# Genome, Transcriptome, and Germplasm Sequencing Uncovers Functional Variation in the Warm-Season Grain Legume Horsegram *Macrotyloma uniflorum* (Lam.) Verdc.

**DOI:** 10.3389/fpls.2021.758119

**Published:** 2021-10-18

**Authors:** H. B. Mahesh, M. K. Prasannakumar, K. G. Manasa, Sampath Perumal, Yogendra Khedikar, Sateesh Kagale, Raju Y. Soolanayakanahally, H. C. Lohithaswa, Annabathula Mohan Rao, Shailaja Hittalmani

**Affiliations:** ^1^Department of Genetics and Plant Breeding, College of Agriculture, Mandya, University of Agricultural Sciences, Bengaluru, India; ^2^Department of Plant Pathology, University of Agricultural Sciences, Bengaluru, India; ^3^Saskatoon Research and Development Centre, Agriculture and Agri-Food Canada, Saskatoon, SK, Canada; ^4^Global Institute for Food Security, University of Saskatchewan, Saskatoon, SK, Canada; ^5^National Research Council Canada, Saskatoon, SK, Canada; ^6^Department of Genetics and Plant Breeding, College of Agriculture, GKVK, University of Agricultural Sciences, Bengaluru, India

**Keywords:** genome assembly, RNA sequencing, underutilized legume, germplasm, ddRAD sequencing, SNP, GWAS, powdery mildew

## Abstract

Horsegram is a grain legume with excellent nutritional and remedial properties and good climate resilience, able to adapt to harsh environmental conditions. Here, we used a combination of short- and long-read sequencing technologies to generate a genome sequence of 279.12Mb, covering 83.53% of the estimated total size of the horsegram genome, and we annotated 24,521 genes. *De novo* prediction of DNA repeats showed that approximately 25.04% of the horsegram genome was made up of repetitive sequences, the lowest among the legume genomes sequenced so far. The major transcription factors identified in the horsegram genome were *bHLH*, *ERF*, *C2H2*, *WRKY*, *NAC*, *MYB*, and *bZIP*, suggesting that horsegram is resistant to drought. Interestingly, the genome is abundant in Bowman–Birk protease inhibitors (BBIs), which can be used as a functional food ingredient. The results of maximum likelihood phylogenetic and estimated synonymous substitution analyses suggested that horsegram is closely related to the common bean and diverged approximately 10.17 million years ago. The double-digested restriction associated DNA (ddRAD) sequencing of 40 germplasms allowed us to identify 3,942 high-quality SNPs in the horsegram genome. A genome-wide association study with powdery mildew identified 10 significant associations similar to the *MLO* and *RPW8*.*2* genes. The reference genome and other genomic information presented in this study will be of great value to horsegram breeding programs. In addition, keeping the increasing demand for food with nutraceutical values in view, these genomic data provide opportunities to explore the possibility of horsegram for use as a source of food and nutraceuticals.

## Introduction

Horsegram [*Macrotyloma uniflorum* (Lam.) Verdc., also known as *Dolichos biflorus* and *Dolichos uniflorus*], is a self-fertile cleistogamous species with a diploid chromosome number of 2*n*=20. It is an annual short-day (SD) climbing legume with a thermo- and photosensitive phenology. However, some lines show day-neutral characteristics as well, and these mature within 120–180days. Horsegram is a member of the family Fabaceae, and it is largely cultivated for food in countries in temperate and subtropical regions, including in India, China, Philippines, Bhutan, Pakistan, Sri Lanka, and Australia (Krishna, 2010). It is an excellent source of phosphorous, iron, molybdenum, vitamins (carotene, thiamine, riboflavin, niacin, and vitamin C), and calcium (Gopalan et al., 1989; Sodani et al., 2006). Its seeds contain about 23% protein and are richer in lysine (an essential amino acid) content than pigeon pea and chickpea, making it a good complement to a cereal-based diet. Its seed and its extracts are widely prescribed in Ayurvedic medicine to treat numerous health disorders, including rheumatism, renal stones, worm conjunctivitis, and piles. The seeds also contain important anti-nutritional proteins, such as trypsin inhibitors and lectins (Liener, 1970). Its high levels of dietary fiber and molecular tannins, low levels of lipids and sodium, and slowly digestible starch make it suitable for a cardio diet and for diabetic and obese patients (Bazzano et al., 2001). Furthermore, horsegram is particularly rich in the antioxidants such as polyphenols, proteins, and flavonoids. Because of its large amount of digestible protein and small amount of digestive inhibitors, it is widely used as feed for milch animals and horses. Overall, horsegram has the potential to serve as food, as forage, and as a nutraceutical to combat malnourishment (Morris, 2008). Despite these benefits, a lack of genomic data has impaired the crop improvement of this species. To date, there have been a very limited number of reports on the transcriptomics and identification of microRNAs and simple sequence repeat (SSR) markers (Bhardwaj et al., 2013; Kaldate et al., 2017) in this species. Genotyping using next-generation sequencing (NGS) technologies has made it feasible for any crop to acquire many genome-wide single nucleotide polymorphism (SNP) markers over a short period, which can be used for genetic estimation of diversity, association mapping, and genetic enhancement through molecular breeding.

The lack of genomic resources and the scarcity of scientific research on this neglected legume prompted us to describe the first whole-genome sequence of the high-yield horsegram variety PHG-9, using both short- and long-read sequencing technologies. We used a ddRADSeq approach to genotype 40 horsegram germplasms to understand genetic diversity and identify markers for molecular breeding.

## Materials and Methods

### High Molecular Weight DNA and RNA Isolation

Genomic DNA (gDNA) was isolated from horsegram variety PHG-9 (Supplementary Figure S1) using the DNAeasy Plant Mini Kit as per the manufacturer’s instructions (Catalog # 69104, Qiagen), and the DNA quality was checked using a NanoDrop device. The RNA from root and leaf tissues was isolated using TRIzol reagent (Catalog # 15596026, Invitrogen), followed by the procedure of the Direct-zol RNA MiniPrep kit (Catalog # R2050, Zymo Research). The integrity and quantity of the RNA were checked using the Agilent 4200 TapeStation system. Then, the RNA from root and leaf tissues was mixed in equimolar proportions and processed as a single sample for RNA-seq library preparation.

### Sequencing Library Preparation and Sequencing

Genomic DNA in the amount of 1μg was fragmented, and a paired-end library was prepared using a NEBNext Ultra DNA library Prep Kit for Illumina (NEB#E7370S/L) following the instructions provided in the user’s manual. In brief, fragmented DNA was end-repaired, and the adapter was ligated, followed by incubation at 20°C for 15min in a thermal cycler. Adapter-ligated DNA ranged from 400 to 500bp was selected using AMPure XP beads, followed by PCR enrichment and PCR cleanup. For RNA-seq library preparation, the TruSeq Stranded Total RNA Library Prep kit was used. Initially, the ribosomal RNA fraction was depleted and processed to prepare the RNA-seq library, as per the instruction manual. The DNA and RNA libraries were sequenced at 2×250, 2×100 ntds using an Illumina HiSeq2500. High-quality and high molecular weight gDNA was used to prepare a 20-kb insert size PacBio library, as detailed in the instruction manual^1^ and was sequenced on the PacBio Sequel system.

### Checking Sequence Data Quality and Filtering

The raw reads for Illumina (181,192,098 paired reads of lengths of 250 ntds) and PacBio (1,074,434 reads) were processed, and low-quality bases were removed. High-quality reads were aligned to the Plant Organelle database, and unmapped reads were considered for nuclear genome assembly. RNA-seq reads (110,054,066 paired reads of length 100 ntds) were pre-processed, and reads belonging to rRNA genes were removed by mapping to the SILVA rRNA database (Quast et al., 2012).

### Genome and Transcriptome Assembly

Illumina short-read data were assembled using MaSuRCA assembler. Hybrid assembly was performed using MaSuRCA with Illumina and PacBio data. The transcripts were assembled using RNA-seq data with a Trinity assembler (Grabherr et al., 2011). Transcriptome assembly was used to scaffold the hybrid assembly using the L_RNA_scaffolder tool (Xue et al., 2013). Genomic completeness was assessed using BUSCO v.3.0.2 (Simão et al., 2015).

### Horsegram Repeat Annotation and Comparative Analyses

A *de novo* and structure-based approach was used to annotate the repeats in horsegram and in five related legume genomes. The genome sequences of five legume genomes, *Medicago truncatula* (barrel medic Mt4.0), *Glycine max* (soybean Williams82-V4), *Phaseolus vulgaris* (common bean, G19833 V1.0), *Vigna angularis* (adzuki bean v1.0 JyYc), and *Vigna radiata* var. *radiata* (mung bean V1.0 VC1973A), were obtained (Schmutz et al., 2010, 2014; Young et al., 2011; Kang et al., 2014, 2015) from the legume information system database.^2^ The EDTA tool (Ou et al., 2019) was used to predict the whole genome repeat proportion in the six legume genomes. The LTR assembly quality of the six legume genomes was estimated using the LTR assembly index (LAI) tool (Ou et al., 2018). In addition, the LTR_retriever (Ou and Jiang, 2018), integrated with the EDTA, was used to estimate the age of full-length LTRs with the default parameters, and the LTR subfamily was classified using the program TEsorter (Zhang et al., 2019). The scaffolds/contigs were used to predict SSRs using the MicroSatellite (MISA) identification tool (Thiel et al., 2003) and classified into Class-I and Class-II SSRs (Temnykh et al., 2001).

### Prediction of Genes and Functional Annotation

The repeat-masked (by RepeatMasker tool) genome was used for gene prediction. *Arabidopsis* was the model, and RNA-seq data were taken as hints to predict the genes using the AUGUSTUS tool (Stanke et al., 2006). The predicted CDSs were compared against the NCBI plant non-redundant protein database using the BLASTX program. Matches with *e*-values ≤10^−5^ and similarity scores of ≥40% were retained for further annotation. RNA-seq evidence for predicted genes was identified by mapping these gene sequences to Trinity-assembled transcripts using the BLAST tool. The protein domains (Pfam), gene ontology (GO) annotations, and KEGG pathways were assigned to predict genes using InterProScan 5.36–75.0 (Jones et al., 2014), and the plant metabolic network was assigned based on BLASTP alignment.

### Orthologous Gene Clustering and Prediction of Transcription Factors

The OrthoVenn2 web platform was used to cluster genes using default parameters (Xu et al., 2019). The predicted genes of the horsegram were compared to previously reported protein sequences of *Phaseolus vulgaris* (common bean), *Vigna angularis* (adzuki bean), *Vigna radiata* var. *radiata* (mung bean), *Glycine max* (soybean), and *Medicago truncatula* (barrel medic). The Transcription Factors (TFs) were predicted by comparing horsegram genes to the plant transcription factor database v5.0. The TFs of the aforementioned legumes were downloaded from the plant TFs database^3^ and clustered using OrthoVenn2 to check the conserved TFs between these important legumes.

### Divergence Analyses

In the phylogenetic analyses, 11,647 orthologous genes were identified between horsegram and its related legume species, and they were compared to construct a data matrix consisting of a concatenated alignment of 14,267,980bps. The sequences of individual orthologous gene sets were aligned using ClustalW version 2.1 (Larkin et al., 2007), and poorly aligned regions were removed using trimAL version 1.2 (Capella-Gutiérrez et al., 2009). Trimmed sequences were concatenated using the Phyutility program (Smith and Dunn, 2008) to produce the final data matrix. The phylogenetic relationships were inferred using the maximum likelihood method implemented in RAxML version 8.0.0 (Stamatakis, 2014) with rapid bootstrapping (100 replications) and a GTRGAMMA substitution model. The resulting phylogenetic tree was visualized using the web server of the Interactive Tree of Life version 4 (Letunic and Bork, 2019).

*Ks* analyses (distribution of synonymous substitutions) were performed as described previously (Kagale et al., 2014). Briefly, for each pair of orthologous genes between horsegram and other legume species, protein sequences were aligned using ClustalW version 2.1 (Larkin et al., 2007), and the corresponding codon alignments were produced using PAL2NAL (Suyama et al., 2006). The *Ks* values for each sequence pair were calculated using the maximum likelihood method, implemented using codeml from the PAML package (Yang, 2007) under the F3x4 model (Goldman and Yang, 1994). Histograms were generated using log-transformed *Ks* values >0.001. Gaussian mixture models were fitted to the ln (*Ks*) values using the R package Mclust, and the number of Gaussian components, the mean for each component, and the data fractions were calculated. The Bayesian information criterion was used to determine the best-fitting model to the data. The fit of the determined models was confirmed using *χ*^2^ tests.

### Horsegram Germplasm Sequencing

Genomic DNA was isolated from young leaves of 40 horsegram germplasm accessions using the DNAeasy Plant Mini Kit (catalog # 69104, Qiagen). The quality and quantity were checked using a NanoDrop device, a Qubit assay, and agarose gel electrophoresis. Double-digested restriction associated DNA (ddRAD) libraries were prepared. In brief, 250–1,000ng DNA was digested with two units *Mluc*I and four units *Sph*I restriction enzymes at 37°C overnight followed by AMPure purification. Adapters specific to *Mluc*I and *Sph*I were ligated to the double-digested DNA using T_4_ DNA ligase at room temperature for 30min and then heat killed at 65°C for 15min. Equal volumes of five samples of ligated DNA were combined to prepare one Illumina library. (In total, eight libraries were prepared for 40 samples.) The samples were size selected (370–470bp) on a 2% SYBR safe gel and purified. The samples were enriched in nine cycles of PCR amplification, and the PCR products were purified using AMPure beads. The concentration of the ddRAD libraries was checked using Qubit, and the quality was assessed using Agilent 2200 TapeStation on a D1000 ScreenTape system. The ddRAD libraries were paired-end (2×100 ntds) sequenced using Illumina HiSeq2500.

### ddRAD Data Pre-processing, Variant Calling, and Functional Annotation of SNPs

The raw reads for all libraries were demultiplexed based on an internal barcode, and sequences were indexed using the process_radtags.pl script in the STACKS tool (Catchen et al., 2013). Low-quality bases were trimmed using the cutadapt tool (Martin, 2011). Processed reads were mapped to the PHG-9 genome as a reference using Bowtie2 (Langmead and Salzberg, 2012), and BAM files were used as an input to call SNPs using SAMtools (Li et al., 2009). All aligned files were converted into the SAM format, and the SNPs were called using SAMtools and bcftools. The alignment data were filtered to retain only high-quality SNPs with a minimum mapping quality of 30, a read depth (DP) of 5, and a minimum allele frequency (MAF) of 0.1 using Tassel-5 (Bradbury et al., 2007). The SnpEff 4.3 (Cingolani et al., 2012) was used to annotate the filtered variants to establish their potential effects on associated genes. Admixture-1.3 (Alexander and Lange, 2011) was used to estimate population structure as detailed previously in other legumes (Li et al., 2018; Wang et al., 2019). To define the most optimal *K* value, the cross-validation (CV) procedure was followed by running *K*=2 to 10. CV error was plotted against all of the assumed population sizes to determine the optimum *K* value.

### Powdery Mildew Disease

In all, 40 germplasms were screened for powdery mildew disease. The severity of the disease was recorded using a rating scale from 0 to 5 measuring the leaf coverage of the powdery mildew, where 0=immune, 1=resistant, 2=moderately resistant, 3=moderately susceptible, 4=susceptible, and 5=highly susceptible. A total of 3,810 SNPs were filtered for a minor allele frequency of 1% and missing genotype of 50%. The missing calls were imputed using Beagle version 5.1 with default criteria (Browning et al., 2018). The imputed SNP genotyping data and log-transformed powdery mildew data were utilized for genome-wide association studies (GWAS). A multi-locus mixed linear model was employed to perform GWAS using GAPIT in R (Lipka et al., 2012). Significant SNPs were identified using at a 5% false discovery rate (FDR). The sequence information of annotated genes was retrieved from the region 200kb upstream and downstream in associated SNPs. The sequence homology search was performed by applying BLAST against the Plant Resistance Genes Database (PRGdb; Osuna-Cruz et al., 2018) and the UniProt database for reviewed genes of powdery mildew disease across different crop species.

## Results

### Genome Sequencing and Assembly

A hybrid genome sequencing approach involving short-read (Illumina) and long-read (PacBio) sequencing technologies was used to assemble the genome sequence of the horsegram variety PHG-9. Illumina paired-end library preparation and sequencing on HiSeq generated 181 million 2×250bp reads (45.30Gb of data). PacBio Sequel sequencing generated 1.07 million reads (6.9Gb of data). Approximately 110 million reads were generated using the strand-specific RNA-sequencing method. A combination approach was used to obtain a reference horsegram genome assembly (Mahesh et al., 2016). First, Illumina short-read sequences were assembled into contigs using the MaSuRCA (Zimin et al., 2013) genome assembler. This resulted in a total of 108,849 contigs, with the largest contig size of 36,091bp and an N50 contig length of 3,372bp. Then, the PacBio long-read sequences were used to build scaffold-level assemblies using MaSuRCA assembler. This enabled us to construct scaffolds by filling gaps left by the Illumina sequence-based assembly.

A combination of genome (Illumina and PacBio Sequel) and transcriptome (Illumina) data with hierarchical scaffolding resulted in a consensus genome size of 279.12Mb and a N50 size of 111,472bp (Table 1). This generated 5,854 scaffolds (>1kb) in the assembly with a largest scaffold size of 754,305bp and 72.6% total assembled genome in the scaffolded contigs. The completeness of the genome and gene repertoire was assessed using BUSCO. Based on a core set of 1,440 single-copy ortholog (SCO) genes from the *Embryophyta* lineage (includes 30 different species), 91.8% were complete in the assembly (84.7% as single copies, 7.1% as duplicates), where 1.8% were fragmented and 6.4% were missing (not found), which indicates that the assembly represented a substantial fraction of the horsegram gene space (Supplementary Figure S2).

**Table 1:** Genome assembly and annotation statistics of horsegram.

Details	Statistics
Number of scaffolds	5,854
Total size of scaffolds (bp)	279,119,361
Assembled genome (%)	83.53
Longest scaffold (bp)	754,305
Shortest scaffold (bp)	1,005
Median scaffold size (bp)	21,357
N50 scaffold length (bp)	111,472
L50 scaffold count	739
GC content (%)	31.40
Scaffold (% *N*)	1.89
Percentage of assembly in scaffolded contigs	72.60
Number of genes predicted	24,521
Total length of genes (bp)	33,902,758
Average gene length (bp)	1,383
Number of mRNA	25,942
Number of exons	147,389
Average exon length (bp)	230
Average exon number per gene	6

### Gene Prediction and Functional Annotation

The prediction of the horsegram genome’s protein-coding genes was done using *ab initio* and homology-based methods. A repeat masked genome was subjected to gene prediction by providing RNA-seq assembled transcripts as hints using the Augustus tool (Stanke et al., 2006). In total, we predicted 24,521 genes with an average gene length of 1,383bp, whose total length was 33,902,758bp (12.15% of the entire assembled genome). We annotated 147,389 exons with an average length of 230bp and an average of six per gene (Table 1).

Among 24,521 predicted genes, 24,143 had significant matches (>60% similarity at the protein level) to existing proteins in the NCBI database. More horsegram proteins showed homology to *Phaseolus vulgaris* (common/French bean), followed by *Vigna angularis* (adzuki bean, syn. *Phaseolus angularis*), *Vigna radiata* var. *radiata* (mung bean or green gram), *Glycine max* (soybean), *Glycine soja* (wild soybean), and *Cajanus cajan* (pigeon pea). More than 69% of predicted genes (17,163 genes) were confirmed by RNA-Seq evidence. The protein family (Pfam) database was used to assign Pfam domain to 19,999 proteins; of these, 11,657 proteins had only one Pfam domain, and 8,342 proteins had more than two Pfam domains. Relative abundance analyses showed that pentatricopeptide repeats (PF01535, PF13041) were the most abundant in horsegram, followed by protein kinase (PF00069), WD domain G-beta repeat (PF00400), leucine-rich repeat (PF13855), protein tyrosine kinase (PF07714), and RNA recognition motif (PF00076) domains.

### Repetitive Sequences and Comparative Analyses With Relative Legume Genomes

**Figure 1:**
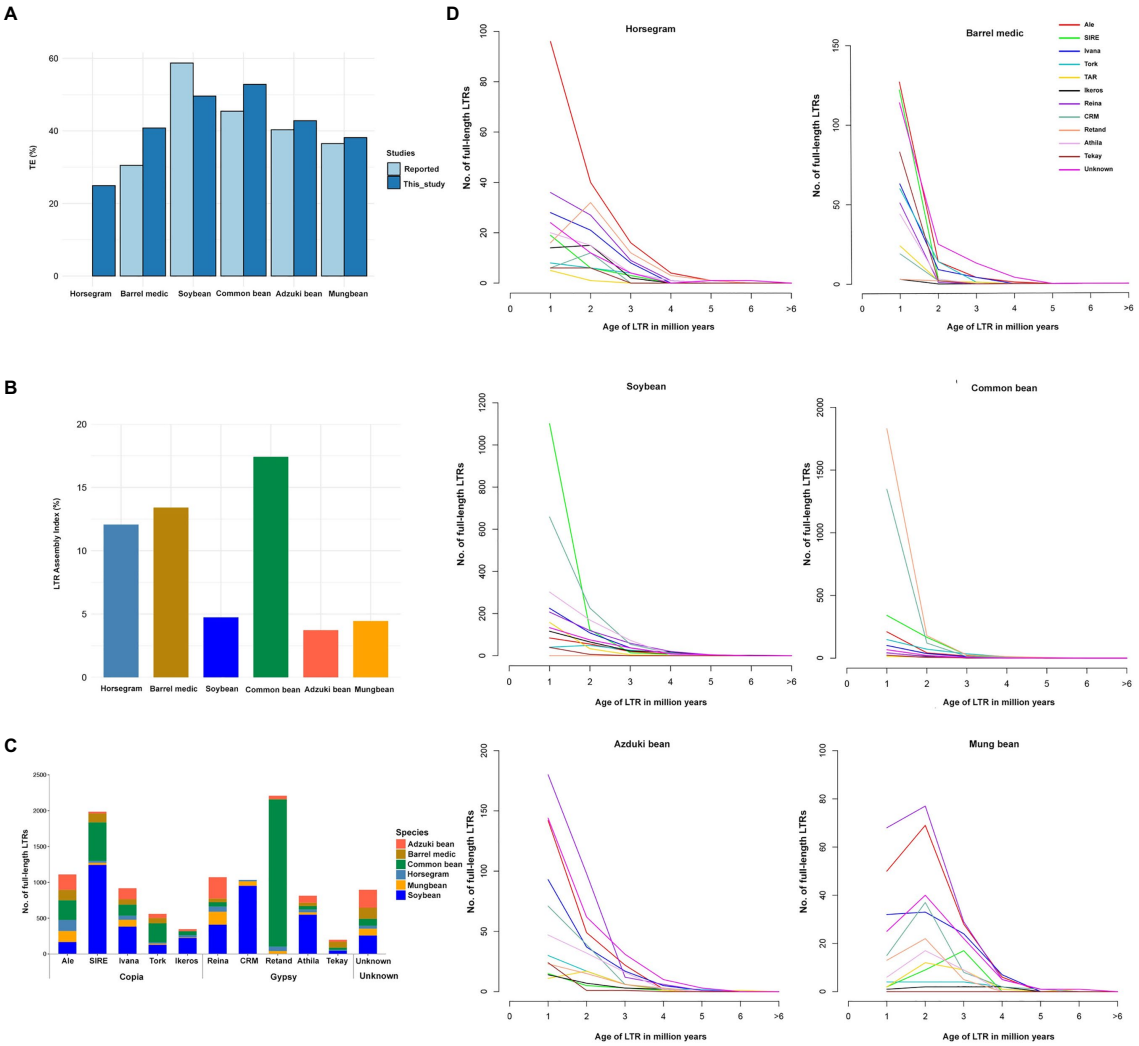
Comparison of repeats in horsegram and the genomes of related legumes. **(A)** Repeat proportions in the six legume crops using the EDTA approach compared to those previously reported. **(B)** LTR assembly index in the six legume genomes. **(C)** Copy numbers of full-length LTRs in six legume genomes. **(D)** Full-length LTRs and age-distribution in six legume genomes. Age of full-length LTRs from 14 different families in horsegram, barrel medic, soybean, common bean, adzuki bean, and mung bean.

*De novo* repeat analyses revealed that approximately 25% of the horsegram genome was occupied by repetitive sequences (Table 2). A major fraction of the genome was occupied by class I retrotransposons (13.57%), followed by class II DNA transposons (11.35%). Among the class I retrotransposons, LTR gypsy (5.57%) and LTR copia (5.35%) were the major contributors to the horsegram genome. A comparison of horsegram genome repeat content to the five previously published legume genomes revealed very low repeat content in the horsegram genome (Figure 1A). The repetitive elements in each of the five legume genomes were annotated using different approaches. To facilitate the comparison and CV, we re-annotated the repeat elements in these species using the same approach as that used for horsegram.

**Table 2:** Summary of repeat proportion in the horsegram genome.

Class	Super family	Count	Coverage (bp)	Masked (%)
LTR	Copia	22,415	14,648,777	5.35
	Gypsy	18,855	15,255,927	5.57
	Unknown	14,539	6,539,966	2.39
LINE	LINE	1,429	705,197	0.26
Sub-total (class I)		57,238	37,149,867	13.57
DNA	DTA	4,316	1,349,900	0.49
	DTC	27,281	7,704,524	2.82
	DTH	366	172,226	0.06
	DTM	15,875	8,660,753	3.17
	DTT	528	155,776	0.06
	Helitron	7,699	2,008,476	0.73
	Unknown	33,472	10,654,223	3.89
MITE	DTA	738	140,381	0.05
	DTC	113	21,000	0.01
	DTH	96	22,381	0.01
	DTM	1,041	156,337	0.06
	DTT	35	7,790	0.00
Sub-total (class II)		91,560	31,053,767	11.35
Total interspersed repeats		148,798	68,203,634	24.91
Others	Low complexity	78	44,478	0.02
	Satellite	3	159	0.00
	Simple repeat	43	5,515	0.00
	rRNA	311	314,595	0.11
	Total	149,233	68,568,381	25.04

The EDTA method predicted a slightly higher repeat content in four of the five legume genomes than the previous estimation (Schmutz et al., 2010, 2014; Young et al., 2011; Kang et al., 2014, 2015). Soybean had 9% fewer repeats than in the previous estimation, but in soybean, >25% unknown LTR fragment corresponded to 9.2% annotated repeats in our current approach, suggesting a more stringent filtering than the EDTA method (Figure 1A). In addition, we evaluated the quality of the assembled genomes using the overall LAI. The genome assemblies developed by long-read assemblies provide a superior assembly of the LTR indices than the short-read assemblies. Our analyses indicated that three long-read-based assemblies (horsegram, barrel medic, and common bean) had higher LAIs (>10), which is indicative of a good genome assembly, while the other three genomes assembled by short reads had lower LAI scores, namely <5 (Figure 1B).

fl-LTRs are not only important for the increase in genome size but also for the evolution of the genome. We identified 547 fl-LTRs belonging to 12 different families in the horsegram genome (Figure 1C). Among the 12 different families, ALE was the most abundant, with 157 copies, followed by the Reina family, with 73 copies. Age analyses of ALE LTR revealed that 96 (61%) copies had less than one million years of age, suggesting recent and continuous proliferation of ALE LTRs in the horsegram (Figure 1D). Similarly, analyses of five other related legume genomes showed a varying number of fl-LTR elements in each genome. For example, common bean contained 2,051 Retand family elements, while soybean contained 1,244 SIRE family elements (Figure 1C). Interestingly, six of the genomes were dominated by different families, each with a recent proliferation (Figure 1D).

### Comparison of Gene Families Among Selected Legumes

**Figure 2:**
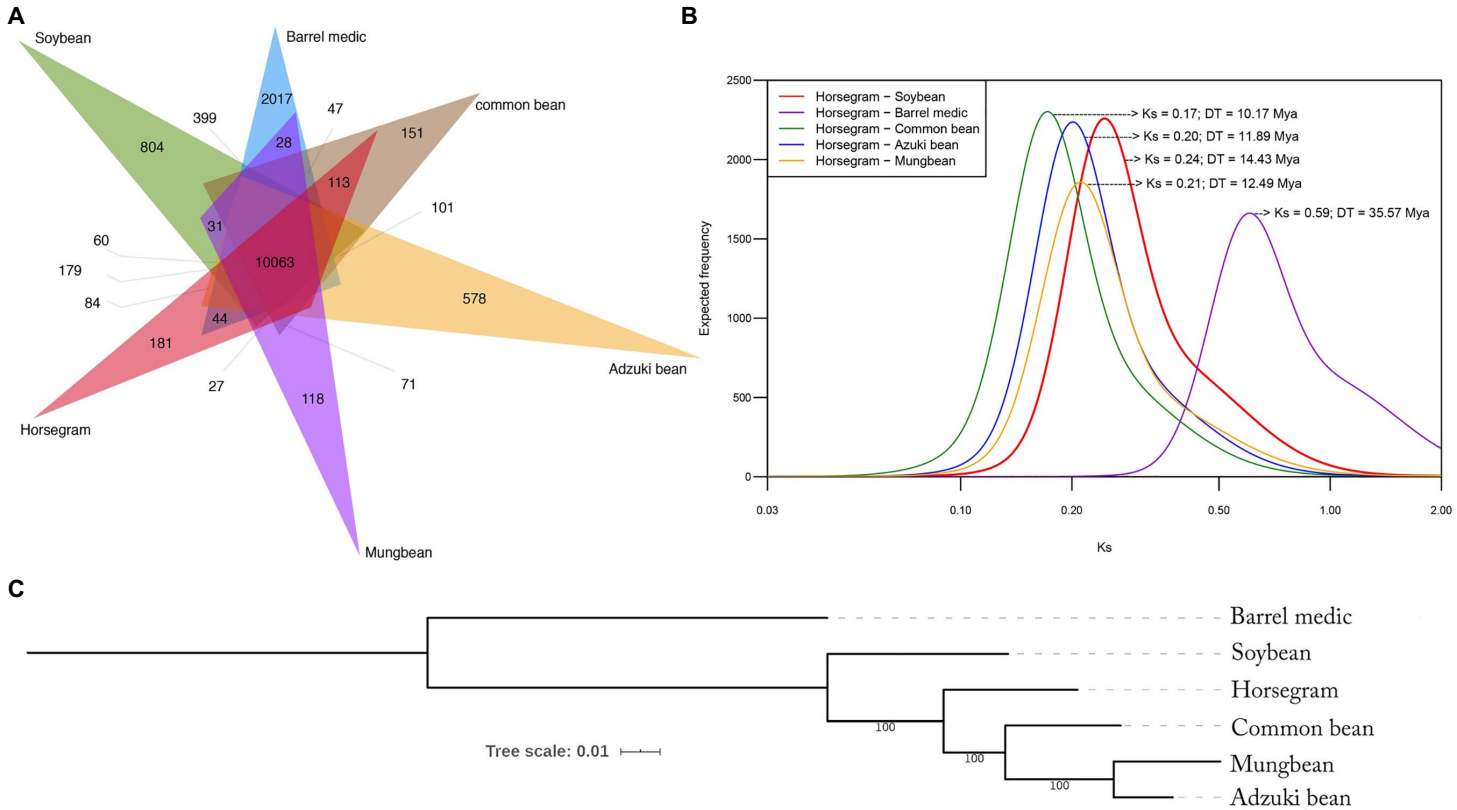
Orthologous gene clustering and phylogenetic relationship. **(A)** Venn diagram showing shared and specific gene clusters among the six major legumes as determined by OrthoVenn. An *e*-value cutoff of 1*e*^−5^ was used for the protein similarity search, and inflation value of 1.5 was used for orthologous clustering. **(B)** Frequency distribution of *Ks* values obtained by pairwise comparison. **(C)** Phylogenetic tree showing evolutionary relationships among six legumes inferred by single-copy orthologs.

A total of 206,480 genes from six legumes, including horsegram (24,521), common bean (27,996), adzuki bean (28,285), mung bean (21,570), soybean (56,209), and barrel medic (47,899), were clustered using the OrthVenn2 tool. Among these species were 26,899 gene clusters (164,679 genes), of which 23,855 core orthologous gene (COG) clusters had genes from at least two species (Figure 2A) and 3,044 SCO gene clusters contained at least one gene from each species. In horsegram, out of a total 24,521 genes, 22,057 genes were clustered into 19,456 COGs and 2,464 singletons that were unique to horsegram. The similarity matrix for pairwise genome comparison showed that the horsegram–soybean combination had more orthologous clusters (21,491), followed by horsegram combinations with common bean (21,425), adzuki bean (21,396), barrel medic/*Medicago* (21,356), and mung bean (21,339).

### Divergence and Evolution

We compared the gene coding sequences of the six legume species to determine their phylogenetic relationships (Figure 2B). A supermatrix was constructed consisting of 11,647 orthologous genes in a concatenated alignment of 14,267,980bp, which was used to define evolutionary relationships among the legume species. Maximum likelihood revealed that horsegram is most closely related to common bean, followed by adzuki bean, mung bean, soybean, and barrel medic (Figure 2B).

To assess the relative age of separation between horsegram and the other species, we estimated *Ks*, the level of synonymous substitutions, using pairs of orthologous sequences between horsegram and other legumes. Major peaks in each *Ks* distribution were identified using mixture model analyses, as described previously (Kagale et al., 2014). Based on a synonymous substitution rate of 8.3×10^−9^, extrapolated using an established age of 19.2 million years for the divergence of *Phaseolus* and *Glycine* (Lavin et al., 2005) and the geometric mean of the peak observed in each *Ks* distribution (Supplementary Table S1), the age of divergence between horsegram and the other leguminous species was estimated. It was 10.17 million years for common bean and 11.89, 12.49, 14.43, and 35.57 million years ago for adzuki bean, mung bean, soybean, and barrel medic, respectively (Figure 2C).

### Transcription Factors

We identified 1,680 genes encoding TFs belonging to 58 families in the horsegram genome. basic helix–loop–helix (*bHLH*), ethylene response factor (*ERF*), *C2H2*, *WRKY*, *NAC*, *MYB*, and *bZIP* were the major TF families. Their relative abundances were compared across the five major legume species, and their proportions were similar in each species (Supplementary Table S2). To confirm the extent of the conservation of TFs among the legume species, the TFs were clustered using OrthoVenn2 (*e*-value 1*e*^−5^ and inflation value 1.5). Out of 1,680 TFs in horsegram, 848 were clustered in 766 orthologous clusters (61 were single-copy gene clusters), which implies their conservation among these legume species, and 75 TFs were unique to the horsegram. *MuNAC4* (NCBI protein accession number HS109648) and *MuWRKY3* (NCBI protein accession number KM520390.1) have previously been cloned in horsegram and over-expressed in groundnut to impart drought tolerance (Pandurangaiah et al., 2014; Kiranmai et al., 2018). These two TFs were predicted *via* BLAST similarity searches to be present in the horsegram. *MuNAC4* showed 99.21% nucleotide identity and 93% query coverage with the *Mu_g21627*.*t1* gene. Similarly, *MuWRKY3* showed 99.8% nucleotide identity and 99% query coverage with the *Mu_g13338*.*t1* gene. This confirms the accuracy of our prediction model.

### Stress Responsive and Anti-nutritional Genes

The inherent stress resilience in horsegram could be partly attributed to 6,347 genes with Pfam domains related to biotic and abiotic stresses (Hittalmani et al., 2017). The PPR repeat, protein kinase, leucine-rich repeat, protein tyrosine kinase, ring finger, salt stress response/antifungal, d-mannose binding lectin, NB-ARC domain, late embryogenesis abundant protein, BTB/PZ domain, CB domain, U-box domain, legume lectin domain, lipoxygenase, NAD-dependent epimerase/dehydratase family, universal stress protein family, HSP70 protein, galactose-binding lectin domain, and dehydrin were some of the major-stress associated Pfam domains enriched in the annotated genes of horsegram.

Out of 34 genes with lipoxygenase function, *Mu_g15332*.*t1* and *Mu_g08128*.*t1* had ~92% sequence homology to previously cloned lipoxygenase gene (NCBI accession number KJ886941.1), exhibiting defense against pests and pathogens (Roopashree et al., 2006). In addition, BLAST analyses with drought-induced ESTs (958 ESTs retrieved from GenBank NCBI accession numbers DR988679–DR989637) showed sequence similarity with previously annotated genes of horsegram (Reddy et al., 2008). Among the 958 ESTs, 461 mapped to 296 genes that indicated a role in stress tolerance (Supplementary Data S1).

Proteinase inhibitors serve as a means of defense against pests and diseases in addition to controlling protease activity in plants. We identified 35 genes based on Pfam domains encoding various protease inhibitors, such as serine Bowman–Birk protease inhibitor (BBI), LTP family/protease inhibitor, serpin (serine protease inhibitor), trypsin, and protease inhibitor (Supplementary Data S2). *Mu_g18571*.*t1*, which codes for BBI, had sequence homology with an already cloned gene (NCBI nucleotide accession JQ259858.1).

### Genes Involved in Photoperiod Sensitivity in Horsegram

The transition of apical meristem from the vegetative to reproductive (flowering) stage is a very crucial stage in the life cycle of any plant. Horsegram is a SD photoperiod-sensitive plant, and it flowers in shorter photoperiods. The development of photoperiod-insensitive horsegram varieties plays a major role in growing this nutri-legume in many cultivation areas and in different cropping seasons. The cloned and characterized *CONSTANS* (*CO*) and *HEADING DATE1* (*Hd1*) genes (Putterill et al., 1995; Yano et al., 2000), which promote flowering under SD conditions in *Arabidopsis* and *Oryza*, respectively, were predicted in horsegram through a protein–protein homology search. The *Mu_g12727*.*t1* gene was found to share 49 and 46% homology with the *CO* and *Hd1* genes, respectively. This gene contains CCT (CONSTANS, CO-like, and TOC1) motif and B-box zinc finger domains based on Pfam domain analyses. The CCT domain contains 43 amino acids near the C-terminal end of the protein often involved in light signal transduction. It has been reported that the CCT domain is associated with other domains, such as the B-box zinc finger, GATA zinc finger, and TIFY (previously known as ZIM) motif. Pfam analyses identified 36 genes with these domains in horsegram (Supplementary Data S3); further, 20 previously validated *Arabidopsis* and *Oryza* protein sequences with a CCT domain were retrieved from UniProt and aligned with *Mu_g12727*.*t1* to determine their evolutionary relationship (Supplementary Figure S3).

### Large-Scale Identification of Genetic Markers

*In silico* prediction of the horsegram genome provided 77,821 SSRs including mono- (63,087), di- (10,755), tri- (3,882), tetra- (3), penta- (0), and hexa- (94) nucleotide repeats. Of 5,854 scaffolds, 4,468 contained SSRs that contributed to 0.874% (2,438,790 bases) of the total horsegram genome. Among di-nucleotides, AT/AT (7,407) repeats were the most abundant, followed by AG/CT (2,309), AC/GT (1,031), and CG/CG (8). Similarly, AAT/ATT (1,864) repeats were followed by AAG/CTT (713), ATC/ATG (411), AAC/GTT (315), ACC/GGT (223), AGG/CCT (135), AGC/CTG (103), ACT/AGT (59), CCG/CGG (35), and ACG/CGT (24). Interestingly, only one type of tetra-repeat (AGAT/ATCT) was identified. The classification of di-, tri-, and tetra- SSRs based on length of repeat motif resulted in 109 (16 di-, one tri-, three tetra-, and 89 hexa-) hypervariable (Class I with ≥20bp repeat motif) and 8,459 (4,962 di-, and 3,497 tri-) potentially variable (Class II with >12 to <19bp) SSRs in the horsegram genome. The remaining 4,211 SSRs were <12 ntds in length (Supplementary Table S3).

### Germplasm Sequencing, SNP Identification, and Genetic Diversity

**Figure 3:**
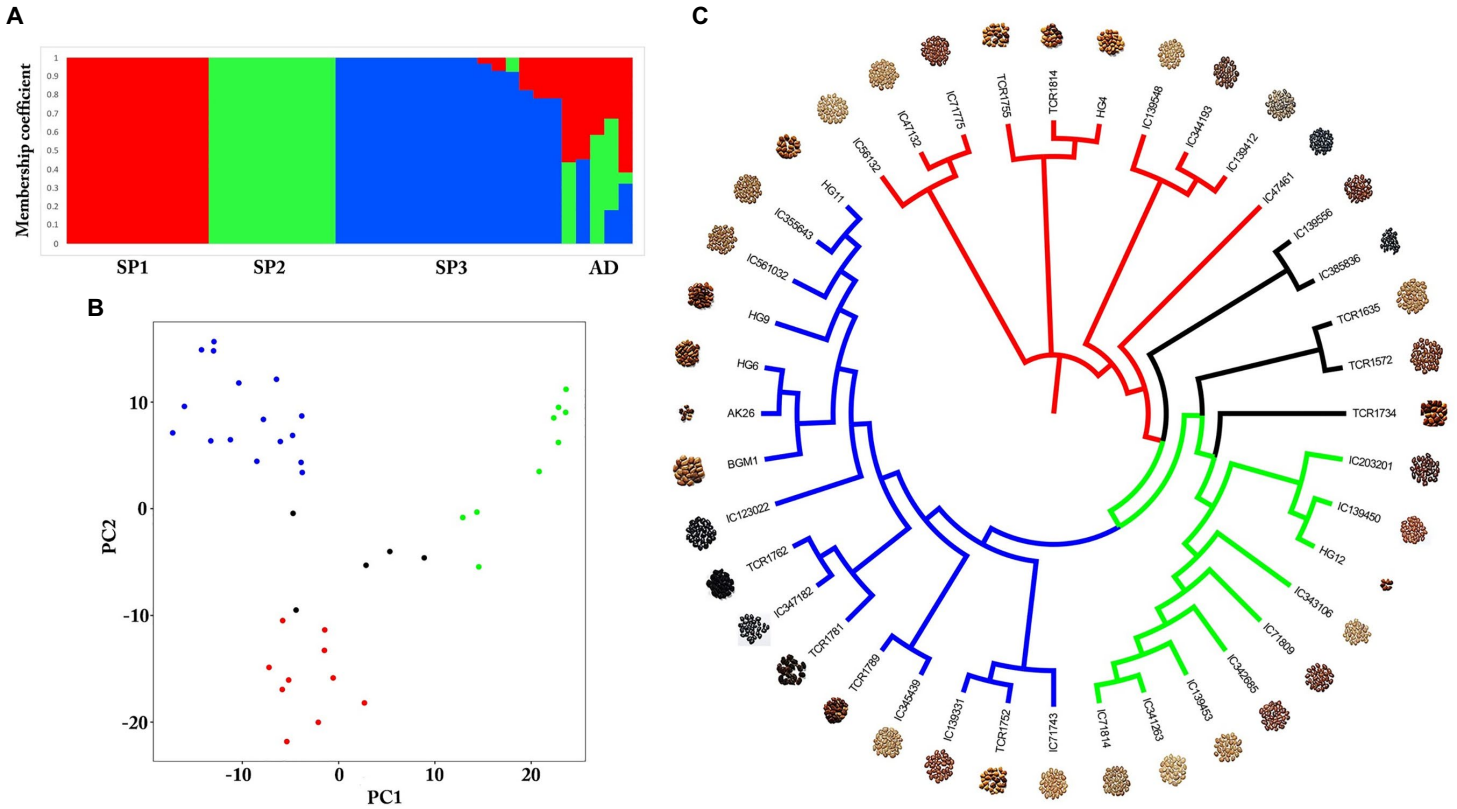
Population structure of horsegram germplasm accessions. **(A)** Population stratification of 40 horsegram germplasm accessions. **(B)** Principal component analyses showing four groups of germplasm. **(C)** Genome-wide single nucleotide polymorphisms (SNPs) based on a dendrogram showing the genetic diversity of horsegram germplasm accessions with reference to PHG-9 reference genome.

Genome-wide SNPs were identified in a total of 40 diverse germplasms using ddRADSeq technology to understand and study the genome-wide variability in the horsegram. Sequence-based barcoding followed by a pooling of eight genotypes per library and sequencing on Illumina HiSeq2500 generated a total of ~162.6 million paired reads with an average of four million reads per genotype (Supplementary Table S4). Filtered and high-quality reads were aligned to the reference genome. A total of 18,032 SNPs were identified across 40 germplasms at a read depth of 10 and an MAF of 0.05. The population structure of 40 germplasms based on multi-locus SNPs was estimated using the ADMIXTURE tool. Using the maximum likelihood method, the optimal partitioning of the population with lowest CV error was obtained at *K*=3 (Figures 3A,B). The dendrogram based on SNP markers grouped 40 germplasms into three distinct clusters (Figure 3C) indicating low genetic diversity among horsegram germplasm selected for ddRADSeq. The estimated Tajima’s *D* value was 3.74, indicating a lack of rare alleles in the germplasm population. Out of 40 germplasms, five germplasms, namely TCR1734, TCR1572, TCR1635, IC385836 and IC139556, were admixed (AD) genotypes. BGM-1 and AK26 varieties were bred systematically and were assigned to cluster III (SP3; Figures 3A,C).

### Functional Annotation of the SNPs

Approximately 3,942 high-quality SNPs (MAF=0.01, DP=5) were retained for downstream analyses, of which 2,277 (57.76%) were transition substitutions (1,145 A/G and 1,132 C/T), and 1,665 (42.24%) were transversion substitutions (404 G/T, 313 G/C, 399 A/C, and A/T 549). This SNP pattern showed that transition substitutions were predominant; the transition-to-transversion rate was 1.37 in the selected horsegram germplasm. These SNPs were annotated using the SnpEff tool to understand their potential effects in their genomic locations. Of 3,942 SNPs, 1,150 (18.73%), 435 (7.085%), 2,931 (47.736%), 528 (8.599%), 986 (16.059%), 58 (0.945%), 32 (0.521%), and 20 (0.326%) spanned the downstream, exon, intergenic, intron, upstream, 3'-UTR, 5'-UTR, and splice site regions, respectively (Supplementary Figure S4). Furthermore, functional classification in the coding region of genes revealed 233 missense/nonsynonymous, 12 nonsense/stop gained, and 178 silent/synonymous mutations (Supplementary Table S5).

### Genome-Wide Association for Powdery Mildew Disease

**Figure 4:**
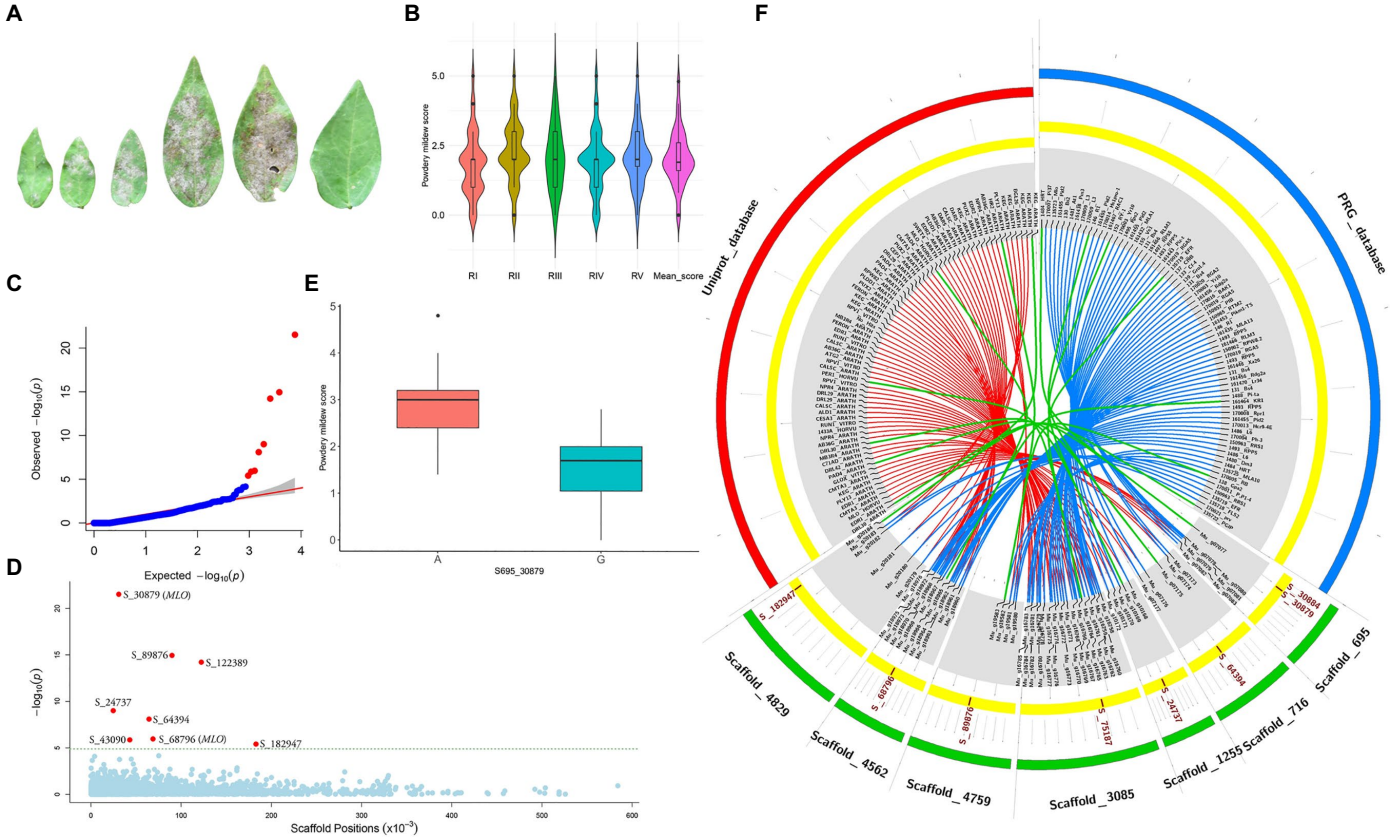
Genome-wide association analyses of powdery mildew disease. **(A)** Different stages of disease progression. **(B)** Violin plots depicting phenotypic distribution of powdery mildew disease scores among 40 horsegram germplasms. **(C)** Quantile–quantile plot of SNPs. **(D)** Manhattan plot of the associated SNPs. The plot shows negative values of *p* on a log 10 scale (*y*-axis) plotted against SNP positions of scaffold (*x*-axis). **(E)** Distribution of G and A alleles of S_30879 SNP. **(F)** Circos diagram showing the homology of SNP-associated horsegram genes with cloned resistance genes against powdery mildew in the Plant Resistance Genes database (blue concentric ring) and UniProt database for powdery mildew (red concentric ring). The SNPs (brown) on scaffolds (green) of horsegram with predicted genes are shown in gray.

Powdery mildew disease caused by the biotrophic parasite *Erysiphe polygoni* causes chlorosis followed by drying of leaves and defoliation and results in 70–80% yield loss and poor-quality grains (Figure 4A). The field evaluation of 40 horsegram germplasms revealed wide variation in disease expression (Figure 4B). A total of 4,624 variants were used for GWAS analyses, and we identified 10 significant SNPs (value of *p*, 5.43×10^−5^ to 4.45×10^−22^) associated with powdery mildew resistance in horsegram, with effect estimates ranging from 0.03 to 0.15 (Table 3; Figures 4C–E). Using the SnpEff annotation tool, the functionality of the associated SNPs was assigned, and we found five SNPs in the downstream region, three in intergenic regions, whereas S_64394 and S_68796 were in intron and upstream regions, respectively. A further comparison of genes was conducted ~200kb upstream and downstream of the scaffold of associated SNPs with PRGdb (Sanseverino et al., 2010), and Uniprot reviewed database for powdery mildew disease resistance genes. The SNPs were highly comparable with the candidate genes governing powdery mildew resistance in *Hordeum vulgare* and *Arabidopsis*. The overall comparison of annotated SNPs with the PRGdb indicated the putativeness of the genomic regions contributing to disease resistance in horsegram and candidate genes from Uniprot database for powdery mildew identified in the several crop species has been represented in the Circos (Figure 4F).

**Table 3:** Significant markers associated with powdery mildew in horsegram.

SNP ID	Alleles	Position	*p*	Minimum allele frequency (MAF)	False discovery rate (FDR) adjusted *p*	Effect estimates	Candidate gene	Model
S_30879	G/A	30,879	4.45E-22	0.39	1.76E-18	−0.15	*MLO (Hordeum vulgare)*	MLMM
S_89876	C/T	89,876	1.51E-15	0.25	2.98E-12	−0.09		MLMM
S_122389	A/G	122,389	8.49E-15	0.13	1.12E-11	−0.23		MLMM
S_24737	A/G	24,737	1.42E-09	0.11	1.40E-06	−0.08		MLMM
S_64394	G/T	64,394	1.26E-08	0.11	9.91E-06	−0.06		MLMM
S_68796	A/G	68,796	1.40E-06	0.08	8.56E-04	−0.07	*MLO (Hordeum vulgare)*	MLMM
S_43090	C/T	43,090	1.52E-06	0.46	8.56E-04	0.03		MLMM
S_182947	G/T	182,947	5.22E-06	0.10	2.57E-03	0.04		MLMM
S_75187	T/A	75,187	5.43E-05	0.13	2.38E-02	–	*RPW8*.*2 (Arabidopsis thaliana)*	MLMM
S_30879	G/A	30,879	2.28E-05	0.3875	4.35E-02	−0.12	*MLO (Hordeum vulgare)*	FarmCPU
S_30884	T/C	30,884	2.28E-05	0.3875	4.35E-02	−0.12		FarmCPU

## Discussion

Horsegram is a nutritious and stress-resilient legume, referred to as an indicator crop. However, to date, little scientific work has been devoted to its improvement. Considering that horsegram is important for future sustainable nutrition and food security, it is important to understand its genetic architecture using genomic resources. The first framework linkage map was recently constructed using 211 SSR markers, and the same study mapped QTLs for agronomically important traits (Chahota et al., 2020). This crop gas many desirable traits, including drought tolerance, antioxidant activity, antimicrobial properties, and high protein and iron contents. The development and utilization of genomic resources for genetic improvement may be extremely useful.

The horsegram variety PHG-9 was chosen for whole genome sequencing, as it has had wide cultivation for its high productivity. The reference genome sequence and re-sequencing of 40 germplasm presented in this manuscript have tremendous scope for the genetic improvement of the horsegram in the near future. The combination of short- and long-read sequencing technologies helped us to assemble up to 83.53% of the total estimated genome size (334Mb). Previous genome sequencing studies of important legumes have reported similar genome coverage (Schmutz et al., 2010, 2014; Varshney et al., 2012, 2013; Kang et al., 2014; Yang et al., 2015). Further advancements in long-read sequencing technologies and construction of high-density linkage map will facilitate building pseudomolecule-level genome assembly for horsegram in near future. The completeness of genome assembly, quality, and gene set of the present study confirmed the presence of ~93.6% of the universal SCO genes useful criteria for further downstream analyses of the horsegram genome. According to our analyses, 25.04% of the horsegram genome is occupied by repetitive sequences. Compared to five legume relatives, horsegram has a low level of repeats, indicating less proliferation in the genome. Furthermore, our long-read-based genome assembly had high (>12) LAI values, suggesting a good assembly of the repeat fraction. However, the unassembled horsegram genome, which usually consists of repeat related sequences, may contribute to a slight increase in repeat content.

fl-LTR analyses revealed recently amplified (<1 Mya) ALE family LTRs specific to the horsegram genome. For example, the genomes of the other five legume analyses showed active amplification of different LTR families, demonstrating a unique role for each genome. Further analyses of these LTRs may provide insight into how their evolution relates to each legume genome. Gene prediction supported by RNA seq data, followed by structural annotations of the horsegram genome, enabled the identification of 25,942 protein-coding genes including 147,389 exons with average gene and exon lengths of 1,383 and 230bp, respectively. Sequence homology-based functional annotation of predicted genes helped to assign gene function, gene ontology, pathway information, and protein family domains. This genomic information can help horsegram breeders understand the genetic architecture of important traits, which can aid the process of marker-assisted selection and candidate gene discovery. Furthermore, this may improve the possibility of unraveling the molecular mechanisms underpinning trait variation in future studies through the establishment of the complete gene repertoire of the horsegram.

The genomic resources of legume crops have opened the door to translational research with greater success in marker-assisted selection, genomic selection, and high-yield varieties. A comparison of the 24,521 gene families of horsegram with five sequenced legumes (adzuki bean, common bean, mung bean, barrel medic, and soybean) showed that 79% (19,456) of the genes are orthologous to those of the other five legumes. These conserved orthologous genes also have conserved gene functions, offering an opportunity for comparative functional genomic studies in horsegram and other species (Varshney et al., 2013). A minimum of 77.54% of the predicted horsegram genes have a history of duplication. Over the same period of time, 3,044 genes remained SCOs without duplication or loss, indicating the essential role of these genes during the evolution of the legumes.

A protein–protein homology search against a plant TFs database revealed that bHLH, ERF, C2H2, WRKY, and NAC transcription factors were abundant in the horsegram genome. These TFs confer tolerance of biotic and abiotic stresses in many crops including horsegram (Nuruzzaman et al., 2013; Pandurangaiah et al., 2014; Kiranmai et al., 2016, 2018; Sun et al., 2018; Han et al., 2020). The inherent drought tolerance mechanism of horsegram is most likely due to the presence of these TFs in its genome. The identification and cloning of novel TFs in horsegram will assist in understanding signaling and transcriptional regulation for various biotic and abiotic stresses, and the same information can be used to develop varieties with broad-spectrum biotic and abiotic tolerance. Horsegram exhibits an innate defense against pest/pathogens from the genes encoding lectin and lipoxygenase-like functions (Roopashree et al., 2006).

Functional annotation allowed us to identify 170 genes that encode lectin and lipoxygenase activity. These lectin proteins have specific affinities for carbohydrate moieties. When insects feed on horsegram plants, lectins bind to glycoproteins in the peritrophic matrix lining of the insect midgut, leading to a disruption of digestive processes and nutrient assimilation (Michiels et al., 2010; Roy et al., 2014). These lectins also serve as a source for protein–carbohydrate interactions in the horsegram. According to our analyses, horsegram is enriched in several drought- and pest/pathogen-responsive genes, which is possibly responsible for its high resistance to several environmental stresses (Roopashree et al., 2006; Bhardwaj et al., 2013).

Since the establishment of the nutraceutical concept and with the growth health consciousness, the demand for nutraceutical and functional foods has been increasing. In recent years, the isolation and utilization of potential antioxidants from legumes such as horsegram have gained relevance, as they decrease the risk of intestinal disease, diabetes, coronary heart disease, and dental caries. Functional annotation of the horsegram gene repertoire has allowed us to identify a number of protease inhibitors, particularly trypsin inhibitors. The BBI is being explored as a functional food ingredient in other legumes such as soybean (Hernández-Ledesma et al., 2009). Interestingly, these BBIs have potential applications in human health for combating cancer, obesity, multiple sclerosis, ulcerative colitis, and several degenerative and autoimmune diseases (Chen et al., 2005; Duranti, 2006; Gran et al., 2006; Lichtenstein et al., 2008; Kumar and Gowda, 2013). These innate nutritional properties of horsegram make it a potentially excellent functional food ingredient.

The utilization of novel genomic tools to investigate variability in the horsegram germplasm is a viable option for improving horsegram’s agronomically important traits. The sequencing of 40 horsegram germplasm accessions with significant plant breeding implications allowed us to identify 18,032 SNPs against the PHG-9 reference genome. Validating this large number of SNPs is laborious and expensive, and discovering high-quality SNPs with a minimum number of false positives remains challenging. Using stringent filtering criteria, 3,942, SNPs were identified, and their genetic effects were annotated. Of these, 427 missense/nonsense nonsynonymous SNPs were identified, spanning 259 annotated horsegram genes. These SNPs can be potential sources for mining functional alleles in different horsegram germplasm accessions and driving genomics-assisted crop improvement through genetic and association mapping (Bajaj et al., 2016). Maps of genetic linkages and of the QTLs that govern the traits are the starting point of any molecular breeding program. In this direction, a framework genetic map for mapping drought and yield-related QTLs has been reported (Chahota et al., 2020). Further, horsegram requires molecular breeding and genomics to overcome the drawbacks of conventional breeding, which is laborious and time-consuming. The adoption of genomics-assisted breeding strategies through NGS and high-throughput genotyping platforms has helped breeders to improve efficiency and accelerate their success (Pandey et al., 2016).

Powdery mildew is a serious limitation for horsegram production. Despite the significant damage caused by powdery mildew disease in horsegram, there have been no efforts to determine the genetic basis of the disease and identify resistant genes. The GWAS results of our study identified the SNPs associated with powdery mildew resistance. A protein–protein homology search identified candidate genes in horsegram that are homologous to the genes *MLO* and *RPW8*.*2* that are conferring resistance to powdery mildew in *H. vulgare* and *Arabidopsis*, respectively. The *MLO* gene has been studied in several monocot and eudicot species including *Triticum aestivum*, *Oryza sativa*, *Brachypodium distachyon*, *Solanum lycopersicum*, *Vitis vinifera*, and *Cucumis sativus*. The mapping of powdery mildew resistance genes has helped to identify QTLs in mung bean (Chaitieng et al., 2002; Kasettranan et al., 2010) and field pea (Katoch et al., 2010; Fondevilla et al., 2011; Pavan et al., 2011), which may be of potential interest in comparative genomic studies in horsegram.

Further assessment of the horsegram germplasm through genomic platforms will lead to the identification of putative disease-resistance regions through GWAS, and detailed tracking of these regions using the reported candidates in other legume/model crops will allow us to work toward identifying possible resistance genes for powdery mildew and the subsequent characterization of candidate genes in horsegram. In addition to SNPs and GWAS, we also identified di-, tri-, and tetra-SSRs within the PHG-9 genome, which can serve the immediate purposes of any molecular biology laboratory with limited resources for genetic diversity analyses and QTL mapping. Overall, these SNPs and SSRs can be used for linkage map construction, gene/QTL mapping, allelic diversity analyses, and population genetic structure analyses. The successful use of these markers can support marker-assisted selection of desirable traits and germplasm accessions for horsegram improvement.

## Data Availability Statement

The whole genome assembly has been deposited at NCBI/DDBJ/EMBL with the accession ID NSKJ00000000. The version described in this paper is NSKJ01000000. The raw sequence reads of Illumina, PacBio, and RNA sequencing are deposited in NCBI SRA database with the accession numbers SRR10854883, SRR10854881, and SRR10854882, respectively. The ddRADSeq raw sequence reads of Illumina is deposited in NCBI SRA database with the accession numbers from SRR14229121 to SRR14229160 under NCBI BioProject ID PRJNA721661.

## Author Contributions

HM designed the experiment, performed the genome assembly and functional annotation, gene family analyses, transcription-factor mining, SSR identification, SNP calling, and other analyses, submitted genome and raw sequence data to NCBI, prepared the tables and figures, and wrote the manuscript. MP performed the powdery mildew disease scoring and revised the manuscript. KM performed the SNP annotation. SP performed the repeat analyses. SK performed the evolutionary divergence analyses. YK performed the SNP imputation, PCA, and GWAS analyses and prepared the figures. RS, HL, and AR revised the manuscript. SH conceived the project and edited the manuscript. All authors contributed to the article and approved the submitted version.

## Funding

This project was supported by the Director of Research, University of Agricultural Sciences, Bengaluru, India.

## Conflict of Interest

The authors declare that the research was conducted in the absence of any commercial or financial relationships that could be construed as a potential conflict of interest.

## Publisher’s Note

All claims expressed in this article are solely those of the authors and do not necessarily represent those of their affiliated organizations, or those of the publisher, the editors and the reviewers. Any product that may be evaluated in this article, or claim that may be made by its manufacturer, is not guaranteed or endorsed by the publisher.

## Footnotes

^1^ https://www.pacb.com

^2^ https://legumeinfo.org/genomes

^3^ http://planttfdb.cbi.pku.edu.cn
