# Supplementary material for: Genome, Transcriptome, and Germplasm Sequencing Uncovers Functional Variation in the Warm-Season Grain Legume Horsegram Macrotyloma uniflorum (Lam.) Verdc
Source: Front Plant Sci. 2021 Oct 18;12:758119. doi: 10.3389/fpls.2021.758119 (PMC8558620; doi:10.3389/fpls.2021.758119)
Supplement: Supplementary file 1 [file Data_Sheet_1.PDF]

Supplementary Data 1:

Protein sequences of predicted genes of PHG-9 variety matched to ESTs.

>Mu_g00116.t1 PREDICTED: chlorophyll a-b binding protein of LHCII type 1-like [Vigna angularis]
MAASTMALSSPSLAGKAMKLSPSTTNLSGGRVSMRKTPSKSVSSGSPWYGPDRVKYLGPFSGEAPSYLTGEFPG
DYGWDTAGLSADPETFARNRELEVIHSRWAMLGALGCVFPELLARNGVKFGEAVWFKAGSQIFSEGGLDYLGN
PSLIHAQSILAIWATQVILMGAVEGYRIAGGPLGDVSDPIYPGGSFDPLGLADDPDAFAELKVKELKNGRLAMFS
MFGFFVQAIVTGKGPLENLADHLADPVNNNAWAYATNFVPGK
>Mu_g00173.t1 protein phosphatase 2C 70 [Vigna radiata var. radiata]
MGPMLQTILTVXXXXXXXXXXXXXXXXXXKPWRFFFSSRFFSSIKGELERPLVADDDEAYASPLNPNNELPRDYDL
EGACYPSEAHFRSPRTHGQGLVHKQRLQSLSLPPTIPQGGDSLVVDVISDPSSEDADYGQTLKLSSAHLAQIQKHG
PPNCQNDRLQDFVRWDISDQRSCLTLEVISGPSQGLRCSVQSINSSRLPLTLGRVSPSDLLIKDSEVSGKHAMIKW
NLDKMKWELVDMGSLNGTLLNSRPINHPDTESRNWGDPMNLANGDIITLGTTSKVMVHITSQTQHHIPFGVG
MASDPMAMRRGGKRFAMEDVCYYQWPLPGLDQFGIFGVCDGHGGDGAAKSASKLFPEIIASILSDSIRRERVLSH
RDASDILRDAFSQTEAHMNHYYEGCTATVLLVWTDGDENFFAQCANVGDSACIMSLNGKQIKMTEDHKITNYS
ERLRIEETGEPLKDGETRLYGINLARMLGDKFLKQQDTRFSSEPYISEVVHIDQASKAFAILASDGLWDVISVKKA
IQLVLQMRERCYSERENTAEKTASLLLNEAKTLRTKDNTSVIFLDFDTFNKFSCKVES
>Mu_g00411.t1 elongation factor 1-alpha [Vigna radiata var. radiata]
MGKEKVHINIVVIGHVDSGKSTTTGHLIYKLGGIDKRVIERFEKEAAEMNKRSFKYAWVLDKLKAERERGITIDIA
LWKFETTKYYCTVIDAPGHRDFIKNMITGTSQADCAVLIIDSTTGGFEAGISKDGQTREHALLAFTLGVKQMICCC
NKMDATTPKYSKARYDEIVKEVSSYLKKVGYNPDKIPFVPISGFEGDNMIERSTNLDWYKGPTLLEALDQINEPK
RPSDKPLRLPLQDVYKIGGIGTVPVGRVETGVLKPGMVVTFAPTGLTTEVKSVEMHHEALQEALPGDNVGFNVK
NVAVKDLKRGFVASNSKDDPAKEAANFTSQVIIMNHPGQIGNGYAPVLDCHTSHIAVKFAELVTKIDRRSGKELE
KEPKFLKNGDAGFVKMIPTKPMVVETFSEYPPLGRFAVRDMRQTVAVGVIKSVEKKDPTGAKVTKAAQKKK
>Mu_g00876.t1 histone H2A [Sesamum indicum]
MDTTGKIKKGAGGRKGGGPKKKPVSRSVKAGLQFPVGRIGRYLKKGRYSQRVGTGAPVYLAAVLEYLAAEVLEL
AGNAARDNKKNRIIPRHVLLAVRNDEELGKLLAGVTIAHGGVLPNINPVLLPKKTEKSAKEPKSPSKATKSPKKS
>Mu_g00877.t1 PREDICTED: histone H2A [Capsicum annuum]
MDAGGKIKKGAGGRKGGGPKKKPVSRSVKAGLQFPVGRIGRYLKKGRYSQRVGTGAPVYLAAVLEYLAAEVLEL
AGNAARDNKKNRIIPRHVLLAVRNDEELGKLLAGVTIAHGGVLPNINPVLLPKKSDKISKEPKSPSKATKSPKKA
>Mu_g01082.t1 hypothetical protein PHAVU_010G132900g [Phaseolus vulgaris]
MMPSSIPQYVTEAFTGPMPDPPGVKLKKEGLRAKHPVVFVPGIVTGGLELWEGHQCAEGLFRKRLWGGTFGEV
YKRPSCWVDHMSLDNETGLDPPGIRVRPVSGLVAADYFAAGYFVWAVLIANLARIGYEEKTMYMAAYDWRIAF
QNTEVRDQTLSRIKSNIELMVATNGGNKAVIIPHSMGVLYFLHFMKWVEAPAPMGGGGGPDWCSKYIKAVINIG
GPFLGVPKAIAGLFSAEARDIAVARTIAPGFLDNDLFRLQTLQHVMKMTRSWDSTMSMIPRGGDTIWGDLDWS
PEEGYHPGKRKHSNNNNTQLTKQETNETNSVNYGRMISFGRDVAEAPSSKIEMTDFRGALKGRSVANTTCRDV
WTEYHEMGIEGVRAVAEHKVYTAGSIIELLNFVAPKMMARGSAHFSYGIADNLDDPKYNHYKYWSNPLETKLP
NAPDMEIFSMYGVGLPTERSYVYKLTPFAECYIPFEIDTTQDGGHDEDSCLRGGVYTVDGDETVPVLSSGFMCAK
GWRGKTRFNPSGIPTYVREYDHSPPANLLEGRGTQSGAHVDIMGNFALIEDVIRVAAGGTGEDLGGDKVYSDIFK
WSEKIKLPL
>Mu_g01479.t1 hypothetical protein PHAVU_003G164100g [Phaseolus vulgaris]
MAVSDGVVKKIVLSYTYVAIWIFLSFTVIVYNKYILDRKMYNWPYPISLTMIHMAFCSSLAYVLVRVFKLVEPVSM
SRDLYFKSVVPIGALYSLSLWFSNSAYIYLSVSFIQMLKALMPVAVYSIGVLFKKEAFKNETMANMVSISLGVAIAA
YGEAKFDTWGVTLQLLAVAFEATRLVLIQILLNSKGISLNPITSLYYIAPCCLVFLTVPWIVMEYPSLRDNSSFHLD
FAIFGTNSACAFALNLAVFLLVGKTSALTMNVAGVVKDWLLIAFSWSVIKDTVTPINLIGYGLAFLGVAYYNHCK
LQALKASEAQKKAQQADEEAGRLLEQKDGEGTGRKNDNQN
>Mu_g01751.t1 PREDICTED: stress-response A/B barrel domain-containing protein UP3 [Vigna angularis]
MLRLRTPLSHPFSQTFSPSRQLRKVQARPFRSSIKMTSAKTVEHVVLFKVKEETEASKVSEMVNGLGSLLSLDTV
LHLSVGPLLRNRSSSLTFTHMLHSRYKSKEDLQAYSAHPSHVSVVKDNVLPIIEDIMAVDWVADGLGTLVPPPGS
ALRLSFLKLKEGAAGDEVLGVVRGIPDIFKEISEFSVGENFSPGRAKGFSIASLAVFPGPSELEAVDSNQELVNYQK
DKVRDHIESVVVVDYVVPPPPPAQTASL
>Mu_g02948.t1 hypothetical protein PHAVU_001G264200g [Phaseolus vulgaris]
MAACSYISAANLNCLVGARNLSNPKLGSADATISLSFSGSDSMGLSVRPASIRAPMRNHFSPLRVVCVDYPRPELE
STVNFIEAAYLSSTFRASPRPLQPLKIVIAGAGLAGLSTAKYLADAGHKPILLESRDVLGGKVAAWKDEDGDWYE
TGLHIFFGAYPNVQNLFGELGINDRLQWKEHSMIFAMPSKPGEFSRFDFPEVLPAPLNGIWAILRNNEMLTWPE
KVKFAIGLLPAMLGGQAYVEAQDGLSVKEWMIKQGVPERVTDEVFIAMSKALNFINPDELSMQCILIALNRFLQE
KNGSKMAFLDGNPPERLCMPIVDHIQSLGGEVHLNSRIQKIELNDDGTVKSFILNNGKVMEGDAYVFATPVDILK
LLLPDNWKGIPYFQRLDKLVGVPVINVHIWFDRKLKNTYDHLLFSRSPLLSVYADMSVTCKEYYDPNRSMLELV

FAPAEEWISRSDEDIIEATMSELAKLFPDEISADQSKAKIVKYHVVKTPRSVYKTVPNCEPCRPVQRSPIEGFYLA
GDYTKQKYLASMEGAVLSGKLCAQAIVQDCELLAGRGQKRMAKASVV
>Mu_g03878.t1 hypothetical protein PHAVU_006G173700g [Phaseolus vulgaris]
MSQPTVILATASYDHTIRFWEAKSGRCYRTIQYPDSQVNRLEITPDKRYLAAAGNPHIRLFDVNSNSPQPVMSYE
SHTNNVMAVGFQCDGNWMYSGSEDGTVKIWDLRAAGCQREYESRAAVNTVVLHPNQTELISGDQNGNIRVWD
LTANSCSCELVPEVDTAVRSLTVMWDGSLVVAANNHGTCYVWRLLRGTQTMTNFEPLHKLQAHKGYILKCLLS
PEFCEPHRYLATASSDHTVKIWNVDGFTLEKTLIGHQRWVWDCVFSVDGAYLITASSDTTARLWSMSTGEDIKV
YQGHHKATICCALHDGAEPATS
>Mu_g04175.t1 photosystem I reaction center subunit IV, chloroplastic [Vigna radiata var. radiata]
MATSNMASAASGFVLSLNVAASTSNSPASRVIFPTKNNAGSRLVVRASDEAAASAPATATPPAEGEAKPKPPPIG
PKRGAKVKILRKESYWYKGTGSVVAVDQDPKTRYPVVVRFNKVNYANVSTNNYALDEIVEVE
>Mu_g04176.t1 photosystem I reaction center subunit IV, chloroplastic [Vigna radiata var. radiata]
MASAASGFVLSLNVNVAAATTNSSPSRVMFPSKNTGSRLVVRASDEAAPAPATATPPAEAEAKPKPPPIGPKRG
AKVKILRKESYWYKGTGSVVAVDQDPKTRYPVVVRFNKVNYANVSTNNYALDEIEEVE
>Mu_g04646.t1 PREDICTED: linoleate 9S-lipoxygenase-like [Vigna angularis]
MFPFGGKTQKVKGTVVLMRKNVLDINSITNPATVVDSVFDIFGSVIDTVTAFATSISIQLISATKADGQGKGKVGS
ATKLRGQISLPTLGAKEEAYDVNFDWNTDFGIPGAFYIKNFMQNEFYLKSVTIEDIPNQGTIHFVCNSWIYNSTK
YKTDRIFFANNTYLPSDTPAPLVKYREEELKTVRGDGTGERQEWDRIYDYDVYNDLGDPDKSADLARPVLGGSV
LPYPRRGRTGRGKTRKDPNSEKPSAFVYLPRDEAFGHLKSSDFLADALKSVSQDVLPVLTDAFDGSLLTLEFDNF
EEVRKLYEGGVTLPTNFLSKYAPIPVLKEILRTDGEQFLKYPPPKVMQVDKSAWMTDEEFARETIAGVNPNVIKI
LEEFPPRSKLDIQTYGDHTSIITKEHLEPNLGGLTVEQAIENKKLFILDHHDYLIPYLRRINSSNTKTYATRTIFFLK
DDGTLTPLAIELSKPHGQGDEYGPLSEVYVPAYDGVEAYIWLLAKAYVVVNDSCYHQIVSHWLNTHAVVEPFVIA
TNRQLSVVHPVYKLLFPHFRDTMNINSLARKSLVNADGIIEKTFLWSRYSLEMSALNYKDWAFPDQALPNDLVK
RGVAVKDPSAPHGVKLLIEDYPYASDGLEIWDAIKSWVQEYVSFYYKSDEVLQKDPELQAWWKELVQVGHGDL
KDKPWWQKMQTREELVEVSTTLIWIASALHAAVNFGQYPYGGLILNRPTLSRRFMPVKGSAEYDALAKNPEKE
FLRTITGKKETLIDLTVIEILSRHASDEFYLGERDGGDYWTSDTVPLEAFKRFGKKLAEIEAKLVEKNNDETLRNR
TGPAKMPYTLLFPSSEEGLTFRGIPNSISI
>Mu_g05224.t1 hypothetical protein VIGAN_01205600 [Vigna angularis var. angularis]
MTPGYEYDINDLHQEAQARWLKPAEVMYILQNHEKFQLTQEPPQQPTSGSLFLFNKRVLRFFRKDGHNWRKK
RDGRTVGEAHERLKVGNVEALNCYYAHGEQNPSFQRRSYWMLDPEYEHIVLVHYRNTSEGKLSSGAGAQLSPS
YSSAFSQSPSPYSNQNPGSTSLPGDSYDPNQSFSSPASTEVTSDIFVLNNKMDHVDGTDAESGTSSELEVTQALRR
LEVQLSLNEDSFEDIAPFCNKLETANDSNPQHNQRVISNQEQSAAFSGPDDQGLFYEEYNGRQGDGGECYRELID
HGYPDGNENALWTGVLESCKSSTAVNLPPKNVNMTAENENSVTSRRVLAPVSNLENSHWLNFNSINSQSSVFS
PPQGVDEVKFPAYSSMVETRVINSDYYETLFDQSQIVEPLNADSSLTVAQKQKFTIKTISPEWGYATETTKVIIVG
SFLCHPSDSTWACMLGDVELPVEIVQDGVICCEAPPHLPGKVTLCITSGNRESCSEVREFEYRDKAYSCTRCTQSE
IEATRSPEELLLLVRLGQMLLSASTIQNDNIESGIPLIKQKADDDSWSHIIEALLVGSGTSTSTIDWLLEELLKDKL
QQWLSCRSQERDEETGCSLSRKEQGIIHMVAGLGFEWALNPILSCGVNINFRDINGWTALHWAARFGREKMVA
SLVASGASAGAVTDPTAQDPAGKTAASIAASKGNKGLAGYLSEVAVTSHLSSLVLEESELSKDSAELQADMTVTS
VSKENLGANEDQASLKDTLAAVRNVTQAAARIQSAFRSHSFRKRRAREAAAGIDRYGTSVGGIGSIPEISAMSKLA
FRNSREHNSAALSIQKKYRGWKGRKDFLSLRQKVVKIQAHVRGYQVRKHYKVIWAVGILDKVVLRWRRKGAGL
RGFRQEMDVTENEDEDILKVFRKQKVDVEIEKAVSRVLSMVDSPDARQQYHRMLEKYRQAKAELAGTSDEASS
TTSVGNALFMEDDFYPFP
>Mu_g05418.t1 PREDICTED: linoleate 9S-lipoxygenase [Vigna angularis]
MFGILGNKGHKIKGTLVIMRKNVLDINSLTSVSGVVGTGINIIGGVVDTVTALASHISIQLISATKTDAHGKGKVGK
ATNLRGQISLPTLGAGQDAYDVNFEWDSDFGIPGAFYIKNYMQVEFYLKSLVLEDIPNHGTIHFVCNSWVYNSKV
YKTDRIFFANNTYLPSETPAALVKYREEELNTVRGDGTGERKEYDRIYDYDVYNDLGDPDSSADLARPVLGGSVL
PYPRRGRTGRGKTRKDPNSEKQGSFVYLPRDEAFGHLKSSDFLTYGLKSVAQDVLPVLTDAFDGNVLSLEFDNF
AEVRKLYEGGITLPTNFLSKIAPLPVIKEIFRTDGEQFLKYPPPKVMQVDKSAWMTDEEFARETIAGVNPNVIKIL
EEFPPRSKLDTQAYGDHTSIITKEHLEPNLGGLTVEQALSSKKLFILDHHDYLIPYLRKINQTTTKTYATRTVLFL
KSDGTLAPLAIELSKPHPQGDEHGPVSEVYVPSSEGVEAYIWLLAKAYVVVNDSCYHQLVSHWLNTHAVVEPFII
ATNRQLSVVHPIYKLLFPHYRDTMNINSLARKSLVNANGIIEKTFLWGRYSLELSAVIYKDWSLYDQALPNDLVK
RGVAVKDPSAAHGLKLVIEDYPYATDGLEIWEAIKSWVEEYVAFYYKTDAELQQDPELKAWWKELVEVGHGDL
KDKPWWPKMQSREDLVQVSTTLIWIASALHAAVNFGQYPYGGLILNRPTISRRFMPEKGSAEYVALGKNPEKEF
LKTITGKKETLIDLTVIEILSRHTSDEFYLGERDGGDYWTSDAGPLEAFKRFGKKLAEIETKLVQKNNDETLRNR
TGPAKMPYTVLYPSSEEGLTFRGIPNSISI
>Mu_g05465.t1 PREDICTED: pectinesterase-like [Vigna angularis]
MLPHLPSILSAVYVLLLFSSTQFSITAEATGTLEQTHPHFHVASSTCEGTLYPDLCVSTLASFPDLTSKTVPQMICS
ALNHTIYEVTLSSSNCSGLRKKLPRLSKLERRALDDCLDLFQETVEELKTTVADLSHTTIASKRYHDSQTLLSGAM
TNLFTCLDGFAYSKGHVRERIEKGLVQISHHVSNSLALLKKVPRTKNQSKNEIFPEYGKMKDGFPSWVSPKDRKL

LQAPVNQTKFDLVVAKDGTGNFTTISEAVAAAPNSSATRFVIHIKAGAYFENVEVIRKKTNLMWVGDGIGKTVV
KASRNVVDGWTTFQSATVAVVGDGFIAKGITFENSAGPSKHQAVALRSGADFSAFYQCSFVAYQDTLYVHSLRQ
FYRECDVYGTVDFIFGNAAVVLQNCNLYARKPDPNQKNLFTAQGREDPNQNTGISIINCKVAAAADLIPVKSEFK
NYLGRPWKKYSRTVYLNSLMEDLIDPAGWLEWDGTFALDTLYYGEYNNRGPGANTSRRVTWPGYRVITNSTE
ASQFTVANFIQGNEWLNSYAIPFFSGLS
>Mu_g05719.t1 PREDICTED: cellulose synthase-like protein G1 [Vigna angularis]
MTTFTYHVETVQPWLVLSRLHIFIHLLAVLALCYYRISHLLLQPPTLPWILITVAELVLCMLWFFNQAFRWHPVS
RTSLTEKLPKEDKLPGLDIFVCTLDPEKEPTVQVMDTVISSIAMDYPSDKLAVYLSDDGGCPVTLYAMREAGEFA
REWVPFCRKYGVKLRCPKVFFSPMGEDEHLLRDEDFKTQCQLIKGKYEKMQKNIEKFSSDPKNLGIVSDRAPRIE
IINDQPGLPLVVYVSRERRPSLPHKFKGGALNTLLRVSGFLSNAPYFLVVDCDMFCNDPTSAKQAMCFFLDPQTS
KTTAFVQFPQMFHNLSKKDIYDSQTRTAFKTMWQGMDGLRGPGLSGSGNYLSRSALLFGSPDQKNDYLLDAQK
YFGNSTGFIESLKVIRGQKTTKTNISREEKLREALAVSSCSYENNTKWGTEVGFSYAILLESSATGYILHTRGWRS
VYLYPKTPCFLGCAPTDIKEGMLQLVKWLSELLLLGFSKYSPFTHGFSSMSILHSFTYCFITMSSLYAIVFILYGMV
PQICLLQGISVFPKVTDPWFAVFAFVYVSTQVQHLVEVLSGEGSVAMWWDEQRIWILKSVMSIFAIIDAVKKGLG
LNKVKFALSNKAIDKEKVKKYEEGRFDFQGAALFMAPLVVLLITNIVGFFGGIWRVVHVKDFEEMFGQLFLVTY
VMVLSYPILEAIINMKSKSG
>Mu_g06091.t1 PREDICTED: geranylgeranyl pyrophosphate synthase 7, chloroplastic [Vigna angularis]
MVSSCASLKSSMLNTPSPPFLTPLFHTPTTLPNTTKLPTPKLSSSFHSLSVSAVPTKVDTTVRIAEIEDSPNFDFKV
YMLHKAHTVNQALDAAIALRDPEKIHKAMRYSLLAGGKRVRPILCIAACELVGGAEVTAIPAACAVEMIHTMSLI
HDDLPCMDNDDLRRGKPTNHKVFGEDVAVLAGDALLAFAFEHVATATEGVPPACVVRAIGELAKSIGGEGLVA
GQVVDLDSEGVSNVGLERLEFIHAHKTAALLEAAVVLGAIVGGGSDEEIEKLRKFARCIGLLFQVVDDILDVTKSS
EELGKTAGKDLVADKVTYPKLLGIHKSKEFADELLQEAKEQLFGFDPRKAVPLFALANYIAYRQN
>Mu_g06317.t1 ribulose bisphosphate carboxylase small chain 1, chloroplastic-like
MASSMISSPAITTVNRAGAGMVAPYTGLKSLGGFPTRKTNNDITSVANNGGRAQCIQVWPTVGKKKFETLSYLP
DLTDAQLAKEVEYLLRNGWIPCLEFELEHGFVYREHNRSPGYYDGRYWTMWKLPMFGCTDASQVLKELEEAR
TAHPNGFVRIIGFDNVRQVQCISFIAYKPPNY
>Mu_g06318.t1 ribulose bisphosphate carboxylase small chain 1, chloroplastic-like
MASSMISSPAITTVNRAGAGMVAPYTGLKSLGGFPTRKTNNDITSVANNGGRAQCIQVWPTVGKKKFETLSYLP
DLTDAQLAKEVEYLLRNGWIPCLEFELEHGFVYREHNRSPGYYDGRYWTMWKLPMFGCTDASQVLKELEEAR
TAHPNGFVRIIGFDNVRQVQCISFIAYKPPNY
>Mu_g06319.t1 ribulose bisphosphate carboxylase small chain 1, chloroplastic-like
MASSMISSPAITTVNRAGAGMVAPYTGLKSLGGFPTRKTNNDITSVANNGGRAQCIQVWPTVGKKKFETLSYLP
DLTDAQLAKEVEYLLRNGWIPCLEFELEHGFVYREHNRSPGYYDGRYWTMWKLPMFGCTDASQVLKELEEAR
TAHPNGFVRIIGFDNVRQVQCISFIAYKPPNY
>Mu_g06835.t1 protein DJ-1 homolog D [Vigna radiata var. radiata]
MASKRVLLLCGDFMEDYEAMVPFQALQAFGVAVDAICPGKKAGDVCRTAVHQLSGAQTYTETVGHNFALNATF
DEVDAASYDGLWLPGGRAPEYLAHIPGVLELVTKFVGLGKQIASICHGQLILAAAAVVVKGRKCTAFPAVKPVLV
AAGAHWVEPDTMAATVVDGNLITAATYDGHPEFIRHFVKALGGAISGFDRKILFICGDYMEDYEVKVPFQSLQA
LGCHVDAVCPSKKAGDTCPTAIHDFEGDQTYSEKPGHAFSLTATFDDVNPSSYDALVIPGGRAPEYLALNESVIA
LVKHFFENKKPVASICHGQQILSAAGVLKGRKCTAYPAVKLNVVLSGATWLEPDPISRCFTDGNLVTGAAWPGH
PEFISQLMALLGIKVSF
>Mu_g07378.t1 hypothetical protein PHAVU_007G214200g [Phaseolus vulgaris]
MATIAGGVSLSSPSRVFANAKGSESAQKAQATIRFPLFRPMQKLPGLVGVAKAWPIRATPEKISEKIEESIKSAEE
TCSGGGADAECAAAWDEVEELSAAASHARDAQKQSDPLETYCKDNPETDECRTYDN
>Mu_g07463.t1 PREDICTED: uncharacterized protein At5g01610-like [Vigna angularis]
MSSSIRLLCILVVCLTASCALSQAQKLSAYDILAEYGFPVGLLPKGAIGYSLNRETGQFAVYFDGACSFVIESYTLKY
KSTITGVISNGRLYNLKGVTVKILLLWLNIVEVTRQGDDIYFSVGIASADFGVENFLESPQCGCGFDCNKLPLNGD
VSSI
>Mu_g07476.t1 actin-depolymerizing factor 2 isoform X2 [Vigna radiata var. radiata]
MLTANYLLTCGILQANAASGMAVHDDCKLKFLELKAKRTYRYIVYKIEEKQKQVIVEKLGDPANGYEDFTASLPA
DECRYAVYDFDFVTEENCQKSRIFFIAWSPDTSRVRSKMIYASSKDRFKRELDGIQVELQATDPTEMGLDVFKSR
VN
>Mu_g08128.t1 hypothetical protein PHAVU_010G134900g [Phaseolus vulgaris]
MFGILGGKGQKIKGTVVLMSKNVLDFNEIVSTANGGVLGVAGGLFGAANKVVGGIVDGATSVFSRNIALQLISATK
TDGLGNGKVGKQTFLEKHLPTLPTLGDRQDAFNIYFEWDESFGIPGAFYIKNFMQAEFFLVSVTLEDIPNHGTIR
FVCNSWVYNAKEYKKDRIFFANKTYIPNETPTPLVKYRKEELENLRGDGTGQRKESDRIYDYDVYNDLGNPDKS
ADLARPVLGGSSAYPYPRRGRTGRKATKRDPKSETPTSDTYIPRDESFGHLKSSDFLTYGIKSLSQNVIPQFQSAF
GLNAEFDKFDDVRGLFEGGLYLPTDVISKISPLPVLKEIFRTDGEQALKFPPPQVIKVSKSAWMTDEEFGREMLA
GVNPCLIQRLQEFPPKSKLDSTVYGDQTSTITKEHLEINLGGLTVEEASDALNGNRLFILDHHDAFLPYLRKINDL

PTAKSYATRTILLLKDDGTLKPLAIELSLPHPRGDEFGAVSRVILPADQGAESTIWLLAKAYVVVNDSCYHQLMS
HWLNTHATIEPFVIATNRHLSVLHPIYKLLLPHYRDTMNINGLARQSLINAGGIIEQSFLPGPFSVEMSSAVYKSW
VFTDQALPADLIKRGMAIEDPSSPYGLRLVIDDYPYAVDGLEIWSAIQTWVKDYVSLYYATDDAIKKDTELQTW
WKEAVEKGHGDLKDKPWWPKLNTLQDLIHICSIIIWTASALHAAVNFGQYPYGGYILNRPTLTRRLLPEPGTKE
YGELSSNHQKAYLRTITGKVEALVDLSVIEILSRHASDEVYLGQRDNPNWTDDTKALQDFQKFGNKLKEIENKIS
GRNNNSSLRNRVGPVQMPYTVLLPTGGEGLTFRGIPNSISI
>Mu_g08421.t1 NAC transcription factor 29 [Vigna radiata var. radiata]
MEGRTSSVLPPGFRFHPTDEELIVYYLCNQATSKPCPASIIPEVDIYKFDPWELPEKTDFGENEWYFFSPRERKYP
NGVRPNRATVSGYWKATGTDKAIYSGSKHIGVKKALVFYKGKPPKGLKTDWIMHEYRLIGSRRQANRQIGSMRL
DDWVLCRIYKKKNIGKSLEAKEDYPIAQINLTPPNNFTEQEVVKFPRTSSLTHLLDMDYLGPISHILSDASYNSTF
DFQLNTANGGIDPFVKPQPLEIPNNPYYAADSGKYHVKQNSTINPTIFVNQVYDQRG
>Mu_g08739.t1 hypothetical protein PHAVU_008G016200g [Phaseolus vulgaris]
MEVEIKLRLPDSATHEKLSNILAPFHTKTLIQENIFFDGKNKELTSNLAVLRIRFYNLEHCVLSLKAKPVISAGISR
MEEHEEPFDPALGRACIAEPWRLLSVNSSMVLKRVKDEYGFGANGVVCLGGFRNVRAVHEWKGLKLELDETNY
DFGTSYELECESADPERHKKLLEEFLQENGISYSYSKVSKFAVFQSRKLSE
>Mu_g09334.t1 PREDICTED: 2-methylene-furan-3-one reductase-like [Glycine max]
MASTPSIPSHIKAWAYSEYGNIEETLKFESNIPIPDIKEDQVLIKVVAAALNPVDYMRANGFSKDTDSPLPTVPGY
DVAGVVVRVGSEVRKFKVGDEVYGEINEDALDNPKTIGTLAEYTAVEEKVLAHKPSNLSFIEAASLPLAIITAYQG
LERVEFSAGKSILVIGGAGGVGSLVIQLAKHVFGASKVAATASTAKLDLLRNLGADLAIDYTKENIEDLEEKFDVV
YDTVGPSEIEKALKAVKESGKVVTIVAFGNAAAISIIGISDSAVLEKLQPYLESGKVKPLLDPKSPFSFSQTVEAFAY
LKTNRAIGKVVIHPIP
>Mu_g09335.t1 2-methylene-furan-3-one reductase [Vigna radiata var. radiata]
MANTPSIPSHIKAWAYSEYGNIEETLKFESNIPIPDIKEDQVLIKVVAAALNPVDYKRALGYFKDTDSPLPTVPGF
DVAGVVVRVGSEVKKFKVGDEVYGDINEYALDNPKTIGTLAEYTAAEEKVLALKPSNLSFIEAASLPLAIITAYQG
LERVEFSAGKSILVIGGAGGVGSLVIQLAKHVFGASKVAATASTAKLDLLRSLGADLAIDYTKENIEDLEEKFDVVY
DAVGPSEIEKALKAVKESGKVLSIATFGNPAVISITLISDSAVLEKLQPYLESGKVKPLLDPKGPFSFSQTVEAFAYL
KTNRAIGKVVIHPIP
>Mu_g09391.t1 hypothetical protein PHAVU_002G032600g [Phaseolus vulgaris]
MGTIGRAFYAVGFWIRETGQALDRLGSRLQGNYLFQEQLSRHRPLMNLFDKGPSVHKDAFVAPSASLLGDVHVG
PASSIWYGCVLRGDVNGIVIGSGTNIQDNSLVHVAKSNLSGKVLPTIIGDNVTVGHSAVLQGCTVEDEAFIGMGAT
LLDGVYVEKHAMVAAGALVRQNTRIPYGEVWGGNPARFLRKLTEDEMTFFSQSALNYSNLAQAHATENAKELE
KTEFVKVLNKKFARPGEEYDSVLGGGQETPAELNLQDNVLLDKAPKA
>Mu_g09763.t1 ascorbate peroxidase [Macrotyloma uniflorum]
MVLFLSNQDKPHPPPEGRLPDATKGSDHLRDVFGKAMGLSDQDIVALSGGHTLGAAHKERSGFEGPWTSNPLIF
DNSYFKELLSGDKEGLLKLPSDKALLSDPVFRPLVEKYAADEDAFFADYAVAHQKLSELGFAEAMGKSYPTVSA
DYQKAVEKAKKRLRGFIAEKRCAPLMLRLAWHSAGTYDVSTKTGGPFGTIKHPSELAHGANNGLDIAVRLLEPL
KAEFPILSYADFYQLAGVVAVEVTGGPEVPFHPGREDKPHPPPEGRLPDATKGSDHLRDVFGKAMGLSDQDIVA
LSGGHTLGAAHKERSGFEGPWTSNPLIFDNSYFKELLSGDKEGLLKLPSDKALLSDPVFRPLVEKYAADEDAFFA
DYAVAHQKLSELGFAEAMGKSYPTVSADYQKAVEKAKKRLRGFIAEKRCAPLMLRLAWHSAGTYDVSTKTGGP
FGTIKHPSELAHGANNGLDIAVRLLEPLKAEFPILSYADFYQLAGVVAVEVTGGPEVPFHPGREV
>Mu_g09994.t1 hypothetical protein PHAVU_003G094500g [Phaseolus vulgaris]
MYTSRKKIHKDKDAEPTEFEESVGQALFDLENTNNELKSDLKDLYINSAVQIDVSGNRKAVVIHVPYRLRKGFRK
IHVRLVRELEKKFSGKDVVLIATRRILRPPKKGSAVQRPRSRTLTAVHEAMLEDIVLPAEIVGKRVRFRIDGSKIM
KVFLDPKERNNTEYKLETFAAVYRKFSGKDVVFDYPTTEA
>Mu_g10380.t1 3'-hydroxy-N-methyl-(S)-coclaurine 4'-O-methyltransferase [Glycine soja]
MTCFTDSVAIKCVVELRIADIIDRYGKPLSLSQIVENIEDAPSPDASLLHRVMRVMVRRKIFSAEQSETGETLYGLT
RASKWILRDTKMTLAPMLLLENHPIHLNPAHYISDIVREGTKNGTAFFRCHGHEQFEMTGLDSEYNRLFNEGM
VCTARVVSKAVIKGYKDGFNQIESLVDVGGGIGGSLSEIVRAYPHIKAINFDLPHVVATAPKFDGITHVGGDMFVS
VPSADAIYMKWILHDWSDEHCIKILKNCRKAIPEKTGKVIIVDHVLRPEGNELFTDVGIAFDMMLLAHNAGGKE
RTEENWKWLFKETGFARYNIIKINALPSIIEAFPI
>Mu_g10516.t1 elongation factor 1-delta [Vigna radiata var. radiata]
MAVTFYELSSASGLKKLDEYLLSRSYITGYQASKDDLTVYAAFPTVPSADYVNVSRWYKHIDALLRISGVSGEGSG
VTVEGSLVAEPVATPPAADTKAAAAEDDDDDDVDLFGEETEEEKKAAEERAAAVKASGKKKESGKSSVLMDVK
PWDDETDMKKLEEAVRSVSMEGLLWGASKLVPVGYGIKKLQIMLTIVDDLVSVDNLIEEYLTVEPINEYVQSCDI
VAFNKICKYF
>Mu_g11074.t1 PREDICTED: chlorophyll a-b binding protein of LHCII type 1-like [Vigna angularis]
MAASTMALSSSSLAGQAIKLSPSTPDLSTGRISMRKTASKSVSSGSPWYGPDRVKYLGPFSGEAPSYLTGEFPGDY
GWDTAGLSADPETFARNRELEVIHSRWAMLGALGCVFPELLARNGVKFGEAVWFKAGSQIFSEGGLDYLGNPSL

IHAQSILAIWATQVILMGAVEGYRIAGGPLGEVTDPIYPGGSFDPLGLADDPEAFAELKVKELKNGRLAMFSMFG
FFVQAIVTGKGPLENLADHIADPVNNNAWAYATNFVPGK
>Mu_g11347.t1 PREDICTED: heat shock cognate 70 kDa protein 2-like [Vigna angularis]
MAGKGEGPAIGIDLGTTYSCVGVWQHDRVEIIANDQGNRTTPSYVGFTDTERLIGDAAKNQVAMNPINTVFDAK
RLIGRRFTDAPVQSDIKLWPFKVIAGPADKPTIVVNYKGEEKQFAAEEISSMVLMKMREIAEAYLGSTVKNAVVT
VPAYFNDSQRQATKDAGVIAGLNVMRIINEPTAAAIAYGLDKKATSVGEKNVLIFDLGGGTFDVSLLTIEEGIFEV
KATAGDTHLGGEDFDNRMVNHFVQEFKRKNKKDISGNPRALRRLRTACERAKRTLSSTAQTTIEIDSLYEGIDF
YSTITRARFEELNMDLFRKCMEPVEKCLRDAKMDKRSVDDVVLVGGSTRIPKVQQLLQDFFNGKELCKSINPDE
AVAYGAAVQAAILSGEGNEKVQDLLLLDVTPLSLGLETAGGVMTVLIPRNTTIPTKKEQVFSTYSDNQPGVLIQVF
EGERARTRDNNLLGKFELSGIPPAPRGVPQITVCFDIDANGILNVSAEDKTTGQKNKITITNDKGRLSKEDIEKMV
QEAEKYKSEDEDHKKKVEAKNALENYAYNMRNTVKDDKIGGKLDPADKKKIEDAIEQAIQWLDSNQLAEADEF
EDKMKELESICNPIIAKMYQGGAGPDMGGAGPAEDGYTPSGGSGAGPKIEEVD
>Mu_g11647.t1 actin-depolymerizing factor 2 [Vigna radiata var. radiata]
MANAASGMAVHDDCKLRFLELKAKRTHRFIVFKIEEQQKQVIVEKLGEPAQGYEDFAGSLPADECRYAVYDFEY
MTEGNVPKSRIFFIAWSPDTSRVRSKMIYASSKDRFKRELDGIQVELQATDPTEMGLDVFKSRAN
>Mu_g11719.t1 RING-H2 finger protein ATL74 isoform X1 [Vigna radiata var. radiata]
MAPHHRFLLDTNSSTPAPSNMNRTRETFTGDANFDTNMVIILAALLCALICALGLNSIVRCALRCSRRFAFETPE
ETAARLVAKGLKKSALHQIPIVVYGSGSVGIAATDCPICLGEFVDGEKVRVLPKCNHGFHVRCIDTWLLSHSSCPN
CRQSLLEQPTISGAVAAGTSNHAGNALGGHEHVSLAVAVEEVG
>Mu_g11909.t1 histone H2A [Sesamum indicum]
METGGKIKKGAGGRKGGGSKKKSVTKSIKAGLQFPVGRIGRYLKKGRYSQRVGAGAPVYLAAVLEYLAAEVLELA
GNAARDNKNRIIPRHVLLAVRNDEELGKLLAGVTIAQVVCCLTLTLFCCLRRLRRLPRSPNLHPRPPSHPRRLRL
YSI
>Mu_g12232.t1 40S ribosomal protein S8 [Vigna radiata var. radiata]
MGISRDSMHKRRATGGKKKAWRKKRKYELGRQSANTKLSSNKTIRRIRVRGGNVKWRALRLDTGNYSWGSEA
VTRKTRILDVVYNASNNELVRTQTLVKSAIVQVDAAPFKQWYLQHYGVEIGRKKKAATKKDSAEEGEAAAEETK
KSNHVQRKLEKRQKERKLDSHIEEQFGGGRLLACISSRPGQCGRADGYILEGKELEFYMKKLQKKKGKGAAA
>Mu_g12512.t1 Glycine-rich RNA-binding protein GRP2A [Cajanus cajan]
MASADVEYRCFVGGLAWATDSDALEKAFSQYGEIVESKVINDRETGRSRGFGFVTFATEQAMKDAIQGMNGHD
LDGRNITVNEAQTXXXXXXXXXXXXXXXXXXXXXXXXXXXXXXXXXXXYGGRRDGGFNRNGGGGGGYGGGRDRGG
YGDSGSRYSRGGEGGGSDGNWRS
>Mu_g12641.t1 hypothetical protein PHAVU_007G250500g [Phaseolus vulgaris]
METKPTAMETKPTEISSGKMFGGYNKRFKHFSPTLGCSMNFHIYFPPSPSPSHKFPVLYWLSGLTCTDENFIFKS
GAQRAAAAEGIALVAPDTSPRGLNVEGEADSWDFGVGAGFYLNATQEKWKNWRMYDYVVKELPKLLSDNFPQ
LETSKASIFGHSMGGHGALTIYLKNQDKYKSVSAFAPVANPINCPWGHKAFTNYLGDNKSDWEEYDATSLVTKF
PNVSSSILIDQGEDDKFLTDQQLLPHKFEEACRKANVPLLLRFQPGYDHGYYFIATFIDDHIQHHAQALRLN
>Mu_g13584.t1 PREDICTED: photosystem II reaction center W protein, chloroplastic-like [Vigna angularis]
MATIIAGTPTTSISRAGLVLKRPFAASPSTVLGLPAMGRAGKVRCSMEEKPSVQESSSNLGMGASLLAAACAATM
SSPAMALVDERLSTEGTGLPFGLSNNLLGWILLGVFALVWALFFVYTNTLEEDEESGMSL
>Mu_g13878.t1 hypothetical protein PHAVU_009G227100g [Phaseolus vulgaris]
MASFSVVWCTVAVLLCAVAGGSWLDDANPIRMVSDLEADVIQVIGQCRSAVTFARFVGKFGKTYRNEEEMKQR
FEIFSQNLRFIRSTNKKRLPYTLAVNHFADWTWEEFKRHRLGAAQNCSATHKGNHKLTDAVLPPTKDWRQENI
VSSVKDQGSCGSCWTFSTTGALEAAYAQAHGKSISLSEQQLVDCAGNFNNFGCNGGLPSQAFEYIKYNGGLETEE
AYPYTAKDGVCKFSAENVAIQVLDSVNITLGAEDELKHAVAFVRPVSVAFQVVNGFRFYEKGVYTSEICGSTSQD
VNHAVLAVGYGVENGVPYWLIKNSWGANWGDSGYFKMELGKNMCGVATCASYPVVA
>Mu_g13956.t1 PREDICTED: methylenetetrahydrofolate reductase 2 [Glycine max]
MKIIEKIHAAGADPNRVVFSFEFFPPKTEDGVDNLFERMDRMVAHNPSFCDITWGAGGTTADLTLEIANRMQNI
VCVETMMHLTCTNMPVEKIDHALHTIKSNGLQNVLALRGDPPHGQDKFVQVEGGFACARDLVKHIRAKYGDYF
GITVAGYPEAHPDVIGSDGLATTEGYQNDLEYLKSKVDAGADLIVTQLFYDTDTFLKFVNDCRQIGITCPIVPGIM
PINNYKGFLRMTGFCKTKIPADIMSALEPIKDNEEAVKAYGIHLGTEMCRKILANGIKTLHLYTLNMEKSALAIL
MNLGLIEESKISRSLPWRRPANVFRVKEDVRPIFWANRPKSYISRTIGWDQYPHGRWSDSCNPSYGALTDYQFM
RPRARDKKLIEEWAVPLKTVEDIYERFRMYCVGKLRSNPWSELDGLQPETKIINEQLERINTKGFLTINSQPAVN
GEKSDSPTVGWGGPGGYVYQKAYVEFFCSKEKLDALIDKCKDRTSLTYMAVNKDGSWKSNVGQIDVNAVTWGV
FPAKEIIQPTIVDPVSFNVWKDEAFEIWSRGWASLYPEGDASRKLVEEVAGSYFLVSLVDNDYVNGDLFAAFVDF
>Mu_g14048.t1 hypothetical protein PHAVU_009G216400g [Phaseolus vulgaris]
MATTVMTTLPQFSGLRPQFSAAPLQNLVAVQPMRRKGKGALGARCDYIGSSTNLIMVASTSLMLFAGRFGLAPS
ANRKATAGLKLEVRDSGLQTGDPAGFTLADTLACGAVGHIIGVGVVLGLKNIGAL
>Mu_g14117.t1 hypothetical protein PHAVU_009G092300g [Phaseolus vulgaris]

MAQESATPPVPFDFELLVGDPEHLRTVKASSSSTDPWIEPERLKLRHRIGRGLYGDVWLATHHQSTEDYDEYHE
VAAKMLPPIREEHMKTALEKFCELYFQCQGVARVCWLLGISILNGRICIIMNFYEGSVGDKMARLREGRISLPDVL
RYGIDLAQGILELHSKGILVLNLKPSNVLLDDADQAIVGDVGIPNLLFGSSFLSSDTANRLGTPNYMAPEQWQPEV
RGPVSFETDSWGFGCTIVEMLIGNQPWYGCPVGEIYQSVVEKYEKPQIPSGLPSSVENVLSGCFEYDLRNRPSMV
DILAVFRSSWNAVVNDGGWRYLGIHKTIAKSSSTGYTQWSLSKDHLQVGDTVRSRKPSNSCNPQNMEVPEGNV
VGNADHGFVLVRLHGVHDPVRIHASTLERVTNGLGAGDWVRMKEEDDKHSPVGILHSINRDGRVTVGFIGLRTL
WKGNSSELEMAESYCVGQFIRLKPNVLSPRFEWSRKRGGAWATGKISWILPNGCLVVKFPGMLNFWDAPSAFL
ADPSEVDVVNFKNCPKMIEKYQHVEDHHWAVRPALIAFGLLTTMKLGMSIGNKIGRNMNVNAMENQTNYTDT
QNATSPTPTWTSSVANILFREGVNLPTAR
>Mu_g14118.t1 PREDICTED: ATP synthase subunit b', chloroplastic [Vigna angularis]
MNYVGDNFSYSGLPSETLNLSIIKLDNSSFRVEVANEATVAELKQAVEAIFAHVPLKGPGKILCFISCAIYLILAVTR
VSSQRNEVLVSNNTGVAMANMIMASTKPLVPVCNNSRSPSSKLPILQISFPKVPTLKLKLPISKPQMLSLLGGIAP
LVLARPSLAAEIEKAALFDFNLTLPIIMVEFLLLMVALDKIWFTPLGKFMDERDAAIREKLSSVKDTSEEVKQLEE
KANAVMVAARAEIAAALNAMKAETQAEVEQKIAEGRKKVEAELQEALANLERQKEETVKALDSQIADLSQEIVK
KVLPIS
>Mu_g14829.t1 PREDICTED: probable zinc metalloprotease EGY1, chloroplastic [Vigna angularis]
MGTLTSCSFMPLNSEFRSNPVRRAFRERIQCHKLTRLRGTSCFFSRVQVPKWGKSKHANYGSLRCFGTKDSDSD
KDNVGAGEESSGDDSKSNVTTTMPEEERGFNSEKSTPPSTSHRSSLSSLGPAYNTFQVDSFKLIELLGPEKVDPA
DVKLIKDKLFGYSTFWVTKEEPFGELGEGTLFIGNLRGNREDVFAKLQNQLVEVTGDKYNLFMLEEPNSESPDP
RGGPRVSFGLLRKEVSDPGPTTLWQYVIALLLFLLTIGSSVELGIASQINRLPPEVVKYFTDPDAIEAPDMELLFPF
VDSALPLAYGVLGVLLFHEVGHFLAAFPKQVKLSIPFFIPNITLGSFGAITQFKSILPDRSTKVDVSLAGPFAGAVLS
FSMFAVGLLLSSNPDTTGDLVQVPSLLFQGSLLLGLISRATLGYAAMHAATVPIHPLVIAGWCGVTIQAFNMLPV
GCLDGGRAVQGAFGKNALVGFGLTTYTLLGLGVLGGPLSLPWGLYVLLCQRTPEKPCLNDVTEVGTWRKALVA
AAIFLVVLTLVPVGDELAEELGIGLVTAF
>Mu_g14985.t1 PREDICTED: 40S ribosomal protein S8-like [Vigna angularis]
MGISRDSMHKRRATGGKKKAWRKKRKYELGRQPANTKLSSNKTVRRIRVRGGNVKWRALRLDTGNYSWGSE
AVTRKTRILDVVYNASNNELVRTQTLVKSAIVQVDAAPFKQWYLQHYGVDIGRKKKTAAKKDSAEEGEAAAAE
EAKKSNHVQRKLEKRQKDRKLDAHIEEQFGGGRLLACISSRPGQCGRSDGYILEGKELEFYMKKLQKKKGKGAA
>Mu_g15188.t1 PREDICTED: chlorophyll a-b binding protein 3, chloroplastic [Vigna angularis]
MAAASSMALSSPSLAGKAVKLAPSAPEVGRVSMRKTVTKQVSSGSPWYGPDRVKYLGPFSGEPPSYLTGEFPGD
YGWDTAGLSADPETFARNRELEVIHSRWAMLGALGCVFPELLSRNGVKFGEAVWFKAGSQIFSEGGLDYLGNPS
LIHAQSILAIWATQVILMGAVEGYRIAGGPLGEVTDPIYPGGSFDPLGLADDPEAFAELKVKELKNGRLAMFSMF
GFFVQAIVTGKGPLENLADHLADPVNNNAWAYATNFVPGK
>Mu_g15332.t1 hypothetical protein PHAVU_010G134900g [Phaseolus vulgaris]
MFGILGGKGQKIKGTVVLMSKNVLDFNEIVSTANGGVLGVAGGLFGAANKVVGGIVDGATSVFSRNIALQLISATK
TDGLGNGKVGKQTFLEKHLPTLPTLGDRQDAFNIYFEWDESFGIPGAFYIKNFMQAEFFLVSVTLEDIPNHGTIR
FVCNSWVYNAKEYKKDRIFFANKTYIPNETPTPLVKYRKEELENLRGDGTGQRKESDRIYDYDVYNDLGNPDKS
ADLARPVLGGSSAYPYPRRGRTGRKATKRDPKSETPTSDTYIPRDESFGHLKSSDFLTYGIKSLSQNVIPQFQSAF
GLNAEFDKFDDVRGLFEGGLYLPTDVISKISPLPVLKEIFRTDGEQALKFPPPQVIKVSKSAWMTDEEFGREMLA
GVNPCLIQRLQEFPPKSKLDSTVYGDQTSTITKEHLEINLGGLTVEEASDALNGNRLFILDHHDAFLPYLRKINDL
PTAKSYATRTILLLKDDGTLKPLAIELSLPHPRGDEFGAVSRVILPADQGAESTIWLLAKAYVVVNDSCYHQLMS
HWLNTHATIEPFVIATNRHLSVLHPIYKLLLPHYRDTMNINGLARQSLINAGGIIEQSFLPGPFSVEMSSAVYKSW
VFTDQALPADLIKRGMAIEDPSSPYGLRLVIDDYPYAVDGLEIWSAIQTWVKDYVSLYYATDDAIKKDTELQTW
WKEAVEKGHGDLKDKPWWPKLNTLQDLIHICSIIIWTASALHAAVNFGQYPYGGYILNRPTLTRRLLPEPGTKE
YGELSSNHQKAYLRTITGKVEALVDLSVIEILSRHASDEVYLGQRDNPNWTDDTKALQDFQKFGNKLKEIENKIS
GRNNNSSLRNRVGPVQMPYTVLLPTGGEGLTFRGIPNSISI
>Mu_g15333.t1 hypothetical protein PHAVU_010G134900g [Phaseolus vulgaris]
MFGILGGKGQKIKGTVVLMSKNVLDFNEIVSTASGGVLGVAGGIFGAANKVVGGIVDGATSIFSRNIAIQLISATKT
DGLGNGKVGKQTFLEKHLPTLPTLGDRQDAFNVYFEWDEKFGIPGAFYIQNFMQSEFFLVSLTLEDIPNHGTIHF
VCNSWVYNAKKYKKDRIFFANKTYLPDDTPTPLVKYRKEELENLRGDGTGKRQEHDRIYDYDVYNDLGNPDKT
ADLARTVLGGSSDFPYPRRGRTGREKTKKDPKSEAPSSDTYIPRDENFGHLKSSDFLTYGIKSLSQNVFPIFESAF
GLNAEFDTFDDVRRLSEGGLYLPTDLLSKISPLPVLKEIFRTDGEQVLKFPPPQVIKVSKSAWMTDEEFGREMLA
GVNPCLIQRLQEFPPKSKLDSTVYGDQTSTITKEHLEINLGGLTVEEASDALNGNRLFILDHHDAFLPYLRKINDL
PTAKSYATRTILLLKDDGTLKPLAIELSLPHPRGDEFGAVSRVILPADQGAESTIWLLAKAYVVVNDSCYHQLMS
HWLNTHAAIEPFVIATNRHLSVLHPIYKLLSPHYRDTMNINGLARQSLINAGGIIEQSFLPGPYAVEMSSVVYKN
WVFTDQAIPADLVKRGMAIEDPSSPYGLRLVIDDYPYAVDGLEIWSAIQTWVKDYVSLYYATDDAIKKDTELQT
WWKEAVEKGHGDLKDKPWWPKLNTLQDLIHICSIIIWTASALHAAVNFGQYPYGGYILNRPTLSRRLLPEPGTK
EYDELSSNHQKAYLRTITGKYEALVDLSVIEILSRHASDEVYLGQRDNPNWTDDTKAIQSFQKFGNKLKEIEKKIS
ERNNNSSLKNRNGPVQMPYTVLLPTSEEGLTFRGIPNSISI

>Mu_g15677.t1 PREDICTED: acyl-CoA oxidase isoform X1 [Glycine max]
MAAPSSKNPEGPVQNAMPSYFYLPPLDVSAAFPQATPASTFPPSASDYFQLDDLLTAEEQAIRRKVRECMEKEIA
PIMAEYWEKAKFPFHVIPKLGALNIAGGTIKDYGCPGLSITGCALAYAEVARVDASCSTFILVHSSLAMLTIALCGS
EAQKQKYLPSLAQLHTISCWALTEPDYGSDASALKTTATKVEGGWILEGQKRWIGNSTFADVLVVFARNATTKQ
INGFLIKKDAPGLTVTKIENKIGLRIVQNGDIVMRKVFVPDEDRLPGVNSFQDTNKVLAVSRVMVAWQPIGLSMG
IYDMCHRYLKERKQFGAPLAAFQISQQKLVKMLGNIQAMILVGWRLCKLYESGKMTPGHASLGKSWITLSARET
AALGRELLGGNGILADFLVAKAFCDLEPIYTFEGTYDINTLVTGREVTGFASFKPVAQRSRL
>Mu_g15841.t1 PREDICTED: chlorophyll a-b binding protein of LHCII type 1-like [Vigna angularis]
MAASTMALSSPSLAGKAMKLSPSTTNLSGGRVSMRKTPSKSVSSGSPWYGPDRVKYLGPFSGEAPSYLTGEFPG
DYGWDTAGLSADPETFARNRELEVIHSRWAMLGALGCVFPELLARNGVKFGEAVWFKAGSQIFSEGGLDYLGN
PSLIHAQSILAIWATQVILMGAVEGYRIAGGPLGDVSDPIYPGGSFDPLGLADDPDAFAELKVKELKNGRLAMFS
MFGFFVQAIVTGKGPLENLADHLADPVNNNAWAYATNFVPGK
>Mu_g16031.t1 hypothetical protein PHAVU_005G109400g [Phaseolus vulgaris]
MASTTLSPITPSQLCSGKSGIFCPSQALLVKPMKRQMMGKSKGMRIACQATSISADRVPDMGKRQLLNLLLLGAI
SLPSAGMLIPYTYFFVPPGSGSSSGGTVAKDAVGNDVIAENWLKTHGPGDRTLTQGLKGDPTYLVVEKDRTLAT
FAINAVCTHLGCVVPWNTAEKKFICPCHGSQYNDQGRVVRGPAPLSLALAHCDIDDGKVVFVPWTETDFRTGD
APWWA
>Mu_g16214.t1 elongation factor 1-alpha [Vigna radiata var. radiata]
MGKEKVHINIVVIGHVDSGKSTTTGHLIYKLGGIDKRVIERFEKEAAEMNKRSFKYAWVLDKLKAERERGITIDIA
LWKFETTKYYCTVIDAPGHRDFIKNMITGTSQADCAVLIIDSTTGGFEAGISKDGQTREHALLAFTLGVKQMICCC
NKMDATTPKYSKARYDEIVKEVSSYLKKVGYNPDKIPFVPISGFEGDNMIERSTNLDWYKGPTLLEALDQINEPK
RPSDKPLRLPLQDVYKIGGIGTVPVGRVETGVLKPGMVVTFAPTGLTTEVKSVEMHHEALQEALPGDNVGFNVK
NVAVKDLKRGFVASNSKDDPAKEAANFTSQVIIMNHPGQIGNGYAPVLDCHTSHIAVKFAELVTKIDRRSGKELE
KEPKFLKNGDAGFVKMIPTKPMVVETFSEYPPLGRFAVRDMRQTVAVGVIKSVEKKDPTGAKVTKAAQKKK
>Mu_g16464.t1 PREDICTED: heat shock cognate 70 kDa protein 2-like [Vigna angularis]
MAGKGEGPAIGIDLGTTYSCVGVWQHDRVEIIANDQGNRTTPSYVGFTDTERLIGDAAKNQVAMNPINTVFDAK
RLIGRRFTDASVQSDIKLWPFKVFSGPGDKPMIQVSYKGEDKQFSAEEISSMVLMKMREIAEAYLGTTIKNAVVT
VPAYFNDSQRQATKDAGVIAGLNVMRIINEPTAAAIAYGLDKKATSVGEKNVLIFDLGGGTFDVSLLTIEEGIFEV
KATAGDTHLGGEDFDNRMVNHFVQEFKRKNKKDISGNPRALRRLRTACERAKRTLSSTAQTTIEIDSLYEGIDF
YSTITRARFEELNMDLFRKCMEPVEKCLRDAKMDKRTVHDVVLVGGSTRIPKVQQLLQDFFNGKELCKSINPDE
AVAYGAAVQAAILSGEGNEKVQDLLLLDVTPLSLGLETAGGVMTVLIPRNTTIPTKKEQVFSTYSDNQPGVLIQVY
EGERTRTRDNNLLGKFELSGIPPAPRGVPQITVCFDIDANGILNVSAEDKTTGQKNKITITNDKGRLSKEEIEKMV
QEAEKYKSEDEEHKKKVEAKNALENYAYNMRNTVKDEKIGGKLDPADKKKIEDAVEQTIQWLDSNHLGEADEF
EDKMKELESICNPIIAKMYQGGAGPDAGGAMDDDVPAGGSGAGPKIEEVD
>Mu_g16844.t1 hypothetical protein PHAVU_010G144300g [Phaseolus vulgaris]
MKVVVLVFATLLVASHGASFRSFPLLLKTDGDDESYQDIRCASWRLAAETRNILTWETIPEECVEATAKYIEGKQ
YRSDSKTVNQQVYFYAREREVHDNDVIVFSIDGTLLSNVPYYSQHGYGVEKFNSTRYDEEFVLKGDAPALPETLK
NYRKLVSLGYKIIILSGRKENKREVTEANLKQAGFDTWEKLILKEPSNSAPNALEYKKAERAKLVQQGYNIVAVV
GDQWSDLRGKPKGTRNFKLPNPMYSIE
>Mu_g16853.t1 hypothetical protein PHAVU_011G126000g [Phaseolus vulgaris]
MADGLEHPSVVQKLAGQSYLASRLSPNFHSRNYSATGSYFNGGLHTSGLAAVSSVSPITVHAPAEKGPGAFMVDF
LMGGVSAAVSKTAAAPIERVKLLIQNQDEMIKSGRLSEPYKGIGDCFARTMKDEGVIALWRGNTANVIRYFPTQA
LNFAFKDYFKRLFNFKKDKDGYWKWFAGNLASGGAAGASSLLFVYSLDYARTRLANDAKAAKKGGERQFNGLI
DVYRKTIKSDGVAGLYRGFNISCVGIIVYRGLYFGMYDSLKPVVLVGGLQDSFFASFLLGWGITIGAGLASYPIDTV
RRRMMMTSGEAVKYKSSLHAFQTIVAKEGTKSLFKGAGANILRAVAGAGVLAGYDKLQLVLFGKKYGSGGG
>Mu_g17498.t1 hypothetical protein PHAVU_008G199700g [Phaseolus vulgaris]
MLTAILRRTCSTLSRRAFPAALISASTAASNDFRPLSAAFISRTFHSKPSPLNFRSSLYHRAEYAVDDIPYEEGSKG
NAEEGLEIAKLGISQDIVSALAKKGIIKLFPIQRAVLEPAMQGRDMIGRARTGTGKTLAFGIPIMDKIIQFNAKHGR
GRDPLALVLAPTRELAKQVEKEFYESAPNLDTICVYGGTPISRQMRQLDYGVDIAVGTPGRIIDLLNRGALNLKD
VQFVVLDEADQMLQVGFQEEVEKILERLPPKRQTLMFSATMPSWIKQISRNYLNDPLTIDLVGDSDQKLADGISL
YSIATDLYVKAGILAQLITEHAKGGKCIVFTQTKRDADRLSYTMAKSVQCEALHGDISQAQREKTLAGFRNGHFN
VLVATDVASRGLDIPNVDLVIHYDLPNSSEIFVHRSGRTGRAGKKGAAILVYTEDQSRAVRLIERDVGCRFTELPR
IAVDSASMDTVGLGGGRFGSFGGSRDRRYGDTGFGRGPGSGRSGGYNSSGFGRSSFGDSGERFDGQNYNRFRGSG
LSQSGGGFSGNSSGGPSSGRFGSSGGFGSGQSGSRSGRSSSGSRFSRPDDFGGFGGSDRSGGFGDFGSGQPSGSRGS
NQNNRRPF
>Mu_g17657.t1 hypothetical protein PHAVU_010G144300g [Phaseolus vulgaris]
MKVVVLVFATLLVASHGASFRSFPLLLKTDGDDESYQDIRCASWRLAAETRNILTWETIPEECVEATAKYIEGKQ
YRSDSKTVNQQVYFYAREREVHDNDVIVFSIDGTLLSNVPYYSQHGYGVEKFNSTRYDEEFVLKGDAPALPETLK

NYRKLVSLGYKIIILSGRKENKREVTEANLKQAGFDTWEKLILKEPSNSAPNALEYKKAERAKLVQQGYNIVAVV
GDQWSDLRGKPKGTRNFKLPNPMYSIE
>Mu_g17750.t1 hypothetical protein PHAVU_009G066000g [Phaseolus vulgaris]
MQGQRGTVGSMPETLEFDCGSTPSNSTVDQPICWNNVNPAENQLPDFILSPGDMNSPYVNPINHEWQNLSGWS
LGEPSSSNAPNEINNNEQKRELGWTSTMTAAALPGARLEERRLEPTNTLSLDNDDASPMYLRSPNTRLMSQNL
NLNAGLADGASDNSQHLELPNLHMSSGSANECLPSNVGSGSFLLPSANNGFLVDDSDDRPVSLDTRRVSCKRKA
VEGNHGQSSDAGSSSYNQHTDGSAWHAIPTQDNAGSSSNRSVTTEQVNARLGLGMGDEASENVPNSNTAGSSE
SFHRNFRLRLNPSNQQNSVPPAAFSTGSMIRQSGISSSSQASQRFHSVDNSLNLRSAPPIDNVVPQSQPLVIHVPA
LPRNRQSFRWSGGSSSRNIHSSNPIICAERDQEDASSRRMSRNILEHPIFVPATDLRNLVQNPTVRASSSSSENLSI
PGNVASSSRTGPNPATNPSSASTWVSRPSPPQHPRRLSEYVRRSLFSPSPDAIGNPSNSYASLRSGLSTSEPRALSS
GTGANPRSSSWMERQGDSEFGIPYSLRTLAVASEGSSRLVSELRNVLGLMRRGGNVRFEDVVILDHQSFLSGIAD
VHDRHRDMRLDVDNMSYEELLALEERIGNVSTGLSEETVLKLLKQKKHSVEKESQIDAEPCCVCQEDYGDGDDI
GTLDCGHDFHSDCIKQWLMHKNLCPICKTTGLAT
>Mu_g17998.t1 PREDICTED: E3 ubiquitin-protein ligase RMA1H1-like [Glycine max]
MALQHYISRDLKTIPNTVTETENSNGCFDCNICFDFAHEPVVTLCGHLYCWPCIYKWLRVQSDSLTTYEHPQCP
VCKAVISNSTMVPLYGRGHTTAEGKTSSCDVLIPPRPPASCAQVLLATSSQRGQRLPYRNPYQGHYFSSHPYQEE
EATSQMLNLGSHHHPVTGMFGEMVYSRVFGNPENLYAYPNSYQLMGSSTPRLRRQEMQAHKSLNRISIFLFCCF
LLCLVVF
>Mu_g18164.t1 PREDICTED: 6-hydroxynicotinate 3-monooxygenase-like [Vigna angularis]
MVGEGKKPKAVIVGGSIAGISSAHALILAGWDVLVLEKTPSPPTGSATGAGLGLDPLSQQIIHSWLPHSQQQLLHS
TTLPLTIDQNHATDSEKKVNCTLTRDENFNFRAAYWPDLHGLLYNALPSDIFLWGHLFLSFHVVDDKDSVIVKA
KVLETGKVVEIVGDLLVAADGCLSSIRKKYLPDFKLRYSGYCAWRGVLDFSKIENSETITAFRKAYPDLGKCLYFD
LASGSHTVLYELKNKKFNWVWYENQPEPEVKGTSVTMKVNSDMIQKMQQEAEKVLIPEFVKVMKETREPFLN
FIYDSDPLEKIFWDNVVLVGDAAHPTTPHCLRSTNMSLLDAAVLGKCMEKWGAEKIGSALEEYQLIRLPVTSKQ
VLHARRLGRLKQGLVLPDREPFDPKLVRPEDYQELLQRNTPFFKDVPLHLV
>Mu_g18165.t1 3-hydroxybenzoate 6-hydroxylase 1 [Cajanus cajan]
MVGEGKPRAVIVGGSIAGISTAHALTLSGWNVTVLEKTNAPPTGTPTGAGLGLDPLSQRIIESWLSQPQLLHNLT
LPLTIDQNQATDSEKKFSRTLARDESFNFRAAHWADLHGLLYNALPSTVFLWGHLFLSFHVSDDKGSVIVKAMV
LHTGTVIEIVGDLLVAADGCFSSIRQKYLHDYKLRYSGYCAWRGVFDFSESEASETVKGIRKAYPDIGKCLYSNLGS
CTHCGFYELLNKKFNWIWYVNQPEPEVMGTSVTMKVSSDMIQKMLQEAEKVWSPELFKVMKETKEPFLNIIYD
GEPLEKIFWDHVVLVGDAAHPTTPHCLRSTNMSILDAAVLGKCLEKFGAEKVGSALEEYQFIRLHVTSKQVLHA
RRLGCLKQGLVLPDREPFNPNSAKKEDCEELLQRNTPFFNDVPLLLATILPSI
>Mu_g18333.t1 hypothetical protein PHAVU_010G134800g [Phaseolus vulgaris]
MFGILNRGHKIKGTVVLMTKNVFDFNEFVSTTRGGITGVAGGLFGAATDIVGGIVDGATAIFSRNIAIQLISATKTD
GLGNGKVGKQTFLEKHLPSLPNLGDRQDAFNVYFEWDENFGIPGAFYIKNFMQSEFFLVSLTLEDIPNHGTIRFV
CNSWVYNAKCYKRDRIFFVNKTYLPNETPTPLVKYRKEELENLRGDGAGERKEHDRIYDYDVYNDLGNPDKSK
NLARTTLGGSSDFPYPRRGRTGRRRTRTDPNCEIPTSDTYIPRDENFGHLKSSDFLTYAIKALTQNVLPSFQKAFG
LNNEFDTFDDVRCLFDGGVYLPTDVISKISPIPVLKEIFRTDGEQALKFPPPQVIKVNKSAWMTDEEFGREMLAG
VNPCLIQRLQEFPPKSKLDVRVYGDQTSTITKEHLEINLGGLTVEQALNSSRLFILDHHDAFIPYLKRINELPTAKS
YATRTILFLKDDGTLKPLAIELSLPHPRGNEFGTISRVILPAKEGAESTIWLLAKAYVVVNDSCYHQLMSHWLNT
HAVMEPFVIATNRHLSVLHPIYKLLLPHYRDTVNINGLARQSLINAGGVIERSFLPGEFAVEMSSAVYKSWVFTD
QALPADLIKRGMAVEDPSSPYGLRLMVEDYPYAVDGLEIWDTIQTWVKDYVSLYYPTNDAVKKDTELQAWWK
EAVEKGHGDLKDKPWWPKLNTPQDLIHTCSIIIWIASALHAAVNFGQYPYGGFILNRPTITRRLLPEPGTKEYAE
LTDNHQKAYLRTITGKVEALVDLSVIEILSRHASDEVYLGQRDNPNWTDDTKALQAFQKFGNKLKEIENKISGR
NKDPSLRNRVGPVQMPYTVLIPSCEDEGLTFRGIPNSISI
>Mu_g18571.t1 RecName: Full=Bowman-Birk type proteinase inhibitor DE-4
MMVLKVCLVLLFLVGVTTARMDLSHHLRSNHHESSDESSESSKPCCDLCTCTKSIPPQCHCQDMRLNSCHSACK
SCICALSEPAQCFCLDTTDFCYKSCHNDADKDLVNKF
>Mu_g19042.t1 hypothetical protein PHAVU_003G251000g [Phaseolus vulgaris]
MTPMATDKIFKPSKWFSNKTLKLSLHRRRPRSSNXXXXXXXXXXSASPRSPMSTCTTPKKGEMNGLMEAFRYLD
GDGDGKISAYELRSYFGSIGEHMSHEEVEEVIHELDSDGDNMLDLEDFTKLMKKDGGDGDEGDLRKAFEMFVW
EKEGCGCITPKGLQRMLHRLGDDKSHDECVAMIGAFDIDHNGVLDFDEFYQMMA
>Mu_g20000.t1 hypothetical protein PHAVU_010G057000g [Phaseolus vulgaris]
MPMASSTLLLPLLLSLIFFHNFHHSSSFSLSVENLEEDIIVSSPKKTFTAGFRAVGENAFCFAIWYTQPPHTVVWM
ANRDNPVNGKRSTLSLLKTGNLVLTDADQFQVWSTNTANFSKHLRLHLHDSGNLVLRDDSSNVDLWRSFDFP
TDTLLPGQTLTKSTNLVSSRSRSNYSSGFYRLFFDFENVLRIMYQGPRVSSVYWPDPWLQNNNFGNGGAGNGRS
TYNDSRVAVLDDLGSFVSSDKFTFKSIDYGTELQRRLTIDHDGGVRVYSKNDGEEKWTMSGEFPSHPCYVHGICG
ANSYCRYEPSSGRKCSCLPGHSWVDSKDWSQGCTPNFQQLCSSSNNTKYDYRFLHIPDVDFYGYDYGYFGNYSY
QQCENLCSQLCECKGFQHSFSEANAFFQCYAKTYLFNGNSQPGFTGSFFLRLPLSSHDEYESTIQNNGLVCGGNSE

GVELLKRSYVQEGENGSVKFMLWFASALGGVELVCIFLVWCFLFRNNRTLPSGAEGQGYVLAAAAGVRGTRGY
MAPEWVFNLPITSKVDVYSYGIVVLEMITGRSPTTGVQITELESESPHHERLVTWVREKRMKGSEVGSSWVDQI
VDPALGSNYDMNELEILATVALECVEEEKNARPSMSQVAEKLQRHMHTIVDSLNKE
>Mu_g20001.t1 hypothetical protein PHAVU_010G057100g [Phaseolus vulgaris]
MASPTLLLPLLLCLIFFDNFHHSSSFSLSVENPKEDIIVSSPKGTFTAGFRAVGENAFCFAIWYTRPPHTVVWMAN
RDHPVNGKRSTLSLLKTGNLVLTDADQFQVWSTNTANFSKHVRLHLHDSGNLVLRDDSSNVDLWQSFDFPTD
TLLPNQPLRGSTNLVSSRSGTNYSSGFYKLFFDFENVLRLMYQGPRVSSVFWPYAWLQSNNFGNGNGRSTFNDS
RVAVLDDFGAVVSSDNFTFRTIDYGTVLQRRLTIDHDGNVRVYSMKDGEDEWLVSGLFRSQPCFIHGICGPNSYC
TNDPTIGRKCSCLPGHIWVDSQDWTQGCTPNFQPWCNTTEQESRFVRLPEFDFYGYDYGFYQNHTYEQCVNQC
LRLCQCKGFQHSFSDQGGLGSQCYLKTQLLNGHHSPGFAGSFILRLPLSHNYENPINNGLLCGENSGGVKVLKRP
YVEEKENESVKFMLWLLVYEYMDNGSLAQNLSSSSNVLDWTKRCNIALGTARGLAYLHEECLEWVLHCDIKPQ
NILLDSDYQPKVADFGLSKLLNRNNLHNSNFSRIRGTRGYMAPEWIFNLPITSKVDVYSYGIVVLEMITGRSPIAGI
PITELEVESHHHERLVTWVREKRRKGSEVGSSWVDQIVDPALGSNYDRSEMEILATLALECVEEDKDVRPSMSH
VVERLQRHEHNS
>Mu_g20175.t1 PREDICTED: probable galacturonosyltransferase 6 [Vigna angularis]
MSKIRRCQRILILSLLFLSVVAPLVLVSHRLNLLTPLGRREFFEDLYRAEDAEELEEPNQVVYTEKDFVSTIGYDSE
SNDSKESRNAGYRAFNQHERKHQGAQQNELSFMSQGRNIHDSQRMLEKNIEVTTKKVQEIKDQLILAKAYLKIA
PPSSNLRLRDLERLTREMEFAVGEATQDSDLSMSALQKMRHMEASLSKVYRAFPDCSNMAAKLNTMKRQVEE
QVRSQRHQATYLVNLAARTAPKGIHCLSMQLTAEYFALRPEERKLPNENNINDPELHHYAVFSDNVLACAAVV
NSTISTAKEKEKLVFHVLTKSLNLPAISMWFLINPPGKATVHIQSIDNFEWSSKYNSYQENNSSDPRYTSELNYLR
FYLPDIFPALNKIVLFDHDVVVQQDLSALWNIDMKGNVIGAVGTCQEGKIPFHKIDMFINFSDPLIGKRFDANSCT
WAFGMNLFDLQQWRRHKLTAVYHHYLQMGLWSVGSLPLGWLTFYNKTELLDRQWHILGLGYSSDVDRNEIE
RAAVIHYDGLRKPCLVILYRFSFNSTMATISAATTTPSITRACLVQKRSLGVSSPVLGLPTMGKMGRVRCSMEEKP
SVKESSSSIGMGASLMAAACAAAMSGPAIALVDERLSTEGTGLPFGLSNNLLGWILFGVFGLIWGLYFVYTSSLEE
DEESGMSL
>Mu_g20409.t1 hypothetical protein PHAVU_011G183800g [Phaseolus vulgaris]
MSLTGKLSTEIPVHATADKWFNTFTNQLHHMQHVADKIHEAKLHEGDDWHTSDSVKHWSYTVDGEAITCHET
IEAIDEQKKTIVFKLYGEHIDDKYKLFNLIFEAIEKDGGNAAIRWSVEYEKVSEDVRPPYGYLEFCDHCINDIDAYL
VKGNQENANK
>Mu_g20509.t1 hypothetical protein PHAVU_002G269000g [Phaseolus vulgaris]
MVTKLVQPPMTDSHVSPLICRTMNVMNIQNIVPARNHSRINTSRFGERPLWFRPVTIIKVAEHNSGSGLVEIETL
AQKKREFYQAVEGINRGIFGMPSAKKSEIESLVQQLESLNPTPYPTQELEMVAGCWRLVYSTISILGSKRTKLGLR
DFISLDEFFQTIDISKSKAVNMIKFSARGLSLLSGQLSIEASFKISSSTRVDINFENSTITPDQLMNLFRKNYDLLLSI
FNPEGWLEITYVDDSMRIGRDDKSNIFVLERFDGSDN
>Mu_g20657.t1 uncharacterized protein LOC106755525 [Vigna radiata var. radiata]
MATFSFGIATTSVSAAVGYGTRSNCRWRRPTILSIGWDPEGVLGPPQTGHLARLEFRRRLERDAEAREAFERQV
NEEKERRQTLRQSRIVPDTPEELIEYFLDTEAQEIEFEIARMRPRLDEGFFAQLKFELGQLRFAVNKTEHMEERQ
IELEALEKAIQEGIEAYDKMQGELVKARAGLTKILTSKDVKATLLDMVEKNELNRSLLALLDENIANAHKGNQKQ
VAEYMEKLRGSVIRYLTV
>Mu_g20942.t1 Translationally-controlled tumor protein like [Glycine soja]
MLLYQDLLTADELLSDSFRYTEIENGMLWEVEGKWVVKGAVDVDIGANPSAEGGDDEGVDDQAVKVVDIVDTF
RLQEQPAYDKKQFVTFMKRFIKNLTPKLDAEKQELFKKHIEGATKFLLPKLKDFQL
>Mu_g21627.t1 hypothetical protein PHAVU_005G084500g [Phaseolus vulgaris]
MGVPEKDPLSQLSLPPGFRFYPTDEELLVQYLCRKVAGHHFSLPIIAEIDLYKFDPWVLPSKAIFGEKEWYFFSPR
DRKYPNGSRPNRVAGSGYWKATGTDKIITTEGRKVGIKKALVFYVGKAPKGTKTNWIMHEYRLLDSSRKTTGTK
LDDWVLCRIYKKNSSAQKAVQNDVVSSREHTQYSNGSSSSSSSHLDDVLESLPAIDERCFAMPRVNTVQQQQEE
KVNVQNLGSSGLVDWANPAVLNSVADFVSGNNQVVQDHTQGMVNYNGCNDLYVPTFCHVDSSLPQKVEEEVQ
SGVRNQNNNSWFLQNDFTQGFQNPVDTCGFKFPVQPVGFGFRQ
>Mu_g21794.t1 hypothetical protein PHAVU_001G010700g [Phaseolus vulgaris]
MASFPMPQLLPCTSLSLSNTTSTISLPSSLFRPRRTLHSLRCSASDKTPPASSHEDAVELPLFPLPLVLFPGAILPLQ
IFEFRYRIMMHTLLHTDLRFGVVYHDAVSGTSEVGCVGEVVKHERLVDDRFFLVCKGQERFRVNDVVRTKPYLV
GRVTWLEDRPSESDGGDDAEGLAREVEGYMKEVIRLSNRLGGKGEKEMGDLRRNLFPTPFSFFVGSTFEGAPRE
QQALLELEDTAKRLKREKETLKNTLNYLTAASAVKDAFPSS
>Mu_g22373.t1 hypothetical protein PHAVU_010G135900g [Phaseolus vulgaris]
MFSGVTGLINRGQKLKGTVVLMRKNVLDINALTSVQSPTGIIGGAIGVVGGAIGSTVDTLTSFLGRSVALRLISATA
ADASGKGKVGKQTFLEGIITSIATLGAGQSAFDIHFEWDSDMGIPGAFYIENFMQVEFFLVSLTLEDVPNQGTIHF
VCNSWVYNDKKYKSDRIFFANKAYLPSETPGPLVKYREEELNTLRGDGTGERKEHERIYDYDVYNDLGDPDSND
RLARPVLGGSTLPYPRRGRTGRKPTKKDPKSESRSDSVYLPRDESFGHLKSSDFLVYILKSASQNVIPQLQSALRL
QFNQPEFTSFDDVRGLYDGGIKLPTDALSKLSPIPLFTELFRTDGEQVLKFPTPKVIQVDQSAWMTDEEFAREMI

AGVNPHIIRKLQEFPPKSKLDSQLYGDNTSTITREHLEPNLGGLTVEQAIQNNRLFILDHHDTLIPYLRRINATET
KAYATRTIIFLQDNGTLRPLAIELSKPHPQGDNFGPISNVYLPANQGVEASIWLLAKAYVIVNDSCYHQLVSHWL
NTHAVVEPFVIATNRHLSVVHPIHKLLLPHYRDTMNINALARNVLVNAEGIIESTFLWGGYSLEMSAVVYKDWV
FPEQALPADLIKRGLAVEDSSSPHGLRLLIEDYPYAADGLEIWATIKSWVQEYVFFYYKSDAAITQDTELQAFWK
ELVQVGHGDKKNEPWWVKMQTREELIESCTTLIWTASALHAAVNFGQYPYGGYILNRPTLSRQFMPEIGSPEYD
ELAKNPQKVYLKTITGKNEALKDLTIIEVLSRHASDELYLGQRDGGEFWTFDKEPLEAFKRFGKRLAEIEQKLIQR
NNDETLRNRYGPVKMPYTLLYPSSEEGLTFRGIPNSISI
>Mu_g23047.t1 hypothetical protein PHAVU_011G021200g [Phaseolus vulgaris]
METQSHEILQQPPISSNDAHNDEASNHRDAEQKHDVSREEVGQILQVIASTGKFWHDWDKLKSMLSFQLKQVL
SEYPEAKLTSEQQYASLGESYIELVNKLDEALTCFIEGPPFTLQRLCEILLDAKNIYPNLSKLALALEKNLLVTSTLT
ICTDPYPQATAKEPVDQEKANEKQNQQSDTAQNGIEPLVSDKDEVMTEADVGDDMTIDMESFEGDKSSETNSE
PNANNV
>Mu_g23578.t1 PREDICTED: uncharacterized protein LOC108342625 [Vigna angularis]
MEEASWEQRLQALTHILTSPTTTPSLHSQFFIATQIPCYLNWDYPPFLCSNPNLLRTWLRSFFLKRVFGTTPQTS
WRSKCPYHQPPPLILAEGVEEPHWGPQERRAYVRKRMARKLRKNVNPVLHIVIPNLLLLSAMIWNPFQSQD
>Mu_g24039.t1 PREDICTED: acyl-coenzyme A oxidase 4, peroxisomal-like [Glycine max]
MTRPNPEIVDSKMPSYFNLPALDVSVAFPQATPASNFPPCVSDYFHYDDLLSLEEHALRNKVRECMENEIAPIM
TKYWEKAEFPFHVIPKFANLHIAGGTIKLALSDSLGYGCPGLSITGSAAAVAEVARVDASCSTFFLVHSSLAMLTIA
LCGSEAQKQKYLPSLAQMKTIACWALTEPDYGSDASSLKTTATKVEGGWILDGQKRWIGNSTFADLLVIFARNT
TTNQINGYIVKKDAPGLTVTKMENKIGLRIVQNGDIVLRKVFVPGDDRIQGVNSFQDTNKVLAVSRVMVAWQPI
GLSMGIYDMCHRYLTERKQFGAPLAAFQISQQKLVQMLGNIQAMILVGWRLCKLYDSGKMTPGRASLGKSWITL
RARETAALGRELLGGNGILADFLVAKAFCDLEPIYTFEGTYDINTLVTGREVTRFASFKPAAQKSRL
>Mu_g24262.t1 multiple organellar RNA editing factor 2, chloroplastic [Vigna radiata var. radiata]
MARAISSSCARLTMRLLSTASTTTFTATLPKPSSLSLPSRRFLFPLSHAIVLPSTRVAGIRCRVNRAGDSAYSPLNS
GSSSFSDRPPTEMAPLFPGCDYNHWLIVMDKPGGEGATKQQMIDCYIQTLAKVLGSEEEAKKKIYNVSCERYFGF
GCEIDEETSNKLEGLPGVLFVLPDSYVDPENKDYGAELFVNGEIVQRSPERQRRVEPQPQRHQDRPRYNDRTRY
VRRRENTQ

Supplementary Dataset S2:
Protein sequences of protease inhibitor genes of PHG-9 variety

>Mu_g02040.t1 PREDICTED: seed trypsin/chymotrypsin inhibitor IVA-like [Vigna angularis]
MELKMKVLLKVASLLFLLGFSATTVDARYDPSSIITSFFAIDDANYKATTKACCDKCFCTSIPPECQCKDIKETCHS
ACKTCICTRSIPPQCRCYDITDFCYRPCDHHGSKGE
>Mu_g03064.t1 uncharacterized LOC100306062
MASSLLVKVTCMVVVLMVGFGQIIPLAEAEIPCGTVQLAVAPCITYIRSPGGGSPPATCCNGVKNINNQAKATSDR
QGVCRCLKSTTFTIPGLNLQKLANLPGNCGVNLSYKISPTIDCTK
>Mu_g03420.t1 PREDICTED: non-specific lipid-transfer protein 1-like [Vigna angularis]
MASMVTRVFFIAIVCLALGSTTIPKAEAVVQCGQVVNYLTPCLSYVMYGGNTVPAQCCNGIKNLYNLAQTTQDR
QSVCNCIKNAVNSSGVNYSPFNLNLAANLPKKCRVNIPYQISPNIDCARVQ
>Mu_g03421.t1 non-specific lipid-transfer protein 1 [Vigna radiata var. radiata]
MASLKFACVLALLCLVVMTAPTARAISCGQVTTALTPCLSFLTKGGPVPPSCCAGVKSLNEAAQTTPDRQTVCNC
LKSTASKISGFNAGNAAALPGRCGVSIPYKISTSTNCATIKF
>Mu_g05032.t1 Non-specific lipid-transfer protein 8 [Glycine soja]
MKSSGAFAALVLLILLASTSEAAISCSDVVKDLRPCLSYLVSGSGQPPAACCSGAKALASAASSSEDKKAACNCIKS
TSKSISINSQLAQSLPGNCGITLPVAISPNADCSKVG
>Mu_g08590.t1 hypothetical protein PHAVU_011G169800g [Phaseolus vulgaris]
MKLLPCVVLLVWLFTATPLLAQYVIDTNGEQVDNDEEYYIKPAITDNGGRFTLISRNEGCPLNVGLENTDLALGY
PVKFTHFAVNIQDEDIGVNRDLKVEFVEVSSTCVQSTEWRVGENETRSGRRPIITGQDADLYGNYFRILETESAGI
YNFQWCPMELCSDCGFVCCTGGILRENGKILFALDGAPLPVVFQKKDD
>Mu_g08591.t1 endogenous alpha-amylase/subtilisin inhibitor [Vigna radiata var. radiata]
MSMKVFTFLTLAVWLFTATSTLSQTNNHVLDTNGDPLESDEEYFIRPAITDNGGRFTLINRNNSCPLYVGLENT
DLPQGYPVRFTPFARDNNDDDDEVKVGRDLKVEFVEVSSTCVQTTEWRLGENDTSIGRRVIITGRDNGIQNAGN
YFRIEETERSNIYNIRWCPTEVCTTCRFICGTGGIVRENGRILFVLDGSALPVVFQKKDD
>Mu_g09591.t1 hypothetical protein VIGAN_02041400 [Vigna angularis var. angularis]
MDLRESISNQTDVALSMTMLLLSKEARDKNLVYSPLSLHVVLSIIAAGSKAATLDQLLSFLRSKSTDHLNSFASQL
VAVVLSDAAPAGGPRLSFANGVWVEQTLSLLPSFKQLVNTDYKATLASVDFQTKAAEVTNEVNSWVEKETNGF
VKELLPAGSVDSRTRLIFANALYFKGAWNEKFDASITKDYDFHLLDGSSVKVPFMTSKKKQFIRALDGFKVLGLP
YKQGEDKRQFTMYFFLPDAKDGLPALAEKLASESGFLERKLPNQKEEVGDFRIPRFKISFGFEASDVLKELGVVL
PFTPGDLTEMVESPVGKKLYVANIFHKSFIEVNEEGTEAAAATAAIVVMQSMRISTKIDFVADHPFLFLIREDLTG
TVLFIGQVLDPRAG
>Mu_g09855.t1 PREDICTED: kunitz-type trypsin inhibitor KTI1-like [Glycine max]
MKMSNTITFFFALFLLCAFTSHLPSATAEIVFDTDGDPVLNGGVGFYYVLPVIKGKGGGIDRTKTGTETCPLTVVQ
SRFEVSKGLPVSFLSPIRLQYIHEHLPLEIQFLFSPSCTRPEWKLVQGLPEGLAVKQIGYNDTVPGWFQIRRVSQEF
FDYKFLFHSADGTSTWDLGIYIDSDGNRRLVATDSDPLLVQFQKLEISTA
>Mu_g10421.t1 kunitz-type trypsin inhibitor-like 2 protein [Arachis duranensis]
MAQSHEKVADFQGAPLVAGTEYYILPSHYGIEGGGVRLDKTGNQTCPLTIVQDYSVSIGDTVKFSIPPVTGPDARL
IFTGRPLDIEFTEKPGCATSSKWSVFEDDYLTYLGVAGPGHVPTMKSGKFNIKRFEGAYKLGFCYPHVCYDVEKF
DHGHEGGWRLGLVNVGNPFKLFFVNVKDVIKHVV
>Mu_g10423.t1 kunitz-type trypsin inhibitor-like 1 protein [Vigna radiata var. radiata]
MKPTLVLAFFFLPLLAFLTNLPLTFCQAEQVLDTKGNPIFPGHTYYIIPAIRGPPGGGLKLAQTGNSKCPLTVLQD
YSEVFRGLPVRFTIPDVSTGIIFTGTPLEIEFVEKPWCAESSKWVSFVDESIQKSCVGIGGAEGHPGQQTFGGKFHI
ERYQFGYKLVFCINGSQTCFDIGRFDAQNGEDGRRLNLTEHEAFDVVFVKASSEADAVIKTVV
>Mu_g10690.t1 hypothetical protein PHAVU_010G066500g [Phaseolus vulgaris]
MASVKFACVVVLCMVVVGADTAQGIACGDVQRNLVPCLGFLKNGGFVPAGCCNGVRSILNSARTTADRRAVCNC
LKSAAAAVRGLNLNNAQALPGKCGVNIPYTISPSTNCARYVYIDSEIIKHRL
>Mu_g10691.t1 hypothetical protein PHAVU_010G066400g [Phaseolus vulgaris]
MASLKIACMVAVVCMVVLSAPMAHAITCGQVASSVGPCITYIRSGGNPSAGCCNGVRSLNAAAKTTADRQATCN
CLKTIATKIGAFNARNVASLPGKCGVSIPYKISTSTNCATIKF
>Mu_g12020.t1 PREDICTED: kunitz-type trypsin inhibitor KTI1-like [Glycine max]
MKMSNTITFFFAPFLLCAFTSHLPSATAEIVFDTDGDPVLNGGVGFYYVLPVIKGKGGGIDRTKTGTETCPLTVVQ
SRFEVSKGLPVSFLSPIRLQYIHEHLPLEIQFLFSPSCTRPEWKLVQGLPEGLAVKQVGYNDTVPGWFQIHRVSKE
FYDYKFLFISADGSSTWDIGIYIDSDGNRRLVATDSDPLLVQFQKLETSTA
>Mu_g12368.t1 PREDICTED: kunitz-type trypsin inhibitor KTI1-like [Vigna angularis]
MKSTTLLSLFLFCVFTTYLPSAIAQEVVDTDGNVLVNGGIYYLRSVIITANGGGVEYTATGNETCPLTVVQNPSPF
ANGSPVQISSPLRIRTIYEGLILYIGFTFGPPCAPTPSYWTVVKGLGEELPIKLTGYNNIVSGWFQIKSVPVEIGGYN
LLFCPTDGSACSYVAVDVNANRQRRLVLTQGEEGRLWIRFEKLSYSSTATNEPQLTLKSYV

>Mu_g12813.t1 PREDICTED: non-specific lipid-transfer protein 1-like [Vigna angularis]
MASLKVAFMVVMCMAVVGAPVMVQGVTCADVVANLSPCLSYLTNGGAASDACCGGVRSILGAAGTTSEKQTVC
NCLKSAANSFGINDDYAQALPGLCGVNVPYKISRSTNCADIRF
>Mu_g14745.t1 PREDICTED: trypsin inhibitor DE-3-like [Vigna angularis]
MASAMVIALFLVSALTLYPPSATAQVTDMYGNIVSNGGRFHILPYIIGNGGGIEKIATGNETTPLTVVQSPFETSKG
LPWRISSIFLPTFIPEPSLVFVKSEQSPLGDAFLQWTVVEGLPEGSVVKVSGYPNTVRGSFYIHRASSPNSYKFLFC
NFDGSLCGNVGIANDDAGNRVLVITQDKPFEFVLEQLPSSSTASA
>Mu_g14746.t1 PREDICTED: chymotrypsin inhibitor 3-like [Vigna angularis]
MASAMVFALFLVSALTLYPPSATAQVTDMYGNIVKNGGRFHILPYIVGNGGGIERIATGNETIPLTVVQSPFETSK
GKPLRITSIFRSGFIPEPSTVFIQFELIPLGDASLEWTVVEGLPEGSVVKVSGYANTVRGSFSIHRASSPNSYKLLFC
NFDRSLCGNVGIVRDDAGNRLLAITQGKELEFVLEELPSSSAASA
>Mu_g14972.t1 PREDICTED: trypsin inhibitor A-like [Vigna angularis]
MKTLIPPTASTLFFFSLFFLSIFTSNIPHAAADAVTDRDGDALQNGGTYHILPLFGVRDGGVELAATGSETCPLTV
VQSRTSQIFRGLPVRISSPYRMAYITEGLILNLAFASSPSCAPTPPKWTVVKDLAEGQAVKLPGYRDTVSGWFKIE
KSSLEYLYKVVFCARSGGRCGDVGISVDDEGMSRLVVTEEEDEGMIVEFMKATSV
>Mu_g16946.t1 PREDICTED: kunitz-type trypsin inhibitor KTI1-like [Vigna angularis]
MKSTTLLSLFLFCVFTTYLPSAIAQDVLDTDGNTLRNGGIYYLRSVIITANGGGVEFAATGNETCPLTVVQNPSPF
SNGLPVKISSLFRIKWISEGLILYIGFTSVPPCAPTPSEWTVVKGLGEELPIKLTGYDNTVRGWFHIKSVPVEIGGY
NILFCPEDGSACGYVAVDVEANRQRRLVLTQSEEDRLWVRFEKLSYSSTATA
>Mu_g16947.t1 PREDICTED: kunitz-type trypsin inhibitor KTI1-like [Vigna angularis]
MKMTTTTFLALFLLSAFTTYHPSAIAQDVLDTDGNVLSNGGIYYLRSVIITGNGGGVEFAATGNETCPLTVVQNP
SPFSNGLPVQISSLAKIPWIYQGLSLYIGFTFVPPCAPTPSEWTVVKGLGEELPIKLTGYDNTVRGGFKIKSVPVEIG
GYNLLFCPVDGSACGYVAVDVDANRQRRLVLTKSEEARLWIRFEKLSYASTATATA
>Mu_g17098.t1 hypothetical protein PHAVU_004G130600g [Phaseolus vulgaris]
MKCALSVTLSFLLFAFTTNFPLAFSDAPQKILDSKGAPISPSGEYYISQLNHGATGGGLKAGLGGKNATCQVTVLQ
DFYEAVRGQAVKFSIQGTNSGGIFIDTPLDIAFVDKPACASSSKWVVVADNYPGKWVGIGDAVDHPGKKIINGVF
KIQKYLFIEGYKFVFCPTITAVRPCFNIGRREDKFGNRLILINETSSQFIFEVAFDKVVLS
>Mu_g17099.t1 PREDICTED: kunitz-type trypsin inhibitor-like 1 protein [Vigna angularis]
MKSALSLTLSFLLAFTTIFPLSFSRAPPEEVKDSEGKPISTSGKYCILPFNGGPKDGSGLILVSNDNSTCYSTILQLPF
DGHDGVAVKFSTQGRSSGGILTGTALGIAFDDGPSCVSSSKWVVVSDDFPKKWLGIGGARDHPGKKIIGGAFKIE
KYNGGYKFVFCVSRRRCYNIGTDHNGRGGHRLILTHDPFHVQFDPKSNITHS
>Mu_g17590.t1 PREDICTED: kunitz-type trypsin inhibitor KTI1-like [Vigna angularis]
MKMTTTTFLALFLLSSFTTYLPSTIAQDVLDTDGNILRNGGIYYLRPVIITANGGGVEFAATGNETCPLTVVQNPS
PFSEGSPVHISSLARIPWISQGLSLYIGFTSVPPCAPTPSEWTVVKGLREEPSIKLTGYDNTERGGFQIKSVPVEIGG
YNLLFCPDDGCACSYVAVGVEANRQRPLVLTKSEEAPLWIRFQKFSDDASTATHEPPLTLKTISTLCNLEKGMVI
KNKIHEISDEAQC
>Mu_g17716.t1 hypothetical protein VIGAN_02041400 [Vigna angularis var. angularis]
MDLRESISNQTDVALSMTKLLLSKEARDKNLVYSPLSLHVVLSIIAAGSKAATLDQLLSFLRSKSTDHLNSFASQL
VAVVLSDAAPAGGPRLSFANGVWVEQTLSLLPSFKQLVNTDYKATLASVDFQTKAVEVANEVNSWAEKETNGL
VKELLPAGSVDSTTRLIFANALYFKGAWNEKFDASITKDYDFHLLDGSSVKFPFMTSKKKQFIRALDGFKVLGLP
YKQGEDKRQFTMYFFLPDAKDGLPALSEKLASESGFLERKLPNQKEEVGDFRIPRFKISFGFEASDVLKELGVVLP
FTTGDLTEMVESPVGQKLCVSDIFHKSFIEVNEEGTEAAAATAATVVLRSMRFPTKIDFVADHPFLFLIREDLTGT
VLFIGQVLDPRAG
>Mu_g18224.t1 PREDICTED: kunitz-type trypsin inhibitor KTI1-like [Vigna angularis]
MKSTTLLSLFLFCVFTTYLPSAIAQDVLDTDGNTLRNGGIYYLRSVIITANGGGVEFAATGNETCPLTVVQNPSPF
SNGLPVKISSLFRIKWISEGLILYIGFTSVPPCAPTPSEWTVVKGLGEELPIKLTGYDNTVRGWFHIKSVPVEIGGY
NILFCPEDGSACGYVAVDVEANRQRRLVLTQSEEDRLWVRFEKLSYSSTATA
>Mu_g18238.t1 PREDICTED: kunitz-type trypsin inhibitor KTI1-like [Vigna angularis]
MKMTTTTFLALFLLSAFTSFLPSATAFVVDTDGKPLRNGGVYYLMPVIITGNGGGVEFAATGNETCPLTVVQNPS
PFANGLPVQISSPVKVLYIGENIILNIGFTYVLPCAPTPSDWTVVNGLGEELAVKLTGYEDTVPGWFRIKRVPVDV
GGYNLLFCPTDGSACGYVSVGSEDRRLVVSQNEGGRLWVRFQSLSSASAAIATVTATA
>Mu_g18571.t1 RecName: Full=Bowman-Birk type proteinase inhibitor DE-4
MMVLKVCLVLLFLVGVTTARMDLSHHLRSNHHESSDESSESSKPCCDLCTCTKSIPPQCHCQDMRLNSCHSACK
SCICALSEPAQCFCLDTTDFCYKSCHNDADKDLVNKF
>Mu_g21982.t1 miraculin-like [Vigna radiata var. radiata]
MMKKTLLAFVFLFALSSQPLLGAAEASPEQVVDTSGKKLRAGLSYYIVPAVPFTRCGRYGRCMNSGGLSLASIGE
ACPLDVVVVPGSRGLPLSFSPVNPKKDVVRVSTDLNIMFSTDHTSCAEYSPVWKLDRFDVSKGQWFLTTGGSMG
NPGWGTIRNWFKIEKCDGAYKIVYCPSVFPSSKHLCKDVGMFVDENGYRRLALSDVPFKVKFQLA
>Mu_g21983.t1 PREDICTED: miraculin-like [Glycine max]

MMKTTLLVFVLLFALSSQTLPGAAEASHEQVVDTSGKVLRAGVNYNILLSMPYTSCRSPQGLGLSKIGNSCPLDV
VVVDRYHSLPLRFIPVNPKKGVIRISTDLKITFPPNVTCPHHSTVWKLDNFHVSKGHWFVTTGGETMGNWFKIE
KYDGAYKLVYCPSVCPSCKHECKNVGMFVDQNGNQRLALSDVPFQVKFLKA
>Mu_g21985.t1 PREDICTED: miraculin-like [Vigna angularis]
MKITLVQLFLLVALSTKALPGEGGPAPEQVVDTSGKKVRAGGFYYIVPASSDVGGLALASTGEKCPLDVVAVDGY
QGQPLTFYLLNDKKGFVRVSTDLNIYFSTYTTCPESIVWKLKDYDYSTSQWFVTTGGGFGIPGPDTISNWFKIEK
YEDAYKLVYCPSVCNDCSHPCSDIGIYQDQYGKRLALSSAPFKVKFQKVISY
>Mu_g23081.t1 hypothetical protein PHAVU_004G137600g [Phaseolus vulgaris]
MKVAMLLHLLPLCFSLLLAFNTQPLLAAEPEPVVDKQQNPLVPGVGYYVWPLWADDGGLTLGQTRNKTCPLDV
IRDPSFIGSPVAFFAPGLDYVPTETDLTIDFPVVTICNQPTVWRLSKEGAGFWFVSTRGNPEDITSKFKIERLAGE
HAYEIYSFKFCPSVPGALCAPVGTFVDSDGTKVMAVGDNLETYYVRFQRAGVNDQEKKRPFSIV
>Mu_g23086.t1 uncharacterized LOC100527288
MKVSSLAFSVLFLAFTIEPFISVAVAAPEPVLDTSGHELRAGVQYYILPVKGGNGGGLTLANDTCPLFVVQEKLEV
HGTPVTFTPYNAKTGVILTSTDLNIQSKPTPSCDKPPVWKLLKALSGVWFLSTGGVEGNPGIDTIDNWFKIEKFQ
NDYVLSFCPSVYPIETLCRGLGLYVGDDGKKHLSLSDQVPFFKVVFKRATIGNPTNVIKQVKSE
>Mu_g23087.t1 hypothetical protein PHAVU_004G138500g [Phaseolus vulgaris]
MKVSSLAFSLLFLAFTIEPFIGVAVAAPEPVLDISGHELRAGVQYYILPAKSGNGGGLTLAKDTCPLFVVQEKLEVH
GTPVTFTPYNAETGVILTSTDLNIQSKPTPSCDKPPVWKLLKALSGVWFLSTGGVEGNPGFDTLFNWFKIEKFQ
NDYVLSFCPSVYPIETLCRGLGLYVGDDGNKHLSLSSEVPFFKVVFKRATIGNPTNVIKQVKSE
>Mu_g23088.t1 hypothetical protein PHAVU_004G138500g [Phaseolus vulgaris]
MKVSSLAFSILFLAFTIELFIGIAVAAPEPVLDTSGQKLRTGVKYYILPVFRGKGGGLTVSSSGNNTCPLLVVQEKLE
VNGTPVTFTPYNAKSGVILTSTDLNIKSYGTTTTCDKPPVWKLLKVLTGVWFLSTGGVEGNPGIDTIVNWFKIEK
AEKDYVISFCPSVCNCQTLCRELGLYVGDDGNKHLSLSDKVPSFRVMFKRA

Supplementary data 3:
Protein sequences of CCT domain genes promoting flowering under short day condition identified in PHG-9 variety
>Mu_g00462.t1 zinc finger protein CONSTANS-LIKE 16 [Vigna radiata var. radiata]
MGLKFEKPMLIHRASGSPQEIGVIREGANEKAENRMNFPSSTNENLDKAFHCGAQSSIPFAVQAKNAEYAVGGK
TARACDSCITKRARWYCAADDAFLCQACDSSVHSANPLARRHERVRLKTASYKSTDEQQQPPTWHTKKARTPR
HGKHSRSNNPFHLVPEVGSEEVNSHDENEEQLLYRVPIFDPFVAELCRTNSSSPSSVTSTDQGVAAALTEAENKG
VHQSNVFYHSNNEMENLHGMLPSDAELAEFAADVESLLGRGLENECMGMEDLGLIDAKEEECSVGSGKVKVEE
EESPQMEMEMVGRDLSFELSFDYETCEEVKEKICDLELGKDKRNEEELGENDEMKKKKISLQLDYEAVIIAWAG
QKSPWTTAHKPNLDPDECWYQCMETCGTAIHHPYGEVGGFGIHPVIIDGGREARVSRYREKRRTRLFSKKIRYE
VRKLNAEKRPRIKGRFVKRASFAAPTFPLLNK
>Mu_g00527.t1 putative zinc finger protein CONSTANS-LIKE 11 [Vigna radiata var. radiata]
MKKCELCKLPARVFCESDQASLCWDCDAKVHGANFLVARHSRNLLCRTCHSPTPWKAAGASLANAATVCDRC
AASAAEEPDETESGNDEEPDTEDDEVTGEVDCDNQVVPWSSTPPPPASSSGSEWSSFSGCKNDDDDEGASVSVC
EPETTTSTLKRQREDDDDDVSVRDPQXXXXXXXXSNEEYXXXXXXXXXXXXXXXXXXVKAGGQKGCASQRRWRV
TESDR
>Mu_g00670.t1 PREDICTED: LOW QUALITY PROTEIN: two-component response regulator-like APRR3 [Vigna angularis]
MEERRQSQIGHLVARFTSLELNLYLRSNVSDWVRNYDLSWRILAVLGLVKRLQVPLFLDQEAFDIMLNNQNSEL
ARKSSGSGSESATHTRKSAKSRSNDASENNSDSSDENDYGSKGLNIRDGSDNGSGTQSSRTKCRAQVGSPHPVSP
HKQLVDAPDSTCAQVMQTKTEKVSSRWVHATEKECLELIDHLDDVAMGKDLAVGISLNMQLEHPLEGLSSHSM
GKGANKMSDVDDMQIVKGQSNVCEKGQLEYNGDTNRTQENQAMNVIDVTDSNSPQAESRYMNTPNGFSGFSL
TKENCCPKEHPSLELTLKRLGEVRDAKNVTVEECNVLRHSDQSAFSKYNTASSGNQVMFFGPLTTLCIVLKAQT
GNVGSCSPLANGSAAPNAEIVHNFPSHSNVTPPNQQSNGSNNINDMASTNTYLGTKPDTFDKKPESVRGMGSFN
SSELHILQNKSICSSQKKTYAREEYMEIVKGQVGGSEHEFQVEHTHYELHHYNHISHKAAVDLQSDHDLFFKSSN
NTAQQCVSSNAFGGPAESNAANYTVDGNTVESDHGSNGQDGTMTMRTVNMENGNVAAGSIGVGGIDRMNIGN
GTDEGRLALREAALTKFRQKRKERCFEKRVRYHSRKKLAEQRPRIRGQFVRRIVSEAKEEKDHRSNNLVPGDNS
DTPRMEERRQSQIGHLVARFTSLELNLYLRSNVSDWVRNYDLSWRILAVLGLVKRLQVPLFLDQEAFDIMLNNQ
NSELARKSSGSGSESATHTRKSAKSRSNDASENNSDSSDENDYGSKGLNIRDGSDNGSGTQSSRTKCRAQVGSPH
PVSPHKQLVDAPDSTCAQVMQTKTEKVSSRWVHATEKECLELIDHLDDVAMGKDLAVGISLNMQLEHPLEGLS
SHSMGKGANKMSDVDDMQIVKGQSNVCEKGQLEYNGDTNRTQENQAMNVIDVTDSNSPQAESRYMNTPNGFS
GFSLTKENCCPKEHPSLELTLKRLGEVRDAKNVTVEECNVLRHSDQSAFSKYNTASSGNQAQTGNVGSCSPLAN
GSAAPNAEIVHNFPSHSNVTPPNQQSNGSNNINDMASTNTYLGTKPDTFDKKPESVRGMGSFNSSELHILQNKSI
CSSQKKTYAREEYMEIVKGQVGGSEHEFQVEHTHYELHHYNHISHKAAVDLQSDHDLFFKSSNNTAQQCVSSNA
FGGPAESNAANYTVDGNTVESDHGSNGQDGTMTMRTVNMENGNVAAGSIGVGGIDRMNIGNGTDEGRLALRE
AALTKFRQKRKERCFEKRVRYHSRKKLAEQRPRIRGQFVRRIVSEAKEEKDHRSNNLVPGDNSDTPR
>Mu_g00678.t1 hypothetical protein PHAVU_011G069800g [Phaseolus vulgaris]
MQYAAAPQPHHHHNHQVALPEFDSEASSNNTYNSCSSGCTSYMGSPSSLASYESRRVQRSVSSHSLQKNSGGPH
HPFSPLFAQFLDSQNGPVRRACSTGDLQRFHGMQHYHHSDSPLSSESSLIIEEMSRTSPYSPEEKKVRIERYRNKR
NQRNFGKKIKYACRKTLADSRPRIRGRFAKNDEIATNPPVQWSHMGPGEEEDEEEENWANFFDSLVPANLAQE
PQGSSSSFGVFY
>Mu_g00828.t1 hypothetical protein PHAVU_001G203000g [Phaseolus vulgaris]
MSAEEPKPARPCDYCGHSTAVLYCRADSAKLCFSCDREVHSTNQLFSKHTRTLLCDACDDSPATILCSTDTSVLC
QNCDWEKHNPALSDSLHQRRPLEGFTGCPSVSELLSVVGFGDLSKKSLLSSPQGSAGDGFLGCEIEGLSDLFVWD
APSLVTLDDLICSSASSHSFQAMEVPPLPKNRKAACGRHREEILSQLRELAKSEPLDIEQYVQSGNLSSGFERDVE
VDIFPSHEWHRESSEPMYQVVPPDPSLRTYTEEISVKQSTSAVAETHTYGDNGGKPSISLKSETIPTTPKAAACEL
TSLERDSALLRYKQKKKTRRYDKHIRYESRKVRAESRVRVKGRFAKMEQEH
>Mu_g02226.t1 hypothetical protein PHAVU_007G166100g [Phaseolus vulgaris]
MSGSPNQKQRTCDYCGDFTALLYCRADSAKLCFFCDRKVHFPNQLFSKHTRTQLCDGCGDSPASVLCYAENSVL
CHYCDCQSHNNHSLSQEHQRRPLEGFSGCPSVTQMLMILGLTEKSLLSTEGGSSHHDGLSALHMWNAPSVVGLE
DLVASSASFHKNLKGACGRQKDEILSQLGELIKLEPDLIHGEADAEQQLQFENLSTGFERDVEANMFHSYEAGVF
CWHGESSDRANQIVPSDTSMSDYSELVSAKDSSFSIPATQTYCNNQGKPSNYFKAENLSPTPKATPYELTSHERD
SALLRYREKKKTRRYDKHIRYESRKVRAESRMRIKGRFVKDETQK
>Mu_g03665.t1 zinc finger protein HD1 [Vigna radiata var. radiata]
MSSDLYSYDTTFHTHSNSDLLSSDGVGDLPFLSDSFPFFPNSPSNVVQDNSNSNSLPSLDPFSPSFFSFSPPSSHLE
SLSLYHANRVQPLSNSQNLANEFGSFSAFDGSEVKSEECQLGVDCVYTQQILPHSYSGAENFSKYMQRSFSSHSFE
GKPGFLSQPYSDTLVDSPKFQRHDLSSPEDTLFSGQMRRVCSTGDLQNIKENHMSPTEAPLLEESNFKVGRYSAE
ERKERISKYRAKRTQRNFNKTIKYACRKTLADNRPRIRGRFARNDEPIDTPKASCSTRDEDDVDFWIEELRLHEE
QEDVTVGAEQYLRSYGGNQFQYGGGF

>Mu_g03888.t1 Zinc finger protein CONSTANS-LIKE 4 [Cajanus cajan]
MFAETELLFPYFRNFSQEFQQLEEYCMTHKSNASMNDLVQSSSVISEYDLLIEGDLFKAPEPIIEEPTMDLDPVLA
AISMISCGEEVSSQGLKSSDIDVLQNEQLLSEVFYECKKDLLEKTVIESPLSDIMEIKVPALNIETNSNQVTKHLSQ
MTLPKSISSESLSSMDWIHGAAMKPGFLDFPGIDFNVVYGMRRAFSEGDIKTLSTGNTSFGQSPQERPFLLSNCTS
EERQEKLSRYRNKKTKRNFGRKIKYACRKALADSQPRIRGRFAKTEELDVKRQMFAETELLFPYFRNFSQEFQQ
LEEYCMTHKSNASMPCTPGFFQSQLNDLVQSSSVISEYDLLIEGDLFKAPEPIIEEPTMDLDPVLAAISMISCGEEV
SSQGLKSSDIDVLQNEQLLSEVFYECKKDLLEKTVIESPLSDIMEIKVPALNIETNSNQVTKHLSQMTLPKSISSESL
SSMDWIHGAAMKPGFLDFPGIDFNVVYGMRRAFSEGDIKTLSTGNTSFGQSPQERPFLLSNCTSEERQEKLSRYR
NKKTKRNFGRKIKYACRKALADSQPRIRGRFAKTEELDVKRQ
>Mu_g04935.t1 zinc finger protein CONSTANS-LIKE 15 isoform X1 [Vigna radiata var. radiata]
MLLCDYCHSKPALLFCRPDSAKLCLLCDQHVHSANALSLKHVRFQICDTCKSDTAVLRCSSHNLLLCHRCDFDA
HAHAPDSLHHRHRLRGLSGCPSIPEIASTLDLDFRPRHSKCRDEVFEQVLEVARRRNENEFARAESVELRFDSGS
DVVDEMLLQQTPFTSLLGMLDSQSEFGAAKSNNNGCGAQEGDLLWSFDPNYQPPQVWDFQLQKSRDCDEPRL
VTFDGLEVPTSFQDVHNMNYSTTGDDILSRNNQSDQSSSSHAKKKAESNKKARCGLSSESKLLESIPYSATKNVV
VMEHLVCGNENVSTLKARVSFEELAKNRGDAMLRYKEKKKTRRYDKHIRYESRKARADTRKRVRGRFVKASDV
QAMLLCDYCHSKPALLFCRPDSAKLCLLCDQHVHSANALSLKHVRFQICDTCKSDTAVLRCSSHNLLLCHRCDF
DAHAHAPDSLHHRHRLRGLSGCPSIPEIASTLDLDFRPRHSKCRDEVFEQVLEVARRRNENEFARAESVELRFDS
GSDVVDEMLLQQTPFTSLLGMLDSQSEFGAAKSNNNGCGAQEGDLLWSFDPNYQPPQVWDFQLQKSRDCDEP
RLVTFDGLEVPTSFQDVHNMNYSTTGDDILSRNACSRSYCLLLL
>Mu_g05805.t1 zinc finger protein CONSTANS-LIKE 2 [Vigna radiata var. radiata]
MLKEGTNNVGGTTSTWSRVCDTCRSAPCIVYCHADSAYLCSPCDARVHAANRVASRHERVWVCEACERAPAAF
LCKADAASLCSSCDADIHSANPLASRHHRVPILPISGSLLGEPDHEPDHRFVNEVEEEEEEVFFEYDDEIEAASWL
LPHPVKVNEEEENCFLYGDEYLDNLLDCNSCGHNDNQFSNVYQHQQNYNTLPQNYAVVPVQLLQQSQHFQPGL
EFDSSKAGFSYDGSLSQSVSVSSMEVGVVPESTISDISMSYSKSPIGTSELFPPLPMPSHLTPLDREARVLRYREKK
KTRKFEKKIRYASRKAYAETRPRIKGRFAKRTDVEAEVDQMLSATLFTEVGGSIFPSF
>Mu_g06909.t1 zinc finger protein CONSTANS-LIKE 9 [Vigna radiata var. radiata]
MLTFAAAEFAELNCSFLHQYHIVEQLRSLVYCRSDAACLCLSCDRNVHSANALSRRHSRALLCERCNSQPAFVRC
VEEKISLCQNCDWLAHGTSPTSSTHKRQTINCYSGCPSAAEFSSIWSFFLDIPSMGEACEQELGLMSINEDGNKSD
WVPLEGQHVSGSAQVTDLPSKGKSWAGTSSIPGSSSEPRILDQPPGPANECLPKLYCPGKKVSGICEDDNYDDFI
MDEVDLELENYEELFGMALSHSEELFENGGIDSLFETKEMSASAGDSHCQGAVAAEGSSAGLVNAIQPACSNAAS
ADSMLSTKTEPIVCFTARQSQSNISFSGVTKDSAGDYQDCDASSMLLMGEPPWCPPCPESSLHSANRSNAVMRY
KEKKKTRKFEKKVRYASRKARADVRRRVKGRFVKAGDVYDYDPLNQTRSC
>Mu_g07789.t1 putative zinc finger protein At1g68190 isoform X1 [Vigna radiata var. radiata]
MEKVCEFCTALRPLVYCKADAAYLCLSCDAKVHLANALSGRHLRNLVCNSCGHHVAYVLCLDHKMLICRDCDQ
KLHNVSLPHQKRAIRSFMGCPSAKDFAALWGIELNEIENPACQDQFDSVSCISADLNVAQVSGKPDIQTGVPSLLS
GAKLDGGSTSQQGQILCNLQERQTIVQQIIDLKWLQQNEEIDYSVKINRLKEKKISPSVYHTLKKLDEKFNGQAQ
NSQDLATNVLEKDCPIVELNTETLPSTFSQLDNLSSSSIIDLPLHGELFWTCKSPLRSNQLWSQNIQDLGICEELVC
LDDFNIPDVDLTFQNFDELFGGDQDPIRILFDDQDVSCSSLEKDKSVDKSDIDNPSAMEESSAAASITISQSDHVN
KDMDPLSQYCPKRMDPMHAIRPFDSDIPLSVLRFSPESSFNGHEDSALSSYSEGKAYSGMRSNFDFYPGNLELM
>Mu_g08007.t1 zinc finger protein CONSTANS-LIKE 4 [Vigna radiata var. radiata]
MASKLCDSCKSATATLYCRPDAAFLCGACDSKVHAANKLASRHPRVALCEVCEQAPAHVTCKADAAALCLACD
RDIHSANPLASRHERLPVAPFYESVHSVKASSPINFLDDHRFFSDADADVSTEEAEAASWLLPTPKTDLNSSQYL
FSESEPVPYIDLDYSVADPKAEQKNSANADGVVPVQSNYEPFAYGYKYNSNLSQSQSQMSQSVSSSSMEVGVVPD
GNTMSEISNCSYSKVAAVTVTAQFSAADREARVLRYREKRKNRKFEKTIRYASRKAYAETRPRIKGRFAKRSDAD
PLSGYGVVPSC
>Mu_g08874.t1 hypothetical protein PHAVU_009G053300g [Phaseolus vulgaris]
MYAETGLLFPYLHNLSQELHQLEEYCKSQKYNAPMDDLVQSSAMSEYDLAAEGDLFKAPEPIIEEPIMDLDPMT
AAISMISCGEDVSNQGLKSTDIDILQNDQFLSEVFYECKKDLLEKAAIESPLSEILEIKVPLLNIDENSIQENKPLPDI
QLGKSVSSGSLSSMDWIRGAVMKPAFIDIPAMDFNAVYGMRRSFSEGDIKTLGNGNMNIVQSPLERPFLISNCTS
EERFQKLSRYRNKRTKRNFGRKIKYACRKALADSQPRIRGRFARTEECESKREMYAETGLLFPYLHNLSQELHQL
EEYCKSQKYNAPMGRCTLTNPPSGSVKGRPSTTDDLVQSSAMSEYDLAAEGDLFKAPEPIIEEPIMDLDPMTAAI
SMISCGEDVSNQGLKSTDIDILQNDQFLSEVFYECKKDLLEKAAIESPLSEILEIKVPLLNIDENSIQENKPLPDIQL
GKSVSSGSLSSMDWIRGAVMKPAFIDIPAMDFNAVYGMRRSFSEGDIKTLGNGNMNIVQSPLERPFLISNCTSEE
RFQKLSRYRNKRTKRNFGRKIKYACRKALADSQPRIRGRFARTEECESKRE
>Mu_g10928.t1 PREDICTED: protein CHLOROPLAST IMPORT APPARATUS 2 [Vigna angularis]
MSSCLTGAGGRTYGFDFEFVKSPSSTRTSHTSSSPSSTISESSNSPLAISTKKPRTPRKRPNQTYNEAAALLSTAY
PNLFSTKNLKTQGKFAKPASENFDYDSSELLLPFRVLDGSSSCFLLDQPGPKPMERPKVVSLQEKACGSPGEISSV
VNFNSLELNDDCEESLDAESILDEEIEEGIDSIMGSRVEEVSNDSVNNFPWIMPFGGKSDFPRPRVSALRHVDDG
NWWNFPAVDILQISPKIITKPPLVTAEKKMKKKKVATIAAAEKSVVVELKKAELPKPKQGLMLKLNYDDIRSAW

SDRGTPFADDSPLADMPENDVTARLSQIDLWWDNGGVREASVQRYKEKRRTRLFSKKIRYQVRKVNADRRPR
MKGRFVRRLNSSSNVHR
>Mu_g11021.t1 zinc finger protein CONSTANS-LIKE 5 [Vigna radiata var. radiata]
MGIERGGLKGFRSGWSVPPKPCDSCKLASAALFCRPDSAFLCIACDSKIHCANKLASRHERVWMCEVCEQAPAA
VTCKADAAALCVTCDSDIHSANPLARRHDRVPVEPFFDXXXXXXXXXXXXXXXXXXXXXXXXXXXXXXXXXXXXXX
WLIPNPNFGSKLMDAPDIKSKEIFYSDMDPFLDFDYSNSFHNSSAGNDSVVPVQTKPSLAPHLINNHHHQSEGCF
DIDFCRSKLSSFNYPSQSISQSVSSSSLDVGVVPDGNNVSDMSYSFGRNSSESSGIGLSGVSGGQGATQLCGMDREA
RVLRYREKRKNRKFEKTIRYASRKAYAETRPRIKGRFAKRTEIDSDVERLYSPGTASMMLESPYGVVPSF
>Mu_g11043.t1 zinc finger protein CONSTANS-LIKE 1 [Vigna radiata var. radiata]
MSSDLYTFDIPFPRHSDTDNMVSYDANGNLMFFSDPCSFPFLTGSPVEDLAQGNISNSLQPSFSSFSPQENESLCH
ANLVQPLSDGPNIKCEFSSFSALHGCDVTSEECQMGVDYSNNQHFLSQTCHASDSASKVIIQRSFSCNSFGGKPGF
PFEPHPDTPMDSSNFQWHALNSPEKSFFTGQMRRVCSTGDLQNTSQMESPLLEEAYFKVGRYSAEERKEKISKY
RAKRSQRKFNKIIKYACRKTLADNRTRIRGRFARNDEISEIPKAPSSTSTTEEYEHEFWVEFIEGLNEEVIG
>Mu_g11979.t1 PREDICTED: GATA transcription factor 28 isoform X1 [Vigna angularis]
MDDIHGGDSRIHISDGQHPIHVPYVQEHEHHGLHHMSNGNGIDEDQNDGGDTNCGGSENLESDVPSSHGNLTD
NHGVIIDQGGDAGDQLTLSFQGQVYVFDSVSPEKVQAVLLLLGGREIPPTMPALPVSPHHNNRGFTGTPQKFSVP
QRLASLIRFREKRKERNFDKKIRYTVRKEVALRMQRNKGQFTSSKPNHDESASTATNWGTNENWSAENNGSQ
QQDIVCRHCSISEKCTPMMRRGPEGPRTLCNACGLMWANKGTLRDLSRTAPISGPIKNENKSLEANQIIVHRVAG
EADHSS
>Mu_g12727.t1 hypothetical protein VIGAN_04028500 [Vigna angularis var. angularis]
MLEGETAGSWARTCDTCRSAASTVFCRAHNAYFCASCDTRAHHASSSSWHERVWVCEACERAPAAFLCKADA
ASLCSSCDADIHSANPLASRHHRVPILPIAAGHHSXXXXXXXXXXXXXXXXXXXXTASWLLLNPVKTGTVPNSNN
NGFLYSGEVDEYLDLVDNCNSCGDTNHFAATTTTATTTDHYTQHHHFDGVSQKSYAGDSVVPVQHHQHFQLGL
EFENSKAAFSYNGSISQSVSISSMDIGVVPESPMRDVSIAHPRPPKGTIDLFSGPPIQMPSHFSPMDREARVLRYRE
KKKTRKFEKTIRYASRKAYAETRPRIKGRFAKRTDVEAEVDQMFSTTLITEVGYGIVPSF
>Mu_g12967.t1 hypothetical protein VIGAN_07206000 [Vigna angularis var. angularis]
MASIPQFYPNYTFTTHDLSEFPTPLMSGNASVIDNAMWAGQDSLIPVLDMNNGALDHIVSLDCDTMACANWM
PSFSEQLGGLSDLAISDCKMGFYGGFQNYNSRYQPHIGEFGDECCGFVEDVKPPAYPNAARENWVCFNFVVGFLI
LFVSLMVLCQGGLQGNQMQAVEEPNIKVGRYSEEERKERILRYLKKRNQRNFNKTIKYACRKTLADRRVRVRGR
FARNNELCEEDMASKKHENHHHKEDFYGGDSIQFQLKNDEEDWLQEAMASLVYLSHSSPEDM
>Mu_g13779.t1 putative zinc finger protein CONSTANS-LIKE 11 [Vigna radiata var. radiata]
MIFVLWEMKKCELCKLSARVFYESDQASLCWDCDAKVHGANFLVARHSRNLLCRTCHSPTPWKAAGASLANA
ATVYDRCAASAAEELNGTVSGNDEEPDTEDNDITGEVDCDNQVVPWSSTLSPSASSSGSEWSSFISNRQTPDQLI
PVLCSLEQPKTWRFTNPTRVVSIWVTESFT
>Mu_g14624.t1 PREDICTED: protein TIFY 3B [Vigna radiata var. radiata]
MAGVNTEAGGGWKVFPSSAMETVPDSVDRRSENMEVHSTLADDGSVMHFSATRPVAVPSSGQIKVTPSPTKFT
ILYKGNMCIYEGIPAEKVREIMVIASAFAKSAEMKSGVPPLTSLIPKKPSSPQGNSTNFAKKSSIRRLQDEFPLARR
QSLQRFLEKRRSRFANKAPYALTKNMAHNIENNFCSDSTPDFGSLNYQKRNFSSALPL
>Mu_g15429.t1 PREDICTED: zinc finger protein CONSTANS-LIKE 9-like [Vigna angularis]
MVYCRSDAACLCLSCDRNVHSANALSRRHSRTLLCERCNSQPAFVRCVEEKISLCQNCDWLGHGASTSSSTHKR
QAISCYSGCPSAAELSSIWSFVLDITSISESTCEQELGLMSINENNNKSVGVPPENRNVSASDQVTDLPALDKSLVG
TSSMPESSSEPRIPDQPAGLANECLPKLYCPVTKCPALCEDDNLYDDFDMDEVDLNLENYEELFGVALSHSEELF
ENGGIDSLFGTKDMSAGDSNCQDAIAAEGSSVGLVNTTQPACSNAASADSILSTKTEPILCFTGRQAQSNLSFSGIT
GESSAAADYQDCGASSMLLMGEPPWFAPCPENSLQSANRSNAVLRYKEKKKARKFDKQVRYASRKARADVRRR
VKGRFVKAGDVYDYDPLSATRSC
>Mu_g15539.t1 GATA transcription factor 24 [Vigna radiata var. radiata]
MDTVNNPCQNGEEPLPLGMALPMQVDEGEQAAAVPSPLAHASSAVHARTSELTISFEGEVYVFPAVTPQKVQA
VLLLLGGQEMPNTAPTSDFLLQQNCQDIREINDPSRSSKLSRRIASLVRFREKRKERCFEKKIRYSCRKEVAQRM
HRKNGQFASLKEDYKSPAENWDSSNGTPCPESIERRCQHCGIGEKSTPAMRRGPAGPRSLCNACGLMWANKGT
LRDLTKTGRMTFEQNELDTSADIKPSKAEAEHSCAKQDKEGSPEETKPVQLDSKRSPEKTNEQFIIGTVESVTEN
LSIQVENHGLSLHEQDPLEDLADASGTEFEIPAGFDEQVDIDDSNMRTYWL
>Mu_g15958.t1 zinc finger protein CONSTANS-LIKE 16 [Vigna radiata var. radiata]
MKDAGALGAKTARACDSCISRRARWFCAADDAFLCHGCDTLVHSANQLASRHERVRLQTASSKVTTTTHAWH
SGFTRKARTPRHNNSKHFALQQRLKEEVLFNNSSILPLVPELGGEEQEPVVVDNEETEEQMLCRVPVFDNFDVR
TDDLDSFSDMDFAEFAADVESLLDKEDDEMSARVGGGVQGAMAKVKDEEEVDGDVACYLESVFDMTNDDAFH
WNSIESVLSDAREEKECVVASDGAVGEEGGTKRDIFLRLNYDEVITAWSSQGSSPWTTSNPPEFNSDYDFCLGLS
GVDGEIRSLRSHLDGGREARVSRYREKRRNRLFAKKIRYEVRKLNAEKRPRMKGRFVKRTCFVGANAFPAYQ
>Mu_g16176.t1 hypothetical protein PHAVU_005G113300g [Phaseolus vulgaris]

MEPLCEFCWVVRAVVYCKSDSARLCLHCDGCVHSANSLSRRHSRSLLCDKCNSEPAMIRCMDHKLSLCQGCDW
NPNDCSALGHRRRTLNCYTGCPSLAEFSRIWSFVFDADSSLGCWKPVSTLPKTESCNSQCMEQPDHNGGSFGLV
TDKLDEIESCVRYEPWMGQSHIIPSNPNYTPYNKEEAFFLPQDSNQPKECTNLAIRDGDGLCEGINVDNVPINLG
SADEIFGCSQAATRYHLEDGGMDCLLMDKNISVTESSSLIESALEASSSIQQDCVPFQSSRPDGSASVMQAMNTNC
SITIPSCSRNISLGFPQGQVHSNIPLQLANIVGENSSTEYQDCGISPMFLTGEFPWESNLEATCPQARDKAIIRYHE
KKKTRMFGKQIRYASRKARADTRKRVKGRFVKAGEAYDYDPLVTRDV
>Mu_g16345.t1 zinc finger protein CONSTANS-LIKE 16 [Vigna radiata var. radiata]
MSSTKNKGNALGAKTARACDSCIRNRARWYCAADDAFLCQACDSSVHSANLLARRHHRVCLKIVPYNNTNKNS
SSGISNIRAPSWHKGFTKKPRTPRHGKRAHKALKSPFDVVVPEVCPEDTNSNSHEESVNQLLYRVPATPSPEEGK
NDLLGCESEFEIAEFGAEVESLLGEGLESECVGMEELGLVDTKKLEEYGSGDCCVDGGNVKVEESQQVALKENDE
QKRKKMLLLRLDYEAVISAWTHQKSPWTTGAKPDLGTHQCWPHCMGTCGVELQHPCGEMGGLLGCQSAMGD
GGREARVLRYREKRRTRLFSKKIRYEVRKLNAEKRPRMKGRFVKRLH
>Mu_g16361.t1 hypothetical protein PHAVU_007G131300g [Phaseolus vulgaris]
MPFTKMSFSEGNMDEISSPLSARIFELCNAEFFPEALPNSEVTSSSNCCYEENSSYGTTTNIPLTVDVENKLNSNS
NTVTTSTSTTTTTNNTANSSNLSIIFDSQEEIDNDISASIDFSLSPSFNVPPFLPVTSQQEQFDFSSMQPQVQLQAC
SVVEGFSQYPTDSVAPLMGAPLPSVFEEDCISSVPSYVPLNPSSPCTYLTPGMPPYMPPGPLTTALSTDSSGFFGG
NILLGSELQTQELEYQGENGRMYCTDSIQRVFNPPDLQALGTESQQLVPGSGSSATLTTEISNLEDSSFKVGKLSV
EQRKEKINRYMKKRNERNFSKKIKYACRKTLADSRPRVRGRFAKNDDFGETNRTTSSNHEEDDEEEVVVKDED
DMVDSSDIFAHISGVNSFKYNYSIQSWI
>Mu_g16656.t1 PREDICTED: zinc finger protein CONSTANS-like [Vigna angularis]
MFAHSTTASPLPSPSSLFPDLTEFDTLSQLNNSDTNYYYNNNNNSNCSSAYSSYGGSPTSVASSNFMQRSVSSNSFN
YISNGTHHPLSALFAELLDSHDAPVRRVCSTGDLQTINGIQHNHLSDSPLSSESSMIIEGMNRACRYSPEEKKVRIE
RYRSKRNQRNFNKKIKYACRKTLADSRPRIRGRFARNDEIDKNTTTQWSQSGEEEDEEDENWVTILDSLVAANF
AQESQGSCSSYGLFY
>Mu_g18384.t1 zinc finger protein CONSTANS-LIKE 16 [Vigna radiata var. radiata]
MTNEMKEASALGARTARACESCLKVRARWYCAADDAFLCHGCDSIVHSANQLASRHERVKLQTASSKVSYSLT
PHNNNKVAWHSGFTRKARTPRHNNNKHSSVQQQQQQKKIHEEGEEEEVFFSNTISLPLVPELGSEEALLNDESE
EQLLCRVPVFDVELCSIYTEVKDEILAGEEAFDLENFSSEFLPSDMDLAEFAADVESLLGNGADEDSSEHVKGPEL
VLDCKEGDDEMDACVDGLGAKDAMVKVKDEEELDADIPCHLDSIVLDMNSEAVNWNDLVESESLAQEQEEEVS
KVGINKKGIFLRLNYEDVITAWASHGSPWTTGTPPKFNSDDCWPDLFLGPNGGDGQCCYGEMRSLRGHADGER
EARVSRYREKRRTRLFAKKIRYEVRKLNAEKRPRMKGRFVKRTSFVGVTA
>Mu_g18779.t1 hypothetical protein PHAVU_008G147400g [Phaseolus vulgaris]
MCNKNSPSSSDIQTTSIMKRTRKPTKRYQXXXXXXXXXXXXLKPHRRGNRTKTRRPKFVSLRLQLSDPKNMPPQP
NPKAQEQPQLNLFPLHPDHHDMHEEQNVALLFSAEGGATLSGLLEEDSTSPSSATTEGSLSALTCPAEDAGNWL
XXXXXXXXXXXXXXXXXXXXXXXXXXXXXXXXXXTSYCVTGKTTNNXXXXXXXSFGLLSLKLDHQQILNAWSDKG
SLYVAGEGAPHTVPDFLNGFLLHNALLPHVAWDGWGSGVVGNAWNVPEECAANKANVKEENGWKLGQREAS
VQRYKEKRQSRLFSKKIRYEVRKLNAEKRPRMKGRFVKRE
>Mu_g21270.t1 hypothetical protein PHAVU_008G182900g [Phaseolus vulgaris]
MNSTLWFAWMHRNKGQFTSKKQDGANNYGTDQDSGHDDSQSETSCTHCGISSKSTPMMRRGPSGPRSLCNAC
GLFWANRGALRDLSKRNHEHALVPVDQVDEGNDSDCRTATNPSHNNLAAYSENNNPALVTDRKVFQSQKMLE
MEPPAMYGHSQPLNMPPQISAAESDDGSGPDPALDGYHHIHYDSHALEDGAAGAVVVVEDVTSDAVYVSGGGG
PEESSQLTLSFRGQVYVFDAVTPDKVQAVLLLLGGCELSSSGSPCVDAVPQQSQRGSMEYPARCSLPQRAASLDR
FRQKRKERCFDKKVRYSVRQEVALRMHRNKGQFTSKKQDGANNYGTDQDSGHDDSQSETSCTHCGISSKSTPM
MRRGPSGPRSLCNACGLFWANRGALRDLSKRNHEHALVPVDQVDEGNDSDCRTATNPSHNNLAAYSENNNPA
LVTDRKVFQSQKMLE
>Mu_g22903.t1 hypothetical protein PHAVU_007G270600g [Phaseolus vulgaris]
MLFTMLICHHSLQDEISSPITARLFELCDPDFFPDTLQNSEVTSSSNCCHEEKSSYATTISPPLDVVXXXXXXXXXX
XXXXXXXXXXXXXXXXXXXXXXXXXXXLSIFFDSQDEIDNDISASIDFSSSPSFVVPPLLPITNQQDQFDFSSAQAQV
QLSAAGSVLKGLSQYSTDPVVAPLIGAPLASVFDDDCISSIPSYMPLNPSSPSCSYLSPGIGVYMPTPGSLSTALSAD
SSGLFGGNMLLGSELQAQELDYQGENGGIYCTDSIQRVFNPPDLQALGTESQNLVSGAGGSATLAPEISHLEDSTL
KVGKLSVEQRKEKIHRYMKKRNERNFSKKIKYACRKTLADSRPRVRGRFAKNDDFGESHRQGSSNHEEDDEEII
VKEDDDMVDSSDIFAHISGVNSFKCNYSIQSLI
>Mu_g22988.t1 PREDICTED: zinc finger protein CONSTANS-LIKE 3-like [Vigna angularis]
MTESCALCEKRAAMLCASDQAKLCWSCDEKVHSANFLVANHSRVLLCCSCHSPTPWKASGAKLTPTVTFCQSC
VGDGDARLQPLLNNVQQHGYCMNPNNDHGEERENRPLPIMSAASATSPPTSVILPCKLLRTHSFSIDSHAETACS
SSVNAAALATRDDDSTFSKVNEVGLSVHRSTSS
>Mu_g23480.t1 hypothetical protein PHAVU_009G035400g [Phaseolus vulgaris]
MYDPMNQIVPVEHNDPAASDHHIHYSSHTIEDVGAAVEDVSADSVYVPPPEISIQDSSQLTLSFRGQVYVFDAVT
PDKVQAVLLLLGGSELISGSQCTELSPQNHTGAAEFPARCSLPQRVASLNRFRQKRKERCFDKKVRYSVRQEVAL

RMHRNKGQFTSSKKQDGTNSWGSDQESGQEAVQSETSCTHCGTSSKSTPMMRRGPSGPRSLCNACGLFWANR
GTLRDLSKRNQEHSLAPPEQVHVGSNNDLDCRSALPAQHNNHVNDNKALVSDR
>Mu_g23481.t1 GATA transcription factor 24 isoform X1 [Vigna radiata var. radiata]
MASVNPQPLQFQDPAIPVXXXXXXXXXXXXXMDELEDAHVSSVNVVGNAASASREVVAAIPSRTSELTLSFEGEV
YVFPAVTPQKVQAVLLLLGGRDVQAGVPTVELPFDQSNRGMGDTPKRSNLSRRIASLVRFREKRKERCFDKKIR
YTVRKEVAQRMHRKNGQFASLKESPDSSNWDSARSSAQDGTSHSESVRRCHHCGVTENNTPAMRRGPAGPRT
LCNACGLMWANKGTLRDLSKGGRNLSIEQSDLDIPIDVKPTSVIEGELPGIQDEQGSSEDPSKSNTADGSSGHAV
NPSDEELPETAENFTKALPLGLDHSSINDTEQEPLVELSNPSDTDIDIPGNF
