# Supplementary material for: Genome, Transcriptome, and Germplasm Sequencing Uncovers Functional Variation in the Warm-Season Grain Legume Horsegram Macrotyloma uniflorum (Lam.) Verdc
Source: Front Plant Sci. 2021 Oct 18;12:758119. doi: 10.3389/fpls.2021.758119 (PMC8558620; doi:10.3389/fpls.2021.758119)
Supplement: Supplementary file 2 [file Data_Sheet_2.PDF]

Genome, Transcriptome, and Germplasm Sequencing Uncovers Functional Variation in the Warm-season Grain Legume Horsegram

***Macrotyloma uniflorum* (Lam.) Verdc.**

H. B. Mahesh[1,*], M K Prasannakumar[2], K. G. Manasa[1], Sampath Perumal[3,4], Yogendra Khedikar[3], Sateesh Kagale[5], Raju Y. Soolanayakanahally[3], H. C. Lohithaswa[1], Annabathula Mohan Rao[6] and Shailaja Hittalmani[6]

Supplementary Table S1. Divergence time estimated based on distributions of *Ks* (synonymous substitutions).

Divergence - Orthologous Ks

	**No. of duplicate pairs**	**G (# Gaussian components)**	**Chisquare**	**p-value**	**mean ln (Ks)**	**variance ln (Ks)**	**fraction of data**	**mean Ks**	**Main orthologous Ks peak**	**Divergence time (million years)***
Horsegram - Soybean	17924	3	110.3947168	3.74E-09	-1.4289052	0.04595189	0.53268009	0.239571061	0.24	14.43
					-1.0124808	0.21791987	0.45568819	0.363316545		
					0.2832131	2.55331582	0.01163172	1.327387998		
Horsegram - Barrel medic	14835	3	17.6200245	5.23E-32	-0.53235705	0.05145016	0.51906165	0.587219232	0.59	35.37
					-	0.19341	0.43088	0.98691		

					0.01316981	811	726	6532		
					1.54749347	1.89388617	0.05005109	4.699675529		
Horsegram - Common bean	17903	3	120.2774365	1.04E-04	-1.7785617	0.04827958	0.53502027	0.168880874	0.17	10.17
					-1.4958913	0.24696245	0.45435989	0.224048821		
					0.2357141	3.38082696	0.01061984	1.265812363		
Horsegram - Adzuki bean	17024	3	152.7349772	8.48E-09	-1.622994	0.04813041	0.56740146	0.197307076	0.20	11.89
					-1.291563	0.21890519	0.41663127	0.274840871		
					0.581352	4.22553279	0.01596727	1.788454788		
Horsegram - Mungbean	14751	3	152.0465392	3.08E-13	-1.5732044	0.05185789	0.58168562	0.207379589	0.21	12.49
					-1.1787707	0.24641069	0.4014414	0.307656709		
					0.7454514	3.16118017	0.01687298	2.107392497		

* The synonymous substitution rate of 8.3 x 10-9 was extrapolated using an established age of 19.2 million years for the divergence of Phaseolus

and Glycine of 19.2 Mya (Lavin et al., 2005)

Lavin, M., Herendeen, P. & Wojciechowski, M. (2005) Evolutionary Rates Analysis of Leguminosae Implicates a Rapid Diversification of Lineages during the Tertiary. Syst. Biol 54, 575–594.

Supplementary Table S2. Relative abundance of transcription factors in horsegram and other legumes.

**TFs families**	**Horsegram**	***Phaseolus vulgaris***	***Vigna angularis***	***Vigna radiata***	***Medicago***	***Glycine max***
AP2	26	31	27	21	37	99
ARF	25	31	32	24	65	107
ARR-B	18	17	16	10	46	55
B3	29	61	40	25	113	148
BBR-BPC	4	9	7	5	4	29
BES1	8	7	5	5	9	19
bHLH	165	203	192	153	259	548
bZIP	76	105	122	63	124	352
C2H2	106	137	135	104	112	321
C3H	42	52	46	32	90	150
CAMTA	7	9	12	8	20	24
CO-like	13	14	14	10	13	40
CPP	5	6	7	7	9	26
DBB	11	16	13	8	15	47
Dof	39	45	38	33	47	97
E2F/DP	7	14	5	7	12	38
EIL	12	7	6	5	13	13
ERF	148	155	253	113	197	338
FAR1	28	32	25	17	91	138
G2-like	49	61	65	45	77	222
GATA	29	35	40	24	53	92
GeBP	8	5	7	6	6	10
GRAS	63	63	88	55	75	151
GRF	6	12	9	7	12	42

HB-other	8	9	14	11	20	30
HB-PHD	3	4	3	1	5	16
HD-ZIP	55	61	59	49	58	180
HRT-like	1	2	1	1	3	1
HSF	30	35	19	22	36	81
LBD	47	53	62	40	67	125
LFY	1	1	1	1	1	2
LSD	4	7	7	3	9	18
M-type_MADS	35	49	51	19	99	87
MIKC_MADS	19	43	47	27	50	209
MYB	47	181	120	117	185	430
MYB_related	79	89	121	85	158	342
NAC	91	106	115	82	123	269
NF-X1	3	2	3	1	5	10
NF-YA	9	19	12	9	16	117
NF-YB	18	22	31	12	24	58
NF-YC	14	18	28	13	17	38
Nin-like	10	15	13	10	21	61
NZZ/SPL	1	2	0	0	0	0
RAV	3	3	7	1	3	5
S1Fa-like	2	2	1	1	4	4
SAP	2	2	1	1	1	2
SBP	20	29	28	23	28	111
SRS	11	13	10	9	12	42
STAT	1	1	0	0	1	2
TALE	33	47	35	21	34	133
TCP	30	31	37	24	27	81

Trihelix	34	47	37	32	41	104
VOZ	3	5	4	3	4	26
Whirly	3	4	4	4	3	18
WOX	17	18	25	18	21	41
WRKY	93	102	92	88	140	296
YABBY	8	9	11	9	8	47
ZF-HD	21	19	30	15	18	58

Supplementary Table S3. Simple sequence repeats predicted in horsegram genome.

Scaffold ID	SSR_ID	SSR type	SSR	size	scaffold_start	scaffold_end	SSR_Category
PHG9_Scaffold_1	1	p2	(AG)8	16	1641	1656	potentially variable
PHG9_Scaffold_1	2	p3	(TTC)5	15	5614	5628	potentially variable
PHG9_Scaffold_1	21	p2	(TA)7	14	88346	88359	potentially variable
PHG9_Scaffold_1	31	p2	(TA)6	12	109096	109107	
PHG9_Scaffold_1	35	p2	(TA)7	14	159240	159253	potentially variable
PHG9_Scaffold_1	41	p3	(AAT)5	15	217005	217019	potentially variable
PHG9_Scaffold_1	58	p2	(TA)7	14	303961	303974	potentially variable
PHG9_Scaffold_1	59	p3	(TTG)5	15	310218	310232	potentially variable
PHG9_Scaffold_1	65	p2	(TA)9	18	338510	338527	potentially variable

PHG9_Scaffold_1	73	p3	(ACC)5	15	430828	430842	potentially variable
PHG9_Scaffold_1	75	p2	(GA)6	12	440871	440882	
PHG9_Scaffold_1	77	p3	(GAG)5	15	460497	460511	potentially variable
PHG9_Scaffold_2	5	p2	(TA)7	14	11580	11593	potentially variable
PHG9_Scaffold_2	9	p3	(AAT)5	15	26584	26598	potentially variable
PHG9_Scaffold_2	10	p2	(AT)6	12	30004	30015	
PHG9_Scaffold_2	19	p3	(TAA)5	15	47359	47373	potentially variable
PHG9_Scaffold_2	21	p2	(CT)6	12	53014	53025	
PHG9_Scaffold_2	28	p2	(TA)8	16	58910	58925	potentially variable
PHG9_Scaffold_2	34	p3	(ATA)5	15	77254	77268	potentially variable
PHG9_Scaffold_2	36	p2	(AT)6	12	78816	78827	
PHG9_Scaffold_2	37	p3	(TCA)5	15	79845	79859	potentially variable
PHG9_Scaffold_2	38	p3	(TAT)5	15	83178	83192	potentially variable
PHG9_Scaffold_2	45	p2	(TA)6	12	88278	88289	
PHG9_Scaffold_2	46	p3	(TTA)5	15	88886	88900	potentially variable
PHG9_Scaffold_2	51	p3	(TAT)5	15	125894	125908	potentially variable
PHG9_Scaffold_2	52	p2	(TA)7	14	130108	130121	potentially variable
PHG9_Scaffold_2	55	p3	(TGA)5	15	141853	141867	potentially

							variable
PHG9_Scaffold_3	3	p3	(ATC)5	15	11198	11212	potentially variable
PHG9_Scaffold_3	13	p2	(AT)7	14	39446	39459	potentially variable
PHG9_Scaffold_3	19	p2	(GT)7	14	58233	58246	potentially variable
PHG9_Scaffold_3	31	p2	(AC)9	18	96581	96598	potentially variable
PHG9_Scaffold_3	32	p2	(TA)8	16	99964	99979	potentially variable
PHG9_Scaffold_3	36	p3	(AAG)5	15	132028	132042	potentially variable
PHG9_Scaffold_3	37	p2	(AT)6	12	132424	132435	
PHG9_Scaffold_3	38	p2	(AT)8	16	133646	133661	potentially variable
PHG9_Scaffold_4	5	p2	(AT)8	16	2757	2772	potentially variable
PHG9_Scaffold_4	7	p3	(TTC)5	15	6537	6551	potentially variable
PHG9_Scaffold_4	20	p2	(AT)6	12	54658	54669	
PHG9_Scaffold_4	21	p2	(AG)7	14	59263	59276	potentially variable
PHG9_Scaffold_4	31	p2	(TA)9	18	99639	99656	potentially variable
PHG9_Scaffold_6	5	p6	(GAACCA)5	30	9111	9140	Hypervariable
PHG9_Scaffold_6	11	p3	(AAT)5	15	39452	39466	potentially variable
PHG9_Scaffold_6	12	p2	(TA)6	12	40579	40590	
PHG9_Scaffold_6	14	p2	(AT)6	12	47978	47989	

PHG9_Scaffold_6	16	p2	(CT)9	18	54872	54889	potentially variable
PHG9_Scaffold_6	23	p3	(AAC)5	15	83628	83642	potentially variable
PHG9_Scaffold_6	24	p2	(TA)9	18	86712	86729	potentially variable
PHG9_Scaffold_6	32	p3	(GAA)5	15	102716	102730	potentially variable
PHG9_Scaffold_6	36	p3	(CTT)6	18	110127	110144	potentially variable
PHG9_Scaffold_6	38	p2	(TG)7	14	110795	110808	potentially variable
PHG9_Scaffold_6	48	p2	(AT)7	14	139811	139824	potentially variable
PHG9_Scaffold_6	55	p3	(TTA)5	15	172225	172239	potentially variable
PHG9_Scaffold_6	56	p3	(TAT)5	15	173879	173893	potentially variable
PHG9_Scaffold_6	57	p2	(AT)6	12	179803	179814	
PHG9_Scaffold_6	58	p2	(AT)7	14	181098	181111	potentially variable
PHG9_Scaffold_7	3	p2	(CA)6	12	4360	4371	
PHG9_Scaffold_7	5	p2	(TA)6	12	7545	7556	
PHG9_Scaffold_7	6	p2	(AT)7	14	7961	7974	potentially variable
PHG9_Scaffold_7	8	p3	(TAT)6	18	12852	12869	potentially variable
PHG9_Scaffold_7	15	p2	(TA)7	14	27676	27689	potentially variable
PHG9_Scaffold_7	20	p2	(TA)6	12	60475	60486	

PHG9_Scaffold_7	25	p2	(AG)7	14	71663	71676	potentially variable
PHG9_Scaffold_7	31	p2	(AT)9	18	97156	97173	potentially variable
PHG9_Scaffold_7	39	p2	(TA)7	14	125800	125813	potentially variable
PHG9_Scaffold_7	40	p2	(AT)6	12	126658	126669	
PHG9_Scaffold_7	42	p3	(ATT)5	15	128108	128122	potentially variable
PHG9_Scaffold_7	46	p2	(AG)6	12	136493	136504	
PHG9_Scaffold_7	50	p2	(TA)7	14	144238	144251	potentially variable
PHG9_Scaffold_8	3	p2	(TA)6	12	8079	8090	
PHG9_Scaffold_8	7	p2	(TC)6	12	19843	19854	
PHG9_Scaffold_9	7	p3	(AAT)5	15	29114	29128	potentially variable
PHG9_Scaffold_9	9	p3	(AAT)6	18	30665	30682	potentially variable
PHG9_Scaffold_9	13	p2	(AG)6	12	40920	40931	
PHG9_Scaffold_9	17	p3	(AAG)5	15	52620	52634	potentially variable
PHG9_Scaffold_9	18	p2	(CT)6	12	53013	53024	
PHG9_Scaffold_9	28	p2	(AT)7	14	93084	93097	potentially variable
PHG9_Scaffold_9	33	p3	(ATC)6	18	104943	104960	potentially variable
PHG9_Scaffold_9	34	p2	(CA)8	16	106848	106863	potentially variable
PHG9_Scaffold_9	36	p3	(TCT)5	15	116526	116540	potentially variable

PHG9_Scaffold_9	48	p3	(AGA)5	15	142606	142620	potentially variable
PHG9_Scaffold_9	52	p2	(AG)7	14	144371	144384	potentially variable
PHG9_Scaffold_9	54	p3	(GAT)5	15	150059	150073	potentially variable
PHG9_Scaffold_9	59	p2	(TA)6	12	158837	158848	
PHG9_Scaffold_9	60	p2	(CT)6	12	159729	159740	
PHG9_Scaffold_9	61	p3	(TCT)5	15	159864	159878	potentially variable
PHG9_Scaffold_9	70	p2	(TA)7	14	182105	182118	potentially variable
PHG9_Scaffold_9	74	p2	(AC)9	18	200892	200909	potentially variable
PHG9_Scaffold_9	90	p2	(AT)7	14	236410	236423	potentially variable
PHG9_Scaffold_9	91	p3	(TTA)5	15	237450	237464	potentially variable
PHG9_Scaffold_9	99	p2	(TA)9	18	262366	262383	potentially variable
PHG9_Scaffold_10	6	p3	(TAT)5	15	9278	9292	potentially variable
PHG9_Scaffold_10	11	p2	(AG)6	12	32950	32961	
PHG9_Scaffold_10	12	p2	(CT)6	12	33358	33369	
PHG9_Scaffold_10	17	p2	(AT)9	18	39449	39466	potentially variable
PHG9_Scaffold_11	1	p3	(ATC)5	15	1036	1050	potentially variable
PHG9_Scaffold_11	13	p2	(CT)6	12	39220	39231	
PHG9_Scaffold_11	17	p3	(AAT)5	15	47302	47316	potentially

								variable
PHG9_Scaffold_11	18	p2	(TA)6	12	49551	49562		
PHG9_Scaffold_11	19	p2	(AG)8	16	51358	51373	potentially variable	
PHG9_Scaffold_11	28	p2	(TA)9	18	64670	64687	potentially variable	
PHG9_Scaffold_11	37	p2	(TG)6	12	76960	76971		
PHG9_Scaffold_11	40	p3	(TTA)5	15	80984	80998	potentially variable	
PHG9_Scaffold_11	41	p3	(CAA)6	18	81361	81378	potentially variable	
PHG9_Scaffold_11	49	p2	(AT)7	14	106014	106027	potentially variable	
PHG9_Scaffold_11	53	p2	(AG)8	16	124635	124650	potentially variable	
PHG9_Scaffold_12	6	p2	(TA)6	12	14328	14339		
PHG9_Scaffold_12	12	p3	(ATA)5	15	32703	32717	potentially variable	
PHG9_Scaffold_12	23	p3	(CAT)5	15	70395	70409	potentially variable	
PHG9_Scaffold_12	32	p2	(TA)7	14	99350	99363	potentially variable	
PHG9_Scaffold_13	4	p2	(AT)7	14	1602	1615	potentially variable	
PHG9_Scaffold_13	5	p3	(ATA)5	15	6883	6897	potentially variable	
PHG9_Scaffold_13	10	p2	(AT)7	14	26030	26043	potentially variable	
PHG9_Scaffold_13	14	p2	(AT)9	18	45495	45512	potentially variable	

PHG9_Scaffold_13	20	p3	(AGG)5	15	61399	61413	potentially variable
PHG9_Scaffold_13	21	p3	(TTC)5	15	63217	63231	potentially variable
PHG9_Scaffold_13	24	p2	(TA)7	14	68043	68056	potentially variable
PHG9_Scaffold_13	25	p2	(AG)7	14	69717	69730	potentially variable
PHG9_Scaffold_13	28	p3	(TGG)6	18	73788	73805	potentially variable
PHG9_Scaffold_13	41	p3	(GAA)5	15	104160	104174	potentially variable
PHG9_Scaffold_13	45	p2	(TG)6	12	114775	114786	
PHG9_Scaffold_13	48	p2	(AT)8	16	122779	122794	potentially variable
PHG9_Scaffold_13	49	p2	(TA)9	18	125019	125036	potentially variable
PHG9_Scaffold_13	59	p2	(GC)6	12	142203	142214	
PHG9_Scaffold_13	63	p2	(TA)7	14	147152	147165	potentially variable
PHG9_Scaffold_13	70	p2	(CA)6	12	173307	173318	
PHG9_Scaffold_13	90	p2	(GT)6	12	241063	241074	
PHG9_Scaffold_13	92	p3	(ATT)5	15	259842	259856	potentially variable
PHG9_Scaffold_13	97	p2	(AT)6	12	269004	269015	
PHG9_Scaffold_13	99	p3	(GAA)5	15	278010	278024	potentially variable
PHG9_Scaffold_13	100	p2	(GA)6	12	279803	279814	
PHG9_Scaffold_13	103	p2	(TA)6	12	291583	291594	

PHG9_Scaffold_14	1	p2	(TA)7	14	15524	15537	potentially variable
PHG9_Scaffold_14	4	p2	(TA)6	12	29235	29246	
PHG9_Scaffold_14	18	p2	(TA)6	12	61148	61159	
PHG9_Scaffold_14	28	p3	(TTC)6	18	88329	88346	potentially variable
PHG9_Scaffold_14	30	p3	(AGA)5	15	97910	97924	potentially variable
PHG9_Scaffold_14	32	p3	(AGA)5	15	108019	108033	potentially variable
PHG9_Scaffold_14	41	p3	(ATT)6	18	155064	155081	potentially variable
PHG9_Scaffold_14	43	p2	(TC)8	16	177018	177033	potentially variable
PHG9_Scaffold_14	48	p2	(TA)6	12	190730	190741	
PHG9_Scaffold_14	49	p2	(TA)7	14	191446	191459	potentially variable
PHG9_Scaffold_14	50	p2	(TC)8	16	192228	192243	potentially variable
PHG9_Scaffold_14	53	p2	(AG)6	12	214884	214895	
PHG9_Scaffold_14	55	p3	(TAT)5	15	220634	220648	potentially variable
PHG9_Scaffold_14	60	p2	(TC)6	12	239695	239706	
PHG9_Scaffold_14	62	p3	(ATT)6	18	256280	256297	potentially variable
PHG9_Scaffold_14	64	p3	(TAT)5	15	264749	264763	potentially variable
PHG9_Scaffold_14	65	p2	(AT)9	18	281528	281545	potentially variable
PHG9_Scaffold_14	68	p3	(TAT)5	15	291831	291845	potentially

							variable
PHG9_Scaffold_14	70	p3	(TAA)5	15	328189	328203	potentially variable
PHG9_Scaffold_15	3	p2	(AT)6	12	2986	2997	
PHG9_Scaffold_15	12	p3	(AAG)5	15	38020	38034	potentially variable
PHG9_Scaffold_15	13	p2	(CA)7	14	50117	50130	potentially variable
PHG9_Scaffold_15	19	p2	(TA)7	14	60506	60519	potentially variable
PHG9_Scaffold_15	23	p2	(AT)6	12	87617	87628	
PHG9_Scaffold_15	28	p2	(CT)6	12	98779	98790	
PHG9_Scaffold_15	37	p2	(TA)6	12	148272	148283	
PHG9_Scaffold_15	44	p2	(TA)7	14	158828	158841	potentially variable
PHG9_Scaffold_15	46	p2	(AG)9	18	167949	167966	potentially variable
PHG9_Scaffold_15	48	p3	(CCT)5	15	171036	171050	potentially variable
PHG9_Scaffold_15	49	p6	(TGAATG)5	30	173339	173368	Hypervariable
PHG9_Scaffold_15	50	p3	(ACA)5	15	175159	175173	potentially variable
PHG9_Scaffold_15	52	p2	(TA)6	12	184471	184482	
PHG9_Scaffold_15	54	p2	(TA)9	18	194866	194883	potentially variable
PHG9_Scaffold_15	58	p2	(AG)6	12	216762	216773	
PHG9_Scaffold_15	59	p3	(GCG)5	15	218651	218665	potentially variable
PHG9_Scaffold_15	66	p2	(AT)6	12	253298	253309	

PHG9_Scaffold_16	2	p2	(AT)6	12	8131	8142	
PHG9_Scaffold_16	3	p2	(AC)8	16	10381	10396	potentially variable
PHG9_Scaffold_16	4	p3	(ACA)6	18	11341	11358	potentially variable
PHG9_Scaffold_16	13	p2	(TA)8	16	28223	28238	potentially variable
PHG9_Scaffold_16	15	p2	(AT)6	12	34336	34347	
PHG9_Scaffold_16	21	p2	(AT)7	14	50167	50180	potentially variable
PHG9_Scaffold_16	27	p3	(GGT)5	15	59907	59921	potentially variable
PHG9_Scaffold_16	28	p2	(TC)6	12	61736	61747	
PHG9_Scaffold_17	2	p2	(TA)7	14	419	432	potentially variable
PHG9_Scaffold_17	3	p2	(TA)8	16	3028	3043	potentially variable
PHG9_Scaffold_17	10	p2	(TA)6	12	29027	29038	
PHG9_Scaffold_17	15	p2	(TA)8	16	49232	49247	potentially variable
PHG9_Scaffold_17	19	p3	(ATC)5	15	57883	57897	potentially variable
PHG9_Scaffold_17	23	p2	(AT)6	12	63493	63504	
PHG9_Scaffold_17	24	p3	(AAT)5	15	66020	66034	potentially variable
PHG9_Scaffold_17	26	p2	(TA)7	14	67528	67541	potentially variable
PHG9_Scaffold_17	27	p2	(AT)7	14	70187	70200	potentially variable
PHG9_Scaffold_17	28	p2	(TA)6	12	70728	70739	

PHG9_Scaffold_17	32	p2	(TA)6	12	95041	95052	
PHG9_Scaffold_17	35	p2	(GA)7	14	99222	99235	potentially variable
PHG9_Scaffold_17	37	p2	(GA)6	12	101500	101511	
PHG9_Scaffold_17	40	p3	(AAT)5	15	106711	106725	potentially variable
PHG9_Scaffold_17	44	p2	(TA)6	12	129414	129425	
PHG9_Scaffold_17	50	p3	(TGA)5	15	136124	136138	potentially variable
PHG9_Scaffold_17	52	p2	(AT)7	14	138724	138737	potentially variable
PHG9_Scaffold_17	55	p6	(AGGTGG)6	36	149704	149739	Hypervariable
PHG9_Scaffold_17	59	p3	(AAT)5	15	165861	165875	potentially variable
PHG9_Scaffold_17	61	p2	(AT)7	14	171204	171217	potentially variable
PHG9_Scaffold_18	3	p3	(ATT)6	18	6290	6307	potentially variable
PHG9_Scaffold_18	4	p3	(GAC)5	15	11019	11033	potentially variable
PHG9_Scaffold_18	6	p2	(TC)8	16	13404	13419	potentially variable
PHG9_Scaffold_18	7	p3	(AGC)5	15	14509	14523	potentially variable
PHG9_Scaffold_18	12	p2	(TA)6	12	25963	25974	
PHG9_Scaffold_19	1	p2	(AG)6	12	513	524	
PHG9_Scaffold_19	2	p3	(ACA)5	15	2285	2299	potentially variable
PHG9_Scaffold_19	14	p3	(AGC)5	15	32296	32310	potentially variable

PHG9_Scaffold_19	19	p3	(TTC)6	18	53656	53673	potentially variable
PHG9_Scaffold_19	28	p2	(AG)8	16	75168	75183	potentially variable
PHG9_Scaffold_19	32	p2	(AT)8	16	82564	82579	potentially variable
PHG9_Scaffold_19	37	p2	(TG)6	12	107569	107580	
PHG9_Scaffold_20	3	p3	(AAT)5	15	3383	3397	potentially variable
PHG9_Scaffold_20	14	p2	(TA)7	14	44812	44825	potentially variable
PHG9_Scaffold_20	17	p2	(TA)8	16	48498	48513	potentially variable
PHG9_Scaffold_20	24	p2	(TA)8	16	55808	55823	potentially variable
PHG9_Scaffold_20	28	p2	(AT)8	16	72730	72745	potentially variable
PHG9_Scaffold_20	30	p2	(AG)8	16	80742	80757	potentially variable
PHG9_Scaffold_20	35	p2	(TA)6	12	95208	95219	
PHG9_Scaffold_20	37	p2	(TA)6	12	96107	96118	
PHG9_Scaffold_20	49	p2	(AT)7	14	146604	146617	potentially variable
PHG9_Scaffold_21	25	p3	(TCT)5	15	91460	91474	potentially variable
PHG9_Scaffold_21	30	p3	(AAG)5	15	96252	96266	potentially variable
PHG9_Scaffold_21	32	p2	(AT)7	14	101685	101698	potentially variable
PHG9_Scaffold_21	54	p2	(AT)6	12	135379	135390	

PHG9_Scaffold_21	55	p3	(GTT)5	15	138030	138044	potentially variable
PHG9_Scaffold_21	68	p3	(ATA)5	15	164757	164771	potentially variable
PHG9_Scaffold_21	74	p2	(AG)7	14	173075	173088	potentially variable
PHG9_Scaffold_21	75	p3	(TAT)5	15	175355	175369	potentially variable
PHG9_Scaffold_21	78	p3	(ATT)5	15	180004	180018	potentially variable
PHG9_Scaffold_21	80	p2	(AT)7	14	182476	182489	potentially variable
PHG9_Scaffold_21	84	p2	(TG)8	16	191981	191996	potentially variable
PHG9_Scaffold_22	1	p2	(AG)8	16	8990	9005	potentially variable
PHG9_Scaffold_22	6	p3	(TAT)6	18	29070	29087	potentially variable
PHG9_Scaffold_23	9	p2	(TC)7	14	33382	33395	potentially variable
PHG9_Scaffold_23	10	p2	(AT)8	16	35254	35269	potentially variable
PHG9_Scaffold_23	13	p2	(TA)6	12	56387	56398	
PHG9_Scaffold_23	17	p2	(AT)8	16	84242	84257	potentially variable
PHG9_Scaffold_23	23	p3	(TAA)5	15	148679	148693	potentially variable
PHG9_Scaffold_23	32	p2	(TA)6	12	204795	204806	
PHG9_Scaffold_23	33	p3	(TAA)6	18	206248	206265	potentially variable

PHG9_Scaffold_24	15	p3	(ATA)6	18	68551	68568	potentially variable
PHG9_Scaffold_24	16	p2	(TA)7	14	70985	70998	potentially variable
PHG9_Scaffold_24	17	p2	(GA)6	12	71270	71281	
PHG9_Scaffold_24	21	p2	(TA)6	12	75626	75637	
PHG9_Scaffold_24	25	p3	(GAT)5	15	92648	92662	potentially variable
PHG9_Scaffold_24	51	p2	(TA)6	12	157228	157239	
PHG9_Scaffold_24	57	p2	(GA)7	14	168634	168647	potentially variable
PHG9_Scaffold_24	59	p2	(AC)6	12	175348	175359	
PHG9_Scaffold_24	61	p2	(AT)7	14	182369	182382	potentially variable
PHG9_Scaffold_24	67	p2	(TA)8	16	198832	198847	potentially variable
PHG9_Scaffold_24	74	p2	(TA)7	14	220245	220258	potentially variable
PHG9_Scaffold_24	76	p2	(CT)7	14	239786	239799	potentially variable
PHG9_Scaffold_24	78	p2	(AT)6	12	244249	244260	
PHG9_Scaffold_25	2	p3	(TCT)6	18	11990	12007	potentially variable
PHG9_Scaffold_25	24	p2	(TA)6	12	73141	73152	
PHG9_Scaffold_25	42	p2	(TC)9	18	97210	97227	potentially variable
PHG9_Scaffold_26	4	p3	(TTA)5	15	22649	22663	potentially variable
PHG9_Scaffold_26	8	p2	(AT)6	12	29966	29977	

PHG9_Scaffold_26	17	p2	(CT)9	18	63296	63313	potentially variable
PHG9_Scaffold_26	18	p2	(AT)8	16	71371	71386	potentially variable
PHG9_Scaffold_27	8	p2	(AT)6	12	16281	16292	
PHG9_Scaffold_27	10	p3	(TAT)5	15	25778	25792	potentially variable
PHG9_Scaffold_27	12	p3	(CAT)5	15	29502	29516	potentially variable
PHG9_Scaffold_27	14	p2	(TA)6	12	42923	42934	
PHG9_Scaffold_27	33	p2	(AT)9	18	107378	107395	potentially variable
PHG9_Scaffold_27	40	p2	(GA)7	14	119110	119123	potentially variable
PHG9_Scaffold_27	41	p2	(AG)6	12	121371	121382	
PHG9_Scaffold_28	1	p2	(TA)6	12	4326	4337	
PHG9_Scaffold_28	3	p2	(AT)7	14	25219	25232	potentially variable
PHG9_Scaffold_28	6	p2	(AT)8	16	26829	26844	potentially variable
PHG9_Scaffold_28	9	p2	(AG)8	16	42828	42843	potentially variable
PHG9_Scaffold_28	10	p2	(TC)9	18	44154	44171	potentially variable
PHG9_Scaffold_28	12	p3	(ATG)5	15	47888	47902	potentially variable
PHG9_Scaffold_28	16	p3	(AGA)5	15	66816	66830	potentially variable
PHG9_Scaffold_28	21	p2	(TG)6	12	81091	81102	
PHG9_Scaffold_28	24	p2	(TG)7	14	98404	98417	potentially

							variable
PHG9_Scaffold_28	32	p2	(TA)9	18	116283	116300	potentially variable
PHG9_Scaffold_28	43	p2	(CT)6	12	163054	163065	
PHG9_Scaffold_28	46	p3	(TAA)5	15	167966	167980	potentially variable
PHG9_Scaffold_28	51	p2	(AG)6	12	192638	192649	
PHG9_Scaffold_28	71	p2	(GA)9	18	253764	253781	potentially variable
PHG9_Scaffold_28	74	p3	(TGG)6	18	264387	264404	potentially variable
PHG9_Scaffold_29	4	p3	(TCG)5	15	13525	13539	potentially variable
PHG9_Scaffold_29	8	p2	(TA)8	16	17721	17736	potentially variable
PHG9_Scaffold_29	12	p3	(ATC)5	15	35371	35385	potentially variable
PHG9_Scaffold_29	13	p2	(TA)6	12	36182	36193	
PHG9_Scaffold_29	21	p3	(TTA)5	15	53779	53793	potentially variable
PHG9_Scaffold_30	6	p2	(TC)8	16	15409	15424	potentially variable
PHG9_Scaffold_30	23	p3	(TGA)6	18	66208	66225	potentially variable
PHG9_Scaffold_30	35	p2	(TA)7	14	99996	100009	potentially variable
PHG9_Scaffold_31	1	p2	(TC)6	12	394	405	
PHG9_Scaffold_31	2	p2	(TG)8	16	2613	2628	potentially variable
PHG9_Scaffold_31	4	p2	(CT)9	18	40593	40610	potentially

							variable
PHG9_Scaffold_31	5	p2	(GT)9	18	41783	41800	potentially variable
PHG9_Scaffold_31	6	p2	(AG)8	16	46460	46475	potentially variable
PHG9_Scaffold_31	7	p2	(AG)7	14	63914	63927	potentially variable
PHG9_Scaffold_31	16	p2	(AT)6	12	101181	101192	
PHG9_Scaffold_31	23	p2	(AG)7	14	112703	112716	potentially variable
PHG9_Scaffold_31	24	p3	(ATA)5	15	114138	114152	potentially variable
PHG9_Scaffold_31	25	p2	(TA)6	12	124103	124114	
PHG9_Scaffold_31	27	p2	(AC)7	14	125886	125899	potentially variable
PHG9_Scaffold_31	28	p2	(TA)7	14	126159	126172	potentially variable
PHG9_Scaffold_31	29	p2	(AT)9	18	126328	126345	potentially variable
PHG9_Scaffold_31	30	p2	(AT)9	18	126574	126591	potentially variable
PHG9_Scaffold_31	48	p2	(CT)9	18	160793	160810	potentially variable
PHG9_Scaffold_31	56	p2	(TA)7	14	169291	169304	potentially variable
PHG9_Scaffold_31	68	p2	(CT)9	18	213275	213292	potentially variable
PHG9_Scaffold_31	77	p2	(TA)7	14	229895	229908	potentially variable
PHG9_Scaffold_31	79	p2	(AG)6	12	239987	239998	

PHG9_Scaffold_31	82	p2	(TA)7	14	242959	242972	potentially variable
PHG9_Scaffold_31	83	p2	(GA)7	14	243399	243412	potentially variable
PHG9_Scaffold_31	86	p3	(AAT)5	15	252647	252661	potentially variable
PHG9_Scaffold_31	89	p3	(TTA)5	15	257914	257928	potentially variable
PHG9_Scaffold_31	96	p2	(TA)7	14	300139	300152	potentially variable
PHG9_Scaffold_31	114	p3	(AAT)6	18	343302	343319	potentially variable
PHG9_Scaffold_31	120	p2	(AT)7	14	366512	366525	potentially variable
PHG9_Scaffold_31	121	p3	(TAT)6	18	366802	366819	potentially variable
PHG9_Scaffold_32	9	p3	(TGA)5	15	33331	33345	potentially variable
PHG9_Scaffold_32	26	p2	(TC)7	14	66330	66343	potentially variable
PHG9_Scaffold_32	28	p2	(AT)9	18	69720	69737	potentially variable
PHG9_Scaffold_32	42	p3	(TAT)6	18	115818	115835	potentially variable
PHG9_Scaffold_32	52	p2	(AT)9	18	139338	139355	potentially variable
PHG9_Scaffold_32	53	p2	(GT)6	12	141023	141034	
PHG9_Scaffold_32	65	p2	(AT)6	12	174260	174271	
PHG9_Scaffold_32	66	p2	(GT)6	12	178640	178651	
PHG9_Scaffold_32	73	p3	(ATA)5	15	187178	187192	potentially

							variable
PHG9_Scaffold_32	74	p3	(ACC)5	15	187450	187464	potentially variable
PHG9_Scaffold_32	81	p2	(AT)9	18	204861	204878	potentially variable
PHG9_Scaffold_32	88	p2	(TA)8	16	245984	245999	potentially variable
PHG9_Scaffold_32	89	p2	(CT)6	12	248674	248685	
PHG9_Scaffold_32	90	p2	(TA)6	12	252480	252491	
PHG9_Scaffold_32	93	p3	(TTA)6	18	269180	269197	potentially variable
PHG9_Scaffold_33	2	p3	(AGA)6	18	6105	6122	potentially variable
PHG9_Scaffold_33	6	p2	(CT)6	12	9625	9636	
PHG9_Scaffold_33	9	p3	(TAA)5	15	20279	20293	potentially variable
PHG9_Scaffold_33	13	p2	(TA)6	12	28904	28915	
PHG9_Scaffold_33	30	p3	(TTC)6	18	84402	84419	potentially variable
PHG9_Scaffold_33	35	p2	(TA)6	12	94559	94570	
PHG9_Scaffold_33	36	p2	(TA)9	18	98368	98385	potentially variable
PHG9_Scaffold_33	67	p3	(ATT)5	15	177253	177267	potentially variable
PHG9_Scaffold_33	69	p2	(TA)9	18	180273	180290	potentially variable
PHG9_Scaffold_34	7	p2	(TC)6	12	23303	23314	
PHG9_Scaffold_34	15	p2	(GT)8	16	50612	50627	potentially variable

PHG9_Scaffold_34	16	p2	(GA)6	12	50991	51002	
PHG9_Scaffold_34	17	p3	(GAT)6	18	55223	55240	potentially variable
PHG9_Scaffold_34	21	p2	(TC)8	16	59254	59269	potentially variable
PHG9_Scaffold_34	32	p3	(TAA)5	15	92086	92100	potentially variable
PHG9_Scaffold_34	34	p3	(ATA)5	15	109896	109910	potentially variable
PHG9_Scaffold_34	42	p2	(CA)6	12	127038	127049	
PHG9_Scaffold_34	44	p3	(TTC)6	18	133743	133760	potentially variable
PHG9_Scaffold_34	52	p3	(CAT)5	15	165052	165066	potentially variable
PHG9_Scaffold_34	56	p2	(AG)6	12	194246	194257	
PHG9_Scaffold_34	57	p3	(ATC)5	15	194628	194642	potentially variable
PHG9_Scaffold_34	60	p3	(TAA)5	15	197954	197968	potentially variable
PHG9_Scaffold_34	61	p2	(TA)6	12	199255	199266	
PHG9_Scaffold_34	65	p3	(TAA)5	15	203643	203657	potentially variable
PHG9_Scaffold_35	3	p3	(CTT)5	15	18807	18821	potentially variable
PHG9_Scaffold_35	9	p2	(TC)7	14	32883	32896	potentially variable
PHG9_Scaffold_35	15	p2	(AT)7	14	45472	45485	potentially variable
PHG9_Scaffold_35	17	p2	(TA)7	14	59532	59545	potentially variable

PHG9_Scaffold_35	26	p2	(TA)7	14	83272	83285	potentially variable
PHG9_Scaffold_35	31	p2	(TA)6	12	91090	91101	
PHG9_Scaffold_35	40	p2	(TA)6	12	111695	111706	
PHG9_Scaffold_35	47	p3	(ATG)6	18	124873	124890	potentially variable
PHG9_Scaffold_35	48	p2	(GA)6	12	126867	126878	
PHG9_Scaffold_35	51	p2	(TA)9	18	131545	131562	potentially variable
PHG9_Scaffold_35	56	p2	(AT)8	16	142346	142361	potentially variable
PHG9_Scaffold_35	71	p2	(AC)7	14	176634	176647	potentially variable
PHG9_Scaffold_35	74	p2	(AT)6	12	188615	188626	
PHG9_Scaffold_35	76	p2	(TA)8	16	195850	195865	potentially variable
PHG9_Scaffold_35	85	p2	(AC)6	12	239887	239898	
PHG9_Scaffold_36	1	p2	(AT)6	12	1323	1334	
PHG9_Scaffold_36	3	p2	(TA)9	18	8004	8021	potentially variable
PHG9_Scaffold_36	6	p2	(TA)7	14	21120	21133	potentially variable
PHG9_Scaffold_36	7	p2	(AT)8	16	21349	21364	potentially variable
PHG9_Scaffold_36	11	p2	(TA)9	18	29288	29305	potentially variable
PHG9_Scaffold_36	12	p3	(ATT)5	15	30108	30122	potentially variable
PHG9_Scaffold_36	27	p3	(TGT)5	15	93645	93659	potentially variable

PHG9_Scaffold_36	28	p2	(AT)7	14	94965	94978	potentially variable
PHG9_Scaffold_36	29	p2	(CT)7	14	95514	95527	potentially variable
PHG9_Scaffold_36	31	p2	(AT)7	14	101077	101090	potentially variable
PHG9_Scaffold_37	4	p2	(TA)6	12	19428	19439	
PHG9_Scaffold_37	32	p2	(TA)7	14	86138	86151	potentially variable
PHG9_Scaffold_37	36	p3	(GAT)5	15	94428	94442	potentially variable
PHG9_Scaffold_37	40	p2	(TG)6	12	111158	111169	
PHG9_Scaffold_37	41	p2	(CT)6	12	111842	111853	
PHG9_Scaffold_37	51	p2	(TA)6	12	153586	153597	
PHG9_Scaffold_37	52	p2	(TC)7	14	158427	158440	potentially variable
PHG9_Scaffold_37	54	p2	(TA)7	14	161627	161640	potentially variable
PHG9_Scaffold_37	55	p3	(GCT)5	15	166539	166553	potentially variable
PHG9_Scaffold_37	57	p3	(TTC)5	15	169066	169080	potentially variable
PHG9_Scaffold_37	61	p3	(ATA)5	15	180851	180865	potentially variable
PHG9_Scaffold_37	67	p2	(GT)6	12	201758	201769	
PHG9_Scaffold_37	73	p2	(AG)6	12	216115	216126	
PHG9_Scaffold_37	74	p3	(TTA)5	15	218679	218693	potentially variable
PHG9_Scaffold_37	75	p2	(TA)7	14	225685	225698	potentially variable

PHG9_Scaffold_37	80	p2	(TA)7	14	238050	238063	potentially variable
PHG9_Scaffold_37	86	p2	(TA)8	16	247931	247946	potentially variable
PHG9_Scaffold_37	88	p3	(CAT)5	15	261889	261903	potentially variable
PHG9_Scaffold_37	90	p3	(TTC)5	15	264685	264699	potentially variable
PHG9_Scaffold_37	94	p2	(TA)6	12	293750	293761	
PHG9_Scaffold_37	96	p2	(TA)9	18	297564	297581	potentially variable
PHG9_Scaffold_37	101	p2	(AC)7	14	322426	322439	potentially variable
PHG9_Scaffold_38	23	p2	(AT)9	18	83422	83439	potentially variable
PHG9_Scaffold_38	31	p3	(TTA)5	15	97897	97911	potentially variable
PHG9_Scaffold_38	34	p2	(GT)7	14	117919	117932	potentially variable
PHG9_Scaffold_39	5	p3	(CAT)5	15	71518	71532	potentially variable
PHG9_Scaffold_39	8	p2	(TA)6	12	82937	82948	
PHG9_Scaffold_40	14	p2	(TA)7	14	51475	51488	potentially variable
PHG9_Scaffold_40	16	p2	(TA)7	14	54966	54979	potentially variable
PHG9_Scaffold_40	20	p3	(CCA)6	18	66797	66814	potentially variable
PHG9_Scaffold_40	21	p2	(TA)8	16	68995	69010	potentially variable

PHG9_Scaffold_40	23	p3	(TCT)5	15	70689	70703	potentially variable
PHG9_Scaffold_40	27	p2	(AT)6	12	78128	78139	
PHG9_Scaffold_40	36	p3	(CCG)5	15	99490	99504	potentially variable
PHG9_Scaffold_40	41	p3	(TTC)5	15	109153	109167	potentially variable
PHG9_Scaffold_40	42	p2	(TA)6	12	109693	109704	
PHG9_Scaffold_41	4	p3	(AAT)6	18	10540	10557	potentially variable
PHG9_Scaffold_41	11	p3	(GTT)5	15	31079	31093	potentially variable
PHG9_Scaffold_41	12	p2	(AT)6	12	33373	33384	
PHG9_Scaffold_41	13	p3	(GAT)5	15	34584	34598	potentially variable
PHG9_Scaffold_41	15	p3	(TTA)5	15	39027	39041	potentially variable
PHG9_Scaffold_41	20	p2	(AG)9	18	50320	50337	potentially variable
PHG9_Scaffold_41	21	p2	(TA)7	14	50947	50960	potentially variable
PHG9_Scaffold_41	22	p2	(TA)8	16	51947	51962	potentially variable
PHG9_Scaffold_41	26	p2	(AT)6	12	77329	77340	
PHG9_Scaffold_41	46	p2	(TA)6	12	155766	155777	
PHG9_Scaffold_41	48	p2	(AG)6	12	173159	173170	
PHG9_Scaffold_41	53	p2	(TA)6	12	176672	176683	
PHG9_Scaffold_41	55	p3	(CCT)5	15	195498	195512	potentially variable

PHG9_Scaffold_42	4	p2	(CA)6	12	8995	9006	
PHG9_Scaffold_42	5	p3	(TGT)5	15	15910	15924	potentially variable
PHG9_Scaffold_42	8	p2	(TA)6	12	23730	23741	
PHG9_Scaffold_42	12	p3	(AAC)5	15	31260	31274	potentially variable
PHG9_Scaffold_42	15	p3	(GTA)5	15	39670	39684	potentially variable
PHG9_Scaffold_43	2	p2	(TG)6	12	8162	8173	
PHG9_Scaffold_43	7	p2	(AG)6	12	27749	27760	
PHG9_Scaffold_43	9	p2	(TA)7	14	28936	28949	potentially variable
PHG9_Scaffold_43	12	p3	(ATT)5	15	63100	63114	potentially variable
PHG9_Scaffold_43	18	p3	(TAC)5	15	72540	72554	potentially variable
PHG9_Scaffold_43	33	p2	(AT)9	18	110977	110994	potentially variable
PHG9_Scaffold_43	34	p2	(AT)7	14	113107	113120	potentially variable
PHG9_Scaffold_43	36	p3	(GTG)5	15	116998	117012	potentially variable
PHG9_Scaffold_43	37	p3	(ACA)5	15	118425	118439	potentially variable
PHG9_Scaffold_43	39	p2	(AT)8	16	130798	130813	potentially variable
PHG9_Scaffold_45	7	p2	(CT)6	12	9134	9145	
PHG9_Scaffold_45	8	p2	(TC)6	12	11041	11052	
PHG9_Scaffold_45	9	p2	(GT)6	12	11634	11645	

PHG9_Scaffold_46	3	p2	(AT)8	16	2423	2438	potentially variable
PHG9_Scaffold_46	5	p2	(AT)7	14	11961	11974	potentially variable
PHG9_Scaffold_46	15	p2	(TA)7	14	31843	31856	potentially variable
PHG9_Scaffold_46	33	p2	(AT)6	12	66610	66621	
PHG9_Scaffold_47	15	p3	(TTC)5	15	34041	34055	potentially variable
PHG9_Scaffold_47	20	p3	(ATA)5	15	40514	40528	potentially variable
PHG9_Scaffold_47	29	p2	(AT)6	12	58950	58961	
PHG9_Scaffold_47	30	p2	(TA)6	12	62658	62669	
PHG9_Scaffold_47	39	p2	(GT)8	16	96906	96921	potentially variable
PHG9_Scaffold_47	41	p6	(CAAAAC)5	30	107827	107856	Hypervariable
PHG9_Scaffold_47	48	p2	(TA)6	12	126150	126161	
PHG9_Scaffold_47	52	p2	(TA)7	14	135587	135600	potentially variable
PHG9_Scaffold_47	54	p3	(TCA)5	15	145471	145485	potentially variable
PHG9_Scaffold_47	59	p2	(AG)6	12	155224	155235	
PHG9_Scaffold_47	61	p2	(TC)6	12	160637	160648	
PHG9_Scaffold_47	69	p2	(GT)8	16	177851	177866	potentially variable
PHG9_Scaffold_47	76	p2	(TC)6	12	205375	205386	
PHG9_Scaffold_48	14	p3	(TAA)5	15	48994	49008	potentially variable
PHG9_Scaffold_48	16	p3	(TAA)5	15	53323	53337	potentially

							variable
PHG9_Scaffold_48	25	p2	(AT)6	12	84102	84113	
PHG9_Scaffold_48	28	p3	(TTA)5	15	87757	87771	potentially variable
PHG9_Scaffold_48	39	p3	(AAT)5	15	126273	126287	potentially variable
PHG9_Scaffold_48	42	p2	(AT)7	14	130047	130060	potentially variable
PHG9_Scaffold_48	43	p2	(TA)8	16	131150	131165	potentially variable
PHG9_Scaffold_48	44	p2	(AT)6	12	132688	132699	
PHG9_Scaffold_48	45	p3	(AAT)5	15	134791	134805	potentially variable
PHG9_Scaffold_48	52	p2	(TC)6	12	146062	146073	
PHG9_Scaffold_48	53	p3	(TAT)6	18	148237	148254	potentially variable
PHG9_Scaffold_48	56	p3	(ATT)5	15	154665	154679	potentially variable
PHG9_Scaffold_48	57	p2	(GA)6	12	157230	157241	
PHG9_Scaffold_48	58	p2	(TA)6	12	158399	158410	
PHG9_Scaffold_48	76	p2	(AT)7	14	193851	193864	potentially variable
PHG9_Scaffold_48	81	p3	(TAT)5	15	213725	213739	potentially variable
PHG9_Scaffold_48	84	p2	(TA)9	18	229936	229953	potentially variable
PHG9_Scaffold_48	86	p2	(AT)7	14	230648	230661	potentially variable
PHG9_Scaffold_48	87	p2	(AT)6	12	239834	239845	

PHG9_Scaffold_48	90	p2	(AT)7	14	244347	244360	potentially variable
PHG9_Scaffold_48	92	p2	(TA)7	14	249527	249540	potentially variable
PHG9_Scaffold_48	101	p2	(TA)6	12	268271	268282	
PHG9_Scaffold_48	103	p3	(GAA)6	18	272495	272512	potentially variable
PHG9_Scaffold_49	1	p2	(TA)7	14	21376	21389	potentially variable
PHG9_Scaffold_49	4	p2	(TA)9	18	25368	25385	potentially variable
PHG9_Scaffold_49	7	p2	(TA)6	12	44865	44876	
PHG9_Scaffold_49	14	p2	(AT)6	12	54425	54436	
PHG9_Scaffold_49	18	p3	(CGG)5	15	61895	61909	potentially variable
PHG9_Scaffold_50	5	p2	(TA)7	14	24707	24720	potentially variable
PHG9_Scaffold_50	7	p2	(TA)7	14	29585	29598	potentially variable
PHG9_Scaffold_50	11	p2	(TA)7	14	36160	36173	potentially variable
PHG9_Scaffold_50	23	p2	(TA)6	12	81208	81219	
PHG9_Scaffold_50	25	p2	(TA)6	12	87318	87329	
PHG9_Scaffold_50	40	p2	(TA)8	16	140160	140175	potentially variable
PHG9_Scaffold_50	48	p3	(GCT)5	15	176779	176793	potentially variable
PHG9_Scaffold_50	54	p2	(GA)9	18	223370	223387	potentially variable
PHG9_Scaffold_50	56	p3	(ATC)5	15	230595	230609	potentially

							variable
PHG9_Scaffold_50	58	p2	(CA)9	18	234545	234562	potentially variable
PHG9_Scaffold_50	60	p2	(TA)8	16	235636	235651	potentially variable
PHG9_Scaffold_50	61	p2	(TA)7	14	235854	235867	potentially variable
PHG9_Scaffold_50	62	p2	(CT)7	14	236037	236050	potentially variable
PHG9_Scaffold_50	63	p2	(AT)6	12	239040	239051	
PHG9_Scaffold_50	66	p2	(TA)9	18	250135	250152	potentially variable
PHG9_Scaffold_50	67	p2	(GT)7	14	255228	255241	potentially variable
PHG9_Scaffold_51	1	p2	(AG)6	12	7353	7364	
PHG9_Scaffold_51	2	p2	(AT)8	16	8945	8960	potentially variable
PHG9_Scaffold_51	3	p2	(TA)8	16	14722	14737	potentially variable
PHG9_Scaffold_51	7	p2	(TA)6	12	19463	19474	
PHG9_Scaffold_51	8	p3	(GAT)5	15	20597	20611	potentially variable
PHG9_Scaffold_51	14	p2	(TA)6	12	34064	34075	
PHG9_Scaffold_51	26	p2	(TA)8	16	50246	50261	potentially variable
PHG9_Scaffold_51	35	p2	(TC)6	12	72585	72596	
PHG9_Scaffold_51	36	p2	(CT)6	12	80876	80887	
PHG9_Scaffold_51	37	p3	(GAA)5	15	81373	81387	potentially variable

PHG9_Scaffold_51	40	p3	(TAA)5	15	84279	84293	potentially variable
PHG9_Scaffold_51	47	p2	(TA)7	14	100816	100829	potentially variable
PHG9_Scaffold_51	49	p2	(TG)6	12	106824	106835	
PHG9_Scaffold_51	56	p3	(ATA)5	15	137478	137492	potentially variable
PHG9_Scaffold_51	60	p2	(AT)8	16	146363	146378	potentially variable
PHG9_Scaffold_51	64	p2	(AT)7	14	164964	164977	potentially variable
PHG9_Scaffold_51	77	p3	(AGA)5	15	205200	205214	potentially variable
PHG9_Scaffold_51	79	p3	(GCA)5	15	206851	206865	potentially variable
PHG9_Scaffold_52	12	p2	(GA)6	12	22809	22820	
PHG9_Scaffold_52	22	p3	(ATA)6	18	52014	52031	potentially variable
PHG9_Scaffold_52	30	p3	(TAA)6	18	71134	71151	potentially variable
PHG9_Scaffold_52	31	p2	(TC)9	18	71448	71465	potentially variable
PHG9_Scaffold_52	37	p2	(AT)6	12	87455	87466	
PHG9_Scaffold_52	44	p3	(TTC)6	18	99135	99152	potentially variable
PHG9_Scaffold_52	46	p2	(AT)6	12	113059	113070	
PHG9_Scaffold_52	50	p2	(AG)6	12	150272	150283	
PHG9_Scaffold_52	57	p2	(AT)6	12	200130	200141	
PHG9_Scaffold_53	2	p2	(AC)6	12	10758	10769	

PHG9_Scaffold_53	32	p2	(AT)7	14	100542	100555	potentially variable
PHG9_Scaffold_53	36	p2	(AT)9	18	109821	109838	potentially variable
PHG9_Scaffold_53	41	p3	(AGA)5	15	115643	115657	potentially variable
PHG9_Scaffold_53	44	p3	(AGA)5	15	120977	120991	potentially variable
PHG9_Scaffold_53	56	p2	(AT)6	12	156650	156661	
PHG9_Scaffold_53	62	p3	(CAC)5	15	185583	185597	potentially variable
PHG9_Scaffold_53	63	p2	(TA)6	12	186483	186494	
PHG9_Scaffold_53	65	p3	(TAT)6	18	187859	187876	potentially variable
PHG9_Scaffold_54	10	p2	(AT)6	12	29126	29137	
PHG9_Scaffold_54	11	p2	(TA)8	16	30571	30586	potentially variable
PHG9_Scaffold_54	15	p3	(CCA)5	15	49356	49370	potentially variable
PHG9_Scaffold_54	17	p2	(TG)6	12	58964	58975	
PHG9_Scaffold_54	23	p3	(ACA)5	15	67721	67735	potentially variable
PHG9_Scaffold_54	34	p2	(TA)6	12	94646	94657	
PHG9_Scaffold_54	40	p2	(TC)7	14	125358	125371	potentially variable
PHG9_Scaffold_55	6	p6	(TGGGAA)6	36	12662	12697	Hypervariable
PHG9_Scaffold_55	8	p2	(AT)6	12	26008	26019	
PHG9_Scaffold_56	2	p3	(ATG)5	15	977	991	potentially variable

PHG9_Scaffold_56	7	p3	(ATT)5	15	16986	17000	potentially variable
PHG9_Scaffold_56	8	p2	(TC)6	12	18443	18454	
PHG9_Scaffold_56	16	p2	(TA)8	16	36251	36266	potentially variable
PHG9_Scaffold_56	20	p2	(AT)8	16	52329	52344	potentially variable
PHG9_Scaffold_56	24	p6	(ATCTTC)5	30	54886	54915	Hypervariable
PHG9_Scaffold_56	32	p2	(TC)6	12	68415	68426	
PHG9_Scaffold_56	43	p2	(AT)6	12	117066	117077	
PHG9_Scaffold_56	44	p2	(TA)6	12	119673	119684	
PHG9_Scaffold_56	46	p2	(AG)6	12	123788	123799	
PHG9_Scaffold_56	47	p2	(AG)7	14	128266	128279	potentially variable
PHG9_Scaffold_57	9	p2	(TG)6	12	91194	91205	
PHG9_Scaffold_57	11	p2	(CA)6	12	96770	96781	
PHG9_Scaffold_58	3	p3	(AAT)5	15	8892	8906	potentially variable
PHG9_Scaffold_58	7	p3	(AAG)5	15	23565	23579	potentially variable
PHG9_Scaffold_58	26	p2	(AG)8	16	88268	88283	potentially variable
PHG9_Scaffold_58	27	p3	(CGG)5	15	92117	92131	potentially variable
PHG9_Scaffold_58	35	p2	(TA)8	16	121745	121760	potentially variable
PHG9_Scaffold_58	44	p3	(TTA)5	15	149665	149679	potentially variable
PHG9_Scaffold_58	48	p2	(TA)6	12	156946	156957	

PHG9_Scaffold_58	57	p2	(TA)7	14	186847	186860	potentially variable
PHG9_Scaffold_59	9	p2	(TA)8	16	24737	24752	potentially variable
PHG9_Scaffold_59	16	p2	(AT)6	12	50723	50734	
PHG9_Scaffold_59	18	p2	(CA)6	12	52083	52094	
PHG9_Scaffold_59	26	p2	(AT)6	12	91496	91507	
PHG9_Scaffold_59	28	p2	(TA)6	12	98898	98909	
PHG9_Scaffold_59	36	p2	(AT)6	12	111106	111117	
PHG9_Scaffold_59	45	p2	(AT)9	18	133673	133690	potentially variable
PHG9_Scaffold_59	48	p2	(AT)6	12	146478	146489	
PHG9_Scaffold_59	49	p2	(TA)8	16	154699	154714	potentially variable
PHG9_Scaffold_59	55	p2	(AT)7	14	180978	180991	potentially variable
PHG9_Scaffold_59	59	p2	(TA)6	12	189291	189302	
PHG9_Scaffold_59	60	p2	(AT)8	16	189926	189941	potentially variable
PHG9_Scaffold_60	21	p3	(GAA)5	15	61832	61846	potentially variable
PHG9_Scaffold_60	29	p2	(TG)6	12	76081	76092	
PHG9_Scaffold_60	30	p3	(TTC)5	15	80111	80125	potentially variable
PHG9_Scaffold_60	39	p2	(AG)7	14	110394	110407	potentially variable
PHG9_Scaffold_60	51	p2	(AT)6	12	139149	139160	
PHG9_Scaffold_60	52	p2	(AT)6	12	144565	144576	
PHG9_Scaffold_60	60	p2	(CT)6	12	177031	177042	

PHG9_Scaffold_60	70	p3	(ATG)5	15	237141	237155	potentially variable
PHG9_Scaffold_60	76	p2	(AG)6	12	260093	260104	
PHG9_Scaffold_60	77	p2	(TG)6	12	261809	261820	
PHG9_Scaffold_60	80	p2	(GA)6	12	266697	266708	
PHG9_Scaffold_60	81	p2	(AC)6	12	267070	267081	
PHG9_Scaffold_60	86	p3	(GAA)6	18	295638	295655	potentially variable
PHG9_Scaffold_60	88	p2	(TC)8	16	300054	300069	potentially variable
PHG9_Scaffold_60	89	p2	(AT)8	16	305033	305048	potentially variable
PHG9_Scaffold_60	100	p3	(GAT)5	15	343943	343957	potentially variable
PHG9_Scaffold_60	107	p2	(TA)6	12	355201	355212	
PHG9_Scaffold_60	111	p2	(CT)8	16	360015	360030	potentially variable
PHG9_Scaffold_60	139	p2	(TA)6	12	452485	452496	
PHG9_Scaffold_61	1	p2	(AT)8	16	11510	11525	potentially variable
PHG9_Scaffold_61	16	p3	(AAT)5	15	91645	91659	potentially variable
PHG9_Scaffold_62	11	p2	(CT)6	12	42887	42898	
PHG9_Scaffold_62	12	p2	(AC)6	12	47597	47608	
PHG9_Scaffold_62	17	p3	(TTA)5	15	65466	65480	potentially variable
PHG9_Scaffold_62	31	p2	(TA)6	12	116480	116491	
PHG9_Scaffold_62	35	p2	(CT)6	12	132291	132302	
PHG9_Scaffold_62	41	p2	(GA)6	12	154154	154165	

PHG9_Scaffold_62	51	p2	(TA)7	14	184039	184052	potentially variable
PHG9_Scaffold_62	52	p2	(TC)6	12	185329	185340	
PHG9_Scaffold_62	60	p2	(TA)6	12	206237	206248	
PHG9_Scaffold_62	61	p2	(TA)8	16	206873	206888	potentially variable
PHG9_Scaffold_62	62	p2	(AT)6	12	210081	210092	
PHG9_Scaffold_62	71	p3	(ACC)5	15	230534	230548	potentially variable
PHG9_Scaffold_62	75	p2	(TA)7	14	238386	238399	potentially variable
PHG9_Scaffold_62	83	p3	(TAA)5	15	268924	268938	potentially variable
PHG9_Scaffold_62	87	p2	(AT)6	12	274502	274513	
PHG9_Scaffold_62	89	p2	(CT)6	12	296410	296421	
PHG9_Scaffold_62	123	p2	(TC)6	12	422322	422333	
PHG9_Scaffold_62	125	p2	(AT)6	12	427764	427775	
PHG9_Scaffold_62	126	p3	(TTA)5	15	429021	429035	potentially variable
PHG9_Scaffold_62	128	p2	(TC)9	18	434265	434282	potentially variable
PHG9_Scaffold_62	131	p2	(TA)6	12	446781	446792	
PHG9_Scaffold_63	2	p3	(AAG)6	18	310	327	potentially variable
PHG9_Scaffold_63	16	p2	(TA)9	18	40126	40143	potentially variable
PHG9_Scaffold_63	19	p2	(TA)6	12	41525	41536	
PHG9_Scaffold_63	20	p2	(TA)6	12	41843	41854	
PHG9_Scaffold_63	35	p2	(TC)6	12	70998	71009	

PHG9_Scaffold_63	40	p2	(TA)8	16	78354	78369	potentially variable
PHG9_Scaffold_63	41	p2	(TA)7	14	82074	82087	potentially variable
PHG9_Scaffold_64	3	p3	(TAA)5	15	2214	2228	potentially variable
PHG9_Scaffold_64	11	p2	(TA)8	16	32225	32240	potentially variable
PHG9_Scaffold_64	14	p2	(TC)7	14	47864	47877	potentially variable
PHG9_Scaffold_64	20	p2	(AC)8	16	62053	62068	potentially variable
PHG9_Scaffold_64	21	p2	(TC)6	12	63722	63733	
PHG9_Scaffold_64	34	p3	(ATT)6	18	89483	89500	potentially variable
PHG9_Scaffold_64	39	p2	(TA)7	14	121713	121726	potentially variable
PHG9_Scaffold_64	44	p2	(AT)6	12	136211	136222	
PHG9_Scaffold_64	49	p2	(AT)6	12	142239	142250	
PHG9_Scaffold_64	51	p3	(ATC)6	18	149942	149959	potentially variable
PHG9_Scaffold_64	56	p2	(AT)9	18	162784	162801	potentially variable
PHG9_Scaffold_64	57	p3	(AAG)5	15	167355	167369	potentially variable
PHG9_Scaffold_64	58	p3	(AGA)5	15	169444	169458	potentially variable
PHG9_Scaffold_64	59	p2	(TA)6	12	169771	169782	
PHG9_Scaffold_65	8	p2	(TC)6	12	20721	20732	
PHG9_Scaffold_65	9	p3	(ATT)6	18	33649	33666	potentially

							variable
PHG9_Scaffold_65	16	p2	(AG)6	12	56775	56786	
PHG9_Scaffold_65	17	p2	(AT)8	16	57150	57165	potentially variable
PHG9_Scaffold_65	24	p2	(CT)7	14	64234	64247	potentially variable
PHG9_Scaffold_65	32	p2	(AT)7	14	81146	81159	potentially variable
PHG9_Scaffold_65	56	p2	(AT)7	14	164915	164928	potentially variable
PHG9_Scaffold_65	60	p3	(TTC)5	15	167841	167855	potentially variable
PHG9_Scaffold_65	63	p2	(AC)8	16	175612	175627	potentially variable
PHG9_Scaffold_65	66	p2	(TA)8	16	178241	178256	potentially variable
PHG9_Scaffold_65	67	p2	(TA)8	16	181130	181145	potentially variable
PHG9_Scaffold_65	69	p3	(ATT)5	15	187193	187207	potentially variable
PHG9_Scaffold_65	75	p2	(AT)9	18	194160	194177	potentially variable
PHG9_Scaffold_65	78	p2	(AT)6	12	201733	201744	
PHG9_Scaffold_66	2	p2	(AT)9	18	7362	7379	potentially variable
PHG9_Scaffold_66	4	p2	(TA)6	12	7788	7799	
PHG9_Scaffold_66	13	p3	(TTC)5	15	50773	50787	potentially variable
PHG9_Scaffold_66	15	p2	(AT)6	12	60603	60614	
PHG9_Scaffold_66	19	p2	(AT)7	14	84779	84792	potentially

							variable
PHG9_Scaffold_66	23	p3	(CTG)6	18	96738	96755	potentially variable
PHG9_Scaffold_66	28	p3	(TTC)6	18	109067	109084	potentially variable
PHG9_Scaffold_67	5	p2	(AT)6	12	32241	32252	
PHG9_Scaffold_67	8	p3	(TAA)5	15	46090	46104	potentially variable
PHG9_Scaffold_67	10	p2	(AT)6	12	52041	52052	
PHG9_Scaffold_67	14	p2	(AG)9	18	62902	62919	potentially variable
PHG9_Scaffold_67	21	p2	(TA)6	12	79056	79067	
PHG9_Scaffold_67	24	p2	(TA)6	12	81733	81744	
PHG9_Scaffold_68	4	p2	(AT)8	16	20394	20409	potentially variable
PHG9_Scaffold_68	5	p2	(AT)6	12	22704	22715	
PHG9_Scaffold_68	6	p2	(AG)7	14	23466	23479	potentially variable
PHG9_Scaffold_68	10	p2	(TA)7	14	48861	48874	potentially variable
PHG9_Scaffold_69	8	p3	(ACA)6	18	21091	21108	potentially variable
PHG9_Scaffold_69	9	p3	(AAT)6	18	22372	22389	potentially variable
PHG9_Scaffold_69	12	p3	(TAA)5	15	26235	26249	potentially variable
PHG9_Scaffold_69	13	p2	(AC)6	12	31919	31930	
PHG9_Scaffold_69	22	p2	(CA)9	18	59005	59022	potentially variable

PHG9_Scaffold_69	28	p2	(TA)8	16	70206	70221	potentially variable
PHG9_Scaffold_69	31	p2	(TA)6	12	73574	73585	
PHG9_Scaffold_69	33	p3	(TGT)5	15	78139	78153	potentially variable
PHG9_Scaffold_69	36	p2	(TA)8	16	87450	87465	potentially variable
PHG9_Scaffold_69	37	p2	(TA)7	14	91141	91154	potentially variable
PHG9_Scaffold_69	40	p2	(AG)6	12	94508	94519	
PHG9_Scaffold_69	42	p2	(AT)8	16	100286	100301	potentially variable
PHG9_Scaffold_69	47	p3	(TTA)5	15	108166	108180	potentially variable
PHG9_Scaffold_69	56	p2	(TA)7	14	131171	131184	potentially variable
PHG9_Scaffold_69	57	p2	(TA)6	12	136795	136806	
PHG9_Scaffold_69	59	p2	(CT)6	12	141025	141036	
PHG9_Scaffold_70	3	p2	(TG)6	12	9320	9331	
PHG9_Scaffold_70	11	p2	(TA)7	14	46557	46570	potentially variable
PHG9_Scaffold_70	13	p3	(TTG)5	15	57019	57033	potentially variable
PHG9_Scaffold_70	16	p2	(TA)7	14	66111	66124	potentially variable
PHG9_Scaffold_70	17	p2	(AT)6	12	66661	66672	
PHG9_Scaffold_70	19	p2	(TA)6	12	71060	71071	
PHG9_Scaffold_70	20	p3	(ATC)6	18	71866	71883	potentially variable

PHG9_Scaffold_70	22	p2	(AT)7	14	77581	77594	potentially variable
PHG9_Scaffold_70	25	p3	(CAT)5	15	82306	82320	potentially variable
PHG9_Scaffold_70	34	p2	(TA)6	12	92698	92709	
PHG9_Scaffold_70	39	p3	(TTA)5	15	117716	117730	potentially variable
PHG9_Scaffold_70	44	p3	(ATT)6	18	136167	136184	potentially variable
PHG9_Scaffold_71	7	p3	(AAT)5	15	22619	22633	potentially variable
PHG9_Scaffold_71	12	p3	(CGT)5	15	39022	39036	potentially variable
PHG9_Scaffold_71	24	p2	(TA)6	12	86545	86556	
PHG9_Scaffold_72	2	p2	(TA)8	16	7835	7850	potentially variable
PHG9_Scaffold_72	21	p2	(TA)6	12	45542	45553	
PHG9_Scaffold_72	33	p3	(ATT)5	15	72533	72547	potentially variable
PHG9_Scaffold_72	35	p3	(TAT)5	15	77180	77194	potentially variable
PHG9_Scaffold_72	40	p3	(TAT)5	15	85358	85372	potentially variable
PHG9_Scaffold_72	41	p2	(TA)9	18	85690	85707	potentially variable
PHG9_Scaffold_72	43	p2	(TA)8	16	87797	87812	potentially variable
PHG9_Scaffold_72	48	p2	(TA)6	12	109004	109015	
PHG9_Scaffold_72	51	p3	(CTT)5	15	110155	110169	potentially variable

PHG9_Scaffold_72	62	p3	(TCA)5	15	133285	133299	potentially variable
PHG9_Scaffold_73	20	p2	(TA)7	14	42053	42066	potentially variable
PHG9_Scaffold_73	22	p2	(AT)6	12	57179	57190	
PHG9_Scaffold_73	23	p3	(GAA)5	15	57551	57565	potentially variable
PHG9_Scaffold_73	24	p2	(TG)7	14	59907	59920	potentially variable
PHG9_Scaffold_73	26	p2	(CT)7	14	69480	69493	potentially variable
PHG9_Scaffold_73	30	p2	(TG)6	12	80547	80558	
PHG9_Scaffold_73	33	p2	(GT)8	16	88573	88588	potentially variable
PHG9_Scaffold_73	38	p2	(TA)8	16	105488	105503	potentially variable
PHG9_Scaffold_73	39	p2	(CA)6	12	109550	109561	
PHG9_Scaffold_73	40	p2	(AT)6	12	111480	111491	
PHG9_Scaffold_73	44	p2	(GA)6	12	122519	122530	
PHG9_Scaffold_73	48	p2	(AT)6	12	147698	147709	
PHG9_Scaffold_73	52	p2	(TA)6	12	156173	156184	
PHG9_Scaffold_73	56	p2	(AT)8	16	161822	161837	potentially variable
PHG9_Scaffold_73	58	p2	(TA)6	12	162459	162470	
PHG9_Scaffold_73	60	p3	(TCA)6	18	173578	173595	potentially variable
PHG9_Scaffold_73	77	p2	(AG)9	18	204655	204672	potentially variable
PHG9_Scaffold_73	81	p2	(TA)6	12	217720	217731	

PHG9_Scaffold_73	88	p2	(TA)7	14	250182	250195	potentially variable
PHG9_Scaffold_73	89	p2	(AG)9	18	253106	253123	potentially variable
PHG9_Scaffold_73	94	p2	(AT)6	12	275675	275686	
PHG9_Scaffold_73	95	p2	(TA)7	14	292465	292478	potentially variable
PHG9_Scaffold_73	99	p2	(TA)6	12	304608	304619	
PHG9_Scaffold_73	100	p2	(AT)7	14	305457	305470	potentially variable
PHG9_Scaffold_73	103	p2	(TA)6	12	314062	314073	
PHG9_Scaffold_73	110	p3	(ATT)5	15	329913	329927	potentially variable
PHG9_Scaffold_73	120	p2	(AT)8	16	369733	369748	potentially variable
PHG9_Scaffold_73	124	p2	(AT)9	18	387123	387140	potentially variable
PHG9_Scaffold_73	125	p2	(GA)6	12	387837	387848	
PHG9_Scaffold_73	128	p2	(TA)9	18	389302	389319	potentially variable
PHG9_Scaffold_73	132	p2	(AT)8	16	397433	397448	potentially variable
PHG9_Scaffold_73	134	p3	(ATT)6	18	408979	408996	potentially variable
PHG9_Scaffold_73	142	p2	(GA)7	14	432907	432920	potentially variable
PHG9_Scaffold_74	3	p2	(CA)9	18	7733	7750	potentially variable
PHG9_Scaffold_74	4	p2	(TA)6	12	9708	9719	
PHG9_Scaffold_74	10	p3	(ATT)5	15	27876	27890	potentially

							variable
PHG9_Scaffold_74	11	p2	(AT)6	12	32811	32822	
PHG9_Scaffold_74	14	p2	(GA)6	12	35844	35855	
PHG9_Scaffold_74	29	p3	(GAA)5	15	71873	71887	potentially variable
PHG9_Scaffold_74	31	p2	(AT)7	14	74302	74315	potentially variable
PHG9_Scaffold_74	33	p2	(TC)6	12	78611	78622	
PHG9_Scaffold_74	34	p3	(TTC)6	18	80588	80605	potentially variable
PHG9_Scaffold_74	35	p2	(AG)6	12	84006	84017	
PHG9_Scaffold_74	36	p2	(AG)6	12	85823	85834	
PHG9_Scaffold_74	37	p2	(TA)6	12	87709	87720	
PHG9_Scaffold_74	39	p2	(AT)6	12	98611	98622	
PHG9_Scaffold_74	50	p2	(TA)7	14	131472	131485	potentially variable
PHG9_Scaffold_74	53	p2	(AT)6	12	137731	137742	
PHG9_Scaffold_74	64	p2	(TA)6	12	166707	166718	
PHG9_Scaffold_74	68	p2	(GT)7	14	175946	175959	potentially variable
PHG9_Scaffold_74	70	p2	(AT)9	18	188926	188943	potentially variable
PHG9_Scaffold_74	73	p3	(CAT)5	15	194532	194546	potentially variable
PHG9_Scaffold_75	2	p2	(TA)7	14	2482	2495	potentially variable
PHG9_Scaffold_75	6	p2	(TA)7	14	11683	11696	potentially variable
PHG9_Scaffold_75	14	p2	(AT)8	16	30130	30145	potentially

							variable
PHG9_Scaffold_75	23	p3	(TTC)6	18	47898	47915	potentially variable
PHG9_Scaffold_75	25	p3	(ATA)5	15	52858	52872	potentially variable
PHG9_Scaffold_75	34	p2	(AT)6	12	90498	90509	
PHG9_Scaffold_75	38	p2	(AT)6	12	97825	97836	
PHG9_Scaffold_75	39	p2	(AT)7	14	97981	97994	potentially variable
PHG9_Scaffold_75	50	p2	(AT)9	18	118974	118991	potentially variable
PHG9_Scaffold_75	55	p2	(TA)6	12	135973	135984	
PHG9_Scaffold_75	61	p3	(ATT)6	18	150572	150589	potentially variable
PHG9_Scaffold_75	65	p2	(AG)7	14	161340	161353	potentially variable
PHG9_Scaffold_75	70	p2	(AT)6	12	174513	174524	
PHG9_Scaffold_75	76	p2	(TA)7	14	200424	200437	potentially variable
PHG9_Scaffold_75	80	p3	(TTC)6	18	202520	202537	potentially variable
PHG9_Scaffold_75	84	p2	(TA)7	14	221477	221490	potentially variable
PHG9_Scaffold_75	88	p3	(TTA)5	15	227583	227597	potentially variable
PHG9_Scaffold_75	90	p3	(AAT)6	18	239563	239580	potentially variable
PHG9_Scaffold_75	102	p2	(TA)7	14	290038	290051	potentially variable
PHG9_Scaffold_75	103	p3	(AAT)5	15	291404	291418	potentially

							variable
PHG9_Scaffold_75	105	p3	(AGA)6	18	298496	298513	potentially variable
PHG9_Scaffold_75	119	p2	(AG)6	12	340914	340925	
PHG9_Scaffold_75	131	p2	(TA)8	16	365998	366013	potentially variable
PHG9_Scaffold_76	1	p2	(TA)7	14	11935	11948	potentially variable
PHG9_Scaffold_76	12	p2	(TA)6	12	61396	61407	
PHG9_Scaffold_76	14	p2	(AT)7	14	63049	63062	potentially variable
PHG9_Scaffold_76	16	p3	(TTA)5	15	63706	63720	potentially variable
PHG9_Scaffold_76	19	p2	(TC)6	12	73233	73244	
PHG9_Scaffold_76	23	p2	(AT)6	12	76210	76221	
PHG9_Scaffold_76	25	p2	(AT)8	16	81679	81694	potentially variable
PHG9_Scaffold_76	34	p2	(TA)6	12	111145	111156	
PHG9_Scaffold_77	4	p3	(ATT)5	15	13852	13866	potentially variable
PHG9_Scaffold_77	6	p2	(TA)7	14	16975	16988	potentially variable
PHG9_Scaffold_77	8	p2	(TA)9	18	30303	30320	potentially variable
PHG9_Scaffold_77	16	p2	(TA)6	12	59044	59055	
PHG9_Scaffold_77	17	p2	(TA)6	12	62799	62810	
PHG9_Scaffold_77	20	p2	(AC)8	16	80474	80489	potentially variable
PHG9_Scaffold_77	21	p2	(AG)7	14	81853	81866	potentially

							variable
PHG9_Scaffold_78	5	p2	(AT)8	16	90683	90698	potentially variable
PHG9_Scaffold_78	14	p2	(AT)8	16	224123	224138	potentially variable
PHG9_Scaffold_79	1	p2	(TA)6	12	2704	2715	
PHG9_Scaffold_79	12	p2	(TA)6	12	18053	18064	
PHG9_Scaffold_79	16	p3	(TAA)6	18	26746	26763	potentially variable
PHG9_Scaffold_79	25	p3	(GCT)5	15	50232	50246	potentially variable
PHG9_Scaffold_79	33	p2	(AT)8	16	64264	64279	potentially variable
PHG9_Scaffold_79	46	p2	(AT)6	12	114389	114400	
PHG9_Scaffold_79	49	p2	(AT)9	18	117525	117542	potentially variable
PHG9_Scaffold_79	53	p2	(AT)6	12	149631	149642	
PHG9_Scaffold_79	55	p2	(TG)8	16	155054	155069	potentially variable
PHG9_Scaffold_79	56	p3	(TAA)5	15	155631	155645	potentially variable
PHG9_Scaffold_80	4	p2	(AT)6	12	9894	9905	
PHG9_Scaffold_81	2	p3	(ATT)5	15	4042	4056	potentially variable
PHG9_Scaffold_81	3	p2	(TA)8	16	19525	19540	potentially variable
PHG9_Scaffold_81	10	p3	(TGA)5	15	39541	39555	potentially variable
PHG9_Scaffold_81	33	p2	(TC)7	14	91588	91601	potentially variable

PHG9_Scaffold_81	36	p2	(AT)8	16	96124	96139	potentially variable
PHG9_Scaffold_81	47	p2	(AT)9	18	110841	110858	potentially variable
PHG9_Scaffold_81	54	p2	(GT)7	14	141969	141982	potentially variable
PHG9_Scaffold_81	58	p2	(CA)6	12	151994	152005	
PHG9_Scaffold_81	59	p2	(TG)8	16	152546	152561	potentially variable
PHG9_Scaffold_81	69	p2	(AT)7	14	186256	186269	potentially variable
PHG9_Scaffold_81	77	p3	(ATT)5	15	204618	204632	potentially variable
PHG9_Scaffold_81	78	p2	(CA)6	12	209771	209782	
PHG9_Scaffold_81	82	p2	(AG)6	12	220408	220419	
PHG9_Scaffold_81	93	p2	(TC)8	16	249344	249359	potentially variable
PHG9_Scaffold_81	94	p2	(AT)7	14	250826	250839	potentially variable
PHG9_Scaffold_81	107	p3	(AAT)5	15	280157	280171	potentially variable
PHG9_Scaffold_81	112	p2	(TA)6	12	288748	288759	
PHG9_Scaffold_82	6	p2	(TA)6	12	30424	30435	
PHG9_Scaffold_82	11	p3	(GCT)5	15	44241	44255	potentially variable
PHG9_Scaffold_82	20	p2	(AT)7	14	73954	73967	potentially variable
PHG9_Scaffold_82	24	p2	(AT)6	12	98127	98138	
PHG9_Scaffold_82	32	p2	(GT)7	14	109135	109148	potentially variable

PHG9_Scaffold_82	35	p2	(AT)9	18	114236	114253	potentially variable
PHG9_Scaffold_82	37	p3	(AGA)5	15	118251	118265	potentially variable
PHG9_Scaffold_83	3	p3	(CTT)5	15	65851	65865	potentially variable
PHG9_Scaffold_83	9	p2	(CT)6	12	105497	105508	
PHG9_Scaffold_83	13	p2	(AC)6	12	158377	158388	
PHG9_Scaffold_84	5	p3	(GGA)6	18	14983	15000	potentially variable
PHG9_Scaffold_84	8	p2	(TA)6	12	17376	17387	
PHG9_Scaffold_84	9	p2	(TA)6	12	28575	28586	
PHG9_Scaffold_84	16	p2	(AT)6	12	39152	39163	
PHG9_Scaffold_84	21	p2	(AT)9	18	68204	68221	potentially variable
PHG9_Scaffold_85	16	p2	(AT)9	18	64429	64446	potentially variable
PHG9_Scaffold_85	29	p2	(TA)6	12	98441	98452	
PHG9_Scaffold_86	1	p2	(AT)7	14	1	14	potentially variable
PHG9_Scaffold_87	3	p2	(TA)6	12	4924	4935	
PHG9_Scaffold_87	14	p2	(TA)8	16	30027	30042	potentially variable
PHG9_Scaffold_87	18	p2	(TA)8	16	53087	53102	potentially variable
PHG9_Scaffold_87	19	p2	(TA)8	16	55639	55654	potentially variable
PHG9_Scaffold_87	34	p2	(TC)6	12	108057	108068	
PHG9_Scaffold_87	40	p2	(TA)8	16	118687	118702	potentially

							variable
PHG9_Scaffold_87	42	p3	(ATA)5	15	127828	127842	potentially variable
PHG9_Scaffold_87	45	p2	(AT)6	12	134772	134783	
PHG9_Scaffold_88	1	p2	(AT)8	16	3191	3206	potentially variable
PHG9_Scaffold_88	6	p3	(TAA)5	15	39821	39835	potentially variable
PHG9_Scaffold_88	15	p3	(ATC)5	15	133402	133416	potentially variable
PHG9_Scaffold_88	16	p2	(AT)6	12	139439	139450	
PHG9_Scaffold_89	4	p3	(TAA)5	15	11690	11704	potentially variable
PHG9_Scaffold_89	10	p2	(AT)6	12	30391	30402	
PHG9_Scaffold_89	12	p2	(AT)6	12	34108	34119	
PHG9_Scaffold_89	13	p2	(AT)9	18	35136	35153	potentially variable
PHG9_Scaffold_89	20	p2	(AT)6	12	51766	51777	
PHG9_Scaffold_89	25	p2	(AC)6	12	67925	67936	
PHG9_Scaffold_89	27	p2	(AC)9	18	71909	71926	potentially variable
PHG9_Scaffold_89	50	p3	(GAT)5	15	146786	146800	potentially variable
PHG9_Scaffold_89	59	p2	(AT)7	14	159842	159855	potentially variable
PHG9_Scaffold_89	83	p3	(AAC)5	15	216553	216567	potentially variable
PHG9_Scaffold_89	87	p2	(TA)8	16	225267	225282	potentially variable

PHG9_Scaffold_89	89	p3	(ATC)5	15	230163	230177	potentially variable
PHG9_Scaffold_89	110	p3	(TTA)5	15	275788	275802	potentially variable
PHG9_Scaffold_89	119	p3	(CCA)5	15	294173	294187	potentially variable
PHG9_Scaffold_89	120	p3	(AAT)5	15	304488	304502	potentially variable
PHG9_Scaffold_89	127	p2	(TA)6	12	319900	319911	
PHG9_Scaffold_89	129	p2	(AC)6	12	324212	324223	
PHG9_Scaffold_90	2	p2	(TA)7	14	1361	1374	potentially variable
PHG9_Scaffold_90	10	p2	(AG)9	18	17206	17223	potentially variable
PHG9_Scaffold_90	13	p3	(TTG)6	18	22261	22278	potentially variable
PHG9_Scaffold_90	23	p3	(ACC)5	15	44045	44059	potentially variable
PHG9_Scaffold_90	30	p2	(AT)9	18	72270	72287	potentially variable
PHG9_Scaffold_90	45	p2	(CA)6	12	138604	138615	
PHG9_Scaffold_90	46	p2	(CT)7	14	140336	140349	potentially variable
PHG9_Scaffold_91	3	p2	(TA)6	12	11677	11688	
PHG9_Scaffold_91	17	p3	(AAG)6	18	40077	40094	potentially variable
PHG9_Scaffold_91	18	p2	(AT)6	12	43459	43470	
PHG9_Scaffold_91	23	p2	(CT)6	12	50323	50334	
PHG9_Scaffold_91	25	p2	(AG)9	18	53903	53920	potentially variable

PHG9_Scaffold_91	36	p3	(ATT)6	18	70902	70919	potentially variable
PHG9_Scaffold_92	3	p2	(TA)6	12	7481	7492	
PHG9_Scaffold_92	4	p2	(CT)6	12	8728	8739	
PHG9_Scaffold_92	7	p2	(AT)8	16	16278	16293	potentially variable
PHG9_Scaffold_92	18	p2	(AT)8	16	43055	43070	potentially variable
PHG9_Scaffold_92	19	p2	(AT)7	14	43387	43400	potentially variable
PHG9_Scaffold_92	23	p2	(TG)6	12	58277	58288	
PHG9_Scaffold_92	27	p2	(CT)7	14	69520	69533	potentially variable
PHG9_Scaffold_92	30	p2	(GA)9	18	88124	88141	potentially variable
PHG9_Scaffold_92	38	p3	(TTC)5	15	113920	113934	potentially variable
PHG9_Scaffold_92	44	p2	(CT)8	16	145117	145132	potentially variable
PHG9_Scaffold_92	45	p3	(ATG)5	15	147121	147135	potentially variable
PHG9_Scaffold_92	48	p3	(AAT)5	15	154176	154190	potentially variable
PHG9_Scaffold_93	1	p2	(TA)6	12	4477	4488	
PHG9_Scaffold_93	3	p2	(TA)9	18	5118	5135	potentially variable
PHG9_Scaffold_93	7	p3	(CAC)5	15	16430	16444	potentially variable
PHG9_Scaffold_93	8	p3	(TAT)5	15	19760	19774	potentially variable

PHG9_Scaffold_93	14	p2	(CT)9	18	47325	47342	potentially variable
PHG9_Scaffold_93	15	p3	(TTA)5	15	47955	47969	potentially variable
PHG9_Scaffold_93	18	p2	(AT)8	16	54360	54375	potentially variable
PHG9_Scaffold_93	27	p6	(GGCTCA)5	30	81098	81127	Hypervariable
PHG9_Scaffold_93	28	p3	(TAA)5	15	88582	88596	potentially variable
PHG9_Scaffold_93	33	p3	(CTT)5	15	101705	101719	potentially variable
PHG9_Scaffold_93	35	p3	(ATT)5	15	105668	105682	potentially variable
PHG9_Scaffold_93	38	p2	(TC)6	12	113787	113798	
PHG9_Scaffold_93	53	p2	(AG)8	16	162912	162927	potentially variable
PHG9_Scaffold_93	57	p2	(TA)8	16	175171	175186	potentially variable
PHG9_Scaffold_93	59	p2	(TG)6	12	181335	181346	
PHG9_Scaffold_93	64	p2	(TA)6	12	224360	224371	
PHG9_Scaffold_93	67	p2	(TA)6	12	228685	228696	
PHG9_Scaffold_93	69	p2	(TC)6	12	240393	240404	
PHG9_Scaffold_93	74	p2	(AT)8	16	248681	248696	potentially variable
PHG9_Scaffold_93	78	p2	(AC)7	14	254987	255000	potentially variable
PHG9_Scaffold_93	88	p2	(TA)7	14	280334	280347	potentially variable
PHG9_Scaffold_93	89	p2	(GA)6	12	280608	280619	

PHG9_Scaffold_93	90	p2	(AT)6	12	280907	280918	
PHG9_Scaffold_93	96	p2	(TA)8	16	301629	301644	potentially variable
PHG9_Scaffold_93	99	p2	(AC)6	12	322330	322341	
PHG9_Scaffold_93	100	p2	(AG)9	18	323734	323751	potentially variable
PHG9_Scaffold_93	105	p2	(TA)8	16	330622	330637	potentially variable
PHG9_Scaffold_93	108	p2	(GA)7	14	333830	333843	potentially variable
PHG9_Scaffold_93	116	p2	(TA)9	18	360042	360059	potentially variable
PHG9_Scaffold_93	119	p2	(CT)6	12	371684	371695	
PHG9_Scaffold_93	120	p2	(AG)6	12	380169	380180	
PHG9_Scaffold_93	123	p2	(AG)8	16	386700	386715	potentially variable
PHG9_Scaffold_93	124	p2	(TC)8	16	386972	386987	potentially variable
PHG9_Scaffold_93	136	p2	(AT)8	16	427479	427494	potentially variable
PHG9_Scaffold_93	138	p2	(TA)6	12	431915	431926	
PHG9_Scaffold_93	144	p2	(TA)6	12	451847	451858	
PHG9_Scaffold_93	145	p2	(CT)6	12	457266	457277	
PHG9_Scaffold_93	147	p2	(GA)6	12	459950	459961	
PHG9_Scaffold_94	2	p3	(ATT)5	15	11869	11883	potentially variable
PHG9_Scaffold_94	3	p3	(CCA)5	15	16221	16235	potentially variable
PHG9_Scaffold_94	13	p3	(AGG)5	15	62928	62942	potentially

							variable
PHG9_Scaffold_94	16	p2	(AG)6	12	74660	74671	
PHG9_Scaffold_94	17	p2	(CT)6	12	74834	74845	
PHG9_Scaffold_94	22	p2	(AT)6	12	80870	80881	
PHG9_Scaffold_94	25	p3	(TTC)5	15	90664	90678	potentially variable
PHG9_Scaffold_94	29	p2	(TA)6	12	107134	107145	
PHG9_Scaffold_94	47	p3	(TTA)6	18	169463	169480	potentially variable
PHG9_Scaffold_94	48	p2	(TA)6	12	174131	174142	
PHG9_Scaffold_94	50	p2	(AT)7	14	179034	179047	potentially variable
PHG9_Scaffold_94	52	p2	(TA)6	12	182123	182134	
PHG9_Scaffold_94	57	p3	(CCT)5	15	193258	193272	potentially variable
PHG9_Scaffold_94	66	p3	(AAT)5	15	226242	226256	potentially variable
PHG9_Scaffold_94	77	p2	(AC)6	12	279885	279896	
PHG9_Scaffold_94	79	p3	(TAT)5	15	302030	302044	potentially variable
PHG9_Scaffold_94	82	p2	(TA)6	12	316269	316280	
PHG9_Scaffold_94	87	p2	(TA)9	18	331647	331664	potentially variable
PHG9_Scaffold_95	9	p2	(TA)6	12	30496	30507	
PHG9_Scaffold_95	10	p2	(AT)8	16	30645	30660	potentially variable
PHG9_Scaffold_95	11	p2	(TA)8	16	38279	38294	potentially variable
PHG9_Scaffold_96	4	p2	(TA)7	14	7384	7397	potentially

							variable
PHG9_Scaffold_96	8	p2	(AC)6	12	26811	26822	
PHG9_Scaffold_96	14	p3	(CTC)5	15	34929	34943	potentially variable
PHG9_Scaffold_96	36	p2	(GA)6	12	93172	93183	
PHG9_Scaffold_96	38	p2	(TA)8	16	97311	97326	potentially variable
PHG9_Scaffold_96	40	p3	(TTA)6	18	104124	104141	potentially variable
PHG9_Scaffold_96	45	p2	(AT)6	12	111926	111937	
PHG9_Scaffold_96	48	p3	(ATA)5	15	138525	138539	potentially variable
PHG9_Scaffold_96	49	p2	(AT)7	14	140545	140558	potentially variable
PHG9_Scaffold_96	53	p2	(TA)6	12	180870	180881	
PHG9_Scaffold_96	61	p3	(TAA)5	15	240694	240708	potentially variable
PHG9_Scaffold_96	65	p3	(TAA)5	15	271448	271462	potentially variable
PHG9_Scaffold_96	69	p3	(TTA)5	15	309970	309984	potentially variable
PHG9_Scaffold_96	70	p2	(AT)8	16	310113	310128	potentially variable
PHG9_Scaffold_96	71	p2	(AT)7	14	312981	312994	potentially variable
PHG9_Scaffold_96	73	p2	(AT)6	12	315653	315664	
PHG9_Scaffold_96	75	p2	(TA)9	18	325658	325675	potentially variable
PHG9_Scaffold_97	1	p2	(TA)8	16	2080	2095	potentially variable

PHG9_Scaffold_97	6	p3	(TAT)5	15	28248	28262	potentially variable
PHG9_Scaffold_97	16	p2	(AT)7	14	105274	105287	potentially variable
PHG9_Scaffold_97	22	p2	(AT)6	12	115930	115941	
PHG9_Scaffold_97	24	p3	(AAG)5	15	135650	135664	potentially variable
PHG9_Scaffold_97	26	p3	(CAG)5	15	142600	142614	potentially variable
PHG9_Scaffold_98	4	p2	(AT)6	12	5928	5939	
PHG9_Scaffold_98	5	p3	(AAT)5	15	6652	6666	potentially variable
PHG9_Scaffold_98	6	p2	(AT)6	12	11437	11448	
PHG9_Scaffold_98	17	p2	(AT)9	18	66605	66622	potentially variable
PHG9_Scaffold_98	33	p2	(TA)7	14	135128	135141	potentially variable
PHG9_Scaffold_98	42	p2	(AT)8	16	167684	167699	potentially variable
PHG9_Scaffold_98	46	p2	(GA)6	12	179624	179635	
PHG9_Scaffold_99	11	p3	(TTA)6	18	29211	29228	potentially variable
PHG9_Scaffold_99	24	p2	(AT)8	16	67662	67677	potentially variable
PHG9_Scaffold_99	29	p2	(AG)6	12	80943	80954	
PHG9_Scaffold_99	35	p2	(TA)9	18	86976	86993	potentially variable
PHG9_Scaffold_99	42	p3	(TTG)5	15	105452	105466	potentially variable
PHG9_Scaffold_100	10	p3	(GAA)6	18	27914	27931	potentially

							variable
PHG9_Scaffold_100	11	p2	(TA)8	16	46780	46795	potentially variable
PHG9_Scaffold_100	13	p2	(GA)8	16	47746	47761	potentially variable
PHG9_Scaffold_100	26	p3	(AAT)6	18	95481	95498	potentially variable
PHG9_Scaffold_100	27	p6	(AGCATG)8	48	95847	95894	Hypervariable
PHG9_Scaffold_100	29	p2	(TC)6	12	102384	102395	
PHG9_Scaffold_100	30	p3	(CAT)5	15	102645	102659	potentially variable
PHG9_Scaffold_100	36	p2	(CT)6	12	122797	122808	
PHG9_Scaffold_100	39	p2	(CT)8	16	139416	139431	potentially variable
PHG9_Scaffold_100	44	p3	(ATT)5	15	145917	145931	potentially variable
PHG9_Scaffold_100	45	p3	(AGC)5	15	165718	165732	potentially variable
PHG9_Scaffold_100	47	p3	(CTT)6	18	173514	173531	potentially variable
PHG9_Scaffold_100	48	p4	(TATC)5	20	174798	174817	Hypervariable
PHG9_Scaffold_100	50	p3	(AAG)6	18	183554	183571	potentially variable
PHG9_Scaffold_100	51	p2	(AC)8	16	191923	191938	potentially variable
PHG9_Scaffold_101	6	p2	(AG)8	16	22269	22284	potentially variable
PHG9_Scaffold_101	22	p3	(CTT)6	18	47804	47821	potentially variable
PHG9_Scaffold_101	23	p3	(CTG)5	15	48044	48058	potentially

							variable
PHG9_Scaffold_101	33	p2	(AT)8	16	84702	84717	potentially variable
PHG9_Scaffold_101	35	p3	(ATA)5	15	96263	96277	potentially variable
PHG9_Scaffold_101	42	p2	(TA)9	18	105930	105947	potentially variable
PHG9_Scaffold_101	44	p2	(TA)7	14	114928	114941	potentially variable
PHG9_Scaffold_101	52	p3	(GTT)5	15	136241	136255	potentially variable
PHG9_Scaffold_101	59	p3	(TAA)5	15	161352	161366	potentially variable
PHG9_Scaffold_101	64	p2	(AT)6	12	180948	180959	
PHG9_Scaffold_102	8	p2	(CT)8	16	51183	51198	potentially variable
PHG9_Scaffold_102	13	p2	(TA)6	12	71443	71454	
PHG9_Scaffold_102	14	p3	(AAT)5	15	74161	74175	potentially variable
PHG9_Scaffold_102	25	p2	(TA)7	14	105981	105994	potentially variable
PHG9_Scaffold_102	52	p2	(AT)8	16	197115	197130	potentially variable
PHG9_Scaffold_102	54	p3	(TTA)5	15	203102	203116	potentially variable
PHG9_Scaffold_102	56	p2	(GT)6	12	203998	204009	
PHG9_Scaffold_102	65	p2	(TA)9	18	231011	231028	potentially variable
PHG9_Scaffold_102	66	p3	(CCA)5	15	232074	232088	potentially variable

PHG9_Scaffold_102	70	p2	(TA)7	14	249042	249055	potentially variable
PHG9_Scaffold_102	75	p2	(AT)9	18	261444	261461	potentially variable
PHG9_Scaffold_102	76	p3	(AGG)5	15	264802	264816	potentially variable
PHG9_Scaffold_102	77	p3	(GGT)5	15	265004	265018	potentially variable
PHG9_Scaffold_102	84	p3	(CAC)5	15	281633	281647	potentially variable
PHG9_Scaffold_102	96	p2	(TA)8	16	328154	328169	potentially variable
PHG9_Scaffold_102	100	p2	(TA)6	12	350558	350569	
PHG9_Scaffold_102	106	p2	(AT)7	14	384373	384386	potentially variable
PHG9_Scaffold_103	11	p3	(TTA)5	15	55600	55614	potentially variable
PHG9_Scaffold_103	15	p2	(TA)6	12	60320	60331	
PHG9_Scaffold_103	21	p2	(AT)9	18	63324	63341	potentially variable
PHG9_Scaffold_103	24	p2	(TA)7	14	79868	79881	potentially variable
PHG9_Scaffold_103	32	p2	(TA)7	14	116447	116460	potentially variable
PHG9_Scaffold_104	8	p2	(AG)9	18	22549	22566	potentially variable
PHG9_Scaffold_104	11	p3	(ATA)5	15	42686	42700	potentially variable
PHG9_Scaffold_104	15	p2	(TA)6	12	47080	47091	
PHG9_Scaffold_104	18	p2	(AT)7	14	61431	61444	potentially

							variable
PHG9_Scaffold_104	19	p2	(AC)7	14	65101	65114	potentially variable
PHG9_Scaffold_104	23	p2	(TA)6	12	73912	73923	
PHG9_Scaffold_104	30	p2	(AT)9	18	102090	102107	potentially variable
PHG9_Scaffold_104	32	p2	(GA)8	16	106066	106081	potentially variable
PHG9_Scaffold_104	33	p2	(AG)6	12	107175	107186	
PHG9_Scaffold_104	34	p3	(TTA)5	15	108629	108643	potentially variable
PHG9_Scaffold_105	10	p3	(TTA)6	18	29613	29630	potentially variable
PHG9_Scaffold_105	20	p2	(TA)6	12	51363	51374	
PHG9_Scaffold_105	32	p3	(AAT)5	15	83792	83806	potentially variable
PHG9_Scaffold_105	33	p2	(GT)7	14	84188	84201	potentially variable
PHG9_Scaffold_105	49	p2	(AT)7	14	125051	125064	potentially variable
PHG9_Scaffold_105	51	p2	(TA)7	14	133121	133134	potentially variable
PHG9_Scaffold_105	55	p2	(AG)7	14	139555	139568	potentially variable
PHG9_Scaffold_105	56	p2	(CT)6	12	140727	140738	
PHG9_Scaffold_105	60	p3	(ACA)5	15	146075	146089	potentially variable
PHG9_Scaffold_105	62	p2	(CA)6	12	160243	160254	
PHG9_Scaffold_105	63	p2	(TA)9	18	160677	160694	potentially variable

PHG9_Scaffold_105	69	p3	(GTA)5	15	182970	182984	potentially variable
PHG9_Scaffold_105	71	p3	(CTT)5	15	188519	188533	potentially variable
PHG9_Scaffold_105	73	p2	(AT)8	16	194592	194607	potentially variable
PHG9_Scaffold_105	77	p2	(TA)7	14	203239	203252	potentially variable
PHG9_Scaffold_105	81	p2	(AT)6	12	207396	207407	
PHG9_Scaffold_105	87	p2	(TC)8	16	217876	217891	potentially variable
PHG9_Scaffold_105	88	p3	(CAT)5	15	229180	229194	potentially variable
PHG9_Scaffold_105	108	p2	(TA)7	14	277391	277404	potentially variable
PHG9_Scaffold_105	109	p2	(CT)7	14	279039	279052	potentially variable
PHG9_Scaffold_105	110	p3	(ATT)6	18	279740	279757	potentially variable
PHG9_Scaffold_105	127	p2	(TC)6	12	316834	316845	
PHG9_Scaffold_105	144	p3	(CAT)5	15	354472	354486	potentially variable
PHG9_Scaffold_105	145	p3	(AGA)5	15	355238	355252	potentially variable
PHG9_Scaffold_105	148	p2	(TA)9	18	361025	361042	potentially variable
PHG9_Scaffold_105	153	p2	(AT)7	14	385937	385950	potentially variable
PHG9_Scaffold_106	3	p2	(AG)6	12	5664	5675	
PHG9_Scaffold_106	4	p2	(AG)6	12	5870	5881	

PHG9_Scaffold_106	5	p2	(AG)9	18	6122	6139	potentially variable
PHG9_Scaffold_106	9	p2	(AT)8	16	16711	16726	potentially variable
PHG9_Scaffold_106	14	p3	(TAT)6	18	44131	44148	potentially variable
PHG9_Scaffold_106	17	p2	(TA)7	14	53406	53419	potentially variable
PHG9_Scaffold_106	19	p2	(TA)6	12	54344	54355	
PHG9_Scaffold_106	28	p2	(GC)7	14	98341	98354	potentially variable
PHG9_Scaffold_106	29	p2	(TC)8	16	106585	106600	potentially variable
PHG9_Scaffold_107	10	p3	(AGA)5	15	28221	28235	potentially variable
PHG9_Scaffold_107	18	p2	(AT)6	12	65981	65992	
PHG9_Scaffold_107	21	p2	(AT)7	14	76034	76047	potentially variable
PHG9_Scaffold_107	32	p2	(TA)8	16	93413	93428	potentially variable
PHG9_Scaffold_107	41	p2	(TA)8	16	121838	121853	potentially variable
PHG9_Scaffold_107	53	p2	(TA)6	12	178720	178731	
PHG9_Scaffold_107	57	p3	(TAA)5	15	184778	184792	potentially variable
PHG9_Scaffold_107	65	p2	(AG)7	14	202162	202175	potentially variable
PHG9_Scaffold_107	68	p2	(AT)8	16	211702	211717	potentially variable
PHG9_Scaffold_107	69	p3	(AAT)5	15	213369	213383	potentially

							variable
PHG9_Scaffold_107	77	p2	(GA)6	12	228714	228725	
PHG9_Scaffold_107	80	p3	(ATA)5	15	230574	230588	potentially variable
PHG9_Scaffold_107	89	p3	(ATG)5	15	253697	253711	potentially variable
PHG9_Scaffold_107	90	p3	(GAT)5	15	259368	259382	potentially variable
PHG9_Scaffold_107	98	p3	(TCC)5	15	275514	275528	potentially variable
PHG9_Scaffold_107	100	p3	(ATA)6	18	280931	280948	potentially variable
PHG9_Scaffold_107	101	p2	(TA)9	18	288706	288723	potentially variable
PHG9_Scaffold_107	103	p3	(TGC)5	15	293200	293214	potentially variable
PHG9_Scaffold_107	110	p2	(AT)9	18	306542	306559	potentially variable
PHG9_Scaffold_107	121	p3	(CCA)5	15	331092	331106	potentially variable
PHG9_Scaffold_108	14	p2	(TA)7	14	23220	23233	potentially variable
PHG9_Scaffold_108	20	p2	(TA)9	18	32620	32637	potentially variable
PHG9_Scaffold_108	28	p3	(AAT)5	15	49846	49860	potentially variable
PHG9_Scaffold_108	32	p2	(TG)6	12	57459	57470	
PHG9_Scaffold_108	35	p2	(TC)6	12	61794	61805	
PHG9_Scaffold_108	41	p2	(TA)9	18	70343	70360	potentially variable

PHG9_Scaffold_108	51	p3	(TTC)6	18	111242	111259	potentially variable
PHG9_Scaffold_108	52	p3	(CTC)6	18	113495	113512	potentially variable
PHG9_Scaffold_109	8	p2	(CT)6	12	35422	35433	
PHG9_Scaffold_109	15	p3	(ATT)5	15	53839	53853	potentially variable
PHG9_Scaffold_109	19	p2	(AG)6	12	65740	65751	
PHG9_Scaffold_109	21	p2	(TA)6	12	69806	69817	
PHG9_Scaffold_109	24	p2	(CT)6	12	73786	73797	
PHG9_Scaffold_110	3	p2	(TC)8	16	13828	13843	potentially variable
PHG9_Scaffold_110	8	p2	(TA)9	18	31803	31820	potentially variable
PHG9_Scaffold_110	10	p2	(AT)6	12	35315	35326	
PHG9_Scaffold_110	13	p2	(TA)9	18	48326	48343	potentially variable
PHG9_Scaffold_110	16	p2	(AT)7	14	74718	74731	potentially variable
PHG9_Scaffold_110	19	p3	(TCT)5	15	77951	77965	potentially variable
PHG9_Scaffold_110	23	p2	(AC)6	12	102482	102493	
PHG9_Scaffold_110	26	p2	(TA)6	12	106426	106437	
PHG9_Scaffold_110	27	p2	(TC)6	12	115667	115678	
PHG9_Scaffold_110	31	p3	(TGG)5	15	118407	118421	potentially variable
PHG9_Scaffold_110	43	p3	(TAT)5	15	152673	152687	potentially variable
PHG9_Scaffold_110	49	p2	(TA)9	18	164672	164689	potentially

							variable
PHG9_Scaffold_110	51	p2	(TA)6	12	168086	168097	
PHG9_Scaffold_111	4	p3	(AAT)5	15	7810	7824	potentially variable
PHG9_Scaffold_111	13	p2	(TA)7	14	29519	29532	potentially variable
PHG9_Scaffold_111	23	p2	(CA)7	14	52457	52470	potentially variable
PHG9_Scaffold_111	25	p3	(TTG)6	18	61067	61084	potentially variable
PHG9_Scaffold_111	33	p2	(TA)6	12	88021	88032	
PHG9_Scaffold_111	34	p2	(TA)6	12	89385	89396	
PHG9_Scaffold_111	42	p2	(AT)6	12	100099	100110	
PHG9_Scaffold_111	44	p2	(TA)6	12	104414	104425	
PHG9_Scaffold_112	3	p2	(GA)6	12	10576	10587	
PHG9_Scaffold_112	4	p2	(TA)7	14	12635	12648	potentially variable
PHG9_Scaffold_112	6	p2	(TA)8	16	22398	22413	potentially variable
PHG9_Scaffold_112	17	p3	(ACC)5	15	41957	41971	potentially variable
PHG9_Scaffold_112	27	p2	(AG)9	18	68988	69005	potentially variable
PHG9_Scaffold_112	39	p2	(TA)8	16	106441	106456	potentially variable
PHG9_Scaffold_112	41	p2	(AT)6	12	111231	111242	
PHG9_Scaffold_112	48	p6	(TCGACC)5	30	135817	135846	Hypervariable
PHG9_Scaffold_112	61	p3	(AGG)6	18	217935	217952	potentially variable

PHG9_Scaffold_113	3	p2	(TA)8	16	6425	6440	potentially variable
PHG9_Scaffold_113	7	p3	(TTC)5	15	16825	16839	potentially variable
PHG9_Scaffold_113	9	p2	(AT)8	16	28039	28054	potentially variable
PHG9_Scaffold_113	12	p2	(TA)7	14	29899	29912	potentially variable
PHG9_Scaffold_113	14	p2	(TC)7	14	50216	50229	potentially variable
PHG9_Scaffold_113	19	p2	(AT)8	16	68713	68728	potentially variable
PHG9_Scaffold_113	22	p2	(CA)6	12	79208	79219	
PHG9_Scaffold_113	38	p2	(AT)6	12	144065	144076	
PHG9_Scaffold_113	42	p2	(TG)8	16	153005	153020	potentially variable
PHG9_Scaffold_113	46	p2	(GA)8	16	164353	164368	potentially variable
PHG9_Scaffold_114	5	p3	(TTA)5	15	15346	15360	potentially variable
PHG9_Scaffold_114	6	p2	(TA)7	14	15641	15654	potentially variable
PHG9_Scaffold_114	22	p3	(TTA)5	15	63184	63198	potentially variable
PHG9_Scaffold_114	37	p3	(GAA)6	18	111288	111305	potentially variable
PHG9_Scaffold_114	38	p2	(TA)8	16	116663	116678	potentially variable
PHG9_Scaffold_115	1	p2	(TA)6	12	959	970	
PHG9_Scaffold_115	10	p2	(TA)9	18	41065	41082	potentially

							variable
PHG9_Scaffold_115	15	p3	(AAC)5	15	53682	53696	potentially variable
PHG9_Scaffold_115	16	p3	(GTT)5	15	55064	55078	potentially variable
PHG9_Scaffold_115	23	p2	(TA)8	16	84718	84733	potentially variable
PHG9_Scaffold_115	24	p2	(AT)8	16	85264	85279	potentially variable
PHG9_Scaffold_115	53	p2	(TA)6	12	240847	240858	
PHG9_Scaffold_115	61	p2	(AT)6	12	266955	266966	
PHG9_Scaffold_115	64	p2	(AT)7	14	270694	270707	potentially variable
PHG9_Scaffold_115	68	p2	(AT)6	12	291471	291482	
PHG9_Scaffold_115	76	p2	(AT)9	18	338039	338056	potentially variable
PHG9_Scaffold_115	78	p2	(TA)6	12	368803	368814	
PHG9_Scaffold_115	84	p3	(ATT)5	15	381357	381371	potentially variable
PHG9_Scaffold_116	2	p3	(CCT)5	15	8577	8591	potentially variable
PHG9_Scaffold_116	3	p2	(CT)9	18	10733	10750	potentially variable
PHG9_Scaffold_116	34	p2	(TA)8	16	93614	93629	potentially variable
PHG9_Scaffold_116	41	p6	(TTTTCA)7	42	117187	117228	Hypervariable
PHG9_Scaffold_116	44	p2	(AT)8	16	121876	121891	potentially variable
PHG9_Scaffold_116	55	p2	(AT)9	18	145775	145792	potentially variable

PHG9_Scaffold_116	60	p2	(AG)7	14	155923	155936	potentially variable
PHG9_Scaffold_116	66	p3	(CAA)5	15	179702	179716	potentially variable
PHG9_Scaffold_116	71	p3	(TTA)6	18	190664	190681	potentially variable
PHG9_Scaffold_116	73	p2	(AC)6	12	193747	193758	
PHG9_Scaffold_116	76	p3	(CAT)6	18	196158	196175	potentially variable
PHG9_Scaffold_116	78	p3	(ATT)5	15	206478	206492	potentially variable
PHG9_Scaffold_116	85	p3	(CAC)6	18	220821	220838	potentially variable
PHG9_Scaffold_117	2	p2	(TA)6	12	12575	12586	
PHG9_Scaffold_117	8	p2	(CA)7	14	21029	21042	potentially variable
PHG9_Scaffold_117	15	p2	(CT)6	12	59531	59542	
PHG9_Scaffold_117	16	p3	(GAG)5	15	60678	60692	potentially variable
PHG9_Scaffold_117	17	p2	(TA)8	16	62417	62432	potentially variable
PHG9_Scaffold_117	19	p2	(AT)7	14	73349	73362	potentially variable
PHG9_Scaffold_117	20	p3	(TAC)5	15	78641	78655	potentially variable
PHG9_Scaffold_117	21	p2	(TA)6	12	83818	83829	
PHG9_Scaffold_117	22	p2	(TC)9	18	85576	85593	potentially variable
PHG9_Scaffold_117	25	p2	(AG)6	12	91476	91487	
PHG9_Scaffold_117	45	p2	(TA)6	12	139600	139611	

PHG9_Scaffold_118	8	p2	(CT)7	14	28751	28764	potentially variable
PHG9_Scaffold_118	9	p3	(TAA)5	15	29047	29061	potentially variable
PHG9_Scaffold_118	11	p2	(AG)9	18	43715	43732	potentially variable
PHG9_Scaffold_118	15	p3	(ACA)5	15	68340	68354	potentially variable
PHG9_Scaffold_118	19	p3	(ATT)5	15	74270	74284	potentially variable
PHG9_Scaffold_118	24	p3	(TAT)5	15	97241	97255	potentially variable
PHG9_Scaffold_118	28	p2	(GT)7	14	127805	127818	potentially variable
PHG9_Scaffold_118	29	p2	(TC)6	12	130491	130502	
PHG9_Scaffold_118	37	p2	(AT)7	14	164936	164949	potentially variable
PHG9_Scaffold_118	40	p2	(AC)9	18	184253	184270	potentially variable
PHG9_Scaffold_118	44	p2	(CA)6	12	191914	191925	
PHG9_Scaffold_118	46	p2	(TA)7	14	202760	202773	potentially variable
PHG9_Scaffold_118	47	p2	(TA)8	16	207030	207045	potentially variable
PHG9_Scaffold_118	50	p2	(TA)6	12	228063	228074	
PHG9_Scaffold_118	56	p3	(TAT)5	15	231063	231077	potentially variable
PHG9_Scaffold_118	75	p2	(AC)8	16	289066	289081	potentially variable
PHG9_Scaffold_119	15	p2	(AT)6	12	51799	51810	

PHG9_Scaffold_119	18	p2	(CT)8	16	61855	61870	potentially variable
PHG9_Scaffold_119	25	p2	(TA)6	12	95001	95012	
PHG9_Scaffold_119	37	p3	(TAA)5	15	130966	130980	potentially variable
PHG9_Scaffold_119	39	p3	(ATG)5	15	137117	137131	potentially variable
PHG9_Scaffold_120	14	p2	(TC)6	12	32163	32174	
PHG9_Scaffold_120	37	p2	(AG)8	16	83355	83370	potentially variable
PHG9_Scaffold_120	40	p2	(TC)9	18	88366	88383	potentially variable
PHG9_Scaffold_120	41	p2	(AT)6	12	89732	89743	
PHG9_Scaffold_120	56	p2	(AT)8	16	129963	129978	potentially variable
PHG9_Scaffold_120	86	p2	(AT)6	12	192911	192922	
PHG9_Scaffold_120	93	p2	(TA)7	14	202139	202152	potentially variable
PHG9_Scaffold_120	103	p2	(AT)7	14	231631	231644	potentially variable
PHG9_Scaffold_120	106	p3	(AAT)5	15	248998	249012	potentially variable
PHG9_Scaffold_120	117	p3	(GCT)5	15	260383	260397	potentially variable
PHG9_Scaffold_120	119	p3	(AAG)5	15	263039	263053	potentially variable
PHG9_Scaffold_120	122	p2	(GA)6	12	268513	268524	
PHG9_Scaffold_120	125	p3	(CTG)6	18	273877	273894	potentially variable
PHG9_Scaffold_120	127	p2	(CT)6	12	276531	276542	

PHG9_Scaffold_120	129	p3	(AAT)5	15	278498	278512	potentially variable
PHG9_Scaffold_120	137	p2	(TC)6	12	285776	285787	
PHG9_Scaffold_120	142	p3	(AAT)5	15	293565	293579	potentially variable
PHG9_Scaffold_121	4	p2	(AT)8	16	47390	47405	potentially variable
PHG9_Scaffold_121	13	p2	(AT)6	12	117620	117631	
PHG9_Scaffold_122	5	p2	(TA)9	18	7895	7912	potentially variable
PHG9_Scaffold_122	10	p2	(TA)6	12	24911	24922	
PHG9_Scaffold_122	15	p2	(TA)7	14	41625	41638	potentially variable
PHG9_Scaffold_122	28	p3	(GTT)6	18	78699	78716	potentially variable
PHG9_Scaffold_122	30	p2	(GT)6	12	90464	90475	
PHG9_Scaffold_122	32	p3	(TTA)6	18	100764	100781	potentially variable
PHG9_Scaffold_122	38	p2	(TA)6	12	115356	115367	
PHG9_Scaffold_122	41	p2	(CT)6	12	135184	135195	
PHG9_Scaffold_122	42	p3	(ATA)6	18	135432	135449	potentially variable
PHG9_Scaffold_122	44	p2	(TC)9	18	140825	140842	potentially variable
PHG9_Scaffold_122	64	p2	(AG)8	16	210104	210119	potentially variable
PHG9_Scaffold_122	74	p2	(AG)6	12	229194	229205	
PHG9_Scaffold_122	75	p2	(AG)7	14	232135	232148	potentially variable

PHG9_Scaffold_122	77	p3	(ATA)5	15	239102	239116	potentially variable
PHG9_Scaffold_122	83	p2	(TC)9	18	249630	249647	potentially variable
PHG9_Scaffold_122	93	p2	(TA)6	12	277453	277464	
PHG9_Scaffold_122	101	p3	(AGA)5	15	301057	301071	potentially variable
PHG9_Scaffold_122	102	p2	(TA)8	16	306579	306594	potentially variable
PHG9_Scaffold_122	108	p2	(AT)6	12	329674	329685	
PHG9_Scaffold_123	3	p2	(TG)6	12	6390	6401	
PHG9_Scaffold_123	4	p2	(AC)8	16	8002	8017	potentially variable
PHG9_Scaffold_123	7	p2	(AT)6	12	13926	13937	
PHG9_Scaffold_123	11	p2	(AG)7	14	25134	25147	potentially variable
PHG9_Scaffold_123	12	p3	(ACA)6	18	25874	25891	potentially variable
PHG9_Scaffold_123	13	p2	(AT)6	12	29072	29083	
PHG9_Scaffold_123	16	p3	(TAA)5	15	33073	33087	potentially variable
PHG9_Scaffold_123	21	p3	(AGA)6	18	39105	39122	potentially variable
PHG9_Scaffold_123	27	p2	(TA)6	12	63949	63960	
PHG9_Scaffold_123	29	p2	(AT)7	14	72383	72396	potentially variable
PHG9_Scaffold_123	32	p2	(GA)7	14	78613	78626	potentially variable
PHG9_Scaffold_123	38	p2	(AT)6	12	98352	98363	

PHG9_Scaffold_123	40	p2	(AT)6	12	99919	99930	
PHG9_Scaffold_123	45	p2	(TC)7	14	113668	113681	potentially variable
PHG9_Scaffold_123	46	p3	(CCA)5	15	114070	114084	potentially variable
PHG9_Scaffold_123	53	p3	(AAT)5	15	145401	145415	potentially variable
PHG9_Scaffold_123	62	p2	(AT)7	14	172455	172468	potentially variable
PHG9_Scaffold_123	71	p2	(GA)6	12	183179	183190	
PHG9_Scaffold_123	83	p3	(TAT)6	18	201753	201770	potentially variable
PHG9_Scaffold_123	85	p2	(AG)6	12	203970	203981	
PHG9_Scaffold_123	88	p2	(CT)6	12	212553	212564	
PHG9_Scaffold_123	89	p2	(CT)6	12	213505	213516	
PHG9_Scaffold_123	100	p3	(GTT)6	18	234578	234595	potentially variable
PHG9_Scaffold_123	104	p3	(GAA)5	15	249846	249860	potentially variable
PHG9_Scaffold_124	8	p2	(CT)6	12	21855	21866	
PHG9_Scaffold_124	19	p2	(TA)7	14	47909	47922	potentially variable
PHG9_Scaffold_124	20	p2	(TG)9	18	50340	50357	potentially variable
PHG9_Scaffold_124	22	p2	(CT)7	14	59749	59762	potentially variable
PHG9_Scaffold_124	27	p2	(TA)6	12	78659	78670	
PHG9_Scaffold_124	33	p3	(TCT)5	15	86823	86837	potentially variable

PHG9_Scaffold_124	35	p3	(TAA)5	15	89563	89577	potentially variable
PHG9_Scaffold_124	37	p3	(GAT)6	18	97388	97405	potentially variable
PHG9_Scaffold_124	39	p3	(ATT)5	15	99862	99876	potentially variable
PHG9_Scaffold_124	41	p2	(TA)6	12	104443	104454	
PHG9_Scaffold_124	43	p3	(ATT)6	18	107094	107111	potentially variable
PHG9_Scaffold_124	49	p2	(AT)6	12	128788	128799	
PHG9_Scaffold_124	50	p2	(AG)6	12	132678	132689	
PHG9_Scaffold_124	53	p2	(AT)7	14	141750	141763	potentially variable
PHG9_Scaffold_124	63	p2	(TA)8	16	158596	158611	potentially variable
PHG9_Scaffold_125	7	p2	(AG)6	12	17423	17434	
PHG9_Scaffold_125	20	p2	(AT)8	16	80905	80920	potentially variable
PHG9_Scaffold_125	32	p2	(AT)7	14	121662	121675	potentially variable
PHG9_Scaffold_125	33	p6	(GGGTCA)6	36	122489	122524	Hypervariable
PHG9_Scaffold_125	41	p2	(TA)6	12	135509	135520	
PHG9_Scaffold_125	44	p2	(TC)6	12	151479	151490	
PHG9_Scaffold_125	49	p2	(TA)8	16	167903	167918	potentially variable
PHG9_Scaffold_125	54	p2	(TA)7	14	181044	181057	potentially variable
PHG9_Scaffold_125	55	p3	(ATT)5	15	181319	181333	potentially variable

PHG9_Scaffold_125	56	p2	(TA)8	16	182596	182611	potentially variable
PHG9_Scaffold_125	58	p3	(AAC)5	15	185551	185565	potentially variable
PHG9_Scaffold_125	59	p2	(AT)8	16	185695	185710	potentially variable
PHG9_Scaffold_125	62	p2	(AG)8	16	195217	195232	potentially variable
PHG9_Scaffold_125	63	p2	(AC)7	14	206870	206883	potentially variable
PHG9_Scaffold_126	3	p3	(AAT)5	15	14327	14341	potentially variable
PHG9_Scaffold_126	22	p3	(TTC)5	15	68785	68799	potentially variable
PHG9_Scaffold_126	24	p3	(TCC)5	15	78147	78161	potentially variable
PHG9_Scaffold_126	26	p2	(TG)7	14	79416	79429	potentially variable
PHG9_Scaffold_126	35	p2	(AT)6	12	89110	89121	
PHG9_Scaffold_127	2	p2	(AT)7	14	1343	1356	potentially variable
PHG9_Scaffold_127	4	p2	(TA)8	16	4348	4363	potentially variable
PHG9_Scaffold_127	5	p2	(AT)9	18	6812	6829	potentially variable
PHG9_Scaffold_127	17	p3	(GAA)5	15	38020	38034	potentially variable
PHG9_Scaffold_127	20	p3	(AGA)6	18	48599	48616	potentially variable
PHG9_Scaffold_127	22	p2	(GA)8	16	56965	56980	potentially

							variable
PHG9_Scaffold_127	25	p2	(AG)8	16	64050	64065	potentially variable
PHG9_Scaffold_127	26	p2	(AT)6	12	67652	67663	
PHG9_Scaffold_127	27	p2	(AT)8	16	67874	67889	potentially variable
PHG9_Scaffold_127	30	p2	(AG)9	18	93344	93361	potentially variable
PHG9_Scaffold_127	31	p3	(CCT)5	15	93564	93578	potentially variable
PHG9_Scaffold_127	34	p2	(AT)9	18	98185	98202	potentially variable
PHG9_Scaffold_127	36	p2	(AT)6	12	103970	103981	
PHG9_Scaffold_127	37	p3	(GGA)5	15	104542	104556	potentially variable
PHG9_Scaffold_127	39	p2	(AT)6	12	108778	108789	
PHG9_Scaffold_127	47	p2	(AT)6	12	123485	123496	
PHG9_Scaffold_127	50	p2	(CT)6	12	128247	128258	
PHG9_Scaffold_127	56	p3	(AAT)5	15	144800	144814	potentially variable
PHG9_Scaffold_127	57	p3	(ATA)6	18	150520	150537	potentially variable
PHG9_Scaffold_127	62	p2	(TA)7	14	171508	171521	potentially variable
PHG9_Scaffold_128	1	p3	(TTA)6	18	2632	2649	potentially variable
PHG9_Scaffold_128	3	p2	(AT)6	12	13026	13037	
PHG9_Scaffold_128	4	p2	(TA)7	14	20893	20906	potentially variable

PHG9_Scaffold_128	11	p2	(AG)7	14	58301	58314	potentially variable
PHG9_Scaffold_128	12	p2	(TC)7	14	58447	58460	potentially variable
PHG9_Scaffold_128	17	p2	(AT)9	18	83447	83464	potentially variable
PHG9_Scaffold_128	30	p3	(GAA)6	18	136733	136750	potentially variable
PHG9_Scaffold_128	34	p3	(TAT)5	15	155229	155243	potentially variable
PHG9_Scaffold_128	36	p2	(AT)6	12	158599	158610	
PHG9_Scaffold_128	41	p3	(ATA)6	18	163027	163044	potentially variable
PHG9_Scaffold_128	44	p2	(AT)9	18	168309	168326	potentially variable
PHG9_Scaffold_128	54	p2	(CT)6	12	196406	196417	
PHG9_Scaffold_128	55	p2	(TA)9	18	200010	200027	potentially variable
PHG9_Scaffold_128	56	p2	(AT)9	18	206953	206970	potentially variable
PHG9_Scaffold_128	59	p2	(AT)6	12	216949	216960	
PHG9_Scaffold_128	61	p3	(CGT)5	15	220067	220081	potentially variable
PHG9_Scaffold_128	69	p2	(TA)7	14	257271	257284	potentially variable
PHG9_Scaffold_128	70	p2	(CT)6	12	261222	261233	
PHG9_Scaffold_128	72	p2	(TA)7	14	265249	265262	potentially variable
PHG9_Scaffold_128	73	p2	(TA)6	12	266732	266743	
PHG9_Scaffold_128	74	p3	(GAA)6	18	272291	272308	potentially

							variable
PHG9_Scaffold_128	77	p3	(ATT)5	15	275763	275777	potentially variable
PHG9_Scaffold_128	92	p2	(AG)8	16	321605	321620	potentially variable
PHG9_Scaffold_128	96	p3	(ATC)5	15	332148	332162	potentially variable
PHG9_Scaffold_128	97	p3	(TTC)5	15	333786	333800	potentially variable
PHG9_Scaffold_128	98	p3	(ATG)5	15	334918	334932	potentially variable
PHG9_Scaffold_128	99	p3	(TAT)5	15	336343	336357	potentially variable
PHG9_Scaffold_128	117	p3	(CTG)5	15	383538	383552	potentially variable
PHG9_Scaffold_129	6	p2	(AC)8	16	24673	24688	potentially variable
PHG9_Scaffold_129	13	p2	(AT)8	16	61899	61914	potentially variable
PHG9_Scaffold_129	15	p3	(CTT)5	15	68066	68080	potentially variable
PHG9_Scaffold_129	18	p3	(TCA)5	15	74520	74534	potentially variable
PHG9_Scaffold_129	19	p2	(AT)6	12	74858	74869	
PHG9_Scaffold_129	25	p2	(TA)8	16	83315	83330	potentially variable
PHG9_Scaffold_129	29	p2	(TA)8	16	88753	88768	potentially variable
PHG9_Scaffold_129	30	p2	(TA)6	12	93284	93295	
PHG9_Scaffold_129	32	p2	(AT)6	12	113882	113893	

PHG9_Scaffold_129	34	p3	(TAT)6	18	124906	124923	potentially variable
PHG9_Scaffold_129	45	p2	(CT)7	14	167222	167235	potentially variable
PHG9_Scaffold_129	54	p3	(ATA)5	15	188851	188865	potentially variable
PHG9_Scaffold_129	56	p2	(TA)6	12	195299	195310	
PHG9_Scaffold_130	5	p3	(AAT)6	18	17405	17422	potentially variable
PHG9_Scaffold_130	14	p3	(TTA)5	15	43547	43561	potentially variable
PHG9_Scaffold_130	17	p2	(AT)6	12	54449	54460	
PHG9_Scaffold_130	18	p2	(TA)9	18	59467	59484	potentially variable
PHG9_Scaffold_130	20	p3	(GAA)5	15	63709	63723	potentially variable
PHG9_Scaffold_130	28	p2	(AG)6	12	102289	102300	
PHG9_Scaffold_130	31	p3	(AAT)5	15	113969	113983	potentially variable
PHG9_Scaffold_130	34	p3	(ATT)6	18	121159	121176	potentially variable
PHG9_Scaffold_130	36	p3	(GTG)5	15	122458	122472	potentially variable
PHG9_Scaffold_131	3	p2	(AT)6	12	1044	1055	
PHG9_Scaffold_131	7	p2	(AT)6	12	41636	41647	
PHG9_Scaffold_131	9	p2	(GT)6	12	58962	58973	
PHG9_Scaffold_131	14	p2	(TG)8	16	83153	83168	potentially variable
PHG9_Scaffold_131	17	p2	(AT)8	16	89051	89066	potentially variable

PHG9_Scaffold_131	24	p3	(AAT)5	15	125699	125713	potentially variable
PHG9_Scaffold_131	25	p2	(CA)7	14	127620	127633	potentially variable
PHG9_Scaffold_132	1	p3	(TCC)6	18	9917	9934	potentially variable
PHG9_Scaffold_132	3	p3	(GTG)5	15	18220	18234	potentially variable
PHG9_Scaffold_132	8	p2	(TA)6	12	26708	26719	
PHG9_Scaffold_132	13	p2	(AT)6	12	45842	45853	
PHG9_Scaffold_132	15	p2	(AT)7	14	56329	56342	potentially variable
PHG9_Scaffold_132	19	p2	(GT)7	14	66668	66681	potentially variable
PHG9_Scaffold_132	22	p3	(AAG)5	15	75328	75342	potentially variable
PHG9_Scaffold_132	23	p2	(TC)6	12	82155	82166	
PHG9_Scaffold_132	25	p3	(ATT)6	18	83756	83773	potentially variable
PHG9_Scaffold_132	39	p3	(ATT)5	15	115038	115052	potentially variable
PHG9_Scaffold_132	46	p2	(AT)7	14	131542	131555	potentially variable
PHG9_Scaffold_132	52	p2	(TA)9	18	140111	140128	potentially variable
PHG9_Scaffold_132	66	p2	(TC)6	12	191117	191128	
PHG9_Scaffold_132	67	p2	(AG)6	12	192498	192509	
PHG9_Scaffold_132	74	p3	(AAT)6	18	210318	210335	potentially variable
PHG9_Scaffold_132	77	p2	(CT)8	16	247838	247853	potentially

							variable
PHG9_Scaffold_132	82	p2	(AT)6	12	268215	268226	
PHG9_Scaffold_133	16	p2	(CT)9	18	94088	94105	potentially variable
PHG9_Scaffold_133	26	p2	(TA)6	12	119357	119368	
PHG9_Scaffold_133	29	p3	(AAG)6	18	122055	122072	potentially variable
PHG9_Scaffold_133	33	p2	(CT)6	12	126313	126324	
PHG9_Scaffold_133	34	p3	(TAT)5	15	132808	132822	potentially variable
PHG9_Scaffold_134	2	p3	(TTC)5	15	1629	1643	potentially variable
PHG9_Scaffold_134	3	p2	(TG)8	16	2703	2718	potentially variable
PHG9_Scaffold_134	6	p3	(AAT)5	15	9215	9229	potentially variable
PHG9_Scaffold_134	12	p2	(TC)6	12	26136	26147	
PHG9_Scaffold_134	16	p2	(TC)7	14	41770	41783	potentially variable
PHG9_Scaffold_134	17	p3	(CTT)5	15	42027	42041	potentially variable
PHG9_Scaffold_134	23	p2	(TA)8	16	62016	62031	potentially variable
PHG9_Scaffold_134	25	p2	(AT)8	16	63490	63505	potentially variable
PHG9_Scaffold_134	26	p2	(TC)6	12	67200	67211	
PHG9_Scaffold_134	44	p2	(AT)6	12	116027	116038	
PHG9_Scaffold_134	46	p3	(ATC)5	15	121925	121939	potentially variable

PHG9_Scaffold_134	49	p2	(TG)7	14	130049	130062	potentially variable
PHG9_Scaffold_134	53	p2	(AG)9	18	135473	135490	potentially variable
PHG9_Scaffold_134	54	p2	(CT)6	12	137223	137234	
PHG9_Scaffold_134	61	p2	(AT)6	12	177249	177260	
PHG9_Scaffold_134	70	p3	(ATT)5	15	213483	213497	potentially variable
PHG9_Scaffold_134	71	p2	(TA)8	16	218129	218144	potentially variable
PHG9_Scaffold_135	3	p2	(TA)6	12	8247	8258	
PHG9_Scaffold_135	5	p2	(TA)6	12	9649	9660	
PHG9_Scaffold_135	7	p2	(AC)8	16	17585	17600	potentially variable
PHG9_Scaffold_135	8	p2	(AT)6	12	23318	23329	
PHG9_Scaffold_135	10	p2	(TA)6	12	25761	25772	
PHG9_Scaffold_135	11	p2	(AT)6	12	32294	32305	
PHG9_Scaffold_135	35	p2	(TA)6	12	106789	106800	
PHG9_Scaffold_135	37	p2	(TA)6	12	121068	121079	
PHG9_Scaffold_135	42	p2	(AT)9	18	143738	143755	potentially variable
PHG9_Scaffold_135	48	p3	(AAT)5	15	165697	165711	potentially variable
PHG9_Scaffold_135	49	p2	(AT)6	12	172645	172656	
PHG9_Scaffold_135	50	p3	(ATT)5	15	180398	180412	potentially variable
PHG9_Scaffold_135	68	p2	(AT)7	14	230499	230512	potentially variable
PHG9_Scaffold_135	72	p2	(TG)6	12	244045	244056	

PHG9_Scaffold_135	77	p2	(AT)7	14	268580	268593	potentially variable
PHG9_Scaffold_135	80	p2	(AT)6	12	274403	274414	
PHG9_Scaffold_136	3	p3	(TAT)5	15	11463	11477	potentially variable
PHG9_Scaffold_136	7	p3	(GAA)5	15	28493	28507	potentially variable
PHG9_Scaffold_136	8	p2	(TA)6	12	33666	33677	
PHG9_Scaffold_136	11	p3	(ATT)5	15	36583	36597	potentially variable
PHG9_Scaffold_136	17	p2	(AG)7	14	51365	51378	potentially variable
PHG9_Scaffold_136	36	p2	(AT)6	12	112047	112058	
PHG9_Scaffold_137	4	p2	(TA)8	16	14951	14966	potentially variable
PHG9_Scaffold_137	16	p2	(TA)8	16	71609	71624	potentially variable
PHG9_Scaffold_137	26	p2	(AC)7	14	118420	118433	potentially variable
PHG9_Scaffold_137	28	p2	(TA)6	12	121914	121925	
PHG9_Scaffold_137	32	p2	(AT)9	18	131610	131627	potentially variable
PHG9_Scaffold_137	34	p2	(TA)7	14	133325	133338	potentially variable
PHG9_Scaffold_138	14	p2	(GT)6	12	56307	56318	
PHG9_Scaffold_139	3	p2	(AG)9	18	6626	6643	potentially variable
PHG9_Scaffold_139	10	p2	(TA)6	12	31597	31608	
PHG9_Scaffold_139	11	p2	(CT)9	18	33198	33215	potentially variable

PHG9_Scaffold_139	15	p3	(TTA)5	15	42926	42940	potentially variable
PHG9_Scaffold_139	21	p2	(AT)6	12	67728	67739	
PHG9_Scaffold_139	25	p2	(GT)8	16	70520	70535	potentially variable
PHG9_Scaffold_140	3	p2	(AT)6	12	2415	2426	
PHG9_Scaffold_140	9	p2	(TA)6	12	19261	19272	
PHG9_Scaffold_140	11	p2	(AT)6	12	21795	21806	
PHG9_Scaffold_140	12	p3	(TCT)5	15	22578	22592	potentially variable
PHG9_Scaffold_140	13	p2	(CT)8	16	22988	23003	potentially variable
PHG9_Scaffold_140	16	p2	(GT)6	12	34573	34584	
PHG9_Scaffold_140	31	p3	(ACC)6	18	78870	78887	potentially variable
PHG9_Scaffold_140	38	p2	(AG)6	12	92546	92557	
PHG9_Scaffold_140	46	p2	(GT)6	12	113913	113924	
PHG9_Scaffold_140	47	p2	(TA)8	16	117939	117954	potentially variable
PHG9_Scaffold_140	48	p2	(TA)6	12	118852	118863	
PHG9_Scaffold_140	50	p3	(GTG)5	15	119921	119935	potentially variable
PHG9_Scaffold_140	55	p2	(TA)6	12	147690	147701	
PHG9_Scaffold_140	73	p3	(CCT)5	15	185685	185699	potentially variable
PHG9_Scaffold_140	87	p2	(AT)6	12	223369	223380	
PHG9_Scaffold_140	88	p3	(ATG)5	15	223490	223504	potentially variable
PHG9_Scaffold_140	89	p2	(TA)7	14	224027	224040	potentially

							variable
PHG9_Scaffold_140	90	p3	(TTA)5	15	225410	225424	potentially variable
PHG9_Scaffold_140	92	p2	(TA)7	14	226951	226964	potentially variable
PHG9_Scaffold_140	95	p2	(AT)6	12	239168	239179	
PHG9_Scaffold_140	103	p2	(TA)7	14	257028	257041	potentially variable
PHG9_Scaffold_140	105	p3	(TAT)5	15	257911	257925	potentially variable
PHG9_Scaffold_140	112	p3	(TAA)6	18	289567	289584	potentially variable
PHG9_Scaffold_140	115	p2	(AT)7	14	293440	293453	potentially variable
PHG9_Scaffold_140	117	p2	(TG)7	14	297033	297046	potentially variable
PHG9_Scaffold_140	126	p2	(AT)7	14	316253	316266	potentially variable
PHG9_Scaffold_140	131	p3	(TTA)5	15	329304	329318	potentially variable
PHG9_Scaffold_140	132	p2	(TA)9	18	329601	329618	potentially variable
PHG9_Scaffold_140	142	p2	(TA)6	12	356037	356048	
PHG9_Scaffold_140	149	p2	(AT)6	12	377884	377895	
PHG9_Scaffold_140	151	p3	(TAA)5	15	397362	397376	potentially variable
PHG9_Scaffold_142	1	p2	(TA)8	16	2070	2085	potentially variable
PHG9_Scaffold_142	3	p3	(AAG)5	15	16580	16594	potentially variable

PHG9_Scaffold_142	6	p2	(AT)9	18	21472	21489	potentially variable
PHG9_Scaffold_142	10	p2	(TC)6	12	34284	34295	
PHG9_Scaffold_142	17	p3	(TTC)5	15	58702	58716	potentially variable
PHG9_Scaffold_142	18	p3	(ATA)5	15	66634	66648	potentially variable
PHG9_Scaffold_142	19	p2	(TA)6	12	72749	72760	
PHG9_Scaffold_142	21	p2	(AT)6	12	73675	73686	
PHG9_Scaffold_142	27	p3	(TTA)5	15	88056	88070	potentially variable
PHG9_Scaffold_142	35	p2	(AT)6	12	105933	105944	
PHG9_Scaffold_143	1	p3	(TAA)5	15	3266	3280	potentially variable
PHG9_Scaffold_143	8	p2	(AT)7	14	11284	11297	potentially variable
PHG9_Scaffold_143	9	p2	(AT)8	16	12750	12765	potentially variable
PHG9_Scaffold_144	7	p2	(TA)6	12	10426	10437	
PHG9_Scaffold_144	13	p2	(TA)6	12	33685	33696	
PHG9_Scaffold_144	17	p2	(TA)6	12	43114	43125	
PHG9_Scaffold_145	6	p2	(TA)7	14	30546	30559	potentially variable
PHG9_Scaffold_145	19	p2	(TA)7	14	53594	53607	potentially variable
PHG9_Scaffold_145	27	p2	(TA)7	14	64344	64357	potentially variable
PHG9_Scaffold_145	37	p2	(GA)7	14	80825	80838	potentially variable

PHG9_Scaffold_145	54	p2	(TA)6	12	132788	132799	
PHG9_Scaffold_145	56	p2	(TA)9	18	138247	138264	potentially variable
PHG9_Scaffold_145	63	p2	(GT)7	14	176717	176730	potentially variable
PHG9_Scaffold_145	71	p3	(TGA)5	15	200217	200231	potentially variable
PHG9_Scaffold_145	77	p2	(TA)8	16	213212	213227	potentially variable
PHG9_Scaffold_145	96	p2	(TA)6	12	247313	247324	
PHG9_Scaffold_145	98	p2	(TA)6	12	255272	255283	
PHG9_Scaffold_145	102	p3	(CTT)5	15	264334	264348	potentially variable
PHG9_Scaffold_145	103	p2	(TA)6	12	277068	277079	
PHG9_Scaffold_146	5	p3	(CAC)5	15	23290	23304	potentially variable
PHG9_Scaffold_146	8	p3	(TCA)5	15	28489	28503	potentially variable
PHG9_Scaffold_146	11	p2	(CA)6	12	47244	47255	
PHG9_Scaffold_146	16	p2	(GA)6	12	69511	69522	
PHG9_Scaffold_146	19	p2	(TA)7	14	72820	72833	potentially variable
PHG9_Scaffold_146	21	p2	(TA)6	12	77064	77075	
PHG9_Scaffold_147	1	p3	(TTG)6	18	492	509	potentially variable
PHG9_Scaffold_148	24	p3	(TAA)5	15	76612	76626	potentially variable
PHG9_Scaffold_148	26	p2	(TA)6	12	78031	78042	
PHG9_Scaffold_148	35	p2	(TA)8	16	98689	98704	potentially

							variable
PHG9_Scaffold_148	40	p2	(AT)9	18	118152	118169	potentially variable
PHG9_Scaffold_148	41	p2	(AT)9	18	119521	119538	potentially variable
PHG9_Scaffold_148	43	p3	(ACT)5	15	144913	144927	potentially variable
PHG9_Scaffold_148	47	p3	(CGG)5	15	155657	155671	potentially variable
PHG9_Scaffold_148	49	p2	(AT)7	14	160604	160617	potentially variable
PHG9_Scaffold_148	54	p2	(CA)6	12	169492	169503	
PHG9_Scaffold_149	5	p2	(TC)6	12	18100	18111	
PHG9_Scaffold_149	11	p2	(AT)8	16	47968	47983	potentially variable
PHG9_Scaffold_149	25	p2	(AT)6	12	115831	115842	
PHG9_Scaffold_149	33	p2	(AT)8	16	157449	157464	potentially variable
PHG9_Scaffold_149	34	p2	(TC)6	12	160066	160077	
PHG9_Scaffold_150	6	p3	(GGA)6	18	14583	14600	potentially variable
PHG9_Scaffold_150	7	p3	(AAG)5	15	14955	14969	potentially variable
PHG9_Scaffold_150	11	p3	(ACC)5	15	20791	20805	potentially variable
PHG9_Scaffold_150	21	p2	(AC)6	12	37344	37355	
PHG9_Scaffold_150	26	p2	(AT)9	18	47879	47896	potentially variable
PHG9_Scaffold_150	28	p2	(TA)7	14	58088	58101	potentially variable

PHG9_Scaffold_150	31	p3	(CTT)5	15	65364	65378	potentially variable
PHG9_Scaffold_150	32	p3	(TTC)6	18	66875	66892	potentially variable
PHG9_Scaffold_150	45	p2	(TA)6	12	97000	97011	
PHG9_Scaffold_151	1	p2	(TA)7	14	1736	1749	potentially variable
PHG9_Scaffold_151	9	p2	(AT)6	12	11504	11515	
PHG9_Scaffold_151	11	p2	(CT)6	12	12886	12897	
PHG9_Scaffold_151	12	p2	(TA)7	14	14014	14027	potentially variable
PHG9_Scaffold_151	14	p2	(CT)6	12	16710	16721	
PHG9_Scaffold_151	15	p2	(AT)6	12	17914	17925	
PHG9_Scaffold_151	16	p2	(AT)6	12	18956	18967	
PHG9_Scaffold_151	18	p2	(TG)9	18	20314	20331	potentially variable
PHG9_Scaffold_151	19	p2	(TA)6	12	20638	20649	
PHG9_Scaffold_151	24	p2	(AT)9	18	27467	27484	potentially variable
PHG9_Scaffold_151	37	p2	(TG)6	12	54483	54494	
PHG9_Scaffold_151	38	p2	(TA)7	14	59170	59183	potentially variable
PHG9_Scaffold_151	42	p2	(TA)8	16	74947	74962	potentially variable
PHG9_Scaffold_151	48	p3	(TGA)5	15	89766	89780	potentially variable
PHG9_Scaffold_151	52	p3	(TTC)5	15	103897	103911	potentially variable
PHG9_Scaffold_151	53	p3	(GGA)6	18	104452	104469	potentially

							variable
PHG9_Scaffold_152	14	p2	(TA)7	14	26288	26301	potentially variable
PHG9_Scaffold_152	17	p2	(TA)9	18	31204	31221	potentially variable
PHG9_Scaffold_152	18	p2	(TA)6	12	32664	32675	
PHG9_Scaffold_152	26	p3	(ATA)5	15	50048	50062	potentially variable
PHG9_Scaffold_152	30	p2	(AT)7	14	58783	58796	potentially variable
PHG9_Scaffold_152	40	p3	(TTC)5	15	71284	71298	potentially variable
PHG9_Scaffold_152	44	p3	(AAT)5	15	79377	79391	potentially variable
PHG9_Scaffold_152	51	p2	(TA)8	16	89396	89411	potentially variable
PHG9_Scaffold_152	55	p2	(TA)6	12	95595	95606	
PHG9_Scaffold_152	56	p2	(TA)6	12	96696	96707	
PHG9_Scaffold_152	58	p3	(TAT)5	15	98036	98050	potentially variable
PHG9_Scaffold_152	59	p3	(GGA)5	15	101666	101680	potentially variable
PHG9_Scaffold_152	64	p2	(AT)9	18	118178	118195	potentially variable
PHG9_Scaffold_152	65	p2	(TA)8	16	118901	118916	potentially variable
PHG9_Scaffold_152	69	p3	(ATG)5	15	125355	125369	potentially variable
PHG9_Scaffold_152	80	p2	(AT)8	16	160099	160114	potentially variable

PHG9_Scaffold_152	87	p2	(AT)7	14	166135	166148	potentially variable
PHG9_Scaffold_152	93	p2	(CA)6	12	178519	178530	
PHG9_Scaffold_152	94	p3	(TCT)6	18	180144	180161	potentially variable
PHG9_Scaffold_152	96	p2	(AT)9	18	182350	182367	potentially variable
PHG9_Scaffold_152	101	p3	(AGA)5	15	200695	200709	potentially variable
PHG9_Scaffold_153	2	p2	(AT)6	12	7179	7190	
PHG9_Scaffold_153	26	p2	(TG)7	14	87271	87284	potentially variable
PHG9_Scaffold_153	37	p2	(CT)6	12	113569	113580	
PHG9_Scaffold_153	38	p3	(GTT)5	15	116391	116405	potentially variable
PHG9_Scaffold_153	45	p2	(TC)6	12	154319	154330	
PHG9_Scaffold_153	46	p2	(TA)7	14	158011	158024	potentially variable
PHG9_Scaffold_153	49	p3	(TTA)5	15	175131	175145	potentially variable
PHG9_Scaffold_154	5	p2	(CA)8	16	6638	6653	potentially variable
PHG9_Scaffold_154	8	p2	(GA)6	12	8619	8630	
PHG9_Scaffold_154	9	p6	(TTCCTA)5	30	8871	8900	Hypervariable
PHG9_Scaffold_154	10	p2	(TA)7	14	9143	9156	potentially variable
PHG9_Scaffold_154	15	p2	(TA)6	12	25203	25214	
PHG9_Scaffold_154	17	p2	(TG)6	12	29275	29286	
PHG9_Scaffold_154	18	p2	(AG)8	16	29951	29966	potentially

							variable
PHG9_Scaffold_154	22	p2	(AG)7	14	37204	37217	potentially variable
PHG9_Scaffold_154	32	p2	(GA)9	18	66039	66056	potentially variable
PHG9_Scaffold_154	36	p2	(AG)7	14	71005	71018	potentially variable
PHG9_Scaffold_154	45	p2	(AT)7	14	91901	91914	potentially variable
PHG9_Scaffold_154	52	p2	(AT)9	18	111542	111559	potentially variable
PHG9_Scaffold_154	53	p3	(GGA)6	18	114258	114275	potentially variable
PHG9_Scaffold_154	55	p2	(TA)9	18	138667	138684	potentially variable
PHG9_Scaffold_154	58	p2	(TA)7	14	147755	147768	potentially variable
PHG9_Scaffold_154	63	p3	(TGA)6	18	181543	181560	potentially variable
PHG9_Scaffold_154	69	p2	(TA)8	16	187925	187940	potentially variable
PHG9_Scaffold_155	14	p2	(AT)8	16	61031	61046	potentially variable
PHG9_Scaffold_155	17	p2	(TA)8	16	71727	71742	potentially variable
PHG9_Scaffold_155	20	p2	(TA)6	12	90132	90143	
PHG9_Scaffold_155	31	p2	(TA)7	14	115025	115038	potentially variable
PHG9_Scaffold_155	32	p2	(AT)6	12	116434	116445	
PHG9_Scaffold_156	5	p2	(TC)8	16	22760	22775	potentially

							variable
PHG9_Scaffold_156	8	p2	(AT)8	16	35426	35441	potentially variable
PHG9_Scaffold_156	19	p2	(AT)6	12	65985	65996	
PHG9_Scaffold_156	24	p2	(AT)6	12	74649	74660	
PHG9_Scaffold_156	28	p2	(TA)8	16	82773	82788	potentially variable
PHG9_Scaffold_156	38	p2	(TA)7	14	123620	123633	potentially variable
PHG9_Scaffold_156	45	p3	(GTC)5	15	139960	139974	potentially variable
PHG9_Scaffold_156	46	p3	(AGA)5	15	140165	140179	potentially variable
PHG9_Scaffold_156	55	p2	(TC)6	12	154341	154352	
PHG9_Scaffold_156	61	p3	(TAG)5	15	167182	167196	potentially variable
PHG9_Scaffold_156	68	p2	(GT)6	12	182649	182660	
PHG9_Scaffold_156	70	p2	(AG)6	12	194364	194375	
PHG9_Scaffold_156	72	p2	(TA)6	12	198179	198190	
PHG9_Scaffold_156	74	p2	(TA)6	12	205075	205086	
PHG9_Scaffold_156	78	p3	(GAT)5	15	212602	212616	potentially variable
PHG9_Scaffold_156	86	p2	(AG)9	18	222524	222541	potentially variable
PHG9_Scaffold_156	88	p2	(TA)6	12	226395	226406	
PHG9_Scaffold_156	92	p2	(AG)6	12	235842	235853	
PHG9_Scaffold_157	5	p3	(TTA)5	15	9919	9933	potentially variable
PHG9_Scaffold_157	9	p3	(TTC)5	15	28451	28465	potentially

							variable
PHG9_Scaffold_157	11	p2	(TA)7	14	35125	35138	potentially variable
PHG9_Scaffold_157	12	p2	(AG)7	14	36130	36143	potentially variable
PHG9_Scaffold_157	13	p2	(CT)8	16	40705	40720	potentially variable
PHG9_Scaffold_157	14	p2	(GA)9	18	45676	45693	potentially variable
PHG9_Scaffold_157	24	p2	(AT)7	14	101080	101093	potentially variable
PHG9_Scaffold_157	25	p2	(AT)6	12	106742	106753	
PHG9_Scaffold_157	28	p2	(TA)7	14	119745	119758	potentially variable
PHG9_Scaffold_157	31	p2	(TA)6	12	123927	123938	
PHG9_Scaffold_157	33	p2	(AT)7	14	127491	127504	potentially variable
PHG9_Scaffold_157	34	p3	(ATT)5	15	131916	131930	potentially variable
PHG9_Scaffold_157	38	p2	(AT)6	12	135425	135436	
PHG9_Scaffold_157	39	p2	(TA)6	12	135856	135867	
PHG9_Scaffold_157	41	p2	(TA)6	12	138957	138968	
PHG9_Scaffold_157	47	p2	(TA)6	12	171283	171294	
PHG9_Scaffold_157	48	p2	(TA)7	14	177097	177110	potentially variable
PHG9_Scaffold_157	61	p6	(GCAGAA)5	30	196223	196252	Hypervariable
PHG9_Scaffold_157	72	p2	(CT)7	14	227758	227771	potentially variable
PHG9_Scaffold_157	76	p3	(GAT)5	15	236750	236764	potentially

							variable
PHG9_Scaffold_157	78	p2	(TA)6	12	240797	240808	
PHG9_Scaffold_157	96	p2	(TA)7	14	296163	296176	potentially variable
PHG9_Scaffold_157	97	p2	(AT)6	12	296486	296497	
PHG9_Scaffold_157	98	p2	(TC)6	12	298140	298151	
PHG9_Scaffold_157	99	p3	(AAT)5	15	299728	299742	potentially variable
PHG9_Scaffold_158	2	p2	(TA)6	12	24337	24348	
PHG9_Scaffold_158	6	p2	(TA)6	12	69981	69992	
PHG9_Scaffold_158	17	p2	(TA)7	14	123566	123579	potentially variable
PHG9_Scaffold_158	19	p2	(GA)6	12	125323	125334	
PHG9_Scaffold_158	26	p2	(AT)7	14	165528	165541	potentially variable
PHG9_Scaffold_158	28	p3	(AAG)5	15	181729	181743	potentially variable
PHG9_Scaffold_159	1	p3	(AAT)5	15	559	573	potentially variable
PHG9_Scaffold_159	14	p2	(AC)7	14	45500	45513	potentially variable
PHG9_Scaffold_159	15	p2	(CA)6	12	46135	46146	
PHG9_Scaffold_159	16	p2	(AT)6	12	46893	46904	
PHG9_Scaffold_159	20	p2	(AT)6	12	62010	62021	
PHG9_Scaffold_159	29	p3	(TCA)5	15	85994	86008	potentially variable
PHG9_Scaffold_159	35	p3	(CCA)5	15	99755	99769	potentially variable
PHG9_Scaffold_159	38	p2	(AT)6	12	111883	111894	

PHG9_Scaffold_159	39	p2	(AT)6	12	112623	112634	
PHG9_Scaffold_159	40	p2	(TG)7	14	113407	113420	potentially variable
PHG9_Scaffold_159	42	p2	(TA)8	16	122912	122927	potentially variable
PHG9_Scaffold_159	50	p3	(AAT)6	18	137766	137783	potentially variable
PHG9_Scaffold_159	54	p2	(AT)7	14	145178	145191	potentially variable
PHG9_Scaffold_159	55	p2	(AC)8	16	148603	148618	potentially variable
PHG9_Scaffold_159	56	p2	(AT)6	12	152043	152054	
PHG9_Scaffold_159	57	p2	(AT)7	14	160595	160608	potentially variable
PHG9_Scaffold_159	63	p3	(AAT)5	15	169921	169935	potentially variable
PHG9_Scaffold_159	67	p2	(AT)7	14	191987	192000	potentially variable
PHG9_Scaffold_159	70	p3	(GTT)5	15	198314	198328	potentially variable
PHG9_Scaffold_159	72	p3	(AAT)5	15	203220	203234	potentially variable
PHG9_Scaffold_159	76	p2	(AT)6	12	218095	218106	
PHG9_Scaffold_160	2	p2	(CT)7	14	1656	1669	potentially variable
PHG9_Scaffold_161	5	p3	(ACC)5	15	24977	24991	potentially variable
PHG9_Scaffold_161	6	p2	(TC)6	12	27432	27443	
PHG9_Scaffold_161	8	p2	(CA)8	16	28193	28208	potentially variable

PHG9_Scaffold_161	13	p2	(AT)7	14	38148	38161	potentially variable
PHG9_Scaffold_161	32	p3	(AGA)5	15	75853	75867	potentially variable
PHG9_Scaffold_161	33	p2	(GA)8	16	75977	75992	potentially variable
PHG9_Scaffold_161	42	p2	(TA)6	12	94322	94333	
PHG9_Scaffold_161	43	p2	(AT)8	16	97281	97296	potentially variable
PHG9_Scaffold_161	45	p2	(TA)6	12	102847	102858	
PHG9_Scaffold_161	46	p3	(ATA)5	15	103207	103221	potentially variable
PHG9_Scaffold_161	48	p2	(AG)6	12	106684	106695	
PHG9_Scaffold_161	50	p2	(AT)6	12	110738	110749	
PHG9_Scaffold_161	52	p2	(TA)6	12	115701	115712	
PHG9_Scaffold_161	54	p2	(GA)7	14	125153	125166	potentially variable
PHG9_Scaffold_161	55	p2	(AT)6	12	126132	126143	
PHG9_Scaffold_161	58	p2	(AT)6	12	128573	128584	
PHG9_Scaffold_161	59	p2	(AT)9	18	132000	132017	potentially variable
PHG9_Scaffold_161	65	p2	(TA)7	14	156101	156114	potentially variable
PHG9_Scaffold_161	70	p2	(AT)7	14	169677	169690	potentially variable
PHG9_Scaffold_161	75	p2	(CT)6	12	179242	179253	
PHG9_Scaffold_161	86	p2	(TA)6	12	221266	221277	
PHG9_Scaffold_161	92	p2	(CT)7	14	227494	227507	potentially variable

PHG9_Scaffold_161	98	p3	(TAT)5	15	242557	242571	potentially variable
PHG9_Scaffold_161	110	p6	(AGTGAG)5	30	299388	299417	Hypervariable
PHG9_Scaffold_161	116	p2	(TC)8	16	303146	303161	potentially variable
PHG9_Scaffold_161	117	p2	(TC)6	12	304016	304027	
PHG9_Scaffold_161	118	p3	(ATT)5	15	305749	305763	potentially variable
PHG9_Scaffold_161	119	p2	(AG)7	14	306940	306953	potentially variable
PHG9_Scaffold_161	128	p2	(TA)7	14	336293	336306	potentially variable
PHG9_Scaffold_161	142	p2	(AG)6	12	376901	376912	
PHG9_Scaffold_161	143	p2	(TA)6	12	377090	377101	
PHG9_Scaffold_162	4	p2	(TA)7	14	7715	7728	potentially variable
PHG9_Scaffold_162	5	p2	(AT)7	14	8030	8043	potentially variable
PHG9_Scaffold_162	7	p2	(TA)6	12	17913	17924	
PHG9_Scaffold_162	20	p6	(TGAAAT)6	36	47523	47558	Hypervariable
PHG9_Scaffold_162	25	p2	(AT)9	18	65203	65220	potentially variable
PHG9_Scaffold_162	26	p2	(AT)8	16	65889	65904	potentially variable
PHG9_Scaffold_162	35	p2	(AT)9	18	89122	89139	potentially variable
PHG9_Scaffold_162	39	p2	(TA)8	16	99176	99191	potentially variable
PHG9_Scaffold_163	6	p2	(AT)6	12	20430	20441	

PHG9_Scaffold_163	16	p3	(GAA)5	15	54833	54847	potentially variable
PHG9_Scaffold_164	14	p2	(TA)6	12	24238	24249	
PHG9_Scaffold_164	16	p3	(AAT)5	15	31919	31933	potentially variable
PHG9_Scaffold_164	18	p2	(AT)6	12	33657	33668	
PHG9_Scaffold_164	24	p2	(TC)6	12	51443	51454	
PHG9_Scaffold_165	11	p3	(ATT)5	15	53747	53761	potentially variable
PHG9_Scaffold_165	12	p3	(AAT)5	15	57769	57783	potentially variable
PHG9_Scaffold_165	20	p2	(TA)6	12	102708	102719	
PHG9_Scaffold_165	31	p2	(AT)6	12	134354	134365	
PHG9_Scaffold_165	34	p2	(GA)6	12	144840	144851	
PHG9_Scaffold_165	42	p3	(ATA)5	15	173391	173405	potentially variable
PHG9_Scaffold_166	8	p2	(TA)6	12	45390	45401	
PHG9_Scaffold_166	10	p3	(AAT)5	15	49385	49399	potentially variable
PHG9_Scaffold_166	12	p2	(CA)6	12	54410	54421	
PHG9_Scaffold_166	14	p3	(AGG)5	15	57208	57222	potentially variable
PHG9_Scaffold_166	16	p2	(TC)8	16	75461	75476	potentially variable
PHG9_Scaffold_166	18	p2	(AT)7	14	80659	80672	potentially variable
PHG9_Scaffold_166	23	p2	(CT)6	12	85689	85700	
PHG9_Scaffold_166	24	p3	(CAG)5	15	85830	85844	potentially variable

PHG9_Scaffold_166	40	p3	(TAA)5	15	142292	142306	potentially variable
PHG9_Scaffold_166	42	p3	(TAT)5	15	144389	144403	potentially variable
PHG9_Scaffold_166	46	p3	(TAA)5	15	167561	167575	potentially variable
PHG9_Scaffold_166	50	p2	(TC)8	16	179426	179441	potentially variable
PHG9_Scaffold_166	55	p2	(TA)8	16	194542	194557	potentially variable
PHG9_Scaffold_166	67	p2	(CT)6	12	211932	211943	
PHG9_Scaffold_166	71	p2	(AT)6	12	228229	228240	
PHG9_Scaffold_166	83	p2	(TA)7	14	263793	263806	potentially variable
PHG9_Scaffold_166	84	p2	(AG)7	14	264895	264908	potentially variable
PHG9_Scaffold_166	102	p2	(AT)6	12	311353	311364	
PHG9_Scaffold_166	110	p3	(GTA)6	18	323994	324011	potentially variable
PHG9_Scaffold_166	114	p2	(TA)7	14	333327	333340	potentially variable
PHG9_Scaffold_166	115	p3	(ATT)5	15	334006	334020	potentially variable
PHG9_Scaffold_167	2	p2	(TA)6	12	6399	6410	
PHG9_Scaffold_167	3	p3	(AAT)6	18	10648	10665	potentially variable
PHG9_Scaffold_167	7	p2	(AT)9	18	18280	18297	potentially variable
PHG9_Scaffold_167	10	p2	(TA)6	12	36755	36766	
PHG9_Scaffold_167	12	p2	(TA)8	16	40358	40373	potentially

							variable
PHG9_Scaffold_167	23	p3	(ATT)6	18	68317	68334	potentially variable
PHG9_Scaffold_167	36	p2	(CT)9	18	82770	82787	potentially variable
PHG9_Scaffold_167	42	p2	(AT)7	14	98787	98800	potentially variable
PHG9_Scaffold_168	3	p2	(AG)6	12	9083	9094	
PHG9_Scaffold_168	4	p2	(CT)7	14	10285	10298	potentially variable
PHG9_Scaffold_168	15	p2	(TA)9	18	55901	55918	potentially variable
PHG9_Scaffold_168	16	p2	(TA)6	12	60451	60462	
PHG9_Scaffold_168	17	p2	(AT)7	14	62591	62604	potentially variable
PHG9_Scaffold_168	25	p2	(AT)8	16	81865	81880	potentially variable
PHG9_Scaffold_168	40	p2	(AT)8	16	136444	136459	potentially variable
PHG9_Scaffold_168	46	p3	(TGC)6	18	154978	154995	potentially variable
PHG9_Scaffold_168	51	p3	(AGG)5	15	171890	171904	potentially variable
PHG9_Scaffold_168	52	p2	(TA)8	16	176398	176413	potentially variable
PHG9_Scaffold_168	55	p2	(AT)7	14	181913	181926	potentially variable
PHG9_Scaffold_169	3	p2	(TA)8	16	11324	11339	potentially variable
PHG9_Scaffold_169	7	p2	(AT)6	12	18436	18447	

PHG9_Scaffold_169	9	p2	(TC)6	12	21953	21964	
PHG9_Scaffold_169	11	p2	(AT)6	12	25511	25522	
PHG9_Scaffold_169	13	p2	(AT)6	12	30507	30518	
PHG9_Scaffold_169	22	p3	(AGA)5	15	62531	62545	potentially variable
PHG9_Scaffold_169	29	p2	(AT)6	12	89784	89795	
PHG9_Scaffold_169	30	p3	(AAT)5	15	95489	95503	potentially variable
PHG9_Scaffold_169	38	p2	(TA)6	12	125293	125304	
PHG9_Scaffold_169	47	p3	(CTC)5	15	150300	150314	potentially variable
PHG9_Scaffold_169	54	p2	(AT)7	14	162552	162565	potentially variable
PHG9_Scaffold_169	55	p2	(AT)6	12	166031	166042	
PHG9_Scaffold_170	3	p2	(TG)8	16	7330	7345	potentially variable
PHG9_Scaffold_170	6	p2	(TA)7	14	14441	14454	potentially variable
PHG9_Scaffold_170	13	p3	(TCT)5	15	27105	27119	potentially variable
PHG9_Scaffold_170	21	p2	(CT)6	12	47337	47348	
PHG9_Scaffold_170	29	p3	(ATC)6	18	59481	59498	potentially variable
PHG9_Scaffold_170	30	p3	(ATG)5	15	68473	68487	potentially variable
PHG9_Scaffold_170	35	p2	(AG)7	14	85121	85134	potentially variable
PHG9_Scaffold_171	22	p3	(CTT)5	15	104623	104637	potentially variable

PHG9_Scaffold_171	23	p2	(TA)7	14	104872	104885	potentially variable
PHG9_Scaffold_171	28	p2	(TA)7	14	120168	120181	potentially variable
PHG9_Scaffold_171	31	p2	(AC)6	12	128726	128737	
PHG9_Scaffold_171	33	p2	(AT)8	16	130602	130617	potentially variable
PHG9_Scaffold_171	35	p2	(TA)8	16	131477	131492	potentially variable
PHG9_Scaffold_171	41	p2	(CT)6	12	149028	149039	
PHG9_Scaffold_171	42	p2	(GT)7	14	150038	150051	potentially variable
PHG9_Scaffold_171	46	p3	(CAA)5	15	161027	161041	potentially variable
PHG9_Scaffold_171	49	p2	(TA)7	14	172325	172338	potentially variable
PHG9_Scaffold_171	52	p2	(TA)6	12	183647	183658	
PHG9_Scaffold_171	64	p2	(TA)7	14	217872	217885	potentially variable
PHG9_Scaffold_171	72	p2	(TA)7	14	257158	257171	potentially variable
PHG9_Scaffold_171	77	p2	(TA)6	12	261292	261303	
PHG9_Scaffold_171	82	p2	(TA)7	14	274255	274268	potentially variable
PHG9_Scaffold_171	83	p3	(CTT)5	15	274622	274636	potentially variable
PHG9_Scaffold_172	4	p3	(CAC)5	15	16428	16442	potentially variable
PHG9_Scaffold_172	13	p2	(AG)7	14	39715	39728	potentially variable

PHG9_Scaffold_172	15	p2	(TA)10	20	43911	43930	Hypervariable
PHG9_Scaffold_172	17	p2	(CA)9	18	45024	45041	potentially variable
PHG9_Scaffold_172	43	p2	(AT)6	12	143064	143075	
PHG9_Scaffold_172	55	p2	(GA)7	14	187185	187198	potentially variable
PHG9_Scaffold_172	58	p3	(TTC)5	15	195455	195469	potentially variable
PHG9_Scaffold_172	66	p2	(AT)7	14	214638	214651	potentially variable
PHG9_Scaffold_172	67	p3	(TTA)5	15	215879	215893	potentially variable
PHG9_Scaffold_172	71	p2	(GA)8	16	226447	226462	potentially variable
PHG9_Scaffold_172	77	p3	(ATA)5	15	244093	244107	potentially variable
PHG9_Scaffold_172	83	p3	(AAT)5	15	273125	273139	potentially variable
PHG9_Scaffold_172	84	p2	(AT)8	16	273426	273441	potentially variable
PHG9_Scaffold_172	90	p2	(CA)7	14	291182	291195	potentially variable
PHG9_Scaffold_172	93	p2	(AT)6	12	294861	294872	
PHG9_Scaffold_172	94	p3	(ATT)6	18	303238	303255	potentially variable
PHG9_Scaffold_172	99	p2	(TA)6	12	311387	311398	
PHG9_Scaffold_172	102	p2	(AT)8	16	315062	315077	potentially variable
PHG9_Scaffold_172	111	p3	(ATT)5	15	342063	342077	potentially variable

PHG9_Scaffold_172	115	p2	(AT)6	12	345446	345457	
PHG9_Scaffold_172	116	p2	(AT)6	12	345584	345595	
PHG9_Scaffold_172	121	p2	(TA)6	12	351396	351407	
PHG9_Scaffold_172	123	p2	(CA)6	12	356555	356566	
PHG9_Scaffold_172	124	p3	(GAA)5	15	357169	357183	potentially variable
PHG9_Scaffold_172	127	p2	(TA)8	16	358623	358638	potentially variable
PHG9_Scaffold_172	133	p2	(TA)8	16	373634	373649	potentially variable
PHG9_Scaffold_172	134	p2	(TA)6	12	377587	377598	
PHG9_Scaffold_172	151	p2	(TA)6	12	413764	413775	
PHG9_Scaffold_172	152	p2	(TA)8	16	414654	414669	potentially variable
PHG9_Scaffold_172	154	p3	(TTA)5	15	418769	418783	potentially variable
PHG9_Scaffold_172	156	p3	(GAG)5	15	425450	425464	potentially variable
PHG9_Scaffold_172	165	p2	(TC)6	12	449845	449856	
PHG9_Scaffold_172	173	p3	(TTC)6	18	470346	470363	potentially variable
PHG9_Scaffold_172	179	p2	(TA)9	18	481108	481125	potentially variable
PHG9_Scaffold_172	181	p2	(AT)8	16	493872	493887	potentially variable
PHG9_Scaffold_172	182	p3	(TTA)6	18	497202	497219	potentially variable
PHG9_Scaffold_172	189	p2	(AT)10	20	504448	504467	Hypervariable
PHG9_Scaffold_172	202	p3	(CCA)5	15	537163	537177	potentially

							variable
PHG9_Scaffold_172	203	p2	(TA)9	18	543966	543983	potentially variable
PHG9_Scaffold_172	204	p2	(AT)7	14	546954	546967	potentially variable
PHG9_Scaffold_172	208	p2	(TA)8	16	560435	560450	potentially variable
PHG9_Scaffold_172	210	p2	(AG)8	16	561367	561382	potentially variable
PHG9_Scaffold_172	221	p3	(TTC)5	15	603547	603561	potentially variable
PHG9_Scaffold_173	1	p2	(TC)6	12	307	318	
PHG9_Scaffold_173	3	p2	(GT)7	14	6891	6904	potentially variable
PHG9_Scaffold_173	7	p3	(AAT)5	15	17236	17250	potentially variable
PHG9_Scaffold_173	11	p3	(TAA)5	15	40357	40371	potentially variable
PHG9_Scaffold_174	4	p2	(TA)9	18	10725	10742	potentially variable
PHG9_Scaffold_174	5	p2	(TA)6	12	17686	17697	
PHG9_Scaffold_174	9	p3	(TAA)6	18	33980	33997	potentially variable
PHG9_Scaffold_174	12	p2	(GA)8	16	45592	45607	potentially variable
PHG9_Scaffold_174	13	p2	(TA)9	18	68250	68267	potentially variable
PHG9_Scaffold_174	15	p2	(TA)7	14	73307	73320	potentially variable
PHG9_Scaffold_175	4	p2	(AT)7	14	13131	13144	potentially

							variable
PHG9_Scaffold_175	7	p2	(AT)7	14	24326	24339	potentially variable
PHG9_Scaffold_175	8	p2	(TC)6	12	25281	25292	
PHG9_Scaffold_175	14	p3	(GTC)5	15	34656	34670	potentially variable
PHG9_Scaffold_175	21	p2	(TA)6	12	45876	45887	
PHG9_Scaffold_175	22	p3	(TCA)5	15	48030	48044	potentially variable
PHG9_Scaffold_175	43	p3	(TAA)6	18	132599	132616	potentially variable
PHG9_Scaffold_176	1	p2	(AT)9	18	3539	3556	potentially variable
PHG9_Scaffold_176	2	p2	(AT)7	14	7738	7751	potentially variable
PHG9_Scaffold_176	3	p2	(AT)6	12	9228	9239	
PHG9_Scaffold_176	12	p3	(TAA)5	15	39717	39731	potentially variable
PHG9_Scaffold_176	13	p2	(AT)8	16	40046	40061	potentially variable
PHG9_Scaffold_176	14	p3	(ATT)5	15	40182	40196	potentially variable
PHG9_Scaffold_176	19	p2	(AT)8	16	53769	53784	potentially variable
PHG9_Scaffold_176	27	p3	(AAT)5	15	77274	77288	potentially variable
PHG9_Scaffold_176	31	p2	(AT)6	12	90058	90069	
PHG9_Scaffold_176	34	p2	(AT)6	12	93242	93253	
PHG9_Scaffold_176	38	p2	(TA)8	16	95379	95394	potentially variable

PHG9_Scaffold_176	40	p2	(TA)9	18	97865	97882	potentially variable
PHG9_Scaffold_176	43	p2	(TA)6	12	116543	116554	
PHG9_Scaffold_176	48	p2	(AT)8	16	132505	132520	potentially variable
PHG9_Scaffold_176	49	p2	(AT)8	16	136703	136718	potentially variable
PHG9_Scaffold_176	54	p3	(AGA)5	15	151327	151341	potentially variable
PHG9_Scaffold_176	60	p2	(TA)6	12	186670	186681	
PHG9_Scaffold_176	75	p2	(TA)7	14	213540	213553	potentially variable
PHG9_Scaffold_177	6	p3	(ATC)5	15	15564	15578	potentially variable
PHG9_Scaffold_177	8	p2	(TA)8	16	19610	19625	potentially variable
PHG9_Scaffold_177	11	p2	(TA)6	12	21228	21239	
PHG9_Scaffold_177	22	p3	(TGG)5	15	43741	43755	potentially variable
PHG9_Scaffold_177	25	p2	(TA)8	16	48396	48411	potentially variable
PHG9_Scaffold_177	27	p2	(CT)6	12	50087	50098	
PHG9_Scaffold_177	28	p3	(TTC)7	21	53950	53970	Hypervariable
PHG9_Scaffold_177	31	p3	(TCT)5	15	64479	64493	potentially variable
PHG9_Scaffold_177	33	p2	(AT)7	14	71361	71374	potentially variable
PHG9_Scaffold_177	34	p3	(ATC)5	15	75960	75974	potentially variable
PHG9_Scaffold_177	55	p3	(AAT)6	18	137977	137994	potentially

							variable
PHG9_Scaffold_177	59	p3	(TAA)5	15	148342	148356	potentially variable
PHG9_Scaffold_178	5	p2	(TA)6	12	13000	13011	
PHG9_Scaffold_178	12	p3	(TTC)6	18	34606	34623	potentially variable
PHG9_Scaffold_178	22	p2	(CT)6	12	55107	55118	
PHG9_Scaffold_178	23	p2	(TA)9	18	61669	61686	potentially variable
PHG9_Scaffold_178	24	p2	(AT)9	18	61849	61866	potentially variable
PHG9_Scaffold_178	26	p3	(ATA)5	15	70585	70599	potentially variable
PHG9_Scaffold_178	27	p2	(TA)9	18	72052	72069	potentially variable
PHG9_Scaffold_178	28	p2	(AT)7	14	72601	72614	potentially variable
PHG9_Scaffold_178	31	p2	(TA)8	16	78895	78910	potentially variable
PHG9_Scaffold_178	33	p3	(ACC)5	15	89593	89607	potentially variable
PHG9_Scaffold_178	37	p2	(AT)6	12	99393	99404	
PHG9_Scaffold_178	38	p3	(ATA)5	15	106949	106963	potentially variable
PHG9_Scaffold_178	40	p2	(AT)6	12	110244	110255	
PHG9_Scaffold_178	41	p2	(AT)9	18	110557	110574	potentially variable
PHG9_Scaffold_179	1	p2	(TC)7	14	6844	6857	potentially variable
PHG9_Scaffold_179	5	p2	(TA)6	12	16140	16151	

PHG9_Scaffold_179	7	p2	(AT)8	16	28289	28304	potentially variable
PHG9_Scaffold_179	17	p2	(TA)9	18	48393	48410	potentially variable
PHG9_Scaffold_179	18	p2	(TC)6	12	49121	49132	
PHG9_Scaffold_179	21	p2	(AT)6	12	61578	61589	
PHG9_Scaffold_179	32	p3	(AGA)5	15	126537	126551	potentially variable
PHG9_Scaffold_179	33	p2	(AT)8	16	132777	132792	potentially variable
PHG9_Scaffold_179	37	p3	(GGT)5	15	146782	146796	potentially variable
PHG9_Scaffold_179	42	p2	(AG)7	14	160341	160354	potentially variable
PHG9_Scaffold_180	7	p3	(TTA)5	15	17235	17249	potentially variable
PHG9_Scaffold_180	10	p2	(TA)6	12	19280	19291	
PHG9_Scaffold_180	12	p2	(AT)6	12	20957	20968	
PHG9_Scaffold_180	14	p2	(CA)6	12	38017	38028	
PHG9_Scaffold_180	16	p2	(TC)6	12	53879	53890	
PHG9_Scaffold_180	19	p3	(ATA)5	15	61316	61330	potentially variable
PHG9_Scaffold_180	26	p2	(TA)6	12	82622	82633	
PHG9_Scaffold_180	43	p2	(CT)7	14	123968	123981	potentially variable
PHG9_Scaffold_180	45	p2	(AT)6	12	127320	127331	
PHG9_Scaffold_181	12	p3	(CTA)6	18	28811	28828	potentially variable
PHG9_Scaffold_181	16	p2	(TA)7	14	43722	43735	potentially

							variable
PHG9_Scaffold_181	19	p2	(AT)6	12	48022	48033	
PHG9_Scaffold_181	22	p3	(ATT)5	15	56635	56649	potentially variable
PHG9_Scaffold_181	29	p2	(TA)7	14	77828	77841	potentially variable
PHG9_Scaffold_181	32	p3	(AAT)5	15	86829	86843	potentially variable
PHG9_Scaffold_181	33	p2	(AT)6	12	93411	93422	
PHG9_Scaffold_181	34	p2	(TC)8	16	97788	97803	potentially variable
PHG9_Scaffold_181	35	p6	(TGAGAG)6	36	98823	98858	Hypervariable
PHG9_Scaffold_181	42	p3	(TAC)6	18	109111	109128	potentially variable
PHG9_Scaffold_181	52	p2	(TA)7	14	132057	132070	potentially variable
PHG9_Scaffold_181	54	p2	(TC)6	12	133432	133443	
PHG9_Scaffold_181	55	p3	(ATT)6	18	136193	136210	potentially variable
PHG9_Scaffold_181	59	p3	(TAT)6	18	144609	144626	potentially variable
PHG9_Scaffold_181	60	p3	(GGC)5	15	145114	145128	potentially variable
PHG9_Scaffold_181	64	p2	(AG)9	18	158513	158530	potentially variable
PHG9_Scaffold_181	66	p2	(AT)7	14	164843	164856	potentially variable
PHG9_Scaffold_181	70	p3	(AGA)5	15	175107	175121	potentially variable
PHG9_Scaffold_181	77	p2	(TA)6	12	181232	181243	

PHG9_Scaffold_181	82	p2	(TA)6	12	217765	217776	
PHG9_Scaffold_181	85	p2	(TC)7	14	219744	219757	potentially variable
PHG9_Scaffold_181	86	p2	(TA)6	12	219977	219988	
PHG9_Scaffold_182	3	p3	(TTC)5	15	8191	8205	potentially variable
PHG9_Scaffold_182	6	p2	(TA)6	12	11423	11434	
PHG9_Scaffold_182	7	p2	(TC)7	14	12232	12245	potentially variable
PHG9_Scaffold_182	8	p2	(AG)6	12	12383	12394	
PHG9_Scaffold_183	4	p3	(CTT)5	15	7737	7751	potentially variable
PHG9_Scaffold_183	6	p2	(TA)9	18	8436	8453	potentially variable
PHG9_Scaffold_183	8	p3	(AAT)5	15	22234	22248	potentially variable
PHG9_Scaffold_183	13	p2	(AT)6	12	32182	32193	
PHG9_Scaffold_183	16	p3	(GGA)5	15	38448	38462	potentially variable
PHG9_Scaffold_183	18	p2	(TA)8	16	42163	42178	potentially variable
PHG9_Scaffold_183	22	p2	(AT)8	16	49048	49063	potentially variable
PHG9_Scaffold_183	29	p2	(GT)6	12	70654	70665	
PHG9_Scaffold_183	35	p3	(TAT)6	18	88852	88869	potentially variable
PHG9_Scaffold_183	41	p2	(TA)7	14	101544	101557	potentially variable
PHG9_Scaffold_183	42	p3	(TAT)5	15	106469	106483	potentially variable

PHG9_Scaffold_183	44	p2	(AT)7	14	126336	126349	potentially variable
PHG9_Scaffold_183	52	p3	(CGT)5	15	150380	150394	potentially variable
PHG9_Scaffold_183	54	p2	(TA)6	12	151697	151708	
PHG9_Scaffold_184	6	p3	(CTT)5	15	20571	20585	potentially variable
PHG9_Scaffold_184	20	p3	(TTC)5	15	118884	118898	potentially variable
PHG9_Scaffold_184	23	p2	(AT)7	14	141735	141748	potentially variable
PHG9_Scaffold_184	24	p3	(AAT)5	15	149005	149019	potentially variable
PHG9_Scaffold_184	30	p2	(CT)9	18	203305	203322	potentially variable
PHG9_Scaffold_184	32	p2	(TA)6	12	228992	229003	
PHG9_Scaffold_184	35	p3	(TTA)5	15	245435	245449	potentially variable
PHG9_Scaffold_184	41	p2	(AT)9	18	292123	292140	potentially variable
PHG9_Scaffold_184	42	p3	(AAT)5	15	292482	292496	potentially variable
PHG9_Scaffold_184	43	p2	(AT)9	18	319667	319684	potentially variable
PHG9_Scaffold_184	47	p2	(TA)7	14	340760	340773	potentially variable
PHG9_Scaffold_184	57	p2	(AT)6	12	389226	389237	
PHG9_Scaffold_184	58	p2	(AT)6	12	391575	391586	
PHG9_Scaffold_184	63	p2	(AT)7	14	423395	423408	potentially variable

PHG9_Scaffold_184	64	p2	(CT)7	14	424008	424021	potentially variable
PHG9_Scaffold_189	3	p2	(CT)6	12	3370	3381	
PHG9_Scaffold_191	1	p2	(TA)6	12	9061	9072	
PHG9_Scaffold_191	4	p2	(TA)6	12	23308	23319	
PHG9_Scaffold_191	22	p3	(CTT)5	15	133031	133045	potentially variable
PHG9_Scaffold_191	24	p2	(TA)7	14	138653	138666	potentially variable
PHG9_Scaffold_194	1	p2	(AT)7	14	12452	12465	potentially variable
PHG9_Scaffold_194	2	p3	(ATC)5	15	16181	16195	potentially variable
PHG9_Scaffold_194	3	p3	(AAT)6	18	18160	18177	potentially variable
PHG9_Scaffold_195	2	p2	(AT)7	14	8031	8044	potentially variable
PHG9_Scaffold_195	3	p2	(AT)9	18	16541	16558	potentially variable
PHG9_Scaffold_196	9	p2	(AT)8	16	106475	106490	potentially variable
PHG9_Scaffold_196	17	p2	(TA)7	14	219814	219827	potentially variable
PHG9_Scaffold_196	26	p2	(TA)6	12	459655	459666	
PHG9_Scaffold_197	4	p2	(TA)7	14	27289	27302	potentially variable
PHG9_Scaffold_197	24	p2	(AT)6	12	156589	156600	
PHG9_Scaffold_197	28	p3	(TTA)5	15	190972	190986	potentially variable
PHG9_Scaffold_197	29	p2	(AT)6	12	208509	208520	

PHG9_Scaffold_198	4	p2	(AT)6	12	18535	18546	
PHG9_Scaffold_198	8	p2	(TA)8	16	112384	112399	potentially variable
PHG9_Scaffold_198	15	p2	(AT)9	18	124086	124103	potentially variable
PHG9_Scaffold_200	2	p2	(TA)7	14	14595	14608	potentially variable
PHG9_Scaffold_200	4	p2	(TC)6	12	27226	27237	
PHG9_Scaffold_200	6	p3	(AAG)6	18	29894	29911	potentially variable
PHG9_Scaffold_200	10	p2	(TA)8	16	53134	53149	potentially variable
PHG9_Scaffold_201	7	p3	(TTC)5	15	61048	61062	potentially variable
PHG9_Scaffold_201	8	p2	(TA)6	12	68002	68013	
PHG9_Scaffold_201	10	p2	(AT)7	14	73269	73282	potentially variable
PHG9_Scaffold_201	13	p2	(AT)6	12	74682	74693	
PHG9_Scaffold_201	21	p2	(TC)7	14	114541	114554	potentially variable
PHG9_Scaffold_203	3	p2	(AT)6	12	23510	23521	
PHG9_Scaffold_203	7	p2	(AT)9	18	39049	39066	potentially variable
PHG9_Scaffold_205	8	p2	(TA)6	12	41073	41084	
PHG9_Scaffold_206	3	p2	(TA)8	16	30633	30648	potentially variable
PHG9_Scaffold_206	6	p2	(AG)8	16	82202	82217	potentially variable
PHG9_Scaffold_207	2	p3	(GGT)5	15	24303	24317	potentially variable

PHG9_Scaffold_207	3	p2	(TA)7	14	25665	25678	potentially variable
PHG9_Scaffold_207	12	p2	(AT)9	18	65938	65955	potentially variable
PHG9_Scaffold_207	16	p3	(TAT)5	15	80424	80438	potentially variable
PHG9_Scaffold_209	1	p2	(AT)8	16	47196	47211	potentially variable
PHG9_Scaffold_210	1	p2	(TA)6	12	866	877	
PHG9_Scaffold_210	3	p2	(CT)6	12	5096	5107	
PHG9_Scaffold_210	12	p3	(AAC)5	15	18012	18026	potentially variable
PHG9_Scaffold_210	18	p2	(GT)6	12	27168	27179	
PHG9_Scaffold_210	25	p2	(AC)8	16	52725	52740	potentially variable
PHG9_Scaffold_210	28	p2	(AT)7	14	61481	61494	potentially variable
PHG9_Scaffold_210	32	p2	(CT)6	12	71150	71161	
PHG9_Scaffold_210	38	p2	(AT)6	12	88776	88787	
PHG9_Scaffold_210	42	p2	(AG)6	12	98224	98235	
PHG9_Scaffold_210	59	p2	(AT)8	16	138834	138849	potentially variable
PHG9_Scaffold_210	60	p2	(TC)7	14	141362	141375	potentially variable
PHG9_Scaffold_210	62	p2	(TA)6	12	142256	142267	
PHG9_Scaffold_210	64	p3	(AAT)5	15	143856	143870	potentially variable
PHG9_Scaffold_210	69	p3	(AAT)5	15	159030	159044	potentially variable

PHG9_Scaffold_210	70	p3	(ATA)5	15	159336	159350	potentially variable
PHG9_Scaffold_212	1	p3	(TTC)5	15	11905	11919	potentially variable
PHG9_Scaffold_217	2	p3	(ATT)5	15	766	780	potentially variable
PHG9_Scaffold_217	13	p3	(CAT)5	15	61776	61790	potentially variable
PHG9_Scaffold_217	36	p3	(ATA)5	15	152982	152996	potentially variable
PHG9_Scaffold_217	40	p3	(TAA)6	18	164666	164683	potentially variable
PHG9_Scaffold_217	48	p3	(AAT)6	18	187221	187238	potentially variable
PHG9_Scaffold_219	4	p2	(TA)6	12	50252	50263	
PHG9_Scaffold_219	8	p3	(TAA)5	15	70368	70382	potentially variable
PHG9_Scaffold_219	11	p2	(TC)6	12	103609	103620	
PHG9_Scaffold_219	12	p2	(AT)8	16	109902	109917	potentially variable
PHG9_Scaffold_219	22	p2	(AT)7	14	206826	206839	potentially variable
PHG9_Scaffold_219	25	p2	(AT)9	18	214696	214713	potentially variable
PHG9_Scaffold_220	1	p3	(TGA)5	15	801	815	potentially variable
PHG9_Scaffold_220	12	p2	(CT)6	12	23271	23282	
PHG9_Scaffold_220	20	p3	(TTA)5	15	58354	58368	potentially variable
PHG9_Scaffold_220	26	p3	(AGT)5	15	72033	72047	potentially

							variable
PHG9_Scaffold_220	27	p2	(TG)6	12	74321	74332	
PHG9_Scaffold_220	28	p2	(AC)7	14	77327	77340	potentially variable
PHG9_Scaffold_220	29	p3	(TCT)5	15	79308	79322	potentially variable
PHG9_Scaffold_220	31	p3	(ATT)6	18	84379	84396	potentially variable
PHG9_Scaffold_220	36	p2	(CT)6	12	92430	92441	
PHG9_Scaffold_220	48	p6	(CCAAAT)5	30	127252	127281	Hypervariable
PHG9_Scaffold_220	49	p3	(AGA)6	18	131466	131483	potentially variable
PHG9_Scaffold_220	58	p3	(CTT)5	15	167312	167326	potentially variable
PHG9_Scaffold_220	66	p3	(TAA)5	15	198139	198153	potentially variable
PHG9_Scaffold_220	68	p3	(AAT)5	15	203278	203292	potentially variable
PHG9_Scaffold_220	70	p2	(TA)9	18	207587	207604	potentially variable
PHG9_Scaffold_220	78	p2	(GT)7	14	225527	225540	potentially variable
PHG9_Scaffold_220	83	p2	(GC)6	12	258277	258288	
PHG9_Scaffold_226	5	p2	(TA)8	16	13178	13193	potentially variable
PHG9_Scaffold_226	12	p3	(TGA)6	18	55933	55950	potentially variable
PHG9_Scaffold_226	14	p2	(AT)8	16	93999	94014	potentially variable
PHG9_Scaffold_226	17	p2	(CA)6	12	97481	97492	

PHG9_Scaffold_226	32	p2	(AC)7	14	205368	205381	potentially variable
PHG9_Scaffold_226	35	p2	(AG)6	12	220520	220531	
PHG9_Scaffold_226	38	p2	(AG)8	16	228258	228273	potentially variable
PHG9_Scaffold_227	1	p3	(CTT)6	18	1863	1880	potentially variable
PHG9_Scaffold_227	2	p4	(TATC)5	20	3147	3166	Hypervariable
PHG9_Scaffold_227	7	p3	(TGC)5	15	36003	36017	potentially variable
PHG9_Scaffold_227	17	p3	(AAT)5	15	73185	73199	potentially variable
PHG9_Scaffold_227	26	p2	(TA)6	12	99108	99119	
PHG9_Scaffold_227	30	p2	(TA)6	12	105644	105655	
PHG9_Scaffold_227	31	p2	(TA)8	16	106779	106794	potentially variable
PHG9_Scaffold_227	32	p2	(AC)6	12	113840	113851	
PHG9_Scaffold_228	13	p2	(AC)6	12	138225	138236	
PHG9_Scaffold_229	2	p2	(TA)6	12	4935	4946	
PHG9_Scaffold_232	1	p3	(AGA)5	15	3845	3859	potentially variable
PHG9_Scaffold_232	20	p2	(CA)8	16	65351	65366	potentially variable
PHG9_Scaffold_232	22	p2	(AT)7	14	72538	72551	potentially variable
PHG9_Scaffold_232	26	p2	(AT)7	14	89291	89304	potentially variable
PHG9_Scaffold_232	29	p2	(CT)6	12	93560	93571	
PHG9_Scaffold_232	30	p3	(CCT)5	15	93678	93692	potentially

							variable
PHG9_Scaffold_232	45	p2	(AT)7	14	121528	121541	potentially variable
PHG9_Scaffold_232	47	p6	(AGTCTC)6	36	124573	124608	Hypervariable
PHG9_Scaffold_232	53	p2	(AT)8	16	151371	151386	potentially variable
PHG9_Scaffold_234	7	p2	(AT)8	16	25732	25747	potentially variable
PHG9_Scaffold_234	10	p2	(AT)7	14	41192	41205	potentially variable
PHG9_Scaffold_237	2	p2	(TA)7	14	2870	2883	potentially variable
PHG9_Scaffold_237	4	p3	(GAT)5	15	11322	11336	potentially variable
PHG9_Scaffold_237	17	p2	(AT)9	18	49954	49971	potentially variable
PHG9_Scaffold_237	23	p2	(AT)6	12	65955	65966	
PHG9_Scaffold_237	28	p2	(TA)7	14	74694	74707	potentially variable
PHG9_Scaffold_237	31	p2	(TA)7	14	87556	87569	potentially variable
PHG9_Scaffold_237	38	p2	(AT)8	16	109746	109761	potentially variable
PHG9_Scaffold_239	2	p2	(TA)6	12	34052	34063	
PHG9_Scaffold_239	8	p2	(AT)6	12	92959	92970	
PHG9_Scaffold_239	10	p2	(TA)8	16	95451	95466	potentially variable
PHG9_Scaffold_239	17	p2	(AT)9	18	140227	140244	potentially variable
PHG9_Scaffold_240	4	p2	(AT)8	16	24072	24087	potentially

							variable
PHG9_Scaffold_240	13	p2	(TC)7	14	46109	46122	potentially variable
PHG9_Scaffold_240	15	p3	(TTG)5	15	57929	57943	potentially variable
PHG9_Scaffold_240	17	p2	(TA)7	14	61577	61590	potentially variable
PHG9_Scaffold_240	19	p2	(TA)7	14	66185	66198	potentially variable
PHG9_Scaffold_240	24	p2	(TC)9	18	74908	74925	potentially variable
PHG9_Scaffold_240	27	p2	(AT)6	12	79990	80001	
PHG9_Scaffold_240	28	p2	(TA)8	16	82020	82035	potentially variable
PHG9_Scaffold_240	31	p3	(ATT)5	15	84809	84823	potentially variable
PHG9_Scaffold_240	39	p3	(AAT)5	15	98948	98962	potentially variable
PHG9_Scaffold_240	48	p2	(AT)6	12	120196	120207	
PHG9_Scaffold_240	49	p2	(TA)6	12	125799	125810	
PHG9_Scaffold_241	1	p2	(TG)9	18	12127	12144	potentially variable
PHG9_Scaffold_241	4	p2	(TA)7	14	16270	16283	potentially variable
PHG9_Scaffold_241	6	p2	(TG)7	14	18155	18168	potentially variable
PHG9_Scaffold_241	24	p2	(TA)8	16	62067	62082	potentially variable
PHG9_Scaffold_241	28	p2	(AT)9	18	70086	70103	potentially variable

PHG9_Scaffold_241	30	p2	(AT)6	12	77436	77447	
PHG9_Scaffold_241	41	p2	(CA)6	12	114303	114314	
PHG9_Scaffold_241	51	p2	(CA)8	16	150082	150097	potentially variable
PHG9_Scaffold_241	61	p2	(TA)6	12	202475	202486	
PHG9_Scaffold_241	62	p3	(TTA)5	15	205358	205372	potentially variable
PHG9_Scaffold_241	66	p2	(AT)8	16	239798	239813	potentially variable
PHG9_Scaffold_241	70	p2	(TA)6	12	261702	261713	
PHG9_Scaffold_242	4	p3	(GTT)5	15	18982	18996	potentially variable
PHG9_Scaffold_242	9	p2	(AT)7	14	28706	28719	potentially variable
PHG9_Scaffold_242	19	p3	(GAA)5	15	63580	63594	potentially variable
PHG9_Scaffold_242	20	p3	(TTA)5	15	69247	69261	potentially variable
PHG9_Scaffold_242	21	p3	(AAG)5	15	73670	73684	potentially variable
PHG9_Scaffold_242	22	p3	(CTT)6	18	74177	74194	potentially variable
PHG9_Scaffold_242	24	p2	(TA)8	16	76465	76480	potentially variable
PHG9_Scaffold_242	25	p6	(CAAATG)5	30	82284	82313	Hypervariable
PHG9_Scaffold_242	31	p3	(ATT)5	15	109295	109309	potentially variable
PHG9_Scaffold_242	32	p2	(TA)9	18	111545	111562	potentially variable
PHG9_Scaffold_242	33	p2	(TA)9	18	117797	117814	potentially

							variable
PHG9_Scaffold_242	40	p2	(AT)6	12	129841	129852	
PHG9_Scaffold_244	5	p3	(TTC)5	15	10503	10517	potentially variable
PHG9_Scaffold_244	11	p2	(AT)7	14	30224	30237	potentially variable
PHG9_Scaffold_244	17	p3	(ATC)5	15	40936	40950	potentially variable
PHG9_Scaffold_244	18	p2	(AT)7	14	48076	48089	potentially variable
PHG9_Scaffold_244	23	p2	(TA)9	18	70781	70798	potentially variable
PHG9_Scaffold_244	30	p3	(TCT)6	18	89629	89646	potentially variable
PHG9_Scaffold_244	35	p2	(AT)7	14	97935	97948	potentially variable
PHG9_Scaffold_244	38	p2	(AT)6	12	102331	102342	
PHG9_Scaffold_244	43	p2	(AT)8	16	125853	125868	potentially variable
PHG9_Scaffold_245	7	p3	(AAT)5	15	20096	20110	potentially variable
PHG9_Scaffold_245	8	p2	(AT)8	16	23674	23689	potentially variable
PHG9_Scaffold_246	5	p2	(AG)6	12	63050	63061	
PHG9_Scaffold_247	2	p2	(AT)7	14	1674	1687	potentially variable
PHG9_Scaffold_247	4	p2	(TA)9	18	8700	8717	potentially variable
PHG9_Scaffold_250	5	p2	(TA)6	12	37293	37304	
PHG9_Scaffold_250	9	p2	(TA)7	14	56604	56617	potentially

							variable
PHG9_Scaffold_250	15	p2	(TG)6	12	73653	73664	
PHG9_Scaffold_250	20	p3	(GAT)5	15	90478	90492	potentially variable
PHG9_Scaffold_251	1	p2	(TA)8	16	7798	7813	potentially variable
PHG9_Scaffold_252	10	p2	(AT)7	14	32474	32487	potentially variable
PHG9_Scaffold_252	19	p2	(AG)6	12	52251	52262	
PHG9_Scaffold_252	23	p2	(TA)6	12	65438	65449	
PHG9_Scaffold_252	24	p2	(TC)7	14	67617	67630	potentially variable
PHG9_Scaffold_252	29	p2	(AT)6	12	89955	89966	
PHG9_Scaffold_252	38	p2	(CA)8	16	108603	108618	potentially variable
PHG9_Scaffold_253	5	p2	(TA)9	18	13780	13797	potentially variable
PHG9_Scaffold_253	9	p2	(AT)7	14	33122	33135	potentially variable
PHG9_Scaffold_253	10	p2	(AT)6	12	36153	36164	
PHG9_Scaffold_254	4	p2	(TA)7	14	19939	19952	potentially variable
PHG9_Scaffold_255	1	p2	(AT)6	12	1604	1615	
PHG9_Scaffold_257	9	p2	(GA)6	12	40004	40015	
PHG9_Scaffold_257	10	p2	(TC)7	14	42041	42054	potentially variable
PHG9_Scaffold_257	13	p3	(TTG)5	15	47681	47695	potentially variable
PHG9_Scaffold_257	14	p2	(TA)7	14	50316	50329	potentially

							variable
PHG9_Scaffold_257	16	p2	(GT)6	12	52903	52914	
PHG9_Scaffold_257	31	p2	(TA)6	12	98768	98779	
PHG9_Scaffold_257	34	p2	(TA)6	12	103828	103839	
PHG9_Scaffold_257	42	p3	(AAT)5	15	128004	128018	potentially variable
PHG9_Scaffold_257	55	p2	(TC)7	14	181357	181370	potentially variable
PHG9_Scaffold_257	56	p2	(GT)6	12	184622	184633	
PHG9_Scaffold_257	60	p2	(GA)6	12	203853	203864	
PHG9_Scaffold_257	63	p2	(AG)6	12	209576	209587	
PHG9_Scaffold_257	69	p2	(TA)6	12	226753	226764	
PHG9_Scaffold_257	72	p2	(AT)9	18	230756	230773	potentially variable
PHG9_Scaffold_259	4	p2	(GT)6	12	21719	21730	
PHG9_Scaffold_259	12	p2	(AT)8	16	54252	54267	potentially variable
PHG9_Scaffold_259	32	p2	(AT)9	18	113798	113815	potentially variable
PHG9_Scaffold_259	34	p2	(AT)6	12	121174	121185	
PHG9_Scaffold_259	35	p2	(AT)7	14	121518	121531	potentially variable
PHG9_Scaffold_263	2	p3	(TAT)5	15	4123	4137	potentially variable
PHG9_Scaffold_263	17	p2	(AT)6	12	63555	63566	
PHG9_Scaffold_264	2	p3	(AGA)6	18	30914	30931	potentially variable
PHG9_Scaffold_264	3	p2	(TA)7	14	48394	48407	potentially variable

PHG9_Scaffold_268	3	p2	(TA)9	18	30807	30824	potentially variable
PHG9_Scaffold_269	1	p2	(TA)7	14	150	163	potentially variable
PHG9_Scaffold_269	4	p2	(AT)7	14	7305	7318	potentially variable
PHG9_Scaffold_269	11	p3	(AAT)5	15	25056	25070	potentially variable
PHG9_Scaffold_269	20	p2	(AT)9	18	54590	54607	potentially variable
PHG9_Scaffold_269	28	p3	(TTA)5	15	91792	91806	potentially variable
PHG9_Scaffold_269	35	p3	(TAA)5	15	126612	126626	potentially variable
PHG9_Scaffold_269	37	p3	(ATA)6	18	131617	131634	potentially variable
PHG9_Scaffold_270	4	p3	(ATA)5	15	17902	17916	potentially variable
PHG9_Scaffold_270	17	p2	(AT)6	12	70318	70329	
PHG9_Scaffold_270	18	p2	(AT)6	12	77001	77012	
PHG9_Scaffold_270	24	p3	(CAT)6	18	106130	106147	potentially variable
PHG9_Scaffold_270	28	p2	(AT)6	12	117136	117147	
PHG9_Scaffold_272	2	p2	(AT)8	16	11125	11140	potentially variable
PHG9_Scaffold_273	6	p2	(CT)6	12	18770	18781	
PHG9_Scaffold_273	8	p2	(TA)6	12	27501	27512	
PHG9_Scaffold_273	9	p2	(TA)7	14	31352	31365	potentially variable
PHG9_Scaffold_273	24	p2	(TA)7	14	76517	76530	potentially

							variable
PHG9_Scaffold_273	29	p2	(AT)6	12	98494	98505	
PHG9_Scaffold_273	34	p2	(TA)7	14	111451	111464	potentially variable
PHG9_Scaffold_273	39	p2	(AT)8	16	121436	121451	potentially variable
PHG9_Scaffold_276	4	p2	(AT)6	12	10957	10968	
PHG9_Scaffold_276	7	p2	(AT)7	14	47779	47792	potentially variable
PHG9_Scaffold_277	9	p2	(TC)9	18	48210	48227	potentially variable
PHG9_Scaffold_277	12	p2	(TA)8	16	57051	57066	potentially variable
PHG9_Scaffold_277	19	p2	(TC)6	12	72118	72129	
PHG9_Scaffold_277	25	p2	(AG)6	12	90587	90598	
PHG9_Scaffold_278	5	p3	(ACC)5	15	10423	10437	potentially variable
PHG9_Scaffold_278	8	p2	(AT)6	12	20629	20640	
PHG9_Scaffold_278	13	p2	(TA)6	12	35835	35846	
PHG9_Scaffold_278	16	p3	(ATG)5	15	64224	64238	potentially variable
PHG9_Scaffold_281	10	p2	(TA)6	12	47769	47780	
PHG9_Scaffold_281	12	p3	(GAG)5	15	62724	62738	potentially variable
PHG9_Scaffold_282	5	p2	(TA)8	16	15791	15806	potentially variable
PHG9_Scaffold_282	22	p2	(AT)8	16	54400	54415	potentially variable
PHG9_Scaffold_282	36	p2	(AT)6	12	86355	86366	

PHG9_Scaffold_282	42	p2	(TA)6	12	106073	106084	
PHG9_Scaffold_282	43	p2	(AT)6	12	108980	108991	
PHG9_Scaffold_282	45	p2	(AT)6	12	110938	110949	
PHG9_Scaffold_283	1	p2	(AT)9	18	9452	9469	potentially variable
PHG9_Scaffold_285	2	p2	(TA)7	14	5463	5476	potentially variable
PHG9_Scaffold_285	13	p2	(AC)7	14	24500	24513	potentially variable
PHG9_Scaffold_285	16	p2	(AG)6	12	25664	25675	
PHG9_Scaffold_285	20	p3	(CCA)5	15	32825	32839	potentially variable
PHG9_Scaffold_285	28	p2	(AT)10	20	50051	50070	Hypervariable
PHG9_Scaffold_285	30	p6	(TTCTAT)5	30	52965	52994	Hypervariable
PHG9_Scaffold_285	32	p2	(GA)7	14	58140	58153	potentially variable
PHG9_Scaffold_285	36	p3	(ATT)5	15	79873	79887	potentially variable
PHG9_Scaffold_285	39	p2	(CT)9	18	97960	97977	potentially variable
PHG9_Scaffold_285	44	p2	(AT)8	16	107695	107710	potentially variable
PHG9_Scaffold_285	45	p2	(TA)7	14	107976	107989	potentially variable
PHG9_Scaffold_285	47	p3	(CAT)5	15	112329	112343	potentially variable
PHG9_Scaffold_285	54	p3	(TAA)5	15	122004	122018	potentially variable
PHG9_Scaffold_286	3	p2	(AG)9	18	9552	9569	potentially variable

PHG9_Scaffold_286	7	p2	(TA)7	14	35039	35052	potentially variable
PHG9_Scaffold_286	9	p2	(AT)8	16	55707	55722	potentially variable
PHG9_Scaffold_286	10	p2	(AT)6	12	86947	86958	
PHG9_Scaffold_287	2	p2	(AT)7	14	12239	12252	potentially variable
PHG9_Scaffold_287	13	p2	(AT)6	12	31133	31144	
PHG9_Scaffold_287	20	p2	(CT)8	16	50215	50230	potentially variable
PHG9_Scaffold_287	40	p2	(TG)6	12	105884	105895	
PHG9_Scaffold_287	44	p2	(AT)9	18	121159	121176	potentially variable
PHG9_Scaffold_287	58	p3	(TCT)5	15	149779	149793	potentially variable
PHG9_Scaffold_287	59	p2	(CT)6	12	153191	153202	
PHG9_Scaffold_287	60	p3	(AAT)5	15	162557	162571	potentially variable
PHG9_Scaffold_287	64	p2	(CT)8	16	182521	182536	potentially variable
PHG9_Scaffold_287	65	p3	(TGT)5	15	182787	182801	potentially variable
PHG9_Scaffold_287	66	p2	(TA)6	12	185227	185238	
PHG9_Scaffold_287	70	p2	(CA)8	16	198499	198514	potentially variable
PHG9_Scaffold_287	71	p6	(AGAAAG)5	30	199008	199037	Hypervariable
PHG9_Scaffold_287	76	p3	(ACA)6	18	220690	220707	potentially variable
PHG9_Scaffold_287	82	p2	(AT)6	12	229100	229111	

PHG9_Scaffold_287	85	p3	(ATT)5	15	233950	233964	potentially variable
PHG9_Scaffold_288	6	p2	(AT)7	14	17808	17821	potentially variable
PHG9_Scaffold_288	9	p3	(AAT)5	15	22021	22035	potentially variable
PHG9_Scaffold_288	12	p2	(CT)8	16	27294	27309	potentially variable
PHG9_Scaffold_288	17	p2	(AT)6	12	40557	40568	
PHG9_Scaffold_288	25	p3	(GAG)5	15	68051	68065	potentially variable
PHG9_Scaffold_288	37	p2	(TA)7	14	124926	124939	potentially variable
PHG9_Scaffold_288	40	p3	(GTG)6	18	142030	142047	potentially variable
PHG9_Scaffold_288	41	p2	(AT)6	12	143479	143490	
PHG9_Scaffold_288	43	p2	(AT)9	18	148272	148289	potentially variable
PHG9_Scaffold_288	50	p2	(AT)6	12	200554	200565	
PHG9_Scaffold_288	55	p2	(AT)10	20	209928	209947	Hypervariable
PHG9_Scaffold_288	57	p2	(AT)10	20	227026	227045	Hypervariable
PHG9_Scaffold_288	59	p3	(AGA)5	15	233363	233377	potentially variable
PHG9_Scaffold_289	10	p2	(AG)7	14	35508	35521	potentially variable
PHG9_Scaffold_293	14	p2	(TA)6	12	28763	28774	
PHG9_Scaffold_293	21	p3	(ATA)6	18	46850	46867	potentially variable
PHG9_Scaffold_293	28	p3	(GGT)5	15	59354	59368	potentially variable

PHG9_Scaffold_293	31	p3	(AAT)5	15	68006	68020	potentially variable
PHG9_Scaffold_293	32	p2	(CT)8	16	68501	68516	potentially variable
PHG9_Scaffold_293	46	p2	(TA)7	14	99644	99657	potentially variable
PHG9_Scaffold_293	51	p2	(TC)6	12	108918	108929	
PHG9_Scaffold_294	3	p3	(ACC)5	15	5806	5820	potentially variable
PHG9_Scaffold_294	15	p2	(AG)7	14	45418	45431	potentially variable
PHG9_Scaffold_294	20	p3	(AGG)6	18	64951	64968	potentially variable
PHG9_Scaffold_294	30	p3	(AAG)5	15	99748	99762	potentially variable
PHG9_Scaffold_294	33	p2	(TA)9	18	114608	114625	potentially variable
PHG9_Scaffold_294	39	p2	(AT)6	12	141638	141649	
PHG9_Scaffold_294	41	p2	(AG)8	16	143561	143576	potentially variable
PHG9_Scaffold_295	8	p2	(AT)6	12	48145	48156	
PHG9_Scaffold_295	9	p2	(TA)7	14	52808	52821	potentially variable
PHG9_Scaffold_295	12	p2	(TA)7	14	73744	73757	potentially variable
PHG9_Scaffold_295	18	p2	(TA)9	18	90692	90709	potentially variable
PHG9_Scaffold_296	1	p2	(CT)6	12	300	311	
PHG9_Scaffold_296	2	p2	(AT)7	14	8935	8948	potentially variable

PHG9_Scaffold_296	9	p2	(TA)6	12	27054	27065	
PHG9_Scaffold_297	17	p2	(AC)9	18	45843	45860	potentially variable
PHG9_Scaffold_297	21	p2	(AT)8	16	59062	59077	potentially variable
PHG9_Scaffold_297	27	p3	(TGA)5	15	70734	70748	potentially variable
PHG9_Scaffold_297	28	p3	(ATT)5	15	78781	78795	potentially variable
PHG9_Scaffold_298	4	p2	(TA)6	12	15948	15959	
PHG9_Scaffold_298	19	p2	(AG)7	14	51005	51018	potentially variable
PHG9_Scaffold_298	23	p2	(AT)8	16	63707	63722	potentially variable
PHG9_Scaffold_298	31	p2	(AT)7	14	75978	75991	potentially variable
PHG9_Scaffold_298	32	p2	(TA)7	14	77765	77778	potentially variable
PHG9_Scaffold_298	33	p3	(CCA)5	15	78532	78546	potentially variable
PHG9_Scaffold_298	40	p2	(AG)6	12	101989	102000	
PHG9_Scaffold_298	45	p2	(CT)6	12	116255	116266	
PHG9_Scaffold_298	48	p2	(AT)6	12	125685	125696	
PHG9_Scaffold_298	54	p2	(TA)6	12	140465	140476	
PHG9_Scaffold_298	68	p3	(GAA)5	15	184692	184706	potentially variable
PHG9_Scaffold_298	70	p2	(CA)7	14	186787	186800	potentially variable
PHG9_Scaffold_298	71	p3	(ATT)5	15	188432	188446	potentially variable

PHG9_Scaffold_298	80	p2	(AC)9	18	208736	208753	potentially variable
PHG9_Scaffold_298	81	p2	(TA)6	12	216967	216978	
PHG9_Scaffold_298	91	p2	(AT)8	16	257044	257059	potentially variable
PHG9_Scaffold_298	92	p2	(AT)6	12	258896	258907	
PHG9_Scaffold_298	95	p2	(AT)7	14	264109	264122	potentially variable
PHG9_Scaffold_298	96	p2	(AT)6	12	265569	265580	
PHG9_Scaffold_298	103	p2	(AG)6	12	279671	279682	
PHG9_Scaffold_298	104	p3	(ATA)5	15	283711	283725	potentially variable
PHG9_Scaffold_298	107	p2	(AT)7	14	289218	289231	potentially variable
PHG9_Scaffold_298	116	p2	(GA)6	12	316877	316888	
PHG9_Scaffold_298	117	p2	(GA)7	14	319078	319091	potentially variable
PHG9_Scaffold_300	2	p2	(TA)6	12	18475	18486	
PHG9_Scaffold_300	10	p2	(TA)6	12	31535	31546	
PHG9_Scaffold_300	12	p3	(TGT)5	15	35220	35234	potentially variable
PHG9_Scaffold_300	28	p2	(TA)6	12	90568	90579	
PHG9_Scaffold_300	39	p3	(TCT)6	18	130770	130787	potentially variable
PHG9_Scaffold_300	41	p3	(TCT)5	15	141088	141102	potentially variable
PHG9_Scaffold_300	48	p2	(AT)6	12	154072	154083	
PHG9_Scaffold_300	49	p3	(CAA)5	15	160311	160325	potentially variable

PHG9_Scaffold_300	50	p2	(AT)9	18	164801	164818	potentially variable
PHG9_Scaffold_300	54	p3	(ATT)5	15	168242	168256	potentially variable
PHG9_Scaffold_301	9	p2	(AT)6	12	17689	17700	
PHG9_Scaffold_302	1	p2	(TG)6	12	1126	1137	
PHG9_Scaffold_304	2	p2	(TA)6	12	3502	3513	
PHG9_Scaffold_304	17	p2	(CA)6	12	60959	60970	
PHG9_Scaffold_304	18	p2	(AG)6	12	61491	61502	
PHG9_Scaffold_304	31	p2	(AT)6	12	148246	148257	
PHG9_Scaffold_304	32	p3	(GCA)5	15	159793	159807	potentially variable
PHG9_Scaffold_304	39	p2	(AT)6	12	165281	165292	
PHG9_Scaffold_304	50	p2	(TA)8	16	218353	218368	potentially variable
PHG9_Scaffold_304	57	p2	(TC)6	12	231673	231684	
PHG9_Scaffold_304	58	p2	(CT)6	12	232875	232886	
PHG9_Scaffold_304	59	p2	(AT)6	12	235523	235534	
PHG9_Scaffold_305	1	p2	(AT)7	14	797	810	potentially variable
PHG9_Scaffold_305	4	p2	(AT)6	12	5408	5419	
PHG9_Scaffold_305	8	p2	(CT)6	12	16628	16639	
PHG9_Scaffold_305	17	p2	(TA)8	16	46949	46964	potentially variable
PHG9_Scaffold_305	18	p2	(CT)6	12	47630	47641	
PHG9_Scaffold_305	21	p3	(GAT)6	18	62962	62979	potentially variable
PHG9_Scaffold_305	26	p3	(TAT)6	18	69893	69910	potentially variable

PHG9_Scaffold_305	29	p2	(AT)6	12	78915	78926	
PHG9_Scaffold_305	35	p3	(AAG)5	15	83971	83985	potentially variable
PHG9_Scaffold_306	8	p2	(AT)8	16	32993	33008	potentially variable
PHG9_Scaffold_306	12	p2	(TA)6	12	44578	44589	
PHG9_Scaffold_306	17	p2	(TG)9	18	55812	55829	potentially variable
PHG9_Scaffold_306	28	p2	(AT)7	14	89765	89778	potentially variable
PHG9_Scaffold_306	29	p2	(GT)7	14	91823	91836	potentially variable
PHG9_Scaffold_306	53	p2	(CT)6	12	142336	142347	
PHG9_Scaffold_306	55	p2	(AT)7	14	144812	144825	potentially variable
PHG9_Scaffold_306	60	p2	(AG)6	12	158966	158977	
PHG9_Scaffold_306	65	p2	(CT)9	18	166059	166076	potentially variable
PHG9_Scaffold_306	68	p2	(TA)8	16	196016	196031	potentially variable
PHG9_Scaffold_306	70	p2	(AT)6	12	201307	201318	
PHG9_Scaffold_306	72	p2	(AT)6	12	211918	211929	
PHG9_Scaffold_306	75	p2	(TA)6	12	223523	223534	
PHG9_Scaffold_307	5	p2	(TA)7	14	8070	8083	potentially variable
PHG9_Scaffold_307	7	p2	(AT)7	14	18949	18962	potentially variable
PHG9_Scaffold_307	8	p2	(TA)7	14	21055	21068	potentially variable

PHG9_Scaffold_307	9	p2	(AT)7	14	26884	26897	potentially variable
PHG9_Scaffold_307	14	p2	(CA)7	14	37579	37592	potentially variable
PHG9_Scaffold_307	30	p2	(AT)6	12	73899	73910	
PHG9_Scaffold_307	50	p2	(TG)6	12	109292	109303	
PHG9_Scaffold_307	53	p2	(AT)6	12	114252	114263	
PHG9_Scaffold_308	4	p3	(AGA)5	15	19870	19884	potentially variable
PHG9_Scaffold_308	5	p2	(AG)6	12	20439	20450	
PHG9_Scaffold_308	10	p2	(TA)7	14	46348	46361	potentially variable
PHG9_Scaffold_308	21	p2	(TA)6	12	62639	62650	
PHG9_Scaffold_308	26	p3	(GAA)5	15	96090	96104	potentially variable
PHG9_Scaffold_308	29	p2	(AG)6	12	108811	108822	
PHG9_Scaffold_308	33	p2	(TC)6	12	123874	123885	
PHG9_Scaffold_308	40	p3	(TAA)6	18	146730	146747	potentially variable
PHG9_Scaffold_308	51	p2	(AT)6	12	178696	178707	
PHG9_Scaffold_308	66	p2	(AC)6	12	224044	224055	
PHG9_Scaffold_308	69	p2	(AT)6	12	234561	234572	
PHG9_Scaffold_308	75	p2	(TC)6	12	255486	255497	
PHG9_Scaffold_308	78	p2	(TA)6	12	266640	266651	
PHG9_Scaffold_308	79	p3	(AAT)6	18	274918	274935	potentially variable
PHG9_Scaffold_308	81	p3	(AAT)5	15	277198	277212	potentially variable
PHG9_Scaffold_308	82	p3	(CTT)5	15	277462	277476	potentially

							variable
PHG9_Scaffold_308	85	p2	(TA)6	12	283184	283195	
PHG9_Scaffold_308	87	p3	(TCT)5	15	284953	284967	potentially variable
PHG9_Scaffold_309	1	p2	(AG)8	16	7862	7877	potentially variable
PHG9_Scaffold_309	2	p2	(AT)6	12	8952	8963	
PHG9_Scaffold_311	3	p3	(AGA)5	15	25329	25343	potentially variable
PHG9_Scaffold_315	8	p3	(TAT)5	15	50600	50614	potentially variable
PHG9_Scaffold_315	15	p3	(TAA)6	18	80999	81016	potentially variable
PHG9_Scaffold_315	21	p2	(AT)8	16	86102	86117	potentially variable
PHG9_Scaffold_316	1	p3	(TAA)6	18	2162	2179	potentially variable
PHG9_Scaffold_316	6	p2	(TA)6	12	7275	7286	
PHG9_Scaffold_316	9	p2	(GT)9	18	13307	13324	potentially variable
PHG9_Scaffold_316	11	p2	(AT)6	12	35710	35721	
PHG9_Scaffold_317	1	p3	(CCA)5	15	3866	3880	potentially variable
PHG9_Scaffold_317	11	p2	(TA)6	12	53265	53276	
PHG9_Scaffold_317	19	p2	(AT)6	12	72485	72496	
PHG9_Scaffold_317	25	p2	(TA)8	16	81806	81821	potentially variable
PHG9_Scaffold_317	28	p2	(TC)7	14	87426	87439	potentially variable

PHG9_Scaffold_317	33	p3	(ATG)5	15	96067	96081	potentially variable
PHG9_Scaffold_317	35	p2	(AT)6	12	101627	101638	
PHG9_Scaffold_319	5	p2	(TA)7	14	58601	58614	potentially variable
PHG9_Scaffold_319	6	p3	(ATT)5	15	68952	68966	potentially variable
PHG9_Scaffold_319	29	p2	(TA)6	12	147739	147750	
PHG9_Scaffold_320	3	p2	(AT)6	12	9762	9773	
PHG9_Scaffold_320	9	p3	(GAA)5	15	34030	34044	potentially variable
PHG9_Scaffold_321	9	p3	(TTA)6	18	26442	26459	potentially variable
PHG9_Scaffold_321	10	p2	(TA)6	12	31123	31134	
PHG9_Scaffold_321	14	p2	(TA)9	18	68195	68212	potentially variable
PHG9_Scaffold_321	16	p3	(ATT)5	15	84639	84653	potentially variable
PHG9_Scaffold_322	4	p2	(GT)8	16	2802	2817	potentially variable
PHG9_Scaffold_323	5	p2	(AG)6	12	46979	46990	
PHG9_Scaffold_325	2	p3	(ATA)5	15	5116	5130	potentially variable
PHG9_Scaffold_326	9	p3	(TTA)5	15	31926	31940	potentially variable
PHG9_Scaffold_326	12	p2	(CT)6	12	37699	37710	
PHG9_Scaffold_326	16	p2	(CT)9	18	47948	47965	potentially variable
PHG9_Scaffold_326	21	p2	(TA)9	18	58147	58164	potentially variable

PHG9_Scaffold_326	27	p2	(TA)7	14	69974	69987	potentially variable
PHG9_Scaffold_326	40	p2	(TA)9	18	112582	112599	potentially variable
PHG9_Scaffold_326	41	p2	(AT)6	12	114667	114678	
PHG9_Scaffold_330	1	p2	(AT)6	12	602	613	
PHG9_Scaffold_330	8	p2	(AT)6	12	28781	28792	
PHG9_Scaffold_330	11	p2	(AC)8	16	36857	36872	potentially variable
PHG9_Scaffold_330	18	p3	(AAC)5	15	61982	61996	potentially variable
PHG9_Scaffold_330	19	p3	(ACA)5	15	62506	62520	potentially variable
PHG9_Scaffold_330	20	p2	(AT)9	18	63214	63231	potentially variable
PHG9_Scaffold_330	25	p2	(AT)8	16	71104	71119	potentially variable
PHG9_Scaffold_330	31	p3	(CTC)5	15	83713	83727	potentially variable
PHG9_Scaffold_330	33	p2	(AT)8	16	84688	84703	potentially variable
PHG9_Scaffold_330	35	p2	(AT)9	18	86799	86816	potentially variable
PHG9_Scaffold_330	38	p2	(TA)8	16	95142	95157	potentially variable
PHG9_Scaffold_330	53	p2	(TA)6	12	137614	137625	
PHG9_Scaffold_330	54	p3	(TAT)5	15	138506	138520	potentially variable
PHG9_Scaffold_330	59	p6	(GAATTG)5	30	151917	151946	Hypervariable
PHG9_Scaffold_330	66	p2	(TA)9	18	158164	158181	potentially

							variable
PHG9_Scaffold_330	69	p2	(TG)6	12	168849	168860	
PHG9_Scaffold_330	74	p2	(TA)7	14	183870	183883	potentially variable
PHG9_Scaffold_330	77	p2	(GA)6	12	206263	206274	
PHG9_Scaffold_330	84	p3	(AAT)6	18	229837	229854	potentially variable
PHG9_Scaffold_330	86	p2	(AT)7	14	232170	232183	potentially variable
PHG9_Scaffold_330	87	p2	(TA)6	12	234098	234109	
PHG9_Scaffold_330	88	p2	(AT)6	12	243007	243018	
PHG9_Scaffold_330	98	p3	(TAT)5	15	273143	273157	potentially variable
PHG9_Scaffold_330	99	p2	(TG)6	12	275086	275097	
PHG9_Scaffold_330	103	p2	(AG)6	12	293084	293095	
PHG9_Scaffold_330	105	p2	(AT)9	18	297789	297806	potentially variable
PHG9_Scaffold_335	4	p3	(CTT)5	15	18395	18409	potentially variable
PHG9_Scaffold_335	7	p2	(TA)7	14	27461	27474	potentially variable
PHG9_Scaffold_335	9	p3	(GAT)6	18	36698	36715	potentially variable
PHG9_Scaffold_335	11	p2	(TA)8	16	39791	39806	potentially variable
PHG9_Scaffold_335	12	p2	(AT)9	18	41907	41924	potentially variable
PHG9_Scaffold_335	26	p2	(AC)7	14	86540	86553	potentially variable

PHG9_Scaffold_335	27	p2	(GT)6	12	87292	87303	
PHG9_Scaffold_335	30	p2	(AG)8	16	97668	97683	potentially variable
PHG9_Scaffold_335	32	p2	(GA)6	12	101096	101107	
PHG9_Scaffold_335	33	p2	(AT)8	16	101761	101776	potentially variable
PHG9_Scaffold_335	37	p3	(CCT)5	15	113073	113087	potentially variable
PHG9_Scaffold_335	38	p2	(AT)8	16	115368	115383	potentially variable
PHG9_Scaffold_335	40	p2	(TC)6	12	129673	129684	
PHG9_Scaffold_335	44	p2	(AT)6	12	142703	142714	
PHG9_Scaffold_335	45	p3	(GTT)5	15	145105	145119	potentially variable
PHG9_Scaffold_335	54	p2	(AT)6	12	190512	190523	
PHG9_Scaffold_335	55	p2	(AT)6	12	190892	190903	
PHG9_Scaffold_335	60	p2	(TG)6	12	202734	202745	
PHG9_Scaffold_335	80	p2	(AG)7	14	271316	271329	potentially variable
PHG9_Scaffold_335	85	p2	(AT)9	18	291347	291364	potentially variable
PHG9_Scaffold_335	98	p2	(CA)8	16	338151	338166	potentially variable
PHG9_Scaffold_335	106	p2	(AT)8	16	356440	356455	potentially variable
PHG9_Scaffold_335	109	p2	(AC)9	18	362833	362850	potentially variable
PHG9_Scaffold_336	3	p3	(AAT)5	15	33281	33295	potentially variable

PHG9_Scaffold_336	6	p2	(TA)8	16	46095	46110	potentially variable
PHG9_Scaffold_336	8	p2	(AT)6	12	69667	69678	
PHG9_Scaffold_338	1	p2	(TC)9	18	1645	1662	potentially variable
PHG9_Scaffold_339	25	p2	(TA)6	12	127381	127392	
PHG9_Scaffold_340	20	p2	(TA)6	12	50913	50924	
PHG9_Scaffold_340	25	p3	(CAA)5	15	64772	64786	potentially variable
PHG9_Scaffold_340	28	p3	(TAT)5	15	76945	76959	potentially variable
PHG9_Scaffold_340	30	p2	(AT)6	12	78977	78988	
PHG9_Scaffold_340	40	p3	(TAA)5	15	114781	114795	potentially variable
PHG9_Scaffold_340	42	p2	(AT)6	12	116213	116224	
PHG9_Scaffold_340	46	p3	(AAT)5	15	133323	133337	potentially variable
PHG9_Scaffold_340	50	p2	(AT)7	14	144157	144170	potentially variable
PHG9_Scaffold_340	52	p3	(CAA)5	15	154823	154837	potentially variable
PHG9_Scaffold_340	59	p2	(TA)9	18	171977	171994	potentially variable
PHG9_Scaffold_340	69	p2	(CA)7	14	205518	205531	potentially variable
PHG9_Scaffold_340	70	p2	(AG)7	14	207576	207589	potentially variable
PHG9_Scaffold_340	71	p2	(TA)6	12	208736	208747	
PHG9_Scaffold_340	75	p2	(AT)8	16	216592	216607	potentially variable

PHG9_Scaffold_340	77	p2	(TG)7	14	220940	220953	potentially variable
PHG9_Scaffold_340	79	p2	(AT)6	12	222595	222606	
PHG9_Scaffold_340	82	p2	(TA)8	16	231610	231625	potentially variable
PHG9_Scaffold_340	87	p2	(GA)8	16	237676	237691	potentially variable
PHG9_Scaffold_340	96	p2	(CA)7	14	261341	261354	potentially variable
PHG9_Scaffold_340	101	p2	(AT)6	12	267683	267694	
PHG9_Scaffold_340	105	p2	(TA)6	12	279118	279129	
PHG9_Scaffold_340	113	p2	(AT)7	14	293793	293806	potentially variable
PHG9_Scaffold_340	114	p2	(TA)8	16	294258	294273	potentially variable
PHG9_Scaffold_340	119	p2	(AT)8	16	317845	317860	potentially variable
PHG9_Scaffold_340	126	p2	(TA)8	16	330198	330213	potentially variable
PHG9_Scaffold_340	137	p3	(GTG)5	15	356080	356094	potentially variable
PHG9_Scaffold_340	139	p2	(TA)9	18	367457	367474	potentially variable
PHG9_Scaffold_340	143	p2	(CA)7	14	379919	379932	potentially variable
PHG9_Scaffold_340	154	p2	(AT)6	12	405301	405312	
PHG9_Scaffold_343	5	p2	(TA)8	16	8534	8549	potentially variable
PHG9_Scaffold_343	9	p2	(AG)6	12	39341	39352	
PHG9_Scaffold_343	11	p3	(TAT)5	15	61027	61041	potentially

							variable
PHG9_Scaffold_343	19	p3	(AAG)5	15	85011	85025	potentially variable
PHG9_Scaffold_343	28	p2	(AT)7	14	151590	151603	potentially variable
PHG9_Scaffold_343	31	p2	(AT)7	14	158785	158798	potentially variable
PHG9_Scaffold_343	33	p3	(CGG)5	15	172623	172637	potentially variable
PHG9_Scaffold_343	34	p2	(TC)6	12	180875	180886	
PHG9_Scaffold_343	42	p2	(AT)6	12	201268	201279	
PHG9_Scaffold_344	4	p2	(AT)8	16	4315	4330	potentially variable
PHG9_Scaffold_344	7	p2	(CT)6	12	15364	15375	
PHG9_Scaffold_344	8	p2	(TA)6	12	19451	19462	
PHG9_Scaffold_344	11	p3	(ATA)5	15	25571	25585	potentially variable
PHG9_Scaffold_345	11	p2	(TC)6	12	26982	26993	
PHG9_Scaffold_345	12	p2	(GA)9	18	29167	29184	potentially variable
PHG9_Scaffold_345	13	p2	(TA)9	18	30326	30343	potentially variable
PHG9_Scaffold_345	29	p2	(TA)10	20	75656	75675	Hypervariable
PHG9_Scaffold_346	4	p3	(TAT)5	15	12897	12911	potentially variable
PHG9_Scaffold_346	5	p2	(TA)7	14	13651	13664	potentially variable
PHG9_Scaffold_348	2	p3	(TTA)5	15	8310	8324	potentially variable

PHG9_Scaffold_348	7	p2	(AG)6	12	12881	12892	
PHG9_Scaffold_348	11	p2	(AT)6	12	30575	30586	
PHG9_Scaffold_348	23	p3	(CTT)5	15	64344	64358	potentially variable
PHG9_Scaffold_348	25	p2	(TA)8	16	70808	70823	potentially variable
PHG9_Scaffold_348	31	p2	(TA)6	12	76126	76137	
PHG9_Scaffold_349	2	p2	(TA)6	12	3820	3831	
PHG9_Scaffold_349	8	p3	(TGT)5	15	19812	19826	potentially variable
PHG9_Scaffold_349	11	p2	(TA)7	14	31316	31329	potentially variable
PHG9_Scaffold_349	24	p2	(AT)6	12	98730	98741	
PHG9_Scaffold_349	41	p2	(TA)6	12	139923	139934	
PHG9_Scaffold_349	47	p2	(AC)7	14	147254	147267	potentially variable
PHG9_Scaffold_350	8	p3	(ATC)5	15	9583	9597	potentially variable
PHG9_Scaffold_350	14	p2	(AT)6	12	22934	22945	
PHG9_Scaffold_350	17	p2	(TA)8	16	26763	26778	potentially variable
PHG9_Scaffold_350	19	p2	(AT)9	18	46458	46475	potentially variable
PHG9_Scaffold_350	26	p2	(TA)6	12	63797	63808	
PHG9_Scaffold_350	27	p2	(GA)6	12	64108	64119	
PHG9_Scaffold_350	28	p2	(TA)8	16	64950	64965	potentially variable
PHG9_Scaffold_350	34	p2	(CT)6	12	82158	82169	
PHG9_Scaffold_350	35	p2	(CA)6	12	84000	84011	

PHG9_Scaffold_350	37	p3	(AAT)5	15	84730	84744	potentially variable
PHG9_Scaffold_351	2	p2	(TA)7	14	1687	1700	potentially variable
PHG9_Scaffold_351	5	p2	(TA)6	12	13018	13029	
PHG9_Scaffold_351	9	p2	(AT)8	16	25606	25621	potentially variable
PHG9_Scaffold_351	11	p3	(ATG)5	15	32080	32094	potentially variable
PHG9_Scaffold_351	12	p3	(CAT)5	15	32598	32612	potentially variable
PHG9_Scaffold_351	13	p2	(TA)7	14	33095	33108	potentially variable
PHG9_Scaffold_351	18	p3	(TAG)5	15	44509	44523	potentially variable
PHG9_Scaffold_351	22	p2	(GA)9	18	57288	57305	potentially variable
PHG9_Scaffold_351	24	p3	(AAT)5	15	70290	70304	potentially variable
PHG9_Scaffold_352	8	p2	(AT)9	18	13239	13256	potentially variable
PHG9_Scaffold_352	9	p3	(TTG)5	15	14669	14683	potentially variable
PHG9_Scaffold_352	15	p3	(GAT)5	15	30796	30810	potentially variable
PHG9_Scaffold_352	21	p2	(TA)6	12	43080	43091	
PHG9_Scaffold_352	23	p3	(AGA)5	15	45940	45954	potentially variable
PHG9_Scaffold_352	33	p2	(AT)6	12	85712	85723	
PHG9_Scaffold_353	2	p2	(CT)9	18	5790	5807	potentially

							variable
PHG9_Scaffold_353	10	p3	(CAG)5	15	25105	25119	potentially variable
PHG9_Scaffold_354	2	p2	(GA)6	12	645	656	
PHG9_Scaffold_354	7	p2	(TA)6	12	9063	9074	
PHG9_Scaffold_357	1	p2	(TC)8	16	1081	1096	potentially variable
PHG9_Scaffold_357	5	p2	(AC)7	14	5439	5452	potentially variable
PHG9_Scaffold_357	6	p2	(AC)8	16	8198	8213	potentially variable
PHG9_Scaffold_357	12	p3	(GGT)5	15	23481	23495	potentially variable
PHG9_Scaffold_357	15	p2	(AT)6	12	48281	48292	
PHG9_Scaffold_357	16	p2	(TA)8	16	63723	63738	potentially variable
PHG9_Scaffold_358	1	p2	(GT)6	12	588	599	
PHG9_Scaffold_358	9	p2	(GT)7	14	17114	17127	potentially variable
PHG9_Scaffold_358	15	p3	(TAT)5	15	36939	36953	potentially variable
PHG9_Scaffold_358	17	p2	(AC)6	12	49210	49221	
PHG9_Scaffold_358	28	p2	(AT)6	12	71464	71475	
PHG9_Scaffold_358	35	p2	(AG)7	14	91026	91039	potentially variable
PHG9_Scaffold_358	36	p3	(ATA)5	15	95455	95469	potentially variable
PHG9_Scaffold_358	43	p3	(AAT)5	15	99681	99695	potentially variable

PHG9_Scaffold_359	2	p2	(TG)8	16	6542	6557	potentially variable
PHG9_Scaffold_359	15	p3	(TTA)5	15	87490	87504	potentially variable
PHG9_Scaffold_360	1	p3	(ATA)5	15	444	458	potentially variable
PHG9_Scaffold_360	5	p3	(ATT)5	15	48758	48772	potentially variable
PHG9_Scaffold_360	16	p2	(AT)8	16	94158	94173	potentially variable
PHG9_Scaffold_362	1	p2	(TC)6	12	5055	5066	
PHG9_Scaffold_362	8	p3	(ATT)6	18	44377	44394	potentially variable
PHG9_Scaffold_362	23	p2	(TA)7	14	92067	92080	potentially variable
PHG9_Scaffold_363	4	p3	(GAT)5	15	9804	9818	potentially variable
PHG9_Scaffold_363	8	p2	(AT)9	18	17737	17754	potentially variable
PHG9_Scaffold_363	12	p2	(TA)8	16	23260	23275	potentially variable
PHG9_Scaffold_363	14	p2	(TA)6	12	29977	29988	
PHG9_Scaffold_363	18	p2	(TC)6	12	35005	35016	
PHG9_Scaffold_363	21	p3	(ATC)5	15	44537	44551	potentially variable
PHG9_Scaffold_363	24	p2	(TA)9	18	49343	49360	potentially variable
PHG9_Scaffold_363	26	p3	(TTA)5	15	50167	50181	potentially variable
PHG9_Scaffold_363	28	p3	(CAA)5	15	56898	56912	potentially

							variable
PHG9_Scaffold_363	30	p3	(TAT)5	15	63260	63274	potentially variable
PHG9_Scaffold_363	32	p2	(TA)8	16	70612	70627	potentially variable
PHG9_Scaffold_363	42	p2	(AC)6	12	92910	92921	
PHG9_Scaffold_363	44	p2	(TA)6	12	95826	95837	
PHG9_Scaffold_363	51	p2	(TA)9	18	118048	118065	potentially variable
PHG9_Scaffold_363	53	p2	(TA)7	14	119982	119995	potentially variable
PHG9_Scaffold_363	55	p2	(TA)7	14	122706	122719	potentially variable
PHG9_Scaffold_363	56	p3	(TAA)5	15	123656	123670	potentially variable
PHG9_Scaffold_363	64	p3	(GTT)5	15	133389	133403	potentially variable
PHG9_Scaffold_363	66	p2	(GA)8	16	137689	137704	potentially variable
PHG9_Scaffold_363	75	p2	(TA)8	16	159003	159018	potentially variable
PHG9_Scaffold_363	81	p2	(AT)9	18	175051	175068	potentially variable
PHG9_Scaffold_364	1	p2	(TA)6	12	2631	2642	
PHG9_Scaffold_364	12	p3	(TGT)5	15	28690	28704	potentially variable
PHG9_Scaffold_364	13	p3	(TTA)6	18	29328	29345	potentially variable
PHG9_Scaffold_364	14	p2	(AT)7	14	29963	29976	potentially variable

PHG9_Scaffold_364	20	p2	(AT)8	16	46403	46418	potentially variable
PHG9_Scaffold_364	28	p2	(TA)9	18	109901	109918	potentially variable
PHG9_Scaffold_364	39	p2	(AT)9	18	173893	173910	potentially variable
PHG9_Scaffold_366	11	p2	(CT)6	12	30745	30756	
PHG9_Scaffold_367	6	p3	(TGT)5	15	11801	11815	potentially variable
PHG9_Scaffold_367	7	p2	(TC)8	16	11958	11973	potentially variable
PHG9_Scaffold_367	14	p2	(AT)7	14	39055	39068	potentially variable
PHG9_Scaffold_367	16	p2	(CT)6	12	44164	44175	
PHG9_Scaffold_367	17	p3	(AGC)5	15	49056	49070	potentially variable
PHG9_Scaffold_369	1	p3	(ATA)5	15	85	99	potentially variable
PHG9_Scaffold_369	4	p2	(GA)6	12	4117	4128	
PHG9_Scaffold_371	5	p2	(AT)8	16	13018	13033	potentially variable
PHG9_Scaffold_378	5	p2	(AG)6	12	9896	9907	
PHG9_Scaffold_378	18	p3	(ATA)5	15	45007	45021	potentially variable
PHG9_Scaffold_378	19	p2	(GA)6	12	53883	53894	
PHG9_Scaffold_378	20	p2	(AT)7	14	63107	63120	potentially variable
PHG9_Scaffold_378	24	p2	(CT)6	12	72730	72741	
PHG9_Scaffold_378	32	p2	(AT)8	16	81311	81326	potentially variable

PHG9_Scaffold_378	33	p2	(AG)8	16	81713	81728	potentially variable
PHG9_Scaffold_379	23	p2	(TA)7	14	90155	90168	potentially variable
PHG9_Scaffold_379	25	p2	(AT)8	16	91575	91590	potentially variable
PHG9_Scaffold_381	4	p2	(TA)8	16	22784	22799	potentially variable
PHG9_Scaffold_382	2	p2	(TA)6	12	2818	2829	
PHG9_Scaffold_382	4	p2	(AT)8	16	7133	7148	potentially variable
PHG9_Scaffold_382	9	p2	(GT)7	14	17905	17918	potentially variable
PHG9_Scaffold_382	16	p2	(AT)6	12	62099	62110	
PHG9_Scaffold_382	17	p2	(TA)9	18	63297	63314	potentially variable
PHG9_Scaffold_383	11	p2	(TA)6	12	111813	111824	
PHG9_Scaffold_383	23	p3	(AAT)5	15	190781	190795	potentially variable
PHG9_Scaffold_383	28	p3	(TAT)5	15	229716	229730	potentially variable
PHG9_Scaffold_383	31	p2	(AT)6	12	236851	236862	
PHG9_Scaffold_383	36	p2	(TA)6	12	255756	255767	
PHG9_Scaffold_384	8	p2	(TA)8	16	61674	61689	potentially variable
PHG9_Scaffold_384	12	p2	(AT)7	14	81035	81048	potentially variable
PHG9_Scaffold_385	3	p3	(GTT)5	15	6080	6094	potentially variable
PHG9_Scaffold_385	6	p3	(ATT)5	15	17165	17179	potentially

							variable
PHG9_Scaffold_385	13	p3	(AAT)5	15	42169	42183	potentially variable
PHG9_Scaffold_385	17	p2	(TA)6	12	57255	57266	
PHG9_Scaffold_386	2	p2	(AT)6	12	26303	26314	
PHG9_Scaffold_386	10	p2	(TA)6	12	72461	72472	
PHG9_Scaffold_387	2	p2	(TA)6	12	3335	3346	
PHG9_Scaffold_387	13	p2	(AT)7	14	12918	12931	potentially variable
PHG9_Scaffold_387	14	p3	(GTA)5	15	18821	18835	potentially variable
PHG9_Scaffold_387	22	p3	(CAT)6	18	30637	30654	potentially variable
PHG9_Scaffold_389	2	p2	(AT)6	12	1265	1276	
PHG9_Scaffold_389	13	p2	(AT)6	12	32673	32684	
PHG9_Scaffold_389	15	p2	(AG)6	12	36378	36389	
PHG9_Scaffold_389	16	p2	(AC)7	14	37182	37195	potentially variable
PHG9_Scaffold_389	22	p2	(AT)6	12	55219	55230	
PHG9_Scaffold_389	30	p3	(GAA)5	15	64707	64721	potentially variable
PHG9_Scaffold_389	54	p2	(AT)8	16	144689	144704	potentially variable
PHG9_Scaffold_389	56	p2	(AG)6	12	148793	148804	
PHG9_Scaffold_389	57	p2	(TA)8	16	151193	151208	potentially variable
PHG9_Scaffold_389	58	p2	(AG)6	12	151964	151975	
PHG9_Scaffold_390	4	p2	(AT)9	18	12221	12238	potentially variable

PHG9_Scaffold_393	6	p2	(AG)6	12	15028	15039	
PHG9_Scaffold_393	9	p2	(AT)6	12	23367	23378	
PHG9_Scaffold_393	27	p3	(AAT)5	15	88872	88886	potentially variable
PHG9_Scaffold_393	35	p2	(AT)7	14	96050	96063	potentially variable
PHG9_Scaffold_394	5	p2	(TC)6	12	13926	13937	
PHG9_Scaffold_394	10	p2	(AG)7	14	26007	26020	potentially variable
PHG9_Scaffold_394	13	p2	(TA)6	12	28660	28671	
PHG9_Scaffold_394	14	p2	(GT)6	12	28859	28870	
PHG9_Scaffold_394	16	p2	(AT)6	12	49271	49282	
PHG9_Scaffold_394	25	p3	(TTG)5	15	62111	62125	potentially variable
PHG9_Scaffold_394	26	p3	(CAC)5	15	63293	63307	potentially variable
PHG9_Scaffold_394	30	p3	(CAA)5	15	73961	73975	potentially variable
PHG9_Scaffold_394	42	p2	(CT)6	12	117313	117324	
PHG9_Scaffold_394	44	p2	(TA)6	12	122891	122902	
PHG9_Scaffold_394	50	p3	(ATC)5	15	143167	143181	potentially variable
PHG9_Scaffold_394	59	p2	(AT)6	12	166180	166191	
PHG9_Scaffold_394	62	p2	(AT)7	14	168009	168022	potentially variable
PHG9_Scaffold_394	66	p2	(AT)6	12	186620	186631	
PHG9_Scaffold_394	70	p2	(TA)6	12	201693	201704	
PHG9_Scaffold_394	76	p3	(GAA)5	15	216488	216502	potentially variable

PHG9_Scaffold_394	79	p2	(CT)8	16	218121	218136	potentially variable
PHG9_Scaffold_394	82	p2	(AT)6	12	239103	239114	
PHG9_Scaffold_395	2	p2	(AT)7	14	11374	11387	potentially variable
PHG9_Scaffold_395	6	p3	(ATG)5	15	28383	28397	potentially variable
PHG9_Scaffold_395	12	p2	(TA)7	14	49313	49326	potentially variable
PHG9_Scaffold_396	5	p2	(TA)7	14	12299	12312	potentially variable
PHG9_Scaffold_396	8	p2	(AT)7	14	29058	29071	potentially variable
PHG9_Scaffold_396	10	p3	(GTG)5	15	35236	35250	potentially variable
PHG9_Scaffold_396	13	p2	(TA)7	14	45738	45751	potentially variable
PHG9_Scaffold_396	19	p2	(AT)7	14	64559	64572	potentially variable
PHG9_Scaffold_399	1	p2	(AT)6	12	1774	1785	
PHG9_Scaffold_399	3	p2	(AT)6	12	4300	4311	
PHG9_Scaffold_399	10	p2	(TA)8	16	45165	45180	potentially variable
PHG9_Scaffold_399	23	p3	(TAT)5	15	85332	85346	potentially variable
PHG9_Scaffold_401	1	p3	(CTT)5	15	31907	31921	potentially variable
PHG9_Scaffold_403	9	p2	(TA)6	12	48776	48787	
PHG9_Scaffold_403	19	p2	(AT)6	12	128651	128662	
PHG9_Scaffold_403	20	p2	(AT)8	16	129313	129328	potentially

							variable
PHG9_Scaffold_403	21	p2	(CT)7	14	136324	136337	potentially variable
PHG9_Scaffold_403	22	p3	(GTT)5	15	137903	137917	potentially variable
PHG9_Scaffold_403	33	p2	(AT)7	14	208731	208744	potentially variable
PHG9_Scaffold_403	40	p3	(TTA)5	15	236956	236970	potentially variable
PHG9_Scaffold_404	3	p3	(AAT)5	15	19853	19867	potentially variable
PHG9_Scaffold_405	6	p2	(AT)6	12	12408	12419	
PHG9_Scaffold_405	8	p2	(TA)6	12	14555	14566	
PHG9_Scaffold_405	9	p2	(AT)7	14	15595	15608	potentially variable
PHG9_Scaffold_405	15	p2	(AT)6	12	38873	38884	
PHG9_Scaffold_405	23	p2	(TG)7	14	60524	60537	potentially variable
PHG9_Scaffold_405	24	p2	(TA)6	12	65646	65657	
PHG9_Scaffold_405	32	p2	(CA)8	16	84762	84777	potentially variable
PHG9_Scaffold_405	34	p3	(ATC)5	15	91688	91702	potentially variable
PHG9_Scaffold_405	37	p2	(AT)8	16	111704	111719	potentially variable
PHG9_Scaffold_405	41	p2	(AT)7	14	114273	114286	potentially variable
PHG9_Scaffold_405	52	p2	(TC)6	12	133882	133893	
PHG9_Scaffold_406	12	p2	(AT)7	14	74760	74773	potentially variable

PHG9_Scaffold_406	17	p2	(TA)6	12	97232	97243	
PHG9_Scaffold_408	4	p3	(ATA)6	18	3408	3425	potentially variable
PHG9_Scaffold_408	16	p2	(TC)8	16	48184	48199	potentially variable
PHG9_Scaffold_408	19	p2	(TG)6	12	57614	57625	
PHG9_Scaffold_408	23	p3	(ATC)5	15	60298	60312	potentially variable
PHG9_Scaffold_408	29	p2	(AT)6	12	67808	67819	
PHG9_Scaffold_408	35	p3	(AAT)5	15	85162	85176	potentially variable
PHG9_Scaffold_408	36	p2	(TG)6	12	85487	85498	
PHG9_Scaffold_408	39	p2	(AT)6	12	97264	97275	
PHG9_Scaffold_408	44	p3	(GAA)6	18	105327	105344	potentially variable
PHG9_Scaffold_408	45	p3	(GAA)6	18	110685	110702	potentially variable
PHG9_Scaffold_408	46	p2	(GA)6	12	117251	117262	
PHG9_Scaffold_410	2	p3	(CCA)5	15	22100	22114	potentially variable
PHG9_Scaffold_410	5	p2	(TA)7	14	28570	28583	potentially variable
PHG9_Scaffold_410	12	p2	(AT)6	12	42731	42742	
PHG9_Scaffold_410	15	p3	(GAT)5	15	47380	47394	potentially variable
PHG9_Scaffold_411	8	p2	(GA)8	16	26157	26172	potentially variable
PHG9_Scaffold_411	19	p2	(AG)9	18	55542	55559	potentially variable

PHG9_Scaffold_411	23	p2	(AT)6	12	60553	60564	
PHG9_Scaffold_411	31	p2	(AT)8	16	78261	78276	potentially variable
PHG9_Scaffold_412	2	p2	(AT)6	12	5590	5601	
PHG9_Scaffold_412	4	p2	(GA)6	12	24328	24339	
PHG9_Scaffold_412	5	p2	(TC)6	12	27035	27046	
PHG9_Scaffold_412	23	p2	(TG)7	14	88859	88872	potentially variable
PHG9_Scaffold_412	28	p2	(AT)7	14	111556	111569	potentially variable
PHG9_Scaffold_412	29	p3	(ATT)6	18	116601	116618	potentially variable
PHG9_Scaffold_412	33	p3	(TGC)5	15	125253	125267	potentially variable
PHG9_Scaffold_412	36	p2	(CA)6	12	133251	133262	
PHG9_Scaffold_412	45	p2	(AT)7	14	173752	173765	potentially variable
PHG9_Scaffold_413	4	p2	(AG)8	16	18124	18139	potentially variable
PHG9_Scaffold_413	5	p2	(TC)8	16	18657	18672	potentially variable
PHG9_Scaffold_413	11	p2	(TA)8	16	40503	40518	potentially variable
PHG9_Scaffold_413	14	p2	(AG)6	12	65693	65704	
PHG9_Scaffold_413	17	p2	(TA)8	16	68483	68498	potentially variable
PHG9_Scaffold_413	18	p2	(TA)8	16	75639	75654	potentially variable
PHG9_Scaffold_415	2	p2	(AT)7	14	5011	5024	potentially variable

PHG9_Scaffold_415	3	p2	(TA)6	12	5996	6007	
PHG9_Scaffold_415	4	p2	(TA)6	12	6672	6683	
PHG9_Scaffold_415	16	p3	(AAT)5	15	37646	37660	potentially variable
PHG9_Scaffold_415	21	p3	(GTT)5	15	70163	70177	potentially variable
PHG9_Scaffold_415	23	p2	(CT)7	14	71379	71392	potentially variable
PHG9_Scaffold_416	2	p3	(ATA)5	15	1662	1676	potentially variable
PHG9_Scaffold_417	14	p2	(TC)6	12	53812	53823	
PHG9_Scaffold_417	16	p2	(TA)6	12	55867	55878	
PHG9_Scaffold_421	2	p3	(AAT)5	15	12147	12161	potentially variable
PHG9_Scaffold_421	3	p2	(TA)8	16	12438	12453	potentially variable
PHG9_Scaffold_423	7	p2	(AT)9	18	15804	15821	potentially variable
PHG9_Scaffold_423	17	p2	(TC)6	12	30187	30198	
PHG9_Scaffold_423	31	p2	(TA)8	16	64675	64690	potentially variable
PHG9_Scaffold_423	32	p2	(AT)7	14	67035	67048	potentially variable
PHG9_Scaffold_425	2	p2	(TA)8	16	3358	3373	potentially variable
PHG9_Scaffold_425	6	p2	(GA)6	12	7004	7015	
PHG9_Scaffold_425	10	p3	(ATC)6	18	17048	17065	potentially variable
PHG9_Scaffold_425	16	p2	(CT)6	12	34839	34850	

PHG9_Scaffold_425	24	p2	(AT)9	18	57958	57975	potentially variable
PHG9_Scaffold_425	28	p2	(GA)9	18	64139	64156	potentially variable
PHG9_Scaffold_425	29	p2	(AT)6	12	66150	66161	
PHG9_Scaffold_425	39	p2	(AT)9	18	103347	103364	potentially variable
PHG9_Scaffold_425	41	p2	(AT)6	12	106162	106173	
PHG9_Scaffold_425	42	p2	(CT)6	12	109353	109364	
PHG9_Scaffold_425	44	p2	(AT)9	18	119081	119098	potentially variable
PHG9_Scaffold_425	47	p2	(TA)7	14	120957	120970	potentially variable
PHG9_Scaffold_425	48	p2	(TC)6	12	128210	128221	
PHG9_Scaffold_425	49	p2	(TC)9	18	128470	128487	potentially variable
PHG9_Scaffold_427	1	p3	(ATT)5	15	5001	5015	potentially variable
PHG9_Scaffold_427	2	p2	(TA)7	14	7930	7943	potentially variable
PHG9_Scaffold_427	5	p2	(AT)6	12	12255	12266	
PHG9_Scaffold_428	4	p2	(TA)7	14	23486	23499	potentially variable
PHG9_Scaffold_428	7	p2	(GT)9	18	36441	36458	potentially variable
PHG9_Scaffold_428	8	p2	(TG)6	12	36840	36851	
PHG9_Scaffold_428	11	p2	(CT)6	12	44081	44092	
PHG9_Scaffold_428	15	p2	(AT)9	18	48531	48548	potentially variable

PHG9_Scaffold_428	28	p2	(TA)9	18	108747	108764	potentially variable
PHG9_Scaffold_428	45	p3	(TTC)5	15	166554	166568	potentially variable
PHG9_Scaffold_428	46	p3	(TAT)5	15	176597	176611	potentially variable
PHG9_Scaffold_429	2	p2	(AT)7	14	5896	5909	potentially variable
PHG9_Scaffold_429	8	p2	(AT)7	14	46486	46499	potentially variable
PHG9_Scaffold_430	2	p2	(AT)7	14	2193	2206	potentially variable
PHG9_Scaffold_430	3	p2	(TA)6	12	4143	4154	
PHG9_Scaffold_430	8	p2	(AT)6	12	8997	9008	
PHG9_Scaffold_430	16	p3	(CAC)6	18	33879	33896	potentially variable
PHG9_Scaffold_430	17	p2	(AT)8	16	36077	36092	potentially variable
PHG9_Scaffold_430	18	p3	(GAA)5	15	39290	39304	potentially variable
PHG9_Scaffold_430	19	p2	(AT)6	12	53271	53282	
PHG9_Scaffold_430	27	p2	(TA)9	18	82967	82984	potentially variable
PHG9_Scaffold_431	6	p2	(AT)9	18	20596	20613	potentially variable
PHG9_Scaffold_431	10	p2	(AT)9	18	34049	34066	potentially variable
PHG9_Scaffold_431	15	p2	(TA)6	12	49312	49323	
PHG9_Scaffold_431	23	p2	(TA)6	12	73621	73632	
PHG9_Scaffold_431	24	p2	(TA)8	16	75953	75968	potentially

							variable
PHG9_Scaffold_431	27	p6	(TTTCTT)5	30	80440	80469	Hypervariable
PHG9_Scaffold_431	29	p2	(TA)7	14	85990	86003	potentially variable
PHG9_Scaffold_431	30	p2	(AT)6	12	86605	86616	
PHG9_Scaffold_431	31	p2	(AT)6	12	94281	94292	
PHG9_Scaffold_431	32	p2	(TA)6	12	97447	97458	
PHG9_Scaffold_431	33	p2	(TA)7	14	100904	100917	potentially variable
PHG9_Scaffold_431	34	p2	(GT)7	14	102910	102923	potentially variable
PHG9_Scaffold_431	43	p2	(CT)6	12	126910	126921	
PHG9_Scaffold_432	12	p2	(CT)6	12	23009	23020	
PHG9_Scaffold_432	13	p2	(AG)7	14	23341	23354	potentially variable
PHG9_Scaffold_432	23	p3	(ACA)5	15	63698	63712	potentially variable
PHG9_Scaffold_432	29	p3	(ATA)5	15	74146	74160	potentially variable
PHG9_Scaffold_432	30	p2	(AT)8	16	74920	74935	potentially variable
PHG9_Scaffold_432	46	p2	(AT)7	14	114568	114581	potentially variable
PHG9_Scaffold_432	47	p2	(TA)7	14	117601	117614	potentially variable
PHG9_Scaffold_432	53	p2	(AT)6	12	141344	141355	
PHG9_Scaffold_433	3	p3	(GAG)5	15	2650	2664	potentially variable
PHG9_Scaffold_433	5	p3	(AAT)5	15	10175	10189	potentially

							variable
PHG9_Scaffold_433	22	p2	(TG)6	12	54286	54297	
PHG9_Scaffold_433	23	p2	(TC)6	12	61171	61182	
PHG9_Scaffold_433	24	p2	(TA)8	16	62392	62407	potentially variable
PHG9_Scaffold_433	31	p2	(TA)8	16	73366	73381	potentially variable
PHG9_Scaffold_433	32	p2	(TA)6	12	73591	73602	
PHG9_Scaffold_433	38	p2	(TA)6	12	93311	93322	
PHG9_Scaffold_433	39	p2	(AT)9	18	99065	99082	potentially variable
PHG9_Scaffold_433	44	p2	(AT)7	14	107713	107726	potentially variable
PHG9_Scaffold_433	45	p2	(AC)7	14	111383	111396	potentially variable
PHG9_Scaffold_433	49	p2	(TA)6	12	120185	120196	
PHG9_Scaffold_436	11	p6	(ATCTCA)5	30	21730	21759	Hypervariable
PHG9_Scaffold_436	15	p3	(ATA)5	15	29035	29049	potentially variable
PHG9_Scaffold_436	18	p2	(TA)6	12	31870	31881	
PHG9_Scaffold_436	22	p2	(AT)6	12	40918	40929	
PHG9_Scaffold_436	23	p2	(TA)6	12	42692	42703	
PHG9_Scaffold_436	24	p2	(GT)6	12	45550	45561	
PHG9_Scaffold_436	26	p2	(AG)6	12	52643	52654	
PHG9_Scaffold_436	38	p2	(TA)6	12	75134	75145	
PHG9_Scaffold_438	7	p2	(TA)6	12	27125	27136	
PHG9_Scaffold_438	9	p2	(TA)7	14	31239	31252	potentially variable
PHG9_Scaffold_438	10	p2	(AT)7	14	31476	31489	potentially

							variable
PHG9_Scaffold_438	17	p3	(AAT)5	15	45548	45562	potentially variable
PHG9_Scaffold_438	20	p3	(ATA)6	18	50044	50061	potentially variable
PHG9_Scaffold_438	28	p2	(TA)6	12	64240	64251	
PHG9_Scaffold_438	32	p2	(TA)9	18	75935	75952	potentially variable
PHG9_Scaffold_438	34	p2	(TA)6	12	83436	83447	
PHG9_Scaffold_438	37	p3	(TTA)5	15	91632	91646	potentially variable
PHG9_Scaffold_438	47	p3	(AAT)5	15	107251	107265	potentially variable
PHG9_Scaffold_442	2	p2	(TA)8	16	4081	4096	potentially variable
PHG9_Scaffold_442	8	p2	(TA)8	16	19743	19758	potentially variable
PHG9_Scaffold_442	19	p2	(AT)8	16	59132	59147	potentially variable
PHG9_Scaffold_444	3	p3	(CTC)5	15	10717	10731	potentially variable
PHG9_Scaffold_445	1	p2	(AG)8	16	2411	2426	potentially variable
PHG9_Scaffold_446	1	p2	(TA)8	16	393	408	potentially variable
PHG9_Scaffold_446	4	p2	(AT)6	12	13638	13649	
PHG9_Scaffold_446	10	p2	(AT)6	12	50248	50259	
PHG9_Scaffold_449	14	p2	(GT)9	18	67556	67573	potentially variable
PHG9_Scaffold_449	17	p2	(AT)6	12	75105	75116	

PHG9_Scaffold_449	19	p2	(TA)6	12	75725	75736	
PHG9_Scaffold_449	20	p2	(TA)6	12	76487	76498	
PHG9_Scaffold_450	1	p2	(TA)8	16	1896	1911	potentially variable
PHG9_Scaffold_450	6	p2	(TA)6	12	53493	53504	
PHG9_Scaffold_450	9	p2	(TA)6	12	60629	60640	
PHG9_Scaffold_450	16	p2	(AT)7	14	80946	80959	potentially variable
PHG9_Scaffold_450	18	p2	(AT)9	18	90529	90546	potentially variable
PHG9_Scaffold_451	1	p2	(TC)7	14	3399	3412	potentially variable
PHG9_Scaffold_452	7	p2	(AT)8	16	47804	47819	potentially variable
PHG9_Scaffold_452	9	p2	(TA)6	12	77863	77874	
PHG9_Scaffold_453	1	p2	(AT)7	14	598	611	potentially variable
PHG9_Scaffold_453	4	p3	(CAT)5	15	3941	3955	potentially variable
PHG9_Scaffold_453	5	p3	(AAT)5	15	6665	6679	potentially variable
PHG9_Scaffold_453	9	p3	(TAT)5	15	27324	27338	potentially variable
PHG9_Scaffold_453	12	p2	(AT)8	16	35679	35694	potentially variable
PHG9_Scaffold_454	12	p2	(AT)7	14	21260	21273	potentially variable
PHG9_Scaffold_454	21	p2	(AT)7	14	51500	51513	potentially variable
PHG9_Scaffold_454	23	p2	(AT)7	14	55804	55817	potentially

							variable
PHG9_Scaffold_454	24	p2	(TA)9	18	57087	57104	potentially variable
PHG9_Scaffold_454	25	p2	(TA)6	12	57667	57678	
PHG9_Scaffold_454	29	p3	(TTA)5	15	67182	67196	potentially variable
PHG9_Scaffold_455	17	p3	(TTA)5	15	57637	57651	potentially variable
PHG9_Scaffold_455	18	p2	(AT)6	12	63760	63771	
PHG9_Scaffold_455	30	p3	(GTT)5	15	102223	102237	potentially variable
PHG9_Scaffold_455	33	p2	(AT)6	12	111086	111097	
PHG9_Scaffold_455	38	p3	(GTT)6	18	122064	122081	potentially variable
PHG9_Scaffold_455	45	p2	(AT)6	12	137342	137353	
PHG9_Scaffold_455	46	p2	(AG)6	12	137836	137847	
PHG9_Scaffold_456	1	p2	(AT)8	16	29871	29886	potentially variable
PHG9_Scaffold_456	3	p2	(AT)6	12	60893	60904	
PHG9_Scaffold_456	5	p2	(CT)6	12	81818	81829	
PHG9_Scaffold_457	4	p2	(TA)8	16	10446	10461	potentially variable
PHG9_Scaffold_457	8	p2	(AC)9	18	15871	15888	potentially variable
PHG9_Scaffold_457	9	p2	(TA)6	12	34162	34173	
PHG9_Scaffold_457	11	p2	(TC)8	16	44302	44317	potentially variable
PHG9_Scaffold_457	22	p2	(TC)7	14	80114	80127	potentially variable

PHG9_Scaffold_457	25	p3	(CAA)5	15	84943	84957	potentially variable
PHG9_Scaffold_457	31	p2	(CT)8	16	108899	108914	potentially variable
PHG9_Scaffold_460	6	p2	(CA)8	16	14200	14215	potentially variable
PHG9_Scaffold_460	8	p2	(AC)6	12	22832	22843	
PHG9_Scaffold_460	9	p2	(CA)8	16	34104	34119	potentially variable
PHG9_Scaffold_460	13	p2	(AT)6	12	47739	47750	
PHG9_Scaffold_460	14	p3	(AAG)5	15	61261	61275	potentially variable
PHG9_Scaffold_460	16	p2	(TC)6	12	69677	69688	
PHG9_Scaffold_460	17	p2	(AT)7	14	72917	72930	potentially variable
PHG9_Scaffold_460	30	p2	(CT)9	18	113220	113237	potentially variable
PHG9_Scaffold_461	1	p3	(AAT)5	15	1829	1843	potentially variable
PHG9_Scaffold_461	12	p3	(TGA)5	15	57543	57557	potentially variable
PHG9_Scaffold_461	18	p2	(TA)8	16	83634	83649	potentially variable
PHG9_Scaffold_461	19	p2	(AT)8	16	86977	86992	potentially variable
PHG9_Scaffold_461	29	p2	(AT)8	16	137165	137180	potentially variable
PHG9_Scaffold_461	30	p2	(AT)6	12	138147	138158	
PHG9_Scaffold_461	31	p2	(CA)6	12	142461	142472	
PHG9_Scaffold_461	34	p2	(TA)9	18	145955	145972	potentially

							variable
PHG9_Scaffold_461	35	p3	(TTC)5	15	146261	146275	potentially variable
PHG9_Scaffold_462	1	p3	(AAT)5	15	346	360	potentially variable
PHG9_Scaffold_463	7	p2	(TA)7	14	10868	10881	potentially variable
PHG9_Scaffold_463	8	p2	(GA)6	12	11142	11153	
PHG9_Scaffold_463	12	p3	(TCT)5	15	17807	17821	potentially variable
PHG9_Scaffold_463	20	p2	(TA)6	12	34895	34906	
PHG9_Scaffold_463	21	p3	(CGC)5	15	41024	41038	potentially variable
PHG9_Scaffold_463	23	p2	(AT)8	16	46719	46734	potentially variable
PHG9_Scaffold_464	1	p2	(TA)7	14	5511	5524	potentially variable
PHG9_Scaffold_465	10	p3	(ATT)6	18	41749	41766	potentially variable
PHG9_Scaffold_465	11	p2	(AT)6	12	48207	48218	
PHG9_Scaffold_466	5	p2	(AG)7	14	25925	25938	potentially variable
PHG9_Scaffold_466	10	p2	(AT)6	12	43984	43995	
PHG9_Scaffold_466	14	p2	(AT)6	12	48022	48033	
PHG9_Scaffold_466	15	p3	(ATC)5	15	51671	51685	potentially variable
PHG9_Scaffold_466	17	p2	(GA)6	12	55137	55148	
PHG9_Scaffold_466	18	p2	(AT)6	12	55254	55265	
PHG9_Scaffold_466	23	p3	(AAT)5	15	65630	65644	potentially

							variable
PHG9_Scaffold_466	27	p6	(GTCTGC)5	30	75283	75312	Hypervariable
PHG9_Scaffold_467	15	p2	(AT)9	18	71438	71455	potentially variable
PHG9_Scaffold_467	23	p3	(TAT)5	15	112220	112234	potentially variable
PHG9_Scaffold_468	2	p2	(TA)9	18	4948	4965	potentially variable
PHG9_Scaffold_468	4	p2	(TC)6	12	10628	10639	
PHG9_Scaffold_468	13	p2	(TA)7	14	59066	59079	potentially variable
PHG9_Scaffold_468	14	p2	(AT)6	12	60076	60087	
PHG9_Scaffold_468	18	p2	(AG)9	18	73119	73136	potentially variable
PHG9_Scaffold_468	19	p3	(GAA)6	18	73789	73806	potentially variable
PHG9_Scaffold_473	3	p2	(AT)6	12	6223	6234	
PHG9_Scaffold_473	13	p2	(TC)6	12	48196	48207	
PHG9_Scaffold_473	20	p2	(TA)7	14	69441	69454	potentially variable
PHG9_Scaffold_473	21	p2	(AT)6	12	69902	69913	
PHG9_Scaffold_473	23	p2	(TA)6	12	72264	72275	
PHG9_Scaffold_473	24	p2	(AT)8	16	73479	73494	potentially variable
PHG9_Scaffold_473	26	p2	(AT)6	12	78676	78687	
PHG9_Scaffold_476	3	p2	(TA)6	12	11462	11473	
PHG9_Scaffold_476	4	p2	(AT)6	12	17341	17352	
PHG9_Scaffold_476	5	p2	(AG)6	12	19572	19583	
PHG9_Scaffold_476	6	p3	(CAC)5	15	20102	20116	potentially

							variable
PHG9_Scaffold_476	7	p3	(ATC)5	15	22279	22293	potentially variable
PHG9_Scaffold_476	12	p2	(AT)7	14	32419	32432	potentially variable
PHG9_Scaffold_476	23	p2	(TA)6	12	59761	59772	
PHG9_Scaffold_476	32	p2	(AT)8	16	83999	84014	potentially variable
PHG9_Scaffold_477	8	p2	(AT)6	12	25413	25424	
PHG9_Scaffold_477	10	p3	(AAT)6	18	27811	27828	potentially variable
PHG9_Scaffold_477	16	p2	(GA)7	14	41864	41877	potentially variable
PHG9_Scaffold_477	26	p2	(AT)7	14	85881	85894	potentially variable
PHG9_Scaffold_478	11	p2	(AT)7	14	40592	40605	potentially variable
PHG9_Scaffold_478	19	p2	(TA)9	18	93999	94016	potentially variable
PHG9_Scaffold_478	22	p2	(TG)7	14	102503	102516	potentially variable
PHG9_Scaffold_478	26	p3	(TAA)6	18	118896	118913	potentially variable
PHG9_Scaffold_478	27	p2	(TA)8	16	130477	130492	potentially variable
PHG9_Scaffold_478	28	p2	(AT)6	12	133992	134003	
PHG9_Scaffold_478	31	p2	(TA)8	16	153072	153087	potentially variable
PHG9_Scaffold_479	3	p2	(AT)8	16	1137	1152	potentially variable

PHG9_Scaffold_479	16	p3	(CAT)5	15	37922	37936	potentially variable
PHG9_Scaffold_479	18	p3	(CAT)6	18	52013	52030	potentially variable
PHG9_Scaffold_480	4	p3	(ATA)5	15	7808	7822	potentially variable
PHG9_Scaffold_480	9	p2	(CT)6	12	21107	21118	
PHG9_Scaffold_480	10	p2	(TC)7	14	25164	25177	potentially variable
PHG9_Scaffold_480	12	p2	(AT)6	12	29004	29015	
PHG9_Scaffold_480	14	p3	(TCT)5	15	32736	32750	potentially variable
PHG9_Scaffold_481	1	p2	(TA)7	14	6265	6278	potentially variable
PHG9_Scaffold_481	10	p3	(ATA)5	15	26834	26848	potentially variable
PHG9_Scaffold_481	13	p2	(TA)6	12	39209	39220	
PHG9_Scaffold_481	21	p2	(CA)7	14	59717	59730	potentially variable
PHG9_Scaffold_481	23	p3	(AAT)5	15	69254	69268	potentially variable
PHG9_Scaffold_482	6	p2	(AT)7	14	41643	41656	potentially variable
PHG9_Scaffold_482	8	p2	(TA)6	12	56753	56764	
PHG9_Scaffold_482	12	p2	(AT)9	18	90963	90980	potentially variable
PHG9_Scaffold_482	14	p2	(AT)6	12	102159	102170	
PHG9_Scaffold_482	17	p2	(AT)6	12	108505	108516	
PHG9_Scaffold_482	22	p2	(AT)7	14	124707	124720	potentially variable

PHG9_Scaffold_483	2	p3	(TAA)6	18	11610	11627	potentially variable
PHG9_Scaffold_483	5	p2	(AT)6	12	18194	18205	
PHG9_Scaffold_483	14	p3	(GGA)5	15	50333	50347	potentially variable
PHG9_Scaffold_483	15	p3	(AGA)5	15	50681	50695	potentially variable
PHG9_Scaffold_483	17	p2	(AT)9	18	58563	58580	potentially variable
PHG9_Scaffold_484	7	p3	(AAT)5	15	20406	20420	potentially variable
PHG9_Scaffold_484	16	p3	(ATA)5	15	64527	64541	potentially variable
PHG9_Scaffold_484	21	p2	(TA)7	14	76994	77007	potentially variable
PHG9_Scaffold_486	2	p2	(TA)6	12	6590	6601	
PHG9_Scaffold_486	7	p3	(AAT)5	15	13644	13658	potentially variable
PHG9_Scaffold_486	20	p2	(AT)7	14	49030	49043	potentially variable
PHG9_Scaffold_488	1	p2	(AG)6	12	13064	13075	
PHG9_Scaffold_488	3	p2	(CT)6	12	14884	14895	
PHG9_Scaffold_488	5	p2	(AT)7	14	51118	51131	potentially variable
PHG9_Scaffold_489	5	p2	(CT)7	14	9878	9891	potentially variable
PHG9_Scaffold_489	18	p2	(TA)6	12	34709	34720	
PHG9_Scaffold_489	30	p3	(AAC)5	15	57595	57609	potentially variable
PHG9_Scaffold_489	38	p3	(AAT)5	15	79042	79056	potentially

							variable
PHG9_Scaffold_489	39	p6	(AGATAT)5	30	84828	84857	Hypervariable
PHG9_Scaffold_490	1	p3	(GGA)5	15	6652	6666	potentially variable
PHG9_Scaffold_492	7	p2	(AT)6	12	16716	16727	
PHG9_Scaffold_492	8	p2	(AT)8	16	22172	22187	potentially variable
PHG9_Scaffold_492	11	p2	(GT)6	12	23277	23288	
PHG9_Scaffold_492	16	p2	(AT)7	14	40066	40079	potentially variable
PHG9_Scaffold_492	22	p2	(GA)7	14	46745	46758	potentially variable
PHG9_Scaffold_492	24	p2	(TA)6	12	51519	51530	
PHG9_Scaffold_492	35	p2	(AT)6	12	71690	71701	
PHG9_Scaffold_492	37	p2	(AT)6	12	76415	76426	
PHG9_Scaffold_492	42	p2	(AG)6	12	98079	98090	
PHG9_Scaffold_493	5	p2	(TA)6	12	49725	49736	
PHG9_Scaffold_493	18	p2	(GA)8	16	105682	105697	potentially variable
PHG9_Scaffold_494	5	p2	(TA)6	12	27824	27835	
PHG9_Scaffold_494	10	p2	(AT)6	12	55462	55473	
PHG9_Scaffold_494	12	p2	(TA)6	12	58611	58622	
PHG9_Scaffold_494	15	p2	(TA)9	18	71188	71205	potentially variable
PHG9_Scaffold_494	21	p2	(CA)6	12	92414	92425	
PHG9_Scaffold_494	22	p3	(TGT)5	15	93044	93058	potentially variable
PHG9_Scaffold_494	24	p3	(AAT)6	18	97922	97939	potentially variable

PHG9_Scaffold_495	1	p2	(AT)8	16	1141	1156	potentially variable
PHG9_Scaffold_495	6	p2	(AG)6	12	15977	15988	
PHG9_Scaffold_495	10	p2	(GA)6	12	22260	22271	
PHG9_Scaffold_495	11	p2	(TC)8	16	27046	27061	potentially variable
PHG9_Scaffold_495	13	p2	(AT)6	12	35429	35440	
PHG9_Scaffold_495	22	p2	(AT)6	12	53616	53627	
PHG9_Scaffold_495	23	p2	(TA)6	12	54710	54721	
PHG9_Scaffold_495	24	p2	(AT)8	16	55836	55851	potentially variable
PHG9_Scaffold_495	25	p3	(ATA)6	18	59943	59960	potentially variable
PHG9_Scaffold_495	28	p2	(AT)9	18	62761	62778	potentially variable
PHG9_Scaffold_495	30	p2	(AT)7	14	66033	66046	potentially variable
PHG9_Scaffold_496	5	p2	(AT)6	12	18921	18932	
PHG9_Scaffold_496	7	p3	(TAT)5	15	28806	28820	potentially variable
PHG9_Scaffold_496	8	p2	(TA)7	14	29903	29916	potentially variable
PHG9_Scaffold_496	9	p3	(TAT)5	15	32560	32574	potentially variable
PHG9_Scaffold_496	13	p3	(AAT)5	15	37748	37762	potentially variable
PHG9_Scaffold_496	33	p2	(AT)8	16	75362	75377	potentially variable
PHG9_Scaffold_497	2	p2	(TC)6	12	5334	5345	

PHG9_Scaffold_497	16	p3	(ATT)5	15	33071	33085	potentially variable
PHG9_Scaffold_497	24	p2	(TG)8	16	49333	49348	potentially variable
PHG9_Scaffold_497	27	p2	(AT)6	12	60973	60984	
PHG9_Scaffold_497	38	p2	(AG)6	12	93883	93894	
PHG9_Scaffold_497	39	p2	(CA)7	14	95076	95089	potentially variable
PHG9_Scaffold_497	49	p3	(TAT)5	15	126527	126541	potentially variable
PHG9_Scaffold_498	2	p2	(TA)6	12	6184	6195	
PHG9_Scaffold_498	4	p2	(AT)9	18	13696	13713	potentially variable
PHG9_Scaffold_498	8	p2	(TC)9	18	33029	33046	potentially variable
PHG9_Scaffold_498	13	p2	(AT)6	12	45761	45772	
PHG9_Scaffold_500	3	p2	(AT)7	14	2838	2851	potentially variable
PHG9_Scaffold_501	7	p2	(TC)6	12	43354	43365	
PHG9_Scaffold_504	20	p3	(TTA)5	15	53035	53049	potentially variable
PHG9_Scaffold_504	25	p2	(AT)6	12	57081	57092	
PHG9_Scaffold_504	27	p2	(TA)6	12	61172	61183	
PHG9_Scaffold_507	4	p2	(TA)9	18	15153	15170	potentially variable
PHG9_Scaffold_507	12	p2	(AT)6	12	38556	38567	
PHG9_Scaffold_507	15	p2	(AT)9	18	78981	78998	potentially variable
PHG9_Scaffold_507	16	p2	(AG)7	14	81444	81457	potentially

							variable
PHG9_Scaffold_507	27	p2	(TA)8	16	111119	111134	potentially variable
PHG9_Scaffold_507	28	p2	(AT)6	12	111286	111297	
PHG9_Scaffold_507	32	p2	(TA)6	12	122333	122344	
PHG9_Scaffold_509	1	p2	(AC)7	14	7849	7862	potentially variable
PHG9_Scaffold_509	2	p2	(AT)6	12	8887	8898	
PHG9_Scaffold_509	10	p2	(TA)7	14	73104	73117	potentially variable
PHG9_Scaffold_509	11	p2	(AT)6	12	79403	79414	
PHG9_Scaffold_509	14	p2	(TA)6	12	93667	93678	
PHG9_Scaffold_509	15	p2	(AT)8	16	118941	118956	potentially variable
PHG9_Scaffold_509	20	p2	(AT)8	16	160808	160823	potentially variable
PHG9_Scaffold_510	1	p3	(GAG)5	15	18271	18285	potentially variable
PHG9_Scaffold_511	24	p2	(TA)8	16	72230	72245	potentially variable
PHG9_Scaffold_511	27	p3	(TTA)5	15	77154	77168	potentially variable
PHG9_Scaffold_511	29	p2	(TA)6	12	82664	82675	
PHG9_Scaffold_511	30	p3	(ATT)5	15	83942	83956	potentially variable
PHG9_Scaffold_512	24	p3	(ATT)5	15	93623	93637	potentially variable
PHG9_Scaffold_513	3	p2	(TA)7	14	6037	6050	potentially variable

PHG9_Scaffold_513	7	p3	(ATA)5	15	9777	9791	potentially variable
PHG9_Scaffold_513	9	p3	(AAC)5	15	17895	17909	potentially variable
PHG9_Scaffold_513	14	p2	(AT)9	18	32389	32406	potentially variable
PHG9_Scaffold_513	21	p2	(TA)7	14	50215	50228	potentially variable
PHG9_Scaffold_514	3	p2	(TA)6	12	22241	22252	
PHG9_Scaffold_514	11	p3	(CCG)5	15	61668	61682	potentially variable
PHG9_Scaffold_514	14	p2	(AT)7	14	73093	73106	potentially variable
PHG9_Scaffold_514	18	p2	(TA)8	16	89439	89454	potentially variable
PHG9_Scaffold_514	20	p3	(TTG)5	15	97683	97697	potentially variable
PHG9_Scaffold_514	26	p2	(AT)9	18	116439	116456	potentially variable
PHG9_Scaffold_514	28	p2	(GA)7	14	118765	118778	potentially variable
PHG9_Scaffold_514	39	p2	(TA)6	12	144231	144242	
PHG9_Scaffold_514	41	p2	(TA)6	12	147037	147048	
PHG9_Scaffold_514	42	p2	(AT)9	18	147299	147316	potentially variable
PHG9_Scaffold_514	49	p3	(TAA)5	15	189845	189859	potentially variable
PHG9_Scaffold_514	62	p2	(TA)6	12	223233	223244	
PHG9_Scaffold_514	63	p2	(TA)8	16	227099	227114	potentially variable

PHG9_Scaffold_514	64	p2	(TA)9	18	228757	228774	potentially variable
PHG9_Scaffold_514	66	p3	(CAT)5	15	238010	238024	potentially variable
PHG9_Scaffold_514	69	p2	(TA)7	14	244073	244086	potentially variable
PHG9_Scaffold_514	72	p2	(AT)6	12	253579	253590	
PHG9_Scaffold_514	80	p2	(AT)6	12	272931	272942	
PHG9_Scaffold_514	82	p2	(CT)7	14	275329	275342	potentially variable
PHG9_Scaffold_514	85	p2	(AT)7	14	279610	279623	potentially variable
PHG9_Scaffold_514	87	p3	(ATC)5	15	282817	282831	potentially variable
PHG9_Scaffold_514	96	p2	(TA)9	18	296252	296269	potentially variable
PHG9_Scaffold_514	97	p2	(TA)6	12	296883	296894	
PHG9_Scaffold_514	101	p3	(GTG)5	15	309238	309252	potentially variable
PHG9_Scaffold_514	105	p2	(TA)6	12	319473	319484	
PHG9_Scaffold_515	4	p2	(CA)9	18	7890	7907	potentially variable
PHG9_Scaffold_515	7	p2	(TA)7	14	15881	15894	potentially variable
PHG9_Scaffold_515	14	p2	(AT)6	12	63325	63336	
PHG9_Scaffold_516	3	p3	(ACA)5	15	9601	9615	potentially variable
PHG9_Scaffold_516	9	p2	(AT)7	14	29945	29958	potentially variable
PHG9_Scaffold_516	12	p6	(AGTTTC)6	36	37610	37645	Hypervariable

PHG9_Scaffold_516	14	p2	(TA)7	14	40409	40422	potentially variable
PHG9_Scaffold_516	15	p2	(AT)8	16	41988	42003	potentially variable
PHG9_Scaffold_516	24	p3	(AGA)5	15	66373	66387	potentially variable
PHG9_Scaffold_517	14	p2	(CT)9	18	26960	26977	potentially variable
PHG9_Scaffold_517	18	p3	(ATT)5	15	42205	42219	potentially variable
PHG9_Scaffold_518	1	p3	(ATA)5	15	1544	1558	potentially variable
PHG9_Scaffold_518	5	p3	(CAC)5	15	6446	6460	potentially variable
PHG9_Scaffold_518	6	p2	(TA)9	18	7641	7658	potentially variable
PHG9_Scaffold_518	10	p2	(CA)7	14	15179	15192	potentially variable
PHG9_Scaffold_518	18	p3	(AAT)5	15	32487	32501	potentially variable
PHG9_Scaffold_518	22	p2	(TA)8	16	38006	38021	potentially variable
PHG9_Scaffold_518	29	p2	(AT)6	12	61964	61975	
PHG9_Scaffold_518	43	p3	(TAA)5	15	129832	129846	potentially variable
PHG9_Scaffold_518	44	p2	(AT)7	14	130534	130547	potentially variable
PHG9_Scaffold_518	45	p2	(GA)9	18	131720	131737	potentially variable
PHG9_Scaffold_518	53	p2	(TA)6	12	151719	151730	

PHG9_Scaffold_518	55	p2	(AT)7	14	156607	156620	potentially variable
PHG9_Scaffold_518	58	p2	(AT)8	16	160908	160923	potentially variable
PHG9_Scaffold_518	59	p2	(AT)8	16	161810	161825	potentially variable
PHG9_Scaffold_518	61	p2	(AG)8	16	167875	167890	potentially variable
PHG9_Scaffold_518	62	p2	(TA)6	12	171302	171313	
PHG9_Scaffold_519	5	p3	(TCA)5	15	32967	32981	potentially variable
PHG9_Scaffold_519	9	p2	(AT)6	12	48772	48783	
PHG9_Scaffold_519	20	p2	(AT)6	12	73288	73299	
PHG9_Scaffold_520	8	p2	(AT)6	12	21577	21588	
PHG9_Scaffold_520	10	p2	(AT)6	12	27428	27439	
PHG9_Scaffold_520	13	p3	(AGA)5	15	33824	33838	potentially variable
PHG9_Scaffold_520	14	p3	(CCA)5	15	34795	34809	potentially variable
PHG9_Scaffold_520	22	p2	(AT)6	12	66081	66092	
PHG9_Scaffold_520	23	p2	(AG)7	14	68134	68147	potentially variable
PHG9_Scaffold_520	24	p2	(TA)8	16	68597	68612	potentially variable
PHG9_Scaffold_521	3	p3	(GTG)5	15	2574	2588	potentially variable
PHG9_Scaffold_521	5	p2	(AT)7	14	4219	4232	potentially variable
PHG9_Scaffold_521	7	p2	(TA)6	12	7527	7538	

PHG9_Scaffold_521	9	p3	(AGC)6	18	10341	10358	potentially variable
PHG9_Scaffold_521	10	p2	(TA)9	18	16067	16084	potentially variable
PHG9_Scaffold_521	15	p2	(AG)6	12	46541	46552	
PHG9_Scaffold_524	4	p2	(AG)7	14	21935	21948	potentially variable
PHG9_Scaffold_524	20	p3	(AAC)5	15	92160	92174	potentially variable
PHG9_Scaffold_525	6	p2	(TA)6	12	31586	31597	
PHG9_Scaffold_526	6	p2	(TA)6	12	29109	29120	
PHG9_Scaffold_526	12	p2	(AT)6	12	51173	51184	
PHG9_Scaffold_526	13	p2	(TA)8	16	53489	53504	potentially variable
PHG9_Scaffold_528	2	p2	(TA)8	16	1863	1878	potentially variable
PHG9_Scaffold_528	8	p2	(AT)8	16	5339	5354	potentially variable
PHG9_Scaffold_528	12	p3	(GAA)5	15	42383	42397	potentially variable
PHG9_Scaffold_528	14	p3	(CTT)5	15	49872	49886	potentially variable
PHG9_Scaffold_528	19	p3	(TCG)5	15	87173	87187	potentially variable
PHG9_Scaffold_528	21	p2	(TA)6	12	138399	138410	
PHG9_Scaffold_528	26	p3	(ATT)5	15	186713	186727	potentially variable
PHG9_Scaffold_529	1	p2	(GT)6	12	2896	2907	
PHG9_Scaffold_530	2	p2	(TA)6	12	4491	4502	

PHG9_Scaffold_531	8	p2	(AT)8	16	25051	25066	potentially variable
PHG9_Scaffold_532	5	p2	(TA)7	14	16633	16646	potentially variable
PHG9_Scaffold_532	18	p2	(TA)7	14	52863	52876	potentially variable
PHG9_Scaffold_532	19	p3	(AAT)5	15	53064	53078	potentially variable
PHG9_Scaffold_532	25	p2	(TG)7	14	78418	78431	potentially variable
PHG9_Scaffold_532	28	p2	(AT)6	12	84316	84327	
PHG9_Scaffold_532	29	p3	(TTA)5	15	84607	84621	potentially variable
PHG9_Scaffold_532	46	p3	(TCT)5	15	133886	133900	potentially variable
PHG9_Scaffold_533	8	p2	(TA)7	14	35139	35152	potentially variable
PHG9_Scaffold_533	11	p2	(TA)6	12	50978	50989	
PHG9_Scaffold_533	14	p2	(TA)9	18	62858	62875	potentially variable
PHG9_Scaffold_535	6	p2	(AT)6	12	12118	12129	
PHG9_Scaffold_535	10	p2	(TG)6	12	24662	24673	
PHG9_Scaffold_535	14	p3	(TGC)5	15	34534	34548	potentially variable
PHG9_Scaffold_535	17	p2	(AT)7	14	42474	42487	potentially variable
PHG9_Scaffold_536	8	p3	(TAA)5	15	61540	61554	potentially variable
PHG9_Scaffold_537	14	p2	(AT)7	14	41960	41973	potentially variable

PHG9_Scaffold_537	21	p3	(ATA)5	15	67943	67957	potentially variable
PHG9_Scaffold_537	22	p2	(AT)6	12	69642	69653	
PHG9_Scaffold_538	2	p3	(AAC)5	15	972	986	potentially variable
PHG9_Scaffold_538	3	p2	(TC)6	12	5923	5934	
PHG9_Scaffold_538	14	p3	(TTA)5	15	59877	59891	potentially variable
PHG9_Scaffold_539	3	p2	(TA)6	12	8218	8229	
PHG9_Scaffold_539	5	p2	(AG)7	14	20073	20086	potentially variable
PHG9_Scaffold_539	10	p3	(TAA)5	15	43423	43437	potentially variable
PHG9_Scaffold_539	16	p3	(AAT)5	15	50883	50897	potentially variable
PHG9_Scaffold_539	17	p3	(TCT)5	15	51361	51375	potentially variable
PHG9_Scaffold_539	27	p2	(TA)6	12	61245	61256	
PHG9_Scaffold_540	6	p2	(TC)8	16	15097	15112	potentially variable
PHG9_Scaffold_540	7	p2	(AT)6	12	18398	18409	
PHG9_Scaffold_540	17	p2	(TA)7	14	36473	36486	potentially variable
PHG9_Scaffold_540	20	p2	(TA)6	12	38593	38604	
PHG9_Scaffold_540	22	p2	(TC)10	20	41871	41890	Hypervariable
PHG9_Scaffold_540	23	p2	(AT)6	12	42178	42189	
PHG9_Scaffold_540	26	p2	(AT)6	12	58383	58394	
PHG9_Scaffold_540	28	p2	(GA)8	16	61751	61766	potentially variable

PHG9_Scaffold_540	29	p2	(TC)8	16	62007	62022	potentially variable
PHG9_Scaffold_542	7	p2	(AG)7	14	17344	17357	potentially variable
PHG9_Scaffold_542	10	p2	(TC)8	16	35627	35642	potentially variable
PHG9_Scaffold_542	12	p2	(TG)6	12	40905	40916	
PHG9_Scaffold_542	14	p2	(TC)6	12	50037	50048	
PHG9_Scaffold_542	15	p2	(AC)6	12	51503	51514	
PHG9_Scaffold_542	28	p2	(TA)8	16	82267	82282	potentially variable
PHG9_Scaffold_542	38	p3	(CTT)5	15	107869	107883	potentially variable
PHG9_Scaffold_542	48	p2	(GA)6	12	137647	137658	
PHG9_Scaffold_542	55	p2	(TA)6	12	180914	180925	
PHG9_Scaffold_543	2	p3	(TTC)5	15	10137	10151	potentially variable
PHG9_Scaffold_543	4	p2	(CA)7	14	36913	36926	potentially variable
PHG9_Scaffold_543	5	p2	(AT)9	18	40821	40838	potentially variable
PHG9_Scaffold_544	1	p2	(TC)8	16	3165	3180	potentially variable
PHG9_Scaffold_544	10	p2	(TA)6	12	29672	29683	
PHG9_Scaffold_546	11	p2	(AT)9	18	31823	31840	potentially variable
PHG9_Scaffold_546	15	p3	(AGT)6	18	35613	35630	potentially variable
PHG9_Scaffold_549	4	p3	(TCA)5	15	16499	16513	potentially variable

PHG9_Scaffold_549	10	p3	(AAT)5	15	38182	38196	potentially variable
PHG9_Scaffold_549	13	p3	(TAA)6	18	48620	48637	potentially variable
PHG9_Scaffold_550	2	p2	(AG)8	16	4305	4320	potentially variable
PHG9_Scaffold_550	3	p2	(AT)7	14	4469	4482	potentially variable
PHG9_Scaffold_550	18	p2	(AT)6	12	37712	37723	
PHG9_Scaffold_550	26	p2	(GT)6	12	44514	44525	
PHG9_Scaffold_550	28	p3	(AAT)5	15	52699	52713	potentially variable
PHG9_Scaffold_551	1	p2	(TA)7	14	7130	7143	potentially variable
PHG9_Scaffold_551	9	p2	(TA)9	18	43954	43971	potentially variable
PHG9_Scaffold_551	10	p3	(TAT)5	15	46074	46088	potentially variable
PHG9_Scaffold_552	10	p2	(CT)6	12	33750	33761	
PHG9_Scaffold_553	1	p2	(TA)7	14	2376	2389	potentially variable
PHG9_Scaffold_553	3	p2	(CT)6	12	14343	14354	
PHG9_Scaffold_553	7	p2	(GT)6	12	35775	35786	
PHG9_Scaffold_553	11	p2	(AT)6	12	80313	80324	
PHG9_Scaffold_553	13	p3	(AGA)5	15	115891	115905	potentially variable
PHG9_Scaffold_554	3	p2	(TA)7	14	18807	18820	potentially variable
PHG9_Scaffold_554	4	p2	(TC)7	14	19747	19760	potentially variable

PHG9_Scaffold_555	5	p2	(AT)6	12	37385	37396	
PHG9_Scaffold_556	2	p2	(GA)7	14	1022	1035	potentially variable
PHG9_Scaffold_556	4	p2	(AT)6	12	1893	1904	
PHG9_Scaffold_556	7	p3	(TTA)6	18	13708	13725	potentially variable
PHG9_Scaffold_556	15	p3	(GCG)5	15	31867	31881	potentially variable
PHG9_Scaffold_559	13	p3	(AGT)5	15	30320	30334	potentially variable
PHG9_Scaffold_559	18	p2	(AT)6	12	51095	51106	
PHG9_Scaffold_561	2	p3	(TAA)6	18	3179	3196	potentially variable
PHG9_Scaffold_561	3	p3	(TTA)6	18	6856	6873	potentially variable
PHG9_Scaffold_562	2	p2	(AT)6	12	2458	2469	
PHG9_Scaffold_562	4	p3	(TTC)6	18	18077	18094	potentially variable
PHG9_Scaffold_562	9	p2	(GT)9	18	26527	26544	potentially variable
PHG9_Scaffold_562	10	p2	(TC)7	14	26735	26748	potentially variable
PHG9_Scaffold_562	12	p2	(AT)6	12	34155	34166	
PHG9_Scaffold_562	16	p2	(AC)8	16	63380	63395	potentially variable
PHG9_Scaffold_562	20	p2	(AG)9	18	76445	76462	potentially variable
PHG9_Scaffold_562	28	p2	(AG)9	18	101117	101134	potentially variable
PHG9_Scaffold_563	9	p2	(TC)6	12	35070	35081	

PHG9_Scaffold_563	24	p2	(TA)9	18	69478	69495	potentially variable
PHG9_Scaffold_563	25	p2	(AG)7	14	71435	71448	potentially variable
PHG9_Scaffold_563	29	p2	(AT)9	18	85001	85018	potentially variable
PHG9_Scaffold_563	31	p2	(AT)9	18	86845	86862	potentially variable
PHG9_Scaffold_564	3	p2	(AT)9	18	8066	8083	potentially variable
PHG9_Scaffold_564	12	p2	(TC)6	12	45726	45737	
PHG9_Scaffold_564	16	p2	(AT)6	12	67593	67604	
PHG9_Scaffold_564	17	p3	(AAT)5	15	69365	69379	potentially variable
PHG9_Scaffold_564	20	p2	(TA)6	12	93771	93782	
PHG9_Scaffold_565	2	p2	(TA)6	12	26102	26113	
PHG9_Scaffold_565	3	p2	(AT)7	14	26528	26541	potentially variable
PHG9_Scaffold_565	10	p2	(TA)6	12	82279	82290	
PHG9_Scaffold_565	11	p2	(AT)6	12	82572	82583	
PHG9_Scaffold_565	22	p2	(GA)6	12	194602	194613	
PHG9_Scaffold_566	1	p3	(GGA)5	15	444	458	potentially variable
PHG9_Scaffold_566	6	p2	(TA)8	16	13959	13974	potentially variable
PHG9_Scaffold_566	12	p3	(TAT)5	15	27935	27949	potentially variable
PHG9_Scaffold_566	21	p2	(CA)7	14	83086	83099	potentially variable

PHG9_Scaffold_566	30	p2	(AT)6	12	107897	107908	
PHG9_Scaffold_566	36	p3	(GTT)6	18	126412	126429	potentially variable
PHG9_Scaffold_566	39	p3	(TAA)6	18	132041	132058	potentially variable
PHG9_Scaffold_566	49	p2	(AT)8	16	168547	168562	potentially variable
PHG9_Scaffold_566	52	p3	(GAA)5	15	182898	182912	potentially variable
PHG9_Scaffold_566	54	p2	(AT)9	18	183829	183846	potentially variable
PHG9_Scaffold_566	58	p3	(TTA)5	15	198296	198310	potentially variable
PHG9_Scaffold_566	61	p2	(AG)6	12	210894	210905	
PHG9_Scaffold_566	71	p2	(TA)8	16	233296	233311	potentially variable
PHG9_Scaffold_566	74	p3	(ATA)5	15	236713	236727	potentially variable
PHG9_Scaffold_566	82	p3	(AAG)5	15	240478	240492	potentially variable
PHG9_Scaffold_566	95	p3	(CTT)5	15	264305	264319	potentially variable
PHG9_Scaffold_566	109	p3	(TAA)5	15	300022	300036	potentially variable
PHG9_Scaffold_566	117	p3	(ATT)5	15	328496	328510	potentially variable
PHG9_Scaffold_566	123	p2	(TA)7	14	341738	341751	potentially variable
PHG9_Scaffold_567	2	p3	(AAG)5	15	2855	2869	potentially variable

PHG9_Scaffold_567	4	p2	(GA)9	18	6935	6952	potentially variable
PHG9_Scaffold_569	12	p2	(TA)7	14	29151	29164	potentially variable
PHG9_Scaffold_569	19	p2	(TA)6	12	43476	43487	
PHG9_Scaffold_569	20	p2	(CT)8	16	45675	45690	potentially variable
PHG9_Scaffold_569	22	p2	(TA)8	16	50764	50779	potentially variable
PHG9_Scaffold_571	2	p2	(AT)9	18	34406	34423	potentially variable
PHG9_Scaffold_572	1	p2	(AT)6	12	1343	1354	
PHG9_Scaffold_575	10	p2	(AT)9	18	20660	20677	potentially variable
PHG9_Scaffold_575	22	p3	(GTT)5	15	70070	70084	potentially variable
PHG9_Scaffold_575	26	p2	(TA)8	16	75516	75531	potentially variable
PHG9_Scaffold_575	28	p3	(TTC)5	15	84308	84322	potentially variable
PHG9_Scaffold_576	5	p2	(TA)6	12	38851	38862	
PHG9_Scaffold_578	17	p3	(CTC)5	15	97103	97117	potentially variable
PHG9_Scaffold_579	4	p2	(TA)7	14	16317	16330	potentially variable
PHG9_Scaffold_579	10	p2	(AT)8	16	41871	41886	potentially variable
PHG9_Scaffold_579	17	p2	(AT)7	14	58312	58325	potentially variable
PHG9_Scaffold_579	18	p3	(ATT)5	15	60262	60276	potentially

							variable
PHG9_Scaffold_580	4	p2	(TA)6	12	1620	1631	
PHG9_Scaffold_580	6	p2	(GA)7	14	5078	5091	potentially variable
PHG9_Scaffold_580	7	p2	(TA)10	20	5450	5469	Hypervariable
PHG9_Scaffold_580	15	p2	(AT)9	18	54270	54287	potentially variable
PHG9_Scaffold_580	18	p2	(AT)6	12	58732	58743	
PHG9_Scaffold_582	2	p2	(AT)9	18	2389	2406	potentially variable
PHG9_Scaffold_583	4	p2	(AT)8	16	5454	5469	potentially variable
PHG9_Scaffold_583	24	p2	(TA)9	18	57974	57991	potentially variable
PHG9_Scaffold_583	28	p2	(AT)7	14	62553	62566	potentially variable
PHG9_Scaffold_583	29	p2	(AG)7	14	63666	63679	potentially variable
PHG9_Scaffold_583	37	p2	(GA)8	16	77648	77663	potentially variable
PHG9_Scaffold_584	17	p3	(ATA)5	15	58350	58364	potentially variable
PHG9_Scaffold_584	38	p2	(TA)8	16	106306	106321	potentially variable
PHG9_Scaffold_584	39	p2	(AT)6	12	107361	107372	
PHG9_Scaffold_584	40	p3	(AAT)5	15	111641	111655	potentially variable
PHG9_Scaffold_584	44	p2	(TA)6	12	125932	125943	
PHG9_Scaffold_584	53	p2	(AT)7	14	141184	141197	potentially variable

PHG9_Scaffold_584	63	p2	(AT)7	14	177726	177739	potentially variable
PHG9_Scaffold_587	9	p2	(TA)7	14	34475	34488	potentially variable
PHG9_Scaffold_587	21	p3	(TAT)5	15	63507	63521	potentially variable
PHG9_Scaffold_588	1	p2	(GA)9	18	1306	1323	potentially variable
PHG9_Scaffold_588	2	p2	(GA)9	18	3392	3409	potentially variable
PHG9_Scaffold_588	14	p2	(TA)8	16	33309	33324	potentially variable
PHG9_Scaffold_588	19	p2	(AT)6	12	44879	44890	
PHG9_Scaffold_588	27	p3	(AGA)5	15	56823	56837	potentially variable
PHG9_Scaffold_588	31	p2	(GA)9	18	73183	73200	potentially variable
PHG9_Scaffold_588	32	p2	(AT)7	14	75435	75448	potentially variable
PHG9_Scaffold_588	43	p2	(CT)6	12	130481	130492	
PHG9_Scaffold_588	47	p2	(TA)7	14	137993	138006	potentially variable
PHG9_Scaffold_588	55	p2	(AG)6	12	153550	153561	
PHG9_Scaffold_588	57	p3	(ACC)6	18	154119	154136	potentially variable
PHG9_Scaffold_588	58	p2	(TA)6	12	157745	157756	
PHG9_Scaffold_588	59	p2	(TA)6	12	158195	158206	
PHG9_Scaffold_588	60	p2	(AT)6	12	158481	158492	
PHG9_Scaffold_588	67	p6	(ATATGA)5	30	182832	182861	Hypervariable

PHG9_Scaffold_588	69	p2	(TG)7	14	190837	190850	potentially variable
PHG9_Scaffold_588	76	p3	(AAC)5	15	213836	213850	potentially variable
PHG9_Scaffold_588	80	p2	(AT)6	12	216851	216862	
PHG9_Scaffold_588	81	p3	(GGA)5	15	218114	218128	potentially variable
PHG9_Scaffold_590	4	p2	(CT)8	16	6822	6837	potentially variable
PHG9_Scaffold_590	9	p2	(TA)7	14	14170	14183	potentially variable
PHG9_Scaffold_590	10	p2	(CT)9	18	18365	18382	potentially variable
PHG9_Scaffold_590	12	p2	(AC)8	16	24260	24275	potentially variable
PHG9_Scaffold_590	19	p3	(ATA)5	15	43338	43352	potentially variable
PHG9_Scaffold_590	24	p3	(AAT)5	15	54523	54537	potentially variable
PHG9_Scaffold_590	29	p2	(TA)7	14	60288	60301	potentially variable
PHG9_Scaffold_590	56	p3	(TTG)5	15	134494	134508	potentially variable
PHG9_Scaffold_590	65	p3	(TAT)5	15	168823	168837	potentially variable
PHG9_Scaffold_590	69	p3	(AAC)5	15	175247	175261	potentially variable
PHG9_Scaffold_590	71	p2	(TC)6	12	178568	178579	
PHG9_Scaffold_590	76	p2	(AG)9	18	194658	194675	potentially variable

PHG9_Scaffold_590	79	p2	(TG)6	12	210763	210774	
PHG9_Scaffold_593	2	p2	(TA)6	12	3524	3535	
PHG9_Scaffold_593	5	p2	(AT)6	12	9465	9476	
PHG9_Scaffold_593	19	p2	(CT)7	14	35984	35997	potentially variable
PHG9_Scaffold_593	22	p3	(TGA)5	15	46230	46244	potentially variable
PHG9_Scaffold_593	26	p2	(TA)7	14	54614	54627	potentially variable
PHG9_Scaffold_594	2	p2	(TA)7	14	8107	8120	potentially variable
PHG9_Scaffold_594	10	p2	(AT)6	12	32388	32399	
PHG9_Scaffold_594	11	p3	(TCA)5	15	33902	33916	potentially variable
PHG9_Scaffold_595	1	p3	(TCT)5	15	1012	1026	potentially variable
PHG9_Scaffold_595	2	p2	(AC)7	14	1258	1271	potentially variable
PHG9_Scaffold_595	3	p3	(AAG)5	15	2119	2133	potentially variable
PHG9_Scaffold_595	4	p2	(AG)8	16	4029	4044	potentially variable
PHG9_Scaffold_595	8	p2	(TA)9	18	17623	17640	potentially variable
PHG9_Scaffold_595	9	p2	(TA)9	18	18290	18307	potentially variable
PHG9_Scaffold_595	18	p3	(TAA)5	15	40312	40326	potentially variable
PHG9_Scaffold_595	23	p2	(TA)6	12	45855	45866	
PHG9_Scaffold_596	3	p2	(GT)8	16	10062	10077	potentially

							variable
PHG9_Scaffold_596	20	p2	(AT)6	12	53408	53419	
PHG9_Scaffold_597	3	p2	(AT)8	16	3621	3636	potentially variable
PHG9_Scaffold_597	4	p3	(TAT)5	15	3893	3907	potentially variable
PHG9_Scaffold_597	5	p2	(AT)8	16	6003	6018	potentially variable
PHG9_Scaffold_597	15	p2	(AT)9	18	45471	45488	potentially variable
PHG9_Scaffold_597	17	p2	(AT)7	14	64438	64451	potentially variable
PHG9_Scaffold_597	23	p2	(AT)7	14	77744	77757	potentially variable
PHG9_Scaffold_597	25	p2	(TA)6	12	81826	81837	
PHG9_Scaffold_601	5	p2	(AT)9	18	33774	33791	potentially variable
PHG9_Scaffold_602	9	p2	(TA)6	12	41527	41538	
PHG9_Scaffold_603	2	p2	(TC)7	14	2700	2713	potentially variable
PHG9_Scaffold_603	6	p2	(AT)7	14	16441	16454	potentially variable
PHG9_Scaffold_603	11	p2	(TC)6	12	33896	33907	
PHG9_Scaffold_603	17	p3	(AGA)5	15	62343	62357	potentially variable
PHG9_Scaffold_603	18	p3	(TAA)5	15	65359	65373	potentially variable
PHG9_Scaffold_603	19	p3	(CAT)6	18	66252	66269	potentially variable
PHG9_Scaffold_603	20	p2	(AT)9	18	70908	70925	potentially

							variable
PHG9_Scaffold_603	21	p2	(TC)8	16	74236	74251	potentially variable
PHG9_Scaffold_603	25	p2	(TA)6	12	79088	79099	
PHG9_Scaffold_603	29	p2	(AT)7	14	98120	98133	potentially variable
PHG9_Scaffold_603	31	p3	(TAT)5	15	102152	102166	potentially variable
PHG9_Scaffold_603	36	p2	(AG)6	12	108146	108157	
PHG9_Scaffold_603	41	p2	(TA)6	12	126829	126840	
PHG9_Scaffold_603	53	p3	(TAA)5	15	149866	149880	potentially variable
PHG9_Scaffold_603	57	p2	(GT)6	12	166652	166663	
PHG9_Scaffold_603	61	p2	(AG)6	12	184014	184025	
PHG9_Scaffold_603	66	p3	(GTT)5	15	193831	193845	potentially variable
PHG9_Scaffold_603	71	p2	(AT)7	14	207909	207922	potentially variable
PHG9_Scaffold_603	72	p2	(GT)7	14	209302	209315	potentially variable
PHG9_Scaffold_603	73	p3	(GTG)5	15	210652	210666	potentially variable
PHG9_Scaffold_603	74	p2	(AT)6	12	215306	215317	
PHG9_Scaffold_603	79	p3	(ATT)5	15	219258	219272	potentially variable
PHG9_Scaffold_603	84	p3	(TAA)5	15	231574	231588	potentially variable
PHG9_Scaffold_603	89	p2	(AC)7	14	239599	239612	potentially variable

PHG9_Scaffold_603	92	p2	(TA)6	12	250181	250192	
PHG9_Scaffold_604	4	p3	(ATT)5	15	10532	10546	potentially variable
PHG9_Scaffold_604	6	p2	(TA)9	18	12905	12922	potentially variable
PHG9_Scaffold_604	7	p2	(AT)6	12	21162	21173	
PHG9_Scaffold_605	1	p2	(TA)6	12	210	221	
PHG9_Scaffold_605	2	p2	(TA)8	16	10837	10852	potentially variable
PHG9_Scaffold_605	3	p2	(AT)6	12	11015	11026	
PHG9_Scaffold_605	8	p3	(GTG)5	15	21779	21793	potentially variable
PHG9_Scaffold_605	10	p2	(AC)7	14	22586	22599	potentially variable
PHG9_Scaffold_605	20	p2	(AT)8	16	41347	41362	potentially variable
PHG9_Scaffold_605	24	p2	(AT)6	12	58527	58538	
PHG9_Scaffold_605	29	p3	(TAT)6	18	81472	81489	potentially variable
PHG9_Scaffold_605	31	p2	(TA)7	14	97837	97850	potentially variable
PHG9_Scaffold_606	8	p2	(TC)7	14	24814	24827	potentially variable
PHG9_Scaffold_608	2	p3	(GAT)5	15	2100	2114	potentially variable
PHG9_Scaffold_608	3	p3	(TTA)5	15	2265	2279	potentially variable
PHG9_Scaffold_609	4	p2	(TA)7	14	10096	10109	potentially variable
PHG9_Scaffold_609	18	p2	(TA)6	12	39986	39997	

PHG9_Scaffold_609	23	p2	(CT)6	12	46576	46587	
PHG9_Scaffold_609	24	p2	(CT)6	12	51330	51341	
PHG9_Scaffold_609	44	p3	(ATA)5	15	117659	117673	potentially variable
PHG9_Scaffold_609	58	p2	(AT)8	16	140444	140459	potentially variable
PHG9_Scaffold_609	59	p2	(AT)7	14	141866	141879	potentially variable
PHG9_Scaffold_609	62	p2	(TA)6	12	153867	153878	
PHG9_Scaffold_609	64	p2	(TG)7	14	156051	156064	potentially variable
PHG9_Scaffold_612	1	p2	(AG)6	12	2911	2922	
PHG9_Scaffold_613	2	p2	(TA)6	12	1776	1787	
PHG9_Scaffold_616	1	p2	(CT)6	12	222	233	
PHG9_Scaffold_616	2	p2	(TC)7	14	4421	4434	potentially variable
PHG9_Scaffold_616	7	p2	(AT)7	14	38594	38607	potentially variable
PHG9_Scaffold_616	8	p2	(AT)7	14	38796	38809	potentially variable
PHG9_Scaffold_616	10	p2	(AT)7	14	55402	55415	potentially variable
PHG9_Scaffold_616	11	p2	(AT)9	18	58688	58705	potentially variable
PHG9_Scaffold_617	5	p2	(TA)6	12	18684	18695	
PHG9_Scaffold_617	8	p2	(TA)6	12	22274	22285	
PHG9_Scaffold_617	9	p2	(AT)9	18	26488	26505	potentially variable
PHG9_Scaffold_617	11	p2	(AC)8	16	31029	31044	potentially

							variable
PHG9_Scaffold_617	12	p2	(GA)6	12	31249	31260	
PHG9_Scaffold_617	13	p2	(TC)7	14	31378	31391	potentially variable
PHG9_Scaffold_617	14	p3	(CAG)5	15	32021	32035	potentially variable
PHG9_Scaffold_617	15	p3	(CAC)5	15	33759	33773	potentially variable
PHG9_Scaffold_617	19	p2	(AT)9	18	43985	44002	potentially variable
PHG9_Scaffold_617	22	p3	(CAA)5	15	51457	51471	potentially variable
PHG9_Scaffold_617	24	p2	(CT)6	12	52192	52203	
PHG9_Scaffold_619	1	p2	(TC)6	12	1450	1461	
PHG9_Scaffold_620	3	p2	(AT)6	12	8949	8960	
PHG9_Scaffold_620	6	p2	(TA)6	12	14827	14838	
PHG9_Scaffold_620	7	p2	(AT)8	16	15783	15798	potentially variable
PHG9_Scaffold_620	21	p2	(AT)8	16	52968	52983	potentially variable
PHG9_Scaffold_620	36	p2	(GA)6	12	97510	97521	
PHG9_Scaffold_622	17	p2	(TA)6	12	53895	53906	
PHG9_Scaffold_622	20	p2	(TA)9	18	63150	63167	potentially variable
PHG9_Scaffold_624	2	p2	(CT)6	12	2064	2075	
PHG9_Scaffold_624	4	p2	(AG)6	12	14105	14116	
PHG9_Scaffold_624	7	p3	(GCA)5	15	17973	17987	potentially variable
PHG9_Scaffold_624	10	p2	(GA)6	12	28028	28039	

PHG9_Scaffold_624	13	p2	(AT)7	14	36061	36074	potentially variable
PHG9_Scaffold_624	18	p2	(AG)7	14	55849	55862	potentially variable
PHG9_Scaffold_624	23	p2	(AG)6	12	70300	70311	
PHG9_Scaffold_624	31	p2	(AT)8	16	88953	88968	potentially variable
PHG9_Scaffold_624	32	p3	(AAT)5	15	93080	93094	potentially variable
PHG9_Scaffold_624	33	p2	(TA)6	12	93321	93332	
PHG9_Scaffold_624	40	p2	(AT)7	14	122115	122128	potentially variable
PHG9_Scaffold_624	45	p2	(TA)7	14	139521	139534	potentially variable
PHG9_Scaffold_624	49	p2	(AT)7	14	142985	142998	potentially variable
PHG9_Scaffold_624	54	p3	(GGT)5	15	149161	149175	potentially variable
PHG9_Scaffold_624	55	p2	(GT)7	14	151996	152009	potentially variable
PHG9_Scaffold_624	56	p3	(GTG)5	15	153652	153666	potentially variable
PHG9_Scaffold_624	59	p2	(AG)6	12	157605	157616	
PHG9_Scaffold_624	62	p2	(TA)7	14	162480	162493	potentially variable
PHG9_Scaffold_624	76	p2	(GT)6	12	186905	186916	
PHG9_Scaffold_624	89	p2	(AT)6	12	212235	212246	
PHG9_Scaffold_624	95	p3	(AAT)5	15	220510	220524	potentially variable
PHG9_Scaffold_625	7	p2	(TC)9	18	33604	33621	potentially

							variable
PHG9_Scaffold_625	10	p2	(AT)8	16	34646	34661	potentially variable
PHG9_Scaffold_625	13	p2	(TA)6	12	42954	42965	
PHG9_Scaffold_626	8	p2	(AT)6	12	38755	38766	
PHG9_Scaffold_626	9	p2	(AT)7	14	42032	42045	potentially variable
PHG9_Scaffold_626	10	p2	(TA)8	16	51306	51321	potentially variable
PHG9_Scaffold_626	12	p2	(TA)6	12	90478	90489	
PHG9_Scaffold_626	19	p2	(AT)6	12	135647	135658	
PHG9_Scaffold_626	22	p2	(TA)6	12	160706	160717	
PHG9_Scaffold_626	23	p3	(GGT)5	15	167683	167697	potentially variable
PHG9_Scaffold_627	7	p2	(TG)6	12	27777	27788	
PHG9_Scaffold_627	14	p3	(AGA)5	15	62154	62168	potentially variable
PHG9_Scaffold_627	17	p3	(TAA)5	15	71205	71219	potentially variable
PHG9_Scaffold_627	18	p2	(TA)6	12	73014	73025	
PHG9_Scaffold_627	19	p3	(AAT)5	15	75820	75834	potentially variable
PHG9_Scaffold_627	21	p2	(AC)6	12	76467	76478	
PHG9_Scaffold_628	1	p2	(CT)6	12	7779	7790	
PHG9_Scaffold_628	2	p2	(AT)8	16	9617	9632	potentially variable
PHG9_Scaffold_628	6	p2	(TA)8	16	17582	17597	potentially variable
PHG9_Scaffold_628	9	p2	(AG)6	12	28508	28519	

PHG9_Scaffold_628	12	p2	(TC)9	18	38103	38120	potentially variable
PHG9_Scaffold_628	16	p2	(TG)8	16	55232	55247	potentially variable
PHG9_Scaffold_628	25	p2	(AG)6	12	102668	102679	
PHG9_Scaffold_628	27	p2	(AT)6	12	107960	107971	
PHG9_Scaffold_629	1	p2	(TA)7	14	790	803	potentially variable
PHG9_Scaffold_629	2	p2	(TA)7	14	1460	1473	potentially variable
PHG9_Scaffold_629	3	p2	(TA)7	14	2838	2851	potentially variable
PHG9_Scaffold_629	4	p2	(TA)6	12	3729	3740	
PHG9_Scaffold_629	5	p2	(AT)7	14	5420	5433	potentially variable
PHG9_Scaffold_629	6	p2	(TA)6	12	5885	5896	
PHG9_Scaffold_629	7	p2	(AT)7	14	6145	6158	potentially variable
PHG9_Scaffold_629	8	p2	(TG)8	16	8412	8427	potentially variable
PHG9_Scaffold_629	9	p2	(TA)9	18	13206	13223	potentially variable
PHG9_Scaffold_629	14	p2	(CT)6	12	39517	39528	
PHG9_Scaffold_629	15	p2	(TA)9	18	41623	41640	potentially variable
PHG9_Scaffold_630	2	p2	(CA)8	16	15066	15081	potentially variable
PHG9_Scaffold_630	3	p3	(TAA)6	18	25039	25056	potentially variable
PHG9_Scaffold_630	4	p2	(AT)7	14	25294	25307	potentially

							variable
PHG9_Scaffold_630	6	p6	(CCATGC)6	36	34711	34746	Hypervariable
PHG9_Scaffold_630	28	p3	(CAC)5	15	130598	130612	potentially variable
PHG9_Scaffold_630	31	p2	(TC)6	12	148533	148544	
PHG9_Scaffold_632	1	p2	(TA)7	14	1729	1742	potentially variable
PHG9_Scaffold_632	2	p2	(TA)9	18	2077	2094	potentially variable
PHG9_Scaffold_632	3	p2	(TA)8	16	2342	2357	potentially variable
PHG9_Scaffold_632	9	p2	(TA)8	16	39172	39187	potentially variable
PHG9_Scaffold_632	15	p2	(TA)9	18	45811	45828	potentially variable
PHG9_Scaffold_632	22	p2	(CT)9	18	60512	60529	potentially variable
PHG9_Scaffold_632	29	p2	(TA)6	12	79939	79950	
PHG9_Scaffold_632	32	p2	(GT)6	12	84682	84693	
PHG9_Scaffold_632	35	p3	(AAG)5	15	87490	87504	potentially variable
PHG9_Scaffold_632	36	p3	(TCT)5	15	97957	97971	potentially variable
PHG9_Scaffold_632	38	p3	(ATA)5	15	108610	108624	potentially variable
PHG9_Scaffold_632	41	p2	(CT)7	14	113006	113019	potentially variable
PHG9_Scaffold_634	3	p2	(AT)9	18	1960	1977	potentially variable
PHG9_Scaffold_634	11	p6	(TGAATT)5	30	40192	40221	Hypervariable

PHG9_Scaffold_634	41	p3	(ATA)5	15	182737	182751	potentially variable
PHG9_Scaffold_634	42	p2	(AC)6	12	211390	211401	
PHG9_Scaffold_635	19	p2	(GT)8	16	78629	78644	potentially variable
PHG9_Scaffold_636	7	p2	(TA)8	16	63160	63175	potentially variable
PHG9_Scaffold_636	10	p2	(TA)6	12	108685	108696	
PHG9_Scaffold_637	2	p3	(TAA)5	15	5531	5545	potentially variable
PHG9_Scaffold_637	5	p2	(GA)7	14	10009	10022	potentially variable
PHG9_Scaffold_637	7	p2	(AT)6	12	14779	14790	
PHG9_Scaffold_637	8	p2	(TG)7	14	21929	21942	potentially variable
PHG9_Scaffold_637	13	p2	(TA)7	14	42922	42935	potentially variable
PHG9_Scaffold_637	15	p2	(AC)9	18	51630	51647	potentially variable
PHG9_Scaffold_637	19	p2	(TA)7	14	71583	71596	potentially variable
PHG9_Scaffold_638	1	p3	(AAC)5	15	3361	3375	potentially variable
PHG9_Scaffold_638	2	p3	(CGG)5	15	3541	3555	potentially variable
PHG9_Scaffold_638	7	p2	(TA)8	16	8041	8056	potentially variable
PHG9_Scaffold_638	13	p2	(TA)6	12	36896	36907	
PHG9_Scaffold_639	11	p3	(ATA)5	15	26376	26390	potentially variable

PHG9_Scaffold_639	19	p2	(AG)6	12	88157	88168	
PHG9_Scaffold_640	1	p3	(TTA)5	15	4756	4770	potentially variable
PHG9_Scaffold_640	8	p3	(AAT)6	18	69225	69242	potentially variable
PHG9_Scaffold_641	5	p2	(AG)6	12	12734	12745	
PHG9_Scaffold_641	12	p2	(CT)9	18	23320	23337	potentially variable
PHG9_Scaffold_641	13	p2	(CA)6	12	24065	24076	
PHG9_Scaffold_642	2	p2	(AT)8	16	4806	4821	potentially variable
PHG9_Scaffold_643	2	p2	(CT)8	16	6625	6640	potentially variable
PHG9_Scaffold_643	3	p2	(CT)6	12	9481	9492	
PHG9_Scaffold_643	11	p2	(AG)9	18	32527	32544	potentially variable
PHG9_Scaffold_643	12	p2	(TA)8	16	45096	45111	potentially variable
PHG9_Scaffold_643	37	p3	(GAT)5	15	160773	160787	potentially variable
PHG9_Scaffold_643	41	p2	(GA)6	12	170550	170561	
PHG9_Scaffold_643	43	p2	(TA)6	12	178805	178816	
PHG9_Scaffold_645	2	p2	(TA)6	12	410	421	
PHG9_Scaffold_645	4	p2	(AT)8	16	2585	2600	potentially variable
PHG9_Scaffold_645	8	p2	(TA)7	14	19056	19069	potentially variable
PHG9_Scaffold_647	7	p2	(AT)7	14	27213	27226	potentially variable

PHG9_Scaffold_647	11	p2	(AT)6	12	36794	36805	
PHG9_Scaffold_647	13	p2	(TA)6	12	49286	49297	
PHG9_Scaffold_647	14	p2	(GA)6	12	49661	49672	
PHG9_Scaffold_648	1	p3	(ATC)5	15	3607	3621	potentially variable
PHG9_Scaffold_648	10	p2	(AT)6	12	32231	32242	
PHG9_Scaffold_648	16	p2	(TG)6	12	57583	57594	
PHG9_Scaffold_648	17	p3	(AAT)5	15	58616	58630	potentially variable
PHG9_Scaffold_651	3	p2	(CT)9	18	5247	5264	potentially variable
PHG9_Scaffold_651	5	p3	(ACA)5	15	6531	6545	potentially variable
PHG9_Scaffold_652	3	p2	(AT)9	18	17763	17780	potentially variable
PHG9_Scaffold_652	10	p2	(AG)7	14	52821	52834	potentially variable
PHG9_Scaffold_653	1	p2	(AT)8	16	1145	1160	potentially variable
PHG9_Scaffold_653	5	p2	(TA)9	18	29229	29246	potentially variable
PHG9_Scaffold_653	12	p3	(GAA)5	15	52860	52874	potentially variable
PHG9_Scaffold_653	14	p2	(AT)8	16	56653	56668	potentially variable
PHG9_Scaffold_653	16	p2	(GA)6	12	76694	76705	
PHG9_Scaffold_654	1	p2	(TA)6	12	1507	1518	
PHG9_Scaffold_654	3	p2	(AT)7	14	17651	17664	potentially variable

PHG9_Scaffold_654	4	p3	(ATA)5	15	20587	20601	potentially variable
PHG9_Scaffold_654	16	p2	(AT)9	18	62613	62630	potentially variable
PHG9_Scaffold_654	17	p2	(AT)6	12	62848	62859	
PHG9_Scaffold_654	19	p6	(TTGGTT)5	30	66511	66540	Hypervariable
PHG9_Scaffold_654	26	p3	(GAA)6	18	84571	84588	potentially variable
PHG9_Scaffold_655	1	p2	(TA)7	14	2613	2626	potentially variable
PHG9_Scaffold_655	6	p2	(AT)9	18	5450	5467	potentially variable
PHG9_Scaffold_655	12	p2	(AT)6	12	27166	27177	
PHG9_Scaffold_655	21	p3	(TTC)6	18	55660	55677	potentially variable
PHG9_Scaffold_655	24	p2	(AT)8	16	58472	58487	potentially variable
PHG9_Scaffold_656	5	p3	(TTA)5	15	11733	11747	potentially variable
PHG9_Scaffold_657	2	p2	(AT)8	16	10437	10452	potentially variable
PHG9_Scaffold_657	5	p2	(TA)6	12	15299	15310	
PHG9_Scaffold_657	7	p3	(CCA)6	18	20627	20644	potentially variable
PHG9_Scaffold_657	8	p2	(TA)7	14	21214	21227	potentially variable
PHG9_Scaffold_657	9	p2	(TA)7	14	22093	22106	potentially variable
PHG9_Scaffold_658	13	p3	(TTA)5	15	27225	27239	potentially variable

PHG9_Scaffold_659	4	p2	(TA)6	12	16937	16948	
PHG9_Scaffold_659	8	p2	(TA)6	12	29436	29447	
PHG9_Scaffold_659	9	p2	(AT)8	16	31347	31362	potentially variable
PHG9_Scaffold_659	13	p2	(AT)6	12	40590	40601	
PHG9_Scaffold_659	19	p2	(AT)6	12	58340	58351	
PHG9_Scaffold_660	2	p2	(AT)9	18	6598	6615	potentially variable
PHG9_Scaffold_660	3	p2	(AT)9	18	27465	27482	potentially variable
PHG9_Scaffold_661	1	p2	(TA)8	16	253	268	potentially variable
PHG9_Scaffold_663	1	p3	(ATA)5	15	2017	2031	potentially variable
PHG9_Scaffold_663	20	p2	(AT)6	12	60009	60020	
PHG9_Scaffold_663	21	p2	(AT)6	12	60402	60413	
PHG9_Scaffold_663	23	p3	(ATT)5	15	61868	61882	potentially variable
PHG9_Scaffold_664	9	p2	(AT)9	18	23309	23326	potentially variable
PHG9_Scaffold_664	11	p3	(TGA)5	15	27507	27521	potentially variable
PHG9_Scaffold_664	25	p2	(TA)6	12	58738	58749	
PHG9_Scaffold_664	30	p2	(TA)6	12	79072	79083	
PHG9_Scaffold_666	18	p2	(TA)6	12	47309	47320	
PHG9_Scaffold_666	22	p2	(TA)6	12	56211	56222	
PHG9_Scaffold_666	24	p3	(ATT)5	15	60129	60143	potentially variable
PHG9_Scaffold_666	28	p2	(TA)6	12	86115	86126	

PHG9_Scaffold_669	3	p3	(AAG)5	15	13080	13094	potentially variable
PHG9_Scaffold_670	2	p2	(AT)9	18	3721	3738	potentially variable
PHG9_Scaffold_670	9	p2	(TC)6	12	29194	29205	
PHG9_Scaffold_673	2	p2	(TA)7	14	4703	4716	potentially variable
PHG9_Scaffold_673	3	p2	(AT)9	18	6234	6251	potentially variable
PHG9_Scaffold_673	4	p3	(ATA)5	15	14755	14769	potentially variable
PHG9_Scaffold_673	7	p2	(AT)8	16	17894	17909	potentially variable
PHG9_Scaffold_673	8	p2	(AC)7	14	19390	19403	potentially variable
PHG9_Scaffold_674	3	p2	(TA)6	12	20260	20271	
PHG9_Scaffold_675	14	p2	(TA)6	12	33958	33969	
PHG9_Scaffold_675	31	p2	(AT)8	16	90129	90144	potentially variable
PHG9_Scaffold_675	33	p3	(ATT)6	18	104699	104716	potentially variable
PHG9_Scaffold_675	47	p2	(TA)6	12	183820	183831	
PHG9_Scaffold_677	1	p2	(AG)6	12	918	929	
PHG9_Scaffold_677	9	p2	(TA)6	12	16804	16815	
PHG9_Scaffold_677	10	p3	(TAT)5	15	17238	17252	potentially variable
PHG9_Scaffold_677	14	p2	(AT)6	12	32712	32723	
PHG9_Scaffold_677	15	p2	(AT)8	16	34585	34600	potentially variable

PHG9_Scaffold_678	1	p2	(TA)6	12	6277	6288	
PHG9_Scaffold_679	2	p2	(AT)6	12	2149	2160	
PHG9_Scaffold_680	1	p2	(AT)6	12	5161	5172	
PHG9_Scaffold_687	4	p2	(TA)8	16	13879	13894	potentially variable
PHG9_Scaffold_687	11	p2	(AC)6	12	37023	37034	
PHG9_Scaffold_687	19	p2	(TC)6	12	72446	72457	
PHG9_Scaffold_688	11	p2	(AT)7	14	31391	31404	potentially variable
PHG9_Scaffold_688	21	p2	(TA)7	14	67290	67303	potentially variable
PHG9_Scaffold_688	27	p3	(TTC)5	15	80721	80735	potentially variable
PHG9_Scaffold_688	38	p2	(AG)8	16	113610	113625	potentially variable
PHG9_Scaffold_688	46	p2	(TG)6	12	146737	146748	
PHG9_Scaffold_688	52	p6	(ACACAA)5	30	175693	175722	Hypervariable
PHG9_Scaffold_688	58	p2	(AT)8	16	200630	200645	potentially variable
PHG9_Scaffold_688	59	p2	(TC)7	14	203521	203534	potentially variable
PHG9_Scaffold_688	61	p2	(GA)6	12	212817	212828	
PHG9_Scaffold_688	63	p2	(CT)6	12	213329	213340	
PHG9_Scaffold_688	68	p3	(AGA)6	18	216521	216538	potentially variable
PHG9_Scaffold_688	69	p2	(TC)9	18	217437	217454	potentially variable
PHG9_Scaffold_689	1	p2	(TA)6	12	314	325	
PHG9_Scaffold_689	9	p2	(AT)6	12	38874	38885	

PHG9_Scaffold_689	12	p3	(TTC)5	15	45704	45718	potentially variable
PHG9_Scaffold_689	16	p3	(TTC)5	15	52214	52228	potentially variable
PHG9_Scaffold_689	20	p3	(ATG)5	15	61687	61701	potentially variable
PHG9_Scaffold_689	24	p3	(TTA)5	15	87697	87711	potentially variable
PHG9_Scaffold_689	27	p3	(TTA)5	15	100424	100438	potentially variable
PHG9_Scaffold_689	32	p2	(AT)9	18	108614	108631	potentially variable
PHG9_Scaffold_689	34	p2	(AT)6	12	118764	118775	
PHG9_Scaffold_689	36	p3	(TAA)5	15	123466	123480	potentially variable
PHG9_Scaffold_691	3	p2	(AT)7	14	13948	13961	potentially variable
PHG9_Scaffold_691	4	p2	(TA)7	14	16073	16086	potentially variable
PHG9_Scaffold_691	6	p2	(AG)6	12	19823	19834	
PHG9_Scaffold_691	8	p3	(AAT)5	15	21623	21637	potentially variable
PHG9_Scaffold_692	9	p2	(TC)6	12	41029	41040	
PHG9_Scaffold_692	11	p3	(GGC)5	15	47807	47821	potentially variable
PHG9_Scaffold_693	3	p3	(CAC)5	15	8380	8394	potentially variable
PHG9_Scaffold_693	12	p3	(GGT)5	15	26935	26949	potentially variable
PHG9_Scaffold_693	17	p2	(GA)6	12	51542	51553	

PHG9_Scaffold_693	19	p2	(AT)7	14	58651	58664	potentially variable
PHG9_Scaffold_693	20	p2	(AT)6	12	60571	60582	
PHG9_Scaffold_693	23	p2	(GT)9	18	66480	66497	potentially variable
PHG9_Scaffold_693	24	p2	(TA)6	12	70196	70207	
PHG9_Scaffold_693	27	p2	(TC)7	14	83741	83754	potentially variable
PHG9_Scaffold_694	4	p2	(TA)9	18	5800	5817	potentially variable
PHG9_Scaffold_695	1	p2	(AT)6	12	1212	1223	
PHG9_Scaffold_695	6	p3	(ATT)5	15	29241	29255	potentially variable
PHG9_Scaffold_695	10	p2	(AT)9	18	58163	58180	potentially variable
PHG9_Scaffold_695	13	p6	(CTCCAT)6	36	71832	71867	Hypervariable
PHG9_Scaffold_695	14	p2	(AC)9	18	73073	73090	potentially variable
PHG9_Scaffold_695	15	p2	(TA)8	16	78546	78561	potentially variable
PHG9_Scaffold_695	20	p2	(TA)6	12	93080	93091	
PHG9_Scaffold_695	25	p2	(TA)7	14	107118	107131	potentially variable
PHG9_Scaffold_695	26	p2	(TA)9	18	107403	107420	potentially variable
PHG9_Scaffold_697	4	p3	(ATC)5	15	10091	10105	potentially variable
PHG9_Scaffold_697	10	p2	(AT)8	16	49551	49566	potentially variable
PHG9_Scaffold_700	1	p2	(TA)6	12	2985	2996	

PHG9_Scaffold_700	4	p3	(TTG)5	15	11546	11560	potentially variable
PHG9_Scaffold_700	6	p3	(TAA)5	15	15447	15461	potentially variable
PHG9_Scaffold_700	7	p2	(TA)6	12	20937	20948	
PHG9_Scaffold_700	27	p2	(TA)8	16	72923	72938	potentially variable
PHG9_Scaffold_700	34	p2	(TA)7	14	80470	80483	potentially variable
PHG9_Scaffold_700	40	p2	(AT)8	16	87416	87431	potentially variable
PHG9_Scaffold_700	41	p2	(AT)7	14	88637	88650	potentially variable
PHG9_Scaffold_700	44	p2	(TA)6	12	97216	97227	
PHG9_Scaffold_700	45	p2	(AT)6	12	99439	99450	
PHG9_Scaffold_700	48	p2	(AT)6	12	106686	106697	
PHG9_Scaffold_702	4	p2	(TA)7	14	8047	8060	potentially variable
PHG9_Scaffold_702	7	p2	(AT)8	16	16000	16015	potentially variable
PHG9_Scaffold_702	15	p3	(CAT)5	15	42542	42556	potentially variable
PHG9_Scaffold_703	2	p3	(TTG)5	15	1527	1541	potentially variable
PHG9_Scaffold_703	4	p2	(GT)6	12	6692	6703	
PHG9_Scaffold_703	10	p2	(AT)6	12	26674	26685	
PHG9_Scaffold_703	18	p2	(AT)7	14	52367	52380	potentially variable
PHG9_Scaffold_704	7	p2	(TC)9	18	61805	61822	potentially variable

PHG9_Scaffold_705	13	p3	(ATT)5	15	28825	28839	potentially variable
PHG9_Scaffold_705	17	p2	(TA)6	12	34697	34708	
PHG9_Scaffold_705	18	p3	(AAT)6	18	37390	37407	potentially variable
PHG9_Scaffold_705	23	p2	(AT)9	18	43989	44006	potentially variable
PHG9_Scaffold_705	24	p2	(AT)8	16	46449	46464	potentially variable
PHG9_Scaffold_705	25	p3	(AAT)5	15	46710	46724	potentially variable
PHG9_Scaffold_705	26	p2	(AT)7	14	56120	56133	potentially variable
PHG9_Scaffold_705	27	p2	(AT)6	12	58930	58941	
PHG9_Scaffold_705	30	p3	(AGA)5	15	66236	66250	potentially variable
PHG9_Scaffold_706	5	p2	(TA)7	14	23421	23434	potentially variable
PHG9_Scaffold_707	2	p2	(AT)8	16	23411	23426	potentially variable
PHG9_Scaffold_707	9	p2	(AT)6	12	48279	48290	
PHG9_Scaffold_708	1	p3	(GAT)5	15	36670	36684	potentially variable
PHG9_Scaffold_709	1	p2	(TA)6	12	3551	3562	
PHG9_Scaffold_709	3	p3	(CAA)5	15	17437	17451	potentially variable
PHG9_Scaffold_709	7	p2	(AT)6	12	28466	28477	
PHG9_Scaffold_709	9	p2	(TA)6	12	35594	35605	
PHG9_Scaffold_709	14	p2	(TA)6	12	49686	49697	

PHG9_Scaffold_709	20	p3	(TAT)6	18	69377	69394	potentially variable
PHG9_Scaffold_710	2	p2	(TA)8	16	4818	4833	potentially variable
PHG9_Scaffold_710	6	p3	(TCA)6	18	10460	10477	potentially variable
PHG9_Scaffold_710	7	p2	(TA)6	12	11094	11105	
PHG9_Scaffold_710	8	p2	(TA)8	16	11270	11285	potentially variable
PHG9_Scaffold_710	22	p2	(CT)6	12	53325	53336	
PHG9_Scaffold_711	5	p2	(AT)7	14	16553	16566	potentially variable
PHG9_Scaffold_712	16	p3	(AGA)5	15	58134	58148	potentially variable
PHG9_Scaffold_713	10	p3	(ACT)5	15	49673	49687	potentially variable
PHG9_Scaffold_714	1	p2	(AT)6	12	1541	1552	
PHG9_Scaffold_715	4	p2	(TA)7	14	38233	38246	potentially variable
PHG9_Scaffold_715	6	p2	(AT)6	12	49742	49753	
PHG9_Scaffold_716	3	p2	(TA)6	12	5093	5104	
PHG9_Scaffold_716	5	p2	(AT)7	14	7562	7575	potentially variable
PHG9_Scaffold_716	13	p3	(AAT)6	18	28014	28031	potentially variable
PHG9_Scaffold_716	17	p2	(AT)7	14	33965	33978	potentially variable
PHG9_Scaffold_716	18	p2	(TA)8	16	56225	56240	potentially variable
PHG9_Scaffold_716	20	p2	(GT)8	16	65528	65543	potentially

							variable
PHG9_Scaffold_716	21	p2	(TA)6	12	80504	80515	
PHG9_Scaffold_716	25	p3	(TTA)5	15	91771	91785	potentially variable
PHG9_Scaffold_716	26	p2	(AT)6	12	96230	96241	
PHG9_Scaffold_717	1	p2	(TA)7	14	14449	14462	potentially variable
PHG9_Scaffold_717	5	p2	(AT)6	12	20736	20747	
PHG9_Scaffold_717	6	p2	(AT)9	18	22065	22082	potentially variable
PHG9_Scaffold_718	4	p2	(TA)7	14	5908	5921	potentially variable
PHG9_Scaffold_718	11	p3	(AGA)5	15	25092	25106	potentially variable
PHG9_Scaffold_719	17	p3	(GAT)6	18	64047	64064	potentially variable
PHG9_Scaffold_721	3	p2	(AT)9	18	41358	41375	potentially variable
PHG9_Scaffold_722	1	p2	(TA)6	12	105901	105912	
PHG9_Scaffold_723	3	p3	(CGC)5	15	5311	5325	potentially variable
PHG9_Scaffold_724	3	p2	(TG)7	14	13649	13662	potentially variable
PHG9_Scaffold_724	8	p3	(TTA)5	15	32929	32943	potentially variable
PHG9_Scaffold_724	13	p3	(ATA)5	15	40026	40040	potentially variable
PHG9_Scaffold_724	14	p2	(AT)8	16	41073	41088	potentially variable
PHG9_Scaffold_724	15	p2	(TA)6	12	41607	41618	

PHG9_Scaffold_729	6	p2	(CT)9	18	60832	60849	potentially variable
PHG9_Scaffold_729	9	p3	(TTA)5	15	69476	69490	potentially variable
PHG9_Scaffold_730	7	p3	(AAT)5	15	11867	11881	potentially variable
PHG9_Scaffold_730	11	p2	(GT)6	12	29114	29125	
PHG9_Scaffold_730	18	p2	(TA)7	14	46659	46672	potentially variable
PHG9_Scaffold_730	20	p3	(ATG)6	18	47678	47695	potentially variable
PHG9_Scaffold_730	22	p2	(AT)7	14	59669	59682	potentially variable
PHG9_Scaffold_730	28	p2	(TA)7	14	84754	84767	potentially variable
PHG9_Scaffold_730	29	p2	(AT)8	16	89409	89424	potentially variable
PHG9_Scaffold_730	34	p3	(AAT)5	15	95665	95679	potentially variable
PHG9_Scaffold_730	37	p2	(AT)8	16	116152	116167	potentially variable
PHG9_Scaffold_730	38	p3	(TAT)5	15	124153	124167	potentially variable
PHG9_Scaffold_730	47	p2	(AG)6	12	178583	178594	
PHG9_Scaffold_730	49	p2	(TA)6	12	182643	182654	
PHG9_Scaffold_731	5	p2	(AT)8	16	23304	23319	potentially variable
PHG9_Scaffold_731	7	p2	(TA)6	12	43858	43869	
PHG9_Scaffold_731	12	p3	(ATA)5	15	72184	72198	potentially variable

PHG9_Scaffold_732	6	p3	(AAT)6	18	16949	16966	potentially variable
PHG9_Scaffold_733	2	p3	(TAA)5	15	51862	51876	potentially variable
PHG9_Scaffold_734	6	p2	(TA)6	12	15059	15070	
PHG9_Scaffold_735	2	p2	(TA)6	12	960	971	
PHG9_Scaffold_735	4	p2	(AG)7	14	21442	21455	potentially variable
PHG9_Scaffold_735	8	p2	(AG)6	12	33580	33591	
PHG9_Scaffold_735	16	p2	(TA)7	14	52239	52252	potentially variable
PHG9_Scaffold_735	17	p2	(AG)6	12	52912	52923	
PHG9_Scaffold_735	19	p3	(ACA)5	15	57649	57663	potentially variable
PHG9_Scaffold_735	21	p2	(AG)8	16	63350	63365	potentially variable
PHG9_Scaffold_735	26	p2	(TA)6	12	70911	70922	
PHG9_Scaffold_735	28	p2	(TA)6	12	72971	72982	
PHG9_Scaffold_736	3	p3	(TAA)5	15	11765	11779	potentially variable
PHG9_Scaffold_736	4	p2	(TC)7	14	13181	13194	potentially variable
PHG9_Scaffold_736	8	p3	(TTC)5	15	19438	19452	potentially variable
PHG9_Scaffold_736	9	p2	(AT)9	18	20928	20945	potentially variable
PHG9_Scaffold_736	15	p2	(AT)7	14	43783	43796	potentially variable
PHG9_Scaffold_736	17	p3	(TAT)5	15	47480	47494	potentially variable

PHG9_Scaffold_736	21	p3	(GAT)5	15	59363	59377	potentially variable
PHG9_Scaffold_736	32	p2	(AG)8	16	88820	88835	potentially variable
PHG9_Scaffold_736	43	p2	(TA)6	12	107773	107784	
PHG9_Scaffold_736	60	p2	(AT)6	12	166887	166898	
PHG9_Scaffold_736	64	p2	(TA)7	14	173922	173935	potentially variable
PHG9_Scaffold_736	69	p2	(TA)6	12	183780	183791	
PHG9_Scaffold_736	70	p3	(ATT)5	15	184703	184717	potentially variable
PHG9_Scaffold_736	73	p3	(TTA)5	15	195243	195257	potentially variable
PHG9_Scaffold_736	105	p3	(ATT)5	15	307942	307956	potentially variable
PHG9_Scaffold_736	111	p2	(TA)8	16	317634	317649	potentially variable
PHG9_Scaffold_736	119	p2	(TA)6	12	335546	335557	
PHG9_Scaffold_738	1	p2	(AG)9	18	9487	9504	potentially variable
PHG9_Scaffold_738	2	p2	(AT)8	16	9975	9990	potentially variable
PHG9_Scaffold_738	4	p2	(TA)9	18	11101	11118	potentially variable
PHG9_Scaffold_738	7	p2	(TA)7	14	31365	31378	potentially variable
PHG9_Scaffold_738	8	p2	(AT)6	12	32303	32314	
PHG9_Scaffold_739	1	p3	(TTA)5	15	652	666	potentially variable
PHG9_Scaffold_739	2	p2	(TG)6	12	8515	8526	

PHG9_Scaffold_739	8	p2	(TA)6	12	17921	17932	
PHG9_Scaffold_739	15	p2	(TA)7	14	38124	38137	potentially variable
PHG9_Scaffold_739	16	p2	(TA)7	14	38938	38951	potentially variable
PHG9_Scaffold_739	18	p2	(AT)9	18	39367	39384	potentially variable
PHG9_Scaffold_739	22	p2	(CT)8	16	42169	42184	potentially variable
PHG9_Scaffold_741	2	p3	(TTG)5	15	3933	3947	potentially variable
PHG9_Scaffold_741	6	p2	(AT)6	12	14152	14163	
PHG9_Scaffold_741	8	p2	(TA)6	12	15542	15553	
PHG9_Scaffold_741	12	p2	(TA)7	14	29094	29107	potentially variable
PHG9_Scaffold_741	16	p2	(GT)6	12	39294	39305	
PHG9_Scaffold_741	22	p3	(AAT)6	18	60142	60159	potentially variable
PHG9_Scaffold_741	23	p2	(AG)7	14	60860	60873	potentially variable
PHG9_Scaffold_741	31	p2	(TA)6	12	105610	105621	
PHG9_Scaffold_748	8	p2	(TC)9	18	29105	29122	potentially variable
PHG9_Scaffold_749	11	p2	(TA)7	14	48721	48734	potentially variable
PHG9_Scaffold_749	12	p2	(TC)7	14	49723	49736	potentially variable
PHG9_Scaffold_749	14	p2	(TA)7	14	56601	56614	potentially variable
PHG9_Scaffold_749	17	p3	(AAT)5	15	61487	61501	potentially

							variable
PHG9_Scaffold_750	2	p2	(TA)7	14	3092	3105	potentially variable
PHG9_Scaffold_750	15	p2	(AT)7	14	28096	28109	potentially variable
PHG9_Scaffold_750	22	p2	(AT)6	12	58163	58174	
PHG9_Scaffold_750	24	p3	(AAT)5	15	61920	61934	potentially variable
PHG9_Scaffold_751	7	p2	(AT)6	12	38026	38037	
PHG9_Scaffold_752	7	p2	(TA)6	12	56161	56172	
PHG9_Scaffold_753	2	p2	(AT)6	12	12300	12311	
PHG9_Scaffold_753	4	p2	(TC)9	18	53337	53354	potentially variable
PHG9_Scaffold_754	3	p3	(TAT)5	15	10175	10189	potentially variable
PHG9_Scaffold_754	7	p3	(TAA)5	15	31381	31395	potentially variable
PHG9_Scaffold_754	21	p3	(TTA)5	15	65046	65060	potentially variable
PHG9_Scaffold_754	23	p2	(AT)6	12	69040	69051	
PHG9_Scaffold_754	26	p2	(TA)6	12	76103	76114	
PHG9_Scaffold_754	28	p3	(TGG)6	18	88021	88038	potentially variable
PHG9_Scaffold_755	23	p3	(TTC)5	15	70684	70698	potentially variable
PHG9_Scaffold_756	1	p2	(AT)7	14	302	315	potentially variable
PHG9_Scaffold_756	9	p2	(TA)6	12	65391	65402	
PHG9_Scaffold_756	19	p2	(TA)8	16	116750	116765	potentially

							variable
PHG9_Scaffold_756	26	p2	(AT)9	18	134792	134809	potentially variable
PHG9_Scaffold_756	33	p2	(GA)7	14	146076	146089	potentially variable
PHG9_Scaffold_756	45	p2	(TC)7	14	176090	176103	potentially variable
PHG9_Scaffold_756	46	p3	(CAA)5	15	177724	177738	potentially variable
PHG9_Scaffold_757	1	p3	(ATA)6	18	1994	2011	potentially variable
PHG9_Scaffold_757	7	p2	(AT)6	12	20943	20954	
PHG9_Scaffold_757	8	p2	(TA)7	14	27237	27250	potentially variable
PHG9_Scaffold_757	9	p3	(CCT)5	15	29971	29985	potentially variable
PHG9_Scaffold_757	13	p3	(AAT)6	18	33917	33934	potentially variable
PHG9_Scaffold_757	15	p3	(TCA)5	15	36517	36531	potentially variable
PHG9_Scaffold_757	19	p2	(TA)6	12	58955	58966	
PHG9_Scaffold_758	1	p2	(CT)8	16	2226	2241	potentially variable
PHG9_Scaffold_758	3	p2	(TA)6	12	7036	7047	
PHG9_Scaffold_758	7	p3	(TCT)5	15	40067	40081	potentially variable
PHG9_Scaffold_758	9	p2	(CT)6	12	49007	49018	
PHG9_Scaffold_758	10	p2	(TG)6	12	57635	57646	
PHG9_Scaffold_758	11	p2	(GT)6	12	57910	57921	

PHG9_Scaffold_758	12	p3	(TTA)5	15	60083	60097	potentially variable
PHG9_Scaffold_758	19	p3	(GAA)5	15	78041	78055	potentially variable
PHG9_Scaffold_759	17	p2	(AT)8	16	69232	69247	potentially variable
PHG9_Scaffold_759	21	p2	(AT)7	14	81456	81469	potentially variable
PHG9_Scaffold_759	33	p2	(TA)9	18	121006	121023	potentially variable
PHG9_Scaffold_759	39	p3	(AAT)5	15	131753	131767	potentially variable
PHG9_Scaffold_759	42	p3	(CAG)5	15	135331	135345	potentially variable
PHG9_Scaffold_759	43	p2	(CT)7	14	139553	139566	potentially variable
PHG9_Scaffold_759	46	p2	(CA)6	12	143484	143495	
PHG9_Scaffold_759	49	p3	(AAT)6	18	165838	165855	potentially variable
PHG9_Scaffold_761	12	p2	(AT)9	18	17751	17768	potentially variable
PHG9_Scaffold_762	12	p2	(AT)8	16	55048	55063	potentially variable
PHG9_Scaffold_763	1	p2	(TA)6	12	4079	4090	
PHG9_Scaffold_763	2	p3	(TTC)5	15	16900	16914	potentially variable
PHG9_Scaffold_763	3	p3	(GCA)6	18	36764	36781	potentially variable
PHG9_Scaffold_764	1	p2	(TA)7	14	2257	2270	potentially variable

PHG9_Scaffold_764	3	p3	(GTA)5	15	5432	5446	potentially variable
PHG9_Scaffold_764	7	p3	(TCA)6	18	15207	15224	potentially variable
PHG9_Scaffold_765	12	p2	(AT)6	12	50190	50201	
PHG9_Scaffold_765	20	p2	(AT)6	12	103241	103252	
PHG9_Scaffold_766	2	p2	(TG)7	14	6870	6883	potentially variable
PHG9_Scaffold_766	6	p2	(GT)7	14	24317	24330	potentially variable
PHG9_Scaffold_766	7	p2	(TA)7	14	25781	25794	potentially variable
PHG9_Scaffold_767	3	p2	(TG)6	12	1442	1453	
PHG9_Scaffold_767	15	p2	(AT)9	18	42857	42874	potentially variable
PHG9_Scaffold_767	19	p3	(CAA)5	15	54410	54424	potentially variable
PHG9_Scaffold_767	25	p2	(AT)8	16	72118	72133	potentially variable
PHG9_Scaffold_767	27	p2	(AT)6	12	81895	81906	
PHG9_Scaffold_767	30	p3	(AAG)5	15	91374	91388	potentially variable
PHG9_Scaffold_769	2	p3	(TTA)5	15	11933	11947	potentially variable
PHG9_Scaffold_770	3	p3	(AAT)6	18	11387	11404	potentially variable
PHG9_Scaffold_770	9	p2	(TA)6	12	53559	53570	
PHG9_Scaffold_770	12	p2	(TA)9	18	92662	92679	potentially variable
PHG9_Scaffold_770	14	p2	(TA)8	16	113728	113743	potentially

							variable
PHG9_Scaffold_770	15	p2	(AT)7	14	124369	124382	potentially variable
PHG9_Scaffold_773	6	p2	(AT)8	16	13502	13517	potentially variable
PHG9_Scaffold_773	17	p2	(TC)7	14	23308	23321	potentially variable
PHG9_Scaffold_773	21	p2	(AT)8	16	36113	36128	potentially variable
PHG9_Scaffold_774	3	p2	(TA)9	18	8991	9008	potentially variable
PHG9_Scaffold_774	4	p2	(TA)7	14	10163	10176	potentially variable
PHG9_Scaffold_774	10	p2	(AT)6	12	16030	16041	
PHG9_Scaffold_774	15	p3	(CAC)5	15	22776	22790	potentially variable
PHG9_Scaffold_775	1	p3	(AAT)5	15	1066	1080	potentially variable
PHG9_Scaffold_775	2	p2	(AG)6	12	18455	18466	
PHG9_Scaffold_775	3	p2	(CA)6	12	23031	23042	
PHG9_Scaffold_775	9	p2	(TA)7	14	49393	49406	potentially variable
PHG9_Scaffold_775	11	p2	(TA)7	14	53683	53696	potentially variable
PHG9_Scaffold_775	13	p3	(ATA)5	15	58025	58039	potentially variable
PHG9_Scaffold_775	15	p2	(AG)6	12	60499	60510	
PHG9_Scaffold_775	22	p3	(TTG)5	15	71882	71896	potentially variable
PHG9_Scaffold_775	23	p2	(TG)8	16	74308	74323	potentially

							variable
PHG9_Scaffold_775	30	p2	(TA)9	18	119397	119414	potentially variable
PHG9_Scaffold_775	33	p3	(AAT)6	18	131823	131840	potentially variable
PHG9_Scaffold_775	39	p3	(ACA)6	18	143055	143072	potentially variable
PHG9_Scaffold_776	10	p2	(AT)6	12	50121	50132	
PHG9_Scaffold_776	14	p2	(TA)7	14	58899	58912	potentially variable
PHG9_Scaffold_776	17	p3	(ATC)6	18	65979	65996	potentially variable
PHG9_Scaffold_776	26	p2	(CT)7	14	87325	87338	potentially variable
PHG9_Scaffold_776	27	p2	(AT)6	12	89472	89483	
PHG9_Scaffold_776	32	p2	(AC)7	14	96814	96827	potentially variable
PHG9_Scaffold_779	2	p2	(TA)8	16	14564	14579	potentially variable
PHG9_Scaffold_779	3	p2	(TA)8	16	25111	25126	potentially variable
PHG9_Scaffold_780	17	p2	(AT)7	14	95678	95691	potentially variable
PHG9_Scaffold_780	21	p2	(TA)8	16	102837	102852	potentially variable
PHG9_Scaffold_780	26	p3	(TAT)5	15	109946	109960	potentially variable
PHG9_Scaffold_780	32	p3	(TCC)5	15	134160	134174	potentially variable
PHG9_Scaffold_780	37	p2	(TA)9	18	138807	138824	potentially

							variable
PHG9_Scaffold_781	3	p2	(AT)6	12	4338	4349	
PHG9_Scaffold_784	5	p2	(AT)8	16	6083	6098	potentially variable
PHG9_Scaffold_784	6	p2	(TA)6	12	6751	6762	
PHG9_Scaffold_784	10	p3	(ACA)5	15	10152	10166	potentially variable
PHG9_Scaffold_784	14	p2	(TA)9	18	26600	26617	potentially variable
PHG9_Scaffold_784	23	p2	(AT)9	18	50314	50331	potentially variable
PHG9_Scaffold_784	25	p3	(ACC)5	15	52923	52937	potentially variable
PHG9_Scaffold_784	37	p3	(TTG)5	15	72691	72705	potentially variable
PHG9_Scaffold_786	10	p2	(TA)8	16	55369	55384	potentially variable
PHG9_Scaffold_786	11	p2	(AT)6	12	56274	56285	
PHG9_Scaffold_786	15	p2	(GA)8	16	68949	68964	potentially variable
PHG9_Scaffold_788	1	p3	(AGA)5	15	6343	6357	potentially variable
PHG9_Scaffold_788	5	p2	(TA)6	12	8167	8178	
PHG9_Scaffold_788	8	p2	(TA)6	12	14523	14534	
PHG9_Scaffold_789	1	p2	(TA)6	12	1209	1220	
PHG9_Scaffold_789	5	p2	(AT)6	12	21676	21687	
PHG9_Scaffold_789	8	p2	(AT)7	14	41172	41185	potentially variable
PHG9_Scaffold_789	11	p2	(AT)6	12	55699	55710	

PHG9_Scaffold_791	6	p2	(AT)8	16	11939	11954	potentially variable
PHG9_Scaffold_791	9	p3	(CAA)5	15	21529	21543	potentially variable
PHG9_Scaffold_791	10	p2	(CT)8	16	22456	22471	potentially variable
PHG9_Scaffold_791	23	p3	(TAT)5	15	74943	74957	potentially variable
PHG9_Scaffold_791	26	p3	(TAG)5	15	84419	84433	potentially variable
PHG9_Scaffold_791	28	p2	(TA)7	14	85411	85424	potentially variable
PHG9_Scaffold_791	34	p2	(AT)9	18	114042	114059	potentially variable
PHG9_Scaffold_791	36	p3	(GAA)6	18	118469	118486	potentially variable
PHG9_Scaffold_791	37	p2	(TA)7	14	121815	121828	potentially variable
PHG9_Scaffold_791	38	p2	(AT)6	12	128682	128693	
PHG9_Scaffold_792	2	p2	(AT)8	16	9412	9427	potentially variable
PHG9_Scaffold_793	14	p3	(ATA)5	15	30460	30474	potentially variable
PHG9_Scaffold_793	24	p2	(AT)6	12	50925	50936	
PHG9_Scaffold_793	25	p2	(AT)6	12	51455	51466	
PHG9_Scaffold_795	3	p3	(ATT)6	18	3878	3895	potentially variable
PHG9_Scaffold_798	1	p3	(ACA)5	15	3246	3260	potentially variable
PHG9_Scaffold_798	4	p2	(AT)6	12	12417	12428	

PHG9_Scaffold_798	11	p2	(TC)6	12	31933	31944	
PHG9_Scaffold_798	12	p3	(CTC)5	15	34614	34628	potentially variable
PHG9_Scaffold_798	15	p3	(TAA)5	15	44324	44338	potentially variable
PHG9_Scaffold_798	16	p3	(TCA)6	18	46201	46218	potentially variable
PHG9_Scaffold_798	17	p3	(AAC)5	15	46552	46566	potentially variable
PHG9_Scaffold_798	22	p2	(GA)9	18	52916	52933	potentially variable
PHG9_Scaffold_798	29	p2	(AT)7	14	71376	71389	potentially variable
PHG9_Scaffold_798	30	p2	(TA)6	12	72839	72850	
PHG9_Scaffold_798	37	p2	(CT)6	12	102199	102210	
PHG9_Scaffold_798	42	p2	(GA)6	12	124917	124928	
PHG9_Scaffold_798	43	p3	(AGA)6	18	125164	125181	potentially variable
PHG9_Scaffold_804	2	p2	(AT)7	14	27123	27136	potentially variable
PHG9_Scaffold_804	4	p2	(AT)6	12	30245	30256	
PHG9_Scaffold_804	16	p2	(AT)6	12	65689	65700	
PHG9_Scaffold_804	23	p2	(GA)6	12	80370	80381	
PHG9_Scaffold_805	9	p3	(ATT)5	15	29825	29839	potentially variable
PHG9_Scaffold_805	16	p2	(CT)6	12	47559	47570	
PHG9_Scaffold_805	19	p2	(AT)6	12	50513	50524	
PHG9_Scaffold_806	4	p2	(TA)6	12	10069	10080	
PHG9_Scaffold_806	12	p3	(AGA)5	15	38698	38712	potentially

							variable
PHG9_Scaffold_806	16	p2	(TA)7	14	45819	45832	potentially variable
PHG9_Scaffold_806	17	p3	(TAA)6	18	50181	50198	potentially variable
PHG9_Scaffold_806	19	p3	(CAA)5	15	55410	55424	potentially variable
PHG9_Scaffold_806	21	p3	(CTT)5	15	70329	70343	potentially variable
PHG9_Scaffold_806	24	p2	(TA)6	12	80626	80637	
PHG9_Scaffold_807	3	p3	(ATT)5	15	15378	15392	potentially variable
PHG9_Scaffold_807	8	p2	(CT)9	18	27189	27206	potentially variable
PHG9_Scaffold_807	18	p2	(GT)6	12	45509	45520	
PHG9_Scaffold_808	6	p2	(TA)7	14	20241	20254	potentially variable
PHG9_Scaffold_808	10	p2	(TA)7	14	25983	25996	potentially variable
PHG9_Scaffold_808	18	p3	(ATA)6	18	42240	42257	potentially variable
PHG9_Scaffold_808	21	p2	(TA)6	12	54459	54470	
PHG9_Scaffold_810	4	p2	(TC)6	12	12058	12069	
PHG9_Scaffold_811	6	p2	(TA)9	18	25723	25740	potentially variable
PHG9_Scaffold_811	11	p2	(AT)9	18	44520	44537	potentially variable
PHG9_Scaffold_813	5	p3	(AAT)5	15	15072	15086	potentially variable
PHG9_Scaffold_813	6	p3	(TAT)5	15	16567	16581	potentially

							variable
PHG9_Scaffold_813	12	p3	(ATT)5	15	29197	29211	potentially variable
PHG9_Scaffold_813	16	p2	(AT)9	18	45065	45082	potentially variable
PHG9_Scaffold_813	18	p2	(AT)9	18	55955	55972	potentially variable
PHG9_Scaffold_813	22	p2	(AT)7	14	68379	68392	potentially variable
PHG9_Scaffold_813	23	p2	(CT)7	14	68827	68840	potentially variable
PHG9_Scaffold_813	24	p3	(TTA)5	15	78896	78910	potentially variable
PHG9_Scaffold_816	5	p2	(CT)6	12	9005	9016	
PHG9_Scaffold_816	10	p2	(AG)8	16	22154	22169	potentially variable
PHG9_Scaffold_816	18	p3	(CTT)6	18	36289	36306	potentially variable
PHG9_Scaffold_816	21	p3	(CTT)6	18	39356	39373	potentially variable
PHG9_Scaffold_816	23	p2	(AT)7	14	40497	40510	potentially variable
PHG9_Scaffold_816	30	p3	(GCT)5	15	67778	67792	potentially variable
PHG9_Scaffold_817	6	p2	(TA)7	14	14909	14922	potentially variable
PHG9_Scaffold_818	10	p2	(TA)6	12	20989	21000	
PHG9_Scaffold_818	19	p2	(TA)8	16	50387	50402	potentially variable
PHG9_Scaffold_818	21	p2	(AT)7	14	51477	51490	potentially

							variable
PHG9_Scaffold_819	2	p2	(TA)7	14	36699	36712	potentially variable
PHG9_Scaffold_820	14	p2	(AT)7	14	58960	58973	potentially variable
PHG9_Scaffold_820	21	p2	(AG)8	16	100367	100382	potentially variable
PHG9_Scaffold_821	4	p2	(AT)9	18	22003	22020	potentially variable
PHG9_Scaffold_821	18	p2	(AT)9	18	78085	78102	potentially variable
PHG9_Scaffold_822	8	p2	(AT)7	14	28276	28289	potentially variable
PHG9_Scaffold_824	2	p2	(AT)8	16	16120	16135	potentially variable
PHG9_Scaffold_824	5	p2	(TA)6	12	26276	26287	
PHG9_Scaffold_824	8	p2	(TA)9	18	33450	33467	potentially variable
PHG9_Scaffold_824	9	p2	(AT)6	12	34300	34311	
PHG9_Scaffold_825	7	p2	(TA)8	16	27791	27806	potentially variable
PHG9_Scaffold_828	7	p2	(AT)6	12	58885	58896	
PHG9_Scaffold_829	6	p2	(GA)6	12	11331	11342	
PHG9_Scaffold_829	11	p2	(TA)6	12	22115	22126	
PHG9_Scaffold_830	2	p2	(AT)7	14	10414	10427	potentially variable
PHG9_Scaffold_831	1	p3	(AAC)5	15	1276	1290	potentially variable
PHG9_Scaffold_832	8	p2	(AT)7	14	30380	30393	potentially variable

PHG9_Scaffold_832	9	p2	(TA)7	14	30620	30633	potentially variable
PHG9_Scaffold_832	10	p2	(AT)7	14	33729	33742	potentially variable
PHG9_Scaffold_832	15	p2	(TA)6	12	51251	51262	
PHG9_Scaffold_832	19	p2	(AT)7	14	64959	64972	potentially variable
PHG9_Scaffold_832	21	p2	(GT)9	18	68126	68143	potentially variable
PHG9_Scaffold_832	23	p3	(TCT)6	18	71156	71173	potentially variable
PHG9_Scaffold_832	25	p2	(CT)7	14	80464	80477	potentially variable
PHG9_Scaffold_832	27	p3	(TTA)5	15	82832	82846	potentially variable
PHG9_Scaffold_832	32	p2	(AG)6	12	91555	91566	
PHG9_Scaffold_832	36	p3	(CAC)6	18	98436	98453	potentially variable
PHG9_Scaffold_832	55	p3	(GTT)5	15	197288	197302	potentially variable
PHG9_Scaffold_832	65	p2	(TA)6	12	243794	243805	
PHG9_Scaffold_832	69	p2	(AC)6	12	261482	261493	
PHG9_Scaffold_832	70	p3	(TTA)5	15	266646	266660	potentially variable
PHG9_Scaffold_832	81	p2	(GA)6	12	286561	286572	
PHG9_Scaffold_832	83	p3	(CTT)5	15	291531	291545	potentially variable
PHG9_Scaffold_832	84	p3	(AAT)5	15	291666	291680	potentially variable
PHG9_Scaffold_832	91	p2	(TA)6	12	312218	312229	

PHG9_Scaffold_832	93	p3	(TTC)5	15	320595	320609	potentially variable
PHG9_Scaffold_832	94	p2	(TG)6	12	321649	321660	
PHG9_Scaffold_832	96	p2	(TA)6	12	322534	322545	
PHG9_Scaffold_832	108	p2	(CT)6	12	370171	370182	
PHG9_Scaffold_832	117	p2	(AG)7	14	381626	381639	potentially variable
PHG9_Scaffold_832	124	p2	(TA)7	14	393464	393477	potentially variable
PHG9_Scaffold_832	125	p2	(AG)6	12	394159	394170	
PHG9_Scaffold_833	1	p3	(AAT)5	15	3312	3326	potentially variable
PHG9_Scaffold_833	2	p2	(AG)6	12	3570	3581	
PHG9_Scaffold_833	4	p3	(ATA)6	18	8083	8100	potentially variable
PHG9_Scaffold_833	5	p2	(AT)9	18	12897	12914	potentially variable
PHG9_Scaffold_833	7	p3	(TAT)5	15	17238	17252	potentially variable
PHG9_Scaffold_834	4	p3	(AAT)5	15	6470	6484	potentially variable
PHG9_Scaffold_834	12	p2	(TC)9	18	48121	48138	potentially variable
PHG9_Scaffold_834	14	p2	(TA)6	12	59767	59778	
PHG9_Scaffold_834	16	p2	(TA)7	14	69961	69974	potentially variable
PHG9_Scaffold_834	18	p2	(AC)6	12	73622	73633	
PHG9_Scaffold_834	23	p2	(AG)7	14	89671	89684	potentially variable

PHG9_Scaffold_834	25	p2	(TA)7	14	99924	99937	potentially variable
PHG9_Scaffold_834	47	p2	(AT)6	12	196838	196849	
PHG9_Scaffold_834	53	p2	(TG)7	14	214082	214095	potentially variable
PHG9_Scaffold_834	54	p2	(CA)6	12	216062	216073	
PHG9_Scaffold_834	56	p2	(TA)6	12	229031	229042	
PHG9_Scaffold_834	68	p3	(TTA)5	15	272143	272157	potentially variable
PHG9_Scaffold_834	75	p2	(TA)8	16	316341	316356	potentially variable
PHG9_Scaffold_834	77	p2	(AT)6	12	321923	321934	
PHG9_Scaffold_834	85	p2	(AT)8	16	365714	365729	potentially variable
PHG9_Scaffold_834	88	p2	(AT)6	12	393082	393093	
PHG9_Scaffold_834	92	p2	(AC)6	12	403569	403580	
PHG9_Scaffold_834	97	p2	(AT)9	18	411603	411620	potentially variable
PHG9_Scaffold_834	98	p3	(ATA)5	15	412433	412447	potentially variable
PHG9_Scaffold_834	99	p2	(TC)7	14	412827	412840	potentially variable
PHG9_Scaffold_834	110	p3	(ATT)5	15	443582	443596	potentially variable
PHG9_Scaffold_834	112	p2	(TC)7	14	444950	444963	potentially variable
PHG9_Scaffold_834	113	p3	(TTA)5	15	445823	445837	potentially variable
PHG9_Scaffold_836	2	p3	(ATG)5	15	6568	6582	potentially variable

PHG9_Scaffold_837	3	p2	(AT)8	16	14683	14698	potentially variable
PHG9_Scaffold_837	14	p2	(AT)8	16	44987	45002	potentially variable
PHG9_Scaffold_838	8	p2	(AT)9	18	32226	32243	potentially variable
PHG9_Scaffold_840	3	p2	(TA)8	16	4608	4623	potentially variable
PHG9_Scaffold_840	9	p3	(GGA)5	15	22530	22544	potentially variable
PHG9_Scaffold_840	11	p2	(AT)6	12	23818	23829	
PHG9_Scaffold_840	25	p3	(TCT)5	15	61593	61607	potentially variable
PHG9_Scaffold_840	41	p3	(ATA)5	15	122211	122225	potentially variable
PHG9_Scaffold_842	4	p2	(TA)8	16	10567	10582	potentially variable
PHG9_Scaffold_842	5	p2	(AT)7	14	13042	13055	potentially variable
PHG9_Scaffold_842	8	p2	(TG)6	12	21094	21105	
PHG9_Scaffold_842	9	p3	(ATA)5	15	22971	22985	potentially variable
PHG9_Scaffold_842	11	p3	(GGA)5	15	24866	24880	potentially variable
PHG9_Scaffold_842	14	p2	(AT)8	16	29960	29975	potentially variable
PHG9_Scaffold_842	16	p2	(AT)6	12	33796	33807	
PHG9_Scaffold_842	17	p3	(TGG)5	15	38040	38054	potentially variable
PHG9_Scaffold_842	20	p2	(TA)7	14	41491	41504	potentially

							variable
PHG9_Scaffold_843	11	p2	(CT)7	14	31129	31142	potentially variable
PHG9_Scaffold_843	15	p2	(AT)6	12	44714	44725	
PHG9_Scaffold_846	2	p2	(TA)8	16	1270	1285	potentially variable
PHG9_Scaffold_846	3	p2	(TA)6	12	20183	20194	
PHG9_Scaffold_846	4	p3	(TGC)5	15	22582	22596	potentially variable
PHG9_Scaffold_846	6	p2	(TA)6	12	26832	26843	
PHG9_Scaffold_846	13	p2	(CA)7	14	39900	39913	potentially variable
PHG9_Scaffold_847	4	p2	(AT)9	18	20304	20321	potentially variable
PHG9_Scaffold_847	6	p2	(TA)8	16	29567	29582	potentially variable
PHG9_Scaffold_848	3	p2	(AT)6	12	18838	18849	
PHG9_Scaffold_848	9	p2	(TA)9	18	35397	35414	potentially variable
PHG9_Scaffold_849	10	p2	(TA)7	14	26513	26526	potentially variable
PHG9_Scaffold_849	11	p3	(ATT)5	15	26699	26713	potentially variable
PHG9_Scaffold_849	23	p2	(TC)8	16	69586	69601	potentially variable
PHG9_Scaffold_850	1	p2	(TA)7	14	7515	7528	potentially variable
PHG9_Scaffold_850	10	p2	(AT)7	14	26605	26618	potentially variable
PHG9_Scaffold_850	15	p2	(AT)6	12	46610	46621	

PHG9_Scaffold_850	27	p2	(AT)8	16	77942	77957	potentially variable
PHG9_Scaffold_850	49	p2	(TA)7	14	150202	150215	potentially variable
PHG9_Scaffold_850	51	p3	(TAA)6	18	151245	151262	potentially variable
PHG9_Scaffold_850	57	p3	(GAA)5	15	173165	173179	potentially variable
PHG9_Scaffold_850	59	p3	(TAA)5	15	176085	176099	potentially variable
PHG9_Scaffold_850	64	p2	(TA)7	14	186618	186631	potentially variable
PHG9_Scaffold_850	75	p3	(TGA)5	15	217961	217975	potentially variable
PHG9_Scaffold_850	77	p2	(CT)7	14	221036	221049	potentially variable
PHG9_Scaffold_850	81	p3	(TCA)5	15	231823	231837	potentially variable
PHG9_Scaffold_850	89	p2	(AT)6	12	250699	250710	
PHG9_Scaffold_850	90	p3	(TAA)5	15	254028	254042	potentially variable
PHG9_Scaffold_850	93	p3	(TTA)5	15	258626	258640	potentially variable
PHG9_Scaffold_850	94	p2	(CT)9	18	259159	259176	potentially variable
PHG9_Scaffold_850	96	p2	(CT)7	14	267939	267952	potentially variable
PHG9_Scaffold_850	113	p2	(AT)6	12	312748	312759	
PHG9_Scaffold_850	118	p2	(AT)7	14	325561	325574	potentially variable

PHG9_Scaffold_850	124	p2	(CT)6	12	347736	347747	
PHG9_Scaffold_850	131	p2	(GA)6	12	367147	367158	
PHG9_Scaffold_850	143	p3	(TTA)5	15	441065	441079	potentially variable
PHG9_Scaffold_850	147	p2	(AT)7	14	446112	446125	potentially variable
PHG9_Scaffold_852	2	p2	(TA)8	16	12286	12301	potentially variable
PHG9_Scaffold_852	7	p2	(TA)7	14	21703	21716	potentially variable
PHG9_Scaffold_852	8	p2	(GT)8	16	28124	28139	potentially variable
PHG9_Scaffold_852	18	p3	(CAA)5	15	56519	56533	potentially variable
PHG9_Scaffold_853	15	p3	(AAG)6	18	25647	25664	potentially variable
PHG9_Scaffold_853	21	p3	(AAT)6	18	47866	47883	potentially variable
PHG9_Scaffold_853	23	p2	(TC)6	12	49606	49617	
PHG9_Scaffold_853	28	p3	(CAA)5	15	63713	63727	potentially variable
PHG9_Scaffold_853	32	p3	(ATA)5	15	69519	69533	potentially variable
PHG9_Scaffold_853	34	p2	(CA)6	12	77393	77404	
PHG9_Scaffold_853	48	p3	(ATA)6	18	103938	103955	potentially variable
PHG9_Scaffold_853	50	p2	(AT)8	16	111581	111596	potentially variable
PHG9_Scaffold_853	54	p3	(CAA)5	15	116862	116876	potentially variable

PHG9_Scaffold_853	59	p2	(AT)6	12	129971	129982	
PHG9_Scaffold_853	65	p2	(TC)6	12	134309	134320	
PHG9_Scaffold_853	68	p3	(ATT)5	15	142568	142582	potentially variable
PHG9_Scaffold_853	72	p2	(TA)7	14	153762	153775	potentially variable
PHG9_Scaffold_853	82	p2	(TA)8	16	183489	183504	potentially variable
PHG9_Scaffold_853	87	p2	(AT)6	12	202697	202708	
PHG9_Scaffold_855	1	p2	(AT)9	18	1025	1042	potentially variable
PHG9_Scaffold_855	7	p2	(CT)8	16	13080	13095	potentially variable
PHG9_Scaffold_855	12	p2	(TG)6	12	20023	20034	
PHG9_Scaffold_856	14	p3	(ATA)6	18	42197	42214	potentially variable
PHG9_Scaffold_856	22	p2	(AT)6	12	57622	57633	
PHG9_Scaffold_856	33	p2	(TA)8	16	81316	81331	potentially variable
PHG9_Scaffold_856	41	p2	(AG)7	14	101598	101611	potentially variable
PHG9_Scaffold_856	43	p2	(AT)6	12	110901	110912	
PHG9_Scaffold_856	48	p3	(TAA)6	18	116588	116605	potentially variable
PHG9_Scaffold_856	50	p2	(AT)7	14	125357	125370	potentially variable
PHG9_Scaffold_856	63	p3	(TAT)5	15	165605	165619	potentially variable
PHG9_Scaffold_856	68	p3	(TGT)5	15	176307	176321	potentially variable

PHG9_Scaffold_856	71	p2	(AC)6	12	181548	181559	
PHG9_Scaffold_856	73	p2	(TA)9	18	186019	186036	potentially variable
PHG9_Scaffold_856	75	p2	(AT)9	18	207506	207523	potentially variable
PHG9_Scaffold_857	6	p2	(AT)6	12	17813	17824	
PHG9_Scaffold_857	8	p2	(TC)6	12	20538	20549	
PHG9_Scaffold_857	15	p3	(AAC)5	15	38766	38780	potentially variable
PHG9_Scaffold_857	24	p2	(TA)9	18	54969	54986	potentially variable
PHG9_Scaffold_859	12	p2	(AT)7	14	55759	55772	potentially variable
PHG9_Scaffold_861	1	p2	(TA)6	12	1540	1551	
PHG9_Scaffold_861	8	p2	(TA)9	18	19970	19987	potentially variable
PHG9_Scaffold_861	9	p3	(AAC)6	18	25857	25874	potentially variable
PHG9_Scaffold_861	13	p3	(TTA)5	15	44204	44218	potentially variable
PHG9_Scaffold_861	17	p2	(TA)6	12	61037	61048	
PHG9_Scaffold_862	6	p2	(TA)6	12	33668	33679	
PHG9_Scaffold_863	4	p2	(TA)8	16	1919	1934	potentially variable
PHG9_Scaffold_863	11	p2	(TC)6	12	16079	16090	
PHG9_Scaffold_863	13	p2	(GT)6	12	22440	22451	
PHG9_Scaffold_863	30	p2	(TA)6	12	67554	67565	
PHG9_Scaffold_863	43	p3	(TAA)6	18	102911	102928	potentially variable

PHG9_Scaffold_863	48	p2	(TA)6	12	117673	117684	
PHG9_Scaffold_864	1	p2	(AT)6	12	38561	38572	
PHG9_Scaffold_864	6	p2	(AT)7	14	62865	62878	potentially variable
PHG9_Scaffold_865	4	p2	(AT)6	12	14579	14590	
PHG9_Scaffold_865	5	p2	(AT)8	16	15956	15971	potentially variable
PHG9_Scaffold_865	6	p2	(AT)7	14	34054	34067	potentially variable
PHG9_Scaffold_866	2	p3	(GTT)5	15	16071	16085	potentially variable
PHG9_Scaffold_868	2	p2	(TG)6	12	6401	6412	
PHG9_Scaffold_868	29	p2	(TG)6	12	70138	70149	
PHG9_Scaffold_868	33	p6	(TGGTGT)5	30	74096	74125	Hypervariable
PHG9_Scaffold_868	37	p2	(GA)6	12	97561	97572	
PHG9_Scaffold_868	39	p2	(TA)7	14	102167	102180	potentially variable
PHG9_Scaffold_870	2	p2	(AT)6	12	3740	3751	
PHG9_Scaffold_870	21	p2	(GT)7	14	79483	79496	potentially variable
PHG9_Scaffold_870	32	p2	(CT)8	16	112632	112647	potentially variable
PHG9_Scaffold_870	44	p3	(ATA)5	15	166308	166322	potentially variable
PHG9_Scaffold_870	52	p2	(AG)8	16	200028	200043	potentially variable
PHG9_Scaffold_872	1	p2	(TA)7	14	909	922	potentially variable
PHG9_Scaffold_872	4	p2	(TA)8	16	6822	6837	potentially

							variable
PHG9_Scaffold_874	4	p2	(AT)8	16	12927	12942	potentially variable
PHG9_Scaffold_874	5	p2	(TA)9	18	14456	14473	potentially variable
PHG9_Scaffold_874	11	p2	(AT)9	18	38582	38599	potentially variable
PHG9_Scaffold_875	2	p2	(TA)9	18	3622	3639	potentially variable
PHG9_Scaffold_879	1	p2	(TA)6	12	10366	10377	
PHG9_Scaffold_880	5	p2	(AT)6	12	12290	12301	
PHG9_Scaffold_880	8	p2	(TA)8	16	27165	27180	potentially variable
PHG9_Scaffold_880	13	p3	(TCT)5	15	33534	33548	potentially variable
PHG9_Scaffold_880	15	p2	(CT)6	12	35060	35071	
PHG9_Scaffold_880	17	p2	(TC)6	12	37510	37521	
PHG9_Scaffold_880	18	p2	(TA)7	14	37752	37765	potentially variable
PHG9_Scaffold_882	4	p2	(AT)9	18	19754	19771	potentially variable
PHG9_Scaffold_882	5	p2	(CT)6	12	22389	22400	
PHG9_Scaffold_882	10	p3	(AGA)6	18	52490	52507	potentially variable
PHG9_Scaffold_882	11	p3	(AGA)5	15	53313	53327	potentially variable
PHG9_Scaffold_882	12	p3	(AGA)6	18	54149	54166	potentially variable
PHG9_Scaffold_882	13	p3	(AGA)6	18	54990	55007	potentially variable

PHG9_Scaffold_883	10	p2	(TA)8	16	14945	14960	potentially variable
PHG9_Scaffold_883	20	p2	(AT)8	16	40358	40373	potentially variable
PHG9_Scaffold_883	21	p2	(TA)6	12	42024	42035	
PHG9_Scaffold_883	25	p2	(TA)6	12	50952	50963	
PHG9_Scaffold_884	5	p2	(AT)8	16	17102	17117	potentially variable
PHG9_Scaffold_884	10	p3	(TAT)5	15	30127	30141	potentially variable
PHG9_Scaffold_884	14	p3	(GAT)6	18	42440	42457	potentially variable
PHG9_Scaffold_884	19	p2	(AG)7	14	62219	62232	potentially variable
PHG9_Scaffold_884	26	p2	(AT)8	16	77494	77509	potentially variable
PHG9_Scaffold_884	28	p2	(GA)6	12	84539	84550	
PHG9_Scaffold_884	30	p2	(CT)6	12	85389	85400	
PHG9_Scaffold_884	41	p3	(TCC)5	15	117403	117417	potentially variable
PHG9_Scaffold_884	43	p2	(TA)6	12	122837	122848	
PHG9_Scaffold_884	45	p3	(CAC)5	15	134357	134371	potentially variable
PHG9_Scaffold_885	16	p2	(AT)7	14	56306	56319	potentially variable
PHG9_Scaffold_885	18	p2	(TA)7	14	73326	73339	potentially variable
PHG9_Scaffold_885	25	p2	(TA)6	12	94343	94354	
PHG9_Scaffold_886	1	p2	(AT)7	14	34	47	potentially variable

PHG9_Scaffold_888	1	p3	(TCA)5	15	1477	1491	potentially variable
PHG9_Scaffold_888	5	p2	(AT)6	12	5516	5527	
PHG9_Scaffold_888	16	p3	(TAA)5	15	42674	42688	potentially variable
PHG9_Scaffold_888	17	p3	(AAG)5	15	45212	45226	potentially variable
PHG9_Scaffold_889	8	p3	(AAT)6	18	19704	19721	potentially variable
PHG9_Scaffold_889	14	p2	(TA)6	12	27966	27977	
PHG9_Scaffold_889	15	p3	(TGT)5	15	28811	28825	potentially variable
PHG9_Scaffold_889	18	p3	(CAA)5	15	43979	43993	potentially variable
PHG9_Scaffold_893	1	p2	(TA)7	14	1638	1651	potentially variable
PHG9_Scaffold_893	2	p3	(ATT)5	15	4688	4702	potentially variable
PHG9_Scaffold_893	4	p2	(TG)6	12	7696	7707	
PHG9_Scaffold_893	5	p2	(TC)6	12	8031	8042	
PHG9_Scaffold_893	8	p2	(TA)9	18	9317	9334	potentially variable
PHG9_Scaffold_893	17	p2	(CT)7	14	23999	24012	potentially variable
PHG9_Scaffold_893	18	p2	(AT)7	14	24583	24596	potentially variable
PHG9_Scaffold_893	20	p3	(TGA)5	15	26543	26557	potentially variable
PHG9_Scaffold_893	24	p3	(TAA)5	15	30501	30515	potentially variable

PHG9_Scaffold_893	26	p2	(TG)7	14	33156	33169	potentially variable
PHG9_Scaffold_893	32	p2	(AT)7	14	47731	47744	potentially variable
PHG9_Scaffold_893	39	p2	(AT)6	12	75177	75188	
PHG9_Scaffold_893	41	p3	(AAT)5	15	90749	90763	potentially variable
PHG9_Scaffold_893	49	p3	(ATC)6	18	112574	112591	potentially variable
PHG9_Scaffold_893	58	p2	(CA)6	12	128180	128191	
PHG9_Scaffold_893	72	p2	(CT)6	12	157405	157416	
PHG9_Scaffold_896	9	p3	(ATT)5	15	20860	20874	potentially variable
PHG9_Scaffold_896	10	p2	(GT)6	12	22182	22193	
PHG9_Scaffold_897	7	p3	(AAT)5	15	38567	38581	potentially variable
PHG9_Scaffold_900	4	p2	(TA)6	12	17405	17416	
PHG9_Scaffold_900	11	p2	(TA)6	12	53128	53139	
PHG9_Scaffold_900	12	p2	(AT)7	14	53470	53483	potentially variable
PHG9_Scaffold_903	5	p2	(TA)8	16	29632	29647	potentially variable
PHG9_Scaffold_903	10	p3	(CAC)5	15	39405	39419	potentially variable
PHG9_Scaffold_903	14	p2	(AT)7	14	75234	75247	potentially variable
PHG9_Scaffold_908	3	p2	(TA)8	16	7656	7671	potentially variable
PHG9_Scaffold_910	2	p2	(AT)6	12	5855	5866	

PHG9_Scaffold_914	2	p2	(AT)6	12	2186	2197	
PHG9_Scaffold_914	9	p2	(TA)6	12	50079	50090	
PHG9_Scaffold_914	12	p3	(ATA)5	15	56937	56951	potentially variable
PHG9_Scaffold_917	8	p3	(CTT)5	15	25374	25388	potentially variable
PHG9_Scaffold_917	10	p2	(TA)7	14	36307	36320	potentially variable
PHG9_Scaffold_918	14	p2	(TG)6	12	53601	53612	
PHG9_Scaffold_919	2	p2	(GA)6	12	3120	3131	
PHG9_Scaffold_919	4	p2	(TA)9	18	4659	4676	potentially variable
PHG9_Scaffold_919	6	p3	(TTC)5	15	17136	17150	potentially variable
PHG9_Scaffold_919	8	p3	(TGG)5	15	27496	27510	potentially variable
PHG9_Scaffold_919	9	p2	(AT)7	14	28256	28269	potentially variable
PHG9_Scaffold_919	11	p3	(TCC)5	15	32912	32926	potentially variable
PHG9_Scaffold_920	3	p2	(AG)6	12	2508	2519	
PHG9_Scaffold_920	4	p3	(CAG)5	15	2789	2803	potentially variable
PHG9_Scaffold_920	5	p3	(TGC)6	18	2911	2928	potentially variable
PHG9_Scaffold_920	10	p2	(TA)8	16	4717	4732	potentially variable
PHG9_Scaffold_923	12	p2	(AG)10	20	23129	23148	Hypervariable
PHG9_Scaffold_923	13	p2	(AG)7	14	26760	26773	potentially variable

PHG9_Scaffold_923	18	p2	(TA)9	18	44451	44468	potentially variable
PHG9_Scaffold_923	20	p3	(ATT)5	15	49419	49433	potentially variable
PHG9_Scaffold_923	22	p3	(AGG)6	18	57767	57784	potentially variable
PHG9_Scaffold_923	23	p3	(GGT)6	18	59031	59048	potentially variable
PHG9_Scaffold_923	24	p2	(AT)9	18	65852	65869	potentially variable
PHG9_Scaffold_923	28	p2	(AT)6	12	75598	75609	
PHG9_Scaffold_924	10	p3	(TAT)5	15	26103	26117	potentially variable
PHG9_Scaffold_924	19	p2	(CT)7	14	48662	48675	potentially variable
PHG9_Scaffold_925	5	p2	(AT)6	12	29872	29883	
PHG9_Scaffold_925	19	p2	(AG)7	14	74367	74380	potentially variable
PHG9_Scaffold_925	30	p2	(TA)6	12	98657	98668	
PHG9_Scaffold_925	34	p3	(CTT)5	15	106044	106058	potentially variable
PHG9_Scaffold_925	39	p2	(TA)7	14	125707	125720	potentially variable
PHG9_Scaffold_925	48	p2	(GA)6	12	142776	142787	
PHG9_Scaffold_925	52	p3	(TGA)6	18	153316	153333	potentially variable
PHG9_Scaffold_926	2	p2	(TA)7	14	19073	19086	potentially variable
PHG9_Scaffold_926	5	p3	(GAT)5	15	25330	25344	potentially variable

PHG9_Scaffold_926	6	p2	(AT)6	12	27614	27625	
PHG9_Scaffold_927	10	p2	(AT)6	12	45877	45888	
PHG9_Scaffold_928	5	p2	(TC)8	16	13583	13598	potentially variable
PHG9_Scaffold_928	12	p2	(AC)7	14	44710	44723	potentially variable
PHG9_Scaffold_928	20	p2	(TA)7	14	64645	64658	potentially variable
PHG9_Scaffold_929	5	p2	(TA)8	16	12908	12923	potentially variable
PHG9_Scaffold_929	6	p2	(TG)6	12	26768	26779	
PHG9_Scaffold_929	9	p3	(AAT)5	15	43387	43401	potentially variable
PHG9_Scaffold_929	11	p2	(AG)6	12	48846	48857	
PHG9_Scaffold_929	12	p6	(CTCAGC)6	36	49013	49048	Hypervariable
PHG9_Scaffold_929	24	p2	(AG)8	16	81410	81425	potentially variable
PHG9_Scaffold_930	4	p3	(TAT)5	15	16381	16395	potentially variable
PHG9_Scaffold_930	12	p3	(TGC)6	18	41162	41179	potentially variable
PHG9_Scaffold_931	1	p2	(AT)9	18	41789	41806	potentially variable
PHG9_Scaffold_932	11	p2	(TA)6	12	37908	37919	
PHG9_Scaffold_932	12	p2	(AC)7	14	38845	38858	potentially variable
PHG9_Scaffold_932	15	p2	(GA)6	12	44604	44615	
PHG9_Scaffold_933	5	p2	(AG)7	14	34097	34110	potentially variable

PHG9_Scaffold_936	13	p2	(TA)9	18	64175	64192	potentially variable
PHG9_Scaffold_936	18	p2	(TA)6	12	76450	76461	
PHG9_Scaffold_937	16	p2	(TA)7	14	107156	107169	potentially variable
PHG9_Scaffold_937	20	p2	(AT)9	18	132589	132606	potentially variable
PHG9_Scaffold_937	27	p2	(AT)6	12	181967	181978	
PHG9_Scaffold_939	1	p2	(TA)7	14	1319	1332	potentially variable
PHG9_Scaffold_939	2	p2	(AT)6	12	6849	6860	
PHG9_Scaffold_939	5	p3	(ATC)5	15	13446	13460	potentially variable
PHG9_Scaffold_939	7	p2	(AT)7	14	29687	29700	potentially variable
PHG9_Scaffold_939	16	p2	(AG)6	12	68456	68467	
PHG9_Scaffold_941	6	p2	(AG)6	12	38234	38245	
PHG9_Scaffold_943	5	p2	(AT)8	16	73615	73630	potentially variable
PHG9_Scaffold_943	8	p3	(ATT)5	15	80170	80184	potentially variable
PHG9_Scaffold_943	9	p2	(TA)7	14	83102	83115	potentially variable
PHG9_Scaffold_943	11	p3	(TAT)5	15	92057	92071	potentially variable
PHG9_Scaffold_945	8	p2	(AT)6	12	8825	8836	
PHG9_Scaffold_945	11	p2	(TA)9	18	28777	28794	potentially variable
PHG9_Scaffold_945	22	p2	(AT)7	14	57002	57015	potentially variable

PHG9_Scaffold_945	25	p2	(CA)6	12	63131	63142	
PHG9_Scaffold_945	26	p3	(TAT)6	18	65920	65937	potentially variable
PHG9_Scaffold_946	9	p2	(TA)6	12	105638	105649	
PHG9_Scaffold_947	2	p3	(TGA)5	15	2606	2620	potentially variable
PHG9_Scaffold_947	10	p2	(TA)7	14	29388	29401	potentially variable
PHG9_Scaffold_947	12	p2	(AT)9	18	34338	34355	potentially variable
PHG9_Scaffold_947	17	p2	(AT)6	12	42754	42765	
PHG9_Scaffold_947	20	p3	(GAA)5	15	47419	47433	potentially variable
PHG9_Scaffold_947	25	p2	(AT)6	12	67291	67302	
PHG9_Scaffold_947	28	p2	(AT)6	12	76890	76901	
PHG9_Scaffold_947	35	p3	(TTC)6	18	96402	96419	potentially variable
PHG9_Scaffold_948	2	p2	(TA)8	16	2072	2087	potentially variable
PHG9_Scaffold_948	3	p2	(TA)6	12	2993	3004	
PHG9_Scaffold_948	6	p2	(TA)9	18	16771	16788	potentially variable
PHG9_Scaffold_948	14	p2	(TA)8	16	39452	39467	potentially variable
PHG9_Scaffold_951	5	p3	(TGT)5	15	9301	9315	potentially variable
PHG9_Scaffold_951	15	p3	(TTA)5	15	37733	37747	potentially variable
PHG9_Scaffold_951	19	p2	(TA)7	14	39935	39948	potentially variable

PHG9_Scaffold_951	20	p2	(TA)7	14	40170	40183	potentially variable
PHG9_Scaffold_952	6	p2	(TA)8	16	19560	19575	potentially variable
PHG9_Scaffold_952	11	p3	(AAT)5	15	36071	36085	potentially variable
PHG9_Scaffold_952	23	p2	(TA)9	18	89174	89191	potentially variable
PHG9_Scaffold_952	28	p2	(AT)7	14	109659	109672	potentially variable
PHG9_Scaffold_952	33	p2	(AT)6	12	115172	115183	
PHG9_Scaffold_953	6	p2	(TG)8	16	15441	15456	potentially variable
PHG9_Scaffold_953	7	p2	(AT)8	16	17003	17018	potentially variable
PHG9_Scaffold_953	11	p3	(GAA)5	15	25186	25200	potentially variable
PHG9_Scaffold_953	14	p3	(ATT)5	15	31687	31701	potentially variable
PHG9_Scaffold_953	22	p2	(CA)8	16	43441	43456	potentially variable
PHG9_Scaffold_953	27	p2	(AT)6	12	71764	71775	
PHG9_Scaffold_953	29	p2	(AC)6	12	83989	84000	
PHG9_Scaffold_953	30	p2	(AG)8	16	106624	106639	potentially variable
PHG9_Scaffold_953	32	p2	(TA)7	14	109939	109952	potentially variable
PHG9_Scaffold_953	34	p2	(AT)6	12	120397	120408	
PHG9_Scaffold_954	3	p2	(AT)7	14	5206	5219	potentially variable

PHG9_Scaffold_954	5	p2	(AT)7	14	12242	12255	potentially variable
PHG9_Scaffold_954	8	p2	(TA)6	12	16221	16232	
PHG9_Scaffold_954	10	p2	(AT)7	14	20857	20870	potentially variable
PHG9_Scaffold_954	12	p2	(AT)6	12	32906	32917	
PHG9_Scaffold_955	5	p2	(TA)7	14	17204	17217	potentially variable
PHG9_Scaffold_955	9	p2	(AT)9	18	26126	26143	potentially variable
PHG9_Scaffold_955	11	p3	(GTT)5	15	27275	27289	potentially variable
PHG9_Scaffold_955	12	p2	(AT)6	12	28201	28212	
PHG9_Scaffold_955	13	p2	(AT)6	12	29220	29231	
PHG9_Scaffold_955	26	p3	(TCT)5	15	75372	75386	potentially variable
PHG9_Scaffold_955	31	p2	(TA)7	14	83936	83949	potentially variable
PHG9_Scaffold_955	38	p2	(TA)6	12	103060	103071	
PHG9_Scaffold_955	43	p2	(TA)9	18	114169	114186	potentially variable
PHG9_Scaffold_955	48	p2	(AT)7	14	130178	130191	potentially variable
PHG9_Scaffold_955	49	p2	(AT)8	16	136083	136098	potentially variable
PHG9_Scaffold_957	2	p2	(AT)6	12	2598	2609	
PHG9_Scaffold_957	4	p3	(AAT)5	15	7711	7725	potentially variable
PHG9_Scaffold_957	12	p2	(AT)8	16	32654	32669	potentially variable

PHG9_Scaffold_957	17	p2	(AT)6	12	39682	39693	
PHG9_Scaffold_957	19	p2	(TA)8	16	44346	44361	potentially variable
PHG9_Scaffold_957	22	p3	(CTA)5	15	48644	48658	potentially variable
PHG9_Scaffold_957	23	p3	(TAT)5	15	49813	49827	potentially variable
PHG9_Scaffold_957	44	p2	(TA)6	12	115956	115967	
PHG9_Scaffold_957	50	p2	(TA)7	14	133567	133580	potentially variable
PHG9_Scaffold_957	62	p2	(TA)6	12	156976	156987	
PHG9_Scaffold_957	63	p3	(ATA)5	15	158365	158379	potentially variable
PHG9_Scaffold_957	68	p3	(CCA)5	15	185454	185468	potentially variable
PHG9_Scaffold_957	70	p2	(TA)6	12	191212	191223	
PHG9_Scaffold_957	71	p2	(AG)6	12	193275	193286	
PHG9_Scaffold_957	76	p3	(TAA)6	18	211217	211234	potentially variable
PHG9_Scaffold_957	80	p3	(AGC)5	15	221887	221901	potentially variable
PHG9_Scaffold_957	83	p2	(AT)9	18	238396	238413	potentially variable
PHG9_Scaffold_957	86	p3	(TCT)5	15	244191	244205	potentially variable
PHG9_Scaffold_957	87	p3	(ATG)5	15	244957	244971	potentially variable
PHG9_Scaffold_958	4	p3	(GGA)5	15	5096	5110	potentially variable
PHG9_Scaffold_959	1	p2	(AT)9	18	332	349	potentially

							variable
PHG9_Scaffold_959	9	p2	(AG)7	14	18711	18724	potentially variable
PHG9_Scaffold_959	16	p3	(AGA)5	15	32747	32761	potentially variable
PHG9_Scaffold_959	18	p2	(TC)7	14	44523	44536	potentially variable
PHG9_Scaffold_960	6	p2	(TA)6	12	11829	11840	
PHG9_Scaffold_960	7	p2	(GA)7	14	13856	13869	potentially variable
PHG9_Scaffold_960	8	p2	(AT)7	14	14765	14778	potentially variable
PHG9_Scaffold_960	14	p2	(GA)7	14	25920	25933	potentially variable
PHG9_Scaffold_960	21	p2	(TA)6	12	31187	31198	
PHG9_Scaffold_960	23	p2	(TA)6	12	37914	37925	
PHG9_Scaffold_960	29	p2	(TA)6	12	48644	48655	
PHG9_Scaffold_961	9	p2	(AT)7	14	14392	14405	potentially variable
PHG9_Scaffold_961	17	p2	(TA)7	14	43788	43801	potentially variable
PHG9_Scaffold_963	3	p2	(AT)7	14	8374	8387	potentially variable
PHG9_Scaffold_963	4	p2	(TA)7	14	8553	8566	potentially variable
PHG9_Scaffold_963	8	p2	(TA)7	14	18746	18759	potentially variable
PHG9_Scaffold_963	11	p2	(TA)7	14	39490	39503	potentially variable
PHG9_Scaffold_964	2	p2	(TA)6	12	2278	2289	

PHG9_Scaffold_964	4	p2	(AT)6	12	7038	7049	
PHG9_Scaffold_964	11	p2	(CT)8	16	30036	30051	potentially variable
PHG9_Scaffold_965	6	p3	(TTG)5	15	11710	11724	potentially variable
PHG9_Scaffold_966	1	p2	(AT)6	12	19067	19078	
PHG9_Scaffold_966	6	p3	(AAT)5	15	35313	35327	potentially variable
PHG9_Scaffold_966	8	p2	(TA)8	16	38193	38208	potentially variable
PHG9_Scaffold_969	1	p3	(CTT)5	15	255	269	potentially variable
PHG9_Scaffold_972	5	p3	(TCA)6	18	19292	19309	potentially variable
PHG9_Scaffold_973	6	p3	(AAT)5	15	14611	14625	potentially variable
PHG9_Scaffold_973	8	p2	(TG)8	16	16788	16803	potentially variable
PHG9_Scaffold_973	12	p2	(AC)6	12	23156	23167	
PHG9_Scaffold_973	13	p2	(TA)6	12	29253	29264	
PHG9_Scaffold_973	19	p2	(AT)7	14	37411	37424	potentially variable
PHG9_Scaffold_973	23	p3	(AGA)5	15	43451	43465	potentially variable
PHG9_Scaffold_974	6	p2	(AT)9	18	42847	42864	potentially variable
PHG9_Scaffold_974	16	p2	(AT)9	18	68491	68508	potentially variable
PHG9_Scaffold_974	18	p2	(CT)6	12	75115	75126	
PHG9_Scaffold_975	5	p2	(AC)7	14	6893	6906	potentially

							variable
PHG9_Scaffold_977	2	p2	(AT)8	16	6163	6178	potentially variable
PHG9_Scaffold_977	3	p2	(TA)7	14	6282	6295	potentially variable
PHG9_Scaffold_977	6	p3	(AAT)5	15	7504	7518	potentially variable
PHG9_Scaffold_977	18	p2	(TA)7	14	86669	86682	potentially variable
PHG9_Scaffold_977	19	p2	(AT)6	12	91137	91148	
PHG9_Scaffold_977	25	p3	(AAT)5	15	121728	121742	potentially variable
PHG9_Scaffold_977	26	p2	(TA)6	12	122031	122042	
PHG9_Scaffold_977	28	p3	(TAA)6	18	135995	136012	potentially variable
PHG9_Scaffold_977	30	p2	(AT)6	12	136682	136693	
PHG9_Scaffold_978	4	p3	(TTA)5	15	6932	6946	potentially variable
PHG9_Scaffold_979	3	p2	(CA)7	14	6467	6480	potentially variable
PHG9_Scaffold_979	7	p2	(AT)6	12	21092	21103	
PHG9_Scaffold_980	2	p2	(GA)6	12	9897	9908	
PHG9_Scaffold_980	8	p3	(AAT)5	15	25288	25302	potentially variable
PHG9_Scaffold_980	10	p2	(TG)8	16	27465	27480	potentially variable
PHG9_Scaffold_981	1	p2	(CT)7	14	1452	1465	potentially variable
PHG9_Scaffold_981	18	p2	(TA)8	16	45933	45948	potentially variable

PHG9_Scaffold_981	23	p2	(AG)6	12	55542	55553	
PHG9_Scaffold_981	43	p2	(CT)7	14	136389	136402	potentially variable
PHG9_Scaffold_981	46	p2	(AT)6	12	137627	137638	
PHG9_Scaffold_983	5	p2	(AT)9	18	15679	15696	potentially variable
PHG9_Scaffold_983	8	p3	(TAA)6	18	33566	33583	potentially variable
PHG9_Scaffold_983	12	p3	(AAT)5	15	45442	45456	potentially variable
PHG9_Scaffold_985	2	p2	(AT)8	16	1093	1108	potentially variable
PHG9_Scaffold_986	5	p3	(GTT)5	15	17228	17242	potentially variable
PHG9_Scaffold_986	20	p2	(GT)6	12	58177	58188	
PHG9_Scaffold_988	4	p2	(AT)7	14	5783	5796	potentially variable
PHG9_Scaffold_988	9	p2	(CT)8	16	25847	25862	potentially variable
PHG9_Scaffold_989	9	p2	(AT)6	12	56559	56570	
PHG9_Scaffold_989	18	p2	(TA)7	14	75554	75567	potentially variable
PHG9_Scaffold_989	19	p3	(ATA)6	18	75956	75973	potentially variable
PHG9_Scaffold_989	20	p2	(TC)9	18	78787	78804	potentially variable
PHG9_Scaffold_989	22	p3	(TAA)5	15	82826	82840	potentially variable
PHG9_Scaffold_989	24	p2	(AT)6	12	85892	85903	
PHG9_Scaffold_991	5	p3	(GAT)5	15	9952	9966	potentially

							variable
PHG9_Scaffold_991	6	p3	(AAT)6	18	10506	10523	potentially variable
PHG9_Scaffold_991	9	p3	(AGA)6	18	17575	17592	potentially variable
PHG9_Scaffold_991	10	p3	(TAT)6	18	18042	18059	potentially variable
PHG9_Scaffold_991	11	p2	(TA)7	14	18489	18502	potentially variable
PHG9_Scaffold_991	13	p2	(TG)8	16	24987	25002	potentially variable
PHG9_Scaffold_991	15	p2	(AT)6	12	34859	34870	
PHG9_Scaffold_991	16	p2	(TA)6	12	35571	35582	
PHG9_Scaffold_991	19	p2	(TC)8	16	43867	43882	potentially variable
PHG9_Scaffold_991	21	p2	(AT)6	12	47424	47435	
PHG9_Scaffold_991	29	p3	(ATA)5	15	66739	66753	potentially variable
PHG9_Scaffold_991	31	p2	(TA)6	12	78197	78208	
PHG9_Scaffold_991	36	p2	(TA)6	12	85162	85173	
PHG9_Scaffold_991	40	p3	(ATT)5	15	100764	100778	potentially variable
PHG9_Scaffold_991	41	p2	(TC)7	14	101488	101501	potentially variable
PHG9_Scaffold_991	43	p2	(GT)9	18	102102	102119	potentially variable
PHG9_Scaffold_991	44	p2	(CT)6	12	116785	116796	
PHG9_Scaffold_991	55	p2	(AG)6	12	136516	136527	
PHG9_Scaffold_991	56	p2	(AT)9	18	143478	143495	potentially

							variable
PHG9_Scaffold_991	58	p2	(CT)9	18	145003	145020	potentially variable
PHG9_Scaffold_991	60	p2	(TA)8	16	151218	151233	potentially variable
PHG9_Scaffold_992	1	p2	(AG)7	14	2207	2220	potentially variable
PHG9_Scaffold_992	4	p3	(TGG)6	18	6251	6268	potentially variable
PHG9_Scaffold_993	9	p3	(TAT)6	18	27742	27759	potentially variable
PHG9_Scaffold_993	10	p2	(AC)7	14	28173	28186	potentially variable
PHG9_Scaffold_993	19	p2	(TA)8	16	69193	69208	potentially variable
PHG9_Scaffold_993	20	p3	(TAT)5	15	70369	70383	potentially variable
PHG9_Scaffold_993	23	p3	(AAT)5	15	72193	72207	potentially variable
PHG9_Scaffold_993	47	p2	(CT)9	18	134548	134565	potentially variable
PHG9_Scaffold_993	50	p2	(TA)6	12	137499	137510	
PHG9_Scaffold_993	51	p2	(AT)7	14	138066	138079	potentially variable
PHG9_Scaffold_993	52	p2	(TG)6	12	140472	140483	
PHG9_Scaffold_993	59	p2	(TC)6	12	156690	156701	
PHG9_Scaffold_993	61	p2	(AT)7	14	157799	157812	potentially variable
PHG9_Scaffold_993	62	p2	(AT)6	12	163539	163550	
PHG9_Scaffold_996	2	p2	(TA)6	12	1347	1358	

PHG9_Scaffold_996	4	p2	(TC)8	16	2692	2707	potentially variable
PHG9_Scaffold_996	14	p2	(AG)6	12	48988	48999	
PHG9_Scaffold_996	20	p2	(AT)7	14	61920	61933	potentially variable
PHG9_Scaffold_996	21	p2	(AT)8	16	66559	66574	potentially variable
PHG9_Scaffold_1000	2	p2	(TA)6	12	1251	1262	
PHG9_Scaffold_1000	3	p2	(AG)6	12	4973	4984	
PHG9_Scaffold_1000	4	p2	(CT)7	14	6348	6361	potentially variable
PHG9_Scaffold_1001	5	p2	(TA)6	12	45690	45701	
PHG9_Scaffold_1002	3	p3	(TGT)5	15	1877	1891	potentially variable
PHG9_Scaffold_1002	27	p2	(AT)7	14	99066	99079	potentially variable
PHG9_Scaffold_1004	15	p3	(ATT)5	15	119492	119506	potentially variable
PHG9_Scaffold_1004	25	p2	(AT)6	12	205184	205195	
PHG9_Scaffold_1005	4	p2	(AG)7	14	41726	41739	potentially variable
PHG9_Scaffold_1006	7	p3	(CAT)5	15	15219	15233	potentially variable
PHG9_Scaffold_1008	2	p2	(AT)6	12	6671	6682	
PHG9_Scaffold_1008	7	p2	(AT)7	14	18507	18520	potentially variable
PHG9_Scaffold_1008	12	p2	(TC)8	16	30953	30968	potentially variable
PHG9_Scaffold_1008	22	p2	(TA)6	12	59009	59020	

PHG9_Scaffold_1008	26	p3	(TCT)6	18	70506	70523	potentially variable
PHG9_Scaffold_1008	30	p3	(AGA)5	15	82991	83005	potentially variable
PHG9_Scaffold_1009	3	p2	(AT)8	16	19630	19645	potentially variable
PHG9_Scaffold_1009	7	p3	(ATC)5	15	27664	27678	potentially variable
PHG9_Scaffold_1009	10	p2	(TC)7	14	34394	34407	potentially variable
PHG9_Scaffold_1009	11	p2	(TC)7	14	44091	44104	potentially variable
PHG9_Scaffold_1010	2	p2	(AT)6	12	7472	7483	
PHG9_Scaffold_1010	7	p3	(ATA)5	15	33355	33369	potentially variable
PHG9_Scaffold_1011	5	p2	(TA)9	18	15861	15878	potentially variable
PHG9_Scaffold_1012	30	p2	(GA)7	14	91838	91851	potentially variable
PHG9_Scaffold_1012	31	p2	(AT)6	12	93064	93075	
PHG9_Scaffold_1013	1	p2	(TA)6	12	120	131	
PHG9_Scaffold_1013	19	p2	(TC)6	12	57609	57620	
PHG9_Scaffold_1013	22	p3	(CCT)5	15	67836	67850	potentially variable
PHG9_Scaffold_1013	32	p3	(TTC)5	15	101066	101080	potentially variable
PHG9_Scaffold_1013	38	p3	(GAA)5	15	115814	115828	potentially variable
PHG9_Scaffold_1013	43	p2	(TA)6	12	128291	128302	
PHG9_Scaffold_1013	44	p2	(AT)6	12	128781	128792	

PHG9_Scaffold_1014	2	p2	(AG)8	16	351	366	potentially variable
PHG9_Scaffold_1014	4	p2	(AT)8	16	12801	12816	potentially variable
PHG9_Scaffold_1014	10	p3	(ATT)5	15	30299	30313	potentially variable
PHG9_Scaffold_1014	11	p3	(CCA)5	15	42894	42908	potentially variable
PHG9_Scaffold_1014	15	p3	(GAA)5	15	57181	57195	potentially variable
PHG9_Scaffold_1014	16	p3	(AAT)6	18	66695	66712	potentially variable
PHG9_Scaffold_1014	17	p2	(AT)9	18	67411	67428	potentially variable
PHG9_Scaffold_1014	21	p3	(TGA)5	15	80494	80508	potentially variable
PHG9_Scaffold_1014	24	p3	(CAT)5	15	88764	88778	potentially variable
PHG9_Scaffold_1015	8	p2	(TA)8	16	27728	27743	potentially variable
PHG9_Scaffold_1017	5	p2	(AT)6	12	39495	39506	
PHG9_Scaffold_1020	2	p2	(TA)6	12	18343	18354	
PHG9_Scaffold_1020	6	p6	(CCCATC)5	30	26276	26305	Hypervariable
PHG9_Scaffold_1021	4	p2	(TA)7	14	9149	9162	potentially variable
PHG9_Scaffold_1023	1	p2	(AT)6	12	1648	1659	
PHG9_Scaffold_1024	7	p2	(TC)7	14	25354	25367	potentially variable
PHG9_Scaffold_1025	16	p2	(AT)7	14	83335	83348	potentially variable

PHG9_Scaffold_1025	23	p2	(AT)6	12	108100	108111	
PHG9_Scaffold_1026	33	p3	(CAT)5	15	61178	61192	potentially variable
PHG9_Scaffold_1026	38	p2	(AT)6	12	75120	75131	
PHG9_Scaffold_1026	54	p2	(AT)7	14	116803	116816	potentially variable
PHG9_Scaffold_1027	3	p3	(CAA)5	15	9390	9404	potentially variable
PHG9_Scaffold_1027	14	p3	(CTT)5	15	33121	33135	potentially variable
PHG9_Scaffold_1027	17	p2	(TC)6	12	50801	50812	
PHG9_Scaffold_1027	28	p2	(TA)6	12	86413	86424	
PHG9_Scaffold_1027	30	p2	(TA)7	14	95224	95237	potentially variable
PHG9_Scaffold_1028	8	p3	(TAT)5	15	19994	20008	potentially variable
PHG9_Scaffold_1030	6	p3	(ATA)6	18	17590	17607	potentially variable
PHG9_Scaffold_1031	14	p2	(TA)6	12	40558	40569	
PHG9_Scaffold_1031	20	p2	(AT)6	12	60941	60952	
PHG9_Scaffold_1031	21	p2	(AT)6	12	62345	62356	
PHG9_Scaffold_1031	23	p2	(TA)6	12	66091	66102	
PHG9_Scaffold_1031	24	p2	(AG)8	16	79475	79490	potentially variable
PHG9_Scaffold_1032	5	p2	(AT)6	12	4538	4549	
PHG9_Scaffold_1032	6	p2	(TA)8	16	13071	13086	potentially variable
PHG9_Scaffold_1032	10	p2	(AT)9	18	23273	23290	potentially variable

PHG9_Scaffold_1032	18	p2	(AT)9	18	64237	64254	potentially variable
PHG9_Scaffold_1032	23	p3	(ATA)5	15	66418	66432	potentially variable
PHG9_Scaffold_1032	24	p2	(TA)8	16	68013	68028	potentially variable
PHG9_Scaffold_1032	29	p2	(CT)6	12	80268	80279	
PHG9_Scaffold_1032	31	p2	(TA)6	12	82968	82979	
PHG9_Scaffold_1032	35	p2	(TA)6	12	89539	89550	
PHG9_Scaffold_1033	4	p2	(TA)9	18	8400	8417	potentially variable
PHG9_Scaffold_1033	8	p3	(TCT)5	15	18561	18575	potentially variable
PHG9_Scaffold_1033	9	p2	(TA)7	14	30436	30449	potentially variable
PHG9_Scaffold_1033	14	p2	(TA)6	12	54315	54326	
PHG9_Scaffold_1034	2	p2	(AT)7	14	913	926	potentially variable
PHG9_Scaffold_1034	3	p2	(AG)8	16	8684	8699	potentially variable
PHG9_Scaffold_1034	8	p3	(TTA)5	15	19353	19367	potentially variable
PHG9_Scaffold_1034	11	p2	(TA)6	12	25495	25506	
PHG9_Scaffold_1034	13	p3	(TTC)5	15	40726	40740	potentially variable
PHG9_Scaffold_1034	14	p2	(AT)6	12	45962	45973	
PHG9_Scaffold_1035	19	p2	(AT)9	18	50599	50616	potentially variable
PHG9_Scaffold_1035	26	p2	(TA)8	16	59780	59795	potentially variable

PHG9_Scaffold_1035	46	p2	(AT)6	12	127752	127763	
PHG9_Scaffold_1035	49	p2	(TA)6	12	130757	130768	
PHG9_Scaffold_1035	57	p2	(TA)8	16	150082	150097	potentially variable
PHG9_Scaffold_1035	63	p2	(AT)7	14	164554	164567	potentially variable
PHG9_Scaffold_1035	69	p2	(AG)9	18	177721	177738	potentially variable
PHG9_Scaffold_1035	70	p2	(AT)6	12	178651	178662	
PHG9_Scaffold_1037	6	p2	(TA)9	18	24749	24766	potentially variable
PHG9_Scaffold_1040	2	p3	(GAT)6	18	1185	1202	potentially variable
PHG9_Scaffold_1040	3	p3	(CAG)6	18	1659	1676	potentially variable
PHG9_Scaffold_1040	5	p2	(AT)9	18	4270	4287	potentially variable
PHG9_Scaffold_1040	7	p2	(GT)6	12	5229	5240	
PHG9_Scaffold_1040	9	p2	(TA)6	12	8829	8840	
PHG9_Scaffold_1040	15	p3	(TGC)5	15	21819	21833	potentially variable
PHG9_Scaffold_1040	18	p2	(TA)9	18	24867	24884	potentially variable
PHG9_Scaffold_1040	22	p2	(TC)7	14	46838	46851	potentially variable
PHG9_Scaffold_1040	25	p2	(TA)6	12	56075	56086	
PHG9_Scaffold_1040	31	p2	(GA)7	14	65791	65804	potentially variable
PHG9_Scaffold_1040	34	p2	(AT)6	12	77103	77114	

PHG9_Scaffold_1040	37	p3	(ATA)5	15	85496	85510	potentially variable
PHG9_Scaffold_1042	4	p3	(AGA)5	15	38779	38793	potentially variable
PHG9_Scaffold_1045	1	p2	(TC)6	12	1480	1491	
PHG9_Scaffold_1045	2	p2	(CA)7	14	2417	2430	potentially variable
PHG9_Scaffold_1045	4	p2	(GA)6	12	15630	15641	
PHG9_Scaffold_1045	6	p2	(TA)6	12	23526	23537	
PHG9_Scaffold_1045	18	p2	(TA)6	12	71908	71919	
PHG9_Scaffold_1045	20	p2	(GA)7	14	79909	79922	potentially variable
PHG9_Scaffold_1045	24	p2	(AG)7	14	87762	87775	potentially variable
PHG9_Scaffold_1046	6	p2	(AT)6	12	28434	28445	
PHG9_Scaffold_1046	7	p2	(TA)6	12	28994	29005	
PHG9_Scaffold_1046	8	p2	(TA)6	12	38869	38880	
PHG9_Scaffold_1047	1	p3	(TTA)5	15	7893	7907	potentially variable
PHG9_Scaffold_1047	3	p3	(ATA)6	18	9397	9414	potentially variable
PHG9_Scaffold_1047	8	p3	(TTA)5	15	30920	30934	potentially variable
PHG9_Scaffold_1048	2	p2	(TC)8	16	1882	1897	potentially variable
PHG9_Scaffold_1048	3	p2	(GA)7	14	8016	8029	potentially variable
PHG9_Scaffold_1048	15	p3	(TCT)6	18	43064	43081	potentially variable

PHG9_Scaffold_1048	18	p2	(TA)6	12	48033	48044	
PHG9_Scaffold_1049	2	p2	(AT)7	14	4635	4648	potentially variable
PHG9_Scaffold_1049	5	p2	(TA)6	12	29642	29653	
PHG9_Scaffold_1049	11	p2	(TA)9	18	40291	40308	potentially variable
PHG9_Scaffold_1049	16	p2	(GT)6	12	72690	72701	
PHG9_Scaffold_1049	20	p2	(TA)9	18	80696	80713	potentially variable
PHG9_Scaffold_1049	21	p2	(AT)7	14	82425	82438	potentially variable
PHG9_Scaffold_1050	8	p2	(CT)7	14	20974	20987	potentially variable
PHG9_Scaffold_1050	9	p2	(AT)7	14	23801	23814	potentially variable
PHG9_Scaffold_1050	11	p2	(TA)6	12	32775	32786	
PHG9_Scaffold_1050	18	p3	(TAT)5	15	53351	53365	potentially variable
PHG9_Scaffold_1050	19	p2	(TA)8	16	55209	55224	potentially variable
PHG9_Scaffold_1050	21	p2	(AT)8	16	61201	61216	potentially variable
PHG9_Scaffold_1050	22	p2	(TA)6	12	61517	61528	
PHG9_Scaffold_1050	23	p2	(CT)6	12	62411	62422	
PHG9_Scaffold_1050	29	p2	(AT)7	14	100380	100393	potentially variable
PHG9_Scaffold_1050	30	p2	(TA)7	14	106939	106952	potentially variable
PHG9_Scaffold_1050	34	p3	(ACA)5	15	114445	114459	potentially variable

PHG9_Scaffold_1051	8	p2	(TG)6	12	18950	18961	
PHG9_Scaffold_1051	9	p2	(GT)7	14	19223	19236	potentially variable
PHG9_Scaffold_1051	22	p3	(ATT)5	15	72159	72173	potentially variable
PHG9_Scaffold_1051	29	p2	(AT)9	18	109266	109283	potentially variable
PHG9_Scaffold_1054	7	p3	(CAA)5	15	8331	8345	potentially variable
PHG9_Scaffold_1054	14	p2	(AT)9	18	26135	26152	potentially variable
PHG9_Scaffold_1054	15	p2	(TA)9	18	30355	30372	potentially variable
PHG9_Scaffold_1054	22	p2	(GT)6	12	48604	48615	
PHG9_Scaffold_1054	23	p2	(AG)8	16	54928	54943	potentially variable
PHG9_Scaffold_1056	3	p2	(TA)6	12	42582	42593	
PHG9_Scaffold_1057	3	p2	(AG)9	18	7945	7962	potentially variable
PHG9_Scaffold_1057	5	p2	(TA)6	12	32831	32842	
PHG9_Scaffold_1057	13	p3	(AAT)5	15	73620	73634	potentially variable
PHG9_Scaffold_1057	18	p2	(AT)7	14	104556	104569	potentially variable
PHG9_Scaffold_1058	3	p3	(ATT)5	15	52157	52171	potentially variable
PHG9_Scaffold_1059	12	p3	(ATG)5	15	43344	43358	potentially variable
PHG9_Scaffold_1059	13	p2	(AG)6	12	43775	43786	
PHG9_Scaffold_1059	16	p2	(AT)9	18	48486	48503	potentially

							variable
PHG9_Scaffold_1060	1	p2	(AT)7	14	956	969	potentially variable
PHG9_Scaffold_1060	7	p3	(CAA)5	15	24786	24800	potentially variable
PHG9_Scaffold_1062	14	p2	(GA)6	12	22671	22682	
PHG9_Scaffold_1062	17	p2	(AG)7	14	30029	30042	potentially variable
PHG9_Scaffold_1062	19	p2	(TA)9	18	32342	32359	potentially variable
PHG9_Scaffold_1062	21	p2	(AT)7	14	33333	33346	potentially variable
PHG9_Scaffold_1065	1	p2	(TA)8	16	3426	3441	potentially variable
PHG9_Scaffold_1065	2	p2	(TG)7	14	4467	4480	potentially variable
PHG9_Scaffold_1065	7	p2	(TA)6	12	11448	11459	
PHG9_Scaffold_1066	6	p3	(AAT)6	18	24689	24706	potentially variable
PHG9_Scaffold_1066	7	p2	(AG)9	18	25677	25694	potentially variable
PHG9_Scaffold_1066	13	p2	(AC)9	18	33373	33390	potentially variable
PHG9_Scaffold_1066	14	p2	(TA)6	12	37442	37453	
PHG9_Scaffold_1066	17	p2	(TA)6	12	42718	42729	
PHG9_Scaffold_1070	11	p2	(TA)7	14	27322	27335	potentially variable
PHG9_Scaffold_1070	17	p2	(AT)8	16	56387	56402	potentially variable
PHG9_Scaffold_1070	24	p3	(ATG)6	18	83692	83709	potentially

							variable
PHG9_Scaffold_1070	31	p2	(TG)8	16	116201	116216	potentially variable
PHG9_Scaffold_1070	33	p2	(TC)6	12	121084	121095	
PHG9_Scaffold_1070	35	p2	(AT)7	14	129208	129221	potentially variable
PHG9_Scaffold_1071	1	p2	(CA)6	12	2255	2266	
PHG9_Scaffold_1072	4	p2	(CT)7	14	10213	10226	potentially variable
PHG9_Scaffold_1072	7	p2	(AG)6	12	13366	13377	
PHG9_Scaffold_1072	13	p2	(AT)7	14	32592	32605	potentially variable
PHG9_Scaffold_1072	15	p2	(TC)6	12	33803	33814	
PHG9_Scaffold_1072	17	p2	(CA)8	16	35651	35666	potentially variable
PHG9_Scaffold_1072	24	p2	(TC)7	14	63395	63408	potentially variable
PHG9_Scaffold_1072	35	p2	(TA)8	16	96094	96109	potentially variable
PHG9_Scaffold_1072	43	p2	(CT)6	12	116182	116193	
PHG9_Scaffold_1072	48	p2	(TA)6	12	129721	129732	
PHG9_Scaffold_1072	51	p2	(AT)6	12	138905	138916	
PHG9_Scaffold_1072	57	p2	(AT)6	12	147552	147563	
PHG9_Scaffold_1072	58	p2	(TC)6	12	150910	150921	
PHG9_Scaffold_1072	60	p3	(GAT)5	15	156873	156887	potentially variable
PHG9_Scaffold_1072	65	p2	(AG)8	16	167895	167910	potentially variable
PHG9_Scaffold_1072	66	p2	(TA)7	14	173453	173466	potentially

							variable
PHG9_Scaffold_1073	3	p2	(AC)9	18	59979	59996	potentially variable
PHG9_Scaffold_1073	5	p2	(TG)6	12	63481	63492	
PHG9_Scaffold_1073	9	p3	(CAC)5	15	72683	72697	potentially variable
PHG9_Scaffold_1074	1	p2	(TA)6	12	50377	50388	
PHG9_Scaffold_1074	7	p2	(AT)6	12	75788	75799	
PHG9_Scaffold_1074	8	p2	(TA)6	12	75904	75915	
PHG9_Scaffold_1075	10	p3	(CAG)5	15	37622	37636	potentially variable
PHG9_Scaffold_1075	11	p2	(CA)6	12	37926	37937	
PHG9_Scaffold_1076	1	p3	(TTA)5	15	70	84	potentially variable
PHG9_Scaffold_1076	6	p2	(TA)6	12	25872	25883	
PHG9_Scaffold_1077	2	p2	(AT)7	14	1656	1669	potentially variable
PHG9_Scaffold_1079	2	p2	(TA)6	12	4858	4869	
PHG9_Scaffold_1079	3	p2	(AG)6	12	5062	5073	
PHG9_Scaffold_1083	1	p2	(TA)6	12	859	870	
PHG9_Scaffold_1083	4	p2	(TA)8	16	7537	7552	potentially variable
PHG9_Scaffold_1083	16	p2	(AT)7	14	48166	48179	potentially variable
PHG9_Scaffold_1083	17	p3	(AAT)5	15	50196	50210	potentially variable
PHG9_Scaffold_1083	21	p3	(ATA)6	18	64951	64968	potentially variable
PHG9_Scaffold_1083	23	p2	(TC)7	14	70295	70308	potentially

							variable
PHG9_Scaffold_1083	32	p2	(GA)6	12	92737	92748	
PHG9_Scaffold_1083	39	p3	(ATA)5	15	118588	118602	potentially variable
PHG9_Scaffold_1083	42	p3	(GTT)5	15	122408	122422	potentially variable
PHG9_Scaffold_1083	61	p2	(TA)6	12	186766	186777	
PHG9_Scaffold_1083	63	p3	(AGA)5	15	188813	188827	potentially variable
PHG9_Scaffold_1083	64	p2	(GT)8	16	189529	189544	potentially variable
PHG9_Scaffold_1083	67	p3	(ATC)6	18	196590	196607	potentially variable
PHG9_Scaffold_1083	70	p3	(GGA)5	15	217142	217156	potentially variable
PHG9_Scaffold_1083	81	p2	(CT)7	14	253493	253506	potentially variable
PHG9_Scaffold_1083	100	p3	(CAA)5	15	295966	295980	potentially variable
PHG9_Scaffold_1083	110	p2	(AT)8	16	327243	327258	potentially variable
PHG9_Scaffold_1083	112	p3	(TAA)5	15	333301	333315	potentially variable
PHG9_Scaffold_1083	114	p3	(CAT)6	18	337321	337338	potentially variable
PHG9_Scaffold_1083	115	p2	(GT)7	14	341528	341541	potentially variable
PHG9_Scaffold_1083	116	p2	(AG)9	18	346894	346911	potentially variable
PHG9_Scaffold_1085	3	p2	(AT)9	18	19333	19350	potentially

							variable
PHG9_Scaffold_1085	6	p3	(TCA)5	15	33122	33136	potentially variable
PHG9_Scaffold_1087	3	p2	(TA)8	16	4200	4215	potentially variable
PHG9_Scaffold_1087	22	p2	(TA)7	14	60742	60755	potentially variable
PHG9_Scaffold_1087	28	p2	(AG)7	14	76748	76761	potentially variable
PHG9_Scaffold_1087	31	p2	(TA)7	14	78837	78850	potentially variable
PHG9_Scaffold_1087	38	p2	(TA)6	12	100796	100807	
PHG9_Scaffold_1087	42	p2	(TA)6	12	106029	106040	
PHG9_Scaffold_1087	54	p2	(AT)9	18	140574	140591	potentially variable
PHG9_Scaffold_1087	55	p3	(TTA)6	18	140755	140772	potentially variable
PHG9_Scaffold_1087	59	p2	(AT)9	18	157945	157962	potentially variable
PHG9_Scaffold_1087	64	p2	(AT)6	12	192109	192120	
PHG9_Scaffold_1087	74	p2	(AT)6	12	233419	233430	
PHG9_Scaffold_1087	77	p2	(AT)6	12	236564	236575	
PHG9_Scaffold_1089	6	p2	(TA)8	16	9694	9709	potentially variable
PHG9_Scaffold_1089	10	p3	(CAC)5	15	20032	20046	potentially variable
PHG9_Scaffold_1089	14	p2	(TA)6	12	37313	37324	
PHG9_Scaffold_1095	3	p2	(TC)8	16	12404	12419	potentially variable

PHG9_Scaffold_1096	10	p2	(CT)6	12	42536	42547	
PHG9_Scaffold_1097	6	p3	(AAT)5	15	21719	21733	potentially variable
PHG9_Scaffold_1097	19	p2	(AT)6	12	53529	53540	
PHG9_Scaffold_1097	23	p2	(AC)6	12	57641	57652	
PHG9_Scaffold_1097	27	p2	(CA)7	14	63793	63806	potentially variable
PHG9_Scaffold_1097	28	p3	(GCA)5	15	66976	66990	potentially variable
PHG9_Scaffold_1097	29	p2	(AC)8	16	68275	68290	potentially variable
PHG9_Scaffold_1097	39	p2	(TA)9	18	121868	121885	potentially variable
PHG9_Scaffold_1097	44	p3	(TGA)6	18	124578	124595	potentially variable
PHG9_Scaffold_1097	46	p3	(ACC)6	18	126555	126572	potentially variable
PHG9_Scaffold_1097	48	p2	(TA)6	12	131153	131164	
PHG9_Scaffold_1097	52	p2	(AT)7	14	132903	132916	potentially variable
PHG9_Scaffold_1097	54	p2	(GA)8	16	137167	137182	potentially variable
PHG9_Scaffold_1097	56	p2	(TC)6	12	142691	142702	
PHG9_Scaffold_1097	62	p2	(AT)7	14	153453	153466	potentially variable
PHG9_Scaffold_1097	65	p2	(AC)6	12	172995	173006	
PHG9_Scaffold_1098	18	p3	(AAC)5	15	39864	39878	potentially variable
PHG9_Scaffold_1098	23	p3	(TTC)5	15	45818	45832	potentially variable

PHG9_Scaffold_1098	24	p2	(TC)9	18	46235	46252	potentially variable
PHG9_Scaffold_1099	4	p2	(AT)8	16	14117	14132	potentially variable
PHG9_Scaffold_1099	8	p3	(TGG)5	15	29480	29494	potentially variable
PHG9_Scaffold_1101	1	p2	(AT)9	18	36493	36510	potentially variable
PHG9_Scaffold_1101	6	p2	(GA)6	12	109845	109856	
PHG9_Scaffold_1101	7	p2	(GT)7	14	124338	124351	potentially variable
PHG9_Scaffold_1101	8	p2	(TA)6	12	126395	126406	
PHG9_Scaffold_1102	1	p2	(AT)7	14	10977	10990	potentially variable
PHG9_Scaffold_1102	5	p3	(ATC)6	18	28133	28150	potentially variable
PHG9_Scaffold_1103	1	p2	(CT)6	12	219	230	
PHG9_Scaffold_1103	3	p2	(AT)7	14	5465	5478	potentially variable
PHG9_Scaffold_1103	22	p2	(AT)7	14	74232	74245	potentially variable
PHG9_Scaffold_1104	5	p2	(AT)9	18	14254	14271	potentially variable
PHG9_Scaffold_1104	6	p2	(TA)9	18	14684	14701	potentially variable
PHG9_Scaffold_1104	20	p2	(TA)7	14	64835	64848	potentially variable
PHG9_Scaffold_1104	25	p2	(AT)8	16	71328	71343	potentially variable
PHG9_Scaffold_1104	29	p3	(AAT)5	15	78408	78422	potentially

							variable
PHG9_Scaffold_1104	34	p3	(AAC)5	15	94179	94193	potentially variable
PHG9_Scaffold_1105	7	p2	(TA)7	14	29264	29277	potentially variable
PHG9_Scaffold_1108	1	p2	(TA)6	12	40876	40887	
PHG9_Scaffold_1109	5	p3	(ATT)5	15	16558	16572	potentially variable
PHG9_Scaffold_1109	11	p2	(AT)6	12	39192	39203	
PHG9_Scaffold_1109	14	p2	(CT)6	12	77413	77424	
PHG9_Scaffold_1109	17	p2	(AT)7	14	84189	84202	potentially variable
PHG9_Scaffold_1110	14	p2	(AT)8	16	53448	53463	potentially variable
PHG9_Scaffold_1110	16	p2	(GA)7	14	55366	55379	potentially variable
PHG9_Scaffold_1110	19	p2	(TA)7	14	62864	62877	potentially variable
PHG9_Scaffold_1110	20	p2	(GA)7	14	64218	64231	potentially variable
PHG9_Scaffold_1110	28	p3	(AAT)5	15	82599	82613	potentially variable
PHG9_Scaffold_1110	30	p2	(TA)9	18	84804	84821	potentially variable
PHG9_Scaffold_1110	34	p3	(CTT)5	15	94098	94112	potentially variable
PHG9_Scaffold_1111	1	p2	(GT)7	14	3869	3882	potentially variable
PHG9_Scaffold_1111	3	p2	(TA)7	14	8036	8049	potentially variable

PHG9_Scaffold_1111	7	p3	(AGC)6	18	18898	18915	potentially variable
PHG9_Scaffold_1111	22	p2	(GT)6	12	56958	56969	
PHG9_Scaffold_1111	26	p3	(TGG)5	15	62903	62917	potentially variable
PHG9_Scaffold_1114	2	p2	(AT)8	16	15359	15374	potentially variable
PHG9_Scaffold_1114	4	p2	(AG)8	16	20708	20723	potentially variable
PHG9_Scaffold_1114	11	p2	(AT)9	18	52773	52790	potentially variable
PHG9_Scaffold_1115	2	p2	(AT)6	12	4970	4981	
PHG9_Scaffold_1115	3	p2	(AT)7	14	5142	5155	potentially variable
PHG9_Scaffold_1115	12	p2	(GA)8	16	37246	37261	potentially variable
PHG9_Scaffold_1115	15	p2	(TA)9	18	42034	42051	potentially variable
PHG9_Scaffold_1116	1	p2	(TA)6	12	646	657	
PHG9_Scaffold_1116	6	p3	(CTC)6	18	26550	26567	potentially variable
PHG9_Scaffold_1116	7	p2	(TA)7	14	29603	29616	potentially variable
PHG9_Scaffold_1116	10	p2	(GA)6	12	38541	38552	
PHG9_Scaffold_1116	13	p2	(TA)9	18	43046	43063	potentially variable
PHG9_Scaffold_1116	15	p2	(AG)9	18	46406	46423	potentially variable
PHG9_Scaffold_1116	16	p2	(TC)6	12	50019	50030	
PHG9_Scaffold_1116	30	p2	(AT)8	16	82321	82336	potentially

							variable
PHG9_Scaffold_1117	6	p3	(TTA)5	15	16714	16728	potentially variable
PHG9_Scaffold_1117	8	p3	(ATC)6	18	21999	22016	potentially variable
PHG9_Scaffold_1117	19	p3	(AAG)5	15	44114	44128	potentially variable
PHG9_Scaffold_1117	23	p2	(AC)9	18	53124	53141	potentially variable
PHG9_Scaffold_1117	29	p2	(TA)7	14	76988	77001	potentially variable
PHG9_Scaffold_1117	31	p2	(AT)6	12	81393	81404	
PHG9_Scaffold_1117	38	p3	(TCT)5	15	92709	92723	potentially variable
PHG9_Scaffold_1117	42	p3	(GAA)6	18	102388	102405	potentially variable
PHG9_Scaffold_1118	1	p3	(AAT)6	18	832	849	potentially variable
PHG9_Scaffold_1119	19	p2	(TA)6	12	42991	43002	
PHG9_Scaffold_1119	21	p2	(CA)8	16	45851	45866	potentially variable
PHG9_Scaffold_1119	32	p3	(ATA)6	18	73303	73320	potentially variable
PHG9_Scaffold_1119	40	p2	(TA)6	12	102725	102736	
PHG9_Scaffold_1120	9	p2	(TA)9	18	35205	35222	potentially variable
PHG9_Scaffold_1120	10	p2	(TA)6	12	35933	35944	
PHG9_Scaffold_1121	9	p2	(AG)9	18	27529	27546	potentially variable
PHG9_Scaffold_1121	10	p2	(AG)7	14	32207	32220	potentially

								variable
PHG9_Scaffold_1122	5	p2	(AT)6	12	23102	23113		
PHG9_Scaffold_1122	21	p3	(TGA)6	18	81058	81075		potentially variable
PHG9_Scaffold_1122	24	p2	(TA)7	14	85923	85936		potentially variable
PHG9_Scaffold_1122	28	p2	(GA)6	12	90713	90724		
PHG9_Scaffold_1122	29	p2	(TA)6	12	91045	91056		
PHG9_Scaffold_1122	30	p2	(TA)7	14	97148	97161		potentially variable
PHG9_Scaffold_1122	32	p2	(CA)6	12	101651	101662		
PHG9_Scaffold_1122	47	p2	(AT)6	12	154198	154209		
PHG9_Scaffold_1122	51	p2	(TA)9	18	161956	161973		potentially variable
PHG9_Scaffold_1122	52	p3	(CTG)5	15	162485	162499		potentially variable
PHG9_Scaffold_1122	53	p2	(AG)6	12	171461	171472		
PHG9_Scaffold_1123	5	p2	(AT)7	14	9151	9164		potentially variable
PHG9_Scaffold_1123	13	p3	(AAT)6	18	27145	27162		potentially variable
PHG9_Scaffold_1123	14	p3	(GTG)5	15	32922	32936		potentially variable
PHG9_Scaffold_1124	1	p2	(CT)7	14	4853	4866		potentially variable
PHG9_Scaffold_1124	2	p2	(CA)6	12	8784	8795		
PHG9_Scaffold_1124	8	p3	(TAA)5	15	56607	56621		potentially variable
PHG9_Scaffold_1124	15	p2	(AT)9	18	75685	75702		potentially

							variable
PHG9_Scaffold_1124	16	p2	(AG)6	12	76042	76053	
PHG9_Scaffold_1124	19	p2	(TA)6	12	79822	79833	
PHG9_Scaffold_1124	39	p3	(CAT)5	15	123271	123285	potentially variable
PHG9_Scaffold_1124	41	p2	(GT)7	14	127111	127124	potentially variable
PHG9_Scaffold_1124	47	p2	(AG)9	18	134493	134510	potentially variable
PHG9_Scaffold_1124	56	p3	(CTT)5	15	147797	147811	potentially variable
PHG9_Scaffold_1125	1	p2	(AT)8	16	557	572	potentially variable
PHG9_Scaffold_1126	2	p2	(AT)7	14	7950	7963	potentially variable
PHG9_Scaffold_1128	3	p2	(AT)7	14	9343	9356	potentially variable
PHG9_Scaffold_1128	11	p2	(AC)8	16	32141	32156	potentially variable
PHG9_Scaffold_1128	14	p2	(AG)7	14	39129	39142	potentially variable
PHG9_Scaffold_1128	15	p3	(TAA)5	15	41248	41262	potentially variable
PHG9_Scaffold_1128	19	p3	(ATA)5	15	45515	45529	potentially variable
PHG9_Scaffold_1129	4	p2	(TA)6	12	7613	7624	
PHG9_Scaffold_1130	4	p2	(AT)7	14	13423	13436	potentially variable
PHG9_Scaffold_1130	5	p2	(TA)8	16	20900	20915	potentially variable

PHG9_Scaffold_1132	3	p4	(ATAG)5	20	9210	9229	Hypervariable
PHG9_Scaffold_1133	3	p2	(TA)6	12	10129	10140	
PHG9_Scaffold_1133	7	p2	(AT)7	14	16705	16718	potentially variable
PHG9_Scaffold_1133	12	p2	(AT)6	12	48289	48300	
PHG9_Scaffold_1133	27	p2	(AG)6	12	103751	103762	
PHG9_Scaffold_1133	28	p2	(TA)7	14	105129	105142	potentially variable
PHG9_Scaffold_1133	32	p2	(TA)9	18	119428	119445	potentially variable
PHG9_Scaffold_1133	33	p2	(AC)9	18	123721	123738	potentially variable
PHG9_Scaffold_1133	34	p2	(GT)6	12	123889	123900	
PHG9_Scaffold_1133	37	p3	(ATA)5	15	147218	147232	potentially variable
PHG9_Scaffold_1134	6	p2	(AT)7	14	5154	5167	potentially variable
PHG9_Scaffold_1134	11	p2	(TA)6	12	20762	20773	
PHG9_Scaffold_1136	2	p6	(TTTTAA)5	30	9372	9401	Hypervariable
PHG9_Scaffold_1136	4	p2	(AC)7	14	11332	11345	potentially variable
PHG9_Scaffold_1136	14	p2	(AG)6	12	44498	44509	
PHG9_Scaffold_1137	6	p3	(AAT)5	15	17837	17851	potentially variable
PHG9_Scaffold_1137	7	p3	(TCA)5	15	18791	18805	potentially variable
PHG9_Scaffold_1137	11	p2	(TA)7	14	23764	23777	potentially variable
PHG9_Scaffold_1137	17	p2	(AG)7	14	38025	38038	potentially

							variable
PHG9_Scaffold_1138	4	p2	(AC)6	12	12475	12486	
PHG9_Scaffold_1138	11	p2	(AT)7	14	25685	25698	potentially variable
PHG9_Scaffold_1139	5	p2	(TA)8	16	28981	28996	potentially variable
PHG9_Scaffold_1139	6	p2	(TA)9	18	35493	35510	potentially variable
PHG9_Scaffold_1139	7	p2	(TA)9	18	37786	37803	potentially variable
PHG9_Scaffold_1140	4	p2	(AG)6	12	13873	13884	
PHG9_Scaffold_1140	5	p2	(TC)6	12	15576	15587	
PHG9_Scaffold_1140	19	p2	(AT)8	16	33321	33336	potentially variable
PHG9_Scaffold_1141	3	p2	(GA)6	12	15548	15559	
PHG9_Scaffold_1141	5	p3	(CAT)5	15	19621	19635	potentially variable
PHG9_Scaffold_1141	11	p2	(TA)9	18	24094	24111	potentially variable
PHG9_Scaffold_1143	1	p3	(TAT)5	15	530	544	potentially variable
PHG9_Scaffold_1143	2	p3	(TTA)5	15	6231	6245	potentially variable
PHG9_Scaffold_1143	6	p3	(AAT)5	15	17939	17953	potentially variable
PHG9_Scaffold_1143	16	p2	(TA)6	12	42521	42532	
PHG9_Scaffold_1144	3	p2	(TA)6	12	5743	5754	
PHG9_Scaffold_1144	16	p2	(AT)9	18	68327	68344	potentially variable

PHG9_Scaffold_1144	24	p2	(TA)7	14	93124	93137	potentially variable
PHG9_Scaffold_1144	32	p2	(AT)8	16	122176	122191	potentially variable
PHG9_Scaffold_1145	7	p2	(TC)6	12	41000	41011	
PHG9_Scaffold_1145	8	p2	(CA)7	14	46816	46829	potentially variable
PHG9_Scaffold_1145	14	p2	(AT)6	12	67666	67677	
PHG9_Scaffold_1145	15	p3	(ATA)5	15	77567	77581	potentially variable
PHG9_Scaffold_1145	18	p3	(TAA)5	15	93487	93501	potentially variable
PHG9_Scaffold_1145	20	p3	(TAT)5	15	102274	102288	potentially variable
PHG9_Scaffold_1145	21	p2	(AT)8	16	111479	111494	potentially variable
PHG9_Scaffold_1146	2	p2	(GT)6	12	9839	9850	
PHG9_Scaffold_1146	6	p2	(AT)6	12	18939	18950	
PHG9_Scaffold_1146	9	p2	(TA)7	14	51505	51518	potentially variable
PHG9_Scaffold_1147	5	p2	(GA)6	12	7564	7575	
PHG9_Scaffold_1147	10	p2	(AT)7	14	18301	18314	potentially variable
PHG9_Scaffold_1147	18	p2	(TA)6	12	32866	32877	
PHG9_Scaffold_1147	23	p2	(AC)9	18	40814	40831	potentially variable
PHG9_Scaffold_1147	28	p3	(GCT)6	18	62129	62146	potentially variable
PHG9_Scaffold_1147	32	p2	(GT)6	12	73446	73457	

PHG9_Scaffold_1147	38	p2	(AT)8	16	93486	93501	potentially variable
PHG9_Scaffold_1149	12	p2	(TA)9	18	28397	28414	potentially variable
PHG9_Scaffold_1150	7	p2	(CT)6	12	32610	32621	
PHG9_Scaffold_1151	2	p3	(ATT)5	15	10986	11000	potentially variable
PHG9_Scaffold_1152	7	p2	(AT)6	12	13521	13532	
PHG9_Scaffold_1154	2	p3	(CTT)5	15	6619	6633	potentially variable
PHG9_Scaffold_1155	2	p3	(AAT)5	15	8779	8793	potentially variable
PHG9_Scaffold_1155	8	p2	(AG)6	12	33182	33193	
PHG9_Scaffold_1155	10	p3	(GAA)5	15	35943	35957	potentially variable
PHG9_Scaffold_1155	18	p3	(AAT)6	18	58607	58624	potentially variable
PHG9_Scaffold_1155	23	p2	(AT)7	14	71081	71094	potentially variable
PHG9_Scaffold_1155	39	p2	(TA)9	18	110004	110021	potentially variable
PHG9_Scaffold_1155	46	p2	(TC)6	12	126722	126733	
PHG9_Scaffold_1156	3	p2	(TA)6	12	26740	26751	
PHG9_Scaffold_1156	4	p2	(TA)9	18	27040	27057	potentially variable
PHG9_Scaffold_1156	16	p2	(TA)7	14	53691	53704	potentially variable
PHG9_Scaffold_1156	17	p2	(AT)6	12	54039	54050	
PHG9_Scaffold_1156	23	p2	(TA)9	18	67127	67144	potentially variable

PHG9_Scaffold_1156	29	p2	(TA)7	14	80481	80494	potentially variable
PHG9_Scaffold_1156	35	p2	(TC)7	14	108413	108426	potentially variable
PHG9_Scaffold_1156	38	p2	(TA)6	12	110227	110238	
PHG9_Scaffold_1156	39	p2	(TA)6	12	110362	110373	
PHG9_Scaffold_1156	40	p2	(AT)6	12	114800	114811	
PHG9_Scaffold_1156	42	p2	(AT)8	16	127535	127550	potentially variable
PHG9_Scaffold_1156	47	p2	(TA)7	14	158530	158543	potentially variable
PHG9_Scaffold_1156	48	p2	(TA)6	12	163897	163908	
PHG9_Scaffold_1156	59	p2	(TC)6	12	198108	198119	
PHG9_Scaffold_1156	62	p2	(GT)6	12	207787	207798	
PHG9_Scaffold_1156	63	p2	(AT)7	14	214033	214046	potentially variable
PHG9_Scaffold_1156	69	p3	(CTT)5	15	225735	225749	potentially variable
PHG9_Scaffold_1156	72	p2	(AT)7	14	228906	228919	potentially variable
PHG9_Scaffold_1156	85	p3	(AGA)5	15	272633	272647	potentially variable
PHG9_Scaffold_1156	93	p2	(GT)6	12	291087	291098	
PHG9_Scaffold_1156	95	p3	(ATA)5	15	297887	297901	potentially variable
PHG9_Scaffold_1156	104	p2	(AG)8	16	320884	320899	potentially variable
PHG9_Scaffold_1156	114	p2	(TG)6	12	355682	355693	
PHG9_Scaffold_1156	117	p2	(TA)6	12	357804	357815	

PHG9_Scaffold_1157	6	p2	(AT)6	12	33800	33811	
PHG9_Scaffold_1157	7	p3	(GAA)5	15	35208	35222	potentially variable
PHG9_Scaffold_1157	9	p2	(TA)9	18	49515	49532	potentially variable
PHG9_Scaffold_1158	1	p2	(TA)6	12	54	65	
PHG9_Scaffold_1158	10	p2	(AT)9	18	44185	44202	potentially variable
PHG9_Scaffold_1158	14	p2	(TA)9	18	53894	53911	potentially variable
PHG9_Scaffold_1158	15	p2	(CA)6	12	61903	61914	
PHG9_Scaffold_1159	7	p2	(AG)7	14	6336	6349	potentially variable
PHG9_Scaffold_1160	10	p2	(AT)8	16	28654	28669	potentially variable
PHG9_Scaffold_1161	1	p2	(AT)7	14	2136	2149	potentially variable
PHG9_Scaffold_1162	6	p2	(AT)6	12	49584	49595	
PHG9_Scaffold_1164	6	p2	(TA)8	16	33521	33536	potentially variable
PHG9_Scaffold_1164	8	p2	(TA)9	18	39877	39894	potentially variable
PHG9_Scaffold_1167	3	p2	(AT)7	14	18506	18519	potentially variable
PHG9_Scaffold_1168	1	p3	(CTC)5	15	2782	2796	potentially variable
PHG9_Scaffold_1168	2	p2	(AT)6	12	4739	4750	
PHG9_Scaffold_1168	8	p3	(ATT)6	18	22599	22616	potentially variable
PHG9_Scaffold_1168	17	p3	(TAG)6	18	37015	37032	potentially

							variable
PHG9_Scaffold_1171	2	p2	(TA)6	12	1512	1523	
PHG9_Scaffold_1171	12	p2	(AT)6	12	22499	22510	
PHG9_Scaffold_1171	14	p2	(TA)6	12	30614	30625	
PHG9_Scaffold_1171	16	p2	(AT)7	14	39986	39999	potentially variable
PHG9_Scaffold_1171	23	p2	(TA)7	14	59672	59685	potentially variable
PHG9_Scaffold_1172	2	p2	(CT)6	12	5826	5837	
PHG9_Scaffold_1173	11	p2	(TA)6	12	31831	31842	
PHG9_Scaffold_1173	12	p3	(TAT)5	15	33989	34003	potentially variable
PHG9_Scaffold_1173	17	p2	(AT)7	14	40157	40170	potentially variable
PHG9_Scaffold_1173	21	p3	(CAA)5	15	51514	51528	potentially variable
PHG9_Scaffold_1173	22	p2	(TA)6	12	51701	51712	
PHG9_Scaffold_1173	30	p2	(CT)6	12	66501	66512	
PHG9_Scaffold_1173	31	p3	(TTC)5	15	74253	74267	potentially variable
PHG9_Scaffold_1173	34	p2	(TA)7	14	78002	78015	potentially variable
PHG9_Scaffold_1173	41	p2	(TC)8	16	95965	95980	potentially variable
PHG9_Scaffold_1173	46	p2	(TA)6	12	117781	117792	
PHG9_Scaffold_1173	47	p2	(TA)6	12	119120	119131	
PHG9_Scaffold_1173	59	p3	(AAT)6	18	148504	148521	potentially variable
PHG9_Scaffold_1174	4	p2	(AT)7	14	4937	4950	potentially

							variable
PHG9_Scaffold_1174	5	p3	(CTT)6	18	5441	5458	potentially variable
PHG9_Scaffold_1174	11	p3	(CAT)5	15	24301	24315	potentially variable
PHG9_Scaffold_1176	7	p2	(AT)6	12	9325	9336	
PHG9_Scaffold_1176	8	p2	(TA)8	16	10184	10199	potentially variable
PHG9_Scaffold_1176	10	p2	(TG)6	12	11462	11473	
PHG9_Scaffold_1176	23	p2	(GA)7	14	48850	48863	potentially variable
PHG9_Scaffold_1176	25	p2	(AC)6	12	54761	54772	
PHG9_Scaffold_1176	32	p2	(AC)6	12	72301	72312	
PHG9_Scaffold_1176	34	p2	(AT)7	14	84118	84131	potentially variable
PHG9_Scaffold_1176	40	p3	(CAT)5	15	110341	110355	potentially variable
PHG9_Scaffold_1176	48	p2	(CT)9	18	132783	132800	potentially variable
PHG9_Scaffold_1176	51	p2	(AT)6	12	134177	134188	
PHG9_Scaffold_1180	3	p3	(ACC)5	15	4151	4165	potentially variable
PHG9_Scaffold_1180	6	p2	(AT)7	14	20506	20519	potentially variable
PHG9_Scaffold_1181	2	p3	(TGA)6	18	10226	10243	potentially variable
PHG9_Scaffold_1181	4	p2	(TA)6	12	12329	12340	
PHG9_Scaffold_1182	1	p2	(AC)7	14	1094	1107	potentially variable

PHG9_Scaffold_1182	6	p3	(ATA)5	15	17602	17616	potentially variable
PHG9_Scaffold_1182	10	p2	(TA)6	12	20208	20219	
PHG9_Scaffold_1182	12	p2	(TC)7	14	26941	26954	potentially variable
PHG9_Scaffold_1182	13	p2	(AT)6	12	27935	27946	
PHG9_Scaffold_1182	16	p2	(AT)6	12	47507	47518	
PHG9_Scaffold_1182	25	p3	(TTA)5	15	56287	56301	potentially variable
PHG9_Scaffold_1183	2	p2	(TA)6	12	15176	15187	
PHG9_Scaffold_1183	3	p2	(AT)6	12	16237	16248	
PHG9_Scaffold_1183	6	p2	(TA)7	14	18875	18888	potentially variable
PHG9_Scaffold_1184	1	p2	(GT)7	14	2448	2461	potentially variable
PHG9_Scaffold_1184	9	p2	(AT)6	12	38317	38328	
PHG9_Scaffold_1186	4	p2	(TA)6	12	13465	13476	
PHG9_Scaffold_1186	5	p2	(AT)9	18	23676	23693	potentially variable
PHG9_Scaffold_1186	20	p2	(CA)7	14	77695	77708	potentially variable
PHG9_Scaffold_1186	22	p3	(AAT)6	18	79441	79458	potentially variable
PHG9_Scaffold_1187	7	p2	(TG)7	14	15696	15709	potentially variable
PHG9_Scaffold_1187	18	p3	(ATA)5	15	43234	43248	potentially variable
PHG9_Scaffold_1187	25	p2	(TG)7	14	57926	57939	potentially variable

PHG9_Scaffold_1187	29	p2	(CA)6	12	64953	64964	
PHG9_Scaffold_1187	33	p3	(GAA)5	15	78292	78306	potentially variable
PHG9_Scaffold_1187	35	p3	(TTA)6	18	83234	83251	potentially variable
PHG9_Scaffold_1188	6	p2	(TC)8	16	13916	13931	potentially variable
PHG9_Scaffold_1188	14	p3	(CCA)5	15	41637	41651	potentially variable
PHG9_Scaffold_1188	15	p2	(TA)6	12	47646	47657	
PHG9_Scaffold_1188	25	p2	(TG)7	14	73519	73532	potentially variable
PHG9_Scaffold_1188	32	p2	(TG)7	14	91884	91897	potentially variable
PHG9_Scaffold_1188	33	p2	(AT)8	16	97058	97073	potentially variable
PHG9_Scaffold_1188	45	p2	(TA)9	18	128448	128465	potentially variable
PHG9_Scaffold_1189	1	p2	(AT)6	12	7624	7635	
PHG9_Scaffold_1189	4	p2	(TA)6	12	15537	15548	
PHG9_Scaffold_1189	8	p2	(AT)8	16	18234	18249	potentially variable
PHG9_Scaffold_1189	16	p3	(AGA)5	15	35309	35323	potentially variable
PHG9_Scaffold_1192	5	p2	(TA)6	12	6251	6262	
PHG9_Scaffold_1192	21	p3	(CAT)5	15	56439	56453	potentially variable
PHG9_Scaffold_1192	24	p2	(AT)7	14	65499	65512	potentially variable
PHG9_Scaffold_1192	38	p2	(AT)6	12	107678	107689	

PHG9_Scaffold_1192	39	p2	(AT)6	12	110820	110831	
PHG9_Scaffold_1192	44	p2	(AT)6	12	121674	121685	
PHG9_Scaffold_1198	9	p2	(AT)6	12	18174	18185	
PHG9_Scaffold_1198	25	p2	(AT)8	16	68970	68985	potentially variable
PHG9_Scaffold_1198	28	p2	(GA)9	18	71084	71101	potentially variable
PHG9_Scaffold_1198	29	p2	(GA)6	12	73978	73989	
PHG9_Scaffold_1198	33	p3	(GTT)5	15	92424	92438	potentially variable
PHG9_Scaffold_1198	46	p2	(AT)6	12	120155	120166	
PHG9_Scaffold_1198	49	p2	(TA)6	12	122193	122204	
PHG9_Scaffold_1198	55	p2	(CT)6	12	141986	141997	
PHG9_Scaffold_1198	67	p3	(ATT)5	15	176779	176793	potentially variable
PHG9_Scaffold_1199	4	p2	(AT)6	12	17376	17387	
PHG9_Scaffold_1199	5	p2	(AT)9	18	17675	17692	potentially variable
PHG9_Scaffold_1199	6	p2	(AT)7	14	29358	29371	potentially variable
PHG9_Scaffold_1199	7	p6	(GTTTTG)8	48	29827	29874	Hypervariable
PHG9_Scaffold_1199	8	p3	(CAA)5	15	30080	30094	potentially variable
PHG9_Scaffold_1199	10	p2	(AT)7	14	33289	33302	potentially variable
PHG9_Scaffold_1199	12	p2	(TA)6	12	35387	35398	
PHG9_Scaffold_1199	14	p2	(AT)9	18	38038	38055	potentially variable
PHG9_Scaffold_1203	3	p2	(TG)6	12	4923	4934	

PHG9_Scaffold_1203	5	p2	(TA)6	12	6287	6298	
PHG9_Scaffold_1203	8	p3	(AAT)5	15	19245	19259	potentially variable
PHG9_Scaffold_1203	10	p3	(ATG)5	15	20232	20246	potentially variable
PHG9_Scaffold_1203	14	p3	(TGT)5	15	27236	27250	potentially variable
PHG9_Scaffold_1203	20	p3	(GAA)5	15	70099	70113	potentially variable
PHG9_Scaffold_1207	2	p2	(CT)8	16	3702	3717	potentially variable
PHG9_Scaffold_1207	11	p2	(TA)7	14	11364	11377	potentially variable
PHG9_Scaffold_1207	17	p3	(TTC)5	15	22857	22871	potentially variable
PHG9_Scaffold_1207	18	p2	(TA)6	12	26335	26346	
PHG9_Scaffold_1207	19	p3	(TAT)6	18	29096	29113	potentially variable
PHG9_Scaffold_1208	5	p2	(AT)7	14	24610	24623	potentially variable
PHG9_Scaffold_1208	16	p2	(AT)6	12	81656	81667	
PHG9_Scaffold_1208	17	p2	(TA)7	14	95621	95634	potentially variable
PHG9_Scaffold_1210	1	p3	(AAG)5	15	1588	1602	potentially variable
PHG9_Scaffold_1211	3	p2	(CT)6	12	16056	16067	
PHG9_Scaffold_1213	1	p2	(TA)8	16	1131	1146	potentially variable
PHG9_Scaffold_1214	1	p2	(AC)6	12	3275	3286	
PHG9_Scaffold_1215	2	p2	(TA)6	12	1427	1438	

PHG9_Scaffold_1215	9	p2	(AG)6	12	12570	12581	
PHG9_Scaffold_1215	14	p2	(GA)7	14	34047	34060	potentially variable
PHG9_Scaffold_1215	18	p2	(CT)6	12	43371	43382	
PHG9_Scaffold_1215	23	p2	(TA)6	12	53512	53523	
PHG9_Scaffold_1215	24	p2	(AG)6	12	54155	54166	
PHG9_Scaffold_1219	6	p3	(TTC)5	15	28688	28702	potentially variable
PHG9_Scaffold_1219	12	p2	(TA)6	12	50235	50246	
PHG9_Scaffold_1222	1	p2	(AT)9	18	30	47	potentially variable
PHG9_Scaffold_1222	31	p2	(TA)7	14	121473	121486	potentially variable
PHG9_Scaffold_1223	19	p2	(TA)6	12	41996	42007	
PHG9_Scaffold_1224	12	p2	(AT)6	12	17646	17657	
PHG9_Scaffold_1224	14	p2	(CT)6	12	18469	18480	
PHG9_Scaffold_1224	22	p2	(AT)8	16	34108	34123	potentially variable
PHG9_Scaffold_1224	23	p2	(AG)6	12	34617	34628	
PHG9_Scaffold_1224	24	p2	(AC)6	12	34859	34870	
PHG9_Scaffold_1224	27	p2	(GA)6	12	38449	38460	
PHG9_Scaffold_1225	3	p2	(AG)9	18	1047	1064	potentially variable
PHG9_Scaffold_1225	18	p2	(TA)9	18	35160	35177	potentially variable
PHG9_Scaffold_1226	7	p2	(TA)6	12	16155	16166	
PHG9_Scaffold_1230	4	p2	(TA)6	12	11803	11814	
PHG9_Scaffold_1230	7	p3	(TGA)5	15	13763	13777	potentially variable

PHG9_Scaffold_1230	8	p2	(TA)6	12	17652	17663	
PHG9_Scaffold_1230	12	p2	(TA)7	14	22895	22908	potentially variable
PHG9_Scaffold_1230	15	p2	(TA)7	14	26947	26960	potentially variable
PHG9_Scaffold_1231	3	p2	(AT)9	18	7788	7805	potentially variable
PHG9_Scaffold_1231	6	p2	(AT)7	14	16084	16097	potentially variable
PHG9_Scaffold_1231	7	p2	(GA)6	12	36711	36722	
PHG9_Scaffold_1231	8	p3	(GAA)5	15	36833	36847	potentially variable
PHG9_Scaffold_1231	13	p3	(TAT)5	15	46128	46142	potentially variable
PHG9_Scaffold_1232	2	p2	(AT)6	12	7709	7720	
PHG9_Scaffold_1232	4	p2	(CA)6	12	14845	14856	
PHG9_Scaffold_1232	9	p2	(AT)8	16	31575	31590	potentially variable
PHG9_Scaffold_1232	10	p2	(AG)8	16	36338	36353	potentially variable
PHG9_Scaffold_1232	12	p2	(AT)8	16	45359	45374	potentially variable
PHG9_Scaffold_1232	13	p2	(AT)7	14	46541	46554	potentially variable
PHG9_Scaffold_1232	14	p2	(CA)7	14	47526	47539	potentially variable
PHG9_Scaffold_1232	18	p2	(GA)8	16	57025	57040	potentially variable
PHG9_Scaffold_1232	20	p2	(TA)9	18	72853	72870	potentially variable

PHG9_Scaffold_1232	23	p2	(TA)6	12	81247	81258	
PHG9_Scaffold_1232	28	p3	(ATT)5	15	89867	89881	potentially variable
PHG9_Scaffold_1232	33	p2	(AT)9	18	96583	96600	potentially variable
PHG9_Scaffold_1232	35	p2	(TA)8	16	98771	98786	potentially variable
PHG9_Scaffold_1232	36	p2	(AT)8	16	103540	103555	potentially variable
PHG9_Scaffold_1232	41	p2	(AT)7	14	115841	115854	potentially variable
PHG9_Scaffold_1232	64	p2	(TA)6	12	168654	168665	
PHG9_Scaffold_1232	74	p2	(TC)7	14	205862	205875	potentially variable
PHG9_Scaffold_1233	1	p2	(TA)6	12	1	12	
PHG9_Scaffold_1233	3	p3	(ATT)5	15	6329	6343	potentially variable
PHG9_Scaffold_1233	10	p2	(TA)8	16	27203	27218	potentially variable
PHG9_Scaffold_1233	13	p2	(TA)6	12	33986	33997	
PHG9_Scaffold_1233	21	p3	(GAG)5	15	78871	78885	potentially variable
PHG9_Scaffold_1233	22	p2	(CT)6	12	79009	79020	
PHG9_Scaffold_1233	23	p3	(TAA)5	15	79689	79703	potentially variable
PHG9_Scaffold_1233	34	p2	(AT)7	14	94685	94698	potentially variable
PHG9_Scaffold_1234	6	p3	(TTG)5	15	13555	13569	potentially variable
PHG9_Scaffold_1234	7	p3	(GAA)6	18	13755	13772	potentially

							variable
PHG9_Scaffold_1234	10	p2	(TA)8	16	37727	37742	potentially variable
PHG9_Scaffold_1235	5	p2	(AG)6	12	29592	29603	
PHG9_Scaffold_1235	6	p2	(AT)8	16	32399	32414	potentially variable
PHG9_Scaffold_1235	9	p2	(AG)6	12	38113	38124	
PHG9_Scaffold_1236	11	p3	(ACG)5	15	36691	36705	potentially variable
PHG9_Scaffold_1236	15	p2	(TA)6	12	42633	42644	
PHG9_Scaffold_1236	23	p3	(TTA)5	15	75693	75707	potentially variable
PHG9_Scaffold_1236	29	p2	(AT)8	16	82892	82907	potentially variable
PHG9_Scaffold_1236	32	p3	(TCA)5	15	101300	101314	potentially variable
PHG9_Scaffold_1236	35	p3	(TCA)5	15	113319	113333	potentially variable
PHG9_Scaffold_1237	3	p2	(AT)7	14	3924	3937	potentially variable
PHG9_Scaffold_1237	7	p2	(AT)6	12	11816	11827	
PHG9_Scaffold_1238	4	p3	(TAT)5	15	15125	15139	potentially variable
PHG9_Scaffold_1238	8	p2	(CT)8	16	22177	22192	potentially variable
PHG9_Scaffold_1238	11	p2	(AG)6	12	31388	31399	
PHG9_Scaffold_1238	14	p2	(AT)6	12	47254	47265	
PHG9_Scaffold_1239	8	p3	(AAG)6	18	20181	20198	potentially variable

PHG9_Scaffold_1239	11	p2	(CT)7	14	23229	23242	potentially variable
PHG9_Scaffold_1239	14	p2	(AT)6	12	33784	33795	
PHG9_Scaffold_1240	3	p2	(TA)7	14	1336	1349	potentially variable
PHG9_Scaffold_1240	4	p2	(TA)8	16	6267	6282	potentially variable
PHG9_Scaffold_1240	8	p2	(TA)6	12	15806	15817	
PHG9_Scaffold_1240	9	p2	(AT)6	12	16478	16489	
PHG9_Scaffold_1240	13	p2	(TA)8	16	32784	32799	potentially variable
PHG9_Scaffold_1240	14	p2	(TA)6	12	41442	41453	
PHG9_Scaffold_1240	17	p3	(TTA)6	18	53151	53168	potentially variable
PHG9_Scaffold_1240	21	p2	(TA)9	18	55935	55952	potentially variable
PHG9_Scaffold_1241	7	p2	(AT)7	14	12972	12985	potentially variable
PHG9_Scaffold_1241	16	p2	(TC)6	12	28634	28645	
PHG9_Scaffold_1241	18	p2	(TA)6	12	43229	43240	
PHG9_Scaffold_1242	8	p2	(TA)9	18	43276	43293	potentially variable
PHG9_Scaffold_1243	3	p3	(TAA)6	18	14913	14930	potentially variable
PHG9_Scaffold_1243	4	p2	(AT)6	12	17731	17742	
PHG9_Scaffold_1243	8	p2	(AT)9	18	27220	27237	potentially variable
PHG9_Scaffold_1244	3	p3	(ATA)6	18	11611	11628	potentially variable

PHG9_Scaffold_1244	7	p2	(AG)7	14	31941	31954	potentially variable
PHG9_Scaffold_1244	17	p2	(CT)7	14	46119	46132	potentially variable
PHG9_Scaffold_1244	25	p2	(TA)7	14	62287	62300	potentially variable
PHG9_Scaffold_1244	27	p3	(AGA)5	15	71604	71618	potentially variable
PHG9_Scaffold_1244	34	p3	(TTC)6	18	99948	99965	potentially variable
PHG9_Scaffold_1244	45	p3	(ATA)5	15	120426	120440	potentially variable
PHG9_Scaffold_1244	52	p2	(AT)6	12	144766	144777	
PHG9_Scaffold_1244	62	p3	(ACA)6	18	167723	167740	potentially variable
PHG9_Scaffold_1244	63	p3	(CCT)5	15	175692	175706	potentially variable
PHG9_Scaffold_1244	71	p2	(AT)6	12	200669	200680	
PHG9_Scaffold_1244	74	p2	(AG)7	14	207515	207528	potentially variable
PHG9_Scaffold_1244	75	p2	(TA)6	12	210138	210149	
PHG9_Scaffold_1244	84	p2	(AG)6	12	251490	251501	
PHG9_Scaffold_1244	86	p2	(TA)6	12	254106	254117	
PHG9_Scaffold_1244	87	p2	(AT)6	12	254998	255009	
PHG9_Scaffold_1244	90	p2	(TA)8	16	263463	263478	potentially variable
PHG9_Scaffold_1244	95	p2	(AT)8	16	276378	276393	potentially variable
PHG9_Scaffold_1244	103	p2	(AT)8	16	292496	292511	potentially variable

PHG9_Scaffold_1244	104	p2	(GA)7	14	297905	297918	potentially variable
PHG9_Scaffold_1244	106	p3	(CAT)5	15	308169	308183	potentially variable
PHG9_Scaffold_1244	109	p2	(GT)6	12	316697	316708	
PHG9_Scaffold_1244	122	p2	(TA)6	12	343507	343518	
PHG9_Scaffold_1245	3	p2	(CT)6	12	24536	24547	
PHG9_Scaffold_1245	5	p2	(CT)6	12	32426	32437	
PHG9_Scaffold_1246	3	p3	(AAT)5	15	27111	27125	potentially variable
PHG9_Scaffold_1246	4	p2	(TA)8	16	27909	27924	potentially variable
PHG9_Scaffold_1247	2	p3	(GCT)5	15	1165	1179	potentially variable
PHG9_Scaffold_1247	8	p2	(AG)8	16	17177	17192	potentially variable
PHG9_Scaffold_1247	13	p2	(TA)6	12	42821	42832	
PHG9_Scaffold_1247	14	p2	(AT)7	14	43260	43273	potentially variable
PHG9_Scaffold_1247	19	p2	(AT)8	16	51941	51956	potentially variable
PHG9_Scaffold_1247	28	p2	(AT)6	12	77836	77847	
PHG9_Scaffold_1247	29	p2	(TC)6	12	79599	79610	
PHG9_Scaffold_1247	30	p3	(GAA)5	15	80206	80220	potentially variable
PHG9_Scaffold_1247	35	p2	(AT)8	16	104096	104111	potentially variable
PHG9_Scaffold_1247	39	p2	(TA)6	12	128436	128447	
PHG9_Scaffold_1248	4	p2	(TA)6	12	8504	8515	

PHG9_Scaffold_1248	29	p3	(ATA)5	15	93418	93432	potentially variable
PHG9_Scaffold_1248	34	p3	(ATA)5	15	100394	100408	potentially variable
PHG9_Scaffold_1248	35	p2	(TC)6	12	101314	101325	
PHG9_Scaffold_1248	39	p3	(AAT)5	15	123482	123496	potentially variable
PHG9_Scaffold_1248	49	p2	(TA)7	14	167171	167184	potentially variable
PHG9_Scaffold_1248	51	p2	(TA)6	12	169916	169927	
PHG9_Scaffold_1248	52	p2	(GT)6	12	173305	173316	
PHG9_Scaffold_1248	54	p3	(GGC)5	15	176530	176544	potentially variable
PHG9_Scaffold_1248	56	p2	(AT)8	16	183034	183049	potentially variable
PHG9_Scaffold_1248	58	p2	(TG)8	16	187371	187386	potentially variable
PHG9_Scaffold_1248	62	p2	(CT)6	12	203155	203166	
PHG9_Scaffold_1248	65	p2	(AT)6	12	219112	219123	
PHG9_Scaffold_1248	69	p3	(CTT)5	15	222377	222391	potentially variable
PHG9_Scaffold_1249	3	p2	(TA)7	14	21461	21474	potentially variable
PHG9_Scaffold_1249	4	p2	(TA)7	14	34496	34509	potentially variable
PHG9_Scaffold_1249	10	p3	(TCT)6	18	96619	96636	potentially variable
PHG9_Scaffold_1252	8	p2	(AT)9	18	27716	27733	potentially variable
PHG9_Scaffold_1254	1	p2	(TC)9	18	9933	9950	potentially

							variable
PHG9_Scaffold_1254	8	p3	(TAA)5	15	36957	36971	potentially variable
PHG9_Scaffold_1254	14	p2	(TA)9	18	52724	52741	potentially variable
PHG9_Scaffold_1254	15	p2	(CT)8	16	56859	56874	potentially variable
PHG9_Scaffold_1255	3	p2	(GA)6	12	13889	13900	
PHG9_Scaffold_1255	7	p3	(CAT)5	15	25929	25943	potentially variable
PHG9_Scaffold_1255	18	p2	(TA)7	14	41601	41614	potentially variable
PHG9_Scaffold_1256	9	p3	(AAG)5	15	16867	16881	potentially variable
PHG9_Scaffold_1259	4	p3	(ATA)6	18	16949	16966	potentially variable
PHG9_Scaffold_1260	11	p3	(ATT)5	15	23789	23803	potentially variable
PHG9_Scaffold_1260	32	p2	(TA)6	12	104891	104902	
PHG9_Scaffold_1260	34	p2	(AT)7	14	113918	113931	potentially variable
PHG9_Scaffold_1260	35	p2	(TA)6	12	118862	118873	
PHG9_Scaffold_1260	37	p2	(GA)7	14	120835	120848	potentially variable
PHG9_Scaffold_1260	42	p2	(AT)6	12	130145	130156	
PHG9_Scaffold_1260	60	p2	(CA)8	16	186676	186691	potentially variable
PHG9_Scaffold_1260	68	p2	(AT)6	12	240118	240129	
PHG9_Scaffold_1260	69	p3	(CTA)5	15	246072	246086	potentially variable

PHG9_Scaffold_1260	76	p2	(AT)7	14	273545	273558	potentially variable
PHG9_Scaffold_1260	79	p2	(CA)9	18	280166	280183	potentially variable
PHG9_Scaffold_1263	14	p2	(AT)6	12	82722	82733	
PHG9_Scaffold_1263	22	p2	(AT)7	14	87112	87125	potentially variable
PHG9_Scaffold_1264	3	p2	(CT)6	12	16982	16993	
PHG9_Scaffold_1264	5	p2	(AT)7	14	30938	30951	potentially variable
PHG9_Scaffold_1264	7	p2	(TA)7	14	37860	37873	potentially variable
PHG9_Scaffold_1265	2	p2	(TA)7	14	2113	2126	potentially variable
PHG9_Scaffold_1265	7	p3	(AAT)6	18	24872	24889	potentially variable
PHG9_Scaffold_1265	8	p3	(ATT)5	15	28733	28747	potentially variable
PHG9_Scaffold_1265	9	p3	(TTA)6	18	30736	30753	potentially variable
PHG9_Scaffold_1266	2	p3	(ATA)5	15	1801	1815	potentially variable
PHG9_Scaffold_1266	4	p2	(AT)7	14	15701	15714	potentially variable
PHG9_Scaffold_1266	5	p2	(TC)6	12	21976	21987	
PHG9_Scaffold_1266	7	p2	(AT)6	12	26362	26373	
PHG9_Scaffold_1266	12	p3	(TAT)5	15	48427	48441	potentially variable
PHG9_Scaffold_1267	2	p2	(AT)6	12	14289	14300	
PHG9_Scaffold_1267	3	p2	(CT)6	12	14755	14766	

PHG9_Scaffold_1267	7	p2	(AT)6	12	39620	39631	
PHG9_Scaffold_1269	4	p2	(AT)6	12	6123	6134	
PHG9_Scaffold_1269	16	p3	(TCA)5	15	37310	37324	potentially variable
PHG9_Scaffold_1269	18	p2	(TA)6	12	37983	37994	
PHG9_Scaffold_1269	19	p3	(TGT)5	15	56121	56135	potentially variable
PHG9_Scaffold_1269	20	p3	(GTT)5	15	57195	57209	potentially variable
PHG9_Scaffold_1269	24	p2	(AT)7	14	71531	71544	potentially variable
PHG9_Scaffold_1270	9	p2	(TA)6	12	31921	31932	
PHG9_Scaffold_1270	12	p2	(GA)7	14	33900	33913	potentially variable
PHG9_Scaffold_1270	14	p2	(AT)6	12	37489	37500	
PHG9_Scaffold_1271	3	p2	(AT)6	12	7799	7810	
PHG9_Scaffold_1271	9	p3	(AAT)5	15	37439	37453	potentially variable
PHG9_Scaffold_1271	10	p3	(CAC)6	18	42100	42117	potentially variable
PHG9_Scaffold_1271	11	p3	(CAT)5	15	42620	42634	potentially variable
PHG9_Scaffold_1272	1	p2	(AT)6	12	488	499	
PHG9_Scaffold_1272	5	p2	(AT)6	12	2737	2748	
PHG9_Scaffold_1272	6	p2	(TA)6	12	2904	2915	
PHG9_Scaffold_1272	10	p3	(GTC)6	18	12938	12955	potentially variable
PHG9_Scaffold_1274	1	p3	(CCA)6	18	4762	4779	potentially variable

PHG9_Scaffold_1274	2	p2	(CT)6	12	6207	6218	
PHG9_Scaffold_1275	3	p2	(AT)8	16	7894	7909	potentially variable
PHG9_Scaffold_1275	5	p3	(AAT)5	15	21328	21342	potentially variable
PHG9_Scaffold_1275	6	p2	(CA)6	12	23328	23339	
PHG9_Scaffold_1277	8	p2	(TC)6	12	23717	23728	
PHG9_Scaffold_1279	1	p3	(AAT)5	15	3524	3538	potentially variable
PHG9_Scaffold_1279	7	p3	(CAA)5	15	12160	12174	potentially variable
PHG9_Scaffold_1280	5	p2	(GA)8	16	10921	10936	potentially variable
PHG9_Scaffold_1280	12	p2	(AT)9	18	37753	37770	potentially variable
PHG9_Scaffold_1280	14	p3	(TAT)5	15	39453	39467	potentially variable
PHG9_Scaffold_1280	18	p2	(AT)6	12	56739	56750	
PHG9_Scaffold_1281	1	p2	(AT)6	12	221	232	
PHG9_Scaffold_1282	2	p2	(TA)9	18	23637	23654	potentially variable
PHG9_Scaffold_1282	18	p2	(AG)8	16	108850	108865	potentially variable
PHG9_Scaffold_1282	20	p2	(AG)8	16	110380	110395	potentially variable
PHG9_Scaffold_1282	23	p2	(TA)7	14	113370	113383	potentially variable
PHG9_Scaffold_1282	28	p3	(TAT)5	15	149423	149437	potentially variable
PHG9_Scaffold_1282	30	p2	(AT)6	12	153453	153464	

PHG9_Scaffold_1282	31	p3	(TAT)5	15	154181	154195	potentially variable
PHG9_Scaffold_1282	36	p2	(AT)6	12	177452	177463	
PHG9_Scaffold_1282	38	p2	(AT)6	12	178183	178194	
PHG9_Scaffold_1282	40	p2	(AT)7	14	184573	184586	potentially variable
PHG9_Scaffold_1284	3	p2	(TC)6	12	4688	4699	
PHG9_Scaffold_1284	4	p3	(CAA)5	15	7149	7163	potentially variable
PHG9_Scaffold_1284	5	p3	(CAA)5	15	7378	7392	potentially variable
PHG9_Scaffold_1284	6	p2	(TA)7	14	10113	10126	potentially variable
PHG9_Scaffold_1284	15	p2	(AG)6	12	26071	26082	
PHG9_Scaffold_1285	1	p2	(AT)7	14	5808	5821	potentially variable
PHG9_Scaffold_1285	9	p3	(TTA)6	18	26553	26570	potentially variable
PHG9_Scaffold_1285	10	p2	(GT)9	18	33790	33807	potentially variable
PHG9_Scaffold_1285	20	p2	(AT)6	12	68453	68464	
PHG9_Scaffold_1285	25	p2	(AT)9	18	100413	100430	potentially variable
PHG9_Scaffold_1286	1	p2	(TC)7	14	4127	4140	potentially variable
PHG9_Scaffold_1286	7	p2	(GA)9	18	37926	37943	potentially variable
PHG9_Scaffold_1287	18	p3	(TTA)5	15	36861	36875	potentially variable
PHG9_Scaffold_1287	24	p2	(GA)6	12	47580	47591	

PHG9_Scaffold_1287	27	p2	(TA)6	12	55665	55676	
PHG9_Scaffold_1287	28	p2	(TA)8	16	56537	56552	potentially variable
PHG9_Scaffold_1287	36	p3	(AAT)5	15	84917	84931	potentially variable
PHG9_Scaffold_1288	2	p2	(TA)6	12	1184	1195	
PHG9_Scaffold_1288	11	p2	(AT)9	18	24589	24606	potentially variable
PHG9_Scaffold_1288	12	p2	(AT)7	14	25618	25631	potentially variable
PHG9_Scaffold_1288	16	p2	(TA)6	12	36883	36894	
PHG9_Scaffold_1288	17	p2	(TC)6	12	37232	37243	
PHG9_Scaffold_1288	22	p2	(AG)8	16	57671	57686	potentially variable
PHG9_Scaffold_1288	25	p2	(TC)6	12	62411	62422	
PHG9_Scaffold_1288	26	p2	(TC)6	12	63041	63052	
PHG9_Scaffold_1289	2	p3	(CTC)6	18	9751	9768	potentially variable
PHG9_Scaffold_1289	5	p2	(AT)7	14	22636	22649	potentially variable
PHG9_Scaffold_1289	10	p2	(TA)6	12	29673	29684	
PHG9_Scaffold_1290	3	p2	(TA)6	12	12524	12535	
PHG9_Scaffold_1290	7	p2	(AT)7	14	29014	29027	potentially variable
PHG9_Scaffold_1291	13	p2	(GT)8	16	36462	36477	potentially variable
PHG9_Scaffold_1292	16	p2	(AT)6	12	80953	80964	
PHG9_Scaffold_1293	5	p3	(TAA)5	15	19290	19304	potentially variable

PHG9_Scaffold_1295	1	p2	(AT)7	14	2944	2957	potentially variable
PHG9_Scaffold_1295	16	p2	(AT)7	14	37056	37069	potentially variable
PHG9_Scaffold_1295	19	p3	(ATA)6	18	45276	45293	potentially variable
PHG9_Scaffold_1295	20	p2	(TA)6	12	58519	58530	
PHG9_Scaffold_1295	23	p2	(TA)6	12	64876	64887	
PHG9_Scaffold_1295	37	p2	(TA)6	12	109437	109448	
PHG9_Scaffold_1296	1	p2	(TA)9	18	364	381	potentially variable
PHG9_Scaffold_1297	1	p2	(AG)7	14	10715	10728	potentially variable
PHG9_Scaffold_1297	4	p2	(AT)6	12	16789	16800	
PHG9_Scaffold_1297	10	p2	(TA)9	18	36147	36164	potentially variable
PHG9_Scaffold_1297	12	p2	(TG)6	12	37305	37316	
PHG9_Scaffold_1297	20	p2	(AT)8	16	48913	48928	potentially variable
PHG9_Scaffold_1297	21	p2	(AT)8	16	50346	50361	potentially variable
PHG9_Scaffold_1297	24	p2	(TC)7	14	59031	59044	potentially variable
PHG9_Scaffold_1297	39	p3	(TTA)5	15	102230	102244	potentially variable
PHG9_Scaffold_1297	41	p2	(AT)6	12	104598	104609	
PHG9_Scaffold_1299	7	p2	(AT)6	12	18558	18569	
PHG9_Scaffold_1300	4	p2	(AT)7	14	17029	17042	potentially variable

PHG9_Scaffold_1301	11	p3	(TTC)5	15	51568	51582	potentially variable
PHG9_Scaffold_1302	8	p3	(TCT)5	15	11859	11873	potentially variable
PHG9_Scaffold_1302	9	p6	(AAGAAA)5	30	16578	16607	Hypervariable
PHG9_Scaffold_1302	17	p2	(CA)6	12	48815	48826	
PHG9_Scaffold_1303	6	p2	(AT)8	16	29118	29133	potentially variable
PHG9_Scaffold_1303	8	p2	(TA)8	16	39135	39150	potentially variable
PHG9_Scaffold_1303	20	p2	(AC)6	12	80714	80725	
PHG9_Scaffold_1303	21	p2	(TA)7	14	83454	83467	potentially variable
PHG9_Scaffold_1303	26	p2	(CA)6	12	102249	102260	
PHG9_Scaffold_1303	28	p2	(TA)9	18	107183	107200	potentially variable
PHG9_Scaffold_1303	30	p2	(CA)8	16	112906	112921	potentially variable
PHG9_Scaffold_1304	1	p2	(AG)6	12	437	448	
PHG9_Scaffold_1304	3	p2	(AT)6	12	2245	2256	
PHG9_Scaffold_1308	2	p2	(TA)6	12	1541	1552	
PHG9_Scaffold_1308	6	p2	(AT)7	14	31350	31363	potentially variable
PHG9_Scaffold_1308	7	p2	(AT)9	18	36708	36725	potentially variable
PHG9_Scaffold_1309	4	p2	(AT)8	16	27097	27112	potentially variable
PHG9_Scaffold_1309	10	p2	(GA)6	12	42107	42118	
PHG9_Scaffold_1311	3	p2	(TA)6	12	38606	38617	

PHG9_Scaffold_1311	12	p3	(ATT)5	15	54972	54986	potentially variable
PHG9_Scaffold_1311	14	p2	(AT)6	12	56050	56061	
PHG9_Scaffold_1313	6	p2	(AT)8	16	23743	23758	potentially variable
PHG9_Scaffold_1313	9	p2	(AC)6	12	27743	27754	
PHG9_Scaffold_1315	7	p2	(CA)6	12	12032	12043	
PHG9_Scaffold_1315	10	p2	(TA)7	14	17819	17832	potentially variable
PHG9_Scaffold_1315	13	p3	(CCT)5	15	23635	23649	potentially variable
PHG9_Scaffold_1315	15	p2	(AT)6	12	28610	28621	
PHG9_Scaffold_1315	23	p2	(TA)6	12	55963	55974	
PHG9_Scaffold_1316	1	p2	(AG)6	12	7034	7045	
PHG9_Scaffold_1316	7	p2	(TC)9	18	16190	16207	potentially variable
PHG9_Scaffold_1316	8	p2	(TA)8	16	16644	16659	potentially variable
PHG9_Scaffold_1317	1	p3	(TTG)5	15	1425	1439	potentially variable
PHG9_Scaffold_1317	10	p2	(AG)6	12	16175	16186	
PHG9_Scaffold_1317	16	p3	(AAG)5	15	27929	27943	potentially variable
PHG9_Scaffold_1317	17	p2	(AT)9	18	32625	32642	potentially variable
PHG9_Scaffold_1317	22	p2	(TA)7	14	61992	62005	potentially variable
PHG9_Scaffold_1317	23	p2	(AT)9	18	64334	64351	potentially variable

PHG9_Scaffold_1317	26	p2	(GA)6	12	69899	69910	
PHG9_Scaffold_1317	34	p2	(AT)8	16	83994	84009	potentially variable
PHG9_Scaffold_1317	35	p2	(CA)6	12	91559	91570	
PHG9_Scaffold_1317	38	p2	(AT)6	12	93506	93517	
PHG9_Scaffold_1317	48	p3	(TAT)5	15	111743	111757	potentially variable
PHG9_Scaffold_1318	1	p2	(TA)7	14	3949	3962	potentially variable
PHG9_Scaffold_1318	4	p3	(AAT)6	18	40790	40807	potentially variable
PHG9_Scaffold_1318	8	p2	(AT)8	16	47406	47421	potentially variable
PHG9_Scaffold_1319	2	p2	(AT)7	14	5702	5715	potentially variable
PHG9_Scaffold_1319	3	p3	(TTA)5	15	13010	13024	potentially variable
PHG9_Scaffold_1320	7	p2	(AG)9	18	9605	9622	potentially variable
PHG9_Scaffold_1320	14	p2	(TA)9	18	66240	66257	potentially variable
PHG9_Scaffold_1320	17	p3	(ATA)5	15	72726	72740	potentially variable
PHG9_Scaffold_1320	18	p2	(AT)9	18	77192	77209	potentially variable
PHG9_Scaffold_1320	20	p2	(TA)8	16	79382	79397	potentially variable
PHG9_Scaffold_1320	25	p2	(AT)6	12	101422	101433	
PHG9_Scaffold_1320	26	p2	(GT)6	12	103260	103271	
PHG9_Scaffold_1320	30	p2	(AC)6	12	122331	122342	

PHG9_Scaffold_1320	33	p2	(TC)7	14	142982	142995	potentially variable
PHG9_Scaffold_1320	36	p2	(TA)8	16	150136	150151	potentially variable
PHG9_Scaffold_1320	43	p2	(TA)7	14	175898	175911	potentially variable
PHG9_Scaffold_1325	3	p2	(CT)6	12	4788	4799	
PHG9_Scaffold_1325	4	p2	(TA)9	18	6352	6369	potentially variable
PHG9_Scaffold_1326	3	p2	(AT)7	14	4544	4557	potentially variable
PHG9_Scaffold_1326	8	p2	(TC)6	12	23290	23301	
PHG9_Scaffold_1326	15	p3	(TAG)5	15	41938	41952	potentially variable
PHG9_Scaffold_1326	16	p2	(AC)7	14	43409	43422	potentially variable
PHG9_Scaffold_1326	29	p2	(AG)7	14	92925	92938	potentially variable
PHG9_Scaffold_1326	34	p2	(TC)8	16	117519	117534	potentially variable
PHG9_Scaffold_1328	2	p2	(TA)6	12	3824	3835	
PHG9_Scaffold_1328	4	p2	(AT)8	16	6632	6647	potentially variable
PHG9_Scaffold_1328	9	p2	(AT)7	14	19735	19748	potentially variable
PHG9_Scaffold_1333	2	p2	(TA)6	12	5487	5498	
PHG9_Scaffold_1333	4	p2	(TA)6	12	8383	8394	
PHG9_Scaffold_1333	15	p2	(AG)7	14	41688	41701	potentially variable
PHG9_Scaffold_1333	20	p2	(TA)8	16	61945	61960	potentially

							variable
PHG9_Scaffold_1333	26	p3	(TAT)6	18	93044	93061	potentially variable
PHG9_Scaffold_1333	28	p2	(TG)6	12	98811	98822	
PHG9_Scaffold_1333	31	p2	(AG)6	12	101629	101640	
PHG9_Scaffold_1333	32	p2	(AC)6	12	102346	102357	
PHG9_Scaffold_1333	35	p2	(CA)9	18	106943	106960	potentially variable
PHG9_Scaffold_1333	44	p2	(AT)7	14	130836	130849	potentially variable
PHG9_Scaffold_1333	53	p3	(TTC)6	18	153337	153354	potentially variable
PHG9_Scaffold_1333	55	p2	(TA)6	12	156172	156183	
PHG9_Scaffold_1333	60	p3	(TTC)5	15	166074	166088	potentially variable
PHG9_Scaffold_1333	62	p2	(AT)8	16	168915	168930	potentially variable
PHG9_Scaffold_1333	69	p2	(GT)7	14	175794	175807	potentially variable
PHG9_Scaffold_1333	70	p3	(CAT)5	15	176114	176128	potentially variable
PHG9_Scaffold_1333	73	p2	(TA)6	12	181303	181314	
PHG9_Scaffold_1333	74	p3	(TGT)5	15	181813	181827	potentially variable
PHG9_Scaffold_1333	79	p2	(TA)6	12	201133	201144	
PHG9_Scaffold_1334	4	p3	(AGA)5	15	26293	26307	potentially variable
PHG9_Scaffold_1335	7	p3	(TTA)5	15	11099	11113	potentially variable

PHG9_Scaffold_1335	8	p2	(AT)6	12	11953	11964	
PHG9_Scaffold_1340	2	p2	(GA)8	16	2746	2761	potentially variable
PHG9_Scaffold_1341	3	p2	(TA)7	14	6606	6619	potentially variable
PHG9_Scaffold_1341	8	p2	(TA)6	12	22169	22180	
PHG9_Scaffold_1341	9	p3	(ATT)5	15	22501	22515	potentially variable
PHG9_Scaffold_1341	11	p2	(AT)6	12	25299	25310	
PHG9_Scaffold_1341	19	p2	(AT)6	12	54789	54800	
PHG9_Scaffold_1343	2	p3	(AAT)6	18	20923	20940	potentially variable
PHG9_Scaffold_1343	6	p2	(TA)6	12	46929	46940	
PHG9_Scaffold_1344	6	p2	(TA)9	18	13466	13483	potentially variable
PHG9_Scaffold_1344	11	p3	(TTA)5	15	31000	31014	potentially variable
PHG9_Scaffold_1346	4	p2	(AT)8	16	2628	2643	potentially variable
PHG9_Scaffold_1346	5	p3	(GGT)5	15	11604	11618	potentially variable
PHG9_Scaffold_1346	7	p2	(TA)7	14	14343	14356	potentially variable
PHG9_Scaffold_1346	13	p2	(TA)8	16	22629	22644	potentially variable
PHG9_Scaffold_1346	20	p3	(AAT)5	15	48676	48690	potentially variable
PHG9_Scaffold_1346	23	p2	(TA)7	14	54410	54423	potentially variable
PHG9_Scaffold_1346	24	p3	(GAA)5	15	63845	63859	potentially

							variable
PHG9_Scaffold_1346	32	p2	(AT)6	12	79557	79568	
PHG9_Scaffold_1347	2	p2	(AT)6	12	2929	2940	
PHG9_Scaffold_1348	2	p2	(TC)9	18	1377	1394	potentially variable
PHG9_Scaffold_1348	3	p2	(TA)6	12	1585	1596	
PHG9_Scaffold_1348	7	p2	(AT)7	14	6068	6081	potentially variable
PHG9_Scaffold_1348	19	p3	(CCA)5	15	18855	18869	potentially variable
PHG9_Scaffold_1348	22	p2	(TA)7	14	36880	36893	potentially variable
PHG9_Scaffold_1348	26	p2	(AT)7	14	43848	43861	potentially variable
PHG9_Scaffold_1348	29	p2	(AT)8	16	47399	47414	potentially variable
PHG9_Scaffold_1348	30	p2	(TA)6	12	52898	52909	
PHG9_Scaffold_1348	36	p2	(AT)9	18	74998	75015	potentially variable
PHG9_Scaffold_1348	43	p2	(TA)6	12	88129	88140	
PHG9_Scaffold_1348	44	p3	(TAT)5	15	88286	88300	potentially variable
PHG9_Scaffold_1351	1	p2	(TG)7	14	1316	1329	potentially variable
PHG9_Scaffold_1351	5	p2	(TA)7	14	8792	8805	potentially variable
PHG9_Scaffold_1358	12	p2	(AT)7	14	27739	27752	potentially variable
PHG9_Scaffold_1364	2	p2	(TA)6	12	4566	4577	

PHG9_Scaffold_1365	2	p2	(AT)7	14	825	838	potentially variable
PHG9_Scaffold_1367	3	p3	(GCG)5	15	14489	14503	potentially variable
PHG9_Scaffold_1368	7	p2	(TA)6	12	12032	12043	
PHG9_Scaffold_1368	10	p2	(TA)8	16	14893	14908	potentially variable
PHG9_Scaffold_1368	12	p2	(TA)7	14	16072	16085	potentially variable
PHG9_Scaffold_1370	8	p3	(GAT)5	15	27526	27540	potentially variable
PHG9_Scaffold_1370	11	p2	(CT)6	12	37179	37190	
PHG9_Scaffold_1370	12	p2	(AT)9	18	41005	41022	potentially variable
PHG9_Scaffold_1370	21	p2	(TA)7	14	62682	62695	potentially variable
PHG9_Scaffold_1370	23	p2	(GT)8	16	69425	69440	potentially variable
PHG9_Scaffold_1370	27	p3	(AAT)6	18	85306	85323	potentially variable
PHG9_Scaffold_1372	5	p2	(TA)9	18	10490	10507	potentially variable
PHG9_Scaffold_1372	7	p2	(TA)6	12	15985	15996	
PHG9_Scaffold_1372	9	p2	(AT)6	12	18692	18703	
PHG9_Scaffold_1373	11	p2	(GA)6	12	35189	35200	
PHG9_Scaffold_1373	14	p2	(TA)6	12	37867	37878	
PHG9_Scaffold_1373	17	p2	(TA)7	14	40194	40207	potentially variable
PHG9_Scaffold_1373	18	p2	(AT)6	12	53253	53264	

PHG9_Scaffold_1373	27	p2	(AT)8	16	101434	101449	potentially variable
PHG9_Scaffold_1374	4	p3	(TAT)5	15	28026	28040	potentially variable
PHG9_Scaffold_1374	5	p2	(AT)6	12	34436	34447	
PHG9_Scaffold_1374	8	p2	(AT)7	14	47455	47468	potentially variable
PHG9_Scaffold_1375	3	p2	(AT)8	16	9040	9055	potentially variable
PHG9_Scaffold_1375	6	p2	(TA)7	14	12227	12240	potentially variable
PHG9_Scaffold_1375	7	p2	(AT)6	12	14367	14378	
PHG9_Scaffold_1375	21	p2	(TA)6	12	34535	34546	
PHG9_Scaffold_1375	23	p2	(TA)9	18	40256	40273	potentially variable
PHG9_Scaffold_1376	1	p3	(TTA)5	15	6953	6967	potentially variable
PHG9_Scaffold_1379	7	p2	(AT)6	12	46136	46147	
PHG9_Scaffold_1379	8	p3	(TAA)5	15	54389	54403	potentially variable
PHG9_Scaffold_1380	2	p2	(AT)9	18	6384	6401	potentially variable
PHG9_Scaffold_1380	3	p2	(TA)7	14	15563	15576	potentially variable
PHG9_Scaffold_1381	3	p2	(AT)8	16	10505	10520	potentially variable
PHG9_Scaffold_1384	2	p3	(CAT)5	15	4512	4526	potentially variable
PHG9_Scaffold_1384	4	p2	(TA)6	12	18235	18246	
PHG9_Scaffold_1385	1	p2	(AT)9	18	3427	3444	potentially

							variable
PHG9_Scaffold_1389	13	p3	(ATA)5	15	36606	36620	potentially variable
PHG9_Scaffold_1389	15	p2	(TA)9	18	40366	40383	potentially variable
PHG9_Scaffold_1389	16	p3	(ATA)5	15	40841	40855	potentially variable
PHG9_Scaffold_1390	1	p2	(AG)6	12	3229	3240	
PHG9_Scaffold_1391	2	p2	(TA)6	12	44800	44811	
PHG9_Scaffold_1392	2	p2	(AT)6	12	1494	1505	
PHG9_Scaffold_1393	14	p2	(TC)7	14	23386	23399	potentially variable
PHG9_Scaffold_1394	3	p2	(TA)8	16	1490	1505	potentially variable
PHG9_Scaffold_1394	4	p3	(ATT)6	18	3012	3029	potentially variable
PHG9_Scaffold_1394	7	p2	(TA)7	14	10176	10189	potentially variable
PHG9_Scaffold_1394	15	p2	(GA)6	12	38181	38192	
PHG9_Scaffold_1394	16	p3	(TAA)5	15	44569	44583	potentially variable
PHG9_Scaffold_1394	19	p2	(AG)7	14	52037	52050	potentially variable
PHG9_Scaffold_1394	22	p2	(AT)6	12	54952	54963	
PHG9_Scaffold_1395	4	p2	(TA)9	18	24295	24312	potentially variable
PHG9_Scaffold_1395	18	p2	(TA)7	14	77839	77852	potentially variable
PHG9_Scaffold_1395	19	p2	(TA)6	12	77981	77992	

PHG9_Scaffold_1395	22	p3	(TGT)5	15	95718	95732	potentially variable
PHG9_Scaffold_1395	23	p2	(TA)6	12	102323	102334	
PHG9_Scaffold_1396	1	p2	(AT)6	12	1754	1765	
PHG9_Scaffold_1396	5	p2	(TA)7	14	9621	9634	potentially variable
PHG9_Scaffold_1396	8	p3	(CCG)5	15	10472	10486	potentially variable
PHG9_Scaffold_1396	10	p2	(AT)8	16	18266	18281	potentially variable
PHG9_Scaffold_1399	1	p2	(AT)9	18	7365	7382	potentially variable
PHG9_Scaffold_1399	2	p2	(AT)6	12	20622	20633	
PHG9_Scaffold_1400	10	p3	(ATA)5	15	14696	14710	potentially variable
PHG9_Scaffold_1400	13	p3	(TGA)5	15	15661	15675	potentially variable
PHG9_Scaffold_1400	16	p3	(ATT)5	15	19006	19020	potentially variable
PHG9_Scaffold_1400	18	p2	(AG)6	12	24231	24242	
PHG9_Scaffold_1400	31	p2	(CT)6	12	70421	70432	
PHG9_Scaffold_1400	32	p2	(TA)6	12	70625	70636	
PHG9_Scaffold_1400	51	p2	(AT)9	18	119451	119468	potentially variable
PHG9_Scaffold_1400	52	p3	(CAT)5	15	124467	124481	potentially variable
PHG9_Scaffold_1400	59	p2	(TC)7	14	135005	135018	potentially variable
PHG9_Scaffold_1400	61	p3	(TAT)6	18	139764	139781	potentially variable

PHG9_Scaffold_1401	9	p2	(TA)8	16	29459	29474	potentially variable
PHG9_Scaffold_1403	3	p2	(TA)7	14	13148	13161	potentially variable
PHG9_Scaffold_1404	1	p2	(CA)6	12	1437	1448	
PHG9_Scaffold_1404	7	p2	(AT)6	12	18384	18395	
PHG9_Scaffold_1404	11	p3	(CCT)5	15	30841	30855	potentially variable
PHG9_Scaffold_1405	3	p2	(AT)6	12	21912	21923	
PHG9_Scaffold_1407	1	p3	(CCA)5	15	8	22	potentially variable
PHG9_Scaffold_1407	2	p2	(AT)8	16	8955	8970	potentially variable
PHG9_Scaffold_1408	2	p2	(AC)6	12	3113	3124	
PHG9_Scaffold_1408	3	p3	(GAA)5	15	3767	3781	potentially variable
PHG9_Scaffold_1409	5	p2	(AT)6	12	15749	15760	
PHG9_Scaffold_1410	7	p2	(TA)6	12	16413	16424	
PHG9_Scaffold_1410	10	p2	(TA)8	16	27073	27088	potentially variable
PHG9_Scaffold_1411	1	p2	(TA)6	12	453	464	
PHG9_Scaffold_1412	4	p2	(TA)6	12	6148	6159	
PHG9_Scaffold_1412	15	p2	(TA)7	14	42063	42076	potentially variable
PHG9_Scaffold_1412	18	p2	(AG)9	18	52833	52850	potentially variable
PHG9_Scaffold_1412	20	p2	(GA)6	12	62765	62776	
PHG9_Scaffold_1412	21	p2	(AT)6	12	72489	72500	
PHG9_Scaffold_1412	23	p2	(AC)6	12	79438	79449	

PHG9_Scaffold_1412	27	p2	(AT)7	14	92188	92201	potentially variable
PHG9_Scaffold_1413	6	p3	(TAA)5	15	14228	14242	potentially variable
PHG9_Scaffold_1413	15	p2	(AT)6	12	44896	44907	
PHG9_Scaffold_1414	5	p2	(GT)6	12	11426	11437	
PHG9_Scaffold_1414	19	p2	(AT)8	16	52884	52899	potentially variable
PHG9_Scaffold_1414	20	p2	(TA)8	16	54346	54361	potentially variable
PHG9_Scaffold_1414	22	p2	(TA)6	12	57444	57455	
PHG9_Scaffold_1414	25	p3	(TTA)5	15	62965	62979	potentially variable
PHG9_Scaffold_1414	30	p2	(GA)6	12	72162	72173	
PHG9_Scaffold_1414	33	p2	(TA)6	12	78888	78899	
PHG9_Scaffold_1414	37	p2	(TA)6	12	88661	88672	
PHG9_Scaffold_1414	38	p3	(ATG)5	15	93325	93339	potentially variable
PHG9_Scaffold_1414	43	p3	(AAT)5	15	105419	105433	potentially variable
PHG9_Scaffold_1414	46	p2	(TA)6	12	107027	107038	
PHG9_Scaffold_1414	69	p3	(AAT)5	15	164825	164839	potentially variable
PHG9_Scaffold_1416	2	p2	(AT)9	18	6571	6588	potentially variable
PHG9_Scaffold_1416	3	p2	(TA)9	18	10800	10817	potentially variable
PHG9_Scaffold_1416	4	p2	(TA)9	18	12279	12296	potentially variable

PHG9_Scaffold_1416	8	p2	(TC)6	12	19289	19300	
PHG9_Scaffold_1416	9	p2	(AT)7	14	25504	25517	potentially variable
PHG9_Scaffold_1416	14	p2	(AT)6	12	39229	39240	
PHG9_Scaffold_1416	16	p3	(CTT)5	15	56899	56913	potentially variable
PHG9_Scaffold_1416	18	p3	(TCT)5	15	62390	62404	potentially variable
PHG9_Scaffold_1416	23	p3	(AAG)6	18	86813	86830	potentially variable
PHG9_Scaffold_1416	27	p2	(TA)7	14	96648	96661	potentially variable
PHG9_Scaffold_1416	32	p3	(AAT)5	15	111716	111730	potentially variable
PHG9_Scaffold_1416	36	p2	(CT)6	12	116566	116577	
PHG9_Scaffold_1417	4	p2	(TA)9	18	5495	5512	potentially variable
PHG9_Scaffold_1417	7	p3	(GAA)5	15	14891	14905	potentially variable
PHG9_Scaffold_1417	13	p2	(TA)8	16	27590	27605	potentially variable
PHG9_Scaffold_1419	1	p3	(TAT)5	15	4	18	potentially variable
PHG9_Scaffold_1419	2	p2	(CA)6	12	1338	1349	
PHG9_Scaffold_1419	3	p3	(CTA)5	15	1709	1723	potentially variable
PHG9_Scaffold_1419	7	p3	(AGA)5	15	20144	20158	potentially variable
PHG9_Scaffold_1419	10	p2	(TA)6	12	33151	33162	
PHG9_Scaffold_1419	22	p3	(CAC)6	18	83890	83907	potentially

							variable
PHG9_Scaffold_1420	4	p2	(AT)8	16	1867	1882	potentially variable
PHG9_Scaffold_1421	2	p3	(CAA)5	15	17853	17867	potentially variable
PHG9_Scaffold_1421	17	p2	(TA)6	12	50339	50350	
PHG9_Scaffold_1422	5	p2	(TC)6	12	28758	28769	
PHG9_Scaffold_1422	8	p2	(TA)8	16	41091	41106	potentially variable
PHG9_Scaffold_1426	9	p2	(TA)6	12	58768	58779	
PHG9_Scaffold_1426	15	p3	(TCT)5	15	76488	76502	potentially variable
PHG9_Scaffold_1428	4	p2	(AT)6	12	8590	8601	
PHG9_Scaffold_1428	5	p2	(TA)6	12	17556	17567	
PHG9_Scaffold_1428	6	p3	(TAA)5	15	23485	23499	potentially variable
PHG9_Scaffold_1429	7	p2	(GA)6	12	17321	17332	
PHG9_Scaffold_1431	1	p2	(AG)6	12	3512	3523	
PHG9_Scaffold_1431	2	p3	(TTC)5	15	4687	4701	potentially variable
PHG9_Scaffold_1431	4	p2	(TA)9	18	13988	14005	potentially variable
PHG9_Scaffold_1431	8	p2	(CA)7	14	30983	30996	potentially variable
PHG9_Scaffold_1431	12	p3	(AGG)5	15	53337	53351	potentially variable
PHG9_Scaffold_1431	16	p3	(ATA)6	18	56883	56900	potentially variable
PHG9_Scaffold_1433	1	p2	(AC)9	18	549	566	potentially

							variable
PHG9_Scaffold_1433	9	p2	(TA)6	12	38889	38900	
PHG9_Scaffold_1434	5	p3	(TTC)5	15	24701	24715	potentially variable
PHG9_Scaffold_1434	6	p2	(TA)7	14	27962	27975	potentially variable
PHG9_Scaffold_1434	13	p3	(ATG)5	15	35736	35750	potentially variable
PHG9_Scaffold_1435	6	p2	(AT)6	12	25436	25447	
PHG9_Scaffold_1435	8	p2	(TA)7	14	33145	33158	potentially variable
PHG9_Scaffold_1436	1	p2	(TA)6	12	992	1003	
PHG9_Scaffold_1436	2	p2	(AC)7	14	2160	2173	potentially variable
PHG9_Scaffold_1436	15	p2	(AT)6	12	37551	37562	
PHG9_Scaffold_1436	16	p2	(TA)6	12	39198	39209	
PHG9_Scaffold_1436	17	p2	(AT)7	14	41167	41180	potentially variable
PHG9_Scaffold_1436	34	p2	(AT)7	14	99758	99771	potentially variable
PHG9_Scaffold_1437	2	p2	(AT)8	16	6072	6087	potentially variable
PHG9_Scaffold_1437	18	p2	(TA)6	12	91820	91831	
PHG9_Scaffold_1437	20	p2	(TA)6	12	97008	97019	
PHG9_Scaffold_1439	1	p3	(AAG)6	18	468	485	potentially variable
PHG9_Scaffold_1439	9	p6	(CGGCGT)5	30	25244	25273	Hypervariable
PHG9_Scaffold_1439	20	p2	(TA)6	12	58430	58441	
PHG9_Scaffold_1441	2	p2	(TA)7	14	3901	3914	potentially

							variable
PHG9_Scaffold_1442	2	p2	(AT)8	16	21362	21377	potentially variable
PHG9_Scaffold_1442	3	p3	(AAT)5	15	22665	22679	potentially variable
PHG9_Scaffold_1442	5	p2	(TA)9	18	25142	25159	potentially variable
PHG9_Scaffold_1442	9	p3	(TTA)5	15	30899	30913	potentially variable
PHG9_Scaffold_1443	3	p2	(CT)6	12	15532	15543	
PHG9_Scaffold_1443	5	p2	(TA)6	12	20281	20292	
PHG9_Scaffold_1444	2	p3	(AAT)5	15	4191	4205	potentially variable
PHG9_Scaffold_1445	9	p2	(TC)6	12	49973	49984	
PHG9_Scaffold_1445	16	p2	(AT)8	16	67522	67537	potentially variable
PHG9_Scaffold_1445	23	p2	(AG)7	14	108108	108121	potentially variable
PHG9_Scaffold_1445	26	p2	(TA)7	14	131800	131813	potentially variable
PHG9_Scaffold_1445	29	p3	(ATA)5	15	138835	138849	potentially variable
PHG9_Scaffold_1445	30	p2	(AT)6	12	150393	150404	
PHG9_Scaffold_1445	33	p2	(AT)6	12	155665	155676	
PHG9_Scaffold_1445	34	p2	(AT)9	18	156902	156919	potentially variable
PHG9_Scaffold_1445	37	p3	(TAA)5	15	170297	170311	potentially variable
PHG9_Scaffold_1445	38	p2	(AT)6	12	179051	179062	

PHG9_Scaffold_1446	3	p3	(TAT)5	15	2576	2590	potentially variable
PHG9_Scaffold_1447	3	p3	(ATT)6	18	5911	5928	potentially variable
PHG9_Scaffold_1447	5	p2	(TA)6	12	7887	7898	
PHG9_Scaffold_1447	16	p2	(TA)8	16	26166	26181	potentially variable
PHG9_Scaffold_1447	17	p2	(CT)6	12	26486	26497	
PHG9_Scaffold_1447	20	p3	(AAT)6	18	42762	42779	potentially variable
PHG9_Scaffold_1447	21	p3	(ATA)6	18	44861	44878	potentially variable
PHG9_Scaffold_1447	25	p3	(AGC)5	15	53572	53586	potentially variable
PHG9_Scaffold_1447	29	p3	(AAT)5	15	58656	58670	potentially variable
PHG9_Scaffold_1447	30	p2	(CT)9	18	62191	62208	potentially variable
PHG9_Scaffold_1448	6	p3	(ACA)5	15	7473	7487	potentially variable
PHG9_Scaffold_1448	7	p2	(AT)6	12	11915	11926	
PHG9_Scaffold_1448	8	p2	(TA)8	16	13324	13339	potentially variable
PHG9_Scaffold_1449	2	p6	(CATTTC)5	30	13918	13947	Hypervariable
PHG9_Scaffold_1449	4	p2	(AT)7	14	22045	22058	potentially variable
PHG9_Scaffold_1449	6	p2	(AT)7	14	27358	27371	potentially variable
PHG9_Scaffold_1450	8	p3	(TAT)6	18	33065	33082	potentially variable

PHG9_Scaffold_1450	10	p2	(AT)8	16	37562	37577	potentially variable
PHG9_Scaffold_1450	12	p2	(AT)7	14	50230	50243	potentially variable
PHG9_Scaffold_1451	3	p2	(AT)9	18	10959	10976	potentially variable
PHG9_Scaffold_1451	11	p3	(TTA)6	18	24313	24330	potentially variable
PHG9_Scaffold_1451	20	p2	(TA)8	16	51334	51349	potentially variable
PHG9_Scaffold_1453	4	p2	(AT)6	12	11280	11291	
PHG9_Scaffold_1454	2	p3	(CTT)5	15	9303	9317	potentially variable
PHG9_Scaffold_1454	5	p2	(GA)6	12	22753	22764	
PHG9_Scaffold_1454	6	p2	(AT)7	14	23071	23084	potentially variable
PHG9_Scaffold_1454	12	p3	(TAA)6	18	42412	42429	potentially variable
PHG9_Scaffold_1455	2	p2	(TA)7	14	6417	6430	potentially variable
PHG9_Scaffold_1456	1	p2	(TA)8	16	8845	8860	potentially variable
PHG9_Scaffold_1456	5	p2	(AT)6	12	23948	23959	
PHG9_Scaffold_1457	4	p3	(GTA)5	15	11525	11539	potentially variable
PHG9_Scaffold_1457	21	p2	(TA)6	12	68112	68123	
PHG9_Scaffold_1460	2	p6	(TAAATC)5	30	11339	11368	Hypervariable
PHG9_Scaffold_1460	5	p2	(AT)7	14	19838	19851	potentially variable
PHG9_Scaffold_1460	12	p2	(TA)7	14	40930	40943	potentially

							variable
PHG9_Scaffold_1460	13	p2	(AT)6	12	42711	42722	
PHG9_Scaffold_1461	9	p2	(TC)8	16	46728	46743	potentially variable
PHG9_Scaffold_1462	4	p3	(CCA)5	15	8491	8505	potentially variable
PHG9_Scaffold_1464	1	p3	(TAT)5	15	4400	4414	potentially variable
PHG9_Scaffold_1464	4	p2	(AT)6	12	8179	8190	
PHG9_Scaffold_1464	13	p2	(CT)6	12	41128	41139	
PHG9_Scaffold_1464	16	p2	(CT)8	16	60599	60614	potentially variable
PHG9_Scaffold_1465	5	p3	(AAT)5	15	3992	4006	potentially variable
PHG9_Scaffold_1465	6	p2	(AT)9	18	9458	9475	potentially variable
PHG9_Scaffold_1465	10	p3	(AAT)5	15	20927	20941	potentially variable
PHG9_Scaffold_1466	2	p2	(TC)6	12	4046	4057	
PHG9_Scaffold_1467	5	p3	(CTG)5	15	23934	23948	potentially variable
PHG9_Scaffold_1468	3	p2	(TA)6	12	5640	5651	
PHG9_Scaffold_1468	5	p2	(TA)8	16	7011	7026	potentially variable
PHG9_Scaffold_1468	7	p3	(GCT)5	15	13727	13741	potentially variable
PHG9_Scaffold_1468	14	p2	(AT)6	12	25622	25633	
PHG9_Scaffold_1468	15	p3	(TTA)5	15	27013	27027	potentially variable

PHG9_Scaffold_1468	26	p2	(TC)6	12	62237	62248	
PHG9_Scaffold_1468	27	p3	(AAT)6	18	63265	63282	potentially variable
PHG9_Scaffold_1468	37	p2	(AT)6	12	90524	90535	
PHG9_Scaffold_1468	38	p3	(ATG)5	15	94330	94344	potentially variable
PHG9_Scaffold_1470	3	p2	(TA)7	14	4005	4018	potentially variable
PHG9_Scaffold_1470	6	p2	(GA)6	12	6810	6821	
PHG9_Scaffold_1470	10	p2	(AG)9	18	12674	12691	potentially variable
PHG9_Scaffold_1470	21	p3	(GAG)5	15	57824	57838	potentially variable
PHG9_Scaffold_1470	28	p2	(GT)6	12	82138	82149	
PHG9_Scaffold_1470	67	p3	(AAC)5	15	204379	204393	potentially variable
PHG9_Scaffold_1470	82	p3	(TAT)5	15	233622	233636	potentially variable
PHG9_Scaffold_1471	5	p3	(ACC)5	15	19664	19678	potentially variable
PHG9_Scaffold_1471	6	p3	(GTT)5	15	20729	20743	potentially variable
PHG9_Scaffold_1471	7	p2	(TA)7	14	23147	23160	potentially variable
PHG9_Scaffold_1472	3	p2	(TA)8	16	15987	16002	potentially variable
PHG9_Scaffold_1472	6	p3	(ATG)5	15	20193	20207	potentially variable
PHG9_Scaffold_1472	7	p2	(AT)6	12	21712	21723	
PHG9_Scaffold_1473	5	p2	(AT)6	12	33374	33385	

PHG9_Scaffold_1473	12	p2	(AC)8	16	49726	49741	potentially variable
PHG9_Scaffold_1473	13	p2	(TC)8	16	50688	50703	potentially variable
PHG9_Scaffold_1473	16	p2	(AT)7	14	63008	63021	potentially variable
PHG9_Scaffold_1473	17	p2	(TA)8	16	65618	65633	potentially variable
PHG9_Scaffold_1474	13	p2	(TA)8	16	45848	45863	potentially variable
PHG9_Scaffold_1474	14	p2	(TA)8	16	50717	50732	potentially variable
PHG9_Scaffold_1474	20	p2	(AT)6	12	56086	56097	
PHG9_Scaffold_1474	22	p2	(AC)8	16	57406	57421	potentially variable
PHG9_Scaffold_1474	26	p2	(TA)7	14	74039	74052	potentially variable
PHG9_Scaffold_1474	36	p2	(TA)6	12	95258	95269	
PHG9_Scaffold_1474	37	p2	(AG)7	14	98558	98571	potentially variable
PHG9_Scaffold_1474	38	p2	(TG)6	12	99231	99242	
PHG9_Scaffold_1474	41	p2	(CA)6	12	115585	115596	
PHG9_Scaffold_1476	1	p2	(TA)6	12	8422	8433	
PHG9_Scaffold_1476	2	p2	(AT)9	18	9312	9329	potentially variable
PHG9_Scaffold_1476	3	p2	(TA)6	12	14473	14484	
PHG9_Scaffold_1476	13	p3	(AAT)5	15	45060	45074	potentially variable
PHG9_Scaffold_1476	26	p3	(AAT)5	15	89768	89782	potentially variable

PHG9_Scaffold_1476	27	p2	(TC)6	12	89988	89999	
PHG9_Scaffold_1476	33	p2	(TA)6	12	103044	103055	
PHG9_Scaffold_1476	37	p3	(TTC)6	18	108042	108059	potentially variable
PHG9_Scaffold_1476	45	p2	(TC)9	18	125574	125591	potentially variable
PHG9_Scaffold_1476	48	p2	(AT)9	18	133403	133420	potentially variable
PHG9_Scaffold_1477	3	p3	(AAT)5	15	8684	8698	potentially variable
PHG9_Scaffold_1479	3	p2	(GA)7	14	4687	4700	potentially variable
PHG9_Scaffold_1480	5	p2	(AG)8	16	7293	7308	potentially variable
PHG9_Scaffold_1480	6	p3	(GCA)5	15	7792	7806	potentially variable
PHG9_Scaffold_1480	17	p2	(AG)6	12	30183	30194	
PHG9_Scaffold_1480	30	p2	(TA)6	12	50545	50556	
PHG9_Scaffold_1480	34	p2	(AT)6	12	69872	69883	
PHG9_Scaffold_1480	35	p3	(TTC)5	15	70781	70795	potentially variable
PHG9_Scaffold_1480	38	p2	(AT)7	14	72535	72548	potentially variable
PHG9_Scaffold_1480	39	p2	(AT)6	12	77288	77299	
PHG9_Scaffold_1480	40	p2	(TA)7	14	78057	78070	potentially variable
PHG9_Scaffold_1480	43	p2	(TA)6	12	93027	93038	
PHG9_Scaffold_1480	46	p2	(AT)7	14	106001	106014	potentially variable

PHG9_Scaffold_1481	2	p2	(TG)7	14	6728	6741	potentially variable
PHG9_Scaffold_1481	3	p3	(ATT)5	15	11872	11886	potentially variable
PHG9_Scaffold_1481	4	p2	(TC)7	14	23439	23452	potentially variable
PHG9_Scaffold_1481	5	p2	(TA)6	12	25494	25505	
PHG9_Scaffold_1483	4	p3	(TTG)5	15	10517	10531	potentially variable
PHG9_Scaffold_1483	8	p2	(AT)8	16	25852	25867	potentially variable
PHG9_Scaffold_1483	11	p3	(TTA)5	15	31718	31732	potentially variable
PHG9_Scaffold_1483	12	p2	(AT)7	14	44659	44672	potentially variable
PHG9_Scaffold_1483	17	p2	(TA)7	14	51441	51454	potentially variable
PHG9_Scaffold_1483	19	p3	(ATA)6	18	57293	57310	potentially variable
PHG9_Scaffold_1483	20	p6	(CTTTTT)5	30	58210	58239	Hypervariable
PHG9_Scaffold_1486	3	p2	(AT)6	12	4465	4476	
PHG9_Scaffold_1486	12	p3	(ATA)5	15	26001	26015	potentially variable
PHG9_Scaffold_1486	15	p2	(AT)8	16	30271	30286	potentially variable
PHG9_Scaffold_1486	22	p3	(TAA)5	15	37128	37142	potentially variable
PHG9_Scaffold_1487	5	p2	(AT)6	12	15769	15780	
PHG9_Scaffold_1490	1	p2	(AT)9	18	6624	6641	potentially variable

PHG9_Scaffold_1490	9	p2	(AT)7	14	35177	35190	potentially variable
PHG9_Scaffold_1490	12	p2	(TA)8	16	39641	39656	potentially variable
PHG9_Scaffold_1493	2	p3	(AAT)5	15	5524	5538	potentially variable
PHG9_Scaffold_1493	3	p2	(TG)6	12	5849	5860	
PHG9_Scaffold_1494	2	p2	(TC)9	18	11803	11820	potentially variable
PHG9_Scaffold_1494	3	p2	(AG)8	16	19681	19696	potentially variable
PHG9_Scaffold_1496	3	p2	(AT)6	12	2054	2065	
PHG9_Scaffold_1496	23	p3	(ATT)5	15	50180	50194	potentially variable
PHG9_Scaffold_1497	5	p3	(AGA)5	15	11245	11259	potentially variable
PHG9_Scaffold_1497	17	p2	(TG)6	12	43919	43930	
PHG9_Scaffold_1497	20	p3	(TTA)6	18	53086	53103	potentially variable
PHG9_Scaffold_1497	25	p2	(TA)9	18	63303	63320	potentially variable
PHG9_Scaffold_1497	32	p2	(TG)6	12	73109	73120	
PHG9_Scaffold_1497	34	p2	(AT)9	18	81373	81390	potentially variable
PHG9_Scaffold_1497	36	p2	(TA)6	12	86242	86253	
PHG9_Scaffold_1497	42	p2	(CT)6	12	100729	100740	
PHG9_Scaffold_1497	47	p2	(TA)6	12	108526	108537	
PHG9_Scaffold_1498	1	p2	(TA)8	16	598	613	potentially variable

PHG9_Scaffold_1498	6	p3	(ATA)6	18	17976	17993	potentially variable
PHG9_Scaffold_1498	9	p3	(TGG)5	15	24334	24348	potentially variable
PHG9_Scaffold_1499	1	p2	(TA)8	16	1462	1477	potentially variable
PHG9_Scaffold_1499	2	p2	(AG)6	12	2233	2244	
PHG9_Scaffold_1501	3	p2	(TG)6	12	6040	6051	
PHG9_Scaffold_1501	10	p3	(AAT)5	15	24493	24507	potentially variable
PHG9_Scaffold_1504	4	p2	(TA)6	12	11591	11602	
PHG9_Scaffold_1504	7	p2	(AG)6	12	16792	16803	
PHG9_Scaffold_1505	1	p2	(TA)6	12	1365	1376	
PHG9_Scaffold_1505	4	p2	(TA)6	12	7626	7637	
PHG9_Scaffold_1506	3	p3	(TTC)5	15	16016	16030	potentially variable
PHG9_Scaffold_1506	5	p3	(AGG)5	15	35585	35599	potentially variable
PHG9_Scaffold_1506	13	p2	(AT)6	12	61223	61234	
PHG9_Scaffold_1506	16	p2	(TA)6	12	73535	73546	
PHG9_Scaffold_1506	17	p3	(TAA)6	18	86593	86610	potentially variable
PHG9_Scaffold_1508	1	p2	(CA)6	12	804	815	
PHG9_Scaffold_1508	8	p3	(AAT)5	15	16682	16696	potentially variable
PHG9_Scaffold_1508	14	p3	(TTA)5	15	36726	36740	potentially variable
PHG9_Scaffold_1508	19	p3	(TAT)5	15	54587	54601	potentially variable

PHG9_Scaffold_1508	35	p2	(AT)9	18	101234	101251	potentially variable
PHG9_Scaffold_1509	1	p3	(AGG)5	15	2177	2191	potentially variable
PHG9_Scaffold_1509	3	p2	(AT)6	12	10802	10813	
PHG9_Scaffold_1509	10	p2	(AT)9	18	29525	29542	potentially variable
PHG9_Scaffold_1509	15	p2	(TA)7	14	66572	66585	potentially variable
PHG9_Scaffold_1509	17	p2	(TA)9	18	77357	77374	potentially variable
PHG9_Scaffold_1510	5	p2	(TC)8	16	8483	8498	potentially variable
PHG9_Scaffold_1510	6	p2	(TA)6	12	8689	8700	
PHG9_Scaffold_1510	18	p2	(AT)6	12	21902	21913	
PHG9_Scaffold_1513	1	p3	(TAA)5	15	903	917	potentially variable
PHG9_Scaffold_1513	4	p2	(AT)6	12	5859	5870	
PHG9_Scaffold_1515	1	p2	(TG)7	14	477	490	potentially variable
PHG9_Scaffold_1515	4	p2	(AG)7	14	4729	4742	potentially variable
PHG9_Scaffold_1515	7	p2	(AT)7	14	8541	8554	potentially variable
PHG9_Scaffold_1515	9	p3	(CCA)6	18	13875	13892	potentially variable
PHG9_Scaffold_1515	13	p3	(GTT)5	15	30003	30017	potentially variable
PHG9_Scaffold_1515	14	p3	(GTT)5	15	30534	30548	potentially variable

PHG9_Scaffold_1515	16	p2	(TA)7	14	34655	34668	potentially variable
PHG9_Scaffold_1516	8	p3	(TAT)5	15	11288	11302	potentially variable
PHG9_Scaffold_1517	10	p2	(TA)6	12	48773	48784	
PHG9_Scaffold_1518	2	p2	(AT)7	14	1759	1772	potentially variable
PHG9_Scaffold_1519	1	p3	(GCA)5	15	7655	7669	potentially variable
PHG9_Scaffold_1519	2	p2	(AT)7	14	8535	8548	potentially variable
PHG9_Scaffold_1519	3	p2	(TA)8	16	10825	10840	potentially variable
PHG9_Scaffold_1522	1	p2	(AT)7	14	4901	4914	potentially variable
PHG9_Scaffold_1522	6	p2	(AT)8	16	32308	32323	potentially variable
PHG9_Scaffold_1531	9	p2	(AT)9	18	16950	16967	potentially variable
PHG9_Scaffold_1533	3	p3	(CAA)5	15	8975	8989	potentially variable
PHG9_Scaffold_1533	4	p2	(CT)8	16	9902	9917	potentially variable
PHG9_Scaffold_1534	2	p2	(TA)8	16	1069	1084	potentially variable
PHG9_Scaffold_1534	3	p2	(TA)9	18	4357	4374	potentially variable
PHG9_Scaffold_1535	1	p3	(GTG)5	15	6546	6560	potentially variable
PHG9_Scaffold_1537	2	p3	(GTT)5	15	2630	2644	potentially

							variable
PHG9_Scaffold_1537	3	p3	(TGC)6	18	2774	2791	potentially variable
PHG9_Scaffold_1537	6	p2	(AT)8	16	15550	15565	potentially variable
PHG9_Scaffold_1537	8	p2	(TA)7	14	19387	19400	potentially variable
PHG9_Scaffold_1542	1	p2	(TA)7	14	2155	2168	potentially variable
PHG9_Scaffold_1543	2	p2	(TA)8	16	4147	4162	potentially variable
PHG9_Scaffold_1544	2	p2	(AT)6	12	5547	5558	
PHG9_Scaffold_1544	7	p2	(AC)8	16	28454	28469	potentially variable
PHG9_Scaffold_1546	10	p2	(TC)6	12	29336	29347	
PHG9_Scaffold_1546	19	p2	(TG)7	14	53539	53552	potentially variable
PHG9_Scaffold_1546	20	p2	(TC)8	16	60475	60490	potentially variable
PHG9_Scaffold_1546	21	p2	(AT)6	12	66296	66307	
PHG9_Scaffold_1546	33	p2	(TC)8	16	93637	93652	potentially variable
PHG9_Scaffold_1546	37	p2	(TG)6	12	107705	107716	
PHG9_Scaffold_1546	40	p2	(AG)6	12	113405	113416	
PHG9_Scaffold_1546	42	p2	(TG)7	14	126015	126028	potentially variable
PHG9_Scaffold_1546	43	p2	(TC)7	14	126178	126191	potentially variable
PHG9_Scaffold_1546	44	p2	(AT)6	12	126869	126880	

PHG9_Scaffold_1546	46	p3	(AAG)5	15	131239	131253	potentially variable
PHG9_Scaffold_1547	2	p2	(TA)8	16	5157	5172	potentially variable
PHG9_Scaffold_1547	11	p2	(TC)6	12	28649	28660	
PHG9_Scaffold_1547	17	p2	(CT)6	12	52753	52764	
PHG9_Scaffold_1548	1	p2	(TA)6	12	3710	3721	
PHG9_Scaffold_1548	11	p3	(TGC)5	15	41509	41523	potentially variable
PHG9_Scaffold_1548	16	p2	(GA)9	18	58739	58756	potentially variable
PHG9_Scaffold_1548	19	p2	(TA)7	14	69189	69202	potentially variable
PHG9_Scaffold_1548	29	p2	(AT)7	14	109993	110006	potentially variable
PHG9_Scaffold_1548	31	p3	(GTT)5	15	122083	122097	potentially variable
PHG9_Scaffold_1548	32	p2	(AT)7	14	125525	125538	potentially variable
PHG9_Scaffold_1548	36	p2	(AG)9	18	142559	142576	potentially variable
PHG9_Scaffold_1548	37	p2	(TG)7	14	148661	148674	potentially variable
PHG9_Scaffold_1549	2	p2	(TA)8	16	5948	5963	potentially variable
PHG9_Scaffold_1549	5	p3	(GAT)5	15	9274	9288	potentially variable
PHG9_Scaffold_1550	12	p2	(TA)8	16	28765	28780	potentially variable
PHG9_Scaffold_1550	18	p2	(AC)8	16	40375	40390	potentially

							variable
PHG9_Scaffold_1550	25	p2	(AT)8	16	60485	60500	potentially variable
PHG9_Scaffold_1550	36	p2	(TA)7	14	103393	103406	potentially variable
PHG9_Scaffold_1550	39	p2	(TA)7	14	117273	117286	potentially variable
PHG9_Scaffold_1550	41	p2	(TA)9	18	127508	127525	potentially variable
PHG9_Scaffold_1550	43	p2	(TA)8	16	130043	130058	potentially variable
PHG9_Scaffold_1550	53	p3	(ATT)5	15	156001	156015	potentially variable
PHG9_Scaffold_1550	72	p2	(TC)6	12	222205	222216	
PHG9_Scaffold_1550	83	p2	(TA)6	12	253859	253870	
PHG9_Scaffold_1550	96	p2	(CT)6	12	286876	286887	
PHG9_Scaffold_1550	98	p2	(TG)6	12	298087	298098	
PHG9_Scaffold_1550	107	p2	(TA)9	18	313271	313288	potentially variable
PHG9_Scaffold_1552	2	p6	(ATTTGG)5	30	10656	10685	Hypervariable
PHG9_Scaffold_1554	4	p3	(ATA)5	15	22922	22936	potentially variable
PHG9_Scaffold_1555	5	p2	(AT)7	14	13165	13178	potentially variable
PHG9_Scaffold_1555	7	p2	(AT)9	18	20694	20711	potentially variable
PHG9_Scaffold_1555	8	p3	(CAT)5	15	25060	25074	potentially variable
PHG9_Scaffold_1555	13	p2	(TA)7	14	32469	32482	potentially variable

PHG9_Scaffold_1556	11	p3	(TGA)5	15	29936	29950	potentially variable
PHG9_Scaffold_1557	1	p3	(GCT)5	15	14155	14169	potentially variable
PHG9_Scaffold_1557	8	p2	(GA)6	12	50610	50621	
PHG9_Scaffold_1558	5	p2	(AT)6	12	27267	27278	
PHG9_Scaffold_1558	9	p2	(AT)7	14	36439	36452	potentially variable
PHG9_Scaffold_1558	16	p2	(TA)6	12	63143	63154	
PHG9_Scaffold_1559	7	p2	(TA)6	12	22720	22731	
PHG9_Scaffold_1559	8	p2	(TA)8	16	26777	26792	potentially variable
PHG9_Scaffold_1560	1	p3	(CAG)5	15	7612	7626	potentially variable
PHG9_Scaffold_1560	2	p2	(AT)6	12	23297	23308	
PHG9_Scaffold_1560	3	p2	(AT)7	14	23442	23455	potentially variable
PHG9_Scaffold_1560	4	p2	(AT)7	14	23572	23585	potentially variable
PHG9_Scaffold_1560	5	p2	(AT)8	16	23815	23830	potentially variable
PHG9_Scaffold_1560	6	p2	(AG)9	18	27010	27027	potentially variable
PHG9_Scaffold_1560	7	p2	(GA)8	16	29524	29539	potentially variable
PHG9_Scaffold_1560	10	p2	(AG)6	12	45988	45999	
PHG9_Scaffold_1560	16	p2	(TA)6	12	62891	62902	
PHG9_Scaffold_1560	18	p2	(AT)8	16	63441	63456	potentially variable

PHG9_Scaffold_1560	23	p3	(TGG)6	18	80517	80534	potentially variable
PHG9_Scaffold_1560	24	p3	(TCC)5	15	90453	90467	potentially variable
PHG9_Scaffold_1560	27	p2	(TG)8	16	96475	96490	potentially variable
PHG9_Scaffold_1560	30	p2	(TA)6	12	102855	102866	
PHG9_Scaffold_1560	33	p3	(TAT)5	15	109689	109703	potentially variable
PHG9_Scaffold_1560	34	p2	(AT)7	14	116040	116053	potentially variable
PHG9_Scaffold_1560	39	p2	(TA)8	16	120700	120715	potentially variable
PHG9_Scaffold_1560	41	p2	(TC)8	16	134402	134417	potentially variable
PHG9_Scaffold_1560	43	p2	(AG)8	16	149638	149653	potentially variable
PHG9_Scaffold_1560	49	p3	(AAT)5	15	160472	160486	potentially variable
PHG9_Scaffold_1560	51	p2	(TC)6	12	165789	165800	
PHG9_Scaffold_1560	52	p2	(TA)7	14	168473	168486	potentially variable
PHG9_Scaffold_1560	55	p2	(TA)6	12	172480	172491	
PHG9_Scaffold_1563	13	p2	(AG)8	16	34729	34744	potentially variable
PHG9_Scaffold_1563	21	p2	(AG)8	16	68122	68137	potentially variable
PHG9_Scaffold_1564	3	p2	(TA)6	12	4700	4711	
PHG9_Scaffold_1564	4	p3	(GAA)5	15	5356	5370	potentially variable

PHG9_Scaffold_1564	16	p3	(AAT)5	15	40793	40807	potentially variable
PHG9_Scaffold_1564	17	p2	(AT)6	12	41009	41020	
PHG9_Scaffold_1564	21	p2	(TA)8	16	49052	49067	potentially variable
PHG9_Scaffold_1565	9	p3	(AGA)6	18	11289	11306	potentially variable
PHG9_Scaffold_1565	10	p2	(CA)6	12	16151	16162	
PHG9_Scaffold_1565	18	p3	(ATT)5	15	34087	34101	potentially variable
PHG9_Scaffold_1565	28	p3	(AAC)5	15	51396	51410	potentially variable
PHG9_Scaffold_1565	31	p2	(AT)6	12	54186	54197	
PHG9_Scaffold_1565	49	p2	(TA)8	16	108801	108816	potentially variable
PHG9_Scaffold_1565	52	p3	(CCT)5	15	116714	116728	potentially variable
PHG9_Scaffold_1565	53	p2	(AT)8	16	121056	121071	potentially variable
PHG9_Scaffold_1565	55	p2	(TA)9	18	123125	123142	potentially variable
PHG9_Scaffold_1566	9	p2	(AT)8	16	18881	18896	potentially variable
PHG9_Scaffold_1566	11	p2	(TA)6	12	28707	28718	
PHG9_Scaffold_1566	12	p2	(AG)7	14	32865	32878	potentially variable
PHG9_Scaffold_1566	18	p2	(TA)9	18	42820	42837	potentially variable
PHG9_Scaffold_1567	10	p3	(TTA)5	15	25443	25457	potentially variable

PHG9_Scaffold_1567	12	p3	(TCT)6	18	30758	30775	potentially variable
PHG9_Scaffold_1568	5	p2	(AT)6	12	25409	25420	
PHG9_Scaffold_1569	6	p2	(AT)6	12	9427	9438	
PHG9_Scaffold_1569	8	p2	(AT)9	18	13464	13481	potentially variable
PHG9_Scaffold_1573	4	p3	(TAT)5	15	2833	2847	potentially variable
PHG9_Scaffold_1573	18	p3	(AAG)5	15	62023	62037	potentially variable
PHG9_Scaffold_1573	19	p2	(TA)7	14	62185	62198	potentially variable
PHG9_Scaffold_1573	22	p2	(AT)7	14	68233	68246	potentially variable
PHG9_Scaffold_1576	4	p2	(TC)8	16	13547	13562	potentially variable
PHG9_Scaffold_1576	6	p3	(AAT)5	15	18367	18381	potentially variable
PHG9_Scaffold_1577	9	p2	(AC)6	12	33275	33286	
PHG9_Scaffold_1577	10	p3	(TAT)5	15	34906	34920	potentially variable
PHG9_Scaffold_1577	13	p2	(CT)8	16	37474	37489	potentially variable
PHG9_Scaffold_1577	14	p2	(TA)6	12	44433	44444	
PHG9_Scaffold_1580	8	p2	(AG)6	12	26229	26240	
PHG9_Scaffold_1583	1	p2	(AT)6	12	2093	2104	
PHG9_Scaffold_1583	3	p3	(GTT)5	15	12765	12779	potentially variable
PHG9_Scaffold_1583	5	p2	(TA)6	12	18431	18442	

PHG9_Scaffold_1584	14	p2	(GA)7	14	19907	19920	potentially variable
PHG9_Scaffold_1584	25	p3	(AAG)5	15	58203	58217	potentially variable
PHG9_Scaffold_1584	41	p2	(GT)6	12	110378	110389	
PHG9_Scaffold_1584	46	p3	(ATT)5	15	132649	132663	potentially variable
PHG9_Scaffold_1585	3	p2	(CA)8	16	4526	4541	potentially variable
PHG9_Scaffold_1585	4	p3	(TCA)6	18	8617	8634	potentially variable
PHG9_Scaffold_1585	7	p2	(AT)7	14	12923	12936	potentially variable
PHG9_Scaffold_1585	8	p3	(ATT)6	18	19041	19058	potentially variable
PHG9_Scaffold_1585	11	p2	(AT)7	14	21948	21961	potentially variable
PHG9_Scaffold_1585	13	p2	(TC)6	12	28801	28812	
PHG9_Scaffold_1585	16	p2	(AT)6	12	35928	35939	
PHG9_Scaffold_1587	7	p2	(TA)8	16	25404	25419	potentially variable
PHG9_Scaffold_1587	11	p2	(TA)6	12	31119	31130	
PHG9_Scaffold_1587	19	p3	(TAT)5	15	54986	55000	potentially variable
PHG9_Scaffold_1587	27	p3	(ATT)5	15	83484	83498	potentially variable
PHG9_Scaffold_1587	33	p2	(AT)6	12	87666	87677	
PHG9_Scaffold_1587	37	p2	(AG)6	12	93719	93730	
PHG9_Scaffold_1587	43	p2	(AT)6	12	110391	110402	

PHG9_Scaffold_1587	44	p3	(AGA)5	15	111476	111490	potentially variable
PHG9_Scaffold_1587	46	p3	(ATG)5	15	118400	118414	potentially variable
PHG9_Scaffold_1588	10	p3	(GAA)5	15	40317	40331	potentially variable
PHG9_Scaffold_1589	4	p3	(AAT)5	15	9243	9257	potentially variable
PHG9_Scaffold_1589	10	p2	(GT)7	14	25527	25540	potentially variable
PHG9_Scaffold_1589	11	p2	(TA)7	14	30176	30189	potentially variable
PHG9_Scaffold_1589	16	p2	(AG)7	14	45445	45458	potentially variable
PHG9_Scaffold_1589	18	p3	(ATT)5	15	50969	50983	potentially variable
PHG9_Scaffold_1590	11	p2	(TC)7	14	31964	31977	potentially variable
PHG9_Scaffold_1591	3	p2	(TA)6	12	6213	6224	
PHG9_Scaffold_1591	8	p2	(AT)6	12	25763	25774	
PHG9_Scaffold_1591	16	p2	(TA)6	12	40545	40556	
PHG9_Scaffold_1591	30	p2	(CT)7	14	94904	94917	potentially variable
PHG9_Scaffold_1591	36	p6	(AAACGC)6	36	110994	111029	Hypervariable
PHG9_Scaffold_1592	7	p2	(AT)7	14	13522	13535	potentially variable
PHG9_Scaffold_1592	11	p3	(TTA)6	18	25975	25992	potentially variable
PHG9_Scaffold_1595	7	p2	(TA)9	18	17865	17882	potentially variable

PHG9_Scaffold_1595	14	p2	(AT)6	12	34294	34305	
PHG9_Scaffold_1595	16	p2	(TA)6	12	35575	35586	
PHG9_Scaffold_1596	19	p2	(AT)6	12	68767	68778	
PHG9_Scaffold_1597	1	p2	(AG)6	12	221	232	
PHG9_Scaffold_1598	3	p2	(CT)9	18	3232	3249	potentially variable
PHG9_Scaffold_1599	6	p2	(TA)6	12	8983	8994	
PHG9_Scaffold_1600	6	p3	(GAA)5	15	5322	5336	potentially variable
PHG9_Scaffold_1600	12	p2	(AT)7	14	18988	19001	potentially variable
PHG9_Scaffold_1600	14	p2	(AT)6	12	24429	24440	
PHG9_Scaffold_1600	17	p3	(ATT)5	15	27023	27037	potentially variable
PHG9_Scaffold_1600	19	p3	(ATT)6	18	31121	31138	potentially variable
PHG9_Scaffold_1600	20	p3	(CTC)5	15	33252	33266	potentially variable
PHG9_Scaffold_1600	21	p2	(CT)6	12	35204	35215	
PHG9_Scaffold_1602	5	p3	(TAT)5	15	20181	20195	potentially variable
PHG9_Scaffold_1603	1	p3	(ATT)5	15	1331	1345	potentially variable
PHG9_Scaffold_1603	4	p2	(AT)6	12	7532	7543	
PHG9_Scaffold_1603	14	p3	(TTA)5	15	38102	38116	potentially variable
PHG9_Scaffold_1604	3	p3	(AAC)6	18	10066	10083	potentially variable
PHG9_Scaffold_1606	3	p2	(TA)8	16	7849	7864	potentially

							variable
PHG9_Scaffold_1606	8	p2	(GA)7	14	25059	25072	potentially variable
PHG9_Scaffold_1606	11	p2	(TA)6	12	45292	45303	
PHG9_Scaffold_1606	14	p2	(AT)8	16	57464	57479	potentially variable
PHG9_Scaffold_1607	10	p2	(TC)7	14	23273	23286	potentially variable
PHG9_Scaffold_1609	1	p2	(AT)8	16	1554	1569	potentially variable
PHG9_Scaffold_1609	4	p2	(AT)6	12	10698	10709	
PHG9_Scaffold_1609	5	p2	(AG)6	12	14612	14623	
PHG9_Scaffold_1611	3	p3	(GAA)5	15	22910	22924	potentially variable
PHG9_Scaffold_1611	4	p2	(TA)8	16	30149	30164	potentially variable
PHG9_Scaffold_1611	9	p3	(CTT)5	15	42889	42903	potentially variable
PHG9_Scaffold_1612	2	p2	(AT)6	12	12246	12257	
PHG9_Scaffold_1612	9	p2	(TA)6	12	16173	16184	
PHG9_Scaffold_1613	2	p3	(ATT)5	15	3892	3906	potentially variable
PHG9_Scaffold_1613	3	p2	(TA)6	12	4241	4252	
PHG9_Scaffold_1613	20	p2	(TA)7	14	41667	41680	potentially variable
PHG9_Scaffold_1616	7	p3	(CAT)6	18	22639	22656	potentially variable
PHG9_Scaffold_1616	8	p2	(TA)6	12	25834	25845	
PHG9_Scaffold_1616	11	p2	(TC)8	16	39805	39820	potentially

							variable
PHG9_Scaffold_1616	16	p2	(AT)6	12	45262	45273	
PHG9_Scaffold_1616	25	p2	(TC)6	12	68008	68019	
PHG9_Scaffold_1618	2	p2	(TA)6	12	10914	10925	
PHG9_Scaffold_1618	11	p2	(TA)7	14	28484	28497	potentially variable
PHG9_Scaffold_1618	13	p3	(TCT)5	15	37733	37747	potentially variable
PHG9_Scaffold_1618	14	p2	(AC)8	16	39478	39493	potentially variable
PHG9_Scaffold_1618	16	p2	(AT)7	14	50381	50394	potentially variable
PHG9_Scaffold_1618	17	p3	(TAA)5	15	51095	51109	potentially variable
PHG9_Scaffold_1618	31	p2	(AT)9	18	102185	102202	potentially variable
PHG9_Scaffold_1618	34	p2	(TA)7	14	122123	122136	potentially variable
PHG9_Scaffold_1618	40	p2	(TA)9	18	141385	141402	potentially variable
PHG9_Scaffold_1618	45	p2	(TA)6	12	152039	152050	
PHG9_Scaffold_1622	5	p3	(ATT)6	18	26788	26805	potentially variable
PHG9_Scaffold_1622	12	p2	(AT)6	12	52595	52606	
PHG9_Scaffold_1622	15	p2	(AT)6	12	60062	60073	
PHG9_Scaffold_1622	20	p2	(TA)6	12	73744	73755	
PHG9_Scaffold_1622	21	p2	(TA)8	16	77081	77096	potentially variable
PHG9_Scaffold_1622	22	p2	(GA)6	12	77217	77228	

PHG9_Scaffold_1623	6	p3	(TAA)5	15	11044	11058	potentially variable
PHG9_Scaffold_1624	2	p2	(AT)6	12	7629	7640	
PHG9_Scaffold_1624	5	p2	(TA)7	14	8987	9000	potentially variable
PHG9_Scaffold_1624	10	p2	(CT)6	12	20101	20112	
PHG9_Scaffold_1624	13	p2	(TC)9	18	24863	24880	potentially variable
PHG9_Scaffold_1624	15	p2	(TA)6	12	35314	35325	
PHG9_Scaffold_1624	20	p2	(TA)7	14	53470	53483	potentially variable
PHG9_Scaffold_1624	27	p2	(TA)7	14	62590	62603	potentially variable
PHG9_Scaffold_1626	4	p2	(TA)6	12	11287	11298	
PHG9_Scaffold_1626	6	p2	(AT)7	14	12266	12279	potentially variable
PHG9_Scaffold_1626	16	p3	(CCG)5	15	28395	28409	potentially variable
PHG9_Scaffold_1627	1	p2	(AT)6	12	698	709	
PHG9_Scaffold_1627	6	p2	(AT)9	18	21305	21322	potentially variable
PHG9_Scaffold_1627	11	p3	(ATG)5	15	27261	27275	potentially variable
PHG9_Scaffold_1628	4	p3	(TGA)5	15	15304	15318	potentially variable
PHG9_Scaffold_1628	5	p3	(ATT)5	15	15651	15665	potentially variable
PHG9_Scaffold_1628	11	p3	(AAC)6	18	35886	35903	potentially variable
PHG9_Scaffold_1629	3	p2	(AT)8	16	7755	7770	potentially

							variable
PHG9_Scaffold_1629	7	p2	(AT)7	14	22008	22021	potentially variable
PHG9_Scaffold_1629	9	p2	(AT)9	18	23044	23061	potentially variable
PHG9_Scaffold_1629	19	p2	(TA)8	16	64670	64685	potentially variable
PHG9_Scaffold_1629	27	p3	(TCT)5	15	88189	88203	potentially variable
PHG9_Scaffold_1631	8	p2	(TC)6	12	17331	17342	
PHG9_Scaffold_1631	9	p3	(AAT)5	15	22419	22433	potentially variable
PHG9_Scaffold_1632	7	p2	(CT)8	16	10680	10695	potentially variable
PHG9_Scaffold_1633	3	p3	(TGT)5	15	18085	18099	potentially variable
PHG9_Scaffold_1633	10	p2	(GA)7	14	35909	35922	potentially variable
PHG9_Scaffold_1633	11	p2	(AT)9	18	36869	36886	potentially variable
PHG9_Scaffold_1633	12	p2	(AT)6	12	36995	37006	
PHG9_Scaffold_1633	20	p2	(TA)7	14	64736	64749	potentially variable
PHG9_Scaffold_1633	25	p3	(TCA)5	15	72902	72916	potentially variable
PHG9_Scaffold_1633	27	p2	(GA)6	12	75246	75257	
PHG9_Scaffold_1634	5	p3	(TAT)5	15	14869	14883	potentially variable
PHG9_Scaffold_1634	8	p2	(CT)6	12	25648	25659	
PHG9_Scaffold_1635	2	p2	(TA)6	12	4123	4134	

PHG9_Scaffold_1635	3	p2	(AT)8	16	5863	5878	potentially variable
PHG9_Scaffold_1635	13	p2	(TA)6	12	13390	13401	
PHG9_Scaffold_1635	21	p2	(TA)7	14	31182	31195	potentially variable
PHG9_Scaffold_1637	15	p2	(AT)6	12	35630	35641	
PHG9_Scaffold_1637	16	p2	(AT)7	14	35770	35783	potentially variable
PHG9_Scaffold_1637	21	p2	(TA)6	12	72383	72394	
PHG9_Scaffold_1637	23	p2	(AT)7	14	80628	80641	potentially variable
PHG9_Scaffold_1640	5	p2	(AC)7	14	15130	15143	potentially variable
PHG9_Scaffold_1642	7	p3	(AAC)6	18	18267	18284	potentially variable
PHG9_Scaffold_1642	9	p2	(AG)7	14	27936	27949	potentially variable
PHG9_Scaffold_1642	11	p2	(TC)8	16	30845	30860	potentially variable
PHG9_Scaffold_1642	20	p2	(AT)7	14	61580	61593	potentially variable
PHG9_Scaffold_1644	1	p2	(TA)7	14	2449	2462	potentially variable
PHG9_Scaffold_1644	2	p2	(AT)6	12	4301	4312	
PHG9_Scaffold_1646	13	p2	(AT)7	14	101035	101048	potentially variable
PHG9_Scaffold_1646	15	p3	(TGA)5	15	123237	123251	potentially variable
PHG9_Scaffold_1649	3	p2	(TA)7	14	14658	14671	potentially variable

PHG9_Scaffold_1650	9	p3	(TTA)5	15	37680	37694	potentially variable
PHG9_Scaffold_1650	15	p2	(AT)6	12	54996	55007	
PHG9_Scaffold_1650	18	p2	(AT)7	14	62513	62526	potentially variable
PHG9_Scaffold_1650	22	p2	(AT)6	12	100476	100487	
PHG9_Scaffold_1650	33	p2	(TA)6	12	134069	134080	
PHG9_Scaffold_1650	34	p2	(GA)7	14	138063	138076	potentially variable
PHG9_Scaffold_1651	7	p2	(AT)7	14	19051	19064	potentially variable
PHG9_Scaffold_1651	18	p2	(TA)9	18	63029	63046	potentially variable
PHG9_Scaffold_1651	27	p2	(TA)7	14	82661	82674	potentially variable
PHG9_Scaffold_1651	33	p2	(TA)7	14	130849	130862	potentially variable
PHG9_Scaffold_1652	4	p2	(TA)6	12	16071	16082	
PHG9_Scaffold_1652	11	p2	(CA)7	14	55906	55919	potentially variable
PHG9_Scaffold_1652	13	p3	(ATT)5	15	58827	58841	potentially variable
PHG9_Scaffold_1652	16	p2	(AT)6	12	61573	61584	
PHG9_Scaffold_1652	39	p3	(ATA)5	15	118559	118573	potentially variable
PHG9_Scaffold_1652	47	p3	(TAA)5	15	137660	137674	potentially variable
PHG9_Scaffold_1652	71	p2	(TA)6	12	219414	219425	
PHG9_Scaffold_1652	79	p2	(AT)6	12	248984	248995	

PHG9_Scaffold_1652	86	p3	(GAA)5	15	293882	293896	potentially variable
PHG9_Scaffold_1652	88	p3	(GAA)6	18	294218	294235	potentially variable
PHG9_Scaffold_1654	3	p3	(TTA)5	15	14393	14407	potentially variable
PHG9_Scaffold_1654	4	p2	(AC)6	12	16751	16762	
PHG9_Scaffold_1655	1	p2	(TA)7	14	1366	1379	potentially variable
PHG9_Scaffold_1655	7	p2	(AT)8	16	13893	13908	potentially variable
PHG9_Scaffold_1656	2	p2	(TA)8	16	8703	8718	potentially variable
PHG9_Scaffold_1656	30	p2	(CT)8	16	83064	83079	potentially variable
PHG9_Scaffold_1656	32	p3	(ATA)5	15	88439	88453	potentially variable
PHG9_Scaffold_1656	36	p3	(ATT)5	15	94245	94259	potentially variable
PHG9_Scaffold_1656	39	p3	(ATA)5	15	108601	108615	potentially variable
PHG9_Scaffold_1656	40	p2	(AT)7	14	110855	110868	potentially variable
PHG9_Scaffold_1656	41	p2	(TA)6	12	116164	116175	
PHG9_Scaffold_1657	6	p3	(ATA)6	18	22411	22428	potentially variable
PHG9_Scaffold_1657	8	p2	(AT)7	14	25589	25602	potentially variable
PHG9_Scaffold_1657	11	p3	(GGA)5	15	35513	35527	potentially variable

PHG9_Scaffold_1657	14	p3	(ATT)5	15	40500	40514	potentially variable
PHG9_Scaffold_1657	56	p2	(AT)7	14	159884	159897	potentially variable
PHG9_Scaffold_1657	57	p2	(AT)6	12	161594	161605	
PHG9_Scaffold_1657	61	p2	(TG)6	12	173035	173046	
PHG9_Scaffold_1657	63	p3	(CAC)6	18	177255	177272	potentially variable
PHG9_Scaffold_1657	66	p3	(AAG)5	15	184896	184910	potentially variable
PHG9_Scaffold_1657	71	p3	(TGA)6	18	204248	204265	potentially variable
PHG9_Scaffold_1657	74	p2	(GA)6	12	206584	206595	
PHG9_Scaffold_1657	79	p3	(AAC)5	15	234153	234167	potentially variable
PHG9_Scaffold_1657	83	p2	(TA)9	18	238593	238610	potentially variable
PHG9_Scaffold_1658	1	p3	(ATA)6	18	5438	5455	potentially variable
PHG9_Scaffold_1660	3	p3	(GTT)5	15	16761	16775	potentially variable
PHG9_Scaffold_1660	8	p2	(AT)7	14	38039	38052	potentially variable
PHG9_Scaffold_1660	12	p3	(TTA)5	15	50549	50563	potentially variable
PHG9_Scaffold_1662	3	p3	(CAA)5	15	7423	7437	potentially variable
PHG9_Scaffold_1662	9	p2	(TA)6	12	28861	28872	
PHG9_Scaffold_1662	10	p2	(AT)8	16	29589	29604	potentially variable

PHG9_Scaffold_1662	11	p2	(TA)7	14	31349	31362	potentially variable
PHG9_Scaffold_1664	5	p2	(AT)8	16	14901	14916	potentially variable
PHG9_Scaffold_1664	7	p2	(TA)6	12	22910	22921	
PHG9_Scaffold_1664	8	p2	(TA)6	12	23945	23956	
PHG9_Scaffold_1664	9	p2	(CT)6	12	26898	26909	
PHG9_Scaffold_1666	9	p3	(TTC)6	18	18386	18403	potentially variable
PHG9_Scaffold_1666	16	p2	(TG)6	12	34244	34255	
PHG9_Scaffold_1666	17	p3	(GAG)5	15	35272	35286	potentially variable
PHG9_Scaffold_1666	23	p3	(ATT)6	18	58751	58768	potentially variable
PHG9_Scaffold_1666	25	p2	(CA)6	12	61821	61832	
PHG9_Scaffold_1666	27	p2	(CA)8	16	68991	69006	potentially variable
PHG9_Scaffold_1666	28	p3	(TTA)5	15	72080	72094	potentially variable
PHG9_Scaffold_1667	7	p2	(AT)7	14	23561	23574	potentially variable
PHG9_Scaffold_1667	20	p2	(TA)7	14	50660	50673	potentially variable
PHG9_Scaffold_1667	21	p2	(TG)9	18	53816	53833	potentially variable
PHG9_Scaffold_1667	27	p2	(TG)7	14	73610	73623	potentially variable
PHG9_Scaffold_1667	33	p2	(TA)6	12	92878	92889	
PHG9_Scaffold_1667	36	p3	(TAA)6	18	94548	94565	potentially variable

PHG9_Scaffold_1667	37	p2	(GT)6	12	95235	95246	
PHG9_Scaffold_1667	41	p2	(AC)6	12	111640	111651	
PHG9_Scaffold_1667	42	p2	(TG)9	18	116520	116537	potentially variable
PHG9_Scaffold_1667	50	p2	(AT)6	12	126497	126508	
PHG9_Scaffold_1668	3	p2	(AT)8	16	9202	9217	potentially variable
PHG9_Scaffold_1668	5	p2	(TA)6	12	15691	15702	
PHG9_Scaffold_1669	5	p2	(AT)7	14	18877	18890	potentially variable
PHG9_Scaffold_1669	6	p2	(AT)9	18	21632	21649	potentially variable
PHG9_Scaffold_1669	10	p3	(GAA)5	15	27964	27978	potentially variable
PHG9_Scaffold_1669	12	p2	(TA)6	12	30901	30912	
PHG9_Scaffold_1669	15	p2	(AT)6	12	35412	35423	
PHG9_Scaffold_1669	17	p2	(TC)6	12	39810	39821	
PHG9_Scaffold_1669	19	p2	(AT)8	16	45072	45087	potentially variable
PHG9_Scaffold_1669	20	p2	(GT)6	12	50975	50986	
PHG9_Scaffold_1669	21	p2	(TA)6	12	51483	51494	
PHG9_Scaffold_1669	31	p2	(TA)7	14	77951	77964	potentially variable
PHG9_Scaffold_1670	3	p2	(TA)7	14	7110	7123	potentially variable
PHG9_Scaffold_1670	6	p2	(GT)7	14	14943	14956	potentially variable
PHG9_Scaffold_1671	1	p2	(TA)6	12	1242	1253	
PHG9_Scaffold_1671	4	p2	(AT)9	18	17336	17353	potentially

							variable
PHG9_Scaffold_1671	7	p2	(AT)6	12	26158	26169	
PHG9_Scaffold_1671	10	p2	(AT)6	12	31407	31418	
PHG9_Scaffold_1674	3	p2	(AT)6	12	10096	10107	
PHG9_Scaffold_1674	8	p3	(ATT)5	15	19032	19046	potentially variable
PHG9_Scaffold_1674	18	p2	(TA)7	14	37809	37822	potentially variable
PHG9_Scaffold_1674	23	p2	(TA)8	16	47923	47938	potentially variable
PHG9_Scaffold_1674	25	p2	(AT)7	14	58129	58142	potentially variable
PHG9_Scaffold_1674	26	p2	(TA)6	12	58406	58417	
PHG9_Scaffold_1674	29	p2	(AT)6	12	65941	65952	
PHG9_Scaffold_1674	30	p3	(GGT)5	15	68607	68621	potentially variable
PHG9_Scaffold_1674	41	p3	(ATT)5	15	103203	103217	potentially variable
PHG9_Scaffold_1674	50	p3	(CTT)5	15	130753	130767	potentially variable
PHG9_Scaffold_1675	4	p2	(GA)6	12	15770	15781	
PHG9_Scaffold_1677	5	p2	(TA)8	16	4340	4355	potentially variable
PHG9_Scaffold_1677	9	p2	(AT)7	14	22000	22013	potentially variable
PHG9_Scaffold_1677	12	p2	(CT)6	12	24877	24888	
PHG9_Scaffold_1677	14	p2	(AT)6	12	27023	27034	
PHG9_Scaffold_1677	22	p2	(TA)6	12	71372	71383	
PHG9_Scaffold_1679	15	p2	(TA)7	14	16190	16203	potentially

							variable
PHG9_Scaffold_1679	21	p2	(AT)7	14	32478	32491	potentially variable
PHG9_Scaffold_1679	39	p3	(TAT)5	15	109065	109079	potentially variable
PHG9_Scaffold_1679	40	p2	(TA)8	16	109454	109469	potentially variable
PHG9_Scaffold_1679	41	p3	(TTA)5	15	110891	110905	potentially variable
PHG9_Scaffold_1679	43	p2	(TA)6	12	113472	113483	
PHG9_Scaffold_1680	14	p2	(AG)6	12	40606	40617	
PHG9_Scaffold_1682	1	p2	(AG)6	12	478	489	
PHG9_Scaffold_1682	2	p2	(TA)9	18	4284	4301	potentially variable
PHG9_Scaffold_1682	6	p2	(AT)6	12	10636	10647	
PHG9_Scaffold_1682	7	p2	(TA)7	14	11572	11585	potentially variable
PHG9_Scaffold_1683	5	p2	(GA)8	16	18118	18133	potentially variable
PHG9_Scaffold_1683	7	p3	(TTA)5	15	26920	26934	potentially variable
PHG9_Scaffold_1683	8	p2	(AT)6	12	27437	27448	
PHG9_Scaffold_1684	4	p3	(GAT)5	15	15761	15775	potentially variable
PHG9_Scaffold_1684	23	p3	(AAT)6	18	73670	73687	potentially variable
PHG9_Scaffold_1685	1	p2	(CT)7	14	1057	1070	potentially variable
PHG9_Scaffold_1685	3	p2	(GA)6	12	5404	5415	

PHG9_Scaffold_1685	5	p2	(TC)6	12	6232	6243	
PHG9_Scaffold_1685	6	p3	(TCC)5	15	7100	7114	potentially variable
PHG9_Scaffold_1685	9	p2	(GA)8	16	9394	9409	potentially variable
PHG9_Scaffold_1688	2	p2	(AT)9	18	20629	20646	potentially variable
PHG9_Scaffold_1688	4	p2	(TA)6	12	47645	47656	
PHG9_Scaffold_1689	1	p2	(GA)8	16	5018	5033	potentially variable
PHG9_Scaffold_1689	3	p2	(AT)7	14	9890	9903	potentially variable
PHG9_Scaffold_1689	16	p2	(AT)7	14	48305	48318	potentially variable
PHG9_Scaffold_1689	20	p2	(TA)8	16	57688	57703	potentially variable
PHG9_Scaffold_1689	22	p3	(TAT)5	15	60684	60698	potentially variable
PHG9_Scaffold_1689	36	p2	(TA)7	14	95904	95917	potentially variable
PHG9_Scaffold_1689	46	p2	(TG)6	12	124369	124380	
PHG9_Scaffold_1689	52	p2	(AT)6	12	142794	142805	
PHG9_Scaffold_1689	53	p2	(TA)6	12	143496	143507	
PHG9_Scaffold_1689	55	p2	(GA)9	18	155157	155174	potentially variable
PHG9_Scaffold_1689	59	p2	(TC)9	18	175420	175437	potentially variable
PHG9_Scaffold_1689	68	p3	(AAT)5	15	214226	214240	potentially variable
PHG9_Scaffold_1689	76	p2	(TG)6	12	250356	250367	

PHG9_Scaffold_1689	79	p2	(AG)6	12	254128	254139	
PHG9_Scaffold_1689	82	p2	(GT)6	12	257362	257373	
PHG9_Scaffold_1689	87	p2	(AG)6	12	265627	265638	
PHG9_Scaffold_1689	88	p3	(TGA)5	15	265741	265755	potentially variable
PHG9_Scaffold_1689	91	p2	(AG)6	12	271328	271339	
PHG9_Scaffold_1689	94	p2	(CT)7	14	281790	281803	potentially variable
PHG9_Scaffold_1689	95	p3	(TTC)5	15	282175	282189	potentially variable
PHG9_Scaffold_1690	2	p3	(TTA)5	15	8156	8170	potentially variable
PHG9_Scaffold_1690	6	p3	(AGG)5	15	22272	22286	potentially variable
PHG9_Scaffold_1690	11	p3	(TTA)5	15	36649	36663	potentially variable
PHG9_Scaffold_1690	12	p3	(AAC)5	15	37311	37325	potentially variable
PHG9_Scaffold_1692	4	p2	(AT)7	14	9956	9969	potentially variable
PHG9_Scaffold_1692	20	p2	(TA)7	14	86193	86206	potentially variable
PHG9_Scaffold_1692	26	p2	(TA)6	12	106146	106157	
PHG9_Scaffold_1692	31	p2	(TC)8	16	130553	130568	potentially variable
PHG9_Scaffold_1692	46	p2	(TA)7	14	185928	185941	potentially variable
PHG9_Scaffold_1692	47	p2	(TA)6	12	193275	193286	
PHG9_Scaffold_1692	52	p3	(ATT)5	15	200679	200693	potentially variable

PHG9_Scaffold_1692	53	p2	(AT)7	14	200974	200987	potentially variable
PHG9_Scaffold_1692	55	p2	(AT)9	18	203634	203651	potentially variable
PHG9_Scaffold_1695	5	p2	(AT)9	18	36769	36786	potentially variable
PHG9_Scaffold_1695	7	p2	(TA)7	14	43631	43644	potentially variable
PHG9_Scaffold_1695	11	p2	(TA)8	16	54037	54052	potentially variable
PHG9_Scaffold_1695	20	p2	(TA)6	12	77743	77754	
PHG9_Scaffold_1697	6	p3	(CTG)5	15	10665	10679	potentially variable
PHG9_Scaffold_1698	18	p2	(AT)8	16	63411	63426	potentially variable
PHG9_Scaffold_1698	19	p3	(TCG)6	18	67949	67966	potentially variable
PHG9_Scaffold_1699	1	p3	(CTT)5	15	598	612	potentially variable
PHG9_Scaffold_1699	2	p2	(TA)7	14	3523	3536	potentially variable
PHG9_Scaffold_1699	8	p2	(AT)6	12	11726	11737	
PHG9_Scaffold_1699	12	p2	(AT)9	18	17793	17810	potentially variable
PHG9_Scaffold_1701	8	p2	(AT)7	14	5618	5631	potentially variable
PHG9_Scaffold_1701	9	p2	(AT)6	12	6076	6087	
PHG9_Scaffold_1701	12	p2	(AT)7	14	16637	16650	potentially variable
PHG9_Scaffold_1701	17	p2	(TA)6	12	23583	23594	

PHG9_Scaffold_1701	22	p2	(TA)7	14	66266	66279	potentially variable
PHG9_Scaffold_1702	6	p2	(AT)6	12	13124	13135	
PHG9_Scaffold_1702	8	p2	(CT)6	12	18207	18218	
PHG9_Scaffold_1702	12	p2	(AT)8	16	22153	22168	potentially variable
PHG9_Scaffold_1702	13	p2	(TA)8	16	26389	26404	potentially variable
PHG9_Scaffold_1702	16	p2	(TA)8	16	30618	30633	potentially variable
PHG9_Scaffold_1702	24	p2	(TA)9	18	51415	51432	potentially variable
PHG9_Scaffold_1703	5	p2	(TA)8	16	11492	11507	potentially variable
PHG9_Scaffold_1703	10	p2	(AT)6	12	22917	22928	
PHG9_Scaffold_1703	11	p2	(GA)6	12	25113	25124	
PHG9_Scaffold_1703	12	p3	(ATC)5	15	26118	26132	potentially variable
PHG9_Scaffold_1703	24	p2	(GA)6	12	65722	65733	
PHG9_Scaffold_1703	30	p3	(AAT)5	15	80170	80184	potentially variable
PHG9_Scaffold_1708	1	p2	(AT)6	12	523	534	
PHG9_Scaffold_1709	7	p3	(AAG)5	15	31046	31060	potentially variable
PHG9_Scaffold_1709	9	p2	(TA)6	12	36486	36497	
PHG9_Scaffold_1709	11	p3	(AAG)5	15	41111	41125	potentially variable
PHG9_Scaffold_1709	14	p3	(TTA)5	15	46044	46058	potentially variable

PHG9_Scaffold_1712	1	p2	(AT)8	16	2667	2682	potentially variable
PHG9_Scaffold_1712	6	p2	(TA)7	14	22349	22362	potentially variable
PHG9_Scaffold_1714	1	p3	(TAT)5	15	4885	4899	potentially variable
PHG9_Scaffold_1718	1	p2	(TC)7	14	8961	8974	potentially variable
PHG9_Scaffold_1718	2	p2	(TC)7	14	15806	15819	potentially variable
PHG9_Scaffold_1718	5	p2	(TC)6	12	31127	31138	
PHG9_Scaffold_1718	7	p2	(GA)9	18	37280	37297	potentially variable
PHG9_Scaffold_1721	2	p2	(AT)8	16	7187	7202	potentially variable
PHG9_Scaffold_1723	1	p2	(AT)7	14	5395	5408	potentially variable
PHG9_Scaffold_1726	13	p2	(AT)7	14	33615	33628	potentially variable
PHG9_Scaffold_1726	17	p2	(TA)8	16	39160	39175	potentially variable
PHG9_Scaffold_1726	19	p2	(TA)6	12	41764	41775	
PHG9_Scaffold_1727	7	p3	(TGA)5	15	31249	31263	potentially variable
PHG9_Scaffold_1733	1	p3	(AAG)5	15	1259	1273	potentially variable
PHG9_Scaffold_1733	12	p2	(GA)8	16	32961	32976	potentially variable
PHG9_Scaffold_1733	13	p2	(TA)6	12	37292	37303	
PHG9_Scaffold_1733	14	p2	(TA)6	12	37554	37565	

PHG9_Scaffold_1734	2	p2	(AT)7	14	2516	2529	potentially variable
PHG9_Scaffold_1735	2	p2	(TA)7	14	3362	3375	potentially variable
PHG9_Scaffold_1735	9	p2	(CT)6	12	18594	18605	
PHG9_Scaffold_1736	1	p2	(TA)6	12	267	278	
PHG9_Scaffold_1736	6	p2	(GA)7	14	6849	6862	potentially variable
PHG9_Scaffold_1736	7	p2	(AT)9	18	8064	8081	potentially variable
PHG9_Scaffold_1736	8	p3	(CAA)6	18	10216	10233	potentially variable
PHG9_Scaffold_1736	13	p2	(TC)6	12	24435	24446	
PHG9_Scaffold_1736	20	p2	(AT)6	12	49061	49072	
PHG9_Scaffold_1736	26	p3	(AAT)6	18	63458	63475	potentially variable
PHG9_Scaffold_1736	27	p3	(ATA)5	15	63643	63657	potentially variable
PHG9_Scaffold_1736	28	p3	(GCG)5	15	64718	64732	potentially variable
PHG9_Scaffold_1738	7	p2	(TC)9	18	39330	39347	potentially variable
PHG9_Scaffold_1738	31	p2	(AT)7	14	97762	97775	potentially variable
PHG9_Scaffold_1738	42	p3	(GAT)5	15	142258	142272	potentially variable
PHG9_Scaffold_1738	44	p2	(GT)7	14	148547	148560	potentially variable
PHG9_Scaffold_1738	46	p3	(TAA)6	18	151720	151737	potentially variable

PHG9_Scaffold_1738	47	p3	(TTA)6	18	155371	155388	potentially variable
PHG9_Scaffold_1738	56	p3	(ACC)6	18	167495	167512	potentially variable
PHG9_Scaffold_1738	63	p3	(AAC)5	15	195454	195468	potentially variable
PHG9_Scaffold_1738	64	p2	(TA)6	12	196379	196390	
PHG9_Scaffold_1738	65	p2	(AT)7	14	197020	197033	potentially variable
PHG9_Scaffold_1738	66	p2	(AT)6	12	199239	199250	
PHG9_Scaffold_1739	6	p2	(TA)6	12	23777	23788	
PHG9_Scaffold_1739	14	p3	(CAA)5	15	59661	59675	potentially variable
PHG9_Scaffold_1739	15	p3	(GCA)5	15	59804	59818	potentially variable
PHG9_Scaffold_1741	4	p2	(TA)6	12	4779	4790	
PHG9_Scaffold_1741	10	p2	(TA)8	16	30380	30395	potentially variable
PHG9_Scaffold_1741	13	p2	(AT)7	14	45229	45242	potentially variable
PHG9_Scaffold_1741	20	p2	(CT)6	12	70130	70141	
PHG9_Scaffold_1743	4	p2	(AT)6	12	5944	5955	
PHG9_Scaffold_1743	6	p2	(AT)6	12	10197	10208	
PHG9_Scaffold_1743	11	p2	(CA)6	12	26000	26011	
PHG9_Scaffold_1743	13	p3	(GGT)5	15	33138	33152	potentially variable
PHG9_Scaffold_1744	3	p3	(TTC)5	15	3839	3853	potentially variable
PHG9_Scaffold_1744	4	p2	(TA)8	16	5930	5945	potentially

							variable
PHG9_Scaffold_1745	4	p2	(TC)9	18	17859	17876	potentially variable
PHG9_Scaffold_1745	5	p3	(ATA)5	15	21942	21956	potentially variable
PHG9_Scaffold_1745	37	p2	(TA)9	18	111092	111109	potentially variable
PHG9_Scaffold_1746	2	p2	(AT)7	14	14510	14523	potentially variable
PHG9_Scaffold_1746	10	p3	(CAA)5	15	41659	41673	potentially variable
PHG9_Scaffold_1747	4	p2	(GA)6	12	8295	8306	
PHG9_Scaffold_1747	10	p3	(CAA)5	15	16142	16156	potentially variable
PHG9_Scaffold_1748	1	p2	(TA)7	14	2003	2016	potentially variable
PHG9_Scaffold_1748	7	p2	(AT)8	16	21659	21674	potentially variable
PHG9_Scaffold_1748	24	p2	(AT)6	12	59821	59832	
PHG9_Scaffold_1748	25	p3	(GGA)5	15	63783	63797	potentially variable
PHG9_Scaffold_1748	26	p2	(CA)6	12	64108	64119	
PHG9_Scaffold_1748	37	p2	(TA)8	16	129665	129680	potentially variable
PHG9_Scaffold_1748	41	p2	(TA)6	12	131898	131909	
PHG9_Scaffold_1748	45	p2	(TA)8	16	145676	145691	potentially variable
PHG9_Scaffold_1750	6	p2	(AT)6	12	88374	88385	
PHG9_Scaffold_1750	15	p2	(GA)7	14	170157	170170	potentially variable

PHG9_Scaffold_1753	4	p2	(AT)9	18	6781	6798	potentially variable
PHG9_Scaffold_1753	12	p3	(TTC)5	15	59846	59860	potentially variable
PHG9_Scaffold_1753	18	p3	(ATT)5	15	96469	96483	potentially variable
PHG9_Scaffold_1754	4	p3	(TAT)5	15	4691	4705	potentially variable
PHG9_Scaffold_1754	13	p2	(TA)6	12	34725	34736	
PHG9_Scaffold_1754	18	p2	(TA)6	12	60295	60306	
PHG9_Scaffold_1754	20	p2	(AT)7	14	63811	63824	potentially variable
PHG9_Scaffold_1754	21	p2	(TG)6	12	64716	64727	
PHG9_Scaffold_1754	22	p3	(ATT)5	15	66126	66140	potentially variable
PHG9_Scaffold_1755	4	p2	(TA)6	12	17945	17956	
PHG9_Scaffold_1755	11	p2	(AT)7	14	47442	47455	potentially variable
PHG9_Scaffold_1755	13	p3	(TAA)5	15	61443	61457	potentially variable
PHG9_Scaffold_1755	17	p2	(CT)6	12	77169	77180	
PHG9_Scaffold_1755	22	p2	(TA)6	12	85768	85779	
PHG9_Scaffold_1755	23	p3	(TAA)5	15	85885	85899	potentially variable
PHG9_Scaffold_1755	24	p3	(TAA)5	15	86023	86037	potentially variable
PHG9_Scaffold_1756	5	p2	(AT)6	12	75337	75348	
PHG9_Scaffold_1757	8	p3	(ATA)5	15	25125	25139	potentially variable

PHG9_Scaffold_1758	4	p2	(AT)8	16	27747	27762	potentially variable
PHG9_Scaffold_1758	5	p2	(TA)7	14	28205	28218	potentially variable
PHG9_Scaffold_1758	6	p3	(GTG)6	18	35512	35529	potentially variable
PHG9_Scaffold_1759	3	p2	(TA)7	14	5856	5869	potentially variable
PHG9_Scaffold_1759	6	p3	(ATC)5	15	13542	13556	potentially variable
PHG9_Scaffold_1759	8	p2	(AG)7	14	23000	23013	potentially variable
PHG9_Scaffold_1759	20	p2	(AT)7	14	63106	63119	potentially variable
PHG9_Scaffold_1759	22	p2	(CA)8	16	68799	68814	potentially variable
PHG9_Scaffold_1759	25	p2	(TA)7	14	79242	79255	potentially variable
PHG9_Scaffold_1759	29	p2	(AT)6	12	86895	86906	
PHG9_Scaffold_1761	6	p2	(TA)6	12	17018	17029	
PHG9_Scaffold_1761	7	p2	(TG)6	12	26256	26267	
PHG9_Scaffold_1761	8	p2	(TC)6	12	29904	29915	
PHG9_Scaffold_1761	20	p2	(TA)9	18	59965	59982	potentially variable
PHG9_Scaffold_1761	22	p3	(TTG)6	18	60583	60600	potentially variable
PHG9_Scaffold_1761	23	p2	(AT)6	12	64924	64935	
PHG9_Scaffold_1762	4	p2	(AT)7	14	13399	13412	potentially variable
PHG9_Scaffold_1762	6	p3	(TTA)6	18	17231	17248	potentially

							variable
PHG9_Scaffold_1762	9	p2	(CT)7	14	19336	19349	potentially variable
PHG9_Scaffold_1762	11	p2	(CT)7	14	22121	22134	potentially variable
PHG9_Scaffold_1762	15	p3	(ACG)5	15	32855	32869	potentially variable
PHG9_Scaffold_1763	2	p2	(TA)6	12	12423	12434	
PHG9_Scaffold_1763	8	p2	(AT)8	16	18667	18682	potentially variable
PHG9_Scaffold_1763	16	p2	(AT)6	12	53934	53945	
PHG9_Scaffold_1763	19	p2	(CA)6	12	68685	68696	
PHG9_Scaffold_1763	25	p2	(AT)8	16	91174	91189	potentially variable
PHG9_Scaffold_1763	26	p2	(TA)6	12	95906	95917	
PHG9_Scaffold_1763	32	p3	(TAT)5	15	127192	127206	potentially variable
PHG9_Scaffold_1764	1	p2	(AT)6	12	723	734	
PHG9_Scaffold_1764	7	p2	(CT)7	14	45986	45999	potentially variable
PHG9_Scaffold_1764	8	p2	(AT)6	12	55808	55819	
PHG9_Scaffold_1764	22	p2	(TA)8	16	89987	90002	potentially variable
PHG9_Scaffold_1765	6	p2	(AT)9	18	20242	20259	potentially variable
PHG9_Scaffold_1765	10	p2	(AT)6	12	23281	23292	
PHG9_Scaffold_1765	14	p2	(TC)7	14	34662	34675	potentially variable
PHG9_Scaffold_1766	2	p3	(AAT)6	18	29098	29115	potentially

							variable
PHG9_Scaffold_1766	3	p2	(AT)8	16	29728	29743	potentially variable
PHG9_Scaffold_1767	8	p2	(AG)6	12	25377	25388	
PHG9_Scaffold_1767	9	p2	(AT)6	12	29296	29307	
PHG9_Scaffold_1768	1	p2	(AT)8	16	2425	2440	potentially variable
PHG9_Scaffold_1768	4	p3	(AAT)5	15	11268	11282	potentially variable
PHG9_Scaffold_1768	9	p2	(TA)7	14	21082	21095	potentially variable
PHG9_Scaffold_1768	11	p2	(TA)7	14	27016	27029	potentially variable
PHG9_Scaffold_1768	19	p2	(GA)6	12	58673	58684	
PHG9_Scaffold_1768	24	p2	(TA)6	12	75690	75701	
PHG9_Scaffold_1768	25	p3	(AAT)5	15	76469	76483	potentially variable
PHG9_Scaffold_1768	26	p2	(TA)7	14	78812	78825	potentially variable
PHG9_Scaffold_1768	30	p2	(TA)6	12	80783	80794	
PHG9_Scaffold_1768	32	p2	(AT)6	12	81909	81920	
PHG9_Scaffold_1769	4	p2	(AT)6	12	15554	15565	
PHG9_Scaffold_1769	6	p2	(GA)8	16	20713	20728	potentially variable
PHG9_Scaffold_1771	8	p2	(TA)6	12	23209	23220	
PHG9_Scaffold_1771	10	p2	(GA)8	16	27869	27884	potentially variable
PHG9_Scaffold_1771	11	p2	(AC)8	16	37633	37648	potentially variable

PHG9_Scaffold_1771	15	p3	(TTG)5	15	59209	59223	potentially variable
PHG9_Scaffold_1771	17	p3	(TAA)5	15	69341	69355	potentially variable
PHG9_Scaffold_1771	18	p3	(CAC)5	15	74157	74171	potentially variable
PHG9_Scaffold_1771	19	p2	(TA)9	18	74880	74897	potentially variable
PHG9_Scaffold_1771	25	p2	(TA)6	12	97736	97747	
PHG9_Scaffold_1771	26	p2	(CT)6	12	99376	99387	
PHG9_Scaffold_1772	3	p2	(TC)6	12	9716	9727	
PHG9_Scaffold_1772	6	p2	(CG)6	12	21208	21219	
PHG9_Scaffold_1772	11	p2	(CA)6	12	36531	36542	
PHG9_Scaffold_1772	12	p3	(AGA)5	15	42094	42108	potentially variable
PHG9_Scaffold_1772	13	p2	(AG)8	16	43120	43135	potentially variable
PHG9_Scaffold_1772	14	p2	(AT)6	12	51193	51204	
PHG9_Scaffold_1772	17	p2	(TA)6	12	57014	57025	
PHG9_Scaffold_1772	19	p2	(TA)6	12	61742	61753	
PHG9_Scaffold_1773	1	p2	(AT)7	14	1769	1782	potentially variable
PHG9_Scaffold_1773	2	p2	(TA)7	14	7070	7083	potentially variable
PHG9_Scaffold_1773	16	p2	(AT)9	18	40217	40234	potentially variable
PHG9_Scaffold_1773	19	p2	(TA)7	14	50694	50707	potentially variable
PHG9_Scaffold_1775	5	p2	(TA)9	18	16271	16288	potentially

							variable
PHG9_Scaffold_1775	18	p2	(AT)7	14	44570	44583	potentially variable
PHG9_Scaffold_1776	1	p3	(TTG)6	18	4199	4216	potentially variable
PHG9_Scaffold_1776	9	p3	(ACC)5	15	26498	26512	potentially variable
PHG9_Scaffold_1776	25	p2	(AG)8	16	91370	91385	potentially variable
PHG9_Scaffold_1776	26	p2	(TA)7	14	103900	103913	potentially variable
PHG9_Scaffold_1776	27	p2	(TA)7	14	104266	104279	potentially variable
PHG9_Scaffold_1776	30	p3	(TAA)5	15	108709	108723	potentially variable
PHG9_Scaffold_1776	35	p3	(TTC)5	15	117751	117765	potentially variable
PHG9_Scaffold_1776	37	p3	(AAT)5	15	122235	122249	potentially variable
PHG9_Scaffold_1776	44	p2	(TA)6	12	137440	137451	
PHG9_Scaffold_1776	45	p3	(TTA)5	15	138108	138122	potentially variable
PHG9_Scaffold_1776	54	p2	(GT)9	18	159846	159863	potentially variable
PHG9_Scaffold_1777	1	p2	(AG)6	12	5358	5369	
PHG9_Scaffold_1777	5	p3	(ACC)5	15	17145	17159	potentially variable
PHG9_Scaffold_1778	3	p2	(AT)8	16	18482	18497	potentially variable
PHG9_Scaffold_1778	7	p3	(TGA)6	18	32260	32277	potentially

							variable
PHG9_Scaffold_1778	11	p2	(TC)7	14	49287	49300	potentially variable
PHG9_Scaffold_1779	1	p3	(TTC)5	15	19104	19118	potentially variable
PHG9_Scaffold_1781	2	p2	(TA)6	12	7376	7387	
PHG9_Scaffold_1782	6	p2	(AT)8	16	6538	6553	potentially variable
PHG9_Scaffold_1783	1	p2	(TA)6	12	5419	5430	
PHG9_Scaffold_1784	1	p2	(AT)6	12	2408	2419	
PHG9_Scaffold_1784	3	p2	(AT)8	16	12601	12616	potentially variable
PHG9_Scaffold_1784	6	p2	(AT)6	12	18226	18237	
PHG9_Scaffold_1784	7	p2	(TA)6	12	18699	18710	
PHG9_Scaffold_1784	17	p2	(AT)7	14	36420	36433	potentially variable
PHG9_Scaffold_1784	18	p2	(TA)6	12	38695	38706	
PHG9_Scaffold_1784	20	p2	(AT)6	12	45376	45387	
PHG9_Scaffold_1784	21	p3	(GGA)5	15	48578	48592	potentially variable
PHG9_Scaffold_1784	24	p3	(ATA)5	15	50326	50340	potentially variable
PHG9_Scaffold_1784	26	p2	(CT)6	12	59388	59399	
PHG9_Scaffold_1784	38	p3	(ATA)5	15	94935	94949	potentially variable
PHG9_Scaffold_1784	41	p2	(AG)6	12	100068	100079	
PHG9_Scaffold_1785	1	p2	(TA)6	12	1612	1623	
PHG9_Scaffold_1785	3	p2	(TA)9	18	4123	4140	potentially variable

PHG9_Scaffold_1785	7	p2	(TA)7	14	6699	6712	potentially variable
PHG9_Scaffold_1785	9	p2	(TA)7	14	18327	18340	potentially variable
PHG9_Scaffold_1787	10	p2	(AT)7	14	59504	59517	potentially variable
PHG9_Scaffold_1787	12	p2	(TA)7	14	73553	73566	potentially variable
PHG9_Scaffold_1790	6	p3	(GAA)5	15	18572	18586	potentially variable
PHG9_Scaffold_1790	21	p2	(TA)6	12	64106	64117	
PHG9_Scaffold_1790	26	p2	(TA)8	16	79704	79719	potentially variable
PHG9_Scaffold_1790	28	p2	(TA)6	12	81967	81978	
PHG9_Scaffold_1790	33	p3	(AGA)5	15	93295	93309	potentially variable
PHG9_Scaffold_1790	35	p2	(GT)7	14	97294	97307	potentially variable
PHG9_Scaffold_1790	36	p2	(AG)7	14	101306	101319	potentially variable
PHG9_Scaffold_1792	8	p3	(TCA)5	15	31535	31549	potentially variable
PHG9_Scaffold_1792	11	p2	(AT)7	14	47286	47299	potentially variable
PHG9_Scaffold_1792	13	p2	(TA)6	12	54689	54700	
PHG9_Scaffold_1792	17	p3	(AGA)5	15	61896	61910	potentially variable
PHG9_Scaffold_1793	6	p2	(TA)8	16	13396	13411	potentially variable
PHG9_Scaffold_1795	6	p2	(TC)9	18	6883	6900	potentially

							variable
PHG9_Scaffold_1796	11	p2	(AT)8	16	30858	30873	potentially variable
PHG9_Scaffold_1796	17	p2	(AT)6	12	40565	40576	
PHG9_Scaffold_1796	19	p3	(AGT)5	15	42911	42925	potentially variable
PHG9_Scaffold_1796	22	p3	(AAT)5	15	49244	49258	potentially variable
PHG9_Scaffold_1801	5	p2	(AT)7	14	46396	46409	potentially variable
PHG9_Scaffold_1805	2	p2	(AT)7	14	2798	2811	potentially variable
PHG9_Scaffold_1805	4	p2	(AG)7	14	5751	5764	potentially variable
PHG9_Scaffold_1806	3	p2	(TA)6	12	10649	10660	
PHG9_Scaffold_1806	8	p2	(TG)8	16	24476	24491	potentially variable
PHG9_Scaffold_1806	10	p2	(CT)7	14	30467	30480	potentially variable
PHG9_Scaffold_1807	2	p3	(AAT)5	15	1416	1430	potentially variable
PHG9_Scaffold_1807	9	p2	(TG)6	12	13922	13933	
PHG9_Scaffold_1807	15	p2	(CA)7	14	27610	27623	potentially variable
PHG9_Scaffold_1807	17	p2	(TC)6	12	29454	29465	
PHG9_Scaffold_1807	18	p2	(TC)7	14	31316	31329	potentially variable
PHG9_Scaffold_1807	19	p2	(AT)6	12	34583	34594	
PHG9_Scaffold_1809	2	p3	(ATG)5	15	2170	2184	potentially variable

PHG9_Scaffold_1809	6	p3	(ATA)5	15	6131	6145	potentially variable
PHG9_Scaffold_1809	15	p2	(AT)7	14	47319	47332	potentially variable
PHG9_Scaffold_1810	3	p2	(TG)9	18	8207	8224	potentially variable
PHG9_Scaffold_1810	4	p3	(GGA)5	15	13263	13277	potentially variable
PHG9_Scaffold_1810	8	p2	(AT)7	14	41485	41498	potentially variable
PHG9_Scaffold_1810	14	p2	(AT)7	14	62491	62504	potentially variable
PHG9_Scaffold_1810	18	p3	(AAT)5	15	80461	80475	potentially variable
PHG9_Scaffold_1810	24	p2	(AT)7	14	101539	101552	potentially variable
PHG9_Scaffold_1811	7	p2	(TA)8	16	35032	35047	potentially variable
PHG9_Scaffold_1811	8	p2	(AT)7	14	37747	37760	potentially variable
PHG9_Scaffold_1813	6	p2	(AT)7	14	33893	33906	potentially variable
PHG9_Scaffold_1813	11	p3	(ATT)5	15	47212	47226	potentially variable
PHG9_Scaffold_1813	13	p2	(AT)9	18	49842	49859	potentially variable
PHG9_Scaffold_1813	17	p3	(ATG)5	15	86082	86096	potentially variable
PHG9_Scaffold_1815	3	p2	(AG)6	12	20696	20707	
PHG9_Scaffold_1816	1	p2	(AT)8	16	11413	11428	potentially

							variable
PHG9_Scaffold_1817	1	p2	(TA)6	12	2329	2340	
PHG9_Scaffold_1817	4	p3	(ACT)6	18	12723	12740	potentially variable
PHG9_Scaffold_1817	5	p2	(AT)8	16	14905	14920	potentially variable
PHG9_Scaffold_1818	2	p2	(TA)7	14	16258	16271	potentially variable
PHG9_Scaffold_1818	8	p2	(GA)7	14	59932	59945	potentially variable
PHG9_Scaffold_1818	14	p2	(AT)6	12	86532	86543	
PHG9_Scaffold_1819	1	p3	(CAT)5	15	4243	4257	potentially variable
PHG9_Scaffold_1819	9	p2	(TC)6	12	26810	26821	
PHG9_Scaffold_1819	14	p2	(TA)6	12	32537	32548	
PHG9_Scaffold_1819	21	p3	(GTG)5	15	43818	43832	potentially variable
PHG9_Scaffold_1820	5	p3	(TGA)6	18	23647	23664	potentially variable
PHG9_Scaffold_1820	25	p3	(AGT)6	18	87960	87977	potentially variable
PHG9_Scaffold_1820	31	p2	(AG)6	12	100801	100812	
PHG9_Scaffold_1821	2	p2	(AT)6	12	12903	12914	
PHG9_Scaffold_1822	2	p2	(AG)8	16	4631	4646	potentially variable
PHG9_Scaffold_1824	10	p2	(TG)7	14	29398	29411	potentially variable
PHG9_Scaffold_1824	11	p3	(TAA)5	15	30173	30187	potentially variable

PHG9_Scaffold_1824	14	p3	(CAG)5	15	46463	46477	potentially variable
PHG9_Scaffold_1824	18	p2	(AT)7	14	55653	55666	potentially variable
PHG9_Scaffold_1824	20	p3	(TTC)5	15	57105	57119	potentially variable
PHG9_Scaffold_1824	27	p3	(AAT)5	15	68700	68714	potentially variable
PHG9_Scaffold_1824	30	p2	(AT)6	12	71146	71157	
PHG9_Scaffold_1824	38	p3	(ATT)5	15	106682	106696	potentially variable
PHG9_Scaffold_1824	51	p2	(TA)6	12	160438	160449	
PHG9_Scaffold_1824	59	p2	(TA)8	16	173704	173719	potentially variable
PHG9_Scaffold_1824	82	p3	(TTA)5	15	242898	242912	potentially variable
PHG9_Scaffold_1824	89	p2	(TC)7	14	272806	272819	potentially variable
PHG9_Scaffold_1824	91	p2	(TA)8	16	280332	280347	potentially variable
PHG9_Scaffold_1824	92	p3	(GAA)5	15	280742	280756	potentially variable
PHG9_Scaffold_1824	93	p2	(TC)7	14	289445	289458	potentially variable
PHG9_Scaffold_1825	1	p2	(AT)8	16	10564	10579	potentially variable
PHG9_Scaffold_1825	5	p2	(TA)7	14	29038	29051	potentially variable
PHG9_Scaffold_1826	1	p3	(TGT)5	15	8775	8789	potentially variable

PHG9_Scaffold_1826	4	p3	(CAT)6	18	13839	13856	potentially variable
PHG9_Scaffold_1826	11	p2	(AT)9	18	45793	45810	potentially variable
PHG9_Scaffold_1826	12	p2	(TG)6	12	47434	47445	
PHG9_Scaffold_1827	4	p2	(AT)8	16	11856	11871	potentially variable
PHG9_Scaffold_1827	6	p2	(TC)6	12	16396	16407	
PHG9_Scaffold_1830	3	p3	(TTA)5	15	25732	25746	potentially variable
PHG9_Scaffold_1830	7	p2	(TA)6	12	44411	44422	
PHG9_Scaffold_1831	6	p2	(TA)8	16	16652	16667	potentially variable
PHG9_Scaffold_1832	5	p3	(TTG)5	15	17563	17577	potentially variable
PHG9_Scaffold_1833	9	p2	(TA)7	14	26151	26164	potentially variable
PHG9_Scaffold_1833	10	p2	(TA)6	12	29068	29079	
PHG9_Scaffold_1834	6	p2	(TA)6	12	21298	21309	
PHG9_Scaffold_1835	2	p2	(AT)6	12	125	136	
PHG9_Scaffold_1835	14	p2	(AT)8	16	24091	24106	potentially variable
PHG9_Scaffold_1835	24	p3	(ATT)5	15	48113	48127	potentially variable
PHG9_Scaffold_1835	26	p3	(TTA)5	15	54311	54325	potentially variable
PHG9_Scaffold_1835	31	p3	(CAA)6	18	70097	70114	potentially variable
PHG9_Scaffold_1835	34	p3	(GAA)6	18	78383	78400	potentially variable

PHG9_Scaffold_1835	38	p2	(GA)6	12	89495	89506	
PHG9_Scaffold_1836	11	p3	(ATA)5	15	42381	42395	potentially variable
PHG9_Scaffold_1836	15	p3	(TAT)5	15	59009	59023	potentially variable
PHG9_Scaffold_1838	4	p3	(TAT)5	15	12331	12345	potentially variable
PHG9_Scaffold_1838	7	p3	(GAA)5	15	22572	22586	potentially variable
PHG9_Scaffold_1838	15	p2	(AG)7	14	58282	58295	potentially variable
PHG9_Scaffold_1840	2	p2	(TA)7	14	6212	6225	potentially variable
PHG9_Scaffold_1840	4	p3	(AAT)5	15	8567	8581	potentially variable
PHG9_Scaffold_1840	6	p2	(AG)6	12	18546	18557	
PHG9_Scaffold_1841	4	p2	(AT)9	18	18191	18208	potentially variable
PHG9_Scaffold_1842	3	p3	(AAT)5	15	5680	5694	potentially variable
PHG9_Scaffold_1842	8	p2	(AC)9	18	16909	16926	potentially variable
PHG9_Scaffold_1842	9	p2	(GA)7	14	17342	17355	potentially variable
PHG9_Scaffold_1842	10	p2	(AT)6	12	17614	17625	
PHG9_Scaffold_1842	11	p2	(AG)7	14	18144	18157	potentially variable
PHG9_Scaffold_1842	12	p3	(CAT)5	15	18901	18915	potentially variable
PHG9_Scaffold_1842	23	p3	(TCT)5	15	41451	41465	potentially

							variable
PHG9_Scaffold_1842	30	p3	(CGT)5	15	70307	70321	potentially variable
PHG9_Scaffold_1842	33	p3	(TTA)5	15	75482	75496	potentially variable
PHG9_Scaffold_1842	37	p2	(AT)7	14	82201	82214	potentially variable
PHG9_Scaffold_1842	39	p2	(GT)7	14	84381	84394	potentially variable
PHG9_Scaffold_1842	41	p2	(AG)9	18	91552	91569	potentially variable
PHG9_Scaffold_1842	43	p2	(AT)7	14	98047	98060	potentially variable
PHG9_Scaffold_1842	45	p2	(TA)6	12	101180	101191	
PHG9_Scaffold_1842	48	p2	(TG)6	12	105869	105880	
PHG9_Scaffold_1842	61	p2	(TA)6	12	169736	169747	
PHG9_Scaffold_1842	65	p2	(AT)6	12	174562	174573	
PHG9_Scaffold_1842	67	p2	(AT)8	16	179935	179950	potentially variable
PHG9_Scaffold_1842	69	p2	(AT)7	14	180771	180784	potentially variable
PHG9_Scaffold_1843	2	p3	(CAA)5	15	2936	2950	potentially variable
PHG9_Scaffold_1846	6	p3	(TTG)5	15	12276	12290	potentially variable
PHG9_Scaffold_1846	13	p3	(AAT)6	18	39620	39637	potentially variable
PHG9_Scaffold_1847	8	p3	(TTC)5	15	21262	21276	potentially variable
PHG9_Scaffold_1847	9	p2	(TA)9	18	21856	21873	potentially

							variable
PHG9_Scaffold_1848	5	p2	(AT)7	14	18501	18514	potentially variable
PHG9_Scaffold_1848	12	p2	(TA)6	12	40218	40229	
PHG9_Scaffold_1848	13	p2	(AT)8	16	48170	48185	potentially variable
PHG9_Scaffold_1848	18	p2	(AT)6	12	51425	51436	
PHG9_Scaffold_1848	22	p3	(GTT)6	18	119362	119379	potentially variable
PHG9_Scaffold_1848	26	p2	(TA)9	18	122299	122316	potentially variable
PHG9_Scaffold_1848	33	p2	(AT)8	16	198447	198462	potentially variable
PHG9_Scaffold_1848	41	p2	(TA)9	18	216616	216633	potentially variable
PHG9_Scaffold_1850	4	p3	(TTA)5	15	8629	8643	potentially variable
PHG9_Scaffold_1851	4	p2	(TA)8	16	9606	9621	potentially variable
PHG9_Scaffold_1851	8	p2	(AT)6	12	21002	21013	
PHG9_Scaffold_1852	21	p2	(AT)6	12	90457	90468	
PHG9_Scaffold_1852	23	p2	(TA)6	12	91749	91760	
PHG9_Scaffold_1852	28	p2	(AT)8	16	99684	99699	potentially variable
PHG9_Scaffold_1852	31	p2	(AT)6	12	103795	103806	
PHG9_Scaffold_1853	3	p2	(TA)6	12	13536	13547	
PHG9_Scaffold_1853	9	p3	(TAA)5	15	35115	35129	potentially variable
PHG9_Scaffold_1853	11	p3	(GAT)6	18	36236	36253	potentially

							variable
PHG9_Scaffold_1853	12	p2	(AT)8	16	39985	40000	potentially variable
PHG9_Scaffold_1853	13	p2	(CT)7	14	41335	41348	potentially variable
PHG9_Scaffold_1853	16	p2	(AG)7	14	49609	49622	potentially variable
PHG9_Scaffold_1853	19	p2	(AT)6	12	60347	60358	
PHG9_Scaffold_1853	20	p2	(AT)8	16	60710	60725	potentially variable
PHG9_Scaffold_1853	23	p2	(AG)8	16	66490	66505	potentially variable
PHG9_Scaffold_1853	33	p2	(AT)8	16	101869	101884	potentially variable
PHG9_Scaffold_1854	2	p2	(AT)7	14	922	935	potentially variable
PHG9_Scaffold_1855	6	p2	(AG)6	12	7753	7764	
PHG9_Scaffold_1856	1	p3	(ATC)5	15	15	29	potentially variable
PHG9_Scaffold_1857	2	p2	(TA)6	12	19951	19962	
PHG9_Scaffold_1857	9	p2	(AT)6	12	35417	35428	
PHG9_Scaffold_1857	13	p2	(CT)7	14	46391	46404	potentially variable
PHG9_Scaffold_1857	14	p3	(CAG)5	15	47705	47719	potentially variable
PHG9_Scaffold_1857	18	p3	(ATC)5	15	67279	67293	potentially variable
PHG9_Scaffold_1857	19	p3	(TTG)5	15	68200	68214	potentially variable
PHG9_Scaffold_1858	3	p2	(AT)6	12	3570	3581	

PHG9_Scaffold_1860	1	p2	(AT)6	12	10918	10929	
PHG9_Scaffold_1860	3	p2	(GA)9	18	15932	15949	potentially variable
PHG9_Scaffold_1862	1	p2	(TA)6	12	244	255	
PHG9_Scaffold_1862	2	p2	(AT)6	12	416	427	
PHG9_Scaffold_1862	6	p3	(CAT)6	18	11021	11038	potentially variable
PHG9_Scaffold_1863	1	p3	(ATA)5	15	9137	9151	potentially variable
PHG9_Scaffold_1863	14	p2	(AT)7	14	46740	46753	potentially variable
PHG9_Scaffold_1863	16	p2	(AT)6	12	50278	50289	
PHG9_Scaffold_1863	21	p2	(AT)8	16	69151	69166	potentially variable
PHG9_Scaffold_1863	36	p2	(CT)6	12	133913	133924	
PHG9_Scaffold_1863	44	p2	(AT)7	14	149698	149711	potentially variable
PHG9_Scaffold_1863	45	p2	(AT)6	12	154088	154099	
PHG9_Scaffold_1863	53	p2	(AG)8	16	170667	170682	potentially variable
PHG9_Scaffold_1867	2	p2	(TA)6	12	11364	11375	
PHG9_Scaffold_1867	3	p2	(AT)6	12	13676	13687	
PHG9_Scaffold_1867	6	p2	(TA)6	12	22683	22694	
PHG9_Scaffold_1867	14	p2	(TA)6	12	34555	34566	
PHG9_Scaffold_1867	25	p2	(AG)8	16	78591	78606	potentially variable
PHG9_Scaffold_1867	26	p2	(TA)6	12	78927	78938	
PHG9_Scaffold_1867	30	p2	(AT)7	14	86879	86892	potentially variable

PHG9_Scaffold_1867	31	p2	(AT)6	12	87703	87714	
PHG9_Scaffold_1867	33	p3	(AAT)5	15	88834	88848	potentially variable
PHG9_Scaffold_1867	34	p2	(AT)6	12	93557	93568	
PHG9_Scaffold_1867	39	p2	(TA)6	12	103222	103233	
PHG9_Scaffold_1867	40	p3	(GTA)5	15	114397	114411	potentially variable
PHG9_Scaffold_1870	5	p2	(GT)7	14	21706	21719	potentially variable
PHG9_Scaffold_1871	1	p2	(TA)6	12	5226	5237	
PHG9_Scaffold_1871	5	p2	(AT)7	14	13665	13678	potentially variable
PHG9_Scaffold_1875	3	p2	(AC)7	14	4450	4463	potentially variable
PHG9_Scaffold_1875	4	p2	(AT)8	16	10229	10244	potentially variable
PHG9_Scaffold_1875	9	p2	(TC)6	12	17754	17765	
PHG9_Scaffold_1875	11	p3	(GAT)5	15	23976	23990	potentially variable
PHG9_Scaffold_1875	19	p2	(TA)6	12	40211	40222	
PHG9_Scaffold_1875	22	p3	(ATC)5	15	45399	45413	potentially variable
PHG9_Scaffold_1875	23	p3	(GTT)6	18	50054	50071	potentially variable
PHG9_Scaffold_1875	25	p2	(AT)7	14	53593	53606	potentially variable
PHG9_Scaffold_1876	1	p2	(TA)8	16	1114	1129	potentially variable
PHG9_Scaffold_1877	2	p2	(TA)9	18	1317	1334	potentially variable

PHG9_Scaffold_1877	5	p2	(GT)6	12	7384	7395	
PHG9_Scaffold_1878	14	p2	(AT)6	12	45145	45156	
PHG9_Scaffold_1878	19	p2	(AT)6	12	63996	64007	
PHG9_Scaffold_1878	20	p2	(AG)6	12	74710	74721	
PHG9_Scaffold_1878	21	p2	(CT)6	12	86725	86736	
PHG9_Scaffold_1879	2	p2	(TC)6	12	5976	5987	
PHG9_Scaffold_1879	8	p2	(TA)7	14	14063	14076	potentially variable
PHG9_Scaffold_1881	15	p3	(ATA)5	15	38452	38466	potentially variable
PHG9_Scaffold_1881	17	p2	(TA)7	14	44211	44224	potentially variable
PHG9_Scaffold_1881	33	p3	(AAT)6	18	89474	89491	potentially variable
PHG9_Scaffold_1882	3	p3	(TTA)5	15	2835	2849	potentially variable
PHG9_Scaffold_1889	6	p2	(TA)9	18	43084	43101	potentially variable
PHG9_Scaffold_1889	9	p2	(AT)6	12	54292	54303	
PHG9_Scaffold_1889	11	p2	(TA)6	12	67360	67371	
PHG9_Scaffold_1889	19	p2	(AT)6	12	77536	77547	
PHG9_Scaffold_1889	25	p3	(CTT)5	15	87496	87510	potentially variable
PHG9_Scaffold_1889	32	p2	(TA)9	18	120162	120179	potentially variable
PHG9_Scaffold_1889	42	p3	(AGA)5	15	178802	178816	potentially variable
PHG9_Scaffold_1889	43	p2	(TA)8	16	179094	179109	potentially variable

PHG9_Scaffold_1890	4	p3	(AAG)5	15	6755	6769	potentially variable
PHG9_Scaffold_1890	18	p3	(ATA)6	18	44220	44237	potentially variable
PHG9_Scaffold_1891	2	p3	(CCT)5	15	559	573	potentially variable
PHG9_Scaffold_1891	7	p2	(GA)6	12	14726	14737	
PHG9_Scaffold_1891	16	p2	(TA)7	14	24426	24439	potentially variable
PHG9_Scaffold_1891	20	p2	(AG)9	18	27598	27615	potentially variable
PHG9_Scaffold_1891	27	p2	(TA)6	12	58136	58147	
PHG9_Scaffold_1892	1	p2	(TA)6	12	2183	2194	
PHG9_Scaffold_1892	2	p2	(TA)8	16	5413	5428	potentially variable
PHG9_Scaffold_1892	6	p3	(GAT)5	15	13914	13928	potentially variable
PHG9_Scaffold_1894	8	p2	(AT)6	12	43250	43261	
PHG9_Scaffold_1895	3	p2	(GA)6	12	4986	4997	
PHG9_Scaffold_1895	8	p3	(CAA)5	15	25626	25640	potentially variable
PHG9_Scaffold_1895	9	p2	(TA)6	12	25770	25781	
PHG9_Scaffold_1895	12	p3	(AAG)5	15	30862	30876	potentially variable
PHG9_Scaffold_1895	16	p2	(AG)6	12	44928	44939	
PHG9_Scaffold_1895	20	p2	(TA)6	12	63025	63036	
PHG9_Scaffold_1895	31	p3	(ATA)5	15	88699	88713	potentially variable
PHG9_Scaffold_1896	4	p2	(CT)7	14	4478	4491	potentially

							variable
PHG9_Scaffold_1896	5	p2	(GA)7	14	10408	10421	potentially variable
PHG9_Scaffold_1899	1	p3	(GAA)5	15	840	854	potentially variable
PHG9_Scaffold_1901	9	p2	(TC)6	12	15840	15851	
PHG9_Scaffold_1901	11	p3	(AAT)5	15	19082	19096	potentially variable
PHG9_Scaffold_1902	1	p3	(TAT)5	15	3566	3580	potentially variable
PHG9_Scaffold_1902	2	p2	(AT)8	16	4053	4068	potentially variable
PHG9_Scaffold_1902	5	p3	(CAC)5	15	12046	12060	potentially variable
PHG9_Scaffold_1903	2	p2	(AT)6	12	17446	17457	
PHG9_Scaffold_1903	6	p2	(TA)6	12	25488	25499	
PHG9_Scaffold_1903	8	p2	(AT)6	12	29144	29155	
PHG9_Scaffold_1903	9	p2	(TA)6	12	29803	29814	
PHG9_Scaffold_1903	11	p2	(TA)7	14	31193	31206	potentially variable
PHG9_Scaffold_1904	2	p2	(TA)7	14	4848	4861	potentially variable
PHG9_Scaffold_1905	5	p2	(TA)7	14	3922	3935	potentially variable
PHG9_Scaffold_1907	2	p3	(TAA)6	18	19390	19407	potentially variable
PHG9_Scaffold_1907	4	p2	(AT)8	16	25878	25893	potentially variable
PHG9_Scaffold_1909	2	p2	(CT)6	12	21983	21994	

PHG9_Scaffold_1910	2	p2	(TA)6	12	668	679	
PHG9_Scaffold_1910	8	p2	(AT)7	14	6236	6249	potentially variable
PHG9_Scaffold_1910	14	p2	(AT)9	18	25173	25190	potentially variable
PHG9_Scaffold_1911	2	p2	(AT)9	18	11874	11891	potentially variable
PHG9_Scaffold_1915	1	p2	(TA)6	12	1142	1153	
PHG9_Scaffold_1917	2	p2	(AT)7	14	8013	8026	potentially variable
PHG9_Scaffold_1917	10	p2	(AT)6	12	25475	25486	
PHG9_Scaffold_1917	13	p3	(AAT)5	15	28045	28059	potentially variable
PHG9_Scaffold_1918	7	p2	(AT)6	12	28438	28449	
PHG9_Scaffold_1924	4	p3	(TTG)5	15	9890	9904	potentially variable
PHG9_Scaffold_1925	3	p2	(AT)6	12	22640	22651	
PHG9_Scaffold_1925	5	p2	(TC)8	16	24600	24615	potentially variable
PHG9_Scaffold_1925	10	p2	(TA)6	12	29667	29678	
PHG9_Scaffold_1925	12	p2	(AT)6	12	31080	31091	
PHG9_Scaffold_1925	15	p2	(TG)6	12	33419	33430	
PHG9_Scaffold_1929	2	p3	(TCT)6	18	335	352	potentially variable
PHG9_Scaffold_1929	3	p3	(CTT)5	15	4238	4252	potentially variable
PHG9_Scaffold_1930	1	p2	(AT)9	18	1460	1477	potentially variable
PHG9_Scaffold_1930	4	p3	(GAA)6	18	11910	11927	potentially

							variable
PHG9_Scaffold_1931	4	p2	(AG)6	12	7993	8004	
PHG9_Scaffold_1932	1	p2	(TA)7	14	16184	16197	potentially variable
PHG9_Scaffold_1932	3	p2	(GA)6	12	35730	35741	
PHG9_Scaffold_1935	1	p2	(TA)6	12	13600	13611	
PHG9_Scaffold_1940	2	p2	(AG)7	14	524	537	potentially variable
PHG9_Scaffold_1941	1	p2	(AT)6	12	61	72	
PHG9_Scaffold_1941	3	p2	(TA)7	14	7901	7914	potentially variable
PHG9_Scaffold_1941	4	p2	(AG)7	14	9017	9030	potentially variable
PHG9_Scaffold_1941	6	p3	(TCA)5	15	12110	12124	potentially variable
PHG9_Scaffold_1942	6	p2	(TC)6	12	15441	15452	
PHG9_Scaffold_1942	7	p3	(ATA)5	15	15641	15655	potentially variable
PHG9_Scaffold_1942	10	p3	(ATA)5	15	31533	31547	potentially variable
PHG9_Scaffold_1943	8	p2	(AT)6	12	15607	15618	
PHG9_Scaffold_1944	10	p2	(AT)7	14	68390	68403	potentially variable
PHG9_Scaffold_1944	11	p2	(AT)7	14	68552	68565	potentially variable
PHG9_Scaffold_1944	12	p2	(AT)6	12	69058	69069	
PHG9_Scaffold_1947	5	p2	(TA)8	16	14616	14631	potentially variable
PHG9_Scaffold_1947	7	p2	(GA)8	16	18200	18215	potentially

							variable
PHG9_Scaffold_1947	8	p2	(AG)8	16	18536	18551	potentially variable
PHG9_Scaffold_1948	1	p2	(AT)7	14	1904	1917	potentially variable
PHG9_Scaffold_1949	5	p2	(GA)8	16	8411	8426	potentially variable
PHG9_Scaffold_1950	1	p6	(GGTTTT)5	30	1064	1093	Hypervariable
PHG9_Scaffold_1954	4	p2	(AT)6	12	8164	8175	
PHG9_Scaffold_1954	9	p3	(AAG)5	15	14045	14059	potentially variable
PHG9_Scaffold_1954	17	p2	(CA)6	12	39524	39535	
PHG9_Scaffold_1955	2	p2	(TA)6	12	1746	1757	
PHG9_Scaffold_1955	5	p2	(AT)6	12	23827	23838	
PHG9_Scaffold_1955	8	p2	(TA)7	14	46989	47002	potentially variable
PHG9_Scaffold_1955	11	p2	(TA)6	12	54153	54164	
PHG9_Scaffold_1956	13	p2	(TA)6	12	32162	32173	
PHG9_Scaffold_1958	6	p2	(AT)8	16	33192	33207	potentially variable
PHG9_Scaffold_1958	15	p3	(ATT)6	18	91989	92006	potentially variable
PHG9_Scaffold_1958	16	p2	(AT)7	14	96352	96365	potentially variable
PHG9_Scaffold_1958	19	p3	(AAT)5	15	116622	116636	potentially variable
PHG9_Scaffold_1958	20	p2	(TA)7	14	121344	121357	potentially variable
PHG9_Scaffold_1958	21	p2	(CA)7	14	123009	123022	potentially

							variable
PHG9_Scaffold_1959	3	p2	(AT)6	12	2353	2364	
PHG9_Scaffold_1959	4	p2	(AT)7	14	7910	7923	potentially variable
PHG9_Scaffold_1959	7	p2	(TA)7	14	12767	12780	potentially variable
PHG9_Scaffold_1960	9	p2	(TC)6	12	14282	14293	
PHG9_Scaffold_1960	13	p2	(TA)7	14	32928	32941	potentially variable
PHG9_Scaffold_1961	6	p2	(AT)8	16	17008	17023	potentially variable
PHG9_Scaffold_1961	7	p2	(TA)6	12	17561	17572	
PHG9_Scaffold_1961	18	p2	(AT)9	18	37568	37585	potentially variable
PHG9_Scaffold_1962	5	p2	(AT)6	12	8808	8819	
PHG9_Scaffold_1962	6	p2	(TC)7	14	9924	9937	potentially variable
PHG9_Scaffold_1962	7	p2	(CT)6	12	12737	12748	
PHG9_Scaffold_1962	8	p2	(AT)9	18	14058	14075	potentially variable
PHG9_Scaffold_1962	17	p3	(TTC)5	15	49826	49840	potentially variable
PHG9_Scaffold_1962	22	p2	(AT)6	12	70899	70910	
PHG9_Scaffold_1962	24	p2	(TG)7	14	72236	72249	potentially variable
PHG9_Scaffold_1962	25	p2	(AT)7	14	76984	76997	potentially variable
PHG9_Scaffold_1962	34	p2	(AG)9	18	98143	98160	potentially variable

PHG9_Scaffold_1962	38	p3	(GAT)5	15	107039	107053	potentially variable
PHG9_Scaffold_1964	4	p2	(TC)6	12	22535	22546	
PHG9_Scaffold_1965	1	p2	(AG)6	12	2840	2851	
PHG9_Scaffold_1965	2	p2	(TA)7	14	16061	16074	potentially variable
PHG9_Scaffold_1965	5	p2	(TA)6	12	32514	32525	
PHG9_Scaffold_1965	8	p2	(AT)6	12	38420	38431	
PHG9_Scaffold_1965	9	p2	(AT)6	12	38917	38928	
PHG9_Scaffold_1968	2	p2	(AG)8	16	8838	8853	potentially variable
PHG9_Scaffold_1968	5	p3	(AAT)5	15	13139	13153	potentially variable
PHG9_Scaffold_1968	7	p2	(TA)8	16	21913	21928	potentially variable
PHG9_Scaffold_1968	34	p2	(GA)7	14	94820	94833	potentially variable
PHG9_Scaffold_1968	41	p3	(TTA)5	15	112750	112764	potentially variable
PHG9_Scaffold_1970	5	p2	(AT)9	18	14664	14681	potentially variable
PHG9_Scaffold_1970	7	p3	(ATG)5	15	19567	19581	potentially variable
PHG9_Scaffold_1971	5	p2	(TA)8	16	48456	48471	potentially variable
PHG9_Scaffold_1971	6	p2	(TA)6	12	52306	52317	
PHG9_Scaffold_1971	10	p2	(AT)8	16	76120	76135	potentially variable
PHG9_Scaffold_1972	1	p2	(TA)7	14	7	20	potentially variable

PHG9_Scaffold_1972	10	p2	(AT)9	18	17476	17493	potentially variable
PHG9_Scaffold_1972	12	p3	(TAT)5	15	20087	20101	potentially variable
PHG9_Scaffold_1973	1	p2	(AT)6	12	660	671	
PHG9_Scaffold_1973	6	p3	(GAA)5	15	26810	26824	potentially variable
PHG9_Scaffold_1973	9	p2	(TA)6	12	33309	33320	
PHG9_Scaffold_1973	11	p2	(TA)7	14	40767	40780	potentially variable
PHG9_Scaffold_1975	3	p2	(AT)6	12	3171	3182	
PHG9_Scaffold_1975	7	p2	(TA)7	14	14731	14744	potentially variable
PHG9_Scaffold_1975	9	p2	(CT)6	12	15998	16009	
PHG9_Scaffold_1975	23	p3	(ATT)5	15	57543	57557	potentially variable
PHG9_Scaffold_1975	26	p6	(CATGCC)5	30	76021	76050	Hypervariable
PHG9_Scaffold_1976	5	p2	(AT)7	14	8842	8855	potentially variable
PHG9_Scaffold_1976	13	p3	(ATT)6	18	23509	23526	potentially variable
PHG9_Scaffold_1980	4	p3	(GTG)5	15	19272	19286	potentially variable
PHG9_Scaffold_1980	12	p2	(AG)6	12	41705	41716	
PHG9_Scaffold_1980	17	p2	(TA)7	14	55762	55775	potentially variable
PHG9_Scaffold_1980	22	p2	(AG)6	12	65138	65149	
PHG9_Scaffold_1981	3	p2	(GT)8	16	6295	6310	potentially variable

PHG9_Scaffold_1981	6	p2	(AT)7	14	24338	24351	potentially variable
PHG9_Scaffold_1982	3	p2	(AT)6	12	2738	2749	
PHG9_Scaffold_1984	6	p2	(TA)7	14	23791	23804	potentially variable
PHG9_Scaffold_1985	2	p2	(TA)9	18	3137	3154	potentially variable
PHG9_Scaffold_1985	7	p2	(TA)6	12	13000	13011	
PHG9_Scaffold_1985	8	p2	(TA)6	12	13113	13124	
PHG9_Scaffold_1985	16	p2	(TA)6	12	48172	48183	
PHG9_Scaffold_1985	17	p2	(AT)7	14	52291	52304	potentially variable
PHG9_Scaffold_1986	1	p2	(GT)6	12	1098	1109	
PHG9_Scaffold_1988	9	p3	(CAA)5	15	15797	15811	potentially variable
PHG9_Scaffold_1988	10	p2	(AT)6	12	17877	17888	
PHG9_Scaffold_1988	17	p3	(CAT)5	15	38432	38446	potentially variable
PHG9_Scaffold_1988	18	p3	(ATA)5	15	43836	43850	potentially variable
PHG9_Scaffold_1988	19	p2	(AT)6	12	56864	56875	
PHG9_Scaffold_1988	21	p2	(TA)6	12	66063	66074	
PHG9_Scaffold_1988	27	p3	(ATT)6	18	80180	80197	potentially variable
PHG9_Scaffold_1988	29	p2	(TA)9	18	85085	85102	potentially variable
PHG9_Scaffold_1988	34	p2	(TA)9	18	98928	98945	potentially variable
PHG9_Scaffold_1989	2	p2	(AT)7	14	15572	15585	potentially

							variable
PHG9_Scaffold_1989	3	p2	(TA)8	16	16001	16016	potentially variable
PHG9_Scaffold_1989	6	p2	(AT)6	12	27795	27806	
PHG9_Scaffold_1990	2	p3	(GAA)5	15	5247	5261	potentially variable
PHG9_Scaffold_1995	2	p2	(AT)8	16	8909	8924	potentially variable
PHG9_Scaffold_1995	6	p3	(AAC)5	15	18275	18289	potentially variable
PHG9_Scaffold_1997	1	p2	(AG)6	12	1334	1345	
PHG9_Scaffold_1997	5	p2	(AT)8	16	43976	43991	potentially variable
PHG9_Scaffold_1997	14	p2	(TA)9	18	71988	72005	potentially variable
PHG9_Scaffold_1997	19	p2	(CA)6	12	74277	74288	
PHG9_Scaffold_1999	1	p2	(AT)6	12	571	582	
PHG9_Scaffold_1999	7	p3	(TAT)5	15	10694	10708	potentially variable
PHG9_Scaffold_1999	8	p2	(TA)6	12	14223	14234	
PHG9_Scaffold_2001	5	p2	(TA)8	16	40947	40962	potentially variable
PHG9_Scaffold_2001	8	p2	(AT)9	18	51827	51844	potentially variable
PHG9_Scaffold_2001	14	p2	(TA)7	14	71192	71205	potentially variable
PHG9_Scaffold_2001	17	p2	(AT)6	12	101723	101734	
PHG9_Scaffold_2001	28	p2	(TA)6	12	113920	113931	
PHG9_Scaffold_2003	3	p2	(AC)8	16	6352	6367	potentially

							variable
PHG9_Scaffold_2003	8	p2	(TG)6	12	28292	28303	
PHG9_Scaffold_2003	22	p2	(AT)6	12	59454	59465	
PHG9_Scaffold_2003	23	p3	(TCT)5	15	62057	62071	potentially variable
PHG9_Scaffold_2003	28	p3	(TTC)5	15	70578	70592	potentially variable
PHG9_Scaffold_2003	35	p2	(GT)6	12	98416	98427	
PHG9_Scaffold_2003	41	p2	(TA)8	16	106111	106126	potentially variable
PHG9_Scaffold_2003	47	p2	(AG)6	12	123784	123795	
PHG9_Scaffold_2004	6	p2	(TA)6	12	23764	23775	
PHG9_Scaffold_2005	3	p2	(TA)6	12	11711	11722	
PHG9_Scaffold_2005	4	p2	(TA)7	14	14591	14604	potentially variable
PHG9_Scaffold_2005	5	p2	(AT)7	14	15775	15788	potentially variable
PHG9_Scaffold_2005	11	p2	(GT)7	14	28032	28045	potentially variable
PHG9_Scaffold_2005	21	p2	(AT)7	14	83602	83615	potentially variable
PHG9_Scaffold_2005	22	p3	(TGT)5	15	85624	85638	potentially variable
PHG9_Scaffold_2005	29	p2	(CT)7	14	104549	104562	potentially variable
PHG9_Scaffold_2005	32	p2	(AG)6	12	109993	110004	
PHG9_Scaffold_2005	34	p2	(AT)6	12	111957	111968	
PHG9_Scaffold_2005	35	p2	(TA)8	16	115486	115501	potentially variable

PHG9_Scaffold_2005	44	p3	(TTA)5	15	136816	136830	potentially variable
PHG9_Scaffold_2005	45	p2	(GT)6	12	139179	139190	
PHG9_Scaffold_2005	49	p2	(AT)6	12	145586	145597	
PHG9_Scaffold_2005	52	p2	(TA)6	12	149193	149204	
PHG9_Scaffold_2005	53	p2	(TA)8	16	153058	153073	potentially variable
PHG9_Scaffold_2005	55	p3	(TTA)5	15	169011	169025	potentially variable
PHG9_Scaffold_2005	57	p2	(AT)7	14	169575	169588	potentially variable
PHG9_Scaffold_2005	64	p2	(TC)6	12	196398	196409	
PHG9_Scaffold_2005	75	p2	(TG)7	14	243292	243305	potentially variable
PHG9_Scaffold_2007	11	p3	(CTA)5	15	56668	56682	potentially variable
PHG9_Scaffold_2007	13	p2	(TA)8	16	63766	63781	potentially variable
PHG9_Scaffold_2008	2	p2	(TA)6	12	1302	1313	
PHG9_Scaffold_2008	6	p3	(AAG)5	15	19550	19564	potentially variable
PHG9_Scaffold_2008	10	p2	(TA)6	12	26118	26129	
PHG9_Scaffold_2009	2	p2	(TA)8	16	12358	12373	potentially variable
PHG9_Scaffold_2009	4	p2	(AG)7	14	14906	14919	potentially variable
PHG9_Scaffold_2011	1	p2	(TA)6	12	1876	1887	
PHG9_Scaffold_2012	3	p2	(GA)6	12	10199	10210	
PHG9_Scaffold_2012	9	p3	(GAA)6	18	47563	47580	potentially

							variable
PHG9_Scaffold_2019	1	p2	(CT)8	16	128	143	potentially variable
PHG9_Scaffold_2019	2	p2	(GA)9	18	5114	5131	potentially variable
PHG9_Scaffold_2019	6	p3	(CTC)6	18	33252	33269	potentially variable
PHG9_Scaffold_2019	8	p2	(TA)6	12	37329	37340	
PHG9_Scaffold_2019	10	p3	(TCA)5	15	61549	61563	potentially variable
PHG9_Scaffold_2019	14	p2	(GA)6	12	88835	88846	
PHG9_Scaffold_2019	24	p2	(AG)6	12	123466	123477	
PHG9_Scaffold_2019	25	p6	(TGAGTT)5	30	124367	124396	Hypervariable
PHG9_Scaffold_2019	30	p3	(ATT)6	18	148074	148091	potentially variable
PHG9_Scaffold_2019	31	p2	(TA)6	12	155114	155125	
PHG9_Scaffold_2019	37	p2	(TG)7	14	167330	167343	potentially variable
PHG9_Scaffold_2020	2	p2	(AT)9	18	11077	11094	potentially variable
PHG9_Scaffold_2021	6	p2	(TA)6	12	21356	21367	
PHG9_Scaffold_2022	5	p2	(AT)9	18	13371	13388	potentially variable
PHG9_Scaffold_2022	6	p2	(AT)6	12	13855	13866	
PHG9_Scaffold_2025	8	p2	(AG)9	18	9387	9404	potentially variable
PHG9_Scaffold_2025	9	p2	(AG)6	12	10601	10612	
PHG9_Scaffold_2025	13	p3	(TTA)5	15	25134	25148	potentially variable

PHG9_Scaffold_2025	14	p3	(TCT)5	15	27968	27982	potentially variable
PHG9_Scaffold_2026	14	p2	(TA)8	16	22564	22579	potentially variable
PHG9_Scaffold_2027	2	p2	(GA)9	18	8631	8648	potentially variable
PHG9_Scaffold_2028	3	p2	(TC)6	12	5461	5472	
PHG9_Scaffold_2028	4	p2	(AT)6	12	15816	15827	
PHG9_Scaffold_2029	18	p2	(TG)8	16	75075	75090	potentially variable
PHG9_Scaffold_2029	23	p3	(ATC)5	15	78341	78355	potentially variable
PHG9_Scaffold_2029	29	p3	(TAA)5	15	89662	89676	potentially variable
PHG9_Scaffold_2029	37	p2	(AT)7	14	115270	115283	potentially variable
PHG9_Scaffold_2029	39	p2	(AG)6	12	122905	122916	
PHG9_Scaffold_2029	42	p2	(TA)7	14	128654	128667	potentially variable
PHG9_Scaffold_2029	47	p2	(TA)6	12	134248	134259	
PHG9_Scaffold_2029	52	p2	(TA)6	12	146159	146170	
PHG9_Scaffold_2029	57	p3	(AAT)5	15	155457	155471	potentially variable
PHG9_Scaffold_2029	64	p3	(TGG)5	15	171268	171282	potentially variable
PHG9_Scaffold_2029	70	p2	(TA)9	18	185583	185600	potentially variable
PHG9_Scaffold_2029	72	p2	(CT)6	12	192256	192267	
PHG9_Scaffold_2030	1	p2	(TA)7	14	121	134	potentially variable

PHG9_Scaffold_2030	9	p2	(AT)7	14	16258	16271	potentially variable
PHG9_Scaffold_2030	11	p2	(AT)6	12	21556	21567	
PHG9_Scaffold_2031	2	p2	(TA)8	16	10060	10075	potentially variable
PHG9_Scaffold_2031	4	p2	(TA)6	12	30590	30601	
PHG9_Scaffold_2031	7	p3	(TAT)5	15	36939	36953	potentially variable
PHG9_Scaffold_2031	23	p2	(TA)6	12	105363	105374	
PHG9_Scaffold_2032	2	p2	(AT)6	12	5966	5977	
PHG9_Scaffold_2032	6	p2	(AT)6	12	8957	8968	
PHG9_Scaffold_2033	2	p3	(TAA)6	18	4454	4471	potentially variable
PHG9_Scaffold_2033	11	p2	(TA)6	12	50824	50835	
PHG9_Scaffold_2033	20	p2	(GA)6	12	101095	101106	
PHG9_Scaffold_2033	21	p3	(TAT)5	15	106873	106887	potentially variable
PHG9_Scaffold_2033	23	p2	(TC)6	12	124933	124944	
PHG9_Scaffold_2034	2	p2	(AT)7	14	19226	19239	potentially variable
PHG9_Scaffold_2034	4	p3	(AAG)5	15	24091	24105	potentially variable
PHG9_Scaffold_2034	5	p3	(ATT)5	15	25940	25954	potentially variable
PHG9_Scaffold_2034	6	p3	(GAA)5	15	32339	32353	potentially variable
PHG9_Scaffold_2035	1	p3	(TAA)5	15	44	58	potentially variable
PHG9_Scaffold_2035	2	p3	(AGT)6	18	843	860	potentially

							variable
PHG9_Scaffold_2035	6	p2	(AT)8	16	19038	19053	potentially variable
PHG9_Scaffold_2036	1	p2	(TA)8	16	7525	7540	potentially variable
PHG9_Scaffold_2037	1	p2	(TA)9	18	3995	4012	potentially variable
PHG9_Scaffold_2037	24	p2	(TA)6	12	65409	65420	
PHG9_Scaffold_2037	27	p3	(TAA)5	15	71195	71209	potentially variable
PHG9_Scaffold_2037	28	p2	(TA)7	14	71997	72010	potentially variable
PHG9_Scaffold_2037	36	p6	(CTTCAA)6	36	83961	83996	Hypervariable
PHG9_Scaffold_2037	41	p2	(AT)6	12	113097	113108	
PHG9_Scaffold_2038	5	p2	(TA)8	16	9361	9376	potentially variable
PHG9_Scaffold_2039	1	p2	(AT)7	14	546	559	potentially variable
PHG9_Scaffold_2039	5	p2	(AG)7	14	18787	18800	potentially variable
PHG9_Scaffold_2040	11	p2	(TC)6	12	25372	25383	
PHG9_Scaffold_2040	16	p3	(GAT)6	18	36053	36070	potentially variable
PHG9_Scaffold_2040	17	p2	(AT)7	14	41262	41275	potentially variable
PHG9_Scaffold_2040	24	p2	(TA)6	12	58540	58551	
PHG9_Scaffold_2040	25	p3	(ATT)5	15	60162	60176	potentially variable
PHG9_Scaffold_2040	26	p2	(TC)7	14	61285	61298	potentially variable

PHG9_Scaffold_2040	28	p2	(CT)7	14	64685	64698	potentially variable
PHG9_Scaffold_2040	32	p2	(CT)6	12	72829	72840	
PHG9_Scaffold_2040	37	p3	(TAA)6	18	92520	92537	potentially variable
PHG9_Scaffold_2040	40	p2	(AT)8	16	98358	98373	potentially variable
PHG9_Scaffold_2040	44	p2	(TA)7	14	111141	111154	potentially variable
PHG9_Scaffold_2040	47	p3	(TAA)5	15	118192	118206	potentially variable
PHG9_Scaffold_2042	8	p2	(TA)9	18	16178	16195	potentially variable
PHG9_Scaffold_2042	12	p3	(ATG)5	15	40206	40220	potentially variable
PHG9_Scaffold_2042	14	p3	(TAA)5	15	42357	42371	potentially variable
PHG9_Scaffold_2043	1	p3	(TCA)5	15	10564	10578	potentially variable
PHG9_Scaffold_2043	5	p2	(TA)6	12	20924	20935	
PHG9_Scaffold_2043	12	p2	(TA)7	14	34574	34587	potentially variable
PHG9_Scaffold_2043	16	p2	(TA)9	18	43857	43874	potentially variable
PHG9_Scaffold_2043	18	p2	(GA)7	14	49349	49362	potentially variable
PHG9_Scaffold_2043	20	p2	(CT)6	12	57999	58010	
PHG9_Scaffold_2043	22	p2	(TA)7	14	62587	62600	potentially variable
PHG9_Scaffold_2044	9	p3	(TCT)5	15	38170	38184	potentially

							variable
PHG9_Scaffold_2044	10	p2	(GA)6	12	44431	44442	
PHG9_Scaffold_2045	6	p2	(AT)9	18	12108	12125	potentially variable
PHG9_Scaffold_2045	8	p2	(TA)7	14	14545	14558	potentially variable
PHG9_Scaffold_2045	9	p2	(AT)6	12	21568	21579	
PHG9_Scaffold_2047	3	p3	(AAT)6	18	13439	13456	potentially variable
PHG9_Scaffold_2047	7	p2	(GT)6	12	32129	32140	
PHG9_Scaffold_2047	8	p2	(AT)8	16	34317	34332	potentially variable
PHG9_Scaffold_2047	9	p2	(GA)6	12	42305	42316	
PHG9_Scaffold_2047	16	p3	(TTC)5	15	56971	56985	potentially variable
PHG9_Scaffold_2048	4	p3	(TTG)5	15	2461	2475	potentially variable
PHG9_Scaffold_2048	5	p3	(TTG)5	15	2668	2682	potentially variable
PHG9_Scaffold_2048	9	p3	(AAT)5	15	19669	19683	potentially variable
PHG9_Scaffold_2048	14	p2	(TA)6	12	35179	35190	
PHG9_Scaffold_2049	2	p2	(TC)6	12	1769	1780	
PHG9_Scaffold_2049	8	p3	(AAT)5	15	8957	8971	potentially variable
PHG9_Scaffold_2051	9	p2	(TA)8	16	15693	15708	potentially variable
PHG9_Scaffold_2051	12	p2	(AT)6	12	17385	17396	
PHG9_Scaffold_2052	6	p2	(AT)6	12	21054	21065	

PHG9_Scaffold_2053	4	p3	(ATA)5	15	16110	16124	potentially variable
PHG9_Scaffold_2053	13	p2	(TA)7	14	51768	51781	potentially variable
PHG9_Scaffold_2053	15	p2	(TG)6	12	57061	57072	
PHG9_Scaffold_2053	18	p2	(TA)8	16	75961	75976	potentially variable
PHG9_Scaffold_2054	2	p2	(GA)6	12	2596	2607	
PHG9_Scaffold_2054	8	p2	(TA)7	14	21678	21691	potentially variable
PHG9_Scaffold_2054	12	p3	(TGG)5	15	28730	28744	potentially variable
PHG9_Scaffold_2054	13	p3	(ATG)6	18	30278	30295	potentially variable
PHG9_Scaffold_2055	1	p2	(AG)6	12	11504	11515	
PHG9_Scaffold_2056	7	p2	(AT)6	12	49688	49699	
PHG9_Scaffold_2058	3	p2	(AT)6	12	16207	16218	
PHG9_Scaffold_2058	5	p2	(GA)6	12	30716	30727	
PHG9_Scaffold_2061	5	p2	(TA)8	16	7705	7720	potentially variable
PHG9_Scaffold_2061	8	p2	(TA)7	14	15414	15427	potentially variable
PHG9_Scaffold_2061	15	p2	(AT)9	18	31035	31052	potentially variable
PHG9_Scaffold_2061	18	p3	(TTA)5	15	35486	35500	potentially variable
PHG9_Scaffold_2061	19	p2	(GA)9	18	39249	39266	potentially variable
PHG9_Scaffold_2063	1	p2	(TA)7	14	5378	5391	potentially variable

PHG9_Scaffold_2063	2	p3	(ATA)5	15	6201	6215	potentially variable
PHG9_Scaffold_2063	3	p2	(AT)9	18	13564	13581	potentially variable
PHG9_Scaffold_2063	13	p3	(TCA)5	15	24637	24651	potentially variable
PHG9_Scaffold_2063	21	p2	(AT)6	12	53755	53766	
PHG9_Scaffold_2063	22	p3	(GGA)5	15	58251	58265	potentially variable
PHG9_Scaffold_2064	1	p3	(ATT)5	15	2683	2697	potentially variable
PHG9_Scaffold_2064	3	p2	(TC)6	12	13117	13128	
PHG9_Scaffold_2066	6	p2	(AG)6	12	15799	15810	
PHG9_Scaffold_2067	2	p2	(TA)6	12	966	977	
PHG9_Scaffold_2067	3	p2	(TA)9	18	4914	4931	potentially variable
PHG9_Scaffold_2067	6	p2	(GA)7	14	14110	14123	potentially variable
PHG9_Scaffold_2068	2	p2	(AT)6	12	815	826	
PHG9_Scaffold_2068	5	p2	(TC)9	18	6752	6769	potentially variable
PHG9_Scaffold_2068	10	p2	(TA)6	12	23087	23098	
PHG9_Scaffold_2068	17	p2	(TA)6	12	44015	44026	
PHG9_Scaffold_2069	17	p2	(TG)6	12	68919	68930	
PHG9_Scaffold_2070	1	p2	(TA)8	16	620	635	potentially variable
PHG9_Scaffold_2070	9	p3	(TTG)5	15	10882	10896	potentially variable
PHG9_Scaffold_2070	15	p2	(TA)9	18	49789	49806	potentially

							variable
PHG9_Scaffold_2070	24	p3	(CAC)5	15	71963	71977	potentially variable
PHG9_Scaffold_2070	25	p2	(TA)6	12	72820	72831	
PHG9_Scaffold_2070	46	p2	(AC)6	12	121098	121109	
PHG9_Scaffold_2071	4	p2	(CT)6	12	17529	17540	
PHG9_Scaffold_2071	8	p2	(TA)6	12	57097	57108	
PHG9_Scaffold_2072	1	p2	(AT)6	12	269	280	
PHG9_Scaffold_2072	2	p2	(AT)7	14	947	960	potentially variable
PHG9_Scaffold_2072	4	p3	(ATA)5	15	7053	7067	potentially variable
PHG9_Scaffold_2072	6	p2	(TA)7	14	14839	14852	potentially variable
PHG9_Scaffold_2072	9	p2	(TA)6	12	18871	18882	
PHG9_Scaffold_2072	16	p2	(AT)8	16	28303	28318	potentially variable
PHG9_Scaffold_2073	7	p2	(AT)6	12	28264	28275	
PHG9_Scaffold_2074	2	p3	(GTC)6	18	6153	6170	potentially variable
PHG9_Scaffold_2079	1	p2	(TG)6	12	636	647	
PHG9_Scaffold_2079	4	p3	(TTA)5	15	10215	10229	potentially variable
PHG9_Scaffold_2079	6	p2	(TA)6	12	11326	11337	
PHG9_Scaffold_2082	1	p2	(TC)6	12	910	921	
PHG9_Scaffold_2084	4	p2	(CT)6	12	12135	12146	
PHG9_Scaffold_2084	6	p2	(TA)7	14	17478	17491	potentially variable
PHG9_Scaffold_2085	2	p3	(GGA)5	15	10551	10565	potentially

							variable
PHG9_Scaffold_2087	5	p3	(TCT)5	15	17204	17218	potentially variable
PHG9_Scaffold_2087	10	p2	(GT)8	16	32003	32018	potentially variable
PHG9_Scaffold_2087	20	p3	(AGC)5	15	54284	54298	potentially variable
PHG9_Scaffold_2087	23	p2	(TA)6	12	64127	64138	
PHG9_Scaffold_2087	24	p2	(AT)7	14	64764	64777	potentially variable
PHG9_Scaffold_2088	5	p2	(GT)6	12	9407	9418	
PHG9_Scaffold_2088	8	p3	(AGT)5	15	14059	14073	potentially variable
PHG9_Scaffold_2088	10	p2	(AT)9	18	28018	28035	potentially variable
PHG9_Scaffold_2089	4	p2	(AT)9	18	4014	4031	potentially variable
PHG9_Scaffold_2089	10	p2	(TA)7	14	13351	13364	potentially variable
PHG9_Scaffold_2090	10	p2	(CT)7	14	38859	38872	potentially variable
PHG9_Scaffold_2093	1	p2	(TA)8	16	5897	5912	potentially variable
PHG9_Scaffold_2094	2	p2	(TA)9	18	3530	3547	potentially variable
PHG9_Scaffold_2094	6	p2	(TA)7	14	15694	15707	potentially variable
PHG9_Scaffold_2094	7	p2	(CA)6	12	18522	18533	
PHG9_Scaffold_2094	8	p2	(TA)9	18	20463	20480	potentially variable

PHG9_Scaffold_2094	10	p2	(AC)6	12	31020	31031	
PHG9_Scaffold_2095	6	p2	(TA)6	12	9651	9662	
PHG9_Scaffold_2095	8	p2	(TA)8	16	13164	13179	potentially variable
PHG9_Scaffold_2097	2	p3	(GAA)5	15	6971	6985	potentially variable
PHG9_Scaffold_2098	12	p3	(ATT)5	15	84703	84717	potentially variable
PHG9_Scaffold_2103	2	p2	(AT)6	12	1425	1436	
PHG9_Scaffold_2104	2	p3	(TGA)5	15	4933	4947	potentially variable
PHG9_Scaffold_2104	10	p2	(TA)7	14	43662	43675	potentially variable
PHG9_Scaffold_2104	11	p2	(TA)9	18	50154	50171	potentially variable
PHG9_Scaffold_2104	13	p3	(GGT)5	15	51903	51917	potentially variable
PHG9_Scaffold_2104	15	p2	(TA)7	14	56513	56526	potentially variable
PHG9_Scaffold_2104	25	p3	(ATT)6	18	173917	173934	potentially variable
PHG9_Scaffold_2104	28	p2	(AT)9	18	213471	213488	potentially variable
PHG9_Scaffold_2107	14	p3	(AAT)5	15	60154	60168	potentially variable
PHG9_Scaffold_2107	16	p2	(AT)8	16	66227	66242	potentially variable
PHG9_Scaffold_2107	21	p2	(TA)6	12	93311	93322	
PHG9_Scaffold_2107	22	p2	(AT)8	16	93952	93967	potentially variable

PHG9_Scaffold_2107	28	p2	(TA)8	16	115184	115199	potentially variable
PHG9_Scaffold_2107	31	p2	(AC)6	12	118204	118215	
PHG9_Scaffold_2107	35	p2	(AT)6	12	126079	126090	
PHG9_Scaffold_2107	39	p3	(GGT)5	15	134781	134795	potentially variable
PHG9_Scaffold_2107	41	p2	(TC)6	12	139486	139497	
PHG9_Scaffold_2107	46	p2	(TG)6	12	166747	166758	
PHG9_Scaffold_2107	49	p2	(TC)7	14	173160	173173	potentially variable
PHG9_Scaffold_2107	53	p2	(AT)7	14	184875	184888	potentially variable
PHG9_Scaffold_2107	55	p2	(GA)6	12	191062	191073	
PHG9_Scaffold_2107	56	p2	(CT)8	16	191259	191274	potentially variable
PHG9_Scaffold_2107	60	p3	(ATT)5	15	200904	200918	potentially variable
PHG9_Scaffold_2107	61	p3	(TAT)6	18	201317	201334	potentially variable
PHG9_Scaffold_2107	62	p3	(ATT)5	15	201592	201606	potentially variable
PHG9_Scaffold_2109	2	p2	(TA)8	16	4042	4057	potentially variable
PHG9_Scaffold_2110	6	p2	(TA)7	14	23923	23936	potentially variable
PHG9_Scaffold_2110	11	p2	(GA)7	14	64069	64082	potentially variable
PHG9_Scaffold_2111	5	p2	(AG)8	16	13132	13147	potentially variable
PHG9_Scaffold_2111	6	p2	(TG)6	12	14113	14124	

PHG9_Scaffold_2111	7	p3	(AGA)5	15	15641	15655	potentially variable
PHG9_Scaffold_2111	8	p2	(TA)6	12	22038	22049	
PHG9_Scaffold_2111	9	p3	(GTT)6	18	26606	26623	potentially variable
PHG9_Scaffold_2111	15	p2	(AG)8	16	46704	46719	potentially variable
PHG9_Scaffold_2111	18	p2	(TA)8	16	55044	55059	potentially variable
PHG9_Scaffold_2111	30	p3	(TTA)5	15	98326	98340	potentially variable
PHG9_Scaffold_2111	34	p2	(AT)8	16	118690	118705	potentially variable
PHG9_Scaffold_2112	15	p2	(AT)7	14	36782	36795	potentially variable
PHG9_Scaffold_2112	16	p2	(TA)7	14	39391	39404	potentially variable
PHG9_Scaffold_2113	4	p2	(AT)8	16	9313	9328	potentially variable
PHG9_Scaffold_2113	7	p3	(ATT)5	15	29240	29254	potentially variable
PHG9_Scaffold_2113	8	p3	(AAT)5	15	31807	31821	potentially variable
PHG9_Scaffold_2113	14	p3	(TCT)5	15	46798	46812	potentially variable
PHG9_Scaffold_2113	20	p2	(AT)6	12	60033	60044	
PHG9_Scaffold_2113	22	p3	(GAA)6	18	65023	65040	potentially variable
PHG9_Scaffold_2115	2	p3	(TAT)5	15	23843	23857	potentially variable

PHG9_Scaffold_2117	3	p3	(TAT)5	15	1839	1853	potentially variable
PHG9_Scaffold_2117	4	p2	(TA)6	12	2417	2428	
PHG9_Scaffold_2119	9	p2	(AT)6	12	38272	38283	
PHG9_Scaffold_2119	17	p2	(TA)8	16	76289	76304	potentially variable
PHG9_Scaffold_2120	1	p2	(TA)6	12	23792	23803	
PHG9_Scaffold_2122	3	p2	(TA)7	14	19471	19484	potentially variable
PHG9_Scaffold_2123	3	p2	(CA)7	14	1194	1207	potentially variable
PHG9_Scaffold_2123	5	p2	(AT)8	16	5665	5680	potentially variable
PHG9_Scaffold_2124	5	p3	(ATA)5	15	14427	14441	potentially variable
PHG9_Scaffold_2125	3	p2	(TA)6	12	14500	14511	
PHG9_Scaffold_2125	6	p2	(TA)6	12	26124	26135	
PHG9_Scaffold_2125	11	p2	(AT)9	18	39562	39579	potentially variable
PHG9_Scaffold_2125	13	p2	(AT)7	14	43376	43389	potentially variable
PHG9_Scaffold_2129	6	p2	(CT)8	16	6986	7001	potentially variable
PHG9_Scaffold_2129	11	p3	(TGA)5	15	17343	17357	potentially variable
PHG9_Scaffold_2129	18	p2	(AT)7	14	28946	28959	potentially variable
PHG9_Scaffold_2129	19	p2	(AT)9	18	33305	33322	potentially variable
PHG9_Scaffold_2130	2	p2	(AT)6	12	14546	14557	

PHG9_Scaffold_2130	11	p2	(TA)8	16	37970	37985	potentially variable
PHG9_Scaffold_2131	5	p2	(TA)7	14	15089	15102	potentially variable
PHG9_Scaffold_2132	3	p3	(TTA)5	15	7307	7321	potentially variable
PHG9_Scaffold_2132	4	p2	(AT)6	12	7681	7692	
PHG9_Scaffold_2132	6	p2	(CT)8	16	10882	10897	potentially variable
PHG9_Scaffold_2132	10	p3	(TTG)5	15	14533	14547	potentially variable
PHG9_Scaffold_2132	11	p2	(AT)8	16	16929	16944	potentially variable
PHG9_Scaffold_2133	6	p2	(TA)7	14	28991	29004	potentially variable
PHG9_Scaffold_2133	8	p2	(TA)7	14	31600	31613	potentially variable
PHG9_Scaffold_2137	2	p3	(CTC)5	15	8628	8642	potentially variable
PHG9_Scaffold_2137	3	p2	(TA)6	12	9747	9758	
PHG9_Scaffold_2137	5	p2	(AT)7	14	20061	20074	potentially variable
PHG9_Scaffold_2137	8	p2	(TA)9	18	21552	21569	potentially variable
PHG9_Scaffold_2137	10	p2	(AT)6	12	23688	23699	
PHG9_Scaffold_2138	1	p2	(TA)6	12	1701	1712	
PHG9_Scaffold_2140	1	p2	(TC)6	12	543	554	
PHG9_Scaffold_2140	5	p3	(CAG)6	18	24265	24282	potentially variable
PHG9_Scaffold_2141	8	p2	(TC)6	12	7588	7599	

PHG9_Scaffold_2141	13	p2	(TC)7	14	14451	14464	potentially variable
PHG9_Scaffold_2141	14	p3	(TAT)5	15	28020	28034	potentially variable
PHG9_Scaffold_2141	17	p2	(TA)7	14	95745	95758	potentially variable
PHG9_Scaffold_2141	18	p2	(CA)6	12	98165	98176	
PHG9_Scaffold_2141	25	p2	(AT)6	12	149911	149922	
PHG9_Scaffold_2143	10	p2	(TA)6	12	42766	42777	
PHG9_Scaffold_2143	19	p2	(TA)8	16	77203	77218	potentially variable
PHG9_Scaffold_2143	35	p3	(AAT)5	15	110315	110329	potentially variable
PHG9_Scaffold_2143	36	p2	(AT)7	14	113785	113798	potentially variable
PHG9_Scaffold_2143	37	p2	(AT)8	16	114309	114324	potentially variable
PHG9_Scaffold_2144	3	p3	(ATC)5	15	14854	14868	potentially variable
PHG9_Scaffold_2145	11	p2	(TA)8	16	61656	61671	potentially variable
PHG9_Scaffold_2145	36	p2	(TC)8	16	122985	123000	potentially variable
PHG9_Scaffold_2145	44	p3	(TTG)5	15	150393	150407	potentially variable
PHG9_Scaffold_2145	47	p3	(AAC)5	15	163847	163861	potentially variable
PHG9_Scaffold_2145	77	p2	(AC)8	16	248413	248428	potentially variable
PHG9_Scaffold_2145	83	p2	(CT)7	14	261549	261562	potentially

							variable
PHG9_Scaffold_2145	85	p3	(GGT)6	18	270487	270504	potentially variable
PHG9_Scaffold_2145	86	p2	(AT)9	18	271239	271256	potentially variable
PHG9_Scaffold_2147	1	p2	(AT)7	14	4172	4185	potentially variable
PHG9_Scaffold_2151	2	p2	(AT)7	14	3195	3208	potentially variable
PHG9_Scaffold_2151	3	p2	(TA)6	12	3377	3388	
PHG9_Scaffold_2157	1	p2	(TA)8	16	1006	1021	potentially variable
PHG9_Scaffold_2157	3	p2	(AT)7	14	8493	8506	potentially variable
PHG9_Scaffold_2157	8	p3	(CTC)5	15	21094	21108	potentially variable
PHG9_Scaffold_2159	7	p2	(AT)6	12	12864	12875	
PHG9_Scaffold_2159	9	p3	(TAT)5	15	14934	14948	potentially variable
PHG9_Scaffold_2160	4	p2	(AT)8	16	16718	16733	potentially variable
PHG9_Scaffold_2160	5	p2	(AT)8	16	20571	20586	potentially variable
PHG9_Scaffold_2162	7	p2	(TA)7	14	9663	9676	potentially variable
PHG9_Scaffold_2163	1	p2	(TA)6	12	665	676	
PHG9_Scaffold_2163	2	p2	(TA)7	14	2062	2075	potentially variable
PHG9_Scaffold_2163	3	p3	(ATT)5	15	2380	2394	potentially variable

PHG9_Scaffold_2163	5	p3	(TAA)5	15	14201	14215	potentially variable
PHG9_Scaffold_2163	11	p2	(TC)6	12	22827	22838	
PHG9_Scaffold_2163	16	p2	(AG)6	12	40144	40155	
PHG9_Scaffold_2163	17	p2	(AT)7	14	40865	40878	potentially variable
PHG9_Scaffold_2165	1	p3	(CTT)5	15	3368	3382	potentially variable
PHG9_Scaffold_2167	4	p2	(TA)6	12	13282	13293	
PHG9_Scaffold_2167	8	p2	(AT)8	16	18334	18349	potentially variable
PHG9_Scaffold_2167	9	p3	(TGT)5	15	18466	18480	potentially variable
PHG9_Scaffold_2167	10	p2	(GT)9	18	20178	20195	potentially variable
PHG9_Scaffold_2170	4	p3	(CAA)5	15	2455	2469	potentially variable
PHG9_Scaffold_2172	6	p2	(AT)8	16	11594	11609	potentially variable
PHG9_Scaffold_2172	7	p2	(TA)8	16	12445	12460	potentially variable
PHG9_Scaffold_2176	1	p2	(AT)6	12	44	55	
PHG9_Scaffold_2176	6	p3	(ACC)5	15	6390	6404	potentially variable
PHG9_Scaffold_2176	9	p2	(GA)7	14	18937	18950	potentially variable
PHG9_Scaffold_2177	1	p3	(ATA)5	15	3570	3584	potentially variable
PHG9_Scaffold_2180	1	p2	(AT)8	16	1751	1766	potentially variable

PHG9_Scaffold_2184	3	p3	(CAC)5	15	9077	9091	potentially variable
PHG9_Scaffold_2184	5	p3	(CTC)5	15	12830	12844	potentially variable
PHG9_Scaffold_2184	9	p2	(GT)7	14	24105	24118	potentially variable
PHG9_Scaffold_2187	2	p3	(TAT)6	18	3373	3390	potentially variable
PHG9_Scaffold_2187	6	p2	(TA)8	16	14283	14298	potentially variable
PHG9_Scaffold_2187	9	p3	(CCA)6	18	18828	18845	potentially variable
PHG9_Scaffold_2187	17	p2	(TA)6	12	56225	56236	
PHG9_Scaffold_2191	3	p2	(TA)6	12	2815	2826	
PHG9_Scaffold_2197	1	p3	(AAG)6	18	371	388	potentially variable
PHG9_Scaffold_2206	4	p3	(TAT)5	15	18376	18390	potentially variable
PHG9_Scaffold_2206	6	p2	(TC)6	12	30995	31006	
PHG9_Scaffold_2206	16	p2	(AC)7	14	50694	50707	potentially variable
PHG9_Scaffold_2206	20	p3	(TAT)5	15	56978	56992	potentially variable
PHG9_Scaffold_2206	41	p2	(TA)8	16	128238	128253	potentially variable
PHG9_Scaffold_2207	3	p3	(AGA)5	15	2407	2421	potentially variable
PHG9_Scaffold_2207	24	p3	(TAT)5	15	46178	46192	potentially variable
PHG9_Scaffold_2207	28	p2	(AT)9	18	56778	56795	potentially

							variable
PHG9_Scaffold_2207	37	p2	(TA)6	12	88341	88352	
PHG9_Scaffold_2207	52	p2	(GA)8	16	136636	136651	potentially variable
PHG9_Scaffold_2207	53	p2	(TC)7	14	137750	137763	potentially variable
PHG9_Scaffold_2207	56	p2	(AT)7	14	143406	143419	potentially variable
PHG9_Scaffold_2207	59	p2	(AT)7	14	148844	148857	potentially variable
PHG9_Scaffold_2207	63	p2	(TA)8	16	160547	160562	potentially variable
PHG9_Scaffold_2207	69	p3	(TAT)5	15	171005	171019	potentially variable
PHG9_Scaffold_2207	70	p3	(AAT)5	15	171724	171738	potentially variable
PHG9_Scaffold_2207	72	p3	(AAG)5	15	176268	176282	potentially variable
PHG9_Scaffold_2207	73	p2	(AT)8	16	179605	179620	potentially variable
PHG9_Scaffold_2207	75	p2	(CT)7	14	183663	183676	potentially variable
PHG9_Scaffold_2207	76	p2	(AT)7	14	189649	189662	potentially variable
PHG9_Scaffold_2207	78	p2	(CA)6	12	198614	198625	
PHG9_Scaffold_2208	4	p2	(AT)7	14	2599	2612	potentially variable
PHG9_Scaffold_2208	5	p2	(TA)6	12	3296	3307	
PHG9_Scaffold_2208	7	p2	(AT)9	18	7481	7498	potentially variable

PHG9_Scaffold_2208	9	p2	(TA)6	12	14782	14793	
PHG9_Scaffold_2211	2	p2	(TG)6	12	14052	14063	
PHG9_Scaffold_2211	5	p2	(TA)7	14	23147	23160	potentially variable
PHG9_Scaffold_2212	5	p2	(TA)6	12	4272	4283	
PHG9_Scaffold_2212	9	p3	(AAT)5	15	11821	11835	potentially variable
PHG9_Scaffold_2212	18	p2	(TA)8	16	22639	22654	potentially variable
PHG9_Scaffold_2214	3	p2	(AT)6	12	3598	3609	
PHG9_Scaffold_2214	4	p2	(TA)7	14	4240	4253	potentially variable
PHG9_Scaffold_2214	8	p2	(TA)9	18	9164	9181	potentially variable
PHG9_Scaffold_2214	10	p3	(AAG)5	15	14417	14431	potentially variable
PHG9_Scaffold_2215	9	p2	(TA)8	16	11773	11788	potentially variable
PHG9_Scaffold_2215	10	p2	(AT)6	12	13813	13824	
PHG9_Scaffold_2215	12	p2	(TC)9	18	18837	18854	potentially variable
PHG9_Scaffold_2216	1	p3	(TGT)5	15	282	296	potentially variable
PHG9_Scaffold_2216	2	p2	(TA)9	18	6939	6956	potentially variable
PHG9_Scaffold_2216	8	p2	(AT)8	16	34123	34138	potentially variable
PHG9_Scaffold_2218	8	p2	(TA)6	12	31349	31360	
PHG9_Scaffold_2221	3	p2	(AT)6	12	20261	20272	

PHG9_Scaffold_2221	4	p2	(TA)6	12	20459	20470	
PHG9_Scaffold_2221	6	p2	(TA)6	12	21507	21518	
PHG9_Scaffold_2221	7	p3	(GGT)5	15	24489	24503	potentially variable
PHG9_Scaffold_2223	4	p2	(AT)7	14	34122	34135	potentially variable
PHG9_Scaffold_2223	5	p3	(AGA)5	15	40493	40507	potentially variable
PHG9_Scaffold_2223	6	p3	(TAA)5	15	40675	40689	potentially variable
PHG9_Scaffold_2224	4	p2	(AT)6	12	6459	6470	
PHG9_Scaffold_2225	6	p2	(AT)8	16	25667	25682	potentially variable
PHG9_Scaffold_2226	3	p3	(TAA)6	18	24210	24227	potentially variable
PHG9_Scaffold_2231	4	p3	(GAG)5	15	10109	10123	potentially variable
PHG9_Scaffold_2235	1	p3	(AAG)6	18	286	303	potentially variable
PHG9_Scaffold_2235	2	p2	(TA)7	14	2043	2056	potentially variable
PHG9_Scaffold_2238	4	p3	(ATA)5	15	6657	6671	potentially variable
PHG9_Scaffold_2240	6	p2	(AT)9	18	15542	15559	potentially variable
PHG9_Scaffold_2240	10	p2	(AT)9	18	27874	27891	potentially variable
PHG9_Scaffold_2240	20	p3	(AAT)5	15	42813	42827	potentially variable
PHG9_Scaffold_2240	25	p2	(TA)6	12	50523	50534	

PHG9_Scaffold_2240	29	p3	(ATA)5	15	55917	55931	potentially variable
PHG9_Scaffold_2241	4	p2	(TA)8	16	3875	3890	potentially variable
PHG9_Scaffold_2241	6	p3	(ACA)6	18	11016	11033	potentially variable
PHG9_Scaffold_2241	14	p2	(TC)7	14	30826	30839	potentially variable
PHG9_Scaffold_2241	16	p2	(AT)7	14	40300	40313	potentially variable
PHG9_Scaffold_2241	26	p2	(TA)7	14	57305	57318	potentially variable
PHG9_Scaffold_2242	4	p3	(CAA)5	15	13130	13144	potentially variable
PHG9_Scaffold_2243	1	p2	(AT)6	12	7902	7913	
PHG9_Scaffold_2243	10	p3	(TAA)5	15	48326	48340	potentially variable
PHG9_Scaffold_2243	13	p2	(AT)6	12	59864	59875	
PHG9_Scaffold_2243	21	p3	(TAT)6	18	91598	91615	potentially variable
PHG9_Scaffold_2245	4	p2	(GA)6	12	4874	4885	
PHG9_Scaffold_2245	7	p3	(GAA)6	18	14357	14374	potentially variable
PHG9_Scaffold_2245	10	p3	(TAT)5	15	21825	21839	potentially variable
PHG9_Scaffold_2246	4	p2	(TC)7	14	2854	2867	potentially variable
PHG9_Scaffold_2246	8	p2	(AT)8	16	10665	10680	potentially variable
PHG9_Scaffold_2246	10	p3	(CTT)5	15	14097	14111	potentially

							variable
PHG9_Scaffold_2246	12	p3	(TCT)5	15	24117	24131	potentially variable
PHG9_Scaffold_2247	3	p2	(AT)7	14	7311	7324	potentially variable
PHG9_Scaffold_2248	1	p2	(GT)7	14	7448	7461	potentially variable
PHG9_Scaffold_2248	5	p2	(TA)6	12	13459	13470	
PHG9_Scaffold_2250	2	p2	(AG)7	14	10587	10600	potentially variable
PHG9_Scaffold_2251	4	p2	(AT)6	12	17007	17018	
PHG9_Scaffold_2252	1	p2	(TA)7	14	168	181	potentially variable
PHG9_Scaffold_2252	4	p3	(TAA)5	15	12391	12405	potentially variable
PHG9_Scaffold_2253	9	p2	(GA)6	12	38549	38560	
PHG9_Scaffold_2253	10	p2	(AT)7	14	38776	38789	potentially variable
PHG9_Scaffold_2253	17	p3	(ATA)5	15	48267	48281	potentially variable
PHG9_Scaffold_2253	24	p3	(CAG)6	18	63403	63420	potentially variable
PHG9_Scaffold_2254	1	p3	(ATA)6	18	142	159	potentially variable
PHG9_Scaffold_2254	3	p3	(AAT)5	15	3663	3677	potentially variable
PHG9_Scaffold_2255	2	p3	(ATA)5	15	7250	7264	potentially variable
PHG9_Scaffold_2259	1	p2	(TA)7	14	401	414	potentially variable

PHG9_Scaffold_2259	2	p2	(AT)8	16	724	739	potentially variable
PHG9_Scaffold_2259	3	p2	(TA)7	14	1613	1626	potentially variable
PHG9_Scaffold_2259	4	p2	(AT)7	14	13123	13136	potentially variable
PHG9_Scaffold_2259	12	p2	(TA)6	12	55732	55743	
PHG9_Scaffold_2259	18	p2	(GA)6	12	72231	72242	
PHG9_Scaffold_2259	23	p2	(TA)9	18	80081	80098	potentially variable
PHG9_Scaffold_2260	2	p2	(CT)7	14	10487	10500	potentially variable
PHG9_Scaffold_2260	8	p2	(TA)9	18	21863	21880	potentially variable
PHG9_Scaffold_2261	6	p2	(AT)6	12	5179	5190	
PHG9_Scaffold_2263	5	p2	(TA)9	18	13756	13773	potentially variable
PHG9_Scaffold_2263	15	p3	(TAT)5	15	39798	39812	potentially variable
PHG9_Scaffold_2265	1	p2	(TA)6	12	11609	11620	
PHG9_Scaffold_2269	4	p2	(AT)6	12	18541	18552	
PHG9_Scaffold_2270	1	p3	(AGA)5	15	969	983	potentially variable
PHG9_Scaffold_2272	1	p2	(CA)7	14	1268	1281	potentially variable
PHG9_Scaffold_2272	2	p2	(TA)6	12	2174	2185	
PHG9_Scaffold_2275	1	p2	(TA)9	18	4095	4112	potentially variable
PHG9_Scaffold_2275	4	p2	(TA)7	14	10624	10637	potentially variable

PHG9_Scaffold_2277	11	p2	(TA)6	12	52883	52894	
PHG9_Scaffold_2277	12	p2	(TC)6	12	55714	55725	
PHG9_Scaffold_2277	19	p2	(AC)6	12	68781	68792	
PHG9_Scaffold_2277	30	p2	(TC)7	14	115675	115688	potentially variable
PHG9_Scaffold_2277	31	p2	(AT)7	14	116410	116423	potentially variable
PHG9_Scaffold_2277	32	p3	(CAC)5	15	121376	121390	potentially variable
PHG9_Scaffold_2277	33	p3	(TAT)5	15	127282	127296	potentially variable
PHG9_Scaffold_2277	34	p3	(TTA)5	15	127453	127467	potentially variable
PHG9_Scaffold_2277	35	p2	(TA)7	14	130353	130366	potentially variable
PHG9_Scaffold_2278	10	p2	(TG)7	14	31266	31279	potentially variable
PHG9_Scaffold_2278	13	p2	(TA)8	16	43574	43589	potentially variable
PHG9_Scaffold_2278	14	p2	(AT)9	18	49298	49315	potentially variable
PHG9_Scaffold_2278	15	p3	(AAT)6	18	53580	53597	potentially variable
PHG9_Scaffold_2278	16	p3	(TGA)5	15	53772	53786	potentially variable
PHG9_Scaffold_2278	17	p2	(TA)6	12	54769	54780	
PHG9_Scaffold_2279	4	p2	(TA)8	16	10044	10059	potentially variable
PHG9_Scaffold_2279	6	p2	(GA)7	14	10635	10648	potentially variable

PHG9_Scaffold_2280	2	p3	(CGA)5	15	4373	4387	potentially variable
PHG9_Scaffold_2280	3	p2	(GT)7	14	6737	6750	potentially variable
PHG9_Scaffold_2280	4	p2	(TA)7	14	8281	8294	potentially variable
PHG9_Scaffold_2280	5	p2	(TC)6	12	15036	15047	
PHG9_Scaffold_2281	2	p2	(AT)6	12	2596	2607	
PHG9_Scaffold_2281	4	p2	(AT)6	12	8802	8813	
PHG9_Scaffold_2281	6	p2	(AT)6	12	15009	15020	
PHG9_Scaffold_2281	8	p2	(AT)6	12	21215	21226	
PHG9_Scaffold_2281	10	p2	(AT)6	12	27421	27432	
PHG9_Scaffold_2283	2	p2	(TA)6	12	1736	1747	
PHG9_Scaffold_2283	5	p2	(GT)6	12	6640	6651	
PHG9_Scaffold_2286	2	p2	(TC)6	12	1749	1760	
PHG9_Scaffold_2286	3	p3	(GGT)6	18	1889	1906	potentially variable
PHG9_Scaffold_2286	5	p2	(AT)7	14	13679	13692	potentially variable
PHG9_Scaffold_2287	9	p2	(TA)6	12	34740	34751	
PHG9_Scaffold_2288	5	p3	(ATA)6	18	8819	8836	potentially variable
PHG9_Scaffold_2289	1	p3	(TTA)5	15	1368	1382	potentially variable
PHG9_Scaffold_2289	14	p2	(TA)6	12	27002	27013	
PHG9_Scaffold_2289	15	p3	(AAT)5	15	35333	35347	potentially variable
PHG9_Scaffold_2289	19	p2	(AT)6	12	43214	43225	
PHG9_Scaffold_2289	20	p2	(TA)6	12	44480	44491	

PHG9_Scaffold_2289	23	p3	(TTA)5	15	49898	49912	potentially variable
PHG9_Scaffold_2289	24	p3	(TTC)5	15	50741	50755	potentially variable
PHG9_Scaffold_2289	27	p2	(AT)9	18	52236	52253	potentially variable
PHG9_Scaffold_2289	28	p2	(AT)8	16	55820	55835	potentially variable
PHG9_Scaffold_2290	9	p3	(TCT)5	15	24923	24937	potentially variable
PHG9_Scaffold_2290	10	p2	(TA)8	16	27212	27227	potentially variable
PHG9_Scaffold_2290	11	p2	(TA)8	16	28133	28148	potentially variable
PHG9_Scaffold_2292	3	p3	(AAT)5	15	5532	5546	potentially variable
PHG9_Scaffold_2292	5	p2	(AT)6	12	25769	25780	
PHG9_Scaffold_2293	1	p2	(AT)8	16	127	142	potentially variable
PHG9_Scaffold_2293	9	p2	(TC)6	12	20815	20826	
PHG9_Scaffold_2293	10	p2	(AT)7	14	21302	21315	potentially variable
PHG9_Scaffold_2295	7	p2	(TA)7	14	33432	33445	potentially variable
PHG9_Scaffold_2296	3	p2	(TA)6	12	2592	2603	
PHG9_Scaffold_2296	6	p2	(CT)7	14	26087	26100	potentially variable
PHG9_Scaffold_2296	8	p2	(TA)7	14	34937	34950	potentially variable
PHG9_Scaffold_2300	5	p2	(AT)6	12	9719	9730	

PHG9_Scaffold_2301	1	p2	(TA)6	12	4985	4996	
PHG9_Scaffold_2303	14	p3	(TAG)5	15	54469	54483	potentially variable
PHG9_Scaffold_2303	23	p2	(AT)7	14	68997	69010	potentially variable
PHG9_Scaffold_2303	25	p2	(AT)6	12	70862	70873	
PHG9_Scaffold_2303	41	p3	(GAT)6	18	107968	107985	potentially variable
PHG9_Scaffold_2303	52	p3	(AAT)6	18	141199	141216	potentially variable
PHG9_Scaffold_2303	60	p3	(GGC)5	15	158376	158390	potentially variable
PHG9_Scaffold_2303	68	p2	(AT)8	16	178537	178552	potentially variable
PHG9_Scaffold_2303	72	p3	(TCA)5	15	197555	197569	potentially variable
PHG9_Scaffold_2305	1	p2	(TA)8	16	840	855	potentially variable
PHG9_Scaffold_2307	2	p2	(TA)7	14	12429	12442	potentially variable
PHG9_Scaffold_2307	5	p2	(TA)7	14	32664	32677	potentially variable
PHG9_Scaffold_2308	5	p2	(TA)9	18	8791	8808	potentially variable
PHG9_Scaffold_2308	16	p2	(AT)6	12	22661	22672	
PHG9_Scaffold_2308	19	p2	(AT)6	12	27917	27928	
PHG9_Scaffold_2308	21	p2	(AT)7	14	36109	36122	potentially variable
PHG9_Scaffold_2308	25	p2	(TA)8	16	45331	45346	potentially variable

PHG9_Scaffold_2308	29	p2	(CT)6	12	52358	52369	
PHG9_Scaffold_2308	32	p2	(TA)6	12	55798	55809	
PHG9_Scaffold_2308	41	p3	(CAA)5	15	74934	74948	potentially variable
PHG9_Scaffold_2308	46	p2	(AT)6	12	86934	86945	
PHG9_Scaffold_2308	52	p3	(GTG)5	15	101842	101856	potentially variable
PHG9_Scaffold_2308	54	p2	(CT)6	12	107073	107084	
PHG9_Scaffold_2312	4	p2	(TA)7	14	5698	5711	potentially variable
PHG9_Scaffold_2313	1	p2	(TC)7	14	633	646	potentially variable
PHG9_Scaffold_2313	8	p2	(TA)7	14	14218	14231	potentially variable
PHG9_Scaffold_2314	8	p2	(TA)7	14	19132	19145	potentially variable
PHG9_Scaffold_2314	18	p2	(AT)8	16	35168	35183	potentially variable
PHG9_Scaffold_2315	1	p2	(AT)7	14	1223	1236	potentially variable
PHG9_Scaffold_2316	4	p3	(ATG)5	15	22545	22559	potentially variable
PHG9_Scaffold_2316	5	p3	(TTA)5	15	27022	27036	potentially variable
PHG9_Scaffold_2318	6	p3	(ATT)5	15	29715	29729	potentially variable
PHG9_Scaffold_2320	2	p2	(TA)6	12	5921	5932	
PHG9_Scaffold_2321	4	p2	(AT)6	12	12351	12362	
PHG9_Scaffold_2321	8	p2	(TA)7	14	28538	28551	potentially variable

PHG9_Scaffold_2321	16	p2	(TG)8	16	42038	42053	potentially variable
PHG9_Scaffold_2321	17	p2	(TA)6	12	49557	49568	
PHG9_Scaffold_2321	21	p3	(TAA)5	15	60531	60545	potentially variable
PHG9_Scaffold_2321	25	p2	(CT)7	14	62952	62965	potentially variable
PHG9_Scaffold_2321	41	p3	(GAA)5	15	117775	117789	potentially variable
PHG9_Scaffold_2321	42	p2	(CT)7	14	118039	118052	potentially variable
PHG9_Scaffold_2321	43	p3	(ATG)5	15	120100	120114	potentially variable
PHG9_Scaffold_2321	45	p2	(AT)6	12	122930	122941	
PHG9_Scaffold_2321	47	p2	(TA)7	14	125026	125039	potentially variable
PHG9_Scaffold_2321	56	p2	(AT)6	12	147436	147447	
PHG9_Scaffold_2321	68	p2	(TA)7	14	183969	183982	potentially variable
PHG9_Scaffold_2321	69	p2	(TA)7	14	186888	186901	potentially variable
PHG9_Scaffold_2321	79	p2	(GA)6	12	224026	224037	
PHG9_Scaffold_2322	2	p3	(ACA)5	15	3038	3052	potentially variable
PHG9_Scaffold_2322	5	p2	(TA)8	16	11651	11666	potentially variable
PHG9_Scaffold_2323	8	p2	(AT)6	12	42220	42231	
PHG9_Scaffold_2323	11	p3	(TTA)6	18	44047	44064	potentially variable
PHG9_Scaffold_2323	14	p3	(TCT)5	15	52855	52869	potentially

							variable
PHG9_Scaffold_2323	27	p3	(AAT)5	15	97120	97134	potentially variable
PHG9_Scaffold_2323	30	p2	(GA)7	14	111120	111133	potentially variable
PHG9_Scaffold_2323	31	p3	(TTA)5	15	120942	120956	potentially variable
PHG9_Scaffold_2323	38	p2	(TA)6	12	194952	194963	
PHG9_Scaffold_2323	39	p2	(AT)8	16	199883	199898	potentially variable
PHG9_Scaffold_2323	40	p2	(AT)8	16	205532	205547	potentially variable
PHG9_Scaffold_2324	1	p2	(AT)6	12	1616	1627	
PHG9_Scaffold_2325	6	p2	(AT)8	16	23610	23625	potentially variable
PHG9_Scaffold_2325	12	p2	(AT)6	12	37202	37213	
PHG9_Scaffold_2325	16	p3	(AAT)5	15	48991	49005	potentially variable
PHG9_Scaffold_2325	17	p2	(AT)6	12	52931	52942	
PHG9_Scaffold_2325	18	p2	(TG)6	12	56825	56836	
PHG9_Scaffold_2325	20	p2	(AT)7	14	63803	63816	potentially variable
PHG9_Scaffold_2326	1	p2	(TA)6	12	438	449	
PHG9_Scaffold_2326	6	p2	(AT)6	12	14813	14824	
PHG9_Scaffold_2327	2	p2	(AG)6	12	3077	3088	
PHG9_Scaffold_2327	9	p2	(TA)6	12	34618	34629	
PHG9_Scaffold_2329	9	p3	(TTA)5	15	19567	19581	potentially variable
PHG9_Scaffold_2329	17	p2	(TC)7	14	31846	31859	potentially

							variable
PHG9_Scaffold_2330	1	p2	(AT)6	12	691	702	
PHG9_Scaffold_2330	2	p2	(TA)8	16	1768	1783	potentially variable
PHG9_Scaffold_2330	13	p2	(AT)6	12	48600	48611	
PHG9_Scaffold_2330	15	p2	(GA)6	12	56132	56143	
PHG9_Scaffold_2330	19	p3	(AAT)5	15	61243	61257	potentially variable
PHG9_Scaffold_2330	27	p3	(CAT)5	15	82394	82408	potentially variable
PHG9_Scaffold_2331	4	p3	(TAC)6	18	5318	5335	potentially variable
PHG9_Scaffold_2333	12	p2	(TA)7	14	30655	30668	potentially variable
PHG9_Scaffold_2335	2	p2	(GA)7	14	5923	5936	potentially variable
PHG9_Scaffold_2336	8	p3	(ATT)6	18	22956	22973	potentially variable
PHG9_Scaffold_2337	1	p2	(TA)6	12	12937	12948	
PHG9_Scaffold_2337	3	p2	(TA)9	18	34236	34253	potentially variable
PHG9_Scaffold_2338	4	p3	(TGG)6	18	19310	19327	potentially variable
PHG9_Scaffold_2338	6	p2	(TA)6	12	22012	22023	
PHG9_Scaffold_2338	10	p2	(AT)7	14	33188	33201	potentially variable
PHG9_Scaffold_2338	11	p2	(TA)7	14	35678	35691	potentially variable
PHG9_Scaffold_2340	2	p3	(TAA)5	15	564	578	potentially variable

PHG9_Scaffold_2340	11	p3	(GAA)5	15	28667	28681	potentially variable
PHG9_Scaffold_2340	18	p2	(TA)6	12	43883	43894	
PHG9_Scaffold_2342	3	p3	(CAA)6	18	2293	2310	potentially variable
PHG9_Scaffold_2343	7	p2	(TA)6	12	14996	15007	
PHG9_Scaffold_2345	11	p2	(GT)7	14	22231	22244	potentially variable
PHG9_Scaffold_2345	16	p3	(ACT)5	15	32891	32905	potentially variable
PHG9_Scaffold_2345	20	p2	(AT)6	12	41714	41725	
PHG9_Scaffold_2345	23	p2	(CT)6	12	48736	48747	
PHG9_Scaffold_2345	32	p2	(AG)6	12	64769	64780	
PHG9_Scaffold_2345	33	p2	(TA)6	12	67968	67979	
PHG9_Scaffold_2345	38	p2	(TC)9	18	89924	89941	potentially variable
PHG9_Scaffold_2346	6	p2	(TA)7	14	10496	10509	potentially variable
PHG9_Scaffold_2346	8	p2	(AT)8	16	18103	18118	potentially variable
PHG9_Scaffold_2348	6	p2	(AT)6	12	13407	13418	
PHG9_Scaffold_2348	16	p2	(TA)7	14	59182	59195	potentially variable
PHG9_Scaffold_2350	3	p2	(AT)7	14	8218	8231	potentially variable
PHG9_Scaffold_2351	5	p2	(TA)6	12	3446	3457	
PHG9_Scaffold_2351	8	p2	(TA)7	14	13525	13538	potentially variable
PHG9_Scaffold_2354	4	p2	(TA)7	14	21231	21244	potentially

							variable
PHG9_Scaffold_2354	5	p2	(AT)6	12	22660	22671	
PHG9_Scaffold_2354	10	p2	(AT)7	14	37027	37040	potentially variable
PHG9_Scaffold_2354	17	p3	(CCA)6	18	52388	52405	potentially variable
PHG9_Scaffold_2354	21	p2	(AT)9	18	63539	63556	potentially variable
PHG9_Scaffold_2355	3	p2	(TG)7	14	3762	3775	potentially variable
PHG9_Scaffold_2355	4	p2	(TA)6	12	8065	8076	
PHG9_Scaffold_2357	2	p2	(TC)8	16	17344	17359	potentially variable
PHG9_Scaffold_2359	8	p2	(AT)7	14	27852	27865	potentially variable
PHG9_Scaffold_2359	9	p3	(AAT)5	15	40177	40191	potentially variable
PHG9_Scaffold_2359	12	p2	(AT)6	12	63817	63828	
PHG9_Scaffold_2359	13	p3	(ATT)5	15	63981	63995	potentially variable
PHG9_Scaffold_2359	17	p2	(TA)8	16	72606	72621	potentially variable
PHG9_Scaffold_2359	18	p2	(TA)6	12	78004	78015	
PHG9_Scaffold_2359	23	p3	(TTA)5	15	88694	88708	potentially variable
PHG9_Scaffold_2359	27	p2	(AC)8	16	102486	102501	potentially variable
PHG9_Scaffold_2359	32	p2	(TA)9	18	107477	107494	potentially variable
PHG9_Scaffold_2361	1	p2	(AT)9	18	135	152	potentially

							variable
PHG9_Scaffold_2361	3	p2	(AC)6	12	3003	3014	
PHG9_Scaffold_2361	10	p2	(TA)9	18	17647	17664	potentially variable
PHG9_Scaffold_2361	11	p3	(AAG)5	15	25771	25785	potentially variable
PHG9_Scaffold_2361	16	p2	(TA)7	14	37825	37838	potentially variable
PHG9_Scaffold_2361	18	p6	(CACTTA)7	42	40073	40114	Hypervariable
PHG9_Scaffold_2361	38	p3	(CGG)5	15	86791	86805	potentially variable
PHG9_Scaffold_2361	40	p2	(TC)6	12	87417	87428	
PHG9_Scaffold_2361	45	p2	(AT)8	16	97626	97641	potentially variable
PHG9_Scaffold_2361	52	p2	(TA)6	12	115014	115025	
PHG9_Scaffold_2361	63	p2	(GT)9	18	153125	153142	potentially variable
PHG9_Scaffold_2361	64	p2	(AT)8	16	155069	155084	potentially variable
PHG9_Scaffold_2361	80	p2	(AT)8	16	212969	212984	potentially variable
PHG9_Scaffold_2361	82	p3	(TTA)5	15	214357	214371	potentially variable
PHG9_Scaffold_2361	88	p2	(GA)9	18	230137	230154	potentially variable
PHG9_Scaffold_2361	95	p3	(GTT)6	18	237498	237515	potentially variable
PHG9_Scaffold_2361	109	p2	(CT)8	16	282548	282563	potentially variable
PHG9_Scaffold_2361	111	p2	(GA)7	14	292048	292061	potentially

							variable
PHG9_Scaffold_2361	112	p2	(AT)7	14	293772	293785	potentially variable
PHG9_Scaffold_2361	115	p2	(AT)8	16	301924	301939	potentially variable
PHG9_Scaffold_2362	2	p2	(TA)7	14	22068	22081	potentially variable
PHG9_Scaffold_2363	8	p3	(CAT)5	15	20588	20602	potentially variable
PHG9_Scaffold_2363	11	p2	(TA)7	14	27125	27138	potentially variable
PHG9_Scaffold_2363	19	p2	(AT)6	12	57414	57425	
PHG9_Scaffold_2363	25	p3	(TAA)5	15	76619	76633	potentially variable
PHG9_Scaffold_2363	26	p3	(ATT)5	15	78451	78465	potentially variable
PHG9_Scaffold_2363	27	p3	(ATA)5	15	79203	79217	potentially variable
PHG9_Scaffold_2363	29	p2	(CA)6	12	79745	79756	
PHG9_Scaffold_2363	30	p2	(TA)6	12	84526	84537	
PHG9_Scaffold_2363	32	p2	(TA)7	14	88964	88977	potentially variable
PHG9_Scaffold_2363	37	p2	(TC)6	12	98178	98189	
PHG9_Scaffold_2364	7	p2	(GA)7	14	31836	31849	potentially variable
PHG9_Scaffold_2364	8	p3	(ATT)5	15	38892	38906	potentially variable
PHG9_Scaffold_2364	12	p2	(AT)6	12	62496	62507	
PHG9_Scaffold_2364	14	p3	(TGA)5	15	68787	68801	potentially variable

PHG9_Scaffold_2365	8	p3	(GAA)5	15	64089	64103	potentially variable
PHG9_Scaffold_2365	13	p3	(TTA)5	15	79072	79086	potentially variable
PHG9_Scaffold_2365	18	p2	(AT)7	14	95690	95703	potentially variable
PHG9_Scaffold_2365	23	p2	(AT)8	16	116553	116568	potentially variable
PHG9_Scaffold_2365	27	p2	(AT)9	18	126269	126286	potentially variable
PHG9_Scaffold_2365	39	p3	(GTT)5	15	157850	157864	potentially variable
PHG9_Scaffold_2365	43	p2	(AT)8	16	169481	169496	potentially variable
PHG9_Scaffold_2365	44	p3	(TAT)5	15	171034	171048	potentially variable
PHG9_Scaffold_2365	46	p2	(TA)6	12	171276	171287	
PHG9_Scaffold_2365	47	p2	(AC)8	16	177940	177955	potentially variable
PHG9_Scaffold_2365	50	p2	(TA)6	12	180956	180967	
PHG9_Scaffold_2365	53	p3	(AGA)5	15	186224	186238	potentially variable
PHG9_Scaffold_2365	59	p2	(AT)9	18	200977	200994	potentially variable
PHG9_Scaffold_2366	25	p3	(AAT)5	15	53161	53175	potentially variable
PHG9_Scaffold_2366	26	p2	(CT)7	14	53594	53607	potentially variable
PHG9_Scaffold_2367	3	p3	(TTG)5	15	2267	2281	potentially variable

PHG9_Scaffold_2368	3	p3	(TTC)5	15	14644	14658	potentially variable
PHG9_Scaffold_2368	8	p3	(TTA)5	15	33992	34006	potentially variable
PHG9_Scaffold_2368	9	p2	(AT)6	12	38382	38393	
PHG9_Scaffold_2368	15	p2	(AT)7	14	53711	53724	potentially variable
PHG9_Scaffold_2372	5	p3	(AAT)5	15	15283	15297	potentially variable
PHG9_Scaffold_2375	11	p2	(TG)9	18	26574	26591	potentially variable
PHG9_Scaffold_2375	12	p2	(TA)6	12	28557	28568	
PHG9_Scaffold_2375	13	p3	(TCA)5	15	29400	29414	potentially variable
PHG9_Scaffold_2375	15	p3	(ATT)5	15	30742	30756	potentially variable
PHG9_Scaffold_2375	21	p3	(ATA)5	15	39157	39171	potentially variable
PHG9_Scaffold_2375	25	p3	(TTA)5	15	42637	42651	potentially variable
PHG9_Scaffold_2375	27	p2	(GT)7	14	46798	46811	potentially variable
PHG9_Scaffold_2375	28	p3	(AAT)6	18	49846	49863	potentially variable
PHG9_Scaffold_2375	35	p2	(AT)7	14	59646	59659	potentially variable
PHG9_Scaffold_2375	36	p3	(TTG)5	15	65586	65600	potentially variable
PHG9_Scaffold_2375	37	p3	(GGA)5	15	67416	67430	potentially variable

PHG9_Scaffold_2375	38	p3	(CAA)5	15	67779	67793	potentially variable
PHG9_Scaffold_2376	1	p3	(AGA)6	18	556	573	potentially variable
PHG9_Scaffold_2376	4	p2	(AT)7	14	6498	6511	potentially variable
PHG9_Scaffold_2377	2	p2	(AT)6	12	5632	5643	
PHG9_Scaffold_2377	6	p2	(TA)7	14	7476	7489	potentially variable
PHG9_Scaffold_2377	10	p3	(TTC)5	15	15681	15695	potentially variable
PHG9_Scaffold_2377	12	p3	(GGT)5	15	27401	27415	potentially variable
PHG9_Scaffold_2377	14	p3	(TAT)5	15	32366	32380	potentially variable
PHG9_Scaffold_2377	17	p3	(TCA)5	15	43763	43777	potentially variable
PHG9_Scaffold_2378	4	p2	(AT)7	14	24410	24423	potentially variable
PHG9_Scaffold_2378	6	p3	(AAT)5	15	27836	27850	potentially variable
PHG9_Scaffold_2386	1	p3	(CTT)5	15	3984	3998	potentially variable
PHG9_Scaffold_2387	12	p2	(TA)6	12	22488	22499	
PHG9_Scaffold_2387	13	p2	(CA)8	16	22782	22797	potentially variable
PHG9_Scaffold_2387	18	p2	(TC)6	12	29674	29685	
PHG9_Scaffold_2388	3	p2	(AT)6	12	69418	69429	
PHG9_Scaffold_2388	4	p2	(AT)7	14	72963	72976	potentially variable

PHG9_Scaffold_2390	3	p2	(AT)6	12	705	716	
PHG9_Scaffold_2390	4	p2	(AT)6	12	4655	4666	
PHG9_Scaffold_2390	5	p2	(AT)8	16	8276	8291	potentially variable
PHG9_Scaffold_2391	6	p2	(TA)6	12	8155	8166	
PHG9_Scaffold_2394	6	p2	(AG)6	12	19978	19989	
PHG9_Scaffold_2396	7	p3	(ATT)5	15	10349	10363	potentially variable
PHG9_Scaffold_2399	1	p2	(TA)7	14	3471	3484	potentially variable
PHG9_Scaffold_2399	4	p2	(AT)7	14	18892	18905	potentially variable
PHG9_Scaffold_2399	6	p2	(TA)6	12	20980	20991	
PHG9_Scaffold_2400	2	p3	(ATT)5	15	7711	7725	potentially variable
PHG9_Scaffold_2401	2	p2	(AT)6	12	4486	4497	
PHG9_Scaffold_2403	3	p3	(CCT)6	18	6617	6634	potentially variable
PHG9_Scaffold_2405	5	p2	(GA)7	14	14541	14554	potentially variable
PHG9_Scaffold_2405	8	p2	(TA)7	14	19018	19031	potentially variable
PHG9_Scaffold_2407	2	p3	(AGA)6	18	9083	9100	potentially variable
PHG9_Scaffold_2409	15	p2	(AT)6	12	34033	34044	
PHG9_Scaffold_2410	1	p2	(TA)7	14	3404	3417	potentially variable
PHG9_Scaffold_2412	7	p2	(AT)6	12	39343	39354	
PHG9_Scaffold_2412	11	p3	(TAC)5	15	47092	47106	potentially

							variable
PHG9_Scaffold_2412	21	p2	(AT)7	14	60413	60426	potentially variable
PHG9_Scaffold_2412	28	p2	(AT)6	12	70927	70938	
PHG9_Scaffold_2412	52	p2	(AC)7	14	165639	165652	potentially variable
PHG9_Scaffold_2414	3	p3	(TTC)5	15	6291	6305	potentially variable
PHG9_Scaffold_2415	5	p2	(TA)6	12	25220	25231	
PHG9_Scaffold_2415	15	p2	(AC)8	16	50733	50748	potentially variable
PHG9_Scaffold_2415	18	p2	(TA)9	18	57966	57983	potentially variable
PHG9_Scaffold_2415	19	p2	(TA)6	12	59185	59196	
PHG9_Scaffold_2415	32	p2	(AT)6	12	112394	112405	
PHG9_Scaffold_2416	4	p2	(AT)8	16	28753	28768	potentially variable
PHG9_Scaffold_2416	5	p2	(TA)6	12	29318	29329	
PHG9_Scaffold_2417	8	p2	(TA)6	12	27109	27120	
PHG9_Scaffold_2417	17	p3	(TGA)6	18	66604	66621	potentially variable
PHG9_Scaffold_2417	21	p3	(TTC)5	15	76868	76882	potentially variable
PHG9_Scaffold_2419	8	p2	(AT)6	12	16050	16061	
PHG9_Scaffold_2419	13	p2	(TA)6	12	29312	29323	
PHG9_Scaffold_2419	14	p3	(TGA)6	18	37745	37762	potentially variable
PHG9_Scaffold_2419	17	p2	(AT)8	16	45110	45125	potentially variable

PHG9_Scaffold_2421	2	p2	(AC)6	12	10730	10741	
PHG9_Scaffold_2421	4	p2	(TA)7	14	26748	26761	potentially variable
PHG9_Scaffold_2421	10	p2	(TA)7	14	59732	59745	potentially variable
PHG9_Scaffold_2425	2	p2	(AT)7	14	2831	2844	potentially variable
PHG9_Scaffold_2427	1	p2	(AT)6	12	470	481	
PHG9_Scaffold_2427	3	p2	(AT)9	18	4168	4185	potentially variable
PHG9_Scaffold_2427	6	p3	(TTA)5	15	28797	28811	potentially variable
PHG9_Scaffold_2427	9	p2	(TA)7	14	41630	41643	potentially variable
PHG9_Scaffold_2428	8	p2	(AG)6	12	15656	15667	
PHG9_Scaffold_2429	2	p2	(AT)8	16	3332	3347	potentially variable
PHG9_Scaffold_2435	2	p2	(AT)8	16	3026	3041	potentially variable
PHG9_Scaffold_2442	1	p3	(CTT)5	15	5693	5707	potentially variable
PHG9_Scaffold_2443	3	p2	(TA)7	14	9627	9640	potentially variable
PHG9_Scaffold_2443	10	p2	(TA)6	12	33232	33243	
PHG9_Scaffold_2443	11	p2	(AT)8	16	35065	35080	potentially variable
PHG9_Scaffold_2443	13	p2	(TA)6	12	42088	42099	
PHG9_Scaffold_2453	4	p2	(AT)6	12	6292	6303	
PHG9_Scaffold_2453	14	p2	(CT)7	14	37125	37138	potentially variable

PHG9_Scaffold_2453	25	p2	(AG)7	14	71713	71726	potentially variable
PHG9_Scaffold_2454	6	p2	(TA)6	12	8951	8962	
PHG9_Scaffold_2454	11	p2	(GA)7	14	27383	27396	potentially variable
PHG9_Scaffold_2455	1	p2	(TA)7	14	6705	6718	potentially variable
PHG9_Scaffold_2455	3	p2	(TA)6	12	20654	20665	
PHG9_Scaffold_2457	6	p2	(TA)6	12	32690	32701	
PHG9_Scaffold_2457	9	p2	(TA)7	14	43409	43422	potentially variable
PHG9_Scaffold_2459	9	p3	(TTC)6	18	35246	35263	potentially variable
PHG9_Scaffold_2461	1	p2	(AC)7	14	2559	2572	potentially variable
PHG9_Scaffold_2462	2	p2	(TA)8	16	983	998	potentially variable
PHG9_Scaffold_2462	9	p2	(AG)6	12	13893	13904	
PHG9_Scaffold_2462	11	p2	(TA)6	12	18914	18925	
PHG9_Scaffold_2462	12	p2	(TA)9	18	26772	26789	potentially variable
PHG9_Scaffold_2462	15	p3	(AAG)6	18	38287	38304	potentially variable
PHG9_Scaffold_2462	16	p2	(TA)7	14	40027	40040	potentially variable
PHG9_Scaffold_2465	1	p2	(TA)6	12	9236	9247	
PHG9_Scaffold_2465	5	p2	(AT)7	14	47790	47803	potentially variable
PHG9_Scaffold_2465	11	p2	(GA)6	12	94948	94959	

PHG9_Scaffold_2467	4	p2	(TA)6	12	5853	5864	
PHG9_Scaffold_2467	10	p2	(AT)6	12	8086	8097	
PHG9_Scaffold_2467	11	p6	(GCTTTG)6	36	9603	9638	Hypervariable
PHG9_Scaffold_2467	12	p2	(AT)6	12	14854	14865	
PHG9_Scaffold_2467	16	p2	(TA)7	14	25515	25528	potentially variable
PHG9_Scaffold_2467	17	p2	(AT)7	14	28000	28013	potentially variable
PHG9_Scaffold_2470	3	p2	(AT)6	12	2321	2332	
PHG9_Scaffold_2470	14	p2	(TC)6	12	35350	35361	
PHG9_Scaffold_2472	7	p2	(AT)6	12	8796	8807	
PHG9_Scaffold_2472	8	p2	(AG)8	16	12861	12876	potentially variable
PHG9_Scaffold_2472	17	p2	(AG)8	16	50684	50699	potentially variable
PHG9_Scaffold_2472	27	p2	(TA)8	16	87652	87667	potentially variable
PHG9_Scaffold_2472	28	p2	(AG)6	12	88740	88751	
PHG9_Scaffold_2472	29	p2	(AG)7	14	89038	89051	potentially variable
PHG9_Scaffold_2472	35	p3	(TAT)6	18	104544	104561	potentially variable
PHG9_Scaffold_2472	38	p2	(GA)7	14	115597	115610	potentially variable
PHG9_Scaffold_2472	40	p2	(CT)8	16	126529	126544	potentially variable
PHG9_Scaffold_2473	1	p2	(AT)6	12	12964	12975	
PHG9_Scaffold_2473	2	p3	(ATA)5	15	17746	17760	potentially variable

PHG9_Scaffold_2473	3	p2	(TC)8	16	18599	18614	potentially variable
PHG9_Scaffold_2474	3	p2	(TA)7	14	2714	2727	potentially variable
PHG9_Scaffold_2474	5	p2	(AC)6	12	14214	14225	
PHG9_Scaffold_2476	5	p2	(TC)9	18	12760	12777	potentially variable
PHG9_Scaffold_2476	12	p2	(TA)6	12	31844	31855	
PHG9_Scaffold_2476	16	p2	(TA)8	16	36616	36631	potentially variable
PHG9_Scaffold_2476	17	p2	(TG)7	14	37190	37203	potentially variable
PHG9_Scaffold_2476	22	p3	(ATT)5	15	47307	47321	potentially variable
PHG9_Scaffold_2476	27	p2	(TA)6	12	62727	62738	
PHG9_Scaffold_2476	32	p2	(TA)7	14	76736	76749	potentially variable
PHG9_Scaffold_2476	33	p2	(TA)8	16	79509	79524	potentially variable
PHG9_Scaffold_2476	40	p3	(CAA)5	15	95869	95883	potentially variable
PHG9_Scaffold_2477	4	p3	(TTC)5	15	1252	1266	potentially variable
PHG9_Scaffold_2480	4	p2	(AG)6	12	34251	34262	
PHG9_Scaffold_2480	6	p2	(AT)7	14	46376	46389	potentially variable
PHG9_Scaffold_2480	12	p2	(AT)7	14	99693	99706	potentially variable
PHG9_Scaffold_2480	19	p2	(AT)8	16	133456	133471	potentially variable

PHG9_Scaffold_2480	20	p2	(TA)6	12	135292	135303	
PHG9_Scaffold_2480	30	p2	(AT)9	18	160242	160259	potentially variable
PHG9_Scaffold_2481	5	p2	(TA)7	14	28715	28728	potentially variable
PHG9_Scaffold_2481	10	p2	(CT)6	12	42240	42251	
PHG9_Scaffold_2481	12	p2	(TA)7	14	43857	43870	potentially variable
PHG9_Scaffold_2481	14	p3	(AAT)6	18	50044	50061	potentially variable
PHG9_Scaffold_2481	18	p3	(ATC)5	15	60462	60476	potentially variable
PHG9_Scaffold_2482	4	p2	(AT)7	14	6465	6478	potentially variable
PHG9_Scaffold_2483	2	p2	(AG)7	14	7133	7146	potentially variable
PHG9_Scaffold_2484	1	p3	(TAA)5	15	6427	6441	potentially variable
PHG9_Scaffold_2485	2	p3	(GAG)5	15	8991	9005	potentially variable
PHG9_Scaffold_2485	5	p3	(TTC)6	18	22559	22576	potentially variable
PHG9_Scaffold_2485	9	p2	(TA)7	14	49239	49252	potentially variable
PHG9_Scaffold_2485	10	p2	(TA)6	12	50511	50522	
PHG9_Scaffold_2486	4	p2	(TA)6	12	12124	12135	
PHG9_Scaffold_2486	9	p2	(TG)7	14	16450	16463	potentially variable
PHG9_Scaffold_2486	10	p3	(TTA)5	15	17621	17635	potentially variable

PHG9_Scaffold_2486	18	p2	(AT)6	12	51121	51132	
PHG9_Scaffold_2486	25	p2	(TA)8	16	75626	75641	potentially variable
PHG9_Scaffold_2486	32	p3	(AAT)6	18	89915	89932	potentially variable
PHG9_Scaffold_2487	4	p2	(AT)8	16	7466	7481	potentially variable
PHG9_Scaffold_2488	4	p2	(AT)6	12	26197	26208	
PHG9_Scaffold_2489	5	p3	(AAG)5	15	45153	45167	potentially variable
PHG9_Scaffold_2489	6	p3	(AGA)5	15	46104	46118	potentially variable
PHG9_Scaffold_2489	9	p2	(TA)8	16	84113	84128	potentially variable
PHG9_Scaffold_2490	4	p2	(CT)6	12	5158	5169	
PHG9_Scaffold_2490	15	p2	(AG)6	12	24483	24494	
PHG9_Scaffold_2490	16	p2	(AT)6	12	26242	26253	
PHG9_Scaffold_2490	19	p2	(AT)7	14	35133	35146	potentially variable
PHG9_Scaffold_2490	20	p2	(TA)6	12	40813	40824	
PHG9_Scaffold_2490	21	p2	(AC)6	12	40941	40952	
PHG9_Scaffold_2490	22	p2	(AC)7	14	41343	41356	potentially variable
PHG9_Scaffold_2490	23	p2	(AT)7	14	42380	42393	potentially variable
PHG9_Scaffold_2490	25	p3	(TAT)5	15	44175	44189	potentially variable
PHG9_Scaffold_2490	29	p3	(ATT)5	15	49170	49184	potentially variable

PHG9_Scaffold_2490	36	p2	(GT)9	18	66516	66533	potentially variable
PHG9_Scaffold_2490	38	p3	(ATA)5	15	71350	71364	potentially variable
PHG9_Scaffold_2490	55	p6	(GCCTCA)5	30	106676	106705	Hypervariable
PHG9_Scaffold_2492	7	p2	(AT)7	14	127247	127260	potentially variable
PHG9_Scaffold_2492	10	p2	(CT)8	16	154851	154866	potentially variable
PHG9_Scaffold_2493	1	p2	(AG)7	14	1113	1126	potentially variable
PHG9_Scaffold_2494	2	p3	(AAT)6	18	603	620	potentially variable
PHG9_Scaffold_2494	13	p2	(TC)6	12	15260	15271	
PHG9_Scaffold_2494	16	p2	(AT)8	16	19459	19474	potentially variable
PHG9_Scaffold_2497	3	p2	(AT)7	14	4238	4251	potentially variable
PHG9_Scaffold_2497	7	p2	(TC)6	12	14375	14386	
PHG9_Scaffold_2497	10	p6	(TTTCAC)5	30	18799	18828	Hypervariable
PHG9_Scaffold_2497	11	p2	(AT)6	12	19348	19359	
PHG9_Scaffold_2497	12	p2	(AT)6	12	20307	20318	
PHG9_Scaffold_2498	1	p6	(TTTCAC)5	30	1732	1761	Hypervariable
PHG9_Scaffold_2498	2	p2	(AT)6	12	2281	2292	
PHG9_Scaffold_2498	3	p2	(AT)6	12	3240	3251	
PHG9_Scaffold_2499	4	p2	(AG)7	14	6875	6888	potentially variable
PHG9_Scaffold_2499	6	p2	(AT)7	14	19541	19554	potentially variable

PHG9_Scaffold_2499	7	p2	(AT)8	16	23388	23403	potentially variable
PHG9_Scaffold_2500	2	p2	(AT)6	12	4650	4661	
PHG9_Scaffold_2500	6	p2	(TG)6	12	20645	20656	
PHG9_Scaffold_2500	7	p3	(AGA)5	15	24040	24054	potentially variable
PHG9_Scaffold_2500	10	p3	(TTA)5	15	34555	34569	potentially variable
PHG9_Scaffold_2501	1	p3	(TTA)5	15	989	1003	potentially variable
PHG9_Scaffold_2501	3	p2	(AT)9	18	9083	9100	potentially variable
PHG9_Scaffold_2502	10	p2	(AG)8	16	27072	27087	potentially variable
PHG9_Scaffold_2502	11	p3	(TAT)5	15	32144	32158	potentially variable
PHG9_Scaffold_2502	14	p3	(AGA)6	18	34583	34600	potentially variable
PHG9_Scaffold_2502	20	p2	(GA)7	14	49136	49149	potentially variable
PHG9_Scaffold_2502	21	p2	(TG)6	12	49687	49698	
PHG9_Scaffold_2502	22	p2	(TA)9	18	52353	52370	potentially variable
PHG9_Scaffold_2502	25	p2	(AT)6	12	58090	58101	
PHG9_Scaffold_2502	26	p2	(AT)8	16	65582	65597	potentially variable
PHG9_Scaffold_2503	7	p2	(AG)9	18	72206	72223	potentially variable
PHG9_Scaffold_2503	8	p3	(AAT)5	15	72855	72869	potentially variable

PHG9_Scaffold_2504	2	p2	(AG)9	18	14517	14534	potentially variable
PHG9_Scaffold_2507	2	p2	(AT)7	14	12697	12710	potentially variable
PHG9_Scaffold_2507	7	p2	(TA)9	18	34611	34628	potentially variable
PHG9_Scaffold_2507	16	p2	(TA)6	12	78890	78901	
PHG9_Scaffold_2509	3	p3	(TAA)5	15	5780	5794	potentially variable
PHG9_Scaffold_2510	1	p2	(TA)7	14	2973	2986	potentially variable
PHG9_Scaffold_2510	3	p2	(AT)9	18	9796	9813	potentially variable
PHG9_Scaffold_2510	4	p3	(CCT)6	18	14993	15010	potentially variable
PHG9_Scaffold_2513	2	p2	(AT)6	12	5738	5749	
PHG9_Scaffold_2514	1	p2	(TA)7	14	13342	13355	potentially variable
PHG9_Scaffold_2516	2	p3	(CAT)5	15	1584	1598	potentially variable
PHG9_Scaffold_2517	2	p2	(AT)7	14	13758	13771	potentially variable
PHG9_Scaffold_2517	3	p2	(AC)6	12	14093	14104	
PHG9_Scaffold_2520	2	p2	(TA)6	12	6373	6384	
PHG9_Scaffold_2520	9	p2	(AT)6	12	38571	38582	
PHG9_Scaffold_2520	17	p3	(ATA)6	18	62005	62022	potentially variable
PHG9_Scaffold_2520	25	p2	(AT)6	12	73529	73540	
PHG9_Scaffold_2520	30	p2	(AT)7	14	90765	90778	potentially variable

PHG9_Scaffold_2522	12	p2	(TA)6	12	76362	76373	
PHG9_Scaffold_2522	14	p2	(AT)6	12	76970	76981	
PHG9_Scaffold_2522	18	p2	(TC)6	12	95948	95959	
PHG9_Scaffold_2528	3	p3	(TTA)5	15	2582	2596	potentially variable
PHG9_Scaffold_2529	11	p2	(AT)6	12	14634	14645	
PHG9_Scaffold_2529	12	p2	(CA)7	14	17431	17444	potentially variable
PHG9_Scaffold_2529	23	p2	(AT)7	14	44484	44497	potentially variable
PHG9_Scaffold_2533	17	p3	(TAT)5	15	84631	84645	potentially variable
PHG9_Scaffold_2533	22	p2	(TA)7	14	100765	100778	potentially variable
PHG9_Scaffold_2535	4	p2	(AT)7	14	24351	24364	potentially variable
PHG9_Scaffold_2535	15	p2	(TA)8	16	45648	45663	potentially variable
PHG9_Scaffold_2535	22	p2	(TA)7	14	78562	78575	potentially variable
PHG9_Scaffold_2535	24	p2	(AT)6	12	84096	84107	
PHG9_Scaffold_2535	25	p2	(AT)6	12	89069	89080	
PHG9_Scaffold_2535	26	p2	(AT)6	12	89997	90008	
PHG9_Scaffold_2535	27	p2	(AT)6	12	90779	90790	
PHG9_Scaffold_2535	44	p2	(TA)7	14	141734	141747	potentially variable
PHG9_Scaffold_2535	45	p2	(TA)8	16	149125	149140	potentially variable
PHG9_Scaffold_2535	49	p2	(AT)6	12	158994	159005	

PHG9_Scaffold_2535	52	p2	(TA)8	16	167946	167961	potentially variable
PHG9_Scaffold_2536	3	p2	(AT)6	12	7910	7921	
PHG9_Scaffold_2537	1	p3	(AAT)6	18	52	69	potentially variable
PHG9_Scaffold_2538	4	p2	(TA)6	12	10424	10435	
PHG9_Scaffold_2539	6	p2	(AT)6	12	24480	24491	
PHG9_Scaffold_2541	3	p2	(AT)6	12	3138	3149	
PHG9_Scaffold_2541	8	p2	(AT)7	14	15537	15550	potentially variable
PHG9_Scaffold_2544	1	p2	(AT)9	18	117	134	potentially variable
PHG9_Scaffold_2544	5	p6	(ATAAAG)5	30	13045	13074	Hypervariable
PHG9_Scaffold_2544	11	p3	(TGT)6	18	32642	32659	potentially variable
PHG9_Scaffold_2544	16	p2	(AT)7	14	41539	41552	potentially variable
PHG9_Scaffold_2544	17	p2	(AT)7	14	52063	52076	potentially variable
PHG9_Scaffold_2544	18	p3	(TTC)5	15	66909	66923	potentially variable
PHG9_Scaffold_2544	23	p3	(TAA)5	15	74032	74046	potentially variable
PHG9_Scaffold_2544	24	p2	(GT)7	14	74708	74721	potentially variable
PHG9_Scaffold_2544	28	p3	(TTC)5	15	93441	93455	potentially variable
PHG9_Scaffold_2544	34	p3	(TCT)5	15	121713	121727	potentially variable
PHG9_Scaffold_2544	35	p2	(AT)6	12	129328	129339	

PHG9_Scaffold_2544	39	p2	(AT)8	16	141515	141530	potentially variable
PHG9_Scaffold_2545	10	p3	(TTC)5	15	39630	39644	potentially variable
PHG9_Scaffold_2545	14	p3	(AAT)5	15	60898	60912	potentially variable
PHG9_Scaffold_2545	25	p3	(AAT)5	15	108063	108077	potentially variable
PHG9_Scaffold_2545	26	p2	(AT)6	12	109343	109354	
PHG9_Scaffold_2546	3	p2	(AG)9	18	8834	8851	potentially variable
PHG9_Scaffold_2547	3	p2	(AT)6	12	12393	12404	
PHG9_Scaffold_2547	7	p2	(TA)8	16	48160	48175	potentially variable
PHG9_Scaffold_2547	9	p3	(TAT)5	15	77287	77301	potentially variable
PHG9_Scaffold_2548	13	p2	(TA)9	18	26775	26792	potentially variable
PHG9_Scaffold_2548	14	p3	(TAA)5	15	26923	26937	potentially variable
PHG9_Scaffold_2549	7	p2	(AT)7	14	16989	17002	potentially variable
PHG9_Scaffold_2550	10	p2	(TA)6	12	15105	15116	
PHG9_Scaffold_2550	13	p2	(AC)6	12	16805	16816	
PHG9_Scaffold_2550	28	p3	(TAA)5	15	48605	48619	potentially variable
PHG9_Scaffold_2550	42	p2	(CT)6	12	71214	71225	
PHG9_Scaffold_2550	49	p6	(ATGCAG)7	42	81619	81660	Hypervariable
PHG9_Scaffold_2550	59	p3	(ATG)6	18	101228	101245	potentially variable

PHG9_Scaffold_2550	64	p2	(AT)7	14	110331	110344	potentially variable
PHG9_Scaffold_2550	66	p2	(AT)6	12	114001	114012	
PHG9_Scaffold_2550	68	p2	(TA)7	14	118836	118849	potentially variable
PHG9_Scaffold_2550	88	p2	(TA)6	12	185924	185935	
PHG9_Scaffold_2551	3	p2	(TA)7	14	2332	2345	potentially variable
PHG9_Scaffold_2551	6	p2	(AT)7	14	22359	22372	potentially variable
PHG9_Scaffold_2551	11	p2	(TC)9	18	28477	28494	potentially variable
PHG9_Scaffold_2551	15	p2	(AT)6	12	41216	41227	
PHG9_Scaffold_2551	18	p2	(AT)6	12	52385	52396	
PHG9_Scaffold_2551	19	p2	(TA)6	12	53937	53948	
PHG9_Scaffold_2551	24	p2	(AT)9	18	67826	67843	potentially variable
PHG9_Scaffold_2551	29	p2	(AT)8	16	75282	75297	potentially variable
PHG9_Scaffold_2551	30	p2	(AT)6	12	77308	77319	
PHG9_Scaffold_2551	31	p2	(GT)6	12	81477	81488	
PHG9_Scaffold_2552	2	p2	(AT)8	16	806	821	potentially variable
PHG9_Scaffold_2552	5	p2	(AT)7	14	6380	6393	potentially variable
PHG9_Scaffold_2552	10	p2	(TA)6	12	17505	17516	
PHG9_Scaffold_2552	14	p2	(TA)6	12	27031	27042	
PHG9_Scaffold_2552	15	p2	(AT)6	12	27943	27954	
PHG9_Scaffold_2552	20	p3	(TAA)5	15	34209	34223	potentially

							variable
PHG9_Scaffold_2552	33	p2	(AT)8	16	80269	80284	potentially variable
PHG9_Scaffold_2552	55	p2	(CT)8	16	135241	135256	potentially variable
PHG9_Scaffold_2552	58	p2	(CT)6	12	141255	141266	
PHG9_Scaffold_2552	73	p3	(CTT)5	15	171252	171266	potentially variable
PHG9_Scaffold_2552	78	p2	(AT)8	16	181705	181720	potentially variable
PHG9_Scaffold_2553	12	p2	(AG)6	12	36543	36554	
PHG9_Scaffold_2554	9	p2	(TA)6	12	38125	38136	
PHG9_Scaffold_2554	10	p2	(TA)6	12	39106	39117	
PHG9_Scaffold_2555	1	p2	(AT)8	16	4558	4573	potentially variable
PHG9_Scaffold_2556	2	p2	(AC)6	12	11071	11082	
PHG9_Scaffold_2557	7	p2	(AT)6	12	66196	66207	
PHG9_Scaffold_2557	8	p2	(AT)6	12	73558	73569	
PHG9_Scaffold_2558	2	p2	(CT)6	12	1997	2008	
PHG9_Scaffold_2559	2	p3	(TCA)5	15	1737	1751	potentially variable
PHG9_Scaffold_2560	1	p3	(TTA)5	15	433	447	potentially variable
PHG9_Scaffold_2560	2	p3	(CCA)5	15	1854	1868	potentially variable
PHG9_Scaffold_2560	10	p2	(CA)8	16	25675	25690	potentially variable
PHG9_Scaffold_2562	1	p3	(CGT)5	15	4639	4653	potentially variable

PHG9_Scaffold_2563	4	p3	(GAT)5	15	4735	4749	potentially variable
PHG9_Scaffold_2563	8	p2	(AT)8	16	13523	13538	potentially variable
PHG9_Scaffold_2564	2	p2	(TA)7	14	13635	13648	potentially variable
PHG9_Scaffold_2569	1	p2	(TA)8	16	3441	3456	potentially variable
PHG9_Scaffold_2570	1	p2	(AT)9	18	1065	1082	potentially variable
PHG9_Scaffold_2576	9	p3	(TTC)5	15	32418	32432	potentially variable
PHG9_Scaffold_2577	1	p2	(AT)8	16	220	235	potentially variable
PHG9_Scaffold_2577	6	p2	(TA)8	16	17774	17789	potentially variable
PHG9_Scaffold_2577	13	p2	(TA)6	12	42547	42558	
PHG9_Scaffold_2577	14	p2	(TC)7	14	42708	42721	potentially variable
PHG9_Scaffold_2577	20	p2	(TA)8	16	57291	57306	potentially variable
PHG9_Scaffold_2577	22	p2	(AT)7	14	63761	63774	potentially variable
PHG9_Scaffold_2577	32	p2	(TC)6	12	102761	102772	
PHG9_Scaffold_2577	34	p2	(AT)7	14	106154	106167	potentially variable
PHG9_Scaffold_2577	36	p2	(TA)6	12	107828	107839	
PHG9_Scaffold_2579	8	p2	(AT)7	14	23185	23198	potentially variable
PHG9_Scaffold_2579	22	p2	(TA)7	14	68916	68929	potentially

							variable
PHG9_Scaffold_2582	5	p2	(TG)6	12	10745	10756	
PHG9_Scaffold_2582	6	p2	(TA)6	12	11183	11194	
PHG9_Scaffold_2582	7	p2	(TA)6	12	13159	13170	
PHG9_Scaffold_2582	8	p2	(AT)8	16	23596	23611	potentially variable
PHG9_Scaffold_2582	14	p2	(TC)7	14	39310	39323	potentially variable
PHG9_Scaffold_2582	15	p2	(AT)6	12	42818	42829	
PHG9_Scaffold_2583	3	p2	(AT)6	12	9062	9073	
PHG9_Scaffold_2584	2	p2	(TA)7	14	3825	3838	potentially variable
PHG9_Scaffold_2584	4	p2	(AG)7	14	13081	13094	potentially variable
PHG9_Scaffold_2585	3	p2	(AT)6	12	4203	4214	
PHG9_Scaffold_2585	6	p2	(TC)6	12	13104	13115	
PHG9_Scaffold_2585	14	p2	(TG)9	18	37701	37718	potentially variable
PHG9_Scaffold_2585	21	p2	(TC)8	16	60577	60592	potentially variable
PHG9_Scaffold_2585	23	p3	(ATT)5	15	65496	65510	potentially variable
PHG9_Scaffold_2585	26	p2	(AT)7	14	68096	68109	potentially variable
PHG9_Scaffold_2585	28	p2	(TA)6	12	73631	73642	
PHG9_Scaffold_2585	32	p2	(AT)6	12	83110	83121	
PHG9_Scaffold_2585	35	p2	(AT)6	12	89732	89743	
PHG9_Scaffold_2585	41	p3	(ATA)5	15	113363	113377	potentially variable

PHG9_Scaffold_2586	2	p2	(TC)7	14	28387	28400	potentially variable
PHG9_Scaffold_2586	5	p2	(TA)6	12	74258	74269	
PHG9_Scaffold_2586	12	p2	(TC)9	18	95796	95813	potentially variable
PHG9_Scaffold_2587	4	p2	(AT)8	16	9147	9162	potentially variable
PHG9_Scaffold_2587	5	p2	(TC)9	18	20874	20891	potentially variable
PHG9_Scaffold_2587	6	p2	(CT)7	14	21236	21249	potentially variable
PHG9_Scaffold_2589	6	p2	(AT)8	16	162014	162029	potentially variable
PHG9_Scaffold_2589	13	p2	(TA)6	12	212707	212718	
PHG9_Scaffold_2589	21	p2	(AT)9	18	240966	240983	potentially variable
PHG9_Scaffold_2589	22	p2	(AT)6	12	243619	243630	
PHG9_Scaffold_2589	29	p2	(TA)6	12	274357	274368	
PHG9_Scaffold_2590	15	p2	(TA)6	12	117110	117121	
PHG9_Scaffold_2591	1	p3	(ATT)6	18	5254	5271	potentially variable
PHG9_Scaffold_2593	2	p2	(TA)9	18	16108	16125	potentially variable
PHG9_Scaffold_2593	6	p3	(TTG)5	15	19605	19619	potentially variable
PHG9_Scaffold_2593	10	p3	(GAT)6	18	27819	27836	potentially variable
PHG9_Scaffold_2593	11	p2	(CT)9	18	34476	34493	potentially variable
PHG9_Scaffold_2595	2	p2	(GA)6	12	3362	3373	

PHG9_Scaffold_2595	3	p3	(TCC)6	18	3648	3665	potentially variable
PHG9_Scaffold_2596	3	p2	(TA)7	14	1101	1114	potentially variable
PHG9_Scaffold_2597	2	p2	(AT)8	16	12423	12438	potentially variable
PHG9_Scaffold_2597	3	p2	(TC)6	12	23459	23470	
PHG9_Scaffold_2597	9	p2	(CT)6	12	59492	59503	
PHG9_Scaffold_2603	4	p3	(TTA)6	18	10327	10344	potentially variable
PHG9_Scaffold_2604	1	p2	(TA)6	12	502	513	
PHG9_Scaffold_2604	4	p2	(AC)6	12	6110	6121	
PHG9_Scaffold_2607	3	p3	(AGA)5	15	4675	4689	potentially variable
PHG9_Scaffold_2607	7	p2	(AT)6	12	19422	19433	
PHG9_Scaffold_2607	12	p2	(AT)7	14	23271	23284	potentially variable
PHG9_Scaffold_2607	18	p3	(TGA)5	15	51196	51210	potentially variable
PHG9_Scaffold_2607	20	p2	(AT)6	12	53376	53387	
PHG9_Scaffold_2607	21	p3	(ATT)5	15	56962	56976	potentially variable
PHG9_Scaffold_2607	22	p2	(TC)6	12	63641	63652	
PHG9_Scaffold_2607	26	p2	(TA)7	14	84460	84473	potentially variable
PHG9_Scaffold_2609	1	p3	(ATA)5	15	10746	10760	potentially variable
PHG9_Scaffold_2609	11	p2	(AT)6	12	51788	51799	
PHG9_Scaffold_2611	1	p2	(TA)6	12	4762	4773	

PHG9_Scaffold_2611	2	p2	(AC)6	12	8648	8659	
PHG9_Scaffold_2611	14	p2	(AT)6	12	27243	27254	
PHG9_Scaffold_2611	23	p2	(TA)7	14	69326	69339	potentially variable
PHG9_Scaffold_2611	27	p3	(TGA)5	15	93893	93907	potentially variable
PHG9_Scaffold_2611	28	p2	(CT)7	14	94685	94698	potentially variable
PHG9_Scaffold_2612	3	p2	(TA)8	16	27579	27594	potentially variable
PHG9_Scaffold_2613	5	p2	(AT)6	12	9806	9817	
PHG9_Scaffold_2613	11	p2	(TA)8	16	29173	29188	potentially variable
PHG9_Scaffold_2613	15	p2	(TA)6	12	43438	43449	
PHG9_Scaffold_2614	1	p2	(AT)6	12	4970	4981	
PHG9_Scaffold_2614	3	p2	(AT)7	14	13866	13879	potentially variable
PHG9_Scaffold_2614	6	p2	(GA)6	12	31187	31198	
PHG9_Scaffold_2614	8	p2	(AT)6	12	65934	65945	
PHG9_Scaffold_2614	9	p2	(TA)6	12	66741	66752	
PHG9_Scaffold_2614	10	p2	(AT)7	14	78942	78955	potentially variable
PHG9_Scaffold_2616	1	p2	(GT)7	14	1936	1949	potentially variable
PHG9_Scaffold_2616	2	p3	(TTA)5	15	5586	5600	potentially variable
PHG9_Scaffold_2616	3	p3	(CTT)5	15	6491	6505	potentially variable
PHG9_Scaffold_2619	3	p2	(AC)7	14	1778	1791	potentially

							variable
PHG9_Scaffold_2619	7	p2	(TA)8	16	17293	17308	potentially variable
PHG9_Scaffold_2621	2	p3	(GAA)6	18	8112	8129	potentially variable
PHG9_Scaffold_2621	5	p2	(AG)9	18	39739	39756	potentially variable
PHG9_Scaffold_2624	3	p2	(TA)6	12	11554	11565	
PHG9_Scaffold_2626	1	p2	(AG)6	12	5309	5320	
PHG9_Scaffold_2626	2	p2	(AT)9	18	5853	5870	potentially variable
PHG9_Scaffold_2626	8	p3	(ATA)5	15	18941	18955	potentially variable
PHG9_Scaffold_2626	9	p2	(AG)6	12	20801	20812	
PHG9_Scaffold_2630	2	p2	(AT)6	12	1350	1361	
PHG9_Scaffold_2631	1	p2	(TC)8	16	445	460	potentially variable
PHG9_Scaffold_2632	5	p2	(AT)6	12	26090	26101	
PHG9_Scaffold_2632	9	p2	(TA)6	12	57501	57512	
PHG9_Scaffold_2632	10	p2	(TA)6	12	58212	58223	
PHG9_Scaffold_2633	1	p3	(CTT)5	15	2217	2231	potentially variable
PHG9_Scaffold_2634	2	p2	(AT)6	12	1893	1904	
PHG9_Scaffold_2634	10	p2	(AT)6	12	24444	24455	
PHG9_Scaffold_2634	17	p3	(CTG)6	18	46343	46360	potentially variable
PHG9_Scaffold_2635	4	p2	(AT)6	12	9150	9161	
PHG9_Scaffold_2635	10	p2	(AT)8	16	18756	18771	potentially variable

PHG9_Scaffold_2635	12	p2	(AT)9	18	25701	25718	potentially variable
PHG9_Scaffold_2635	16	p2	(TG)8	16	31718	31733	potentially variable
PHG9_Scaffold_2635	22	p2	(TC)6	12	44758	44769	
PHG9_Scaffold_2635	26	p3	(CTT)6	18	65772	65789	potentially variable
PHG9_Scaffold_2635	27	p3	(GCT)5	15	71506	71520	potentially variable
PHG9_Scaffold_2635	28	p3	(GAT)5	15	73007	73021	potentially variable
PHG9_Scaffold_2635	29	p3	(TTG)5	15	74367	74381	potentially variable
PHG9_Scaffold_2635	37	p2	(AG)6	12	83485	83496	
PHG9_Scaffold_2635	41	p2	(TA)6	12	95836	95847	
PHG9_Scaffold_2635	45	p2	(TA)6	12	104936	104947	
PHG9_Scaffold_2636	1	p2	(TA)6	12	8222	8233	
PHG9_Scaffold_2638	3	p2	(AT)9	18	12034	12051	potentially variable
PHG9_Scaffold_2638	7	p2	(AG)6	12	19584	19595	
PHG9_Scaffold_2638	9	p2	(TA)6	12	30491	30502	
PHG9_Scaffold_2640	3	p2	(TC)8	16	3279	3294	potentially variable
PHG9_Scaffold_2642	4	p2	(GA)6	12	12957	12968	
PHG9_Scaffold_2642	5	p2	(AC)6	12	15726	15737	
PHG9_Scaffold_2642	6	p2	(TA)6	12	21713	21724	
PHG9_Scaffold_2643	2	p2	(CT)6	12	9876	9887	
PHG9_Scaffold_2644	10	p2	(AT)7	14	46437	46450	potentially variable

PHG9_Scaffold_2647	4	p2	(TA)9	18	5933	5950	potentially variable
PHG9_Scaffold_2648	1	p6	(TAATAT)5	30	4587	4616	Hypervariable
PHG9_Scaffold_2648	5	p3	(AAT)5	15	16808	16822	potentially variable
PHG9_Scaffold_2648	8	p2	(TA)7	14	54540	54553	potentially variable
PHG9_Scaffold_2650	5	p2	(AG)6	12	12199	12210	
PHG9_Scaffold_2650	7	p2	(TA)6	12	15199	15210	
PHG9_Scaffold_2650	12	p2	(TA)6	12	41449	41460	
PHG9_Scaffold_2650	13	p2	(TA)7	14	41584	41597	potentially variable
PHG9_Scaffold_2650	18	p3	(TGG)5	15	55876	55890	potentially variable
PHG9_Scaffold_2650	19	p2	(AG)7	14	56318	56331	potentially variable
PHG9_Scaffold_2650	26	p2	(TA)6	12	67366	67377	
PHG9_Scaffold_2650	32	p2	(AT)6	12	94943	94954	
PHG9_Scaffold_2650	33	p2	(TA)8	16	95990	96005	potentially variable
PHG9_Scaffold_2650	35	p3	(ATT)5	15	98879	98893	potentially variable
PHG9_Scaffold_2650	40	p2	(AG)9	18	110918	110935	potentially variable
PHG9_Scaffold_2650	42	p2	(AT)6	12	113727	113738	
PHG9_Scaffold_2652	8	p2	(TA)6	12	7325	7336	
PHG9_Scaffold_2652	12	p2	(TA)6	12	15584	15595	
PHG9_Scaffold_2655	5	p2	(TA)6	12	5744	5755	
PHG9_Scaffold_2655	10	p2	(CA)7	14	37321	37334	potentially

							variable
PHG9_Scaffold_2657	5	p2	(AG)6	12	3971	3982	
PHG9_Scaffold_2657	7	p2	(AT)6	12	8995	9006	
PHG9_Scaffold_2657	23	p2	(AG)6	12	74192	74203	
PHG9_Scaffold_2657	28	p2	(AG)6	12	86412	86423	
PHG9_Scaffold_2659	14	p2	(TA)9	18	18172	18189	potentially variable
PHG9_Scaffold_2659	33	p2	(TA)6	12	92923	92934	
PHG9_Scaffold_2661	6	p2	(CT)6	12	35462	35473	
PHG9_Scaffold_2662	3	p2	(TA)6	12	19004	19015	
PHG9_Scaffold_2666	5	p3	(AAT)5	15	23707	23721	potentially variable
PHG9_Scaffold_2668	6	p2	(AT)6	12	11741	11752	
PHG9_Scaffold_2668	11	p2	(AC)6	12	31197	31208	
PHG9_Scaffold_2668	17	p2	(AT)8	16	55150	55165	potentially variable
PHG9_Scaffold_2668	19	p2	(TA)6	12	62173	62184	
PHG9_Scaffold_2668	29	p3	(TTC)6	18	97836	97853	potentially variable
PHG9_Scaffold_2669	3	p3	(TTA)5	15	2283	2297	potentially variable
PHG9_Scaffold_2671	2	p2	(AT)7	14	2775	2788	potentially variable
PHG9_Scaffold_2673	6	p2	(TA)8	16	5897	5912	potentially variable
PHG9_Scaffold_2673	10	p2	(TA)6	12	13213	13224	
PHG9_Scaffold_2675	3	p3	(ATT)5	15	6799	6813	potentially variable
PHG9_Scaffold_2675	6	p2	(AT)6	12	13912	13923	

PHG9_Scaffold_2676	6	p2	(AT)6	12	17970	17981	
PHG9_Scaffold_2676	12	p3	(TTA)5	15	30380	30394	potentially variable
PHG9_Scaffold_2678	5	p2	(TA)6	12	38330	38341	
PHG9_Scaffold_2678	15	p2	(AG)6	12	85588	85599	
PHG9_Scaffold_2678	16	p2	(CT)7	14	86792	86805	potentially variable
PHG9_Scaffold_2682	6	p2	(TA)6	12	5699	5710	
PHG9_Scaffold_2682	8	p2	(TA)6	12	7547	7558	
PHG9_Scaffold_2682	9	p2	(AT)7	14	8831	8844	potentially variable
PHG9_Scaffold_2682	11	p2	(AT)6	12	13015	13026	
PHG9_Scaffold_2682	12	p2	(TA)8	16	24156	24171	potentially variable
PHG9_Scaffold_2683	22	p2	(GA)6	12	57585	57596	
PHG9_Scaffold_2688	1	p2	(TA)6	12	199	210	
PHG9_Scaffold_2690	2	p3	(AAT)5	15	3363	3377	potentially variable
PHG9_Scaffold_2690	7	p6	(AAATCC)6	36	15568	15603	Hypervariable
PHG9_Scaffold_2690	20	p2	(TA)7	14	41809	41822	potentially variable
PHG9_Scaffold_2690	33	p2	(CT)6	12	70553	70564	
PHG9_Scaffold_2690	37	p2	(TA)6	12	83314	83325	
PHG9_Scaffold_2690	41	p2	(CA)6	12	90694	90705	
PHG9_Scaffold_2690	42	p3	(TTC)5	15	92978	92992	potentially variable
PHG9_Scaffold_2690	47	p2	(AT)8	16	105633	105648	potentially variable
PHG9_Scaffold_2693	1	p2	(AT)6	12	71	82	

PHG9_Scaffold_2693	23	p2	(AG)6	12	76622	76633	
PHG9_Scaffold_2693	24	p3	(ATA)5	15	83693	83707	potentially variable
PHG9_Scaffold_2693	25	p3	(CAA)5	15	84092	84106	potentially variable
PHG9_Scaffold_2693	26	p2	(TA)7	14	86530	86543	potentially variable
PHG9_Scaffold_2693	28	p2	(TC)9	18	88766	88783	potentially variable
PHG9_Scaffold_2693	32	p2	(AG)6	12	99811	99822	
PHG9_Scaffold_2693	35	p2	(AC)6	12	111287	111298	
PHG9_Scaffold_2693	39	p2	(AT)6	12	118062	118073	
PHG9_Scaffold_2693	42	p2	(TG)7	14	131844	131857	potentially variable
PHG9_Scaffold_2693	48	p2	(TA)6	12	154164	154175	
PHG9_Scaffold_2694	3	p2	(TA)6	12	9808	9819	
PHG9_Scaffold_2694	9	p2	(CT)8	16	16904	16919	potentially variable
PHG9_Scaffold_2701	1	p2	(TC)6	12	6737	6748	
PHG9_Scaffold_2701	2	p2	(TA)8	16	7365	7380	potentially variable
PHG9_Scaffold_2702	1	p2	(AT)9	18	9150	9167	potentially variable
PHG9_Scaffold_2706	10	p2	(AT)7	14	37445	37458	potentially variable
PHG9_Scaffold_2708	1	p2	(TA)7	14	2502	2515	potentially variable
PHG9_Scaffold_2709	1	p2	(AT)6	12	1136	1147	
PHG9_Scaffold_2710	4	p3	(CGG)5	15	4531	4545	potentially

							variable
PHG9_Scaffold_2711	1	p2	(CT)6	12	244	255	
PHG9_Scaffold_2716	13	p2	(AC)6	12	45100	45111	
PHG9_Scaffold_2716	19	p2	(AT)9	18	62948	62965	potentially variable
PHG9_Scaffold_2716	21	p2	(GA)6	12	65538	65549	
PHG9_Scaffold_2716	36	p3	(CAT)5	15	104369	104383	potentially variable
PHG9_Scaffold_2718	3	p2	(AT)6	12	7830	7841	
PHG9_Scaffold_2721	2	p2	(TA)7	14	11484	11497	potentially variable
PHG9_Scaffold_2724	5	p3	(AGT)5	15	10434	10448	potentially variable
PHG9_Scaffold_2724	6	p2	(GT)6	12	13546	13557	
PHG9_Scaffold_2724	7	p2	(CT)6	12	13912	13923	
PHG9_Scaffold_2724	8	p2	(TC)7	14	14768	14781	potentially variable
PHG9_Scaffold_2726	1	p2	(TA)8	16	903	918	potentially variable
PHG9_Scaffold_2734	2	p2	(AG)6	12	4314	4325	
PHG9_Scaffold_2734	11	p2	(TA)9	18	36249	36266	potentially variable
PHG9_Scaffold_2734	31	p2	(AT)6	12	99861	99872	
PHG9_Scaffold_2735	1	p2	(CT)6	12	2240	2251	
PHG9_Scaffold_2743	3	p2	(TA)6	12	4243	4254	
PHG9_Scaffold_2743	4	p2	(AT)7	14	4381	4394	potentially variable
PHG9_Scaffold_2743	5	p2	(TA)6	12	6272	6283	
PHG9_Scaffold_2744	2	p3	(CAA)6	18	10554	10571	potentially

							variable
PHG9_Scaffold_2745	2	p3	(AAT)5	15	3920	3934	potentially variable
PHG9_Scaffold_2745	12	p2	(AT)9	18	36394	36411	potentially variable
PHG9_Scaffold_2747	4	p3	(CCA)5	15	24138	24152	potentially variable
PHG9_Scaffold_2747	17	p2	(AT)9	18	77082	77099	potentially variable
PHG9_Scaffold_2747	20	p2	(AT)7	14	86146	86159	potentially variable
PHG9_Scaffold_2747	28	p2	(TA)6	12	96990	97001	
PHG9_Scaffold_2749	1	p3	(CAC)5	15	3881	3895	potentially variable
PHG9_Scaffold_2749	4	p3	(ATC)5	15	10076	10090	potentially variable
PHG9_Scaffold_2750	1	p2	(AG)6	12	11336	11347	
PHG9_Scaffold_2751	2	p2	(TA)6	12	8683	8694	
PHG9_Scaffold_2752	4	p2	(TA)9	18	11613	11630	potentially variable
PHG9_Scaffold_2752	8	p3	(ATA)6	18	25923	25940	potentially variable
PHG9_Scaffold_2752	13	p3	(CAT)5	15	31014	31028	potentially variable
PHG9_Scaffold_2752	17	p2	(AT)6	12	35657	35668	
PHG9_Scaffold_2752	22	p2	(TA)7	14	60661	60674	potentially variable
PHG9_Scaffold_2752	23	p2	(TA)6	12	62596	62607	
PHG9_Scaffold_2754	1	p2	(TC)7	14	4934	4947	potentially variable

PHG9_Scaffold_2757	2	p3	(CCA)5	15	6306	6320	potentially variable
PHG9_Scaffold_2757	5	p3	(GGA)5	15	15938	15952	potentially variable
PHG9_Scaffold_2757	18	p2	(TA)7	14	77920	77933	potentially variable
PHG9_Scaffold_2757	21	p3	(GGT)6	18	81525	81542	potentially variable
PHG9_Scaffold_2759	3	p2	(TA)9	18	19730	19747	potentially variable
PHG9_Scaffold_2759	4	p2	(CT)8	16	20524	20539	potentially variable
PHG9_Scaffold_2759	5	p3	(GTG)6	18	22745	22762	potentially variable
PHG9_Scaffold_2759	6	p2	(AT)6	12	23029	23040	
PHG9_Scaffold_2760	1	p2	(TA)6	12	8713	8724	
PHG9_Scaffold_2761	8	p2	(TA)7	14	22842	22855	potentially variable
PHG9_Scaffold_2761	11	p2	(AT)7	14	25488	25501	potentially variable
PHG9_Scaffold_2761	12	p2	(AT)9	18	28064	28081	potentially variable
PHG9_Scaffold_2761	16	p2	(AG)9	18	40480	40497	potentially variable
PHG9_Scaffold_2762	2	p3	(ATA)5	15	4426	4440	potentially variable
PHG9_Scaffold_2762	7	p2	(CT)7	14	9985	9998	potentially variable
PHG9_Scaffold_2763	27	p2	(GA)6	12	63472	63483	
PHG9_Scaffold_2763	29	p2	(AT)9	18	69330	69347	potentially

							variable
PHG9_Scaffold_2763	30	p2	(AT)9	18	76955	76972	potentially variable
PHG9_Scaffold_2764	5	p2	(AT)6	12	3301	3312	
PHG9_Scaffold_2764	6	p2	(TA)7	14	10949	10962	potentially variable
PHG9_Scaffold_2764	17	p3	(AGA)5	15	27038	27052	potentially variable
PHG9_Scaffold_2764	21	p2	(AT)7	14	36883	36896	potentially variable
PHG9_Scaffold_2764	26	p2	(AT)7	14	56276	56289	potentially variable
PHG9_Scaffold_2765	11	p2	(AT)7	14	18183	18196	potentially variable
PHG9_Scaffold_2766	1	p2	(TA)6	12	1080	1091	
PHG9_Scaffold_2767	12	p3	(GAA)5	15	46001	46015	potentially variable
PHG9_Scaffold_2768	2	p2	(TG)9	18	5044	5061	potentially variable
PHG9_Scaffold_2768	14	p2	(AT)9	18	36258	36275	potentially variable
PHG9_Scaffold_2768	21	p3	(TTG)5	15	49122	49136	potentially variable
PHG9_Scaffold_2768	39	p2	(AG)7	14	120584	120597	potentially variable
PHG9_Scaffold_2768	41	p2	(CT)6	12	136467	136478	
PHG9_Scaffold_2770	5	p2	(AT)6	12	22580	22591	
PHG9_Scaffold_2770	6	p2	(AT)8	16	26406	26421	potentially variable
PHG9_Scaffold_2770	10	p2	(GA)8	16	37543	37558	potentially

							variable
PHG9_Scaffold_2772	1	p2	(TA)6	12	3195	3206	
PHG9_Scaffold_2773	1	p2	(GT)7	14	4857	4870	potentially variable
PHG9_Scaffold_2775	6	p2	(TA)9	18	16264	16281	potentially variable
PHG9_Scaffold_2776	14	p3	(AAT)5	15	28672	28686	potentially variable
PHG9_Scaffold_2778	11	p2	(AT)6	12	54810	54821	
PHG9_Scaffold_2778	33	p2	(TA)7	14	195332	195345	potentially variable
PHG9_Scaffold_2782	2	p3	(ATA)5	15	2197	2211	potentially variable
PHG9_Scaffold_2782	4	p2	(GA)6	12	6013	6024	
PHG9_Scaffold_2782	6	p2	(CA)6	12	8751	8762	
PHG9_Scaffold_2782	9	p2	(TA)9	18	13853	13870	potentially variable
PHG9_Scaffold_2782	10	p3	(AAG)5	15	15044	15058	potentially variable
PHG9_Scaffold_2786	1	p3	(ATA)5	15	4633	4647	potentially variable
PHG9_Scaffold_2787	15	p2	(TA)6	12	39992	40003	
PHG9_Scaffold_2787	18	p2	(AT)9	18	42009	42026	potentially variable
PHG9_Scaffold_2787	23	p2	(TC)7	14	61617	61630	potentially variable
PHG9_Scaffold_2787	24	p2	(TA)8	16	61844	61859	potentially variable
PHG9_Scaffold_2788	5	p2	(TA)6	12	9666	9677	

PHG9_Scaffold_2790	4	p2	(AT)7	14	16201	16214	potentially variable
PHG9_Scaffold_2791	1	p2	(TA)6	12	2057	2068	
PHG9_Scaffold_2791	3	p3	(TAT)5	15	9587	9601	potentially variable
PHG9_Scaffold_2792	5	p2	(AT)6	12	6264	6275	
PHG9_Scaffold_2792	8	p3	(TTC)5	15	17000	17014	potentially variable
PHG9_Scaffold_2793	2	p2	(CT)7	14	6404	6417	potentially variable
PHG9_Scaffold_2794	2	p2	(TC)6	12	824	835	
PHG9_Scaffold_2794	3	p2	(AT)6	12	4940	4951	
PHG9_Scaffold_2794	7	p2	(TA)6	12	25631	25642	
PHG9_Scaffold_2795	3	p2	(TA)6	12	7562	7573	
PHG9_Scaffold_2795	4	p3	(GAA)5	15	8815	8829	potentially variable
PHG9_Scaffold_2795	12	p3	(AGT)5	15	36737	36751	potentially variable
PHG9_Scaffold_2795	13	p2	(CT)6	12	38012	38023	
PHG9_Scaffold_2796	1	p2	(TA)8	16	3545	3560	potentially variable
PHG9_Scaffold_2796	4	p2	(TA)9	18	13572	13589	potentially variable
PHG9_Scaffold_2796	5	p2	(TA)7	14	15215	15228	potentially variable
PHG9_Scaffold_2797	2	p2	(TA)6	12	7344	7355	
PHG9_Scaffold_2797	7	p3	(TTA)5	15	14602	14616	potentially variable
PHG9_Scaffold_2797	11	p2	(AT)6	12	17784	17795	

PHG9_Scaffold_2799	1	p2	(TG)7	14	5713	5726	potentially variable
PHG9_Scaffold_2800	1	p2	(AT)8	16	22	37	potentially variable
PHG9_Scaffold_2800	4	p3	(TGG)5	15	9057	9071	potentially variable
PHG9_Scaffold_2800	12	p2	(AT)7	14	28747	28760	potentially variable
PHG9_Scaffold_2800	15	p2	(CT)7	14	33328	33341	potentially variable
PHG9_Scaffold_2800	17	p2	(TA)7	14	36573	36586	potentially variable
PHG9_Scaffold_2800	18	p2	(TC)6	12	37670	37681	
PHG9_Scaffold_2800	24	p3	(ACG)5	15	65160	65174	potentially variable
PHG9_Scaffold_2800	32	p3	(TTA)5	15	87260	87274	potentially variable
PHG9_Scaffold_2801	1	p2	(TA)8	16	1	16	potentially variable
PHG9_Scaffold_2801	3	p2	(AT)6	12	3161	3172	
PHG9_Scaffold_2801	6	p2	(CT)7	14	6641	6654	potentially variable
PHG9_Scaffold_2802	2	p2	(AT)7	14	7937	7950	potentially variable
PHG9_Scaffold_2802	6	p3	(ATT)5	15	16655	16669	potentially variable
PHG9_Scaffold_2802	10	p3	(CAT)5	15	46012	46026	potentially variable
PHG9_Scaffold_2802	12	p3	(TTA)5	15	48066	48080	potentially variable

PHG9_Scaffold_2802	14	p2	(TA)8	16	56038	56053	potentially variable
PHG9_Scaffold_2802	15	p2	(TA)6	12	57746	57757	
PHG9_Scaffold_2804	5	p2	(GA)6	12	10870	10881	
PHG9_Scaffold_2804	6	p2	(GT)6	12	12654	12665	
PHG9_Scaffold_2804	7	p2	(TA)7	14	13561	13574	potentially variable
PHG9_Scaffold_2804	8	p3	(ATT)6	18	14361	14378	potentially variable
PHG9_Scaffold_2804	17	p2	(GT)6	12	42225	42236	
PHG9_Scaffold_2804	20	p3	(TTA)5	15	60674	60688	potentially variable
PHG9_Scaffold_2804	22	p2	(AT)8	16	64757	64772	potentially variable
PHG9_Scaffold_2804	23	p3	(ACC)6	18	65244	65261	potentially variable
PHG9_Scaffold_2807	9	p2	(TA)6	12	58975	58986	
PHG9_Scaffold_2807	13	p2	(TA)6	12	78544	78555	
PHG9_Scaffold_2809	1	p2	(TA)7	14	2146	2159	potentially variable
PHG9_Scaffold_2809	3	p2	(TA)6	12	14127	14138	
PHG9_Scaffold_2809	4	p2	(TA)6	12	23840	23851	
PHG9_Scaffold_2809	6	p3	(ACA)5	15	39376	39390	potentially variable
PHG9_Scaffold_2809	7	p2	(TA)6	12	40411	40422	
PHG9_Scaffold_2809	9	p2	(TA)8	16	51517	51532	potentially variable
PHG9_Scaffold_2810	1	p2	(AT)6	12	946	957	
PHG9_Scaffold_2810	2	p2	(TC)9	18	5940	5957	potentially

							variable
PHG9_Scaffold_2810	3	p3	(AAT)5	15	8778	8792	potentially variable
PHG9_Scaffold_2813	7	p2	(GA)6	12	37332	37343	
PHG9_Scaffold_2813	12	p2	(CT)7	14	43159	43172	potentially variable
PHG9_Scaffold_2813	13	p2	(AT)7	14	50603	50616	potentially variable
PHG9_Scaffold_2814	4	p2	(AG)7	14	8275	8288	potentially variable
PHG9_Scaffold_2814	5	p2	(AG)6	12	12996	13007	
PHG9_Scaffold_2815	4	p3	(TTG)5	15	7714	7728	potentially variable
PHG9_Scaffold_2815	8	p2	(GA)8	16	21524	21539	potentially variable
PHG9_Scaffold_2815	9	p2	(TA)7	14	22075	22088	potentially variable
PHG9_Scaffold_2816	4	p2	(AT)6	12	6290	6301	
PHG9_Scaffold_2817	2	p2	(CT)8	16	2832	2847	potentially variable
PHG9_Scaffold_2817	19	p2	(CT)6	12	28992	29003	
PHG9_Scaffold_2817	22	p2	(TA)6	12	33289	33300	
PHG9_Scaffold_2817	24	p2	(TA)8	16	37985	38000	potentially variable
PHG9_Scaffold_2817	26	p2	(TA)7	14	38475	38488	potentially variable
PHG9_Scaffold_2817	32	p2	(TC)7	14	56951	56964	potentially variable
PHG9_Scaffold_2817	33	p2	(AT)6	12	57189	57200	

PHG9_Scaffold_2817	43	p2	(TC)6	12	79410	79421	
PHG9_Scaffold_2817	47	p2	(TA)6	12	95670	95681	
PHG9_Scaffold_2817	55	p3	(TAT)5	15	106319	106333	potentially variable
PHG9_Scaffold_2817	57	p2	(AT)9	18	110052	110069	potentially variable
PHG9_Scaffold_2817	58	p2	(TA)7	14	111142	111155	potentially variable
PHG9_Scaffold_2817	72	p2	(AG)9	18	147887	147904	potentially variable
PHG9_Scaffold_2817	78	p2	(TC)6	12	162072	162083	
PHG9_Scaffold_2817	82	p3	(ATA)6	18	172338	172355	potentially variable
PHG9_Scaffold_2819	15	p2	(TA)9	18	25065	25082	potentially variable
PHG9_Scaffold_2819	17	p3	(TTC)5	15	26868	26882	potentially variable
PHG9_Scaffold_2819	22	p3	(TAA)5	15	41801	41815	potentially variable
PHG9_Scaffold_2819	24	p2	(TA)7	14	51900	51913	potentially variable
PHG9_Scaffold_2819	37	p3	(AAC)5	15	83583	83597	potentially variable
PHG9_Scaffold_2820	1	p2	(TA)7	14	3351	3364	potentially variable
PHG9_Scaffold_2822	1	p2	(TG)7	14	722	735	potentially variable
PHG9_Scaffold_2822	3	p2	(TA)6	12	5365	5376	
PHG9_Scaffold_2824	4	p3	(TAA)5	15	13672	13686	potentially variable

PHG9_Scaffold_2826	9	p2	(TA)6	12	20203	20214	
PHG9_Scaffold_2831	3	p2	(TG)6	12	9562	9573	
PHG9_Scaffold_2832	1	p3	(ATA)5	15	8774	8788	potentially variable
PHG9_Scaffold_2834	1	p3	(AAT)5	15	18255	18269	potentially variable
PHG9_Scaffold_2834	2	p2	(AT)7	14	24451	24464	potentially variable
PHG9_Scaffold_2834	5	p2	(GA)9	18	42125	42142	potentially variable
PHG9_Scaffold_2834	6	p2	(AT)8	16	43043	43058	potentially variable
PHG9_Scaffold_2834	9	p3	(ATT)5	15	44669	44683	potentially variable
PHG9_Scaffold_2836	4	p2	(AT)6	12	19860	19871	
PHG9_Scaffold_2836	10	p2	(AT)7	14	51361	51374	potentially variable
PHG9_Scaffold_2836	11	p2	(TA)6	12	53235	53246	
PHG9_Scaffold_2837	4	p2	(AT)7	14	21637	21650	potentially variable
PHG9_Scaffold_2837	11	p2	(AT)6	12	52221	52232	
PHG9_Scaffold_2838	3	p2	(TA)7	14	9281	9294	potentially variable
PHG9_Scaffold_2839	2	p2	(TA)7	14	5257	5270	potentially variable
PHG9_Scaffold_2841	1	p2	(TA)6	12	812	823	
PHG9_Scaffold_2841	2	p2	(AT)6	12	2273	2284	
PHG9_Scaffold_2842	2	p3	(TTA)5	15	8614	8628	potentially variable

PHG9_Scaffold_2842	5	p3	(TGG)6	18	12120	12137	potentially variable
PHG9_Scaffold_2843	3	p2	(TA)8	16	3296	3311	potentially variable
PHG9_Scaffold_2843	6	p2	(TA)7	14	4747	4760	potentially variable
PHG9_Scaffold_2844	5	p3	(GAA)5	15	22799	22813	potentially variable
PHG9_Scaffold_2844	7	p3	(TAT)5	15	37238	37252	potentially variable
PHG9_Scaffold_2844	9	p3	(ATC)5	15	44934	44948	potentially variable
PHG9_Scaffold_2845	3	p2	(AG)7	14	6804	6817	potentially variable
PHG9_Scaffold_2846	8	p2	(AG)9	18	18885	18902	potentially variable
PHG9_Scaffold_2846	14	p2	(AG)7	14	81275	81288	potentially variable
PHG9_Scaffold_2846	15	p3	(AAT)5	15	82010	82024	potentially variable
PHG9_Scaffold_2846	18	p3	(TCA)5	15	91632	91646	potentially variable
PHG9_Scaffold_2849	4	p2	(CT)7	14	6690	6703	potentially variable
PHG9_Scaffold_2849	5	p2	(AT)7	14	15775	15788	potentially variable
PHG9_Scaffold_2857	6	p2	(AT)6	12	14052	14063	
PHG9_Scaffold_2857	8	p2	(AC)6	12	15594	15605	
PHG9_Scaffold_2857	10	p2	(TC)9	18	18966	18983	potentially variable

PHG9_Scaffold_2857	11	p3	(CTG)5	15	21944	21958	potentially variable
PHG9_Scaffold_2857	13	p3	(ATT)5	15	27056	27070	potentially variable
PHG9_Scaffold_2857	16	p2	(AT)6	12	32666	32677	
PHG9_Scaffold_2858	4	p2	(TA)8	16	45958	45973	potentially variable
PHG9_Scaffold_2860	2	p2	(AT)8	16	1061	1076	potentially variable
PHG9_Scaffold_2860	4	p3	(TAA)5	15	11873	11887	potentially variable
PHG9_Scaffold_2861	4	p2	(AC)7	14	12322	12335	potentially variable
PHG9_Scaffold_2864	2	p2	(AT)6	12	5620	5631	
PHG9_Scaffold_2864	7	p2	(AT)9	18	13757	13774	potentially variable
PHG9_Scaffold_2864	12	p3	(AGA)5	15	18871	18885	potentially variable
PHG9_Scaffold_2864	17	p3	(AAT)5	15	23016	23030	potentially variable
PHG9_Scaffold_2864	25	p2	(TC)7	14	54303	54316	potentially variable
PHG9_Scaffold_2864	39	p2	(AT)7	14	87911	87924	potentially variable
PHG9_Scaffold_2864	50	p2	(CT)8	16	118031	118046	potentially variable
PHG9_Scaffold_2864	56	p2	(AT)8	16	130142	130157	potentially variable
PHG9_Scaffold_2864	61	p2	(AT)6	12	141346	141357	
PHG9_Scaffold_2864	66	p2	(GA)6	12	151705	151716	

PHG9_Scaffold_2864	75	p2	(AT)7	14	171940	171953	potentially variable
PHG9_Scaffold_2864	83	p3	(CTT)6	18	198859	198876	potentially variable
PHG9_Scaffold_2864	88	p3	(ATT)5	15	219115	219129	potentially variable
PHG9_Scaffold_2864	91	p2	(TA)6	12	223621	223632	
PHG9_Scaffold_2864	98	p2	(AT)6	12	236280	236291	
PHG9_Scaffold_2864	100	p2	(AT)8	16	236692	236707	potentially variable
PHG9_Scaffold_2864	102	p3	(TTA)5	15	238643	238657	potentially variable
PHG9_Scaffold_2864	107	p2	(TA)7	14	249674	249687	potentially variable
PHG9_Scaffold_2868	7	p3	(AAT)5	15	31188	31202	potentially variable
PHG9_Scaffold_2868	10	p3	(TCA)5	15	46886	46900	potentially variable
PHG9_Scaffold_2868	11	p2	(AT)6	12	47318	47329	
PHG9_Scaffold_2869	5	p2	(AT)8	16	7504	7519	potentially variable
PHG9_Scaffold_2869	6	p2	(AT)6	12	16468	16479	
PHG9_Scaffold_2871	5	p2	(TA)7	14	10729	10742	potentially variable
PHG9_Scaffold_2872	2	p2	(TA)6	12	6228	6239	
PHG9_Scaffold_2872	3	p2	(TA)7	14	6375	6388	potentially variable
PHG9_Scaffold_2872	7	p3	(ATA)5	15	10560	10574	potentially variable
PHG9_Scaffold_2872	9	p2	(TA)7	14	14607	14620	potentially

							variable
PHG9_Scaffold_2872	10	p3	(ATG)5	15	14921	14935	potentially variable
PHG9_Scaffold_2872	13	p2	(TC)9	18	17481	17498	potentially variable
PHG9_Scaffold_2872	20	p2	(TC)8	16	26971	26986	potentially variable
PHG9_Scaffold_2874	2	p2	(AT)6	12	3247	3258	
PHG9_Scaffold_2876	2	p3	(CGG)5	15	2797	2811	potentially variable
PHG9_Scaffold_2878	9	p2	(TA)8	16	41150	41165	potentially variable
PHG9_Scaffold_2878	14	p2	(AC)6	12	57703	57714	
PHG9_Scaffold_2880	1	p2	(AG)9	18	16965	16982	potentially variable
PHG9_Scaffold_2880	4	p2	(AG)7	14	35225	35238	potentially variable
PHG9_Scaffold_2880	5	p3	(ATC)5	15	35595	35609	potentially variable
PHG9_Scaffold_2882	5	p2	(CT)7	14	23875	23888	potentially variable
PHG9_Scaffold_2882	7	p3	(GAA)5	15	27961	27975	potentially variable
PHG9_Scaffold_2882	8	p2	(AG)6	12	30421	30432	
PHG9_Scaffold_2882	17	p3	(TAT)5	15	62370	62384	potentially variable
PHG9_Scaffold_2883	1	p2	(TA)8	16	55	70	potentially variable
PHG9_Scaffold_2883	8	p2	(TC)6	12	60052	60063	
PHG9_Scaffold_2883	11	p3	(CAG)5	15	82396	82410	potentially

							variable
PHG9_Scaffold_2885	3	p3	(AAG)5	15	1651	1665	potentially variable
PHG9_Scaffold_2885	5	p2	(AG)6	12	16930	16941	
PHG9_Scaffold_2885	6	p2	(TA)6	12	19286	19297	
PHG9_Scaffold_2885	7	p2	(CT)7	14	19793	19806	potentially variable
PHG9_Scaffold_2885	10	p2	(AT)6	12	34756	34767	
PHG9_Scaffold_2885	22	p2	(AT)6	12	72771	72782	
PHG9_Scaffold_2885	24	p3	(TAT)5	15	80619	80633	potentially variable
PHG9_Scaffold_2885	31	p2	(AT)9	18	116302	116319	potentially variable
PHG9_Scaffold_2885	45	p2	(CT)7	14	197270	197283	potentially variable
PHG9_Scaffold_2885	54	p2	(TC)8	16	229643	229658	potentially variable
PHG9_Scaffold_2885	58	p3	(TGA)5	15	245921	245935	potentially variable
PHG9_Scaffold_2885	59	p3	(CAT)5	15	256662	256676	potentially variable
PHG9_Scaffold_2886	2	p2	(AT)8	16	9651	9666	potentially variable
PHG9_Scaffold_2887	3	p2	(AC)8	16	5734	5749	potentially variable
PHG9_Scaffold_2889	2	p2	(TC)6	12	6419	6430	
PHG9_Scaffold_2889	9	p2	(TA)7	14	24034	24047	potentially variable
PHG9_Scaffold_2889	13	p3	(TTA)6	18	34078	34095	potentially variable

PHG9_Scaffold_2889	20	p2	(AT)6	12	76232	76243	
PHG9_Scaffold_2889	21	p2	(TA)7	14	83766	83779	potentially variable
PHG9_Scaffold_2889	30	p2	(AT)8	16	135092	135107	potentially variable
PHG9_Scaffold_2889	32	p2	(CT)7	14	139226	139239	potentially variable
PHG9_Scaffold_2889	34	p2	(AT)7	14	147806	147819	potentially variable
PHG9_Scaffold_2889	40	p2	(AT)8	16	173872	173887	potentially variable
PHG9_Scaffold_2889	41	p2	(TA)6	12	176841	176852	
PHG9_Scaffold_2889	43	p3	(GAT)5	15	179602	179616	potentially variable
PHG9_Scaffold_2889	44	p2	(TA)7	14	179993	180006	potentially variable
PHG9_Scaffold_2889	45	p2	(AT)7	14	184153	184166	potentially variable
PHG9_Scaffold_2889	48	p2	(AT)7	14	202448	202461	potentially variable
PHG9_Scaffold_2889	50	p2	(CA)6	12	205815	205826	
PHG9_Scaffold_2890	5	p3	(TGA)5	15	11797	11811	potentially variable
PHG9_Scaffold_2890	9	p3	(ATG)5	15	21843	21857	potentially variable
PHG9_Scaffold_2892	4	p3	(TTA)5	15	19352	19366	potentially variable
PHG9_Scaffold_2892	22	p3	(TAT)5	15	94902	94916	potentially variable
PHG9_Scaffold_2896	1	p2	(AG)9	18	3666	3683	potentially

							variable
PHG9_Scaffold_2896	2	p3	(AAT)5	15	3848	3862	potentially variable
PHG9_Scaffold_2898	14	p2	(TA)6	12	43446	43457	
PHG9_Scaffold_2898	20	p2	(GA)6	12	68542	68553	
PHG9_Scaffold_2900	3	p2	(TA)7	14	7284	7297	potentially variable
PHG9_Scaffold_2900	4	p2	(AT)8	16	10025	10040	potentially variable
PHG9_Scaffold_2901	4	p2	(GT)6	12	6249	6260	
PHG9_Scaffold_2904	4	p3	(TAT)6	18	8520	8537	potentially variable
PHG9_Scaffold_2906	4	p2	(GA)9	18	3530	3547	potentially variable
PHG9_Scaffold_2906	9	p2	(GA)6	12	8555	8566	
PHG9_Scaffold_2908	9	p2	(TA)6	12	13566	13577	
PHG9_Scaffold_2908	11	p2	(TC)6	12	15309	15320	
PHG9_Scaffold_2908	12	p3	(CAC)5	15	17202	17216	potentially variable
PHG9_Scaffold_2908	14	p2	(AT)6	12	24374	24385	
PHG9_Scaffold_2909	11	p2	(TA)6	12	33341	33352	
PHG9_Scaffold_2911	2	p2	(TC)9	18	8702	8719	potentially variable
PHG9_Scaffold_2916	2	p2	(TA)6	12	7122	7133	
PHG9_Scaffold_2919	1	p2	(TA)6	12	12096	12107	
PHG9_Scaffold_2921	2	p2	(TA)6	12	6679	6690	
PHG9_Scaffold_2921	4	p2	(AG)7	14	39596	39609	potentially variable
PHG9_Scaffold_2921	5	p2	(TA)7	14	41544	41557	potentially

							variable
PHG9_Scaffold_2922	4	p2	(TA)6	12	9797	9808	
PHG9_Scaffold_2922	5	p3	(ATT)5	15	10921	10935	potentially variable
PHG9_Scaffold_2926	17	p2	(CT)7	14	47782	47795	potentially variable
PHG9_Scaffold_2926	18	p2	(AC)6	12	50171	50182	
PHG9_Scaffold_2926	30	p2	(AT)7	14	82772	82785	potentially variable
PHG9_Scaffold_2926	31	p2	(AT)6	12	83938	83949	
PHG9_Scaffold_2926	34	p3	(TTA)5	15	101785	101799	potentially variable
PHG9_Scaffold_2926	36	p2	(TA)7	14	102562	102575	potentially variable
PHG9_Scaffold_2927	1	p2	(AT)6	12	795	806	
PHG9_Scaffold_2928	4	p2	(TA)6	12	16945	16956	
PHG9_Scaffold_2928	12	p2	(CT)9	18	31464	31481	potentially variable
PHG9_Scaffold_2928	14	p2	(AT)7	14	42307	42320	potentially variable
PHG9_Scaffold_2929	1	p3	(TTG)5	15	2554	2568	potentially variable
PHG9_Scaffold_2929	23	p2	(TA)6	12	80950	80961	
PHG9_Scaffold_2930	5	p2	(TA)6	12	4768	4779	
PHG9_Scaffold_2931	5	p2	(AT)7	14	1621	1634	potentially variable
PHG9_Scaffold_2932	7	p2	(GT)8	16	11268	11283	potentially variable
PHG9_Scaffold_2934	3	p2	(CA)7	14	1857	1870	potentially

							variable
PHG9_Scaffold_2934	9	p2	(AC)6	12	14594	14605	
PHG9_Scaffold_2935	7	p2	(TA)8	16	44572	44587	potentially variable
PHG9_Scaffold_2935	8	p2	(AT)9	18	45436	45453	potentially variable
PHG9_Scaffold_2938	1	p3	(TAA)5	15	15865	15879	potentially variable
PHG9_Scaffold_2938	2	p2	(AT)6	12	17083	17094	
PHG9_Scaffold_2938	3	p2	(TA)6	12	17981	17992	
PHG9_Scaffold_2939	1	p2	(AT)6	12	11436	11447	
PHG9_Scaffold_2943	2	p2	(AT)7	14	6490	6503	potentially variable
PHG9_Scaffold_2945	2	p2	(TA)6	12	2424	2435	
PHG9_Scaffold_2946	3	p2	(TA)7	14	13802	13815	potentially variable
PHG9_Scaffold_2948	5	p2	(TC)6	12	5151	5162	
PHG9_Scaffold_2948	9	p3	(AAT)6	18	20025	20042	potentially variable
PHG9_Scaffold_2948	10	p2	(AT)8	16	21263	21278	potentially variable
PHG9_Scaffold_2948	12	p2	(AT)6	12	26747	26758	
PHG9_Scaffold_2948	15	p2	(AT)9	18	36768	36785	potentially variable
PHG9_Scaffold_2948	16	p2	(AT)8	16	37755	37770	potentially variable
PHG9_Scaffold_2949	6	p2	(TC)7	14	11086	11099	potentially variable
PHG9_Scaffold_2956	5	p2	(AT)9	18	7016	7033	potentially

							variable
PHG9_Scaffold_2956	16	p2	(TA)8	16	35152	35167	potentially variable
PHG9_Scaffold_2956	30	p2	(AC)7	14	64222	64235	potentially variable
PHG9_Scaffold_2956	31	p2	(TA)8	16	68054	68069	potentially variable
PHG9_Scaffold_2957	1	p3	(AAC)5	15	4080	4094	potentially variable
PHG9_Scaffold_2959	1	p2	(TA)6	12	806	817	
PHG9_Scaffold_2959	2	p2	(AT)6	12	1240	1251	
PHG9_Scaffold_2959	3	p2	(TA)7	14	18019	18032	potentially variable
PHG9_Scaffold_2959	10	p3	(TAT)5	15	37391	37405	potentially variable
PHG9_Scaffold_2959	20	p3	(TCT)6	18	89950	89967	potentially variable
PHG9_Scaffold_2959	25	p2	(TA)8	16	103135	103150	potentially variable
PHG9_Scaffold_2959	30	p2	(AT)6	12	134606	134617	
PHG9_Scaffold_2960	1	p2	(TA)7	14	4500	4513	potentially variable
PHG9_Scaffold_2960	2	p2	(TA)7	14	8578	8591	potentially variable
PHG9_Scaffold_2960	3	p2	(TA)6	12	8803	8814	
PHG9_Scaffold_2963	3	p2	(TA)6	12	8803	8814	
PHG9_Scaffold_2964	1	p3	(TTC)6	18	1404	1421	potentially variable
PHG9_Scaffold_2964	3	p2	(TA)9	18	20385	20402	potentially variable

PHG9_Scaffold_2965	3	p2	(TA)7	14	7660	7673	potentially variable
PHG9_Scaffold_2965	9	p2	(AT)8	16	17850	17865	potentially variable
PHG9_Scaffold_2965	15	p2	(AT)6	12	27162	27173	
PHG9_Scaffold_2965	27	p2	(AG)8	16	46414	46429	potentially variable
PHG9_Scaffold_2965	32	p2	(AG)6	12	50856	50867	
PHG9_Scaffold_2965	35	p3	(CTT)5	15	55438	55452	potentially variable
PHG9_Scaffold_2967	3	p2	(TA)6	12	2755	2766	
PHG9_Scaffold_2967	10	p2	(TA)7	14	12208	12221	potentially variable
PHG9_Scaffold_2967	17	p2	(AG)6	12	31566	31577	
PHG9_Scaffold_2967	18	p3	(ATT)5	15	33798	33812	potentially variable
PHG9_Scaffold_2967	19	p2	(TA)9	18	34799	34816	potentially variable
PHG9_Scaffold_2968	3	p2	(TG)6	12	8009	8020	
PHG9_Scaffold_2968	5	p2	(TA)6	12	19118	19129	
PHG9_Scaffold_2970	4	p2	(TA)6	12	18651	18662	
PHG9_Scaffold_2970	5	p2	(TA)7	14	22789	22802	potentially variable
PHG9_Scaffold_2971	4	p2	(AT)6	12	17696	17707	
PHG9_Scaffold_2973	4	p3	(TTA)5	15	18927	18941	potentially variable
PHG9_Scaffold_2974	2	p2	(TA)6	12	5038	5049	
PHG9_Scaffold_2974	3	p2	(CT)7	14	8443	8456	potentially variable

PHG9_Scaffold_2974	4	p2	(AT)6	12	11365	11376	
PHG9_Scaffold_2976	6	p2	(TA)9	18	34139	34156	potentially variable
PHG9_Scaffold_2976	8	p2	(AG)6	12	38346	38357	
PHG9_Scaffold_2976	18	p3	(ATT)5	15	65720	65734	potentially variable
PHG9_Scaffold_2978	2	p2	(TA)7	14	5837	5850	potentially variable
PHG9_Scaffold_2978	8	p2	(TA)9	18	25561	25578	potentially variable
PHG9_Scaffold_2978	12	p2	(TA)9	18	39589	39606	potentially variable
PHG9_Scaffold_2978	14	p2	(AT)7	14	42793	42806	potentially variable
PHG9_Scaffold_2978	15	p3	(CAC)5	15	44034	44048	potentially variable
PHG9_Scaffold_2978	18	p3	(AAG)5	15	46953	46967	potentially variable
PHG9_Scaffold_2978	21	p3	(TTG)6	18	52315	52332	potentially variable
PHG9_Scaffold_2978	23	p2	(AC)7	14	63677	63690	potentially variable
PHG9_Scaffold_2978	24	p2	(TA)8	16	67976	67991	potentially variable
PHG9_Scaffold_2978	27	p2	(TA)7	14	72279	72292	potentially variable
PHG9_Scaffold_2981	9	p2	(TA)6	12	19408	19419	
PHG9_Scaffold_2982	2	p3	(GGT)5	15	6458	6472	potentially variable
PHG9_Scaffold_2982	3	p2	(AT)6	12	12818	12829	

PHG9_Scaffold_2982	4	p2	(TA)6	12	13601	13612	
PHG9_Scaffold_2986	1	p2	(TA)8	16	1803	1818	potentially variable
PHG9_Scaffold_2986	6	p2	(CA)8	16	11403	11418	potentially variable
PHG9_Scaffold_2986	7	p3	(GAT)5	15	12362	12376	potentially variable
PHG9_Scaffold_2987	5	p2	(TA)6	12	19752	19763	
PHG9_Scaffold_2989	1	p2	(CT)7	14	504	517	potentially variable
PHG9_Scaffold_2992	4	p2	(TA)7	14	11592	11605	potentially variable
PHG9_Scaffold_2992	5	p3	(AAC)5	15	18557	18571	potentially variable
PHG9_Scaffold_2992	7	p3	(TAA)5	15	20776	20790	potentially variable
PHG9_Scaffold_2992	8	p2	(CA)7	14	22490	22503	potentially variable
PHG9_Scaffold_2992	16	p2	(CT)6	12	41177	41188	
PHG9_Scaffold_2992	21	p2	(TC)7	14	59489	59502	potentially variable
PHG9_Scaffold_2992	23	p3	(GAA)5	15	67609	67623	potentially variable
PHG9_Scaffold_2992	28	p3	(CAT)5	15	76803	76817	potentially variable
PHG9_Scaffold_2992	32	p2	(AT)6	12	97232	97243	
PHG9_Scaffold_2992	35	p2	(AC)6	12	117857	117868	
PHG9_Scaffold_2992	36	p6	(ACCTTA)5	30	118714	118743	Hypervariable
PHG9_Scaffold_2992	38	p2	(GT)8	16	124239	124254	potentially variable

PHG9_Scaffold_2992	41	p2	(GT)7	14	143437	143450	potentially variable
PHG9_Scaffold_2992	50	p2	(TG)6	12	160170	160181	
PHG9_Scaffold_2992	52	p2	(TA)6	12	162839	162850	
PHG9_Scaffold_2992	53	p3	(CTT)5	15	163720	163734	potentially variable
PHG9_Scaffold_2992	56	p3	(TAA)6	18	167773	167790	potentially variable
PHG9_Scaffold_2993	1	p3	(GAA)5	15	659	673	potentially variable
PHG9_Scaffold_2994	2	p2	(TA)6	12	3392	3403	
PHG9_Scaffold_2996	3	p2	(TA)9	18	6218	6235	potentially variable
PHG9_Scaffold_2998	1	p2	(TA)6	12	1362	1373	
PHG9_Scaffold_2998	5	p2	(AT)6	12	5028	5039	
PHG9_Scaffold_3000	9	p3	(TGT)5	15	10177	10191	potentially variable
PHG9_Scaffold_3003	7	p3	(GAA)6	18	73328	73345	potentially variable
PHG9_Scaffold_3006	3	p2	(AT)9	18	9715	9732	potentially variable
PHG9_Scaffold_3006	5	p3	(ATT)5	15	12979	12993	potentially variable
PHG9_Scaffold_3007	1	p2	(TA)6	12	256	267	
PHG9_Scaffold_3012	5	p2	(AT)8	16	9936	9951	potentially variable
PHG9_Scaffold_3012	6	p2	(AT)7	14	12520	12533	potentially variable
PHG9_Scaffold_3012	15	p2	(AT)7	14	37273	37286	potentially variable

PHG9_Scaffold_3012	18	p2	(TA)6	12	51580	51591	
PHG9_Scaffold_3020	2	p2	(TA)8	16	3811	3826	potentially variable
PHG9_Scaffold_3020	3	p2	(TA)6	12	4093	4104	
PHG9_Scaffold_3024	2	p2	(AT)8	16	1362	1377	potentially variable
PHG9_Scaffold_3024	4	p2	(TA)7	14	9279	9292	potentially variable
PHG9_Scaffold_3026	8	p3	(TTG)5	15	24742	24756	potentially variable
PHG9_Scaffold_3026	15	p2	(TC)6	12	46007	46018	
PHG9_Scaffold_3026	22	p2	(AG)9	18	61257	61274	potentially variable
PHG9_Scaffold_3026	24	p3	(TTC)5	15	64948	64962	potentially variable
PHG9_Scaffold_3026	27	p2	(TA)7	14	82993	83006	potentially variable
PHG9_Scaffold_3026	35	p2	(GT)8	16	104705	104720	potentially variable
PHG9_Scaffold_3026	38	p2	(AT)9	18	113117	113134	potentially variable
PHG9_Scaffold_3026	39	p2	(TC)6	12	113368	113379	
PHG9_Scaffold_3026	40	p3	(CTT)5	15	115549	115563	potentially variable
PHG9_Scaffold_3026	44	p2	(AT)7	14	120609	120622	potentially variable
PHG9_Scaffold_3026	46	p2	(TA)9	18	122564	122581	potentially variable
PHG9_Scaffold_3026	47	p3	(ATA)5	15	124533	124547	potentially variable

PHG9_Scaffold_3027	1	p2	(AG)7	14	2	15	potentially variable
PHG9_Scaffold_3027	2	p2	(CT)6	12	199	210	
PHG9_Scaffold_3027	3	p2	(CA)6	12	2041	2052	
PHG9_Scaffold_3027	6	p3	(TAA)5	15	3824	3838	potentially variable
PHG9_Scaffold_3027	7	p2	(GA)7	14	9651	9664	potentially variable
PHG9_Scaffold_3027	15	p3	(GTG)5	15	36189	36203	potentially variable
PHG9_Scaffold_3029	3	p2	(AT)7	14	4932	4945	potentially variable
PHG9_Scaffold_3030	4	p2	(AG)6	12	5881	5892	
PHG9_Scaffold_3030	5	p2	(TA)6	12	10442	10453	
PHG9_Scaffold_3030	8	p2	(AT)6	12	15363	15374	
PHG9_Scaffold_3030	9	p2	(TA)9	18	15906	15923	potentially variable
PHG9_Scaffold_3030	31	p2	(AG)8	16	67861	67876	potentially variable
PHG9_Scaffold_3030	41	p2	(AG)6	12	98781	98792	
PHG9_Scaffold_3030	43	p2	(AG)6	12	102305	102316	
PHG9_Scaffold_3031	6	p2	(TA)6	12	4208	4219	
PHG9_Scaffold_3032	1	p3	(CAC)5	15	120	134	potentially variable
PHG9_Scaffold_3033	1	p2	(AT)6	12	341	352	
PHG9_Scaffold_3033	2	p3	(AGA)5	15	1292	1306	potentially variable
PHG9_Scaffold_3033	4	p3	(TTA)5	15	2137	2151	potentially variable

PHG9_Scaffold_3033	7	p2	(TA)6	12	4156	4167	
PHG9_Scaffold_3033	10	p2	(CT)6	12	14149	14160	
PHG9_Scaffold_3033	22	p2	(AT)9	18	62511	62528	potentially variable
PHG9_Scaffold_3033	28	p2	(AT)7	14	76954	76967	potentially variable
PHG9_Scaffold_3033	29	p2	(AT)9	18	77298	77315	potentially variable
PHG9_Scaffold_3033	37	p2	(TC)6	12	106965	106976	
PHG9_Scaffold_3033	51	p3	(ATA)5	15	137496	137510	potentially variable
PHG9_Scaffold_3033	61	p2	(AT)9	18	160057	160074	potentially variable
PHG9_Scaffold_3033	63	p2	(AT)7	14	170767	170780	potentially variable
PHG9_Scaffold_3033	67	p2	(GA)6	12	177593	177604	
PHG9_Scaffold_3033	71	p2	(AG)7	14	188793	188806	potentially variable
PHG9_Scaffold_3033	72	p2	(AG)6	12	192643	192654	
PHG9_Scaffold_3033	78	p3	(ATC)5	15	208867	208881	potentially variable
PHG9_Scaffold_3037	2	p2	(TA)6	12	9264	9275	
PHG9_Scaffold_3040	8	p2	(AT)9	18	18897	18914	potentially variable
PHG9_Scaffold_3040	17	p2	(AT)6	12	34573	34584	
PHG9_Scaffold_3040	19	p2	(TA)6	12	37874	37885	
PHG9_Scaffold_3040	23	p2	(GA)7	14	45993	46006	potentially variable
PHG9_Scaffold_3040	24	p2	(AT)7	14	47345	47358	potentially

							variable
PHG9_Scaffold_3046	1	p2	(TA)7	14	3048	3061	potentially variable
PHG9_Scaffold_3047	2	p2	(AC)8	16	8631	8646	potentially variable
PHG9_Scaffold_3049	8	p2	(TA)6	12	15233	15244	
PHG9_Scaffold_3049	9	p2	(TA)7	14	15742	15755	potentially variable
PHG9_Scaffold_3049	10	p2	(TA)9	18	29275	29292	potentially variable
PHG9_Scaffold_3049	11	p2	(CT)8	16	32538	32553	potentially variable
PHG9_Scaffold_3062	2	p2	(AT)6	12	18006	18017	
PHG9_Scaffold_3062	3	p2	(TA)6	12	19745	19756	
PHG9_Scaffold_3065	5	p2	(AG)6	12	19822	19833	
PHG9_Scaffold_3066	2	p2	(TA)6	12	4966	4977	
PHG9_Scaffold_3066	6	p2	(AT)7	14	9871	9884	potentially variable
PHG9_Scaffold_3066	7	p2	(TA)6	12	12594	12605	
PHG9_Scaffold_3066	8	p2	(AT)9	18	14066	14083	potentially variable
PHG9_Scaffold_3066	9	p3	(AAT)5	15	21412	21426	potentially variable
PHG9_Scaffold_3066	10	p3	(AAT)5	15	24119	24133	potentially variable
PHG9_Scaffold_3066	13	p2	(TA)8	16	31682	31697	potentially variable
PHG9_Scaffold_3066	16	p2	(CT)6	12	50051	50062	
PHG9_Scaffold_3074	2	p2	(AT)8	16	9803	9818	potentially

							variable
PHG9_Scaffold_3075	1	p2	(CT)6	12	2491	2502	
PHG9_Scaffold_3075	2	p2	(AT)6	12	6203	6214	
PHG9_Scaffold_3075	9	p3	(CAA)5	15	30968	30982	potentially variable
PHG9_Scaffold_3075	11	p2	(TG)6	12	34616	34627	
PHG9_Scaffold_3075	17	p2	(GT)9	18	52933	52950	potentially variable
PHG9_Scaffold_3075	19	p2	(TG)6	12	57988	57999	
PHG9_Scaffold_3075	20	p3	(TAA)5	15	63563	63577	potentially variable
PHG9_Scaffold_3075	24	p2	(TA)9	18	70360	70377	potentially variable
PHG9_Scaffold_3080	4	p2	(AT)6	12	30180	30191	
PHG9_Scaffold_3080	13	p2	(GA)6	12	69596	69607	
PHG9_Scaffold_3084	4	p2	(AT)7	14	14404	14417	potentially variable
PHG9_Scaffold_3084	7	p3	(TCC)5	15	19575	19589	potentially variable
PHG9_Scaffold_3085	15	p3	(TTC)5	15	54267	54281	potentially variable
PHG9_Scaffold_3085	25	p3	(TAA)5	15	105174	105188	potentially variable
PHG9_Scaffold_3085	29	p3	(TAA)6	18	119884	119901	potentially variable
PHG9_Scaffold_3085	40	p3	(GGT)5	15	166735	166749	potentially variable
PHG9_Scaffold_3085	47	p3	(TAC)5	15	185757	185771	potentially variable

PHG9_Scaffold_3085	48	p2	(AG)6	12	194008	194019	
PHG9_Scaffold_3085	49	p3	(CTA)6	18	199644	199661	potentially variable
PHG9_Scaffold_3085	56	p2	(TA)6	12	214087	214098	
PHG9_Scaffold_3085	66	p2	(TC)6	12	233534	233545	
PHG9_Scaffold_3085	67	p3	(ATT)5	15	235390	235404	potentially variable
PHG9_Scaffold_3091	7	p3	(ATT)5	15	16704	16718	potentially variable
PHG9_Scaffold_3092	6	p3	(TTC)5	15	9227	9241	potentially variable
PHG9_Scaffold_3095	5	p3	(AAT)5	15	4290	4304	potentially variable
PHG9_Scaffold_3099	6	p2	(TA)7	14	12790	12803	potentially variable
PHG9_Scaffold_3101	2	p3	(TGA)5	15	2725	2739	potentially variable
PHG9_Scaffold_3108	1	p2	(CA)6	12	6878	6889	
PHG9_Scaffold_3110	2	p2	(TA)8	16	12880	12895	potentially variable
PHG9_Scaffold_3112	1	p3	(AAC)5	15	5194	5208	potentially variable
PHG9_Scaffold_3114	3	p2	(AG)7	14	5185	5198	potentially variable
PHG9_Scaffold_3114	8	p2	(TA)7	14	11920	11933	potentially variable
PHG9_Scaffold_3114	12	p2	(TA)7	14	18386	18399	potentially variable
PHG9_Scaffold_3114	14	p2	(TA)8	16	21274	21289	potentially variable

PHG9_Scaffold_3114	16	p2	(TA)7	14	27536	27549	potentially variable
PHG9_Scaffold_3114	25	p2	(CG)7	14	46408	46421	potentially variable
PHG9_Scaffold_3114	27	p2	(CA)6	12	51897	51908	
PHG9_Scaffold_3114	49	p3	(GAT)5	15	112710	112724	potentially variable
PHG9_Scaffold_3116	3	p2	(GA)6	12	6317	6328	
PHG9_Scaffold_3117	3	p2	(TA)6	12	55885	55896	
PHG9_Scaffold_3117	7	p2	(TA)8	16	69606	69621	potentially variable
PHG9_Scaffold_3118	5	p2	(TA)6	12	5854	5865	
PHG9_Scaffold_3118	7	p3	(CAA)5	15	7909	7923	potentially variable
PHG9_Scaffold_3120	1	p3	(AAG)5	15	432	446	potentially variable
PHG9_Scaffold_3121	2	p3	(CAT)5	15	4402	4416	potentially variable
PHG9_Scaffold_3126	2	p2	(TA)6	12	5725	5736	
PHG9_Scaffold_3126	26	p3	(AGA)6	18	80819	80836	potentially variable
PHG9_Scaffold_3127	7	p2	(AG)9	18	16564	16581	potentially variable
PHG9_Scaffold_3129	1	p3	(TAT)5	15	2499	2513	potentially variable
PHG9_Scaffold_3130	10	p2	(TA)7	14	43185	43198	potentially variable
PHG9_Scaffold_3131	1	p3	(GGT)5	15	1771	1785	potentially variable
PHG9_Scaffold_3134	3	p2	(CT)7	14	7867	7880	potentially

							variable
PHG9_Scaffold_3138	1	p2	(AT)6	12	2412	2423	
PHG9_Scaffold_3138	3	p2	(AT)7	14	10799	10812	potentially variable
PHG9_Scaffold_3138	5	p2	(TC)7	14	14705	14718	potentially variable
PHG9_Scaffold_3139	5	p2	(TG)6	12	2566	2577	
PHG9_Scaffold_3140	3	p3	(ATT)5	15	6636	6650	potentially variable
PHG9_Scaffold_3140	5	p3	(TCA)5	15	17201	17215	potentially variable
PHG9_Scaffold_3142	3	p2	(AT)6	12	7254	7265	
PHG9_Scaffold_3144	1	p2	(AT)6	12	3030	3041	
PHG9_Scaffold_3144	3	p2	(TA)6	12	11085	11096	
PHG9_Scaffold_3144	5	p2	(AG)6	12	21057	21068	
PHG9_Scaffold_3146	1	p2	(CT)6	12	537	548	
PHG9_Scaffold_3148	8	p3	(GTG)5	15	16405	16419	potentially variable
PHG9_Scaffold_3150	4	p3	(ATT)5	15	7414	7428	potentially variable
PHG9_Scaffold_3150	5	p2	(TA)6	12	9377	9388	
PHG9_Scaffold_3151	5	p2	(GA)6	12	8731	8742	
PHG9_Scaffold_3154	6	p2	(TA)6	12	23913	23924	
PHG9_Scaffold_3154	17	p3	(TTC)5	15	67400	67414	potentially variable
PHG9_Scaffold_3154	26	p2	(AG)6	12	88712	88723	
PHG9_Scaffold_3154	28	p2	(AT)6	12	94483	94494	
PHG9_Scaffold_3154	30	p2	(AT)7	14	95969	95982	potentially variable

PHG9_Scaffold_3154	32	p2	(GT)9	18	116739	116756	potentially variable
PHG9_Scaffold_3154	35	p2	(TC)6	12	120524	120535	
PHG9_Scaffold_3154	39	p2	(TA)6	12	141042	141053	
PHG9_Scaffold_3154	40	p2	(AT)8	16	143262	143277	potentially variable
PHG9_Scaffold_3154	42	p2	(TA)6	12	165229	165240	
PHG9_Scaffold_3154	43	p2	(GT)8	16	165745	165760	potentially variable
PHG9_Scaffold_3154	44	p2	(AG)6	12	172073	172084	
PHG9_Scaffold_3154	49	p3	(AAG)5	15	188349	188363	potentially variable
PHG9_Scaffold_3155	1	p3	(TTA)6	18	9	26	potentially variable
PHG9_Scaffold_3155	3	p2	(AT)8	16	2894	2909	potentially variable
PHG9_Scaffold_3158	4	p2	(AT)7	14	14094	14107	potentially variable
PHG9_Scaffold_3158	9	p2	(TC)6	12	28470	28481	
PHG9_Scaffold_3158	11	p3	(ATA)5	15	30555	30569	potentially variable
PHG9_Scaffold_3158	16	p2	(TA)8	16	42364	42379	potentially variable
PHG9_Scaffold_3159	5	p2	(AT)8	16	36472	36487	potentially variable
PHG9_Scaffold_3160	1	p2	(AT)6	12	1780	1791	
PHG9_Scaffold_3160	3	p2	(AT)9	18	9373	9390	potentially variable
PHG9_Scaffold_3162	2	p3	(AAC)5	15	9443	9457	potentially variable

PHG9_Scaffold_3162	4	p3	(AAT)5	15	12402	12416	potentially variable
PHG9_Scaffold_3166	2	p3	(GTG)5	15	758	772	potentially variable
PHG9_Scaffold_3169	1	p3	(AGC)5	15	2060	2074	potentially variable
PHG9_Scaffold_3171	1	p3	(CAC)5	15	3903	3917	potentially variable
PHG9_Scaffold_3172	2	p2	(AG)7	14	2640	2653	potentially variable
PHG9_Scaffold_3174	2	p2	(CT)8	16	10023	10038	potentially variable
PHG9_Scaffold_3174	9	p2	(AT)6	12	28028	28039	
PHG9_Scaffold_3174	13	p2	(TC)7	14	42963	42976	potentially variable
PHG9_Scaffold_3174	19	p3	(AGA)6	18	57637	57654	potentially variable
PHG9_Scaffold_3174	20	p2	(GA)6	12	57978	57989	
PHG9_Scaffold_3177	2	p3	(TGG)5	15	6376	6390	potentially variable
PHG9_Scaffold_3181	3	p3	(AAT)6	18	2273	2290	potentially variable
PHG9_Scaffold_3184	5	p3	(CAT)5	15	18213	18227	potentially variable
PHG9_Scaffold_3187	1	p2	(AT)6	12	480	491	
PHG9_Scaffold_3188	2	p2	(AT)8	16	32290	32305	potentially variable
PHG9_Scaffold_3191	1	p2	(GA)7	14	1400	1413	potentially variable
PHG9_Scaffold_3191	3	p3	(TAA)5	15	7182	7196	potentially

							variable
PHG9_Scaffold_3198	9	p2	(TA)8	16	32449	32464	potentially variable
PHG9_Scaffold_3198	10	p3	(TCA)5	15	34875	34889	potentially variable
PHG9_Scaffold_3198	15	p2	(AT)7	14	50816	50829	potentially variable
PHG9_Scaffold_3198	24	p2	(TA)6	12	74465	74476	
PHG9_Scaffold_3198	28	p2	(GA)7	14	82470	82483	potentially variable
PHG9_Scaffold_3198	30	p2	(AC)6	12	86880	86891	
PHG9_Scaffold_3199	1	p3	(GAA)5	15	1390	1404	potentially variable
PHG9_Scaffold_3200	4	p3	(AAT)6	18	15717	15734	potentially variable
PHG9_Scaffold_3200	5	p2	(AC)8	16	17902	17917	potentially variable
PHG9_Scaffold_3200	6	p2	(TA)8	16	18894	18909	potentially variable
PHG9_Scaffold_3200	10	p2	(TA)7	14	26473	26486	potentially variable
PHG9_Scaffold_3200	11	p2	(TA)7	14	26908	26921	potentially variable
PHG9_Scaffold_3200	12	p2	(AT)8	16	30015	30030	potentially variable
PHG9_Scaffold_3200	21	p2	(AT)7	14	37390	37403	potentially variable
PHG9_Scaffold_3200	22	p3	(ATA)5	15	40418	40432	potentially variable
PHG9_Scaffold_3200	23	p2	(AG)7	14	42336	42349	potentially

							variable
PHG9_Scaffold_3200	25	p2	(TA)6	12	45199	45210	
PHG9_Scaffold_3200	27	p6	(TGAAGG)5	30	46565	46594	Hypervariable
PHG9_Scaffold_3200	28	p2	(AT)6	12	47852	47863	
PHG9_Scaffold_3200	29	p2	(AT)6	12	48458	48469	
PHG9_Scaffold_3204	1	p2	(AT)6	12	3798	3809	
PHG9_Scaffold_3204	3	p3	(AAG)5	15	6429	6443	potentially variable
PHG9_Scaffold_3204	9	p2	(TA)7	14	18541	18554	potentially variable
PHG9_Scaffold_3204	11	p2	(TA)6	12	27201	27212	
PHG9_Scaffold_3204	12	p2	(TA)6	12	27365	27376	
PHG9_Scaffold_3205	5	p2	(CA)7	14	11907	11920	potentially variable
PHG9_Scaffold_3205	16	p3	(CTT)5	15	60677	60691	potentially variable
PHG9_Scaffold_3205	27	p2	(AG)6	12	109654	109665	
PHG9_Scaffold_3209	2	p3	(TTA)5	15	3499	3513	potentially variable
PHG9_Scaffold_3209	3	p2	(AT)6	12	5319	5330	
PHG9_Scaffold_3213	1	p2	(AT)8	16	272	287	potentially variable
PHG9_Scaffold_3213	3	p3	(AAG)5	15	1895	1909	potentially variable
PHG9_Scaffold_3213	5	p2	(AT)6	12	4553	4564	
PHG9_Scaffold_3213	6	p3	(GAA)5	15	5940	5954	potentially variable
PHG9_Scaffold_3213	12	p3	(AGT)6	18	26217	26234	potentially variable

PHG9_Scaffold_3213	13	p2	(TA)9	18	27293	27310	potentially variable
PHG9_Scaffold_3213	20	p2	(TA)6	12	39555	39566	
PHG9_Scaffold_3213	21	p3	(TAA)5	15	40685	40699	potentially variable
PHG9_Scaffold_3213	22	p3	(AGG)5	15	42409	42423	potentially variable
PHG9_Scaffold_3215	4	p2	(TA)8	16	6458	6473	potentially variable
PHG9_Scaffold_3218	2	p2	(CA)6	12	6459	6470	
PHG9_Scaffold_3219	3	p2	(AT)6	12	11369	11380	
PHG9_Scaffold_3220	2	p3	(TAA)5	15	1605	1619	potentially variable
PHG9_Scaffold_3220	7	p3	(TGC)5	15	19187	19201	potentially variable
PHG9_Scaffold_3220	20	p2	(AT)6	12	50108	50119	
PHG9_Scaffold_3220	25	p2	(TA)6	12	102859	102870	
PHG9_Scaffold_3220	29	p2	(TA)7	14	111088	111101	potentially variable
PHG9_Scaffold_3220	30	p2	(TA)9	18	115804	115821	potentially variable
PHG9_Scaffold_3220	33	p2	(AT)6	12	124445	124456	
PHG9_Scaffold_3222	4	p2	(TG)6	12	6252	6263	
PHG9_Scaffold_3224	1	p3	(TGA)5	15	1745	1759	potentially variable
PHG9_Scaffold_3224	13	p2	(AT)6	12	56734	56745	
PHG9_Scaffold_3225	5	p3	(ATA)5	15	5027	5041	potentially variable
PHG9_Scaffold_3225	6	p2	(AT)8	16	6074	6089	potentially

							variable
PHG9_Scaffold_3225	7	p2	(TA)6	12	6608	6619	
PHG9_Scaffold_3230	3	p3	(TCT)5	15	6645	6659	potentially variable
PHG9_Scaffold_3230	4	p3	(ATT)6	18	8209	8226	potentially variable
PHG9_Scaffold_3233	1	p3	(TTA)6	18	899	916	potentially variable
PHG9_Scaffold_3233	3	p2	(AT)6	12	3149	3160	
PHG9_Scaffold_3234	3	p2	(TA)6	12	7757	7768	
PHG9_Scaffold_3234	4	p2	(AG)9	18	9925	9942	potentially variable
PHG9_Scaffold_3234	5	p2	(GA)6	12	10716	10727	
PHG9_Scaffold_3236	1	p2	(TA)7	14	1798	1811	potentially variable
PHG9_Scaffold_3236	10	p2	(GA)6	12	19318	19329	
PHG9_Scaffold_3237	8	p2	(AT)7	14	19349	19362	potentially variable
PHG9_Scaffold_3238	4	p2	(AT)7	14	8112	8125	potentially variable
PHG9_Scaffold_3239	2	p2	(AT)6	12	1889	1900	
PHG9_Scaffold_3240	1	p2	(CA)8	16	1430	1445	potentially variable
PHG9_Scaffold_3240	11	p2	(TA)6	12	19712	19723	
PHG9_Scaffold_3240	12	p2	(AT)8	16	20238	20253	potentially variable
PHG9_Scaffold_3241	2	p2	(AT)6	12	492	503	
PHG9_Scaffold_3247	3	p2	(AT)6	12	2999	3010	
PHG9_Scaffold_3247	9	p2	(AT)6	12	12151	12162	

PHG9_Scaffold_3248	9	p2	(TA)6	12	114805	114816	
PHG9_Scaffold_3248	12	p2	(TA)6	12	121605	121616	
PHG9_Scaffold_3248	14	p2	(AG)7	14	141004	141017	potentially variable
PHG9_Scaffold_3251	5	p2	(AT)7	14	11452	11465	potentially variable
PHG9_Scaffold_3251	6	p3	(TTC)5	15	12275	12289	potentially variable
PHG9_Scaffold_3252	5	p3	(TAA)5	15	35453	35467	potentially variable
PHG9_Scaffold_3252	16	p2	(TA)9	18	65689	65706	potentially variable
PHG9_Scaffold_3252	18	p3	(TTA)5	15	71344	71358	potentially variable
PHG9_Scaffold_3252	21	p6	(TCCTAT)5	30	82091	82120	Hypervariable
PHG9_Scaffold_3253	1	p2	(TA)6	12	1242	1253	
PHG9_Scaffold_3253	3	p2	(GA)7	14	6301	6314	potentially variable
PHG9_Scaffold_3257	2	p2	(TA)7	14	4132	4145	potentially variable
PHG9_Scaffold_3257	3	p2	(TA)6	12	6432	6443	
PHG9_Scaffold_3257	4	p2	(TA)8	16	6805	6820	potentially variable
PHG9_Scaffold_3257	14	p2	(TA)7	14	48625	48638	potentially variable
PHG9_Scaffold_3257	16	p2	(TG)6	12	58094	58105	
PHG9_Scaffold_3257	17	p2	(AC)8	16	60845	60860	potentially variable
PHG9_Scaffold_3257	20	p2	(AG)9	18	67849	67866	potentially variable

PHG9_Scaffold_3261	16	p2	(TA)8	16	41569	41584	potentially variable
PHG9_Scaffold_3261	28	p3	(ATG)5	15	89019	89033	potentially variable
PHG9_Scaffold_3261	29	p3	(GAA)5	15	94045	94059	potentially variable
PHG9_Scaffold_3261	33	p2	(AT)8	16	98820	98835	potentially variable
PHG9_Scaffold_3262	1	p2	(AT)9	18	1700	1717	potentially variable
PHG9_Scaffold_3262	2	p2	(AT)6	12	6041	6052	
PHG9_Scaffold_3263	1	p2	(AT)7	14	1251	1264	potentially variable
PHG9_Scaffold_3264	2	p3	(AAG)5	15	4090	4104	potentially variable
PHG9_Scaffold_3266	2	p2	(AT)9	18	5325	5342	potentially variable
PHG9_Scaffold_3266	3	p2	(TA)8	16	5746	5761	potentially variable
PHG9_Scaffold_3267	3	p2	(AT)8	16	3186	3201	potentially variable
PHG9_Scaffold_3267	4	p2	(TA)8	16	6113	6128	potentially variable
PHG9_Scaffold_3267	8	p2	(AT)6	12	11892	11903	
PHG9_Scaffold_3273	1	p3	(ACA)5	15	7142	7156	potentially variable
PHG9_Scaffold_3275	2	p2	(TA)6	12	2412	2423	
PHG9_Scaffold_3276	1	p2	(TC)6	12	283	294	
PHG9_Scaffold_3276	2	p2	(TC)7	14	1531	1544	potentially variable

PHG9_Scaffold_3276	4	p2	(AT)7	14	3418	3431	potentially variable
PHG9_Scaffold_3280	1	p3	(AAT)5	15	390	404	potentially variable
PHG9_Scaffold_3281	2	p2	(AT)7	14	1101	1114	potentially variable
PHG9_Scaffold_3282	11	p2	(TA)9	18	23331	23348	potentially variable
PHG9_Scaffold_3282	12	p3	(TAA)5	15	24116	24130	potentially variable
PHG9_Scaffold_3282	15	p2	(CT)7	14	29702	29715	potentially variable
PHG9_Scaffold_3284	4	p3	(TGC)5	15	5317	5331	potentially variable
PHG9_Scaffold_3290	1	p3	(AAT)5	15	1402	1416	potentially variable
PHG9_Scaffold_3290	9	p2	(TA)6	12	8511	8522	
PHG9_Scaffold_3290	20	p2	(AT)6	12	24253	24264	
PHG9_Scaffold_3292	9	p3	(TTG)5	15	21311	21325	potentially variable
PHG9_Scaffold_3292	10	p3	(TGT)5	15	21459	21473	potentially variable
PHG9_Scaffold_3292	11	p2	(TA)6	12	21973	21984	
PHG9_Scaffold_3292	12	p3	(GTT)5	15	22280	22294	potentially variable
PHG9_Scaffold_3292	14	p2	(AT)8	16	24597	24612	potentially variable
PHG9_Scaffold_3292	16	p2	(TG)6	12	25948	25959	
PHG9_Scaffold_3292	23	p2	(AT)6	12	55800	55811	
PHG9_Scaffold_3293	4	p2	(TA)7	14	17773	17786	potentially

							variable
PHG9_Scaffold_3293	7	p3	(TCT)5	15	36390	36404	potentially variable
PHG9_Scaffold_3293	12	p3	(GTG)5	15	42477	42491	potentially variable
PHG9_Scaffold_3293	15	p2	(TC)7	14	57949	57962	potentially variable
PHG9_Scaffold_3293	22	p2	(AT)9	18	73012	73029	potentially variable
PHG9_Scaffold_3293	31	p2	(AT)6	12	94369	94380	
PHG9_Scaffold_3293	38	p3	(TGA)5	15	110885	110899	potentially variable
PHG9_Scaffold_3293	51	p2	(TG)6	12	148733	148744	
PHG9_Scaffold_3293	53	p2	(TA)9	18	149647	149664	potentially variable
PHG9_Scaffold_3293	54	p3	(TAT)5	15	150314	150328	potentially variable
PHG9_Scaffold_3293	57	p2	(AT)9	18	159875	159892	potentially variable
PHG9_Scaffold_3293	58	p2	(TC)6	12	163480	163491	
PHG9_Scaffold_3293	65	p2	(TA)6	12	196669	196680	
PHG9_Scaffold_3295	2	p2	(TA)9	18	15369	15386	potentially variable
PHG9_Scaffold_3295	9	p3	(AAG)6	18	42957	42974	potentially variable
PHG9_Scaffold_3295	11	p2	(AT)8	16	43825	43840	potentially variable
PHG9_Scaffold_3297	2	p2	(TA)6	12	21141	21152	
PHG9_Scaffold_3298	1	p2	(CT)9	18	9569	9586	potentially variable

PHG9_Scaffold_3298	6	p2	(CA)8	16	21251	21266	potentially variable
PHG9_Scaffold_3298	10	p2	(AT)7	14	40642	40655	potentially variable
PHG9_Scaffold_3298	16	p3	(ACA)5	15	58879	58893	potentially variable
PHG9_Scaffold_3298	17	p2	(AT)6	12	63321	63332	
PHG9_Scaffold_3298	19	p2	(AT)7	14	68507	68520	potentially variable
PHG9_Scaffold_3298	20	p2	(TA)6	12	69560	69571	
PHG9_Scaffold_3298	30	p2	(TA)6	12	91709	91720	
PHG9_Scaffold_3304	3	p3	(AAT)5	15	10286	10300	potentially variable
PHG9_Scaffold_3304	14	p2	(GT)8	16	68667	68682	potentially variable
PHG9_Scaffold_3304	15	p2	(AT)6	12	77066	77077	
PHG9_Scaffold_3304	21	p2	(CA)6	12	98711	98722	
PHG9_Scaffold_3306	1	p2	(AT)7	14	1872	1885	potentially variable
PHG9_Scaffold_3306	3	p2	(TA)9	18	5195	5212	potentially variable
PHG9_Scaffold_3306	4	p2	(TA)6	12	8606	8617	
PHG9_Scaffold_3306	6	p2	(TA)8	16	11219	11234	potentially variable
PHG9_Scaffold_3306	7	p3	(TTC)6	18	11863	11880	potentially variable
PHG9_Scaffold_3307	4	p3	(GAA)6	18	13498	13515	potentially variable
PHG9_Scaffold_3307	5	p2	(AT)7	14	22433	22446	potentially variable

PHG9_Scaffold_3308	1	p3	(TTA)6	18	82	99	potentially variable
PHG9_Scaffold_3314	6	p3	(AAT)6	18	5718	5735	potentially variable
PHG9_Scaffold_3314	18	p3	(TAA)5	15	37284	37298	potentially variable
PHG9_Scaffold_3314	30	p2	(TA)6	12	64409	64420	
PHG9_Scaffold_3314	35	p2	(TC)8	16	75280	75295	potentially variable
PHG9_Scaffold_3314	41	p2	(AT)8	16	86525	86540	potentially variable
PHG9_Scaffold_3315	17	p2	(TA)8	16	48674	48689	potentially variable
PHG9_Scaffold_3315	20	p2	(AT)8	16	52071	52086	potentially variable
PHG9_Scaffold_3315	22	p2	(TA)9	18	56383	56400	potentially variable
PHG9_Scaffold_3315	23	p2	(TC)6	12	57226	57237	
PHG9_Scaffold_3315	26	p2	(TA)7	14	82841	82854	potentially variable
PHG9_Scaffold_3315	27	p2	(CT)6	12	86168	86179	
PHG9_Scaffold_3315	29	p3	(TAA)6	18	89433	89450	potentially variable
PHG9_Scaffold_3316	8	p2	(TA)7	14	15137	15150	potentially variable
PHG9_Scaffold_3318	1	p2	(AT)6	12	5985	5996	
PHG9_Scaffold_3319	7	p2	(TA)7	14	17698	17711	potentially variable
PHG9_Scaffold_3320	1	p2	(AT)6	12	32191	32202	
PHG9_Scaffold_3320	2	p2	(TA)8	16	37296	37311	potentially

							variable
PHG9_Scaffold_3323	2	p2	(AT)6	12	2996	3007	
PHG9_Scaffold_3324	2	p2	(TA)6	12	4198	4209	
PHG9_Scaffold_3324	4	p3	(ATT)5	15	11460	11474	potentially variable
PHG9_Scaffold_3324	8	p2	(AT)6	12	21801	21812	
PHG9_Scaffold_3328	1	p2	(TA)6	12	1714	1725	
PHG9_Scaffold_3328	2	p2	(TA)6	12	4079	4090	
PHG9_Scaffold_3328	4	p2	(AT)6	12	7604	7615	
PHG9_Scaffold_3330	1	p3	(TGG)5	15	2921	2935	potentially variable
PHG9_Scaffold_3331	2	p3	(TAA)5	15	7067	7081	potentially variable
PHG9_Scaffold_3333	1	p2	(AT)6	12	508	519	
PHG9_Scaffold_3334	1	p3	(TTC)5	15	1006	1020	potentially variable
PHG9_Scaffold_3335	8	p3	(TAA)5	15	32257	32271	potentially variable
PHG9_Scaffold_3335	11	p3	(ATA)5	15	56065	56079	potentially variable
PHG9_Scaffold_3335	12	p2	(TA)9	18	56535	56552	potentially variable
PHG9_Scaffold_3335	13	p2	(TA)6	12	65530	65541	
PHG9_Scaffold_3340	3	p2	(TC)6	12	10726	10737	
PHG9_Scaffold_3340	4	p2	(AT)7	14	11509	11522	potentially variable
PHG9_Scaffold_3340	6	p2	(AG)8	16	17690	17705	potentially variable
PHG9_Scaffold_3342	1	p3	(TGA)6	18	11671	11688	potentially

							variable
PHG9_Scaffold_3344	3	p2	(AT)6	12	3123	3134	
PHG9_Scaffold_3347	3	p3	(ATA)5	15	5051	5065	potentially variable
PHG9_Scaffold_3350	3	p3	(ACA)6	18	34618	34635	potentially variable
PHG9_Scaffold_3355	9	p2	(AG)6	12	15921	15932	
PHG9_Scaffold_3355	11	p2	(TA)9	18	18793	18810	potentially variable
PHG9_Scaffold_3364	8	p2	(TA)7	14	59234	59247	potentially variable
PHG9_Scaffold_3364	10	p2	(TA)6	12	77958	77969	
PHG9_Scaffold_3373	1	p2	(CA)7	14	2389	2402	potentially variable
PHG9_Scaffold_3375	1	p2	(AT)6	12	1968	1979	
PHG9_Scaffold_3381	4	p2	(TA)7	14	2437	2450	potentially variable
PHG9_Scaffold_3381	6	p2	(AT)7	14	4865	4878	potentially variable
PHG9_Scaffold_3384	3	p2	(TA)6	12	4169	4180	
PHG9_Scaffold_3386	4	p3	(AAG)6	18	10411	10428	potentially variable
PHG9_Scaffold_3386	11	p3	(ATT)5	15	28543	28557	potentially variable
PHG9_Scaffold_3386	12	p3	(ATC)5	15	30681	30695	potentially variable
PHG9_Scaffold_3386	20	p3	(TAA)5	15	51901	51915	potentially variable
PHG9_Scaffold_3386	34	p3	(GTT)5	15	83627	83641	potentially variable

PHG9_Scaffold_3386	35	p2	(AT)6	12	84207	84218	
PHG9_Scaffold_3386	47	p2	(AT)6	12	111241	111252	
PHG9_Scaffold_3387	6	p2	(CT)8	16	44215	44230	potentially variable
PHG9_Scaffold_3393	4	p3	(CAT)6	18	5492	5509	potentially variable
PHG9_Scaffold_3396	1	p2	(AT)8	16	10290	10305	potentially variable
PHG9_Scaffold_3396	2	p2	(AT)9	18	10668	10685	potentially variable
PHG9_Scaffold_3399	3	p2	(AT)6	12	719	730	
PHG9_Scaffold_3399	18	p3	(TGG)5	15	37681	37695	potentially variable
PHG9_Scaffold_3400	4	p2	(TA)6	12	19951	19962	
PHG9_Scaffold_3400	5	p2	(TA)7	14	23726	23739	potentially variable
PHG9_Scaffold_3401	2	p2	(TA)8	16	5399	5414	potentially variable
PHG9_Scaffold_3403	8	p2	(TA)6	12	17485	17496	
PHG9_Scaffold_3403	9	p6	(CAAATG)5	30	19211	19240	Hypervariable
PHG9_Scaffold_3405	4	p2	(AG)7	14	8753	8766	potentially variable
PHG9_Scaffold_3407	11	p2	(TA)7	14	35568	35581	potentially variable
PHG9_Scaffold_3407	13	p2	(TA)7	14	42758	42771	potentially variable
PHG9_Scaffold_3407	18	p3	(ACC)5	15	48903	48917	potentially variable
PHG9_Scaffold_3410	1	p3	(ATA)5	15	13616	13630	potentially variable

PHG9_Scaffold_3413	1	p2	(TA)8	16	7053	7068	potentially variable
PHG9_Scaffold_3413	11	p2	(TA)8	16	33822	33837	potentially variable
PHG9_Scaffold_3413	13	p2	(AT)6	12	39774	39785	
PHG9_Scaffold_3413	18	p2	(AT)7	14	65773	65786	potentially variable
PHG9_Scaffold_3413	22	p3	(AAC)5	15	75225	75239	potentially variable
PHG9_Scaffold_3413	23	p2	(AT)8	16	75834	75849	potentially variable
PHG9_Scaffold_3413	28	p3	(ATC)6	18	94860	94877	potentially variable
PHG9_Scaffold_3413	30	p2	(CA)9	18	160882	160899	potentially variable
PHG9_Scaffold_3413	36	p2	(AT)8	16	182720	182735	potentially variable
PHG9_Scaffold_3413	37	p2	(AT)9	18	183494	183511	potentially variable
PHG9_Scaffold_3413	38	p2	(TA)8	16	194445	194460	potentially variable
PHG9_Scaffold_3413	43	p3	(ATA)5	15	213600	213614	potentially variable
PHG9_Scaffold_3415	5	p3	(AAT)5	15	9131	9145	potentially variable
PHG9_Scaffold_3421	1	p2	(TA)6	12	1826	1837	
PHG9_Scaffold_3421	3	p2	(TA)8	16	4449	4464	potentially variable
PHG9_Scaffold_3421	4	p3	(TTC)6	18	5093	5110	potentially variable

PHG9_Scaffold_3421	5	p2	(TA)7	14	7770	7783	potentially variable
PHG9_Scaffold_3423	2	p3	(AAG)6	18	7973	7990	potentially variable
PHG9_Scaffold_3423	5	p2	(AT)7	14	12298	12311	potentially variable
PHG9_Scaffold_3425	2	p2	(AT)8	16	12229	12244	potentially variable
PHG9_Scaffold_3425	3	p2	(AG)7	14	14440	14453	potentially variable
PHG9_Scaffold_3425	4	p2	(AG)7	14	19187	19200	potentially variable
PHG9_Scaffold_3425	11	p2	(TA)6	12	31578	31589	
PHG9_Scaffold_3425	12	p2	(AT)7	14	33032	33045	potentially variable
PHG9_Scaffold_3425	13	p2	(AT)8	16	45774	45789	potentially variable
PHG9_Scaffold_3426	6	p2	(TC)8	16	24312	24327	potentially variable
PHG9_Scaffold_3426	8	p2	(TA)6	12	33306	33317	
PHG9_Scaffold_3426	20	p2	(TC)6	12	78930	78941	
PHG9_Scaffold_3426	21	p3	(ACA)5	15	79155	79169	potentially variable
PHG9_Scaffold_3426	28	p2	(TA)7	14	113564	113577	potentially variable
PHG9_Scaffold_3426	31	p2	(GT)6	12	125182	125193	
PHG9_Scaffold_3427	1	p3	(AGA)6	18	616	633	potentially variable
PHG9_Scaffold_3428	2	p2	(TA)6	12	10514	10525	
PHG9_Scaffold_3429	1	p3	(TTA)6	18	281	298	potentially

							variable
PHG9_Scaffold_3429	2	p2	(TA)6	12	1352	1363	
PHG9_Scaffold_3429	6	p2	(AT)6	12	14156	14167	
PHG9_Scaffold_3429	7	p2	(AT)6	12	15835	15846	
PHG9_Scaffold_3429	10	p2	(CT)6	12	19519	19530	
PHG9_Scaffold_3436	1	p2	(AT)9	18	1940	1957	potentially variable
PHG9_Scaffold_3436	2	p2	(TA)8	16	6140	6155	potentially variable
PHG9_Scaffold_3442	1	p2	(TA)6	12	738	749	
PHG9_Scaffold_3442	3	p2	(TA)7	14	2684	2697	potentially variable
PHG9_Scaffold_3442	6	p2	(TA)6	12	14926	14937	
PHG9_Scaffold_3445	3	p2	(AT)9	18	4064	4081	potentially variable
PHG9_Scaffold_3445	5	p2	(AT)6	12	5198	5209	
PHG9_Scaffold_3446	1	p2	(CA)6	12	3585	3596	
PHG9_Scaffold_3448	7	p3	(TAA)6	18	21013	21030	potentially variable
PHG9_Scaffold_3452	1	p3	(CTT)5	15	7923	7937	potentially variable
PHG9_Scaffold_3452	2	p2	(AT)8	16	8827	8842	potentially variable
PHG9_Scaffold_3458	16	p2	(AC)6	12	51450	51461	
PHG9_Scaffold_3458	17	p3	(TTA)5	15	52468	52482	potentially variable
PHG9_Scaffold_3458	20	p2	(TC)6	12	64035	64046	
PHG9_Scaffold_3459	1	p2	(AT)6	12	3348	3359	
PHG9_Scaffold_3459	5	p3	(CAT)5	15	34136	34150	potentially

							variable
PHG9_Scaffold_3459	11	p3	(AAT)6	18	68994	69011	potentially variable
PHG9_Scaffold_3459	15	p3	(TGA)6	18	75695	75712	potentially variable
PHG9_Scaffold_3463	3	p3	(AAT)5	15	3130	3144	potentially variable
PHG9_Scaffold_3463	4	p2	(AT)6	12	3434	3445	
PHG9_Scaffold_3464	8	p2	(AG)6	12	24574	24585	
PHG9_Scaffold_3465	9	p3	(TTG)5	15	13129	13143	potentially variable
PHG9_Scaffold_3465	11	p2	(TA)7	14	19428	19441	potentially variable
PHG9_Scaffold_3469	7	p2	(TA)8	16	25801	25816	potentially variable
PHG9_Scaffold_3470	7	p2	(AT)7	14	18440	18453	potentially variable
PHG9_Scaffold_3475	13	p2	(AT)9	18	31303	31320	potentially variable
PHG9_Scaffold_3475	15	p2	(TA)6	12	41123	41134	
PHG9_Scaffold_3475	16	p2	(TA)6	12	41689	41700	
PHG9_Scaffold_3475	17	p2	(AT)6	12	43754	43765	
PHG9_Scaffold_3475	20	p2	(TA)8	16	64793	64808	potentially variable
PHG9_Scaffold_3475	22	p2	(AT)7	14	68835	68848	potentially variable
PHG9_Scaffold_3475	25	p2	(AT)9	18	76462	76479	potentially variable
PHG9_Scaffold_3478	1	p2	(AT)9	18	7483	7500	potentially variable

PHG9_Scaffold_3478	2	p2	(AT)7	14	8127	8140	potentially variable
PHG9_Scaffold_3480	2	p2	(TA)9	18	7514	7531	potentially variable
PHG9_Scaffold_3485	5	p3	(AAT)6	18	16385	16402	potentially variable
PHG9_Scaffold_3487	5	p2	(AT)6	12	13585	13596	
PHG9_Scaffold_3489	1	p2	(TA)7	14	487	500	potentially variable
PHG9_Scaffold_3490	1	p2	(TA)7	14	181	194	potentially variable
PHG9_Scaffold_3490	3	p2	(AT)6	12	10279	10290	
PHG9_Scaffold_3492	3	p2	(TA)8	16	5631	5646	potentially variable
PHG9_Scaffold_3492	6	p2	(TC)6	12	12814	12825	
PHG9_Scaffold_3492	11	p3	(ATC)5	15	28082	28096	potentially variable
PHG9_Scaffold_3492	21	p3	(TTA)5	15	52375	52389	potentially variable
PHG9_Scaffold_3492	24	p3	(TAA)5	15	63090	63104	potentially variable
PHG9_Scaffold_3492	36	p2	(TA)7	14	109299	109312	potentially variable
PHG9_Scaffold_3492	38	p3	(TAA)6	18	116154	116171	potentially variable
PHG9_Scaffold_3494	1	p3	(TGT)5	15	463	477	potentially variable
PHG9_Scaffold_3494	3	p2	(TA)6	12	2523	2534	
PHG9_Scaffold_3499	1	p2	(AT)6	12	1083	1094	
PHG9_Scaffold_3506	1	p2	(AG)8	16	4774	4789	potentially

							variable
PHG9_Scaffold_3507	4	p2	(TA)6	12	13439	13450	
PHG9_Scaffold_3509	1	p2	(AG)7	14	287	300	potentially variable
PHG9_Scaffold_3509	2	p2	(TA)6	12	4754	4765	
PHG9_Scaffold_3509	3	p3	(AAT)5	15	5195	5209	potentially variable
PHG9_Scaffold_3509	4	p3	(AAT)5	15	5366	5380	potentially variable
PHG9_Scaffold_3522	1	p2	(TA)6	12	23918	23929	
PHG9_Scaffold_3522	3	p2	(AT)6	12	26394	26405	
PHG9_Scaffold_3526	1	p2	(CT)7	14	443	456	potentially variable
PHG9_Scaffold_3530	1	p3	(TTA)5	15	285	299	potentially variable
PHG9_Scaffold_3533	1	p2	(AT)7	14	19	32	potentially variable
PHG9_Scaffold_3534	2	p2	(AT)6	12	1297	1308	
PHG9_Scaffold_3537	1	p3	(CAC)5	15	1104	1118	potentially variable
PHG9_Scaffold_3542	2	p3	(ATA)5	15	3055	3069	potentially variable
PHG9_Scaffold_3546	9	p2	(TA)6	12	36287	36298	
PHG9_Scaffold_3546	18	p2	(GA)7	14	61915	61928	potentially variable
PHG9_Scaffold_3546	19	p2	(TA)8	16	68814	68829	potentially variable
PHG9_Scaffold_3552	6	p2	(AT)8	16	9066	9081	potentially variable

PHG9_Scaffold_3555	2	p2	(TC)7	14	4469	4482	potentially variable
PHG9_Scaffold_3557	8	p2	(GA)7	14	120864	120877	potentially variable
PHG9_Scaffold_3558	8	p3	(TCA)5	15	34048	34062	potentially variable
PHG9_Scaffold_3558	12	p2	(AT)7	14	42161	42174	potentially variable
PHG9_Scaffold_3558	14	p2	(GA)7	14	46476	46489	potentially variable
PHG9_Scaffold_3558	19	p2	(AG)6	12	50961	50972	
PHG9_Scaffold_3558	25	p2	(AT)7	14	59173	59186	potentially variable
PHG9_Scaffold_3558	30	p3	(GAA)5	15	87439	87453	potentially variable
PHG9_Scaffold_3558	33	p2	(AT)6	12	99221	99232	
PHG9_Scaffold_3558	35	p2	(TG)6	12	102821	102832	
PHG9_Scaffold_3560	6	p3	(AAG)5	15	11773	11787	potentially variable
PHG9_Scaffold_3560	7	p3	(AAT)5	15	12202	12216	potentially variable
PHG9_Scaffold_3561	2	p2	(AG)6	12	4166	4177	
PHG9_Scaffold_3561	6	p3	(GAT)5	15	18286	18300	potentially variable
PHG9_Scaffold_3561	13	p2	(TC)7	14	37958	37971	potentially variable
PHG9_Scaffold_3561	18	p3	(GAT)6	18	58469	58486	potentially variable
PHG9_Scaffold_3561	20	p2	(AG)6	12	66123	66134	
PHG9_Scaffold_3561	21	p3	(TGA)5	15	67937	67951	potentially

							variable
PHG9_Scaffold_3561	22	p3	(CTT)6	18	70667	70684	potentially variable
PHG9_Scaffold_3561	23	p2	(TC)7	14	71804	71817	potentially variable
PHG9_Scaffold_3561	24	p6	(CCCTAC)6	36	75645	75680	Hypervariable
PHG9_Scaffold_3562	1	p2	(CT)7	14	798	811	potentially variable
PHG9_Scaffold_3562	2	p2	(TA)7	14	3277	3290	potentially variable
PHG9_Scaffold_3566	2	p2	(AT)6	12	2261	2272	
PHG9_Scaffold_3569	1	p2	(AT)7	14	5339	5352	potentially variable
PHG9_Scaffold_3570	1	p2	(AT)6	12	1453	1464	
PHG9_Scaffold_3570	4	p2	(AT)6	12	13509	13520	
PHG9_Scaffold_3570	18	p3	(TAA)5	15	50162	50176	potentially variable
PHG9_Scaffold_3577	2	p2	(TA)7	14	7611	7624	potentially variable
PHG9_Scaffold_3577	3	p2	(TA)9	18	11053	11070	potentially variable
PHG9_Scaffold_3577	5	p2	(CT)6	12	16216	16227	
PHG9_Scaffold_3577	14	p2	(AT)7	14	52250	52263	potentially variable
PHG9_Scaffold_3578	2	p2	(CG)6	12	3367	3378	
PHG9_Scaffold_3578	4	p2	(TA)7	14	9186	9199	potentially variable
PHG9_Scaffold_3582	2	p2	(AT)7	14	3091	3104	potentially variable

PHG9_Scaffold_3582	3	p2	(CT)8	16	6243	6258	potentially variable
PHG9_Scaffold_3583	4	p2	(TA)6	12	5420	5431	
PHG9_Scaffold_3583	8	p3	(CTT)6	18	22815	22832	potentially variable
PHG9_Scaffold_3583	9	p2	(TA)6	12	23666	23677	
PHG9_Scaffold_3583	12	p2	(AT)6	12	33236	33247	
PHG9_Scaffold_3583	17	p2	(TA)9	18	42806	42823	potentially variable
PHG9_Scaffold_3583	27	p3	(CTT)6	18	58907	58924	potentially variable
PHG9_Scaffold_3583	28	p2	(AT)8	16	60140	60155	potentially variable
PHG9_Scaffold_3583	36	p3	(AAG)5	15	76773	76787	potentially variable
PHG9_Scaffold_3583	39	p2	(AG)7	14	90440	90453	potentially variable
PHG9_Scaffold_3584	6	p3	(TAT)5	15	27811	27825	potentially variable
PHG9_Scaffold_3584	11	p2	(GA)7	14	45838	45851	potentially variable
PHG9_Scaffold_3584	15	p2	(AT)6	12	63062	63073	
PHG9_Scaffold_3584	17	p3	(ATT)5	15	70810	70824	potentially variable
PHG9_Scaffold_3584	22	p3	(TTA)5	15	84340	84354	potentially variable
PHG9_Scaffold_3584	27	p2	(TC)8	16	108681	108696	potentially variable
PHG9_Scaffold_3588	1	p2	(TA)7	14	456	469	potentially variable

PHG9_Scaffold_3589	2	p2	(TA)7	14	1094	1107	potentially variable
PHG9_Scaffold_3592	1	p3	(AAG)5	15	723	737	potentially variable
PHG9_Scaffold_3594	1	p3	(ATA)5	15	1145	1159	potentially variable
PHG9_Scaffold_3594	9	p2	(AT)7	14	22731	22744	potentially variable
PHG9_Scaffold_3594	11	p3	(GAA)6	18	26160	26177	potentially variable
PHG9_Scaffold_3594	20	p3	(AAT)5	15	40901	40915	potentially variable
PHG9_Scaffold_3595	6	p2	(AT)6	12	16363	16374	
PHG9_Scaffold_3597	1	p3	(AAG)5	15	376	390	potentially variable
PHG9_Scaffold_3597	5	p2	(AT)7	14	3823	3836	potentially variable
PHG9_Scaffold_3598	6	p2	(AT)7	14	13991	14004	potentially variable
PHG9_Scaffold_3600	2	p3	(TAT)5	15	1913	1927	potentially variable
PHG9_Scaffold_3600	9	p2	(TA)7	14	9562	9575	potentially variable
PHG9_Scaffold_3600	10	p2	(TA)9	18	10256	10273	potentially variable
PHG9_Scaffold_3600	11	p3	(GGT)6	18	11805	11822	potentially variable
PHG9_Scaffold_3600	12	p3	(ATA)6	18	13379	13396	potentially variable
PHG9_Scaffold_3601	3	p2	(AT)9	18	3015	3032	potentially

							variable
PHG9_Scaffold_3601	9	p2	(CT)6	12	20930	20941	
PHG9_Scaffold_3607	2	p2	(CT)6	12	9415	9426	
PHG9_Scaffold_3612	3	p2	(AT)8	16	4877	4892	potentially variable
PHG9_Scaffold_3617	11	p3	(AAG)6	18	41620	41637	potentially variable
PHG9_Scaffold_3617	15	p2	(TA)7	14	48569	48582	potentially variable
PHG9_Scaffold_3617	16	p3	(TAA)5	15	50636	50650	potentially variable
PHG9_Scaffold_3617	17	p2	(TA)7	14	59539	59552	potentially variable
PHG9_Scaffold_3618	1	p3	(ATT)5	15	7255	7269	potentially variable
PHG9_Scaffold_3621	2	p2	(AT)8	16	3664	3679	potentially variable
PHG9_Scaffold_3624	3	p3	(TTA)5	15	5797	5811	potentially variable
PHG9_Scaffold_3624	5	p2	(TA)8	16	19172	19187	potentially variable
PHG9_Scaffold_3629	2	p2	(CA)6	12	5269	5280	
PHG9_Scaffold_3636	1	p2	(GA)6	12	45	56	
PHG9_Scaffold_3637	1	p2	(TA)9	18	55	72	potentially variable
PHG9_Scaffold_3637	2	p6	(AAATCA)5	30	2481	2510	Hypervariable
PHG9_Scaffold_3645	2	p2	(TA)6	12	6310	6321	
PHG9_Scaffold_3645	5	p2	(TA)6	12	12984	12995	
PHG9_Scaffold_3651	1	p3	(AAT)5	15	2498	2512	potentially

							variable
PHG9_Scaffold_3653	1	p2	(TG)6	12	16176	16187	
PHG9_Scaffold_3655	6	p2	(TA)6	12	21520	21531	
PHG9_Scaffold_3655	7	p2	(TC)6	12	22305	22316	
PHG9_Scaffold_3655	10	p2	(GT)6	12	31017	31028	
PHG9_Scaffold_3655	11	p3	(TAA)5	15	32038	32052	potentially variable
PHG9_Scaffold_3655	16	p2	(TA)8	16	38394	38409	potentially variable
PHG9_Scaffold_3655	20	p3	(GAT)5	15	47702	47716	potentially variable
PHG9_Scaffold_3655	30	p2	(AT)6	12	84506	84517	
PHG9_Scaffold_3655	31	p2	(TG)6	12	88221	88232	
PHG9_Scaffold_3655	34	p2	(AT)6	12	95208	95219	
PHG9_Scaffold_3655	45	p2	(CT)7	14	123793	123806	potentially variable
PHG9_Scaffold_3655	46	p2	(TA)8	16	124018	124033	potentially variable
PHG9_Scaffold_3655	48	p2	(CA)8	16	126420	126435	potentially variable
PHG9_Scaffold_3655	57	p2	(AT)6	12	161897	161908	
PHG9_Scaffold_3655	58	p3	(CTT)5	15	167810	167824	potentially variable
PHG9_Scaffold_3655	74	p2	(AT)7	14	211616	211629	potentially variable
PHG9_Scaffold_3655	75	p3	(GTT)5	15	212243	212257	potentially variable
PHG9_Scaffold_3655	76	p2	(AT)9	18	213152	213169	potentially variable

PHG9_Scaffold_3655	81	p2	(TC)6	12	221113	221124	
PHG9_Scaffold_3655	82	p2	(TC)6	12	222409	222420	
PHG9_Scaffold_3655	83	p2	(TC)7	14	225134	225147	potentially variable
PHG9_Scaffold_3655	85	p2	(CA)6	12	229386	229397	
PHG9_Scaffold_3655	89	p2	(AG)7	14	240857	240870	potentially variable
PHG9_Scaffold_3661	3	p2	(CA)7	14	1686	1699	potentially variable
PHG9_Scaffold_3661	11	p2	(AT)6	12	13018	13029	
PHG9_Scaffold_3661	12	p2	(CT)7	14	13663	13676	potentially variable
PHG9_Scaffold_3661	13	p2	(AC)7	14	13810	13823	potentially variable
PHG9_Scaffold_3661	14	p3	(GAA)5	15	21056	21070	potentially variable
PHG9_Scaffold_3661	19	p2	(AT)6	12	46195	46206	
PHG9_Scaffold_3668	3	p2	(AT)6	12	5470	5481	
PHG9_Scaffold_3669	1	p3	(CCA)6	18	5945	5962	potentially variable
PHG9_Scaffold_3671	1	p2	(CA)6	12	2227	2238	
PHG9_Scaffold_3671	5	p2	(GA)6	12	11756	11767	
PHG9_Scaffold_3672	1	p3	(TGG)6	18	657	674	potentially variable
PHG9_Scaffold_3672	4	p2	(AT)8	16	5226	5241	potentially variable
PHG9_Scaffold_3677	3	p3	(TTC)5	15	4398	4412	potentially variable
PHG9_Scaffold_3679	4	p3	(GGT)5	15	12862	12876	potentially

							variable
PHG9_Scaffold_3680	2	p2	(AT)6	12	3933	3944	
PHG9_Scaffold_3682	5	p2	(AG)6	12	9426	9437	
PHG9_Scaffold_3683	5	p2	(TC)6	12	6704	6715	
PHG9_Scaffold_3683	6	p2	(CT)6	12	15215	15226	
PHG9_Scaffold_3686	2	p3	(TTC)5	15	8660	8674	potentially variable
PHG9_Scaffold_3686	8	p2	(AT)6	12	40269	40280	
PHG9_Scaffold_3688	2	p2	(TA)6	12	2675	2686	
PHG9_Scaffold_3689	2	p2	(AG)6	12	7695	7706	
PHG9_Scaffold_3693	2	p2	(AG)9	18	3966	3983	potentially variable
PHG9_Scaffold_3694	2	p2	(AT)7	14	1637	1650	potentially variable
PHG9_Scaffold_3694	3	p2	(AT)7	14	2628	2641	potentially variable
PHG9_Scaffold_3694	11	p2	(TA)6	12	59844	59855	
PHG9_Scaffold_3698	2	p2	(TA)7	14	2000	2013	potentially variable
PHG9_Scaffold_3701	2	p3	(CCA)5	15	8399	8413	potentially variable
PHG9_Scaffold_3706	4	p2	(AT)6	12	8237	8248	
PHG9_Scaffold_3706	5	p2	(TA)8	16	8513	8528	potentially variable
PHG9_Scaffold_3707	1	p2	(AT)7	14	2012	2025	potentially variable
PHG9_Scaffold_3710	2	p2	(TA)6	12	2502	2513	
PHG9_Scaffold_3710	4	p3	(TTA)6	18	6440	6457	potentially variable

PHG9_Scaffold_3712	1	p2	(TG)7	14	3672	3685	potentially variable
PHG9_Scaffold_3712	9	p2	(AT)8	16	40406	40421	potentially variable
PHG9_Scaffold_3714	1	p3	(CAA)5	15	1260	1274	potentially variable
PHG9_Scaffold_3718	3	p3	(AAT)6	18	5860	5877	potentially variable
PHG9_Scaffold_3719	1	p2	(AT)6	12	3008	3019	
PHG9_Scaffold_3724	4	p2	(GA)6	12	21402	21413	
PHG9_Scaffold_3724	14	p2	(TA)6	12	115158	115169	
PHG9_Scaffold_3725	2	p2	(TA)6	12	557	568	
PHG9_Scaffold_3725	4	p2	(TA)8	16	2305	2320	potentially variable
PHG9_Scaffold_3726	1	p2	(AT)8	16	2585	2600	potentially variable
PHG9_Scaffold_3730	3	p2	(GA)6	12	4685	4696	
PHG9_Scaffold_3731	8	p2	(AT)7	14	24838	24851	potentially variable
PHG9_Scaffold_3731	12	p2	(AT)6	12	31607	31618	
PHG9_Scaffold_3732	3	p3	(GAT)5	15	4709	4723	potentially variable
PHG9_Scaffold_3734	3	p3	(AAT)5	15	2333	2347	potentially variable
PHG9_Scaffold_3737	1	p2	(TA)6	12	1777	1788	
PHG9_Scaffold_3737	5	p2	(TA)6	12	4664	4675	
PHG9_Scaffold_3739	2	p3	(GGT)6	18	9013	9030	potentially variable
PHG9_Scaffold_3742	4	p3	(TAA)6	18	6506	6523	potentially

							variable
PHG9_Scaffold_3743	1	p2	(AT)7	14	421	434	potentially variable
PHG9_Scaffold_3749	1	p3	(ATA)5	15	1060	1074	potentially variable
PHG9_Scaffold_3749	3	p2	(TA)7	14	8287	8300	potentially variable
PHG9_Scaffold_3749	12	p3	(TTA)5	15	37655	37669	potentially variable
PHG9_Scaffold_3750	2	p2	(AT)8	16	2446	2461	potentially variable
PHG9_Scaffold_3750	3	p2	(TA)6	12	2626	2637	
PHG9_Scaffold_3752	2	p2	(AC)9	18	8030	8047	potentially variable
PHG9_Scaffold_3752	3	p2	(TA)8	16	10145	10160	potentially variable
PHG9_Scaffold_3752	6	p2	(AT)6	12	18283	18294	
PHG9_Scaffold_3752	10	p3	(CTC)6	18	36640	36657	potentially variable
PHG9_Scaffold_3752	18	p2	(AC)6	12	70328	70339	
PHG9_Scaffold_3752	20	p2	(AT)8	16	74433	74448	potentially variable
PHG9_Scaffold_3760	2	p2	(AT)6	12	6292	6303	
PHG9_Scaffold_3765	2	p2	(TA)7	14	2919	2932	potentially variable
PHG9_Scaffold_3765	4	p3	(AAT)5	15	5219	5233	potentially variable
PHG9_Scaffold_3773	1	p2	(AT)8	16	1514	1529	potentially variable
PHG9_Scaffold_3775	17	p2	(AT)9	18	51336	51353	potentially

							variable
PHG9_Scaffold_3775	18	p2	(TA)8	16	51640	51655	potentially variable
PHG9_Scaffold_3775	25	p3	(AAT)5	15	60919	60933	potentially variable
PHG9_Scaffold_3775	33	p3	(TTC)6	18	87579	87596	potentially variable
PHG9_Scaffold_3775	35	p2	(TA)7	14	90600	90613	potentially variable
PHG9_Scaffold_3775	37	p3	(TAT)6	18	92781	92798	potentially variable
PHG9_Scaffold_3775	38	p2	(TA)9	18	95092	95109	potentially variable
PHG9_Scaffold_3775	49	p3	(ATA)6	18	129210	129227	potentially variable
PHG9_Scaffold_3779	1	p3	(AGA)5	15	1109	1123	potentially variable
PHG9_Scaffold_3782	2	p3	(GAT)5	15	8584	8598	potentially variable
PHG9_Scaffold_3783	12	p2	(TG)6	12	32530	32541	
PHG9_Scaffold_3783	13	p2	(TA)6	12	39653	39664	
PHG9_Scaffold_3783	16	p2	(AG)8	16	47496	47511	potentially variable
PHG9_Scaffold_3783	27	p2	(TA)7	14	70011	70024	potentially variable
PHG9_Scaffold_3787	2	p2	(TA)6	12	2962	2973	
PHG9_Scaffold_3790	2	p3	(ATT)6	18	2722	2739	potentially variable
PHG9_Scaffold_3790	4	p2	(AT)6	12	5152	5163	
PHG9_Scaffold_3799	8	p2	(TA)7	14	19225	19238	potentially

							variable
PHG9_Scaffold_3801	1	p2	(AT)6	12	5856	5867	
PHG9_Scaffold_3808	6	p2	(TA)8	16	49554	49569	potentially variable
PHG9_Scaffold_3810	4	p2	(AC)7	14	1467	1480	potentially variable
PHG9_Scaffold_3818	4	p3	(ACA)5	15	8026	8040	potentially variable
PHG9_Scaffold_3822	2	p3	(ATA)5	15	5568	5582	potentially variable
PHG9_Scaffold_3822	16	p2	(TC)6	12	38737	38748	
PHG9_Scaffold_3822	18	p3	(AGT)5	15	40402	40416	potentially variable
PHG9_Scaffold_3822	19	p2	(CA)7	14	46085	46098	potentially variable
PHG9_Scaffold_3822	20	p2	(CT)6	12	46248	46259	
PHG9_Scaffold_3822	21	p2	(AT)7	14	46639	46652	potentially variable
PHG9_Scaffold_3822	23	p3	(GTA)5	15	60732	60746	potentially variable
PHG9_Scaffold_3823	1	p2	(TA)8	16	2753	2768	potentially variable
PHG9_Scaffold_3824	1	p2	(AG)6	12	3013	3024	
PHG9_Scaffold_3826	5	p3	(ATT)5	15	17631	17645	potentially variable
PHG9_Scaffold_3832	5	p2	(AT)7	14	12702	12715	potentially variable
PHG9_Scaffold_3836	7	p2	(TA)7	14	7845	7858	potentially variable
PHG9_Scaffold_3839	2	p2	(TA)6	12	2637	2648	

PHG9_Scaffold_3839	4	p2	(AT)6	12	5150	5161	
PHG9_Scaffold_3840	1	p2	(AT)7	14	4536	4549	potentially variable
PHG9_Scaffold_3840	16	p3	(CTA)5	15	47813	47827	potentially variable
PHG9_Scaffold_3840	20	p2	(TA)7	14	64874	64887	potentially variable
PHG9_Scaffold_3840	22	p2	(AT)8	16	69841	69856	potentially variable
PHG9_Scaffold_3840	26	p2	(AT)6	12	72540	72551	
PHG9_Scaffold_3840	40	p3	(ACA)5	15	100533	100547	potentially variable
PHG9_Scaffold_3840	47	p2	(CT)6	12	114646	114657	
PHG9_Scaffold_3840	49	p3	(TTA)5	15	123388	123402	potentially variable
PHG9_Scaffold_3842	1	p2	(TA)7	14	2457	2470	potentially variable
PHG9_Scaffold_3843	21	p3	(AAT)5	15	88984	88998	potentially variable
PHG9_Scaffold_3845	5	p2	(TA)6	12	5700	5711	
PHG9_Scaffold_3845	6	p2	(AT)9	18	8530	8547	potentially variable
PHG9_Scaffold_3847	1	p2	(TA)7	14	1189	1202	potentially variable
PHG9_Scaffold_3852	17	p3	(ATT)5	15	41771	41785	potentially variable
PHG9_Scaffold_3852	36	p2	(AT)7	14	135945	135958	potentially variable
PHG9_Scaffold_3852	37	p3	(GTT)6	18	141386	141403	potentially variable

PHG9_Scaffold_3853	1	p2	(TA)6	12	1152	1163	
PHG9_Scaffold_3855	8	p2	(TA)6	12	15023	15034	
PHG9_Scaffold_3855	12	p3	(ATC)6	18	25958	25975	potentially variable
PHG9_Scaffold_3855	15	p2	(TA)6	12	38603	38614	
PHG9_Scaffold_3855	21	p2	(TA)6	12	60505	60516	
PHG9_Scaffold_3856	2	p3	(CTC)5	15	1926	1940	potentially variable
PHG9_Scaffold_3858	1	p2	(TA)6	12	1962	1973	
PHG9_Scaffold_3861	1	p2	(TA)7	14	178	191	potentially variable
PHG9_Scaffold_3862	4	p3	(GAA)5	15	5856	5870	potentially variable
PHG9_Scaffold_3862	20	p2	(TC)9	18	52588	52605	potentially variable
PHG9_Scaffold_3862	25	p2	(TA)6	12	63547	63558	
PHG9_Scaffold_3862	29	p2	(AG)9	18	86915	86932	potentially variable
PHG9_Scaffold_3862	37	p2	(GA)8	16	108341	108356	potentially variable
PHG9_Scaffold_3862	42	p2	(AC)6	12	116423	116434	
PHG9_Scaffold_3862	47	p2	(TA)6	12	126256	126267	
PHG9_Scaffold_3865	1	p2	(TG)6	12	818	829	
PHG9_Scaffold_3865	3	p2	(AT)6	12	7822	7833	
PHG9_Scaffold_3865	18	p3	(GTG)5	15	50363	50377	potentially variable
PHG9_Scaffold_3865	19	p2	(GT)8	16	50532	50547	potentially variable
PHG9_Scaffold_3865	20	p2	(AT)6	12	51130	51141	

PHG9_Scaffold_3865	24	p2	(TA)6	12	57212	57223	
PHG9_Scaffold_3866	4	p3	(ATC)5	15	23724	23738	potentially variable
PHG9_Scaffold_3866	7	p2	(GT)6	12	47148	47159	
PHG9_Scaffold_3866	11	p3	(AAT)5	15	66734	66748	potentially variable
PHG9_Scaffold_3866	17	p2	(TA)8	16	77430	77445	potentially variable
PHG9_Scaffold_3871	4	p2	(AT)6	12	5302	5313	
PHG9_Scaffold_3873	1	p3	(TAT)6	18	6049	6066	potentially variable
PHG9_Scaffold_3874	16	p3	(TTA)5	15	43608	43622	potentially variable
PHG9_Scaffold_3875	5	p2	(AT)6	12	5111	5122	
PHG9_Scaffold_3879	1	p2	(TC)6	12	601	612	
PHG9_Scaffold_3879	9	p2	(TA)8	16	36089	36104	potentially variable
PHG9_Scaffold_3882	1	p2	(AT)6	12	503	514	
PHG9_Scaffold_3888	5	p2	(AT)6	12	4423	4434	
PHG9_Scaffold_3894	2	p2	(AT)7	14	2802	2815	potentially variable
PHG9_Scaffold_3896	2	p3	(GTT)5	15	6610	6624	potentially variable
PHG9_Scaffold_3896	5	p2	(TC)9	18	16173	16190	potentially variable
PHG9_Scaffold_3896	18	p2	(AC)8	16	60480	60495	potentially variable
PHG9_Scaffold_3896	36	p2	(TA)7	14	115554	115567	potentially variable

PHG9_Scaffold_3896	37	p3	(CTC)5	15	121130	121144	potentially variable
PHG9_Scaffold_3896	44	p3	(TCT)6	18	133183	133200	potentially variable
PHG9_Scaffold_3896	49	p2	(AT)7	14	146254	146267	potentially variable
PHG9_Scaffold_3896	52	p2	(TA)6	12	162571	162582	
PHG9_Scaffold_3897	1	p3	(TAT)6	18	50	67	potentially variable
PHG9_Scaffold_3898	4	p2	(TA)6	12	10554	10565	
PHG9_Scaffold_3898	9	p3	(ATC)5	15	24844	24858	potentially variable
PHG9_Scaffold_3898	14	p6	(CCATGC)12	72	32139	32210	Hypervariable
PHG9_Scaffold_3901	1	p3	(ATG)6	18	430	447	potentially variable
PHG9_Scaffold_3902	5	p2	(AT)7	14	7062	7075	potentially variable
PHG9_Scaffold_3902	7	p2	(AT)7	14	9846	9859	potentially variable
PHG9_Scaffold_3903	9	p2	(TA)7	14	23946	23959	potentially variable
PHG9_Scaffold_3906	1	p3	(GAG)5	15	2906	2920	potentially variable
PHG9_Scaffold_3917	3	p2	(AT)9	18	17865	17882	potentially variable
PHG9_Scaffold_3917	4	p2	(AT)6	12	29133	29144	
PHG9_Scaffold_3917	10	p3	(TTA)5	15	55038	55052	potentially variable
PHG9_Scaffold_3917	12	p2	(TA)6	12	55564	55575	
PHG9_Scaffold_3917	13	p2	(GA)6	12	56089	56100	

PHG9_Scaffold_3917	14	p3	(TAT)6	18	58572	58589	potentially variable
PHG9_Scaffold_3917	15	p2	(GA)6	12	59130	59141	
PHG9_Scaffold_3917	21	p2	(TA)7	14	75005	75018	potentially variable
PHG9_Scaffold_3917	26	p2	(AT)6	12	81325	81336	
PHG9_Scaffold_3917	33	p3	(GCT)5	15	100262	100276	potentially variable
PHG9_Scaffold_3917	43	p2	(AT)6	12	196080	196091	
PHG9_Scaffold_3918	1	p2	(AT)7	14	2558	2571	potentially variable
PHG9_Scaffold_3921	1	p2	(TG)6	12	5944	5955	
PHG9_Scaffold_3921	6	p2	(AC)6	12	29483	29494	
PHG9_Scaffold_3921	9	p2	(AC)7	14	50819	50832	potentially variable
PHG9_Scaffold_3921	17	p3	(AAT)5	15	65654	65668	potentially variable
PHG9_Scaffold_3921	20	p2	(AT)6	12	68818	68829	
PHG9_Scaffold_3921	30	p3	(ATA)5	15	108028	108042	potentially variable
PHG9_Scaffold_3921	40	p2	(GT)8	16	133452	133467	potentially variable
PHG9_Scaffold_3921	43	p3	(ATA)5	15	139537	139551	potentially variable
PHG9_Scaffold_3922	2	p2	(TA)7	14	3466	3479	potentially variable
PHG9_Scaffold_3922	3	p2	(TA)8	16	4184	4199	potentially variable
PHG9_Scaffold_3945	1	p2	(CA)8	16	2987	3002	potentially variable

PHG9_Scaffold_3947	4	p2	(AT)6	12	30103	30114	
PHG9_Scaffold_3948	1	p2	(TG)9	18	1160	1177	potentially variable
PHG9_Scaffold_3951	1	p2	(TA)8	16	3696	3711	potentially variable
PHG9_Scaffold_3953	2	p3	(TGG)5	15	10127	10141	potentially variable
PHG9_Scaffold_3953	24	p2	(AG)6	12	62990	63001	
PHG9_Scaffold_3953	28	p3	(TCA)5	15	80987	81001	potentially variable
PHG9_Scaffold_3954	1	p2	(TA)8	16	7320	7335	potentially variable
PHG9_Scaffold_3964	1	p2	(TA)6	12	2405	2416	
PHG9_Scaffold_3965	2	p2	(AT)7	14	1864	1877	potentially variable
PHG9_Scaffold_3965	6	p2	(TC)8	16	14606	14621	potentially variable
PHG9_Scaffold_3965	11	p2	(TC)6	12	23661	23672	
PHG9_Scaffold_3965	12	p2	(AT)7	14	28936	28949	potentially variable
PHG9_Scaffold_3967	1	p3	(ATT)5	15	3601	3615	potentially variable
PHG9_Scaffold_3968	5	p2	(GA)9	18	29910	29927	potentially variable
PHG9_Scaffold_3968	8	p2	(TA)7	14	33833	33846	potentially variable
PHG9_Scaffold_3981	2	p2	(AT)7	14	547	560	potentially variable
PHG9_Scaffold_3981	6	p3	(AAT)5	15	2981	2995	potentially variable

PHG9_Scaffold_3984	1	p2	(AT)6	12	200	211	
PHG9_Scaffold_3984	11	p2	(TA)7	14	16099	16112	potentially variable
PHG9_Scaffold_3984	12	p2	(TC)9	18	16542	16559	potentially variable
PHG9_Scaffold_3984	13	p2	(TA)6	12	22640	22651	
PHG9_Scaffold_3987	1	p2	(AT)6	12	3425	3436	
PHG9_Scaffold_3988	2	p2	(TC)7	14	978	991	potentially variable
PHG9_Scaffold_3991	1	p2	(TA)6	12	2915	2926	
PHG9_Scaffold_3997	3	p2	(AT)6	12	3212	3223	
PHG9_Scaffold_4001	4	p2	(CT)6	12	11802	11813	
PHG9_Scaffold_4002	2	p3	(ATC)5	15	1090	1104	potentially variable
PHG9_Scaffold_4008	4	p2	(TA)6	12	6597	6608	
PHG9_Scaffold_4008	7	p2	(AT)8	16	14673	14688	potentially variable
PHG9_Scaffold_4012	7	p2	(AT)6	12	27672	27683	
PHG9_Scaffold_4016	1	p2	(AT)6	12	2801	2812	
PHG9_Scaffold_4016	14	p2	(GT)6	12	40019	40030	
PHG9_Scaffold_4026	2	p2	(AT)6	12	4482	4493	
PHG9_Scaffold_4026	3	p2	(TC)8	16	20900	20915	potentially variable
PHG9_Scaffold_4031	1	p2	(TC)6	12	164	175	
PHG9_Scaffold_4045	3	p2	(AT)6	12	3225	3236	
PHG9_Scaffold_4051	4	p3	(AAC)5	15	4193	4207	potentially variable
PHG9_Scaffold_4051	9	p2	(AG)6	12	17349	17360	
PHG9_Scaffold_4051	16	p3	(TAA)6	18	38704	38721	potentially

							variable
PHG9_Scaffold_4053	2	p2	(TA)7	14	1109	1122	potentially variable
PHG9_Scaffold_4053	3	p2	(AT)7	14	3831	3844	potentially variable
PHG9_Scaffold_4054	2	p2	(TA)8	16	1089	1104	potentially variable
PHG9_Scaffold_4054	10	p2	(TC)7	14	32227	32240	potentially variable
PHG9_Scaffold_4054	11	p2	(GA)6	12	35190	35201	
PHG9_Scaffold_4056	2	p2	(TA)8	16	5496	5511	potentially variable
PHG9_Scaffold_4059	1	p3	(ATA)6	18	1120	1137	potentially variable
PHG9_Scaffold_4060	1	p2	(TA)6	12	25	36	
PHG9_Scaffold_4061	5	p3	(TAA)5	15	10305	10319	potentially variable
PHG9_Scaffold_4065	3	p2	(TA)6	12	6488	6499	
PHG9_Scaffold_4066	1	p2	(CA)7	14	1208	1221	potentially variable
PHG9_Scaffold_4067	3	p2	(CT)6	12	18262	18273	
PHG9_Scaffold_4067	10	p2	(CT)6	12	47670	47681	
PHG9_Scaffold_4067	11	p2	(AT)6	12	48307	48318	
PHG9_Scaffold_4068	2	p2	(AG)6	12	6324	6335	
PHG9_Scaffold_4068	3	p2	(AT)6	12	8437	8448	
PHG9_Scaffold_4072	3	p2	(TA)8	16	2876	2891	potentially variable
PHG9_Scaffold_4075	5	p2	(TA)7	14	9574	9587	potentially variable

PHG9_Scaffold_4077	3	p2	(AT)7	14	3125	3138	potentially variable
PHG9_Scaffold_4082	2	p2	(TG)7	14	4681	4694	potentially variable
PHG9_Scaffold_4083	4	p2	(TA)8	16	8295	8310	potentially variable
PHG9_Scaffold_4084	3	p3	(TAA)5	15	8590	8604	potentially variable
PHG9_Scaffold_4087	1	p3	(GGA)5	15	859	873	potentially variable
PHG9_Scaffold_4089	1	p2	(CA)6	12	146	157	
PHG9_Scaffold_4089	2	p3	(TGT)5	15	776	790	potentially variable
PHG9_Scaffold_4089	3	p3	(AAT)6	18	5599	5616	potentially variable
PHG9_Scaffold_4108	1	p2	(AG)9	18	1331	1348	potentially variable
PHG9_Scaffold_4108	6	p2	(AT)9	18	5725	5742	potentially variable
PHG9_Scaffold_4108	8	p2	(TA)6	12	7856	7867	
PHG9_Scaffold_4109	2	p2	(AT)7	14	5223	5236	potentially variable
PHG9_Scaffold_4111	2	p2	(TA)6	12	4054	4065	
PHG9_Scaffold_4113	1	p3	(CCT)5	15	1336	1350	potentially variable
PHG9_Scaffold_4114	4	p2	(TA)8	16	2305	2320	potentially variable
PHG9_Scaffold_4120	4	p3	(TTC)5	15	39338	39352	potentially variable
PHG9_Scaffold_4120	5	p2	(AT)6	12	39752	39763	

PHG9_Scaffold_4123	11	p2	(AG)6	12	34580	34591	
PHG9_Scaffold_4123	30	p2	(TA)8	16	100225	100240	potentially variable
PHG9_Scaffold_4123	31	p2	(AT)8	16	101698	101713	potentially variable
PHG9_Scaffold_4123	37	p3	(ATC)5	15	110425	110439	potentially variable
PHG9_Scaffold_4123	38	p3	(AAT)5	15	113549	113563	potentially variable
PHG9_Scaffold_4126	4	p2	(TA)7	14	20820	20833	potentially variable
PHG9_Scaffold_4126	5	p3	(TTA)5	15	23276	23290	potentially variable
PHG9_Scaffold_4126	12	p2	(TA)8	16	62597	62612	potentially variable
PHG9_Scaffold_4126	14	p3	(TAT)5	15	64134	64148	potentially variable
PHG9_Scaffold_4126	17	p2	(TC)6	12	98953	98964	
PHG9_Scaffold_4134	1	p3	(TCC)5	15	1170	1184	potentially variable
PHG9_Scaffold_4134	10	p3	(GAA)6	18	39765	39782	potentially variable
PHG9_Scaffold_4134	11	p2	(TC)8	16	40826	40841	potentially variable
PHG9_Scaffold_4134	16	p2	(CT)6	12	49957	49968	
PHG9_Scaffold_4134	19	p2	(AT)7	14	62522	62535	potentially variable
PHG9_Scaffold_4134	20	p3	(CTC)5	15	63292	63306	potentially variable
PHG9_Scaffold_4134	26	p3	(TTG)5	15	75294	75308	potentially

							variable
PHG9_Scaffold_4134	44	p2	(CT)7	14	115963	115976	potentially variable
PHG9_Scaffold_4134	45	p2	(AT)9	18	123730	123747	potentially variable
PHG9_Scaffold_4134	46	p2	(AT)8	16	128514	128529	potentially variable
PHG9_Scaffold_4134	47	p2	(AT)6	12	129481	129492	
PHG9_Scaffold_4134	57	p3	(CAT)5	15	157679	157693	potentially variable
PHG9_Scaffold_4140	3	p2	(CT)6	12	4922	4933	
PHG9_Scaffold_4142	1	p2	(AT)7	14	2375	2388	potentially variable
PHG9_Scaffold_4150	2	p3	(TAA)5	15	5818	5832	potentially variable
PHG9_Scaffold_4155	1	p2	(CT)6	12	1585	1596	
PHG9_Scaffold_4156	2	p2	(TA)6	12	3159	3170	
PHG9_Scaffold_4158	1	p2	(AT)9	18	3022	3039	potentially variable
PHG9_Scaffold_4161	2	p2	(CA)7	14	8425	8438	potentially variable
PHG9_Scaffold_4161	11	p2	(TC)8	16	34049	34064	potentially variable
PHG9_Scaffold_4161	12	p3	(TTA)5	15	39345	39359	potentially variable
PHG9_Scaffold_4161	13	p2	(GA)8	16	42580	42595	potentially variable
PHG9_Scaffold_4161	31	p3	(TCT)5	15	86227	86241	potentially variable
PHG9_Scaffold_4161	33	p2	(AT)6	12	97153	97164	

PHG9_Scaffold_4161	34	p2	(AG)7	14	98762	98775	potentially variable
PHG9_Scaffold_4161	38	p2	(TA)6	12	103826	103837	
PHG9_Scaffold_4161	41	p2	(AT)8	16	117236	117251	potentially variable
PHG9_Scaffold_4161	44	p3	(ATA)5	15	141759	141773	potentially variable
PHG9_Scaffold_4161	45	p3	(AGA)5	15	143477	143491	potentially variable
PHG9_Scaffold_4161	51	p2	(AT)7	14	158848	158861	potentially variable
PHG9_Scaffold_4171	1	p2	(AT)6	12	1667	1678	
PHG9_Scaffold_4172	3	p3	(TTA)6	18	9208	9225	potentially variable
PHG9_Scaffold_4175	1	p2	(TA)6	12	1127	1138	
PHG9_Scaffold_4178	2	p2	(TA)6	12	2332	2343	
PHG9_Scaffold_4179	1	p2	(TA)9	18	316	333	potentially variable
PHG9_Scaffold_4187	1	p2	(TA)6	12	1416	1427	
PHG9_Scaffold_4189	1	p2	(AT)7	14	8379	8392	potentially variable
PHG9_Scaffold_4189	12	p3	(TCA)6	18	49559	49576	potentially variable
PHG9_Scaffold_4189	13	p2	(AT)8	16	58172	58187	potentially variable
PHG9_Scaffold_4189	14	p3	(TAA)6	18	60836	60853	potentially variable
PHG9_Scaffold_4189	15	p3	(TAA)6	18	61892	61909	potentially variable
PHG9_Scaffold_4189	17	p2	(AT)8	16	72201	72216	potentially

							variable
PHG9_Scaffold_4189	21	p2	(AT)8	16	95546	95561	potentially variable
PHG9_Scaffold_4191	2	p2	(AT)6	12	2696	2707	
PHG9_Scaffold_4192	1	p2	(TC)6	12	421	432	
PHG9_Scaffold_4192	2	p2	(CT)6	12	2944	2955	
PHG9_Scaffold_4194	6	p2	(AT)6	12	25473	25484	
PHG9_Scaffold_4194	17	p2	(TC)7	14	83961	83974	potentially variable
PHG9_Scaffold_4194	20	p2	(AT)6	12	88976	88987	
PHG9_Scaffold_4204	4	p2	(AG)6	12	6379	6390	
PHG9_Scaffold_4210	3	p3	(AGG)5	15	4856	4870	potentially variable
PHG9_Scaffold_4217	2	p3	(TAT)5	15	11799	11813	potentially variable
PHG9_Scaffold_4218	1	p2	(TA)7	14	6906	6919	potentially variable
PHG9_Scaffold_4218	2	p2	(GA)6	12	7180	7191	
PHG9_Scaffold_4224	3	p2	(CT)7	14	867	880	potentially variable
PHG9_Scaffold_4243	3	p3	(ATG)5	15	4314	4328	potentially variable
PHG9_Scaffold_4244	1	p3	(GAA)5	15	3537	3551	potentially variable
PHG9_Scaffold_4249	1	p2	(AT)9	18	4305	4322	potentially variable
PHG9_Scaffold_4251	2	p2	(TA)7	14	210	223	potentially variable
PHG9_Scaffold_4256	1	p2	(TA)7	14	5	18	potentially

							variable
PHG9_Scaffold_4257	7	p3	(ATT)6	18	24730	24747	potentially variable
PHG9_Scaffold_4259	7	p2	(AT)8	16	79404	79419	potentially variable
PHG9_Scaffold_4259	10	p3	(ATA)6	18	138349	138366	potentially variable
PHG9_Scaffold_4259	12	p2	(AT)6	12	146629	146640	
PHG9_Scaffold_4259	19	p2	(AT)6	12	213664	213675	
PHG9_Scaffold_4260	11	p2	(AC)7	14	35399	35412	potentially variable
PHG9_Scaffold_4265	4	p2	(AT)8	16	2988	3003	potentially variable
PHG9_Scaffold_4272	1	p2	(GA)9	18	459	476	potentially variable
PHG9_Scaffold_4272	2	p2	(AT)6	12	1210	1221	
PHG9_Scaffold_4273	1	p2	(TA)6	12	3963	3974	
PHG9_Scaffold_4276	1	p3	(TGA)5	15	318	332	potentially variable
PHG9_Scaffold_4281	1	p2	(TA)6	12	148	159	
PHG9_Scaffold_4281	4	p2	(GA)6	12	6617	6628	
PHG9_Scaffold_4282	1	p2	(AT)7	14	5223	5236	potentially variable
PHG9_Scaffold_4284	1	p2	(TA)6	12	3525	3536	
PHG9_Scaffold_4293	1	p2	(TG)8	16	2130	2145	potentially variable
PHG9_Scaffold_4293	2	p3	(CTT)5	15	2502	2516	potentially variable
PHG9_Scaffold_4295	3	p2	(AT)6	12	8404	8415	

PHG9_Scaffold_4299	2	p2	(TG)9	18	1675	1692	potentially variable
PHG9_Scaffold_4301	1	p3	(ATT)5	15	554	568	potentially variable
PHG9_Scaffold_4303	2	p3	(CAT)5	15	2766	2780	potentially variable
PHG9_Scaffold_4312	1	p2	(TA)7	14	2331	2344	potentially variable
PHG9_Scaffold_4313	2	p2	(CA)7	14	1382	1395	potentially variable
PHG9_Scaffold_4318	1	p2	(TA)7	14	371	384	potentially variable
PHG9_Scaffold_4318	6	p2	(AT)6	12	8302	8313	
PHG9_Scaffold_4322	4	p2	(TA)6	12	13987	13998	
PHG9_Scaffold_4325	1	p2	(AC)6	12	1810	1821	
PHG9_Scaffold_4334	1	p2	(TA)6	12	2659	2670	
PHG9_Scaffold_4335	2	p2	(AG)7	14	13790	13803	potentially variable
PHG9_Scaffold_4335	5	p2	(CA)8	16	22694	22709	potentially variable
PHG9_Scaffold_4336	2	p2	(CT)7	14	2729	2742	potentially variable
PHG9_Scaffold_4338	1	p3	(TCA)6	18	890	907	potentially variable
PHG9_Scaffold_4341	2	p2	(TA)6	12	1884	1895	
PHG9_Scaffold_4346	1	p3	(ATT)5	15	2757	2771	potentially variable
PHG9_Scaffold_4346	4	p2	(AT)7	14	10233	10246	potentially variable
PHG9_Scaffold_4346	6	p2	(TA)6	12	11519	11530	

PHG9_Scaffold_4348	1	p3	(ATA)5	15	451	465	potentially variable
PHG9_Scaffold_4351	1	p2	(AT)6	12	2351	2362	
PHG9_Scaffold_4358	3	p2	(GA)7	14	4622	4635	potentially variable
PHG9_Scaffold_4363	1	p2	(TA)8	16	16907	16922	potentially variable
PHG9_Scaffold_4363	5	p3	(CGC)5	15	36744	36758	potentially variable
PHG9_Scaffold_4363	15	p2	(AT)7	14	119196	119209	potentially variable
PHG9_Scaffold_4365	3	p2	(AT)8	16	5593	5608	potentially variable
PHG9_Scaffold_4365	10	p3	(AGA)5	15	18317	18331	potentially variable
PHG9_Scaffold_4365	26	p2	(TA)9	18	56302	56319	potentially variable
PHG9_Scaffold_4365	29	p3	(ATA)5	15	67850	67864	potentially variable
PHG9_Scaffold_4365	34	p2	(TA)8	16	83974	83989	potentially variable
PHG9_Scaffold_4365	35	p3	(ATT)6	18	84137	84154	potentially variable
PHG9_Scaffold_4365	37	p2	(AT)7	14	93571	93584	potentially variable
PHG9_Scaffold_4365	39	p3	(TAT)5	15	94265	94279	potentially variable
PHG9_Scaffold_4365	46	p2	(AT)8	16	121479	121494	potentially variable
PHG9_Scaffold_4399	2	p2	(GA)7	14	4796	4809	potentially

							variable
PHG9_Scaffold_4406	7	p2	(TA)8	16	24889	24904	potentially variable
PHG9_Scaffold_4406	8	p3	(TTG)5	15	31971	31985	potentially variable
PHG9_Scaffold_4406	12	p2	(AT)7	14	46399	46412	potentially variable
PHG9_Scaffold_4406	21	p2	(TC)6	12	54574	54585	
PHG9_Scaffold_4406	26	p2	(AT)6	12	72249	72260	
PHG9_Scaffold_4407	6	p2	(TA)6	12	15975	15986	
PHG9_Scaffold_4407	8	p2	(AT)9	18	18026	18043	potentially variable
PHG9_Scaffold_4412	1	p3	(TTA)5	15	1055	1069	potentially variable
PHG9_Scaffold_4420	2	p2	(TA)7	14	4480	4493	potentially variable
PHG9_Scaffold_4420	4	p2	(AT)7	14	10631	10644	potentially variable
PHG9_Scaffold_4420	6	p2	(AT)7	14	13812	13825	potentially variable
PHG9_Scaffold_4420	9	p2	(TA)6	12	22424	22435	
PHG9_Scaffold_4420	11	p3	(TAA)5	15	31592	31606	potentially variable
PHG9_Scaffold_4422	1	p2	(TA)6	12	1413	1424	
PHG9_Scaffold_4425	1	p3	(TAA)5	15	857	871	potentially variable
PHG9_Scaffold_4432	1	p2	(AT)8	16	586	601	potentially variable
PHG9_Scaffold_4432	2	p2	(TA)8	16	1981	1996	potentially variable

PHG9_Scaffold_4437	9	p2	(TA)9	18	23099	23116	potentially variable
PHG9_Scaffold_4437	10	p3	(GAA)5	15	28170	28184	potentially variable
PHG9_Scaffold_4437	24	p2	(AG)6	12	101756	101767	
PHG9_Scaffold_4437	29	p2	(TG)6	12	119622	119633	
PHG9_Scaffold_4437	34	p2	(TA)6	12	141715	141726	
PHG9_Scaffold_4437	38	p2	(TA)9	18	162907	162924	potentially variable
PHG9_Scaffold_4437	39	p3	(TAT)5	15	163105	163119	potentially variable
PHG9_Scaffold_4437	41	p2	(AG)8	16	177871	177886	potentially variable
PHG9_Scaffold_4437	42	p2	(TC)6	12	178019	178030	
PHG9_Scaffold_4437	43	p3	(TTG)5	15	178578	178592	potentially variable
PHG9_Scaffold_4437	44	p3	(GGT)5	15	181161	181175	potentially variable
PHG9_Scaffold_4437	52	p2	(AG)7	14	211885	211898	potentially variable
PHG9_Scaffold_4437	58	p2	(TA)6	12	228469	228480	
PHG9_Scaffold_4437	60	p2	(TA)6	12	230205	230216	
PHG9_Scaffold_4437	70	p3	(ATG)5	15	262954	262968	potentially variable
PHG9_Scaffold_4440	2	p2	(GA)6	12	2198	2209	
PHG9_Scaffold_4444	1	p2	(AT)8	16	880	895	potentially variable
PHG9_Scaffold_4445	6	p3	(AAT)6	18	19965	19982	potentially variable

PHG9_Scaffold_4451	1	p3	(CCG)5	15	2577	2591	potentially variable
PHG9_Scaffold_4462	5	p6	(ATGAAC)8	48	9990	10037	Hypervariable
PHG9_Scaffold_4462	6	p3	(TTC)5	15	11681	11695	potentially variable
PHG9_Scaffold_4462	12	p2	(TC)6	12	29722	29733	
PHG9_Scaffold_4462	13	p2	(TA)6	12	30895	30906	
PHG9_Scaffold_4462	17	p2	(AT)6	12	38571	38582	
PHG9_Scaffold_4473	9	p2	(GA)6	12	22555	22566	
PHG9_Scaffold_4473	14	p2	(AC)8	16	35724	35739	potentially variable
PHG9_Scaffold_4473	20	p2	(AC)6	12	56665	56676	
PHG9_Scaffold_4473	25	p2	(AT)7	14	72207	72220	potentially variable
PHG9_Scaffold_4473	29	p2	(AC)6	12	88804	88815	
PHG9_Scaffold_4473	35	p2	(TA)8	16	109765	109780	potentially variable
PHG9_Scaffold_4473	38	p3	(TAA)5	15	119049	119063	potentially variable
PHG9_Scaffold_4473	40	p2	(AT)6	12	123629	123640	
PHG9_Scaffold_4473	44	p3	(TAT)6	18	134261	134278	potentially variable
PHG9_Scaffold_4473	47	p2	(TA)6	12	165171	165182	
PHG9_Scaffold_4473	48	p3	(CAA)6	18	171825	171842	potentially variable
PHG9_Scaffold_4473	51	p2	(TA)6	12	185813	185824	
PHG9_Scaffold_4474	3	p2	(AT)7	14	4810	4823	potentially variable
PHG9_Scaffold_4475	1	p2	(AT)8	16	383	398	potentially

							variable
PHG9_Scaffold_4479	1	p2	(AG)6	12	653	664	
PHG9_Scaffold_4492	1	p2	(TA)8	16	1450	1465	potentially variable
PHG9_Scaffold_4492	7	p3	(ATA)6	18	12019	12036	potentially variable
PHG9_Scaffold_4492	11	p2	(TA)8	16	18737	18752	potentially variable
PHG9_Scaffold_4492	27	p2	(AT)6	12	74471	74482	
PHG9_Scaffold_4492	36	p2	(TC)7	14	100192	100205	potentially variable
PHG9_Scaffold_4492	43	p3	(ATT)5	15	139198	139212	potentially variable
PHG9_Scaffold_4499	1	p3	(ATA)5	15	2862	2876	potentially variable
PHG9_Scaffold_4501	2	p2	(TA)6	12	531	542	
PHG9_Scaffold_4502	2	p2	(TA)6	12	2054	2065	
PHG9_Scaffold_4507	1	p3	(CTT)5	15	1406	1420	potentially variable
PHG9_Scaffold_4518	1	p2	(AC)8	16	1232	1247	potentially variable
PHG9_Scaffold_4518	2	p2	(GA)6	12	3456	3467	
PHG9_Scaffold_4518	3	p3	(CAT)5	15	3934	3948	potentially variable
PHG9_Scaffold_4518	4	p3	(TCA)5	15	4104	4118	potentially variable
PHG9_Scaffold_4518	11	p2	(AG)7	14	27351	27364	potentially variable
PHG9_Scaffold_4518	12	p6	(TTGCAT)5	30	27681	27710	Hypervariable

PHG9_Scaffold_4518	13	p2	(CA)7	14	28015	28028	potentially variable
PHG9_Scaffold_4518	16	p3	(CTT)6	18	49035	49052	potentially variable
PHG9_Scaffold_4518	22	p3	(GAA)5	15	66075	66089	potentially variable
PHG9_Scaffold_4518	23	p2	(AT)6	12	68941	68952	
PHG9_Scaffold_4518	24	p2	(TC)9	18	78111	78128	potentially variable
PHG9_Scaffold_4518	25	p6	(GGTGTT)5	30	79121	79150	Hypervariable
PHG9_Scaffold_4518	26	p2	(AT)9	18	80350	80367	potentially variable
PHG9_Scaffold_4518	27	p3	(TAA)5	15	82022	82036	potentially variable
PHG9_Scaffold_4518	28	p2	(TC)6	12	83979	83990	
PHG9_Scaffold_4518	29	p2	(TA)6	12	86380	86391	
PHG9_Scaffold_4518	32	p3	(CTT)5	15	110658	110672	potentially variable
PHG9_Scaffold_4518	38	p3	(GAA)5	15	122710	122724	potentially variable
PHG9_Scaffold_4518	39	p2	(CT)6	12	123291	123302	
PHG9_Scaffold_4518	40	p2	(GA)7	14	129623	129636	potentially variable
PHG9_Scaffold_4518	41	p2	(CT)7	14	129829	129842	potentially variable
PHG9_Scaffold_4518	42	p2	(TC)8	16	138038	138053	potentially variable
PHG9_Scaffold_4518	43	p3	(TCG)5	15	138398	138412	potentially variable
PHG9_Scaffold_4518	46	p2	(CT)7	14	151403	151416	potentially

							variable
PHG9_Scaffold_4518	48	p2	(TC)8	16	152962	152977	potentially variable
PHG9_Scaffold_4518	49	p3	(ATT)5	15	154326	154340	potentially variable
PHG9_Scaffold_4518	50	p2	(AT)7	14	156196	156209	potentially variable
PHG9_Scaffold_4518	51	p2	(TA)8	16	163366	163381	potentially variable
PHG9_Scaffold_4522	2	p3	(TCT)6	18	758	775	potentially variable
PHG9_Scaffold_4522	3	p2	(GT)6	12	6453	6464	
PHG9_Scaffold_4525	1	p2	(TA)7	14	3085	3098	potentially variable
PHG9_Scaffold_4528	1	p2	(TA)6	12	20149	20160	
PHG9_Scaffold_4529	3	p2	(AT)6	12	3235	3246	
PHG9_Scaffold_4529	5	p2	(TA)6	12	7550	7561	
PHG9_Scaffold_4532	1	p3	(AGA)5	15	6269	6283	potentially variable
PHG9_Scaffold_4542	9	p2	(AT)6	12	84954	84965	
PHG9_Scaffold_4542	12	p2	(AT)6	12	104567	104578	
PHG9_Scaffold_4542	19	p3	(TAA)6	18	149110	149127	potentially variable
PHG9_Scaffold_4543	6	p3	(ATA)5	15	23159	23173	potentially variable
PHG9_Scaffold_4543	19	p2	(AG)6	12	67043	67054	
PHG9_Scaffold_4543	26	p3	(TTA)5	15	84422	84436	potentially variable
PHG9_Scaffold_4543	29	p3	(TTA)5	15	93072	93086	potentially

								variable
PHG9_Scaffold_4543	31	p2	(AC)6	12	103282	103293		
PHG9_Scaffold_4543	44	p2	(AT)8	16	128071	128086	potentially variable	
PHG9_Scaffold_4543	46	p3	(ATA)5	15	131035	131049	potentially variable	
PHG9_Scaffold_4543	50	p2	(AT)6	12	138597	138608		
PHG9_Scaffold_4543	56	p2	(TC)6	12	167366	167377		
PHG9_Scaffold_4543	57	p2	(AT)7	14	168149	168162	potentially variable	
PHG9_Scaffold_4545	5	p3	(TTA)6	18	19891	19908	potentially variable	
PHG9_Scaffold_4545	7	p2	(TA)6	12	35837	35848		
PHG9_Scaffold_4545	12	p2	(AG)6	12	44340	44351		
PHG9_Scaffold_4545	14	p3	(GAA)5	15	58751	58765	potentially variable	
PHG9_Scaffold_4545	20	p2	(TG)7	14	65954	65967	potentially variable	
PHG9_Scaffold_4545	23	p2	(AT)6	12	79514	79525		
PHG9_Scaffold_4547	10	p2	(TA)6	12	62399	62410		
PHG9_Scaffold_4547	27	p2	(AT)6	12	145554	145565		
PHG9_Scaffold_4547	28	p2	(GA)8	16	146292	146307	potentially variable	
PHG9_Scaffold_4547	29	p2	(AT)6	12	147223	147234		
PHG9_Scaffold_4547	38	p3	(TAA)5	15	206549	206563	potentially variable	
PHG9_Scaffold_4548	1	p3	(AAT)5	15	16	30	potentially variable	
PHG9_Scaffold_4548	6	p2	(TA)8	16	18421	18436	potentially	

							variable
PHG9_Scaffold_4548	7	p3	(AGT)5	15	20185	20199	potentially variable
PHG9_Scaffold_4548	17	p2	(TA)7	14	80737	80750	potentially variable
PHG9_Scaffold_4550	7	p2	(CT)7	14	25409	25422	potentially variable
PHG9_Scaffold_4550	12	p2	(TA)7	14	31545	31558	potentially variable
PHG9_Scaffold_4553	4	p2	(AT)8	16	2328	2343	potentially variable
PHG9_Scaffold_4553	19	p2	(AT)7	14	72187	72200	potentially variable
PHG9_Scaffold_4553	28	p2	(AT)8	16	87688	87703	potentially variable
PHG9_Scaffold_4553	38	p2	(AT)6	12	110502	110513	
PHG9_Scaffold_4553	41	p2	(TA)6	12	125409	125420	
PHG9_Scaffold_4553	45	p2	(AT)9	18	129960	129977	potentially variable
PHG9_Scaffold_4553	47	p2	(TA)8	16	132266	132281	potentially variable
PHG9_Scaffold_4553	53	p2	(AT)6	12	149772	149783	
PHG9_Scaffold_4553	61	p2	(TA)7	14	182103	182116	potentially variable
PHG9_Scaffold_4553	65	p3	(CTC)6	18	189856	189873	potentially variable
PHG9_Scaffold_4553	67	p2	(TA)6	12	193939	193950	
PHG9_Scaffold_4553	71	p3	(CTT)6	18	222601	222618	potentially variable
PHG9_Scaffold_4553	72	p2	(AT)7	14	223425	223438	potentially

							variable
PHG9_Scaffold_4553	75	p2	(AC)8	16	231355	231370	potentially variable
PHG9_Scaffold_4553	78	p2	(TA)8	16	241053	241068	potentially variable
PHG9_Scaffold_4553	87	p3	(CAC)6	18	292871	292888	potentially variable
PHG9_Scaffold_4553	88	p3	(CAC)5	15	294726	294740	potentially variable
PHG9_Scaffold_4553	92	p2	(AC)6	12	309258	309269	
PHG9_Scaffold_4553	97	p2	(TA)7	14	322453	322466	potentially variable
PHG9_Scaffold_4553	99	p2	(TA)9	18	326687	326704	potentially variable
PHG9_Scaffold_4553	102	p2	(AT)9	18	340787	340804	potentially variable
PHG9_Scaffold_4553	103	p2	(AT)8	16	341265	341280	potentially variable
PHG9_Scaffold_4553	109	p2	(TA)7	14	359557	359570	potentially variable
PHG9_Scaffold_4553	111	p3	(GAA)5	15	360442	360456	potentially variable
PHG9_Scaffold_4553	113	p6	(TGGGCT)6	36	363448	363483	Hypervariable
PHG9_Scaffold_4553	114	p3	(TAT)5	15	369015	369029	potentially variable
PHG9_Scaffold_4553	115	p2	(TA)7	14	369317	369330	potentially variable
PHG9_Scaffold_4553	116	p2	(AT)8	16	372005	372020	potentially variable
PHG9_Scaffold_4553	126	p3	(ATA)5	15	404924	404938	potentially

							variable
PHG9_Scaffold_4553	129	p2	(GA)7	14	424784	424797	potentially variable
PHG9_Scaffold_4553	139	p2	(AT)6	12	469188	469199	
PHG9_Scaffold_4553	144	p2	(TA)9	18	491158	491175	potentially variable
PHG9_Scaffold_4553	147	p2	(AT)9	18	509032	509049	potentially variable
PHG9_Scaffold_4553	158	p3	(AAT)6	18	544761	544778	potentially variable
PHG9_Scaffold_4553	161	p2	(TC)9	18	551016	551033	potentially variable
PHG9_Scaffold_4553	166	p2	(AT)6	12	568666	568677	
PHG9_Scaffold_4553	177	p2	(GA)6	12	592485	592496	
PHG9_Scaffold_4553	182	p3	(AGA)6	18	612134	612151	potentially variable
PHG9_Scaffold_4553	184	p3	(TGC)5	15	614302	614316	potentially variable
PHG9_Scaffold_4553	190	p2	(AT)6	12	636290	636301	
PHG9_Scaffold_4554	8	p2	(TA)6	12	33647	33658	
PHG9_Scaffold_4554	14	p2	(TA)6	12	59075	59086	
PHG9_Scaffold_4554	18	p2	(AG)6	12	74609	74620	
PHG9_Scaffold_4554	20	p2	(TA)8	16	82189	82204	potentially variable
PHG9_Scaffold_4554	22	p2	(AT)6	12	85562	85573	
PHG9_Scaffold_4554	23	p2	(AT)7	14	90287	90300	potentially variable
PHG9_Scaffold_4555	3	p3	(TTC)5	15	1692	1706	potentially variable

PHG9_Scaffold_4555	15	p2	(AG)8	16	46518	46533	potentially variable
PHG9_Scaffold_4555	17	p3	(TAT)6	18	70553	70570	potentially variable
PHG9_Scaffold_4557	1	p2	(AG)7	14	1372	1385	potentially variable
PHG9_Scaffold_4557	3	p2	(TA)6	12	1921	1932	
PHG9_Scaffold_4557	13	p2	(TA)7	14	27890	27903	potentially variable
PHG9_Scaffold_4557	20	p3	(TTA)5	15	52273	52287	potentially variable
PHG9_Scaffold_4557	24	p2	(TA)6	12	67023	67034	
PHG9_Scaffold_4558	18	p3	(TAT)5	15	83935	83949	potentially variable
PHG9_Scaffold_4558	28	p2	(AT)6	12	125165	125176	
PHG9_Scaffold_4560	2	p2	(TA)6	12	18522	18533	
PHG9_Scaffold_4560	4	p2	(AT)8	16	22817	22832	potentially variable
PHG9_Scaffold_4560	5	p3	(TTA)5	15	40841	40855	potentially variable
PHG9_Scaffold_4560	8	p3	(ATA)6	18	42331	42348	potentially variable
PHG9_Scaffold_4561	7	p3	(TTA)5	15	22148	22162	potentially variable
PHG9_Scaffold_4561	9	p2	(AT)7	14	27734	27747	potentially variable
PHG9_Scaffold_4561	12	p3	(ATT)5	15	32091	32105	potentially variable
PHG9_Scaffold_4561	19	p3	(TAA)6	18	51233	51250	potentially variable

PHG9_Scaffold_4561	24	p2	(TA)7	14	68991	69004	potentially variable
PHG9_Scaffold_4561	25	p2	(TA)6	12	69707	69718	
PHG9_Scaffold_4561	26	p2	(AT)6	12	70723	70734	
PHG9_Scaffold_4561	42	p2	(TA)7	14	139982	139995	potentially variable
PHG9_Scaffold_4561	54	p2	(AG)9	18	179569	179586	potentially variable
PHG9_Scaffold_4561	56	p3	(TCA)5	15	180347	180361	potentially variable
PHG9_Scaffold_4561	57	p3	(GAT)5	15	181711	181725	potentially variable
PHG9_Scaffold_4561	61	p2	(AT)6	12	209377	209388	
PHG9_Scaffold_4561	64	p2	(AT)6	12	235969	235980	
PHG9_Scaffold_4561	65	p3	(TCA)5	15	237383	237397	potentially variable
PHG9_Scaffold_4561	70	p3	(TGC)5	15	295026	295040	potentially variable
PHG9_Scaffold_4561	71	p2	(AG)6	12	299256	299267	
PHG9_Scaffold_4561	79	p2	(CA)6	12	345303	345314	
PHG9_Scaffold_4561	84	p2	(TA)9	18	380369	380386	potentially variable
PHG9_Scaffold_4561	92	p2	(TA)6	12	418922	418933	
PHG9_Scaffold_4561	97	p3	(CAT)5	15	438762	438776	potentially variable
PHG9_Scaffold_4562	3	p2	(GT)7	14	18849	18862	potentially variable
PHG9_Scaffold_4562	14	p3	(AGA)5	15	41418	41432	potentially variable

PHG9_Scaffold_4562	15	p2	(AT)6	12	42314	42325	
PHG9_Scaffold_4562	27	p2	(AT)6	12	69187	69198	
PHG9_Scaffold_4563	2	p2	(AT)6	12	21997	22008	
PHG9_Scaffold_4564	7	p2	(TA)9	18	11581	11598	potentially variable
PHG9_Scaffold_4565	8	p3	(AAT)5	15	5972	5986	potentially variable
PHG9_Scaffold_4565	9	p3	(ATC)5	15	7514	7528	potentially variable
PHG9_Scaffold_4565	10	p2	(AG)6	12	8029	8040	
PHG9_Scaffold_4565	24	p2	(TA)8	16	36353	36368	potentially variable
PHG9_Scaffold_4566	1	p2	(AT)8	16	1048	1063	potentially variable
PHG9_Scaffold_4566	5	p3	(GAT)6	18	25100	25117	potentially variable
PHG9_Scaffold_4566	13	p2	(TA)6	12	48837	48848	
PHG9_Scaffold_4566	21	p2	(CT)7	14	77868	77881	potentially variable
PHG9_Scaffold_4566	22	p2	(AT)7	14	80627	80640	potentially variable
PHG9_Scaffold_4566	27	p3	(GAT)5	15	86827	86841	potentially variable
PHG9_Scaffold_4566	29	p2	(TC)7	14	89900	89913	potentially variable
PHG9_Scaffold_4566	30	p2	(AT)6	12	90604	90615	
PHG9_Scaffold_4566	31	p2	(AT)7	14	91030	91043	potentially variable
PHG9_Scaffold_4566	34	p2	(AT)6	12	98881	98892	

PHG9_Scaffold_4566	38	p2	(AT)8	16	104364	104379	potentially variable
PHG9_Scaffold_4567	2	p3	(AGA)5	15	10323	10337	potentially variable
PHG9_Scaffold_4567	4	p3	(TCT)5	15	14574	14588	potentially variable
PHG9_Scaffold_4570	4	p3	(GTT)6	18	4798	4815	potentially variable
PHG9_Scaffold_4570	5	p2	(AG)7	14	5041	5054	potentially variable
PHG9_Scaffold_4570	11	p2	(AT)6	12	15687	15698	
PHG9_Scaffold_4570	13	p2	(AG)8	16	18388	18403	potentially variable
PHG9_Scaffold_4570	16	p2	(TA)6	12	21899	21910	
PHG9_Scaffold_4570	29	p2	(TC)7	14	57445	57458	potentially variable
PHG9_Scaffold_4570	36	p2	(CG)6	12	71778	71789	
PHG9_Scaffold_4571	4	p2	(TA)7	14	27592	27605	potentially variable
PHG9_Scaffold_4571	9	p3	(GAA)5	15	39128	39142	potentially variable
PHG9_Scaffold_4571	23	p2	(TA)7	14	116828	116841	potentially variable
PHG9_Scaffold_4571	24	p2	(AG)6	12	127683	127694	
PHG9_Scaffold_4574	1	p2	(TA)6	12	4584	4595	
PHG9_Scaffold_4576	2	p2	(TG)6	12	5960	5971	
PHG9_Scaffold_4576	18	p2	(AT)7	14	45111	45124	potentially variable
PHG9_Scaffold_4576	26	p2	(AT)6	12	67682	67693	

PHG9_Scaffold_4576	28	p2	(TA)6	12	84100	84111	
PHG9_Scaffold_4576	29	p2	(AT)6	12	84430	84441	
PHG9_Scaffold_4577	9	p2	(GA)8	16	20371	20386	potentially variable
PHG9_Scaffold_4577	11	p2	(TA)6	12	25870	25881	
PHG9_Scaffold_4577	18	p2	(TA)6	12	47214	47225	
PHG9_Scaffold_4581	6	p2	(AT)6	12	9075	9086	
PHG9_Scaffold_4581	9	p2	(TA)6	12	20778	20789	
PHG9_Scaffold_4581	11	p2	(TC)8	16	21998	22013	potentially variable
PHG9_Scaffold_4581	12	p2	(CA)7	14	22403	22416	potentially variable
PHG9_Scaffold_4581	13	p2	(TC)7	14	22800	22813	potentially variable
PHG9_Scaffold_4581	15	p3	(ATT)5	15	27026	27040	potentially variable
PHG9_Scaffold_4582	8	p2	(AT)8	16	21363	21378	potentially variable
PHG9_Scaffold_4582	9	p2	(AG)9	18	27238	27255	potentially variable
PHG9_Scaffold_4582	23	p2	(CA)6	12	79069	79080	
PHG9_Scaffold_4583	4	p2	(AG)8	16	7315	7330	potentially variable
PHG9_Scaffold_4583	6	p3	(GGT)5	15	12913	12927	potentially variable
PHG9_Scaffold_4583	10	p3	(ATA)5	15	18756	18770	potentially variable
PHG9_Scaffold_4585	15	p2	(GA)6	12	46744	46755	
PHG9_Scaffold_4586	11	p2	(TC)6	12	22138	22149	

PHG9_Scaffold_4586	12	p3	(GGT)5	15	23352	23366	potentially variable
PHG9_Scaffold_4586	13	p2	(AT)6	12	24656	24667	
PHG9_Scaffold_4588	2	p2	(TA)6	12	12427	12438	
PHG9_Scaffold_4588	12	p3	(ATT)6	18	50781	50798	potentially variable
PHG9_Scaffold_4589	6	p2	(GA)9	18	38730	38747	potentially variable
PHG9_Scaffold_4589	7	p2	(AT)6	12	39327	39338	
PHG9_Scaffold_4590	4	p2	(TA)6	12	14693	14704	
PHG9_Scaffold_4590	9	p2	(GA)7	14	32024	32037	potentially variable
PHG9_Scaffold_4590	13	p2	(TG)8	16	68937	68952	potentially variable
PHG9_Scaffold_4590	23	p3	(CTT)5	15	92749	92763	potentially variable
PHG9_Scaffold_4590	43	p3	(TCT)5	15	160966	160980	potentially variable
PHG9_Scaffold_4590	46	p3	(CAA)5	15	174098	174112	potentially variable
PHG9_Scaffold_4590	47	p3	(TAT)6	18	175008	175025	potentially variable
PHG9_Scaffold_4590	52	p3	(GAA)5	15	186775	186789	potentially variable
PHG9_Scaffold_4590	53	p2	(CA)6	12	198982	198993	
PHG9_Scaffold_4591	3	p2	(AT)6	12	11563	11574	
PHG9_Scaffold_4591	12	p2	(TA)7	14	30981	30994	potentially variable
PHG9_Scaffold_4592	7	p2	(CT)6	12	24304	24315	

PHG9_Scaffold_4593	2	p2	(AT)6	12	710	721	
PHG9_Scaffold_4593	4	p2	(TA)7	14	2834	2847	potentially variable
PHG9_Scaffold_4593	13	p2	(AG)6	12	16200	16211	
PHG9_Scaffold_4593	15	p2	(TA)8	16	22017	22032	potentially variable
PHG9_Scaffold_4594	4	p2	(CA)6	12	7122	7133	
PHG9_Scaffold_4594	8	p2	(AT)6	12	25347	25358	
PHG9_Scaffold_4595	14	p3	(TTC)5	15	32789	32803	potentially variable
PHG9_Scaffold_4595	15	p2	(AG)6	12	34786	34797	
PHG9_Scaffold_4596	3	p2	(AT)7	14	729	742	potentially variable
PHG9_Scaffold_4596	4	p3	(AAT)5	15	1723	1737	potentially variable
PHG9_Scaffold_4596	6	p2	(AT)6	12	3207	3218	
PHG9_Scaffold_4596	11	p2	(TA)7	14	16177	16190	potentially variable
PHG9_Scaffold_4596	18	p2	(AT)6	12	27481	27492	
PHG9_Scaffold_4596	26	p2	(TG)6	12	62935	62946	
PHG9_Scaffold_4596	36	p2	(TA)7	14	108885	108898	potentially variable
PHG9_Scaffold_4596	47	p3	(ATT)5	15	147494	147508	potentially variable
PHG9_Scaffold_4596	60	p3	(TAT)5	15	174930	174944	potentially variable
PHG9_Scaffold_4596	65	p2	(TA)10	20	186850	186869	Hypervariable
PHG9_Scaffold_4596	66	p2	(TA)8	16	187232	187247	potentially variable

PHG9_Scaffold_4596	75	p2	(AG)6	12	214332	214343	
PHG9_Scaffold_4596	80	p2	(TA)7	14	229819	229832	potentially variable
PHG9_Scaffold_4598	3	p3	(CAC)5	15	7237	7251	potentially variable
PHG9_Scaffold_4598	12	p3	(ACC)5	15	30903	30917	potentially variable
PHG9_Scaffold_4598	17	p2	(TA)8	16	47378	47393	potentially variable
PHG9_Scaffold_4598	24	p3	(AGA)5	15	60679	60693	potentially variable
PHG9_Scaffold_4598	26	p2	(TA)6	12	64650	64661	
PHG9_Scaffold_4598	27	p3	(GGA)5	15	65267	65281	potentially variable
PHG9_Scaffold_4598	29	p2	(TC)7	14	75448	75461	potentially variable
PHG9_Scaffold_4598	42	p3	(AAT)5	15	117930	117944	potentially variable
PHG9_Scaffold_4602	5	p2	(CT)6	12	7136	7147	
PHG9_Scaffold_4602	7	p3	(AAT)6	18	11208	11225	potentially variable
PHG9_Scaffold_4602	10	p2	(TA)6	12	17160	17171	
PHG9_Scaffold_4602	14	p2	(TA)7	14	22946	22959	potentially variable
PHG9_Scaffold_4602	22	p3	(ATA)5	15	31828	31842	potentially variable
PHG9_Scaffold_4608	5	p3	(AAT)5	15	17628	17642	potentially variable
PHG9_Scaffold_4608	6	p2	(AT)9	18	18430	18447	potentially variable

PHG9_Scaffold_4609	1	p2	(AT)7	14	3179	3192	potentially variable
PHG9_Scaffold_4609	5	p3	(TTA)5	15	16150	16164	potentially variable
PHG9_Scaffold_4609	9	p2	(TA)7	14	31515	31528	potentially variable
PHG9_Scaffold_4609	10	p2	(AT)6	12	33776	33787	
PHG9_Scaffold_4611	7	p6	(CATAAA)5	30	19777	19806	Hypervariable
PHG9_Scaffold_4611	9	p3	(GAA)5	15	30095	30109	potentially variable
PHG9_Scaffold_4611	10	p2	(AG)6	12	31168	31179	
PHG9_Scaffold_4611	16	p2	(AG)6	12	53689	53700	
PHG9_Scaffold_4611	17	p2	(TC)8	16	57230	57245	potentially variable
PHG9_Scaffold_4612	4	p2	(TC)7	14	17026	17039	potentially variable
PHG9_Scaffold_4612	8	p2	(TA)8	16	28649	28664	potentially variable
PHG9_Scaffold_4615	11	p2	(AT)8	16	46868	46883	potentially variable
PHG9_Scaffold_4616	1	p3	(AGA)5	15	2878	2892	potentially variable
PHG9_Scaffold_4617	2	p2	(AT)7	14	4720	4733	potentially variable
PHG9_Scaffold_4619	2	p2	(TC)9	18	5434	5451	potentially variable
PHG9_Scaffold_4619	3	p2	(TA)6	12	8286	8297	
PHG9_Scaffold_4619	7	p2	(AT)8	16	15254	15269	potentially variable
PHG9_Scaffold_4621	1	p3	(TAT)5	15	531	545	potentially

							variable
PHG9_Scaffold_4622	5	p2	(AT)6	12	8254	8265	
PHG9_Scaffold_4622	10	p2	(GA)7	14	27520	27533	potentially variable
PHG9_Scaffold_4622	12	p3	(AGC)5	15	31126	31140	potentially variable
PHG9_Scaffold_4622	17	p3	(AAT)5	15	41908	41922	potentially variable
PHG9_Scaffold_4622	19	p2	(TA)6	12	49976	49987	
PHG9_Scaffold_4622	21	p2	(TA)8	16	50385	50400	potentially variable
PHG9_Scaffold_4627	12	p3	(AAT)5	15	81036	81050	potentially variable
PHG9_Scaffold_4629	3	p2	(AT)6	12	25741	25752	
PHG9_Scaffold_4629	12	p2	(TA)7	14	48849	48862	potentially variable
PHG9_Scaffold_4632	7	p2	(TA)7	14	3843	3856	potentially variable
PHG9_Scaffold_4632	12	p2	(TA)7	14	45824	45837	potentially variable
PHG9_Scaffold_4632	17	p2	(TG)6	12	102113	102124	
PHG9_Scaffold_4632	19	p2	(AT)6	12	112085	112096	
PHG9_Scaffold_4634	1	p2	(TA)6	12	10530	10541	
PHG9_Scaffold_4634	6	p2	(AT)6	12	31120	31131	
PHG9_Scaffold_4634	19	p2	(TA)7	14	84458	84471	potentially variable
PHG9_Scaffold_4634	22	p2	(TA)9	18	91765	91782	potentially variable
PHG9_Scaffold_4634	34	p3	(TTA)5	15	124216	124230	potentially

							variable
PHG9_Scaffold_4634	35	p2	(TA)8	16	127036	127051	potentially variable
PHG9_Scaffold_4634	42	p2	(AT)6	12	148744	148755	
PHG9_Scaffold_4638	1	p2	(AT)8	16	10560	10575	potentially variable
PHG9_Scaffold_4638	5	p2	(CT)7	14	29850	29863	potentially variable
PHG9_Scaffold_4640	1	p3	(TCA)5	15	2000	2014	potentially variable
PHG9_Scaffold_4640	4	p2	(AT)9	18	5120	5137	potentially variable
PHG9_Scaffold_4640	7	p3	(TTA)6	18	8409	8426	potentially variable
PHG9_Scaffold_4640	25	p2	(AC)6	12	52557	52568	
PHG9_Scaffold_4641	2	p2	(AT)8	16	17454	17469	potentially variable
PHG9_Scaffold_4641	7	p2	(AT)6	12	27233	27244	
PHG9_Scaffold_4645	2	p2	(AT)6	12	9770	9781	
PHG9_Scaffold_4646	3	p2	(TA)9	18	8603	8620	potentially variable
PHG9_Scaffold_4646	4	p2	(TA)6	12	25105	25116	
PHG9_Scaffold_4651	1	p2	(TA)9	18	2046	2063	potentially variable
PHG9_Scaffold_4651	3	p2	(AT)6	12	5875	5886	
PHG9_Scaffold_4651	4	p2	(AT)9	18	6981	6998	potentially variable
PHG9_Scaffold_4651	5	p2	(AT)8	16	7744	7759	potentially variable

PHG9_Scaffold_4651	7	p2	(AT)8	16	10431	10446	potentially variable
PHG9_Scaffold_4651	10	p2	(AT)7	14	12898	12911	potentially variable
PHG9_Scaffold_4651	11	p3	(GAA)5	15	14278	14292	potentially variable
PHG9_Scaffold_4651	14	p2	(GA)6	12	27795	27806	
PHG9_Scaffold_4651	27	p2	(TA)9	18	75486	75503	potentially variable
PHG9_Scaffold_4651	28	p3	(ACT)6	18	83034	83051	potentially variable
PHG9_Scaffold_4655	5	p3	(AGC)5	15	23454	23468	potentially variable
PHG9_Scaffold_4656	3	p2	(AT)6	12	14710	14721	
PHG9_Scaffold_4656	18	p2	(TA)8	16	108524	108539	potentially variable
PHG9_Scaffold_4656	22	p2	(AG)6	12	120280	120291	
PHG9_Scaffold_4656	38	p3	(ACA)5	15	172110	172124	potentially variable
PHG9_Scaffold_4656	41	p2	(CA)6	12	198395	198406	
PHG9_Scaffold_4656	43	p2	(AT)6	12	223682	223693	
PHG9_Scaffold_4659	2	p2	(TA)7	14	840	853	potentially variable
PHG9_Scaffold_4659	7	p3	(TAC)5	15	28891	28905	potentially variable
PHG9_Scaffold_4659	8	p3	(CCA)5	15	55183	55197	potentially variable
PHG9_Scaffold_4659	12	p2	(GA)7	14	63968	63981	potentially variable
PHG9_Scaffold_4660	4	p2	(AT)7	14	7072	7085	potentially

							variable
PHG9_Scaffold_4660	8	p3	(AAC)6	18	20972	20989	potentially variable
PHG9_Scaffold_4666	4	p2	(AG)6	12	15860	15871	
PHG9_Scaffold_4666	5	p2	(AG)6	12	17570	17581	
PHG9_Scaffold_4666	18	p3	(CAT)6	18	77171	77188	potentially variable
PHG9_Scaffold_4666	21	p3	(ATT)5	15	82027	82041	potentially variable
PHG9_Scaffold_4666	32	p2	(TA)6	12	115108	115119	
PHG9_Scaffold_4666	33	p2	(AG)8	16	119794	119809	potentially variable
PHG9_Scaffold_4666	34	p3	(AGC)5	15	126423	126437	potentially variable
PHG9_Scaffold_4666	37	p2	(AT)6	12	127905	127916	
PHG9_Scaffold_4666	43	p3	(TTA)5	15	147255	147269	potentially variable
PHG9_Scaffold_4666	44	p2	(TC)7	14	159446	159459	potentially variable
PHG9_Scaffold_4667	4	p2	(AT)6	12	8429	8440	
PHG9_Scaffold_4667	11	p2	(TA)7	14	29910	29923	potentially variable
PHG9_Scaffold_4667	20	p2	(TA)7	14	47531	47544	potentially variable
PHG9_Scaffold_4667	21	p3	(TTG)6	18	51136	51153	potentially variable
PHG9_Scaffold_4667	35	p2	(TA)8	16	90173	90188	potentially variable
PHG9_Scaffold_4667	36	p3	(TGG)5	15	94647	94661	potentially variable

PHG9_Scaffold_4667	39	p3	(TTC)5	15	99159	99173	potentially variable
PHG9_Scaffold_4667	42	p2	(AT)9	18	113750	113767	potentially variable
PHG9_Scaffold_4667	48	p6	(TGTTGG)5	30	133557	133586	Hypervariable
PHG9_Scaffold_4667	50	p2	(TG)6	12	139640	139651	
PHG9_Scaffold_4667	51	p2	(CT)6	12	149255	149266	
PHG9_Scaffold_4667	55	p3	(TTA)5	15	160532	160546	potentially variable
PHG9_Scaffold_4667	56	p3	(CGG)5	15	162043	162057	potentially variable
PHG9_Scaffold_4670	6	p2	(AT)7	14	14042	14055	potentially variable
PHG9_Scaffold_4670	7	p2	(TA)6	12	18258	18269	
PHG9_Scaffold_4671	3	p2	(TA)8	16	13516	13531	potentially variable
PHG9_Scaffold_4671	5	p2	(TA)6	12	25538	25549	
PHG9_Scaffold_4671	9	p2	(AT)7	14	47343	47356	potentially variable
PHG9_Scaffold_4671	15	p2	(AT)6	12	60606	60617	
PHG9_Scaffold_4671	19	p3	(CAA)5	15	74893	74907	potentially variable
PHG9_Scaffold_4671	25	p3	(TAT)5	15	93761	93775	potentially variable
PHG9_Scaffold_4678	3	p2	(TG)6	12	13143	13154	
PHG9_Scaffold_4679	3	p2	(AT)6	12	10946	10957	
PHG9_Scaffold_4679	23	p3	(CTG)5	15	76271	76285	potentially variable
PHG9_Scaffold_4679	24	p3	(TTC)5	15	79084	79098	potentially

							variable
PHG9_Scaffold_4680	1	p2	(CA)6	12	3833	3844	
PHG9_Scaffold_4690	4	p2	(CA)6	12	12666	12677	
PHG9_Scaffold_4690	5	p2	(TA)7	14	13364	13377	potentially variable
PHG9_Scaffold_4690	6	p2	(TA)6	12	13537	13548	
PHG9_Scaffold_4706	4	p2	(TA)6	12	16484	16495	
PHG9_Scaffold_4706	17	p2	(TG)6	12	80565	80576	
PHG9_Scaffold_4722	1	p2	(TA)7	14	259	272	potentially variable
PHG9_Scaffold_4722	2	p3	(ATA)5	15	928	942	potentially variable
PHG9_Scaffold_4726	1	p2	(TA)6	12	258	269	
PHG9_Scaffold_4727	4	p2	(AT)6	12	260427	260438	
PHG9_Scaffold_4727	19	p2	(TA)7	14	436643	436656	potentially variable
PHG9_Scaffold_4727	21	p3	(TTA)5	15	443889	443903	potentially variable
PHG9_Scaffold_4728	3	p2	(AT)8	16	645234	645249	potentially variable
PHG9_Scaffold_4728	7	p2	(TA)8	16	676640	676655	potentially variable
PHG9_Scaffold_4732	7	p2	(AT)7	14	52609	52622	potentially variable
PHG9_Scaffold_4734	1	p3	(ATT)5	15	619	633	potentially variable
PHG9_Scaffold_4735	3	p2	(TA)6	12	42639	42650	
PHG9_Scaffold_4735	6	p2	(TA)8	16	109638	109653	potentially variable

PHG9_Scaffold_4745	1	p2	(AT)9	18	46979	46996	potentially variable
PHG9_Scaffold_4745	12	p2	(TA)7	14	134119	134132	potentially variable
PHG9_Scaffold_4745	16	p3	(AGG)6	18	154583	154600	potentially variable
PHG9_Scaffold_4745	20	p3	(TGT)5	15	166851	166865	potentially variable
PHG9_Scaffold_4745	22	p2	(AT)7	14	195243	195256	potentially variable
PHG9_Scaffold_4745	23	p2	(TC)6	12	209812	209823	
PHG9_Scaffold_4746	4	p2	(AT)6	12	53852	53863	
PHG9_Scaffold_4746	7	p2	(TA)6	12	73384	73395	
PHG9_Scaffold_4749	14	p3	(TTA)5	15	28093	28107	potentially variable
PHG9_Scaffold_4749	23	p3	(TCT)5	15	55518	55532	potentially variable
PHG9_Scaffold_4749	25	p2	(TA)6	12	60691	60702	
PHG9_Scaffold_4749	35	p3	(ATG)6	18	75324	75341	potentially variable
PHG9_Scaffold_4749	52	p2	(TA)6	12	112010	112021	
PHG9_Scaffold_4749	61	p2	(AT)7	14	146315	146328	potentially variable
PHG9_Scaffold_4749	64	p3	(ATT)5	15	149131	149145	potentially variable
PHG9_Scaffold_4749	76	p2	(TA)6	12	213920	213931	
PHG9_Scaffold_4749	80	p3	(GCT)5	15	226411	226425	potentially variable
PHG9_Scaffold_4749	85	p2	(AT)9	18	243810	243827	potentially variable

PHG9_Scaffold_4749	86	p2	(AT)6	12	249975	249986	
PHG9_Scaffold_4749	88	p3	(ACA)6	18	261984	262001	potentially variable
PHG9_Scaffold_4749	92	p2	(TA)7	14	267905	267918	potentially variable
PHG9_Scaffold_4749	99	p3	(TAT)6	18	294107	294124	potentially variable
PHG9_Scaffold_4749	106	p2	(TA)8	16	320246	320261	potentially variable
PHG9_Scaffold_4751	1	p2	(TC)6	12	12146	12157	
PHG9_Scaffold_4751	2	p2	(TA)7	14	18731	18744	potentially variable
PHG9_Scaffold_4751	19	p2	(CT)7	14	80058	80071	potentially variable
PHG9_Scaffold_4751	20	p2	(CT)6	12	80434	80445	
PHG9_Scaffold_4751	21	p2	(AT)8	16	85915	85930	potentially variable
PHG9_Scaffold_4751	26	p2	(AT)6	12	112499	112510	
PHG9_Scaffold_4751	35	p2	(CT)9	18	145392	145409	potentially variable
PHG9_Scaffold_4751	49	p3	(AAT)5	15	172993	173007	potentially variable
PHG9_Scaffold_4751	54	p2	(TC)6	12	194021	194032	
PHG9_Scaffold_4751	56	p2	(TG)6	12	199217	199228	
PHG9_Scaffold_4751	58	p2	(GA)7	14	202240	202253	potentially variable
PHG9_Scaffold_4752	4	p2	(TA)7	14	161858	161871	potentially variable
PHG9_Scaffold_4754	3	p2	(CT)7	14	9172	9185	potentially variable

PHG9_Scaffold_4754	4	p3	(TAG)5	15	15668	15682	potentially variable
PHG9_Scaffold_4754	10	p3	(AAT)5	15	36569	36583	potentially variable
PHG9_Scaffold_4754	13	p2	(AT)6	12	38228	38239	
PHG9_Scaffold_4754	14	p3	(TTA)5	15	39350	39364	potentially variable
PHG9_Scaffold_4754	15	p3	(TTA)5	15	41407	41421	potentially variable
PHG9_Scaffold_4754	22	p2	(AG)6	12	64566	64577	
PHG9_Scaffold_4754	25	p2	(TC)7	14	82798	82811	potentially variable
PHG9_Scaffold_4754	26	p2	(AT)7	14	83925	83938	potentially variable
PHG9_Scaffold_4754	27	p2	(TA)8	16	85081	85096	potentially variable
PHG9_Scaffold_4754	30	p2	(TA)6	12	109049	109060	
PHG9_Scaffold_4754	35	p3	(GAT)5	15	126570	126584	potentially variable
PHG9_Scaffold_4754	48	p2	(TC)9	18	158696	158713	potentially variable
PHG9_Scaffold_4754	51	p2	(AT)6	12	164884	164895	
PHG9_Scaffold_4754	52	p3	(TAT)6	18	165877	165894	potentially variable
PHG9_Scaffold_4754	53	p2	(TC)7	14	173274	173287	potentially variable
PHG9_Scaffold_4755	15	p3	(CTT)5	15	57421	57435	potentially variable
PHG9_Scaffold_4755	16	p3	(CAT)5	15	58639	58653	potentially variable

PHG9_Scaffold_4755	35	p2	(AG)7	14	117211	117224	potentially variable
PHG9_Scaffold_4755	51	p2	(AT)8	16	172113	172128	potentially variable
PHG9_Scaffold_4755	62	p3	(TAA)5	15	200940	200954	potentially variable
PHG9_Scaffold_4758	9	p3	(ATA)6	18	11416	11433	potentially variable
PHG9_Scaffold_4758	17	p2	(TC)7	14	106595	106608	potentially variable
PHG9_Scaffold_4758	22	p2	(AT)9	18	173127	173144	potentially variable
PHG9_Scaffold_4759	4	p2	(AT)9	18	18678	18695	potentially variable
PHG9_Scaffold_4760	4	p2	(AT)8	16	67214	67229	potentially variable
PHG9_Scaffold_4760	9	p3	(TGT)5	15	116567	116581	potentially variable
PHG9_Scaffold_4760	10	p2	(CT)7	14	116924	116937	potentially variable
PHG9_Scaffold_4760	22	p3	(ATG)5	15	161899	161913	potentially variable
PHG9_Scaffold_4761	4	p2	(AT)7	14	19502	19515	potentially variable
PHG9_Scaffold_4761	12	p2	(GT)6	12	77627	77638	
PHG9_Scaffold_4763	8	p2	(AT)9	18	14475	14492	potentially variable
PHG9_Scaffold_4763	28	p3	(TTA)5	15	79521	79535	potentially variable
PHG9_Scaffold_4763	40	p2	(AG)7	14	121687	121700	potentially

							variable
PHG9_Scaffold_4763	41	p2	(AT)8	16	122734	122749	potentially variable
PHG9_Scaffold_4763	45	p2	(GT)7	14	135982	135995	potentially variable
PHG9_Scaffold_4763	54	p2	(AT)7	14	167922	167935	potentially variable
PHG9_Scaffold_4764	7	p2	(AG)9	18	26569	26586	potentially variable
PHG9_Scaffold_4764	9	p2	(CA)6	12	27561	27572	
PHG9_Scaffold_4764	10	p3	(AAT)5	15	28433	28447	potentially variable
PHG9_Scaffold_4764	15	p2	(TA)7	14	38204	38217	potentially variable
PHG9_Scaffold_4764	16	p2	(TA)6	12	38723	38734	
PHG9_Scaffold_4764	17	p2	(AG)6	12	39032	39043	
PHG9_Scaffold_4764	22	p2	(AG)6	12	55230	55241	
PHG9_Scaffold_4764	26	p2	(CA)9	18	62127	62144	potentially variable
PHG9_Scaffold_4764	27	p2	(CT)9	18	63610	63627	potentially variable
PHG9_Scaffold_4764	28	p2	(AT)7	14	65945	65958	potentially variable
PHG9_Scaffold_4764	31	p3	(TGC)5	15	69959	69973	potentially variable
PHG9_Scaffold_4764	41	p2	(AT)6	12	94803	94814	
PHG9_Scaffold_4764	47	p2	(TA)8	16	115753	115768	potentially variable
PHG9_Scaffold_4764	56	p2	(AT)8	16	144387	144402	potentially variable

PHG9_Scaffold_4764	66	p2	(AG)9	18	172822	172839	potentially variable
PHG9_Scaffold_4764	69	p2	(GA)7	14	186109	186122	potentially variable
PHG9_Scaffold_4764	73	p2	(GA)7	14	200794	200807	potentially variable
PHG9_Scaffold_4764	82	p2	(TA)6	12	229835	229846	
PHG9_Scaffold_4764	90	p2	(TA)9	18	244462	244479	potentially variable
PHG9_Scaffold_4764	98	p3	(ATA)5	15	252804	252818	potentially variable
PHG9_Scaffold_4764	100	p2	(AT)8	16	268397	268412	potentially variable
PHG9_Scaffold_4765	3	p3	(ATT)5	15	31150	31164	potentially variable
PHG9_Scaffold_4765	7	p2	(TA)6	12	54953	54964	
PHG9_Scaffold_4765	16	p3	(AAT)5	15	100846	100860	potentially variable
PHG9_Scaffold_4765	20	p2	(AT)6	12	105661	105672	
PHG9_Scaffold_4765	28	p2	(TA)7	14	130655	130668	potentially variable
PHG9_Scaffold_4765	32	p2	(AT)6	12	134418	134429	
PHG9_Scaffold_4769	3	p3	(AAT)5	15	20766	20780	potentially variable
PHG9_Scaffold_4769	5	p2	(AT)9	18	44218	44235	potentially variable
PHG9_Scaffold_4771	8	p3	(TGC)5	15	23227	23241	potentially variable
PHG9_Scaffold_4771	13	p2	(AG)6	12	47779	47790	
PHG9_Scaffold_4771	16	p3	(AAT)5	15	56616	56630	potentially

							variable
PHG9_Scaffold_4771	17	p2	(TA)6	12	57701	57712	
PHG9_Scaffold_4771	23	p2	(TG)6	12	65621	65632	
PHG9_Scaffold_4771	24	p2	(AT)6	12	74432	74443	
PHG9_Scaffold_4771	26	p2	(AT)7	14	87504	87517	potentially variable
PHG9_Scaffold_4771	33	p2	(AC)9	18	102809	102826	potentially variable
PHG9_Scaffold_4771	44	p3	(TTA)5	15	135122	135136	potentially variable
PHG9_Scaffold_4771	49	p2	(AT)7	14	145631	145644	potentially variable
PHG9_Scaffold_4771	50	p2	(TA)8	16	147138	147153	potentially variable
PHG9_Scaffold_4772	3	p3	(TAA)5	15	1556	1570	potentially variable
PHG9_Scaffold_4772	5	p2	(AT)6	12	6752	6763	
PHG9_Scaffold_4772	15	p2	(AT)8	16	58215	58230	potentially variable
PHG9_Scaffold_4772	22	p2	(TA)6	12	63180	63191	
PHG9_Scaffold_4772	29	p2	(TA)8	16	78603	78618	potentially variable
PHG9_Scaffold_4772	33	p2	(TC)9	18	87596	87613	potentially variable
PHG9_Scaffold_4772	34	p3	(TTA)6	18	93173	93190	potentially variable
PHG9_Scaffold_4772	35	p3	(CAT)6	18	96572	96589	potentially variable
PHG9_Scaffold_4772	44	p3	(TAT)5	15	127855	127869	potentially variable

PHG9_Scaffold_4772	45	p2	(AT)6	12	128939	128950	
PHG9_Scaffold_4772	46	p3	(TTG)6	18	129968	129985	potentially variable
PHG9_Scaffold_4772	49	p3	(TCA)5	15	136014	136028	potentially variable
PHG9_Scaffold_4772	50	p3	(CAA)5	15	136324	136338	potentially variable
PHG9_Scaffold_4772	55	p2	(CT)9	18	147891	147908	potentially variable
PHG9_Scaffold_4772	56	p2	(TA)7	14	150917	150930	potentially variable
PHG9_Scaffold_4772	60	p2	(AT)7	14	164040	164053	potentially variable
PHG9_Scaffold_4774	4	p2	(TA)8	16	15740	15755	potentially variable
PHG9_Scaffold_4774	9	p2	(TA)6	12	64446	64457	
PHG9_Scaffold_4774	12	p3	(GGT)5	15	81373	81387	potentially variable
PHG9_Scaffold_4774	13	p2	(AT)7	14	83413	83426	potentially variable
PHG9_Scaffold_4774	16	p2	(TA)6	12	87963	87974	
PHG9_Scaffold_4774	18	p2	(TA)6	12	99877	99888	
PHG9_Scaffold_4777	1	p2	(TA)7	14	14221	14234	potentially variable
PHG9_Scaffold_4778	7	p2	(TA)8	16	18098	18113	potentially variable
PHG9_Scaffold_4778	12	p2	(GA)7	14	34589	34602	potentially variable
PHG9_Scaffold_4778	13	p3	(TAT)5	15	36662	36676	potentially variable

PHG9_Scaffold_4778	14	p3	(TAT)5	15	37479	37493	potentially variable
PHG9_Scaffold_4778	25	p2	(TA)7	14	56984	56997	potentially variable
PHG9_Scaffold_4778	30	p2	(TA)9	18	65594	65611	potentially variable
PHG9_Scaffold_4778	32	p2	(AG)6	12	69092	69103	
PHG9_Scaffold_4778	34	p3	(ATT)5	15	71605	71619	potentially variable
PHG9_Scaffold_4778	39	p2	(AT)7	14	79455	79468	potentially variable
PHG9_Scaffold_4778	40	p3	(GGT)5	15	84257	84271	potentially variable
PHG9_Scaffold_4781	1	p3	(AAG)5	15	32533	32547	potentially variable
PHG9_Scaffold_4781	2	p3	(AAG)5	15	49059	49073	potentially variable
PHG9_Scaffold_4783	10	p2	(TA)9	18	105452	105469	potentially variable
PHG9_Scaffold_4784	3	p2	(AT)6	12	12771	12782	
PHG9_Scaffold_4784	4	p2	(TC)6	12	19881	19892	
PHG9_Scaffold_4784	9	p3	(CTT)5	15	33152	33166	potentially variable
PHG9_Scaffold_4784	14	p3	(ATA)5	15	45826	45840	potentially variable
PHG9_Scaffold_4784	21	p3	(TAT)5	15	69800	69814	potentially variable
PHG9_Scaffold_4784	39	p3	(ACT)5	15	105773	105787	potentially variable
PHG9_Scaffold_4784	45	p2	(AG)8	16	138989	139004	potentially

							variable
PHG9_Scaffold_4784	47	p2	(TC)9	18	155665	155682	potentially variable
PHG9_Scaffold_4784	51	p3	(ATT)5	15	166016	166030	potentially variable
PHG9_Scaffold_4784	62	p2	(TA)9	18	201671	201688	potentially variable
PHG9_Scaffold_4784	66	p3	(CTC)5	15	206759	206773	potentially variable
PHG9_Scaffold_4784	67	p2	(CT)6	12	207744	207755	
PHG9_Scaffold_4784	68	p3	(GAA)5	15	210367	210381	potentially variable
PHG9_Scaffold_4784	69	p3	(CTT)6	18	211113	211130	potentially variable
PHG9_Scaffold_4784	74	p2	(AT)7	14	222706	222719	potentially variable
PHG9_Scaffold_4785	2	p2	(AC)7	14	10775	10788	potentially variable
PHG9_Scaffold_4785	3	p2	(TA)7	14	13785	13798	potentially variable
PHG9_Scaffold_4785	11	p2	(TA)6	12	36207	36218	
PHG9_Scaffold_4785	24	p2	(AT)6	12	104534	104545	
PHG9_Scaffold_4786	4	p3	(AAG)5	15	18477	18491	potentially variable
PHG9_Scaffold_4786	8	p2	(AT)6	12	27209	27220	
PHG9_Scaffold_4786	12	p3	(ATT)5	15	52557	52571	potentially variable
PHG9_Scaffold_4786	13	p2	(TA)8	16	69141	69156	potentially variable
PHG9_Scaffold_4786	14	p2	(TC)8	16	70595	70610	potentially

							variable
PHG9_Scaffold_4786	28	p2	(AT)8	16	142621	142636	potentially variable
PHG9_Scaffold_4788	8	p2	(TA)6	12	21626	21637	
PHG9_Scaffold_4788	19	p2	(AT)7	14	38507	38520	potentially variable
PHG9_Scaffold_4788	33	p2	(TG)8	16	72321	72336	potentially variable
PHG9_Scaffold_4788	34	p2	(TA)7	14	73643	73656	potentially variable
PHG9_Scaffold_4788	38	p2	(TG)6	12	86145	86156	
PHG9_Scaffold_4788	48	p2	(TA)6	12	114718	114729	
PHG9_Scaffold_4788	50	p2	(TA)9	18	121670	121687	potentially variable
PHG9_Scaffold_4788	60	p2	(TA)6	12	136743	136754	
PHG9_Scaffold_4788	62	p2	(TC)7	14	147363	147376	potentially variable
PHG9_Scaffold_4788	67	p2	(AT)6	12	152058	152069	
PHG9_Scaffold_4789	2	p3	(AAT)5	15	5769	5783	potentially variable
PHG9_Scaffold_4789	6	p2	(TG)7	14	17443	17456	potentially variable
PHG9_Scaffold_4789	13	p3	(TGC)5	15	38300	38314	potentially variable
PHG9_Scaffold_4789	18	p2	(GA)7	14	52965	52978	potentially variable
PHG9_Scaffold_4789	32	p3	(TAT)5	15	90312	90326	potentially variable
PHG9_Scaffold_4790	9	p2	(AT)6	12	34907	34918	

PHG9_Scaffold_4790	10	p2	(AG)7	14	41584	41597	potentially variable
PHG9_Scaffold_4790	13	p2	(TA)7	14	143231	143244	potentially variable
PHG9_Scaffold_4791	5	p3	(TAA)5	15	36429	36443	potentially variable
PHG9_Scaffold_4791	17	p2	(TA)7	14	82090	82103	potentially variable
PHG9_Scaffold_4791	21	p2	(GA)7	14	105924	105937	potentially variable
PHG9_Scaffold_4791	23	p2	(TA)6	12	113876	113887	
PHG9_Scaffold_4792	3	p3	(TAT)6	18	6956	6973	potentially variable
PHG9_Scaffold_4792	12	p2	(AT)6	12	105919	105930	
PHG9_Scaffold_4792	18	p3	(AAT)5	15	126192	126206	potentially variable
PHG9_Scaffold_4792	19	p2	(TC)6	12	131391	131402	
PHG9_Scaffold_4792	21	p2	(TA)6	12	140903	140914	
PHG9_Scaffold_4792	31	p2	(TC)6	12	237803	237814	
PHG9_Scaffold_4792	34	p2	(AT)6	12	253035	253046	
PHG9_Scaffold_4792	38	p2	(AT)6	12	273625	273636	
PHG9_Scaffold_4793	1	p2	(TA)6	12	208	219	
PHG9_Scaffold_4793	2	p2	(TA)7	14	458	471	potentially variable
PHG9_Scaffold_4793	5	p2	(GA)6	12	9934	9945	
PHG9_Scaffold_4793	14	p2	(TA)7	14	24222	24235	potentially variable
PHG9_Scaffold_4793	22	p2	(TA)7	14	39140	39153	potentially variable

PHG9_Scaffold_4793	28	p2	(TA)8	16	61794	61809	potentially variable
PHG9_Scaffold_4793	32	p2	(GA)6	12	67152	67163	
PHG9_Scaffold_4795	2	p3	(ATC)5	15	2023	2037	potentially variable
PHG9_Scaffold_4796	3	p3	(AGA)5	15	5725	5739	potentially variable
PHG9_Scaffold_4796	9	p3	(TTC)5	15	14682	14696	potentially variable
PHG9_Scaffold_4796	11	p3	(TTC)5	15	16835	16849	potentially variable
PHG9_Scaffold_4796	18	p2	(AT)6	12	35713	35724	
PHG9_Scaffold_4796	20	p2	(TA)6	12	46129	46140	
PHG9_Scaffold_4797	1	p3	(TAA)5	15	2261	2275	potentially variable
PHG9_Scaffold_4797	4	p2	(TA)9	18	7730	7747	potentially variable
PHG9_Scaffold_4797	6	p3	(GGA)5	15	12038	12052	potentially variable
PHG9_Scaffold_4797	8	p2	(TC)6	12	14498	14509	
PHG9_Scaffold_4797	13	p3	(TCT)6	18	23318	23335	potentially variable
PHG9_Scaffold_4797	16	p2	(GA)6	12	24172	24183	
PHG9_Scaffold_4797	18	p2	(AG)8	16	43577	43592	potentially variable
PHG9_Scaffold_4797	19	p2	(AT)6	12	44712	44723	
PHG9_Scaffold_4797	31	p2	(TC)6	12	79359	79370	
PHG9_Scaffold_4797	34	p3	(CAT)5	15	84194	84208	potentially variable

PHG9_Scaffold_4797	41	p2	(AT)7	14	117161	117174	potentially variable
PHG9_Scaffold_4797	45	p2	(TA)7	14	132678	132691	potentially variable
PHG9_Scaffold_4799	5	p3	(TTA)6	18	26577	26594	potentially variable
PHG9_Scaffold_4799	6	p2	(AG)8	16	29717	29732	potentially variable
PHG9_Scaffold_4799	25	p2	(TA)6	12	71331	71342	
PHG9_Scaffold_4799	26	p2	(TA)8	16	71562	71577	potentially variable
PHG9_Scaffold_4799	33	p2	(TA)9	18	98199	98216	potentially variable
PHG9_Scaffold_4799	42	p2	(TA)6	12	116161	116172	
PHG9_Scaffold_4799	43	p2	(TA)8	16	123660	123675	potentially variable
PHG9_Scaffold_4799	47	p2	(AT)6	12	145903	145914	
PHG9_Scaffold_4799	58	p2	(AT)9	18	166977	166994	potentially variable
PHG9_Scaffold_4799	66	p2	(GA)6	12	231320	231331	
PHG9_Scaffold_4799	73	p3	(TAA)5	15	239862	239876	potentially variable
PHG9_Scaffold_4799	81	p3	(ATA)5	15	253968	253982	potentially variable
PHG9_Scaffold_4799	83	p2	(CT)6	12	275838	275849	
PHG9_Scaffold_4800	10	p2	(AT)6	12	25108	25119	
PHG9_Scaffold_4800	20	p2	(AT)6	12	68656	68667	
PHG9_Scaffold_4800	26	p2	(GA)6	12	98456	98467	
PHG9_Scaffold_4801	10	p3	(TGC)5	15	24669	24683	potentially

							variable
PHG9_Scaffold_4801	13	p3	(ATT)6	18	29072	29089	potentially variable
PHG9_Scaffold_4801	16	p2	(TA)6	12	48228	48239	
PHG9_Scaffold_4801	23	p3	(TAA)5	15	65721	65735	potentially variable
PHG9_Scaffold_4801	28	p2	(AC)6	12	79828	79839	
PHG9_Scaffold_4801	35	p2	(CT)8	16	112876	112891	potentially variable
PHG9_Scaffold_4802	4	p2	(TA)7	14	15972	15985	potentially variable
PHG9_Scaffold_4802	14	p2	(AT)6	12	104681	104692	
PHG9_Scaffold_4802	28	p2	(TG)6	12	167375	167386	
PHG9_Scaffold_4802	37	p2	(AT)8	16	201559	201574	potentially variable
PHG9_Scaffold_4802	40	p2	(TA)6	12	216624	216635	
PHG9_Scaffold_4802	45	p2	(CA)6	12	253110	253121	
PHG9_Scaffold_4802	47	p2	(TA)6	12	271650	271661	
PHG9_Scaffold_4802	54	p2	(TA)7	14	340532	340545	potentially variable
PHG9_Scaffold_4806	12	p3	(TTG)5	15	19836	19850	potentially variable
PHG9_Scaffold_4806	15	p2	(AT)7	14	27641	27654	potentially variable
PHG9_Scaffold_4806	18	p2	(TA)7	14	47526	47539	potentially variable
PHG9_Scaffold_4806	22	p2	(TA)8	16	79396	79411	potentially variable
PHG9_Scaffold_4807	2	p2	(AC)6	12	14083	14094	

PHG9_Scaffold_4807	3	p2	(TA)7	14	16226	16239	potentially variable
PHG9_Scaffold_4807	4	p2	(AT)9	18	20173	20190	potentially variable
PHG9_Scaffold_4807	10	p2	(TA)9	18	48164	48181	potentially variable
PHG9_Scaffold_4808	2	p3	(TGA)6	18	4051	4068	potentially variable
PHG9_Scaffold_4808	9	p2	(AT)6	12	31374	31385	
PHG9_Scaffold_4808	10	p2	(TA)7	14	36129	36142	potentially variable
PHG9_Scaffold_4808	11	p2	(TC)9	18	38933	38950	potentially variable
PHG9_Scaffold_4808	19	p3	(TCT)5	15	70868	70882	potentially variable
PHG9_Scaffold_4811	1	p3	(TCT)5	15	2235	2249	potentially variable
PHG9_Scaffold_4811	8	p2	(CA)6	12	42058	42069	
PHG9_Scaffold_4811	11	p2	(GA)7	14	51373	51386	potentially variable
PHG9_Scaffold_4811	13	p3	(GCT)5	15	53644	53658	potentially variable
PHG9_Scaffold_4811	16	p3	(GAA)5	15	67024	67038	potentially variable
PHG9_Scaffold_4811	18	p2	(AG)6	12	74244	74255	
PHG9_Scaffold_4811	22	p2	(TA)8	16	104163	104178	potentially variable
PHG9_Scaffold_4811	34	p2	(AT)8	16	131586	131601	potentially variable
PHG9_Scaffold_4811	37	p2	(TA)6	12	138735	138746	

PHG9_Scaffold_4811	47	p2	(AT)7	14	165010	165023	potentially variable
PHG9_Scaffold_4811	51	p2	(AT)9	18	189624	189641	potentially variable
PHG9_Scaffold_4812	2	p3	(TTA)6	18	5176	5193	potentially variable
PHG9_Scaffold_4812	5	p2	(TA)9	18	8001	8018	potentially variable
PHG9_Scaffold_4813	20	p3	(AAT)5	15	54664	54678	potentially variable
PHG9_Scaffold_4813	32	p6	(GAAAAC)5	30	82475	82504	Hypervariable
PHG9_Scaffold_4813	51	p2	(TA)8	16	116351	116366	potentially variable
PHG9_Scaffold_4813	54	p2	(AT)6	12	123366	123377	
PHG9_Scaffold_4814	1	p2	(AT)6	12	8085	8096	
PHG9_Scaffold_4814	3	p2	(AT)6	12	21112	21123	
PHG9_Scaffold_4814	4	p3	(CTT)5	15	22649	22663	potentially variable
PHG9_Scaffold_4814	14	p3	(ATA)5	15	52204	52218	potentially variable
PHG9_Scaffold_4814	17	p2	(TA)7	14	60219	60232	potentially variable
PHG9_Scaffold_4814	18	p2	(TA)7	14	63214	63227	potentially variable
PHG9_Scaffold_4814	22	p2	(TA)6	12	65216	65227	
PHG9_Scaffold_4814	25	p2	(AT)6	12	70093	70104	
PHG9_Scaffold_4814	26	p2	(AG)6	12	74106	74117	
PHG9_Scaffold_4814	27	p2	(AT)6	12	86313	86324	
PHG9_Scaffold_4814	28	p2	(TA)7	14	86826	86839	potentially

							variable
PHG9_Scaffold_4814	30	p2	(AG)7	14	97601	97614	potentially variable
PHG9_Scaffold_4815	5	p2	(AT)7	14	29186	29199	potentially variable
PHG9_Scaffold_4815	7	p6	(ATTTCT)5	30	39844	39873	Hypervariable
PHG9_Scaffold_4816	1	p2	(AT)6	12	1152	1163	
PHG9_Scaffold_4816	6	p2	(AT)8	16	15909	15924	potentially variable
PHG9_Scaffold_4816	9	p2	(AT)7	14	36224	36237	potentially variable
PHG9_Scaffold_4816	11	p3	(TGT)5	15	44658	44672	potentially variable
PHG9_Scaffold_4816	13	p2	(TA)7	14	46445	46458	potentially variable
PHG9_Scaffold_4816	27	p3	(ATT)5	15	80209	80223	potentially variable
PHG9_Scaffold_4816	30	p2	(AG)8	16	95700	95715	potentially variable
PHG9_Scaffold_4816	39	p2	(TA)6	12	117010	117021	
PHG9_Scaffold_4817	11	p2	(TA)7	14	26126	26139	potentially variable
PHG9_Scaffold_4817	15	p2	(AT)6	12	29601	29612	
PHG9_Scaffold_4818	5	p2	(AG)6	12	25268	25279	
PHG9_Scaffold_4818	18	p2	(TA)9	18	157162	157179	potentially variable
PHG9_Scaffold_4819	15	p3	(TTG)5	15	57569	57583	potentially variable
PHG9_Scaffold_4821	5	p2	(AT)8	16	83317	83332	potentially variable

PHG9_Scaffold_4822	1	p3	(TAT)5	15	220	234	potentially variable
PHG9_Scaffold_4822	2	p3	(TTA)6	18	6507	6524	potentially variable
PHG9_Scaffold_4822	9	p2	(TC)6	12	19781	19792	
PHG9_Scaffold_4822	12	p2	(GT)7	14	25798	25811	potentially variable
PHG9_Scaffold_4822	16	p2	(AC)6	12	42142	42153	
PHG9_Scaffold_4822	17	p3	(TCC)5	15	60188	60202	potentially variable
PHG9_Scaffold_4822	19	p2	(AC)6	12	69558	69569	
PHG9_Scaffold_4822	20	p2	(AT)9	18	75877	75894	potentially variable
PHG9_Scaffold_4823	13	p2	(TA)6	12	25409	25420	
PHG9_Scaffold_4823	15	p2	(AT)6	12	29402	29413	
PHG9_Scaffold_4823	18	p2	(CA)6	12	31862	31873	
PHG9_Scaffold_4823	25	p2	(AT)6	12	58466	58477	
PHG9_Scaffold_4823	45	p2	(TC)7	14	113023	113036	potentially variable
PHG9_Scaffold_4823	48	p2	(AT)8	16	130013	130028	potentially variable
PHG9_Scaffold_4823	49	p3	(TTC)5	15	132305	132319	potentially variable
PHG9_Scaffold_4823	54	p2	(AG)6	12	151158	151169	
PHG9_Scaffold_4823	56	p3	(AGA)5	15	167604	167618	potentially variable
PHG9_Scaffold_4825	3	p2	(CA)9	18	4297	4314	potentially variable
PHG9_Scaffold_4825	5	p2	(GA)9	18	7139	7156	potentially

							variable
PHG9_Scaffold_4825	9	p2	(TA)6	12	21169	21180	
PHG9_Scaffold_4825	17	p3	(ATT)6	18	38309	38326	potentially variable
PHG9_Scaffold_4826	1	p2	(CT)6	12	3170	3181	
PHG9_Scaffold_4826	2	p3	(AGC)5	15	8062	8076	potentially variable
PHG9_Scaffold_4826	4	p2	(TA)8	16	25596	25611	potentially variable
PHG9_Scaffold_4826	6	p2	(AT)8	16	34234	34249	potentially variable
PHG9_Scaffold_4826	11	p3	(TTA)5	15	43063	43077	potentially variable
PHG9_Scaffold_4826	13	p2	(AT)6	12	55276	55287	
PHG9_Scaffold_4826	14	p2	(TA)6	12	56918	56929	
PHG9_Scaffold_4826	27	p2	(AG)6	12	93334	93345	
PHG9_Scaffold_4826	28	p2	(AG)6	12	93964	93975	
PHG9_Scaffold_4827	8	p3	(AAG)5	15	28262	28276	potentially variable
PHG9_Scaffold_4827	12	p2	(AT)6	12	57400	57411	
PHG9_Scaffold_4827	22	p2	(AT)6	12	120464	120475	
PHG9_Scaffold_4827	25	p2	(TA)6	12	122613	122624	
PHG9_Scaffold_4829	8	p3	(AAG)5	15	40125	40139	potentially variable
PHG9_Scaffold_4829	19	p2	(GA)6	12	67852	67863	
PHG9_Scaffold_4829	21	p2	(AT)8	16	75000	75015	potentially variable
PHG9_Scaffold_4829	25	p3	(TTA)5	15	106913	106927	potentially variable

PHG9_Scaffold_4829	26	p2	(AT)8	16	113624	113639	potentially variable
PHG9_Scaffold_4829	35	p3	(ATG)5	15	130917	130931	potentially variable
PHG9_Scaffold_4829	38	p3	(AAT)5	15	142262	142276	potentially variable
PHG9_Scaffold_4829	43	p2	(TA)7	14	174932	174945	potentially variable
PHG9_Scaffold_4829	44	p3	(ATA)5	15	175073	175087	potentially variable
PHG9_Scaffold_4830	4	p3	(AAT)5	15	10760	10774	potentially variable
PHG9_Scaffold_4830	21	p2	(AT)8	16	112190	112205	potentially variable
PHG9_Scaffold_4831	9	p2	(AC)6	12	37368	37379	
PHG9_Scaffold_4831	33	p2	(GA)6	12	123557	123568	
PHG9_Scaffold_4831	37	p3	(ATA)5	15	131895	131909	potentially variable
PHG9_Scaffold_4831	39	p2	(TC)6	12	142900	142911	
PHG9_Scaffold_4831	41	p3	(TTA)5	15	148029	148043	potentially variable
PHG9_Scaffold_4831	48	p3	(TTC)6	18	162891	162908	potentially variable
PHG9_Scaffold_4831	54	p2	(AC)6	12	176923	176934	
PHG9_Scaffold_4831	56	p2	(AG)6	12	181347	181358	
PHG9_Scaffold_4834	2	p2	(AT)6	12	2765	2776	
PHG9_Scaffold_4834	3	p2	(AT)6	12	8011	8022	
PHG9_Scaffold_4834	16	p2	(TA)8	16	46492	46507	potentially variable

PHG9_Scaffold_4834	23	p2	(CA)6	12	68334	68345	
PHG9_Scaffold_4834	24	p2	(AT)7	14	75482	75495	potentially variable
PHG9_Scaffold_4834	25	p3	(AGC)6	18	76347	76364	potentially variable
PHG9_Scaffold_4835	1	p3	(CAC)5	15	4612	4626	potentially variable
PHG9_Scaffold_4835	2	p2	(AG)6	12	9990	10001	
PHG9_Scaffold_4835	4	p3	(GAA)5	15	13951	13965	potentially variable
PHG9_Scaffold_4835	9	p2	(AT)7	14	24753	24766	potentially variable
PHG9_Scaffold_4835	42	p2	(AT)8	16	143779	143794	potentially variable
PHG9_Scaffold_4836	2	p2	(AT)8	16	14764	14779	potentially variable
PHG9_Scaffold_4836	6	p3	(AAT)5	15	47373	47387	potentially variable
PHG9_Scaffold_4837	1	p2	(AT)6	12	3436	3447	
PHG9_Scaffold_4837	5	p2	(AT)6	12	25110	25121	
PHG9_Scaffold_4838	5	p2	(AT)9	18	14223	14240	potentially variable
PHG9_Scaffold_4838	6	p2	(TA)6	12	15782	15793	
PHG9_Scaffold_4838	13	p3	(AGA)5	15	29691	29705	potentially variable
PHG9_Scaffold_4838	14	p3	(ATT)6	18	30562	30579	potentially variable
PHG9_Scaffold_4838	18	p2	(TA)9	18	38491	38508	potentially variable
PHG9_Scaffold_4838	20	p2	(TA)8	16	40088	40103	potentially

							variable
PHG9_Scaffold_4838	31	p2	(TA)6	12	70503	70514	
PHG9_Scaffold_4839	4	p2	(TA)6	12	11759	11770	
PHG9_Scaffold_4839	6	p3	(TTA)5	15	12713	12727	potentially variable
PHG9_Scaffold_4839	7	p3	(CAC)5	15	13869	13883	potentially variable
PHG9_Scaffold_4839	17	p2	(AT)6	12	39200	39211	
PHG9_Scaffold_4839	23	p2	(CA)7	14	53470	53483	potentially variable
PHG9_Scaffold_4839	24	p2	(AT)7	14	54426	54439	potentially variable
PHG9_Scaffold_4840	1	p6	(TGAAGA)5	30	616	645	Hypervariable
PHG9_Scaffold_4840	16	p3	(ACC)5	15	30235	30249	potentially variable
PHG9_Scaffold_4840	28	p3	(TGG)6	18	71353	71370	potentially variable
PHG9_Scaffold_4840	30	p2	(TA)6	12	77854	77865	
PHG9_Scaffold_4840	36	p2	(AT)9	18	94751	94768	potentially variable
PHG9_Scaffold_4840	46	p2	(TA)6	12	115972	115983	
PHG9_Scaffold_4840	49	p3	(AAT)5	15	119289	119303	potentially variable
PHG9_Scaffold_4840	52	p2	(TA)6	12	132174	132185	
PHG9_Scaffold_4841	9	p3	(ATA)5	15	21141	21155	potentially variable
PHG9_Scaffold_4841	11	p2	(TA)7	14	22786	22799	potentially variable
PHG9_Scaffold_4841	13	p2	(TA)8	16	33190	33205	potentially

							variable
PHG9_Scaffold_4841	18	p2	(TA)6	12	34902	34913	
PHG9_Scaffold_4841	23	p2	(TC)6	12	59560	59571	
PHG9_Scaffold_4842	4	p2	(AT)6	12	28265	28276	
PHG9_Scaffold_4842	6	p2	(AT)9	18	34230	34247	potentially variable
PHG9_Scaffold_4842	17	p2	(AT)8	16	66977	66992	potentially variable
PHG9_Scaffold_4843	4	p2	(AT)7	14	40606	40619	potentially variable
PHG9_Scaffold_4843	9	p2	(AT)7	14	75999	76012	potentially variable
PHG9_Scaffold_4844	2	p2	(TA)8	16	1732	1747	potentially variable
PHG9_Scaffold_4844	8	p2	(AT)6	12	11083	11094	
PHG9_Scaffold_4844	10	p2	(TG)6	12	17619	17630	
PHG9_Scaffold_4844	14	p3	(TGT)5	15	34288	34302	potentially variable
PHG9_Scaffold_4844	22	p3	(TAA)5	15	56528	56542	potentially variable
PHG9_Scaffold_4846	2	p2	(TA)6	12	7393	7404	
PHG9_Scaffold_4846	3	p2	(GA)7	14	7637	7650	potentially variable
PHG9_Scaffold_4846	7	p2	(AG)6	12	13572	13583	
PHG9_Scaffold_4846	8	p3	(ATT)5	15	14647	14661	potentially variable
PHG9_Scaffold_4846	13	p2	(CA)9	18	21079	21096	potentially variable
PHG9_Scaffold_4846	15	p2	(TA)7	14	24309	24322	potentially

							variable
PHG9_Scaffold_4846	16	p2	(TA)6	12	31804	31815	
PHG9_Scaffold_4846	17	p3	(TTA)5	15	39084	39098	potentially variable
PHG9_Scaffold_4846	20	p2	(AT)8	16	64490	64505	potentially variable
PHG9_Scaffold_4846	24	p2	(GA)7	14	81915	81928	potentially variable
PHG9_Scaffold_4846	26	p2	(TA)8	16	95607	95622	potentially variable
PHG9_Scaffold_4849	1	p2	(CT)6	12	3385	3396	
PHG9_Scaffold_4849	14	p2	(CT)6	12	45296	45307	
PHG9_Scaffold_4849	15	p3	(ATA)5	15	46706	46720	potentially variable
PHG9_Scaffold_4849	16	p2	(TA)9	18	47151	47168	potentially variable
PHG9_Scaffold_4849	23	p2	(CA)9	18	66129	66146	potentially variable
PHG9_Scaffold_4849	24	p3	(TAA)5	15	72766	72780	potentially variable
PHG9_Scaffold_4849	29	p2	(CA)6	12	83321	83332	
PHG9_Scaffold_4849	42	p2	(GA)6	12	134052	134063	
PHG9_Scaffold_4851	7	p2	(TA)6	12	25721	25732	
PHG9_Scaffold_4851	17	p2	(AG)7	14	61349	61362	potentially variable
PHG9_Scaffold_4852	6	p3	(TAT)5	15	12029	12043	potentially variable
PHG9_Scaffold_4852	27	p2	(AT)8	16	78110	78125	potentially variable

PHG9_Scaffold_4853	12	p2	(AT)6	12	48069	48080	
PHG9_Scaffold_4853	22	p2	(CT)6	12	82718	82729	
PHG9_Scaffold_4854	1	p2	(AT)6	12	1264	1275	
PHG9_Scaffold_4855	1	p3	(TAA)5	15	568	582	potentially variable
PHG9_Scaffold_4855	2	p2	(TA)7	14	4031	4044	potentially variable
PHG9_Scaffold_4855	24	p2	(AT)6	12	48333	48344	
PHG9_Scaffold_4855	27	p2	(TA)6	12	64778	64789	
PHG9_Scaffold_4855	30	p2	(AT)7	14	71597	71610	potentially variable
PHG9_Scaffold_4857	6	p2	(TA)6	12	15026	15037	
PHG9_Scaffold_4857	7	p2	(AT)7	14	17517	17530	potentially variable
PHG9_Scaffold_4857	10	p3	(GTA)6	18	25935	25952	potentially variable
PHG9_Scaffold_4857	11	p2	(CT)9	18	27827	27844	potentially variable
PHG9_Scaffold_4858	12	p2	(AT)7	14	85785	85798	potentially variable
PHG9_Scaffold_4861	2	p2	(TC)7	14	11167	11180	potentially variable
PHG9_Scaffold_4861	6	p2	(TC)6	12	32142	32153	
PHG9_Scaffold_4861	11	p2	(TA)6	12	49166	49177	
PHG9_Scaffold_4861	19	p3	(GAA)5	15	88482	88496	potentially variable
PHG9_Scaffold_4862	4	p2	(TA)6	12	12559	12570	
PHG9_Scaffold_4862	14	p3	(TAC)6	18	41098	41115	potentially variable

PHG9_Scaffold_4862	17	p2	(GA)6	12	46225	46236	
PHG9_Scaffold_4862	18	p3	(AAT)6	18	48059	48076	potentially variable
PHG9_Scaffold_4862	26	p3	(TAA)5	15	66872	66886	potentially variable
PHG9_Scaffold_4863	3	p2	(TA)6	12	3483	3494	
PHG9_Scaffold_4863	7	p3	(TGA)6	18	23326	23343	potentially variable
PHG9_Scaffold_4864	6	p3	(TAT)5	15	16993	17007	potentially variable
PHG9_Scaffold_4865	2	p3	(TGC)5	15	2786	2800	potentially variable
PHG9_Scaffold_4865	3	p2	(CT)8	16	3284	3299	potentially variable
PHG9_Scaffold_4865	14	p2	(TA)6	12	17097	17108	
PHG9_Scaffold_4865	20	p2	(CT)6	12	46945	46956	
PHG9_Scaffold_4866	13	p3	(TTG)5	15	31118	31132	potentially variable
PHG9_Scaffold_4866	16	p3	(TAT)6	18	44963	44980	potentially variable
PHG9_Scaffold_4866	20	p2	(TA)6	12	56131	56142	
PHG9_Scaffold_4866	28	p3	(CTG)5	15	64936	64950	potentially variable
PHG9_Scaffold_4866	29	p2	(TC)8	16	65674	65689	potentially variable
PHG9_Scaffold_4866	31	p2	(AG)6	12	66472	66483	
PHG9_Scaffold_4866	36	p2	(AT)6	12	78853	78864	
PHG9_Scaffold_4866	40	p3	(CTT)5	15	84116	84130	potentially variable

PHG9_Scaffold_4866	43	p2	(AT)7	14	86287	86300	potentially variable
PHG9_Scaffold_4866	44	p2	(AT)7	14	89207	89220	potentially variable
PHG9_Scaffold_4866	49	p2	(GA)8	16	97211	97226	potentially variable
PHG9_Scaffold_4868	2	p2	(AT)6	12	3552	3563	
PHG9_Scaffold_4870	2	p2	(AT)8	16	6950	6965	potentially variable
PHG9_Scaffold_4870	5	p2	(TA)8	16	12321	12336	potentially variable
PHG9_Scaffold_4870	9	p2	(AG)6	12	27560	27571	
PHG9_Scaffold_4870	12	p2	(TA)6	12	32139	32150	
PHG9_Scaffold_4870	14	p3	(TGA)5	15	36617	36631	potentially variable
PHG9_Scaffold_4870	17	p3	(AGA)5	15	51835	51849	potentially variable
PHG9_Scaffold_4870	28	p2	(TA)7	14	70417	70430	potentially variable
PHG9_Scaffold_4871	1	p2	(TC)7	14	2164	2177	potentially variable
PHG9_Scaffold_4871	4	p3	(TAA)5	15	11500	11514	potentially variable
PHG9_Scaffold_4871	7	p3	(AAG)5	15	14621	14635	potentially variable
PHG9_Scaffold_4871	13	p2	(CT)6	12	34661	34672	
PHG9_Scaffold_4871	19	p2	(AT)7	14	51802	51815	potentially variable
PHG9_Scaffold_4871	28	p2	(AT)9	18	83964	83981	potentially variable

PHG9_Scaffold_4871	29	p2	(TA)9	18	84125	84142	potentially variable
PHG9_Scaffold_4871	30	p2	(AT)7	14	89980	89993	potentially variable
PHG9_Scaffold_4871	39	p3	(AGA)5	15	105376	105390	potentially variable
PHG9_Scaffold_4871	41	p2	(TC)6	12	106749	106760	
PHG9_Scaffold_4871	42	p2	(TC)6	12	109058	109069	
PHG9_Scaffold_4871	43	p2	(TA)9	18	109623	109640	potentially variable
PHG9_Scaffold_4871	47	p2	(GT)6	12	119471	119482	
PHG9_Scaffold_4871	48	p2	(TC)6	12	119607	119618	
PHG9_Scaffold_4871	51	p2	(AT)7	14	131465	131478	potentially variable
PHG9_Scaffold_4871	56	p2	(TA)7	14	151392	151405	potentially variable
PHG9_Scaffold_4871	61	p3	(TTA)6	18	171248	171265	potentially variable
PHG9_Scaffold_4871	66	p3	(TTA)5	15	175426	175440	potentially variable
PHG9_Scaffold_4871	76	p2	(TA)6	12	201173	201184	
PHG9_Scaffold_4873	8	p2	(TA)6	12	33225	33236	
PHG9_Scaffold_4873	12	p2	(AT)8	16	64824	64839	potentially variable
PHG9_Scaffold_4876	4	p2	(AT)7	14	9308	9321	potentially variable
PHG9_Scaffold_4876	13	p2	(AT)6	12	42244	42255	
PHG9_Scaffold_4876	14	p2	(AT)8	16	42921	42936	potentially variable

PHG9_Scaffold_4876	20	p2	(TC)6	12	52521	52532	
PHG9_Scaffold_4876	24	p2	(GT)6	12	62632	62643	
PHG9_Scaffold_4876	25	p3	(TTA)6	18	66207	66224	potentially variable
PHG9_Scaffold_4876	30	p2	(TA)7	14	72999	73012	potentially variable
PHG9_Scaffold_4877	6	p3	(AAT)5	15	23567	23581	potentially variable
PHG9_Scaffold_4877	13	p2	(GA)9	18	52395	52412	potentially variable
PHG9_Scaffold_4877	21	p2	(TA)8	16	74894	74909	potentially variable
PHG9_Scaffold_4877	34	p2	(TA)8	16	124961	124976	potentially variable
PHG9_Scaffold_4877	35	p2	(TA)8	16	129375	129390	potentially variable
PHG9_Scaffold_4877	43	p3	(AAT)5	15	147844	147858	potentially variable
PHG9_Scaffold_4878	2	p2	(AT)8	16	516	531	potentially variable
PHG9_Scaffold_4878	3	p2	(TA)6	12	3787	3798	
PHG9_Scaffold_4878	5	p2	(TA)9	18	8577	8594	potentially variable
PHG9_Scaffold_4878	8	p2	(TG)8	16	19951	19966	potentially variable
PHG9_Scaffold_4878	9	p2	(TA)7	14	21353	21366	potentially variable
PHG9_Scaffold_4878	12	p2	(AT)6	12	26420	26431	
PHG9_Scaffold_4878	20	p2	(TA)8	16	37316	37331	potentially variable

PHG9_Scaffold_4878	27	p3	(AAT)6	18	50007	50024	potentially variable
PHG9_Scaffold_4878	38	p3	(ATT)5	15	70851	70865	potentially variable
PHG9_Scaffold_4878	46	p2	(AT)6	12	90119	90130	
PHG9_Scaffold_4878	48	p3	(ATG)5	15	91820	91834	potentially variable
PHG9_Scaffold_4879	5	p2	(AT)6	12	15140	15151	
PHG9_Scaffold_4879	7	p2	(AC)6	12	19025	19036	
PHG9_Scaffold_4879	13	p3	(ATA)5	15	34588	34602	potentially variable
PHG9_Scaffold_4879	16	p3	(ATA)6	18	43777	43794	potentially variable
PHG9_Scaffold_4879	25	p2	(TA)8	16	72916	72931	potentially variable
PHG9_Scaffold_4879	31	p2	(TA)6	12	89098	89109	
PHG9_Scaffold_4879	34	p3	(GCT)5	15	93256	93270	potentially variable
PHG9_Scaffold_4880	17	p2	(CT)6	12	47358	47369	
PHG9_Scaffold_4880	22	p2	(TA)9	18	52748	52765	potentially variable
PHG9_Scaffold_4880	28	p2	(AT)6	12	62325	62336	
PHG9_Scaffold_4880	29	p3	(ATA)5	15	64288	64302	potentially variable
PHG9_Scaffold_4882	4	p2	(GA)6	12	3128	3139	
PHG9_Scaffold_4882	8	p3	(AGA)6	18	41858	41875	potentially variable
PHG9_Scaffold_4882	16	p2	(TC)6	12	68813	68824	
PHG9_Scaffold_4882	20	p2	(TA)7	14	87790	87803	potentially

							variable
PHG9_Scaffold_4882	22	p3	(ATT)5	15	97324	97338	potentially variable
PHG9_Scaffold_4882	24	p2	(TA)6	12	106120	106131	
PHG9_Scaffold_4883	3	p2	(AC)6	12	18462	18473	
PHG9_Scaffold_4883	13	p2	(TA)8	16	33489	33504	potentially variable
PHG9_Scaffold_4883	18	p2	(CT)7	14	48563	48576	potentially variable
PHG9_Scaffold_4883	22	p2	(TC)7	14	68503	68516	potentially variable
PHG9_Scaffold_4884	3	p2	(TA)6	12	19190	19201	
PHG9_Scaffold_4885	12	p2	(TA)7	14	74379	74392	potentially variable
PHG9_Scaffold_4885	17	p2	(AT)9	18	87315	87332	potentially variable
PHG9_Scaffold_4887	3	p2	(AT)6	12	17628	17639	
PHG9_Scaffold_4888	3	p2	(AG)7	14	5914	5927	potentially variable
PHG9_Scaffold_4888	6	p3	(TAT)5	15	9400	9414	potentially variable
PHG9_Scaffold_4888	12	p2	(TA)9	18	40205	40222	potentially variable
PHG9_Scaffold_4893	8	p2	(TA)6	12	28579	28590	
PHG9_Scaffold_4893	12	p2	(TA)8	16	38677	38692	potentially variable
PHG9_Scaffold_4893	13	p3	(GAA)5	15	46209	46223	potentially variable
PHG9_Scaffold_4893	16	p2	(AT)9	18	53953	53970	potentially variable

PHG9_Scaffold_4895	2	p2	(AT)6	12	25758	25769	
PHG9_Scaffold_4896	3	p3	(ATT)5	15	4697	4711	potentially variable
PHG9_Scaffold_4896	18	p3	(ATA)6	18	47132	47149	potentially variable
PHG9_Scaffold_4896	20	p3	(TAT)5	15	50553	50567	potentially variable
PHG9_Scaffold_4896	23	p2	(AC)7	14	59736	59749	potentially variable
PHG9_Scaffold_4896	39	p2	(TC)9	18	101500	101517	potentially variable
PHG9_Scaffold_4896	41	p3	(TCT)5	15	109607	109621	potentially variable
PHG9_Scaffold_4896	44	p3	(AAT)5	15	115156	115170	potentially variable
PHG9_Scaffold_4896	45	p2	(TA)8	16	121674	121689	potentially variable
PHG9_Scaffold_4896	51	p2	(TA)8	16	134975	134990	potentially variable
PHG9_Scaffold_4896	52	p2	(TA)6	12	135774	135785	
PHG9_Scaffold_4896	56	p2	(AT)6	12	142654	142665	
PHG9_Scaffold_4896	57	p2	(TA)6	12	143670	143681	
PHG9_Scaffold_4896	61	p2	(TA)8	16	153063	153078	potentially variable
PHG9_Scaffold_4896	63	p2	(TA)7	14	157611	157624	potentially variable
PHG9_Scaffold_4897	2	p2	(TA)7	14	30846	30859	potentially variable
PHG9_Scaffold_4897	3	p2	(TA)7	14	31590	31603	potentially variable

PHG9_Scaffold_4897	7	p2	(AT)8	16	43671	43686	potentially variable
PHG9_Scaffold_4898	3	p3	(GAA)5	15	4304	4318	potentially variable
PHG9_Scaffold_4898	12	p2	(TA)9	18	34374	34391	potentially variable
PHG9_Scaffold_4898	13	p2	(AT)7	14	36884	36897	potentially variable
PHG9_Scaffold_4898	15	p6	(GATGAA)5	30	40020	40049	Hypervariable
PHG9_Scaffold_4898	17	p3	(AAG)5	15	46600	46614	potentially variable
PHG9_Scaffold_4898	19	p3	(TCT)5	15	59232	59246	potentially variable
PHG9_Scaffold_4899	3	p2	(AG)7	14	9966	9979	potentially variable
PHG9_Scaffold_4899	10	p3	(GAT)6	18	41422	41439	potentially variable
PHG9_Scaffold_4899	15	p3	(TAT)6	18	59938	59955	potentially variable
PHG9_Scaffold_4900	1	p3	(TTA)5	15	758	772	potentially variable
PHG9_Scaffold_4900	2	p3	(TCT)5	15	3591	3605	potentially variable
PHG9_Scaffold_4902	3	p2	(TA)8	16	68198	68213	potentially variable
PHG9_Scaffold_4903	18	p2	(AG)7	14	63098	63111	potentially variable
PHG9_Scaffold_4903	20	p2	(TA)9	18	65399	65416	potentially variable
PHG9_Scaffold_4903	21	p3	(CTT)5	15	66656	66670	potentially

							variable
PHG9_Scaffold_4904	2	p3	(TAA)5	15	76276	76290	potentially variable
PHG9_Scaffold_4908	3	p2	(TA)6	12	7557	7568	
PHG9_Scaffold_4908	11	p3	(TTC)6	18	41047	41064	potentially variable
PHG9_Scaffold_4911	1	p2	(AT)8	16	24	39	potentially variable
PHG9_Scaffold_4913	19	p3	(CCA)5	15	69958	69972	potentially variable
PHG9_Scaffold_4913	27	p3	(CTT)5	15	102965	102979	potentially variable
PHG9_Scaffold_4913	32	p2	(TA)6	12	144960	144971	
PHG9_Scaffold_4913	35	p2	(AT)9	18	155903	155920	potentially variable
PHG9_Scaffold_4913	36	p2	(TA)7	14	158749	158762	potentially variable
PHG9_Scaffold_4913	46	p2	(CA)9	18	199911	199928	potentially variable
PHG9_Scaffold_4913	53	p2	(GT)7	14	238921	238934	potentially variable
PHG9_Scaffold_4913	55	p2	(AT)6	12	247674	247685	
PHG9_Scaffold_4913	57	p2	(TA)6	12	249328	249339	
PHG9_Scaffold_4913	59	p2	(TA)7	14	257583	257596	potentially variable
PHG9_Scaffold_4914	3	p2	(CT)8	16	8592	8607	potentially variable
PHG9_Scaffold_4914	12	p2	(TA)6	12	29773	29784	
PHG9_Scaffold_4914	21	p2	(AT)6	12	55557	55568	

PHG9_Scaffold_4914	24	p2	(AT)8	16	73525	73540	potentially variable
PHG9_Scaffold_4915	5	p3	(ATC)5	15	19659	19673	potentially variable
PHG9_Scaffold_4915	8	p3	(TGT)5	15	28355	28369	potentially variable
PHG9_Scaffold_4915	10	p2	(TA)6	12	31406	31417	
PHG9_Scaffold_4915	18	p2	(TA)9	18	62012	62029	potentially variable
PHG9_Scaffold_4915	21	p3	(GTT)5	15	71680	71694	potentially variable
PHG9_Scaffold_4915	22	p3	(AAG)5	15	72135	72149	potentially variable
PHG9_Scaffold_4918	1	p2	(CT)6	12	130	141	
PHG9_Scaffold_4918	12	p2	(AT)9	18	25444	25461	potentially variable
PHG9_Scaffold_4918	23	p2	(AG)7	14	56275	56288	potentially variable
PHG9_Scaffold_4919	2	p3	(AGA)6	18	1651	1668	potentially variable
PHG9_Scaffold_4919	3	p2	(TC)9	18	2567	2584	potentially variable
PHG9_Scaffold_4919	6	p2	(TA)7	14	16779	16792	potentially variable
PHG9_Scaffold_4921	4	p3	(CTG)5	15	2767	2781	potentially variable
PHG9_Scaffold_4921	8	p2	(GA)8	16	11492	11507	potentially variable
PHG9_Scaffold_4921	11	p2	(TA)8	16	15986	16001	potentially variable

PHG9_Scaffold_4921	12	p2	(AT)9	18	19164	19181	potentially variable
PHG9_Scaffold_4921	24	p2	(AG)8	16	57493	57508	potentially variable
PHG9_Scaffold_4921	26	p3	(AGA)5	15	65082	65096	potentially variable
PHG9_Scaffold_4923	4	p3	(CCT)5	15	11287	11301	potentially variable
PHG9_Scaffold_4923	7	p2	(TA)6	12	15299	15310	
PHG9_Scaffold_4923	8	p2	(CT)8	16	16871	16886	potentially variable
PHG9_Scaffold_4923	10	p2	(CT)6	12	26340	26351	
PHG9_Scaffold_4923	14	p2	(TG)6	12	34509	34520	
PHG9_Scaffold_4924	2	p2	(TA)8	16	23469	23484	potentially variable
PHG9_Scaffold_4924	5	p3	(TCG)6	18	44261	44278	potentially variable
PHG9_Scaffold_4925	8	p2	(AT)6	12	50183	50194	
PHG9_Scaffold_4926	3	p2	(TA)6	12	16463	16474	
PHG9_Scaffold_4926	6	p2	(TA)6	12	21168	21179	
PHG9_Scaffold_4926	7	p2	(TA)6	12	24614	24625	
PHG9_Scaffold_4927	6	p2	(TA)6	12	34869	34880	
PHG9_Scaffold_4930	1	p2	(TA)6	12	1456	1467	
PHG9_Scaffold_4930	4	p3	(TCT)5	15	13189	13203	potentially variable
PHG9_Scaffold_4930	11	p2	(AT)6	12	33284	33295	
PHG9_Scaffold_4930	15	p2	(AT)6	12	35994	36005	
PHG9_Scaffold_4930	17	p3	(TAA)5	15	40819	40833	potentially variable

PHG9_Scaffold_4930	18	p3	(TAT)5	15	45067	45081	potentially variable
PHG9_Scaffold_4930	20	p2	(TA)7	14	57053	57066	potentially variable
PHG9_Scaffold_4933	2	p2	(TC)7	14	2346	2359	potentially variable
PHG9_Scaffold_4933	8	p2	(TA)8	16	32376	32391	potentially variable
PHG9_Scaffold_4933	11	p3	(AAT)6	18	37865	37882	potentially variable
PHG9_Scaffold_4933	13	p2	(TA)9	18	46234	46251	potentially variable
PHG9_Scaffold_4933	15	p3	(TTA)5	15	51924	51938	potentially variable
PHG9_Scaffold_4933	21	p2	(AT)6	12	76540	76551	
PHG9_Scaffold_4934	4	p2	(AG)6	12	10404	10415	
PHG9_Scaffold_4934	5	p2	(AC)7	14	11208	11221	potentially variable
PHG9_Scaffold_4934	7	p2	(TA)6	12	11980	11991	
PHG9_Scaffold_4936	2	p2	(TA)8	16	1498	1513	potentially variable
PHG9_Scaffold_4936	4	p3	(TAA)5	15	7080	7094	potentially variable
PHG9_Scaffold_4936	7	p2	(AT)8	16	33292	33307	potentially variable
PHG9_Scaffold_4936	9	p2	(TA)7	14	35000	35013	potentially variable
PHG9_Scaffold_4936	16	p2	(AT)8	16	65013	65028	potentially variable
PHG9_Scaffold_4936	17	p2	(TA)6	12	72457	72468	

PHG9_Scaffold_4937	1	p2	(TA)9	18	16013	16030	potentially variable
PHG9_Scaffold_4938	1	p2	(TA)6	12	4420	4431	
PHG9_Scaffold_4938	2	p2	(AT)8	16	6756	6771	potentially variable
PHG9_Scaffold_4938	6	p2	(AT)6	12	16482	16493	
PHG9_Scaffold_4938	7	p2	(GA)6	12	17079	17090	
PHG9_Scaffold_4938	16	p3	(TCA)5	15	29901	29915	potentially variable
PHG9_Scaffold_4938	17	p2	(TC)7	14	30318	30331	potentially variable
PHG9_Scaffold_4938	19	p2	(TA)6	12	39993	40004	
PHG9_Scaffold_4938	20	p3	(ATA)5	15	41788	41802	potentially variable
PHG9_Scaffold_4938	25	p2	(AT)6	12	53533	53544	
PHG9_Scaffold_4938	28	p2	(AT)6	12	61038	61049	
PHG9_Scaffold_4938	30	p2	(GT)6	12	63259	63270	
PHG9_Scaffold_4938	34	p2	(AG)6	12	68846	68857	
PHG9_Scaffold_4941	5	p2	(TA)6	12	64839	64850	
PHG9_Scaffold_4941	13	p2	(AT)8	16	111904	111919	potentially variable
PHG9_Scaffold_4943	1	p2	(TA)6	12	1894	1905	
PHG9_Scaffold_4943	3	p3	(TAA)5	15	2711	2725	potentially variable
PHG9_Scaffold_4943	10	p2	(TA)9	18	10751	10768	potentially variable
PHG9_Scaffold_4943	13	p2	(AT)6	12	30912	30923	
PHG9_Scaffold_4944	1	p2	(TC)9	18	2105	2122	potentially variable

PHG9_Scaffold_4944	8	p2	(AT)6	12	28014	28025	
PHG9_Scaffold_4944	11	p2	(AT)7	14	57468	57481	potentially variable
PHG9_Scaffold_4945	2	p2	(TA)6	12	3649	3660	
PHG9_Scaffold_4946	2	p2	(AT)6	12	6717	6728	
PHG9_Scaffold_4946	5	p2	(TA)6	12	14871	14882	
PHG9_Scaffold_4946	9	p2	(TA)6	12	31031	31042	
PHG9_Scaffold_4947	3	p2	(TA)6	12	10707	10718	
PHG9_Scaffold_4947	14	p2	(AT)6	12	47180	47191	
PHG9_Scaffold_4947	19	p2	(AT)9	18	79541	79558	potentially variable
PHG9_Scaffold_4947	22	p2	(TA)6	12	80596	80607	
PHG9_Scaffold_4947	24	p2	(AT)9	18	84363	84380	potentially variable
PHG9_Scaffold_4948	2	p2	(TA)9	18	7666	7683	potentially variable
PHG9_Scaffold_4948	16	p2	(TA)6	12	28586	28597	
PHG9_Scaffold_4948	22	p2	(TA)7	14	42160	42173	potentially variable
PHG9_Scaffold_4948	38	p2	(AT)6	12	78305	78316	
PHG9_Scaffold_4948	46	p2	(TC)7	14	96288	96301	potentially variable
PHG9_Scaffold_4948	47	p2	(CT)9	18	97075	97092	potentially variable
PHG9_Scaffold_4949	3	p2	(CT)7	14	18920	18933	potentially variable
PHG9_Scaffold_4949	4	p3	(TAT)5	15	19242	19256	potentially variable
PHG9_Scaffold_4949	14	p3	(GCC)5	15	24604	24618	potentially

							variable
PHG9_Scaffold_4949	18	p3	(GGA)5	15	50897	50911	potentially variable
PHG9_Scaffold_4949	22	p3	(CAA)5	15	71093	71107	potentially variable
PHG9_Scaffold_4950	9	p2	(GT)7	14	51133	51146	potentially variable
PHG9_Scaffold_4951	1	p2	(AT)7	14	649	662	potentially variable
PHG9_Scaffold_4951	10	p3	(ATA)5	15	13226	13240	potentially variable
PHG9_Scaffold_4953	4	p3	(ACA)5	15	7740	7754	potentially variable
PHG9_Scaffold_4953	8	p2	(GT)9	18	34424	34441	potentially variable
PHG9_Scaffold_4953	9	p2	(TA)7	14	35828	35841	potentially variable
PHG9_Scaffold_4953	22	p3	(TTC)5	15	89598	89612	potentially variable
PHG9_Scaffold_4953	23	p2	(TA)9	18	92428	92445	potentially variable
PHG9_Scaffold_4953	28	p3	(AAT)5	15	105275	105289	potentially variable
PHG9_Scaffold_4953	29	p3	(AAT)5	15	105950	105964	potentially variable
PHG9_Scaffold_4954	2	p2	(TA)8	16	2246	2261	potentially variable
PHG9_Scaffold_4954	3	p2	(AT)7	14	22532	22545	potentially variable
PHG9_Scaffold_4958	1	p3	(CTT)5	15	8358	8372	potentially

							variable
PHG9_Scaffold_4960	1	p2	(TA)7	14	8972	8985	potentially variable
PHG9_Scaffold_4960	17	p2	(AT)7	14	65401	65414	potentially variable
PHG9_Scaffold_4961	9	p2	(AT)9	18	29154	29171	potentially variable
PHG9_Scaffold_4961	14	p2	(GT)6	12	39809	39820	
PHG9_Scaffold_4961	18	p2	(GT)6	12	70220	70231	
PHG9_Scaffold_4961	19	p2	(TA)6	12	70395	70406	
PHG9_Scaffold_4962	11	p2	(TA)6	12	51727	51738	
PHG9_Scaffold_4963	6	p3	(ATT)5	15	42047	42061	potentially variable
PHG9_Scaffold_4964	5	p2	(AT)7	14	31910	31923	potentially variable
PHG9_Scaffold_4965	16	p2	(AT)6	12	46725	46736	
PHG9_Scaffold_4966	3	p3	(GGT)5	15	10360	10374	potentially variable
PHG9_Scaffold_4966	7	p2	(TA)6	12	47045	47056	
PHG9_Scaffold_4967	6	p3	(TCG)5	15	17802	17816	potentially variable
PHG9_Scaffold_4967	10	p3	(TAT)6	18	30908	30925	potentially variable
PHG9_Scaffold_4967	12	p3	(TAA)5	15	39536	39550	potentially variable
PHG9_Scaffold_4967	14	p2	(AT)6	12	50658	50669	
PHG9_Scaffold_4967	16	p2	(TA)6	12	52426	52437	
PHG9_Scaffold_4967	21	p2	(GA)7	14	74182	74195	potentially variable

PHG9_Scaffold_4968	3	p3	(TTA)5	15	11630	11644	potentially variable
PHG9_Scaffold_4968	8	p3	(GAA)6	18	27591	27608	potentially variable
PHG9_Scaffold_4968	11	p2	(AT)9	18	37612	37629	potentially variable
PHG9_Scaffold_4968	16	p2	(TA)9	18	47227	47244	potentially variable
PHG9_Scaffold_4969	1	p3	(ACC)5	15	182	196	potentially variable
PHG9_Scaffold_4969	11	p2	(TC)10	20	30486	30505	Hypervariable
PHG9_Scaffold_4969	15	p3	(GCA)5	15	51830	51844	potentially variable
PHG9_Scaffold_4969	17	p2	(TA)7	14	55969	55982	potentially variable
PHG9_Scaffold_4972	1	p2	(GA)8	16	6629	6644	potentially variable
PHG9_Scaffold_4972	3	p2	(TA)9	18	9123	9140	potentially variable
PHG9_Scaffold_4972	5	p2	(TA)6	12	14494	14505	
PHG9_Scaffold_4972	6	p3	(GCG)6	18	17218	17235	potentially variable
PHG9_Scaffold_4972	11	p2	(TA)7	14	37774	37787	potentially variable
PHG9_Scaffold_4972	18	p3	(ATT)5	15	50644	50658	potentially variable
PHG9_Scaffold_4973	4	p2	(AC)6	12	14154	14165	
PHG9_Scaffold_4973	8	p2	(AT)9	18	34835	34852	potentially variable
PHG9_Scaffold_4973	12	p3	(TAA)5	15	48196	48210	potentially

							variable
PHG9_Scaffold_4973	14	p3	(AGA)6	18	55924	55941	potentially variable
PHG9_Scaffold_4973	15	p2	(AT)6	12	58921	58932	
PHG9_Scaffold_4973	18	p3	(AAC)6	18	70427	70444	potentially variable
PHG9_Scaffold_4973	19	p3	(CAC)5	15	71857	71871	potentially variable
PHG9_Scaffold_4973	20	p2	(GT)7	14	72437	72450	potentially variable
PHG9_Scaffold_4973	21	p3	(ACA)5	15	75146	75160	potentially variable
PHG9_Scaffold_4974	1	p3	(CAC)5	15	565	579	potentially variable
PHG9_Scaffold_4974	9	p2	(TG)6	12	21091	21102	
PHG9_Scaffold_4974	16	p2	(TC)6	12	55678	55689	
PHG9_Scaffold_4974	21	p3	(GAT)5	15	66551	66565	potentially variable
PHG9_Scaffold_4974	29	p2	(TA)7	14	77034	77047	potentially variable
PHG9_Scaffold_4976	1	p3	(TCT)5	15	854	868	potentially variable
PHG9_Scaffold_4976	7	p3	(GGT)6	18	14163	14180	potentially variable
PHG9_Scaffold_4976	14	p3	(TCT)5	15	28709	28723	potentially variable
PHG9_Scaffold_4976	16	p3	(AAG)5	15	32992	33006	potentially variable
PHG9_Scaffold_4976	17	p2	(AT)8	16	34653	34668	potentially variable

PHG9_Scaffold_4976	28	p2	(GT)6	12	58652	58663	
PHG9_Scaffold_4977	5	p2	(AT)6	12	8325	8336	
PHG9_Scaffold_4977	10	p2	(AT)7	14	22308	22321	potentially variable
PHG9_Scaffold_4977	13	p2	(TC)7	14	24787	24800	potentially variable
PHG9_Scaffold_4977	15	p3	(TAT)5	15	29015	29029	potentially variable
PHG9_Scaffold_4977	19	p2	(AT)6	12	45887	45898	
PHG9_Scaffold_4977	24	p3	(GTT)5	15	58434	58448	potentially variable
PHG9_Scaffold_4977	26	p2	(AG)9	18	59717	59734	potentially variable
PHG9_Scaffold_4979	2	p3	(TAT)5	15	4180	4194	potentially variable
PHG9_Scaffold_4979	9	p2	(TA)7	14	25996	26009	potentially variable
PHG9_Scaffold_4981	2	p3	(TAA)6	18	1509	1526	potentially variable
PHG9_Scaffold_4981	6	p2	(AT)6	12	27355	27366	
PHG9_Scaffold_4981	15	p2	(TA)6	12	48511	48522	
PHG9_Scaffold_4982	1	p3	(TGC)5	15	3266	3280	potentially variable
PHG9_Scaffold_4982	15	p2	(TA)6	12	55693	55704	
PHG9_Scaffold_4982	36	p3	(AAT)5	15	133879	133893	potentially variable
PHG9_Scaffold_4982	38	p2	(AT)7	14	135696	135709	potentially variable
PHG9_Scaffold_4983	6	p2	(TA)6	12	5786	5797	

PHG9_Scaffold_4983	7	p2	(AT)7	14	22540	22553	potentially variable
PHG9_Scaffold_4983	8	p2	(TA)8	16	26484	26499	potentially variable
PHG9_Scaffold_4987	5	p2	(TA)6	12	16802	16813	
PHG9_Scaffold_4987	6	p2	(TA)8	16	19192	19207	potentially variable
PHG9_Scaffold_4987	8	p2	(AT)6	12	24686	24697	
PHG9_Scaffold_4987	11	p2	(AT)6	12	30556	30567	
PHG9_Scaffold_4987	15	p3	(TTA)5	15	41497	41511	potentially variable
PHG9_Scaffold_4988	1	p2	(TA)6	12	1437	1448	
PHG9_Scaffold_4988	2	p3	(TAT)6	18	1825	1842	potentially variable
PHG9_Scaffold_4988	4	p2	(GT)8	16	14212	14227	potentially variable
PHG9_Scaffold_4988	6	p2	(CT)6	12	27260	27271	
PHG9_Scaffold_4988	7	p3	(TAT)5	15	29436	29450	potentially variable
PHG9_Scaffold_4988	11	p2	(TC)9	18	47210	47227	potentially variable
PHG9_Scaffold_4988	13	p3	(AAC)5	15	58269	58283	potentially variable
PHG9_Scaffold_4990	2	p2	(AT)9	18	7033	7050	potentially variable
PHG9_Scaffold_4990	4	p2	(TA)7	14	12013	12026	potentially variable
PHG9_Scaffold_4990	5	p2	(AT)8	16	12189	12204	potentially variable
PHG9_Scaffold_4992	1	p2	(AT)6	12	836	847	

PHG9_Scaffold_4992	2	p2	(AT)7	14	7123	7136	potentially variable
PHG9_Scaffold_4992	10	p2	(TA)8	16	34902	34917	potentially variable
PHG9_Scaffold_4993	2	p3	(AGA)5	15	13952	13966	potentially variable
PHG9_Scaffold_4993	9	p2	(TA)6	12	41291	41302	
PHG9_Scaffold_4993	10	p2	(AG)6	12	41478	41489	
PHG9_Scaffold_4994	5	p2	(GA)6	12	28500	28511	
PHG9_Scaffold_4995	1	p3	(AGA)5	15	16251	16265	potentially variable
PHG9_Scaffold_4997	9	p2	(TA)9	18	16287	16304	potentially variable
PHG9_Scaffold_4997	14	p3	(ATT)5	15	33030	33044	potentially variable
PHG9_Scaffold_4997	23	p3	(AAT)5	15	45577	45591	potentially variable
PHG9_Scaffold_4999	1	p3	(AGA)5	15	1253	1267	potentially variable
PHG9_Scaffold_4999	6	p3	(AGA)5	15	9720	9734	potentially variable
PHG9_Scaffold_4999	13	p3	(TTA)5	15	40137	40151	potentially variable
PHG9_Scaffold_4999	14	p2	(TA)6	12	48403	48414	
PHG9_Scaffold_4999	18	p2	(TA)7	14	55982	55995	potentially variable
PHG9_Scaffold_5000	3	p2	(TA)7	14	36570	36583	potentially variable
PHG9_Scaffold_5000	5	p2	(TA)6	12	38217	38228	
PHG9_Scaffold_5000	11	p2	(AT)6	12	71989	72000	

PHG9_Scaffold_5000	20	p2	(AT)7	14	121708	121721	potentially variable
PHG9_Scaffold_5000	27	p2	(TA)6	12	150318	150329	
PHG9_Scaffold_5000	30	p2	(AT)6	12	151686	151697	
PHG9_Scaffold_5000	32	p2	(AG)8	16	163131	163146	potentially variable
PHG9_Scaffold_5000	35	p2	(TA)6	12	179893	179904	
PHG9_Scaffold_5000	37	p2	(AG)8	16	182241	182256	potentially variable
PHG9_Scaffold_5000	39	p2	(GT)7	14	186218	186231	potentially variable
PHG9_Scaffold_5000	40	p2	(AT)6	12	191185	191196	
PHG9_Scaffold_5000	44	p3	(TAT)6	18	203718	203735	potentially variable
PHG9_Scaffold_5002	6	p2	(TC)7	14	10736	10749	potentially variable
PHG9_Scaffold_5002	13	p2	(AT)7	14	43101	43114	potentially variable
PHG9_Scaffold_5002	14	p2	(AT)6	12	44022	44033	
PHG9_Scaffold_5002	18	p2	(TC)9	18	50256	50273	potentially variable
PHG9_Scaffold_5002	19	p2	(TA)10	20	54985	55004	Hypervariable
PHG9_Scaffold_5002	28	p2	(AT)7	14	65404	65417	potentially variable
PHG9_Scaffold_5002	30	p3	(ATT)5	15	77437	77451	potentially variable
PHG9_Scaffold_5002	32	p2	(TC)7	14	86339	86352	potentially variable
PHG9_Scaffold_5002	33	p2	(AT)6	12	87435	87446	

PHG9_Scaffold_5002	43	p2	(AT)8	16	98968	98983	potentially variable
PHG9_Scaffold_5002	50	p2	(TA)8	16	116106	116121	potentially variable
PHG9_Scaffold_5002	56	p2	(TA)7	14	132756	132769	potentially variable
PHG9_Scaffold_5002	72	p3	(TAA)5	15	184338	184352	potentially variable
PHG9_Scaffold_5002	108	p2	(CA)6	12	262926	262937	
PHG9_Scaffold_5002	125	p2	(GA)6	12	294577	294588	
PHG9_Scaffold_5002	131	p3	(GGC)5	15	307470	307484	potentially variable
PHG9_Scaffold_5002	132	p2	(AG)6	12	309285	309296	
PHG9_Scaffold_5002	136	p2	(TA)6	12	315370	315381	
PHG9_Scaffold_5002	138	p2	(TA)6	12	323851	323862	
PHG9_Scaffold_5002	139	p2	(AT)6	12	326294	326305	
PHG9_Scaffold_5003	9	p2	(TA)6	12	12485	12496	
PHG9_Scaffold_5003	11	p2	(GA)6	12	17782	17793	
PHG9_Scaffold_5009	1	p2	(AT)9	18	4752	4769	potentially variable
PHG9_Scaffold_5009	5	p2	(AT)6	12	21186	21197	
PHG9_Scaffold_5009	10	p2	(CA)6	12	39201	39212	
PHG9_Scaffold_5009	11	p3	(TAT)5	15	39488	39502	potentially variable
PHG9_Scaffold_5009	17	p2	(TA)8	16	65539	65554	potentially variable
PHG9_Scaffold_5009	24	p2	(GA)9	18	75076	75093	potentially variable
PHG9_Scaffold_5009	25	p2	(GT)8	16	75440	75455	potentially

							variable
PHG9_Scaffold_5009	26	p2	(TA)6	12	80063	80074	
PHG9_Scaffold_5009	27	p2	(AT)9	18	85652	85669	potentially variable
PHG9_Scaffold_5009	28	p3	(ATT)5	15	89282	89296	potentially variable
PHG9_Scaffold_5010	5	p3	(TTC)5	15	20252	20266	potentially variable
PHG9_Scaffold_5010	6	p3	(TTC)6	18	21886	21903	potentially variable
PHG9_Scaffold_5010	7	p3	(TTC)6	18	22705	22722	potentially variable
PHG9_Scaffold_5010	8	p3	(TTC)5	15	23524	23538	potentially variable
PHG9_Scaffold_5011	1	p3	(ATT)5	15	23	37	potentially variable
PHG9_Scaffold_5011	7	p2	(AG)7	14	27260	27273	potentially variable
PHG9_Scaffold_5011	14	p2	(CA)6	12	74433	74444	
PHG9_Scaffold_5012	4	p2	(AT)9	18	6871	6888	potentially variable
PHG9_Scaffold_5012	5	p2	(AT)7	14	7618	7631	potentially variable
PHG9_Scaffold_5012	19	p3	(GCC)5	15	38231	38245	potentially variable
PHG9_Scaffold_5012	26	p3	(TAA)5	15	54066	54080	potentially variable
PHG9_Scaffold_5012	29	p2	(AT)6	12	59735	59746	
PHG9_Scaffold_5012	31	p2	(AC)7	14	64394	64407	potentially variable

PHG9_Scaffold_5012	34	p2	(AT)8	16	75715	75730	potentially variable
PHG9_Scaffold_5014	2	p3	(ATA)5	15	4629	4643	potentially variable
PHG9_Scaffold_5014	4	p2	(AT)7	14	15242	15255	potentially variable
PHG9_Scaffold_5014	5	p3	(AGA)5	15	19146	19160	potentially variable
PHG9_Scaffold_5014	11	p3	(TAA)5	15	61388	61402	potentially variable
PHG9_Scaffold_5014	12	p3	(GGC)6	18	62516	62533	potentially variable
PHG9_Scaffold_5015	4	p2	(TA)6	12	4773	4784	
PHG9_Scaffold_5015	6	p3	(CTT)6	18	9849	9866	potentially variable
PHG9_Scaffold_5015	7	p2	(AT)7	14	10258	10271	potentially variable
PHG9_Scaffold_5015	19	p2	(CT)7	14	31621	31634	potentially variable
PHG9_Scaffold_5016	3	p3	(TTA)6	18	17966	17983	potentially variable
PHG9_Scaffold_5016	8	p2	(AT)7	14	43275	43288	potentially variable
PHG9_Scaffold_5016	9	p2	(AT)6	12	47322	47333	
PHG9_Scaffold_5017	1	p2	(AT)6	12	457	468	
PHG9_Scaffold_5017	4	p2	(TA)7	14	3240	3253	potentially variable
PHG9_Scaffold_5017	5	p2	(AT)8	16	4753	4768	potentially variable
PHG9_Scaffold_5017	7	p2	(TA)7	14	5874	5887	potentially

							variable
PHG9_Scaffold_5018	1	p2	(AG)7	14	1607	1620	potentially variable
PHG9_Scaffold_5018	4	p2	(AT)6	12	18231	18242	
PHG9_Scaffold_5018	8	p6	(GAGCAT)5	30	39226	39255	Hypervariable
PHG9_Scaffold_5020	1	p3	(CTT)5	15	4412	4426	potentially variable
PHG9_Scaffold_5022	2	p2	(TC)6	12	14436	14447	
PHG9_Scaffold_5022	3	p3	(CAT)6	18	19415	19432	potentially variable
PHG9_Scaffold_5023	1	p2	(TC)6	12	770	781	
PHG9_Scaffold_5023	2	p2	(AT)6	12	8875	8886	
PHG9_Scaffold_5023	3	p2	(TA)6	12	9544	9555	
PHG9_Scaffold_5023	9	p2	(TA)7	14	19617	19630	potentially variable
PHG9_Scaffold_5023	11	p2	(CA)8	16	43156	43171	potentially variable
PHG9_Scaffold_5024	10	p3	(ATC)5	15	37713	37727	potentially variable
PHG9_Scaffold_5025	2	p2	(AT)6	12	2832	2843	
PHG9_Scaffold_5025	4	p2	(AT)9	18	7933	7950	potentially variable
PHG9_Scaffold_5025	7	p2	(AT)6	12	24580	24591	
PHG9_Scaffold_5025	14	p3	(CCT)5	15	39245	39259	potentially variable
PHG9_Scaffold_5027	1	p3	(ATT)5	15	424	438	potentially variable
PHG9_Scaffold_5027	7	p2	(AT)8	16	19697	19712	potentially variable

PHG9_Scaffold_5028	3	p2	(AT)8	16	6925	6940	potentially variable
PHG9_Scaffold_5028	11	p2	(AT)9	18	17537	17554	potentially variable
PHG9_Scaffold_5028	13	p2	(TC)6	12	20227	20238	
PHG9_Scaffold_5028	18	p2	(TC)7	14	26343	26356	potentially variable
PHG9_Scaffold_5028	27	p2	(AG)8	16	47602	47617	potentially variable
PHG9_Scaffold_5029	1	p2	(TA)9	18	1694	1711	potentially variable
PHG9_Scaffold_5029	3	p2	(GA)7	14	5166	5179	potentially variable
PHG9_Scaffold_5029	9	p2	(AC)6	12	15757	15768	
PHG9_Scaffold_5029	11	p3	(ATT)5	15	17237	17251	potentially variable
PHG9_Scaffold_5029	17	p2	(AT)8	16	43531	43546	potentially variable
PHG9_Scaffold_5029	38	p3	(CTA)5	15	117101	117115	potentially variable
PHG9_Scaffold_5029	40	p2	(AT)7	14	125290	125303	potentially variable
PHG9_Scaffold_5031	2	p2	(GA)7	14	5018	5031	potentially variable
PHG9_Scaffold_5031	5	p2	(TA)7	14	17780	17793	potentially variable
PHG9_Scaffold_5031	8	p2	(AT)6	12	24133	24144	
PHG9_Scaffold_5031	14	p3	(TCC)5	15	31789	31803	potentially variable
PHG9_Scaffold_5031	27	p3	(GAG)5	15	64773	64787	potentially

							variable
PHG9_Scaffold_5031	30	p2	(AT)7	14	68858	68871	potentially variable
PHG9_Scaffold_5031	32	p2	(TA)8	16	70550	70565	potentially variable
PHG9_Scaffold_5031	34	p2	(AT)8	16	72774	72789	potentially variable
PHG9_Scaffold_5032	6	p2	(TA)8	16	67573	67588	potentially variable
PHG9_Scaffold_5033	3	p2	(AT)7	14	8735	8748	potentially variable
PHG9_Scaffold_5033	16	p2	(AC)6	12	38291	38302	
PHG9_Scaffold_5033	17	p2	(TA)7	14	42598	42611	potentially variable
PHG9_Scaffold_5033	31	p2	(TA)7	14	76036	76049	potentially variable
PHG9_Scaffold_5034	3	p2	(TA)9	18	38988	39005	potentially variable
PHG9_Scaffold_5034	4	p2	(AT)7	14	40616	40629	potentially variable
PHG9_Scaffold_5034	6	p3	(ATA)5	15	51007	51021	potentially variable
PHG9_Scaffold_5034	8	p3	(AAT)6	18	68379	68396	potentially variable
PHG9_Scaffold_5034	13	p2	(TC)7	14	92373	92386	potentially variable
PHG9_Scaffold_5034	19	p2	(TA)6	12	117797	117808	
PHG9_Scaffold_5036	11	p2	(TA)9	18	58606	58623	potentially variable
PHG9_Scaffold_5037	3	p2	(AG)6	12	21513	21524	

PHG9_Scaffold_5037	4	p3	(AAT)6	18	27483	27500	potentially variable
PHG9_Scaffold_5039	3	p2	(AT)9	18	6292	6309	potentially variable
PHG9_Scaffold_5039	5	p3	(TTC)5	15	15798	15812	potentially variable
PHG9_Scaffold_5039	7	p2	(AT)6	12	16409	16420	
PHG9_Scaffold_5039	8	p2	(AT)7	14	16911	16924	potentially variable
PHG9_Scaffold_5039	9	p2	(AT)7	14	19477	19490	potentially variable
PHG9_Scaffold_5039	10	p2	(GT)9	18	23323	23340	potentially variable
PHG9_Scaffold_5039	12	p3	(AGG)5	15	24796	24810	potentially variable
PHG9_Scaffold_5041	1	p2	(CT)6	12	3779	3790	
PHG9_Scaffold_5041	8	p2	(AT)8	16	30894	30909	potentially variable
PHG9_Scaffold_5041	19	p3	(ATT)6	18	80473	80490	potentially variable
PHG9_Scaffold_5041	29	p2	(CA)7	14	131359	131372	potentially variable
PHG9_Scaffold_5041	35	p3	(ATC)5	15	150106	150120	potentially variable
PHG9_Scaffold_5041	37	p3	(TAA)5	15	155957	155971	potentially variable
PHG9_Scaffold_5041	39	p3	(CAT)6	18	156620	156637	potentially variable
PHG9_Scaffold_5043	7	p2	(AT)7	14	32360	32373	potentially variable

PHG9_Scaffold_5044	7	p2	(TA)6	12	35486	35497	
PHG9_Scaffold_5045	8	p2	(AG)6	12	32973	32984	
PHG9_Scaffold_5045	10	p3	(CGG)5	15	40199	40213	potentially variable
PHG9_Scaffold_5048	2	p3	(GAT)5	15	27814	27828	potentially variable
PHG9_Scaffold_5049	12	p2	(TA)6	12	48387	48398	
PHG9_Scaffold_5049	13	p2	(TA)7	14	57592	57605	potentially variable
PHG9_Scaffold_5049	14	p2	(AT)9	18	58774	58791	potentially variable
PHG9_Scaffold_5049	17	p2	(AT)6	12	65500	65511	
PHG9_Scaffold_5051	12	p3	(TAA)5	15	29423	29437	potentially variable
PHG9_Scaffold_5051	14	p3	(TGT)5	15	41839	41853	potentially variable
PHG9_Scaffold_5053	4	p2	(TA)7	14	4006	4019	potentially variable
PHG9_Scaffold_5053	5	p3	(TTA)5	15	7532	7546	potentially variable
PHG9_Scaffold_5053	9	p2	(AG)7	14	33142	33155	potentially variable
PHG9_Scaffold_5053	11	p3	(CTC)5	15	43588	43602	potentially variable
PHG9_Scaffold_5054	4	p3	(AAC)5	15	17155	17169	potentially variable
PHG9_Scaffold_5058	5	p3	(CAT)5	15	40037	40051	potentially variable
PHG9_Scaffold_5060	6	p2	(AT)6	12	12044	12055	
PHG9_Scaffold_5060	9	p2	(AT)7	14	16184	16197	potentially

							variable
PHG9_Scaffold_5061	4	p2	(AT)6	12	5620	5631	
PHG9_Scaffold_5061	5	p2	(TA)9	18	5763	5780	potentially variable
PHG9_Scaffold_5061	7	p2	(AT)7	14	12868	12881	potentially variable
PHG9_Scaffold_5061	12	p3	(TTA)5	15	38005	38019	potentially variable
PHG9_Scaffold_5061	15	p2	(AT)7	14	43472	43485	potentially variable
PHG9_Scaffold_5061	19	p2	(AT)6	12	50846	50857	
PHG9_Scaffold_5061	22	p2	(TC)7	14	62927	62940	potentially variable
PHG9_Scaffold_5061	25	p2	(TA)8	16	73604	73619	potentially variable
PHG9_Scaffold_5062	10	p2	(TG)6	12	22704	22715	
PHG9_Scaffold_5062	19	p2	(AT)6	12	42333	42344	
PHG9_Scaffold_5062	26	p2	(CT)6	12	56707	56718	
PHG9_Scaffold_5062	30	p2	(TA)6	12	66438	66449	
PHG9_Scaffold_5062	31	p3	(GTT)5	15	80516	80530	potentially variable
PHG9_Scaffold_5062	38	p2	(AT)7	14	100837	100850	potentially variable
PHG9_Scaffold_5062	40	p2	(AT)6	12	105689	105700	
PHG9_Scaffold_5062	42	p2	(TA)6	12	108110	108121	
PHG9_Scaffold_5062	51	p2	(TA)6	12	121104	121115	
PHG9_Scaffold_5062	52	p2	(TA)7	14	124577	124590	potentially variable
PHG9_Scaffold_5062	66	p2	(TA)7	14	152122	152135	potentially

							variable
PHG9_Scaffold_5062	73	p2	(TA)6	12	170738	170749	
PHG9_Scaffold_5062	78	p2	(AT)7	14	175843	175856	potentially variable
PHG9_Scaffold_5062	84	p2	(CT)6	12	183856	183867	
PHG9_Scaffold_5062	87	p3	(ATT)5	15	192111	192125	potentially variable
PHG9_Scaffold_5062	88	p3	(GGT)5	15	196280	196294	potentially variable
PHG9_Scaffold_5062	89	p2	(TG)6	12	199299	199310	
PHG9_Scaffold_5062	91	p3	(GAA)5	15	203970	203984	potentially variable
PHG9_Scaffold_5063	7	p3	(GTT)5	15	9075	9089	potentially variable
PHG9_Scaffold_5063	10	p2	(TA)7	14	17421	17434	potentially variable
PHG9_Scaffold_5064	7	p2	(GA)7	14	41453	41466	potentially variable
PHG9_Scaffold_5067	3	p2	(TA)7	14	9266	9279	potentially variable
PHG9_Scaffold_5067	5	p2	(TG)6	12	14458	14469	
PHG9_Scaffold_5068	4	p3	(TAT)6	18	5164	5181	potentially variable
PHG9_Scaffold_5068	5	p3	(ATA)5	15	5335	5349	potentially variable
PHG9_Scaffold_5068	7	p2	(AT)6	12	5841	5852	
PHG9_Scaffold_5068	9	p3	(CAA)5	15	7519	7533	potentially variable
PHG9_Scaffold_5068	19	p3	(TTC)5	15	43063	43077	potentially variable

PHG9_Scaffold_5070	1	p2	(TG)6	12	4600	4611	
PHG9_Scaffold_5071	2	p2	(CT)7	14	13816	13829	potentially variable
PHG9_Scaffold_5071	8	p2	(TA)6	12	61870	61881	
PHG9_Scaffold_5071	9	p3	(TAA)5	15	62955	62969	potentially variable
PHG9_Scaffold_5071	18	p2	(AT)6	12	118978	118989	
PHG9_Scaffold_5071	25	p2	(AG)7	14	148573	148586	potentially variable
PHG9_Scaffold_5072	9	p3	(TCA)6	18	6948	6965	potentially variable
PHG9_Scaffold_5072	21	p2	(TA)6	12	55787	55798	
PHG9_Scaffold_5072	26	p2	(TA)6	12	67065	67076	
PHG9_Scaffold_5072	31	p3	(AGA)5	15	75816	75830	potentially variable
PHG9_Scaffold_5072	32	p2	(CT)8	16	80172	80187	potentially variable
PHG9_Scaffold_5072	33	p3	(ATA)5	15	80806	80820	potentially variable
PHG9_Scaffold_5072	34	p2	(TG)6	12	83331	83342	
PHG9_Scaffold_5072	39	p2	(AG)8	16	94431	94446	potentially variable
PHG9_Scaffold_5072	50	p2	(TA)6	12	116066	116077	
PHG9_Scaffold_5074	2	p6	(GAGAAA)5	30	7287	7316	Hypervariable
PHG9_Scaffold_5074	3	p2	(TA)6	12	8376	8387	
PHG9_Scaffold_5075	7	p2	(AT)7	14	49101	49114	potentially variable
PHG9_Scaffold_5078	6	p2	(AT)8	16	30546	30561	potentially variable

PHG9_Scaffold_5079	5	p2	(TC)6	12	11237	11248	
PHG9_Scaffold_5081	18	p2	(AG)8	16	32957	32972	potentially variable
PHG9_Scaffold_5082	5	p2	(AG)7	14	16949	16962	potentially variable
PHG9_Scaffold_5082	8	p2	(AT)6	12	30354	30365	
PHG9_Scaffold_5084	11	p2	(AT)6	12	49211	49222	
PHG9_Scaffold_5085	1	p2	(AT)6	12	13100	13111	
PHG9_Scaffold_5085	3	p2	(CT)7	14	16074	16087	potentially variable
PHG9_Scaffold_5085	5	p2	(CT)6	12	17783	17794	
PHG9_Scaffold_5085	6	p2	(AT)8	16	19006	19021	potentially variable
PHG9_Scaffold_5085	16	p2	(TA)7	14	38340	38353	potentially variable
PHG9_Scaffold_5088	7	p3	(AAT)6	18	30859	30876	potentially variable
PHG9_Scaffold_5090	9	p2	(TA)8	16	20390	20405	potentially variable
PHG9_Scaffold_5090	13	p2	(TA)7	14	27004	27017	potentially variable
PHG9_Scaffold_5090	16	p2	(TA)6	12	38506	38517	
PHG9_Scaffold_5091	2	p2	(TA)6	12	3039	3050	
PHG9_Scaffold_5091	6	p2	(GT)6	12	11775	11786	
PHG9_Scaffold_5091	12	p2	(AT)7	14	23886	23899	potentially variable
PHG9_Scaffold_5093	3	p3	(ATA)5	15	8620	8634	potentially variable
PHG9_Scaffold_5093	4	p2	(AT)6	12	16202	16213	

PHG9_Scaffold_5093	5	p2	(CT)8	16	17083	17098	potentially variable
PHG9_Scaffold_5093	6	p3	(AAT)5	15	21875	21889	potentially variable
PHG9_Scaffold_5094	4	p2	(AT)7	14	9715	9728	potentially variable
PHG9_Scaffold_5094	11	p2	(TA)6	12	39571	39582	
PHG9_Scaffold_5094	15	p2	(TA)6	12	44133	44144	
PHG9_Scaffold_5097	6	p2	(TA)6	12	16260	16271	
PHG9_Scaffold_5097	18	p3	(CGG)5	15	45959	45973	potentially variable
PHG9_Scaffold_5097	19	p3	(TCT)5	15	46404	46418	potentially variable
PHG9_Scaffold_5097	25	p2	(AT)9	18	60914	60931	potentially variable
PHG9_Scaffold_5097	26	p2	(AT)8	16	63091	63106	potentially variable
PHG9_Scaffold_5098	2	p2	(AG)6	12	23678	23689	
PHG9_Scaffold_5099	1	p2	(AT)7	14	3545	3558	potentially variable
PHG9_Scaffold_5099	3	p3	(ACA)5	15	10936	10950	potentially variable
PHG9_Scaffold_5100	1	p2	(TC)6	12	3790	3801	
PHG9_Scaffold_5100	3	p2	(CT)6	12	8235	8246	
PHG9_Scaffold_5100	8	p2	(TA)6	12	31126	31137	
PHG9_Scaffold_5100	9	p3	(GTT)5	15	31861	31875	potentially variable
PHG9_Scaffold_5100	10	p2	(TC)7	14	32133	32146	potentially variable

PHG9_Scaffold_5100	13	p2	(AT)9	18	40320	40337	potentially variable
PHG9_Scaffold_5100	15	p2	(TA)9	18	43119	43136	potentially variable
PHG9_Scaffold_5102	6	p2	(GT)9	18	39368	39385	potentially variable
PHG9_Scaffold_5102	7	p3	(GAT)5	15	41335	41349	potentially variable
PHG9_Scaffold_5105	2	p2	(TG)6	12	2014	2025	
PHG9_Scaffold_5105	10	p3	(TAA)5	15	33292	33306	potentially variable
PHG9_Scaffold_5105	12	p3	(AAG)5	15	43900	43914	potentially variable
PHG9_Scaffold_5107	9	p2	(TA)6	12	32191	32202	
PHG9_Scaffold_5107	13	p2	(TA)7	14	35595	35608	potentially variable
PHG9_Scaffold_5109	1	p2	(AT)6	12	751	762	
PHG9_Scaffold_5109	2	p2	(AC)9	18	1895	1912	potentially variable
PHG9_Scaffold_5109	12	p2	(AT)7	14	25666	25679	potentially variable
PHG9_Scaffold_5109	15	p2	(TA)8	16	32501	32516	potentially variable
PHG9_Scaffold_5109	16	p2	(TC)7	14	33041	33054	potentially variable
PHG9_Scaffold_5110	1	p2	(TA)8	16	610	625	potentially variable
PHG9_Scaffold_5110	11	p2	(TA)6	12	22792	22803	
PHG9_Scaffold_5110	14	p3	(TGC)5	15	25778	25792	potentially variable

PHG9_Scaffold_5110	17	p2	(TA)7	14	35056	35069	potentially variable
PHG9_Scaffold_5110	18	p2	(CT)6	12	35813	35824	
PHG9_Scaffold_5110	19	p2	(AT)7	14	35977	35990	potentially variable
PHG9_Scaffold_5110	20	p3	(TCA)5	15	42741	42755	potentially variable
PHG9_Scaffold_5110	25	p3	(TAT)5	15	50404	50418	potentially variable
PHG9_Scaffold_5111	8	p3	(ATT)6	18	22415	22432	potentially variable
PHG9_Scaffold_5111	12	p2	(TA)6	12	42438	42449	
PHG9_Scaffold_5112	2	p2	(TA)8	16	35196	35211	potentially variable
PHG9_Scaffold_5113	2	p3	(AAT)5	15	5244	5258	potentially variable
PHG9_Scaffold_5113	7	p2	(AG)8	16	24124	24139	potentially variable
PHG9_Scaffold_5113	10	p2	(AG)6	12	36101	36112	
PHG9_Scaffold_5113	13	p2	(AT)6	12	52984	52995	
PHG9_Scaffold_5114	6	p2	(AT)9	18	23408	23425	potentially variable
PHG9_Scaffold_5114	8	p3	(AAC)5	15	26581	26595	potentially variable
PHG9_Scaffold_5114	11	p2	(CT)6	12	29362	29373	
PHG9_Scaffold_5114	19	p3	(ATC)5	15	43745	43759	potentially variable
PHG9_Scaffold_5115	6	p3	(ATA)5	15	27038	27052	potentially variable
PHG9_Scaffold_5120	6	p2	(TA)8	16	16203	16218	potentially

							variable
PHG9_Scaffold_5120	17	p3	(GAT)5	15	74594	74608	potentially variable
PHG9_Scaffold_5120	18	p2	(AT)6	12	78200	78211	
PHG9_Scaffold_5120	19	p2	(CA)7	14	81113	81126	potentially variable
PHG9_Scaffold_5120	20	p2	(TA)7	14	81428	81441	potentially variable
PHG9_Scaffold_5121	11	p2	(GA)7	14	34973	34986	potentially variable
PHG9_Scaffold_5121	13	p2	(AT)8	16	36878	36893	potentially variable
PHG9_Scaffold_5123	1	p3	(CTT)5	15	675	689	potentially variable
PHG9_Scaffold_5124	1	p2	(GT)6	12	824	835	
PHG9_Scaffold_5124	2	p3	(ATA)5	15	1940	1954	potentially variable
PHG9_Scaffold_5125	1	p2	(TA)7	14	400	413	potentially variable
PHG9_Scaffold_5125	3	p3	(TAA)5	15	6567	6581	potentially variable
PHG9_Scaffold_5125	6	p2	(TA)7	14	12794	12807	potentially variable
PHG9_Scaffold_5125	9	p2	(CT)6	12	23842	23853	
PHG9_Scaffold_5126	8	p2	(TA)7	14	13967	13980	potentially variable
PHG9_Scaffold_5129	2	p2	(TA)6	12	2721	2732	
PHG9_Scaffold_5133	1	p3	(GAA)5	15	6616	6630	potentially variable
PHG9_Scaffold_5134	6	p3	(TTA)6	18	14493	14510	potentially

							variable
PHG9_Scaffold_5134	13	p2	(TC)6	12	36565	36576	
PHG9_Scaffold_5134	15	p2	(AT)7	14	37335	37348	potentially variable
PHG9_Scaffold_5134	18	p2	(TA)9	18	40558	40575	potentially variable
PHG9_Scaffold_5134	23	p3	(TGA)6	18	60887	60904	potentially variable
PHG9_Scaffold_5137	2	p3	(ATA)6	18	4820	4837	potentially variable
PHG9_Scaffold_5138	1	p3	(ATG)5	15	459	473	potentially variable
PHG9_Scaffold_5142	7	p3	(CTT)5	15	13600	13614	potentially variable
PHG9_Scaffold_5142	8	p3	(TTA)5	15	18019	18033	potentially variable
PHG9_Scaffold_5142	12	p2	(TC)7	14	24032	24045	potentially variable
PHG9_Scaffold_5143	2	p2	(AC)8	16	8048	8063	potentially variable
PHG9_Scaffold_5143	4	p2	(TA)7	14	14700	14713	potentially variable
PHG9_Scaffold_5143	12	p2	(GA)9	18	28530	28547	potentially variable
PHG9_Scaffold_5144	3	p2	(AG)6	12	5346	5357	
PHG9_Scaffold_5144	6	p2	(TA)8	16	18410	18425	potentially variable
PHG9_Scaffold_5145	1	p2	(GA)7	14	204	217	potentially variable
PHG9_Scaffold_5145	11	p2	(GA)6	12	19715	19726	

PHG9_Scaffold_5146	3	p2	(TA)6	12	8725	8736	
PHG9_Scaffold_5146	7	p2	(TA)7	14	12918	12931	potentially variable
PHG9_Scaffold_5147	5	p3	(ATC)5	15	17856	17870	potentially variable
PHG9_Scaffold_5147	7	p3	(GTG)5	15	23482	23496	potentially variable
PHG9_Scaffold_5150	1	p2	(TA)8	16	1	16	potentially variable
PHG9_Scaffold_5150	12	p2	(AT)9	18	29645	29662	potentially variable
PHG9_Scaffold_5151	6	p2	(AT)6	12	21814	21825	
PHG9_Scaffold_5153	1	p3	(ATA)6	18	3937	3954	potentially variable
PHG9_Scaffold_5153	5	p2	(TC)6	12	13081	13092	
PHG9_Scaffold_5153	10	p3	(CCA)6	18	29597	29614	potentially variable
PHG9_Scaffold_5155	16	p2	(AG)6	12	31837	31848	
PHG9_Scaffold_5158	3	p3	(TCC)5	15	6979	6993	potentially variable
PHG9_Scaffold_5158	10	p2	(GT)6	12	32847	32858	
PHG9_Scaffold_5158	11	p3	(AAG)5	15	33274	33288	potentially variable
PHG9_Scaffold_5159	7	p3	(TTA)6	18	14957	14974	potentially variable
PHG9_Scaffold_5159	10	p2	(TA)6	12	19176	19187	
PHG9_Scaffold_5161	5	p3	(CAT)5	15	17562	17576	potentially variable
PHG9_Scaffold_5162	1	p2	(TA)6	12	1997	2008	

PHG9_Scaffold_5162	5	p2	(TA)7	14	11099	11112	potentially variable
PHG9_Scaffold_5162	8	p2	(AT)6	12	17390	17401	
PHG9_Scaffold_5163	5	p2	(AT)7	14	20457	20470	potentially variable
PHG9_Scaffold_5163	7	p2	(AG)8	16	26308	26323	potentially variable
PHG9_Scaffold_5163	9	p2	(TC)7	14	35318	35331	potentially variable
PHG9_Scaffold_5166	4	p2	(TA)6	12	9278	9289	
PHG9_Scaffold_5171	1	p2	(TG)9	18	16	33	potentially variable
PHG9_Scaffold_5176	6	p2	(AG)6	12	5976	5987	
PHG9_Scaffold_5176	8	p2	(AC)9	18	9669	9686	potentially variable
PHG9_Scaffold_5178	4	p2	(AT)7	14	3133	3146	potentially variable
PHG9_Scaffold_5178	15	p3	(GTG)5	15	16083	16097	potentially variable
PHG9_Scaffold_5179	9	p2	(TC)7	14	18152	18165	potentially variable
PHG9_Scaffold_5179	14	p2	(CT)7	14	38126	38139	potentially variable
PHG9_Scaffold_5180	6	p2	(AC)6	12	11351	11362	
PHG9_Scaffold_5180	8	p3	(GGT)6	18	14056	14073	potentially variable
PHG9_Scaffold_5180	13	p3	(TGA)5	15	32244	32258	potentially variable
PHG9_Scaffold_5181	2	p2	(AT)9	18	2508	2525	potentially variable

PHG9_Scaffold_5183	3	p2	(AT)7	14	15463	15476	potentially variable
PHG9_Scaffold_5183	12	p2	(AT)7	14	85908	85921	potentially variable
PHG9_Scaffold_5183	23	p3	(TTA)5	15	186471	186485	potentially variable
PHG9_Scaffold_5183	24	p2	(CT)8	16	202879	202894	potentially variable
PHG9_Scaffold_5183	27	p2	(TC)6	12	220926	220937	
PHG9_Scaffold_5183	36	p2	(TA)6	12	258922	258933	
PHG9_Scaffold_5186	2	p2	(AG)6	12	16888	16899	
PHG9_Scaffold_5186	3	p2	(TA)6	12	17266	17277	
PHG9_Scaffold_5187	6	p3	(TAA)5	15	31249	31263	potentially variable
PHG9_Scaffold_5187	8	p3	(AGA)5	15	44489	44503	potentially variable
PHG9_Scaffold_5187	9	p2	(AT)9	18	47767	47784	potentially variable
PHG9_Scaffold_5187	11	p2	(AT)7	14	53460	53473	potentially variable
PHG9_Scaffold_5187	12	p2	(AT)6	12	55109	55120	
PHG9_Scaffold_5191	2	p2	(AT)6	12	9067	9078	
PHG9_Scaffold_5194	1	p2	(TA)7	14	486	499	potentially variable
PHG9_Scaffold_5194	5	p3	(TAT)5	15	14449	14463	potentially variable
PHG9_Scaffold_5194	7	p2	(AT)9	18	18122	18139	potentially variable
PHG9_Scaffold_5194	13	p2	(TA)6	12	50955	50966	

PHG9_Scaffold_5194	14	p2	(TA)6	12	54610	54621	
PHG9_Scaffold_5195	4	p3	(AAT)5	15	6481	6495	potentially variable
PHG9_Scaffold_5195	9	p3	(TTC)6	18	16544	16561	potentially variable
PHG9_Scaffold_5195	11	p2	(AG)6	12	21587	21598	
PHG9_Scaffold_5197	8	p3	(ATT)6	18	31344	31361	potentially variable
PHG9_Scaffold_5199	2	p2	(TA)7	14	19546	19559	potentially variable
PHG9_Scaffold_5201	3	p2	(AT)6	12	28423	28434	
PHG9_Scaffold_5204	4	p2	(AT)6	12	18103	18114	
PHG9_Scaffold_5204	6	p2	(TA)6	12	22740	22751	
PHG9_Scaffold_5204	12	p2	(TA)6	12	28518	28529	
PHG9_Scaffold_5205	2	p2	(AG)6	12	3704	3715	
PHG9_Scaffold_5208	1	p3	(GAT)5	15	2613	2627	potentially variable
PHG9_Scaffold_5209	2	p2	(GA)6	12	7146	7157	
PHG9_Scaffold_5211	8	p2	(AG)6	12	22084	22095	
PHG9_Scaffold_5211	9	p3	(TAA)5	15	22830	22844	potentially variable
PHG9_Scaffold_5211	10	p2	(TA)7	14	24072	24085	potentially variable
PHG9_Scaffold_5214	5	p2	(TA)6	12	4659	4670	
PHG9_Scaffold_5214	9	p2	(GA)7	14	15094	15107	potentially variable
PHG9_Scaffold_5214	14	p2	(TA)6	12	46878	46889	
PHG9_Scaffold_5218	1	p2	(AG)9	18	11633	11650	potentially variable

PHG9_Scaffold_5221	1	p3	(AAG)5	15	10589	10603	potentially variable
PHG9_Scaffold_5223	2	p2	(TA)6	12	7192	7203	
PHG9_Scaffold_5229	2	p2	(AT)9	18	10922	10939	potentially variable
PHG9_Scaffold_5230	1	p3	(AAT)5	15	3340	3354	potentially variable
PHG9_Scaffold_5230	3	p2	(TA)6	12	12332	12343	
PHG9_Scaffold_5231	3	p3	(CTT)5	15	3239	3253	potentially variable
PHG9_Scaffold_5231	4	p2	(TA)6	12	15975	15986	
PHG9_Scaffold_5231	5	p2	(GT)6	12	19980	19991	
PHG9_Scaffold_5231	8	p2	(TA)9	18	23909	23926	potentially variable
PHG9_Scaffold_5232	3	p3	(TTA)6	18	7347	7364	potentially variable
PHG9_Scaffold_5232	11	p2	(AT)6	12	32297	32308	
PHG9_Scaffold_5233	3	p2	(AG)8	16	14789	14804	potentially variable
PHG9_Scaffold_5233	4	p3	(AGA)5	15	15869	15883	potentially variable
PHG9_Scaffold_5237	4	p2	(AT)7	14	17158	17171	potentially variable
PHG9_Scaffold_5238	5	p2	(TA)6	12	17320	17331	
PHG9_Scaffold_5238	6	p3	(TTA)5	15	22987	23001	potentially variable
PHG9_Scaffold_5238	9	p3	(TAA)5	15	25043	25057	potentially variable
PHG9_Scaffold_5238	14	p3	(ATT)6	18	35992	36009	potentially variable

PHG9_Scaffold_5239	5	p3	(CAT)5	15	17542	17556	potentially variable
PHG9_Scaffold_5240	3	p2	(TA)7	14	11301	11314	potentially variable
PHG9_Scaffold_5241	7	p2	(AT)8	16	31064	31079	potentially variable
PHG9_Scaffold_5241	8	p2	(AT)7	14	31548	31561	potentially variable
PHG9_Scaffold_5245	3	p2	(CT)6	12	9863	9874	
PHG9_Scaffold_5246	1	p2	(TA)6	12	13979	13990	
PHG9_Scaffold_5247	2	p2	(TA)6	12	1767	1778	
PHG9_Scaffold_5247	9	p2	(AT)6	12	25174	25185	
PHG9_Scaffold_5248	9	p2	(AT)6	12	17845	17856	
PHG9_Scaffold_5251	2	p3	(TTA)5	15	7260	7274	potentially variable
PHG9_Scaffold_5251	3	p2	(AG)6	12	9078	9089	
PHG9_Scaffold_5251	7	p3	(TGT)5	15	13226	13240	potentially variable
PHG9_Scaffold_5252	2	p2	(TA)6	12	5724	5735	
PHG9_Scaffold_5254	2	p2	(TA)7	14	5801	5814	potentially variable
PHG9_Scaffold_5254	6	p2	(TA)6	12	8447	8458	
PHG9_Scaffold_5254	8	p3	(ACT)5	15	23657	23671	potentially variable
PHG9_Scaffold_5256	4	p2	(TA)7	14	6322	6335	potentially variable
PHG9_Scaffold_5256	9	p2	(AC)6	12	18820	18831	
PHG9_Scaffold_5257	4	p3	(TAT)5	15	9841	9855	potentially variable

PHG9_Scaffold_5260	4	p2	(TA)6	12	8320	8331	
PHG9_Scaffold_5263	7	p2	(AT)8	16	17159	17174	potentially variable
PHG9_Scaffold_5264	5	p2	(AG)8	16	3504	3519	potentially variable
PHG9_Scaffold_5268	7	p2	(AT)8	16	18498	18513	potentially variable
PHG9_Scaffold_5270	1	p2	(TA)7	14	487	500	potentially variable
PHG9_Scaffold_5272	1	p2	(TA)8	16	3560	3575	potentially variable
PHG9_Scaffold_5276	2	p3	(GTG)5	15	5031	5045	potentially variable
PHG9_Scaffold_5277	3	p2	(TA)6	12	10464	10475	
PHG9_Scaffold_5277	4	p2	(AT)6	12	11689	11700	
PHG9_Scaffold_5277	5	p2	(TA)6	12	12271	12282	
PHG9_Scaffold_5280	7	p2	(GA)8	16	24250	24265	potentially variable
PHG9_Scaffold_5287	3	p2	(AT)8	16	10964	10979	potentially variable
PHG9_Scaffold_5288	4	p2	(AT)6	12	10472	10483	
PHG9_Scaffold_5288	5	p3	(AAT)5	15	17497	17511	potentially variable
PHG9_Scaffold_5288	6	p3	(ATA)5	15	17755	17769	potentially variable
PHG9_Scaffold_5288	7	p2	(TA)6	12	19285	19296	
PHG9_Scaffold_5289	8	p2	(AT)10	20	17119	17138	Hypervariable
PHG9_Scaffold_5289	9	p2	(TA)6	12	18456	18467	
PHG9_Scaffold_5289	10	p2	(AT)9	18	19258	19275	potentially

							variable
PHG9_Scaffold_5289	11	p3	(CAC)5	15	19787	19801	potentially variable
PHG9_Scaffold_5296	5	p3	(AAT)5	15	13420	13434	potentially variable
PHG9_Scaffold_5296	7	p2	(TA)6	12	17698	17709	
PHG9_Scaffold_5297	2	p2	(AT)8	16	1214	1229	potentially variable
PHG9_Scaffold_5308	5	p2	(AG)8	16	35289	35304	potentially variable
PHG9_Scaffold_5308	7	p2	(AG)7	14	49846	49859	potentially variable
PHG9_Scaffold_5313	1	p3	(ATA)5	15	1121	1135	potentially variable
PHG9_Scaffold_5313	3	p3	(TGG)5	15	16487	16501	potentially variable
PHG9_Scaffold_5321	3	p2	(AT)8	16	4302	4317	potentially variable
PHG9_Scaffold_5327	1	p3	(ATT)5	15	135	149	potentially variable
PHG9_Scaffold_5330	2	p2	(AT)7	14	8951	8964	potentially variable
PHG9_Scaffold_5331	1	p2	(TA)7	14	7349	7362	potentially variable
PHG9_Scaffold_5341	9	p2	(AT)7	14	26348	26361	potentially variable
PHG9_Scaffold_5342	2	p2	(TA)8	16	1612	1627	potentially variable
PHG9_Scaffold_5344	3	p2	(TA)6	12	6031	6042	
PHG9_Scaffold_5351	1	p2	(TA)6	12	2385	2396	

PHG9_Scaffold_5351	2	p2	(AT)7	14	2604	2617	potentially variable
PHG9_Scaffold_5351	5	p2	(AG)7	14	13307	13320	potentially variable
PHG9_Scaffold_5355	1	p2	(TA)6	12	2945	2956	
PHG9_Scaffold_5357	1	p2	(TA)7	14	2211	2224	potentially variable
PHG9_Scaffold_5366	2	p2	(GT)6	12	15736	15747	
PHG9_Scaffold_5375	3	p2	(AT)8	16	1701	1716	potentially variable
PHG9_Scaffold_5376	1	p2	(AT)7	14	746	759	potentially variable
PHG9_Scaffold_5376	4	p2	(AG)7	14	12035	12048	potentially variable
PHG9_Scaffold_5378	1	p2	(AT)6	12	6977	6988	
PHG9_Scaffold_5380	4	p3	(ATT)5	15	6807	6821	potentially variable
PHG9_Scaffold_5380	8	p3	(ATG)5	15	8399	8413	potentially variable
PHG9_Scaffold_5382	3	p2	(AT)9	18	5759	5776	potentially variable
PHG9_Scaffold_5382	4	p2	(CT)7	14	6559	6572	potentially variable
PHG9_Scaffold_5383	2	p2	(AT)7	14	9398	9411	potentially variable
PHG9_Scaffold_5383	5	p2	(AG)9	18	12714	12731	potentially variable
PHG9_Scaffold_5388	2	p2	(AC)7	14	5336	5349	potentially variable
PHG9_Scaffold_5389	4	p2	(GA)6	12	8727	8738	

PHG9_Scaffold_5391	2	p2	(GA)7	14	1550	1563	potentially variable
PHG9_Scaffold_5392	1	p2	(TA)7	14	11946	11959	potentially variable
PHG9_Scaffold_5396	6	p2	(AC)9	18	15110	15127	potentially variable
PHG9_Scaffold_5396	11	p2	(CA)7	14	20442	20455	potentially variable
PHG9_Scaffold_5399	1	p2	(AT)8	16	3679	3694	potentially variable
PHG9_Scaffold_5399	3	p2	(TA)8	16	5536	5551	potentially variable
PHG9_Scaffold_5410	1	p3	(AGA)5	15	502	516	potentially variable
PHG9_Scaffold_5427	2	p3	(TGG)5	15	6360	6374	potentially variable
PHG9_Scaffold_5431	2	p2	(AG)6	12	2891	2902	
PHG9_Scaffold_5434	2	p2	(AT)6	12	4474	4485	
PHG9_Scaffold_5435	3	p3	(AAT)5	15	1259	1273	potentially variable
PHG9_Scaffold_5439	6	p3	(TAT)5	15	13850	13864	potentially variable
PHG9_Scaffold_5441	6	p3	(ATT)5	15	9202	9216	potentially variable
PHG9_Scaffold_5442	3	p2	(TA)7	14	6015	6028	potentially variable
PHG9_Scaffold_5442	5	p2	(AT)8	16	8192	8207	potentially variable
PHG9_Scaffold_5442	10	p2	(TC)7	14	13941	13954	potentially variable

PHG9_Scaffold_5450	1	p2	(GA)6	12	4214	4225	
PHG9_Scaffold_5452	3	p3	(TTA)6	18	9266	9283	potentially variable
PHG9_Scaffold_5460	3	p2	(AG)6	12	11633	11644	
PHG9_Scaffold_5460	4	p3	(CAA)6	18	11959	11976	potentially variable
PHG9_Scaffold_5461	1	p3	(TAA)5	15	5824	5838	potentially variable
PHG9_Scaffold_5463	2	p3	(ATC)5	15	5335	5349	potentially variable
PHG9_Scaffold_5465	2	p2	(AT)6	12	15085	15096	
PHG9_Scaffold_5509	1	p3	(TTC)5	15	11352	11366	potentially variable
PHG9_Scaffold_5514	2	p2	(AC)6	12	19992	20003	
PHG9_Scaffold_5543	3	p2	(AT)6	12	5917	5928	
PHG9_Scaffold_5543	4	p2	(AT)7	14	6261	6274	potentially variable
PHG9_Scaffold_5543	8	p6	(AAAACT)6	36	12620	12655	Hypervariable
PHG9_Scaffold_5543	10	p2	(GA)6	12	20754	20765	
PHG9_Scaffold_5543	23	p2	(TA)6	12	52084	52095	
PHG9_Scaffold_5558	3	p3	(AAG)5	15	35589	35603	potentially variable
PHG9_Scaffold_5582	1	p3	(AAG)5	15	1174	1188	potentially variable
PHG9_Scaffold_5619	4	p3	(GAA)5	15	8751	8765	potentially variable
PHG9_Scaffold_5619	8	p3	(GTT)5	15	21778	21792	potentially variable
PHG9_Scaffold_5619	12	p2	(TA)7	14	51635	51648	potentially

							variable
PHG9_Scaffold_5619	27	p2	(TA)6	12	97019	97030	
PHG9_Scaffold_5619	32	p2	(TA)6	12	109456	109467	
PHG9_Scaffold_5619	39	p3	(AAT)5	15	134794	134808	potentially variable
PHG9_Scaffold_5619	41	p2	(TA)9	18	139807	139824	potentially variable
PHG9_Scaffold_5619	42	p3	(GAT)6	18	142854	142871	potentially variable
PHG9_Scaffold_5619	43	p2	(TA)6	12	144929	144940	
PHG9_Scaffold_5619	44	p2	(TA)6	12	145813	145824	
PHG9_Scaffold_5619	45	p2	(CA)6	12	148724	148735	
PHG9_Scaffold_5619	46	p3	(AGA)6	18	150700	150717	potentially variable
PHG9_Scaffold_5619	57	p2	(TA)9	18	170465	170482	potentially variable
PHG9_Scaffold_5619	58	p2	(TA)6	12	170955	170966	
PHG9_Scaffold_5619	63	p2	(CT)7	14	182464	182477	potentially variable
PHG9_Scaffold_5619	64	p2	(TA)6	12	184565	184576	
PHG9_Scaffold_5619	70	p2	(AG)6	12	199102	199113	
PHG9_Scaffold_5620	2	p2	(TA)8	16	13260	13275	potentially variable
PHG9_Scaffold_5621	1	p2	(TA)6	12	1530	1541	
PHG9_Scaffold_5621	6	p2	(TA)7	14	21538	21551	potentially variable
PHG9_Scaffold_5622	2	p2	(AT)9	18	1758	1775	potentially variable
PHG9_Scaffold_5622	3	p2	(TA)8	16	2191	2206	potentially

							variable
PHG9_Scaffold_5622	5	p2	(AT)7	14	7625	7638	potentially variable
PHG9_Scaffold_5623	9	p2	(TA)6	12	65461	65472	
PHG9_Scaffold_5623	15	p2	(TA)6	12	106599	106610	
PHG9_Scaffold_5623	17	p2	(TA)7	14	127004	127017	potentially variable
PHG9_Scaffold_5625	2	p2	(TA)6	12	28352	28363	
PHG9_Scaffold_5625	5	p2	(TA)7	14	53540	53553	potentially variable
PHG9_Scaffold_5625	12	p3	(AGG)5	15	74348	74362	potentially variable
PHG9_Scaffold_5625	13	p3	(ATA)5	15	79888	79902	potentially variable
PHG9_Scaffold_5626	1	p2	(AT)8	16	13751	13766	potentially variable
PHG9_Scaffold_5626	10	p2	(TA)6	12	74949	74960	
PHG9_Scaffold_5627	27	p2	(TA)8	16	105675	105690	potentially variable
PHG9_Scaffold_5627	32	p2	(TA)6	12	124674	124685	
PHG9_Scaffold_5627	38	p3	(ATC)5	15	139272	139286	potentially variable
PHG9_Scaffold_5627	50	p2	(AT)9	18	166715	166732	potentially variable
PHG9_Scaffold_5628	3	p2	(TA)6	12	19057	19068	
PHG9_Scaffold_5628	23	p2	(AT)6	12	228954	228965	
PHG9_Scaffold_5628	28	p2	(AT)6	12	248556	248567	
PHG9_Scaffold_5628	37	p3	(TAT)5	15	295370	295384	potentially variable

PHG9_Scaffold_5628	38	p3	(GCC)5	15	297428	297442	potentially variable
PHG9_Scaffold_5630	3	p3	(TGT)6	18	19134	19151	potentially variable
PHG9_Scaffold_5630	6	p2	(AT)6	12	22693	22704	
PHG9_Scaffold_5630	13	p2	(GA)6	12	39596	39607	
PHG9_Scaffold_5630	17	p2	(AC)7	14	84295	84308	potentially variable
PHG9_Scaffold_5630	30	p2	(TC)8	16	169814	169829	potentially variable
PHG9_Scaffold_5630	33	p3	(ATG)5	15	179312	179326	potentially variable
PHG9_Scaffold_5630	34	p2	(TA)7	14	185091	185104	potentially variable
PHG9_Scaffold_5632	4	p3	(CTT)6	18	7443	7460	potentially variable
PHG9_Scaffold_5632	17	p3	(TTA)5	15	122390	122404	potentially variable
PHG9_Scaffold_5633	3	p3	(AAT)5	15	12082	12096	potentially variable
PHG9_Scaffold_5633	5	p3	(CTT)6	18	13363	13380	potentially variable
PHG9_Scaffold_5633	7	p3	(AAT)5	15	16462	16476	potentially variable
PHG9_Scaffold_5633	9	p3	(AAG)5	15	19245	19259	potentially variable
PHG9_Scaffold_5633	10	p3	(AAT)5	15	21644	21658	potentially variable
PHG9_Scaffold_5633	12	p3	(AGA)6	18	31177	31194	potentially variable

PHG9_Scaffold_5633	14	p2	(CT)6	12	36832	36843	
PHG9_Scaffold_5633	17	p3	(GAG)5	15	43689	43703	potentially variable
PHG9_Scaffold_5633	18	p2	(AT)8	16	49224	49239	potentially variable
PHG9_Scaffold_5633	19	p2	(TA)9	18	55311	55328	potentially variable
PHG9_Scaffold_5633	22	p3	(AAG)5	15	65345	65359	potentially variable
PHG9_Scaffold_5633	23	p3	(TAG)6	18	66164	66181	potentially variable
PHG9_Scaffold_5633	26	p3	(CAT)5	15	78435	78449	potentially variable
PHG9_Scaffold_5633	31	p2	(GT)8	16	86312	86327	potentially variable
PHG9_Scaffold_5633	33	p3	(GAA)5	15	93394	93408	potentially variable
PHG9_Scaffold_5633	40	p3	(TAA)6	18	115952	115969	potentially variable
PHG9_Scaffold_5633	48	p2	(TC)7	14	139504	139517	potentially variable
PHG9_Scaffold_5633	49	p2	(GA)6	12	145706	145717	
PHG9_Scaffold_5633	54	p2	(AT)9	18	154678	154695	potentially variable
PHG9_Scaffold_5633	58	p3	(AAG)6	18	168321	168338	potentially variable
PHG9_Scaffold_5633	59	p2	(CT)9	18	168575	168592	potentially variable
PHG9_Scaffold_5633	62	p2	(TA)7	14	169584	169597	potentially variable

PHG9_Scaffold_5633	68	p3	(TAT)6	18	183131	183148	potentially variable
PHG9_Scaffold_5633	79	p2	(AT)9	18	206416	206433	potentially variable
PHG9_Scaffold_5633	82	p3	(ATG)5	15	209409	209423	potentially variable
PHG9_Scaffold_5634	5	p2	(AT)6	12	15045	15056	
PHG9_Scaffold_5634	8	p2	(TA)6	12	18889	18900	
PHG9_Scaffold_5634	18	p2	(TA)7	14	41904	41917	potentially variable
PHG9_Scaffold_5634	24	p2	(GT)6	12	74323	74334	
PHG9_Scaffold_5634	30	p2	(TA)9	18	96558	96575	potentially variable
PHG9_Scaffold_5634	31	p2	(AT)6	12	110492	110503	
PHG9_Scaffold_5634	39	p2	(AT)8	16	128203	128218	potentially variable
PHG9_Scaffold_5635	10	p2	(AT)7	14	50861	50874	potentially variable
PHG9_Scaffold_5635	13	p2	(TA)8	16	61669	61684	potentially variable
PHG9_Scaffold_5635	16	p2	(TA)7	14	65515	65528	potentially variable
PHG9_Scaffold_5635	19	p3	(TTG)5	15	76486	76500	potentially variable
PHG9_Scaffold_5635	23	p3	(AAT)5	15	89620	89634	potentially variable
PHG9_Scaffold_5635	29	p2	(TG)7	14	129144	129157	potentially variable
PHG9_Scaffold_5636	3	p2	(AT)7	14	3055	3068	potentially variable

PHG9_Scaffold_5636	13	p2	(AT)7	14	48083	48096	potentially variable
PHG9_Scaffold_5637	3	p2	(AT)6	12	5956	5967	
PHG9_Scaffold_5637	4	p2	(AT)6	12	8274	8285	
PHG9_Scaffold_5637	7	p3	(TTA)5	15	11955	11969	potentially variable
PHG9_Scaffold_5637	11	p3	(TAA)5	15	29872	29886	potentially variable
PHG9_Scaffold_5637	21	p3	(TAA)5	15	60688	60702	potentially variable
PHG9_Scaffold_5637	22	p2	(AT)6	12	61329	61340	
PHG9_Scaffold_5637	28	p2	(TC)6	12	73905	73916	
PHG9_Scaffold_5637	32	p2	(TC)6	12	90111	90122	
PHG9_Scaffold_5638	4	p2	(AT)7	14	12700	12713	potentially variable
PHG9_Scaffold_5638	5	p2	(TC)6	12	13734	13745	
PHG9_Scaffold_5638	8	p3	(GAA)5	15	20210	20224	potentially variable
PHG9_Scaffold_5638	25	p2	(AT)6	12	44407	44418	
PHG9_Scaffold_5638	26	p2	(AG)6	12	45350	45361	
PHG9_Scaffold_5638	27	p3	(TTA)5	15	46870	46884	potentially variable
PHG9_Scaffold_5638	30	p3	(AGA)5	15	62957	62971	potentially variable
PHG9_Scaffold_5638	32	p2	(TG)6	12	65345	65356	
PHG9_Scaffold_5638	41	p3	(TAA)5	15	91137	91151	potentially variable
PHG9_Scaffold_5638	45	p2	(AT)6	12	97282	97293	
PHG9_Scaffold_5638	55	p2	(TA)7	14	129100	129113	potentially

							variable
PHG9_Scaffold_5639	4	p2	(CA)6	12	30067	30078	
PHG9_Scaffold_5640	6	p2	(AC)6	12	16626	16637	
PHG9_Scaffold_5641	3	p2	(TA)6	12	15804	15815	
PHG9_Scaffold_5641	6	p2	(TA)7	14	21591	21604	potentially variable
PHG9_Scaffold_5641	18	p3	(GTG)5	15	40121	40135	potentially variable
PHG9_Scaffold_5641	24	p3	(GTG)5	15	56681	56695	potentially variable
PHG9_Scaffold_5641	28	p2	(TA)6	12	63655	63666	
PHG9_Scaffold_5641	32	p2	(AT)6	12	87411	87422	
PHG9_Scaffold_5644	5	p2	(AT)6	12	34126	34137	
PHG9_Scaffold_5645	10	p2	(TA)7	14	60349	60362	potentially variable
PHG9_Scaffold_5645	14	p2	(TA)7	14	74949	74962	potentially variable
PHG9_Scaffold_5646	2	p2	(TA)6	12	3157	3168	
PHG9_Scaffold_5646	3	p2	(AT)8	16	4858	4873	potentially variable
PHG9_Scaffold_5646	4	p2	(AT)7	14	6579	6592	potentially variable
PHG9_Scaffold_5646	9	p2	(TC)7	14	32937	32950	potentially variable
PHG9_Scaffold_5646	17	p2	(AT)6	12	49269	49280	
PHG9_Scaffold_5647	10	p2	(CA)7	14	18527	18540	potentially variable
PHG9_Scaffold_5647	31	p2	(AT)6	12	101827	101838	
PHG9_Scaffold_5648	1	p2	(AT)8	16	1735	1750	potentially

							variable
PHG9_Scaffold_5648	4	p3	(GGA)6	18	10193	10210	potentially variable
PHG9_Scaffold_5648	5	p2	(AT)8	16	13832	13847	potentially variable
PHG9_Scaffold_5648	19	p2	(TA)9	18	30839	30856	potentially variable
PHG9_Scaffold_5648	20	p2	(TA)6	12	31648	31659	
PHG9_Scaffold_5648	21	p2	(AG)6	12	31942	31953	
PHG9_Scaffold_5648	24	p2	(AT)6	12	39807	39818	
PHG9_Scaffold_5648	27	p2	(TA)8	16	42849	42864	potentially variable
PHG9_Scaffold_5649	10	p2	(TA)6	12	25390	25401	
PHG9_Scaffold_5651	8	p3	(AAT)6	18	28071	28088	potentially variable
PHG9_Scaffold_5653	1	p3	(CAA)5	15	10864	10878	potentially variable
PHG9_Scaffold_5654	7	p3	(GTA)5	15	27358	27372	potentially variable
PHG9_Scaffold_5655	8	p2	(CA)6	12	20661	20672	
PHG9_Scaffold_5655	11	p2	(TA)8	16	35318	35333	potentially variable
PHG9_Scaffold_5655	23	p2	(CT)8	16	82543	82558	potentially variable
PHG9_Scaffold_5655	25	p2	(AT)6	12	91534	91545	
PHG9_Scaffold_5655	28	p2	(AC)6	12	108613	108624	
PHG9_Scaffold_5655	37	p3	(CGT)5	15	141502	141516	potentially variable
PHG9_Scaffold_5655	38	p2	(AT)9	18	141710	141727	potentially

							variable
PHG9_Scaffold_5655	41	p3	(GAA)5	15	152711	152725	potentially variable
PHG9_Scaffold_5655	44	p3	(TAG)6	18	172711	172728	potentially variable
PHG9_Scaffold_5655	49	p3	(AAT)6	18	187468	187485	potentially variable
PHG9_Scaffold_5655	51	p2	(CA)8	16	193888	193903	potentially variable
PHG9_Scaffold_5655	52	p2	(TA)7	14	197089	197102	potentially variable
PHG9_Scaffold_5655	54	p2	(TA)6	12	199334	199345	
PHG9_Scaffold_5655	55	p2	(TC)9	18	201002	201019	potentially variable
PHG9_Scaffold_5655	57	p3	(ATC)5	15	205056	205070	potentially variable
PHG9_Scaffold_5655	60	p2	(AT)7	14	214532	214545	potentially variable
PHG9_Scaffold_5655	61	p3	(AAG)5	15	218171	218185	potentially variable
PHG9_Scaffold_5655	66	p2	(TA)6	12	234294	234305	
PHG9_Scaffold_5655	75	p3	(GAA)6	18	270193	270210	potentially variable
PHG9_Scaffold_5655	77	p2	(AT)6	12	273785	273796	
PHG9_Scaffold_5657	9	p3	(GAA)5	15	33689	33703	potentially variable
PHG9_Scaffold_5657	24	p2	(AT)7	14	69250	69263	potentially variable
PHG9_Scaffold_5658	2	p2	(TA)6	12	3963	3974	
PHG9_Scaffold_5659	1	p3	(ATA)5	15	221	235	potentially

							variable
PHG9_Scaffold_5659	3	p3	(TTC)6	18	1419	1436	potentially variable
PHG9_Scaffold_5659	12	p2	(AT)8	16	18932	18947	potentially variable
PHG9_Scaffold_5659	17	p2	(AT)6	12	23649	23660	
PHG9_Scaffold_5659	40	p3	(AAT)5	15	105938	105952	potentially variable
PHG9_Scaffold_5659	45	p2	(GT)6	12	128997	129008	
PHG9_Scaffold_5659	49	p2	(CA)6	12	148981	148992	
PHG9_Scaffold_5660	3	p2	(TA)8	16	62415	62430	potentially variable
PHG9_Scaffold_5662	1	p3	(TAT)5	15	4677	4691	potentially variable
PHG9_Scaffold_5662	8	p2	(TA)6	12	47662	47673	
PHG9_Scaffold_5662	17	p2	(AT)6	12	92354	92365	
PHG9_Scaffold_5664	3	p2	(AT)9	18	18790	18807	potentially variable
PHG9_Scaffold_5664	10	p2	(TA)7	14	42843	42856	potentially variable
PHG9_Scaffold_5664	19	p2	(AT)6	12	84346	84357	
PHG9_Scaffold_5664	23	p2	(CT)6	12	88789	88800	
PHG9_Scaffold_5665	8	p2	(AT)6	12	50987	50998	
PHG9_Scaffold_5665	10	p2	(AT)7	14	56766	56779	potentially variable
PHG9_Scaffold_5665	16	p2	(AC)7	14	65137	65150	potentially variable
PHG9_Scaffold_5665	19	p2	(TA)7	14	67520	67533	potentially variable

PHG9_Scaffold_5665	22	p3	(AAT)5	15	79819	79833	potentially variable
PHG9_Scaffold_5665	26	p3	(ACC)5	15	90439	90453	potentially variable
PHG9_Scaffold_5665	29	p2	(CT)6	12	102382	102393	
PHG9_Scaffold_5666	2	p2	(TA)6	12	15889	15900	
PHG9_Scaffold_5666	18	p2	(CT)8	16	70326	70341	potentially variable
PHG9_Scaffold_5666	22	p2	(TC)8	16	81206	81221	potentially variable
PHG9_Scaffold_5666	23	p2	(TA)7	14	81611	81624	potentially variable
PHG9_Scaffold_5666	26	p3	(ATT)5	15	107419	107433	potentially variable
PHG9_Scaffold_5666	37	p2	(AT)6	12	167129	167140	
PHG9_Scaffold_5666	54	p2	(GA)6	12	219499	219510	
PHG9_Scaffold_5667	2	p2	(TA)8	16	3417	3432	potentially variable
PHG9_Scaffold_5667	10	p2	(AT)6	12	42921	42932	
PHG9_Scaffold_5667	15	p3	(AAT)5	15	79123	79137	potentially variable
PHG9_Scaffold_5667	22	p2	(AT)9	18	107916	107933	potentially variable
PHG9_Scaffold_5667	25	p3	(ATA)6	18	120340	120357	potentially variable
PHG9_Scaffold_5667	27	p2	(TA)9	18	127304	127321	potentially variable
PHG9_Scaffold_5667	28	p2	(TG)6	12	128471	128482	
PHG9_Scaffold_5667	37	p2	(TA)6	12	154183	154194	

PHG9_Scaffold_5667	40	p2	(AT)7	14	158528	158541	potentially variable
PHG9_Scaffold_5667	41	p3	(ACT)5	15	161108	161122	potentially variable
PHG9_Scaffold_5667	62	p2	(AG)9	18	237009	237026	potentially variable
PHG9_Scaffold_5667	66	p2	(TA)6	12	255237	255248	
PHG9_Scaffold_5667	70	p3	(ATT)6	18	262129	262146	potentially variable
PHG9_Scaffold_5667	72	p3	(ACA)6	18	264924	264941	potentially variable
PHG9_Scaffold_5667	73	p2	(CT)7	14	265326	265339	potentially variable
PHG9_Scaffold_5667	75	p2	(TC)6	12	269705	269716	
PHG9_Scaffold_5667	78	p2	(TA)6	12	282362	282373	
PHG9_Scaffold_5667	79	p2	(TA)7	14	305513	305526	potentially variable
PHG9_Scaffold_5667	82	p2	(AG)8	16	312111	312126	potentially variable
PHG9_Scaffold_5667	84	p3	(AAT)5	15	313484	313498	potentially variable
PHG9_Scaffold_5668	4	p2	(TA)7	14	8161	8174	potentially variable
PHG9_Scaffold_5668	8	p2	(AT)6	12	39006	39017	
PHG9_Scaffold_5668	11	p2	(AT)6	12	50245	50256	
PHG9_Scaffold_5669	7	p2	(TC)6	12	24317	24328	
PHG9_Scaffold_5669	13	p2	(GA)8	16	32212	32227	potentially variable
PHG9_Scaffold_5669	19	p2	(CT)6	12	41607	41618	

PHG9_Scaffold_5669	20	p2	(CT)6	12	41935	41946	
PHG9_Scaffold_5669	23	p3	(AAG)5	15	49097	49111	potentially variable
PHG9_Scaffold_5669	30	p2	(TA)6	12	85785	85796	
PHG9_Scaffold_5669	31	p2	(AT)6	12	86657	86668	
PHG9_Scaffold_5669	32	p2	(TA)6	12	86834	86845	
PHG9_Scaffold_5669	36	p2	(TA)7	14	106038	106051	potentially variable
PHG9_Scaffold_5669	38	p2	(TA)6	12	115337	115348	
PHG9_Scaffold_5669	46	p2	(AT)6	12	138230	138241	
PHG9_Scaffold_5669	51	p3	(AAT)5	15	141813	141827	potentially variable
PHG9_Scaffold_5670	2	p2	(AT)6	12	2781	2792	
PHG9_Scaffold_5670	17	p3	(TAA)5	15	46495	46509	potentially variable
PHG9_Scaffold_5670	20	p2	(AT)7	14	89918	89931	potentially variable
PHG9_Scaffold_5670	27	p2	(AT)9	18	112802	112819	potentially variable
PHG9_Scaffold_5672	18	p2	(AT)6	12	38072	38083	
PHG9_Scaffold_5672	30	p3	(AAT)5	15	71044	71058	potentially variable
PHG9_Scaffold_5672	31	p2	(TA)9	18	76997	77014	potentially variable
PHG9_Scaffold_5672	34	p3	(CCA)5	15	85257	85271	potentially variable
PHG9_Scaffold_5673	1	p2	(TG)7	14	475	488	potentially variable
PHG9_Scaffold_5673	4	p2	(TA)7	14	20754	20767	potentially

							variable
PHG9_Scaffold_5673	7	p2	(TA)9	18	29935	29952	potentially variable
PHG9_Scaffold_5673	40	p2	(TA)6	12	180790	180801	
PHG9_Scaffold_5673	43	p2	(AT)6	12	187150	187161	
PHG9_Scaffold_5673	52	p2	(AT)7	14	227616	227629	potentially variable
PHG9_Scaffold_5673	54	p2	(GA)9	18	262794	262811	potentially variable
PHG9_Scaffold_5674	1	p2	(AT)6	12	1116	1127	
PHG9_Scaffold_5674	2	p3	(ATT)5	15	21903	21917	potentially variable
PHG9_Scaffold_5674	3	p2	(AT)6	12	29413	29424	
PHG9_Scaffold_5674	4	p2	(TA)7	14	35689	35702	potentially variable
PHG9_Scaffold_5674	5	p2	(TA)8	16	38858	38873	potentially variable
PHG9_Scaffold_5674	10	p2	(TA)6	12	48080	48091	
PHG9_Scaffold_5674	12	p2	(TA)9	18	50984	51001	potentially variable
PHG9_Scaffold_5674	16	p3	(ATG)5	15	58543	58557	potentially variable
PHG9_Scaffold_5674	20	p2	(AT)7	14	65201	65214	potentially variable
PHG9_Scaffold_5675	9	p2	(TA)6	12	20134	20145	
PHG9_Scaffold_5675	11	p3	(GAT)5	15	25695	25709	potentially variable
PHG9_Scaffold_5675	13	p2	(TA)6	12	28007	28018	
PHG9_Scaffold_5675	14	p2	(TC)6	12	29482	29493	

PHG9_Scaffold_5675	15	p2	(TA)9	18	30026	30043	potentially variable
PHG9_Scaffold_5675	23	p2	(AG)6	12	53757	53768	
PHG9_Scaffold_5675	38	p2	(TA)6	12	103513	103524	
PHG9_Scaffold_5675	40	p2	(AG)6	12	105001	105012	
PHG9_Scaffold_5675	51	p2	(AG)6	12	139173	139184	
PHG9_Scaffold_5675	52	p2	(GT)6	12	139324	139335	
PHG9_Scaffold_5675	53	p3	(TTC)5	15	144087	144101	potentially variable
PHG9_Scaffold_5675	59	p2	(AT)9	18	166735	166752	potentially variable
PHG9_Scaffold_5675	63	p2	(AG)6	12	172189	172200	
PHG9_Scaffold_5675	66	p2	(AT)8	16	177421	177436	potentially variable
PHG9_Scaffold_5675	74	p3	(ATT)5	15	208861	208875	potentially variable
PHG9_Scaffold_5675	77	p2	(AT)6	12	223986	223997	
PHG9_Scaffold_5675	85	p3	(ATG)5	15	247610	247624	potentially variable
PHG9_Scaffold_5675	96	p3	(TTC)6	18	289354	289371	potentially variable
PHG9_Scaffold_5675	100	p2	(AT)6	12	291851	291862	
PHG9_Scaffold_5675	102	p3	(CTG)5	15	302032	302046	potentially variable
PHG9_Scaffold_5676	20	p2	(AT)9	18	66606	66623	potentially variable
PHG9_Scaffold_5676	22	p2	(CA)6	12	67209	67220	
PHG9_Scaffold_5676	25	p2	(TA)9	18	70669	70686	potentially variable

PHG9_Scaffold_5676	34	p2	(AT)7	14	83949	83962	potentially variable
PHG9_Scaffold_5676	42	p3	(GAA)6	18	94926	94943	potentially variable
PHG9_Scaffold_5676	46	p2	(AT)7	14	104468	104481	potentially variable
PHG9_Scaffold_5676	51	p3	(AAT)6	18	112107	112124	potentially variable
PHG9_Scaffold_5677	5	p2	(TA)7	14	12377	12390	potentially variable
PHG9_Scaffold_5677	7	p2	(AG)6	12	19073	19084	
PHG9_Scaffold_5677	12	p2	(AT)6	12	48719	48730	
PHG9_Scaffold_5677	15	p2	(AT)9	18	55060	55077	potentially variable
PHG9_Scaffold_5677	25	p2	(TA)6	12	95023	95034	
PHG9_Scaffold_5677	27	p2	(TC)7	14	101901	101914	potentially variable
PHG9_Scaffold_5677	28	p2	(CT)6	12	102053	102064	
PHG9_Scaffold_5678	5	p2	(AT)6	12	27751	27762	
PHG9_Scaffold_5678	27	p2	(AT)6	12	94800	94811	
PHG9_Scaffold_5678	36	p2	(AT)6	12	144994	145005	
PHG9_Scaffold_5679	21	p3	(TTC)5	15	50028	50042	potentially variable
PHG9_Scaffold_5679	23	p2	(TC)6	12	62504	62515	
PHG9_Scaffold_5679	28	p3	(TCT)5	15	76776	76790	potentially variable
PHG9_Scaffold_5679	32	p2	(TA)7	14	82569	82582	potentially variable
PHG9_Scaffold_5679	34	p3	(TGT)5	15	91690	91704	potentially

							variable
PHG9_Scaffold_5679	35	p3	(TGT)5	15	92757	92771	potentially variable
PHG9_Scaffold_5679	48	p2	(TA)6	12	136305	136316	
PHG9_Scaffold_5680	5	p3	(TCA)6	18	20474	20491	potentially variable
PHG9_Scaffold_5680	15	p2	(TA)6	12	60959	60970	
PHG9_Scaffold_5680	18	p2	(TA)6	12	95530	95541	
PHG9_Scaffold_5680	22	p2	(AT)7	14	143094	143107	potentially variable
PHG9_Scaffold_5681	1	p2	(AT)9	18	33	50	potentially variable
PHG9_Scaffold_5681	4	p2	(TA)9	18	8558	8575	potentially variable
PHG9_Scaffold_5681	10	p2	(TA)6	12	22477	22488	
PHG9_Scaffold_5681	12	p3	(TTG)5	15	32458	32472	potentially variable
PHG9_Scaffold_5681	18	p3	(GAA)6	18	44948	44965	potentially variable
PHG9_Scaffold_5681	22	p3	(CGG)5	15	50892	50906	potentially variable
PHG9_Scaffold_5681	35	p2	(CT)7	14	92727	92740	potentially variable
PHG9_Scaffold_5681	36	p3	(ATG)5	15	96389	96403	potentially variable
PHG9_Scaffold_5681	48	p3	(TAT)5	15	112956	112970	potentially variable
PHG9_Scaffold_5682	2	p2	(TA)7	14	2175	2188	potentially variable
PHG9_Scaffold_5682	10	p2	(GA)7	14	58981	58994	potentially

							variable
PHG9_Scaffold_5682	17	p2	(TA)6	12	71170	71181	
PHG9_Scaffold_5682	26	p2	(AT)8	16	117742	117757	potentially variable
PHG9_Scaffold_5683	4	p2	(AT)6	12	11713	11724	
PHG9_Scaffold_5684	5	p2	(AT)7	14	19613	19626	potentially variable
PHG9_Scaffold_5684	8	p3	(ACA)5	15	34385	34399	potentially variable
PHG9_Scaffold_5684	10	p2	(AG)7	14	38175	38188	potentially variable
PHG9_Scaffold_5684	12	p3	(TTA)5	15	47521	47535	potentially variable
PHG9_Scaffold_5684	13	p3	(ATG)5	15	51497	51511	potentially variable
PHG9_Scaffold_5684	14	p2	(CA)6	12	51907	51918	
PHG9_Scaffold_5684	21	p2	(AT)6	12	65602	65613	
PHG9_Scaffold_5684	25	p3	(ATA)5	15	72878	72892	potentially variable
PHG9_Scaffold_5684	33	p2	(AG)6	12	85936	85947	
PHG9_Scaffold_5684	34	p2	(CT)9	18	86400	86417	potentially variable
PHG9_Scaffold_5684	35	p2	(AG)9	18	91680	91697	potentially variable
PHG9_Scaffold_5684	38	p2	(TA)6	12	101274	101285	
PHG9_Scaffold_5684	39	p2	(TC)6	12	103402	103413	
PHG9_Scaffold_5684	47	p3	(TTC)5	15	125824	125838	potentially variable
PHG9_Scaffold_5685	5	p3	(ACA)5	15	11380	11394	potentially

							variable
PHG9_Scaffold_5685	6	p3	(GAG)5	15	15084	15098	potentially variable
PHG9_Scaffold_5685	15	p2	(TA)7	14	42990	43003	potentially variable
PHG9_Scaffold_5685	16	p3	(TAT)5	15	43388	43402	potentially variable
PHG9_Scaffold_5685	21	p2	(AT)6	12	53606	53617	
PHG9_Scaffold_5686	13	p2	(TA)8	16	36431	36446	potentially variable
PHG9_Scaffold_5687	3	p2	(AT)7	14	5624	5637	potentially variable
PHG9_Scaffold_5687	7	p2	(AT)7	14	56333	56346	potentially variable
PHG9_Scaffold_5687	9	p3	(TTA)5	15	74376	74390	potentially variable
PHG9_Scaffold_5687	14	p2	(GA)7	14	148680	148693	potentially variable
PHG9_Scaffold_5689	17	p2	(TA)8	16	55627	55642	potentially variable
PHG9_Scaffold_5690	2	p3	(ATA)5	15	4719	4733	potentially variable
PHG9_Scaffold_5690	4	p2	(AT)9	18	13255	13272	potentially variable
PHG9_Scaffold_5690	5	p2	(GA)9	18	14695	14712	potentially variable
PHG9_Scaffold_5690	6	p3	(AAT)6	18	15345	15362	potentially variable
PHG9_Scaffold_5690	14	p2	(TA)7	14	35673	35686	potentially variable

PHG9_Scaffold_5690	16	p2	(AT)7	14	36912	36925	potentially variable
PHG9_Scaffold_5690	20	p2	(TG)6	12	50106	50117	
PHG9_Scaffold_5690	26	p3	(GAT)5	15	75143	75157	potentially variable
PHG9_Scaffold_5691	12	p2	(GT)6	12	29819	29830	
PHG9_Scaffold_5691	24	p2	(CT)7	14	65072	65085	potentially variable
PHG9_Scaffold_5692	14	p3	(GAG)5	15	39304	39318	potentially variable
PHG9_Scaffold_5693	5	p2	(AT)8	16	15602	15617	potentially variable
PHG9_Scaffold_5693	11	p2	(CT)8	16	26268	26283	potentially variable
PHG9_Scaffold_5693	18	p2	(TC)9	18	44780	44797	potentially variable
PHG9_Scaffold_5693	19	p3	(TAA)5	15	50683	50697	potentially variable
PHG9_Scaffold_5693	20	p3	(TAA)5	15	57545	57559	potentially variable
PHG9_Scaffold_5693	24	p3	(TAT)6	18	67932	67949	potentially variable
PHG9_Scaffold_5693	29	p2	(TA)6	12	83985	83996	
PHG9_Scaffold_5693	40	p2	(AC)7	14	101454	101467	potentially variable
PHG9_Scaffold_5693	43	p2	(TA)6	12	110097	110108	
PHG9_Scaffold_5693	45	p3	(AGA)5	15	110764	110778	potentially variable
PHG9_Scaffold_5693	52	p3	(TCT)6	18	143954	143971	potentially variable

PHG9_Scaffold_5693	54	p2	(CT)8	16	148637	148652	potentially variable
PHG9_Scaffold_5693	60	p2	(TA)8	16	171208	171223	potentially variable
PHG9_Scaffold_5693	64	p2	(AT)6	12	177451	177462	
PHG9_Scaffold_5693	68	p2	(AT)8	16	182609	182624	potentially variable
PHG9_Scaffold_5693	70	p2	(AT)9	18	183303	183320	potentially variable
PHG9_Scaffold_5693	72	p3	(GCA)5	15	199283	199297	potentially variable
PHG9_Scaffold_5693	75	p2	(CA)6	12	201497	201508	
PHG9_Scaffold_5693	91	p2	(AT)7	14	257408	257421	potentially variable
PHG9_Scaffold_5693	95	p2	(TC)7	14	265287	265300	potentially variable
PHG9_Scaffold_5693	97	p2	(TA)6	12	271778	271789	
PHG9_Scaffold_5694	4	p2	(TA)6	12	17636	17647	
PHG9_Scaffold_5694	10	p2	(CA)6	12	43074	43085	
PHG9_Scaffold_5694	21	p3	(ATT)5	15	71942	71956	potentially variable
PHG9_Scaffold_5694	26	p2	(TA)6	12	88394	88405	
PHG9_Scaffold_5694	28	p2	(TA)6	12	99036	99047	
PHG9_Scaffold_5695	4	p3	(TGA)5	15	11796	11810	potentially variable
PHG9_Scaffold_5695	8	p2	(TA)6	12	22352	22363	
PHG9_Scaffold_5695	21	p3	(TTA)5	15	57678	57692	potentially variable
PHG9_Scaffold_5695	22	p3	(ATT)5	15	60247	60261	potentially

							variable
PHG9_Scaffold_5695	28	p3	(TAA)6	18	75806	75823	potentially variable
PHG9_Scaffold_5695	32	p2	(AT)6	12	79764	79775	
PHG9_Scaffold_5695	37	p2	(AT)6	12	92964	92975	
PHG9_Scaffold_5695	46	p3	(AGA)5	15	108991	109005	potentially variable
PHG9_Scaffold_5696	10	p2	(AT)6	12	28466	28477	
PHG9_Scaffold_5697	1	p2	(TA)8	16	11541	11556	potentially variable
PHG9_Scaffold_5698	2	p3	(TGG)5	15	3714	3728	potentially variable
PHG9_Scaffold_5698	7	p2	(TC)7	14	29429	29442	potentially variable
PHG9_Scaffold_5698	15	p2	(AG)9	18	70412	70429	potentially variable
PHG9_Scaffold_5698	19	p2	(AT)9	18	95249	95266	potentially variable
PHG9_Scaffold_5699	3	p2	(GA)7	14	9440	9453	potentially variable
PHG9_Scaffold_5699	5	p3	(TAA)5	15	12945	12959	potentially variable
PHG9_Scaffold_5699	8	p2	(AT)8	16	18210	18225	potentially variable
PHG9_Scaffold_5699	12	p3	(TAA)5	15	23150	23164	potentially variable
PHG9_Scaffold_5699	14	p2	(AT)6	12	24658	24669	
PHG9_Scaffold_5699	15	p2	(TA)8	16	27381	27396	potentially variable
PHG9_Scaffold_5700	12	p2	(TA)8	16	36367	36382	potentially

							variable
PHG9_Scaffold_5701	1	p2	(AG)7	14	2725	2738	potentially variable
PHG9_Scaffold_5701	3	p2	(TA)9	18	6037	6054	potentially variable
PHG9_Scaffold_5701	23	p3	(AAT)5	15	39939	39953	potentially variable
PHG9_Scaffold_5701	30	p2	(TA)6	12	56414	56425	
PHG9_Scaffold_5702	2	p3	(TAA)5	15	5843	5857	potentially variable
PHG9_Scaffold_5702	5	p2	(TA)7	14	31974	31987	potentially variable
PHG9_Scaffold_5702	12	p2	(AT)8	16	60765	60780	potentially variable
PHG9_Scaffold_5703	7	p2	(TC)8	16	39237	39252	potentially variable
PHG9_Scaffold_5703	9	p3	(ATA)6	18	49419	49436	potentially variable
PHG9_Scaffold_5703	24	p2	(TC)7	14	89182	89195	potentially variable
PHG9_Scaffold_5703	36	p2	(TA)7	14	124045	124058	potentially variable
PHG9_Scaffold_5704	2	p2	(AT)6	12	2133	2144	
PHG9_Scaffold_5704	12	p3	(TGG)5	15	37386	37400	potentially variable
PHG9_Scaffold_5704	17	p2	(AG)6	12	53631	53642	
PHG9_Scaffold_5705	5	p2	(CA)6	12	13884	13895	
PHG9_Scaffold_5705	6	p2	(TA)6	12	17287	17298	
PHG9_Scaffold_5705	28	p2	(TA)9	18	81518	81535	potentially variable

PHG9_Scaffold_5705	29	p2	(AT)6	12	86455	86466	
PHG9_Scaffold_5705	32	p2	(AT)9	18	93590	93607	potentially variable
PHG9_Scaffold_5706	10	p2	(TA)7	14	32319	32332	potentially variable
PHG9_Scaffold_5706	17	p2	(AT)8	16	41451	41466	potentially variable
PHG9_Scaffold_5706	18	p2	(AT)6	12	41573	41584	
PHG9_Scaffold_5706	19	p2	(TA)6	12	42098	42109	
PHG9_Scaffold_5706	20	p2	(TA)6	12	44675	44686	
PHG9_Scaffold_5706	21	p2	(TA)8	16	46379	46394	potentially variable
PHG9_Scaffold_5707	1	p2	(AT)6	12	2301	2312	
PHG9_Scaffold_5707	6	p2	(AT)7	14	57856	57869	potentially variable
PHG9_Scaffold_5707	7	p2	(TC)6	12	65989	66000	
PHG9_Scaffold_5707	8	p2	(TC)6	12	73178	73189	
PHG9_Scaffold_5707	12	p2	(TA)6	12	210155	210166	
PHG9_Scaffold_5707	13	p2	(AT)6	12	212912	212923	
PHG9_Scaffold_5708	5	p3	(AGC)6	18	23888	23905	potentially variable
PHG9_Scaffold_5708	13	p2	(GT)7	14	52913	52926	potentially variable
PHG9_Scaffold_5708	14	p2	(AG)6	12	53329	53340	
PHG9_Scaffold_5708	20	p2	(TA)7	14	72859	72872	potentially variable
PHG9_Scaffold_5709	5	p3	(AGC)5	15	5130	5144	potentially variable
PHG9_Scaffold_5710	9	p2	(CA)6	12	13050	13061	

PHG9_Scaffold_5710	17	p2	(TC)6	12	97535	97546	
PHG9_Scaffold_5710	18	p2	(TA)6	12	98283	98294	
PHG9_Scaffold_5711	8	p3	(CAC)6	18	12944	12961	potentially variable
PHG9_Scaffold_5711	9	p2	(GT)8	16	14242	14257	potentially variable
PHG9_Scaffold_5711	17	p2	(AT)8	16	38302	38317	potentially variable
PHG9_Scaffold_5711	18	p2	(AT)9	18	40055	40072	potentially variable
PHG9_Scaffold_5711	20	p2	(TC)8	16	48581	48596	potentially variable
PHG9_Scaffold_5711	23	p2	(AT)6	12	71067	71078	
PHG9_Scaffold_5711	24	p2	(AT)9	18	72811	72828	potentially variable
PHG9_Scaffold_5711	29	p3	(GAA)6	18	99189	99206	potentially variable
PHG9_Scaffold_5711	30	p2	(TA)8	16	99754	99769	potentially variable
PHG9_Scaffold_5711	39	p2	(AT)6	12	123332	123343	
PHG9_Scaffold_5711	51	p3	(CTT)5	15	138292	138306	potentially variable
PHG9_Scaffold_5711	58	p2	(CT)6	12	162826	162837	
PHG9_Scaffold_5711	62	p3	(AAT)5	15	177103	177117	potentially variable
PHG9_Scaffold_5711	63	p2	(TA)6	12	177676	177687	
PHG9_Scaffold_5711	66	p2	(TA)7	14	181065	181078	potentially variable
PHG9_Scaffold_5711	67	p2	(AT)9	18	182169	182186	potentially variable

PHG9_Scaffold_5711	77	p2	(AT)6	12	208651	208662	
PHG9_Scaffold_5711	78	p2	(TA)8	16	208803	208818	potentially variable
PHG9_Scaffold_5711	88	p2	(AT)8	16	248399	248414	potentially variable
PHG9_Scaffold_5711	98	p6	(CATCTT)7	42	279896	279937	Hypervariable
PHG9_Scaffold_5711	99	p3	(TGA)5	15	280334	280348	potentially variable
PHG9_Scaffold_5711	102	p2	(AT)6	12	283532	283543	
PHG9_Scaffold_5711	122	p2	(TA)7	14	335246	335259	potentially variable
PHG9_Scaffold_5711	125	p2	(TA)8	16	338180	338195	potentially variable
PHG9_Scaffold_5711	129	p2	(TA)7	14	360988	361001	potentially variable
PHG9_Scaffold_5711	134	p6	(CATCTT)7	42	380601	380642	Hypervariable
PHG9_Scaffold_5711	135	p3	(TGA)5	15	381039	381053	potentially variable
PHG9_Scaffold_5711	152	p2	(TC)6	12	435792	435803	
PHG9_Scaffold_5711	153	p2	(AG)6	12	438136	438147	
PHG9_Scaffold_5711	159	p2	(GA)8	16	459639	459654	potentially variable
PHG9_Scaffold_5711	173	p2	(AT)8	16	492282	492297	potentially variable
PHG9_Scaffold_5711	174	p3	(TCC)5	15	498292	498306	potentially variable
PHG9_Scaffold_5711	180	p3	(ATA)5	15	521878	521892	potentially variable
PHG9_Scaffold_5711	184	p3	(AAT)6	18	528668	528685	potentially variable

PHG9_Scaffold_5711	187	p2	(TA)7	14	537003	537016	potentially variable
PHG9_Scaffold_5711	189	p2	(TA)7	14	544045	544058	potentially variable
PHG9_Scaffold_5711	197	p2	(AT)7	14	571850	571863	potentially variable
PHG9_Scaffold_5711	202	p2	(AG)6	12	576235	576246	
PHG9_Scaffold_5711	211	p2	(TA)9	18	620347	620364	potentially variable
PHG9_Scaffold_5711	213	p3	(CAT)5	15	622047	622061	potentially variable
PHG9_Scaffold_5711	214	p2	(TA)7	14	623303	623316	potentially variable
PHG9_Scaffold_5711	216	p2	(TG)7	14	631374	631387	potentially variable
PHG9_Scaffold_5712	15	p2	(TA)6	12	22851	22862	
PHG9_Scaffold_5712	22	p2	(TA)6	12	42047	42058	
PHG9_Scaffold_5712	25	p2	(AT)7	14	43151	43164	potentially variable
PHG9_Scaffold_5713	1	p2	(TA)8	16	1445	1460	potentially variable
PHG9_Scaffold_5713	20	p2	(TA)8	16	57612	57627	potentially variable
PHG9_Scaffold_5713	21	p2	(TA)7	14	60550	60563	potentially variable
PHG9_Scaffold_5713	23	p2	(TA)9	18	65566	65583	potentially variable
PHG9_Scaffold_5713	26	p2	(TG)7	14	77940	77953	potentially variable
PHG9_Scaffold_5713	27	p2	(AG)8	16	78056	78071	potentially

							variable
PHG9_Scaffold_5713	31	p2	(AT)7	14	95835	95848	potentially variable
PHG9_Scaffold_5713	34	p2	(AT)6	12	102827	102838	
PHG9_Scaffold_5713	35	p2	(TA)7	14	106472	106485	potentially variable
PHG9_Scaffold_5713	50	p3	(CTT)6	18	204028	204045	potentially variable
PHG9_Scaffold_5714	3	p2	(AT)9	18	21596	21613	potentially variable
PHG9_Scaffold_5716	1	p2	(AT)8	16	744	759	potentially variable
PHG9_Scaffold_5716	2	p2	(TC)7	14	3734	3747	potentially variable
PHG9_Scaffold_5716	5	p2	(TA)7	14	8021	8034	potentially variable
PHG9_Scaffold_5716	7	p2	(TA)6	12	19395	19406	
PHG9_Scaffold_5716	8	p2	(TC)6	12	19800	19811	
PHG9_Scaffold_5716	9	p2	(TC)7	14	19985	19998	potentially variable
PHG9_Scaffold_5716	12	p2	(AT)8	16	25957	25972	potentially variable
PHG9_Scaffold_5716	15	p3	(ATA)5	15	42155	42169	potentially variable
PHG9_Scaffold_5716	18	p2	(CA)7	14	48244	48257	potentially variable
PHG9_Scaffold_5718	2	p2	(AT)8	16	3592	3607	potentially variable
PHG9_Scaffold_5718	6	p3	(AAT)5	15	13622	13636	potentially variable

PHG9_Scaffold_5718	13	p2	(AT)9	18	41889	41906	potentially variable
PHG9_Scaffold_5718	20	p3	(CCA)5	15	72957	72971	potentially variable
PHG9_Scaffold_5718	21	p2	(AT)8	16	74255	74270	potentially variable
PHG9_Scaffold_5718	31	p2	(TA)7	14	120420	120433	potentially variable
PHG9_Scaffold_5718	36	p2	(TG)8	16	137854	137869	potentially variable
PHG9_Scaffold_5718	41	p6	(CCAGCA)5	30	165265	165294	Hypervariable
PHG9_Scaffold_5718	43	p2	(GA)6	12	173436	173447	
PHG9_Scaffold_5718	45	p3	(TAT)5	15	175016	175030	potentially variable
PHG9_Scaffold_5718	48	p3	(CAT)5	15	178110	178124	potentially variable
PHG9_Scaffold_5718	52	p3	(ATA)5	15	187896	187910	potentially variable
PHG9_Scaffold_5718	56	p2	(CT)6	12	195509	195520	
PHG9_Scaffold_5718	62	p2	(TA)6	12	209674	209685	
PHG9_Scaffold_5718	63	p2	(TA)6	12	211036	211047	
PHG9_Scaffold_5718	68	p2	(TA)8	16	221857	221872	potentially variable
PHG9_Scaffold_5718	72	p2	(AT)7	14	239129	239142	potentially variable
PHG9_Scaffold_5719	10	p2	(AT)7	14	33023	33036	potentially variable
PHG9_Scaffold_5720	1	p2	(TA)8	16	2723	2738	potentially variable
PHG9_Scaffold_5720	3	p2	(TA)6	12	37549	37560	

PHG9_Scaffold_5720	4	p2	(AT)9	18	38940	38957	potentially variable
PHG9_Scaffold_5721	1	p2	(CT)7	14	317	330	potentially variable
PHG9_Scaffold_5721	10	p2	(TA)7	14	18325	18338	potentially variable
PHG9_Scaffold_5721	15	p3	(AAT)6	18	26187	26204	potentially variable
PHG9_Scaffold_5721	22	p2	(AT)6	12	44827	44838	
PHG9_Scaffold_5722	1	p2	(AG)8	16	344	359	potentially variable
PHG9_Scaffold_5723	2	p2	(AC)6	12	18304	18315	
PHG9_Scaffold_5723	4	p2	(TA)8	16	20623	20638	potentially variable
PHG9_Scaffold_5724	5	p2	(AT)9	18	18448	18465	potentially variable
PHG9_Scaffold_5724	6	p3	(TAA)5	15	21980	21994	potentially variable
PHG9_Scaffold_5724	7	p2	(AT)7	14	26781	26794	potentially variable
PHG9_Scaffold_5724	8	p2	(TA)6	12	27639	27650	
PHG9_Scaffold_5724	9	p2	(AC)7	14	32939	32952	potentially variable
PHG9_Scaffold_5724	10	p3	(AAG)5	15	34778	34792	potentially variable
PHG9_Scaffold_5724	15	p2	(CT)9	18	39786	39803	potentially variable
PHG9_Scaffold_5724	19	p2	(AT)9	18	57476	57493	potentially variable
PHG9_Scaffold_5724	23	p2	(GT)7	14	60658	60671	potentially

							variable
PHG9_Scaffold_5724	31	p2	(AT)6	12	74877	74888	
PHG9_Scaffold_5724	40	p2	(AT)9	18	89662	89679	potentially variable
PHG9_Scaffold_5725	1	p2	(AT)6	12	2750	2761	
PHG9_Scaffold_5725	5	p3	(CTC)5	15	10916	10930	potentially variable
PHG9_Scaffold_5725	6	p2	(CA)8	16	16159	16174	potentially variable
PHG9_Scaffold_5726	4	p2	(TC)7	14	9375	9388	potentially variable
PHG9_Scaffold_5726	11	p2	(AT)6	12	23382	23393	
PHG9_Scaffold_5728	11	p2	(TA)6	12	21145	21156	
PHG9_Scaffold_5730	8	p2	(TA)6	12	16388	16399	
PHG9_Scaffold_5730	11	p2	(GA)6	12	33897	33908	
PHG9_Scaffold_5730	13	p2	(TA)6	12	38884	38895	
PHG9_Scaffold_5730	16	p2	(AT)6	12	53078	53089	
PHG9_Scaffold_5732	6	p2	(AT)7	14	6670	6683	potentially variable
PHG9_Scaffold_5732	7	p3	(GAA)5	15	12314	12328	potentially variable
PHG9_Scaffold_5732	8	p2	(AT)9	18	12795	12812	potentially variable
PHG9_Scaffold_5732	9	p2	(AT)6	12	13593	13604	
PHG9_Scaffold_5732	11	p2	(TA)6	12	28139	28150	
PHG9_Scaffold_5732	14	p2	(TA)6	12	29609	29620	
PHG9_Scaffold_5732	24	p3	(ATA)5	15	54933	54947	potentially variable
PHG9_Scaffold_5732	47	p2	(AT)9	18	119019	119036	potentially

							variable
PHG9_Scaffold_5732	48	p3	(ATT)6	18	119845	119862	potentially variable
PHG9_Scaffold_5732	49	p2	(GA)9	18	125777	125794	potentially variable
PHG9_Scaffold_5732	54	p2	(AT)6	12	140295	140306	
PHG9_Scaffold_5732	60	p2	(TA)6	12	153170	153181	
PHG9_Scaffold_5732	74	p3	(TAA)5	15	180088	180102	potentially variable
PHG9_Scaffold_5733	1	p2	(TC)7	14	440	453	potentially variable
PHG9_Scaffold_5733	3	p2	(TA)6	12	3185	3196	
PHG9_Scaffold_5733	5	p2	(AT)6	12	15828	15839	
PHG9_Scaffold_5734	1	p2	(TA)7	14	4583	4596	potentially variable
PHG9_Scaffold_5734	13	p3	(TAA)5	15	32268	32282	potentially variable
PHG9_Scaffold_5734	17	p2	(AT)6	12	43472	43483	
PHG9_Scaffold_5734	28	p2	(TA)7	14	57649	57662	potentially variable
PHG9_Scaffold_5736	2	p2	(TA)7	14	7948	7961	potentially variable
PHG9_Scaffold_5736	4	p2	(AT)6	12	18268	18279	
PHG9_Scaffold_5736	13	p2	(AC)8	16	74773	74788	potentially variable
PHG9_Scaffold_5736	17	p2	(TA)6	12	84058	84069	
PHG9_Scaffold_5737	3	p3	(AAT)5	15	16867	16881	potentially variable
PHG9_Scaffold_5737	12	p3	(TAA)5	15	52624	52638	potentially

							variable
PHG9_Scaffold_5737	14	p2	(TA)6	12	55832	55843	
PHG9_Scaffold_5737	17	p2	(TA)6	12	70664	70675	
PHG9_Scaffold_5738	1	p2	(AT)7	14	9916	9929	potentially variable
PHG9_Scaffold_5738	2	p3	(ATT)5	15	14925	14939	potentially variable
PHG9_Scaffold_5740	5	p2	(AT)6	12	6741	6752	
PHG9_Scaffold_5740	8	p2	(TA)8	16	8359	8374	potentially variable
PHG9_Scaffold_5740	9	p2	(TC)7	14	9833	9846	potentially variable
PHG9_Scaffold_5740	16	p3	(TTC)5	15	46707	46721	potentially variable
PHG9_Scaffold_5740	20	p2	(AG)6	12	73262	73273	
PHG9_Scaffold_5740	23	p2	(TA)9	18	77812	77829	potentially variable
PHG9_Scaffold_5740	27	p2	(AT)7	14	84708	84721	potentially variable
PHG9_Scaffold_5740	33	p3	(AAT)5	15	97520	97534	potentially variable
PHG9_Scaffold_5740	42	p2	(AT)6	12	111709	111720	
PHG9_Scaffold_5740	49	p3	(TAA)5	15	121333	121347	potentially variable
PHG9_Scaffold_5740	55	p2	(AG)7	14	131949	131962	potentially variable
PHG9_Scaffold_5740	56	p3	(TAT)5	15	132719	132733	potentially variable
PHG9_Scaffold_5740	64	p2	(GA)6	12	162596	162607	

PHG9_Scaffold_5740	71	p2	(TA)8	16	186167	186182	potentially variable
PHG9_Scaffold_5740	73	p2	(AT)7	14	186645	186658	potentially variable
PHG9_Scaffold_5740	77	p3	(TAT)5	15	200574	200588	potentially variable
PHG9_Scaffold_5740	80	p2	(GA)9	18	204961	204978	potentially variable
PHG9_Scaffold_5740	83	p3	(GCA)5	15	220331	220345	potentially variable
PHG9_Scaffold_5740	84	p2	(AT)7	14	223298	223311	potentially variable
PHG9_Scaffold_5740	88	p2	(AG)6	12	246372	246383	
PHG9_Scaffold_5740	90	p2	(AT)6	12	255123	255134	
PHG9_Scaffold_5740	93	p2	(GA)6	12	263996	264007	
PHG9_Scaffold_5740	94	p2	(TA)6	12	265284	265295	
PHG9_Scaffold_5741	2	p2	(AT)6	12	30007	30018	
PHG9_Scaffold_5742	4	p3	(TAA)5	15	16700	16714	potentially variable
PHG9_Scaffold_5742	8	p2	(AT)9	18	25195	25212	potentially variable
PHG9_Scaffold_5742	16	p2	(TA)8	16	64187	64202	potentially variable
PHG9_Scaffold_5742	20	p2	(TA)7	14	67134	67147	potentially variable
PHG9_Scaffold_5742	22	p3	(AAT)5	15	70303	70317	potentially variable
PHG9_Scaffold_5742	23	p3	(CAC)5	15	70884	70898	potentially variable
PHG9_Scaffold_5743	3	p2	(AG)9	18	4804	4821	potentially

							variable
PHG9_Scaffold_5743	7	p2	(AG)6	12	6609	6620	
PHG9_Scaffold_5743	8	p3	(ATT)6	18	8881	8898	potentially variable
PHG9_Scaffold_5743	9	p2	(AC)7	14	31638	31651	potentially variable
PHG9_Scaffold_5743	10	p2	(AT)7	14	32630	32643	potentially variable
PHG9_Scaffold_5743	11	p3	(TTG)5	15	36179	36193	potentially variable
PHG9_Scaffold_5743	13	p3	(CAT)5	15	37496	37510	potentially variable
PHG9_Scaffold_5743	29	p2	(GA)8	16	86885	86900	potentially variable
PHG9_Scaffold_5743	30	p3	(ATA)5	15	88846	88860	potentially variable
PHG9_Scaffold_5743	32	p2	(AT)6	12	90811	90822	
PHG9_Scaffold_5743	33	p2	(AG)8	16	90992	91007	potentially variable
PHG9_Scaffold_5743	34	p2	(TA)6	12	93749	93760	
PHG9_Scaffold_5743	39	p2	(CT)7	14	112994	113007	potentially variable
PHG9_Scaffold_5743	43	p2	(AT)6	12	123901	123912	
PHG9_Scaffold_5743	45	p3	(TAA)5	15	129448	129462	potentially variable
PHG9_Scaffold_5743	53	p3	(TAA)5	15	141496	141510	potentially variable
PHG9_Scaffold_5744	16	p2	(TA)9	18	99306	99323	potentially variable
PHG9_Scaffold_5745	1	p2	(TA)6	12	10448	10459	

PHG9_Scaffold_5745	2	p2	(AT)7	14	14234	14247	potentially variable
PHG9_Scaffold_5745	7	p2	(TA)6	12	32119	32130	
PHG9_Scaffold_5746	8	p2	(TA)9	18	38295	38312	potentially variable
PHG9_Scaffold_5746	10	p2	(CA)8	16	47587	47602	potentially variable
PHG9_Scaffold_5746	12	p2	(AT)6	12	72026	72037	
PHG9_Scaffold_5747	7	p2	(TG)8	16	16164	16179	potentially variable
PHG9_Scaffold_5747	10	p2	(TA)7	14	20834	20847	potentially variable
PHG9_Scaffold_5747	35	p2	(CA)6	12	95508	95519	
PHG9_Scaffold_5747	36	p3	(ATA)5	15	98152	98166	potentially variable
PHG9_Scaffold_5747	37	p2	(TA)6	12	99775	99786	
PHG9_Scaffold_5747	38	p2	(CT)6	12	104694	104705	
PHG9_Scaffold_5747	49	p2	(TA)8	16	130201	130216	potentially variable
PHG9_Scaffold_5747	54	p2	(TA)6	12	147931	147942	
PHG9_Scaffold_5747	55	p3	(AAT)5	15	149237	149251	potentially variable
PHG9_Scaffold_5747	57	p2	(AT)8	16	154694	154709	potentially variable
PHG9_Scaffold_5747	60	p2	(TA)9	18	175005	175022	potentially variable
PHG9_Scaffold_5747	62	p2	(AT)7	14	175730	175743	potentially variable
PHG9_Scaffold_5747	63	p2	(TA)8	16	176259	176274	potentially variable

PHG9_Scaffold_5747	66	p2	(GA)6	12	182394	182405	
PHG9_Scaffold_5747	67	p2	(TC)9	18	182938	182955	potentially variable
PHG9_Scaffold_5747	71	p2	(AT)9	18	187040	187057	potentially variable
PHG9_Scaffold_5747	72	p2	(GT)6	12	188381	188392	
PHG9_Scaffold_5747	73	p3	(AAT)5	15	189108	189122	potentially variable
PHG9_Scaffold_5747	76	p2	(TA)6	12	207488	207499	
PHG9_Scaffold_5747	79	p3	(TAA)6	18	215760	215777	potentially variable
PHG9_Scaffold_5747	80	p2	(TA)6	12	215911	215922	
PHG9_Scaffold_5747	94	p2	(AT)6	12	265607	265618	
PHG9_Scaffold_5747	96	p2	(AT)6	12	268595	268606	
PHG9_Scaffold_5747	100	p3	(AGA)5	15	284353	284367	potentially variable
PHG9_Scaffold_5747	101	p3	(CTC)5	15	288145	288159	potentially variable
PHG9_Scaffold_5748	3	p2	(TA)6	12	18579	18590	
PHG9_Scaffold_5748	11	p2	(AT)7	14	36360	36373	potentially variable
PHG9_Scaffold_5748	17	p2	(TC)8	16	63610	63625	potentially variable
PHG9_Scaffold_5751	2	p2	(TA)8	16	25552	25567	potentially variable
PHG9_Scaffold_5751	8	p3	(TAT)5	15	85243	85257	potentially variable
PHG9_Scaffold_5751	13	p2	(TA)7	14	168387	168400	potentially variable

PHG9_Scaffold_5751	16	p3	(AAG)5	15	171901	171915	potentially variable
PHG9_Scaffold_5751	19	p3	(CAC)5	15	181481	181495	potentially variable
PHG9_Scaffold_5751	20	p3	(CAT)6	18	181700	181717	potentially variable
PHG9_Scaffold_5753	4	p2	(TA)8	16	13224	13239	potentially variable
PHG9_Scaffold_5753	5	p2	(AT)8	16	13690	13705	potentially variable
PHG9_Scaffold_5753	6	p2	(AT)9	18	27864	27881	potentially variable
PHG9_Scaffold_5753	10	p2	(TA)7	14	34470	34483	potentially variable
PHG9_Scaffold_5753	14	p2	(TA)6	12	48632	48643	
PHG9_Scaffold_5753	16	p3	(TAA)5	15	52190	52204	potentially variable
PHG9_Scaffold_5753	23	p3	(TTC)5	15	109035	109049	potentially variable
PHG9_Scaffold_5753	30	p2	(GA)6	12	126698	126709	
PHG9_Scaffold_5753	31	p2	(AT)6	12	128026	128037	
PHG9_Scaffold_5753	41	p2	(AT)6	12	158602	158613	
PHG9_Scaffold_5753	42	p2	(AT)7	14	162019	162032	potentially variable
PHG9_Scaffold_5753	46	p3	(TTC)6	18	184006	184023	potentially variable
PHG9_Scaffold_5753	47	p2	(AT)7	14	191709	191722	potentially variable
PHG9_Scaffold_5754	3	p3	(TTA)5	15	16496	16510	potentially variable

PHG9_Scaffold_5754	10	p3	(CAT)5	15	72588	72602	potentially variable
PHG9_Scaffold_5756	4	p2	(AT)8	16	6283	6298	potentially variable
PHG9_Scaffold_5756	5	p2	(AT)7	14	6437	6450	potentially variable
PHG9_Scaffold_5756	13	p2	(TA)6	12	28613	28624	
PHG9_Scaffold_5756	26	p3	(ATC)5	15	86260	86274	potentially variable
PHG9_Scaffold_5756	29	p2	(GT)6	12	91580	91591	
PHG9_Scaffold_5756	34	p3	(AAT)5	15	108902	108916	potentially variable
PHG9_Scaffold_5756	45	p2	(AT)8	16	126701	126716	potentially variable
PHG9_Scaffold_5756	57	p3	(AAT)6	18	155767	155784	potentially variable
PHG9_Scaffold_5756	65	p3	(ATC)5	15	161393	161407	potentially variable
PHG9_Scaffold_5757	3	p2	(TC)6	12	24808	24819	
PHG9_Scaffold_5758	6	p2	(AT)7	14	16590	16603	potentially variable
PHG9_Scaffold_5758	13	p2	(TG)6	12	32309	32320	
PHG9_Scaffold_5758	29	p2	(TG)7	14	86722	86735	potentially variable
PHG9_Scaffold_5758	35	p2	(TC)6	12	100334	100345	
PHG9_Scaffold_5758	36	p2	(AG)8	16	101721	101736	potentially variable
PHG9_Scaffold_5758	38	p3	(CTC)5	15	104338	104352	potentially variable
PHG9_Scaffold_5759	5	p2	(TA)6	12	62708	62719	

PHG9_Scaffold_5759	8	p2	(TG)6	12	69868	69879	
PHG9_Scaffold_5759	12	p2	(TA)8	16	99451	99466	potentially variable
PHG9_Scaffold_5759	13	p2	(AT)7	14	99684	99697	potentially variable
PHG9_Scaffold_5759	23	p2	(TA)6	12	186308	186319	
PHG9_Scaffold_5759	25	p2	(TA)6	12	195405	195416	
PHG9_Scaffold_5760	5	p2	(AT)6	12	17351	17362	
PHG9_Scaffold_5761	7	p2	(TA)6	12	24788	24799	
PHG9_Scaffold_5761	8	p2	(TG)8	16	27081	27096	potentially variable
PHG9_Scaffold_5761	9	p3	(ATT)5	15	33271	33285	potentially variable
PHG9_Scaffold_5761	13	p3	(TGC)5	15	48281	48295	potentially variable
PHG9_Scaffold_5761	14	p2	(TA)9	18	49898	49915	potentially variable
PHG9_Scaffold_5761	21	p2	(AT)7	14	66496	66509	potentially variable
PHG9_Scaffold_5761	23	p3	(TGT)5	15	68615	68629	potentially variable
PHG9_Scaffold_5761	34	p3	(TCT)6	18	105142	105159	potentially variable
PHG9_Scaffold_5761	39	p2	(AT)6	12	125849	125860	
PHG9_Scaffold_5761	47	p3	(AAT)6	18	153758	153775	potentially variable
PHG9_Scaffold_5761	58	p3	(TTA)5	15	189815	189829	potentially variable
PHG9_Scaffold_5761	59	p2	(AG)6	12	190241	190252	

PHG9_Scaffold_5761	67	p2	(AT)7	14	203949	203962	potentially variable
PHG9_Scaffold_5761	78	p2	(AT)6	12	247416	247427	
PHG9_Scaffold_5762	12	p2	(GT)8	16	35065	35080	potentially variable
PHG9_Scaffold_5762	20	p2	(TA)8	16	64837	64852	potentially variable
PHG9_Scaffold_5762	22	p2	(GA)6	12	65346	65357	
PHG9_Scaffold_5762	27	p2	(TA)6	12	71341	71352	
PHG9_Scaffold_5763	5	p2	(TC)6	12	21996	22007	
PHG9_Scaffold_5763	9	p3	(TTA)6	18	30775	30792	potentially variable
PHG9_Scaffold_5763	12	p2	(AT)7	14	51822	51835	potentially variable
PHG9_Scaffold_5763	14	p3	(TTA)6	18	60282	60299	potentially variable
PHG9_Scaffold_5763	19	p2	(TG)7	14	96380	96393	potentially variable
PHG9_Scaffold_5763	23	p3	(ATT)5	15	107663	107677	potentially variable
PHG9_Scaffold_5763	25	p2	(TA)9	18	112435	112452	potentially variable
PHG9_Scaffold_5763	26	p3	(TAA)5	15	136175	136189	potentially variable
PHG9_Scaffold_5763	28	p2	(TA)8	16	139920	139935	potentially variable
PHG9_Scaffold_5763	29	p6	(TTGGTG)5	30	147393	147422	Hypervariable
PHG9_Scaffold_5763	31	p2	(TA)8	16	159573	159588	potentially variable
PHG9_Scaffold_5763	38	p2	(AT)8	16	169278	169293	potentially

							variable
PHG9_Scaffold_5763	42	p2	(TA)9	18	173956	173973	potentially variable
PHG9_Scaffold_5763	46	p2	(AT)6	12	186211	186222	
PHG9_Scaffold_5763	53	p2	(TA)6	12	202560	202571	
PHG9_Scaffold_5763	54	p2	(TA)7	14	203882	203895	potentially variable
PHG9_Scaffold_5763	59	p3	(TAT)5	15	210175	210189	potentially variable
PHG9_Scaffold_5763	62	p2	(CA)9	18	211237	211254	potentially variable
PHG9_Scaffold_5763	63	p2	(AG)6	12	212201	212212	
PHG9_Scaffold_5763	65	p3	(ACC)5	15	225690	225704	potentially variable
PHG9_Scaffold_5763	66	p3	(ATA)5	15	227491	227505	potentially variable
PHG9_Scaffold_5763	71	p2	(TA)7	14	233667	233680	potentially variable
PHG9_Scaffold_5763	80	p3	(AGA)5	15	269820	269834	potentially variable
PHG9_Scaffold_5763	82	p3	(ATA)5	15	272461	272475	potentially variable
PHG9_Scaffold_5763	84	p3	(TAT)5	15	273533	273547	potentially variable
PHG9_Scaffold_5764	4	p2	(AT)7	14	72896	72909	potentially variable
PHG9_Scaffold_5765	5	p2	(TC)6	12	31683	31694	
PHG9_Scaffold_5765	9	p3	(AAT)5	15	39490	39504	potentially variable
PHG9_Scaffold_5765	12	p2	(TA)8	16	44555	44570	potentially

							variable
PHG9_Scaffold_5765	15	p2	(AT)6	12	57350	57361	
PHG9_Scaffold_5765	19	p2	(AT)8	16	101882	101897	potentially variable
PHG9_Scaffold_5765	28	p3	(TTA)5	15	146196	146210	potentially variable
PHG9_Scaffold_5765	29	p2	(AT)8	16	147177	147192	potentially variable
PHG9_Scaffold_5765	39	p2	(TC)6	12	166355	166366	
PHG9_Scaffold_5766	5	p3	(TTA)6	18	10832	10849	potentially variable
PHG9_Scaffold_5767	6	p2	(AT)6	12	6603	6614	
PHG9_Scaffold_5767	8	p2	(TA)8	16	12501	12516	potentially variable
PHG9_Scaffold_5767	10	p3	(TTC)5	15	17896	17910	potentially variable
PHG9_Scaffold_5767	15	p2	(AC)6	12	31980	31991	
PHG9_Scaffold_5767	23	p2	(TA)6	12	45664	45675	
PHG9_Scaffold_5767	28	p2	(TG)6	12	51054	51065	
PHG9_Scaffold_5767	29	p3	(TGA)5	15	52819	52833	potentially variable
PHG9_Scaffold_5767	32	p3	(TAT)5	15	56929	56943	potentially variable
PHG9_Scaffold_5767	37	p2	(TC)6	12	65587	65598	
PHG9_Scaffold_5767	38	p2	(AT)6	12	65883	65894	
PHG9_Scaffold_5767	43	p2	(AT)7	14	77882	77895	potentially variable
PHG9_Scaffold_5767	44	p2	(AC)6	12	78342	78353	
PHG9_Scaffold_5767	45	p3	(ATA)5	15	79983	79997	potentially

							variable
PHG9_Scaffold_5767	46	p2	(AT)9	18	80719	80736	potentially variable
PHG9_Scaffold_5767	59	p2	(AT)8	16	120091	120106	potentially variable
PHG9_Scaffold_5767	62	p2	(AT)7	14	122539	122552	potentially variable
PHG9_Scaffold_5767	65	p2	(AT)9	18	128048	128065	potentially variable
PHG9_Scaffold_5767	73	p2	(AT)6	12	140408	140419	
PHG9_Scaffold_5767	78	p2	(TA)6	12	149733	149744	
PHG9_Scaffold_5767	84	p2	(CT)7	14	159270	159283	potentially variable
PHG9_Scaffold_5767	98	p3	(TTA)5	15	199780	199794	potentially variable
PHG9_Scaffold_5768	2	p3	(TAT)5	15	5781	5795	potentially variable
PHG9_Scaffold_5768	7	p2	(TA)6	12	28022	28033	
PHG9_Scaffold_5768	15	p2	(TC)6	12	58124	58135	
PHG9_Scaffold_5768	20	p2	(AT)7	14	73609	73622	potentially variable
PHG9_Scaffold_5768	22	p2	(AT)6	12	76142	76153	
PHG9_Scaffold_5769	18	p2	(CA)6	12	43817	43828	
PHG9_Scaffold_5769	21	p2	(GT)6	12	50433	50444	
PHG9_Scaffold_5769	24	p2	(CA)7	14	63061	63074	potentially variable
PHG9_Scaffold_5769	38	p3	(AAT)5	15	116531	116545	potentially variable
PHG9_Scaffold_5769	40	p2	(AT)6	12	124401	124412	

PHG9_Scaffold_5769	44	p2	(AG)6	12	132833	132844	
PHG9_Scaffold_5769	60	p2	(AT)9	18	200095	200112	potentially variable
PHG9_Scaffold_5769	72	p2	(TA)8	16	234780	234795	potentially variable
PHG9_Scaffold_5769	76	p3	(TAA)5	15	239893	239907	potentially variable
PHG9_Scaffold_5769	77	p2	(TA)9	18	240368	240385	potentially variable
PHG9_Scaffold_5770	3	p2	(AT)7	14	9247	9260	potentially variable
PHG9_Scaffold_5770	6	p2	(TA)6	12	12240	12251	
PHG9_Scaffold_5772	24	p2	(AT)9	18	63581	63598	potentially variable
PHG9_Scaffold_5772	33	p2	(AT)6	12	77080	77091	
PHG9_Scaffold_5772	45	p2	(TA)8	16	147945	147960	potentially variable
PHG9_Scaffold_5772	49	p3	(AAC)5	15	165199	165213	potentially variable
PHG9_Scaffold_5772	50	p3	(ATG)5	15	166556	166570	potentially variable
PHG9_Scaffold_5772	55	p2	(TA)7	14	182595	182608	potentially variable
PHG9_Scaffold_5773	2	p2	(GA)8	16	7298	7313	potentially variable
PHG9_Scaffold_5773	5	p2	(TA)6	12	16767	16778	
PHG9_Scaffold_5773	9	p2	(AT)8	16	27736	27751	potentially variable
PHG9_Scaffold_5773	12	p3	(ATA)5	15	43117	43131	potentially variable

PHG9_Scaffold_5773	13	p3	(TCT)5	15	43612	43626	potentially variable
PHG9_Scaffold_5773	23	p2	(AT)8	16	89707	89722	potentially variable
PHG9_Scaffold_5773	47	p3	(CTT)5	15	140925	140939	potentially variable
PHG9_Scaffold_5773	53	p2	(TA)9	18	172446	172463	potentially variable
PHG9_Scaffold_5773	55	p3	(TAA)5	15	176460	176474	potentially variable
PHG9_Scaffold_5773	56	p3	(ATA)5	15	176735	176749	potentially variable
PHG9_Scaffold_5773	57	p2	(TA)9	18	178926	178943	potentially variable
PHG9_Scaffold_5773	70	p2	(GA)8	16	227435	227450	potentially variable
PHG9_Scaffold_5773	71	p2	(AT)8	16	228187	228202	potentially variable
PHG9_Scaffold_5775	7	p2	(GA)6	12	56246	56257	
PHG9_Scaffold_5775	15	p2	(TC)7	14	66787	66800	potentially variable
PHG9_Scaffold_5775	22	p2	(TA)8	16	80954	80969	potentially variable
PHG9_Scaffold_5775	32	p3	(GAA)5	15	108503	108517	potentially variable
PHG9_Scaffold_5776	3	p2	(AT)6	12	52881	52892	
PHG9_Scaffold_5776	4	p2	(TA)8	16	87042	87057	potentially variable
PHG9_Scaffold_5776	22	p2	(TG)6	12	241805	241816	
PHG9_Scaffold_5776	29	p2	(TA)6	12	284890	284901	

PHG9_Scaffold_5776	32	p2	(TA)6	12	386701	386712	
PHG9_Scaffold_5776	40	p3	(CGA)6	18	466995	467012	potentially variable
PHG9_Scaffold_5777	8	p2	(AT)7	14	68772	68785	potentially variable
PHG9_Scaffold_5777	9	p2	(AT)7	14	71531	71544	potentially variable
PHG9_Scaffold_5777	15	p2	(AT)6	12	76381	76392	
PHG9_Scaffold_5777	17	p2	(AT)10	20	81109	81128	Hypervariable
PHG9_Scaffold_5777	27	p2	(GT)6	12	102696	102707	
PHG9_Scaffold_5777	28	p3	(TAA)5	15	103672	103686	potentially variable
PHG9_Scaffold_5777	37	p2	(AT)6	12	113382	113393	
PHG9_Scaffold_5777	48	p2	(CA)6	12	156060	156071	
PHG9_Scaffold_5777	51	p2	(TA)6	12	157952	157963	
PHG9_Scaffold_5778	1	p2	(AT)6	12	5934	5945	
PHG9_Scaffold_5778	4	p2	(GA)7	14	30404	30417	potentially variable
PHG9_Scaffold_5778	6	p3	(AAT)5	15	50043	50057	potentially variable
PHG9_Scaffold_5778	9	p3	(TGT)5	15	67802	67816	potentially variable
PHG9_Scaffold_5778	12	p2	(TA)8	16	71547	71562	potentially variable
PHG9_Scaffold_5778	18	p2	(TA)9	18	91377	91394	potentially variable
PHG9_Scaffold_5778	19	p3	(AAT)5	15	106812	106826	potentially variable
PHG9_Scaffold_5778	20	p3	(AAT)6	18	109885	109902	potentially

							variable
PHG9_Scaffold_5778	21	p2	(TA)6	12	122046	122057	
PHG9_Scaffold_5778	25	p3	(TAT)5	15	162081	162095	potentially variable
PHG9_Scaffold_5778	29	p2	(CA)8	16	176926	176941	potentially variable
PHG9_Scaffold_5778	35	p2	(AT)7	14	237079	237092	potentially variable
PHG9_Scaffold_5778	44	p2	(AT)8	16	302301	302316	potentially variable
PHG9_Scaffold_5778	45	p2	(TA)6	12	303248	303259	
PHG9_Scaffold_5778	50	p3	(ATG)5	15	341362	341376	potentially variable
PHG9_Scaffold_5778	52	p2	(AT)7	14	342183	342196	potentially variable
PHG9_Scaffold_5778	54	p2	(TA)7	14	349648	349661	potentially variable
PHG9_Scaffold_5779	6	p2	(CT)6	12	19431	19442	
PHG9_Scaffold_5779	8	p2	(AT)7	14	26963	26976	potentially variable
PHG9_Scaffold_5779	11	p6	(TTATCA)9	54	46388	46441	Hypervariable
PHG9_Scaffold_5780	3	p2	(TC)6	12	25269	25280	
PHG9_Scaffold_5780	4	p2	(TC)9	18	25702	25719	potentially variable
PHG9_Scaffold_5781	9	p2	(TA)9	18	27553	27570	potentially variable
PHG9_Scaffold_5781	12	p2	(TA)6	12	36008	36019	
PHG9_Scaffold_5781	14	p2	(AT)9	18	37955	37972	potentially variable

PHG9_Scaffold_5781	15	p2	(GA)7	14	38885	38898	potentially variable
PHG9_Scaffold_5781	28	p3	(TAA)5	15	80777	80791	potentially variable
PHG9_Scaffold_5781	29	p2	(TA)8	16	81523	81538	potentially variable
PHG9_Scaffold_5781	35	p2	(AT)6	12	95302	95313	
PHG9_Scaffold_5781	41	p2	(AG)8	16	109894	109909	potentially variable
PHG9_Scaffold_5781	44	p2	(TA)9	18	124394	124411	potentially variable
PHG9_Scaffold_5781	48	p2	(TA)9	18	127519	127536	potentially variable
PHG9_Scaffold_5781	49	p3	(AAT)5	15	128044	128058	potentially variable
PHG9_Scaffold_5781	50	p2	(TC)7	14	128875	128888	potentially variable
PHG9_Scaffold_5781	51	p2	(CA)6	12	129594	129605	
PHG9_Scaffold_5781	52	p2	(GA)7	14	130839	130852	potentially variable
PHG9_Scaffold_5781	63	p2	(TC)6	12	146044	146055	
PHG9_Scaffold_5781	71	p2	(GA)9	18	170984	171001	potentially variable
PHG9_Scaffold_5781	73	p2	(AT)6	12	174161	174172	
PHG9_Scaffold_5781	82	p2	(CT)8	16	210592	210607	potentially variable
PHG9_Scaffold_5781	83	p2	(AC)8	16	212748	212763	potentially variable
PHG9_Scaffold_5781	91	p2	(TA)7	14	227155	227168	potentially variable

PHG9_Scaffold_5781	96	p2	(TA)9	18	253364	253381	potentially variable
PHG9_Scaffold_5781	97	p2	(TA)7	14	253558	253571	potentially variable
PHG9_Scaffold_5781	106	p2	(AT)7	14	290414	290427	potentially variable
PHG9_Scaffold_5781	111	p2	(AT)6	12	297022	297033	
PHG9_Scaffold_5781	112	p2	(AT)7	14	297724	297737	potentially variable
PHG9_Scaffold_5781	119	p2	(AC)8	16	307869	307884	potentially variable
PHG9_Scaffold_5781	125	p2	(AG)6	12	318384	318395	
PHG9_Scaffold_5782	3	p3	(AAT)5	15	6708	6722	potentially variable
PHG9_Scaffold_5782	6	p3	(GTT)5	15	18936	18950	potentially variable
PHG9_Scaffold_5782	9	p2	(AT)7	14	29146	29159	potentially variable
PHG9_Scaffold_5782	10	p3	(AAG)5	15	33672	33686	potentially variable
PHG9_Scaffold_5782	17	p2	(CT)9	18	71808	71825	potentially variable
PHG9_Scaffold_5782	18	p3	(AAT)6	18	73336	73353	potentially variable
PHG9_Scaffold_5783	21	p3	(AAT)5	15	92493	92507	potentially variable
PHG9_Scaffold_5783	25	p2	(TA)8	16	124304	124319	potentially variable
PHG9_Scaffold_5784	7	p2	(CA)6	12	20897	20908	
PHG9_Scaffold_5784	10	p2	(TA)6	12	27708	27719	

PHG9_Scaffold_5784	15	p3	(TAT)5	15	40911	40925	potentially variable
PHG9_Scaffold_5785	12	p2	(TA)7	14	33280	33293	potentially variable
PHG9_Scaffold_5785	27	p3	(TGA)6	18	96446	96463	potentially variable
PHG9_Scaffold_5785	28	p2	(CA)6	12	99425	99436	
PHG9_Scaffold_5785	31	p3	(CAA)6	18	109079	109096	potentially variable
PHG9_Scaffold_5785	38	p2	(TA)6	12	145381	145392	
PHG9_Scaffold_5785	40	p2	(TC)6	12	150126	150137	
PHG9_Scaffold_5785	47	p3	(TAT)5	15	164769	164783	potentially variable
PHG9_Scaffold_5785	53	p3	(TAT)5	15	177579	177593	potentially variable
PHG9_Scaffold_5785	60	p3	(GAA)5	15	189250	189264	potentially variable
PHG9_Scaffold_5785	76	p3	(GTG)5	15	253638	253652	potentially variable
PHG9_Scaffold_5785	77	p3	(GAA)5	15	259055	259069	potentially variable
PHG9_Scaffold_5785	79	p2	(TA)7	14	265510	265523	potentially variable
PHG9_Scaffold_5786	1	p2	(TA)6	12	3519	3530	
PHG9_Scaffold_5786	3	p3	(ATA)5	15	5592	5606	potentially variable
PHG9_Scaffold_5786	7	p3	(AAT)5	15	9920	9934	potentially variable
PHG9_Scaffold_5786	11	p2	(TA)7	14	16216	16229	potentially variable

PHG9_Scaffold_5786	12	p2	(TC)6	12	16694	16705	
PHG9_Scaffold_5786	14	p2	(TA)8	16	26698	26713	potentially variable
PHG9_Scaffold_5786	36	p3	(TTC)6	18	72074	72091	potentially variable
PHG9_Scaffold_5786	40	p2	(TA)9	18	76212	76229	potentially variable
PHG9_Scaffold_5786	47	p3	(AAT)5	15	90464	90478	potentially variable
PHG9_Scaffold_5787	6	p3	(TAA)5	15	8426	8440	potentially variable
PHG9_Scaffold_5787	10	p2	(AT)7	14	14734	14747	potentially variable
PHG9_Scaffold_5787	12	p3	(TAA)5	15	23697	23711	potentially variable
PHG9_Scaffold_5787	13	p2	(TA)6	12	28164	28175	
PHG9_Scaffold_5787	22	p2	(TC)7	14	37943	37956	potentially variable
PHG9_Scaffold_5787	24	p2	(TA)9	18	44517	44534	potentially variable
PHG9_Scaffold_5788	2	p3	(GAA)5	15	4159	4173	potentially variable
PHG9_Scaffold_5788	3	p2	(AT)6	12	5396	5407	
PHG9_Scaffold_5788	21	p2	(AT)6	12	40440	40451	
PHG9_Scaffold_5788	22	p3	(TCT)5	15	42165	42179	potentially variable
PHG9_Scaffold_5788	35	p2	(TA)8	16	70364	70379	potentially variable
PHG9_Scaffold_5788	37	p3	(TGT)5	15	77635	77649	potentially variable

PHG9_Scaffold_5788	42	p3	(GCA)5	15	87969	87983	potentially variable
PHG9_Scaffold_5788	52	p2	(AT)6	12	112137	112148	
PHG9_Scaffold_5788	65	p2	(AT)9	18	156204	156221	potentially variable
PHG9_Scaffold_5788	71	p2	(TC)6	12	176191	176202	
PHG9_Scaffold_5788	72	p3	(AAT)6	18	176310	176327	potentially variable
PHG9_Scaffold_5788	80	p2	(TC)6	12	190460	190471	
PHG9_Scaffold_5788	81	p3	(ATA)5	15	190761	190775	potentially variable
PHG9_Scaffold_5788	90	p3	(AAT)5	15	207557	207571	potentially variable
PHG9_Scaffold_5789	3	p2	(TA)7	14	6704	6717	potentially variable
PHG9_Scaffold_5790	1	p3	(CTT)6	18	275	292	potentially variable
PHG9_Scaffold_5790	7	p2	(AT)6	12	15624	15635	
PHG9_Scaffold_5790	9	p2	(AG)6	12	20486	20497	
PHG9_Scaffold_5790	16	p2	(AT)7	14	34516	34529	potentially variable
PHG9_Scaffold_5790	23	p2	(TA)6	12	52744	52755	
PHG9_Scaffold_5790	27	p2	(AT)9	18	68740	68757	potentially variable
PHG9_Scaffold_5790	28	p3	(TAT)6	18	68959	68976	potentially variable
PHG9_Scaffold_5790	30	p2	(AT)9	18	80478	80495	potentially variable
PHG9_Scaffold_5790	48	p3	(ATG)5	15	167358	167372	potentially variable

PHG9_Scaffold_5790	53	p2	(CA)7	14	193086	193099	potentially variable
PHG9_Scaffold_5791	2	p2	(TA)8	16	3734	3749	potentially variable
PHG9_Scaffold_5791	5	p2	(AG)7	14	10884	10897	potentially variable
PHG9_Scaffold_5791	14	p3	(GGT)6	18	79224	79241	potentially variable
PHG9_Scaffold_5791	19	p2	(CT)6	12	90526	90537	
PHG9_Scaffold_5791	25	p2	(AT)7	14	111190	111203	potentially variable
PHG9_Scaffold_5791	26	p3	(TTC)6	18	111869	111886	potentially variable
PHG9_Scaffold_5791	28	p2	(AT)6	12	122957	122968	
PHG9_Scaffold_5791	29	p2	(AT)8	16	123602	123617	potentially variable
PHG9_Scaffold_5791	30	p3	(TCT)6	18	124150	124167	potentially variable
PHG9_Scaffold_5791	32	p2	(AT)6	12	126006	126017	
PHG9_Scaffold_5791	44	p3	(CAA)5	15	157967	157981	potentially variable
PHG9_Scaffold_5792	6	p2	(AT)6	12	27273	27284	
PHG9_Scaffold_5792	10	p2	(AT)6	12	36031	36042	
PHG9_Scaffold_5792	15	p2	(CT)8	16	55316	55331	potentially variable
PHG9_Scaffold_5793	5	p2	(CA)8	16	22071	22086	potentially variable
PHG9_Scaffold_5793	8	p2	(AT)7	14	26792	26805	potentially variable
PHG9_Scaffold_5793	9	p2	(TG)7	14	29875	29888	potentially

							variable
PHG9_Scaffold_5793	20	p3	(TCC)5	15	57123	57137	potentially variable
PHG9_Scaffold_5793	28	p2	(GA)6	12	91984	91995	
PHG9_Scaffold_5793	34	p2	(AT)8	16	118307	118322	potentially variable
PHG9_Scaffold_5793	42	p2	(AG)6	12	165864	165875	
PHG9_Scaffold_5793	51	p2	(TC)8	16	185817	185832	potentially variable
PHG9_Scaffold_5793	53	p2	(TA)6	12	190299	190310	
PHG9_Scaffold_5794	4	p2	(TA)9	18	3742	3759	potentially variable
PHG9_Scaffold_5794	5	p2	(CT)8	16	7877	7892	potentially variable
PHG9_Scaffold_5794	14	p2	(CA)8	16	30222	30237	potentially variable
PHG9_Scaffold_5794	26	p3	(CAC)6	18	58109	58126	potentially variable
PHG9_Scaffold_5794	28	p2	(TA)7	14	64864	64877	potentially variable
PHG9_Scaffold_5794	31	p3	(TTA)5	15	68748	68762	potentially variable
PHG9_Scaffold_5794	37	p2	(TA)7	14	92740	92753	potentially variable
PHG9_Scaffold_5794	42	p2	(GA)6	12	101869	101880	
PHG9_Scaffold_5794	45	p2	(AG)8	16	118522	118537	potentially variable
PHG9_Scaffold_5794	47	p3	(ATT)5	15	120909	120923	potentially variable
PHG9_Scaffold_5794	51	p3	(CAA)5	15	129953	129967	potentially

							variable
PHG9_Scaffold_5794	52	p2	(AT)6	12	131006	131017	
PHG9_Scaffold_5794	53	p2	(TA)6	12	138106	138117	
PHG9_Scaffold_5794	60	p2	(TC)6	12	143409	143420	
PHG9_Scaffold_5794	62	p2	(TA)9	18	147143	147160	potentially variable
PHG9_Scaffold_5795	4	p2	(AT)6	12	9393	9404	
PHG9_Scaffold_5795	17	p2	(AG)9	18	48183	48200	potentially variable
PHG9_Scaffold_5795	24	p3	(TAT)5	15	82121	82135	potentially variable
PHG9_Scaffold_5795	29	p2	(TC)6	12	101461	101472	
PHG9_Scaffold_5795	32	p3	(TTC)5	15	108059	108073	potentially variable
PHG9_Scaffold_5795	33	p3	(AAG)5	15	116820	116834	potentially variable
PHG9_Scaffold_5795	36	p2	(CT)7	14	119385	119398	potentially variable
PHG9_Scaffold_5795	39	p3	(ATT)5	15	126550	126564	potentially variable
PHG9_Scaffold_5795	57	p2	(AT)7	14	167775	167788	potentially variable
PHG9_Scaffold_5797	4	p2	(AT)7	14	12398	12411	potentially variable
PHG9_Scaffold_5797	5	p2	(TA)9	18	12740	12757	potentially variable
PHG9_Scaffold_5797	6	p2	(AT)7	14	15398	15411	potentially variable
PHG9_Scaffold_5797	8	p3	(TAC)5	15	17600	17614	potentially variable

PHG9_Scaffold_5797	15	p2	(TA)8	16	36317	36332	potentially variable
PHG9_Scaffold_5797	17	p2	(AT)6	12	39333	39344	
PHG9_Scaffold_5797	22	p2	(GA)9	18	57121	57138	potentially variable
PHG9_Scaffold_5797	26	p2	(TA)7	14	73420	73433	potentially variable
PHG9_Scaffold_5797	28	p2	(AG)8	16	84195	84210	potentially variable
PHG9_Scaffold_5797	33	p2	(AT)9	18	95785	95802	potentially variable
PHG9_Scaffold_5798	6	p2	(CA)6	12	29629	29640	
PHG9_Scaffold_5798	10	p3	(TTC)5	15	48059	48073	potentially variable
PHG9_Scaffold_5798	13	p2	(TA)6	12	52436	52447	
PHG9_Scaffold_5799	8	p2	(AT)6	12	18233	18244	
PHG9_Scaffold_5799	15	p3	(GTT)6	18	30537	30554	potentially variable
PHG9_Scaffold_5799	27	p2	(AT)6	12	85315	85326	
PHG9_Scaffold_5799	28	p2	(TA)9	18	86950	86967	potentially variable
PHG9_Scaffold_5801	4	p2	(TA)8	16	4618	4633	potentially variable
PHG9_Scaffold_5801	16	p2	(TA)7	14	29599	29612	potentially variable
PHG9_Scaffold_5801	18	p2	(TA)8	16	35814	35829	potentially variable
PHG9_Scaffold_5801	22	p3	(TAA)5	15	47149	47163	potentially variable
PHG9_Scaffold_5801	24	p3	(GAT)5	15	47907	47921	potentially

							variable
PHG9_Scaffold_5801	27	p2	(TA)8	16	50640	50655	potentially variable
PHG9_Scaffold_5802	11	p3	(GTT)5	15	28981	28995	potentially variable
PHG9_Scaffold_5802	15	p2	(TA)6	12	66710	66721	
PHG9_Scaffold_5802	16	p2	(TG)6	12	68710	68721	
PHG9_Scaffold_5802	18	p2	(TA)7	14	69699	69712	potentially variable
PHG9_Scaffold_5802	20	p3	(TAT)5	15	80559	80573	potentially variable
PHG9_Scaffold_5802	28	p2	(TA)6	12	95269	95280	
PHG9_Scaffold_5802	38	p2	(AT)7	14	137000	137013	potentially variable
PHG9_Scaffold_5802	45	p2	(TA)7	14	170440	170453	potentially variable
PHG9_Scaffold_5802	47	p2	(TC)9	18	173078	173095	potentially variable
PHG9_Scaffold_5802	48	p3	(TGC)5	15	173499	173513	potentially variable
PHG9_Scaffold_5802	56	p3	(CTC)5	15	197111	197125	potentially variable
PHG9_Scaffold_5802	59	p2	(CT)6	12	210189	210200	
PHG9_Scaffold_5802	63	p2	(TC)8	16	225712	225727	potentially variable
PHG9_Scaffold_5803	8	p2	(TG)6	12	35598	35609	
PHG9_Scaffold_5803	18	p3	(GCT)5	15	74177	74191	potentially variable
PHG9_Scaffold_5803	26	p2	(AT)9	18	90209	90226	potentially variable

PHG9_Scaffold_5803	31	p2	(AT)6	12	92398	92409	
PHG9_Scaffold_5803	32	p2	(GA)6	12	94013	94024	
PHG9_Scaffold_5803	41	p3	(AAT)5	15	119702	119716	potentially variable
PHG9_Scaffold_5804	9	p2	(AT)6	12	37621	37632	
PHG9_Scaffold_5806	1	p3	(TCA)5	15	1845	1859	potentially variable
PHG9_Scaffold_5806	16	p2	(TA)6	12	34483	34494	
PHG9_Scaffold_5806	24	p3	(AAT)5	15	50198	50212	potentially variable
PHG9_Scaffold_5806	51	p2	(AC)8	16	145850	145865	potentially variable
PHG9_Scaffold_5806	53	p6	(TCTTTC)5	30	149242	149271	Hypervariable
PHG9_Scaffold_5806	55	p2	(GA)6	12	162495	162506	
PHG9_Scaffold_5806	59	p2	(TA)8	16	171913	171928	potentially variable
PHG9_Scaffold_5806	62	p3	(TTC)5	15	176410	176424	potentially variable
PHG9_Scaffold_5807	10	p2	(AT)7	14	49322	49335	potentially variable
PHG9_Scaffold_5807	15	p2	(CT)9	18	74107	74124	potentially variable
PHG9_Scaffold_5807	18	p3	(TTA)5	15	84515	84529	potentially variable
PHG9_Scaffold_5807	23	p3	(TTA)6	18	89647	89664	potentially variable
PHG9_Scaffold_5808	3	p2	(TA)7	14	6330	6343	potentially variable
PHG9_Scaffold_5808	24	p3	(TTA)5	15	63598	63612	potentially variable

PHG9_Scaffold_5809	9	p2	(TA)6	12	26731	26742	
PHG9_Scaffold_5809	10	p6	(AAAGCC)5	30	29331	29360	Hypervariable
PHG9_Scaffold_5809	11	p3	(TCA)5	15	37115	37129	potentially variable
PHG9_Scaffold_5809	15	p2	(AT)6	12	50551	50562	
PHG9_Scaffold_5809	20	p2	(AT)9	18	64042	64059	potentially variable
PHG9_Scaffold_5809	23	p6	(CACAGC)5	30	96244	96273	Hypervariable
PHG9_Scaffold_5809	24	p2	(TA)9	18	96931	96948	potentially variable
PHG9_Scaffold_5809	37	p2	(AG)6	12	129046	129057	
PHG9_Scaffold_5809	39	p3	(CAG)6	18	132707	132724	potentially variable
PHG9_Scaffold_5809	43	p2	(TA)6	12	149921	149932	
PHG9_Scaffold_5809	48	p2	(TA)6	12	162693	162704	
PHG9_Scaffold_5809	49	p2	(TA)6	12	162931	162942	
PHG9_Scaffold_5809	51	p2	(AT)9	18	175046	175063	potentially variable
PHG9_Scaffold_5809	53	p2	(TA)6	12	175684	175695	
PHG9_Scaffold_5809	56	p3	(TTC)5	15	190872	190886	potentially variable
PHG9_Scaffold_5810	5	p3	(TGA)6	18	12709	12726	potentially variable
PHG9_Scaffold_5811	7	p2	(AT)6	12	16892	16903	
PHG9_Scaffold_5811	8	p2	(AT)6	12	22960	22971	
PHG9_Scaffold_5811	9	p2	(AT)6	12	25413	25424	
PHG9_Scaffold_5812	4	p2	(AT)7	14	35246	35259	potentially variable
PHG9_Scaffold_5812	15	p2	(AT)6	12	100382	100393	

PHG9_Scaffold_5812	25	p3	(AAG)5	15	125248	125262	potentially variable
PHG9_Scaffold_5813	4	p3	(GAG)5	15	21665	21679	potentially variable
PHG9_Scaffold_5814	9	p3	(ATA)5	15	68783	68797	potentially variable
PHG9_Scaffold_5814	11	p2	(AT)8	16	70266	70281	potentially variable
PHG9_Scaffold_5814	12	p2	(AT)7	14	70495	70508	potentially variable
PHG9_Scaffold_5815	1	p3	(TTC)5	15	34	48	potentially variable
PHG9_Scaffold_5816	1	p2	(TA)6	12	1648	1659	
PHG9_Scaffold_5816	4	p2	(AT)6	12	14762	14773	
PHG9_Scaffold_5816	5	p2	(AT)6	12	16157	16168	
PHG9_Scaffold_5816	18	p2	(TA)9	18	64536	64553	potentially variable
PHG9_Scaffold_5818	10	p2	(GA)6	12	21604	21615	
PHG9_Scaffold_5818	12	p6	(TCATGC)5	30	32603	32632	Hypervariable
PHG9_Scaffold_5818	14	p6	(TGAGCA)8	48	34404	34451	Hypervariable
PHG9_Scaffold_5818	15	p2	(CT)7	14	34655	34668	potentially variable
PHG9_Scaffold_5818	17	p2	(AG)6	12	38478	38489	
PHG9_Scaffold_5818	30	p2	(TA)7	14	89387	89400	potentially variable
PHG9_Scaffold_5818	33	p2	(TA)9	18	92478	92495	potentially variable
PHG9_Scaffold_5818	37	p3	(TCT)5	15	112659	112673	potentially variable

PHG9_Scaffold_5818	39	p2	(TG)7	14	121463	121476	potentially variable
PHG9_Scaffold_5818	47	p3	(TTA)5	15	151081	151095	potentially variable
PHG9_Scaffold_5818	48	p3	(TGA)5	15	154215	154229	potentially variable
PHG9_Scaffold_5819	3	p2	(AT)9	18	7842	7859	potentially variable
PHG9_Scaffold_5819	4	p2	(AT)6	12	8519	8530	
PHG9_Scaffold_5819	11	p2	(TA)6	12	26682	26693	
PHG9_Scaffold_5819	19	p2	(TA)7	14	39420	39433	potentially variable
PHG9_Scaffold_5819	30	p2	(TA)6	12	73546	73557	
PHG9_Scaffold_5819	34	p2	(AT)7	14	93244	93257	potentially variable
PHG9_Scaffold_5819	42	p2	(AT)6	12	119760	119771	
PHG9_Scaffold_5819	56	p2	(TA)7	14	173295	173308	potentially variable
PHG9_Scaffold_5821	5	p2	(TA)7	14	13399	13412	potentially variable
PHG9_Scaffold_5821	6	p2	(TA)7	14	15499	15512	potentially variable
PHG9_Scaffold_5821	11	p2	(AT)6	12	55350	55361	
PHG9_Scaffold_5822	4	p2	(CT)6	12	8712	8723	
PHG9_Scaffold_5822	5	p3	(ATT)5	15	9108	9122	potentially variable
PHG9_Scaffold_5822	9	p2	(TA)6	12	13684	13695	
PHG9_Scaffold_5822	25	p2	(TA)6	12	75859	75870	
PHG9_Scaffold_5822	27	p2	(TA)7	14	77217	77230	potentially

							variable
PHG9_Scaffold_5823	2	p3	(GTA)5	15	9427	9441	potentially variable
PHG9_Scaffold_5823	11	p3	(ATA)6	18	45494	45511	potentially variable
PHG9_Scaffold_5823	16	p2	(TG)9	18	62235	62252	potentially variable
PHG9_Scaffold_5823	20	p3	(TAT)5	15	69788	69802	potentially variable
PHG9_Scaffold_5823	26	p2	(AT)9	18	84044	84061	potentially variable
PHG9_Scaffold_5823	29	p3	(AAT)5	15	95370	95384	potentially variable
PHG9_Scaffold_5823	32	p2	(TA)6	12	106092	106103	
PHG9_Scaffold_5823	39	p3	(TTA)5	15	127224	127238	potentially variable
PHG9_Scaffold_5823	41	p3	(TTC)5	15	128672	128686	potentially variable
PHG9_Scaffold_5823	43	p3	(AAT)6	18	138227	138244	potentially variable
PHG9_Scaffold_5823	48	p2	(CT)9	18	142273	142290	potentially variable
PHG9_Scaffold_5823	56	p2	(AG)10	20	168167	168186	Hypervariable
PHG9_Scaffold_5823	59	p3	(GTG)5	15	172505	172519	potentially variable
PHG9_Scaffold_5823	61	p2	(CT)7	14	172897	172910	potentially variable
PHG9_Scaffold_5823	71	p3	(TAA)6	18	212733	212750	potentially variable
PHG9_Scaffold_5823	76	p3	(ATT)6	18	223448	223465	potentially

							variable
PHG9_Scaffold_5825	13	p3	(ATT)5	15	61029	61043	potentially variable
PHG9_Scaffold_5825	16	p2	(GA)8	16	76196	76211	potentially variable
PHG9_Scaffold_5826	6	p2	(TA)7	14	15387	15400	potentially variable
PHG9_Scaffold_5826	8	p3	(CTG)5	15	16643	16657	potentially variable
PHG9_Scaffold_5826	10	p2	(AT)9	18	24551	24568	potentially variable
PHG9_Scaffold_5826	13	p2	(TA)7	14	30429	30442	potentially variable
PHG9_Scaffold_5828	7	p2	(CT)6	12	36822	36833	
PHG9_Scaffold_5828	16	p2	(TA)6	12	57703	57714	
PHG9_Scaffold_5828	25	p3	(TAT)6	18	109361	109378	potentially variable
PHG9_Scaffold_5829	15	p3	(TAA)5	15	40150	40164	potentially variable
PHG9_Scaffold_5829	27	p2	(TA)6	12	66932	66943	
PHG9_Scaffold_5829	30	p2	(TA)7	14	75514	75527	potentially variable
PHG9_Scaffold_5829	35	p2	(TA)8	16	89377	89392	potentially variable
PHG9_Scaffold_5829	36	p3	(ATA)5	15	91864	91878	potentially variable
PHG9_Scaffold_5829	41	p3	(TAA)5	15	95551	95565	potentially variable
PHG9_Scaffold_5829	46	p2	(AT)6	12	103581	103592	
PHG9_Scaffold_5829	49	p2	(TA)7	14	125429	125442	potentially

							variable
PHG9_Scaffold_5829	54	p2	(TA)6	12	140045	140056	
PHG9_Scaffold_5829	57	p2	(TA)9	18	143270	143287	potentially variable
PHG9_Scaffold_5829	66	p2	(AT)6	12	165239	165250	
PHG9_Scaffold_5829	67	p2	(CT)6	12	169206	169217	
PHG9_Scaffold_5829	73	p2	(TA)6	12	183628	183639	
PHG9_Scaffold_5829	75	p3	(ATT)5	15	188976	188990	potentially variable
PHG9_Scaffold_5829	76	p2	(TA)7	14	189166	189179	potentially variable
PHG9_Scaffold_5829	86	p2	(AT)6	12	222952	222963	
PHG9_Scaffold_5830	2	p2	(TA)8	16	4704	4719	potentially variable
PHG9_Scaffold_5830	4	p2	(TG)7	14	11062	11075	potentially variable
PHG9_Scaffold_5831	11	p3	(AGA)5	15	46991	47005	potentially variable
PHG9_Scaffold_5831	17	p2	(TC)6	12	57585	57596	
PHG9_Scaffold_5831	18	p3	(CTT)5	15	59964	59978	potentially variable
PHG9_Scaffold_5831	21	p2	(TA)7	14	74342	74355	potentially variable
PHG9_Scaffold_5831	22	p2	(GA)8	16	79854	79869	potentially variable
PHG9_Scaffold_5831	23	p3	(CAC)5	15	81131	81145	potentially variable
PHG9_Scaffold_5831	29	p2	(TC)7	14	106240	106253	potentially variable

PHG9_Scaffold_5831	32	p3	(TGA)5	15	114130	114144	potentially variable
PHG9_Scaffold_5831	33	p2	(CT)6	12	114443	114454	
PHG9_Scaffold_5831	36	p3	(ATT)5	15	118987	119001	potentially variable
PHG9_Scaffold_5831	42	p2	(AT)6	12	141896	141907	
PHG9_Scaffold_5831	48	p3	(GAT)5	15	162083	162097	potentially variable
PHG9_Scaffold_5831	50	p2	(CT)8	16	168108	168123	potentially variable
PHG9_Scaffold_5831	54	p2	(AC)6	12	174196	174207	
PHG9_Scaffold_5831	62	p2	(TA)6	12	193566	193577	
PHG9_Scaffold_5831	67	p2	(AG)6	12	220300	220311	
PHG9_Scaffold_5831	68	p2	(TA)7	14	220974	220987	potentially variable
PHG9_Scaffold_5831	69	p2	(TC)6	12	235193	235204	
PHG9_Scaffold_5831	71	p3	(ATA)5	15	242050	242064	potentially variable
PHG9_Scaffold_5831	72	p3	(TTG)6	18	243654	243671	potentially variable
PHG9_Scaffold_5831	77	p3	(GAA)5	15	249813	249827	potentially variable
PHG9_Scaffold_5831	84	p2	(CA)6	12	281791	281802	
PHG9_Scaffold_5832	2	p3	(TAT)6	18	5870	5887	potentially variable
PHG9_Scaffold_5834	1	p2	(AG)6	12	2569	2580	
PHG9_Scaffold_5834	3	p3	(TTA)5	15	14863	14877	potentially variable
PHG9_Scaffold_5835	2	p2	(AT)7	14	7944	7957	potentially

							variable
PHG9_Scaffold_5835	3	p3	(TTA)5	15	11077	11091	potentially variable
PHG9_Scaffold_5835	5	p2	(TA)8	16	14787	14802	potentially variable
PHG9_Scaffold_5835	7	p3	(TGC)5	15	21389	21403	potentially variable
PHG9_Scaffold_5835	12	p2	(AT)6	12	50982	50993	
PHG9_Scaffold_5835	16	p2	(TA)7	14	67160	67173	potentially variable
PHG9_Scaffold_5835	34	p2	(CT)7	14	106512	106525	potentially variable
PHG9_Scaffold_5835	38	p2	(TC)6	12	129229	129240	
PHG9_Scaffold_5836	3	p2	(TA)9	18	4225	4242	potentially variable
PHG9_Scaffold_5836	4	p2	(TC)7	14	5760	5773	potentially variable
PHG9_Scaffold_5836	12	p2	(TC)6	12	24110	24121	
PHG9_Scaffold_5836	14	p2	(AT)10	20	26312	26331	Hypervariable
PHG9_Scaffold_5837	4	p2	(TA)6	12	5772	5783	
PHG9_Scaffold_5837	22	p2	(TA)6	12	48425	48436	
PHG9_Scaffold_5837	26	p3	(ATC)5	15	66000	66014	potentially variable
PHG9_Scaffold_5837	28	p2	(TA)9	18	84065	84082	potentially variable
PHG9_Scaffold_5837	31	p3	(TTC)6	18	91104	91121	potentially variable
PHG9_Scaffold_5837	34	p2	(TC)7	14	94199	94212	potentially variable

PHG9_Scaffold_5837	39	p2	(TC)8	16	102042	102057	potentially variable
PHG9_Scaffold_5837	44	p2	(AT)8	16	116235	116250	potentially variable
PHG9_Scaffold_5837	59	p2	(AT)6	12	167913	167924	
PHG9_Scaffold_5837	60	p2	(TA)8	16	168792	168807	potentially variable
PHG9_Scaffold_5837	65	p2	(GA)6	12	185555	185566	
PHG9_Scaffold_5837	78	p3	(AAT)5	15	210503	210517	potentially variable
PHG9_Scaffold_5837	87	p2	(AT)6	12	235216	235227	
PHG9_Scaffold_5837	92	p2	(TA)7	14	249367	249380	potentially variable
PHG9_Scaffold_5837	94	p2	(CT)8	16	254478	254493	potentially variable
PHG9_Scaffold_5837	98	p3	(ATT)5	15	258868	258882	potentially variable
PHG9_Scaffold_5837	100	p2	(CT)8	16	260580	260595	potentially variable
PHG9_Scaffold_5837	111	p2	(AT)6	12	292668	292679	
PHG9_Scaffold_5837	113	p3	(AAC)6	18	293567	293584	potentially variable
PHG9_Scaffold_5837	120	p6	(GAACAT)5	30	302501	302530	Hypervariable
PHG9_Scaffold_5837	121	p2	(AT)6	12	303583	303594	
PHG9_Scaffold_5837	126	p2	(AG)6	12	312949	312960	
PHG9_Scaffold_5837	140	p3	(TAT)5	15	358004	358018	potentially variable
PHG9_Scaffold_5838	1	p2	(TA)6	12	1670	1681	
PHG9_Scaffold_5841	23	p2	(AT)6	12	39952	39963	

PHG9_Scaffold_5841	24	p2	(CA)6	12	41020	41031	
PHG9_Scaffold_5841	26	p2	(TC)8	16	44052	44067	potentially variable
PHG9_Scaffold_5842	9	p2	(GT)6	12	52643	52654	
PHG9_Scaffold_5842	18	p2	(AT)9	18	61577	61594	potentially variable
PHG9_Scaffold_5842	29	p3	(TAT)6	18	78737	78754	potentially variable
PHG9_Scaffold_5842	38	p3	(TCA)5	15	99124	99138	potentially variable
PHG9_Scaffold_5842	42	p3	(AAG)5	15	106495	106509	potentially variable
PHG9_Scaffold_5842	59	p2	(AT)7	14	137494	137507	potentially variable
PHG9_Scaffold_5842	72	p2	(AT)6	12	165153	165164	
PHG9_Scaffold_5842	97	p2	(TA)8	16	219827	219842	potentially variable
PHG9_Scaffold_5843	4	p2	(AT)9	18	3251	3268	potentially variable
PHG9_Scaffold_5843	5	p2	(AT)6	12	4000	4011	
PHG9_Scaffold_5843	13	p2	(AT)7	14	30164	30177	potentially variable
PHG9_Scaffold_5843	18	p3	(AGC)5	15	36839	36853	potentially variable
PHG9_Scaffold_5843	19	p2	(CT)9	18	36989	37006	potentially variable
PHG9_Scaffold_5843	31	p3	(CCT)5	15	67352	67366	potentially variable
PHG9_Scaffold_5844	4	p2	(TC)7	14	25933	25946	potentially variable

PHG9_Scaffold_5844	9	p2	(GA)7	14	60246	60259	potentially variable
PHG9_Scaffold_5844	29	p2	(TA)6	12	124322	124333	
PHG9_Scaffold_5844	31	p2	(AT)7	14	126240	126253	potentially variable
PHG9_Scaffold_5844	34	p2	(TA)7	14	129074	129087	potentially variable
PHG9_Scaffold_5844	37	p2	(TA)6	12	130254	130265	
PHG9_Scaffold_5845	9	p2	(TA)8	16	46271	46286	potentially variable
PHG9_Scaffold_5845	13	p3	(ATT)6	18	53942	53959	potentially variable
PHG9_Scaffold_5846	1	p2	(AT)8	16	2877	2892	potentially variable
PHG9_Scaffold_5846	6	p2	(TA)8	16	10726	10741	potentially variable
PHG9_Scaffold_5846	11	p3	(CAG)5	15	29630	29644	potentially variable
PHG9_Scaffold_5846	15	p2	(TA)9	18	38571	38588	potentially variable
PHG9_Scaffold_5846	17	p2	(TA)6	12	45269	45280	
PHG9_Scaffold_5847	3	p2	(AT)7	14	10173	10186	potentially variable
PHG9_Scaffold_5847	4	p2	(TA)7	14	10431	10444	potentially variable
PHG9_Scaffold_5847	10	p2	(AT)6	12	19371	19382	
PHG9_Scaffold_5847	11	p2	(GA)9	18	22221	22238	potentially variable
PHG9_Scaffold_5847	16	p2	(AT)9	18	27494	27511	potentially variable

PHG9_Scaffold_5847	19	p3	(TTC)5	15	48518	48532	potentially variable
PHG9_Scaffold_5847	21	p2	(TA)7	14	50707	50720	potentially variable
PHG9_Scaffold_5847	22	p2	(TA)9	18	58328	58345	potentially variable
PHG9_Scaffold_5847	23	p3	(ATA)5	15	61811	61825	potentially variable
PHG9_Scaffold_5847	24	p2	(AT)6	12	68283	68294	
PHG9_Scaffold_5847	28	p2	(GA)6	12	84630	84641	
PHG9_Scaffold_5847	30	p2	(CT)6	12	90891	90902	
PHG9_Scaffold_5847	34	p2	(GA)9	18	103534	103551	potentially variable
PHG9_Scaffold_5847	35	p2	(TA)7	14	108739	108752	potentially variable
PHG9_Scaffold_5847	41	p2	(TA)7	14	115524	115537	potentially variable
PHG9_Scaffold_5848	2	p2	(AG)9	18	3523	3540	potentially variable
PHG9_Scaffold_5848	4	p2	(CT)7	14	7123	7136	potentially variable
PHG9_Scaffold_5849	3	p2	(TG)7	14	32565	32578	potentially variable
PHG9_Scaffold_5851	2	p2	(TG)8	16	28108	28123	potentially variable
PHG9_Scaffold_5851	6	p3	(AAT)6	18	58253	58270	potentially variable
PHG9_Scaffold_5851	10	p3	(ATA)5	15	72251	72265	potentially variable
PHG9_Scaffold_5851	18	p2	(TA)8	16	115673	115688	potentially

							variable
PHG9_Scaffold_5851	24	p2	(TA)6	12	140591	140602	
PHG9_Scaffold_5851	45	p3	(ATA)6	18	203960	203977	potentially variable
PHG9_Scaffold_5851	62	p2	(AT)6	12	245323	245334	
PHG9_Scaffold_5851	63	p2	(TA)6	12	247007	247018	
PHG9_Scaffold_5851	64	p2	(AT)8	16	247260	247275	potentially variable
PHG9_Scaffold_5851	71	p3	(ATT)5	15	259724	259738	potentially variable
PHG9_Scaffold_5852	2	p3	(TGA)5	15	10689	10703	potentially variable
PHG9_Scaffold_5852	4	p3	(TTC)5	15	20889	20903	potentially variable
PHG9_Scaffold_5852	10	p2	(TA)7	14	42208	42221	potentially variable
PHG9_Scaffold_5852	11	p3	(TTC)5	15	43736	43750	potentially variable
PHG9_Scaffold_5852	17	p3	(AAT)5	15	56099	56113	potentially variable
PHG9_Scaffold_5852	22	p3	(TTC)5	15	64293	64307	potentially variable
PHG9_Scaffold_5852	24	p3	(ACA)5	15	82735	82749	potentially variable
PHG9_Scaffold_5852	29	p2	(TG)7	14	101628	101641	potentially variable
PHG9_Scaffold_5854	2	p2	(AT)6	12	1522	1533	

Supplementary Table S4. Details of ddRAD-Seq data generated for horsegram germplasm accessions.

Sample Code	Germplasm accession	Raw Reads generated	GC %	Q30 bases	Read length	No. of passed reads	No. of aligned reads	% mapping	No. of uniquely aligned reads	% uniquely mapped reads	Origin
G1	IC47132	47,20,572	42.59	92.63	100 x 2	46,14,018	29,15,807	63.20	28,76,054	62.33	Andhra Pradesh
G2	IC47461	42,11,322	41.79	91.80	100 x 2	41,13,250	27,11,298	65.92	26,78,362	65.12	Uttar Pradesh
G3	IC56132	59,46,032	43.59	92.57	100 x 2	57,99,768	34,46,837	59.43	34,00,173	58.63	Andhra Pradesh
G4	IC71743	50,10,950	44.05	92.78	100 x 2	49,06,404	32,06,900	65.36	31,74,882	64.71	Tamil Nadu
G5	IC71775	11,60,606	43.87	93.03	100 x 2	11,08,938	6,74,064	60.79	6,65,049	59.97	Tamil Nadu
G6	IC71809	49,65,970	41.32	92.29	100 x 2	47,96,084	30,10,374	62.77	29,70,109	61.93	Tamil Nadu
G7	IC71814	52,38,808	43.82	93.11	100 x 2	50,55,362	31,67,815	62.66	31,25,430	61.82	Tamil Nadu
G8	IC123022	18,43,838	44.11	92.20	100 x 2	17,58,630	10,99,994	62.55	10,91,693	62.08	
G9	IC139412	52,72,228	42.57	92.25	100 x 2	50,77,596	30,70,677	60.48	30,30,925	59.69	Rajasthan
G10	IC139453	49,08,014	42.41	92.47	100 x 2	48,04,256	31,13,185	64.80	30,73,824	63.98	Himachal Pradesh
G11	IC139548	45,98,882	40.98	92.75	100 x 2	45,03,636	26,48,012	58.80	25,83,146	57.36	Himachal Pradesh
G12	IC139556	49,92,708	42.11	91.98	100 x 2	48,67,084	31,07,647	63.85	30,62,177	62.92	Himachal Pradesh
G13	IC344193	43,04,054	41.53	92.85	100 x 2	42,00,794	23,98,083	57.09	23,40,347	55.71	Maharashtra

G14	IC355643	35,28,052	42.37	92.93	100 x 2	34,55,060	19,84,677	57.44	19,36,949	56.06	
G15	IC561032	49,35,330	41.94	93.29	100 x 2	48,38,360	28,47,169	58.85	27,77,252	57.40	Jharkhand
G16	IC343106	34,44,248	42.30	93.53	100 x 2	33,88,326	21,04,595	62.11	20,71,970	61.15	Jharkhand
G17	IC139450	30,30,262	42.59	93.81	100 x 2	29,74,264	16,94,315	56.97	16,67,730	56.07	Jharkhand
G18	IC347182	38,32,100	39.79	93.39	100 x 2	37,64,864	22,91,541	60.87	22,52,389	59.83	Jharkhand
G19	IC345439	43,81,806	42.28	94.17	100 x 2	43,10,282	26,11,073	60.58	25,69,695	59.62	
G20	IC203201	37,45,226	41.93	93.65	100 x 2	36,87,564	22,21,099	60.23	21,91,123	59.42	Odisha
G21	IC341263	26,69,516	44.34	92.77	100 x 2	26,14,258	17,05,236	65.23	16,94,871	64.83	Maharashtra
G22	IC342685	51,92,638	42.97	92.04	100 x 2	50,94,376	33,60,295	65.96	33,15,314	65.08	
G23	IC385836	32,02,934	44.59	92.85	100 x 2	31,32,638	21,07,747	67.28	20,95,752	66.90	Jharkhand
G24	TCR1635	50,65,728	45.65	92.86	100 x 2	49,70,134	31,54,868	63.48	31,31,197	63.00	Tamil Nadu
G25	TCR1734	15,94,014	44.84	93.50	100 x 2	15,53,296	10,41,589	67.06	10,33,073	66.51	
G26	TCR1752	43,24,696	43.46	94.89	100 x 2	42,39,168	26,31,310	62.07	25,89,882	61.09	
G27	TCR1755	52,04,180	44.86	94.86	100 x 2	50,76,950	30,55,556	60.19	30,31,844	59.72	
G28	TCR1762	38,45,598	41.96	94.68	100 x 2	37,58,102	23,27,804	61.94	23,06,826	61.38	

G29	TCR1781	34,49,842	44.11	95.21	100 x 2	33,60,250	21,49,214	63.96	21,26,859	63.30	
G30	TCR1789	65,59,266	43.85	94.74	100 x 2	64,20,830	38,21,706	59.52	37,73,877	58.78	
G31	TCR1814	42,23,704	41.53	94.61	100 x 2	41,56,524	23,88,636	57.47	23,42,086	56.35	
G32	BGM 1	32,60,776	43.08	92.48	100 x 2	31,76,192	22,26,851	70.11	22,07,873	69.51	
G33	AK 26	51,87,640	42.38	94.58	100 x 2	50,93,420	29,35,919	57.64	28,75,068	56.45	
G34	HG 4	40,21,738	42.74	94.72	100 x 2	39,59,736	22,59,014	57.05	22,11,197	55.84	
G35	HG 6	23,78,542	42.09	95.08	100 x 2	23,43,452	13,95,284	59.54	13,58,347	57.96	
G36	HG 9	36,23,582	42.13	94.69	100 x 2	35,65,726	21,97,968	61.64	21,57,355	60.50	
G37	HG 11	31,58,606	42.62	94.98	100 x 2	31,09,460	19,04,594	61.25	18,77,397	60.38	
G38	HG 12	35,53,174	40.23	94.59	100 x 2	34,99,106	19,95,016	57.02	19,61,122	56.05	
G39	IC385837	46,99,560	43.25	95.10	100 x 2	46,27,166	24,69,759	53.38	24,31,271	52.54	Jharkhand
G40	IC139331	33,45,864	41.44	94.79	100 x 2	32,93,136	20,30,017	61.64	19,95,763	60.60	Maharashtra

Supplementary Table S5. Missense and non-sense substitutions/SNPs in the exonic region of genes.

Scaffold ID	SNP_position	Reference allele	Alternate allele	SNP type	Gene ID	Gene annotation	Pfam_domain	SNP_position_up_downstream_sequence
PHG9_Scaffold_1031	5866			missense_variant	Mu_g08904.t1	hypothetical protein LR48_Vigan07g228600 [Vigna angularis]	Zinc-binding dehydrogenase	AATAGAGGATTACCTATAAAAAATTTATTATCTCTCTTCACAATTTAGAGAGACACGTGTCCCCCAACAGAGTCATGTACTATTTCAGCCATGTTAATCT[T/A]CATGCGGATAAGACACCTAATGGACTCTCCTTTCAGCATGTATTCAAATGCTTTGTTGATCTCTGCGAATGGAACTGAGTGAGTGATGAATTTGTCCACT
PHG9_Scaffold_105	66796			missense_variant	Mu_g02201.t1	hypothetical protein PHAVU_007G183900g [Phaseolus vulgaris]	MSP (Major sperm protein) domain	CTAACAGCAAGTAGCAGGGATAAAGGGAGGTGCTCAGACCTTGAAGGCCACATAGTCAAAGGTGTTATTAGCCAACCGAACCGAGCAAGAACTTTGCTTC[T/C]CAATTCAACTACAAGGCGAAAAATCACGGCATATTATTCAACCGATCACCCAAACACACAAGTTATAATAGATTCAACAACATCAGCATGCATGGCCAGA

PHG9_Scaffold_1070	0244			missense_variant	Mu_g09076.t1	hypothetical protein PHAVU_001G163700g [Phaseolus vulgaris]	Protein kinase domain	ACCTGTGAGTAATTGTGCTCTATAAACACTTCCAAATCCCCCCTTTCCGATGCAGTGCTTGTCATTGAAGTCATCGGTGGCCTTAACAAGATCAGAAAAT[G/C]GAATTTTCCATCTCTTCCCCACACCACGCTAATAGGTTGATCACTTTTCTCAATGCTTTTGGACTCTTCATCAAGGTGTTTTTGGCATGCCGCCGCCAA
PHG9_Scaffold_1070	0245			missense_variant	Mu_g09076.t1	hypothetical protein PHAVU_001G163700g [Phaseolus vulgaris]	Protein kinase domain	CCTGTGAGTAATTGTGCTCTATAAACACTTCCAAATCCCCCCTTTCCGATGCAGTGCTTGTCATTGAAGTCATCGGTGGCCTTAACAAGATCAGAAAATG[T/C]AATTTTCCATCTCTTCCCCACACCACGCTAATAGGTTGATCACTTTTCTCAATGCTTTTGGACTCTTCATCAAGGTGTTTTTGGCATGCCGCCGCCAAA
PHG9_Scaffold_1070	1005			missense_variant	Mu_g09077.t1	PREDICTED: MDIS1-interacting receptor like kinase	Protein kinase domain	TTGGCGGCGGCATGCCAAAAAACACCTTGATGAAGAGTCCAAAAGCATTGATAAAAGTGATCAACCTATTAGCGTGGTGTGGGAAGAGATGGAAAATTC[A/G]ATTTCTGATCTTGTTAAGGCCACCGATGACTTCAATGACAAGTACTGCATCGGAAAGGGAGGATTTGGAAGTGTTTATAGAGCACAATTACTCACAGGC

						2-like isoform X1 [Vigna angularis]		
PHG9_Scaffold_1070	1006			missense_variant	Mu_g09077.t1	PREDICTED: MDIS1-interacting receptor like kinase 2-like isoform X1 [Vigna angularis]	Protein kinase domain	TGGCGGCGGCATGCCAAAAAACACCTTGATGAAGAGTCCAAAAGCATTGATAAAAGTGATCAACCTATTAGCGTGGTGTGGGAAGAGATGGAAAATTCA[C/G]TTTTCTGATCTTGTTAAGGCCACCGATGACTTCAATGACAAGTACTGCATCGGAAAGGGAGGATTTGGAAGTGTTTATAGAGCACAATTACTCACAGGCC
PHG9_Scaffold_1083	03122			missense_variant	Mu_g09172.t1	hypothetical protein TSUD_113870 [Trifolium subterraneum	Protein tyrosine kinase	ACTTGCAATTCTATGTATGTTGATGTGTGAACACATGTAGATTGCAGTGAAAAGATTGAAAGTTTGGAGCAACAAAGCAGACATGGAATTTGCTGTTGAA[G/C]TGAGATATTGGCTAGAGTACGACACAAGAATCTTCTCAGTCTACGTGGCTATTGTGCTGAAGGTCAGGAACGATTAATTGTATATGATTATATGCCGAAT

						]		
PHG9_Scaffold_111	6597			missense_variant	Mu_g02313.t1	ATP synthase CF1 alpha subunit (chloroplast) [Pachyrhizus erosus]	ATP synthase alpha/beta family, nucleotide-binding domain	CGTACTTCAAGTAGGCGATGGTATTGCTCGTATTTTTGGTCTTGATGAAGTAATGGCAGGTGAATTGGTGGAATTTGAAGAAGGTACTATAGGCATTGCT[T/A]GAATTTGGAATCAAAAAATGTTGGTGTTGTGTTAATGGGTGATGGTTTGATGATACAAGAGGGAAGTTCAGTAAAAGCAACAGGAAGAATTGCTCAGATA
PHG9_Scaffold_1156	12249			missense_variant	Mu_g09621.t1	PREDICTED: WRKY transcription factor 44 isoform X2 [Vigna angularis]	WRKY DNA -binding domain	TCTATCACAATTCGCTGAGGATGTTAAAGTCTTTTGGTCCTCCTCTGTGTTCTGCTGCACCATCTTATTAGGTTCACTGATCTGATTTATTTCTGCATGC[G/T]GACAATACTCTGATCAAGATGTGAACTCATGTTGGTTCTAAATTTTTCATTGATGGAATGTTGAAAATTGGCCTCCATTGGTTGCCGAGTTTGCTGCTGA
PHG9_Scaffold_116	3001			missense_variant	Mu_g02392.t1	hypothetical protein PHAVU_002G2146	TB2/DP1, HVA22 family	CACATTGATCATAGAGTCCAATCAGGTTTTCAGTCCCAGTCAGAGAAAAAAAAAAAAAAACTTGACTCCATTATTGGGAAAAGAATGTTCTTACCATTGA[A/G]AAGCCTAGAAAATGCATGCTCAAATAGGTATATTAGCGAGAGAAGTATCCAATATGAGATCAAATCCCTAGTTTCTTTATAGGAATCAGTCTCAATTGCT

						00g [Phaseolus vulgaris]		
PHG9_Scaffold_118	58152			missense_variant	Mu_g02469.t1	hypothetical protein PHAVU_001G265700g [Phaseolus vulgaris]	AMP-binding enzyme	CCGCATGAGAAAATGAAATACTCACTAGCTCTTTCATACGCATGCCAGGAAGGCAACATGCTTAGAAATCTATCCCCAACTTCAGCAGGTACAACATCCC[C/A]AAGTTTTTATCTAGAAATTCATTAACAAGTGATTAGCAACATCAATGATCACTTGAAACCAAGAATTATCTTTAACTGAAACCAATTTAAAACCTGGAA
PHG9_Scaffold_120	07837			missense_variant	Mu_g02523.t1	hypothetical protein PHAVU_007G081900g [Phaseolus vulgaris]	RNA recognition motif. (a.k.a. RRM, RBD, or RNP domain)	GATTCATATATTCTGTAACTGCGAAAAGGAGATACGGATTACTGAGCCAGAAAGTGTGCTGGCATGCTTGCACACAAGGGCTTCGGTAGCCTGCTCCTCA[T/G]CTCAAACTGAACCAGGGCCTGTTTTTCCCATTCATCTCAAAGACTTTACTGTTGAGTATGGTTCCATGCTCCTCTACAAGGCTCACAATCTCCTCCTCA
PHG9_Scaffold_	7625			missense_varian	Mu_g10001.t	PREDICTED: protein SAR	Calmodulin binding protein-like	AAGTCACAATCAAGCATGCAATGACTTGTGAGATGGGGAGCAAGTTGTACATTTATAGAGGACCACAGTTTACTATTTTTCTGGATCCAATATGCAAATT[A/T]TTAGAGCTGATGTCAATGGACAGACATTTTCCAACAGAGATCCTATGAGCCATTTGAATAAGGTACAATGAATCATCTACTCTCATGAACTCAA

1236				t	1	DEFICIENT 1-like [Vigna angularis]		TTGTTC
PHG9_Scaffold_1236	11927			missense_variant	Mu_g10004.t1	hypothetical protein PHAVU_004G081800g [Phaseolus vulgaris]	PPR repeat family	ATTCTCTGCCTTGTCAGTTTCATTCTTTTCAAATAATGCACAGATAATTATTTCGAAAGTTATAGCATTAGGCATGCAACCACTGTCTTCCATTTTTAAC[C/A]CAAGTCCAATGCCTCATCAACCAATCCTTCTTTACATAATCCATTCATCATAATGTTATAAGTACATACGTTGACCGGGTAGCCTTTAATCAAAAGATTT
PHG9_Scaffold_1236	11960			missense_variant	Mu_g10004.t1	hypothetical protein PHAVU_004G081800g [Phaseolus vulgaris]	PPR repeat family	TAATGCACAGATAATTATTTCGAAAGTTATAGCATTAGGCATGCAACCACTGTCTTCCATTTTTAACCACAAGTCCAATGCCTCATCAACCAATCCTTCT[T/A]ACATAATCCATTCATCATAATGTTATAAGTACATACGTTGACCGGGTAGCCTTTAATCAAAAGATTTTGAAAAACTTCTTTTGCATCCTTTAGCCTTCTC
PHG9_Scaf	4798			missense_v	Mu_g1018	26S proteasome	26S proteasome	CGTTTTGAAATTTATTTCAGCTGGTCTGACCGAGCAGATTAAGTTGGACCGCTATCTGCATCCTCATTTCCGATATTACATGAGGGAGGTCAGAACTGTT[T/G]GTACTCCCAATTTTTGGAATCTTACAAGAGTGTGACAATTGAGGCC

fold_ 1258				arian t	0.t 1	non-ATPas e regulat ory subunit 6 homol og [Vigna radiata var. radiata]	subunit RPN7	ATGGCCAAAGCTTTTGGAGTGACAGTAGATTTCATTGATCTGTAAGTAA ATGCA
P HG9 _Scaf fold_ 126	92 53			m issen se_v arian t	Mu _g0 266 8.t 1	hypoth etical protein PHAV U_008 G0507 00g [Phase olus vulgari s]	Protein tyrosine kinase	CTGAAGGTTGCATGCAACTGTGTTACAGAAATGCCAAAGGAGAGGCCTA CCATGTTTGAAGTATACCAGCTTCTAAGAGCCATTGGCATGGATTATAA TT[T/G]ACGACTGAGGATGAGATAATGCTGCCCGTGGACATTGGTGATGC TGATAACTTAGAAGAACTTATTGTAGCTCGAGATGGACATGATTGAAAA GGTATGT
P HG9 _Scaf fold_ 1260	03 59 6			m issen se_v arian t	Mu _g1 020 5.t 1	hypoth etical protein PHAV U_003 G2910	Ubiquitin -2 like Rad60 SUMO-like	GATTATCTTGGAGAAAAGAAAGAATCATGCATGCACCTGAAAGCTGATG GATAAGTCCACATCAGTGCACACATCATCTGAGGGAGATTTTCTCTTAA GT[G/A]CGTAGTGGTAGCCATATTCTTCTTCTGCACACACAATGGGAAGA GATATATGTAAGATTCCACTATCTCAATCAATCAAAGAAGAAAACAAAA GAACCCT

						00g [Phaseolus vulgaris]		
PHG9_Scaffold_1275	1985			missense_variant	Mu_g10264.t1	PREDICTED: probable LRR receptor-like serine/threonine-protein kinase IRK [Vigna angularis]	Protein tyrosine kinase	TGCTGTCATTGTAATTGGTGTTATTAGCATCACTGTTCTCAATCTCCGGGTTCGCTCCTCCACATCTCGAGATGCAGCTGCCCTCACATTTTCTGCGGGA[G/A]TGAATTCAGCCGTTCCCCAACTACAGATGCTAACTCTGGCAAGCTTGTCATGTTTTCAGGTGAGCCTGATTTCAGTTCTGGCGCGCATGCTTTGCTTAAT
PHG9_Scaffold_128	2722			missense_variant	Mu_g02712.t1	magnesium transporter MRS2-2-like	CorA-like Mg2+ transporter protein	ATTTCAAGCTCTTCCACATCATTTTCATCGAAACGAACTGTTGCTAGACTTGCTCTAGATATCTTTGATCCTATGGTGGGGGAGGCAGTAAACCAATTTG[C/A]TTTGCAGCACCTGATCCACTGATTGGTGATGATGAACCTGCTTTTCTTGACAGGTATAGGTCAGCCATATCATCATCATCTTCCATCAATTGTTCAAGCT
PHG9_Scaffold_128	2730			missense_variant	Mu_g02712.t1	magnesium transporter MRS2-	CorA-like Mg2+ transporter protein	CTCTTCCACATCATTTTCATCGAAACGAACTGTTGCTAGACTTGCTCTAGATATCTTTGATCCTATGGTGGGGGAGGCAGTAAACCAATTTGCATTTGCA[G/C]ACCTGATCCACTGATTGGTGATGATGAACCTGCTTTTCTTGACAGGTATAGGTCAGCCATATCATCATCATCTTCCATCAATTGTTCAAGCTCGTCTCTG

						2-like		
PHG9_Scaffold_128	2746			missense_variant	Mu_g02712.t1	magnesium transporter MRS2-2-like	CorA-like Mg2+ transporter protein	TCATCGAAACGAACTGTTGCTAGACTTGCTCTAGATATCTTTGATCCTATGGTGGGGGAGGCAGTAAACCAATTTGCATTTGCAGCACCTGATCCACTGA[T/C]GGTGATGATGAACCTGCTTTTCTTGACAGGTATAGGTCAGCCATATCATCATCATCTTCCATCAATTGTTCAAGCTCGTCTCTGACCTGTACCAGATAAA
PHG9_Scaffold_128	2755			missense_variant	Mu_g02712.t1	magnesium transporter MRS2-2-like	CorA-like Mg2+ transporter protein	CGAACTGTTGCTAGACTTGCTCTAGATATCTTTGATCCTATGGTGGGGGAGGCAGTAAACCAATTTGCATTTGCAGCACCTGATCCACTGATTGGTGATG[A/C]GAACCTGCTTTTCTTGACAGGTATAGGTCAGCCATATCATCATCATCTTCCATCAATTGTTCAAGCTCGTCTCTGACCTGTACCAGATAAAAATAATTGG
PHG9_Scaffold_128	2804			missense_variant	Mu_g02712.t1	magnesium transporter MRS2-2-like	CorA-like Mg2+ transporter protein	AGGCAGTAAACCAATTTGCATTTGCAGCACCTGATCCACTGATTGGTGATGATGAACCTGCTTTTCTTGACAGGTATAGGTCAGCCATATCATCATCATC[T/A]CCATCAATTGTTCAAGCTCGTCTCTGACCTGTACCAGATAAAAATAATTGGTAATGGGAGAGGAAAAAGGTATATCTGAGAAAGGGGATGCAGAATACAT
PHG9_Scaffold_128	2809			missense_variant	Mu_g02712.t1	magnesium transporter MRS2-2-like	CorA-like Mg2+ transporter protein	GTAAACCAATTTGCATTTGCAGCACCTGATCCACTGATTGGTGATGATGAACCTGCTTTTCTTGACAGGTATAGGTCAGCCATATCATCATCATCTTCCA[T/A]AATTGTTCAAGCTCGTCTCTGACCTGTACCAGATAAAAATAATTGGTAATGGGAGAGGAAAAAGGTATATCTGAGAAAGGGGATGCAGAATACATTTAGA
PHG9_Scaffold_1292	940			missense_variant	Mu_g10335.t1	polygalacturonase 1 beta-like	BURP domain	GAACCAGGTAAGTTCTTCCGCGAGAGCATGCTTAAGGAAGGAACTGTTATGCCAATGCCAGATATAAGAGATAAAATGCCGCAAAGGTCGTTTTTACCCC[G/C]TCCATTTTGGACAAATTACCCTTCTCTTCTTCGAAGGTTGAGGAGTTGATGAAGGTGTTCAAGGTGTCTGATAACTCCTCGATGGAGAAGATGATCATCG

						protein 3 [Vigna radiata var. radiata]		
PHG9_Scaffold_1292	952			missense_variant	Mu_g10335.t1	polygalacturonase 1 beta-like protein 3 [Vigna radiata var. radiata]	BURP domain	TTCTTCCGCGAGAGCATGCTTAAGGAAGGAACTGTTATGCCAATGCCAGATATAAGAGATAAAATGCCGCAAAGGTCGTTTTACCCCGCTCCATTTTGG[A/C]AAATTACCCTTCTCTTCTTCGAAGGTTGAGGAGTTGATGAAGGTGTTCAAGGTGTCTGATAACTCCTCGATGGAGAAGATGATCATCGACTCTTTGTCTG
PHG9_Scaffold_1298	4862			missense_variant	Mu_g10393.t1	protein SIEVE ELEMENT OCCLUSION B [Vigna radiata var. radiata]	Sieve element occlusion N-terminus	CACTGTAAAGATAATTTAAATTGCTGACAAAGGATATGCTAATAAACAAGCATAAAAGCTTTGAAAAAGATTGGGAAAGCATACGCATATCCATTAGTGA[G/T]CTTGTAATCTGAGTTGTGCAAGCAACAATACTTCTGATGGTCCAATAGCAAACAATTGGGATATAGTCATTGGCAGTGGCGTGTTCGGTTGGCAGATCTT

PHG9_Scaffold_1298	4867			missense_variant	Mu_g10393.t1	protein SIEVE ELEMENT OCCLUSION B [Vigna radiata var. radiata]	Sieve element occlusion N-terminus	TAAAGATAATTTAAATTGCTGACAAAGGATATGCTAATAAACAAGCATAAAAGCTTTGAAAAAGATTGGGAAAGCATACGCATATCCATTAGTGAGGCTT[G/T]AATCTGAGTTGTGCAAGCAACAATACTTCTGATGGTCCAATAGCAAACAATTGGGATATAGTCATTGGCAGTGGCGTGTTCGGTTGGCAGATCTTGAACC
PHG9_Scaffold_1298	5140			missense_variant	Mu_g10393.t1	protein SIEVE ELEMENT OCCLUSION B [Vigna radiata var. radiata]	Sieve element occlusion N-terminus	CGAACTTGCATTTTCCATGATACTAGGAAATTGCTTCAGAATTGCCAATTTTTTTGCAAGCTGGTCGCTATCTTGAACATGAGCAAACAGACAAAATTCA[C/G]GTAGGTCAAAGTAAAAGCGGCTAAGGCCAAAACTATCTTCATATCCCACTTGTACATTGTAAGCATGTCTAATACGGCAATTGTTGTCCGGTGTTCATCT
PHG9_Scaffold_1298	5184			missense_variant	Mu_g10393.t1	protein SIEVE ELEMENT OCCLUSION B	Sieve element occlusion N-terminus	CCAATTTTTTTGCAAGCTGGTCGCTATCTTGAACATGAGCAAACAGACAAAATTCACCGTAGGTCAAAGTAAAAGCGGCTAAGGCCAAAACTATCTTCAT[A/T]CCCACTTGTACATTGTAAGCATGTCTAATACGGCAATTGTTGTCCGGTGTTCATCTACACCATTCAGAATCTTGTAGGAAATCTGAAATTACATTTTTTA

						[Vigna radiata var. radiata]		
PHG9_Scaffold_1298	5828			missense_variant	Mu_g10393.t1	protein SIEVE ELEMENT OCCLUSION B [Vigna radiata var. radiata]	Sieve element occlusion N-terminus	TATTTTAAATAGAACCAATCTTCAATGACATGCGTGTCTATCGTAAATTTCAATTGCATAAGCAACTTTTTAAGCAATTAGCTTCATACCTTTGTAGCTC[C/T]TCAAACCTCAAAGCGGAATGTATAAGCGTGTCCTCAACAAAACGGAAAATTGGCTTCACGTCATATTCTATTCCCTCGTCGGGTTTGTGCTTATCGCCTA
PHG9_Scaffold_13	31644			missense_variant	Mu_g00209.t1	F-box/kelch-repeat protein At1g57790 [Vigna radiata var. radiata]	F-box-like	TTTTATCTTGCATGCTTATAGTTAATACTTATTATTCAGGCTAAGCGACACAATCACAGATAATAGAAGAGCAGTTGATGAAGTGAAAAGTGAAAATTTA[G/A]GCTGCAATCCTGGGCTGATCTCCCTGCTGAACTCTTAGAATTAATCTTGTCCCGATTGATCCTAGCAGATAACATCCGTGCTTCTTCTGTTTGCAAGAGA
PHG9	86			missen	Mu_g1	kunitz-type	Trypsin and	ACCAAACTTGCCCACTCACTATCGTTCAAGACTATTCAGTTTCTATTGGTGACACAGTCAAATTCAGCATACCCCCTGTTACAGGCCCTGATGCAAGGT

_Scaffold_1303	1			se_variant	0421.t1	trypsin inhibitor-like 2 protein [Arachis duranensis]	protease inhibitor	T[A/C]TCTTTACAGGTACCACTGATGATATCAGTAAATCTTCTTCATACATCAATCTTTTTGGTAACATGGTTCTAAATTGCAGTTACTGTCAGTTGGAAACTG
PHG9_Scaffold_134	23002			missense_variant	Mu_g02881.t1	hypothetical protein PHAVU_009G163700g [Phaseolus vulgaris]	NA	TCCAGAATCCAGCACCCGGAGAAGGTGGTTCAGAAGAAGCAAACCCTCGGCGAGGAGCACAGTCTTGGCGCTTTCAACGGTAGAGACTTTGGCGATGGCA[A/G]GACGGTTTTCTCCTTCATGTCGGAGGGGGAATCGAGCAGGCGCACGAGCACGGGAACAACGCCCTGGGCCACAGCAATTGTAACATTTTTGTCATCTTCA
PHG9_Scaffold_136	7941			missense_variant	Mu_g02919.t1	hypothetical protein PHAVU_007G261300g [Phaseolus vulgaris]	NA	CAGACTATTAACCAGTAACAACAAAGAAAAACATGCCTGCTCCTTAAATATTACAGTAGCAGCATCTAGAAGAAATTCTCGAGCAAGTTCAGGACTCTTC[G/T]ATGCTTTTGAGGACGGGAGCATTCTCCATCAAGCTCATTTCCATCTTCATCAATCAGATCATCATCCTAGAAACAAATGTTGCATAATTAACAAAGGAAC

PHG9_Scaffold_139	9204			missense_variant	Mu_g02950.t1	hypothetical protein PHAVU_001G264500g [Phaseolus vulgaris]	Kinesin motor domain	TCTCTTTCTGCTTTTAAAGCTGAAATTTCAGCAGCTAGGGCATTTATGAACTTAGACTCTGCCCCTCTTACTCCTGCTCTGGAAGCCGCTTTCTTAACAT[C/T]TCAATTCCATCTTGGATCTGCCTATGCCTTGCAAGCAAATGGATGTGTTTCTCTTCCAAATCTGCATATTGCTCTAAAAGTCTTGCATGCCCTTCGATAG
PHG9_Scaffold_14	0615			missense_variant	Mu_g00233.t1	hypothetical protein PHAVU_002G322100g [Phaseolus vulgaris]	GatB/GatE catalytic domain	AGATCTCACAGCACCACTTGGTCAACTTGCATTTTGAAATTGTGGGTAACCAGAAGCTCTAGTATTGTACTGATTGAGCATGACTGCATCTGTGTTGTTT[A/C]ACCTTGCATTTGAACGGCACCGAATGAATTTCTTCTAGGTTGAGGTAATATTAAACCTTGATTATTGTTCATGCCAAAGTTGTAATTCTGGGTGACAGGA
PHG9_Scaffold_14	1631			missense_variant	Mu_g00233.t1	hypothetical protein PHAVU_002G322100g [Phaseolus	GatB/GatE catalytic domain	AAATACAGATATTTGATGGATAAGAAAAATCAAATAATTGTTGAAGAATCATACTAGCAAGTCCAATTTGATCGATAGCCTTAAGAAATAGTCCATGCAG[T/A]TTTCTGACCATACTACTCGCTTCTTTGTCGAGGGTGGATAATTAATCTTTGATTCATCATCAATAGTTTTTCTGGAAGAGCTATTGCTCCTTTCAACAAC

						vulgaris]		
PHG9_Scaffold_14	2100			missense_variant	Mu_g00233.t1	hypothetical protein PHAVU_002G322100g [Phaseolus vulgaris]	GatB/GatE catalytic domain	ACATTGATTTTCTCCTTATTAACCAATTCTAGGAATTTATAACCATCCATCATTGGCATATGAACCTCTATCAGTATCAAATTAATATGAATTTTGCCTT[C/T]CGCAACACCTCAGCAGCAAGTAAAGATTCGGAGCATGCTGTAACTGTTAAAAAAAAGTTACATTTTTTTCTTCTAAGAAATAAAAATCAACTAACTTTTG
PHG9_Scaffold_1426	1841			missense_variant	Mu_g10869.t1	PREDICTED: 1-phosphatidylinositol-3-phosphate 5-kinase FAB1B-like [Glycine max]	Phosphatidylinositol-4-phosphate 5-Kinase	TGATTCTCAGAAAAGTTTTGGATCGATAGAGGAGATGATATTTTCCATGTCTGGGTCTCGAAGTTCATCATTATTTGACCCAATGTCATATACTAAGGCT[A/G]GCATGCCAGAGTTTCCTTTGGAGAAGACGGTCCTCTTGGCAAGGTAAAATATTCTGTGACTTGTTACTATGCCAAGCGTTTTGAAGCCTTAAGAAGGGTG
PHG9_Scaffold	9671			missense_v	Mu_g1092	PREDICTED: probab	Protein phosphatase 2C	TAGCAATGACACGGGCATTTGGAGATGGAAAACTGAAGAAGCACATTTCGGCAGAACCAGATGTGACCATTCGGAAGATTGATGAAGACACTGAATTCAT[A/G]TTTTGGCAAGTGATGGTCTGTGGAAGGTACGAGAATATTGCAT

fold_1437	1			ariant	1.t1	le protein phosphatase 2C 39 isoform X4 [Glycine max]		GCATCTTATAATTACTGATGAGCACATCTTGGCTGAAGTGGAGCAAGCATAAATCTA
PHG9_Scaffold_144	1347			missense_variant	Mu_g03044.t1	hypothetical protein PHAVU_002G115400g [Phaseolus vulgaris]	NA	TCTTCTCAGCATGCAATGCAGAAGTCTTGGCTTTCACTTCATCATCAGAAAAAGGGTCGGATTCTTTGTTCGGTTCGCAATGAAGCTGCAGTGCAATTTT[A/T]CAACCGGGGAAGCAGGAAGGGTCACTTCTGTGTTCTCAACAACAGTAGAAGGCTCTCCTTCGAGTTTCTTTATGGCAACTTCGATGTTTTCTGCAATATA
PHG9_Scaffold_1445	9613			missense_variant	Mu_g10937.t1	uncharacterized protein LOC106754701 [Vigna radiata var.	SAM domain (Sterile alpha motif)	GAGCTTCTCATGGATTCCATGCGCATTAAATCATCAGAACTTCTAGCTGAGGGAATTTGCCTCAACAAACTTGTTTCCCGTGATTCTGGCATGTGCTGCT[T/C]GAATTAAAACTATTTGTCAGAGGGTGAGTTGCCTTCGAAAGTTTTTCACGGAGATCCATATGCCTCTTACTACCATTGCTGTCTGCATGCTTAGATGCAC

						radiata]		
PHG9_Scaffold_145	8967			missense_variant	Mu_g03055.t1	hypothetical protein PHAVU_008G082500g [Phaseolus vulgaris]	Leucine rich repeat N-terminal domain	GGGTGCATTGGAAGAAGGCAAGGTGGACCAATGTGTTGATGGAAGGCTCCTTGGTAACTTTGCAGCAGAGGAAGCAATTCCTGTGATAAAATTGGGGTTG[A/G]TTGTGCATCACAAGTGCCATCAAACCGTCCAGATATGGCTGAGGTAGTCAACATACTAGAATTAATCCAATGTCCTTCAGAAGGACTAGAGGAATTATAA
PHG9_Scaffold_145	9015			missense_variant	Mu_g03055.t1	hypothetical protein PHAVU_008G082500g [Phaseolus vulgaris]	Leucine rich repeat N-terminal domain	CCTTGGTAACTTTGCAGCAGAGGAAGCAATTCCTGTGATAAAATTGGGGTTGATTTGTGCATCACAAGTGCCATCAAACCGTCCAGATATGGCTGAGGTA[G/A]CAACATACTAGAATTAATCCAATGTCCTTCAGAAGGACTAGAGGAATTATAATGAGTTTTGTTTCAGTCTTTGAGAAACAGCATATGTTGCAGTACCTGG
PHG9_Scaffold_145	64990			missense_variant	Mu_g03068.t1	PREDICTED: probable serine/threonine-	Protein kinase domain	TTCTGCGACCCGGGCCACGAAGAGGAGAACGACGGAACCGAACGCAACAAGTGCACCACATTATTGTACCGTGGGGGCGAAGAAGCAGAACCCGGTTTGG[C/T]AAACCGGTTTGTACCCGCCACGGATGGAGTCTTCGTGCGGGTGCATGCTCGGATCGGGTCACAGGTGCCCGAGACTGACTCGGATCAGGTCGTGCCCGC

						protein kinase WNK5 [Vigna angularis]		
PHG9_Scaffold_145	65065			missense_variant	Mu_g03068.t1	PREDICTED: probable serine/threonine-protein kinase WNK5 [Vigna angularis]	Protein kinase domain	GGCGAAGAAGCAGAACCCGGTTTGGCGAAACCGGTTTTGTACCCGCCACGGATGGAGTCTTCGTGCGGGTGCATGCTCGGATCGGGTCACAGGTGCCCGA[G/C]CTGACTCGGATCAGGTCGTGCCCGCACGAACGGCGGAGCATGCAGCTCCAGCGGTCGATGATGTTGGAGGAGATTTACAAGTACAAAAGACGCTTCTTCA
PHG9_Scaffold_1454	0350			missense_variant	Mu_g10982.t1	hypothetical protein KK1_003069 [Cajanus cajan]	MlaD protein	TTTCCAGTTCAACATTGGGAGATACTAGTCTTTGGGATTTAGGACATTTGATCAAAGATGATTAGGAACTCTCCATTGCATGCTTCAACATTGCCAAGTG[T/C]TTATCTTCGTCTCGGATTACTCTACATGGAAGTTCAGTAAATTGTATGCCCTGCCTTCCTTTTAGACCCCAGAGAAAAATCAGCACCATAAGAGCTACAT
PHG9_Scaffold_	7355			missense_variant	Mu_g10991.t	PREDICTED: uncharacteriz	Plant transposase (Ptta/En/S	TGCATCACTAATTTTGTGTTAATTGCAGATGGCAGATAAAGATCAAAATAAACGAAAAACTTCCAAGAGTCATCCAACGAAACAACATGCTACACATCTA[G/A]GCCAATGACTTCTCAAAATCCATCTTATGCTTCGCAAGCAATAAATTCAATGGAAGGGGTACACTCACCTGCTGGGACATCTGATGTATCGC

1457				t	1	ed protein LOC106794106 isoform X1 [Glycine max]	pm family)	CTCCACCT
PHG9_Scaffold_1457	7420			missense_variant	Mu_g10991.t1	PREDICTED: uncharacterized protein LOC106794106 isoform X1 [Glycine max]	Plant transposase (Ptta/En/Spm family)	AGAGTCATCCAACGAAACAACATGCTACACATCTAGCGCCAATGACTTCTCAAAATCCATCTTATGCTTCGCAAGCAATAAATTCAATGGAAGGGGTACA[C/G]CACCTGCTGGGACATCTGATGTATCGCCTCCACCTTCAATGGAAGTGGTACGCTTACCACCTTCCTCACAACCAACAAATTCAGATGCAGGACGTACTGG
PHG9_Scaffold_1457	7707			missense_variant	Mu_g10991.t1	PREDICTED: uncharacterized protein LOC1	Plant transposase (Ptta/En/Spm family)	ACTTATTTGATATTGGCTTTTTAAAATTGTGACATTCATGCAATATTAGCAGATTCTTACCTTCTCGCCCTGCTGCTAATGTGATTGGGGATATCCTCAA[G/C]GGCATTTTACTGATCCCTGGCCATCATGGAAGAAGATACCAATTAGCACAAGAGACTCGTGGTTTGAAGAGTTTCTAGTTAAGTTTCATATCTACATTAT

						06794106 isoform X1 [Glycine max]		
PHG9_Scaffold_151	3595			missense_variant	Mu_g03162.t1	WAT1-related protein At4g30420-like [Vigna radiata var. radiata]	EamA-like transporter family	AGTGAATATTGGAAGCTGGAGATCCATAGCGAAGATAGTAGGGACAGTGATATGTGTGAGTGGAGCAGTGTCCATGGCTTTGTTGAAGGGTCAAAAGCTA[C/A]AAATTCAGAAAACCTGCCATCAAAATCCATCATCATGGCCTCATCAGAAACTGACACTTGGTTGCTGGGTTGTCTGATTCTCACTGCATGCTGCTGTGCT
PHG9_Scaffold_151	3596			missense_variant	Mu_g03162.t1	WAT1-related protein At4g30420-like [Vigna radiata var. radiata]	EamA-like transporter family	GTGAATATTGGAAGCTGGAGATCCATAGCGAAGATAGTAGGGACAGTGATATGTGTGAGTGGAGCAGTGTCCATGGCTTTGTTGAAGGGTCAAAAGCTAC[T/A]AATTCAGAAAACCTGCCATCAAAATCCATCATCATGGCCTCATCAGAAACTGACACTTGGTTGCTGGGTTGTCTGATTCTCACTGCATGCTGCTGTGCTT

PHG9_Scaffold_1560	337	CA	AA	missense_variant	Mu_g11399.t1	hypothetical protein PHAVU_007G006600g [Phaseolus vulgaris]	Transketolase, thiamine diphosphate binding domain	AATGGCTGCACGAATTTCATCATACCCGGTATTTCCATTCTTCACCCAAATCACATGCCATCCAAGTGCCTCAAAACGTTGATCAACATTCTCAGTGAAT[GCA/GAA]AATTTCAGTGTCACCATCAATGGAAATGTGGTTGTCATCATATAACGCAATAAGCTTCCCCAGACCCCAGTGACCGGCAAGTGAGCATGCTTCATTCGAA
PHG9_Scaffold_1587	4947			missense_variant	Mu_g11553.t1	hypothetical protein PHAVU_011G110500g [Phaseolus vulgaris]	NA	GTTGTAATTGTATGAGTAGGCACTTGAAAGTGAGTTAAGTCTAGTGGTGGGGCAACTTGGTTTTCTGCATAGTTGTGTGAAGCCAAATTTGGATTCTCTA[T/G]AGAGAGGCTGTGGCAGCAAGGTTTAACAACCCTTGGTTCATCAAAACATGGGCTCCTAATAAGCCTTGCATGCTAAGGGAGGGGTTCAACACTGACCTTA
PHG9_Scaffold_159	23220			missense_variant	Mu_g03347.t1	hypothetical protein PHAVU_005G145800g [Phaseolus	Multicopper oxidase	CTTTGTCCAACTATGCTGAAGTTGTACACATAACTCTGACCACTTTGTATGGGACATTGAGTTATGTACGATGGTCCATCCGACCATCCACTCCGTAGCT[G/T]TTCACTCCGTGCCTATAAATATGTTGTCATACCACAGACAAAAAAAAAAAAAAACTAAAATATCCATCGCATGCATGCACAACATGAGGTAAATTGGTTC

						vulgaris]		
PHG9_Scaffold_1591	1977			missense_variant	Mu_g11587.t1	hypothetical protein PHAVU_008G117800g [Phaseolus vulgaris]	Legume lectin domain	AGGCCAAGCATGCGCCAAGTTGTGCAGTACTTGGAGAGGGATGTGCCTCTGCCAGACCTGTCTTTGCTTACCTTATCTTCCACTGGCTTAACTTTTGGTC[G/T]CATGAAGACTTTCAGGATTGTCCAATGTCTTATCCTTCATCTATGGATAGGCCAATCTCCCATACTTCTTCAATTGCTGAATCACTTCTCTCTGGGGGGC
PHG9_Scaffold_1604	591			missense_variant	Mu_g11649.t1	hypothetical protein PHAVU_006G090200g [Phaseolus vulgaris]	Ferric reductase NAD binding domain	ATTTCCACAGACTTGGATGTATCTTGCAATTCCTGTGATACTATATGCATGCGAGCGATTGCTTCGTGCTTTTAGGTCTGGCTACAAAAGTGTCAAGATT[T/A]GAAGGTACAACATTAACACCGAAGAATTCACCTTCCCACTTTAAGTTTTAAGTAAATTCAATTTTGCATTTTTGCTATTAATGACGATGGTACACTAAAA
PHG9_Scaffold_1606	7989			missense_variant	Mu_g11659.t1	VAN3-binding protein [Vigna radiata	Auxin canalisation	TGCCACTGAAATAGTGGCATGCAGCTGAGCAGTATGGGTTCTGTTTTCTTCTTTCTTCTTTTCTCTTCGGTCCTTCAACCATCTTCCAACAGTTTTAGGT[C/A]TGAGCATGCTGTGCCATTGCCAATAGTTGCGCTGGCTCGTCCACCATTGAATAAAGGGTGAATGGAATTATTTGCCCGAAAAAACTGCTTAGACATTGAT

						var. radiata]		
PHG9_Scaffold_1606	7994			missense_variant	Mu_g11659.t1	VAN3-binding protein [Vigna radiata var. radiata]	Auxin canalisation	CTGAAATAGTGGCATGCAGCTGAGCAGTATGGGTTCTGTTTTCTTCTTTCTTCTTTTCTCTTCGGTCCTTCAACCATCTTCCAACAGTTTTAGGTCCTGA[G/C]ATGCTGTGCCATTGCCAATAGTTGCGCTGGCTCGTCCACCATTGAATAAAGGGTGAATGGAATTATTTGCCCGAAAAAACTGCTTAGACATTGATAAGGA
PHG9_Scaffold_1623	2004			missense_variant	Mu_g11740.t1	hypothetical protein PHAVU_008G111900g [Phaseolus vulgaris]	NA	AGAACAAGAGAAGAACCGGGTACAGATGTTGTTGCAAGGCATGCAGAATGTAATGACTGCTTACAATGCAAGTGGAAACCTCCCTGCTCCAGCGCCCGCT[G/A]AAAATCTGCAGTTGTAGCGATTCCTCAACCAGCATTTACTCTCAAATCTCAACCAGGTACAACCTTTGCCCCTACCTTAGTTGTTTTCTTAATATATGA
PHG9_Scaffold_1667	4034			missense_variant	Mu_g11994.t1	hypothetical protein VIGAN_05178900 [Vigna	Region in Clathrin and VPS	TGGAGGTTATTCCCCTTCCCCGTCTCCAATGCAAGGAATGGGTGGCGGCTATACACCTCCCCCACCGCCACCACCTATGGGTGGAATGGGGATGCCACCA[A/G]GCCTCCTTTTGGCATGCCTCCAATGGGGGGCGGCTACTGATCTCCCTAATCACATCAGAGGAGAGAATTTTGCAATTTCTTTTACTGGTGAAGCTAGATA

						angularis var. angularis]		
PHG9_Scaffold_171	3628			missense_variant	Mu_g03578.t1	hypothetical protein PHAVU_002G057800g [Phaseolus vulgaris]	Agenet domain	ACAGTGTACAACGATTTAGACAGTCAATATATCAAGATTAAACCCTTATAATGTACCTGATTTGTTGACAGGTATAGCATGGGTTGATACTGCAGAAGTT[T/C]CAATTCAGTTTCCATGCTACCTTCACTAAGACATGATGAAACAACTACTTTATCTACAAGATGGGATTGCTCAACATCACATTGCATGCAGCTAGAATCA
PHG9_Scaffold_1718	6037			missense_variant	Mu_g12210.t1	putative disease resistance protein At3g14460 [Arachis duranensis]	NA	CGTAAGATTGGTCATATAATTAGGAAGTGACCTAAGATTTTTGCAATTGGAGATTGTAAGTGATCTAAGATTGGGAGAACTCAATCCCATTGTTGGGAAG[G/A]CACTAGATTGGAGCAATCCCGAATTTCCAAAGACTCAAGTGATAGATCATTATCTTGGCATCCCTCTATAGAAAGGGACTCTAAATTGCAACAATCCCAG
PHG9_Scaffold_	25905			missense_varian	Mu_g03682.t	PREDICTED: uncharacteriz	WD domain, G-beta repeat	TTTGATGTGCTGCCAAACAAAGTACAGCACCTGTATGTCCTGAGAAGTATTGTTTGCATGCAGTTGGCTTTTCATCAGGATTGGAACTAGCATCATCCAA[C/G]AAATCCCTTGAAACAGATCAAATCTTACAACCTCTATATCTCCACTTATAAAACCAAATACAACAGCATAAGGAGTAAAGAGGTTCTCAGAGA

172				t	1	ed protein LOC108347372 isoform X1 [Vigna angularis]		TAATCAT
PHG9_Scaffold_1736	6259			missense_variant	Mu_g12239.t1	PREDICTED: microtubule-associated protein RP/EB family member 1B-like [Glycine max]	EB1-like C-terminal motif	TAACAACCAAAAAGAACTTTTATTTTTAATTTTAACAATGTGGGCAAGAACAAGAACCAAGAGGATTACCTCCTCAATGCGAGAGAGGTTAAGTTGAAGG[C/T]ATTGTTGATCCAATTGAGAATCTCATTTCTGCCGACAAAGTATGCGCTATCCATCATCCCTATACTCGTGGCCATTTCTTTCCACAAAGAATGAAGACCA
PHG9_Scaffold_1736	6271			missense_variant	Mu_g12239.t1	PREDICTED: microtubule-associated	EB1-like C-terminal motif	AGAACTTTTATTTTTAATTTTAACAATGTGGGCAAGAACAAGAACCAAGAGGATTACCTCCTCAATGCGAGAGAGGTTAAGTTGAAGGCGATTGTTGATC[C/G]ATTGAGAATCTCATTTCTGCCGACAAAGTATGCGCTATCCATCATCCCTATACTCGTGGCCATTTCTTTCCACAAAGAATGAAGACCAAACAGCAACAAC

						protein RP/EB family member 1B-like [Glycine max]		
PHG9_Scaffold_1736	2630			missense_variant	Mu_g12243.t1	organic cation/carnitine transporter 3 [Vigna radiata var. radiata]	Sugar (and other) transporter	ATTCTCTCTTCCAGCACTCACCAGCATTGGACTGAACGCGCCACCAAACACCACTGCTAACCTTGCCATAGACAGTGCTGAATTTCTTACACAAGTTGGG[A/G]AAGTTCCGTGGTGTAAATGAGGTACACGTTAAACGAAGAACAAGCACTGAAGAATGATATCAACTCGAACCCTATTTGCAGATTTTTCATTGGCTTAACT
PHG9_Scaffold_1771	8083			missense_variant	Mu_g12489.t1	hypothetical protein PHAVU_011G124300g [Phaseolus vulgari	RPN1/RPN2 N-terminal domain	AAATGGCAAAGGGGAAGAACGACTGGAAAGAAGAAATGAAACTCAAAACAAACCTGCATTGCCAAAACGAGGAAGTAGAGAACATAGTGATATTTCCCCA[A/T]ACAATAGCTTTCATATCTAGGCATGCGTGCAACATGGTAATTAGTCCAGCAAGCGCTGTCCTGTAAAAATGATACAGAATTTGAATTAGAAAATATTACA

						s]		
PHG9_Scaffold_178	8064			missense_variant	Mu_g03786.t1	PREDICTED: uncharacterized protein LOC106773219 [Vigna radiata var. radiata]	Cellulase (glycosyl hydrolase family 5)	ACAGAGTCACATATCCAATTAATTGGTTGCCTTGTCCATATATCTTTCATTTTGAGTACTCCAACTCCAGACCATGAATATAAATGTATCTCAAACACCA[G/T]TTCTTACCCAAATTTATCTTCAATGGTTTATTCTTTAAATAGTGCAAGTCAGAATCATAACCTATACCTGAAATAACCACAAGCACATTTGGGTTTGTCT
PHG9_Scaffold_178	8172			missense_variant	Mu_g03786.t1	PREDICTED: uncharacterized protein LOC106773219 [Vigna radiata var. radiata]	Cellulase (glycosyl hydrolase family 5)	CCCAAATTTATCTTCAATGGTTTATTCTTTAAATAGTGCAAGTCAGAATCATAACCTATACCTGAAATAACCACAAGCACATTTGGGTTTGTCTTATGTA[C/T]GCTAATGCTGCTTGGGTCATATACTTGTACCAATCAGGCAAGTTTTGGCGTGGACCACGCAATTCATTCCTCAAACTCATTGCCACAACCTATTAGTGAA
P				m	Mu	PREDI	Cellulase	AACAATCTCTTTCAAAGGCCTTTTATCAAGACCCTCAGGGATCATTGGTT

HG9_Scaffold_178	8891			issense_variant	_g03786.t1	CTED: uncharacterized protein LOC106773219 [Vigna radiata var. radiata]	(glycosyl hydrolase family 5)	GGAGGTGACCAGCCCAATTGGCACATGCCAACTTGGTTCGTTGTCCTGTG[G/A]TTCATCTATGATCCATCTCTTATGTGTTGATAAAGGATATGTATTGGAATATGATGTAGAGATGAATAAAAGGAGAAGAAGAAAACCAATTCGTGAAATT
PHG9_Scaffold_178	8934			missense_variant	Mu_g03786.t1	PREDICTED: uncharacterized protein LOC106773219 [Vigna radiata var. radiata]	Cellulase (glycosyl hydrolase family 5)	ATTGGTTGGAGGTGACCAGCCCAATTGGCACATGCCAACTTGGTTCGTTGTCCTGTGGCTTCATCTATGATCCATCTCTTATGTGTTGATAAAGGATATG[T/C]TTGGAATATGATGTAGAGATGAATAAAAGGAGAAGAAGAAAACCAATTCGTGAAATTATTCTAAACATCTTCGTTTTGAACGGAAAATAAATTTTTCTAA
PHG9_Scaffold_	8948			missense_varian	Mu_g03786.t	PREDICTED: uncharacteriz	Cellulase (glycosyl hydrolase family 5)	ACCAGCCCAATTGGCACATGCCAACTTGGTTCGTTGTCCTGTGGCTTCATCTATGATCCATCTCTTATGTGTTGATAAAGGATATGTATTGGAATATGAT[G/A]AGAGATGAATAAAAGGAGAAGAAGAAAACCAATTCGTGAAATTATTCTAAACATCTTCGTTTTGAACGGAAAATAAATTTTTCTAAGATGGAGA

178				t	1	ed protein LOC106773219 [Vigna radiata var. radiata]		AGAAAT
PHG9_Scaffold_1812	1464			missense_variant	Mu_g12691.t1	hypothetical protein PHAVU_006G017100g [Phaseolus vulgaris]	Lipoxygenase	TTCTAACAAGATATAAAAGCAAGTGATGCAATAAGCAGTTTTCCAGCATTATCAAATTTAAAAATAAGCACTTGTAATAAGTTCTTACTTTTAATTGTTT[T/C]GGGGTATCAAAGCGCAAAAAGTTCTGGCTATCATTAGCAACCTTGATCAACTTGGGAATGGCACTCTGGAGGAAATTTAGACTATTGTCTTTAAGAGTTG
PHG9_Scaffold_1812	1526			missense_variant	Mu_g12691.t1	hypothetical protein PHAVU_006G017100g [Phaseolus vulgari	Lipoxygenase	AATAAGCACTTGTAATAAGTTCTTACTTTTAATTGTTTTGGGGGTATCAAAGCGCAAAAAGTTCTGGCTATCATTAGCAACCTTGATCAACTTGGGAATG[G/A]ACTCTGGAGGAAATTTAGACTATTGTCTTTAAGAGTTGGTAAGTGAAACCCTTCTTTGTAGATTGTGTCTATGTCTTCGAAGCTTCTAAATCCAAGATCT

						s]		
PHG9_Scaffold_1842	81263			missense_variant	Mu_g12862.t1	hypothetical protein PHAVU_010G014100g [Phaseolus vulgaris]	Transferase family	CTCGGTGAAAATAACTCCCTCGTCGTTGCACTCAACTATTGAATTGCCTCTCAGGCTTCCACAAAAGTGAGGGTAGAGAGTGAGGACTTGTGACAAAGAA[C/G]CTTCAACTTGTCAGATATTGTTCTGAAGTCTGTGGCATCGGAAGCAGAGTAGAAGAAGAGAATAGGGATGTACAATGGAGGAGCCAATTGATCGAGAAGG
PHG9_Scaffold_1842	81287			missense_variant	Mu_g12862.t1	hypothetical protein PHAVU_010G014100g [Phaseolus vulgaris]	Transferase family	GTTGCACTCAACTATTGAATTGCCTCTCAGGCTTCCACAAAAGTGAGGGTAGAGAGTGAGGACTTGTGACAAAGAACCCTTCAACTTGTCAGATATTGTT[C/T]GAAGTCTGTGGCATCGGAAGCAGAGTAGAAGAAGAGAATAGGGATGTACAATGGAGGAGCCAATTGATCGAGAAGGGAAAGTTTGAAGTGTCGAAGATTA
PHG9_Scaffold_1853	623			missense_variant	Mu_g12896.t1	hypothetical protein PHAVU_004G175900g	Protein kinase domain	TTGATTCTGTGCATGCTGGCAGTAGCCTTCTTGCTGGCTTTGTTTATAGCAATCTTGATTACTAAACTTTACAGAAGAAGGAAACAAGGATTGGATAATT[C/T]TGGAAACTCATTTCATTTCAAAGGCTGAGTTTCACTGAATCAAACATAGTGTCATCAATGACAGAACATAATGTTATTGGAAGTGGTGGATTTGGCACAG

						[Phaseolus vulgaris]		
PHG9_Scaffold_1867	2100			missense_variant	Mu_g12943.t1	Hypothetical protein	NA	TAGCAGCCCAATCAATCCTATAACTATTACTAGCTATTATCTGGGCCCCATCAGTGTCAACTACTCGAAGTTCAACTTTGTTGGCTTGTCCACAAATTTG[A/C]ATTGGGGAACTGAGATGAGATGGTGTCCAGACTTCCCATCATTCCCTTTCTGTTTTGGCTTTCTTACCAAGATTCCAAAATCTTGCATGCATGCATCATC
PHG9_Scaffold_2005	18813			missense_variant	Mu_g13407.t1	PREDICTED: serine/threonine-protein phosphatase 4 regulatory subunit 3-like [Vigna angularis]	Component of IIS longevity pathway SMK-1	TATCTGATTTCGCATGCAAGCAATCAGAAAAGCTTCTAGAGTCATCAGCCTCTTTCTCTATGTGATTGTCTTCTGCTGCATTGTTGCTATCATCGGAATT[G/T]TTCTAGAATTACTTTGTACATCTCCCTCATTATTTTCACTTGAGCCTATCCTCCCCTCAACTGTTCGTGCACTTGTCTGAATGCTGATTGATGATGTTTT
PHG9_Scaffold_2040	07154			missense_variant	Mu_g13593.t1	hypothetical protein PHAVU_001G155900g	PDDEXK-like family of unknown function	GAGCCATTTTGCTTTGACGTATTCTACTTTTCTCCAAGGAGGAACGTGCATGCACCTTTTCTTCAGACTCAGTTTCACTGCCTCCGAAGCGATGGTCACA[G/A]ACTCTGCAACCGATCGCACTTACCGACAAAGATGTAAGGAATGGTTTGGAGGATTGCTTTGTATGTCTTGGTGGGTCGAGCAATCTCGAATTCTGATCTG

						[Phaseolus vulgaris]		
PHG9_Scaffold_2080	176			missense_variant	Mu_g13725.t1	PREDICTED: ankyrin repeat-containing protein At2g01680-like [Vigna radiata var. radiata]	Ankyrin repeats (3 copies)	AGCTACTTTTAACAGTATATTTCGCACATCACTCATTGAATCCCTGTTGGGTTTGTATCCGAATAACTCTTTCAATTCTTTCATTTGTGGCCAGTACCGT[G/A]TCTTCTAGGTAGGTAGGTGCTTTGTTCACGAGTAGAGGTGTTTGTTTTGGAGGAACTTGATTATCATGAACGATCTCCAATTCCAAAGGTGCTACTGATT
PHG9_Scaffold_2080	201			missense_variant	Mu_g13725.t1	PREDICTED: ankyrin repeat-containing protein At2g01680-like	Ankyrin repeats (3 copies)	ACATCACTCATTGAATCCCTGTTGGGTTTGTATCCGAATAACTCTTTCAATTCTTTCATTTGTGGCCAGTACCGTGTTCTTCTAGGTAGGTAGGTGCTTT[G/C]TCACGAGTAGAGGTGTTTGTTTTGGAGGAACTTGATTATCATGAACGATCTCCAATTCCAAAGGTGCTACTGATTGCCCTCTGTTGTTTGATATTCTACC

						[Vigna radiata var. radiata]		
PHG9_Scaffold_2080	209			missense_variant	Mu_g13725.t1	PREDICTED: ankyrin repeat-containing protein At2g01680-like [Vigna radiata var. radiata]	Ankyrin repeats (3 copies)	CATTGAATCCCTGTTGGGTTTGTATCCGAATAACTCTTTCAATTCTTTCATTTGTGGCCAGTACCGTGTTCTTCTAGGTAGGTAGGTGCTTTGTTCACGA[G/T]AGAGGTGTTTGTTTTGGAGGAACTTGATTATCATGAACGATCTCCAATTCCAAAGGTGCTACTGATTGCCCTCTGTTGTTTGATATTCTACCAACTTCAT
PHG9_Scaffold_2231	594			missense_variant	Mu_g14179.t1	hypothetical protein PHAVU_002G333600g [Phaseolus vulgari	NA	CATAGTTTACGATGTGTCTTTGACTTGATTTGTTTTAATTTGCTTGATTTTTACTTGAGTCGTTACTTACAAAAATTGAGCGAGTCTAAGATTGCAGAAT[C/G]CAAAGAGACTTATCAGAGGCAAAATCTCTGGTCGAGGAAGCAGAACGCTCTCTATTGCTAAATGTGGGAGGTCCTGACGGTGGCAGTACATCCATGAGAA

						s]		
PHG9_Scaffold_2231	609			missense_variant	Mu_g14179.t1	hypothetical protein PHAVU_002G333600g [Phaseolus vulgaris]	NA	GTCTTTGACTTGATTTGTTTTAATTTGCTTGATTTTTACTTGAGTCGTTACTTACAAAAATTGAGCGAGTCTAAGATTGCAGAATCTCAAAGAGACTTAT[C/T]GAGGCAAAATCTCTGGTCGAGGAAGCAGAACGCTCTCTATTGCTAAATGTGGGAGGTCCTGACGGTGGCAGTACATCCATGAGAATGAAAAGTGAAGAAA
PHG9_Scaffold_2263	1087			missense_variant	Mu_g14261.t1	PREDICTED: butyrate--CoA ligase AAE11, peroxisomal-like [Vigna angularis]	AMP-binding enzyme	ATCTCTTGGCAACATACAGAGGCACAAATATCTTTCTTGCTCATATTTCATCATAGCCTCTAACATGGATAACCTTCCAAAATGCCAAGCAAACTACACC[T/G]ACTCACCCCTTTAACGTTTCTGTCAAGAGCTGCTGCATGCTATGCAAACAGAACCTCAGTCATCCATGAAGGGACCCGTTTCACTTGGGCACAGACTTAT
PHG9_Scaffold_	6458			missense_variant	Mu_g14479.t	uncharacterized protein	EF hand	GTCCTTGTTGGTATCAGCTTTTCTGAAAGCACAGAAGGCTCTCCAAGCAGGGAAGAAGGCACCTAAATCTTTGAGAGCATGCTTGAGCTCATCCATGCTG[A/G]ACAACCATCTTTGTTTCTGTCAACATTCTTCAATGCTTCCATTATTTTATAGAAGGTGACAATATCAGAAATGGGCTTTCCTTGTATACCTGAC

2327				t	1	LOC106764438 [Vigna radiata var. radiata]		ATGATT
PHG9_Scaffold_2331	653			missense_variant	Mu_g14495.t1	pentatricopeptide repeat-containing protein At2g27610 [Vigna radiata var. radiata]	DYW family of nucleic acid deaminases	TTTCAGAGCATATTCCAACCTGCCACATTTGGAGTATAAACTACAAAGCGCATTGCCAACATTGGTGTCAAACTCAATATGGTATTTGATTATGTAAGCA[T/A]GAATTGATCTCCTAACTTAAGAGAGTATAAAGAAGCACAAGCATTTAGAACAACAGCAAGAGTGTAAGCAGAAGGGAAACTTCCTGCATGCAACATCTCT
PHG9_Scaffold_2331	655			missense_variant	Mu_g14495.t1	pentatricopeptide repeat-containing protein At2g27610	DYW family of nucleic acid deaminases	TCAGAGCATATTCCAACCTGCCACATTTGGAGTATAAACTACAAAGCGCATTGCCAACATTGGTGTCAAACTCAATATGGTATTTGATTATGTAAGCATG[G/C]ATTGATCTCCTAACTTAAGAGAGTATAAAGAAGCACAAGCATTTAGAACAACAGCAAGAGTGTAAGCAGAAGGGAAACTTCCTGCATGCAACATCTCTTG

						[Vigna radiata var. radiata]		
PHG9_Scaffold_2346	0653			missense_variant	Mu_g14542.t1	hypothetical protein PHAVU_004G166700g [Phaseolus vulgaris]	NA	GGCTGTCTCGCTGCGGAGTTGTAAGAGTAAACCCTTGACATCACCAGAATTGTTCACAAGTCTTGTTAGTTCGAGTGAACAGGCTGAAACACCTACTTCG[A/T]TGAGATGTTCCACTTCTCCAAGATTCCTCCATTTTGTTGCATGCTAACCTGTTTTTCTGACAAACTAATGGCAGCATGTTTTCCCAGCTGAACTTGGTAA
PHG9_Scaffold_2354	7928			missense_variant	Mu_g14577.t1	hypothetical protein PHAVU_005G017000g [Phaseolus vulgaris]	Choline/ethanolamine kinase	ATCGCCATTTTGGAGAAGGAACTATCAGGCTCACACCAAAGGATAGGATTTTGCCACAATGATTTACAATATGGTAACATAATGCTTGATGAAGAGACCA[A/G]TCTGTGACCATCATAGTGAGTTTCTCTTTGTGCTCGTTTATGGGCGAAATAATATAATATTAATGTTTCAAGGGTGCTGCACTGTGGAAATAATTAGTTC
PHG9_Scaffold_	2746			missense_variant	Mu_g14599.t	uncharacterized protein	2Fe-2S iron-sulfur cluster	TTAATATTAATTTCAAGCAACATTTCACTGCATAGATAGCCCATGATGTACCATGACTATAACATGGCAGGTTGAACACGCAAGCGAGCCCTCGCATGCT[C/T]TGCAATAACATCAAGTAAGAAATCATTTAAGGCAAAAGGGAAAGTAAACTGAATCAATTATGAATATGCTTGGCCACTTCAGAAATGGCCATA

2361				t	1	LOC106765610 isoform X1 [Vigna radiata var. radiata]	binding domain	CTTGACA
PHG9_Scaffold_2361	71686			missense_variant	Mu_g14616.t1	PREDICTED: alpha-aminoadipic semialdehyde synthase [Vigna angularis]	Saccharopine dehydrogenase C-terminal domain	TTTCTTTCATTTTTTCTTGGTTTTTAAATTATTATCTTCCTGCCAGAGTCTGTTTAGTTGATCAGAGATTTAATGATATACATGACTTTTATGCAGCTGG[A/G]ATGATATTGCCAGATAGAGCTTATGCCTTCTTCTCCCATACTCATAAAGCACAGAAAGAGAACATGCCTTTGCTGGATAAGGTGTTAATCTTATATNNNN
PHG9_Scaffold_2378	9275			missense_variant	Mu_g14745.t1	PREDICTED: trypsin inhibitor DE-3-like [Vigna angularis]	Trypsin and protease inhibitor	TACATCCAAAGCATAACAAAGACACGTCTTTCACAAAGAGAAAGTGCAGAAATTTTCATGGACAGTGGTTATGCAGATGCAGTAGATGATGATGGTAGTT[G/C]TCAAGAACAAACTCGAATGGTTTGTCCTGAGTGATGACCAAAACCCTATTTCCTGCATCGTCGTTAGCAATTCCAACATTACCACAGAGGCTGCCGTCAA

PHG9_Scaffold_2378	9339			missense_variant	Mu_g14745.t1	PREDICTED: trypsin inhibitor DE-3-like [Vigna angularis]	Trypsin and protease inhibitor	GTGGTTATGCAGATGCAGTAGATGATGATGGTAGTTGCTCAAGAACAAACTCGAATGGTTTGTCCTGAGTGATGACCAAAACCCTATTTCCTGCATCGTC[G/C]TAGCAATTCCAACATTACCACAGAGGCTGCCGTCAAAATTGCAGAACAAAAACTTATAGCTGTTGGGTGAGGAAGCTCTGTGAATATAGAAGGAACCTCT
PHG9_Scaffold_2428	5366			missense_variant	Mu_g14856.t1	hypothetical protein PHAVU_010G000300g [Phaseolus vulgaris]	non-haem dioxygenase in morphine synthesis N-terminal	CTTTTTCTGTGCAACAAAAACACAAAAATGATGGCATGCCAAGATTGGCCTGAGCCAGTGATTCGAGTTCAAGCTTTGGCTGAAAGTGGTCTAAACACAA[T/C]CCAGAACGTTTCATAAAGCCTCAGTCCCAAAGGGTCACTAGTTACACCACTCATGCTTTAGACCAACCCAATTCCAATTCCTTTCACACTACTCCTCCTG
PHG9_Scaffold_244	0728			missense_variant	Mu_g04188.t1	PREDICTED: methylesterase 17-like [Vigna angularis]	NA	TGGAGGGTTGAGCATTACTCAGGCTTGTCGCAAGTTTGCAAATAAGATCCGTTTATCTGTTTATGTGGCAGCTACTATGCTCAAATTGGGATTCTTGACC[A/G]TCAAGATCTTAAAGATGTGAGTCCTTTTCATAAACTCTCTCATCTGCTCTGATTCTTTATGTGTACTGGTATGTTTTACTATTGAATATAATTCATTCTC
PHG9	27			missen	Mu_g1	hypothetical	Domain of	TCCCAGCGTGTCACACGGCAATGATTAACGATTCCGTGGGCACTGCACTGCACGTGGCGGTGGATTTGGACGAAGAAGGTGTGGTTCAAGACCTTGTG

_Scaffold_2461	9			se_variant	4912.t1	protein PHAVU_008G214000g [Phaseolus vulgaris]	unknown function	AA[T/G]CAATTATCGATTACAGCAGTGAAGCGAAGATCAAGGCTTTAGAAATGGGGAACGATCGAGGGGATACCCCTCTGCATGTTGCAGCTTCAAGGGGTTTCGC
PHG9_Scaffold_2574	926			missense_variant	Mu_g15377.t1	hypothetical protein LR48_Vigan01g070900 [Vigna angularis]	Terpene synthase family, metal binding domain	ACCCTCTCTTTTCCACTTTTAGAAATATGGACATTTGGTTTCAATGCAAAAGTAAAATGGTTTTTACTACAGTAGGCATGCTGAGGGATGAGCTTTATAG[A/C]GAGCATTTGTTTGGAGAAAATATAGGGTGATTTGGAGGCACAAAGATAGAGTGAGTGAAAGCCATAGGTGTAGTATATATTAAGGTTTGGTGTTGTTGAG
PHG9_Scaffold_2574	931			missense_variant	Mu_g15377.t1	hypothetical protein LR48_Vigan01g070900 [Vigna angularis]	Terpene synthase family, metal binding domain	CTCTTTTCCACTTTTAGAAATATGGACATTTGGTTTCAATGCAAAAGTAAAATGGTTTTTACTACAGTAGGCATGCTGAGGGATGAGCTTTATAGATGAG[C/T]TTTGTTTGGAGAAAATATAGGGTGATTTGGAGGCACAAAGATAGAGTGAGTGAAAGCCATAGGTGTAGTATATATTAAGGTTTGGTGTTGTTGAGGAAGA
PHG9	98			missen	Mu_g1	hypothetical	Terpene synthase	ATGGTTTTTACTACAGTAGGCATGCTGAGGGATGAGCTTTATAGATGAGCATTTGTTTGGAGAAAATATAGGGTGATTTGGAGGCACAAAGATAGAGT

_Scaffold_2574	2			se_variant	5377.t1	protein LR48_Vigan01g070900 [Vigna angularis]	family, metal binding domain	GA[G/A]GAAAGCCATAGGTGTAGTATATATTAAGGTTTGGTGTTGTTGAGGAAGATGAATGGGAACACAAGAGGAATGCAGAAAATAAGGAATTTGAGTCTATTTA
PHG9_Scaffold_2596	4643			missense_variant	Mu_g15453.t1	low affinity sulfate transporter 3 [Vigna radiata var. radiata]	Sulfate permease family	TGTGCAATAGATGAAGTTCAGAATTGGTGCACAGACACCTGATATCAATCAAGCTTTTGCAATCTTGGCAGAGAGGCATGCGTCTACAGCTTCTCCAACA[G/C]AAGGAAAACCCATTCTTTTCCAATCTTGTCTACAAAGTGTGCAAGTTTAAGCTTGTGGATTACTAGCCACCTTGGGTTCACCATAGCTAACTATAAAAAC
PHG9_Scaffold_2678	395			missense_variant	Mu_g15656.t1	U-box domain-containing protein 3 [Glycine soja]	Armadillo/beta-catenin-like repeat	ACTGGTTGTTGCATGCACAGGAACTTGTTCCTGTGGCTCTTGCTAAGGCAAGAGAGGTTAAGGGATTCCCTGGTAGGTGGAAGATGATCATTTCAAAATT[G/T]AGCAGATTCCTTCCTGCTTATCGGATTTGTCTAGCCACCCTTGTTTCTCCAAGAATGCTCTTTGCAAGGAGCAATTGCAGGCTGTGTCAAAGACACTTAA
PHG9_Scaf	396			missense_v	Mu_g1565	U-box domain-	Armadillo/beta-catenin-	CTGGTTGTTGCATGCACAGGAACTTGTTCCTGTGGCTCTTGCTAAGGCAAGAGAGGTTAAGGGATTCCCTGGTAGGTGGAAGATGATCATTTCAAAATTG[G/C]GCAGATTCCTTCCTGCTTATCGGATTTGTCTAGCCACCCTTGTTTC

fold_2678				ariant	6.t1	containing protein 3 [Glycine soja]	like repeat	TCCAAGAATGCTCTTTGCAAGGAGCAATTGCAGGCTGTGTCAAAGACACTTAAA
PHG9_Scaffold_2678	397			missense_variant	Mu_g15656.t1	U-box domain-containing protein 3 [Glycine soja]	Armadillo/beta-catenin-like repeat	TGGTTGTTGCATGCACAGGAACTTGTTCCTGTGGCTCTTGCTAAGGCAAGAGAGGTTAAGGGATTCCCTGGTAGGTGGAAGATGATCATTTCAAAATTGG[A/T]CAGATTCCTTCCTGCTTATCGGATTTGTCTAGCCACCCTTGTTTCTCCAAGAATGCTCTTTGCAAGGAGCAATTGCAGGCTGTGTCAAAGACACTTAAAG
PHG9_Scaffold_269	1028			missense_variant	Mu_g04320.t1	phospholipase A1-Ibeta2, chloroplastic [Vigna radiata var. radiata]	Lipase (class 3)	GTTCCTGGGATGTTTGTGAGCGAAGAGGTTGAAAAGAAACTGAGAAGTTCGAAGGTTGGGGGTGTACTGAATATGGTGGATGAGTACTCGCACATGGGAA[G/C]GAACTGCGGGTGGAGACGAAGATGTCACCGTTTTTGAAGCCGGATGCTGACATGGCATGCTGTCATGACTTGGAAGCATACCTTCACCTTGTTGATGGGT
PHG9_Scaf	0368			missense_v	Mu_g0432	hypothetical protein	GATA zinc finger	ATACCCCTTTGCATGCTCGGGCAGAAAATGTTGATTATGAGGATCAAAAGGTTTCCAGAGTGAAAAACATATCATTAAATAAGAGCAAAGAAGTTAAATT[G/T]TCAAGCGAAAGCAGAACTATGATAATTCTGCATCTGGAGGGTT

fold_ 269	4			arian t	8.t 1	PHAV U_003 G2245 00g [Phase olus vulgari s]		TGTTCCAGATTATAATCAAGGACATCGAAAGGTTGTGGATGAAGATACG AGCAATAG
P HG9 _Scaf fold_ 269	03 68 5			m issen se_v arian t	Mu _g0 432 8.t 1	hypoth etical protein PHAV U_003 G2245 00g [Phase olus vulgari s]	GATA zinc finger	TACCCCTTTGCATGCTCGGGCAGAAAATGTTGATTATGAGGATCAAAAG GTTTCCAGAGTGAAAAACATATCATTAAATAAGAGCAAAGAAGTTAAAT TG[G/A]CAAGCGAAAGCAGAACTATGATAATTCTGCATCTGGAGGGTTT GTTCCAGATTATAATCAAGGACATCGAAAGGTTGTGGATGAAGATACGA GCAATAGA
P HG9 _Scaf fold_ 2747	11 12 7			m issen se_v arian t	Mu _g1 579 9.t 1	O- acyltra nsferas e WSD1 [Glyci ne soja]	Protein of unknown function (DUF129 8)	TGCCTGCAAGAGAAATAAAAAAGGTTTTGCATTACTTTCATAAATTTTTC CTCCATCAATTTCGGTGTTGTTTAGTAAGTTGAATCAACTATATTATTAC[C/G]TAGGGTGTCCATATACACTAGGAGCAATGTATGCCACGGGATGACC ATATAAGCTAATTTCTTCCATGGGACCAGGCATATTAGAGAAGGCTATG GTAGT
P HG9 _Scaf fold_	11 22 2			m issen se_v arian	Mu _g1 579 9.t	O- acyltra nsferas e	Protein of unknown function (DUF129	ATTACCTTAGGGTGTCCATATACACTAGGAGCAATGTATGCCACGGGAT GACCATATAAGCTAATTTCTTCCATGGGACCAGGCATATTAGAGAAGGC TA[T/C]GTAGTGTTAAAGAGAACTTTGTGTGTTATGGCAGCAGCAACCTA CATGAAAGTCCAAAAAGTAAAAATAATTTGCAGAAGATGAACAATACA

2747				t	1	WSD1 [Glycine soja]	8)	TAAAAGCG
PHG9_Scaffold_2747	11543			missense_variant	Mu_g15799.t1	O-acyltransferase WSD1 [Glycine soja]	Protein of unknown function (DUF1298)	TTAGCACCAAATAAAATGAGTACTATCTTTGCACAAGCGTAGGAGCAAATTGCTTCCAATGAGTGCTTTTTCGGTCGATTCTATCCTTAGCTTTACGAA[C/T]TATTCCAATGGATCTTCATATAAAGCAATATAGAATGGGAGGATGATGTATCCCAAGCAATTAGCCCATTTTACTTTTGATTTCTCTGTCATCATATCTG
PHG9_Scaffold_2778	7200			missense_variant	Mu_g15919.t1	early endosome antigen 1 [Vigna radiata var. radiata]	NA	GCTTGCACAGGTTCCAATGGAAGAGAATCAACCTGTGTAGACATGTCCATTGATTAATTTTGTAATTTCTGTAACCTTTCTGCATCCTTTCTCATATCTT[G/C]AGCTGTTGTTGTACTTGAGCACTAGCTTTTGTAGTTAACTCAATTTGCTCATCCTTCTCAGACAATAAGGCTAACTTCTCTTTCTCAGCTTTATTGGCAG
PHG9_Scaffold_2778	7223			missense_variant	Mu_g15919.t1	early endosome antigen 1 [Vigna radiata var. radiata	NA	AGAATCAACCTGTGTAGACATGTCCATTGATTAATTTTGTAATTTCTGTAACCTTTCTGCATCCTTTCTCATATCTTGTAGCTGTTGTTGTACTTGAGCA[C/G]AGCTTTTGTAGTTAACTCAATTTGCTCATCCTTCTCAGACAATAAGGCTAACTTCTCTTTCTCAGCTTTATTGGCAGCTAGTTTTGAATTGTTGATAACT

						]		
PHG9_Scaffold_2778	7706			missense_variant	Mu_g15919.t1	early endosome antigen 1 [Vigna radiata var. radiata]	NA	GTTCATTTTCTTGTTTCTTTCAATGTCAGATTTCTTCAATAGGGTGGACAATTCTTGAATCATCTTTTGGTTGTGTTCTAGCTTAAGAAGTGCTTGCATG[A/T]TGCAGTATGCTTTGCATCTAATTGTACCTTGCAGGGGGCCAATTCTTTTGTCAACTCCTCAAATTCTTTTCCACAATCACTCTGACCTACAAATAAAGCA
PHG9_Scaffold_2864	9425			missense_variant	Mu_g16169.t1	hypothetical protein KK1_034202 [Cajanus cajan]	Protein of unknown function (DUF1218)	TGGATTGAGCATGCTGGTGATAGGGACTTCATCAAACAACAAGTCAAGTGGTTCCTGTGGTTTCACTCACCACCACTTCCTATCCATTGGAGGAATTTTG[T/G]TTTTGTGCATGCCCTATTCAGTGTTGTGTATTACGTTTCTGCCACTGCCTCTTAATTAGCGATTAAGATAACTGAACTTTGAGGCCATCTCTGCACCCTT
PHG9_Scaffold_2864	18688			missense_variant	Mu_g16179.t1	hypothetical protein PHAVU_005G113600g [Phaseolus vulgaris]	NA	TTTGTTTTGAGCATGCCTTTTTCAATGATTTTTGCAATACTATGCTTTGTATTAGGATATACGCTCTATTGGGGCAGTTGCTGGTCTTGCTGTTGCAATT[G/T]TTTCACATGGAGGCTGTTGAGATCTCCTTCTGGATCTCAACGGAGGCAACAAAAACGGCAAGGTGCTTCATCTAGTAATCCTGGAGTCACTGCGCATTCG

PHG9_Scaffold_2864	18689			missense_variant	Mu_g16179.t1	hypothetical protein PHAVU_005G113600g [Phaseolus vulgaris]	NA	TTGTTTTGAGCATGCCTTTTTCAATGATTTTTGCAATACTATGCTTTGTATTAGGATATACGCTCTATTGGGGCAGTTGCTGGTCTTGCTGTTGCAATTG[T/A]TTCACATGGAGGCTGTTGAGATCTCCTTCTGGATCTCAACGGAGGCAACAAAAACGGCAAGGTGCTTCATCTAGTAATCCTGGAGTCACTGCGCATTCGA
PHG9_Scaffold_288	57822			missense_variant	Mu_g04486.t1	hypothetical protein VIGAN_05023500 [Vigna angularis var. angularis]	NA	GGGATTGCAGGCAATGTTGGGTTAGGCAAAGAGGGAATGCCTGGGAAATTTGATGCAGTTGCTTGCATAAGATGGCGAGCTGCTAGGCTCATGCTCATGC[T/A]GAGAAGGTCAGAGCAAGGAGAAAAGCTGTGATCAATGATTTGCTAGAGGCCATAGCTGAATTAATTGATTGCAAAAACTAATGAAGAAGAACTGCAAAAT
PHG9_Scaffold_288	57858			missense_variant	Mu_g04486.t1	hypothetical protein VIGAN_05023500 [Vigna angularis var.	NA	ATGCCTGGGAAATTTGATGCAGTTGCTTGCATAAGATGGCGAGCTGCTAGGCTCATGCTCATGCTTGAGAAGGTCAGAGCAAGGAGAAAAGCTGTGATCA[A/C]GATTTGCTAGAGGCCATAGCTGAATTAATTGATTGCAAAAACTAATGAAGAAGAACTGCAAAATATGAAAATGGTATGTATTAGCTTTTGAGATTGTATG

						angularis]		
PHG9_Scaffold_2956	5713			missense_variant	Mu_g16450.t1	hypothetical protein PHAVU_006G055500g [Phaseolus vulgaris]	Agenet domain	ATTACACCTTATTCTCAAGTCATTAGTGTAGAGTGCGTAGATGGTCCTGCCACAGTCTTGACTCGTGAACACTATGAAAAGTGCATGCCTTTTTTTCGC[C/T]ACTTCAAGAGACAGAATACATTTGTGCTTTAGGCAATTTAGAAGCAACAAAGTAAAGCCGTTTGACTTGAGTAAACTGCGTGGCTACTATGCTCAACCAA
PHG9_Scaffold_3	45220			missense_variant	Mu_g00053.t1	protein FLX-like 1 isoform X1 [Vigna radiata var. radiata]	NA	AACTGGTGCGTCCACCACATTCCCAATTTCCCTCACTCCCCCTTTTCTTCCATTGTTATTAGTTAAAACGATGTCGGGCAGAAACCGCGGGCAGCCCCTT[C/T]TCATCCGCATGCGGCGGGGCTATCGCCGCCTATTCACGACCACCCGCTGTTCGGTGCACGAGCCCATCACCATCTAGTGGGGCCCATTGTCCCACCCCAT
PHG9_Scaffold_300	7186			missense_variant	Mu_g04626.t1	uncharacterized protein LOC106778462 [Vigna	NA	ATAGAACTGATTTTGCCGAGAGCAATTCATCACAAGGGAGGATTGAGCTTAGGCTGTTGTTATAGGGATTTGCTTGAAAGTTGTCAAGAGCTGTGCATGC[A/T]CGCCAGAAAATCTGTATGAAAAAAGAACTCTAAACATTTAAACTCAAACATCCACCTTATAAACATATTTATTACAGGAAAAAGTTTTACACTATTGGAA

						radiata var. radiata]		
PHG9_Scaffold_3072	927			missense_variant	Mu_g16728.t1	hypothetical protein PHAVU_004G005800g [Phaseolus vulgaris]	Cytochrome P450	AATTGCGTATAAAAATTGATCAAATGTTTTGCATGCTTCCATCAACTTCTTCTCTTGACCAATTCGAAGCCATTTCTCCAGCTTCCAAACACACTTTGGC[G/A]TGTATATCTGTTGAATATAGACTTTAGTACTTGGCTGTAGGCCTCCTCAGCACTAACTTGTGGAAAATCATTGGAAAGGCAATTAGGATCATGTCCAAAA
PHG9_Scaffold_3072	955			missense_variant	Mu_g16728.t1	hypothetical protein PHAVU_004G005800g [Phaseolus vulgaris]	Cytochrome P450	TTGCATGCTTCCATCAACTTCTTCTCTTGACCAATTCGAAGCCATTTCTCCAGCTTCCAAACACACTTTGGCGTTGTATATCTGTTGAATATAGACTTTA[G/T]ACTTGGCTGTAGGCCTCCTCAGCACTAACTTGTGGAAAATCATTGGAAAGGCAATTAGGATCATGTCCAAAAATTATTGAACATGAGTTATCAAAAGAAA
PHG9_Scaffold_3072	963			missense_variant	Mu_g16728.t1	hypothetical protein PHAVU_004	Cytochrome P450	TTCCATCAACTTCTTCTCTTGACCAATTCGAAGCCATTTCTCCAGCTTCCAAACACACTTTGGCGTTGTATATCTGTTGAATATAGACTTTAGTACTTGG[C/G]GTAGGCCTCCTCAGCACTAACTTGTGGAAAATCATTGGAAAGGCAATTAGGATCATGTCCAAAAATTATTGAACATGAGTTATCAAAAGAAAAGCGACCG

						G005800g [Phaseolus vulgaris]		
PHG9_Scaffold_3072	974			missense_variant	Mu_g16728.t1	hypothetical protein PHAVU_004G005800g [Phaseolus vulgaris]	Cytochrome P450	TCTTCTCTTGACCAATTCGAAGCCATTTCTCCAGCTTCCAAACACACTTTGGCGTTGTATATCTGTTGAATATAGACTTTAGTACTTGGCTGTAGGCCTC[C/A]CAGCACTAACTTGTGGAAAATCATTGGAAAGGCAATTAGGATCATGTCCAAAAATTATTGAACATGAGTTATCAAAAGAAAAGCGACCGAATACATCTTG
PHG9_Scaffold_3072	385			missense_variant	Mu_g16728.t1	hypothetical protein PHAVU_004G005800g [Phaseolus vulgaris]	Cytochrome P450	GTCCCTTGACGTAGTTGTTGAAATTTTTGCTCAAGACATGGTGCACGTTGAGGGGGTCACTAGTGACCAAATAGTTCATGTTGGTAAACCAAGGTCCAAG[T/A]ACTCACCAGTGCCCCCATGTTGTTTCAGAATCTCAAATGCACCATCATGGATGCGCCACAAATTACAGAGTACCTGTGGTAGCATGCCAAGGATGGGGTA
PHG9_Scaf	844			missense_v	Mu_g1672	hypothetical protein	Cytochrome P450	TCGTATACATATTGATCAAATATTTTGCATGCTTCCCTCAACTTCTTCTCTTGACCAATTTGAAGCCATTTCTTCAGCTTCCAAACACACTTTGGCATTG[T/C]TATCTGTGGAATATAGACTTTAGTACTTCGCCGAAGGCCTCCTCAAC

fold_3072				ariant	9.t1	PHAVU_004G005800g [Phaseolus vulgaris]		ACTAACATGTGGAAAATCATTGGAAAGGCAATTAGGATCATGTCCAAAAATTA
PHG9_Scaffold_3072	891			missense_variant	Mu_g16729.t1	hypothetical protein PHAVU_004G005800g [Phaseolus vulgaris]	Cytochrome P450	CTCTTGACCAATTTGAAGCCATTTCTTCAGCTTCCAAACACACTTTGGCATTGTATATCTGTGGAATATAGACTTTAGTACTTCGCCGAAGGCCTCCTCA[A/G]ACTAACATGTGGAAAATCATTGGAAAGGCAATTAGGATCATGTCCAAAAATTATTGAAGATGAGTTATCAAAAGAAAAGCGACCGAATACATCTTGAAGA
PHG9_Scaffold_3072	263			missense_variant	Mu_g16729.t1	hypothetical protein PHAVU_004G005800g [Phaseolus vulgaris]	Cytochrome P450	ATCCCCGAAAGGCTGAAAAATCTCGTGAAACTCTGGTCCCTTGACGTAGTTATTGAAATTCTTGCTCAAGACATGGTGCACGTTGAGGGGGTCACTAGTG[G/A]CAAATAGTTCATGTTGGTAAACCAAGGTCCAAGAAACTCACCAGTGCCCCCATGTTGTTTCAGAATCTCAAATGCATAGTCATGGATGCGCCACAAATTA

PHG9_Scaffold_3072	296			missense_variant	Mu_g16729.t1	hypothetical protein PHAVU_004G005800g [Phaseolus vulgaris]	Cytochrome P450	TGGTCCCTTGACGTAGTTATTGAAATTCTTGCTCAAGACATGGTGCACGTTGAGGGGGTCACTAGTGGCCAAATAGTTCATGTTGGTAAACCAAGGTCCA[A/C]AAACTCACCAGTGCCCCCATGTTGTTTCAGAATCTCAAATGCATAGTCATGGATGCGCCACAAATTACAGAGTGCCTGTGGTAGCATGCCAAGGATGGGG
PHG9_Scaffold_3072	357			missense_variant	Mu_g16729.t1	hypothetical protein PHAVU_004G005800g [Phaseolus vulgaris]	Cytochrome P450	CTAGTGGCCAAATAGTTCATGTTGGTAAACCAAGGTCCAAGAAACTCACCAGTGCCCCCATGTTGTTTCAGAATCTCAAATGCATAGTCATGGATGCGCC[A/T]AAATTACAGAGTGCCTGTGGTAGCATGCCAAGGATGGGGTAGTCAGTGAGGAGGGGGTGTCTGCAACATAGTCTCCGACGGAAGAAGTACATGATGCAGA
PHG9_Scaffold_3072	405			missense_variant	Mu_g16729.t1	hypothetical protein PHAVU_004G005800g [Phaseolus	Cytochrome P450	CCAGTGCCCCCATGTTGTTTCAGAATCTCAAATGCATAGTCATGGATGCGCCACAAATTACAGAGTGCCTGTGGTAGCATGCCAAGGATGGGGTAGTCAG[T/G]AGGAGGGGGTGTCTGCAACATAGTCTCCGACGGAAGAAGTACATGATGCAGAAGAGTGCTGCAATGGTTGCTGCTGCATAGCCAAGATTGACCATTGTAA

						vulgaris]		
PHG9_Scaffold_3158	606			missense_variant	Mu_g16909.t1	PREDICTED: U-box domain-containing protein 33-like isoform X1 [Glycine max]	Protein kinase domain	AAGGTCCTTGCATGCTGTTGGAGTGTAACCTTTTTATAGCCACCTCAGTGTATCTCAAGACACCTTTGAATATACTTCCATATCCACCTTCTCCAATTTT[T/A]AGGATGGATTGAAGTTACTTGTTGCTTCTTTAATCTCTGAAAAAGAGAATTCTGAGAAAAATTGAGAGACATGACTGCATGAGGCCTCTCCTTGCTTTTT
PHG9_Scaffold_3364	9023			missense_variant	Mu_g17314.t1	PREDICTED: UDP-glycosyltransferase 83A1-like [Vigna angularis]	NA	GAAGCATAGCATGCATGTTGCTTTTGATAGAGAAAATAACTTTCTTGGGGTCATTTCTGTCATCTTCAGGGCCCAAACCATCTGGCAGTGTCACAAACTT[C/T]TCTGGGGTCCCTTGGGGTTGTCTAGCCCAGCACCTGCAGCATTTTCTCGTTTCTGGTTGAACTCTGTATTCAGAAAAGTGATCTTGCAGCCATGTATGGC
PHG9_Scaffold_	4734			missense_varian	Mu_g17354.t	PREDICTED: DDRGK	DDRGK domain	CGCTTTTAAAGTCATAAACGAATTGTTTGATCATCGATAACCAATCGACAGGCTCCGCGGCGAGAAACCGTGGTGCGCGCCACTGCCACTCGTCGCATGC[G/T]CGCCGACCTGCCGCGTCTGGAGCTAGCACGTCGGCAGATCAACCTGCGACGCTAGAAGGTTTCTCATTTTACTCTTTTTTATTTAATTTGTTT

3399				t	1	domain-containing protein 1 [Vigna angularis]		TGTTTC
PHG9_Scaffold_340	30800			missense_variant	Mu_g04971.t1	hypothetical protein PHAVU_004G022500g [Phaseolus vulgaris]	Glycosyl hydrolases family 38 N-terminal domain	GCTTCCGCTTCTTCGACATTAATATACCGAAAATCATCTCCAAGAGGAACAAGAAGTGTATTGGTTCGGTACAAAGTTGACTTTTTCCTGTATTGATCCA[G/T]AATTTAAGAGCTCTTTCCTGAACATTTTCTTGAGTGGTCTCCACAGGATATTGCCCCCATGGGCATTGTTCATAAACAAAACCCTGCATGCGTGCAAAGT
PHG9_Scaffold_351	5280			missense_variant	Mu_g05083.t1	hypothetical protein PHAVU_009G124800g [Phaseolus vulgaris]	AMP-binding enzyme	TATCATGTTGCATGCTGATCCTTTTCACAGCTACTGTGTGGCATTAGTGGCAGTTTCTCATCCCGCATTGGAGCAATGGGCTTCACAGCAAGGAATTGCA[T/C]TTCTGATCTTTCAGAACTGTGCAAGAAAGAAGAAACTGTGAAGGAAGTGCATGCATCACTCGTAAAGGTATGCCTTTAGTATGAATTAAGACACAGTGTT

PHG9_Scaffold_3558	7204			missense_variant	Mu_g17577.t1	hypothetical protein PHAVU_003G287200g [Phaseolus vulgaris]	Rhamnogalacturonate lyase family	ATTTACGGGCATTTTGAACTTTGAACCAACAAAAGGTAAAACAGCCAAAAAAATATTGGCATATAAGACAAGCATTCAGAAAAGTGCTGCATACCTCTCC[T/G]TGAATTCTGGGTTGGTTGGTCTCGTTAATAGAACTGCTTCTGGGTATGCCAGGGTTTGACCAGTTTCTCTATCTTTCATTGTTGGCATGCTTCTCTGCCT
PHG9_Scaffold_3601	240			missense_variant	Mu_g17662.t1	hypothetical protein PHAVU_003G174700g [Phaseolus vulgaris]	Protein tyrosine kinase	TGATCAGAATTCCGTTCCGTTGGATCTTCGTCCGTTGAACGTACCCGCGGCGGTGGCGGAGGAGCCGATGATTTCGCCGGCCACCGTCACGCCGCCGACT[C/G]GAATTCTATGGGCGAGCTTTTTACCAGCCAGCGGCGTCCGCTGGCGGCACAACATGGTGCGTCCGCCCCATTGCGCATGCCAACGTGAGCCCTGCCGCG
PHG9_Scaffold_37	4314			missense_variant	Mu_g00737.t1	hypothetical protein LR48_Vigan01g303800 [Vigna angula	Pectinesterase	TTTGAGATCGCTAACTCCACTGAGCGTGACGCTTGGATCCTTGACTCAGGGGTACCCTACAACCTGGGCCCACTTCCCACACCAATCCCTATGCCTGCAG[C/T]TAATTCATTTCTTCAAATCTAATCTCATAGACTATATATGTATGTAGTCATGCATATGTAGTTACAAATATATATTTAAATCCCTGCATGCTTGCATGCT

						ris]		
PHG9_Scaffold_3752	6254			missense_variant	Mu_g17865.t1	hypothetical protein PHAVU_009G128700g [Phaseolus vulgaris]	NA	ATCATCAAAATTAGCTGTTTTGGGGTTTGAACTTTTGGTGTTTGTATTTAATCCAACAATCTTATCAAGCAGATCATCAACACCGACACTCTTCGCATGC[A/T]TCCACCGAATATGTCATCCACATAAGACGACGTGGAAACCGGTTTATTAGATCCGGCGAATATGGAATCGTAGTTGAACGAAGAGCCATTGGATTTCCCG
PHG9_Scaffold_3905	2135			missense_variant	Mu_g18151.t1	PREDICTED: uncharacterized protein LOC100305687 isoform X1 [Glycine max]	Ribosomal protein L7Ae/L30e/S12e/Gadd45 family	CGAGTGTTTCTCTTGAAAATAGTTTTTCATTATCCAAGTATAAATTCTTATATGGTTGCCCTGTTCATGATTTATTTTTCTCCCTGCAGTGAAGAGGGTG[T/C]GTTGCAGCTGAACCAGTTGCTTCCATTCCAGGTGAGCCCATGGATATCATGACTGCCTTACAACTTGTTCTGAGAAAATCTCTGGCTTATGGTGGTCTTG
PHG9_Scaffold_	2158			missense_varian	Mu_g18151.t	PREDICTED: uncharacteriz	Ribosomal protein L7Ae/L30e/S12e/	TTTTCATTATCCAAGTATAAATTCTTATATGGTTGCCCTGTTCATGATTTATTTTTCTCCCTGCAGTGAAGAGGGTGTTGTTGCAGCTGAACCAGTTGCT[T/G]CATTCCAGGTGAGCCCATGGATATCATGACTGCCTTACAACTTGTTCTGAGAAAATCTCTGGCTTATGGTGGTCTTGCACGAGGCCTTCATGAATCT

3905				t	1	ed protein LOC100305687 isoform X1 [Glycine max]	Gadd45 family	GCA
PHG9_Scaffold_3953	8712			missense_variant	Mu_g18207.t1	helicase-like transcription factor CHR28 isoform X1 [Vigna radiata var. radiata]	SNF2 family N-terminal domain	GTGCTTGAGGGCATGCCACTACTCATACATGTAATATCAGCATTTAACTCCACGTTTTCAGCCTTCATGTTATTCATCATTTCATCATTTGGAACCAATT[G/C]GAACAAAAGTCATTAGGCAAAATACAAGGAGAAGAGTCAGCAACAAAGGGTCCATCATGCGTTCCCTGGTAACAGGCTGAATATCCATGGTAGTCAATGA
PHG9_Scaffold_3965	6960			missense_variant	Mu_g18221.t1	hypothetical protein PHAVU_003G202900g	Quinolinate synthetase A protein	TGGGCGTTCGAGGGTGAACACTTGGCACAACGTGCTGGTCAGCATGCAGAAGAGGACCAAGCAGCTGGTGGCTCAGAGGGAAGGGAAGGTTCCCTTTGAC[A/C]TTTTCCTTCATTGGTTGTTACCTCGGATGGGGTTTTTCCCAAGGGTAGCTACGCTGAAGCCCAGGTTAGACACCGTTTGAATTTTTTAATTCTTCCCCTC

						[Phaseolus vulgaris]		
PHG9_Scaffold_41	9923			missense_variant	Mu_g00835.t1	peptide chain release factor APG3, chloroplastic [Vigna radiata var. radiata]	PCRF domain	AAGTATATGCATGCCTTCCTTTCTTTTAAGCTTTAAATCAACATCTTAATCTATTGATCTGGAATATTTACTTAGGTTGATGAAGTTGAAGTCGAAATTG[A/C]CCCAAAGATATTGAACTGACAACAGCGCGTTCTGGGGGTGCTGGAGGTAAATATCTCTGAGAGACCATTGTTGTCAAGTAATCAATCTTTCTATTCCTTG
PHG9_Scaffold_4282	0334			missense_variant	Mu_g18492.t1	Putative WRKY transcription factor 72, partial [Glycine soja]	WRKY DNA -binding domain	TGTGAAATAACAAATGTTATGCCTTTCAAACAGATGCATGATGGATGTCACTGGAGGAAATATGGACAGAAGATAGCAAAAGGAAATCCATGTCCAAGAG[G/C]TACTATCGTTGCACGCTTGCTCCAGCATGCCCGGTCAGGAAACAGGTATTCATCAACATCACATTTTTAAGTCCTATTTGAATAAAATTCTCAGGAAGTA
PHG9_Scaf	3712			missense_v	Mu_g0565	BRO1 domain-	NA	AGTATAAATTCATGAATTATTGGCTATGTTAAAGTTATATTTTTGGTAGGAAGTATTTAATCAATCTTGATACCATTACTAACCTTAGCTTCGAGGAATT[T/G]CATTTGATGAATCGGAGATGCTTCCTTCCACGCCCTTGGTTGGTGTC

fold_450				ariant	8.t1	containing protein BROX homolog isoform X2 [Vigna radiata var. radiata]		GCATCCTGACAAAGAGTGATAAGCCTGCTCAAGAAAGTGAAACATTTTTATTT
PHG9_Scaffold_4543	4463			missense_variant	Mu_g18733.t1	hypothetical protein PHAVU_003G204700g [Phaseolus vulgaris]	Leucine rich repeat	ATTCTGATCCAACTTTAGCTTGGTCAAGTTATGAAGGCGACCAAAAGAATCAGGAAGTGCATGAATTTGATTGTTGCTGAGATCCAAGTCCCTGAGGTTA[A/G]CAAATCACCAAATGTCTCTGGAAGTTCTTTAAGGTCACTGAAGTTACTGCTCAGGTTTAGAACTTGAAGATTAGTCAATTTCCCAATTGCAATAGGAAGA
PHG9_Scaffold_4543	4530			missense_variant	Mu_g18733.t1	hypothetical protein PHAVU_003G204700g	Leucine rich repeat	TGATTGTTGCTGAGATCCAAGTCCCTGAGGTTAATCAAATCACCAAATGTCTCTGGAAGTTCTTTAAGGTCACTGAAGTTACTGCTCAGGTTTAGAACTT[G/C]AGATTAGTCAATTTCCCAATTGCAATAGGAAGACCGCGCAGCTCGTTAAAGTGAGCATCCAGATGGCGCAAGGATTTCATCTCACAAACAGACGAGGGAA

						[Phaseolus vulgaris]		
PHG9_Scaffold_4553	03018			missense_variant	Mu_g18882.t1	hypothetical protein PHAVU_005G005000g [Phaseolus vulgaris]	NB-ARC domain	AGCCACTGAGCAAATGACTCCATCAGCAATCTCATGGCTCTGACTGTCTGAGTACTTTGTTCTATCAGCAAGCATGCAATCGATCCCTTGAAGCTCAAGC[T/G]AGACTTAAGCCACTTGCAGAAGCGCACCAAGTTGGGGTTCTGGCCATGGAATCCAATGTAGACATCACAGCTTCTGAGTTTGGCATTCGTAGCAGGTGAG
PHG9_Scaffold_4561	40390			missense_variant	Mu_g18958.t1	hypothetical protein PHAVU_001G090100g [Phaseolus vulgaris]	Exo70 exocyst complex subunit	CGGGAAATTCGTCTCTTGTTCACCACCTTCTTGATCTTGCACCACCTCTTGTTGTTGTTGTTCCGATGACGCCACATGCTGTTTCCATTTGTTATCGACG[C/A]GTTCTGATAATTTCCTCCGGGATCCAATTCGGTTGGCATTCTAGATTCTTCCATCAGAAACCGGAAGTCCTCCTCTAGATACATCATCAAACGCTGGTGG
PHG9_Scaffold_4562	5184			missense_variant	Mu_g18967.t1	PREDICTED: ultraviolet-B recepto	Regulator of chromosome condensat	TTCAATTTTGAAGCAGACTCTACCAAGATTAGTGGATTGTCCGAGTTTGGAAAATATGCATGCCAAGAATATATCGTGTGGTGCTCGACACAGTGCTTTA[G/A]AACAGGTATACCAACATATAGCTACCTGCTTATGTCATTTTAATAGCTGTCTAGTCAGAGTTAGGCTTCCATTATTGAAGTCATTTAATACATCTCTAAG

						r UVR8-like isoform X1 [Vigna angularis]	ion (RCC1) repeat	
PHG9_Scaffold_4565	2878			missense_variant	Mu_g18980.t1	PREDICTED: copper transport protein ATX1 [Vigna angularis]	Heavy-metal-associated domain	AGCAACTGAATACACTGCAGGGGTGTGGAAGAAAGTGTGGACTCCATTGGTTGCTTCACTTTTGTATGCATCATAGTAATGGTTAACAATGTAACTAGGG[T/C]CTTGTAAAATGAGGAAAAATGAGCATGCCCTGGAAATGGCCATGGTTCTGCTACTTTCCCTGCACGTTTGATTGCCTTCAGAACCTTCTTTTCCTCCAAG
PHG9_Scaffold_4598	5015			missense_variant	Mu_g19163.t1	uncharacterized protein LOC106780260 [Vigna radiata var. radiata]	NA	NNNNNNNNNNNNNNNNNNNNNNNNNNNNNNNNNNNNNNNNNNNNNNNNNNNNNNNNNNNNNNNNNNNNNNNNNNNNNNGATTAGTTGTCAATGACAACAAGT[C/G]GAATTTCTCCCAGAGAGCAATAACCTCGCTCTGGCTTCTATATTGATGCCCTTCTCTGTCATAGTAGTACTGCATGTCCTCACATGCATGCATACCAATT

PHG9_Scaffold_46	1544			missense_variant	Mu_g00876.t1	histone H2A [Sesamum indicum]	C-terminus of histone H2A	GGGCGGTGGCCCGAAGAAGAAGCCAGTTTCAAGGTCCGTCAAGGCCGGTCTCCAATTCCCCGTCGGAAGAATTGGCCGTTATTTGAAGAAAGGAAGGTAT[T/G]ACAGCGTGTGGGAACCGGTGCTCCTGTTTACCTGGCTGCAGTTCTTGAATACCTAGCTGCTGAGGTAACCTAGCATTAATTGAATCTCTCAGAGATTGTT
PHG9_Scaffold_4666	02996			missense_variant	Mu_g19328.t1	PREDICTED: RNA-dependent RNA polymerase 1 [Glycine max]	RNA dependent RNA polymerase	TGTGCCTTCAACGAATCTGTTAGTATAGTGTTCAGTTGATCCACAGCTTCTCTTTGTTTTTTCTCAAAAGCACCATCCTTGATACCAAGAGTGGATAAGA[G/A]GAAATTAACTGCCGATTCAGATAACAAGGCTGATACTTACTACGAGCCAAAACATCTAACTTCGTGTTATCTGATTCATACTTCAGCATGCTCTTCCTCA
PHG9_Scaffold_4746	5117			missense_variant	Mu_g19421.t1	Nucleolar complex protein 2 like [Glycine soja]	NA	CTATCTGTGGGTTGGGATATGATATACTCATCATGGGTAAATACCCACCTAAATTATGGTTAAGATATATACTGATCAGTTTGAGTTTTTCATACAGGAC[A/T]AGTCAGGATATTGAAGAAAGCATAGAGAATGATACAACAAATCCATCAATTGGAAGGTAAAATGGTAACGTAAATACTATTTATCTGTTGATTCTATTCA
PHG9_Scaffold_4784	5941			missense_variant	Mu_g19752.t1	quinone oxidoreductase	Zinc-binding dehydrogenase	ATTTAGAATTTAGAATATGATGACATACCAAGAAGGTTTCCCCGTGACTTAAACGACTGGTCATGAAAACAGTAGACCATACAGTGCATGCGACCTCAGG[A/C]AACTAGCTGCATCAACGAGGGAAATACCTGGTGGAACATAAAGCACTTGCCCCTCGGGAACTGCAACTTTCTGGGCATATCCTCCACCGGCGAGAAGAGC

						PIG3 [Vigna radiata var. radiata]		
PHG9_Scaffold_4797	408			missense_variant	Mu_g19872.t1	PREDICTED: aldehyde dehydrogenase family 2 member C4-like [Glycine max]	Aldehyde dehydrogenase family	GAGGTAATTGGAAGAGTTAGTGAGGGAGATAAGGAAGACATTGATGTTGCTGTTGAAGCAGCACGTCAAGCATTTGACCAAGGTCCATGGCCTCGCATGC[C/T]GCCTGTGTACGTCATTCTTCTTCCTTTCTTCAATTTAATCTCTTTATACTTCAAAAAAAAAAACTCAATATATTTTAAACCATTTACTTTTTCTTGCTTT
PHG9_Scaffold_4816	0434			missense_variant	Mu_g20072.t1	hypothetical protein PHAVU_004G090900g [Phaseolus vulgari	pre-mRNA splicing factor component	CCATATTGAAATATATTATAATTTGGTTACTGACATTTTTCAGATGAGGGTAAAGAAAAGCCTCTGGCCTCAAATTGAGGCCACTTTCAAGCAGATGGAC[A/G]AGCTGCAACTGAGTTAGAATGTTTTAAAGCTTTGCAAAAACAAGAGCAATTAGCAGCATCACATAGGATAAACAATCTATGGGCTGAAGTACAGAAGCAA

						s]		
PHG9_Scaffold_4816	0484			missense_variant	Mu_g20072.t1	hypothetical protein PHAVU_004G090900g [Phaseolus vulgaris]	pre-mRNA splicing factor component	TAAAGAAAAGCCTCTGGCCTCAAATTGAGGCCACTTTCAAGCAGATGGACATAGCTGCAACTGAGTTAGAATGTTTTAAAGCTTTGCAAAAACAAGAGCA[A/C]TAGCAGCATCACATAGGATAAACAATCTATGGGCTGAAGTACAGAAGCAAAAGGAACTTGAGAAAACTTTGCAAAATAGGTACGGGAGTCTAATTGAAGA
PHG9_Scaffold_4826	0036			missense_variant	Mu_g20161.t1	hypothetical protein PHAVU_007G010900g [Phaseolus vulgaris]	F-box domain	TTCAACCTGAGGCATGCCATGCTTGATTTCAGACAAGGACCAAGCTGTGCCGGCTTGAAGGCTCAAGATTTCAACCAAACACTGTTAACGATAAAGAATT[G/C]GAAACTCTCACTTTATGTGAATGGACTTTTGAGGTATCTATATGTTCTTTCAATTATTTCCCATCTACTGTTGCTGATTTAACACAATTATCAAGCGAAT
PHG9_Scaffold_4835	1716			missense_variant	Mu_g20232.t1	hypothetical protein PHAVU_003G185600g	NA	ATTGAATAAATGCTAACTAAGTTGTTAACTCTGATCAAATTTCTCATTCAAAAAGAAAAAATATAAATCTTACAGGCTTTTGCTGCCAGGGAACACGCGT[G/C]GTCATTTTCTGATGGAGAGATGGAACTGCATGCATCTCCTAACACTAATCTAGCAACAGAGTAGACCACACTTTCATGATGCAACTTGACATCTGCAGAG

						[Phaseolus vulgaris]		
PHG9_Scaffold_4856	4589			missense_variant	Mu_g20409.t1	hypothetical protein PHAVU_011G183800g [Phaseolus vulgaris]	Pathogenesis-related protein Bet v I family	AATGTCACTCACAGGAAAACTTAGCACTGAAATTCCTGTTCATGCAACTGCAGACAAATGGTTTAATACCTTCACAAATCAACTCCACCATATGCAACAC[G/A]TGCTGATAAAATTCATGAAGCCAAACTCCATGAAGGTGATGATTGGCATACCTCTGATTCGGTCAAACACTGGTCTTATACAGTAGGTAATTAAGCTTAT
PHG9_Scaffold_4879	6459			missense_variant	Mu_g20583.t1	4-coumarate:coenzyme A ligase 3 [Phaseolus vulgaris]	AMP-binding enzyme	AAAACCACAACAACGATTTTGCTGTGTTTGCTCTGTCACAGGGACCTAATTGGCCACCACCACCAGACCTTCGTTCAGTCTTGCAGTTAATACCTTCCGC[A/T]AATTTTTCCTGAAGCTGCTTTAGGAATAGAGTCTGTGAAGAACACCCTACCGATTCTCTTGTAAAATACCACCTGCCACAAACGCATGCTTATTAGCCCC
PHG9_Scaffold_4898	3043			missense_variant	Mu_g20671.t1	hypothetical protein PHAVU_007	Beige/BEACH domain	CAAGATTGGGCATGCTGCCATTCAGGTTTCATTGGGTGAAAGATCATGGCCTCCAGCTGCTGGTTATTCATTTGTTTGTTGGTTTCAGTTTCGTAATTTT[T/A]AAAATCACAGTCAAAAGACACTGACATTTCAAAATTTGCCTCTTCAAAGAAGCGCTCTGGTTCAAGTGGATTGCATGAGCGACATATCTTAAGGATTTTT

						G175300g [Phaseolus vulgaris]		
PHG9_Scaffold_4915	5331			missense_variant	Mu_g20799.t1	hypothetical protein PHAVU_008G008100g [Phaseolus vulgaris]	Peptidase C26	ACCCAAATATCCATTTTTCCATCTTCCAAAGAATCCTTAAACCAATGATGCAAAGGGGTGTCTTCAACCACCTCCACATCGTGTCTATGCCCATCATAAT[C/G]TCATAGTTTATGTGCATCACCCTTTTGCTATCGGGGCACTTAACTGAAAGCTCTTTCCCTATGTCCTGGTAAAGGGTACCGCCGCATGCAACATTAAGGA
PHG9_Scaffold_4960	4769			missense_variant	Mu_g20986.t1	PREDICTED: metacaspase-3-like [Vigna angularis]	Caspase domain	CCGTTGATTATGAAGATCAAGGGAAGATAATCGATGATGAGATTAACACTGCAATTGTTAGACCCTTACCACCTGGAGCTAAACTTCATGCCATTGTCGA[T/A]CATGCCATAGTGGGACTGTTCTTGATTTACCTTTTGTCTGCAAGATGAACAGGTCAGTTTCGGAAAGCTTGCTTTGCCAACAACAATTTAACACCATCTT
PHG9_Scaffold_4982	7543			missense_variant	Mu_g21122.t1	inactive beta-amylase 9 [Arachis	Glycosyl hydrolase family 14	CGCTGCTTGCATGCTAACATTTGATTCTGAGCTTTTAGTTGGTGAATCAGCTGCTTCTTCCTCAGAAAGTAGATCAGCTGAATGCAACTCCTGATAATTA[A/T]GCTCCTAACAAACTCAGCGAATGAAGGAAAATGTTCTGGTGAAAAGAAATAAGCTCCCATTCTCTGATAAATTAACAAGTCCAACACATTATCTGCACAC

						durane nsis]		
PHG9_Scaffold_5022	28			missense_variant	Mu_g21326.t1	expansin-A16-like [Vigna radiata var. radiata]	Pollen allergen	TGAACTCCTAGCATGCAAATAAGGTATGATATATGTACGTATGTATGTAAGTAACCTGCGATATTTGATTGGGACGATGCCAGCCTCGTATTCTGCAATT[T/C]AAGATACGCAGGTTTGGCCAAATCAAAGTGATCGCGTGGTGGGTTGCACCATCCTCCATTGTCACCTGGTAGATCGTAATTTGGAGGACAAAAATTGGTT
PHG9_Scaffold_51	0321			missense_variant	Mu_g00995.t1	PREDICTED: ethylene-responsive transcription factor ERF112-like [Glycine max]	AP2 domain	CTTCTTAAGCATGCAGTTATGAAGAACCATGCACTTTCATCATCTCTTTATTCCACATTCAAGCCTTGGAAAAACCAGATCATGATGAGAGATGAATTTG[A/G]TCCCTTGTTGAGGAAACTATAGTTTGTTCCATTCCTGAACAAGGCTCTGGCTGTTGTGGAATTTCATTTGGAAGTTCTAAGGTTTACTCTTCTGTTGTTG
PHG9_Scaffold_5105	7316			missense_variant	Mu_g21676.t1	hypothetical protein PHAVU_001G1472	Sugar (and other) transporter	AACTAGTAATTGATTACGCATAGAAGTGAACATGGCTATCAAAGATGTGGAAGAAGGCATGCAGAAAGGAATCAGAGAGCCACTGGTGGGGGAACAGGAC[G/C]CCGGTTGGTCCATGCAAGCAAAGGGCATCCATGGATGGTTTATTTTTGTACATTCGTTGCAGTGTGTGGTTCTTATGAATTTGGTGCTTGCGTAAGTGTT

						00g [Phaseolus vulgaris]		
PHG9_Scaffold_512	6768			missense_variant	Mu_g06049.t1	PREDICTED: laccase-4-like [Glycine max]	Multicopper oxidase	AGGCACACTTCTCTGGCATGCACATATCTCTTGGCTAAGAGCCACGGTGTATGGTGGCATTGTCATATTGCCTAAACGAGGCATTTCTTATCCATTTCCC[A/G]ACCAGACAAAGAAAAGATAATCATTCTAGGTAAGTTTTATGTAGGATNNNNNNNNNNNNNNNNNNNNNNNNNNNNNNNNNNNNNNNNNNNNNNNNNNNNNN
PHG9_Scaffold_5140	4356			missense_variant	Mu_g21765.t1	hypothetical protein PHAVU_010G000700g [Phaseolus vulgaris]	Sympleki n tight junction protein C terminal	TTCCCTTGAAAAAGGTAAGGGTATCTGATGATAATATGTTTTATATAAAAATGAAAAAACATTTTGCATAACCTAGGTGTTTTCATATGCAGGTCACAGA[T/A]CATGCAATGCTTGTTTTGAGCAGCGACAAACTTTTACTCAAGAAGTTCTTGCCAGAGTTTTGAATCAGTTGGTGTGTTATGTGAAAATTGTTTTATATGT
PHG9_Scaffold_515	9433			missense_variant	Mu_g06103.t1	VuP5CS [Vigna unguiculata]	Amino acid kinase family	CACAGAACCAAACCCTCCCCCGAGATACCCCTCGTGAATGGCACCGCGTTAACTCACCTCAACTCCCTCTACGAATCTCACTTCTTCGCCAACATCGATC[T/C]TCTAGAGCTTTTGTCTCCAAGGTGAAGCGTCTCATTGTAAAGGTACTAATTTTCATTGCTTTTCGTTTTTTACTCTTTCTCTCTCCATATATAATTCATA
PHG9_Scaf	2582			missense_v	Mu_g2179	PREDICTED: floweri	Sulfotransferase domain	AGGTTACTGCATATTTTCTTCTACAGTGGCAGCAGTGATCATACAACAAAGTTTTGGTGCAGAAATAGGCCAGGAGACACGGCTCGTGATAGATTTAACA[T/C]GGCATGCAAGGTTTGTTCATTTTTCATCTAAATATTGAACTGTAT

fold_5155				ariant	8.t1	ng time control protein FY [Vigna angularis]		GTTGAAGGGTTTGGATAGTTTACTCTTCCAACATTTCATGGTAATGTAAATACC
PHG9_Scaffold_5230	036			missense_variant	Mu_g21904.t1	Putative calcium-binding protein CML23 [Glycine soja]	EF-hand domain pair	AGAGATTATGCATGCTGTGGCTCGTGGCATTGGTGGCTCTCCCATCATTGTTGTTCTTGGAAAAGATAGCTTACTCATGAAGGCTGTTCAGCATGAATTA[G/T]TACAGTTTCTTCTTCTTCATGAATTTACATCCTCCTTCTTANNNNNNNNNNNNNNNNNNNNNNNNNNNNNNNNNNNNNNNNNNNNNNNNNNNNNNNNNNNNNNN
PHG9_Scaffold_544	7161			missense_variant	Mu_g06309.t1	hypothetical protein PHAVU_003G235400g [Phaseolus vulgaris]	NPH3 family	CCGCTCGTGCATGCCTTTACTTCCCGAGGCAGAAACGACGGCGTCTCTCGTTAGCAGATGCATTGAAGCGCTTGTTTCAGTTCACGGCATCACTCGTTTA[A/C]TGAAGTGAACGAAATGCAGCCTCGAGATTTTCAAATTGTGGCTGAATCAATGGGTAGAAGGTTCGAGAACCACGACGTCGTCTACAAGATGGTAGATTCG

PHG9_Scaffold_555	5620			missense_variant	Mu_g06355.t1	uncharacterized LOC100527249	Cupin	TATATTCATCTACTGAAGAGTTATTTGAACAAAAACATTGTCTTTGTAATATGCAGTATTTGTGAATGGAAAATTCTGCAAAGACCCTAAGGATGTGAA[A/C]CTGAAGATTTCTTCAAGCATACGGAACCTGCGAACACTGACAATGAACTAGGAATAGCAGCGACTCAAGTGACTGTTAATGAATTACCTGGACTAAACAC
PHG9_Scaffold_5627	0440			missense_variant	Mu_g22215.t1	hypothetical protein LR48_Vigan09g106600 [Vigna angularis]	Domain of unknown function (DUF4487)	AAGATCCTTATGCATGCTTTTAACCTTTGGTGCGCAATCAATGGTTAACGAGGTGTACACGTCTCTTATTGGTGATGGGAGGTCACAGTTGTCACCAATT[A/T]TTGTTTGGCTCTGTTCATTGAAGGTTTTCCACTTGATTTACTGACAGATGAGTTGAGAAACACTTCTATTCAAAGAATTAAGTCTGATTATTTAGACTTC
PHG9_Scaffold_5665	560			missense_variant	Mu_g22484.t1	hypothetical protein PHAVU_007G218900g [Phaseolus vulgaris]	Family of unknown function (DUF5311)	AGTGAGTGTGCATGCAACTCAAGTTCAAGATGTATACCTCTTCAGTGCAGAACCCTTGAAAAATCTGAGAAGGACTGTAAAGGCCATATCATATAAATTG[T/A]TTGGACAAGAAGATCAGCCAAGTCTGATAGAGCTCCATGTTTTCCTTTAAAATGAAAGAGGACTGGGTGAAAACTGGTTTAAAATTCTATCNNNNNNNNN
PHG9_Scaffold_	6074			missense_varian	Mu_g22807.t	hypothetical protein PHAV	Chlorophyll A-B binding protein	TGGGGTGGCAAGCATGCTGACTTGGCCATGGTGTGATATCTCAACAACAACTGTGTCATCATCTTTTGGGAGGGGTTGTTTCCATGGCTCTCCATCAATT[C/T]CATGAATGTGCACTCAGCTGCTCCCTTGTGAAACTCAAATCGGATTCTGCTTGTCTGCATCGTGAGAGAAGCATACAAACGTAAGTTTGAGCAA

5689				t	1	U_011 G1558 00g [Phase olus vulgari s]		AAAGGA
PHG9 _Scaf fold_ 5693	14 66			missen se_v arian t	Mu _g2 283 9.t 1	hypoth etical protein PHAV U_003 G0848 00g [Phase olus vulgari s]	Sugar (and other) transporte r	TGACAGTGACTTAACAATATTAACATATGATAAGAGGAATTTGGAAGAACCTACCTCATTGTCGCTGTTGGCACAGAAGGCACACTCTGCACGCAAACAT[T/A]CATGCAGTTCCACGAAGAGAAATCAGGGGCATTTGTATAAGCCTTGCATGTAAAGTTGGCACCAAAATCAAGTGTGTCCTGGTTACTAATTGCAGGAGCA
PHG9 _Scaf fold_ 5697	49 65			missen se_v arian t	Mu _g2 289 9.t 1	PREDI CTED: alkane hydrox ylase MAH1 -like [Glyci ne max]	Cytochro me P450	CTTTGATTATTTTACTTCAATTTTGAAACAACAAGGAGGTAATTTCATGTTTGAAGGACCCTGGCTATCAGATATGAATATGTTCATCACCAGCGACCCT[G/A]GAATGTGCAATACATCACAAGCACTAACTTTGGAAACTATGGGAAGGGACGTAATTTTAGTGAAATTTTTGAAGTTCTGGGCGACGGGATTTTCAGGTCT
PHG9 _Scaf	41 53			missen se_v	Mu _g2 301	hypoth etical protein	NA	GGAAAGAAATTTGACAATCATTCTATGATTCAGAACGTTGATATGAGACTCGAGTCAGGGACTTGTAATGTGTGCTCAGCTCCTTGTTCATCGTGCATGC[A/T]CTTAACCGAGCTCTCATGGGGTCAAAGGCCAAAGAATTCTCTGAT

fold_ 5711	4			arian t	5.t 1	PHAV U_011 G0179 00g [Phase olus vulgari s]		GAAAACTGTCGTGTAGGGGAGGCTAATCAGTATTGTATTGATGAGGCTG ATGGAC
P HG9 _Scaf fold_ 5713	70 35			m issen se_v arian t	Mu _g2 308 3.t 1	Beta- glucosi dase D4	Glycosyl hydrolase family 1	ATGATTGTTTATTTATGAATACTAATTAAACAATTAGATTGAAAGTAAAT GCAATTGGTATCTTACCATTTTCAGTGATGTAGATCAAAGGATTGTTATA [C/G]TTGTTTTGATATAGAGCAACAGCTCGTGAATTCCTTTTGGATAAAC TGACAACCATTTTGAAGCAGCCTACAAAAGTAGTTATCCTTTTATTCATC AAGC
P HG9 _Scaf fold_ 5713	70 46			m issen se_v arian t	Mu _g2 308 3.t 1	Beta- glucosi dase D4	Glycosyl hydrolase family 1	TTTATGAATACTAATTAAACAATTAGATTGAAAGTAAATGCAATTGGTA TCTTACCATTTTCAGTGATGTAGATCAAAGGATTGTTATACTTTGTTTTG A[T/C]TAGAGCAACAGCTCGTGAATTCCTTTTGGATAAACTGACAACCAT TTTGAAGCAGCCTACAAAAGTAGTTATCCTTTTATTCATCAAGCAGAGT AACTTT
P HG9 _Scaf fold_ 5713	79 32			m issen se_v arian t	Mu _g2 308 3.t 1	Beta- glucosi dase D4	Glycosyl hydrolase family 1	AGGTTCATAAAGCAGTTACCATCCTAACATGAAATCAATGACTCTTTGT GCAGCCTGCCGATCCAATTCTGTATCATGGAGTGGTATCGTCCAATGAC AA[G/A]TAGTGGTATCCCTATCCATCCCTTTTGAGTTGCCTAGGTGCAGT GGAACACAAAGTAAAGGTAAGTTTAGGTCTCTATATTAATTCAGTAAAT GCATACA
P HG9 _Scaf fold_ 5713	79 39			m issen se_v arian t	Mu _g2 308 3.t 1	Beta- glucosi dase D4	Glycosyl hydrolase family 1	TAAAGCAGTTACCATCCTAACATGAAATCAATGACTCTTTGTGCAGCCT GCCGATCCAATTCTGTATCATGGAGTGGTATCGTCCAATGACAAGCTAG TG[G/C]ATCCCTATCCATCCCTTTTGAGTTGCCTAGGTGCAGTGGAACAC AAAGTAAAGGTAAGTTTAGGTCTCTATATTAATTCAGTAAATGCATACA CTTCATG
P HG9 _Scaf	79 63			m issen se_v	Mu _g2 308	Beta- glucosi dase	Glycosyl hydrolase family 1	AAATCAATGACTCTTTGTGCAGCCTGCCGATCCAATTCTGTATCATGGAG TGGTATCGTCCAATGACAAGCTAGTGGTATCCCTATCCATCCCTTTTGAG [T/A]GCCTAGGTGCAGTGGAACACAAAGTAAAGGTAAGTTTAGGTCTCT

fold_ 5713				arian t	3.t 1	D4		ATATTAATTCAGTAAATGCATACACTTCATGAATTGAATTATCAATATAA ATGTA
P HG9 _Scaf fold_ 5724	70 92			m issen se_v arian t	Mu _g2 318 4.t 1	PREDI CTED: unchar acteriz ed protein At1g2 8695- like [Glyci ne max]	Nucleotid e- diphosph o-sugar transferas e	TTTCACTGCTAATTCACATGTAACATCTTTCTCACAGGATACTGATGTAA TGTGGTTAAGAAATCCATTTACAAGATTGAGTAAGGATGAGACAGAAG AC[A/C]TCAAATGAGCACAGACGTGTACCATGGTGATCCTTGGTCAAGG AATAACCTAATCAACACTGGATTTTACTTTGTGAGGTCAAACAACAAAA CTATCTCC
P HG9 _Scaf fold_ 5724	72 04			m issen se_v arian t	Mu _g2 318 4.t 1	PREDI CTED: unchar acteriz ed protein At1g2 8695- like [Glyci ne max]	Nucleotid e- diphosph o-sugar transferas e	ACAGACGTGTACCATGGTGATCCTTGGTCAAGGAATAACCTAATCAACA CTGGATTTTACTTTGTGAGGTCAAACAACAAAACTATCTCCTTGTTTGAA A[C/A]TGGTATGACAAAAAGGACAAATCCAAAGGAAAAAAAGAGCAAG ATGTGCTTTATGAACTCATTAGAGGTGGCATGCTTGAATATTTGGGACTC AGGGTTA
P HG9 _Scaf fold_ 5737	10 79			m issen se_v arian t	Mu _g2 325 9.t 1	PREDI CTED: midasi n-like [Glyci	Midasin AAA lid domain	AGTAGAATAAAATATTAAGCGTAGTGTTCAATACAGGAGAAAACAAAG AAAAAATAGCAATCGATTTGAAGATCTTTATAGAATACCTTAATTTCAA GTT[C/T]AGTGTCGATATTGTTTCCATGAGCTGAGAGTACTTGCAATGGT ACTTCATGACGGGATCAATTTCATTACAATGGTACTTCATGACGGGATC AATTTCAT

						ne max]		
PHG9_Scaffold_5737	5801			missense_variant	Mu_g23259.t1	PREDICTED: midasin-like [Glycine max]	Midasin AAA lid domain	AGGAGCTGAAACAAAAAATTTTTTAAGTATTTCATCTTGCAAAACATGAAGGCACTTCAAAAGTGGAATGAAGCGACTGGCTTCTTCATCAAAATTATGT[T/A]GCTCTCAATATTTCGTTGCTGGAATGTTAGCCTTAGTGGATCAAAACAACTAATAGCATTAAAGAGATATTTAGTTTTGCCATTTGATGGAGCCAAATTA
PHG9_Scaffold_5737	9833			missense_variant	Mu_g23259.t1	PREDICTED: midasin-like [Glycine max]	Midasin AAA lid domain	AGAAACATACCAGGCTTTGGAAGCTGCATAGCCCGCAAAACTCTCAGGGCATTCCTGCAAGTAGTAGGTGCCATGAATTCAAACCCACCATCCTCAACAA[C/T]CCAATACCTATATGATTAAACAAAGTAATGCAAAATAAACACAAGAGACAATCATCATGGCAGTATTTCACAATGCAAAAGTAATTATAAAAGAAATAAG
PHG9_Scaffold_5763	77780			missense_variant	Mu_g23506.t1	hypothetical protein PHAVU_009G032800g [Phaseolus vulgaris]	GDP-fucose protein O-fucosyltransferase	GTTTCTCGCCGCGTCAATATTCTCCTTAGTTGTCACTTCTTTCTTCTTTGTTCACGTTCACGTTTCTCCTTCTCCCACTGATCACAGGTTCAATGACAAG[A/T]CCCTTCGGTGTGCCTCTTCTCTCTGTTCCTCTGTTTTTTTTTTTTTCTTTTTTTGCAAAAAAAAAAAAGTTTTTAGGGCTCTGCGCAATTCTTTCATCT
PHG9_Scaffold_	3254			missense_varian	Mu_g23798.t	PREDICTED: vacuolar	Armadillo/beta-catenin-like	ATTGGTTGTTGCATGCTCAGGAACTTGTTCCTGTTGTCCTTGATAAGGCAAGAGAGGTTAAGGGATTTGCGGGTAGATGGAAGATGATCATTGCGAAATT[G/T]AACAGATCCCGTCGCGATTATCAGATTTGTCAAGCCATCCATGTTTCTCAAAGAATGCTCTGTGCAAGGAGCAGTTGCAGGCTGTGTCAAAGA

5784				t	1	protein 8 [Vigna angularis]	repeat	CACTTGC
PHG9_Scaffold_58	45318			missense_variant	Mu_g01153.t1	Hypothetical protein	NA	TGCTTCTGTTAAATAAAAACTATTCCACGCACACAGACATATACAATTTCAAAGCATTAGACAAGTCGAATTCACCGGAGTATAATTATCCTCCATTGGA[T/A]CGCAGCCTCCGTTTTCTTCCTCGACTCTTCCTCTTTCTCTTCCACCGGTGACTCCAAACGGGAATCGCTTGCCAAATTCTTGTCGCTGCTTCCGTCGTCA
PHG9_Scaffold_5802	67609			missense_variant	Mu_g24022.t1	PREDICTED: putative multidrug resistance protein [Vigna angularis]	ABC transporter transmembrane region	CAACAAACACGTACAAAAGATGTTACCAGAGAGTTAAGAAAAAAATGCTGGAAATTGAGTATGTATATATACCTTATAGGCCATAGTATCTTGAATTGTT[T/C]AGCGTCAGAAGTTATGGTGGCAATAACTCGGAAAGTTGTAGAAGAGTCAGTTTGCTTGTCGAAGAAACCAACCTCTTGCCTTAGAACTGATTTAAGATAT
PHG9_Scaffold_5802	67630			missense_variant	Mu_g24022.t1	PREDICTED: putative multidrug resistance protein	ABC transporter transmembrane region	GTTACCAGAGAGTTAAGAAAAAAATGCTGGAAATTGAGTATGTATATATACCTTATAGGCCATAGTATCTTGAATTGTTTGAGCGTCAGAAGTTATGGTG[G/A]AATAACTCGGAAAGTTGTAGAAGAGTCAGTTTGCTTGTCGAAGAAACCAACCTCTTGCCTTAGAACTGATTTAAGATATTCAATTCTCATGCGGGAAGTT

						[Vigna angularis]		
PHG9_Scaffold_5802	82777			missense_variant	Mu_g24024.t1	hypothetical protein PHAVU_001G256400g [Phaseolus vulgaris]	Pyridoxal-phosphate dependent enzyme	AATCAAAATGGCATGCTGCAAGCTGAAGCGCACCCTAAGCAACGGGCTGCAAGTCCTATACCAACAGCTGTTGTGCCAGTACCAGCATCTACAACAAATT[T/G]ATGGGCCTTTCTTTTCCAAGTAAATGATTCTGTGATAAGTATTGCACTAAGCGACTAACACCTACACAGAAAGAATATTTTCATAAGTTATTGAACAACC
PHG9_Scaffold_5806	77267			missense_variant	Mu_g24073.t1	putative phospholipid-transporting ATPase 9 [Vigna radiata var. radiata]	Phospholipid-translocating P-type ATPase C-terminal	GTTGTGTTGAATTTTGTTAATCAGTGGGATAAAGGATAAAGAATGAGTGGTGGCAGGAAGAGGAAGCTGCGTTTAAGCAAGATCTACTCATTCGCATGCT[G/T]AAGGCATCTTTCAAGGGAGATGATACACAGATTGGAGGAAAAGGTTTTTCCAGGGTGGTGTTCTGCAATGAGCCAGATATCTTTGGGGATGGTGATAGGA
PHG9_Scaffold_	3155			missense_variant	Mu_g24207.t	Scarecrow-like protein	GRAS domain family	TGGACCCAGATTTGAAAATGTCTTCTTTGATCAAAGTAGGAAATTTGATTATTTTCAATCTGATCCAAATCCAATCGCAGCTAACACATCTTCATCCTCC[A/G]TGTGACCCATGAGGAGAACTCTCCAGAGGACTGTGATTTTTCTGATGCAGTTTTGAGTTACATCAGCCAGATCCTAATGGAGGAAGATATGGAGG

5822				t	1	9 [Glycine soja]		ACAAG
PHG9_Scaffold_5831	88549			missense_variant	Mu_g24320.t1	hypothetical protein PHAVU_002G331600g [Phaseolus vulgaris]	NF-X1 type zinc finger	TTTGCCTATTCAGAAGAAAGAATCCCTCGTATCTGCTTCAGTAAACCCTTGGAGTGTCTTAAATCAAGACTCTTCTCCAAGTTCATCGGTTGCAGCTGTT[A/G]AATTGATTTTTCTAGGGAACACTCCGAAAGTAGTGCTGTTACAAAGTTGGAGCCTCATAATGGTGGTTCAAATCTAAGAGGACAGCATGCAGGAAACTTG
PHG9_Scaffold_62	92812			missense_variant	Mu_g01276.t1	ras-related protein RABC1 [Vigna radiata var. radiata]	Ras family	GGAAGGAGAAGGAAACTAGACAGGGGCAAAGAGAAGAGGAACAGACGCCTATTTGGAAGTTGAAGAACTCTGCTCTGAGAATTCTGGATTCTGGCTTTGG[G/A]TTCCGCGGAGTAAGATGGGAAATGGGTGCTCTGCTGCATGCACCCGCCATAGTTGTGTTGTGAGTTAGGAACCACAATCTAATCTAAGGAATCTCTGAAA
PHG9_Scaffold_64	9096			missense_variant	Mu_g01308.t1	PREDICTED: cytochrome b561 and	DOMON domain	GATCCTTGTGCCTAAAATATCTAGCAATAATTGCCCCTACAGGCAGGATTAGACCCCACCCTATTATGCCCAAAATTCCATGGTTCCTCCTCTTCTGAAT[C/G]ATTCATGGGATACAGGACTCGTAGAACCTGTCAAAGTATAGCTCATCAAGTTTCTGCACTCAAAGGTTACATCATAATAATGGCATGCTTCAGACACGAG

						DOMON domain-containing protein At3g61750-like [Vigna angularis]		
PHG9_Scaffold_664	0951			missense_variant	Mu_g06952.t1	hypothetical protein PHAVU_010G152200g [Phaseolus vulgaris]	2OG-Fe(II) oxygenase superfamily	CCACAAGCACCCTGACTTCACCTCTCTACAAGAAAAACTCCCTGACTCTTACGCATGGACACAACCAGATTCTGACCACCATGCTGATTACTATCACCAC[A/T]AAATTACCCTTCCATTAACAACAACACCAACAATGTCCCTGTCATCGATTTCAACGACCCTAATGCTCCAAACCTCATAGCTCATGCATGCAAAACATGG
PHG9_Scaffold_688	939			missense_variant	Mu_g07027.t1	PREDICTED: putative pectinesterase/pectin	Pectinesterase	GACTTGTCTGCTTGGACTCTCAATGCCACCGCTTGATGTTTTTCAGGTCCAGCAGAGTTCTCAAATCCCATGTTGATGGCCACAAAGTAATCTCCTTGAA[C/T]GCTACATGCATAAAGTGATCAATTAATTTATTCAATTAAAATTCCATAAATTACAACTGTTTTATAGTTTTAGTTTTAAAAACTTTTACTCAAATGCATA

						esterase inhibitor 28 [Glycine max]		
PHG9_Scaffold_688	482			missense_variant	Mu_g07027.t1	PREDICTED: putative pectinesterase/pectinesterase inhibitor 28 [Glycine max]	Pectinesterase	AGAATCTTGAAGAAGACGACGCCCAACTAAATTTGTGACATTTGAATTTGTAACAGCATTAGCCAAATCTATTACAATGGCAAGAGCATTGCTTGTCATA[T/A]CATGCCTGTCATTAATAAATCCTTCATCTTCTTACCAGCTTCACTAGTTGTGTTCTCAAACCCATCCAAGCAAGTATCTTGGTACGTAATTGCACCACTA
PHG9_Scaffold_688	5705			missense_variant	Mu_g07028.t1	PREDICTED: putative pectinesterase/pectinesterase inhibit	Pectinesterase	AGAATCTTGAAGAAGACGACGCCCAACTAAATTTGTGACATTTGAATTTGTAACAACATTAGCAAAATATGTTACAATGCCAAGAGCATTGCTTGTCATA[T/A]CATGCCTGTCATTAATAAATCCTTCATCTTCTTACCAGCTTCACTAGTTATGTTTTCAAACCCATCCAAACAAGTATCTTGGTACGTAATTGCACCACTA

						or 28 [Glycine max]		
PHG9_Scaffold_691	1111			missense_variant	Mu_g07062.t1	hypothetical protein PHAVU_001G154100g [Phaseolus vulgaris]	KIP1-like protein	AAGATAGGACGGCTGCAGTTGGAGGTGCAAAGACTGCAATTTTTACTTCTAAAGTTGAATGATGAAAAAGAAGGCAGAGGAAAGACAGTGATGGACGAAC[G/T]AATTCAAAAGTCCTTTTGCGAGATTATCTCTATGGTGGGACGAGAAGAAACTTCCAGAAGACGAAGAAAAAAGCACCCTTTTGTGCATGCATGCAGCCTC
PHG9_Scaffold_70	8978			missense_variant	Mu_g01414.t1	hypothetical protein PHAVU_011G206300g [Phaseolus vulgaris]	Phosphatidylinositol-4-phosphate 5-Kinase	TCTTGTAATATGTCAATGATCCCGAAAAAGATGATGATCTCATATAACTCGCCGGTTGGTTCTCCAACCAGCTGAGGAGTGTCACAACTTTTTCGCACTG[T/G]AATTCGGCCCGTGCCGGCATGTTTATACCTAACTGAATCCACCTGAAACATGAAACAGAATGCAAATGTTTTCAGAACATTCAATGCATGCAACCTTTCC
PHG9_Scaffold_712	492			missense_variant	Mu_g07157.t1	hypothetical protein PHAVU_001	DnaJ domain	CCTGGTTCACTTGTATGGTGTGCATGCTTTTATTATATGTCTAACAAGTGTTACTACATGTTAACAGGAAGTGGTCAAATGTATAAATAATGCAGCTGAA[C/G]TTTAAAAGTGAGGACTTCTGATGCAGCTGTTACAGCTTTAGAACTAGTTTCTAAGGCATTGTCTATAAGTTTATACTCAGAAAAATTGCTGCAAATGAAG

						G129000g [Phaseolus vulgaris]		
PHG9_Scaffold_731	9533			missense_variant	Mu_g07230.t1	PREDICTED: uncharacterized protein LOC100787799 isoform X1 [Glycine max]	WD domain, G-beta repeat	GAAATAGATAGATTATACATTTCAGTTTACTTACGATGGTAAATCATACATGCGAATCGAATTGTCTTTGCAAGAGCAAAATAATATATGTCTATCTTCT[A/G]ATCAGGCATCCCAAAAAGTGAAACAACACCCTATATAAAAAGAAAACAAGAAGTGAATTGACCAACTCCTCTTATTTTTGCAACAAAGGTTGTAGTGATC
PHG9_Scaffold_756	27338			missense_variant	Mu_g07383.t1	PREDICTED: uncharacterized protein LOC108325651 [Vigna angula	Plant invertase/pectin methylesterase inhibitor	CCATGATTACAACTTGCTACTTTTAACAATGGTGTCCATGATGGCAAGGCAATTGCCACCTAACTGGAACAGAGCCGTGGAGTCCTGGGCGAAGGGCATG[G/A]GACTCCAGGAGACTCTGAGAAAGAGTCTTCGCAGGCGGATTTGTACGATATCACAGCGCTCAATTTGCTTTGAGCCTCCACAAAGTTCATCTTCGCCACC

						ris]		
PHG9_Scaffold_758	7741			missense_variant	Mu_g07403.t1	PREDICTED: probable galactinol--sucrose galactosyltransferase 2 [Vigna angularis]	Raffinose synthase or seed imbibition protein Sip1	ACTTACATGAAACATATCCCAGTCAGGTTGCATGAACTCTCCCAGGAAAAGTGTGTTATATGCAACAGATGAAATGTGAATTGTGTGGGAAGCAGGATCA[T/C]GGGATAAAAGTCATCAGATGCTCTCACAACAGCAGTCTGCTTGGAACTATAAAGGCCATCAGTGTTATGACACATACAGGCAATGCATCCGTTGTCAGGA
PHG9_Scaffold_761	9382			missense_variant	Mu_g07442.t1	hypothetical protein PHAVU_003G194700g [Phaseolus vulgaris]	WSTF, HB1, Itc1p, MBD9 motif 1	CAATCCCTCAAACCATGTCTCCGATTCGAAGGAGGAAACCGCCGCGGCTCCTCTCCCAACTACAAGGAGTTCCCGCCCTTCCCGGGCATGCACCATGCGC[G/A]CGCCTCTCGCCTCCATTTCTCGCCGGCGCCGGCGAAGAAGGAGGATTCGCCGCCTCCGCAATGCGGCAAGATTGTGACTCCGCTAGTGGAGCCGCCGTCG
PHG9_Scaf	5677			missense_v	Mu_g0163	ATP synthase beta	ATP synthase alpha/beta	ATTTTATTAACTGTAAAAAAGAAAGACCCTTTCACGACCGCAGAGGAATTCATTCTTCTTCCTCTCTTCGGAGAGCCCTTTCGCGTGGGCGTAGGAAAGA[T/A]AATTCCCAGTCGAAGGTCAAATCCTAGTAAGACGGACTTTGTTGC

fold_78	1			ariant	8.t1	subunit, partial (chloroplast) [Debregeasia saeneb]	family, nucleotide-binding domain	AGCAGATGAAAGCTGCGAATAAGAGAACTGCGACAAGAGAGCAATTCTTGCCCCT
PHG9_Scaffold_78	56865			missense_variant	Mu_g01638.t1	ATP synthase beta subunit, partial (chloroplast) [Debregeasia saeneb]	ATP synthase alpha/beta family, nucleotide-binding domain	GAAAGATCAATTCCCAGTCGAAGGTCAAATCCTAGTAAGACGGACTTTGTTGCAGCAGATGAAAGCTGCGAATAAGAGAACTGCGACAAGAGAGCAATTC[T/C]GCCCCTTCTAACTCCAACCCTGACAGTGAGACCAGTCGAGCCATTTCATACCTTTCTCTATTCCCTATCAGTCGACTTTGTACTCGCTTCGGTCGAGCAA
PHG9_Scaffold_79	1135			missense_variant	Mu_g01659.t1	uncharacterized LOC100816015	zinc-finger of the FCS-type, C2-C2	CTTTCTCATTGCAGATTTCAAGAGAACATTTGATGACCCCTTTGAACTAGTAATTTGGGGGAGAAGTCTCAAACCAACATCCATGTCGGTAATATCAAAT[G/A]TCTTGCATGCTTTCTTTTGTTCACATTCTTCCTTTCTGGGGATTCCACCTTGAAGGGTCTAATTATGAACTTTGAAAGCATGGTGAGTGGAGAAAGAAGA
PHG9_Scaffold_	1141			missense_varian	Mu_g01659.t	uncharacterized LOC1	zinc-finger of the FCS-type, C2-	CATTGCAGATTTCAAGAGAACATTTGATGACCCCTTTGAACTAGTAATTTGGGGGAGAAGTCTCAAACCAACATCCATGTCGGTAATATCAAATGATCTT[G/A]ATGCTTTCTTTTGTTCACATTCTTCCTTTCTGGGGATTCCACCTTGAAGGGTCTAATTATGAACTTTGAAAGCATGGTGAGTGGAGAAAGAAGA

79				t	1	00816015	C2	GAAGGA
PHG9_Scaffold_8	8918			missense_variant	Mu_g00125.t1	PREDICTED: AUGMIN subunit 6 [Vigna angularis]	HAUS augmin-like complex subunit 6 N-terminus	CGATTTTCGCAAGGTATATCGANNNNNNNNNNNNNNNNNNNNNNNNNNNNNNNNNNNNNNATGTAATTTGAATGAATGTGTTTGGATGGCGCTGGAACAGGTT[G/A]GCAAGGGATTATTAGCGAGCTTGAGTCGCAGGGAGCGTTACCGAGGAGTAATTCGAGGGTTTCTTCACTTGCTACCTGCTGTGGACCTAGGTCAGTGTGT
PHG9_Scaffold_843	2726			missense_variant	Mu_g07949.t1	PREDICTED: PI-PLC X domain-containing protein At5g67130-like [Vigna angularis]	NA	GTGGTTCTTGTACACACAGAAAAGAATCAAAGCCGCTAAATTCTAGAAGTACATCACTTTTCTTGCAGAATTACTTTCCAACATATCCAGTTGAAGCTGA[A/C]CATGCAAAGAGCATTCGGCTCCACTTGCTGATATGGTGAACACATGTTACAAAGCTGCAGGAAATGTGTTGCCTAATTTTATAGCAGTTAATTTTTACAT
PHG9_Scaffold_85	1732			missense_variant	Mu_g01743.t1	uncharacterized protein LOC1	NA	ATAGGGTTTTCAATTTGAAAGGCTGCATGCAGATCTTGATGGTGAGAGGTGACATTGTCTATACGACCGCGTTTGAGGGTAATTGCAGAGTGGGAAGAAT[C/A]TCGGCGGTGTCGAATCCTCTTTTGAGGGATGATTCTTTGGCGTCAGAGGTGGATCCTTGGAGGTGATCGAGATTGGGGAATTGAGGGTTAGGTTCAAGAA

						06769554 [Vigna radiata var. radiata]		
PHG9_Scaffold_91	2485			missense_variant	Mu_g01838.t1	hypothetical protein PHAVU_003G232200g [Phaseolus vulgaris]	NA	GTTAGCAAAATAAATATATGAAAAAGCAAGCATGTATGATTTTGCAAGGGGGGAAATAGTCCATTAACTTACACTTGCAATGCATGCATCTTTGACGCTT[A/G]TGTCTTCTTCAAACTTTCTTCACGTTTCCTTGATTCAGTTAATGCAGATTTATTCATTGCAGAGTCATGTTTATGCGTTAACTCGGATGATTCAACCAAG
PHG9_Scaffold_955	4414			missense_variant	Mu_g08473.t1	hypothetical protein PHAVU_010G118800g [Phaseolus vulgaris]	Subtilase family	CATGCAGCTAAGATTGATGTACCAGGAGCAGTGATGTCCGGTTTCAACACAAATGGACAACTATTTGATGGCCCTCTAGAACTGTAAGAATCCACACTTG[G/C]GCTGGTTTAATACCTAAAGCTGTTATCTTAAAAGACATGCTTGCGTTTGCATTAGAGTTACTCTTGATGTAGGCTTTGAGAATTTCTCCATTTCTTGGGT
PHG9	46			missen	Mu_g0	hypothetical	NA	GTATCTGGGCATGACACAAATGGTGTCACTATGGCATCCCAAGATACAAATTTCTTTTGCCCTGCTACCTATGCACAATTTTATTTAGACCAAAATCCA

_Scaffold_96	925			se_variant	2009.t1	protein PHAVU_001G000300g [Phaseolus vulgaris]		T[C/T]CCTCATAATGGTGGGAGATTAAGTGTTTCAAAAGATTCAGATGTTCAATCTACTGGTGGAAATGGACATCAGAATACGCATGCTAGAAGTCCTAAGCAAG
PHG9_Scaffold_981	0042			missense_variant	Mu_g08589.t1	hypothetical protein PHAVU_011G169700g [Phaseolus vulgaris]	recA bacterial DNA recombination protein	GGTCGGCCATCATGTTCGCATTGAATATTGGCATGCTTGAAAGCGGTGACAGCACGAAATCTGAGAGGAAGAGGGAACAATAGAGAAGAAGAGAATGGTG[C/G]TTGAGAATAACACTGTGGGGTTTCAGAGGAAACATCAAATCCATTATTGCAGGAACAAAATTGAAGATACAAAAAGACGTGTTTTTTTGTTGTAACAGA
PHG9_Scaffold_1104	8752			stop_gained	Mu_g09295.t1	hypothetical protein PHAVU_006G199700g [Phaseolus vulgaris]	DNA polymerase phi	CTTACAACAAATTACGAGTTCGGATGCAGCTTCAGAGTACTCCCCTGGACAAAGAAGTACTTGCAGAAGCAGCTGGATAAGCAAGTATCTCAGTGCATGC[A/T]TCTATTAGCACCAGTGCTACAGTCTTGGCTCCTTTCCTGCTTAATATTACCACAAAATATAAAAGAGAAGAATATTATATAAATGAATAAAATAAATGAT

PHG9_Scaffold_111	6693			stop_gained	Mu_g02313.t1	ATP synthase CF1 alpha subunit (chloroplast) [Pachyrhizus erosus]	ATP synthase alpha/beta family, nucleotide-binding domain	TGCTTTGAATTTGGAATCAAAAAATGTTGGTGTTGTGTTAATGGGTGATGGTTTGATGATACAAGAGGGAAGTTCAGTAAAAGCAACAGGAAGAATTGCT[C/T]GATACCAGTAAGTGAGGCTTATTTGGGTCGTGTTATAAATGCCCTGGCTAAACCAATTGATGGTCGAGGAGAAATTTCAGCTTCGGAATCTCGATTAATC
PHG9_Scaffold_1186	5068			stop_gained	Mu_g09774.t1	probable acyl-activating enzyme 18, peroxisomal [Vigna radiata var. radiata]	NA	TGTCAATCTGCATGCACTCACTTCTCATTTTCCTTTTAGGGACCACTCCAAACGCACCAACCTTGGCCGTCTCATGGAAACTCATGCCCCTCAACTTTTA[T/G]ACCTTCTTACGAAGACCCTATTACCAGTTTTCCTCTCTTTCACAAGTTTTCTGTTCAACACCCTCAGGCAAGCAAAGCAACACGACTCTACCCTCACTCC
PHG9_Scaffold_13	31642			stop_gained	Mu_g00209.t1	F-box/kelch-repeat protein At1g57790	F-box-like	ATTTTTATCTTGCATGCTTATAGTTAATACTTATTATTCAGGCTAAGCGACACAATCACAGATAATAGAAGAGCAGTTGATGAAGTGAAAAGTGAAAATT[T/G]GAGCTGCAATCCTGGGCTGATCTCCCTGCTGAACTCTTAGAATTAATCTTGTCCCGATTGATCCTAGCAGATAACATCCGTGCTTCTTCTGTTTGCAAGA

						[Vigna radiata var. radiata]		
PHG9_Scaffold_2271	2997			stop_gained	Mu_g14274.t1	unknown [Glycine max]	Major intrinsic protein	CACCAAATTAACTAGTACTCTTCAGCAGGCACAGGTTCATGCTCAGGAGGCTCAATTGGGACCATAACGTATTCATATAGGACTGCTGCTAGGGCTGCTC[C/A]ATTAATGGGCCCACCCAGAAGATCCAGTGGTAGTTCCACCTCCAGCCCACCAATGCAGGCCCAAAAGCCCGGGCAGGGTTCATGCATGCTCCATCAAAAG
PHG9_Scaffold_2361	79221			stop_gained	Mu_g14616.t1	PREDICTED: alpha-aminoadipic semialdehyde synthase [Vigna angularis]	Saccharopine dehydrogenase C-terminal domain	TCCAAATAAAATTGGTCTTGTAATAAGATTATATTTGTTTGATGATCCAGATCATATGATGGCAATGAAGATGATCAACCAAGCACATGTGCGGAAGGGG[A/T]AATAAAGTCTTTCACTTCTTATTGCGGTGGACTTCCATCACCTGAAGCAGCTAACAATCCTTTAGCATATAAATTCAGGTACTCTTGGAATGGCTTTGTG
PHG9_Scaffold_2462	5520			stop_gained	Mu_g14923.t1	hypothetical protein PHAVU_001G040600g [Phaseolus	Legume lectin domain	AGCATGTAGGGATCAACAATAACTCTGAGGTATCTCTCAATTATACCAGGTTTGATATTGAAACAAACTTAGGTAACATGGGGCATGCCTTGATAACGTA[C/G]ATGCTTCTGCTAAACTCCTTGCTGTCTCGTGGTTCTTCGATGGCACTAGTTCTCGTTTTATGCCTAACGCTTCTCTTTCTTACAAGATTGACCTAGAGGA

						vulgaris]		
PHG9_Scaffold_248	22551			stop_gained	Mu_g04202.t1	PREDICTED: uncharacterized protein LOC107470435 [Arachis duranensis]	Retroviral aspartyl protease	TATCGCTCTGATGGGGGGTAGTGTGGTTGGGACAGCAAAGATGTCATCAAACTCTTGCAACAGTTTCTGTATTACAGGCGAAGGTGCTGGTTCTGGGTTC[C/T]AGAGTGTTGGAGTAGTAATAAATGAAAACATGGCCCAGCGTCTTCTAGTGTTACTAACTGATTTAATTCCCGCAATTTCAAGGGTCTGAAAGTAGGGTGA
PHG9_Scaffold_3158	607			stop_gained	Mu_g16909.t1	PREDICTED: U-box domain-containing protein 33-like isoform X1 [Glycine max]	Protein kinase domain	AGGTCCTTGCATGCTGTTGGAGTGTAACCTTTTTATAGCCACCTCAGTGTATCTCAAGACACCTTTGAATATACTTCCATATCCACCTTCTCCAATTTTT[A/T]GGATGGATTGAAGTTACTTGTTGCTTCTTTAATCTCTGAAAAAGAGAATTCTGAGAAAAATTGAGAGACATGACTGCATGAGGCCTCTCCTTGCTTTTTC
PHG9	00			stop_	Mu_g2	hypothetical	F-box domain	TCAACCTGAGGCATGCCATGCTTGATTTCAGACAAGGACCAAGCTGTGCCGGCTTGAAGGCTCAAGATTTCAACCAAACACTGTTAACGATAAAGAAT

_Scaffold_4826	37			gained	0161.t1	protein PHAVU_007G010900g [Phaseolus vulgaris]		TG[T/A]AAACTCTCACTTTATGTGAATGGACTTTTGAGGTATCTATATGTTCTTTCAATTATTTCCCATCTACTGTTGCTGATTTAACACAATTATCAAGCGAATT
PHG9_Scaffold_4844	3224			stop_gained	Mu_g20330.t1	hypothetical protein PHAVU_001G211800g [Phaseolus vulgaris]	DnaJ C terminal domain	CATATTTCTGAATTTACCTGTCCAGGTCTGTGAGCAATGTCCTAATGTCAAATACGTAAGGGAGGGGTATTTCGTCACTGTTGATATTGAGAAAGGCATG[C/T]AGATGGGCAGGTAATCTTTAAAGCTTATACACAGACTTTAAGTATGCATTTGATGTCTTATTACCTGTTTTCATAAAAAGCTTTCTACCATGGACTATA
PHG9_Scaffold_4972	1150			stop_gained	Mu_g21049.t1	PREDICTED: transcription factor TGA3 [Vigna angularis]	Seed dormancy control	TGAACCACTACATTGAGCTGAACATCAGGCGCTTGCGTTTGAAATGCAAGTCTGAGCTCTTCATTCCGCCTTTGTTGCTCTTCAACCCATTGTCCGTATT[C/A]ATTTCAAAAGCAACAATCCCTGTTTCATATAACAGAAGGAGTACTAAGAAGAATGTAACAAGCTATAATAAGCACAGTGCTGCATGCACAAAGGGAACAA
PHG9	04			synon	Mu_g0	PREDICTED:	DYW family of	TGCACATCTACAAAACTGGGCTTACCAATGATATTTTCTGGTGTAATACTCTTATCAACATATATGTCAGAGTTGGTAACTTGGTTTCCGCACAGAAGCT

_Scaffold_100	28			ymous_variant	2068.t1	putative pentatricopeptide repeat-containing protein At5g09950 [Vigna angularis]	nucleic acid deaminases	[G/C]TTGATGAAATGCCGCAAAAGAACCTCGTTTCTTGGTCTTGTTTGATTTCTGGGTATGCACAAAATGGCATGCCTGATGAGGCATGTGCTTTATTCAAAGG
PHG9_Scaffold_100	8588			synonymous_variant	Mu_g02077.t1	PREDICTED: anthocyanidin 3-O-glucosyltransferase 2-like [Vigna angularis]	UDP-glucoronosyl and UDP-glucosyl transferase	AATTGAGGATGTCAGTAGTAACGGCATCAGCAGTATTGGGCTTGTAGTTTAGAATGGGCCCCACTGGATAAATGGGCTGAGGCCCGTCAAGGAAGGAGTG[A/T]CTGCATGCGATTCTAGCTCCTCGAATGAATTTACTATAATGCCATCGGCTTTCTTCAGATTACCCCCGAAGGAGACAAAAAACGAGTCCCACTCCTTGTT
PHG9_Scaffold_1035	76023			splice_region_varian	Mu_g08933.t1	PREDICTED: probable leucine	Malectin domain	ACATTTGAAGAACAAAACAACTATTAGTCTATATGTTGTACAATAAATTCAATTTGGATATATATCAGAACTAAGTTGTTTCTATTTTGTTTTTGCAGGT[T/G]ATCTATTGAAAGAACAAGGTAATCTAATGGACCTAGTTGATGAGAGGTTGGGTAAAGATTTCAAAAGAAATGAAGTAGTGGTCATGATTAATGTGGCTCT

				t		-rich repeat receptor-like serine/threonine-protein kinase At3g14840 [Vigna angularis]		
PHG9_Scaffold_1049	2639			synonymous_variant	Mu_g08989.t1	PREDICTED: ferredoxin-dependent glutamate synthase, chloroplastic isoform X2 [Vigna angularis]	Glutamine amidotransferases class-II	GGGTAAAAATTTGGGACTTCACCTCAGAGGCAACATAGACCATATTGTCTGATGTTCGCCAATACCTAGCAGGTCTAAGTCCATTACGATCAAGGCATGC[A/T]CAACAGTTTTCCCATCACTGTGACACAAAAATTCGGATTACCAGCAATCACAGAAAAATGATAACTTCGGGTTAAATCAAAACAAAATAAAATAGAAGTA

PHG9_Scaffold_1070	047773			synonymous_variant	Mu_g09083.t1	cysteine-rich repeat secretory protein 56 isoform X2 [Vigna radiata var. radiata]	Salt stress response/ antifungal	AGGGAGGAGGACATCCACACGCGGAGAGGACGGTGGCGCTTGCGGTGGGTGGGGTGGCGGCTCTGGGATTCTTGATTGTTTGCATGCTGTTTCTGAAGTC[G/C]TGTTGAAGAGAAGAGGTGGGAAGCGTTAGGATCAGAGAGTATCATAGTATTATTTGTTGATACTTGTTTCATTCTCACCATTTTGTTCTTAAAAAGTTGT
PHG9_Scaffold_1083	03166			synonymous_variant	Mu_g09172.t1	hypothetical protein TSUD_113870 [Trifolium subterraneum]	Protein tyrosine kinase	CAGTGAAAAGATTGAAAGTTTGGAGCAACAAAGCAGACATGGAATTTGCTGTTGAAGTTGAGATATTGGCTAGAGTACGACACAAGAATCTTCTCAGTCT[A/T]GTGGCTATTGTGCTGAAGGTCAGGAACGATTAATTGTATATGATTATATGCCGAATTTGAGCCTGCTCTCTCATCTTCATGGACAGCACTCATCAGAATC
PHG9_Scaffold_1083	03175			synonymous_variant	Mu_g09172.t1	hypothetical protein TSUD_113870	Protein tyrosine kinase	GATTGAAAGTTTGGAGCAACAAAGCAGACATGGAATTTGCTGTTGAAGTTGAGATATTGGCTAGAGTACGACACAAGAATCTTCTCAGTCTACGTGGCTA[T/C]GTGCTGAAGGTCAGGAACGATTAATTGTATATGATTATATGCCGAATTTGAGCCTGCTCTCTCATCTTCATGGACAGCACTCATCAGAATCCCTTCTTGA

						[Trifolium subterraneum]		
PHG9_Scaffold_1083	03187			synonymous_variant	Mu_g09172.t1	hypothetical protein TSUD_113870 [Trifolium subterraneum]	Protein tyrosine kinase	GGAGCAACAAAGCAGACATGGAATTTGCTGTTGAAGTTGAGATATTGGCTAGAGTACGACACAAGAATCTTCTCAGTCTACGTGGCTATTGTGCTGAAGG[T/A]AGGAACGATTAATTGTATATGATTATATGCCGAATTTGAGCCTGCTCTCTCATCTTCATGGACAGCACTCATCAGAATCCCTTCTTGATTGGAACCGACG
PHG9_Scaffold_1083	03196			synonymous_variant	Mu_g09172.t1	hypothetical protein TSUD_113870 [Trifolium subterraneum]	Protein tyrosine kinase	AAGCAGACATGGAATTTGCTGTTGAAGTTGAGATATTGGCTAGAGTACGACACAAGAATCTTCTCAGTCTACGTGGCTATTGTGCTGAAGGTCAGGAACG[A/G]TAATTGTATATGATTATATGCCGAATTTGAGCCTGCTCTCTCATCTTCATGGACAGCACTCATCAGAATCCCTTCTTGATTGGAACCGACGGATGAACAT
PHG9_Scaffold_	6599			synonymous_v	Mu_g02313.t	ATP synthase CF1 alpha	ATP synthase alpha/beta family,	TACTTCAAGTAGGCGATGGTATTGCTCGTATTTTTGGTCTTGATGAAGTAATGGCAGGTGAATTGGTGGAATTTGAAGAAGGTACTATAGGCATTGCTTT[G/A]ATTTGGAATCAAAAAATGTTGGTGTTGTGTTAATGGGTGATGGTTTGATGATACAAGAGGGAAGTTCAGTAAAAGCAACAGGAAGAATTGCTC

111				ariant	1	subunit (chloroplast) [Pachyrhizus erosus]	nucleotide-binding domain	AGATACC
PHG9_Scaffold_1122	9380			synonymous_variant	Mu_g09411.t1	hypothetical protein PHAVU_004G151200g [Phaseolus vulgaris]	Protein kinase domain	TTGAATGTGCATGAAAAATGAAGTTGAAAATACTTAAATATTGATGTACGTGTGTAATTTTAAATCCTTGCCTTTTGCAGTATACCCCTGCGATTGATAT[T/A]GGAGTATTGGATGCATATTCGCAGAAATGCTCACAGGGAAGCCATTGTTTCCTGGAAAAAATGTAGTGCACCAATTGGATCTCATAACTGACCTGCTTGG
PHG9_Scaffold_1122	9386			synonymous_variant	Mu_g09411.t1	hypothetical protein PHAVU_004G151200g [Phaseolus vulgaris]	Protein kinase domain	GTGCATGAAAAATGAAGTTGAAAATACTTAAATATTGATGTACGTGTGTAATTTTAAATCCTTGCCTTTTGCAGTATACCCCTGCGATTGATATTTGGAG[T/C]TTGGATGCATATTCGCAGAAATGCTCACAGGGAAGCCATTGTTTCCTGGAAAAAATGTAGTGCACCAATTGGATCTCATAACTGACCTGCTTGGTACTCC
PHG9_Scaf	9416			synonymo	Mu_g0941	hypothetical protein	Protein kinase domain	AATATTGATGTACGTGTGTAATTTTAAATCCTTGCCTTTTGCAGTATACCCCTGCGATTGATATTTGGAGTATTGGATGCATATTCGCAGAAATGCTCAC[A/T]GGAAGCCATTGTTTCCTGGAAAAAATGTAGTGCACCAATTGGATC

fold_1122				us_variant	1.t1	PHAVU_004G151200g [Phaseolus vulgaris]		TCATAACTGACCTGCTTGGTACTCCTCCTGCTGAAACCATTTCCAAGGTCAGTTT
PHG9_Scaffold_116	99385			synonymous_variant	Mu_g02405.t1	plant intracellular Ras-group-related LRR protein 6 [Vigna radiata var. radiata]	Leucine rich repeat	TTAGGTATACCACCTTCAACGGACACAGTTCAAGTGGATAAACGAGGAACGTGAGGCATTCATCGTGTCTGATTACAGACCAATCGATAGCCTCGCTTC[G/C]CGCGCTACACGGCCATGTTCTCGCCTCGCCGCCTCTTCTCATCCCGCAATTACTTCTCCACTTGAGCCTCAACGTCGCCGTTTTGCGCTATGAGTAAGAT
PHG9_Scaffold_1187	7262			synonymous_variant	Mu_g09785.t1	PREDICTED: CSC1-like protein At4g35870 [Vigna angula	Late exocytosis, associated with Golgi transport	CCGGGGACGACGGCGACCCCTTCGGCACCTGGTACGGTAACATCGACTACCTCCTCAACATCTCGGCGATCGGGTCCGCCTGCTGCCTCCTGATCTTCCT[C/A]TCGTCAAGCTCCGCAGCGACCACCGCCGCATGCCGGGCCCTGCTGGCCTCGCCTCCAAGCTCCTCGCGGTCTGGCATGCCACCAGCCGCGAAATCGCCCG

						ris]		
PHG9_Scaffold_120	07815			synonymous_variant	Mu_g02523.t1	hypothetical protein PHAVU_007G081900g [Phaseolus vulgaris]	RNA recognition motif. (a.k.a. RRM, RBD, or RNP domain)	TTGTCCACAACTCCTTAACAAAGATTCATATATTCTGTAACTGCGAAAAGGAGATACGGATTACTGAGCCAGAAAGTGTGCTGGCATGCTTGCACACAAG[G/A]CTTCGGTAGCCTGCTCCTCATTCTCAAACTGAACCAGGGCCTGTTTTTTCCCATTCATCTCAAAGACTTTACTGTTGAGTATGGTTCCATGCTCCTCTAC
PHG9_Scaffold_120	07821			synonymous_variant	Mu_g02523.t1	hypothetical protein PHAVU_007G081900g [Phaseolus vulgaris]	RNA recognition motif. (a.k.a. RRM, RBD, or RNP domain)	ACAACTCCTTAACAAAGATTCATATATTCTGTAACTGCGAAAAGGAGATACGGATTACTGAGCCAGAAAGTGTGCTGGCATGCTTGCACACAAGGGCTTC[G/A]TAGCCTGCTCCTCATTCTCAAACTGAACCAGGGCCTGTTTTTTCCCATTCATCTCAAAGACTTTACTGTTGAGTATGGTTCCATGCTCCTCTACAAGGCT
PHG9_Scaffold_120	07884			synonymous_variant	Mu_g02523.t1	hypothetical protein PHAVU_007G081900g	RNA recognition motif. (a.k.a. RRM, RBD, or RNP	CAGAAAGTGTGCTGGCATGCTTGCACACAAGGGCTTCGGTAGCCTGCTCCTCATTCTCAAACTGAACCAGGGCCTGTTTTTTCCCATTCATCTCAAAGAC[T/C]TACTGTTGAGTATGGTTCCATGCTCCTCTACAAGGCTCACAATCTCCTCCTCAGTGATGTCTTGTGGGAGTGTGGACAAATGGATAATCTTTGTTGGGGA

						[Phaseolus vulgaris]	domain)	
PHG9_Scaffold_1213	011			synonymous_variant	Mu_g09876.t1	polyneuridine-aldehyde esterase-like	Alpha/beta hydrolase family	AGTTGATGCCAGAAGCTGCAAGGTCCAGCACTGTGACCTTGTGGCCTGCAGATTCCAACCGTGGCTTGAGCTTGTACCAACACCAAGCTCCATGGCATGC[C/T]CATGCACCAGAACATAGTGCTTCCTTTCTATACTGTTTTCTGAACCCAACATGATGTTAAATTAGTCTTTGTGATTGCTGCTACTGCTGTGTGATCCTTT
PHG9_Scaffold_122	8917			synonymous_variant	Mu_g02539.t1	ABC transporter C family member 10 [Vigna radiata var. radiata]	ABC transporter	GATTTATTGGCATGGCTCTCTCTTATGGCCTTTCACTTAACATGTCCTTGGTATTTTCAATTCAAAATCAATGCAATGTAGCGAATTATATAATATCAGT[G/A]AGAGGCTAAATCAGTATATGCATATACCAAGTGAGGCCCCAGAAGTAATAGAAGGAAATCGTCCTCCTGTGAATTGGCCGGTTGCTGGTCGAGTGCAAAT
PHG9_Scaffold_1236	11989			synonymous_variant	Mu_g10004.t1	hypothetical protein PHAVU_004G081800g [Phaseolus	PPR repeat family	TAGCATTAGGCATGCAACCACTGTCTTCCATTTTTAACCACAAGTCCAATGCCTCATCAACCAATCCTTCTTTACATAATCCATTCATCATAATGTTATA[A/C]TACATACGTTGACCGGGTAGCCTTTAATCAAAAGATTTTGAAAAACTTCTTTTGCATCCTTTAGCCTTCTCCCTTTACACATTCCATCAATAAGTAT ATT

						vulgaris]		
PHG9_Scaffold_1238	3370			synonymous_variant	Mu_g10017.t1	hypothetical protein PHAVU_003G197800g [Phaseolus vulgaris]	Permease family	ACGAGCTAGGCTTTCCTGGGGTAAGTACATATTAAATTGCATGGGTTCAAAGCTAATATAAATATTGATGTCTGGCTGGTTTGTAGGTTGCTAAATGTGT[G/A]AGATTGGATTGCCGGAACTCATATTGCTAGTATTTGTTTCTCAGGTAGAGTTCTTTTTAGTGTCAAAAACTCTTGGCATCATGATCTAAGTTCATTTTGT
PHG9_Scaffold_1238	3395			synonymous_variant	Mu_g10017.t1	hypothetical protein PHAVU_003G197800g [Phaseolus vulgaris]	Permease family	TACATATTAAATTGCATGGGTTCAAAGCTAATATAAATATTGATGTCTGGCTGGTTTGTAGGTTGCTAAATGTGTGGAGATTGGATTGCCGGAACTCATA[T/C]GCTAGTATTTGTTTCTCAGGTAGAGTTCTTTTTAGTGTCAAAAACTCTTGGCATCATGATCTAAGTTCATTTTGTTCTCTTGATTTCCTTACATTTATGG
PHG9_Scaffold_1258	4764			synonymous_variant	Mu_g10180.t1	26S proteasome non-ATPase regulat	26S proteasome subunit RPN7	ATCTGTTTTGGACGCATTGTTTATAGTTTTGTTTCGTTTTGAAATTTATTTCAGCTGGTCTGACCGAGCAGATTAAGTTGGACCGCTATCTGCATCCTCA[T/C]TCCGATATTACATGAGGGAGGTCAGAACTGTTTTGTACTCCCAATTTTTGGAATCTTACAAGAGTGTGACAATTGAGGCCATGGCCAAAGCTTTTGGAGT

						ory subunit 6 homolog [Vigna radiata var. radiata]		
PHG9_Scaffold_128	2771			synonymous_variant	Mu_g02712.t1	magnesium transporter MRS2-2-like	CorA-like Mg2+ transporter protein	TTGCTCTAGATATCTTTGATCCTATGGTGGGGGAGGCAGTAAACCAATTTGCATTTGCAGCACCTGATCCACTGATTGGTGATGATGAACCTGCTTTTCT[T/A]ACAGGTATAGGTCAGCCATATCATCATCATCTTCCATCAATTGTTCAAGCTCGTCTCTGACCTGTACCAGATAAAAATAATTGGTAATGGGAGAGGAAAA
PHG9_Scaffold_128	7875			synonymous_variant	Mu_g02720.t1	PREDICTED: probable LRR receptor-like serine/threonine-protein kinase At1g34110 [Vigna radiata	Leucine rich repeat	CTGACACTGGGATTTCTCCTGTAAAAGAATTGAAGTTCAAGTCCAAACTGATGGTTAAGCTTATAACATGACCAATCTCAGGGGGTATGCCACCAGAGAG[G/A]TGTTGTAACTCAAATCCAGAATAGTTAACTTCTGCAAATTTCTAATGGATTTTGAGATTGATCCTGTCAGTAAATTATTGTTGAGGATGAGCTTGTTCAA

						var. radiata]		
PHG9_Scaffold_128	26806			synonymous_variant	Mu_g02729.t1	laccase-15-like [Vigna radiata var. radiata]	Multicopper oxidase	TGGGAATCTCTGCATGAGGTTGGGGAAAAGGGTAGGGAGTATTCTTGGTTGGATAGACAAAGATAGCACCATGGACAGTGGCTCTTGCCCAGTCACTATG[A/C]CATGCCACCATATTGTCCCTTCCTCGAATGAAAAAACCAACTTTTGTCTGAATTTTCTTCCAGGTTGAATGGGGCACTGAGTGATGTATGCAGGACCATC
PHG9_Scaffold_1292	893			synonymous_variant	Mu_g10335.t1	polygalacturonase 1 beta-like protein 3 [Vigna radiata var. radiata]	BURP domain	CTTCATCTTCTAAAACTGTTGATGGTAGTTCGGTAAAAAGGTGGGTCGAACCAGGTAAGTTCTTCCGCGAGAGCATGCTTAAGGAAGGAACTGTTATGCC[A/C]TGCCAGATATAAGAGATAAAATGCCGCAAAGGTCGTTTTTACCCCGCTCCATTTTGGACAAATTACCCTTCTCTTCTTCGAAGGTTGAGGAGTTGATGAA
PHG9_Scaffold_1292	920			synonymous_variant	Mu_g10335.t1	polygalacturonase 1 beta-like protein 3 [Vigna	BURP domain	GTTCGGTAAAAAGGTGGGTCGAACCAGGTAAGTTCTTCCGCGAGAGCATGCTTAAGGAAGGAACTGTTATGCCAATGCCAGATATAAGAGATAAAATGCC[G/A]AAAGGTCGTTTTTACCCCGCTCCATTTTGGACAAATTACCCTTCTCTTCTTCGAAGGTTGAGGAGTTGATGAAGGTGTTCAAGGTGTCTGATAACTCCTC

						radiata var. radiata]		
PHG9_Scaffold_1292	935			synonymous_variant	Mu_g10335.t1	polygalacturonase 1 beta-like protein 3 [Vigna radiata var. radiata]	BURP domain	GGGTCGAACCAGGTAAGTTCTTCCGCGAGAGCATGCTTAAGGAAGGAACTGTTATGCCAATGCCAGATATAAGAGATAAAATGCCGCAAAGGTCGTTTTT[A/G]CCCGCTCCATTTGGACAAATTACCCTTCTCTTCTTCGAAGGTTGAGGAGTTGATGAAGGTGTTCAAGGTGTCTGATAACTCCTCGATGGAGAAGATGAT
PHG9_Scaffold_1292	947			synonymous_variant	Mu_g10335.t1	polygalacturonase 1 beta-like protein 3 [Vigna radiata var. radiata]	BURP domain	GTAAGTTCTTCCGCGAGAGCATGCTTAAGGAAGGAACTGTTATGCCAATGCCAGATATAAGAGATAAAATGCCGCAAAGGTCGTTTTTACCCCGCTCCAT[T/C]TGGACAAATTACCCTTCTCTTCTTCGAAGGTTGAGGAGTTGATGAAGGTGTTCAAGGTGTCTGATAACTCCTCGATGGAGAAGATGATCATCGACTCTTT
PHG9_Scaf	5150			synonymo	Mu_g1039	protein SIEVE ELEM	Sieve element occlusion	TTTTCCATGATACTAGGAAATTGCTTCAGAATTGCCAATTTTTTTGCAAGCTGGTCGCTATCTTGAACATGAGCAAACAGACAAAATTCACCGTAGGTCA[A/G]GTAAAAGCGGCTAAGGCCAAAACTATCTTCATATCCCACTTGTAC

fold_1298				us_variant	3.t1	ENT OCCLUSION B [Vigna radiata var. radiata]	N-terminus	ATTGTAAGCATGTCTAATACGGCAATTGTTGTCCGGTGTTCATCTACACCATTCA
PHG9_Scaffold_1298	5220			synonymous_variant	Mu_g10393.t1	protein SIEVE ELEMENT OCCLUSION B [Vigna radiata var. radiata]	Sieve element occlusion N-terminus	GAGCAAACAGACAAAATTCACCGTAGGTCAAAGTAAAAGCGGCTAAGGCCAAAACTATCTTCATATCCCACTTGTACATTGTAAGCATGTCTAATACGGC[A/T]TTGTTGTCCGGTGTTCATCTACACCATTCAGAATCTTGTAGGAAATCTGAAATTACATTTTTTATCTTTTTAATTTGTTATATTGATGAAAATGTACTTA
PHG9_Scaffold_130	8900			synonymous_variant	Mu_g02801.t1	hypothetical protein PHAVU_006G107200g [Phaseolus vulgari	GDSL-like Lipase/Acylhydrolase	ACACAAAATGATTAATTATTCAAAGACTGTAACTATTGTGTGTGGTGATGCATACCCTCAGAACTTTGCGGTACTCAGATATGAGATAGCGCACATAGTC[T/C]GGAGAGAAAATTGGCGAGATCTTGCTGAATAGGGCACCAAGTAGTAATTGTTGACAAAATCATTGCCTCCAAGAGTAATGAGGACGAGTGCTCTGTTTAC

						s]		
PHG9_Scaffold_130	8912			synonymous_variant	Mu_g02801.t1	hypothetical protein PHAVU_006G107200g [Phaseolus vulgaris]	GDSL-like Lipase/Acylhydrolase	TAATTATTCAAAGACTGTAACTATTGTGTGTGGTGATGCATACCCTCAGAACTTTGCGGTACTCAGATATGAGATAGCGCACATAGTCTGGGAGAGAAAA[T/C]GGCGAGATCTTGCTGAATAGGGCACCAAGTAGTAATTGTTGACAAAATCATTGCCTCCAAGAGTAATGAGGACGAGTGCTCTGTTTACTAGGTTTCTGGC
PHG9_Scaffold_1389	0257			synonymous_variant	Mu_g10715.t1	secoisolariciresinol dehydrogenase [Vigna radiata var. radiata]	short chain dehydrogenase	AGCATGCAGCTAGAGTGATGATCCCAGCCAAAAAGGGTAGCATAATAACACTAGGAAGTGTTAGCTCAAGTGTTGGAGGGGTTTCAAGTCACGCATACAC[A/T]GTTCTAAACATGCCATTGTGGGACTGGCAAAGAATACAGCAGCTGAGCTTGGACAATTTGGTATCAGAGTGAATTCTCTGTCATGTTATTGCATTAGAAA
PHG9_Scaffold_14	0596			synonymous_variant	Mu_g00233.t1	hypothetical protein PHAVU_002G322100g	GatB/GatE catalytic domain	CAAACGCTGATGCAGCATAAGATCTCACAGCACCACTTGGTCAACTTGCATTTTGAAATTGTGGGTAACCAGAAGCTCTAGTATTGTACTGATTGAGCAT[G/T]CTGCATCTGTGTTGTTTATACCTTGCATTTGAACGGCACCGAATGAATTTCTTCTAGGTTGAGGTAATATTAAACCTTGATTATTGTTCATGCCAAAGTT

						[Phaseolus vulgaris]		
PHG9_Scaffold_1412	606			synonymous_variant	Mu_g10794.t1	hypothetical protein LR48_Vigan02g261100 [Vigna angularis]	Protein of unknown function, DUF617	TGAAACCTTGATATGATCTAACAGATTCAAACTCAACAATTTGAAACCTTGTTGATCCATTTTCTCACTTGCATGGCACATATTAAACCCTAACGAAGAA[G/C]CACTAAGTTCAGGTCCCTTGATATTTCCATCAGGCATCATCATGTAATAAGTCTCAGAGTCTTTAGACCCAACAACACGTTCAAAATGAGCCCTCATGTA
PHG9_Scaffold_1412	9089			synonymous_variant	Mu_g10801.t1	hypothetical protein PHAVU_011G201200g [Phaseolus vulgaris]	Retinoblastoma-associated protein A domain	AAATGACAGTTCGAAGCCACTTTGCTGTAGTCATTGCGGTGCTTACAGGGGTCGCAGCCATCTTTGAGTTTGCACTACCTGGCATGCCATTAGCATATGA[T/C]AGGGGGAGCGGTGAGGTGACAATGGACTTGTAATGGTCTTAGCAGGTGATGCCAGTGAATCAAATTTCCTCTATAGAATACAACAATAGATAATTTTAGG
PHG9_Scaffold_1431	5873			synonymous_variant	Mu_g10888.t1	hypothetical protein PHAVU_010G1650	Protein kinase domain	CAATCTCAATAACATCATTACCATATGCACATACCGTCTGTTGCCTTTCTTGTACATCTTTGCATGCATTTGTGTACATCTCCATTGTTCGCTTTAACTC[C/T]GCTTCAACCTCCTCATATCAGCTTCTACTTCGTCCTGAATAAGGATTAATCATCACCTCTAATACATACTTGGGCTTAAGTATATTTTATTGAATTCAAT

						00g [Phaseolus vulgaris]		
PHG9_Scaffold_1436	5544			synonymous_variant	Mu_g10912.t1	PREDICTED: choline transporter-like protein 2 [Glycine max]	Plasma-membrane choline transporter	TTGGACAAGGCATGCAACTTACAGATGCTTCACCGTGGTTCCAGTAATAAGAGGCGACAGACCCAGCAATCACAGTTGAGGAGCTAGCTATGAAAAATTG[T/A]TAGCCCAATAACACCCAAATAGGTGGAAAAGGATGGCAACTCCAATATGTGGTGTGTAATGAATGCTATAACCACAACAACGATCACAGTTTACCCGTTT
PHG9_Scaffold_144	5821			synonymous_variant	Mu_g03040.t1	PREDICTED: probable serine/threonine-protein kinase Cx32, chloroplastic [Glycine max]	Protein tyrosine kinase	TGGAAATGCGGAACTTGAAGCAGTTCAATTTTGCGGATCTGAAAGCAGCAACCAAGAGTTTCAGGTCTGATGCATTGCTTGGAGAAGGGGGTTTTGGGAA[A/G]TTTACAAAGGATGGTTGCATGAGAAGACACTGACACCCACCAAAGCTGGGTCTGGAATGGTGGTTGCTGTTAAGAAGTTGAATTCCGAGAGTTTGCAGGG

PHG9_Scaffold_1445	9707			synonymous_variant	Mu_g10937.t1	uncharacterized protein LOC106754701 [Vigna radiata var. radiata]	SAM domain (Sterile alpha motif)	GCTGCTTTGAATTAAAACTATTTGTCAGAGGGTGAGTTGCCTTCGAAAGTTTTTCACGGAGATCCATATGCCTCTTACTACCATTGCTGTCTGCATGCTT[A/C]ATGCACTTTTTTGCATGAGCTTCAACCGAAGATCATCTTTAACAATATGTCCATATAAGTAAATTAAGAGGAACAAGATTAGAAATATAAAAATCATTGA
PHG9_Scaffold_145	8987			synonymous_variant	Mu_g03055.t1	hypothetical protein PHAVU_008G082500g [Phaseolus vulgaris]	Leucine rich repeat N-terminal domain	AGGTGGACCAATGTGTTGATGGAAGGCTCCTTGGTAACTTTGCAGCAGAGGAAGCAATTCCTGTGATAAAATTGGGGTTGATTTGTGCATCACAAGTGCC[A/C]CAAACCGTCCAGATATGGCTGAGGTAGTCAACATACTAGAATTAATCCAATGTCCTTCAGAAGGACTAGAGGAATTATAATGAGTTTTGTTTCAGTCTTT
PHG9_Scaffold_1476	07278			synonymous_variant	Mu_g11126.t1	PREDICTED: jasmonic acid-amido synthetase	GH3 auxin-responsive promoter	CTTTTGTTTATATCACTGTTTATAGGGTTTATCCGACAAGGGACGGAGGTAGGATTCTTGAATTCATATACAGCAGCAACCAGTTCAAAACAAAAGGAGG[C/T]TAACAGTTGGAACAGCCACAACACACTACTATGCAAGTGAGGAATTCAAAACCAAACAAGAGAAAACAAAGGCATTCACTTGCAGTCCATACGAAGTTAT

						JAR1-like [Vigna angularis]		
PHG9_Scaffold_1476	07293			synonymous_variant	Mu_g11126.t1	PREDICTED: jasmonic acid-amido synthetase JAR1-like [Vigna angularis]	GH3 auxin-responsive promoter	CTGTTTATAGGGTTTATCCGACAAGGGACGGAGGTAGGATTCTTGAATTCATATACAGCAGCAACCAGTTCAAAACAAAAGGAGGCTTAACAGTTGGAAC[A/C]CCACAACACACTACTATGCAAGTGAGGAATTCAAAACCAAACAAGAGAAAACAAAGGCATTCACTTGCAGTCCATACGAAGTTATATCCGGAGGGGATTA
PHG9_Scaffold_1476	07314			synonymous_variant	Mu_g11126.t1	PREDICTED: jasmonic acid-amido synthetase JAR1-like [Vigna angularis]	GH3 auxin-responsive promoter	CAAGGGACGGAGGTAGGATTCTTGAATTCATATACAGCAGCAACCAGTTCAAAACAAAAGGAGGCTTAACAGTTGGAACAGCCACAACACACTACTATGC[A/C]GTGAGGAATTCAAAACCAAACAAGAGAAAACAAAGGCATTCACTTGCAGTCCATACGAAGTTATATCCGGAGGGGATTACAAACAATCCACGTACTGTCA

PHG9_Scaffold_1476	07320			synonymous_variant	Mu_g11126.t1	PREDICTED: jasmonic acid-amido synthetase JAR1-like [Vigna angularis]	GH3 auxin-responsive promoter	ACGGAGGTAGGATTCTTGAATTCATATACAGCAGCAACCAGTTCAAAACAAAAGGAGGCTTAACAGTTGGAACAGCCACAACACACTACTATGCAAGTGA[G/A]AATTCAAAACCAAACAAGAGAAAACAAAGGCATTCACTTGCAGTCCATACGAAGTTATATCCGGAGGGGATTACAAACAATCCACGTACTGTCACCTCCT
PHG9_Scaffold_1494	085			synonymous_variant	Mu_g11189.t1	hypothetical protein PHAVU_006G099000g [Phaseolus vulgaris]	Proteasome subunit	TGTATGTTTGTTTTCCAGTTCCACGTTTGGTATACCTGCAATTTTTTTCCTTCCAACAATTAAAATTTGTGTATCTTATCCAGGTTAACATGATTGGCAT[A/T]ACTTTGAGGACAACCATGTCGCAACTGGGCTTGGAAATCACCTTGCTAGGCCAATTCTTCGTGATGAGTGGCATGAAAACTTGACCTTCGACGAGGGGGT
PHG9_Scaffold_1494	106			synonymous_variant	Mu_g11189.t1	hypothetical protein PHAVU_006G099000g	Proteasome subunit	CACGTTTGGTATACCTGCAATTTTTTTCCTTCCAACAATTAAAATTTGTGTATCTTATCCAGGTTAACATGATTGGCATAAACTTTGAGGACAACCATGT[C/A]CAACTGGGCTTGGAAATCACCTTGCTAGGCCAATTCTTCGTGATGAGTGGCATGAAAACTTGACCTTCGACGAGGGGGTCAAGTTACTAGAAAAGTGCAT

						[Phaseolus vulgaris]		
PHG9_Scaffold_1494	127			synonymous_variant	Mu_g11189.t1	hypothetical protein PHAVU_006G099000g [Phaseolus vulgaris]	Proteasome subunit	TTTTTTCCTTCCAACAATTAAAATTTGTGTATCTTATCCAGGTTAACATGATTGGCATAAACTTTGAGGACAACCATGTCGCAACTGGGCTTGGAAATCA[C/T]TTGCTAGGCCAATTCTTCGTGATGAGTGGCATGAAAACTTGACCTTCGACGAGGGGGTCAAGTTACTAGAAAAGTGCATGCGTGTGCTTTTATACCGGGA
PHG9_Scaffold_1494	148			synonymous_variant	Mu_g11189.t1	hypothetical protein PHAVU_006G099000g [Phaseolus vulgaris]	Proteasome subunit	AATTTGTGTATCTTATCCAGGTTAACATGATTGGCATAAACTTTGAGGACAACCATGTCGCAACTGGGCTTGGAAATCACCTTGCTAGGCCAATTCTTCG[T/C]ATGAGTGGCATGAAAACTTGACCTTCGACGAGGGGGTCAAGTTACTAGAAAAGTGCATGCGTGTGCTTTTATACCGGGATAGGTCTGCAGTCAACAAAAT
PHG9_Scaffold_1494	184			synonymous_variant	Mu_g11189.t1	hypothetical protein PHAVU_006	Proteasome subunit	TAAACTTTGAGGACAACCATGTCGCAACTGGGCTTGGAAATCACCTTGCTAGGCCAATTCTTCGTGATGAGTGGCATGAAAACTTGACCTTCGACGAGGG[G/C]TCAAGTTACTAGAAAAGTGCATGCGTGTGCTTTTATACCGGGATAGGTCTGCAGTCAACAAAATTCAGGTTTTATACTGTTTCCTTTTCCTCCTCTGCTG

				t		G099000g [Phaseolus vulgaris]		
PHG9_Scaffold_1494	193			synonymous_variant	Mu_g11189.t1	hypothetical protein PHAVU_006G099000g [Phaseolus vulgaris]	Proteasome subunit	AGGACAACCATGTCGCAACTGGGCTTGGAAATCACCTTGCTAGGCCAATTCTTCGTGATGAGTGGCATGAAAACTTGACCTTCGACGAGGGGGTCAAGTT[A/G]TAGAAAAGTGCATGCGTGTGCTTTTATACCGGATAGGTCTGCAGTCAACAAAATTCAGGTTTTATACTGTTTCCTTTTCCTCCTCTGCTGTGGTCTTCC
PHG9_Scaffold_1497	2159			synonymous_variant	Mu_g11206.t1	Leucine-rich repeat protein soc-2-like protein	NB-ARC domain	TGCGAAAGTTGTCTCTTTATAGTTGTAACACGAGGGAGGCTTTCGAAAGTGACAGCATTCCAATTTCTGAGGCACTGCCAAATCTAGTGGAATTGTGTAT[T/C]ATTATTGCAAAGATTTAGTGACACTACCCAATGGATTGTGTGATATTACTTCTATCGAGAAGCTTAGTATCACTAGGTGCATGCGTTTTATTGCGTTGCC
PHG9_Scaffold_1515	0142			synonymous_variant	Mu_g11264.t1	18.5 kDa class I heat shock protein	Hsp20/alpha crystallin family	CATCTTACTGCAACCTGTGAAGTTTCAAATCTCTTCCAAGTATTATAAGCATGTCGCTAATTCCAAGTTTCTTCGGTGGCAGAAGGAGCAACGTCTTTGA[C/T]CTTTCTCATTGGACGTGTGGGATCCCTTCAAGGATTTTCCTTTTCCCAATTCTCTTTCTGCATCCTTCCCTGAATTTTCTCGGGAAAATTCTGCATTTGT

PHG9_Scaffold_1515	0151			synonymous_variant	Mu_g11264.t1	18.5 kDa class I heat shock protein	Hsp20/alpha crystallin family	GCAACCTGTGAAGTTTCAAATCTCTTCCAAGTATTATAAGCATGTCGCTAATTCCAAGTTTCTTCGGTGGCAGAAGGAGCAACGTCTTTGACCCTTTCTC[A/C]TGGACGTGTGGGATCCCTTCAAGGATTTTCCTTTTCCCAATTCTCTTTCTGCATCCTTCCCTGAATTTTCTCGGGAAAATTCTGCATTTGTGAGCACCCA
PHG9_Scaffold_157	7852			synonymous_variant	Mu_g03282.t1	PREDICTED: multiple organellar RNA editing factor 1, mitochondrial-like [Vigna angularis]	NA	AGAGAAGTGAAATTGAGGATGACCTGAACGAACGGCGCTGAACCACGCCCTTCGGAGAGTGGCAACTCCATGAAACGGGGGCGGAAAACGACGCCGCATG[C/A]ACGCGGAGATGGACTGGGTGATGCTGGAGAGTGCTGTGAGGGTACGGCGGAGGCGAATTAAGGACGACGCCATGGGAGAGTGTAGGGTTTTGGAAGATTA
PHG9_Scaffold_1736	2998			synonymous_variant	Mu_g12243.t1	organic cation/carnitine transporter 3 [Vigna radiata	Sugar (and other) transporter	GAGATAGTGGCATGCCATAATAGACCAATCCAATACCTATTCCCATAGCCATGATTGACACAAGCCTTCTCGAAGACCATTTCTTCTGTAAAAGAATTTT[G/T]GGGCAGAGAACAAATCCTCGTTCCAAGTTTCTTCCTTGTAGGACATGTTACTAATGGCCAAATCGAGGTTGCTTAGAGTGACTGATGTGATGCATTTCAG

						var. radiata]		
PHG9_Scaffold_1745	1603			synonymous_variant	Mu_g12303.t1	hypothetical protein PHAVU_004G119800g [Phaseolus vulgaris]	Ankyrin repeats (many copies)	ATGCTTGTGACTGGCGAGGCGATTCATCTCGTGTTTCTGCTTCCCGGGGTTCAAATGATGGACAAGGACCATCATTACAAGTACACAGAGGAATTGCTAC[T/A]ATGGAAATGGAGTACAAGTGACAGGGCAAAGCATCTCTGCCGGAACTGAAGCTACACGGTTCCTTCTAATCTCAGGGGTATGAGAAAGAGACGAGCGATC
PHG9_Scaffold_1766	880			synonymous_variant	Mu_g12458.t1	PREDICTED: protein AIG1-like [Glycine max]	AIG1 family	GTCCAAACAATGCTTGCAATGCAAGAAAAGTAGCATATTCCTCTTCAGAAAAACGAGTTTTCACTGAAAATACCATCAGAATTGCATGAATTCCATCTTT[T/A]CCATATCAATACATTTCACTATTTCTTTCCCAGCTGAATGAGTTCCATCATAAAGTCCTGCAAAACTTGTCAAGATATTTTGATTCAGAATTAAATCTAT
PHG9_Scaffold_1766	892			synonymous_variant	Mu_g12458.t1	PREDICTED: protein AIG1-like [Glycine max]	AIG1 family	CTTGCAATGCAAGAAAAGTAGCATATTCCTCTTCAGAAAAACGAGTTTTCACTGAAAATACCATCAGAATTGCATGAATTCCATCTTTTGCCATATCAAT[A/G]ATTTCACTATTTCTTTCCCAGCTGAATGAGTTCCATCATAAAGTCCTGCAAAACTTGTCAAGATATTTTGATTCAGAATTAAATCTATTTTATGTTCATT

PHG9_Scaffold_178	8155			synonymous_variant	Mu_g03786.t1	PREDICTED: uncharacterized protein LOC106773219 [Vigna radiata var. radiata]	Cellulase (glycosyl hydrolase family 5)	CAAACACCAGTTTCTTACCCAAATTTATCTTCAATGGTTTATTCTTTAAATAGTGCAAGTCAGAATCATAACCTATACCTGAAATAACCACAAGCACATT[T/C]GGTTTGTCTTATGTACAGCTAATGCTGCTTGGGTCATATACTTGTACCAATCAGGCAAGTTTGGCGTGGACCACGCAATTCATTCCTCAAACTCATTGC
PHG9_Scaffold_178	8178			synonymous_variant	Mu_g03786.t1	PREDICTED: uncharacterized protein LOC106773219 [Vigna radiata var. radiata]	Cellulase (glycosyl hydrolase family 5)	TTTATCTTCAATGGTTTATTCTTTAAATAGTGCAAGTCAGAATCATAACCTATACCTGAAATAACCACAAGCACATTTGGGTTTGTCTTATGTACAGCTA[A/G]GCTGCTTGGGTCATATACTTGTACCAATCAGGCAAGTTTTGGCGTGGACCACGCAATTCATTCCTCAAACTCATTGCCACAACCTATTAGTGAACCAAAA
PHG9_Scaf	8215			synonymo	Mu_g0378	PREDICTED: unchar	Cellulase (glycosyl hydrolase	CAGAATCATAACCTATACCTGAAATAACCACAAGCACATTTGGGTTTGTCTTATGTACAGCTAATGCTGCTTGGGTCATATACTTGTACCAATCAGGCAA[G/A]TTTGGCGTGGACCACGCAATTCATTCCTCAAACTCATTGCCACAA

fold_178				us_variant	6.t1	acterized protein LOC106773219 [Vigna radiata var. radiata]	family 5)	CCTATTAGTGAACCAAAAGTAATAATAAAGTCATAATAATAAAGATAAAGTATTA
PHG9_Scaffold_178	8890			synonymous_variant	Mu_g03786.t1	PREDICTED: uncharacterized protein LOC106773219 [Vigna radiata var. radiata]	Cellulase (glycosyl hydrolase family 5)	CAACAATCTCTTTCAAAGGCCTTTTATCAAGACCCTCAGGGATCATTGGTTGGAGGTGACCAGCCCAATTGGCACATGCCAACTTGGTTCGTTGTCCTGT[G/A]CTTCATCTATGATCCATCTCTTATGTGTTGATAAAGGATATGTATTGGAATATGATGTAGAGATGAATAAAAGGAGAAGAAAACCAATTCGTGAAAT
PHG9_Scaffold_178	8899			synonymous_variant	Mu_g03786.t1	PREDICTED: uncharacterized protein	Cellulase (glycosyl hydrolase family 5)	CTTTCAAAGGCCTTTTATCAAGACCCTCAGGGATCATTGGTTGGAGGTGACCAGCCCAATTGGCACATGCCAACTTGGTTCGTTGTCCTGTGGCTTCATC[T/A]TGATCCATCTCTTATGTGTTGATAAAGGATATGTATTGGAATATGATGTAGAGATGAATAAAAGGAGAAGAAAACCAATTCGTGAAATTATTCTAAA

						LOC106773219 [Vigna radiata var. radiata]		
PHG9_Scaffold_178	8929			synonymous_variant	Mu_g03786.t1	PREDICTED: uncharacterized protein LOC106773219 [Vigna radiata var. radiata]	Cellulase (glycosyl hydrolase family 5)	GGATCATTGGTTGGAGGTGACCAGCCCAATTGGCACATGCCAACTTGGTTCGTTGTCCTGTGGCTTCATCTATGATCCATCTCTTATGTGTTGATAAAGG[A/G]ATGTATTGGAATATGATGTAGAGATGAATAAAAGGAGAAGAAGAAAACCAATTCGTGAAATTATTCTAAACATCTTCGTTTTGAACGGAAAATAAATTTT
PHG9_Scaffold_1812	1477			synonymous_variant	Mu_g12691.t1	hypothetical protein PHAVU_006G017100g [Phaseolus	Lipoxygenase	TAAAAGCAAGTGATGCAATAAGCAGTTTTCCAGCATTATCAAATTTAAAAATAAGCACTTGTAATAAGTTCTTACTTTTAATTGTTTTGGGGGTATCAAA[G/A]GCAAAAAGTTCTGGCTATCATTAGCAACCTTGATCAACTTGGGAATGGCACTCTGGAGGAAATTTAGACTATTGTCTTTAAGAGTTGGTAAGTGAAACCC

						vulgaris]		
PHG9_Scaffold_1853	624			synonymous_variant	Mu_g12896.t1	hypothetical protein PHAVU_004G175900g [Phaseolus vulgaris]	Protein kinase domain	TGATTCTGTGCATGCTGGCAGTAGCCTTCTTGCTGGCTTTGTTTATAGCAATCTTGATTACTAAACTTTACAGAAGAAGGAAACAAGGATTGGATAATTC[A/T]GGAAACTCATTTCATTTCAAAGGCTGAGTTTCACTGAATCAAACATAGTGTCATCAATGACAGAACATAATGTTATTGGAAGTGGTGGATTTGGCACAGT
PHG9_Scaffold_1901	3251			synonymous_variant	Mu_g13059.t1	PREDICTED: nucleolar and coiled-body phosphoprotein 1 isoform X1 [Vigna angularis]	SRP40, C-terminal domain	CTACAATCTTTGTTTTCACCCCTCGTCAAGTCTTCTTAAGCAACCAAACCATGAAGCACACTGTTCAGAACGGAACTCCAAACTCCAAGCGGGAAACCAC[G/A]TTCTCCTTCACCAATCCATTGCCCATTACTTGGAACTCAGTGGCTTCAACAAGACGCTAAAGAAATTCCGCTCTGAAGCGCAAATTGAGGTCGGTTACAT
PHG9_Scaffold_	18511			synonymous_v	Mu_g03942.t	PREDICTED: probable	DHHC palmitoyltransferase	TTTCTGAGATTACTTTTCAGTTATGATTTACTTTGTATCATTCTTTAATATGCTTTATGTATTCTAATTGTTTGCACATTGTTATGCAGCGTAATTATCG[G/C]TCTTCTTCATGTTTGTCTTCTCAACTACGCTACTGTGTATATATGTTTTTGCGTTTTGCTGGGTTTATATTGTAAGAATTATGACAGCAGAAGAGACA

201				ariant	1	protein S-acyltransferase 7 [Vigna angularis]		AC
PHG9_Scaffold_201	18541			synonymous_variant	Mu_g03942.t1	PREDICTED: probable protein S-acyltransferase 7 [Vigna angularis]	DHHC palmitoyltransferase	CTTTGTATCATTCTTTAATATGCTTTATGTATTCTAATTGTTTGCACATTGTTATGCAGCGTAATTATCGGTTCTTCTTCATGTTTGTCTTCTCAACTAC[G/A]TACTGTGTATATATGTTTTTGCGTTTTGCTGGGTTTATATTGTAAGAATTATGACAGCAGAAGAGACAACAATTTGGAAGGCAATGATAAAAACTCCAGC
PHG9_Scaffold_201	18547			splice_region_variant	Mu_g03942.t1	PREDICTED: probable protein S-acyltransferase 7 [Vigna angula	DHHC palmitoyltransferase	ATCATTCTTTAATATGCTTTATGTATTCTAATTGTTTGCACATTGTTATGCAGCGTAATTATCGGTTCTTCTTCATGTTTGTCTTCTCAACTACGCTACT[G/T]GTATATATGTTTTTGCGTTTTGCTGGGTTTATATTGTAAGAATTATGACAGCAGAAGAGACAACAATTTGGAAGGCAATGATAAAAACTCCAGCGTCCAT

						ris]		
PHG9_Scaffold_201	18571			synonymous_variant	Mu_g03942.t1	PREDICTED: probable protein S-acyltransferase 7 [Vigna angularis]	DHHC palmitoyltransferase	ATTCTAATTGTTTGCACATTGTTATGCAGCGTAATTATCGGTTCTTCTTCATGTTTGTCTTCTCAACTACGCTACTGTGTATATATGTTTTTGCGTTTTG[C/T]GGGTTTATATTGTAAGAATTATGACAGCAGAAGAGACAACAATTTGGAAGGCAATGATAAAAACTCCAGCGTCCATAGTATTAATAATTTACACCTTTAT
PHG9_Scaffold_201	18577			synonymous_variant	Mu_g03942.t1	PREDICTED: probable protein S-acyltransferase 7 [Vigna angularis]	DHHC palmitoyltransferase	ATTGTTTGCACATTGTTATGCAGCGTAATTATCGGTTCTTCTTCATGTTTGTCTTCTCAACTACGCTACTGTGTATATATGTTTTTGCGTTTTGCTGGGT[T/C]ATATTGTAAGAATTATGACAGCAGAAGAGACAACAATTTGGAAGGCAATGATAAAAACTCCAGCGTCCATAGTATTAATAATTTACACCTTTATATCGAT
PHG9_Scaffold_2064	3090			synonymous_varian	Mu_g13667.t1	mitochondrial inner membrane	60Kd inner membrane protein	TAGCTTCTCAGGCTGCTACTGTTAATGAAGTTGCAATTGCTGCCGCTGATTCTTTTTTGCCTGTAATGGGCTTGCAGTACGTGATAGATGCTGTGCATTC[C/T]ACACTGGCTTAAATTGGTGAGTCTATCTCTCTCTCTCTTGCACGATATATATATACAGACACACATTGGCTTTCAATGGTGATTCTTAATCTTATTTCTT

				t		protein OXA1 [Vigna radiata var. radiata]		
PHG9_Scaffold_2129	936			synonymous_variant	Mu_g13896.t1	hypothetical protein LR48_Vigan10g032900 [Vigna angularis]	MatE	ATTTTGTTGAAAAAAGAATATCATACCAGACCATGAGTGCTGACGGAAAGGCAAGCCTGAGAAATTGAGGAATATTATGCAAGGATTCCCTTGAGAAACC[C/A]TCCAAGTGCTTTTACAGGAAGAGGAGAGCCTGATGTAAAGTGCTAGTAGCACTGTGTTAAGCCAATTTGAAATGCAAAATGCAATGGCAGCTCCTTTCAT
PHG9_Scaffold_2129	945			synonymous_variant	Mu_g13896.t1	hypothetical protein LR48_Vigan10g032900 [Vigna angularis]	MatE	AAAAAAGAATATCATACCAGACCATGAGTGCTGACGGAAAGGCAAGCCTGAGAAATTGAGGAATATTATGCAAGGATTCCCTTGAGAAACCCGTCCAAGT[G/A]TTTTACAGGAAGAGGAGAGCCTGATGTAAAGTGCTAGTAGCACTGTGTTAAGCCAATTTGAAATGCAAAATGCAATGGCAGCTCCTTTCATGCCGAGCCT
PHG9_Scaffold_	951			synonymous_v	Mu_g13896.t	hypothetical protein LR48_	MatE	GAATATCATACCAGACCATGAGTGCTGACGGAAAGGCAAGCCTGAGAAATTGAGGAATATTATGCAAGGATTCCCTTGAGAAACCCGTCCAAGTGCTTTT[A/G]AGGAAGAGGAGAGCCTGATGTAAAGTGCTAGTAGCACTGTGTTAAGCCAATTTGAAATGCAAAATGCAATGGCAGCTCCTTTCATGCCGAG

2129				ariant	1	Vigan10g032900 [Vigna angularis]		CCTAAGTTT
PHG9_Scaffold_2231	628			synonymous_variant	Mu_g14179.t1	hypothetical protein PHAVU_002G333600g [Phaseolus vulgaris]	NA	TTAATTTGCTTGATTTTTACTTGAGTCGTTACTTACAAAAATTGAGCGAGTCTAAGATTGCAGAATCTCAAAGAGACTTATCAGAGGCAAAATCTCTGGT[C/G]AGGAAGCAGAACGCTCTCTATTGCTAAATGTGGGAGGTCCTGACGGTGGCAGTACATCCATGAGAATGAAAAGTGAAGAAATTGATCGAGATGCAGAGAG
PHG9_Scaffold_2276	3211			synonymous_variant	Mu_g14277.t1	hypothetical protein PHAVU_009G204200g [Phaseolus vulgaris]	Cupin	CTCTTTCTCTCTCTTATTTTTTGGGTCCACTGAAAAAGAATATGGAGATTGATCTTTCTCCCCAGTTGGCGAAGAAAGTTTATGGAGGCAATGGTGGGTC[C/T]ACCACGCCTGGTCCCCCTCTAAGCTTCGCATGCTTCGCCAAGGCAACATTGGTGCCGTCAAACTCTCTCTACACAAAAATGGTTTCGCCCTTCCTCGTTA
PHG9_Scaf	2960			synonymo	Mu_g1444	hypothetical protein	Dirigent-like protein	GTATATTTATAAGCAAGCGAATGATGGAGGTTGCAGTTTGTGGATCATACACCACAGTTTGCAATGTAGCATAGCCCCTTACAAAACGAAATTTACCAGT[T/C]CAGACACCACAGAGACTTCTCTAACATTCTCATGTTGGCGACTA

fold_2322				us_variant	6.t1	PHAVU_011G098600g [Phaseolus vulgaris]		ATGCCTTGGAGCTCCAAGGTGCTACCATTGTACTGCGAATTTTGTCGAACACAAT
PHG9_Scaffold_2330	829			synonymous_variant	Mu_g14489.t1	solute carrier family 40 member 2 [Vigna radiata var. radiata]	Ferroportin1 (FPN1)	CCCATCTTCCAATTATGGGACCAAACAGTGCAATGGAGGCAGATTCAATGGCACCATATATTGCTGCATACAGCAAAGAGTCTGGCCATATGTTGATCAT[A/G]ATAAACCAACAGAGAATTCCCACATCCTGAAAACTTAGATGCTCAGTGAATTTAACTAGTAAAAAGCAAACGAAAGCAAACAAAATAGTGGAACCCATTT
PHG9_Scaffold_2354	7935			synonymous_variant	Mu_g14577.t1	hypothetical protein PHAVU_005G017000g [Phaseolus vulgaris]	Choline/ethanolamine kinase	TTTTGGAGAAGGAACTATCAGGCTCACACCAAAGGATAGGATTTTGCCACAATGATTTACAATATGGTAACATAATGCTTGATGAAGAGACCAATTCTGT[G/C]CCATCATAGTGAGTTTCTCTTTGTGCTCGTTTATGGGCGAAATAATATAATATTAATGTTTCAAGGGTGCTGCACTGTGGAAATAATTAGTTCCTCTATC

PHG9_Scaffold_2354	7941			synonymous_variant	Mu_g14577.t1	hypothetical protein PHAVU_005G017000g [Phaseolus vulgaris]	Choline/ethanolamine kinase	AGAAGGAACTATCAGGCTCACACCAAAGGATAGGATTTTGCCACAATGATTTACAATATGGTAACATAATGCTTGATGAAGAGACCAATTCTGTGACCAT[C/A]TAGTGAGTTTCTCTTTGTGCTCGTTTATGGGCGAAATAATATAATATTAATGTTTCAAGGGTGCTGCACTGTGGAAATAATTAGTTCCTCTATCATAATA
PHG9_Scaffold_2365	09177			synonymous_variant	Mu_g14691.t1	PREDICTED: inositol-3-phosphate synthase-like [Glycine max]	Myo-inositol-1-phosphate synthase	GAAACCTTTTGCATGCAAACATTTATGGGTCATTTTTGTGTTTTATGATTCTCAGGGGCATTTCATGGGCAACAAAGGACAAGATTCAACAAGCCAATTA[C/T]TTGGCTCCCTCACCCAAGCTTCCGCCATCCGAGTTGGGTCTTTCCAGGGAGAGGAAATCTATGCCCCATTCAAGAGCCTTCTTCCAATGGTATTCTATCA
PHG9_Scaffold_239	4049			synonymous_variant	Mu_g04125.t1	putative purine permease 4 [Cajanus cajan]	NA	CCATGAATGAAGAAGAAAGGTTTGGAATTGATTCTTCTGTATGAACAGTTTCAATATCGTTTGTGCTGTTCAGAAAAGTGCTTGTATTCAGAAACTGATT[A/G]TCAATCCTGTGGATTGAGATGTGGCTGCCATGGATGAAGAATTGTTTTTGTTTTTTGATCTGCAATAGAAGAGGATTGATTTGCAATAGGTAAAACTATC

PHG9_Scaffold_2461	213			synonymous_variant	Mu_g14912.t1	hypothetical protein PHAVU_008G214000g [Phaseolus vulgaris]	Domain of unknown function	TTGGCAATCTAGCCGAGTACACGTTGGAAGGGAAATGGAGTAAGGTTGTGGAAATGTACAGACAATTCCCAGCGTGTCACACGGCAATGATTAACGATTC[C/T]TGGGCACTGCACTGCACGTGGCGGTGGATTTGGACGAAGAAGGTGTGGTTCAAGACCTTGTGAATGCAATTATCGATTACAGCAGTGAAGCGAAGATCAA
PHG9_Scaffold_2461	264			synonymous_variant	Mu_g14912.t1	hypothetical protein PHAVU_008G214000g [Phaseolus vulgaris]	Domain of unknown function	AAATGTACAGACAATTCCCAGCGTGTCACACGGCAATGATTAACGATTCCGTGGGCACTGCACTGCACGTGGCGGTGGATTTGGACGAAGAAGGTGTGGT[T/C]AAGACCTTGTGAATGCAATTATCGATTACAGCAGTGAAGCGAAGATCAAGGCTTTAGAAATGGGGAACGATCGAGGGGATACCCCTCTGCATGTTGCAGC
PHG9_Scaffold_2486	445			synonymous_variant	Mu_g15019.t1	early nodulin-like protein 3 [Vigna radiata var. radiata	Plastocyanin-like domain	CCTATGAATTCGTAGTTGGAGGTCAAAAGGGTTGGAGTGTTCCAAGTAACCCCAATTCCAATCCTTATAGTCAATGGGCACAAAAGAGCCGATTTCAAAT[A/T]GAGACTCCTTAGGTAACTTCTTATTCAAACTCAACTCACCAACCTTGTGTATTTCTTCATTGGTTTTAACTTCAGTCTAAGTCAAATAATTTTGTGAAAC

						]		
PHG9_Scaffold_2502	6462			synonymous_variant	Mu_g15100.t1	uncharacterized protein LOC106774339 [Vigna radiata var. radiata]	CRS1 / YhbY (CRM) domain	AGGAAAAGGGGTTGATTGTGAATTATTTTTGTTCAGAAATGTTGTACCATTAGCTCACACACCTGAGTGCAGCAACATTGTTAAAGTTGAAGAGGCATG[C/T]AGAGAGTGGGTTTGTATTGCTATGTTAAGCAGTTGAATCTTTTGAATGGATCATTGTTGTCCCGGGCAATGTCAACTTCCAAAGGAAGGAGTATGCGCAG
PHG9_Scaffold_2533	0013			synonymous_variant	Mu_g15169.t1	PREDICTED: probable protein phosphatase 2C 74 [Vigna angularis]	Protein phosphatase 2C	GACCAATGAAGGTGCTTGAAGGGATGTTATTGTTCCCACTCACATTGGATCATGATTGGGTTAAAACACTCTAATCCTTCAGGAACAAGCATACAGCTGT[A/G]AATCATCAACCTTGAAACCATACTTAAATCTCCAAGCCTGAACTGCATGCTTTACTAACAATTTGGCTGCCATGCACCTTTTTGGTGCTGAAGCAACTAT
PHG9_Scaffold_2533	0025			synonymous_variant	Mu_g15169.t1	PREDICTED: probable protein phosph	Protein phosphatase 2C	TGCTTGAAGGGATGTTATTGTTCCCACTCACATTGGATCATGATTGGGTTAAAACACTCTAATCCTTCAGGAACAAGCATACAGCTGTACAATCATCAAC[C/T]TGAAACCATACTTAAATCTCCAAGCCTGAACTGCATGCTTTACTAACAATTTGGCTGCCATGCACCTTTTTGGTGCTGAAGCAACTATGTTTATTACTTC

						atase 2C 74 [Vigna angularis]		
PHG9_Scaffold_2533	0037			synonymous_variant	Mu_g15169.t1	PREDICTED: probable protein phosphatase 2C 74 [Vigna angularis]	Protein phosphatase 2C	TGTTATTGTTCCCACTCACATTGGATCATGATTGGGTTAAAACACTCTAATCCTTCAGGAACAAGCATACAGCTGTACAATCATCAACCTTGAAACCATA[C/T]TAAATCTCCAAGCCTGAACTGCATGCTTTACTAACAATTTGGCTGCCATGCACCTTTTTGGTGCTGAAGCAACTATGTTTATTACTTCACTGTTTGTTAG
PHG9_Scaffold_2574	951			synonymous_variant	Mu_g15377.t1	hypothetical protein LR48_Vigan01g070900 [Vigna angularis]	Terpene synthase family, metal binding domain	TATGGACATTTGGTTTCAATGCAAAAGTAAAATGGTTTTTACTACAGTAGGCATGCTGAGGGATGAGCTTTATAGATGAGCATTTGTTTGGAGAAAATAT[A/G]GGTGATTTGGAGGCACAAAGATAGAGTGAGTGAAAGCCATAGGTGTAGTATATATTAAGGTTTGGTGTTGTTGAGGAAGATGAATGGGAACACAAGAGGA
PHG9_Scaffold_2661	2384			synonymous_varian	Mu_g15618.t1	PREDICTED: AT-rich interac	ARID/BRIGHT DNA binding domain	TGAGGCCAATCCCTCAATCTCAGAGACACAGGATCCTCCCCTTTTCTGGATTGTCTGTGGGCACAAGATATGATGGAGATCATGGAAAATCAAATCAGCC[G/T]ATGCTCCTATTAAGCATAATGTTTCAGGCCTTACTTCAGTTACAAATAGGAAATCCGTGTCGAACTCTTTTCAAGCTAACCAAATTATTTCTTTGAATCC

				t		tive domain-containing protein 4 isoform X1 [Vigna angularis]		
PHG9_Scaffold_2747	11157			synonymous_variant	Mu_g15799.t1	O-acyltransferase WSD1 [Glycine soja]	Protein of unknown function (DUF1298)	ATTACTTTCATAAATTTTTCCTCCATCAATTTCGGTGTTGTTTAGTAAGTTGAATCAACTATATTATTACCTTAGGGTGTCCATATACACTAGGAGCAAT[G/A]ATGCCACGGGATGACCATATAAGCTAATTTCTTCCATGGGACCAGGCATATTAGAGAAGGCTATGGTAGTGTTAAAGAGAACTTTGTGTGTTATGGCAGC
PHG9_Scaffold_2747	11565			synonymous_variant	Mu_g15799.t1	O-acyltransferase WSD1 [Glycine soja]	Protein of unknown function (DUF1298)	CTATCTTTGCACAAGCGTAGGAGCAAATTGCTTCCAATGAGTGCTTTTTTCGGTCGATTCTATCCTTAGCTTTACGAACATATTCCAATGGATCTTCATA[T/C]AAGCAATATAGAATGGGAGGATGATGTATCCCAAGCAATTAGCCCATTTTACTTTTGATTTCTCTGTCATCATATCTGCTAGATCCTAATTAGCACAATA
PHG9_Scaf	5779			synonymo	Mu_g0448	hypothetical protein	NA	ATGGAAAGGTAGGCTGTGGCAGTGTTGGGATTGCAGGCAATGTTGGGTTAGGCAAAGAGGGAATGCCTGGGAAATTTGATGCAGTTGCTTGCATAAGATG[G/A]GAGCTGCTAGGCTCATGCTCATGCTTGAGAAGGTCAGAGCAA

fold_288	6			us_variant	6.t1	VIGAN_05023500 [Vigna angularis var. angularis]		GGAGAAAAGCTGTGATCAATGATTTGCTAGAGGCCATAGCTGAATTAATTGATTGCAA
PHG9_Scaffold_294	1961			synonymous_variant	Mu_g04525.t1	nuclear transcription factor Y subunit C-9 [Vigna radiata var. radiata]	Histone-like transcription factor (CBF/NF-Y) and archaeal histone	CTGGGGATGGTTGACGTTGATCTGGAGGCTGGGGCCACATTTGTGGTGCCATGTAGGGGTGAGACTGCTGAGCATACATGTTTGGGTCCATCACAGGCTT[A/G]CCATTATGACACCAGCAGTCCCAACTTGAGGCGCATGCTGAGGTGGCATGTAGCAGTATGGAAGTGCATCAGCGGGACCTCCAACAGGCATTGTTCCTCT
PHG9_Scaffold_3003	1897			synonymous_variant	Mu_g16576.t1	hypothetical protein VIGAN_01195100 [Vigna angularis var. angularis]	Urb2/Npa2 family	TCAATTTTGTGCGGTCAAGAGTAGATAATGGTGTTGACTCTTGTCAGGATTGTAATACTATAAAATTTCCAAGACTAATATGTTACCTTAATGATTGGAT[T/C]TGACATTGCTATTCCCTCCAAATGGGAAGAAAAACTGGGGTGATGGGAAGACACCACAGTTGGAGGGAATTGAGGCATATATGGATATTCGTTGTTGGGA

PHG9_Scaffold_3072	388			synonymous_variant	Mu_g16729.t1	hypothetical protein PHAVU_004G005800g [Phaseolus vulgaris]	Cytochrome P450	AAGGTCCAAGAAACTCACCAGTGCCCCCATGTTGTTTCAGAATCTCAAATGCATAGTCATGGATGCGCCACAAATTACAGAGTGCCTGTGGTAGCATGCC[A/C]GGATGGGGTAGTCAGTGAGGAGGGGGTGTCTGCAACATAGTCTCCGACGGAAGAAGTACATGATGCAGAAGAGTGCTGCAATGGTTGCTGCTGCATAGCC
PHG9_Scaffold_3076	8362			synonymous_variant	Mu_g16741.t1	zinc finger CCCH domain-containing protein 48-like	WD domain, G-beta repeat	ATGGTTTTTAGGAGTATAATATCATGGATGGAAAGTTTGCAAGAAGGATTGAGCGCATTGGTGGAACAACATGCCTCTTTTGGCTGGCTGGGAGATGCAA[C/T]GAAACCCTTGTAGGTTTTTGCACAGAGAGACACCCTCACCACAAACTGCATGTGCTAATGCCAATACTTCCTATAGATATAGGAAAAATTCCTATAAGCG
PHG9_Scaffold_32	5665			synonymous_variant	Mu_g00614.t1	hypothetical protein PHAVU_002G258500g [Phaseolus vulgaris]	Cellulase (glycosyl hydrolase family 5)	TAACATCATTTCACACACAAATTCTTCAAAGCAACTATATTGAGCTCAATGGTTTCCACAAAAAGTTTGATGAATTCGAAATGGATATTGTTGAGGTTAC[T/A]AGATTGACCTCCTTCCGCTGTCAATAAGTTTGAACCATTGACTTGCAGGATCACATTTGTTATCTTTACTCAAGCATTTACAAGTATTAGTCACAATGAT

PHG9_Scaffold_3251	935			synonymous_variant	Mu_g17082.t1	SNW domain-containing protein 1 [Glycine soja]	NA	TCACGATCCCAATCACGTGTAATCTTTCTCCTCTTCCCCATAGAGGCATCCTTAGCTTTCAANNNNNNNNNNNNNNNNNNNNNNNNNNNNNNNNNNNNNNNNNN[G/A]NNNNNNNNNNNNNNNNNNNNNNNNNNNNNNNNNNNNNNNNNNNNNNNNNNNNNNNNNNNNATATCATCCTCATCCACTGACACAGTCGCATGCGGGGCGGGCA
PHG9_Scaffold_3251	026			synonymous_variant	Mu_g17082.t1	SNW domain-containing protein 1 [Glycine soja]	NA	NNNNNNNNNNNNNNNNNNNNNNNNNNNNNNNNNNNNNNNNNNNNNNNNNNNNNNNNNNNNNNNNNNNNNNNNNNATATCATCCTCATCCACTGACACAGTCGCATG[C/A]GGGCGGCACAACCCCAATTCTCTCCGACCGTGCCTTCTGCGCCAATGCCCTCAACTCCTGCTCCTTCTTCTCTTTCTCCTTCAAAATCATCTCTTTCTG
PHG9_Scaffold_3293	19162			synonymous_variant	Mu_g17176.t1	hypothetical protein PHAVU_002G132800g [Phaseolus vulgaris]	LrgB-like family	TTATCACCAATTTAGTTTCACTGTATACATAATGATTGTGATGAATGGCCATGCATAACATGATGTTTACCTTCAAAAAAAGACACAATGCTGAGAGCCA[A/G]GCCACTGTTATACATCTGGGTAGAATGGATATGGTTAAGGATGGTTCTAAAGCAACTAGACGTCCAACAAGGGCAGTTGAGTACAATGAGAATATGGTAG

PHG9_Scaffold_33	1351			synonymous_variant	Mu_g00641.t1	PREDICTED: cellulose synthase-like protein E1 [Vigna angularis]	Cellulose synthase	TCTTAGTGACATTCTCAAAATTCTGAGGGAACTGCACATAAGCAATATTGTGGCCTTTCTCTTCATCCATGAAAAAGCATAAAGCATCTCTCACAGACTG[T/C]AATTGTTTGAGTACATGTCACAGTCTACGTTTAGAATGATTTTCCCGTTACTAATATTCGAAGACACCCTTAACTGCATGCATTCAAACAGAAGAATTAT
PHG9_Scaffold_3411	7697			synonymous_variant	Mu_g17374.t1	hypothetical protein PHAVU_004G100100g [Phaseolus vulgaris]	Leucine rich repeat N-terminal domain	TTTTCCTTGACAATAACAAATTGCAAGGCGAGATTCCAGCTTCACTTGGGAACATATGCACATTACAGGAATTATACCTCAGCAATAACAACCTGACTGG[G/T]AAATTTCTAGCTTCAATCAGTATTCTTCATGGTGCAACAGAGCTGTATTTGGGATTCTTGATTTATCTTATAACCGGATTATTGGCATGCTGCCTAATCT
PHG9_Scaffold_3434	220			synonymous_variant	Mu_g17424.t1	PREDICTED: NEP1-interacting protein 1-like [Vigna angula	Ring finger domain	CAGCTGAGTTTTGTTTATAATTTAGAATTGCATGTGGTGTAGCCTGATCTTTTTGAGATATGCAGATTGATGTTATCGCCAGCTTACTGAGTGGGAGACT[T/A]TGCGTGAGAGGATAGGTCCAGCCATGTTGAGTGCTGTTCAAAGTCAGGTAATTTTCTTTCCCAAAGGACTTCTTAGGCTATGATTGGTTTAAGGTCACTT

						ris]		
PHG9_Scaffold_3434	229			synonymous_variant	Mu_g17424.t1	PREDICTED: NEP1-interacting protein 1-like [Vigna angularis]	Ring finger domain	TTTGTTTATAATTTAGAATTGCATGTGGTGTAGCCTGATCTTTTTGAGATATGCAGATTGATGTTATCGCCAGCTTACTGAGTGGGAGACTTGTGCGTGA[G/A]GGATAGGTCCAGCCATGTTGAGTGCTGTTCAAAGTCAGGTAATTTTCTTTCCCAAAGGACTTCTTAGGCTATGATTGGTTTAAGGTCACTTCCAATTTTC
PHG9_Scaffold_348	0960			synonymous_variant	Mu_g05037.t1	PREDICTED: tafazzin [Vigna angularis]	Acyltransferase	AGTGTAAAACATGTTTCTTGCTTTAAACTTTCCCCGTCAGGTTTGTCCTGGATGGAGATAGCATGCCCCTTGTTGTCCCATTTGTACATACAGGGATGCA[A/G]AGATTATGCCTATAGGTGCTAACTTTCCCAGAATCGGCAAGATGGTAAGCATTCTTGATTCGTTTCTTAAAATATTTTATCATCATATATGGGTTTTTTT
PHG9_Scaffold_3558	7299			synonymous_variant	Mu_g17577.t1	hypothetical protein PHAVU_003G287200g [Phaseolus vulgaris]	Rhamnogalacturonate lyase family	TCTCCTCTGAATTCTGGGTTGGTTGGTCTCGTTAATAGAACTGCTTCTGGGTATGCCAGGGTTTGACCAGTTTCTCTATCTTTCATTGTTGGCATGCTTC[T/G]TGCCTTTCATCTGACATTGCCATATAGTTGAACCTGCGCCGTTCAATTTTCTATTAGTAAATCACATAGTTCCATTTTCTGTTGCTTTCAAAAAGTTTAG
P				s	Mu	hypoth	Pectineste	TTGAGATCGCTAACTCCACTGAGCGTGACGCTTGGATCCTTGACTCAGG

HG9_Scaffold_37	4315			ynonymous_variant	_g00737.t1	etical protein LR48_Vigan01g303800 [Vigna angularis]	rase	GGTACCCTACAACCTGGGCCCACTTCCCACACCAATCCCTATGCCTGCAGC[T/A]AATTCATTTCTTCAAATCTAATCTCATAGACTATATATGTATGTAGTCATGCATATGTAGTTACAAATATATATTTAAATCCCTGCATGCTTGCATGCTC
PHG9_Scaffold_37	4509			synonymous_variant	Mu_g00742.t1	hypothetical protein PHAVU_003G106800g [Phaseolus vulgaris]	Fumarylacetoacetate (FAA) hydrolase family	CACTTGCAATCTGTAATTAGATGATGATGATGATGAAGGGGAAAGGGAAATTGTGTGTGTGAGAGAGAGAAGGTACCTTGGGAACGGCGTTGCCTAGCTC[T/C]TGGCGTGAGCAGCATAGTTTCTGCCAACAGCGACGATCTTGGTGCTCAATTCCAGAAGCTTCTGACAAGCTACCGCTGCCATTTCCCTCCTTACTCTATC
PHG9_Scaffold_3822	534			synonymous_variant	Mu_g17948.t1	hypothetical protein PHAVU_001G146800g [Phaseolus vulgaris]	Protein kinase domain	ATTTTTTCAAGAGTTCAGAATCAGAAACTAGCTCTTCCAAAGAACCCGGTACTGTGGATTCTGATAAGTCAAAACCCGGTTCTAGGTGGCACGCGTTTCT[T/G]AATTGTTGAGGGTCAAATCGAAGAAGCCAATAGCCATATTGCATCCTCTCAGTGTCCTCAAGCTCTCCAGAAGATTGAGCAGTGGCATGCGGGAGACTAT

PHG9_Scaffold_3822	625			synonymous_variant	Mu_g17948.t1	hypothetical protein PHAVU_001G146800g [Phaseolus vulgaris]	Protein kinase domain	CGCGTTTCTTCAATTGTTGAGGGTCAAATCGAAGAAGCCAATAGCCATATTGCATCCTCTCAGTGTCCTCAAGCTCTCCAGAAGATTGAGCAGTGGCATG[C/A]GGAGACTATAATAGATGCAGATTCGTCCTTGCATAGGCCACCCTGGAAGATCTTCAGTCACCATGAGATACAAATTGCAACCAATTACTTCGCCCAAGGT
PHG9_Scaffold_3843	1973			synonymous_variant	Mu_g17995.t1	hypothetical protein PHAVU_006G185600g [Phaseolus vulgaris]	1,3-beta-glucan synthase component	CCCAAAGGAAGGCATAAACCATCAGTACAATAACCATAGAGTTAAAATAAAATCCTATGGTCGTATAGAACACTGAAAGCATGCGAAAGAAGTCTAATCT[A/G]GGCCTAGACGGTACACATCTCTGCTCAGGACCTGCTCACCATTGCCACTAGCAACCTTGGCCTCAAACATGGATATCTGATTCAAACCAACATCTCTTCC
PHG9_Scaffold_3861	350			synonymous_variant	Mu_g18048.t1	Flavin-containing monooxygenase YUCCA8 [Glyci	Flavin-binding monooxygenase-like	TTGATGCTATCAAAATTGCTCAAGATATTGCCCAAGTTTGGAAACAAGAGACCAAGCAAAATAAACATCGTACCAGTGCTTGTCACAAACGTTGCATTTC[C/T]AGTTCTGAATTAACTCTCAGTGATGATGTTTCTTTTAGCTAGGAATTTGTTCATGTAAAAACAAGTTAACAATATCTTTTGTAAGAATAACAATAGCATA

						ne soja]		
PHG9_Scaffold_4543	4480			synonymous_variant	Mu_g18733.t1	hypothetical protein PHAVU_003G204700g [Phaseolus vulgaris]	Leucine rich repeat	GCTTGGTCAAGTTATGAAGGCGACCAAAAGAATCAGGAAGTGCATGAATTTGATTGTTGCTGAGATCCAAGTCCCTGAGGTTAATCAAATCACCAAATGT[C/T]CTGGAAGTTCTTTAAGGTCACTGAAGTTACTGCTCAGGTTTAGAACTTGAAGATTAGTCAATTTCCCAATTGCAATAGGAAGACCGCGCAGCTCGTTAAA
PHG9_Scaffold_4543	4486			synonymous_variant	Mu_g18733.t1	hypothetical protein PHAVU_003G204700g [Phaseolus vulgaris]	Leucine rich repeat	TCAAGTTATGAAGGCGACCAAAAGAATCAGGAAGTGCATGAATTTGATTGTTGCTGAGATCCAAGTCCCTGAGGTTAATCAAATCACCAAATGTCTCTGG[A/T]GTTCTTTAAGGTCACTGAAGTTACTGCTCAGGTTTAGAACTTGAAGATTAGTCAATTTCCCAATTGCAATAGGAAGACCGCGCAGCTCGTTAAAGTGAGC
PHG9_Scaffold_4550	3190			synonymous_variant	Mu_g18801.t1	PREDICTED: uncharacterized protein LOC1	Type II intron maturase	CGTAGCAAAACGTGACGAAGCGTTCGTCGTACACGATTTCCAACACCATCCGAATGGCCTCAACCACGACCTTCAGCTTCAAATTGGGTAAAACCAAAGG[C/T]AAGAAGAAGTGGATTTACTGGAGGGCGTAAATGTGACGCAGCATGCTTCCACGTTGAACCGATTTTCACGGAGCTCCCGGGACGTGGTCTCGATGCTGAG

						06774737 [Vigna radiata var. radiata]		
PHG9_Scaffold_4594	3853			synonymous_variant	Mu_g19107.t1	PREDICTED: DEAD-box ATP-dependent RNA helicase 37 [Glycine max]	DEAD/DEAH box helicase	AGAGAACAAGCGCTAGCGGATACACAGTTCGCACCCCACGAGGTGGCCTCTGGACAGGTTGGCCCCTCATAATTCCACTGATGATCGGGAAACAGAATGC[A/G]CTGTCTTTCCAGAACCAGTCTGTGCACAAGCCATCAAATCCCGTCCAGCTAGAGATATTGGTATGGCATGCCGCTGAACAGGTGTTGGTTTCACATATTT
PHG9_Scaffold_46	1570			synonymous_variant	Mu_g00876.t1	histone H2A [Sesamum indicum]	C-terminus of histone H2A	TTTCAAGGTCCGTCAAGGCCGGTCTCCAATTCCCCGTCGGAAGAATTGGCCGTTATTTGAAGAAAGGAAGGTATTCACAGCGTGTGGGAACCGGTGCTCC[T/C]TTTACCTGGCTGCAGTTCTTGAATACCTAGCTGCTGAGGTAACCTAGCATTAATTGAATCTCTCAGAGATTGTTAATTGTTGTCAATTTAGTGTTTTGAT
PHG9_Scaffold_46	1577			synonymous_variant	Mu_g00876.t1	histone H2A [Sesamum indicu	C-terminus of histone H2A	GTCCGTCAAGGCCGGTCTCCAATTCCCCGTCGGAAGAATTGGCCGTTATTTGAAGAAAGGAAGGTATTCACAGCGTGTGGGAACCGGTGCTCCTGTTTAC[C/T]GGCTGCAGTTCTTGAATACCTAGCTGCTGAGGTAACCTAGCATTAATTGAATCTCTCAGAGATTGTTAATTGTTGTCAATTTAGTGTTTTGATGATGGAT

				t		m]		
PHG9_Scaffold_4678	0074			splice_region_variant	Mu_g19369.t1	PREDICTED: F-box/WD-40 repeat-containing protein At3g52030 isoform X1 [Glycine max]	F-box domain	GCATTATTGCATGCCCTTTTGTGTGCATCTGTGTTGTAAGTGCTTATCAAGTCAACATGCAAGGTTTATATTGATGTTCAATACTTTCCTTTCAATTTAG[G/T]TTGCTCAGTGCCGAATGAAAATGGGCATGCTTGTTACTGGCGTGGGTGATAAGGTATGGGTGGTATTTCATCTCCTACATTTCCAATTTCTGATTTTTAA
PHG9_Scaffold_473	1372			synonymous_variant	Mu_g05796.t1	hypothetical protein PHAVU_001G066400g [Phaseolus vulgaris]	Protein kinase domain	TCAATGCAGGTGCATGCATAGCTTTCATAGCGTTGCTGTTGATTTCCTCGTTATACTTATTATATCATAAAAGGAGACTTAATAAACAAAGAGAAAATTT[C/T]TTCGACAAAATGGTGGTTTCATTTTGCAAAAACGACTTGCTACACAACAAGACACTCCCAGTATGGCACAGATTTTTGCAGCAAAAGAGCTAAAGAAGGC
PHG9	54			synonon	Mu_g1	hypothetical	N-terminal	CACTCGTTATAACCACATAATCCAGACCCCATGATGCAATTGCTTCAGCCACATTGGTGGGCTCATCAGGGTCTGGTGGTGGAGGAGTCCTCGATGTC

_Scaffold_4759	08			ymous_variant	9580.t1	protein KK1_043119 [Cajanus cajan]	domain of lipoyl synthase of Radical_SAM family	TT[A/G]CATTACAAAATCTGAAAATTAACAAGAAATAATTAAATGGAAGTGTTCTTGTGAAAAAACTAATCCATGTAAAACTATAACGAGTCTTGGGTATCATACT
PHG9_Scaffold_4816	7244			synonymous_variant	Mu_g20072.t1	hypothetical protein PHAVU_004G090900g [Phaseolus vulgaris]	pre-mRNA splicing factor component	TTTATTGCATTTTTCCATCCACAGAATTATAGTTATTACTATTAATTCTTTGCTAATAAGGAGTGAATTTTCAATTTTGTGCCTTTTGTGTGTAGACTGA[A/G]GGACGAGAGAAGAGGAGGAAAAGCTACTTCATCTTGCTAAGCTTATGCCCACACAGTGGAGAACAATTGCTCCAATTGTGGGTCGTACCCCTTCCCAGTG
PHG9_Scaffold_4816	7250			synonymous_variant	Mu_g20072.t1	hypothetical protein PHAVU_004G090900g [Phaseolus vulgaris]	pre-mRNA splicing factor component	GCATTTTTCCATCCACAGAATTATAGTTATTACTATTAATTCTTTGCTAATAAGGAGTGAATTTTCAATTTTGTGCCTTTTGTGTGTAGACTGAATGGAC[G/A]GAGAAGAGGAGGAAAAGCTACTTCATCTTGCTAAGCTTATGCCCACACAGTGGAGAACAATTGCTCCAATTGTGGGTCGTACCCCTTCCCAGTGTCTTGA
PHG9	73			synon	Mu_g2	hypothetical	pre-mRNA	ATCTTGCTAAGCTTATGCCCACACAGTGGAGAACAATTGCTCCAATTGTGGGTCGTACCCCTTCCCAGTGTCTTGAGCGGTATGAGAAGCTCCTTGAT

_Scaffold_4816	76			ymous_variant	0072.t1	protein PHAVU_004G090900g [Phaseolus vulgaris]	splicing factor component	GC[A/T]CCTGTGTGAAAGACGAGAACTATGAGCCTGGTGATGATCCTCGGAAATTGCGTCCTGGGGAGATTGATCCAAACCCCGAGTCAAAACCTGCACGCCCTGA
PHG9_Scaffold_4816	7385			synonymous_variant	Mu_g20072.t1	hypothetical protein PHAVU_004G090900g [Phaseolus vulgaris]	pre-mRNA splicing factor component	AGCTTATGCCCACACAGTGGAGAACAATTGCTCCAATTGTGGGTCGTACCCCTTCCCAGTGTCTTGAGCGGTATGAGAAGCTCCTTGATGCAGCCTGTGT[G/A]AAGACGAGAACTATGAGCCTGGTGATGATCCTCGGAAATTGCGTCCTGGGGAGATTGATCCAAACCCCGAGTCAAAACCTGCACGCCCTGATCCTGTTGA
PHG9_Scaffold_4816	7391			synonymous_variant	Mu_g20072.t1	hypothetical protein PHAVU_004G090900g [Phaseolus vulgaris]	pre-mRNA splicing factor component	TGCCCACACAGTGGAGAACAATTGCTCCAATTGTGGGTCGTACCCCTTCCCAGTGTCTTGAGCGGTATGAGAAGCTCCTTGATGCAGCCTGTGTGAAAGA[C/T]AGAACTATGAGCCTGGTGATGATCCTCGGAAATTGCGTCCTGGGGAGATTGATCCAAACCCCGAGTCAAAACCTGCACGCCCTGATCCTGTTGATATGGA

PHG9_Scaffold_4816	7916			synonymous_variant	Mu_g20072.t1	hypothetical protein PHAVU_004G090900g [Phaseolus vulgaris]	pre-mRNA splicing factor component	ATATTGAGGCACAATTAAGAAAGCAGGACATTGCAAAAAATAAAATTGCAGAAAGGCAAGATGCCCCATCTGCCATATTGCATGCCAACAAGTTGAATGA[C/T]CAGAAACAGTAAGAAAGAGATCAAAATTGATGCTTCCACCACCCCAGATTTCTGATCAGGAATTGGATGAAATTGCAAAGATGGGTTATACTAGTGACGT
PHG9_Scaffold_4816	7937			synonymous_variant	Mu_g20072.t1	hypothetical protein PHAVU_004G090900g [Phaseolus vulgaris]	pre-mRNA splicing factor component	AGCAGGACATTGCAAAAAATAAAATTGCAGAAAGGCAAGATGCCCCATCTGCCATATTGCATGCCAACAAGTTGAATGACCCAGAAACAGTAAGAAAGAG[A/G]CAAAATTGATGCTTCCACCACCCCAGATTTCTGATCAGGAATTGGATGAAATTGCAAAGATGGGTTATACTAGTGACGTCACAGGAAGTCAGGAACTTGC
PHG9_Scaffold_4816	0457			synonymous_variant	Mu_g20072.t1	hypothetical protein PHAVU_004G090900g [Phaseolus	pre-mRNA splicing factor component	TGGTTACTGACATTTTTCAGATGAGGGTAAAGAAAAGCCTCTGGCCTCAAATTGAGGCCACTTTCAAGCAGATGGACATAGCTGCAACTGAGTTAGAATG[T/C]TTAAAGCTTTGCAAAAACAAGAGCAATTAGCAGCATCACATAGGATAAACAATCTATGGGCTGAAGTACAGAAGCAAAAGGAACTTGAGAAAACTTTGCA

						vulgaris]		
PHG9_Scaffold_4831	67376			synonymous_variant	Mu_g20206.t1	Plasma membrane ATPase 4 [Glycine soja]	Cation transporter/ATPase, N-terminus	CCCCTGCATTAAAGAAAGCAGATATTGGAATTGCTGTTGCTGACGCCACAGATGCTGCAAGAAGTGCTTCTGACATTGTCCTCACTGAACCTGGTCTGAG[T/C]TCATTATTAGTGCAGTGCTCACCAGCAGGGCAATTTTCCAAAGGATGAAGAATTATACTGTTAGTTTCCTTGCATGCATGATTTAATTGCTTGACTATTT
PHG9_Scaffold_4861	1981			synonymous_variant	Mu_g20425.t1	hypothetical protein PHAVU_001G140900g [Phaseolus vulgaris]	Protein tyrosine kinase	CGAGTAATAAATTTGGATGGCAACATGGAACTTACTCCAGAGCCTGAGCAACACCTAGAGCAATCCTGAGACGGTGAACCCATGACAGAGGTTTGCATGC[C/T]CACTGTGAAGAGCGTCGTTTAGAGTTACATTTCCAACATAATCATAAACAAATAGACCCTTTCCATTCTCGATACAGTAACCATCGAGTGCTACAATATT
PHG9_Scaffold_4913	34933			synonymous_variant	Mu_g20756.t1	hypothetical protein PHAVU_011G061700g [Phaseolus vulgari	PWI domain	CAAGTATCTCCCCCAAGAGGTCTCCTCGAGATGAATGGAGTTCTCAATCTCCAATACGGAATGTATCAACCTCTCCAATTAGGAAAAATTCTCCAAGACA[C/T]AAAGAAGTCCCATGCAGTCTTCTATGAGAGTCAGGTATAGTGGCATCTAGGAAATGTTTGAATAATATTATCGTCTGTTTTGTCTCTGGCATGCTTGAAT

						s]		
PHG9_Scaffold_4936	71466			synonymous_variant	Mu_g20887.t1	hypothetical protein PHAVU_011G184700g [Phaseolus vulgaris]	POPLD (NUC188) domain	AACTTAAGAAAAACCTAGAGAATGGTTTTTGCACTTCTGGCGATGGAACCAAGAGGCTGAGAACTCATGTTTGGCATGCAAAGCGGTTTGCTATGACCAA[G/A]TTTGGGGTTACCATCTTCCTTTGTGTCTTCAAGGAAGGTAGGTTTTTTTTTGGTTAAAAGGAAAGATATGATAATGTTAATGGGGTAACTTGAAGCTAAT
PHG9_Scaffold_5022	30			synonymous_variant	Mu_g21326.t1	expansin-A16-like [Vigna radiata var. radiata]	Pollen allergen	AACTCCTAGCATGCAAATAAGGTATGATATATGTACGTATGTATGTAAGTAACCTGCGATATTTGATTGGGACGATGCCAGCCTCGTATTCTGCAATTTT[A/C]GATACGCAGGTTTGGCCAAATCAAAGTGATCGCGTGGTGGGTTGCACCATCCTCCATTGTCACCTGGTAGATCGTAATTTGGAGGACAAAAATTGGTTGC
PHG9_Scaffold_511	9408			synonymous_variant	Mu_g06026.t1	hypothetical protein PHAVU_002G108900g [Phaseolus	FAT domain	ATGGTGGAAACTCACATGGCCAAGAATCTGAAAGGTCTACAAGTGCAGAAAGCAGCATGCACAATGGAACTGATCAACCTTTGCAACAAGGTTCTGCAAA[C/T]TCAATGAAGGAGGTCAAAATACTTTAAGACGTGCTGCTGGTGCTTTAGGTTTTGTAGCTTCAGCTGCCAGTGCTTTTGATGCTGCTAAAGATATAATGGA

						vulgaris]		
PHG9_Scaffold_5113	3493			synonymous_variant	Mu_g21706.t1	hypothetical protein PHAVU_011G123200g [Phaseolus vulgaris]	NA	CAAACAGTTTGAAGATGCTGAGAAAGATGGATGAAGGAGAAATCATCACAAATGCTTTCCCTCAGAGCCTAACTACTTCTGCTGCATCTAATAGTAACAA[C/T]CTCCTCCTGCTTTGAAGAGGAAGAGAAACCTACCAGGAAACCCAGGCATGCTTGTTTCTTTCATCTTCTTCAGCCTCTTTATTTCTCTTCATTAATATA
PHG9_Scaffold_5183	22515			synonymous_variant	Mu_g21845.t1	hypothetical protein PHAVU_002G180200g [Phaseolus vulgaris]	Sugar (and other) transporter	TCACTGTCTCGCCATACTATTATTTTTCAACTATTTCTCCATATATGCTTGTTCTTACACTACTGTCTATTGTACATGCATGCAGCTCTTACCTGGGGC[T/C]GTTTGAGTTCTAATGACGTCACAAAGGCTATGTGTGGCAACGAGCATAGAGCATGGTACACAAAAGGGTGTCCTAGTAAAATCGGTTGGCTTGCAATAGT
PHG9_Scaffold_520	7798			splice_region_variant	Mu_g06180.t1	40S ribosomal protein S7 [Vigna radiata	Ribosomal protein S7e	TTATACATTAATTCAGCAATGTAAGCATGCCTGTTTGTGTTTCTGAGCAAGCTTGATTAGTTTTGTCTCTGACTTGTGAACGTCGTTTAACTATATGCAG[C/A]AAATTGATGTTTCTGGGAACCGCAAGGCTGTTGTTATTCACGTTCCGTACAGATTAAGGAAAGGTTTTCGCAAGATTCATGTTAGGCTTGTCAGGGAGCT

						var. radiata]		
PHG9_Scaffold_5307	2512			synonymous_variant	Mu_g22026.t1	cation/H(+) antiporter 4-like [Vigna radiata var. radiata]	Sodium/hydrogen exchanger family	GTTTCAAGCTCATGATGTTCCTTCTTTGATACCCAGCGTATTTCCTTGTAGGGTCATACAATGCCTTCACACCAAGCTGCGAAGCTGTTCCTAGGAACAT[C/G]CCGAGATGATCAAAATACTTATTACTTTTTATTTGTGCCTGCAATGGTTGAAGAATCGAAACCAGTTAATTAGTTAAACAATTGAACAAACACCAGGA
PHG9_Scaffold_532	8636			synonymous_variant	Mu_g06229.t1	hypothetical protein PHAVU_009G185200g [Phaseolus vulgaris]	NA	TTTTGGACCACATCAACTTCTGCGGGAAAGGCTTCCGGCGCTACGCGAAACTCTGCATGCACATGCTCTCACGGGGCAAGTTCAAGACACTGGAAAGGCC[G/A]TGGGGTTTATGTCTTCTTCTTCTTTTACTTTATTAATGTTTTCGAGTATTTCCCCTGATTTTTTAGGGAAAAGGGTAAAAAACTTGTACTAATTTTGATT
PHG9_Scaffold_5627	2634			synonymous_variant	Mu_g22214.t1	hypothetical protein PHAVU_007G134500g	Protein tyrosine kinase	TATAGTTCTCATCATTCTCATTAAGTGTTGAGTCAGAGCGTGCATGCAACATTCTGTACCGAGATCTCTCTCTAAAATCAGATTCTGACAATAAACTTGC[A/G]ACTCAGGAGGATGAGGGACAATCTTGTTAAAATGATATGAACCATCATTGGTAATTAAAGGTCTCTCATAACTCATACTATCTAGATTGCATACTGCAGA

						[Phaseolus vulgaris]		
PHG9_Scaffold_566	86487			synonymous_variant	Mu_g06444.t1	hypothetical protein PHAVU_006G114300g [Phaseolus vulgaris]	Auxin response factor	AATAGATACCTTCTCTCAGGTGAGTCCTCCACCTCAAATCTCATCTTGAAACGCATGCCAACTGAAAACTTGCAGTTGACGGCCTCCAGATATTTGTTTA[A/G]CCAATAATAAACTGGCTAGTCCTGAATGTATCAAAGGTTTACACATTTTGTTACATTTTCATTATAAGCTTATGTGCCACCTAAAACAAATTTCAACAT
PHG9_Scaffold_5675	34015			synonymous_variant	Mu_g22683.t1	hypothetical protein PHAVU_009G097600g [Phaseolus vulgaris]	Protein phosphatase 2A regulatory B subunit (B56 family)	AGGAATGATCAATGTACTTCTTGGCCACCTTAGCATCAAGAGAATTTGAGGTTATAAACTTGAGCAGCAACTCATACACGAGCTGCAAATGAGGCCAAGC[G/A]GATCAAACGCTGGCTCATCATCATCATTCTCACCACCACGATTGGACCTATAATTTGGCGGGAAAACGCGAAAGAGATTAATAGCACACATTCTACACAC
PHG9_Scaffold_5689	6075			synonymous_varian	Mu_g22807.t1	hypothetical protein PHAVU_011	Chlorophyll A-B binding protein	GGGGTGGCAAGCATGCTGACTTGGCCATGGTGTGATATCTCAACAACAACTGTGTCATCATCTTTTGGGAGGGGTTGTTTCCATGGCTCTCCATCAATTC[T/G]ATGAATGTGCACTCAGCTGCTCCCTTGTGAAACTCAAATCGGATTCTGCTTGTCTGCATCGTGAGAGAAGCATACAAACGTAAGTTTGAGCAAAAAGGAG

				t		G155800g [Phaseolus vulgaris]		
PHG9_Scaffold_5695	0558			synonymous_variant	Mu_g22882.t1	PREDICTED: DNA oxidative demethylase ALKBH2 [Glycine max]	2OG-Fe(II) oxygenase superfamily	TTAATAGCAGAATTGATGAGGAACAATGTTGTTTGAGAATCATTCTTACAGCATCCAACATGTCTTTAAGTCGTGGATAATCATCCCAAGAATATGCATG[C/A]GCTGATATCCACTGTAAGTCAACTCGGTCAATCCTGGAGTTGCAACATAACATGTGTCTCGAGGCTGCAGAGAATTACATTGTATTACATTACACAAAAT
PHG9_Scaffold_5711	29364			synonymous_variant	Mu_g23044.t1	PREDICTED: protein EXPORTIN 1A [Vigna angularis]	CRM1 C terminal	TTTGCAGTGAATTTCAACTGATACATGAGTTATGCTTGTATGTGTTATCGGCATCCCAGAGGACTGAACTTATCCGTGCGACTCTATCTACACTGCATGC[C/T]TTTTATCATGGATCCCCTTGGGTTATATATTTGAATCACCATTGGTAGGTAAAATTTTCAGCTAAAATGTACTTGTTTTGGACGATTCATAAACTGAACT
PHG9_Scaffold_	8136			synonymous_v	Mu_g23083.t	Beta-glucosidase D4	Glycosyl hydrolase family 1	TCATGAATTGAATTATCAATATAAATGTAATGCCAAAGCTTACTTGATACTTAGCCCTGTAGATGTTAGCAGCAGCTGCATGAGCAAGTAACTGATTGTG[G/A]CCACAATATAGGGTTCAGTGGCTGAATCCCCACCAGTGCAATTTAGATTTTGCCAAGGTGAACATCTTCCAGGTGCACCAATCCCTTTAGCAT

5713				arian t	1			AGCCATG
PHG9_Scaffold_5724	7067			synonymous_variant	Mu_g23184.t1	PREDICTED: uncharacterized protein At1g28695-like [Glycine max]	Nucleotide-diphospho-sugar transferase	TGAAATACTACTTTTACTTCTCAACTTTCACTGCTAATTCACATGTAACATCTTTCTCACAGGATACTGATGTAATGTGGTTAAGAAATCCATTTACAAG[A/G]TGAGTAAGGATGAGACAGAAGACATTCAAATGAGCACAGACGTGTACCATGGTGATCCTTGGTCAAGGAATAACCTAATCAACACTGGATTTTACTTTGT
PHG9_Scaffold_5724	7073			synonymous_variant	Mu_g23184.t1	PREDICTED: uncharacterized protein At1g28695-like [Glycine max]	Nucleotide-diphospho-sugar transferase	ACTACTTTTACTTCTCAACTTTCACTGCTAATTCACATGTAACATCTTTCTCACAGGATACTGATGTAATGTGGTTAAGAAATCCATTTACAAGATTGAG[T/C]AGGATGAGACAGAAGACATTCAAATGAGCACAGACGTGTACCATGGTGATCCTTGGTCAAGGAATAACCTAATCAACACTGGATTTTACTTTGTGAGGTC
PHG9_Scaffold_5737	5815			synonymous_variant	Mu_g23259.t1	PREDICTED: midasin-like [Glyci	Midasin AAA lid domain	AAAATTTTTTAAGTATTTCATCTTGCAAAACATGAAGGCACTTCAAAAGTGGAATGAAGCGACTGGCTTCTTCATCAAAATTATGTTGGCTCTCAATATT[T/C]GTTGCTGGAATGTTAGCCTTAGTGGATCAAAACAACTAATAGCATTAAAGAGATATTTAGTTTTGCCATTTGATGGAGCCAAATTAACTATATCAAGTGA

				t		ne max]		
PHG9_Scaffold_5737	5863			synonymous_variant	Mu_g23259.t1	PREDICTED: midasin-like [Glycine max]	Midasin AAA lid domain	GTGGAATGAAGCGACTGGCTTCTTCATCAAAATTATGTTGGCTCTCAATATTTCGTTGCTGGAATGTTAGCCTTAGTGGATCAAAACAACTAATAGCATT[A/G]AGAGATATTTAGTTTTGCCATTTGATGGAGCCAAATTAACTATATCAAGTGACAACAACGGCATTAACTGCAAATCAACATCTAGTTGGTCACGGCGTGA
PHG9_Scaffold_5737	2455			synonymous_variant	Mu_g23259.t1	PREDICTED: midasin-like [Glycine max]	Midasin AAA lid domain	GCATTTAACATAAGATCAAAAAATAAAGATATCATATAAGAAATAAGGGAAGTAGAACAAACTAAATCATAAACAAAAAGATCATACCTAGACTTAACCC[A/G]CAAGCACAACAAGAAATACTCCTTGAAGAAGTGCATGCTCTGGTCCTAAGCTTTTCTCCATTGCAATAAAAAAGTCAACCCAAGAAATGAGATCTCTCAC
PHG9_Scaffold_5747	8533			synonymous_variant	Mu_g23338.t1	soyasapogenol B glucuronide galactosyltransferase [Vigna radiata var. radiata]	UDP-glucoronosyl and UDP-glucosyl transferase	AAAACTGTTCAGCAAAAAGAGGCCATGTCACCATAGGCAAACCTGCATCCACAGTTTCAATAATTGTGTTCATCCCACAATGAGTCACCACACCTCCAAT[A/G]CAGGATGCTCCAGAATGAGAAGTTGTGGAGCCCAACCCCATATCAGATAACCCTTGTTGCTTGCTTCTACCCTCTTCTCAAACTCCTCCACAAAAACTCT
PHG9	18			synonymous	Mu_g2	hypothetical	NA	TCAGGTCCTTTAGGAACCATACTTTCTGCAGCAAAGTTGGCAAATATGCCTTGAGTCCATCCACTAGATCGAATCCGTAAAATCCTTTCACTATGTCGC

_Scaffold_5773	63			ymous_variant	3643.t1	protein VIGAN_10176300 [Vigna angularis var. angularis]		C[T/G]AATTCTGAGTCAAGAGCTTCTTCTTCCAACTTCTGAGCAAGACGCCTCATGGCACTAGGATTAAGGTGGCATATAAATAGATCAAGCATGCTTTCATAAT
PHG9_Scaffold_5778	5462			synonymous_variant	Mu_g23711.t1	hypothetical protein PHAVU_007G173700g [Phaseolus vulgaris]	Endonuclease/Exonuclease/phosphatase family	CATGAACCCGGGGAAACCTTGTCTTTCTGAGAATCTCCATTACATCTGAATTTCTCCTTACCTCGTCACCCTCCTTTTGTCCAGAAGTCAAATGACTACA[G/T]TGAAGCAAAAGCTTGTTTGGTGCAAAGACATGCTAATTGATATTGAACCCTGGAAAACTCAAGGCAATGAGAATGTTGTCTCCAGGTTCTCAATTAACCT
PHG9_Scaffold_5782	6667			synonymous_variant	Mu_g23776.t1	uncharacterized protein LOC106776651 [Vigna radiata var. radiata	Protein of unknown function (DUF620)	CATCACGAATTGCTGGACTTGTTTCCAATTTCAAGATGAAGCACTCTTCATCATTGATTATTTTCTCTCCTATGCATGCAGCGTCCAAAAATAAGTTAGC[A/C]TTGCCCTCGGATCCAATCCCTATAATAAACAAATAATGGTGGATTAAATGTTGGAGGAAGAAAAATATTTGTGGATTAAACCATTTTGTTTAAAGAAATT

						]		
PHG9_Scaffold_5802	67602			synonymous_variant	Mu_g24022.t1	PREDICTED: putative multidrug resistance protein [Vigna angularis]	ABC transporter transmembrane region	GCACCTGCAACAAACACGTACAAAAGATGTTACCAGAGAGTTAAGAAAAAAATGCTGGAAATTGAGTATGTATATATACCTTATAGGCCATAGTATCTTG[A/G]TTGTTTGAGCGTCAGAAGTTATGGTGGCAATAACTCGGAAAGTTGTAGAAGAGTCAGTTTGCTTGTCGAAGAAACCAACCTCTTGCCTTAGAACTGATTT
PHG9_Scaffold_5831	88548			synonymous_variant	Mu_g24320.t1	hypothetical protein PHAVU_002G331600g [Phaseolus vulgaris]	NF-X1 type zinc finger	ATTTGCCTATTCAGAAGAAAGAATCCCTCGTATCTGCTTCAGTAAACCCTTGGAGTGTCTTAAATCAAGACTCTTCTCCAAGTTCATCGGTTGCAGCTGT[T/A]AAATTGATTTTTCTAGGGAACACTCCGAAAGTAGTGCTGTTACAAAGTTGGAGCCTCATAATGGTGGTTCAAATCTAAGAGGACAGCATGCAGGAAACTT
PHG9_Scaffold_630	2984			synonymous_variant	Mu_g06782.t1	hypothetical protein PHAVU_011G2162	Protein kinase domain	AGTTGTAATAAACATAGACACTAGAGATAGAGGTTTTTCAATCAACAAAATAAAATTACCTTGTTTATTATAAGATCAGCATGCTTCACAAGAAGTAACA[G/A]GTCTCACCAGCAGCAGTACTCAACACAGGGACAAGGGCTGGAAGTGTAGATTGCTCAAAATCATTCTTGTCCTGGAAAGTAAATTGTTCTTTGCACTTCA

						00g [Phaseolus vulgaris]		
PHG9_Scaffold_66	724			synonymous_variant	Mu_g01359.t1	PREDICTED: F-box/LRR-repeat protein 10 [Vigna angularis]	Leucine Rich repeat	ATTCATCCACATCATCATAATCATGTGTATATAACCCATCTGAAGTGTCATAGGGATCAGCCCCAAGCTCCTCACCATGGCATGCCACATGTAAGAAAGG[T/C]TGCTTCTAGCTAAAGCATCTACCATGTCTCTATTAACATTTCCGGTCACTCCCAACGATTTGAGCCTTGGAAAATATGGTTTCTTCAACCAGCGAAATGC
PHG9_Scaffold_688	895			synonymous_variant	Mu_g07027.t1	PREDICTED: putative pectinesterase/pectinesterase inhibitor 28 [Glycine max]	Pectinesterase	AAAGTGTATCTTGATATGCATCCATTGAACAATTGTAGAAGATAGACTTGTCTGCTTGGACTCTCAATGCCACCGCTTGATGTTTTTCAGGTCCAGCAGA[G/A]TCTCAAATCCCATGTTGATGGCCACAAAGTAATCTCCTTGAACAGCTACATGCATAAAGTGATCAATTAATTTATTCAATTAAAATTCCATAAATTACAA

PHG9_Scaffold_74	2337			synonymous_variant	Mu_g01518.t1	hypothetical protein PHAVU_004G173700g [Phaseolus vulgaris]	Gamma-glutamyltranspeptidase	TCTTTATGGCATGCAGCTAATACCAAATGTAGTTCGTTATGAGAACTTGACTGCACTGAGTGGTGATCAGATTGAGCTTTCGAAAGAAAGAAGGAATTTC[C/T]AGAAGAAAGAGGGCATGAACTGAGTGAGTGTGAAGCACTAGCTGTCACTCAACTTGTTGTCCAAAATCTCAAAACCCCTACCAACAAGAATAGGAAAATT
PHG9_Scaffold_756	27310			synonymous_variant	Mu_g07383.t1	PREDICTED: uncharacterized protein LOC108325651 [Vigna angularis]	Plant invertase/pectin methylesterase inhibitor	ATATAGCAATAGTAACCTAATTCCTCCTCCATGATTACAACTTGCTACTTTTAACAATGGTGTCCATGATGGCAAGGCAATTGCCACCTAACTGGAACAG[A/C]CCGTGGAGTCCTGGGCGAAGGGCATGGCGACTCCAGGAGACTCTGAGAAAGAGTCTTCGCAGGCGGATTTGTACGATATCACAGCGCTCAATTTGCTTTG
PHG9_Scaffold_756	27376			synonymous_variant	Mu_g07383.t1	PREDICTED: uncharacterized protein LOC108325	Plant invertase/pectin methylesterase inhibitor	TGATGGCAAGGCAATTGCCACCTAACTGGAACAGAGCCGTGGAGTCCTGGGCGAAGGGCATGGCGACTCCAGGAGACTCTGAGAAAGAGTCTTCGCAGGC[G/C]ATTTGTACGATATCACAGCGCTCAATTTGCTTTGAGCCTCCACAAAGTTCATCTTCGCCACCAGATCCAGTGCCTCCTTGATGGAGTCCAACATGTCATT

						651 [Vigna angularis]		
PHG9_Scaffold_758	7743			synonymous_variant	Mu_g07403.t1	PREDICTED: probable galactinol--sucrose galactosyltransferase 2 [Vigna angularis]	Raffinose synthase or seed imbibition protein Sip1	TTACATGAAACATATCCCAGTCAGGTTGCATGAACTCTCCCAGGAAAAGTGTGTTATATGCAACAGATGAAATGTGAATTGTGTGGGAAGCAGGATCATG[G/A]GATAAAAGTCATCAGATGCTCTCACAACAGCAGTCTGCTTGGAACTATAAAGGCCATCAGTGTTATGACACATACAGGCAATGCATCCGTTGTCAGGAAA
PHG9_Scaffold_758	7761			synonymous_variant	Mu_g07403.t1	PREDICTED: probable galactinol--sucrose galactosyltransferase 2	Raffinose synthase or seed imbibition protein Sip1	AGTCAGGTTGCATGAACTCTCCCAGGAAAAGTGTGTTATATGCAACAGATGAAATGTGAATTGTGTGGGAAGCAGGATCATGGGGATAAAAGTCATCAGA[T/G]CTCTCACAACAGCAGTCTGCTTGGAACTATAAAGGCCATCAGTGTTATGACACATACAGGCAATGCATCCGTTGTCAGGAAAATTCCGAGCTACTGAGGC

						[Vigna angularis]		
PHG9_Scaffold_758	7809			synonymous_variant	Mu_g07403.t1	PREDICTED: probable galactinol--sucrose galactosyltransferase 2 [Vigna angularis]	Raffinose synthase or seed imbibition protein Sip1	ATGAAATGTGAATTGTGTGGGAAGCAGGATCATGGGGATAAAAGTCATCAGATGCTCTCACAACAGCAGTCTGCTTGGAACTATAAAGGCCATCAGTGTT[A/G]GACACATACAGGCAATGCATCCGTTGTCAGGAAAATTCCGAGCTACTGAGGCCTCAAGTGCCTGATGATAGCTCCGCGTGAGTGTGACCCTACCACCATG
PHG9_Scaffold_758	7920			synonymous_variant	Mu_g07403.t1	PREDICTED: probable galactinol--sucrose galactosyltransferase 2 [Vigna	Raffinose synthase or seed imbibition protein Sip1	AGGCAATGCATCCGTTGTCAGGAAAATTCCGAGCTACTGAGGCCTCAAGTGCCTGATGATAGCTCCGCGTGAGTGTGACCCTACCACCATGTCCACTACC[T/A]GGGTCTCAATAATGTTTTGGACATCAACCTTCACTCCATCTACTCCACATGAAGCTAAGTAAGCATGAAGCTCATTGTAGAAATCAAAAACCTTCTTCGG

						angularis]		
PHG9_Scaffold_834	39157			synonymous_variant	Mu_g07910.t1	PREDICTED: cellulose synthase-like protein E6 [Vigna angularis]	Cellulose synthase	ATTGGTTACCTTTCTGCAGAAGATTCAATGTTGAACCTAGGTCCCCAGAGGTTTTCTTTACTCATTCCCAAAACTATAGCAGTAGCACTAGCACTGAATA[T/C]GCAAAGCATGCTTATTTATCAAGGTAACCAAGTTATTAACTATGCCCATATAATGCTATCTTCATTAAGCCGTGAAAACTAATGAGATTATATTCCATTC
PHG9_Scaffold_843	2744			synonymous_variant	Mu_g07949.t1	PREDICTED: PI-PLC X domain-containing protein At5g67130-like [Vigna angularis]	NA	GAAAAGAATCAAAGCCGCTAAATTCTAGAAGTACATCACTTTTCTTGCAGAATTACTTTCCAACATATCCAGTTGAAGCTGAATCATGCAAAGAGCATTC[G/A]CTCCACTTGCTGATATGGTGAACACATGTTACAAAGCTGCAGGAAATGTGTTGCCTAATTTTATAGCAGTTAATTTTTACATGGTATTCCTTTTTCTTCT
PHG9_Scaf	8814			synonymo	Mu_g0812	protein FAR-RED	NA	AGAGCATGCAGATTTATGTACTTAAGGGTAGAACAGATGTAGAAAGCTCAGAATCTGATTCTGTCAAAGACAGCAACTGTTTCTCTGAAGATTCCCTTAC[T/G]CCATGTCTGTAAATGAAGAAGGTAAACTTGTTGCCGATTCTGCGA

fold_864				us_variant	7.t1	ELONGATED HYPOCOTYL 1 isoform X1 [Vigna radiata var. radiata]		TGACATTCCAACATGAGAGGCCTTCCAATTTGGTTGGTTTCGATGGCAATAATAA
PHG9_Scaffold_93	81943			synonymous_variant	Mu_g01892.t1	hypothetical protein PHAVU_009G005500g [Phaseolus vulgaris]	NA	CATCCGGCAGTTTCTCTCTGACTGAAGAACGGCCAGTGTTTCCTACATGGAGCAATGAAGTGGGCATGCGCTCCTATGGTGGATCTTCAGATGGTTGGTC[A/T]TACCTGGTTTTTCTGAGCTGATGGGAACTCCTCACAGAGAAAGGTGGTCATTTGATAGTGAGTCCTTTGGTTTTAACCATGAAAGATTAGTTAGACCCAG
PHG9_Scaffold_947	9845			synonymous_variant	Mu_g08426.t1	uncharacterized protein LOC106779136	NA	AAGGGGCTCTGCATGCACCGCCACGCAATGCTATGTTGCTGCTTAATCGAAATCGTTTAAAGAAGAACCTGTCTTTGAAAGGTAGTTTAAGCAAGGAATT[T/C]CAGGTAAGGTGAGGAAAAATGGGACATTTTCTTCACAGCTGTTTGGTGGAAGTTCAGCTGATGGGGAGGAAGTTGGTTGCTCCAAGGCGACCGCAGGTCC

						[Vigna radiata var. radiata]		
PHG9_Scaffold_999	094			synonymous_variant	Mu_g08714.t1	PREDICTED: ACT domain-containing protein ACR10-like [Vigna angularis]	NA	ATGAAGATGTTTCTACGGATGGGAAATGGTGCTACATAGTTTTCTGGGTGGTTGGGAAACAGAGGACGAGGTGGAGTTTGCTGAAGAAGAGGCTGATTGA[G/A]CATGCCCTTCTTGCTCCTCGGCTTCTGGAATCTCTTATTATCGATCTGAGTTGCAGCCGTCCAAGCCTCCTGATGTCTTTCTTTTGAATTTTTGCTGTCA
