# Supplementary material for: Genome, Transcriptome, and Germplasm Sequencing Uncovers Functional Variation in the Warm-Season Grain Legume Horsegram Macrotyloma uniflorum (Lam.) Verdc
Source: Front Plant Sci. 2021 Oct 18;12:758119. doi: 10.3389/fpls.2021.758119 (PMC8558620; doi:10.3389/fpls.2021.758119)

Genome, Transcriptome, and Germplasm Sequencing Uncovers Functional Variation in the Warm-season Grain Legume Horse Gram *Macrotyloma uniflorum* (Lam.) Verdc.

H. B. Mahesh[1,*], M K Prasannakumar[2], K. G. Manasa[1], Sampath Perumal[3,4], Yogendra Khedikar[3], Sateesh Kagale[5], Raju Y. Soolanayakanahally[3], H. C. Lohithaswa[1], Annabathula Mohan Rao[6] and Shailaja Hittalmani[6]

Supplementary Figure S1. Morphological features of horse gram variety PHG-9.

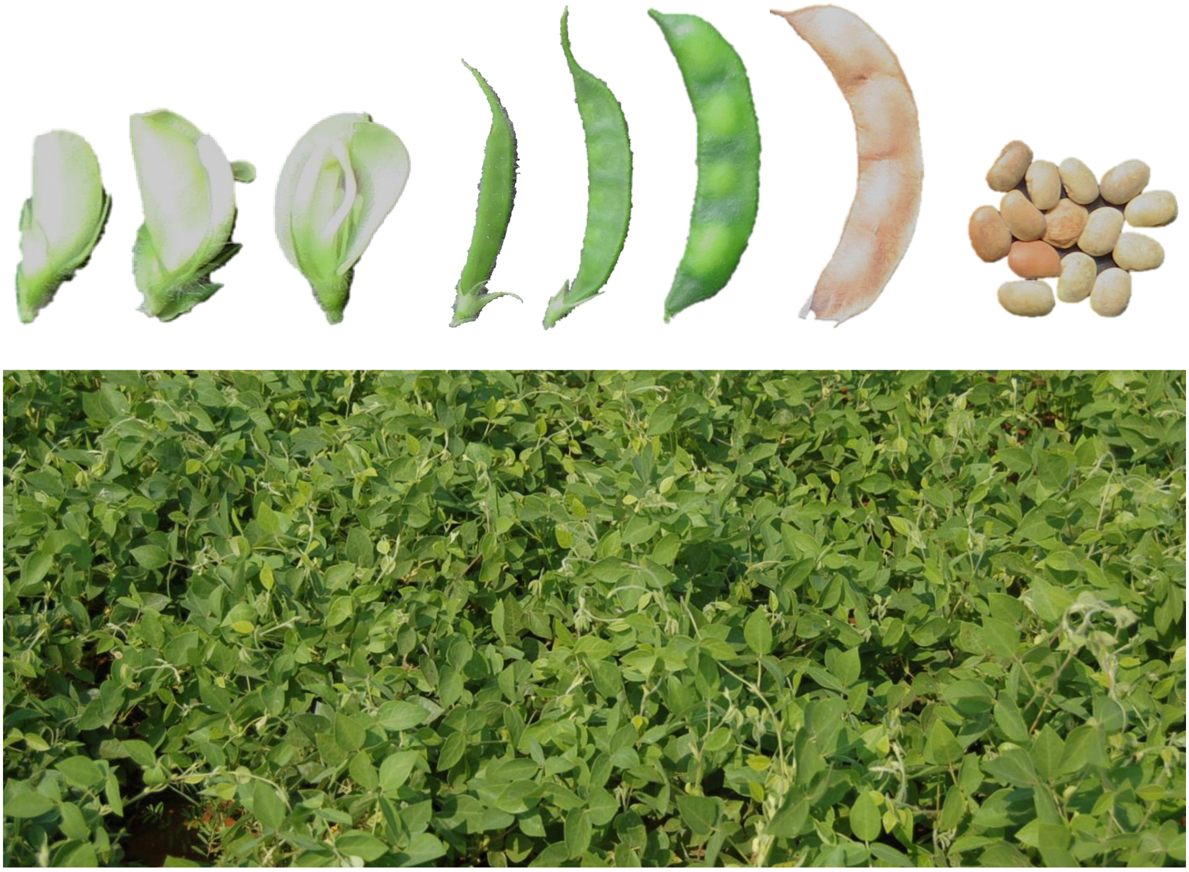

Supplementary Figure S2. BUSCO assessment of the horse gram genome.

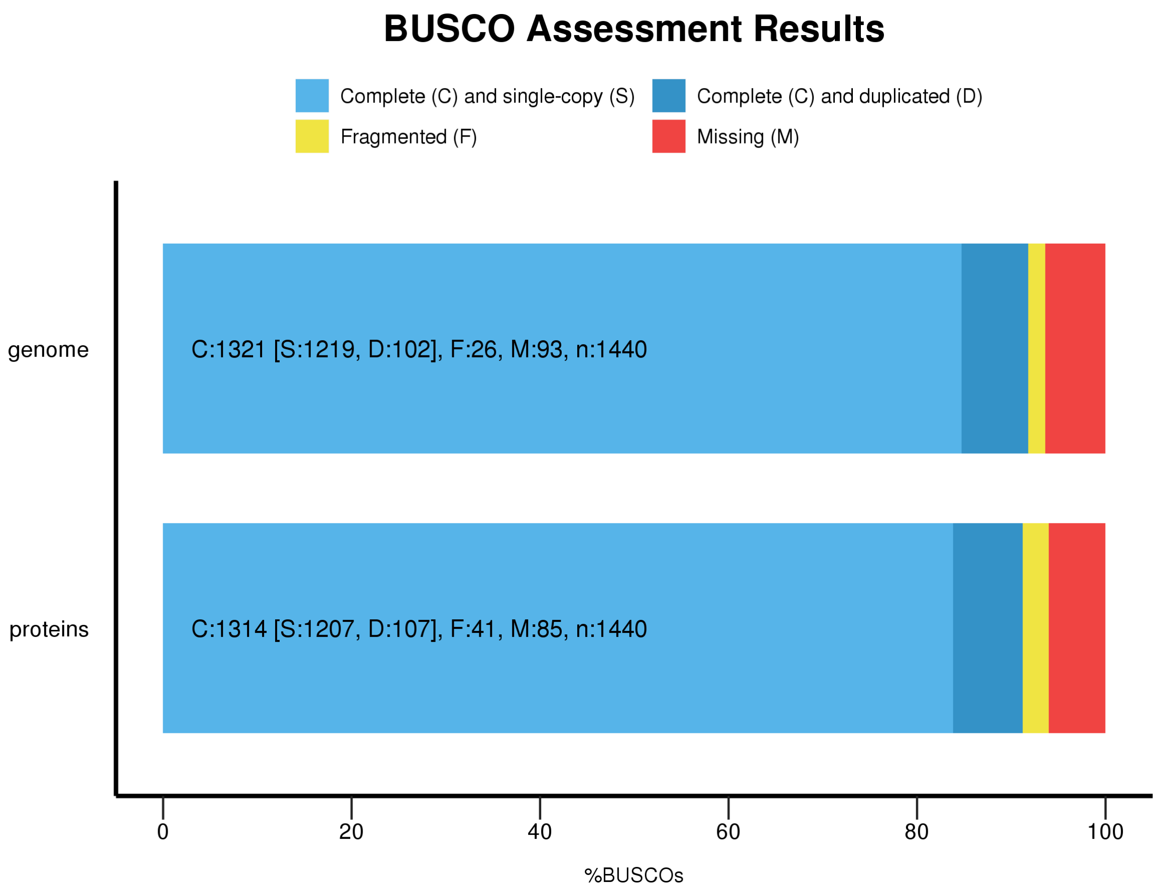

Supplementary Figure S3. Evolutionary relationship of CCT-domain proteins of horse gram with rice and *Arabidopsis* based on protein sequences. Genes highlighted in red, green, and black are from *Arabidopsis*, rice, and horse gram, respectively.

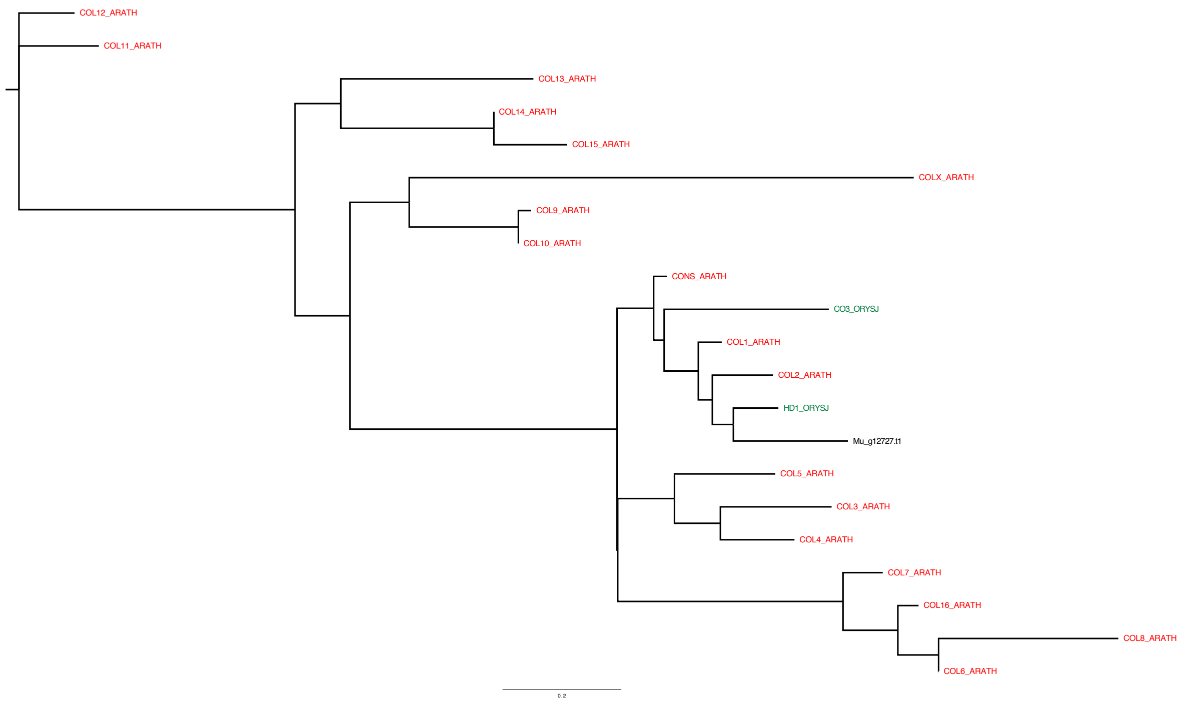

Supplementary Figure S4. Functional annotation of SNPs.

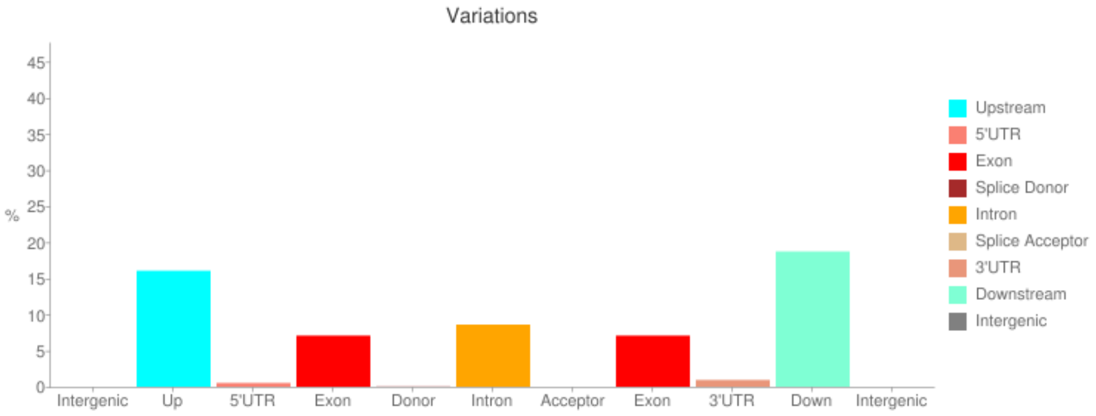

Supplement: Supplementary file 3 [file Data_Sheet_3.PDF]
